# Proliferation-Independent Initiation of Biliary Cysts in Polycystic Liver Diseases

**DOI:** 10.1371/journal.pone.0132295

**Published:** 2015-06-30

**Authors:** Jean-Bernard Beaudry, Sabine Cordi, Céline Demarez, Sébastien Lepreux, Christophe E. Pierreux, Frédéric P. Lemaigre

**Affiliations:** 1 Université catholique de Louvain, de Duve Institute, Brussels, Belgium; 2 INSERM U889, Université Bordeaux 2, Bordeaux, France; Zhejiang University, CHINA

## Abstract

Biliary cysts in adult patients affected by polycystic liver disease are lined by cholangiocytes that proliferate, suggesting that initiation of cyst formation depends on proliferation. Here, we challenge this view by analyzing cyst-lining cell proliferation and differentiation in *Cpk* mouse embryos and in livers from human fetuses affected by Autosomal Recessive Polycystic Kidney Disease (ARPKD), at early stages of cyst formation. Proliferation of fetal cholangiocyte precursors, measured by immunostaining in human and mouse livers, was low and did not differ between normal and ARPKD or *Cpk* livers, excluding excessive proliferation as an initiating cause of liver cysts. Instead, our analyses provide evidence that the polycystic livers exhibit increased and accelerated differentiation of hepatoblasts into cholangiocyte precursors, eventually coalescing into large biliary cysts. Lineage tracing experiments, performed in mouse embryos, indicated that the cholangiocyte precursors in *Cpk* mice generate cholangiocytes and periportal hepatocytes, like in wild-type animals. Therefore, contrary to current belief, cyst formation in polycystic liver disease does not necessarily depend on overproliferation. Combining our prenatal data with available data from adult livers, we propose that polycystic liver can be initiated by proliferation-independent mechanisms at a fetal stage, followed by postnatal proliferation-dependent cyst expansion.

## Introduction

Polycystic disease of the liver is characterized by the presence of biliary cysts leading to hepatomegaly. It is found in patients with polycystic kidney disease—both the autosomal dominant and recessive forms—but when associated with mutations in the Protein kinase C substrate 80K-H, *SEC63* or Low density lipoprotein receptor-related protein 5 genes, it presents as an isolated liver disease. Liver cysts are also found in syndromes that are collectively characterized by dysfunction of primary cilia [1, 2].

The large size of hepatic cysts and their connection to the biliary tree led to the hypothesis that hepatic cysts start to develop in fetuses through excessive proliferation of biliary precursors, although this was not experimentally demonstrated [3–6]. Indeed, Autosomal Recessive Polycystic Kidney Disease (ARPKD) is caused by mutations in *PKHD1*, which codes for fibrocystin/polyductin, a protein associated with the apical membrane and the primary cilium [7–9]. The association of fibrocystin/polyductin with primary cilia led to the speculation that cyst formation and expansion result from ciliary dysfunction. Since primary cilia regulate multiple functions including proliferation, it was suggested that hyperproliferation initiates liver cyst formation in ARPKD [3, 5, 6, 10–12]. This suggestion was supported by reports indicating that cyst-lining cells in PCK rat livers overproliferate [13–15]. However, all data where proliferation was measured, *i*.*e*. not simply deduced from histological phenotypes, were collected in adult livers, and fetal studies were not statistically validated [15]. Therefore, careful analysis of proliferation in mouse models and in human ARPKD at fetal stages is lacking.

During normal liver development, cholangiocytes differentiate from hepatoblasts and from ductal plate cells located around the portal vein [16]. Primitive ductal structures (PDS) then develop within the ductal plate. Their lumina are lined on the portal side by cholangiocytes and on the parenchymal side by hepatoblasts [17]. By the end of gestation PDS differentiate to bile ducts entirely lined by cholangiocytes and surrounded by mesenchyme, while the ductal plate cells not involved in bile duct formation differentiate into periportal hepatocytes, canals of Hering and adult progenitors [18]. Duct development progresses from the hilum to the periphery of the lobes, explaining why distinct stages of duct development can be identified within the same lobe in fetal liver. Importantly, cholangiocyte proliferation in fetal liver is low, indicating that bile ducts grow by recruiting hepatoblasts that differentiate into cholangiocytes [18]. This raises the possibility that cyst development in polycystic diseases is initiated in a proliferation-independent manner. Here, we address this hypothesis by analysing liver samples from human fetuses affected by ARPKD. We also investigate cyst formation in *Cpk* mice, which develop cysts as a result from mutations in *Cystin1*, a gene coding for a cilium-associated protein [19, 20].

## Materials and Methods

### Animals

*Cpk* [21] and *Sox9-CreER;Rosa* ^*YFP*^ mice [18] were backcrossed for at least 6 generations in a Balb/C background. Bromodeoxyuridine (BrdU) labeling was performed by injecting pregnant females at the indicated time with a single intraperitoneal dose of BrdU (20 mg/kg). Embryos were collected 1 h after injection. Tamoxifen (Sigma (T5648), Bornem, Belgium) was dissolved in corn oil (Sigma (C8267), Bornem, Belgium) at a concentration of 30 mg/ml and intraperitoneally injected into embryonic day (E)14.5 pregnant mothers at 100 mg/kg body weight. Tamoxifen had no adverse effects on embryos but induced late delivery and caused the mothers to devour the pups at birth. Thus, following cesarean birth at E19.0, newborn pups were transferred to foster mothers and collected 24 h later.

### Human fetal liver

ARPKD fetuses had mutations in the *PKHD1* gene [22] and showed kidneys with radially oriented dilation of the medullary collecting ducts [23]. The human samples have been studied in previous studies and were collected prior to the start of the present work [23, 24]. We analyzed 1 normal liver at 13 weeks of gestation (W), 1 normal liver at 22W, 1 ARPKD liver at 13W and 2 ARPKD livers at 22W. The normal livers were from a fetus suffering from trisomia 18 (13W) and from a foetus suffering from infection (22W).

### Ethics statement

The work on human tissue samples was performed in compliance with the French regulation (section L. 1241–5 Code of Public Health), and with the 1975 Declaration of Helsinki. The samples were collected in France by a pathologist who is not co-author of the present paper, prior to the start of the present analysis. An anonymous registration number was tagged on each sample at the time of collection, and researchers who analyzed the samples had no access to the name of the donor. The French regulation does not require referral to a research ethic committee for tissue samples used for scientific purposes aimed to investigate the causes of the spontaneous or therapeutic abortions. The women who underwent an abortion gave their written consent after receiving adequate information about the purposes of such samples.

All experimental procedures involving the use of animals were approved by the Animal Ethics Committee of the Université catholique de Louvain (permit number 2012/UCL/MD/021) and were conducted in compliance with the animal welfare regulations of Belgium. Animals received humane care according to the criteria outlined in the "Guide for the Care and Use of Laboratory Animals" prepared by the National Academy of Sciences (NIH publication 86–23 revised 1985).

### Immunofluorescence

Liver tissues were formalin-fixed and paraffin-embedded. Five μm-thick slides were stained as described [18] using antibodies and conditions listed in S1 Table. Pictures were taken with an Axiovert 200 fluorescent microscope using AxioVision system or a Cell Observer Spinning Disk confocal microscope (both from Carl Zeiss, Zaventem, Belgium). The number of proliferating cholangiocytes was established by determining the proportion of Ki67^+^;Sox9^+^ cells compared to the total number of Sox9^+^ cells ((n^Ki67+;Sox9+^/n^Sox9+^) x 100).

In human embryos, numbers of Sox9^+^ cells counted were 726 (control 13W), 498 (control 22W), 830 (ARPKD 13W), 906 and 1086 (ARPKD 22W samples). The percentage of Ki67^+^;Sox9^+^/Sox9^+^ cells was measured per image field for each sample. A total of 9 (control 13W), 11 (control 22W), 6 (ARPKD 13W), 8 and 5 image fields (ARPKD 22W samples) were analyzed.

In mouse embryos, we counted 275 and 600 Sox9^+^ cells in WT and *Cpk* embryos, respectively (n = 3), and calculated the percentage of Ki67^+^;Sox9^+^/Sox9^+^ cells per animal. We applied Student’s t test to the mouse samples to determine statistically significant differences.

### Microarray

Total RNA was extracted from WT and *Cpk* embryos at E18.5 (n = 3) using TriPure reagent (Roche). Quality and integrity of RNA samples were assessed using a Bioanalyzer RNA 6000 Nano kit (Agilent Technologies). Genome-wide expression was assessed using GeneChip Mouse Genome 2.0 ST arrays (Affymetrix). Results are provided in S2 Table. Gene set enrichment analysis was performed as described [25].

### Microdissection of embryonic portal spaces

WT and *Cpk* livers at E17.5 were formalin-fixed and paraffin-embedded. Series of adjacent 10 μm-thick sections were cut, the first of which was subjected to H&E staining to locate portal spaces, which were manually dissected out of the adjacent slides. Approximately 45 portal spaces from as many tissue slides were pooled from each liver and subjected to RNA extraction using RecoverAll Total Nucleic Acid Isolation Kit (Ambion). The experiment was performed separately on 2 WT and 2 *Cpk* embryos. A total of 500 ng RNA was reverse-transcribed using MMLV (Invitrogen). qPCR was performed on 5 ng RNA on a C1000 Themocycler coupled to a CFX96 Real-Time system (BioRad) using Kapa SYBR FAST qPCR Master Mix (Sopachem). Two-tailed Student’s t-test was applied to determine statistically significant differences (p<0.05). qPCR primer sequences are available upon request.

## Results

### Developing biliary cells do not proliferate in two models of polycystic liver

**Fig 1:**
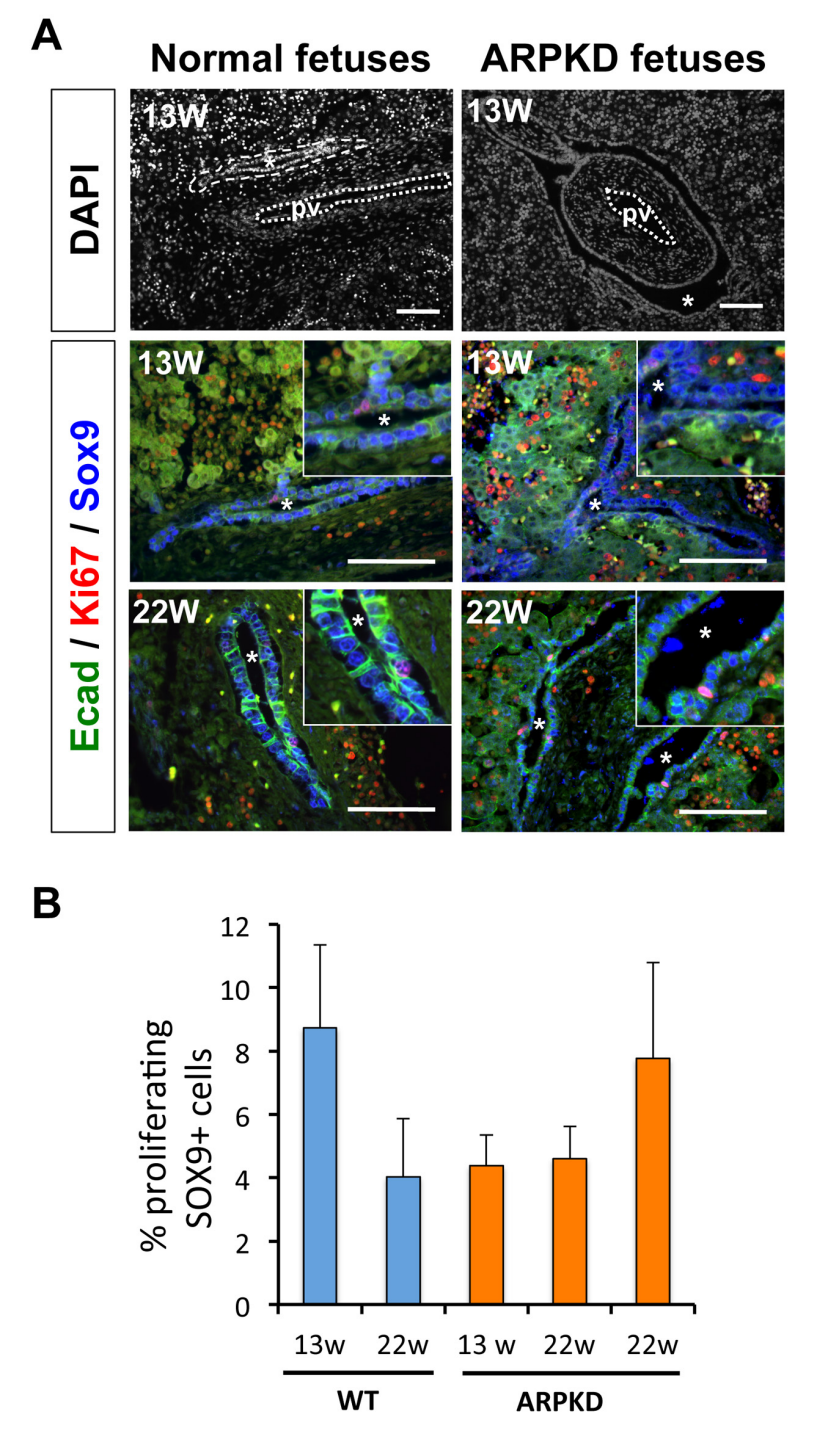
Liver cysts in human ARPKD fetuses do not exhibit increased proliferation. **(A)** *(Upper panels)* Comparison of WT and ARPKD fetal livers. Tissue sections were stained with DAPI. Cysts in ARPKD fetuses almost entirely surround the portal vein. Asterisks indicate cysts and ducts. *(Middle and lower panels)* Immunofluorescent stainings showing the occasional presence of Sox9^+^/Ki67^+^ cells in WT and ARPKD livers. Bars: 100 μm. **(B)** Percentage of proliferating Sox9^+^ cholangiocyte precursors, using the following formula: (n^Ki67+;Sox9+^/n^Sox9+^) x 100. Over 500 (WT) and 800 (ARPKD) Sox9^+^ cells per liver were counted. The two ARPKD livers at 22W of gestation were analysed separately. Data are shown as mean +/- SEM (see methods).

Human fetuses affected by ARPKD develop cysts around the portal vein, which is consistent with a ductal plate origin [23]. No subcapsular cysts are detected. In many instances the ductal plate was almost entirely cystic from 13 weeks of gestation (13W) onwards, suggesting that most cholangiocytes in the ductal plate are involved in cyst formation (Fig 1A). We assessed proliferation of differentiating cholangiocytes in normal fetuses and ARPKD individuals at 13W and 22W of gestation. We measured proliferation of biliary cells by coimmunofluorescent staining against Sex-determining region Y–related HMG box transcription factor 9 (Sox9) and Ki67, which mark biliary cells and cycling cells from G1- to M-phase, respectively. Biliary cells from wild-type (WT) fetuses exhibited low proliferation, extending earlier data on mouse fetal liver [18] (Fig 1A). Importantly, Sox9/Ki67 co-stainings revealed no difference in proliferation of biliary cells between WT and ARPKD fetuses (Fig 1A), irrespective of gestational stage. This was confirmed by quantification (Fig 1B). We reached similar conclusions by analyzing proliferation using staining against the phosphorylated form of histone H3 (P.H3), which specifically marks cells in M-phase (data not shown). The percentage of proliferating biliary cells in human fetuses was similar to that measured in the mouse model (see below). Thus, biliary cysts in ARPKD fetuses, despite their significant size, do not result from excessive proliferation of biliary precursors.

**Fig 2:**
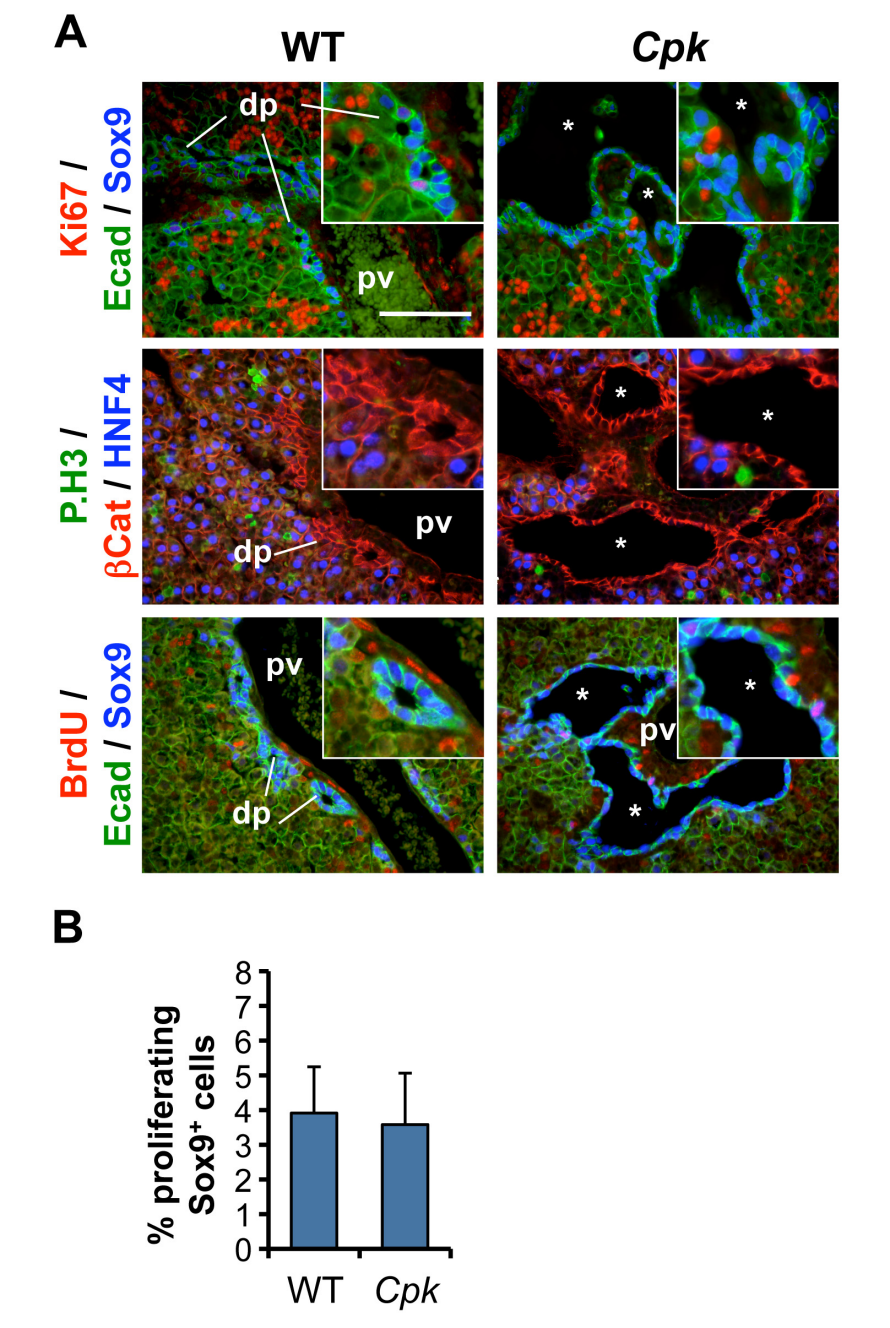
No excessive proliferation during biliary cyst formation in mouse *Cpk* embryonic livers. **(A)** Immunofluorescent staining against the indicated markers was performed on tissue slides from WT and *Cpk* mouse livers at E17.5. Proliferation rates, revealed by Ki67, P.H3 and BrdU stainings, are low in WT and *Cpk* cholangiocytes, while the rest of the liver exhibits higher levels of proliferation. *, cysts; pv, portal vein; dp, ductal plate. Bars: 100 μm. Insets: 2-fold higher magnifications. **(B)** Percentage of proliferating Sox9^+^ cholangiocyte precursors, calculated using the formula (n^Ki67+;Sox9+^/n^Sox9+^) x 100, like in Fig 1B (n = 3 for each genotype). Data are mean +/- SD. Two-tailed t-test was performed, and a p-value <0.05 was considered significant.

To extend these observations in a second model of polycystic liver, we measured the proliferation of cholangiocytes in embryonic livers from WT and *Cpk* mice [19]. *Cpk* mice develop periportal liver cysts when bred in the appropriate genetic background [19, 21, 26–28]. In these mice, large cysts are readily detected around portal veins by E17.5 [24]. We measured proliferation of Sox9^+^ biliary cells by staining for Ki67, P.H3, and BrdU. The latter, which marks nuclei of cells during S-phase, had been injected one hour prior to collection of the fetuses. Embryonic biliary cells exhibited very limited proliferation in WT embryos (Fig 2). Moreover, staining for BrdU, Ki67 and P.H3 in *Cpk* livers at E17.5 did not reveal any increase in proliferation within cysts (Fig 2A and 2B).

### Accelerated differentiation of biliary cells in *Cpk* liver

**Fig 3:**
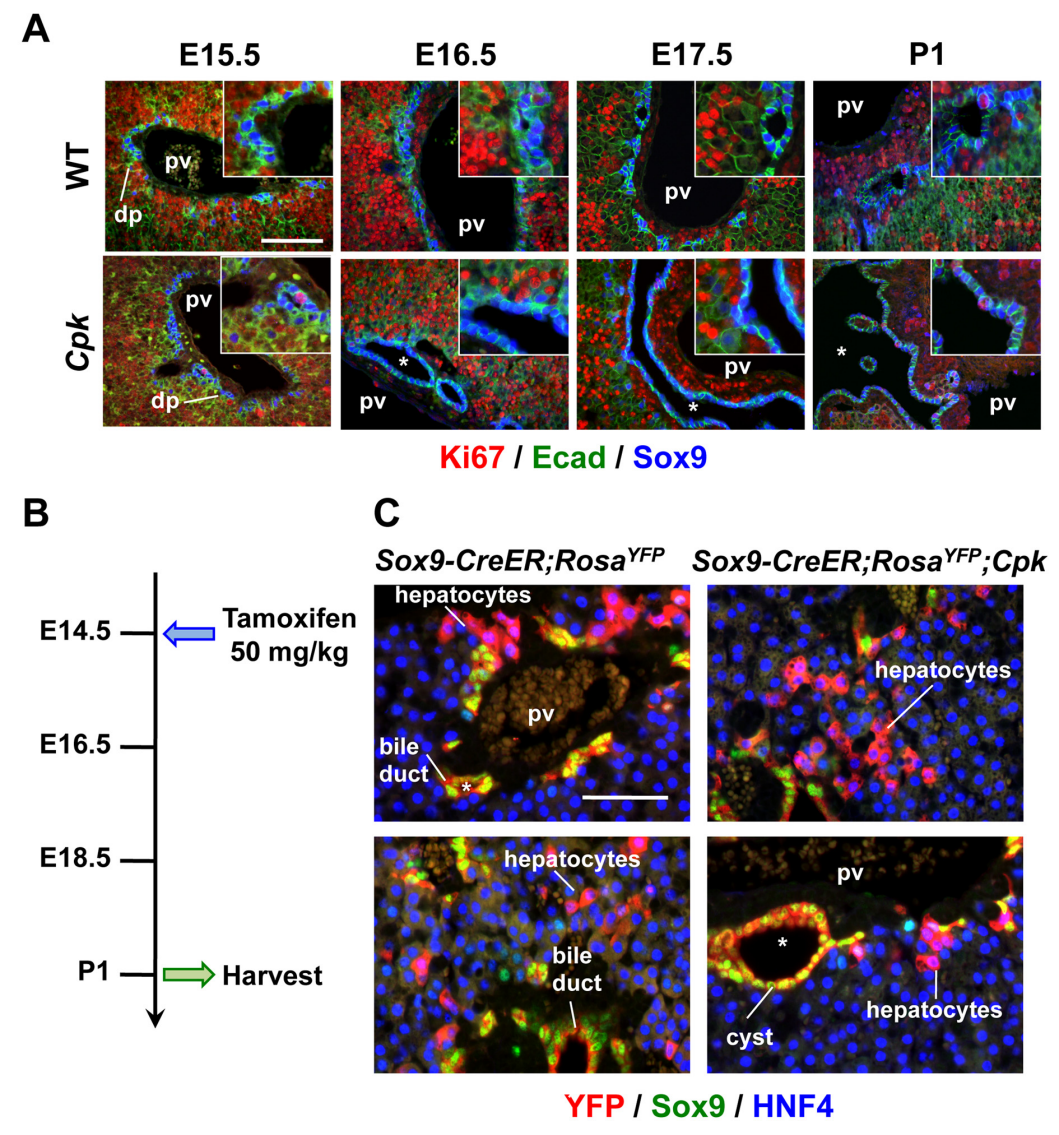
Cysts form in *Cpk* embryos, without excessive proliferation and with normal differentiation of ductal plate cells into periportal hepatocytes. **(A)** Time-course analysis of biliary cyst formation in *Cpk* mouse embryos. First signs of biliary cysts can be observed at E16.5; full-blown cysts are observed with 100% penetrance from E17.5. *, cysts, pv, portal vein; dp, ductal plate. Bars: 100 μm. **(B)** Scheme of the Sox9 lineage tracing experiment experiment. Pregnant mothers bearing *Sox9-CreER;Rosa* ^*YFP*^ *;Cpk* ^*mut*^ embryos were injected at E14.5 with a single dose of tamoxifen, and newborn pups were collected at P1. **(C)** Sox9^+^ ductal plate cells can give rise to periportal hepatocytes in *Cpk* livers. Lineage tracing of *Sox9-CreER;Rosa* ^*YFP*^ *;Cpk* ^*mut*^ ductal plate cells detected differentiation of periportal HNF4^+^/Sox9^-^/YFP^+^ hepatocytes in *Cpk* mouse livers at P1. Bars: 100 μm.

Analysis of embryos at several stages of liver development revealed that cyst formation in homozygous *Cpk* livers occurred very abruptly. Indeed, while no cyst was detected at E15.5 (Fig 3A), at E16.5 the ductal plate became locally irregular and showed some dilated PDS; full-blown cysts were detected at E17.5 with a near 100% penetrance. Importantly, at E15.5 and E16.5, prior to cyst formation, there was no increase in biliary cell proliferation in *Cpk* livers (Fig 3A). At birth, the number of proliferating biliary cells increased slightly in WT pups, as expected [18], but did not differ between WT and *Cpk* pups (Fig 3A). These data indicate that prenatal initiation of cyst formation in *Cpk* livers does not result from increased proliferation of biliary precursors.

Development of cysts cannot result from reduced apoptosis, since the ductal plate and developing bile ducts do not undergo apoptosis [18]. Therefore we envisaged that abnormal cell fate determination might lead to accumulation of biliary cells in *Cpk* livers. Indeed, by the end of gestation the ductal plate normally generates bile ducts but also periportal hepatocytes that progressively lose expression of Sox9 and acquire hepatocytic markers. In polycystic liver, ductal plate cells may fail to differentiate to periportal hepatocytes, leading to an accumulation of differentiating biliary cells and expansion of cysts. To test this hypothesis, we traced the fate of Sox9^+^ ductal plate cells from the fetal period until birth in *Sox9-CreER;Rosa* ^*YFP*^ mice [18] backcrossed in the *Cpk* background. To label Sox9^+^ cells, we induced CreER-mediated YFP expression at E14.5 by a single injection of Tamoxifen in pregnant mothers (Fig 3B), and analyzed the livers at postnatal day P1. YFP^+^ hepatocytes were still detected in the periportal areas *of Cpk* pups with near maximal YFP-labeling of biliary cells (Fig 3C). Thus, lack of differentiation of ductal plate cells to hepatocytes in *Cpk* mice is unlikely to be a major cause of biliary cyst formation. Low and variable efficiency of ductal plate cell labeling by Sox9-CreER in a *Cpk* background prevented us to perform accurate quantifications of the lineage tracings. Therefore, our lineage tracings do not exclude that a small proportion of ductal plate cells maintains its biliary fate and fails to convert to hepatocytes, thereby contributing to some extent to perinatal cyst expansion.

**Fig 4:**
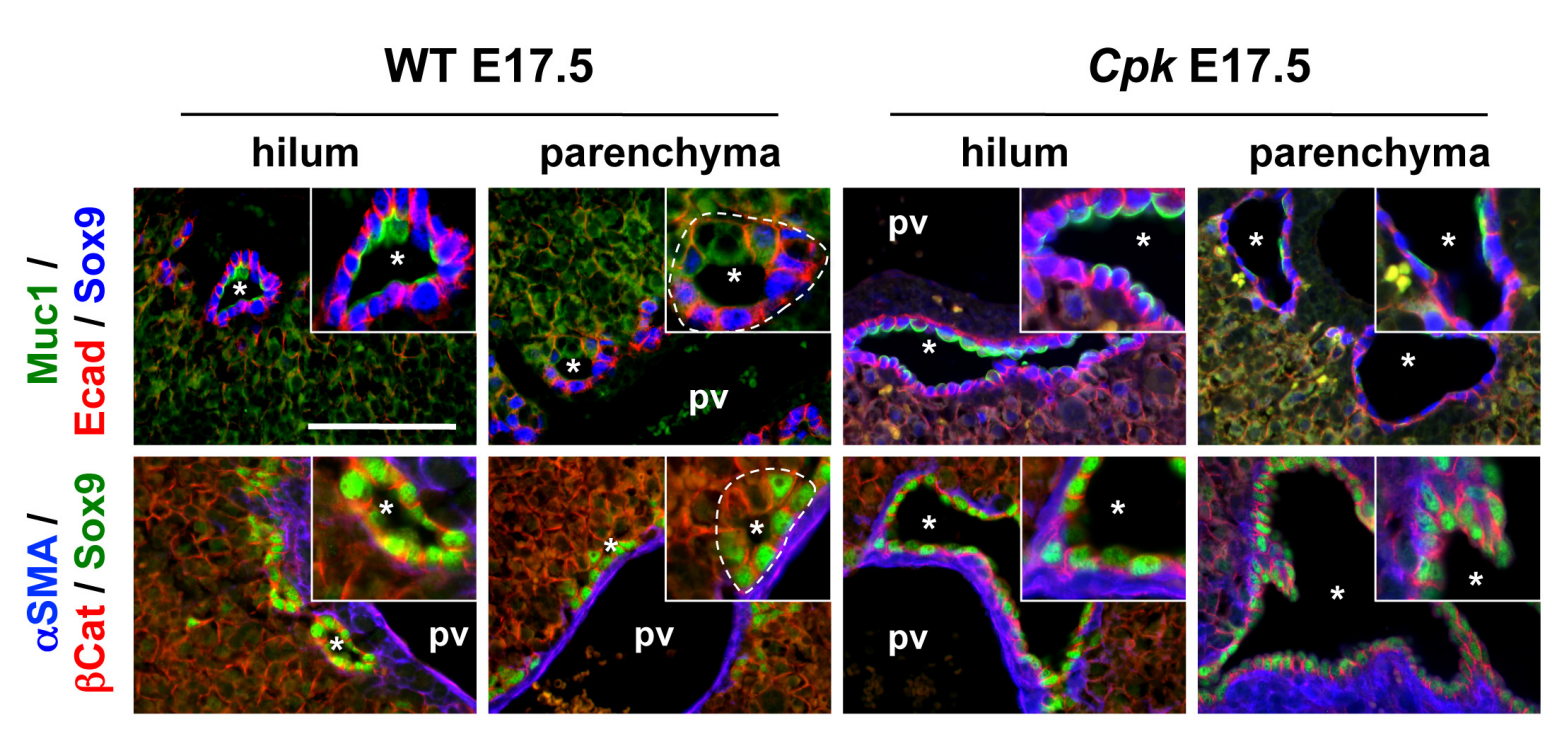
*Cpk* embryonic livers show excessive and accelerated biliary differentiation. Biliary differentiation was assessed by immunofluorescent staining against the indicated markers in E17.5 WT and *Cpk* livers. In WT livers, most PDS were asymmetrical, lacked expression of the maturation marker Muc1 and were not surrounded by mesenchyme, except for some PDS adjacent to the hilum. In contrast, all cystic structures in *Cpk* livers were symmetrical both at the hilum and in the parenchyma, expressed biliary maturation markers, and were surrounded by mesenchyme. Bars: 100 μm. Insets: 2-fold higher magnifications.

Since defective differentiation of ductal plate cells to periportal hepatocytes is unlikely to explain the formation of biliary cysts, we reasoned that excessive and accelerated differentiation of hepatoblasts into biliary cells might cause cyst formation. Our earlier work has shown that biliary cells lining cysts in *Cpk* mice and human ARPKD were well differentiated [24]. Further analysis now revealed that biliary differentiation was accelerated in *Cpk* livers. Indeed, at E17.5 the biliary cysts were invariably symmetrical throughout the lobes (Fig 4): all cells lining the cysts were Sox9^+^ and the cysts were nearly completely surrounded by mesenchyme. In contrast, the WT littermates showed the expected hilar-to-peripheral gradient of differentiation: PDS adjacent to the hilum were entirely lined by Sox9^+^ cells, but did not yet show alpha-Smooth Muscle Actin (αSMA)^+^ mesenchyme on their parenchymal side; at a distance of the hilum, most PDS in WT livers were still asymmetrical since they were lined by Sox9^-^ cells on their parenchymal side; a limited number of symmetrical PDS could be observed but lacked αSMA^+^ mesenchyme. We concluded that excessive and accelerated biliary differentiation contributed to cystogenesis in *Cpk* mice.

**Fig 5:**
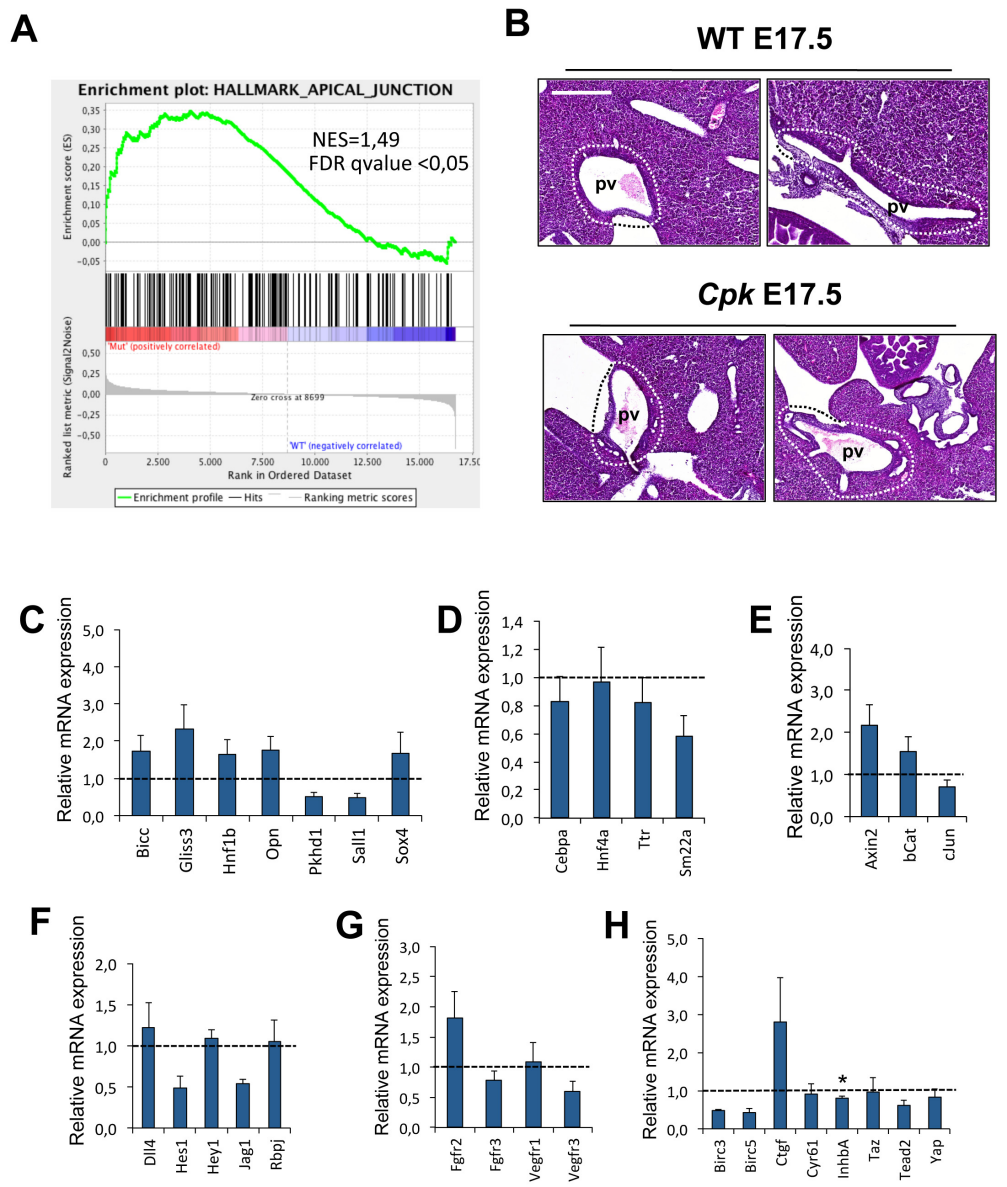
Expression of genes in *Cpk* livers. **(A)** Gene set enrichment analysis revealed enrichment of apical junction proteins. The gene set is shown in S3 Table. **(B)** Manual microdissection of WT and *Cpk* portal tracts at E17.5 on formalin-fixed, paraffin-embedded livers. Representative H&E staining of 2 individuals of each genotype are shown. The area of microdissected tissue is delineated by a dotted line. Bar, 400 μm. **(C-J)** qRT-PCR analysis in WT and *Cpk* portal tracts. Shown here are differentiation markers of **C:** cholangiocytes, **D:** hepatocytes and mesenchyme (*Sm22a*); markers of signaling activity of the **E:** Wnt/βCatenin, **F:** Notch, **G:** FGF and VEGF, **H:** YAP/Hippo pathways (n = 3 for WT, n = 5 for cpk). All gene expression data were normalized for the expression for the biliary marker Sox9. Student’s t-test was applied to determine significant difference (*, p<0.05).

To identify potential genes and pathways involved in this phenomenon we performed genome-wide microarray expression assays on WT and *Cpk* embryonic whole livers at E18.5. Mutants could be identified at dissection by the cystic extrahepatic biliary tissue and the pancreatic duct, while PCR confirmed the *Cpk* genotype. Nonetheless, WT and *Cpk* livers exhibited similar expression profiles, indicating that global levels of gene expression remain unaltered in *Cpk* livers. Known regulators of biliary differentiation were not significantly up- or downregulated (S2 Table), and gene set enrichment analysis did not uncover significant anomalies of signaling pathways known to promote biliary differentiation. However, the latter analysis uncovered that a gene set comprising apical junction genes is enriched in the *cpk* livers (Fig 5A; S3 Table). This gene set comprises several claudin genes, including Claudin 7 which in liver is biliary-specific [29]; claudins are associated with tight junctions and were shown to be required for normal bile duct formation [30].

Since gene expression is minimally affected in whole *Cpk* embryonic livers, we focused on the portal tracts. The latter were microdissected from WT and *Cpk* livers at E18.5 (Fig 5B) and qRT-PCR analyses were performed to analyze the expression of transcriptional regulators and signaling effectors known to control biliary differentiation (Fig 5C–5H). Since biliary cells are more numerous in *Cpk* liver portal tracts than in controls, we normalized the expression of the tested genes to the expression of the biliary marker Sox9. While the mean values for hepatocyte and mesenchymal gene expression were slightly reduced, consistent with a lower number of hepatocytes and mesenchymal cells in the *Cpk* portal tracts, this reduction did not reach statistical significance (Fig 5D). Similarly, mean values of biliary markers (Fig 5C) and of Wnt signaling effectors (Fig 5E) were upregulated in *Cpk* portal tracts, but the data were not statistically significant. Analysis of Notch (Fig 5F), FGF and VEGF (Fig 5G), and of Hippo signaling (Fig 5H) did not support an involvement of these pathways. When the measured values were not normalized for Sox9 expression, only *Osteopontin*, *Axin2*, *Hes1*, *Fgfr2 and Ctgf* showed slight but statistically significant upregulation (not shown). Together, these observations do not identify a specific mechanism promoting biliary differentiation in *Cpk* livers but leave open the possibility that combined alterations of several mechanisms contribute to the formation of cysts in ARPKD embryos. These alterations should preferentially occur in periportal cells, explaining why cysts are systematically located near periportal areas.

## Discussion

Initiation of biliary cyst formation in polycystic liver disease is usually considered to result from overproliferation of cyst-lining cells. This commonly held view is based on histological stainings of fetal liver without *bona fide* measurements of proliferation or without statistical validation of proliferation data [15, 31]; it also relies on analyses of postnatal liver cells, and on the observation that cysts in adult liver contain growth factors and cytokines known to promote proliferation [13–15, 32, 33]. Therefore, analysis of proliferation in polycystic livers at fetal stages was lacking. We decided to address this question in human ARPKD and in a mouse model with polycystic liver. ARPKD is caused by mutation in *PKHD1*. To broaden the scope of our analysis, we selected a mouse model that has a mutation in a distinct gene, namely the *Cpk* mouse which has a mutated *Cystin1* gene. Here we show that there is no increase in proliferation rate in ductal plates before and during cyst initiation in two models of polycystic livers, in human and mouse. We propose that cyst formation is proliferation-independent, and provide evidence that excessive differentiation contributes to cystogenesis at least in a subset of polycystic liver diseases.

Cysts develop around branches of the portal veins, suggesting that in human ARPKD and *Cpk* embryos hepatoblasts display an excessive response to a biliary differentiation-inducing signal emitted by the portal endothelium or surrounding mesenchyme. Cystin1 is associated with the cilium [19, 20] and the latter organelle regulates the activity of several signaling pathways involved in cellular differentiation, including pathways known to stimulate biliary differentiation such as the Notch and Wnt pathways [34–37]. However, overexpression of Notch or Wnt effectors does not induce polycystic livers [35, 38]. Thus, it is likely that none of the known pathways involved in biliary differentiation can, upon perturbation, lead to liver cyst formation single-handedly. However, we cannot rule out that slight perturbations of mutiple pathways combinatorially induce polycystic liver. In this context, our gene set enrichment analysis revealed that apical junction proteins are enriched in *Cpk* livers. Defective cilia function is associated with perturbed expression of the tight junction-associated proteins claudins, including upregulation of a subset of claudins [39]. Claudins are required for normal bile duct development [30]. Taken together, data in human ARPKD and in *Cpk* mice open the possibility that tight junction regulation and biliary differentiation are intimately connected.

Enrichment of cystin and/or fibrocystin in cells located close to the periportal area may explain why cysts develop only around portal veins. In line with this, purified embryonic cholangiocytes show a dramatic enrichment of cystin and fibrocystin mRNA expression as compared to hepatoblasts (data not shown). Dynamic three-dimensional analysis of bile duct development revealed that it normally starts in close vicinity of the portal vein with formation of small cysts, defined by a size that is smaller than 40 μm and with a luminal diameter larger than 5 μm; subsequently, segments of larger size form, most likely by connection and elongation of small cysts [40]. In the present work, we noticed that polycystosis in *Cpk* livers starts at E16.5, a developmental stage at which cyst and segment formation increases in normal embryos. Therefore, acceleration of this early stage of biliary construction is a hallmark of the polycystic liver models studied here. The dynamic model of biliary development revealed that after birth, biliary network density decreases while bile ducts enlarge, elongate and move at some distance away from the portal vein [40]. Takashima and coworkers suggested that postnatal reduction of the biliary network may result from conversion of a subset of biliary cells to hepatocytes [18, 40]. We therefore hypothesized that cyst formation or expansion may result from failure to reduce the network by lack of conversion of a subset of biliary cells to hepatocytes. However, our lineage tracings do not support this hypothesis.

In the present work we provide evidence that cyst initiation results from increased differentiation of biliary cells.

Several mechanisms are proposed to contribute to cyst development. These are increased cell proliferation, enhanced fluid secretion, abnormal cell–matrix interactions, alterations in cell polarity, abnormal ciliary structure or function, and autocrine and paracrine angiogenic signaling [4, 6]. In the models studied here, ciliary dysfunction is likely a key driver of cyst initiation, as mentioned above. However, most studies link ciliary dysfunction with hyperproliferation and altered fluid secretion, for instance via perturbed Ca^2+^ and cAMP levels. Our data do not show biliary cell hyperproliferation and the histology of the cysts do not show evidence for fluid-induced tension. Still, further work is required to determine whether Ca^2+^ and cAMP levels, and fluid secretion might regulate cholangiocyte differentiation.

## Conclusions

Our data indicate that initiation of liver cyst formation does not necessarily depend on overproliferation of cyst-lining cells at fetal stages. Since overproliferation occurs postnatally, cyst formation thus appears to be a two-step mechanism, most likely initiated by excessive differentiation of biliary precursors *in utero* followed by proliferative growth after birth. Therefore, in addition to controlling the proliferative growth of pre-established cysts, efforts aiming at preventing the excessive differentiation that causes biliary cyst formation may provide therapeutic benefits.

## Supporting Information

S1 Table List of antibodies used in this study. (PDF)

S2 Table Microarray data comparing liver RNA from E18.5 wild-type and *Cpk* embryos. (PDF)

S3 Table Apical junction gene set enriched in E18.5 *Cpk* embryos as compared to wild-type embryos. (PDF)
