# Supplementary material for: Proliferation-Independent Initiation of Biliary Cysts in Polycystic Liver Diseases
Source: PLoS One. 2015 Jun 30;10(6):e0132295. doi: 10.1371/journal.pone.0132295 (PMC4488361; doi:10.1371/journal.pone.0132295)
Supplement: S1 Table — (PDF) [file pone.0132295.s001.pdf]

Supplemental Table 1: List of antibodies used in this study

Primary antibody	Species/Isotype	Dilution	Manufacturer	Catalog number
HNF4	mouse IgG2a	1/300	R&D Systems	PP-H1415-OO
Ecad	mouse	1/250	BD Biosciences	610182
Sox9	rabbit	1/500	Chemicon	AB5535
Muc1	hamster	1/500	Neomarkers	HM-1630-P
Ki67	mouse IgG1	1/250	BD Biosciences	556003
Brdu	mouse IgG1	1/20	DSHB	G3G4
P.H3	rabbit	1/100	Cell Signaling	9701
aSMA	mouse IgG2a	1/50	Dako	M0851
bCat	mouse IgG1	1/1000	BD Biosciences	610154
YFP	goat	1/250	Abcam	ab6673

Secondary antibody	Dilution	Manufacturer	Catalog number
donkey anti-mouse Alexa Fluor 647	1/1000	Invitrogen	A31571
goat anti-mouse IgG2a Alexa Fluor 488	1/1000	Invitrogen	A21131
goat anti-mouse IgG2a Alexa Fluor 594	1/1000	Invitrogen	A21135
goat anti-mouse IgG2a Alexa Fluor 647	1/1000	Invitrogen	A21241
goat anti-mouse IgG1 Alexa Fluor 594	1/1000	Invitrogen	A21125
donkey anti-rabbit Alexa Fluor 488	1/1000	Invitrogen	A21206
donkey anti-rabbit Alexa Fluor 647	1/1000	Invitrogen	A31573
donkey anti-goat Alexa Fluor 594	1/1000	Invitrogen	A11058
biotinylated anti-hamster	1/500	Jackson ImmunoResearch	127-065-160
streptavidin Alexa Fluor 488	1/1000	Invitrogen	S11223
