# Supplementary material for: Proliferation-Independent Initiation of Biliary Cysts in Polycystic Liver Diseases
Source: PLoS One. 2015 Jun 30;10(6):e0132295. doi: 10.1371/journal.pone.0132295 (PMC4488361; doi:10.1371/journal.pone.0132295)
Supplement: S3 Table — (PDF) [file pone.0132295.s003.pdf]

NAME	PROBE	GENE SYMBOL	GENE_TITLE	RANK IN GENE LIST	RANK METRIC SCORE	RUNNING ES	CORE ENRICHMENT
row_0	ACTN2	ACTN2	actinin, alpha 2	14	0.26711270213127136	0.03381263	Yes
row_1	ACTA1	ACTA1	actin, alpha 1, skel	18	0.25375592708587646	0.06655846	Yes
row_2	ACTC1	ACTC1	actin, alpha, cardia	31	0.22748596966266632	0.09535025	Yes
row_3	CDH6	CDH6	cadherin 6, type 2,	43	0.21503448486328125	0.12258691	Yes
row_4	CLDN6	CLDN6	claudin 6	120	0.16846275329589844	0.13984267	Yes
row_5	ADAM23	ADAM23	ADAM metallopep	217	0.1439531296491623	0.15270643	Yes
row_6	DMP1	DMP1	dentin matrix acid	218	0.1437109112739563	0.17135447	Yes
row_7	KRT31	KRT31	keratin 31	255	0.13610656559467316	0.18683487	Yes
row_8	MPZL1	MPZL1	myelin protein zer	448	0.11123429238796234	0.18963729	Yes
row_9	CDH3	CDH3	cadherin 3, type 1,	531	0.10421505570411682	0.19819273	Yes
row_10	CLDN7	CLDN7	claudin 7	548	0.10297323763370514	0.21058533	Yes
row_11	CDSN	CDSN	corneodesmosin	551	0.10292921215295792	0.22382034	Yes
row_12	THY1	THY1	Thy-1 cell surface a	566	0.10212336480617523	0.23622383	Yes
row_13	IRS1	IRS1	insulin receptor su	615	0.09851538389921188	0.24609941	Yes
row_14	ITGA9	ITGA9	integrin, alpha 9	766	0.09089526534080505	0.248807	Yes
row_15	LAMC2	LAMC2	laminin, gamma 2	808	0.08918736129999161	0.2578962	Yes
row_16	BAIAP2	BAIAP2	BAI1-associated pr	836	0.08802057057619095	0.26768216	Yes
row_17	CDH11	CDH11	cadherin 11, type 2	859	0.08699466288089752	0.2776379	Yes
row_18	STX4	STX4	syntaxin 4	943	0.08343082666397095	0.2834358	Yes
row_19	NRAP	NRAP	nebulin-related an	985	0.08153420686721802	0.29153192	Yes
row_20	NLGN2	NLGN2	neuroligin 2	1355	0.06978347897529602	0.27823293	Yes
row_21	MMP2	MMP2	matrix metallopep	1521	0.06582680344581604	0.2767789	Yes
row_22	LAMA3	LAMA3	laminin, alpha 3	1572	0.06459055095911026	0.28213122	Yes
row_23	PARD6G	PARD6G	par-6 partitioning	1631	0.06351348757743835	0.28685912	Yes
row_24	ATP1A3	ATP1A3	ATPase, Na+/K+ tr	1642	0.06341354548931122	0.2944819	Yes
row_25	ITGA2	ITGA2	integrin, alpha 2 (C	1904	0.05759163573384285	0.28614357	Yes
row_26	RRAS	RRAS	related RAS viral (r	2013	0.05552288889884949	0.28680557	Yes
row_27	LAMB3	LAMB3	laminin, beta 3	2086	0.05450043827295303	0.28951582	Yes
row_28	SYMPK	SYMPK	symplekin	2113	0.05400937423110008	0.29494902	Yes
row_29	CTNND1	CTNND1	catenin (cadherin-	2139	0.053675029426813126	0.30039942	Yes
row_30	FBN1	FBN1	fibrillin 1	2153	0.05340554192662239	0.30654183	Yes
row_31	SORBS3	SORBS3	sorbin and SH3 do	2216	0.05241628736257553	0.30958742	Yes
row_32	ADAMTS5	ADAMTS5	ADAM metallopep	2313	0.051168348640203476	0.31041136	Yes
row_33	CLDN8	CLDN8	claudin 8	2349	0.05050664395093918	0.31484482	Yes
row_34	AMH	AMH	anti-Mullerian hor	2480	0.04861927404999733	0.31327826	Yes
row_35	CDH1	CDH1	cadherin 1, type 1,	2483	0.04859618842601776	0.31946298	Yes
row_36	PKD1	PKD1	polycystic kidney c	2558	0.04756420850753784	0.321152	Yes
row_37	CDH8	CDH8	cadherin 8, type 2	2589	0.047227855771780014	0.3254629	Yes
row_38	CLDN4	CLDN4	claudin 4	2595	0.04712264984846115	0.3312747	Yes
row_39	IKBKG	IKBKG	inhibitor of kappa	2648	0.04660945385694504	0.33417258	Yes
row_40	ARHGEF6	ARHGEF6	Rac/Cdc42 guanin	2727	0.04555357247591019	0.33535838	Yes
row_41	PTPRC	PTPRC	protein tyrosine pl	2778	0.04484875127673149	0.33814898	Yes
row_42	SLIT2	SLIT2	slit homolog 2 (Dr	2818	0.044225409626960754	0.34152505	Yes
row_43	SRC	SRC	v-src sarcoma (Sch	3072	0.041022252291440964	0.3315213	Yes
row_44	TJP1	TJP1	tight junction prot	3144	0.04025885462760925	0.33244413	Yes
row_45	PDZD3	PDZD3	PDZ domain conta	3254	0.03892922401428223	0.33089235	Yes
row_46	GNAI2	GNAI2	guanine nucleotide	3386	0.037437111139297485	0.3278142	Yes
row_47	MYH10	MYH10	myosin, heavy cha	3398	0.037328943610191345	0.33199164	Yes
row_48	PLCG1	PLCG1	phospholipase C, g	3546	0.03582628443837166	0.3277352	Yes
row_49	CERCAM	null	null	3577	0.03553472086787224	0.33052877	Yes
row_50	ICAM5	ICAM5	intercellular adhes	3670	0.03481919318437576	0.32947356	Yes
row_51	CTNNA1	CTNNA1	catenin (cadherin-	3708	0.034388329833745956	0.33169433	Yes
row_52	VAV2	VAV2	vav 2 oncogene	3713	0.034321241080760956	0.33590555	Yes
row_53	CDH15	CDH15	cadherin 15, M-ca	3735	0.03417474776506424	0.3390679	Yes
row_54	GRB7	GRB7	growth factor rece	3869	0.032969579100608826	0.33528888	Yes
row_55	VWF	VWF	von Willebrand fac	3877	0.03282499313354492	0.33912423	Yes
row_56	CLDN19	CLDN19	claudin 19	3948	0.03211452066898346	0.3390508	Yes
row_57	COL17A1	COL17A1	collagen, type XVII	3970	0.031971294432878494	0.34192723	Yes
row_58	GAMT	GAMT	guanidinoacetate	4007	0.031581830233335495	0.34384444	Yes
row_59	MAP4K2	MAP4K2	mitogen-activated	4022	0.031447071582078934	0.3470769	Yes
row_60	AKT3	AKT3	v-akt murine thym	4049	0.031169315800070763	0.34954637	Yes
row_61	PIK3R3	PIK3R3	phosphoinositide-	4381	0.028484424576163292	0.33319044	No
row_62	ALOX15B	ALOX15B	arachidonate 15-li	4446	0.027888525277376175	0.3329321	No
row_63	EVL	EVL	Enah/Vasp-like	4461	0.027803312987089157	0.33569178	No
row_64	PCDH1	PCDH1	protocadherin 1 (c	4496	0.027591347694396973	0.33721232	No
row_65	CD276	CD276	CD276 molecule	4498	0.027588864788413048	0.34073168	No
row_66	TRO	TRO	trophinin	4575	0.027000075206160545	0.33963114	No
row_67	VASP	VASP	vasodilator-stimul	4584	0.026935797184705734	0.3426417	No
row_68	SPEG	SPEG	SPEG complex locu	4617	0.02654712274670601	0.34414792	No
row_69	SDC3	SDC3	syndecan 3 (N-syn	4699	0.02587352693080902	0.34259826	No
row_70	SHROOM2	SHROOM2	shroom family me	4780	0.025398878380656242	0.3410476	No
row_71	MYH9	MYH9	myosin, heavy cha	4822	0.025073468685150146	0.34181738	No
row_72	AMIGO2	AMIGO2	adhesion molecule	4826	0.025048598647117615	0.34488598	No
row_73	CDH4	CDH4	cadherin 4, type 1,	5057	0.023211216554045677	0.33396438	No
row_74	SGCE	SGCE	sarcoglycan, epsilo	5066	0.02313755825161934	0.33648208	No

row_75	MSN	MSN	moesin	5161	0.022545630112290382	0.33371308	No
row_76	ACTG1	ACTG1	actin, gamma 1	5255	0.02184957265853882	0.33091432	No
row_77	EPB41L2	EPB41L2	erythrocyte memb	5334	0.021164024248719215	0.32893533	No
row_78	WASL	WASL	Wiskott-Aldrich sy	5347	0.02104313299059868	0.3309389	No
row_79	VCAN	null	null	5452	0.020276620984077454	0.32726967	No
row_80	PARVA	PARVA	parvin, alpha	5635	0.018984748050570488	0.31870753	No
row_81	ADAM15	ADAM15	ADAM metallopep	5664	0.01870023086667061	0.31943783	No
row_82	FYB	FYB	FYN binding prote	5731	0.018305890262126923	0.31781492	No
row_83	CLDN9	CLDN9	claudin 9	5751	0.01812940649688244	0.31901637	No
row_84	CADM2	null	null	5760	0.01806326024234295	0.32087564	No
row_85	CLDN5	CLDN5	claudin 5 (transme	5772	0.0179949589073658	0.32254428	No
row_86	ACTG2	ACTG2	actin, gamma 2, sr	5840	0.017471861094236374	0.32075256	No
row_87	NEXN	NEXN	nexilin (F actin bin	5879	0.017107892781496048	0.32067046	No
row_88	PPP2R2C	PPP2R2C	protein phosphata	5954	0.016642002388834953	0.31834698	No
row_89	INPPL1	INPPL1	inositol polyphosp	5989	0.016450630500912666	0.3184219	No
row_90	NLGN3	NLGN3	neuroligin 3	6201	0.015151012688875198	0.30760545	No
row_91	CAP1	CAP1	CAP, adenylate cy	6572	0.012627212330698967	0.28682923	No
row_92	FSCN1	FSCN1	fascin homolog 1,	6853	0.010892949067056179	0.2712802	No
row_93	PTK2	PTK2	PTK2 protein tyros	6878	0.010760599747300148	0.2712226	No
row_94	RAC2	RAC2	ras-related C3 bot	6916	0.010544607415795326	0.27034938	No
row_95	EXOC4	EXOC4	exocyst complex c	6964	0.010272497311234474	0.26883507	No
row_96	NF1	NF1	neurofibromin 1 (	7009	0.009988432750105858	0.26746565	No
row_97	DHX16	DHX16	DEAH (Asp-Glu-Ala	7159	0.00888897106051445	0.25959262	No
row_98	ACTB	ACTB	actin, beta	7160	0.00888433400541544	0.26074544	No
row_99	DSC1	DSC1	desmocollin 1	7175	0.008716538548469543	0.26102838	No
row_100	NF2	NF2	neurofibromin 2 (	7193	0.008609936572611332	0.26111576	No
row_101	CLDN15	CLDN15	claudin 15	7205	0.008549223653972149	0.2615587	No
row_102	TSC1	TSC1	tuberous sclerosis	7258	0.008318696171045303	0.259488	No
row_103	MAPK13	MAPK13	mitogen-activated	7348	0.007725995499640703	0.25509888	No
row_104	PVRL4	PVRL4	poliovirus recepto	7361	0.007651152554899454	0.25536472	No
row_105	TMEM8B	null	null	7384	0.007519801147282123	0.2550077	No
row_106	MPP5	MPP5	membrane protei	7486	0.006916715297847986	0.24978663	No
row_107	VCL	VCL	vinculin	7580	0.006409883499145508	0.2449844	No
row_108	ACTN3	ACTN3	actinin, alpha 3	7780	0.005330526269972324	0.23362061	No
row_109	TUBG1	TUBG1	tubulin, gamma 1	7859	0.004861128982156515	0.22952613	No
row_110	TAOK2	TAOK2	TAO kinase 2	7930	0.004489051643759012	0.225868	No
row_111	MMP9	MMP9	matrix metallopep	7988	0.004110051784664392	0.22294824	No
row_112	PFN1	PFN1	profilin 1	8026	0.003897694637998938	0.22121254	No
row_113	PVRL2	PVRL2	poliovirus recepto	8073	0.003687557764351368	0.21890435	No
row_114	ARPC2	ARPC2	actin related prote	8125	0.003396095475181937	0.21625543	No
row_115	CD34	CD34	CD34 molecule	8132	0.003365738783031702	0.21632868	No
row_116	DLG1	DLG1	discs, large homol	8158	0.003146451199427247	0.21522246	No
row_117	WNK4	WNK4	WNK lysine deficie	8252	0.002555466722697019	0.2099201	No
row_118	FLNC	FLNC	filamin C, gamma	8307	0.00231961440294981	0.20694974	No
row_119	MYL12B	null	null	8359	0.001947141601704061	0.20411281	No
row_120	ADRA1B	ADRA1B	adrenergic, alpha-	8371	0.001867240760475397	0.20368871	No
row_121	CX3CL1	CX3CL1	chemokine (C-X3-	8374	0.001861267141066491	0.20380908	No
row_122	CLDN11	CLDN11	claudin 11 (oligod	8399	0.001726338407024741	0.20257916	No
row_123	MDK	MDK	midkine (neurite g	8467	0.001304792007431388	0.19868958	No
row_124	ACTN4	ACTN4	actinin, alpha 4	8472	0.001263816608116030	0.19861126	No
row_125	GTF2F1	GTF2F1	general transcripti	8560	7,56E+11	0.19343892	No
row_126	ITGB1	ITGB1	integrin, beta 1 (fi	8607	4,57E+12	0.19071147	No
row_127	HADH	HADH	hydroxyacyl-Coenz	8614	4,16E+11	0.19040197	No
row_128	ZYX	ZYX	zyxin	8640	2,85E+11	0.18892448	No
row_129	GNAI1	GNAI1	guanine nucleotid	8709	-7,40E+09	0.18481462	No
row_130	TRAF1	TRAF1	TNF receptor-asso	8952	-0.00149780663195997	0.17034853	No
row_131	ICAM2	ICAM2	intercellular adhes	8967	-0.00155828625429421	0.1697026	No
row_132	SYK	SYK	spleen tyrosine kir	9207	-0.00288836192339658	0.15559869	No
row_133	LAYN	LAYN	layilin	9253	-0.00315565872006118	0.15328205	No
row_134	ACTN1	ACTN1	actinin, alpha 1	9255	-0.00318475067615509	0.15363473	No
row_135	COL16A1	COL16A1	collagen, type XVI,	9262	-0.00322184152901172	0.15368931	No
row_136	TSPAN4	TSPAN4	tetraspanin 4	9521	-0.00480336649343371	0.13868287	No
row_137	CD209	CD209	CD209 molecule	9560	-0.00502900639548897	0.13703339	No
row_138	SLC30A3	SLC30A3	solute carrier fami	9752	-0.00612033763900399	0.12625672	No
row_139	SIRPA	SIRPA	signal-regulatory	9759	-0.00617086142301559	0.12669396	No
row_140	MPZL2	null	null	9793	-0.00637040752917528	0.12552145	No
row_141	INSIG1	INSIG1	insulin induced ge	10030	-0.00792972650378942	0.11225345	No
row_142	YWHAH	YWHAH	tyrosine 3-monoo	10058	-0.00813588127493858	0.1116735	No
row_143	AMIGO1	AMIGO1	adhesion molecule	10086	-0.00832285452634096	0.11111781	No
row_144	RSU1	RSU1	Ras suppressor pr	10248	-0.00919717922806739	0.1025578	No
row_145	CDK8	CDK8	cyclin-dependent	10252	-0.00924716982990503	0.10357598	No
row_146	PTEN	PTEN	phosphatase and	10668	-0.01171608269214630	0.07995542	No
row_147	PVRL1	PVRL1	poliovirus recepto	10701	-0.01190018374472856	0.079561025	No
row_148	VCAM1	VCAM1	vascular cell adhes	10777	-0.01234873477369546	0.07661988	No
row_149	MYL9	MYL9	myosin, light chair	10791	-0.01243281457573175	0.077445626	No
row_150	HRAS	HRAS	v-Ha-ras Harvey ra	10821	-0.01266385149210691	0.077332065	No

row_151	MAPK14	MAPK14	mitogen-activated	10992	-0.013714555650949478	0.06881302	No
row_152	CD86	CD86	CD86 molecule	11016	-0.01387321203947067:	0.069219865	No
row_153	EGFR	EGFR	epidermal growth	11069	-0.0143082831054925 9	0.06792635	No
row_154	ADAM9	ADAM9	ADAM metallopep	11122	-0.0145917572081089 0	0.066669606	No
row_155	MADCAM1	MADCAM1	mucosal vascular a	11497	-0.0173923019319772 7	0.046269387	No
row_156	THBS3	THBS3	thrombospondin 3	11624	-0.0184043142944574 3	0.04102442	No
row_157	ITGB4	ITGB4	integrin, beta 4	11630	-0.0184619426727294 9	0.043117154	No
row_158	CNTN1	CNTN1	contactin 1	11644	-0.0185779202729463 5	0.044740293	No
row_159	AKT2	AKT2	v-akt murine thym	11835	-0.01998194120824337	0.0358229	No
row_160	JAM3	JAM3	junctional adhesio	11959	-0.0209232307970523 8	0.031086529	No
row_161	CNN2	CNN2	calponin 2	11996	-0.02122393064200878	0.03165967	No
row_162	ITGA3	ITGA3	integrin, alpha 3 (a	12049	-0.0216756276786327 3	0.031322137	No
row_163	DSC3	DSC3	desmocollin 3	12506	-0.02535995841026306	0.0069882213	No
row_164	KCNH2	KCNH2	potassium voltage	12511	-0.02539883181452751	0.010041673	No
row_165	PECAM1	PECAM1	platelet/endotheli	12526	-0.02551436796784401	0.012504312	No
row_166	SHC1	SHC1	SHC (Src homology	12579	-0.0259580630809068 6	0.012722472	No
row_167	PIK3CB	PIK3CB	phosphoinositide-	12645	-0.0264928713440895 0	0.012222485	No
row_168	SKAP2	SKAP2	src kinase associat	13033	-0.0299303680658340 4	-0.007338327	No
row_169	LDLRAP1	LDLRAP1	low density lipopr	13043	-0.03003254532814026	-0.0039865035	No
row_170	NFASC	NFASC	neurofascin homo	13228	-0.03172461315989494	-0.011016679	No
row_171	CRAT	CRAT	carnitine acetyltra	13258	-0.03196200728416443	-0.008626094	No
row_172	NRXN2	NRXN2	neurexin 2	13383	-0.0330627821385860 4	-0.011847805	No
row_173	CLDN14	CLDN14	claudin 14	13394	-0.03316264972090721	-0.008150398	No
row_174	PVRL3	PVRL3	poliovirus recepto	13417	-0.03338585048913956	-0.005150991	No
row_175	PBX2	PBX2	pre-B-cell leukemi	13462	-0.03374621644616127	-0.003437591	No
row_176	RASA1	RASA1	RAS p21 protein a	13589	-0.03522463142871857	-0.0064999405	No
row_177	B4GALT1	B4GALT1	UDP-Gal:betaGlcN	13709	-0.03654562681913376	-0.008966814	No
row_178	LIMA1	LIMA1	LIM domain and a	13811	-0.03805079683661461	-0.010147929	No
row_179	NEGR1	NEGR1	neuronal growth r	14169	-0.0425711646676063 5	-0.02625105	No
row_180	JUP	JUP	junction plakoglob	14181	-0.04279167577624321	-0.021364752	No
row_181	CRB3	CRB3	crumbs homolog 3	14223	-0.04340951144695282	-0.018215695	No
row_182	BMP1	BMP1	bone morphogene	14273	-0.04411891847848892	-0.015459226	No
row_183	ZAK	ZAK	-	14664	-0.0503553748130798 3	-0.032551415	No
row_184	CALB2	CALB2	calbindin 2, 29kDa	14716	-0.05101010575890541	-0.0290219	No
row_185	TIAL1	TIAL1	TIA1 cytotoxic gra	14944	-0.0555643327534198 7	-0.035563573	No
row_186	CADM3	null	null	14950	-0.0556875914335250 8	-0.028640408	No
row_187	COL9A1	COL9A1	collagen, type IX, a	15047	-0.05762537196278572	-0.026978608	No
row_188	CD274	CD274	CD274 molecule	15153	-0.06025306507945061	-0.02552106	No
row_189	ICAM1	ICAM1	intercellular adhes	15234	-0.06221110001206398	-0.022294927	No
row_190	TGFBI	TGFBI	transforming grow	15445	-0.0675186961889267	-0.026255524	No
row_191	MVD	MVD	mevalonate (diphc	15806	-0.08156796544790268	-0.03748013	No
row_192	CLDN18	CLDN18	claudin 18	16018	-0.09117062389850616	-0.038432214	No
row_193	TNFRSF11B	TNFRSF11B	tumor necrosis fac	16311	-0.11351769417524338	-0.041391533	No
row_194	RHOF	RHOF	ras homolog gene	16325	-0.1158490926027298	-0.027146408	No
row_195	MAPK11	MAPK11	mitogen-activated	16351	-0.1180209144949913	-0.013346431	No
row_196	ICAM4	ICAM4	intercellular adhes	16361	-0.11986483633518219	0.001662102	No
row_197	NRTN	NRTN	neurturin	16511	-0.14685648679733276	0.011691842	No
