# Supplementary material for: Proliferation-Independent Initiation of Biliary Cysts in Polycystic Liver Diseases
Source: PLoS One. 2015 Jun 30;10(6):e0132295. doi: 10.1371/journal.pone.0132295 (PMC4488361; doi:10.1371/journal.pone.0132295)
Supplement: S2 Table — (PDF) [file pone.0132295.s002.pdf]

Transcript Cluster I	WT 1 (log2)	Wt 2 (log2	Wt 3 (log2	Mut 1 (log2)	Mut 2 (log2)	Mut 3 (log2)	Mean WT	Mean Mut	log FC	Fold Change	Gene Symbol
17387210	7,784241	7,881441	7,611751	5,291137	5,079281	5,661697	7,7591443	5,344038333	-2,415106	0,187491098	Cwc22
17549296	4,235503	10,17276	9,746921	4,108086	9,900191	3,355511	8,051728	5,787929333	-2,26379867	0,208222999	---
17372564	9,586961	9,599075	9,534802	7,446698	7,359002	7,542281	9,5736127	7,449327	-2,12428567	0,22936455	Cwc22
17372566	9,586961	9,599075	9,534802	7,446698	7,359002	7,542281	9,5736127	7,449327	-2,12428567	0,22936455	Cwc22
17372568	9,586961	9,599075	9,534802	7,446698	7,359002	7,542281	9,5736127	7,449327	-2,12428567	0,22936455	Cwc22
17372570	9,586961	9,599075	9,534802	7,446698	7,359002	7,542281	9,5736127	7,449327	-2,12428567	0,22936455	Cwc22
17372572	9,586961	9,599075	9,534802	7,446698	7,359002	7,542281	9,5736127	7,449327	-2,12428567	0,22936455	Cwc22
17372574	9,586961	9,599075	9,534802	7,446698	7,359002	7,542281	9,5736127	7,449327	-2,12428567	0,22936455	Cwc22
17372576	9,586961	9,599075	9,534802	7,446698	7,359002	7,542281	9,5736127	7,449327	-2,12428567	0,22936455	Cwc22
17548055	5,800866	11,80124	5,429154	5,982451	5,27668	6,098981	7,6770867	5,786037333	-1,89104933	0,269610888	Gm4841 // Gm4841
17548738	8,991055	9,953093	9,036399	9,412313	6,274323	6,97011	9,326849	7,552248667	-1,77460033	0,292275269	---
17548740	8,991055	9,953093	9,036399	9,412313	6,274323	6,97011	9,326849	7,552248667	-1,77460033	0,292275269	LOC100503226
17548742	8,991055	9,953093	9,036399	9,412313	6,274323	6,97011	9,326849	7,552248667	-1,77460033	0,292275269	LOC100503226
17231174	7,40343	8,38357	8,295452	7,35981	5,611552	6,117855	8,027484	6,363072333	-1,66441167	0,315472976	Syt14
17270117	6,264108	4,641033	6,129892	4,443655	3,499334	4,103771	5,6783443	4,015586667	-1,66275767	0,315834863	---
17546316	3,956707	8,844461	8,620378	3,859521	8,595602	4,271415	7,1405153	5,575512667	-1,56500267	0,337977084	Eif2s3y
17285521	5,105148	7,673165	6,461238	6,442901	4,117887	4,126011	6,4131837	4,895599667	-1,517584	0,34927033	---
17350932	5,560041	9,860603	4,956504	5,42364	5,009104	5,435662	6,7923827	5,289468667	-1,502914	0,352839993	BC023105
17391784	7,065943	7,070832	6,71022	5,599654	5,161558	5,637124	6,9489983	5,466112	-1,48288633	0,357772317	A730017L22Rik // A730017L22Rik
17475385	9,239648	9,284535	9,572921	9,490564	6,849339	7,318446	9,3657013	7,886116333	-1,479585	0,358591948	Cyp2b9
17231186	5,547811	6,40684	6,685794	5,384283	4,561631	4,25879	6,2134817	4,734901333	-1,47858033	0,358841752	---
17201491	6,285836	6,366714	6,019597	6,248388	3,553077	4,449352	6,224049	4,750272333	-1,47377667	0,36003856	---
17318815	6,9218	8,64792	6,960404	6,584286	5,836019	5,765717	7,5100413	6,062007333	-1,448034	0,366520551	Apol11b
17203943	6,29518	5,443325	6,403235	4,457116	4,122057	5,223727	6,0472467	4,600966667	-1,44628	0,366966431	---
17546834	5,084801	9,190183	8,903146	4,815366	9,099625	5,052479	7,7260433	6,32249	-1,40355333	0,377996994	Ddx3y
17248348	6,412095	5,825885	6,425203	4,921083	5,111623	4,545745	6,221061	4,859483667	-1,36157733	0,389156583	---
17428878	6,00474	5,026613	5,199988	3,081503	4,691483	4,381765	5,410447	4,051583667	-1,35886333	0,389889354	---
17209159	6,944724	6,508142	5,872196	5,758152	3,094266	6,398411	6,4416873	5,083609667	-1,35807767	0,390101739	---
17546797	4,011665	8,210645	8,049984	3,844165	8,417809	3,953659	6,7574313	5,405211	-1,35222033	0,391688769	Uty
17245890	9,146396	10,01198	10,20394	8,676801	8,157782	8,522139	9,7874387	8,452240667	-1,335198	0,39633767	Inhbe
17210639	4,85029	3,535062	4,165556	2,906405	2,900901	2,748616	4,183636	2,851974	-1,331662	0,397310272	---
17206379	4,951212	4,841394	4,836087	3,120542	3,815553	3,733761	4,876231	3,556618667	-1,31961233	0,400642581	---
17207801	6,355254	5,103727	5,940931	4,318244	4,000248	5,188844	5,7999707	4,502445333	-1,29752533	0,406823428	---
17285523	7,808598	10,19588	9,353825	9,029349	7,170012	7,267439	9,1194343	7,822266667	-1,29716767	0,406924298	---
17448780	6,712254	8,25198	7,80964	5,809862	5,924267	7,19004	7,5912913	6,308056333	-1,283235	0,410873161	---
17290928	6,304203	6,858252	6,944724	7,203154	3,794019	5,276218	6,702393	5,424463667	-1,27792933	0,412386974	---
17209059	5,33836	5,365572	5,300767	4,132059	3,886679	4,21364	5,3348997	4,077459333	-1,25744033	0,418285435	---
17513149	7,722065	5,838178	8,395429	6,167607	5,946561	6,109657	7,3185573	6,074608333	-1,243949	0,422215369	Ctrb1
17475070	5,347478	5,778196	5,259404	5,212085	3,692528	3,774371	5,4616927	4,226328	-1,23536467	0,424735127	BC049730
17404350	4,899531	3,387565	5,6826	3,40836	3,403327	3,505629	4,6565653	3,439105333	-1,21746	0,430039176	Cpb1
17322075	4,711737	4,881794	8,308611	4,845667	4,750136	4,661388	5,9673807	4,752397	-1,21498367	0,430777957	Krt4
17384447	9,404123	9,288532	8,932772	9,205847	5,403666	9,389499	9,2084757	7,999670667	-1,208805	0,432626817	---
17202969	6,64897	4,426515	4,752242	4,129992	3,694432	4,426515	5,275909	4,083646333	-1,19226267	0,437615982	---
17475218	7,747221	8,050874	8,173369	6,866518	5,58268	7,968792	7,990488	6,805996667	-1,18449133	0,439979639	---
17546666	4,404106	6,761608	6,399405	3,889007	6,451023	3,791937	5,8550397	4,710655667	-1,144384	0,452382805	---
17200707	5,12595	5,850974	5,747217	4,916395	4,045261	4,395241	5,5747137	4,452299	-1,12241467	0,459324401	---
17546287	3,765132	7,401871	7,216651	3,77528	7,575977	3,670953	6,1278847	5,007403333	-1,12048133	0,459940348	Kdm5d
17547156	4,835454	7,192956	6,66672	4,317955	6,727709	4,330164	6,23171	5,125276	-1,106434	0,4644406	Srsy
17546647	4,658806	7,212288	6,893659	4,285067	6,913514	4,248201	6,2549177	5,148927333	-1,10599033	0,46458345	---
17496345	7,482792	7,800019	7,877153	6,803331	6,59686	6,443253	7,719988	6,614481333	-1,10550667	0,464739229	Nupr1
17208275	4,757235	3,969094	5,530822	3,680513	4,061434	3,275583	4,7523837	3,67251	-1,07987367	0,473070247	---
17203357	7,235661	7,208937	7,309497	6,368595	6,035913	6,11474	7,251365	6,173082667	-1,07828233	0,473592345	---
17284469	4,938732	4,59831	3,419823	3,292939	3,346907	3,131567	4,318955	3,257137667	-1,06181733	0,479028257	---
17546586	4,741688	7,190568	6,91746	4,29174	7,137506	4,248201	6,2832387	5,225815667	-1,057423	0,480489563	---
17203333	5,134701	5,435645	4,982416	3,900705	4,338725	4,167576	5,184254	4,135668667	-1,04858533	0,483441982	---
17445565	8,404526	8,028464	7,633153	6,436426	7,447803	7,099182	8,0220477	6,994470333	-1,02757733	0,490533193	---
17546255	3,592448	5,441312	4,541817	3,380584	4,14485	2,980704	4,5251923	3,502046	-1,02314633	0,4920421	LOC100862038 // LOC100862038
17547281	4,812349	7,888718	7,203522	4,746493	7,517185	4,575768	6,634863	5,613148667	-1,02171433	0,492530737	LOC100041256 // LOC100041256
17206603	5,374272	4,550835	4,84743	4,165818	3,327113	4,217631	4,924179	3,903520667	-1,02065833	0,492891384	---
17547039	3,788274	6,133003	5,31458	3,456564	5,117899	3,604631	5,078619	4,059698	-1,018921	0,493485295	Sly // Sly
17547044	3,788274	6,133003	5,31458	3,456564	5,117899	3,604631	5,078619	4,059698	-1,018921	0,493485295	Sly // Sly
17547049	3,788274	6,133003	5,31458	3,456564	5,117899	3,604631	5,078619	4,059698	-1,018921	0,493485295	Sly // Sly
17547054	3,788274	6,133003	5,31458	3,456564	5,117899	3,604631	5,078619	4,059698	-1,018921	0,493485295	Sly // Sly
17219474	7,822444	9,297348	9,139181	7,455506	8,053592	7,715539	8,752991	7,741545667	-1,01144533	0,496049043	---
17274394	5,501451	6,285979	5,79319	4,780788	4,993148	4,780232	5,8602067	4,851389333	-1,00881733	0,496953464	Gm10330
17546467	4,14892	7,293936	6,502848	4,106634	6,810344	4,006107	5,9819013	4,974361667	-1,00753967	0,497393767	LOC434960 // LOC434960
17547464	4,16263	7,0774	6,442226	3,991422	6,76471	3,961605	5,8940853	4,905912333	-0,988173	0,504115773	LOC100041256 // LOC100041256
17310237	9,417042	9,299064	8,536732	8,160937	7,999187	8,143525	9,0842793	8,101216333	-0,983063	0,505904508	Ugt3a2
17205199	5,133227	5,074743	5,327167	4,684922	4,000446	3,90244	5,178379	4,195936	-0,982443	0,506121968	---
17209389	5,133227	5,074743	5,327167	4,684922	4,000446	3,90244	5,178379	4,195936	-0,982443	0,506121968	---
17548781	8,232029	8,692688	8,447721	7,446393	7,416485	7,576782	8,4574793	7,479886667	-0,97759267	0,507826411	Ywhaq // Ywhaq
17204765	4,545767	5,776639	5,412296	3,707109	4,62743	4,495965	5,2449007	4,276834667	-0,968066	0,511190879	---
17201657	5,572714	5,944931	3,927872	4,256627	4,167733	4,121517	5,1485057	4,181959	-0,96654667	0,511729509	---
17220755	6,285836	5,071432	5,974602	5,884357	3,553077	5,021388	5,77729	4,819607333	-0,95768267	0,514883282	---
17208067	4,387951	4,335142	4,726285	3,463729	3,845428	3,289047	4,483126	3,532734667	-0,95039133	0,517492072	---
17504601	11,01463	11,19681	11,22051	10,82464	9,52327	10,2498	11,143983	10,19923667	-0,94474667	0,51952077	Ces3a
17491193	8,895329	8,165276	7,168362	6,907793	7,139461	7,361514	8,0763223	7,136256	-0,94006633	0,521208915	Saa3
17490899	6,437825	7,225819	7,744981	6,443573	6,127667	6,045243	7,1362083	6,205494333	-0,930714	0,52459865	Fgf21
17205953	6,472093	6,425515	6,299912	6,125361	5,260089	5,022686	6,3991733	5,469378667	-0,92979467	0,524933048	---
17547229	4,71482	7,812593	7,140853	4,741022	7,635428	4,516254	6,5560887	5,630901333	-0,92518733	0,526612133	LOC100039614 // LOC100039614
17547443	4,71482	7,812593	7,140853	4,741022	7,635428	4,516254	6,5560887	5,630901333	-0,92518733	0,526612133	LOC100041256 // LOC100041256
17547450	4,71482	7,812593	7,140853	4,741022	7,635428	4,516254	6,5560887	5,630901333	-0,92518733	0,526612133	LOC100041256 // LOC100041256
17204283	4,267131	4,674734	4,984709	3,560316	3,48833	4,103771	4,6421913	3,717472333	-0,924719	0,526783112	---
17210489	4,255931	5,20091	4,611299	3,962576	3,094266	4,268632	4,68938	3,775158	-0,914222	0,530629946	---
17203145	5,568312	5,397368	5,486913	4,827169	4,090684	4,797938	5,4841977	4,571930333	-0,91226733	0,531349368	---
17393184	7,628788	7,77245	7,937093	6,707891	6,953764	6,942091	7,7794437	6,867915333	-0,91152833	0,531621614	Zfp341 // Zfp341
17462566	4,543962	7,009733	4,197292	4,984871	4,096458	3,938302	5,250329	4,339877	-0,910452	0,532018382	Mug2
17201371	4,870544	4,900139	5,213286	3,870322	4,344512	4,040766	4,9946563	4,0852	-0,90945633	0,532385678	---
17472385	5,927675	5,631138	6,541571	5,916665	4,778792	4,68122	6,0334613	5,125559	-0,90790233	0,532959447	Slco1a1
17547170	3,499335	7,272895	5,924268	3,261558	7,18967	3,533464	5,5654993	4,661564	-0,90393533	0,53442695	LOC380994 // LOC380994
17547333	3,499335	7,272895	5,924268	3,261558	7,18967	3,533464	5,5654993	4,661564	-0,90393533	0,53442695	LOC380994 // LOC380994
17207163	6,153277	5,35544	5,616086	4,934934	4,936789	4,545818	5,7082677	4,805847	-0,90242067	0,534988332	---
17547174	4,606083	7,28194	6,779145	4,57299	6,716908	4,671982	6,2223893	5,320626667	-0,90176267	0,535232391	Srsy
17546459	3,806185	7,299528	6,341484	4,245879	6,53815	3,976135	5,8157323	4,920054667	-0,89567767	0,537494662	---
17546461	4,408773	7,119131	6,516418	4,296305	7,087074	3,994833	6,014774	5,126070667	-0,88870333	0,540099331	LOC382133 // LOC382133
17546463	4,408773	7,119131	6,516418	4,296305	7,087074	3,994833	6,014774	5,126070667	-0,88870333	0,540099331	LOC382133 // LOC382133
17546465	4,408773	7,119131	6,516418	4,296305	7,087074	3,994833	6,014774	5,126070667	-0,88870333	0,540099331	LOC382133 // LOC382133
17204657	6,051011	4,936523	5,804813	4,940075	4,074475	5,14749	5,597449	4,72068	-0,876769	0,544585698	---
17546567	3,999115	6,481046	5,446589	3,316533	6,384085	3,600082	5,3089167	4,433566667	-0,87535	0,545121603	---
17546759	3,999115	6,481046	5,446589	3,316533	6,384085	3,600082	5,3089167	4,433566667	-0,87535	0,545121603	---
17546452	4,674409	7,725236	7,004985	4,671883	7,577065	4,54354	6,46821	5,597496	-0,870714	0,546876131	LOC100041256 // LOC100041256
17391444	10,31414	10,50451	10,34894	10,56019	7,211289	10,78819	10,389197	9,519889667	-0,869307	0,547409736	---
17202701	4,457385	4,596228	4,40694	3,614747	3,575785	3,670007	4,486851	3,620179667	-0,86667133	0,548410716	---
17396238	4,429435	3,95057	4,821748	3,552686	3,900761	3,160961	4,4005843	3,538136	-0,86244833	0,550018354	---
17359952	5,672859	7,007739	7,165126	4,943341	5,892473	6,426635	6,6152413	5,754149667	-0,86109167	0,550535818	---
17547351	4,653702	6,890249	5,796762	4,393915	6,159597	4,204927	5,7802377	4,919479667	-0,860758	0,55066316	---
17547378	4,653702	6,890249	5,796762	4,393915	6,159597	4,204927	5,7802377	4,919479667	-0,860758	0,55066316	---
17546592	3,297479	6,100185	5,069816	3,106604	5,714133	3,066084	4,8224933	3,962273667	-0,86021967	0,550868676	Sly
17546681	3,297479	6,100185	5,069816	3,106604	5,714133	3,066084	4,8224933	3,962273667	-0,86021967	0,550868676	Sly
17546690	3,297479	6,100185	5,069816	3,106604	5,714133	3,066084	4,8224933	3,962273667	-0,86021967	0,550868676	Sly
17546695	3,297479	6,100185	5,069816	3,106604	5,714133	3,066084	4,8224933	3,962273667	-0,86021967	0,550868676	Sly
17546702	3,297479	6,100185	5,069816	3,106604	5,714133	3,066084	4,8224933	3,962273667	-0,86021967	0,550868676	Sly // Sly
17546710	3,297479	6,100185	5,069816	3,106604	5,714133	3,066084	4,8224933	3,962273667	-0,86021967	0,550868676	Sly
17546719	3,297479	6,100185	5,069816	3,106604	5,714133	3,066084	4,8224933	3,962273667	-0,86021967	0,550868676	Sly
17547034	3,297479	6,100185	5,069816	3,106604	5,714133	3,066084	4,8224933	3,962273667	-0,86021967	0,550868676	Sly
17547071	3,297479	6,100185	5,069816	3,106604	5,714133	3,066084	4,8224933	3,962273667	-0,86021967	0,550868676	Sly
17547151	3,297479	6,100185	5,069816	3,106604	5,714133	3,066084	4,8224933	3,962273667	-0,86021967	0,550868676	Sly
17547158	3,297479	6,100185	5,069816	3,106604	5,714133	3,066084	4,8224933	3,962273667	-0,86021967	0,550868676	Sly
17546984	3,819956	6,603892	5,688056	3,808316	5,901648	3,829311	5,3706347	4,513091667	-0,857543	0,551891664	LOC100039753 // LOC100039753
17547456	3,430213	7,142767	6,072444	3,42825	6,957794	3,68891	5,5484747	4,691651333	-0,85682333	0,552167036	Ssty2 // Ssty2
17547353	3,904781	7,365297	6,44008	4,295897	6,742358	4,108459	5,903386	5,048904667	-0,85448133	0,553064124	---
17547383	3,904781	7,365297	6,44008	4,295897	6,742358	4,108459	5,903386	5,048904667	-0,85448133	0,553064124	---
17208657	4,255931	5,839297	5,276218	4,000248	3,226817	5,58268	5,1238153	4,269915	-0,85390033	0,553286898	---
17204755	6,168057	6,523769	6,899465	5,725948	5,263679	6,041483	6,5304303	5,677036667	-0,85339367	0,553481244	---
17202183	4,156521	5,127447	3,651848	3,535062	3,053003	3,788274	4,3119387	3,458779667	-0,853159	0,55357128	---
17547022	4,60723	7,300417	6,786286	4,580131	6,872087	4,690459	6,231311	5,380892333	-0,85041867	0,554623762	Srsy
17547030	4,60723	7,300417	6,786286	4,580131	6,872087	4,690459	6,231311	5,380892333	-0,85041867	0,554623762	Srsy
17201859	5,332694	5,661129	4,940925	4,375771	4,569787	4,456336	5,3115827	4,467298	-0,84428467	0,556986912	---
17210597	5,415656	5,600383	5,175145	4,855229	3,873588	4,948991	5,3970613	4,559269333	-0,837792	0,55949921	---
17475223	6,346176	6,739512	5,276218	4,478904	5,071432	6,30116	6,1206353	5,283832	-0,83680333	0,559882762	---
17207351	6,329433	6,715394	6,593207	5,247363	5,964538	5,91663	6,5460113	5,709510333	-0,836501	0,560000104	---
17232489	3,33795	4,219289	6,462747	4,218918	3,897973	3,402478	4,6733287	3,839789667	-0,833539	0,561151022	Sult3a1
17547231	3,523305	7,048563	5,905824	3,393527	6,899693	3,690264	5,492564	4,661161333	-0,83140267	0,561982587	LOC100039574

17544316	5,267457	7,191197	6,769214	5,321858	6,542293	4,870653	6,4092893	5,578268	-0,83102133	0,56213115	Astx // Astx // Astx
17546550	3,388394	6,133251	5,54091	3,028619	5,945688	3,60816	5,0208517	4,194155667	-0,826696	0,563819	LOC380994 // LOC380994
17546733	3,557591	6,526745	5,580652	3,286362	6,612576	3,286538	5,2216627	4,395158667	-0,826504	0,56389404	Sly // Sly
17546700	3,33702	5,941303	4,321185	2,840911	5,276218	3,005726	4,5331693	3,707618333	-0,825551	0,564266654	Sly
17546737	3,33702	5,941303	4,321185	2,840911	5,276218	3,005726	4,5331693	3,707618333	-0,825551	0,564266654	Sly // Sly
17546739	3,33702	5,941303	4,321185	2,840911	5,276218	3,005726	4,5331693	3,707618333	-0,825551	0,564266654	Sly
17547149	3,33702	5,941303	4,321185	2,840911	5,276218	3,005726	4,5331693	3,707618333	-0,825551	0,564266654	Sly // Sly
17546741	3,33702	5,941303	4,321185	2,840911	5,276218	3,005726	4,5331693	3,707618333	-0,825551	0,564266654	---
17204535	4,831812	5,312044	5,574833	4,271067	4,088634	4,884809	5,239563	4,414836667	-0,82472633	0,56458929	---
17204365	6,325342	5,900469	5,812974	5,672129	4,044016	5,849154	6,0129283	5,188433	-0,82449533	0,564679697	---
17547387	3,784174	7,075294	6,069888	4,125587	6,269927	4,063505	5,6431187	4,819673	-0,82344567	0,565090693	---
17546538	3,35464	6,452721	5,527612	3,067313	6,375113	3,425389	5,1116577	4,289271667	-0,822386	0,565505907	LOC380994 // LOC380994
17546541	3,35464	6,452721	5,527612	3,067313	6,375113	3,425389	5,1116577	4,289271667	-0,822386	0,565505907	LOC380994 // LOC380994
17547205	3,35464	6,452721	5,527612	3,067313	6,375113	3,425389	5,1116577	4,289271667	-0,822386	0,565505907	LOC380994 // LOC380994
17475498	10,38855	10,50738	10,30518	9,612389	9,295098	9,8269	10,40037	9,578129	-0,822241	0,565562747	Cyp2f2
17546989	3,477252	6,249974	5,285896	3,524823	5,526796	3,498688	5,004374	4,183435667	-0,82093833	0,566073647	LOC100039753 // LOC100039753
17200453	4,860666	4,191156	5,316798	3,767399	4,083385	4,06878	4,78954	3,973188	-0,816352	0,567876061	---
17365098	9,513264	9,740421	9,101692	8,68905	7,865733	9,355438	9,4517923	8,636740333	-0,815052	0,568387999	Scd1
17203543	6,582784	5,435662	4,308971	6,173804	4,936306	2,779487	5,4424723	4,629865667	-0,81260667	0,56935222	---
17547723	8,374012	8,787478	8,635737	7,73604	7,781415	7,846176	8,5990757	7,787877	-0,81119867	0,569908152	Ywhaq // Ywhaq
17547026	4,983136	7,481906	6,956645	4,969537	6,971817	5,046755	6,4738957	5,662703	-0,81119267	0,569910522	Srsy
17307117	9,272886	9,716673	9,55653	8,975078	8,557271	8,588086	9,515363	8,706811667	-0,80855133	0,570954888	Gjb2
17547269	4,228975	7,536777	6,538465	4,324965	7,205297	4,353359	6,1014057	5,294540333	-0,80686533	0,571622523	---
17547371	4,228975	7,536776	6,538465	4,324965	7,205297	4,353359	6,1014053	5,294540333	-0,806865	0,571622655	---
17546498	3,946838	7,025564	5,919765	3,749016	6,840199	3,883127	5,6307223	4,824114	-0,80660833	0,57172436	Ssty2
17547434	4,096217	7,295145	6,253305	4,228714	6,813716	4,184257	5,8815557	5,075562333	-0,80599333	0,57196813	---
17547291	3,280609	7,036769	5,968871	3,314757	6,940433	3,61885	5,4287497	4,62468	-0,80406967	0,572731292	LOC100039574
17546454	3,994581	7,120702	6,048251	3,981761	6,937217	3,832477	5,721178	4,917151667	-0,80402633	0,572748495	Ssty2
17547365	3,994581	7,120702	6,048251	3,981761	6,937217	3,832477	5,721178	4,917151667	-0,80402633	0,572748495	Ssty2
17546425	5,023037	7,217721	6,987842	4,715924	7,078928	5,023101	6,4095333	5,605984333	-0,803549	0,572938027	Srsy
17547283	4,330376	7,388045	6,500236	4,375998	7,227492	4,205274	6,0728857	5,269588	-0,80329767	0,573037848	LOC100039810 // LOC100039810
17329423	7,196228	7,242578	6,601696	6,108513	6,332161	6,1903	7,0135007	6,210324667	-0,803176	0,573086176	Sst
17537423	5,23636	7,784663	7,023537	5,431755	7,105444	5,099244	6,68152	5,878814333	-0,80270567	0,573273038	Astx
17537437	5,23636	7,784663	7,023537	5,431755	7,105444	5,099244	6,68152	5,878814333	-0,80270567	0,573273038	Astx
17537483	5,23636	7,784663	7,023537	5,431755	7,105444	5,099244	6,68152	5,878814333	-0,80270567	0,573273038	Astx
17537502	5,23636	7,784663	7,023537	5,431755	7,105444	5,099244	6,68152	5,878814333	-0,80270567	0,573273038	Astx
17428875	3,744872	3,372462	4,112525	3,053003	2,674052	3,105647	3,7432863	2,944234	-0,79905233	0,574726576	---
17203017	3,721965	4,206904	4,419985	3,32912	3,298916	3,325969	4,1162847	3,318001667	-0,798283	0,575033137	---
17208051	3,924175	3,788539	3,660829	3,237269	2,768478	2,976326	3,791181	2,994024333	-0,79715667	0,575482249	---
17342013	4,752242	3,609703	6,415411	3,744872	3,955553	4,691483	4,9257853	4,130636	-0,79514933	0,576283519	---
17449710	6,122308	7,396677	5,970575	5,282601	5,887056	5,944746	6,49652	5,704801	-0,791719	0,577655394	Cxcl9
17546502	4,106062	6,048833	4,909355	3,75686	5,413983	3,518548	5,0214167	4,229797	-0,79161967	0,577695168	LOC434960
17546671	3,904863	7,23025	6,231939	4,027783	6,879412	4,103757	5,7890173	5,003650667	-0,78536667	0,580204477	---
17205213	5,119939	4,878115	5,14678	4,127086	4,72905	3,9456	5,048278	4,267245333	-0,78103267	0,58195009	---
17209403	5,119939	4,878115	5,14678	4,127086	4,72905	3,9456	5,048278	4,267245333	-0,78103267	0,58195009	---
17334404	9,932952	10,32019	9,270746	9,092921	9,136926	8,952314	9,841296	9,060720333	-0,78057567	0,582134463	Gm8842 // Gm10420 // Gm5978 // Rps2
17208285	4,752242	4,039969	5,899795	4,896334	3,630012	3,824566	4,8973353	4,116970667	-0,78036467	0,582219608	---
17204585	3,786086	3,972867	4,975281	3,266285	3,446347	3,68821	4,2447447	3,466947333	-0,77779733	0,583256614	---
17547428	3,731006	6,958322	5,83412	3,802197	6,628288	3,765747	5,507816	4,732077333	-0,77573867	0,584089491	MGC107098 // MGC107098
17321622	6,633123	6,957055	6,892505	6,301378	5,909403	5,945428	6,827561	6,052069667	-0,77549133	0,584189635	---
17202451	5,462433	4,726288	5,760652	5,160044	3,987967	4,481207	5,3164577	4,543072667	-0,773385	0,585043174	---
17200021	5,105613	5,425519	5,838076	4,549642	4,570985	4,93761	5,4564027	4,686079	-0,77032367	0,586285927	---
17449346	9,095186	9,01479	9,045819	8,566771	7,853645	8,429847	9,0519317	8,283421	-0,76851067	0,587023162	Ugt2b5
17201695	5,788575	6,218571	5,658532	5,464206	5,430978	4,473131	5,8885593	5,122771667	-0,76578767	0,588132179	---
17547396	4,015222	7,214149	6,203393	4,042662	6,879412	4,215838	5,8109213	5,045970667	-0,76495067	0,588473491	---
17546575	4,13821	6,789948	5,842088	3,531282	7,069899	3,885298	5,590082	4,828826333	-0,76125567	0,589982609	Sly // Sly
17546651	4,13821	6,789948	5,842088	3,531282	7,069899	3,885298	5,590082	4,828826333	-0,76125567	0,589982609	Sly // Sly
17546678	4,13821	6,789948	5,842088	3,531282	7,069899	3,885298	5,590082	4,828826333	-0,76125567	0,589982609	Sly // Sly
17547163	4,13821	6,789948	5,842088	3,531282	7,069899	3,885298	5,590082	4,828826333	-0,76125567	0,589982609	Sly // Sly
17202929	5,773761	6,270919	5,178196	5,081163	4,916772	4,943211	5,7409587	4,980382	-0,76057667	0,590260348	---
17202921	6,372814	6,211418	5,812723	5,977304	4,927751	5,215456	6,1323183	5,373503667	-0,75881467	0,590981688	---
17205363	6,914315	6,035198	5,588985	5,038571	5,006053	6,217675	6,1794993	5,420766333	-0,758733	0,591015143	---
17547413	3,427644	7,069532	6,111503	3,769971	6,901469	3,670987	5,5362263	4,780809	-0,75541733	0,592375002	LOC100039574
17547251	3,953892	6,908075	6,129179	3,984914	6,819952	3,924048	5,6637153	4,909638	-0,75407733	0,592925466	LOC382133 // LOC382133
17293245	6,551016	7,075148	6,838996	5,684194	6,607962	5,916797	6,82172	6,069651	-0,752069	0,593751435	---
17547417	4,00157	7,054011	6,077339	3,991448	6,923265	3,963762	5,7109733	4,959491667	-0,75148167	0,593993205	Ssty2
17318950	6,929474	7,384344	6,717796	6,374639	6,24382	6,163717	7,010538	6,260725333	-0,74981267	0,594680772	Csf2rb2
17546982	5,115634	6,273634	6,221198	4,654041	5,816318	4,893476	5,8701553	5,121278333	-0,748877	0,59506658	LOC100040196
17210551	3,457789	3,854728	3,720061	2,861059	2,792099	3,138675	3,677526	2,930611	-0,746915	0,595876394	---
17547256	3,748794	6,637788	5,738283	3,660122	6,615822	3,60884	5,374955	4,628261333	-0,74669367	0,595967818	LOC100040744 // LOC100040744
17203913	4,365077	3,997701	4,528192	3,980606	3,105208	3,572155	4,29699	3,552656333	-0,74433367	0,596943516	---
17209681	5,609313	4,015206	4,822145	4,811331	3,594496	3,808356	4,8155547	4,071394333	-0,74416033	0,597015241	---
17371310	6,461539	7,43668	7,031465	6,511024	6,219338	5,969197	6,9765613	6,233186333	-0,743375	0,597340315	B3galt1
17546476	3,331408	6,400557	5,54091	3,161691	6,428431	3,454665	5,0909583	4,348262333	-0,742696	0,597621518	LOC380994 // LOC380994
17546479	3,331408	6,400557	5,54091	3,161691	6,428431	3,454665	5,0909583	4,348262333	-0,742696	0,597621518	LOC380994 // LOC380994
17546482	3,331408	6,400557	5,54091	3,161691	6,428431	3,454665	5,0909583	4,348262333	-0,742696	0,597621518	LOC380994 // LOC380994
17547321	3,331408	6,400557	5,54091	3,161691	6,428431	3,454665	5,0909583	4,348262333	-0,742696	0,597621518	LOC380994 // LOC380994
17208897	4,575855	5,02801	4,572237	3,863076	3,907809	4,181187	4,7253673	3,984024	-0,74134333	0,598182109	---
17546469	4,488775	6,828381	5,870141	4,561078	6,268688	4,133514	5,729099	4,98776	-0,741339	0,598183906	---
17537513	4,941097	7,931485	7,039886	5,111472	7,399142	5,179509	6,6374893	5,896707667	-0,74078167	0,598415037	Astx // Astx // Astx
17489250	6,401258	6,212561	5,762578	5,170062	5,038213	5,951588	6,1254657	5,386621	-0,73884467	0,599219024	---
17201627	4,704674	4,504286	4,868264	4,063245	3,694432	4,10377	4,692408	3,953815667	-0,73859233	0,599323839	---
17387742	4,101408	3,861211	4,203001	3,350389	3,402958	3,198678	4,0552067	3,317341667	-0,737865	0,599626064	Olfr259
17547368	4,093962	7,263824	6,471894	4,274889	7,185934	4,155443	5,9432267	5,205422	-0,73780467	0,599651141	Ssty2 // Ssty2
17547420	4,093962	7,263824	6,471894	4,274889	7,185934	4,155443	5,9432267	5,205422	-0,73780467	0,599651141	Ssty2 // Ssty2
17449322	10,51506	10,01117	10,08616	9,815335	9,191863	9,391888	10,20413	9,466362	-0,737768	0,599666382	Ugt2b1
17200791	3,788273	4,471067	5,351386	3,494043	3,694432	4,210737	4,5369087	3,799737333	-0,73717133	0,599914442	---
17476671	6,254813	5,407842	5,776452	6,066749	3,778249	5,387142	5,8130357	5,07738	-0,73565567	0,600545031	---
17280054	7,437006	7,411407	7,377545	6,972363	6,33753	6,711101	7,4086527	6,673664667	-0,734988	0,600823022	Trib2
17546597	3,256958	5,672079	4,151712	3,156386	4,506548	3,222982	4,3602497	3,628638667	-0,731611	0,602231051	Sly // Sly
17274558	7,889462	8,077404	7,787146	7,132787	7,209461	7,224119	7,918004	7,188789	-0,729215	0,603232056	Mboat2
17547389	5,548428	7,24824	6,684917	5,108084	7,357006	4,831536	6,4938617	5,765542	-0,72831967	0,603606536	LOC382133 // LOC382133
17547235	5,426045	7,125857	6,562534	4,985702	7,234623	4,709154	6,3714787	5,643159667	-0,728319	0,603606815	LOC382133
17547355	5,426045	7,125857	6,562534	4,985702	7,234623	4,709154	6,3714787	5,643159667	-0,728319	0,603606815	LOC382133
17205439	5,546922	5,314497	5,129913	4,36305	4,458366	4,985072	5,330444	4,602162667	-0,72828133	0,603622575	---
17547344	4,641142	7,031464	6,605944	4,586595	6,901658	4,611138	6,09285	5,366463667	-0,72638633	0,604415962	---
17203289	3,885252	4,145768	3,226613	2,984589	2,962985	3,132181	3,7525443	3,026585	-0,72595933	0,60459488	---
17219449	4,752242	3,815353	3,694432	3,859212	2,644673	3,583673	4,0873423	3,362519333	-0,724823	0,605071274	---
17203127	4,525661	4,926874	5,265556	4,327602	3,805929	4,410374	4,9060303	4,181301667	-0,72472867	0,605110839	---
17547288	3,546318	7,223688	6,151903	3,957731	7,048448	3,743233	5,6406363	4,916470667	-0,72416567	0,605347025	Ssty2
17547362	3,546318	7,223688	6,151903	3,957731	7,048448	3,743233	5,6406363	4,916470667	-0,72416567	0,605347025	MGC107098 // MGC107098
17312186	4,456084	3,996922	6,121368	4,265794	4,071561	4,064632	4,8581247	4,133995667	-0,724129	0,60536241	Psca
17247652	7,126163	7,193124	6,985162	6,252187	6,129012	6,752906	7,101483	6,378035	-0,723448	0,605648229	Zrsr1
17343685	7,816056	7,335585	5,403666	6,193632	6,150321	6,045242	6,851769	6,129731667	-0,72203733	0,606240721	---
17201183	6,98988	7,616096	6,630798	5,558317	6,448824	7,085926	7,0789247	6,364355667	-0,714569	0,609387157	---
17364440	12,20119	12,22446	12,36585	12,13527	11,16726	11,35902	12,263833	11,55385	-0,70998333	0,611327201	Cyp2c69
17428881	5,36541	5,435662	6,790361	5,997268	3,794019	5,672859	5,863811	5,154715333	-0,70909567	0,611703457	---
17404191	5,824476	5,158732	5,924267	4,896333	4,781291	5,110693	5,635825	4,929439	-0,706386	0,612853436	Car3 // Car3
17406186	8,616286	8,641311	8,259656	7,937413	7,348929	8,12844	8,505751	7,804927333	-0,70082367	0,615220863	Tdo2
17275704	6,876822	8,104382	6,220948	6,796378	6,40327	5,909441	7,067384	6,369696333	-0,69768767	0,616559629	---
17546457	3,515664	6,957532	6,034226	3,779763	6,961081	3,680575	5,502474	4,807139667	-0,69533433	0,617566186	MGC107098
17547415	3,515664	6,957532	6,034226	3,779763	6,961081	3,680575	5,502474	4,807139667	-0,69533433	0,617566186	Ssty2
17342999	4,674509	4,505275	5,165382	4,067684	3,99554	4,204596	4,781722	4,089273333	-0,69244867	0,618802673	Cpne5
17547445	3,755743	6,954411	5,738675	3,881363	6,69539	3,7952	5,482943	4,790651	-0,692292	0,618869874	Ssty2
17507306	4,357661	5,349718	4,732299	4,522561	3,84661	3,994832	4,813226	4,121334333	-0,69189167	0,619041628	---
17396152	9,257109	8,150538	8,933417	8,092195	7,888917	8,284731	8,7803547	8,088614333	-0,69174033	0,619106567	Car3
17547277	4,488224	6,560135	5,844674	4,167531	6,496611	4,155639	5,631011	4,939927	-0,691084	0,619388284	LOC100039810 // LOC100039810
17207033	6,492808	7,007739	6,705773	5,437501	6,363653	6,336692	6,73544	6,045948667	-0,68949133	0,620072437	---
17200243	5,746084	4,640232	5,963283	4,63739	4,982369	4,663263	5,4498663	4,761007333	-0,688859	0,620344274	---
17205597	4,752242	5,982514	4,287179	4,084911	3,516992	5,354499	5,0073117	4,318800667	-0,688511	0,620493929	---
17200723	4,846175	4,385405	4,164721	3,563018	3,858659	3,909374	4,4654337	3,777017	-0,68841667	0,620534502	---
17348457	8,085735	8,593071	7,897244	7,22871	6,592972	8,689156	8,1920167	7,503612667	-0,688404	0,620539951	---
17547065	4,300256	6,731734	6,730835	4,676303	6,834465	4,187409	5,9209417	5,232725667	-0,688216	0,62062082	Srsy // Srsy
17546522	4,233007	6,533343	5,628524	4,243317	5,802847	4,28803	5,464958	4,778064667	-0,68689333	0,621190067	Ssty1
17207691	3,397199	4,27058	4,165556	3,449936	3,018262	3,309125	3,944445	3,259107667	-0,68533733	0,621860405	---
17200085	4,259609	3,720061	4,623882	3,820643	3,187765	3,541349	4,201184	3,516585667	-0,68459833	0,622179026	---
17529398	7,389519	8,204382	7,59624	7,073011	6,833712	7,229671	7,730047	7,045464667	-0,68458233	0,622185926	Me1
17453131	4,127944	4,256861	5,952887	4,010303	3,978356	4,296252	4,7792307	4,094970333	-0,68426033	0,622324809	---
17200615	3,413514	3,859212	2,906405	2,674052	2,514424	2,939525	3,3930437	2,709333667	-0,68371	0,622562248	---
17202899	6,917395	7,247293	7,233191	6,499241	6,660277	6,188279	7,1326263	6,449265667	-0,68336067	0,622713013	---
17544837	11,30776	11,52596	11,48292	11,24634	10,56349	10,46185	11,43888	10,75722667	-0,68165333	0,623450389	Serpina7
17203971	5,021697	6,075118	5,227309	5,196726	4,387037	4,695881	5,4413747	4,759881333	-0,68149333	0,623519535	---
17205175	4,726725	5,678807	5,059009	4,361305	4,714345	4,344891	5,154847	4,473513667	-0,68133333	0,62358869	---
17209365	4,726725	5,678807	5,059009	4,361305	4,714345	4,344891	5,154847	4,473513667	-0,68133333	0,62358869	---

17260244	7,318207	7,81357	7,862424	6,972416	6,95267	7,02662	7,6647337	6,983902	-0,68083167	0,623805567	Gck
17547285	4,152369	6,816653	5,784469	4,222793	6,49684	3,997179	5,584497	4,905604	-0,678893	0,624644389	MGC107098
17547295	4,152369	6,816653	5,784469	4,222793	6,49684	3,997179	5,584497	4,905604	-0,678893	0,624644389	MGC107098
17204883	5,787069	5,071173	4,272777	4,323795	4,194252	4,576886	5,043673	4,364977667	-0,67869533	0,624729979	---
17546724	3,460468	5,871801	4,493953	3,40574	4,876668	3,517717	4,6087407	3,933375	-0,67536567	0,626173489	Sly // Sly
17202627	4,293671	4,213825	3,982373	3,774254	3,322185	3,369793	4,1632897	3,488744	-0,67454567	0,626529495	---
17204881	3,930006	3,926645	3,97442	3,759401	3,00172	3,04681	3,9436903	3,269310333	-0,67438	0,626601444	---
17537409	4,983349	7,676478	7,082869	5,431227	7,22757	5,064414	6,5808987	5,907737	-0,67316167	0,627130823	Astx // Astx // Astx
17537450	4,983349	7,676478	7,082869	5,431227	7,22757	5,064414	6,5808987	5,907737	-0,67316167	0,627130823	Astx // Astx // Astx
17537488	4,983349	7,676478	7,082869	5,431227	7,22757	5,064414	6,5808987	5,907737	-0,67316167	0,627130823	Astx // Astx // Astx
17205061	5,178856	4,699502	5,692377	4,372723	4,747007	4,43516	5,190245	4,518296667	-0,67194833	0,627658473	---
17209251	5,178856	4,699502	5,692377	4,372723	4,747007	4,43516	5,190245	4,518296667	-0,67194833	0,627658473	---
17206969	5,23392	5,86485	5,804757	5,476586	4,168885	5,245574	5,634509	4,963681667	-0,67082733	0,628146365	---
17546509	4,743961	7,649426	7,118808	5,001966	7,710026	4,790411	6,504065	5,834134333	-0,66993067	0,628536893	LOC382133
17453125	7,515022	7,618724	7,991293	7,496029	7,244037	6,378941	7,7083463	7,039669	-0,66867733	0,629083168	---
17205663	5,078465	4,40623	5,074753	4,248525	4,106807	4,199266	4,8531493	4,184866	-0,66828333	0,629254994	---
17538817	10,03368	10,10594	9,875014	10,15685	7,681977	10,17474	10,004878	9,337855667	-0,66702233	0,62980524	---
17200887	4,10106	3,983837	4,278485	3,379821	3,446074	3,542053	4,1211273	3,455982667	-0,66514467	0,630625465	---
17200149	5,163722	4,830859	5,337566	4,231361	4,485421	4,623057	5,1107157	4,446613	-0,66410267	0,631081105	---
17204815	6,074015	5,490748	6,133166	5,263353	5,154176	5,300349	5,8993097	5,239292667	-0,660017	0,63287084	---
17445359	6,401241	6,030603	6,131693	5,601192	5,346761	5,636834	6,1878457	5,528262333	-0,65958333	0,633061106	---
17546578	3,400945	5,268951	4,05542	3,125987	4,525874	3,095798	4,241772	3,582553	-0,659219	0,633220997	Sly
17546582	3,400945	5,268951	4,05542	3,125987	4,525874	3,095798	4,241772	3,582553	-0,659219	0,633220997	Sly // Sly
17546654	3,400945	5,268951	4,05542	3,125987	4,525874	3,095798	4,241772	3,582553	-0,659219	0,633220997	Sly // Sly
17546658	3,400945	5,268951	4,05542	3,125987	4,525874	3,095798	4,241772	3,582553	-0,659219	0,633220997	Sly // Sly
17546686	3,400945	5,268951	4,05542	3,125987	4,525874	3,095798	4,241772	3,582553	-0,659219	0,633220997	Sly // Sly
17547480	3,400945	5,268951	4,05542	3,125987	4,525874	3,095798	4,241772	3,582553	-0,659219	0,633220997	Sly // Sly
17202797	4,432372	4,835528	4,408291	3,641547	3,695411	4,362635	4,5587303	3,899864333	-0,658866	0,633375953	---
17220753	5,004043	5,103868	5,530568	4,212061	4,622746	4,831217	5,2128263	4,555341333	-0,657485	0,633982534	---
17207213	5,567118	5,821293	5,159505	4,729646	4,782649	5,06429	5,515972	4,858861667	-0,65711033	0,6341472	---
17204163	4,304471	3,776823	4,124932	2,946004	3,699835	3,590744	4,068742	3,412194333	-0,65654767	0,634394572	---
17220898	5,586968	5,918357	6,29862	4,719311	5,315005	5,800282	5,9346483	5,278199333	-0,656449	0,634437961	---
17208357	4,752241	5,44201	4,913456	4,461433	4,321582	4,368409	5,0359023	4,383808	-0,65209433	0,636355857	---
17201005	4,755358	6,336725	3,920464	4,062173	4,062173	4,941199	5,0041823	4,355181667	-0,64900067	0,637721901	---
17342006	6,90314	6,229296	5,625984	6,497536	4,641033	5,672859	6,2528067	5,603809333	-0,64899733	0,637723375	---
17547226	4,887726	7,694474	7,056654	5,081751	7,722532	4,89115	6,5462847	5,898477667	-0,647807	0,638249762	LOC382133
17547346	4,887726	7,694474	7,056654	5,081751	7,722532	4,89115	6,5462847	5,898477667	-0,647807	0,638249762	LOC382133
17486261	6,22594	6,333539	5,890089	5,376865	5,570133	5,560167	6,149856	5,502388333	-0,64746767	0,638399901	Vmn2r41 // Vmn2r40
17206405	3,536524	4,139839	3,076932	3,039092	3,083259	2,689701	3,5844317	2,937350667	-0,647081	0,638571026	---
17206535	5,427646	6,424494	5,386197	5,382571	4,645918	5,269491	5,7461123	5,099326667	-0,64678567	0,638701761	---
17210087	4,643565	5,371809	4,630563	4,896829	3,373057	4,436681	4,881979	4,235522333	-0,64645667	0,638847431	---
17204981	6,983438	7,347759	6,999029	6,837591	6,285168	6,276805	7,1100753	6,466521333	-0,643554	0,64013407	---
17491568	5,021487	4,457771	5,362721	4,660763	3,962316	4,288902	4,9473263	4,303993667	-0,64333267	0,640232284	---
17292772	4,727339	4,00635	4,541928	3,551874	3,854571	3,941315	4,4252057	3,782586667	-0,642619	0,64054907	---
17205341	4,447205	4,703767	4,120782	3,53532	3,575966	4,237472	4,423918	3,782919333	-0,64099867	0,641268894	---
17206701	5,224471	4,024617	5,872196	3,947802	4,795366	4,456803	5,040428	4,399990333	-0,64043767	0,641518304	---
17389917	6,467565	7,187689	6,668549	5,818748	6,112241	6,473892	6,774601	6,134960333	-0,63964067	0,641872801	1500003O03Rik // 1500003O03Rik
17206079	5,627688	4,857363	5,66271	5,467115	4,165556	4,597743	5,382587	4,743471333	-0,63911567	0,642106422	---
17201165	6,008298	5,478945	4,81299	3,283478	5,601769	5,499623	5,433411	4,794956667	-0,63845433	0,642400832	---
17201871	2,946385	4,176476	4,537863	3,135209	3,180315	3,432944	3,886908	3,249489333	-0,63741867	0,642862158	---
17204251	4,389776	3,359448	3,88652	3,148219	3,576496	2,999964	3,8785813	3,241559667	-0,63702167	0,643039084	---
17200907	3,654273	4,871458	3,233618	2,918716	3,824993	3,105063	3,919783	3,282924	-0,636859	0,643111592	---
17204875	4,633688	3,435157	3,832037	3,744872	3,115334	3,130107	3,9669607	3,330104333	-0,63685633	0,643112781	---
17341412	5,598032	5,544364	5,422324	4,860015	4,756995	5,03838	5,5215733	4,88513	-0,63644333	0,643296911	Zfp944
17547349	4,11277	6,601901	6,058821	4,129219	6,470588	4,265673	5,591164	4,95516	-0,636004	0,64349284	---
17547385	4,343616	6,745325	5,755117	4,458865	6,185633	4,292995	5,614686	4,979164333	-0,63552167	0,643708013	LOC100039574
17538757	9,726255	10,43639	10,1954	10,39145	7,412401	10,64883	10,119348	9,484227	-0,63512133	0,64388666	---
17357510	6,532134	7,783028	7,67679	7,003245	5,314334	7,769606	7,3306507	6,695728333	-0,63492233	0,643975482	---
17204539	3,950151	5,726359	4,079821	4,431582	3,028111	4,392881	4,5854437	3,950858	-0,63458567	0,644125777	---
17546715	3,331688	5,29068	4,207741	3,107796	4,580208	3,23998	4,276703	3,642661333	-0,63404167	0,644368705	Sly // Sly
17546729	3,331688	5,29068	4,207741	3,107796	4,580208	3,23998	4,276703	3,642661333	-0,63404167	0,644368705	Sly
17547059	3,331688	5,29068	4,207741	3,107796	4,580208	3,23998	4,276703	3,642661333	-0,63404167	0,644368705	Sly // Sly
17547067	3,331688	5,29068	4,207741	3,107796	4,580208	3,23998	4,276703	3,642661333	-0,63404167	0,644368705	Sly // Sly
17526268	5,417981	5,885863	5,915543	4,828286	5,392308	5,100054	5,7397957	5,106882667	-0,632913	0,644873012	---
17370367	4,943632	4,749231	4,305414	4,308971	3,704662	4,088143	4,6660923	4,033925333	-0,632167	0,645206555	---
17204821	4,431543	4,653671	4,073528	3,972061	3,97915	3,313537	4,3862473	3,754916	-0,63133133	0,645580392	---
17208403	4,779548	4,102294	4,73378	3,96961	4,044357	3,708807	4,5385407	3,907591333	-0,63094933	0,645751353	---
17477693	5,109385	4,649654	5,242573	4,527585	4,224903	4,357681	5,0005373	4,370056333	-0,630481	0,645961014	Mir150
17206819	9,040402	8,729889	8,897469	8,326742	8,113855	8,339203	8,8892533	8,259933333	-0,62932	0,646481056	---
17358640	10,45092	10,17131	10,29441	9,420476	9,555015	10,05793	10,305547	9,677807	-0,62773967	0,647189602	Mbl2
17208871	4,177016	4,188535	4,499523	3,682435	3,893411	3,406265	4,288358	3,660703667	-0,62765433	0,647227883	---
17208521	4,051254	4,246337	4,243835	3,641064	3,249243	3,768711	4,1804753	3,553006	-0,62746933	0,647310884	---
17206943	5,072577	4,830276	3,432901	3,223936	4,625971	3,605016	4,4452513	3,818307667	-0,62694367	0,647546784	---
17530501	4,057967	4,801021	4,138816	3,662517	3,696625	3,757842	4,3326013	3,705661333	-0,62694	0,64754843	---
17204707	3,707285	5,041899	5,051791	4,310043	3,949111	3,66416	4,600325	3,974438	-0,625887	0,648021237	---
17206953	5,937541	5,928308	5,661214	5,655496	5,185118	4,811843	5,8423543	5,217485667	-0,62486867	0,648478808	---
17540325	6,583641	6,365627	6,516911	6,030272	5,728793	5,833134	6,4887263	5,864066333	-0,62466	0,648572608	---
17202491	7,598571	6,974214	7,106296	7,047428	5,91086	6,84719	7,2263603	6,601826	-0,62453433	0,648629105	---
17270099	6,634522	7,13636	7,175143	6,129892	5,403666	7,5432	6,9820083	6,358919333	-0,623089	0,649279246	---
17342014	7,469074	6,844181	7,286203	6,79666	6,534885	6,398925	7,1998193	6,576823333	-0,622996	0,649321102	---
17219629	5,253721	5,299371	5,642926	5,276219	4,493001	4,559497	5,3986727	4,776239	-0,62243367	0,649574243	Olfr16
17208603	3,83504	3,804474	3,527052	3,192905	3,079415	3,027408	3,7221887	3,099909333	-0,62227933	0,649643736	---
17546450	3,808097	7,364269	6,475394	4,423414	7,233444	4,125372	5,8825867	5,260743333	-0,62184333	0,649840096	Ssty2 // Ssty2
17547261	3,808097	7,364269	6,475394	4,423414	7,233444	4,125372	5,8825867	5,260743333	-0,62184333	0,649840096	Ssty2 // Ssty2
17547394	3,808097	7,364269	6,475394	4,423414	7,233444	4,125372	5,8825867	5,260743333	-0,62184333	0,649840096	Ssty2 // Ssty2
17551087	7,3652	7,797523	7,538991	6,877935	6,612575	7,347445	7,567238	6,945985	-0,621253	0,650106057	---
17200515	5,325091	4,988576	5,39275	5,052213	4,605537	4,187366	5,2354723	4,615038667	-0,62043367	0,650475369	---
17463640	3,8726	3,653062	3,902339	3,196189	3,001968	3,37476	3,8093337	3,190972333	-0,61836133	0,651410404	Tas2r110
17205911	5,833282	5,107733	5,074058	3,879455	5,022736	5,270535	5,3383577	4,724242	-0,61411567	0,653330245	---
17498750	7,288272	8,015219	7,425038	7,178652	6,848557	6,859041	7,5761763	6,962083333	-0,614093	0,65334051	Cd209g
17221248	3,602498	4,032043	4,391096	3,494044	3,270892	3,418746	4,0085457	3,394560667	-0,613985	0,65338942	---
17546588	3,657115	5,578735	5,181997	3,630099	5,396122	3,550381	4,805949	4,192200667	-0,61374833	0,653496614	Sly
17546662	3,657115	5,578735	5,181997	3,630099	5,396122	3,550381	4,805949	4,192200667	-0,61374833	0,653496614	Sly
17202159	4,741705	4,690765	4,664652	3,763747	3,727498	4,76507	4,6990407	4,085438333	-0,61360233	0,653562751	---
17200109	5,78502	5,206208	4,994603	4,523136	4,864604	4,757839	5,3286103	4,715193	-0,61341733	0,653646564	---
17206103	7,234707	6,754609	7,090241	6,472928	6,255451	6,511163	7,026519	6,413180667	-0,61333833	0,653682358	---
17208417	5,642663	5,241036	5,104214	4,78705	5,074207	4,289805	5,3293043	4,717020667	-0,61228367	0,6541604	---
17205977	6,549954	5,989792	6,34003	6,375044	4,747641	5,921672	6,2932587	5,681452333	-0,61180633	0,654376873	---
17204689	6,803945	7,044979	6,938224	6,513995	6,090428	6,348302	6,9290493	6,317575	-0,61147433	0,654527479	---
17200783	4,938427	3,907379	6,138235	4,551262	3,981789	4,617241	4,9946803	4,383430667	-0,61124967	0,654629414	---
17546419	4,798768	6,959266	6,138408	4,745858	6,691691	4,629742	5,9654807	5,355763667	-0,609717	0,655325238	Ssty1
17546440	4,798768	6,959266	6,138408	4,745858	6,691691	4,629742	5,9654807	5,355763667	-0,609717	0,655325238	Ssty1
17546446	4,798768	6,959266	6,138408	4,745858	6,691691	4,629742	5,9654807	5,355763667	-0,609717	0,655325238	Ssty1
17203891	4,983554	5,819384	5,434077	5,276218	4,263869	4,873567	5,4123383	4,804551333	-0,607787	0,656202502	---
17478958	10,97339	11,28325	11,20352	10,84759	10,14512	10,64411	11,153387	10,54560667	-0,60778	0,656205686	1810008I18Rik
17203753	5,905418	5,386045	6,629614	5,781452	5,222217	5,095726	5,9736923	5,366465	-0,60722733	0,656457113	---
17383381	5,581587	5,120999	6,638279	5,387721	5,010295	5,121948	5,7802883	5,173321333	-0,606967	0,656575581	Cel
17284414	5,735585	6,057041	5,227585	5,387621	5,227585	4,584757	5,6734037	5,066654333	-0,60674933	0,656674649	---
17202611	4,065085	4,945574	5,121948	3,816674	4,473316	4,027943	4,710869	4,105977667	-0,60489133	0,657520904	---
17546668	4,531627	7,705984	7,106485	5,007867	7,709208	4,816653	6,448032	5,844576	-0,603456	0,658175395	LOC382133
17238359	8,673971	9,18926	8,597946	8,340385	8,213619	8,097589	8,8203923	8,217197667	-0,60319467	0,65829463	Apon
17547223	4,485297	7,717566	7,118067	5,019449	7,711031	4,78373	6,44031	5,83807	-0,60224	0,658730383	LOC382133
17547341	4,485297	7,717566	7,118067	5,019449	7,711031	4,78373	6,44031	5,83807	-0,60224	0,658730383	LOC382133
17547375	4,485297	7,717566	7,118067	5,019449	7,711031	4,78373	6,44031	5,83807	-0,60224	0,658730383	LOC382133
17547407	4,485297	7,717566	7,118067	5,019449	7,711031	4,78373	6,44031	5,83807	-0,60224	0,658730383	LOC382133
17547410	4,485297	7,717566	7,118067	5,019449	7,711031	4,78373	6,44031	5,83807	-0,60224	0,658730383	LOC382133
17209119	4,796451	5,149118	4,259067	4,195672	3,941889	4,262881	4,7348787	4,133480667	-0,601398	0,65911495	---
17243019	6,642828	7,481659	7,488444	7,166253	6,328498	6,31423	7,2043103	6,602993667	-0,60131667	0,65915211	---
17209915	5,769317	5,329129	5,792907	5,533587	4,862967	4,691484	5,630451	5,029346	-0,601105	0,659248825	---
17200585	3,585931	4,980228	3,912974	3,403615	3,745765	3,530747	4,159711	3,560042333	-0,59966867	0,659905494	---
17219633	4,383051	4,096879	4,054191	3,826877	3,578802	3,331213	4,1780403	3,578964	-0,59907633	0,660176489	Olfr218
17208563	6,150321	5,755008	5,637327	4,930556	5,745151	5,075871	5,847552	5,250526	-0,597026	0,661115388	---
17208309	7,199886	7,650729	7,149604	7,061825	6,605571	6,542081	7,3334063	6,736492333	-0,596914	0,661166714	---
17210465	5,104258	5,465283	5,633634	4,565513	5,151933	4,695136	5,4010583	4,804194	-0,59686433	0,661189476	---
17270116	6,983242	7,026304	6,934931	7,203154	5,924267	6,026959	6,9814923	6,384793333	-0,596699	0,661265253	---
17549298	3,435184	4,849085	3,651848	3,053003	3,866327	3,226818	3,9787057	3,382049333	-0,59665633	0,66128481	---
17314679	7,841655	8,038385	7,783657	7,234957	7,215379	7,42424	7,887899	7,291525333	-0,59637367	0,661414387	Tuba1b // Tuba1b
17491634	5,181779	4,771572	4,603392	4,336216	3,579368	4,853591	4,8522477	4,256391667	-0,595856	0,661651758	---
17449517	8,589488	8,805445	8,15082	7,961494	7,28704	8,511629	8,515251	7,920054333	-0,59519667	0,661954212	---
17486707	9,290318	9,052325	9,174664	8,779521	8,446836	8,506184	9,1724357	8,577513667	-0,594922	0,66208025	2810007J24Rik
17208379	3,962794	4,836417	4,562243	3,964985	3,853187	3,759163	4,453818	3,859111667	-0,59470633	0,662179231	---
17438737	5,939619	5,367744	5,96834	5,353889	5,059542	5,078325	5,7585677	5,163918667	-0,594649	0,662205547	Gm7714
17510583	5,907071	6,411982	6,42004	5,877633	5,197193	5,88038	6,2463643	5,651735333	-0,594629	0,662214727	---
17350921	7,835482	7,887252	6,771608	6,879133	6,698367	7,133552	7,498114	6,903684	-0,59443	0,662306077	F830016B08Rik // F830016B08Rik

17320035	6,816287	6,800766	4,797316	5,470645	6,046152	5,118797	6,138123	5,545198	-0,592925	0,662997346	Mir1249
17324303	5,855784	5,668561	5,920378	6,087567	4,905549	4,673732	5,8149077	5,222282667	-0,592625	0,663135227	---
17205495	4,287173	3,086156	2,930133	2,674052	2,973156	2,882678	3,4344873	2,843295333	-0,591192	0,663794233	---
17421396	5,017322	4,834324	4,140557	4,802366	3,496346	3,922576	4,6640677	4,073762667	-0,590305	0,664202473	---
17254041	5,929696	6,735865	6,207006	5,687034	5,435379	5,983813	6,2908557	5,702075333	-0,58878033	0,664904786	Ccl2
17205359	6,747958	5,986207	5,558704	5,389195	5,838293	5,301215	6,097623	5,509567667	-0,58805533	0,665239005	---
17398565	7,243706	6,703051	6,575846	5,839297	6,611698	6,310274	6,8408677	6,253756333	-0,58711133	0,665674434	Gm6525
17201161	5,656941	6,208749	5,6396	4,927943	5,085439	5,730766	5,8350967	5,248049333	-0,58704733	0,665703965	---
17538831	7,723526	7,628301	7,693639	7,300933	6,696112	7,288682	7,681822	7,095242333	-0,58657967	0,665919796	---
17236288	9,079985	8,972974	8,384923	7,765357	8,00754	8,906921	8,8126273	8,226606	-0,58602133	0,666177562	Igf1
17208337	4,45315	4,992811	4,026103	3,765076	3,951914	3,997508	4,490688	3,904832667	-0,58585533	0,666254218	---
17547423	4,428051	6,741466	6,01912	4,43801	6,815901	4,17751	5,7295457	5,143807	-0,58573867	0,666308098	LOC382133 // LOC382133
17285922	6,16664	5,992223	5,851145	5,840495	4,96393	5,44921	6,003336	5,417878333	-0,58545767	0,666437891	Slc17a1
17479485	5,104444	4,698954	5,158818	4,586279	4,533037	4,087363	4,9874053	4,402226333	-0,585179	0,66656663	---
17203985	6,214975	6,044613	6,85437	6,748542	4,679689	5,931149	6,3713193	5,78646	-0,58485933	0,666714342	---
17341701	8,26063	8,638515	8,335488	8,366417	7,561136	7,554756	8,4115443	7,827436333	-0,584108	0,667061647	---
17546748	3,192813	5,208249	4,331776	3,018035	4,716232	3,247824	4,2442793	3,660697	-0,58358233	0,667304745	Sly
17204213	6,209943	7,005093	7,260991	6,410147	5,815805	6,500562	6,8253423	6,242171333	-0,583171	0,66749503	---
17342007	7,476134	7,785392	7,674918	6,815626	7,163717	7,207723	7,6454813	7,062355333	-0,583126	0,667515851	---
17201765	4,633203	4,993078	5,318521	4,642179	4,062492	4,491469	4,9816007	4,398713333	-0,58288733	0,667626288	---
17201941	4,633203	4,993078	5,318521	4,642179	4,062492	4,491469	4,9816007	4,398713333	-0,58288733	0,667626288	---
17203005	6,488592	7,363645	6,556417	6,59826	6,186492	5,875396	6,8028847	6,220049333	-0,58283533	0,667650352	---
17204629	3,15996	3,998113	3,629714	2,823604	3,136468	3,081331	3,595929	3,013801	-0,582128	0,667977772	---
17208413	4,815142	5,533358	4,74843	4,616211	4,790011	3,944565	5,03231	4,450262333	-0,58204767	0,668014968	---
17203451	2,961785	3,725352	3,122024	2,676543	2,487258	2,902229	3,2697203	2,688676667	-0,58104367	0,668480014	---
17200579	4,093076	4,089271	4,422558	3,458821	3,776118	3,626918	4,201635	3,620619	-0,581016	0,668492834	---
17291177	7,34438	7,513692	7,556413	6,775074	6,904896	6,992282	7,471495	6,890750667	-0,58074433	0,668618726	Hist1h2bh
17522789	5,836481	5,5608	5,793471	4,903234	5,339928	5,206941	5,7302507	5,150034333	-0,58021633	0,668863473	---
17207419	3,962665	4,156663	4,813063	3,237459	3,666473	4,287838	4,310797	3,73059	-0,580207	0,6688678	---
17407509	4,033041	4,989244	4,169493	4,01222	3,579703	3,859734	4,3972593	3,817219	-0,58004033	0,668945075	Tdpoz1
17548536	6,081096	6,634851	6,365769	5,605902	5,625155	6,111789	6,360572	5,780948667	-0,57962333	0,669138457	Gm20176
17219473	6,565117	7,628301	6,570187	6,381208	5,677307	6,967957	6,9212017	6,342157333	-0,57904433	0,669407058	---
17352601	3,460815	3,850826	4,364533	3,379243	3,203628	3,358038	3,892058	3,313636333	-0,57842167	0,669696036	---
17547402	4,299768	6,596501	5,887503	4,267921	6,667022	4,114306	5,5945907	5,016416333	-0,57817433	0,669810857	LOC382133 // LOC382133
17547271	4,050878	6,825177	5,764431	3,978902	6,9323	3,995822	5,5468287	4,969008	-0,57782067	0,669975077	Ssty2
17548673	7,25443	7,463063	7,37258	6,526548	6,864458	6,966403	7,3633577	6,785803	-0,57755467	0,670098616	LOC100862010
17467401	4,056556	4,082434	4,400416	3,69418	3,591392	3,527007	4,179802	3,604193	-0,575609	0,671002943	---
17206985	4,307302	4,462806	4,865544	3,994359	4,038774	3,878498	4,5452173	3,970543667	-0,57467367	0,671438112	---
17209173	6,941041	7,293727	7,033288	6,544199	6,878302	6,127344	7,089352	6,516615	-0,572737	0,672340052	---
17401623	5,180242	4,883236	4,346164	4,07609	4,117187	4,499447	4,803214	4,230908	-0,572306	0,672540941	---
17208219	6,602836	7,031946	6,403087	6,187582	5,921193	6,213324	6,6792897	6,107366333	-0,57192333	0,672719352	---
17204261	5,80348	4,807562	4,993113	5,016023	4,428652	4,444048	5,201385	4,629574333	-0,57181067	0,67277189	---
17208883	4,412981	4,273292	4,951948	4,18618	3,863147	3,873905	4,5460737	3,974410667	-0,571663	0,672840755	---
17547392	5,165865	6,807764	6,128985	4,888319	6,624321	4,877919	6,0342047	5,463519667	-0,570685	0,673297027	LOC100041223
17340433	5,241418	5,958026	5,107224	4,398333	4,735848	5,462249	5,435556	4,865476667	-0,57007933	0,673579747	---
17547246	4,279881	6,628525	5,820096	4,263422	6,770514	3,984629	5,5761673	5,006188333	-0,569979	0,673626594	LOC382133
17204645	5,451859	4,876266	4,792891	4,819905	4,350952	4,240964	5,0403387	4,470607	-0,56973167	0,673742089	---
17209675	5,94499	5,69243	5,614613	5,173913	5,054038	5,316515	5,7506777	5,181488667	-0,569189	0,673995563	---
17203819	4,761857	5,669188	4,745647	4,410022	4,658957	4,404845	5,0588973	4,491274667	-0,56762267	0,674727717	---
17546673	4,457299	6,760273	5,972446	4,47086	6,818881	4,19914	5,730006	5,162960333	-0,56704567	0,674997626	LOC382133 // LOC382133
17343488	5,901573	5,568076	5,097406	5,027767	4,67044	5,167763	5,5223517	4,955323333	-0,56702833	0,675005736	Cyp4f14
17207929	3,872685	5,36541	3,74106	3,513437	3,602498	4,165556	4,326385	3,760497	-0,565888	0,675539484	---
17207147	4,481867	4,6542	4,656201	4,562509	3,334012	4,19874	4,5974227	4,031753667	-0,565669	0,675642038	---
17502330	6,514654	6,487931	6,63249	5,571555	6,127861	6,238874	6,545025	5,97943	-0,565595	0,675676695	Zfp709
17232547	3,860271	3,918902	4,345752	3,368555	3,653081	3,409292	4,0416417	3,476976	-0,56466567	0,676112082	---
17209903	5,963388	5,201574	5,094918	4,063553	5,712147	4,794674	5,41996	4,856791333	-0,56316867	0,676814008	---
17207671	5,094232	5,126599	6,429617	5,394965	4,272648	5,294771	5,5501493	4,987461333	-0,562688	0,677039542	---
17468531	8,812247	8,672503	8,485917	7,906019	7,816056	8,562325	8,656889	8,0948	-0,562089	0,677320703	---
17449332	7,267192	7,216733	6,426416	6,357931	5,921294	6,946515	6,9701137	6,40858	-0,56153367	0,677581473	Ugt2b36
17204549	3,25252	4,032043	5,113989	3,494044	4,326697	2,894129	4,1328507	3,571623333	-0,56122733	0,677725362	---
17448751	6,634522	7,359209	6,451573	5,503294	6,429427	6,833166	6,8151013	6,255295667	-0,55980567	0,678393538	---
17200851	4,355606	5,980546	4,919571	4,178976	4,468184	4,930899	5,085241	4,526019667	-0,55922133	0,678668363	---
17438987	6,395637	7,711463	6,423502	5,864317	6,638485	6,350146	6,843534	6,284316	-0,559218	0,678669931	Cxcl1
17200571	6,357809	6,083107	5,854873	6,05294	5,351203	5,214048	6,0985963	5,539397	-0,55919933	0,678678712	---
17359053	3,683466	4,41345	4,290625	3,518979	3,718629	3,473125	4,1291803	3,570244333	-0,558936	0,678802602	---
17229965	3,694432	3,859212	3,714786	3,373194	2,960838	3,259748	3,7561433	3,197926667	-0,55821667	0,67914114	---
17201297	3,044796	3,211247	4,01853	2,695192	2,973334	2,931629	3,4248577	2,866718333	-0,55813933	0,679177545	---
17203767	6,573987	5,654226	6,253994	5,974099	5,557544	5,276218	6,1607357	5,602620333	-0,55811533	0,679188844	---
17209135	5,726458	5,799252	6,663275	5,763719	4,691483	6,059676	6,062995	5,504959333	-0,55803567	0,67922635	---
17216244	5,156363	5,766586	5,348518	4,644443	4,924292	5,031138	5,4238223	4,866624333	-0,557198	0,679620841	Gm20253
17547166	3,392398	5,009389	4,058626	2,970814	4,552012	3,267109	4,153471	3,596645	-0,556826	0,679796105	Sly // Sly
17206685	5,750333	6,344943	6,903479	5,827521	6,001937	5,507392	6,3329183	5,77895	-0,55396833	0,681143968	---
17549848	4,887463	5,521923	4,863388	4,212307	4,346159	5,052585	5,0909247	4,537017	-0,55390767	0,681172611	---
17546415	4,557808	7,041091	6,177568	4,563278	6,882019	4,675042	5,925489	5,373446333	-0,55204267	0,682053746	Ssty1
17546436	4,557808	7,041091	6,177568	4,563278	6,882019	4,675042	5,925489	5,373446333	-0,55204267	0,682053746	Ssty1
17284420	5,607482	4,726737	4,667176	3,992026	4,573123	4,780344	5,000465	4,448497667	-0,55196733	0,682089362	---
17229960	12,02837	12,52565	12,42247	11,86481	11,77963	11,67786	12,325497	11,7741	-0,55139667	0,68235922	Apcs
17210351	5,843914	5,90937	5,668578	6,086692	4,697921	4,989246	5,8072873	5,257953	-0,54933433	0,68333535	---
17243016	7,256221	6,800601	7,014852	6,468454	6,271823	6,68466	7,0238913	6,474979	-0,54891233	0,68353526	---
17315743	7,010621	7,756962	7,093237	6,862055	6,549	6,803792	7,28694	6,738282333	-0,54865767	0,68365593	Osmr
17203097	5,020936	6,14817	5,397884	5,00667	4,829263	5,085447	5,52233	4,973793333	-0,54853667	0,683713271	---
17486461	4,938956	5,542902	4,759755	4,000248	4,723012	4,873382	5,0805377	4,532214	-0,54832367	0,683814222	---
17488105	8,332018	8,476911	8,663323	8,254869	7,754002	7,819169	8,4907507	7,94268	-0,54807067	0,68393415	Cyp2a22
17202467	6,463817	5,605587	6,965932	6,463481	4,651536	6,281169	6,345112	5,798728667	-0,54638333	0,684734527	---
17340631	7,068801	7,624933	6,519286	6,411387	6,594896	6,569084	7,0710067	6,525122333	-0,54588433	0,684971405	Rsph3b
17232162	8,665014	8,764541	9,321584	8,777474	8,427236	7,910662	8,9170463	8,371790667	-0,54525567	0,685269952	Vnn1
17302537	5,426246	5,709298	6,100588	5,616543	5,427243	4,557463	5,7453773	5,200416333	-0,544961	0,685409931	---
17254269	4,891019	5,745962	4,973465	4,646956	4,488059	4,84409	5,203482	4,659701667	-0,54378033	0,685971083	---
17200317	4,936287	4,938005	4,443763	4,185753	4,027579	4,474958	4,772685	4,22943	-0,543255	0,686220914	---
17227103	5,062436	4,873991	4,067858	3,576596	4,537863	4,265223	4,668095	4,126560667	-0,54153433	0,687039841	---
17549946	5,062436	4,873991	4,067858	3,576596	4,537863	4,265223	4,668095	4,126560667	-0,54153433	0,687039841	---
17279159	8,833794	9,297956	8,852829	8,140301	7,89654	9,32738	8,9948597	8,454740333	-0,54011933	0,687714022	---
17475373	6,47681	5,617497	6,012421	5,679998	5,626195	5,18028	6,035576	5,495491	-0,540085	0,687730389	Cyp2b13
17201849	6,634522	5,73373	6,114858	5,758152	4,995864	6,109176	6,1610367	5,621064	-0,53997267	0,68778394	---
17203227	6,822559	6,58701	7,038197	6,051012	6,026959	6,750814	6,815922	6,276261667	-0,53966033	0,687932856	---
17205913	4,603574	5,336217	5,346286	4,401719	4,329748	4,936127	5,095359	4,555864667	-0,53949433	0,688012016	---
17209663	6,198839	6,025017	6,445454	5,700425	6,012772	5,337755	6,2231033	5,683650667	-0,53945267	0,688031887	---
17210171	4,532563	5,670953	4,769433	4,858514	3,957599	4,538727	4,990983	4,451613333	-0,53936967	0,688071471	---
17320581	7,544231	7,330991	7,669982	6,975571	6,879239	7,076454	7,515068	6,977088	-0,53798	0,688734571	---
17549252	4,913333	5,513535	4,875823	4,255311	4,358593	5,078455	5,100897	4,564119667	-0,53677733	0,689308957	---
17321481	6,12808	5,49771	5,584899	5,113981	5,316051	5,170351	5,7368963	5,200127667	-0,53676867	0,689313098	Tuba1c // Tuba1c
17284517	3,540513	4,296431	3,126067	3,141489	2,842582	3,370035	3,654337	3,118035333	-0,53630167	0,689536264	---
17290288	7,34944	8,051905	7,636565	7,351621	6,989859	7,088465	7,6793033	7,143315	-0,53598833	0,689686038	Akr1c20
17488117	6,485035	6,554057	6,666218	5,922306	5,994493	6,182363	6,5684367	6,033054	-0,53538267	0,68997564	Cyp2f2
17206959	3,018262	3,754982	3,104575	2,598193	2,708583	2,966691	3,2926063	2,757822333	-0,534784	0,690262015	---
17207401	4,048461	3,420638	4,460224	3,008854	3,380187	3,936319	3,976441	3,441786667	-0,53465433	0,690324057	---
17201201	7,062977	7,588504	6,747768	6,175404	6,783197	6,839583	7,133083	6,599394667	-0,53368833	0,690786439	---
17203665	5,321312	6,153702	6,502848	5,387045	5,114735	5,878076	5,9926207	5,459952	-0,53266867	0,691274845	---
17204175	4,319051	4,471957	4,482578	4,117743	3,847735	3,710778	4,4245287	3,892085333	-0,53244333	0,691382823	---
17290850	3,526175	5,293095	3,821853	3,77433	3,613543	3,65675	4,2137077	3,681541	-0,53216667	0,691515423	---
17263157	3,997017	4,02791	4,160837	3,505863	3,753625	3,330801	4,0619213	3,530096333	-0,531825	0,691679211	Olfr330
17204187	5,026492	5,126685	4,639132	4,294394	4,401863	4,50072	4,9307697	4,398992333	-0,53177733	0,691702064	---
17516298	5,960674	5,33512	5,686987	5,489903	4,572143	5,32735	5,660927	5,129798667	-0,53112833	0,692013298	Olfr978
17547360	5,432292	6,798369	6,472271	5,052428	6,99826	5,063694	6,2343107	5,704794	-0,52951667	0,692786793	LOC382133
17283932	5,92822	6,76184	7,392504	6,381208	5,36541	6,748485	6,694188	6,165034333	-0,52915367	0,692961129	---
17204471	5,427937	4,897521	4,286689	4,245163	4,102909	4,676758	4,8707157	4,34161	-0,52910567	0,692984185	---
17547124	5,068865	6,667437	6,410071	4,940373	6,541265	5,078325	6,048791	5,519987667	-0,52880333	0,693129423	---
17408323	7,447809	7,824304	7,524183	7,206895	7,012729	6,990718	7,5987653	7,070114	-0,52865133	0,693202454	Hsd3b2 // Hsd3b2
17254660	6,055317	6,233993	5,876941	5,212093	5,687784	5,680684	6,055417	5,526853667	-0,52856333	0,693244738	Vmp1 // Vmp1
17203649	4,923019	4,429832	4,308488	4,190043	3,559504	4,326128	4,5537797	4,025225	-0,52855467	0,693248903	---
17202735	4,707659	3,840701	5,765896	5,199925	3,573287	3,956214	4,7714187	4,243142	-0,52827667	0,693382501	---
17204139	4,449352	4,381765	4,210738	2,906405	5,241523	3,309126	4,347285	3,819018	-0,528267	0,693387147	---
17278312	11,50494	11,97052	12,04309	11,55972	11,19124	11,18416	11,839517	11,31170667	-0,52781	0,693606825	Serpina3k
17547008	2,81212	4,453254	3,140929	2,928611	3,116903	2,777734	3,4687677	2,941082667	-0,527685	0,693666924	---
17421390	4,413438	4,529612	4,73042	3,839909	4,330608	3,921306	4,5578233	4,030607667	-0,52721567	0,693892623	Gm13238
17549386	10,16074	10,62359	10,51291	10,04139	9,82158	9,854229	10,432413	9,905733	-0,52668033	0,69415015	---
17549764	10,16074	10,62359	10,51291	10,04139	9,82158	9,854229	10,432413	9,905733	-0,52668033	0,69415015	---
17423505	7,020275	7,040523	6,968137	6,509281	6,165575	6,775206	7,009645	6,483354	-0,526291	0,694337502	---
17204767	5,867538	5,492311	5,657976	5,426977	5,0986	4,913602	5,6726083	5,146393	-0,52621533	0,694373919	---
17204357	5,341004	5,297526	5,793262	4,545825	4,949747	5,358387	5,477264	4,951319667	-0,52594433	0,694504365	---
17208395	6,126494	5,675971	5,94996	5,840539	5,254993	5,079437	5,917475	5,391656333	-0,52581867	0,694564863	---
17401660	4,89668	4,508678	4,602144	4,036385	4,034039	4,36013	4,6691673	4,143518	-0,52564933	0,694646391	Psma5
17203421	5,827633	5,803116	5,129732	4,822995	4,928525	5,432487	5,586827	5,061335667	-0,52549133	0,694722471	---
17425202	6,300933	7,421616	7,829262	6,832946	6,663095	6,479779	7,183937	6,658606667	-0,52533033	0,694800004	Acnat2

17204849	4,286314	5,090543	4,425447	3,58432	4,591868	4,050884	4,600768	4,075690667	-0,52507733	0,694921859	---
17454193	8,164371	9,480928	9,056052	8,763777	8,190535	8,174614	8,9004503	8,376308667	-0,52414167	0,6953727	Cyp3a13
17293928	5,992511	6,043744	5,767231	5,936437	5,165795	5,130404	5,9344953	5,410878667	-0,52361667	0,695625794	Zfp457 // Zfp457
17203033	4,60716	3,621499	4,289198	3,994454	3,782193	3,171652	4,172619	3,649433	-0,523186	0,69583348	---
17509697	6,318913	6,783301	6,351888	5,739067	5,831607	6,315375	6,4847007	5,962016333	-0,52268433	0,696075483	1810029B16Rik
17203655	7,434897	7,427813	7,188015	6,629688	6,616906	7,237311	7,3502417	6,827968333	-0,52227333	0,696273812	---
17205467	4,921643	4,387468	5,379202	4,273775	4,561132	4,287616	4,8961043	4,374174333	-0,52193	0,696439531	---
17323081	7,020759	6,833166	5,538308	6,605185	5,508072	5,713886	6,4640777	5,942381	-0,52169667	0,696552178	---
17206971	5,089349	5,371203	5,375796	5,22192	5,13995	3,910199	5,2787827	4,757356333	-0,52142633	0,696682711	---
17206935	3,416614	4,009632	3,513561	2,982861	3,282395	3,111897	3,6466023	3,125717667	-0,52088467	0,696944333	---
17395777	4,849394	5,027521	4,923394	4,487326	4,098676	4,652914	4,9334363	4,412972	-0,52046433	0,697147419	Ptk6
17200271	7,047937	6,547032	6,156217	6,850633	5,316747	6,02243	6,5837287	6,06327	-0,52045867	0,697150158	---
17546516	4,547061	6,770752	6,055995	4,750463	6,311105	4,751435	5,7912693	5,271001	-0,52026833	0,697242138	Ssty1
17206923	4,403339	5,503144	5,031413	4,626157	4,611512	4,140821	4,9792987	4,459496667	-0,519802	0,697467549	---
17208527	4,588118	4,566282	4,625772	4,098788	4,219378	3,903801	4,5933907	4,073989	-0,51940167	0,697661116	---
17208207	5,805377	6,960361	6,220994	5,521448	5,911467	5,995777	6,3289107	5,809564	-0,51934667	0,697687714	---
17210629	5,046238	4,648752	5,13073	4,912492	4,115573	4,240062	4,9419067	4,422709	-0,51919767	0,697759774	---
17377122	7,035374	6,955306	6,782381	6,271253	6,361047	6,584653	6,9243537	6,405651	-0,51870267	0,697999222	Gm561
17205541	6,476089	6,417012	6,731826	6,256225	5,773415	6,042061	6,5416423	6,023900333	-0,517742	0,698464163	---
17344860	4,185701	3,226818	4,19884	3,415551	3,248722	3,401585	3,870453	3,355286	-0,515167	0,699711933	Olfr99
17510579	6,900045	7,175779	6,550771	6,460835	6,103133	6,520993	6,8755317	6,361653667	-0,513878	0,700337381	---
17207345	7,212881	6,707944	7,018426	6,648757	6,500397	6,249759	6,9797503	6,466304333	-0,513446	0,700547121	---
17549794	6,669817	6,933906	6,611831	5,814404	6,342933	6,51867	6,738518	6,225335667	-0,51318233	0,700675165	---
17518238	6,843365	7,386507	6,901947	6,440632	6,607757	6,546189	7,0439397	6,531526	-0,51241367	0,701048584	Calml4
17475399	4,672431	5,429965	4,866024	4,746301	4,612985	4,073166	4,9894733	4,477484	-0,51198933	0,70125481	Cyp2a4
17549460	4,931317	5,534937	4,926905	4,256162	4,606644	4,995095	5,131053	4,619300333	-0,51175267	0,701369857	---
17549850	4,931317	5,534937	4,926905	4,256162	4,606644	4,995095	5,131053	4,619300333	-0,51175267	0,701369857	---
17204293	4,922667	4,614711	4,770725	3,844815	4,579815	4,349749	4,7693677	4,258126333	-0,51124133	0,701618487	---
17300225	5,80371	6,13308	5,733652	5,85665	5,168451	5,112452	5,8901473	5,379184333	-0,510963	0,70175386	---
17400800	6,059676	6,465245	6,019597	5,677307	5,276218	6,059676	6,181506	5,671067	-0,510439	0,70200879	---
17247933	5,763277	6,064104	5,875374	5,531918	5,307538	5,33219	5,9009183	5,390548667	-0,51036967	0,702042528	2810471M01Rik
17547012	3,739499	6,496925	5,851717	4,324662	5,878142	4,354332	5,3627137	4,852378667	-0,510335	0,702059398	Ssty1
17507221	6,66317	6,802503	6,591667	6,161081	6,0622	6,304415	6,68578	6,175898667	-0,50988133	0,7022802	Cd209f
17201539	5,956877	6,02423	5,014073	5,013878	5,364319	5,091072	5,66506	5,156423	-0,508637	0,702886183	---
17401557	5,790413	5,211	5,537372	5,558316	4,860985	4,594363	5,5129283	5,004554667	-0,50837367	0,703014491	---
17547466	3,347864	3,777367	4,147856	3,107418	3,460814	3,183238	3,7576957	3,25049	-0,50720567	0,70358388	---
17204253	4,128921	3,910221	3,171064	3,273435	3,333795	3,083686	3,7367353	3,230305333	-0,50643	0,703962264	---
17544295	5,49393	6,346832	6,304908	5,149344	5,860595	5,616929	6,0485567	5,542289333	-0,50626733	0,704041642	Srsx
17203045	5,653344	4,872947	5,493894	4,758642	4,71136	5,032715	5,3400617	4,834239	-0,50582267	0,704258674	---
17407336	8,295486	8,578769	8,249612	7,942642	7,982167	7,681977	8,3746223	7,868928667	-0,50569367	0,704321649	---
17359950	7,666674	7,805346	7,595109	7,222603	7,129508	7,19825	7,689043	7,183453667	-0,50558933	0,704372586	---
17200187	5,146292	3,995751	4,745383	4,295682	3,821913	4,253344	4,629142	4,123646333	-0,50549567	0,704418319	---
17248282	6,485247	6,441524	6,703112	5,730794	6,025339	6,358367	6,5432943	6,038166667	-0,50512767	0,704598024	Hbq1a
17486254	4,295079	4,167507	4,271524	3,799765	3,662501	3,756549	4,2447033	3,739605	-0,50509833	0,70461235	2810047C21Rik1
17489989	7,840282	8,208764	7,81964	7,383296	7,389031	7,581873	7,9562287	7,4514	-0,50482867	0,704744067	AI987944
17355951	3,651848	3,439657	3,604519	3,081503	3,102925	2,99858	3,5653413	3,061002667	-0,50433867	0,704983469	---
17475458	5,889004	5,752433	6,061175	5,582622	5,173432	5,436223	5,9008707	5,397425667	-0,503445	0,705420301	Cyp2g1
17205381	5,300885	4,817373	6,471496	5,152711	4,947488	4,979864	5,529918	5,026687667	-0,50323033	0,705525272	---
17464704	5,226212	4,988486	5,521929	4,787141	4,800721	4,639689	5,2455423	4,742517	-0,50302533	0,705625531	Dlx6as2
17206921	6,497513	6,305866	6,090378	5,669095	6,617548	5,098053	6,297919	5,794898667	-0,50302033	0,705627977	---
17283938	8,909459	9,255473	8,672503	8,397411	8,691819	8,239305	8,9458117	8,442845	-0,50296667	0,705654226	---
17206965	6,977842	7,156041	7,172548	6,610817	6,837507	6,349577	7,1021437	6,599300333	-0,50284333	0,705714553	---
17200555	4,989439	4,90374	5,082239	4,586743	5,013327	3,867794	4,991806	4,489288	-0,502518	0,705873713	---
17206527	5,040256	3,731021	3,970773	3,709341	3,852875	3,672638	4,24735	3,744951333	-0,50239867	0,705932102	---
17533934	6,903049	5,567698	6,092236	5,927049	5,15742	5,974097	6,187661	5,686188667	-0,50147233	0,706385516	Gm2790
17278998	8,333659	8,814821	8,638921	7,930556	8,037276	8,31573	8,5958003	8,094520667	-0,50127967	0,706479857	LOC100861856
17209033	6,03927	5,714581	5,401708	5,597348	4,885856	5,169267	5,7185197	5,217490333	-0,50102933	0,706602455	---
17435245	6,194204	6,858252	6,346519	6,59029	5,557103	5,74914	6,466325	5,965511	-0,500814	0,706707929	2700038G22Rik
17202449	4,900637	4,238094	4,169279	4,000374	3,95473	3,850575	4,4360033	3,935226333	-0,500777	0,706726053	---
17200759	4,251887	4,346653	4,745024	3,796353	4,046264	3,999382	4,4478547	3,947333	-0,50052167	0,706851143	---
17257053	5,596907	4,360271	4,507355	4,59366	4,165648	4,205909	4,821511	4,321739	-0,499772	0,707218539	---
17203263	4,340323	5,320293	4,221908	3,883463	4,269402	4,230826	4,627508	4,127897	-0,499611	0,707297467	---
17208333	6,216431	6,987038	6,193632	4,982644	6,964111	5,951588	6,4657003	5,966114333	-0,499586	0,707309724	---
17425221	7,364973	7,972998	6,617482	6,931328	6,528693	6,997178	7,3184843	6,819066333	-0,499418	0,707392094	Baat
17245169	6,206649	6,073726	6,112989	5,577681	6,05317	5,265006	6,1311213	5,631952333	-0,499169	0,707514196	Gm5781
17203375	6,910736	6,379581	6,362725	6,450892	6,012343	5,694029	6,551014	6,052421333	-0,49859267	0,707796893	---
17407407	3,853348	3,834174	5,487873	3,676949	4,122347	3,880351	4,3917983	3,893215667	-0,49858267	0,707801799	Sprr3
17538747	6,366714	6,193632	5,762578	4,391096	6,150321	6,285836	6,1076413	5,609084333	-0,498557	0,707814391	---
17202241	4,355304	4,438601	3,657531	3,161947	3,849804	3,94448	4,1504787	3,652077	-0,49840167	0,707890605	---
17208849	3,413515	3,875813	3,488808	3,066344	3,141637	3,076242	3,592712	3,094741	-0,497971	0,708101953	---
17387762	5,183871	4,139656	5,127447	4,874518	3,980214	4,103771	4,8169913	4,319501	-0,49749033	0,708337912	Olfr1133
17208597	5,412878	5,128603	5,896739	5,058314	4,408152	5,480461	5,4794067	4,982309	-0,49709767	0,708530731	---
17391423	5,784219	3,413514	3,047793	3,123178	4,112525	3,51964	4,081842	3,585114333	-0,49672767	0,708712467	---
17219374	6,967527	6,818686	6,95746	6,024654	6,629674	6,602315	6,9145577	6,418881	-0,49567667	0,709228951	Alyref2
17270053	4,871527	5,02797	4,527689	4,373172	4,174903	4,392185	4,809062	4,31342	-0,495642	0,709245993	Pyy
17208851	5,214744	5,645019	5,454188	5,108472	5,122985	4,595681	5,4379837	4,942379333	-0,49560433	0,709264511	---
17200157	4,606453	4,206904	4,383975	4,453913	3,432627	3,824565	4,3991107	3,903701667	-0,495409	0,709360548	---
17200345	3,290493	3,407483	3,284928	2,585474	2,800693	3,111476	3,3276347	2,832547667	-0,495087	0,70951889	---
17200807	6,800455	6,954348	6,658527	6,686844	6,065275	6,177525	6,8044433	6,309881333	-0,494562	0,709777133	---
17206761	5,618544	5,345841	5,068769	5,343379	4,53982	4,666915	5,3443847	4,850038	-0,49434667	0,70988308	---
17206007	5,594434	4,945107	6,192988	4,77162	5,133095	5,34524	5,5775097	5,083318333	-0,49419133	0,709959517	---
17204545	7,200816	8,019016	7,484744	6,80713	6,963489	7,454317	7,568192	7,074978667	-0,49321333	0,71044096	---
17547014	4,35864	5,919791	4,920921	4,132894	5,439122	4,148199	5,0664507	4,573405	-0,49304567	0,710523531	LOC434960
17251639	6,158537	5,808632	6,04448	5,484936	5,484726	5,564485	6,003883	5,511382333	-0,49250067	0,710791992	Chd3 // Chd3
17200763	3,170408	4,41971	3,571795	2,906832	3,215488	3,562352	3,7206377	3,228224	-0,49241367	0,710834857	---
17204747	3,284791	3,804474	2,833257	2,850968	2,762316	2,832425	3,3075073	2,815236333	-0,492271	0,710905154	---
17467062	7,996759	7,293921	7,354674	7,006259	6,756499	7,40699	7,5484513	7,056582667	-0,49186867	0,711103436	Inmt
17262644	7,278522	7,649656	7,327929	6,969137	6,876749	6,935069	7,4187023	6,926985	-0,49171733	0,711178032	Leap2
17210751	3,474163	4,604023	4,308971	4,263869	3,175654	3,474163	4,1290523	3,637895333	-0,491157	0,711454303	---
17546603	3,237225	5,642094	4,514694	3,127814	5,299474	3,493459	4,464671	3,973582333	-0,49108867	0,711488002	Sly
17546607	3,237225	5,642094	4,514694	3,127814	5,299474	3,493459	4,464671	3,973582333	-0,49108867	0,711488002	Sly
17202385	5,788747	5,596748	6,200249	5,388032	5,567163	5,157535	5,8619147	5,37091	-0,49100467	0,711529429	---
17426621	3,995981	3,986948	3,955672	3,520022	3,504889	3,443963	3,9795337	3,489624667	-0,489909	0,712070011	2310002L09Rik
17203689	6,555724	4,829289	6,281557	5,371985	5,997533	4,829289	5,8888567	5,399602333	-0,48925433	0,712393208	---
17375976	5,163833	5,2385	5,941901	5,059126	5,262234	4,555168	5,448078	4,958842667	-0,48923533	0,71240259	---
17412719	4,313012	3,807662	4,346232	4,066916	3,651848	3,280688	4,1556353	3,666484	-0,48915133	0,71244407	---
17358008	8,415887	8,146467	8,224716	7,183438	7,959562	8,176697	8,2623567	7,773232333	-0,48912433	0,712457404	---
17209877	5,676223	6,653538	6,799272	6,018278	5,297194	6,350163	6,3763443	5,888545	-0,48779933	0,71311204	---
17525731	5,450376	4,696949	5,492402	4,752287	4,642446	4,781837	5,2132423	4,725523333	-0,487719	0,713151749	Olfr955
17511714	6,913212	6,629101	6,425972	6,206429	5,807046	6,491856	6,656095	6,168443667	-0,48765133	0,713185199	Ces1e
17202091	3,83987	4,305736	2,879331	2,978203	2,879331	3,704701	3,674979	3,187411667	-0,48756733	0,713226725	---
17385396	5,961725	6,072231	5,650052	5,534814	5,229043	5,458574	5,8946693	5,407477	-0,48719233	0,713412138	---
17549216	6,10605	6,48352	5,771139	5,586539	5,816838	5,496003	6,1202363	5,633126667	-0,48710967	0,713453018	---
17202479	5,830154	6,086012	5,415642	5,220756	5,183536	5,467394	5,7772693	5,290562	-0,48670733	0,71365201	---
17247948	7,531551	7,183613	7,333877	6,98506	6,765517	6,838938	7,3496803	6,863171667	-0,48650867	0,713750291	Efemp1
17342016	6,501947	6,144849	6,891137	5,653609	6,438134	5,989274	6,5126443	6,027005667	-0,48563867	0,714180839	---
17210397	5,469478	5,33813	5,821674	5,076567	5,033184	5,062659	5,543094	5,05747	-0,485624	0,7141881	---
17200915	4,14515	5,792676	6,03594	4,861815	4,647209	5,009593	5,3245887	4,839539	-0,48504967	0,714472473	---
17206059	5,230131	4,565663	5,419664	5,086276	4,588493	4,086116	5,0718193	4,586961667	-0,48485767	0,714567564	---
17302054	7,84851	7,897085	7,828574	7,210549	7,299167	7,610855	7,8580563	7,373523667	-0,48453267	0,714728555	Snora31
17467447	4,550068	3,411897	3,925152	3,472768	3,245365	3,718019	3,9623723	3,478717333	-0,483655	0,715163494	---
17207325	6,003709	6,42333	5,907019	6,106167	5,490058	5,289689	6,1113527	5,628638	-0,48271467	0,715629782	---
17205989	6,473207	5,859544	6,361544	5,354137	5,652404	6,240394	6,2314317	5,748978333	-0,48245333	0,715759425	---
17546471	6,406477	6,499097	6,386973	5,535474	6,347697	5,967917	6,430849	5,950362667	-0,48048633	0,716735971	LOC100039659
17200737	3,310346	3,897227	3,857498	3,348828	3,207685	3,069799	3,688357	3,208770667	-0,47958633	0,717183234	---
17278253	6,219621	6,769377	6,407405	5,640525	6,157334	6,161025	6,4654677	5,986294667	-0,479173	0,717388737	Serpina3a
17203727	5,362245	5,282216	5,325659	5,029224	4,33426	5,171808	5,3233733	4,845097333	-0,478276	0,717834914	---
17201577	4,627295	3,532959	4,568705	3,910465	3,332728	4,051007	4,2429863	3,764733333	-0,478253	0,717846358	---
17200027	5,77154	6,255422	6,005219	4,638786	5,617635	6,342905	6,010727	5,533108667	-0,47761833	0,718162221	---
17428864	6,193632	4,987889	7,031826	5,467115	5,780025	5,533587	6,0711157	5,593575667	-0,47754	0,718201216	---
17309302	4,687148	4,018054	4,5228	3,917735	3,936855	3,940843	4,409334	3,931811	-0,477523	0,718209679	---
17201177	4,259736	5,53927	4,616425	4,35918	4,375258	4,24888	4,8051437	4,327772667	-0,477371	0,718285352	---
17205847	6,427539	5,954487	6,243937	5,614449	6,147368	5,432841	6,2086543	5,731552667	-0,47710167	0,718419459	---
17313575	6,401719	6,9442	6,756305	5,436918	6,480387	6,75485	6,7007413	6,224051667	-0,47668967	0,718624653	---
17202645	3,84539	6,112654	6,041597	5,467115	4,794018	4,308971	5,3332137	4,856701333	-0,47651233	0,71871299	---
17205689	8,917856	8,386011	8,647273	8,476992	8,358329	7,687102	8,65038	8,174141	-0,476239	0,71884917	---
17203301	4,456524	3,602692	3,534095	3,301071	3,398066	3,465888	3,864437	3,388341667	-0,47609533	0,718920759	---
17200847	6,601854	5,986245	6,346236	6,368189	5,492524	5,649707	6,311445	5,836806667	-0,47463833	0,719647174	---
17203917	4,253526	3,569434	3,699374	3,558363	3,348577	3,191719	3,840778	3,366219667	-0,47455833	0,719687081	---
17201807	5,069461	4,606536	4,825785	4,657737	4,170125	4,251908	4,8339273	4,359923333	-0,474004	0,719963663	---
17201983	5,069461	4,606536	4,825785	4,657737	4,170125	4,251908	4,8339273	4,359923333	-0,474004	0,719963663	---
17299979	5,747506	4,570146	4,942075	4,894727	4,096449	4,846981	5,0865757	4,612719	-0,47385667	0,720037192	---
17489908	9,192774	9,204432	8,971177	9,049662	8,472539	8,425708	9,1227943	8,649303	-0,47349133	0,72021955	Pop4
17484095	4,226962	4,608126	3,249463	4,02775	3,814848	2,82209	4,0281837	3,554896	-0,47328767	0,720321231	---

17206033	6,088409	6,53826	6,345493	5,769817	6,102929	5,680128	6,324054	5,850958	-0,473096	0,720416935	---
17206159	5,841202	5,676335	6,118151	5,888448	4,8796	5,448509	5,8785627	5,405519	-0,47304367	0,720443068	---
17515335	10,01088	10,39931	10,61671	10,3864	9,554171	9,668422	10,3423	9,869664333	-0,47263567	0,720646841	Gm6484
17200801	6,506081	7,626572	6,484711	7,545775	5,709298	5,944513	6,8724547	6,399862	-0,47259267	0,720668321	---
17445751	3,868535	4,124475	4,350758	3,453883	3,941724	3,532583	4,1145893	3,64273	-0,47185933	0,721034735	---
17549462	4,91641	5,427098	4,71583	4,241254	4,488059	4,915217	5,0197793	4,548176667	-0,47160267	0,721163024	---
17549852	4,91641	5,427098	4,71583	4,241254	4,488059	4,915217	5,0197793	4,548176667	-0,47160267	0,721163024	---
17248071	4,510143	6,015886	5,974602	5,951588	3,602498	5,533587	5,5002103	5,029224333	-0,470986	0,721471345	---
17239695	6,520222	6,536155	6,53475	6,165228	6,209521	5,803544	6,5303757	6,059431	-0,47094467	0,721492015	4930444G20Rik
17204719	2,87792	3,00937	4,012852	2,661918	2,640071	3,185762	3,3000473	2,829250333	-0,470797	0,721565867	---
17269347	5,329684	5,234686	6,479877	5,215886	5,247171	5,168832	5,6814157	5,210629667	-0,470786	0,721571369	Krt13
17506510	9,391092	9,879253	9,529259	9,175348	9,126926	9,086044	9,599868	9,129439333	-0,47042867	0,721750113	Snord68
17501516	4,186282	3,653691	4,504353	3,508086	3,895117	3,529914	4,1147753	3,644372333	-0,470403	0,721762954	---
17203983	4,710085	4,576525	5,009639	4,471865	4,007305	4,406481	4,7654163	4,295217	-0,47019933	0,721864853	---
17486152	4,851129	6,057173	5,440851	5,084565	4,800372	5,055635	5,4497177	4,980190667	-0,469527	0,722201339	Zfp772
17306419	7,875859	8,009566	7,44864	7,033369	7,429182	7,463269	7,7780217	7,308606667	-0,469415	0,722257407	---
17203237	4,830202	4,434976	4,674108	4,619347	4,06691	3,845446	4,6464287	4,177234333	-0,46919433	0,722367888	---
17201761	5,820714	6,175137	6,644573	5,489131	6,080019	5,664109	6,2134747	5,744419667	-0,469055	0,722437657	---
17201937	5,820714	6,175137	6,644573	5,489131	6,080019	5,664109	6,2134747	5,744419667	-0,469055	0,722437657	---
17204845	7,123898	7,796708	7,337084	7,582254	6,354617	6,914414	7,41923	6,950428333	-0,46880167	0,722564526	---
17201357	4,400565	3,965828	3,714833	3,781952	3,411783	3,482604	4,0270753	3,558779667	-0,46829567	0,722817997	---
17358092	4,272263	3,859402	4,031098	3,698949	3,702916	3,357221	4,0542543	3,586362	-0,46789233	0,723020103	---
17204987	5,894816	5,419996	5,792027	6,289951	4,65644	4,757605	5,7022797	5,234665333	-0,46761433	0,723159439	---
17337667	4,165459	4,5541	3,52118	3,629194	3,542713	3,666882	4,0802463	3,612929667	-0,46731667	0,723308662	Gm9041
17205771	4,860327	4,765142	5,29401	4,260978	4,536546	4,721506	4,9731597	4,506343333	-0,46681633	0,723559552	---
17207543	3,282758	3,270892	4,053041	2,973156	3,181319	3,053003	3,5355637	3,069159333	-0,46640433	0,723766213	---
17452204	5,030497	5,320117	4,914304	4,701341	4,673287	4,492081	5,088306	4,622236333	-0,46606967	0,723934127	Adam1a
17295588	6,326244	6,700847	7,026575	5,924055	6,377593	6,35397	6,6845553	6,218539333	-0,466016	0,723961057	Naip5
17342674	4,825878	4,814763	4,687986	4,207221	4,570195	4,15362	4,776209	4,310345333	-0,46586367	0,724037504	Mir3083
17317393	7,249801	7,308514	7,15693	6,462986	6,861152	6,994695	7,238415	6,772944333	-0,46547067	0,724234763	9930014A18Rik // 9930014A18Rik
17412858	4,80187	5,108264	5,235647	5,164009	4,180023	4,407088	5,0485937	4,583706667	-0,464887	0,724527824	---
17208029	4,429867	3,15996	3,838879	3,395604	3,033785	3,605834	3,8095687	3,345074333	-0,46449433	0,72472505	---
17271537	7,278854	7,280125	7,02579	6,818539	6,194041	7,17879	7,194923	6,730456667	-0,46446633	0,724739116	---
17201569	7,618281	7,301711	7,503222	7,327447	6,741235	6,963386	7,4744047	7,010689333	-0,46371533	0,725116479	---
17395193	5,249703	4,936229	5,125684	5,025569	4,583461	4,313844	5,103872	4,640958	-0,462914	0,725519351	---
17372925	3,773824	4,119013	3,668201	3,460505	3,284836	3,427831	3,8536793	3,391057333	-0,462622	0,725666211	Olfr1205
17399858	4,87388	4,76255	5,692496	4,979702	4,306565	4,656318	5,109642	4,647528333	-0,46211367	0,725921944	Sprr2a2 // Sprr2a1
17202769	6,196117	6,335834	5,98095	4,973326	6,512189	5,641568	6,170967	5,709027667	-0,46193933	0,726009669	---
17207525	5,411207	5,404942	6,30833	5,699605	4,510911	5,529019	5,7081597	5,246511667	-0,461648	0,726156292	---
17446058	5,225131	5,166605	4,402393	4,622435	4,822114	3,965026	4,9313763	4,469858333	-0,461518	0,726221728	---
17205137	5,115634	5,736159	5,083676	5,165991	4,481107	4,906521	5,311823	4,851206333	-0,46061667	0,726675582	---
17209327	5,115634	5,736159	5,083676	5,165991	4,481107	4,906521	5,311823	4,851206333	-0,46061667	0,726675582	---
17467404	6,459866	6,315927	5,777647	5,616543	5,832603	5,72467	6,18448	5,724605333	-0,45987467	0,727049418	---
17520667	4,93588	4,907344	4,986747	4,442293	4,515675	4,493796	4,9433237	4,483921333	-0,45940233	0,72728749	2410012M07Rik
17201447	5,464539	4,30564	3,393883	4,084911	3,847332	3,853813	4,3880207	3,928685333	-0,45933533	0,727321267	---
17462832	4,028426	4,200104	4,192499	3,507688	3,738307	3,797139	4,140343	3,681044667	-0,45929833	0,72733992	Vmn2r26
17202621	4,611758	5,343584	4,630342	4,601157	3,990655	4,617172	4,8618947	4,402994667	-0,4589	0,727540769	---
17207645	5,186413	4,584025	4,487227	4,385082	4,124351	4,372334	4,752555	4,293922333	-0,45863267	0,727675596	---
17494177	5,865926	5,594874	5,555569	5,223141	5,249876	5,167671	5,672123	5,213562667	-0,45856033	0,727712081	Olfr609
17370339	3,805418	4,052865	4,26604	3,737903	3,40319	3,607766	4,041441	3,582953	-0,458488	0,727748568	Olfr345
17549796	6,822053	7,194508	6,898902	6,102795	6,479218	6,960254	6,971821	6,514089	-0,457732	0,728130022	---
17262234	5,80594	6,333899	5,777295	5,472638	5,672146	5,401463	5,972378	5,515415667	-0,45696233	0,728518577	Tgtp2 // Tgtp1
17208617	4,610737	4,36863	4,513465	3,75553	4,414134	3,952355	4,4976107	4,040673	-0,45693767	0,728531033	---
17523682	4,877946	5,439826	5,053451	4,748363	4,423152	4,8292	5,123741	4,666905	-0,456836	0,728582375	4930433N12Rik
17276926	6,069755	6,519684	6,068982	5,585891	5,933672	5,769185	6,2194737	5,762916	-0,45655767	0,728722951	Synj2bp // Synj2bp
17206337	5,220432	4,141747	3,862243	3,535351	4,303122	4,017467	4,4081407	3,95198	-0,45616067	0,728923508	---
17461103	6,986987	7,183558	7,299626	6,6487	6,650894	6,802886	7,1567237	6,700826667	-0,455897	0,729056738	Kbtbd8
17439622	8,043542	8,093956	8,081907	7,570285	7,597635	7,68417	8,073135	7,617363333	-0,45577167	0,729120077	Agpat9
17487552	4,605545	4,146559	4,973606	3,878829	4,430603	4,04989	4,5752367	4,119774	-0,45546267	0,729276259	Vmn1r115
17468143	8,025867	8,584311	8,418222	7,99577	7,740803	7,925807	8,3428	7,88746	-0,45534	0,729338269	Mthfd2
17203203	3,912082	4,825075	4,955911	4,426692	3,564007	4,336702	4,564356	4,109133667	-0,45522233	0,729397756	---
17398989	5,571172	5,202195	5,603399	4,960806	4,953115	5,099813	5,458922	5,004578	-0,454344	0,729841959	AW047730
17526267	6,803337	7,602551	7,854651	7,16345	6,503794	7,232635	7,4201797	6,966626333	-0,45355333	0,730242057	---
17434490	8,756126	9,749619	8,769106	8,80363	8,669525	8,442228	9,091617	8,638461	-0,453156	0,730443201	Steap4
17208599	6,259996	5,675694	5,305734	5,793494	4,579659	5,510301	5,7471413	5,294484667	-0,45265667	0,73069606	---
17203675	4,665705	4,160967	4,912291	4,176969	4,197323	4,00724	4,5796543	4,127177333	-0,452477	0,730787063	---
17205693	3,897973	3,737434	3,651848	3,309126	3,226817	3,393883	3,7624183	3,309942	-0,45247633	0,730787401	---
17480588	4,708653	4,249913	4,822919	4,540686	3,773512	4,109988	4,5938283	4,141395333	-0,452433	0,730809351	Mir326
17208789	5,942502	5,561135	5,831298	5,291986	5,442769	5,243443	5,7783117	5,326066	-0,45224567	0,730904253	---
17448757	8,397411	8,074258	7,850843	7,967833	7,04281	7,955292	8,107504	7,655311667	-0,45219233	0,730931273	---
17244543	4,243017	3,964122	4,455243	3,686755	4,036604	3,582896	4,220794	3,768751667	-0,45204233	0,731007274	---
17427367	4,632991	4,91482	4,61219	4,174352	4,611357	4,018408	4,7200003	4,268039	-0,45196133	0,731048317	Cyp2j11
17520396	6,677733	7,349061	7,223764	6,802331	6,567009	6,525816	7,0835193	6,631718667	-0,45180067	0,731129735	Slc9a9
17467519	4,968434	4,637893	4,497026	4,462049	3,966912	4,320045	4,7011177	4,249668667	-0,451449	0,731307975	---
17549622	6,280295	5,487291	6,235691	5,897622	5,580705	5,171452	6,0010923	5,549926333	-0,451166	0,731451443	---
17343153	8,506455	7,957621	7,864539	7,613502	7,735914	7,62749	8,1095383	7,658968667	-0,45056967	0,731753849	1700097N02Rik
17428870	7,19004	6,015886	5,467115	6,063276	4,381765	6,876784	6,224347	5,773941667	-0,45040533	0,731837205	---
17295859	5,153391	4,67519	4,711497	4,578229	4,372403	4,239411	4,8466927	4,396681	-0,45001167	0,732036928	---
17200591	4,316994	5,886609	4,833022	5,13389	4,507947	4,045497	5,0122083	4,562444667	-0,44976367	0,732162777	---
17210839	4,584994	3,640475	3,785465	3,36452	3,658631	3,639053	4,0036447	3,554068	-0,44957667	0,732257685	---
17202999	5,324692	6,196771	5,30265	4,432581	5,408449	5,635967	5,6080377	5,158999	-0,44903867	0,732530804	---
17547172	3,922308	4,8076	4,791525	3,891366	4,475111	3,808401	4,5071443	4,058292667	-0,44885167	0,73262576	---
17326919	6,473205	6,373127	6,067307	6,028265	5,732541	5,807322	6,3045463	5,856042667	-0,44850367	0,732802501	---
17200621	5,102504	4,950767	4,382264	4,451722	4,338262	4,305537	4,811845	4,365173667	-0,44667133	0,733733808	---
17208755	6,654492	6,522513	6,70235	6,386271	6,210817	5,94227	6,6264517	6,179786	-0,44666567	0,73373669	---
17203511	4,173596	4,038795	4,636629	3,929229	3,479784	4,100143	4,2830067	3,836385333	-0,44662133	0,733759238	---
17203705	4,915711	4,803455	4,47018	4,191869	3,703138	4,955123	4,729782	4,283376667	-0,44640533	0,733869104	---
17323820	4,866364	4,384702	4,042609	4,130271	3,470972	4,35608	4,431225	3,985774333	-0,44545067	0,734354884	---
17210473	4,912802	6,10273	6,074745	6,150562	4,366859	5,238494	5,696759	5,251971667	-0,44478733	0,734692609	---
17487270	4,757906	4,834263	5,17752	4,313452	4,427137	4,695561	4,9232297	4,478716667	-0,444513	0,734832327	---
17312035	5,063361	4,464777	4,578592	4,050125	4,286391	4,436956	4,7022433	4,257824	-0,44441933	0,734880037	---
17238914	4,806196	5,724792	5,136331	4,995092	4,112341	5,227819	5,2224397	4,778417333	-0,44402233	0,735082289	Syne1
17420162	5,095632	5,178196	5,027302	4,480553	4,771	4,71941	5,1003767	4,656987667	-0,443389	0,735405056	Cdc42 // Cdc42 // Cdc42 // Cdc42
17316157	3,952397	4,327257	3,861099	3,812052	3,511745	3,486943	4,0469177	3,60358	-0,44333767	0,735431223	Gm5803
17206901	5,43713	5,451399	4,082438	4,803101	4,34397	4,494923	4,9903223	4,547331333	-0,442991	0,735607962	---
17551111	6,235893	6,238601	6,495177	5,690014	5,836164	6,115141	6,3232237	5,880439667	-0,442784	0,735713516	---
17204167	4,003386	3,986555	4,076488	3,456754	4,028898	3,2525	4,022143	3,579384	-0,442759	0,735726265	---
17340831	5,752122	5,895236	5,530128	5,595304	5,053763	5,200758	5,7258287	5,283275	-0,44255367	0,735830985	Slc22a1
17203329	3,478229	4,786085	3,796554	3,583389	3,356373	3,793612	4,0202893	3,577791333	-0,442498	0,735859378	---
17200365	4,792131	5,483977	5,065029	4,48916	4,647311	4,877503	5,1137123	4,671324667	-0,44238767	0,735915657	---
17551127	5,723377	6,028202	5,730412	5,385652	5,313352	5,457083	5,8273303	5,385362333	-0,441968	0,736129759	---
17203963	4,830472	4,742331	3,885835	4,631394	3,687566	3,814373	4,4862127	4,044444333	-0,44176833	0,736231645	---
17277710	5,789885	5,198335	5,861077	5,305413	4,836691	5,382543	5,6164323	5,174882333	-0,44155	0,736343073	---
17208113	4,330834	3,988771	3,532615	3,248854	4,314555	2,96535	3,95074	3,509586333	-0,44115367	0,736545387	---
17204079	5,470896	5,147315	6,005462	5,745189	4,840809	4,714394	5,5412243	5,100130667	-0,44109367	0,736576019	---
17208791	4,065414	3,838404	3,992542	3,521108	3,283392	3,769571	3,9654533	3,524690333	-0,440763	0,736744862	---
17306687	4,501021	4,274495	5,179131	4,341577	4,236763	4,055291	4,651549	4,211210333	-0,44033867	0,73696159	Gm10876
17204859	4,652034	5,619151	5,03821	4,627948	4,772828	4,588828	5,1031317	4,663201333	-0,43993033	0,737170205	---
17201107	6,844486	7,760588	6,415412	6,150321	6,227896	7,323454	7,0068287	6,567223667	-0,439605	0,737336459	---
17370518	4,485646	3,843472	3,9651	3,580373	3,915882	3,480523	4,0980727	3,658926	-0,43914667	0,737570742	---
17206913	4,549569	4,705248	5,260755	4,96204	3,99401	4,243445	4,838524	4,399831667	-0,43869233	0,737803054	---
17200779	3,756694	3,733783	4,221665	3,64824	2,748616	3,99935	3,9040473	3,465402	-0,43864533	0,737827091	---
17372552	5,666718	5,46767	5,514264	5,184741	4,955088	5,193134	5,5495507	5,110987667	-0,438563	0,737869199	Fam171b
17202363	6,388506	5,692657	6,412983	6,441881	4,742891	5,994529	6,1647153	5,726433667	-0,43828167	0,738013102	---
17201319	5,623173	5,2212	4,893399	5,001467	4,660598	4,761668	5,245924	4,807911	-0,438013	0,738150551	---
17549192	5,538308	6,13199	5,896438	5,241523	5,203598	5,808065	5,8555787	5,417728667	-0,43785	0,738233955	---
17445992	4,385117	3,772439	3,57716	3,478566	3,493398	3,45081	3,911572	3,474258	-0,437314	0,738508279	---
17206063	5,528179	4,063338	4,376066	4,372248	4,152033	4,132123	4,655861	4,218801333	-0,43705967	0,738638483	---
17207573	5,826114	5,692597	6,053488	6,066244	4,786277	5,408572	5,8573997	5,420364333	-0,43703533	0,738650941	---
17401333	6,600367	6,123423	6,266177	5,732941	5,980882	5,965503	6,329989	5,893108667	-0,43688033	0,738730304	---
17240665	3,385921	3,354721	3,98959	3,245522	3,044787	3,130106	3,576744	3,140138333	-0,43660567	0,738870961	---
17389295	4,305963	4,025021	3,614046	3,804236	3,252561	3,579442	3,9816767	3,545413	-0,43626367	0,739046135	Olfr1307
17546490	2,946522	5,470755	4,158805	3,06083	5,20736	2,999188	4,1920273	3,755792667	-0,43623467	0,739060991	LOC380994 // LOC380994
17546617	2,946522	5,470755	4,158805	3,06083	5,20736	2,999188	4,1920273	3,755792667	-0,43623467	0,739060991	LOC380994 // LOC380994
17547214	2,946522	5,470755	4,158805	3,06083	5,20736	2,999188	4,1920273	3,755792667	-0,43623467	0,739060991	LOC380994 // LOC380994
17547220	2,946522	5,470755	4,158805	3,06083	5,20736	2,999188	4,1920273	3,755792667	-0,43623467	0,739060991	LOC380994 // LOC380994
17547338	2,946522	5,470755	4,158805	3,06083	5,20736	2,999188	4,1920273	3,755792667	-0,43623467	0,739060991	LOC380994 // LOC380994
17290263	6,794316	7,708412	8,254847	7,911363	6,832573	6,706677	7,5858583	7,150204333	-0,435654	0,739358514	Akr1c18
17200743	4,236469	4,623706	4,807918	3,536071	4,927751	3,89791	4,556031	4,120577333	-0,43545367	0,739461189	---
17370326	4,205725	3,624348	3,492922	3,490023	2,940809	3,586912	3,7743317	3,339248	-0,43508367	0,739650859	Olfr338
17208737	5,032954	3,752388	3,988129	3,818353	3,884446	3,76571	4,2578237	3,822836333	-0,43498733	0,739700249	---
17453136	6,944724	7,414145	7,103524	7,044632	6,362465	6,750795	7,154131	6,719297333	-0,43483367	0,739779041	---
17439901	7,380843	7,47846	7,633153	7,171628	7,0112	7,005869	7,4974853	7,062899	-0,43458633	0,739905879	Lrrc8b
17487919	6,952566	6,760272	8,315088	6,806728	6,978226	6,940228	7,342642	6,908394	-0,434248	0,740079418	Cnfn

17201787	5,191318	4,512626	6,173803	5,100153	5,282483	4,192534	5,2925823	4,85839	-0,43419233	0,740107975	---
17201963	5,191318	4,512626	6,173803	5,100153	5,282483	4,192534	5,2925823	4,85839	-0,43419233	0,740107975	---
17206649	5,002947	5,563994	4,960634	5,057208	4,839804	4,328431	5,1758583	4,741814333	-0,434044	0,740184074	---
17519965	7,052332	6,663276	6,918274	6,398411	6,508786	6,424754	6,8779607	6,443983667	-0,433977	0,74021845	---
17207669	3,63917	3,306917	4,318306	3,2336	3,584432	3,144748	3,7547977	3,320926667	-0,433871	0,740272838	---
17207923	4,729871	4,440973	4,972295	4,047891	4,277531	4,518068	4,7143797	4,281163333	-0,43321633	0,740608836	---
17208741	4,037175	3,457502	4,210738	3,309788	3,602498	3,494044	3,901805	3,468776667	-0,43302833	0,740705352	---
17204547	7,04829	6,769529	6,489531	6,429428	6,193632	6,385379	6,7691167	6,336146333	-0,43297033	0,740735131	---
17200413	5,87941	5,469619	5,399251	5,504824	5,341669	4,603501	5,58276	5,149998	-0,432762	0,740842105	---
17547937	5,003178	4,825366	5,636776	4,744623	4,804161	4,618684	5,1551067	4,722489333	-0,43261733	0,740916397	2310042E22Rik
17204533	5,468678	5,865654	6,126096	5,793477	4,533209	5,836485	5,8201427	5,387723667	-0,432419	0,741018261	---
17459520	6,908623	6,800998	6,786823	6,619904	6,099754	6,480327	6,832148	6,399995	-0,432153	0,7411549	Reep1
17362717	8,262255	8,632597	8,596069	8,488986	7,91052	7,794958	8,4969737	8,064821333	-0,43215233	0,741155243	Dak
17202109	4,479288	5,949872	5,3663	5,381438	4,790542	4,327434	5,2651533	4,833138	-0,43201533	0,741225627	---
17284437	4,933434	4,912636	4,334079	4,29368	4,301841	4,288774	4,7267163	4,294765	-0,43195133	0,741258509	---
17305937	4,492967	4,258015	4,635015	4,214684	3,896611	3,979256	4,461999	4,030183667	-0,43181533	0,74132839	Olfr726
17337570	4,313538	4,184959	3,813346	3,938165	3,699826	3,378527	4,1039477	3,672172667	-0,431775	0,741349115	Olfr108
17207195	4,609582	4,935933	4,607315	3,99633	4,43736	4,423885	4,71761	4,285858333	-0,43175167	0,741361106	---
17430451	5,865971	5,538694	5,66201	5,177516	5,324404	5,26972	5,6888917	5,257213333	-0,43167833	0,74139879	Dcdc2b
17388016	6,923346	6,903864	6,666572	6,503354	6,804181	5,891808	6,8312607	6,399781	-0,43147967	0,741500892	Olfr1269
17547315	5,776464	6,689275	6,709809	5,411424	6,621858	5,85172	6,3918493	5,961667333	-0,430182	0,742168153	LOC100039675
17217158	3,740765	3,797163	4,733028	3,54437	3,804862	3,631876	4,0903187	3,660369333	-0,42994933	0,742287854	---
17333416	3,720353	3,272174	3,601087	2,998486	3,251443	3,054088	3,5312047	3,101339	-0,42986567	0,742330903	---
17204151	3,173281	3,574462	3,299309	2,85842	3,104501	2,795997	3,3490173	2,919639333	-0,429378	0,742581871	---
17204847	4,470513	5,593732	4,691545	4,083145	4,7834	4,601223	4,9185967	4,489256	-0,42934067	0,742601088	---
17323069	10,1915	10,0985	9,536937	9,506846	9,426311	9,606445	9,9423123	9,513200667	-0,42911167	0,742718971	---
17202571	3,514621	4,355134	4,760762	3,649445	3,28089	4,413462	4,2101723	3,781265667	-0,42890667	0,742824515	---
17202789	6,970767	6,758776	6,835364	6,563897	6,456044	6,258761	6,854969	6,426234	-0,428735	0,742912909	---
17207815	4,933709	5,395121	4,853752	4,764191	4,181048	4,951487	5,0608607	4,632242	-0,42861867	0,742972817	---
17417180	9,128721	8,997526	9,100718	8,767084	8,492746	8,68229	9,075655	8,647373333	-0,42828167	0,743146389	Cyp4a12a
17389285	6,318121	5,934688	6,022356	5,726735	5,799562	5,46494	6,0917217	5,663745667	-0,427976	0,743303857	Olfr1303
17209545	5,210355	4,965588	4,605362	4,300917	4,578559	4,619105	4,9271017	4,499527	-0,42757467	0,743510661	---
17206661	5,618734	5,116446	6,095318	5,686422	4,940727	4,92071	5,610166	5,182619667	-0,42754633	0,743525263	---
17205257	5,333408	5,546991	5,029126	4,689672	4,776134	5,161115	5,303175	4,875640333	-0,42753467	0,743531275	---
17209447	5,333408	5,546991	5,029126	4,689672	4,776134	5,161115	5,303175	4,875640333	-0,42753467	0,743531275	---
17262218	4,332535	5,082573	4,414354	4,099971	4,397282	4,049742	4,6098207	4,182331667	-0,427489	0,743554811	Gm12185
17200043	4,811504	5,405013	4,881598	4,866245	4,311575	4,638491	5,032705	4,605437	-0,427268	0,743668722	---
17210241	3,070189	4,835312	4,493739	3,859212	3,226817	4,032043	4,13308	3,706024	-0,427056	0,74377801	---
17426086	4,939641	4,790318	4,136055	4,741079	3,916306	3,928355	4,6220047	4,195246667	-0,426758	0,743931659	Mup20
17353252	6,681392	6,689781	6,596276	6,165912	6,104308	6,418027	6,6558163	6,229415667	-0,42640067	0,744115942	Ammecr1l
17216659	5,520202	5,66917	5,945393	5,237037	5,39138	5,229196	5,7115883	5,285871	-0,42571733	0,744468477	3110009E18Rik
17204873	3,123792	3,647643	3,467793	3,284111	2,992065	2,686839	3,413076	2,987671667	-0,42540433	0,74463001	---
17472420	4,467355	4,524719	4,062693	3,862832	4,183132	3,733974	4,351589	3,926646	-0,424943	0,74486816	---
17210559	5,01891	5,308756	5,285468	4,626156	4,865162	4,847401	5,204378	4,779573	-0,424805	0,744939414	---
17479947	3,863274	3,160541	3,335362	3,23491	3,100149	2,749765	3,453059	3,028274667	-0,42478433	0,744950085	Olfr293
17205281	6,399364	6,301937	6,514697	5,447802	6,29954	6,195179	6,4053327	5,980840333	-0,42449233	0,745100877	---
17209471	6,399364	6,301937	6,514697	5,447802	6,29954	6,195179	6,4053327	5,980840333	-0,42449233	0,745100877	---
17425211	6,748302	7,067988	7,105019	6,872376	6,351206	6,425861	6,9737697	6,549814333	-0,42395533	0,74537827	Acnat1
17206727	7,324001	7,299773	7,052178	6,972343	6,953613	6,478787	7,2253173	6,801581	-0,42373633	0,745491427	---
17450944	6,25094	5,90111	6,2707	6,203728	5,11514	5,832767	6,1409167	5,717211667	-0,423705	0,745507618	---
17337816	11,0917	11,20616	11,3189	11,0533	10,71433	10,57924	11,205587	10,78229	-0,42329667	0,745718653	Cyp39a1
17547327	5,068932	5,993295	6,308926	4,997622	6,076009	5,027634	5,7903843	5,367088333	-0,423296	0,745718997	LOC100039675
17205145	5,079005	4,494898	5,377819	4,058252	5,08519	4,539104	4,9839073	4,560848667	-0,42305867	0,745841683	---
17209335	5,079005	4,494898	5,377819	4,058252	5,08519	4,539104	4,9839073	4,560848667	-0,42305867	0,745841683	---
17274378	6,989427	6,514248	6,31887	6,245092	6,198462	6,110256	6,607515	6,184603333	-0,42291167	0,745917683	Gm3979 // Gm3979
17453128	8,854937	9,035202	9,059191	8,763533	8,405251	8,511839	8,98311	8,560207667	-0,42290233	0,745922509	---
17236194	3,704676	3,763944	3,670473	3,44302	2,874734	3,553703	3,713031	3,290485667	-0,42254533	0,746107113	Gm5174
17339388	5,480113	5,313821	4,92956	4,645784	4,709739	5,101114	5,2411647	4,818879	-0,42228567	0,746241414	---
17204799	4,366381	4,206548	4,453931	3,951137	3,46203	4,347328	4,3422867	3,920165	-0,42212167	0,746326249	---
17202181	5,515648	5,15466	5,772025	4,51575	5,258632	5,402076	5,4807777	5,058819333	-0,42195833	0,746410749	---
17207359	4,070496	3,073634	5,089107	3,590997	4,058822	3,317701	4,0777457	3,65584	-0,42190567	0,746437997	---
17205613	6,057734	5,781638	5,363479	5,478745	5,212921	5,245683	5,7342837	5,312449667	-0,421834	0,746475078	---
17481504	4,528108	4,037589	4,597654	3,823745	4,309242	3,766486	4,3877837	3,966491	-0,42129267	0,746755226	Olfr713
17207121	5,808764	5,219679	5,912293	5,794824	4,901732	4,980453	5,646912	5,225669667	-0,42124233	0,746781279	---
17210225	4,181911	4,820733	3,959568	3,714392	4,325218	3,658945	4,3207373	3,899518333	-0,421219	0,746793357	---
17208509	5,58291	5,982626	6,133377	5,166059	5,515676	5,754062	5,8996377	5,478599	-0,42103867	0,74688671	---
17255674	5,337487	5,183789	5,452174	5,072981	4,935425	4,702633	5,3244833	4,903679667	-0,42080367	0,74700838	Mir152
17476881	6,117692	5,875707	6,018752	5,645377	5,566071	5,538645	6,0040503	5,583364333	-0,420686	0,747069309	Zfp939
17207711	5,219285	4,798896	4,648025	4,843259	4,359073	4,201923	4,8887353	4,468085	-0,42065033	0,747087779	---
17475695	5,539183	5,647608	5,775954	5,263027	5,363397	5,075603	5,6542483	5,234009	-0,42023933	0,747300642	Gm10046
17209527	3,53787	3,866492	3,443736	3,277339	2,9677	3,344341	3,6160327	3,19646	-0,41957267	0,747646048	---
17259526	4,752242	6,845763	6,310274	6,088162	3,853813	6,70766	5,9694263	5,549878333	-0,419548	0,747658831	---
17535096	3,96455	3,982733	4,474581	3,787596	3,509258	3,8667	4,1406213	3,721184667	-0,41943667	0,74771653	---
17337589	3,789682	3,823756	4,006809	3,571241	3,719542	3,071519	3,8734157	3,454100667	-0,419315	0,74777959	Olfr120
17548744	10,24932	10,53417	10,45607	9,866718	9,959605	10,15546	10,413187	9,993927667	-0,419259	0,747808617	Hspa8 // Hspa8
17549414	6,803336	7,161957	6,877807	6,144212	6,458122	6,983193	6,9477	6,528509	-0,419191	0,747843865	---
17428866	8,372234	8,75774	8,393126	8,324279	7,553477	8,388111	8,5077	8,088622333	-0,41907767	0,747902615	---
17420426	6,115651	5,727647	6,33348	5,866714	5,438648	5,614733	6,058926	5,640031667	-0,41889433	0,747997662	---
17546633	4,02065	6,399254	5,035028	4,208386	5,742877	4,247001	5,151644	4,732754667	-0,41888933	0,748000255	LOC434960
17201417	6,073452	6,323661	5,722292	5,429615	5,522816	5,910898	6,0398017	5,621109667	-0,418692	0,748102574	---
17209155	5,746368	4,677307	4,731437	4,394413	4,727452	4,77738	5,051704	4,633081667	-0,41862233	0,7481387	---
17257937	5,470078	5,536405	5,061002	5,025214	4,995453	4,791147	5,3558283	4,937271333	-0,418557	0,748172581	Kcnj2
17200159	6,344146	6,417976	5,99511	5,480361	5,437698	6,583646	6,2524107	5,833901667	-0,418509	0,748197474	---
17525740	5,107747	4,981825	5,308311	4,895125	4,613926	4,633519	5,1326277	4,71419	-0,41843767	0,748234469	Olfr958
17205289	6,733111	7,195341	7,305326	6,570024	7,043412	6,365062	7,077926	6,659499333	-0,41842667	0,748240174	---
17209479	6,733111	7,195341	7,305326	6,570024	7,043412	6,365062	7,077926	6,659499333	-0,41842667	0,748240174	---
17205943	4,575873	5,33847	5,008477	4,610862	4,439449	4,617293	4,9742733	4,555868	-0,41840533	0,748251238	---
17401226	4,740895	4,406818	4,285657	4,139714	4,184165	3,854842	4,47779	4,059573667	-0,41821633	0,748349269	---
17308875	5,529931	5,473562	6,039173	5,346161	5,232478	5,209688	5,6808887	5,262775667	-0,418113	0,748402872	Zfp957
17201227	6,827005	7,558865	6,856822	5,859511	7,089649	7,041115	7,0808973	6,663425	-0,41747233	0,748735294	---
17200937	3,103977	4,418987	4,254349	3,591497	3,581489	3,352084	3,925771	3,508356667	-0,41741433	0,748765395	---
17207505	4,449065	5,368167	5,043946	4,710325	4,075739	4,822958	4,953726	4,536340667	-0,41738533	0,748780447	---
17549424	5,477465	5,418096	5,560586	5,249124	4,844145	5,113	5,4853823	5,068756333	-0,416626	0,749174656	---
17491685	4,122223	4,071109	4,54036	3,871415	3,876979	3,736557	4,244564	3,828317	-0,416247	0,749371492	B230209E15Rik
17403255	6,992363	7,858647	6,624342	6,703946	6,615205	6,908735	7,1584507	6,742628667	-0,415822	0,74959228	Gbp1
17269291	5,441905	5,062507	5,770377	4,862398	5,097759	5,06815	5,4249297	5,009435667	-0,415494	0,749762721	Krt34
17205895	5,678815	5,955143	6,026468	6,248312	5,187845	4,979691	5,8868087	5,471949333	-0,41485933	0,750092627	---
17491438	3,676326	3,919682	3,641738	3,157348	3,431939	3,405624	3,7459153	3,331637	-0,41427833	0,750394764	---
17548379	10,24897	10,53449	10,45063	9,866385	9,961814	10,16317	10,411363	9,997123	-0,41424033	0,75041453	Hspa8 // Hspa8
17434724	3,553341	3,776493	3,843309	3,192266	3,625321	3,11375	3,724381	3,310445667	-0,41393533	0,750573191	4933402N22Rik
17411948	5,068038	5,163039	4,963866	4,654041	4,602628	4,69649	5,064981	4,651053	-0,413928	0,750577007	Gm11837
17329494	6,397616	6,712837	6,70155	6,582236	5,887263	6,101886	6,604001	6,190461667	-0,41353933	0,750779242	Il1rap
17206171	5,096241	5,577567	4,998185	5,088487	4,991087	4,351957	5,2239977	4,810510333	-0,41348733	0,750806303	---
17210723	4,352456	4,117068	4,300916	4,405734	3,333361	3,791308	4,2568133	3,843467667	-0,41334567	0,750880033	---
17270091	9,515414	9,483859	9,912423	9,485538	8,948917	9,238855	9,637232	9,224436667	-0,41279533	0,751166519	---
17342001	7,278696	7,010502	6,637934	6,52369	6,414083	6,751329	6,9757107	6,563034	-0,41267667	0,751228308	---
17254059	4,288485	5,35275	4,511425	4,235211	4,561299	4,118343	4,7175533	4,304951	-0,41260233	0,751267015	Ccl12
17333999	6,501351	7,238622	6,192741	6,342641	5,847587	6,505678	6,644238	6,231968667	-0,41226933	0,751440441	Zfp758
17297372	6,577839	5,980534	6,519257	5,897236	5,970759	5,97314	6,35921	5,947045	-0,412165	0,751494786	---
17336962	9,345084	9,216394	9,082207	9,313311	7,872552	9,22164	9,2145617	8,802501	-0,41206067	0,751549135	---
17516420	3,291787	3,440545	3,739784	3,313027	2,924459	2,998672	3,4907053	3,078719333	-0,411986	0,751588032	Mir125b-1
17200407	7,648483	7,246912	7,991847	7,690795	6,822064	7,138433	7,6290807	7,217097333	-0,41198333	0,751589422	---
17468714	7,280925	7,661933	7,216463	6,983798	6,679787	7,259943	7,3864403	6,974509333	-0,411931	0,751616686	---
17343703	8,441422	7,948465	7,62465	7,606861	8,131112	7,040985	8,0048457	7,592986	-0,41185967	0,75165385	H2-K2
17353576	3,807574	4,629339	4,632945	3,81602	3,942098	4,076245	4,3566193	3,944787667	-0,41183167	0,751668438	---
17202459	3,725525	3,922985	4,20321	3,687247	3,467282	3,4622	3,9505733	3,538909667	-0,41166367	0,751755974	---
17398018	7,87571	6,712057	6,890524	7,693096	6,443368	6,106925	7,1594303	6,747796333	-0,411634	0,751771433	---
17521068	4,892012	4,493087	4,177416	3,952621	3,660357	4,7148	4,5208383	4,109259333	-0,411579	0,751800094	---
17308759	5,199988	4,666	5,111671	4,642712	4,60693	4,493739	4,992553	4,581127	-0,411426	0,751879827	4930444M15Rik
17378313	7,880737	8,688326	6,570187	7,044632	6,580274	8,280099	7,7130833	7,301668333	-0,411415	0,75188556	---
17210809	3,125964	4,501858	3,867982	3,718559	3,386723	3,156833	3,8319347	3,420705	-0,41122967	0,751982156	---
17207393	4,749971	4,602544	4,278326	4,526659	4,277508	3,593333	4,5436137	4,1325	-0,41111367	0,752042622	---
17420446	5,65461	5,379777	5,245811	4,720165	5,455454	4,871431	5,4267327	5,015683333	-0,41104933	0,752076158	---
17399854	6,518243	6,377601	7,670324	6,431573	6,484742	6,416968	6,8553893	6,444427667	-0,41096167	0,75212186	Sprr2a1
17208267	6,764866	6,070712	6,38037	6,608099	4,847833	6,527156	6,405316	5,994362667	-0,41095333	0,752126204	---
17200871	4,191657	4,459336	4,532684	3,792101	3,599802	4,559171	4,394559	3,983691333	-0,41086767	0,752170867	---
17466286	5,151974	5,305938	5,506091	4,909575	4,975312	4,846623	5,3213343	4,910503333	-0,410831	0,752189983	2210010C04Rik
17449525	6,513415	7,05869	6,539538	6,412591	6,267233	6,200254	6,703881	6,293359333	-0,41052167	0,75235128	---
17293238	8,961882	9,54565	9,361034	9,296687	8,427404	8,913014	9,289522	8,879035	-0,410487	0,752369359	---
17336788	7,020627	7,235679	6,71564	6,603341	6,574686	6,563289	6,9906487	6,580438667	-0,41021	0,752513829	Slc44a4
17290083	10,04095	8,952484	8,713736	8,847647	8,927692	8,704386	9,2357233	8,826575	-0,40914833	0,753067801	Emb
17201391	5,854404	5,729237	5,244043	5,798883	4,318223	5,483372	5,609228	5,200159333	-0,40906867	0,753109387	---
17462529	10,74166	10,36739	10,74497	10,25309	9,894721	10,47975	10,618007	10,209187	-0,40881967	0,753239381	Mug1
17387740	3,763855	4,221947	3,750834	3,523608	3,403202	3,583501	3,912212	3,503437	-0,408775	0,753262702	Olfr1109

17507915	4,887845	4,077024	3,727483	4,013818	3,787906	3,6652	4,230784	3,822308	-0,408476	0,753418832	---
17373448	5,566957	4,988943	5,265772	4,793928	5,074162	4,72857	5,2738907	4,865553333	-0,40833733	0,753491252	Dgkz // Dgkz
17372670	6,452506	6,592107	6,854013	6,039098	6,279234	6,356399	6,6328753	6,224910333	-0,407965	0,753685739	Prg3
17269156	6,347953	6,264482	6,328335	6,090288	5,736436	5,890511	6,31359	5,905745	-0,407845	0,753748432	Gm11937
17371956	5,139204	5,427876	5,473682	5,122691	4,753406	4,941787	5,3469207	4,939294667	-0,407626	0,753862859	8430437L04Rik
17525718	5,516911	4,649819	4,46278	4,875786	4,518057	4,01345	4,8765033	4,469097667	-0,40740567	0,753978	Olfr1537-ps1
17274735	8,093132	7,751637	7,927437	7,612187	7,417883	7,521631	7,9240687	7,517233667	-0,406835	0,754276299	Tmem18
17336963	8,391695	9,356133	8,046333	7,923952	8,046333	8,603707	8,5980537	8,191330667	-0,406723	0,754334858	---
17473724	3,692044	3,591945	3,907741	3,372462	3,553077	3,046779	3,7305767	3,324106	-0,40647067	0,754466806	Vmn1r73
17548900	4,042761	3,970387	3,736218	3,574327	3,442238	3,513406	3,9164553	3,509990333	-0,406465	0,754469769	---
17481350	5,362337	5,170803	5,108745	5,041254	4,310955	5,070989	5,2139617	4,807732667	-0,406229	0,754593198	Olfr679
17551123	7,066216	7,351449	6,783387	6,507779	6,735344	6,739512	7,0670173	6,660878333	-0,406139	0,754640273	---
17221549	5,400074	5,1553	5,283571	4,908794	4,898586	4,815728	5,2796483	4,874369333	-0,405279	0,755090253	---
17205941	6,422296	5,918293	5,980031	6,039415	5,161215	5,904181	6,1068733	5,701603667	-0,40526967	0,755095138	---
17202699	4,232263	3,986003	4,081157	3,598995	4,072484	3,412603	4,0998077	3,694694	-0,40511367	0,755176792	---
17545883	6,077851	6,188234	6,140753	5,830842	5,518657	5,842085	6,1356127	5,730528	-0,40508467	0,755191972	---
17284564	3,973873	3,155006	2,981466	2,997115	2,966793	2,932262	3,370115	2,96539	-0,404725	0,755380266	Ighv1-47 // Ighv1-47
17449363	4,99041	4,621456	4,809937	4,443655	4,320177	4,444296	4,8072677	4,402709333	-0,40455833	0,755467536	Ugt2a3
17205927	3,543908	3,95978	4,826269	4,035938	3,399437	3,681058	4,1099857	3,705477667	-0,404508	0,755493894	---
17201703	5,174123	4,668264	5,398152	5,158588	4,216627	4,651828	5,0801797	4,675681	-0,40449867	0,755498781	---
17201879	5,174123	4,668264	5,398152	5,158588	4,216627	4,651828	5,0801797	4,675681	-0,40449867	0,755498781	---
17206587	3,766573	3,267259	4,139114	3,282794	3,110098	3,566753	3,7243153	3,319881667	-0,40443367	0,755532821	---
17201377	4,40634	3,714786	3,066231	3,419292	3,43491	3,120456	3,729119	3,324886	-0,404233	0,755637916	---
17352680	8,479218	8,532413	8,302781	8,127167	7,917587	8,057153	8,4381373	8,033969	-0,40416833	0,755671787	Abhd3
17523949	3,654955	3,362523	3,781173	3,643127	2,863423	3,079884	3,5995503	3,195478	-0,40407233	0,755722073	---
17204749	4,892258	4,237226	5,738039	4,757616	4,595473	4,302553	4,955841	4,551880667	-0,40396033	0,755780744	---
17215762	7,118263	7,650753	7,324693	7,187887	6,604482	7,089653	7,3645697	6,960674	-0,40389567	0,755814621	Fam132b
17435567	5,38622	5,444263	5,370379	4,777819	4,997623	5,213969	5,4002873	4,996470333	-0,403817	0,755855835	Paxip1 // Paxip1
17206221	4,385117	3,526668	3,893266	3,552251	3,693331	3,349422	3,935017	3,531668	-0,403349	0,756101069	---
17308963	6,707467	6,01233	5,443957	5,602677	5,611916	5,739133	6,0545847	5,651242	-0,40334267	0,756104389	---
17548759	7,522425	7,674934	7,609235	7,084955	7,222196	7,290743	7,602198	7,199298	-0,4029	0,756336422	Rpl4 // Rpl4
17204703	5,006005	5,244867	5,355194	4,705358	4,546723	5,145474	5,202022	4,799185	-0,402837	0,756369451	---
17206659	5,665616	5,803881	6,015742	5,439985	5,363085	5,473817	5,828413	5,425629	-0,402784	0,756397238	---
17366343	4,068161	4,95141	3,724731	4,035617	3,696776	3,804117	4,2481007	3,845503333	-0,40259733	0,756495112	---
17237978	7,229415	7,383538	7,282815	6,999884	6,814693	6,873598	7,2985893	6,896058333	-0,402531	0,756529896	Ddit3
17282932	3,214991	3,674552	3,705542	3,009408	3,152039	3,226363	3,531695	3,12927	-0,402425	0,756585483	---
17549312	6,084135	6,018361	6,243685	5,747404	5,828091	5,563925	6,1153937	5,71314	-0,40225367	0,75667534	---
17210815	6,034388	4,719195	4,329648	4,746055	4,437939	4,693648	5,0277437	4,625880667	-0,401863	0,756880267	---
17509962	4,332314	4,441278	4,57387	4,092182	3,927021	4,123474	4,449154	4,047559	-0,401595	0,757020881	---
17306950	7,188426	7,463607	7,227792	6,61004	6,767524	7,298203	7,293275	6,891922333	-0,40135267	0,757148051	Gm5801 // Gm5801 // Gm5801 // Gm58
17205915	4,09678	4,080331	3,746378	3,553702	3,431102	3,735434	3,9744963	3,573412667	-0,40108367	0,757289239	---
17300233	4,444288	4,659982	4,127262	3,796385	3,664651	4,568124	4,4105107	4,00972	-0,40079067	0,757443054	---
17319401	8,590775	8,987823	8,744624	8,398046	8,318651	8,404232	8,7744073	8,373643	-0,40076433	0,75745688	Snord83b
17450461	5,611507	6,873073	4,541961	5,361083	5,115829	5,347783	5,6755137	5,274898333	-0,40061533	0,757535113	Gbp4
17516352	6,808947	6,999245	6,763307	6,803473	6,260549	6,30608	6,8571663	6,456700667	-0,40046567	0,757613705	9030425E11Rik
17527409	4,963843	4,054834	4,670093	4,190939	4,313481	3,983515	4,5629233	4,162645	-0,40027833	0,757712087	---
17250734	9,332743	9,686887	9,635272	9,287508	9,091435	9,075228	9,551634	9,151390333	-0,40024367	0,757730294	2410006H16Rik
17299152	4,151566	3,769045	4,694002	3,596976	3,75076	4,067469	4,204871	3,805068333	-0,39980267	0,757961951	LOC666692
17209047	5,584374	5,439576	6,634951	5,253465	5,693934	5,513305	5,8863003	5,486901333	-0,399399	0,758174059	---
17459784	4,24637	4,008759	4,363801	3,75937	3,762868	3,898881	4,20631	3,807039667	-0,39927033	0,758241679	Gm20594
17305943	4,379348	4,003196	3,79735	3,808729	3,660474	3,514229	4,0599647	3,661144	-0,39882067	0,758478049	Olfr730
17204705	4,165162	4,766432	6,131797	4,419281	5,034168	4,414021	5,0211303	4,62249	-0,39864033	0,758572863	---
17204069	4,63137	5,51266	5,322178	4,297566	5,282699	4,692283	5,1554027	4,757516	-0,39788667	0,758969246	---
17293635	4,760004	5,208333	5,28854	4,68784	4,706781	4,668759	5,0856257	4,687793333	-0,39783233	0,75899783	Hsd17b3
17200863	5,07585	4,014746	3,917544	4,053606	3,822425	3,938795	4,3360467	3,938275333	-0,39777133	0,759029923	---
17208779	5,75941	5,638818	5,749212	6,25788	4,561089	5,135543	5,7158133	5,318170667	-0,39764267	0,75909762	---
17478801	4,498712	4,184757	4,567052	3,835996	4,163508	4,059504	4,4168403	4,019669333	-0,397171	0,759345836	Otud7a
17248081	9,78889	9,870655	9,675996	9,681863	8,899926	9,562443	9,7785137	9,381410667	-0,397103	0,759381628	---
17209667	5,628625	4,825585	4,827862	4,414331	4,425886	5,251153	5,094024	4,697123333	-0,39690067	0,759488136	---
17376970	5,326545	4,783741	4,862825	4,591751	4,57671	4,614161	4,991037	4,594207333	-0,39682967	0,759525514	---
17491695	6,678819	6,590829	6,749465	6,331448	6,155899	6,341322	6,6730377	6,276223	-0,39681467	0,759533411	---
17546407	3,906352	5,472795	5,034702	4,014399	5,154482	4,054594	4,8046163	4,407825	-0,39679133	0,759545695	LOC100041550 // LOC100041550 // LOC
17546431	3,906352	5,472795	5,034702	4,014399	5,154482	4,054594	4,8046163	4,407825	-0,39679133	0,759545695	LOC100041550 // LOC100041550 // LOC
17347983	4,743808	5,875287	4,961456	4,532404	4,474023	5,38382	5,193517	4,796749	-0,396768	0,75955798	---
17276255	10,63487	10,4679	10,23631	9,823324	10,20195	10,12366	10,44636	10,04964467	-0,39671533	0,759585708	---
17204211	4,667337	4,318276	4,580435	4,580471	4,223825	3,571774	4,522016	4,125356667	-0,39665933	0,759615193	---
17277766	5,500844	5,014632	5,392197	4,659168	4,982339	5,077845	5,3025577	4,906450667	-0,396107	0,759906066	Tshr
17217193	5,438877	5,139874	5,564666	4,99339	4,842391	5,120178	5,381139	4,985319667	-0,39581933	0,760057603	Tmcc2
17325361	5,36602	5,486224	5,443154	5,00737	5,078336	5,022612	5,4317993	5,036106	-0,39569333	0,760123987	BC100530
17200803	4,214637	3,376265	3,457587	3,677287	2,869877	3,314402	3,6828297	3,287188667	-0,395641	0,760151561	---
17207173	4,1485	4,939023	4,395535	4,616079	3,769179	3,911541	4,4943527	4,098933	-0,39541967	0,760268189	---
17333380	5,831878	5,502862	5,924267	5,440104	5,24644	5,386506	5,7530023	5,357683333	-0,395319	0,76032124	Smok2b
17210149	5,289365	4,484402	3,72029	3,914456	3,814368	4,579466	4,498019	4,102763333	-0,39525567	0,760354619	---
17205675	3,270892	3,404864	3,143532	2,475628	2,948698	3,209432	3,273096	2,877919333	-0,39517667	0,760396256	---
17366892	5,179806	4,251952	5,12155	4,552374	4,445863	4,369741	4,8511027	4,455992667	-0,39511	0,760431394	---
17546707	3,440967	4,838683	4,061091	3,440967	4,271845	3,443157	4,1135803	3,718656333	-0,394924	0,76052944	Sly // Sly
17547146	3,440967	4,838683	4,061091	3,440967	4,271845	3,443157	4,1135803	3,718656333	-0,394924	0,76052944	Sly
17204757	5,831707	7,014739	7,239306	6,70168	5,83392	6,365725	6,6952507	6,300441667	-0,394809	0,760590065	---
17516316	4,327132	4,027996	4,474353	3,916173	4,064435	3,664993	4,2764937	3,881867	-0,39462667	0,760686198	Olfr986
17202477	3,175447	3,446959	3,418926	2,582663	3,122673	3,152351	3,3471107	2,952562333	-0,39454833	0,760727501	---
17207167	5,023674	4,732786	4,523785	4,459789	3,634391	5,002699	4,7600817	4,365626333	-0,39445533	0,760776542	---
17420432	6,385838	6,907103	6,684996	6,539381	5,528283	6,727015	6,6593123	6,264893	-0,39441933	0,760795526	---
17468876	4,25016	3,615051	3,724644	3,768046	3,438441	3,200583	3,863285	3,469023333	-0,39426167	0,760878675	Vmn1r42
17547086	3,557483	5,723179	5,04776	3,787285	5,829587	3,528891	4,7761407	4,381921	-0,39421967	0,760900826	LOC100041346
17547130	3,557483	5,723179	5,04776	3,787285	5,829587	3,528891	4,7761407	4,381921	-0,39421967	0,760900826	LOC100041346
17546625	3,557483	5,723179	5,04776	3,787285	5,829587	3,528891	4,7761407	4,381921	-0,39421967	0,760900826	---
17546639	3,557483	5,723179	5,04776	3,787285	5,829587	3,528891	4,7761407	4,381921	-0,39421967	0,760900826	---
17203123	5,504384	5,561542	4,44733	4,598018	4,971061	4,761711	5,1710853	4,77693	-0,39415533	0,760934757	---
17548030	7,077724	7,254369	6,458408	6,369832	6,842453	6,395921	6,930167	6,536068667	-0,39409833	0,760964822	LOC100862184
17203853	4,434103	4,042366	4,994963	4,095324	4,088766	4,105423	4,4904773	4,096504333	-0,393973	0,761030933	---
17232499	4,006277	4,106252	4,366408	3,99994	3,538147	3,75958	4,1596457	3,765889	-0,39375667	0,761145059	Rsph4a
17456918	6,459631	6,233281	6,922828	6,101477	6,086623	6,246475	6,53858	6,144858333	-0,39372167	0,761163524	Cpa1
17453130	7,984844	8,390463	7,941665	7,523666	7,828803	7,783417	8,1056573	7,711962	-0,39369533	0,761177418	---
17230174	7,726485	7,630951	7,702256	7,351831	7,181944	7,345853	7,686564	7,293209333	-0,39335467	0,761357178	Chml
17384657	4,340789	3,987342	4,624777	4,162758	3,838331	3,7722	4,317636	3,924429667	-0,39320633	0,761435462	Mir5128
17486692	3,802987	3,312165	3,555102	3,117014	2,991679	3,382679	3,5567513	3,163790667	-0,39296067	0,761565133	Sult2a6
17203703	6,456128	5,470291	5,692607	5,323379	5,551481	5,566437	5,8730087	5,480432333	-0,39257633	0,76176804	---
17428457	11,69042	11,57189	11,59984	11,67121	10,77116	11,24238	11,620717	11,22825	-0,39246667	0,761825948	Cyp4a14
17397426	7,19004	7,544007	7,337329	6,949507	7,039436	6,905147	7,3571253	6,964696667	-0,39242867	0,761846015	Ccrn4l
17370596	4,259461	3,726896	4,143317	3,516662	3,8256	3,610357	4,0432247	3,650873	-0,39235167	0,761886678	Gm19922 // Gm19922
17358821	7,034998	6,886487	7,24204	6,750363	6,683435	6,554132	7,0545083	6,662643333	-0,391865	0,762143729	Ifit3
17235462	8,098643	8,162057	8,36749	7,99202	7,774053	7,68655	8,2093967	7,817541	-0,39185567	0,76214866	Oaz1
17362595	11,38725	11,60612	11,41272	11,29849	10,70046	11,23163	11,468697	11,07686	-0,39183667	0,762158697	Fads2
17284965	7,019335	6,745108	6,976447	6,662884	6,401314	6,502817	6,91363	6,522338333	-0,39129167	0,762446669	Akr1c6
17421469	4,218944	4,681395	3,908798	3,599199	4,1161	3,920621	4,2697123	3,87864	-0,39107233	0,762562593	Rex2 // Rex2
17372007	5,259122	5,575141	5,243701	4,833023	5,223701	4,848477	5,3593213	4,968400333	-0,390921	0,762642587	---
17206201	7,457184	6,499287	7,388299	6,760668	6,712122	6,700165	7,1149233	6,724318333	-0,390605	0,76280965	---
17225320	8,398191	8,546847	8,49461	8,177146	7,916358	8,174354	8,4798827	8,089286	-0,39059667	0,762814056	Ugt1a1 // Ugt1a1
17365801	4,380173	3,516226	3,507334	3,650538	3,42671	3,155014	3,8012443	3,410754	-0,39049033	0,762870281	---
17487112	3,879847	4,203475	3,889484	3,470927	3,29884	4,032043	3,9909353	3,600603333	-0,390332	0,76295401	Psg17
17346599	9,809469	10,22069	10,13121	9,748886	9,29879	9,943751	10,05379	9,663809	-0,38998067	0,763139831	---
17465339	4,303943	4,642385	3,916862	3,856585	3,855036	3,981716	4,28773	3,897779	-0,389951	0,763155524	---
17389079	4,937428	5,28855	5,062333	4,816872	4,78293	4,518773	5,0961037	4,706191667	-0,389912	0,763176154	LOC433461
17206753	4,84383	4,585525	4,518053	4,01303	4,561357	4,20358	4,649136	4,259322333	-0,38981367	0,763228174	---
17353200	4,826598	4,744834	5,133202	4,345424	4,527263	4,664011	4,9015447	4,512232667	-0,389312	0,763493617	---
17455498	5,559514	6,024391	5,807365	5,125578	5,493598	5,604175	5,79709	5,407783667	-0,38930633	0,763496615	Alox5ap // Alox5ap
17202431	3,836395	4,243217	3,918973	3,56323	3,464666	3,80348	3,9995283	3,610458667	-0,38906967	0,763621873	---
17417115	4,645407	4,279498	4,152018	4,000817	4,180029	3,729247	4,3589743	3,970031	-0,38894333	0,763688745	---
17547436	4,477164	6,619089	6,637815	4,472336	6,768877	5,326406	5,911356	5,522539667	-0,38881633	0,763755975	LOC100861873 // LOC100861871 // LOC
17200577	3,218598	3,442718	3,474532	3,043303	3,051303	2,875147	3,378616	2,989917667	-0,38869833	0,763818446	---
17309965	7,93469	7,351352	6,888767	6,650858	7,155328	7,202737	7,391603	7,002974333	-0,38862867	0,763855331	C9
17201257	4,256217	3,819144	4,219087	3,633269	4,070184	3,426024	4,0981493	3,709825667	-0,38832367	0,764016835	---
17546444	3,891019	6,482652	5,495113	4,011369	6,10686	4,585977	5,2895947	4,901402	-0,38819267	0,764086213	Ssty1
17357508	7,213812	7,222908	7,617558	6,909439	6,505619	7,474767	7,351426	6,963275	-0,388151	0,764108281	---
17205477	5,233119	5,959924	6,317805	5,510429	6,242148	4,594089	5,8369493	5,448888667	-0,38806067	0,764156126	---
17203015	6,679401	6,805619	6,928022	6,57148	6,56385	6,114135	6,8043473	6,416488333	-0,387859	0,764262951	---
17203277	6,704275	6,835326	6,422983	6,780007	5,799495	6,21994	6,6541947	6,266480667	-0,387714	0,764339768	---
17547903	5,559778	5,540491	5,763969	5,20187	5,244854	5,255874	5,6214127	5,234199333	-0,38721333	0,764605067	Gm9948
17202671	3,483149	4,032043	4,369395	4,097801	3,552479	3,074184	3,961529	3,574821333	-0,38670767	0,764873109	---
17469026	4,468838	3,92189	3,951359	3,804984	3,571353	3,807097	4,114029	3,727811333	-0,38621767	0,765132937	4930471M09Rik
17206265	4,99297	4,015425	4,974637	4,048052	4,122209	4,65415	4,6610107	4,274803667	-0,386207	0,765138594	---
17432514	4,483914	4,147714	4,062618	3,668821	3,907136	3,960147	4,2314153	3,845368	-0,38604733	0,765223278	Oog3
17202303	4,288147	3,519545	3,344213	3,495867	3,270802	3,227684	3,7173017	3,331451	-0,38585067	0,7653276	---
17449641	8,001499	7,860604	8,450788	8,024405	7,145616	7,988953	8,104297	7,719658	-0,384639	0,76597064	---

17206013	3,931648	4,477245	4,315312	4,030528	3,858963	3,681035	4,2414017	3,856842	-0,38455967	0,766012762	---
17330506	8,616213	8,673642	8,710105	8,445766	8,390285	8,010417	8,6666533	8,282156	-0,38449733	0,766045859	Gramd1c
17210407	3,429523	3,491195	3,833342	3,631212	3,129829	2,839628	3,5846867	3,200223	-0,38446367	0,766063736	---
17201483	5,297316	4,940073	5,372565	4,546641	5,186712	4,723269	5,203318	4,818874	-0,384444	0,766074179	---
17260806	4,818751	5,127446	4,284576	3,911102	4,631614	4,535344	4,743591	4,359353333	-0,38423767	0,76618375	---
17430449	6,879055	7,322126	7,206722	6,778445	6,67998	6,796779	7,1359677	6,751734667	-0,384233	0,766186229	Tmem234 // Tmem234
17328935	6,742402	6,23351	6,72425	6,287672	6,27268	5,987185	6,5667207	6,182512333	-0,38420833	0,766199329	---
17400118	3,311243	3,878045	3,67844	3,15293	3,234612	3,327665	3,622576	3,238402333	-0,38417367	0,76621774	---
17355133	3,369592	3,356263	3,565217	2,821989	3,036719	3,279992	3,4303573	3,046233333	-0,384124	0,766244118	---
17422697	8,325319	8,528312	8,198048	7,746608	8,033248	8,120132	8,3505597	7,966662667	-0,383897	0,766364692	Mrpl20
17200603	5,599746	4,858324	6,53841	4,684267	6,00022	5,16035	5,6654933	5,281612333	-0,383881	0,766373191	---
17289553	6,759912	6,660176	6,737375	6,271495	6,317163	6,418357	6,7191543	6,335671667	-0,38348267	0,766584819	Sgtb
17201677	3,072852	3,95015	2,890512	3,081503	3,037135	2,644673	3,3045047	2,921103667	-0,383401	0,766628214	---
17244424	5,148435	5,63642	5,426189	5,086384	4,968513	5,006114	5,4036813	5,020337	-0,38334433	0,766658327	---
17270115	4,103771	3,651848	5,896438	3,576596	4,165556	4,760218	4,5506857	4,167456667	-0,383229	0,766719618	---
17335540	9,399504	9,795181	9,510769	9,3531	9,121145	9,081869	9,5684847	9,185371333	-0,38311333	0,766781092	Pim1
17206875	6,665425	7,111128	6,154262	5,666499	6,798255	6,317327	6,643605	6,260693667	-0,38291133	0,766888461	---
17233062	6,396714	5,490939	5,509452	4,438131	6,045779	5,765459	5,799035	5,416456333	-0,38257867	0,767065315	---
17210577	4,937976	4,847507	4,971668	4,166162	4,866367	4,577005	4,9190503	4,536511333	-0,382539	0,767086406	---
17421215	5,01283	5,137193	5,492933	4,813506	4,603647	5,078204	5,2143187	4,831785667	-0,382533	0,767089596	BC080695
17494282	4,769343	4,548421	5,163678	4,475389	4,400557	4,458838	4,8271473	4,444928	-0,38221933	0,767256393	Olfr644
17277265	7,9328	7,922496	7,855995	7,410334	7,514508	7,639833	7,9037637	7,521558333	-0,38220533	0,767263838	Rnf113a2
17275062	4,752987	4,841359	4,547709	4,523136	3,955553	4,51695	4,7140183	4,331879667	-0,38213867	0,767299294	Meox2
17514920	3,257033	3,684851	3,54428	3,169649	3,20831	2,963574	3,495388	3,113844333	-0,38154367	0,767615811	Olfr837
17249724	4,443672	4,642348	3,764618	4,133579	3,724938	3,847593	4,283546	3,902036667	-0,38150933	0,767634079	---
17208463	5,676244	5,830812	5,79219	5,310875	5,439332	5,404816	5,7664153	5,385007667	-0,38140767	0,767688176	---
17342015	8,6487	8,420949	8,79662	8,680925	7,885331	8,155999	8,6220897	8,240751667	-0,381338	0,767725248	---
17205261	4,659667	4,210738	4,930833	4,039721	3,453524	5,164009	4,6004127	4,219084667	-0,381328	0,76773057	---
17209451	4,659667	4,210738	4,930833	4,039721	3,453524	5,164009	4,6004127	4,219084667	-0,381328	0,76773057	---
17354363	3,459375	4,020081	4,06882	3,658111	3,513653	3,23353	3,8494253	3,468431333	-0,380994	0,767908328	---
17429512	4,165111	4,545468	4,303874	4,223651	3,694524	3,953572	4,338151	3,957249	-0,380902	0,767957299	Mir30c-1
17210485	4,075572	4,268749	4,277687	3,753593	3,556949	4,169845	4,207336	3,826795667	-0,38054033	0,768149841	---
17333812	3,85052	3,513008	3,626945	3,377388	3,256954	3,214656	3,663491	3,282999333	-0,38049167	0,768175754	Vmn2r101
17299158	3,65341	3,605938	3,728294	3,322028	3,43237	3,092453	3,6625473	3,282283667	-0,38026367	0,768297164	LOC100044625 // LOC100044610
17206017	5,051434	4,417684	5,164009	3,867327	5,279042	4,346232	4,877709	4,497533667	-0,38017533	0,768344207	---
17208237	4,827738	4,729505	4,864999	4,213199	4,060104	5,008532	4,807414	4,427278333	-0,38013567	0,768365333	---
17372797	6,523758	5,279288	5,044714	5,003704	5,400931	5,303476	5,61592	5,236037	-0,379883	0,768499912	Olfr1034
17548761	6,178723	5,997268	6,199682	5,725385	5,880493	5,63128	6,1252243	5,745719333	-0,379505	0,768701293	Gm10144
17406440	6,329859	6,168582	6,350231	6,048649	5,832625	5,828986	6,2828907	5,90342	-0,37947067	0,768719587	Rps3a
17464487	5,763691	6,153502	5,841121	5,58204	5,541202	5,496869	5,919438	5,540037	-0,379401	0,768756708	Dennd5b // Dennd5b
17352018	5,67316	5,534842	5,544352	5,230535	5,242102	5,141753	5,584118	5,204796667	-0,37932133	0,768799161	4930465K10Rik
17202291	3,820037	3,817407	4,317976	3,930531	3,06242	3,824993	3,98514	3,605981333	-0,37915867	0,768885849	---
17203143	4,276561	4,950306	4,679211	4,403275	4,55775	3,807826	4,6353593	4,256283667	-0,37907567	0,768930086	---
17248079	5,97338	3,585645	3,810155	4,890744	3,726886	3,6145	4,4563933	4,077376667	-0,37901667	0,768961532	---
17211258	7,369006	7,53815	7,274198	6,869153	7,092543	7,082766	7,3937847	7,014820667	-0,378964	0,768989604	Rdh10
17290682	12,2482	12,33834	12,35312	11,87263	11,95632	11,9744	12,31322	11,93445	-0,37877	0,769093018	Ywhaq // Ywhaq
17202683	3,935678	5,104537	3,62353	3,799253	3,86992	3,858478	4,2212483	3,842550333	-0,378698	0,769131401	---
17268299	4,674551	4,425387	4,839795	4,390224	4,052835	4,361936	4,6465777	4,268331667	-0,378246	0,76937241	---
17546423	3,854947	6,446579	5,463488	3,979744	6,085836	4,564953	5,2550047	4,876844333	-0,37816033	0,769418096	Ssty1
17204305	3,465108	3,453779	4,062301	3,060736	3,625518	3,160829	3,660396	3,282361	-0,378035	0,769484942	---
17205559	3,312647	4,296252	4,027781	3,443799	3,551991	3,506842	3,8788933	3,500877333	-0,378016	0,769495076	---
17373220	3,541427	3,980225	3,867249	3,354701	3,746644	3,153557	3,7963003	3,418300667	-0,37799967	0,769503788	---
17315386	7,342033	7,283498	7,218229	6,532846	7,087121	7,090623	7,2812533	6,90353	-0,37772333	0,769651192	---
17205119	4,807346	5,533933	5,115168	4,39714	5,229653	4,696696	5,152149	4,774496333	-0,37765267	0,769688893	---
17209309	4,807346	5,533933	5,115168	4,39714	5,229653	4,696696	5,152149	4,774496333	-0,37765267	0,769688893	---
17251583	5,295616	5,334327	5,857653	4,982644	5,239865	5,133231	5,4958653	5,11858	-0,37728533	0,769884893	Alox12b
17240075	5,213504	4,720647	5,214151	4,937939	4,614653	4,464656	5,049434	4,672416	-0,377018	0,770027567	---
17321661	3,454604	3,248474	3,442846	2,974957	3,188072	2,852102	3,3819747	3,005043667	-0,376931	0,770074004	---
17204839	3,716811	4,918239	4,500135	4,917632	3,821489	3,265327	4,378395	4,001482667	-0,37691233	0,770083968	---
17384334	6,326448	6,162993	6,309432	5,848151	5,817032	6,003013	6,266291	5,889398667	-0,37689233	0,770094643	Phf19
17204659	7,024608	6,710213	6,732101	6,945833	6,384583	6,00655	6,8223073	6,445655333	-0,376652	0,770222941	---
17306968	5,735995	6,077358	5,807462	5,765679	5,345311	5,379891	5,873605	5,496960333	-0,37664467	0,770226856	Mcpt8
17365001	9,5742	10,56945	10,30792	10,73357	9,287093	9,301589	10,150523	9,774084	-0,37643933	0,770336488	Cyp2c44
17290238	4,63191	4,471602	5,313972	4,34779	4,456819	4,484505	4,805828	4,429704667	-0,37612333	0,770505236	Calml3
17520073	9,022286	9,059345	8,986412	8,708947	8,575415	8,655432	9,022681	8,646598	-0,376083	0,770526778	Nt5e
17549290	8,233416	9,001641	7,677776	7,714469	8,099299	7,97255	8,3042777	7,928772667	-0,375505	0,770835543	---
17262046	6,588723	7,681977	7,312451	6,707363	6,866518	6,88308	7,1943837	6,818987	-0,37539667	0,770893428	Sft2d1
17310922	7,326746	6,565769	7,352294	7,228892	6,427108	6,463118	7,081603	6,706372667	-0,37523033	0,770982312	Matn2
17334184	4,501699	4,230834	5,024604	4,556311	4,064624	4,010527	4,5857123	4,210487333	-0,375225	0,770985162	Sbp
17291337	4,487421	4,878376	5,117963	4,52519	4,271616	4,561564	4,82792	4,45279	-0,37513	0,771035932	Gm11346
17508848	5,822762	3,904742	5,405972	4,917099	4,689661	4,402035	5,044492	4,669598333	-0,37489367	0,771162249	---
17475863	7,155039	6,706277	6,982417	6,64897	6,357442	6,712949	6,947911	6,573120333	-0,37479067	0,771217307	Il28b
17280327	10,36914	10,69187	10,09683	10,31791	9,887144	9,828852	10,385947	10,011302	-0,37464467	0,771295358	Rsad2
17208033	5,224799	4,735501	4,922086	4,290068	4,862271	4,606698	4,9607953	4,586345667	-0,37444967	0,771399616	---
17405671	3,40878	3,896341	4,346232	3,240409	3,553077	3,734863	3,8837843	3,509449667	-0,37433467	0,771461108	---
17402539	3,330931	3,559396	3,874005	3,387735	3,121347	3,132551	3,5881107	3,213877667	-0,374233	0,771515475	---
17478296	6,974168	6,718091	6,688134	6,230904	6,506433	6,520397	6,7934643	6,419244667	-0,37421967	0,771522606	Gm2788
17525548	9,336246	9,557013	9,078247	9,289443	8,777735	8,781902	9,3238353	8,949693333	-0,374142	0,771564141	Ei24
17525704	6,770283	6,060318	6,040854	5,871064	6,0223	5,856322	6,290485	5,916562	-0,373923	0,771681273	Olfr935
17273564	4,890183	4,852485	4,67826	4,366977	4,165937	4,766363	4,806976	4,433092333	-0,37388367	0,771702312	---
17238528	4,702881	4,567886	4,844082	4,707238	3,897973	4,388501	4,7049497	4,331237333	-0,37371233	0,771793964	Gm9182
17348463	7,653877	7,580297	7,644299	6,79779	7,289015	7,670544	7,6261577	7,252449667	-0,373708	0,771796282	---
17494684	6,21139	5,922232	5,619109	5,710929	5,32302	5,598233	5,917577	5,544060667	-0,37351633	0,771898825	Olfr693
17344892	5,1829	4,77288	4,766777	4,576873	4,686154	4,3401	4,907519	4,534375667	-0,37314333	0,77209842	Olfr113
17207409	5,250596	4,407692	5,491858	4,69694	4,272648	5,061249	5,0500487	4,676945667	-0,373103	0,772120006	---
17408315	4,547431	4,6709	4,688447	4,640563	4,034222	4,112703	4,6355927	4,262496	-0,37309667	0,772123396	Hsd3b5
17260907	4,850802	4,043924	4,361037	3,95355	4,120759	4,062242	4,1858877	4,045517	-0,37307067	0,772137311	---
17210685	4,54313	4,486583	4,842868	4,382246	3,988475	4,382775	4,6241937	4,251165333	-0,37302833	0,772159968	---
17207541	7,495173	7,670137	7,434273	7,137202	6,942092	7,401345	7,5331943	7,160213	-0,37298133	0,772185124	---
17207233	4,792904	5,609533	5,438958	5,205704	4,125566	5,391579	5,280465	4,907616333	-0,37284867	0,772256136	---
17547577	6,343518	6,13272	6,416853	5,863519	5,581799	6,329693	6,297697	5,925003667	-0,37269333	0,772339288	Derl2 // Derl2
17200781	4,784091	4,46507	4,948044	4,469663	4,263869	4,346232	4,7324017	4,359921333	-0,37248033	0,772453325	---
17309358	6,73218	6,442612	6,789854	6,553059	6,157526	6,136631	6,654882	6,282405333	-0,37247667	0,772455288	---
17207531	5,449999	5,632378	5,800608	5,302679	5,237227	5,226254	5,6276617	5,255386667	-0,372275	0,772563273	---
17203701	5,153553	5,433761	5,614034	4,942014	5,222518	4,920445	5,4004493	5,028325667	-0,37212367	0,772644316	---
17546212	5,203382	5,868627	4,982008	4,870946	5,056559	5,010727	5,351339	4,979410667	-0,37192833	0,772748935	G530011O06Rik
17302360	4,211967	4,983282	4,419598	4,334487	3,932012	4,232748	4,5382823	4,166415667	-0,37186667	0,772781967	---
17547525	6,71525	6,766537	6,454976	6,222303	6,356167	6,242781	6,6455877	6,273750333	-0,37183733	0,772797679	Rap1b // Rap1b
17205539	3,786085	3,123178	3,651848	3,270892	3,367237	2,808289	3,5203703	3,148806	-0,37156433	0,772943929	---
17484553	6,359356	6,106317	6,346097	6,120526	5,960141	5,616545	6,27059	5,899070667	-0,37151933	0,772968039	Olfr527
17204233	5,107424	5,654146	5,18388	5,378603	4,951525	4,501118	5,31515	4,943748667	-0,37140133	0,773031263	---
17491893	5,940657	5,609251	5,914771	5,495205	5,291585	5,563754	5,8215597	5,450181333	-0,37137833	0,773043587	Chsy1 // Chsy1
17396492	7,515958	7,795819	7,572068	7,307759	7,173241	7,289303	7,6279483	7,256767667	-0,37118067	0,773149511	Eif5a2
17207915	4,283379	3,730051	3,765442	3,446767	3,029151	4,190548	3,9262907	3,555488667	-0,370802	0,773352467	---
17301932	6,91322	6,470378	6,578777	6,330098	6,110667	6,409547	6,654125	6,283437333	-0,37068767	0,773413758	Esd
17353337	8,821458	8,91245	8,845063	8,471494	8,449453	8,546064	8,859657	8,489003667	-0,37065333	0,773432164	Gypc
17525700	4,693534	4,215453	4,184228	3,944119	4,037711	3,999821	4,364405	3,993883667	-0,37052133	0,773502933	Olfr919
17300201	4,501525	3,296967	3,51964	3,236028	3,5294	3,441872	3,7727107	3,402433333	-0,37027733	0,773633765	---
17206371	6,086649	4,457204	5,305866	4,777066	4,662604	5,299588	5,2832397	4,913086	-0,37015367	0,773700083	---
17203253	4,473149	3,99417	4,095086	3,788274	3,4873	4,1765	4,1874683	3,817358	-0,37011033	0,773723322	---
17201449	6,513647	6,096103	6,37241	6,426116	5,555468	5,890314	6,3273867	5,957299333	-0,37008733	0,773735657	---
17336964	6,790361	7,057325	6,582784	6,828942	5,864055	6,627244	6,8101567	6,440080333	-0,37007633	0,773741557	---
17208241	3,299596	3,128425	4,022921	3,125324	3,08655	3,129375	3,4836473	3,113749667	-0,36989767	0,773837385	---
17372153	5,611664	5,427535	5,991435	5,311923	5,266932	5,342237	5,676878	5,307030667	-0,36984733	0,773864383	E030042O20Rik
17204231	3,568099	4,175547	4,122402	3,445529	3,304866	4,006749	3,9553493	3,585714667	-0,36963467	0,773978466	---
17207023	4,582848	6,123526	5,273438	5,174199	4,953825	4,743373	5,326604	4,957132333	-0,36947167	0,774065918	---
17391430	7,409576	7,436199	6,69379	6,562576	7,107009	6,76184	7,179855	6,810475	-0,36938	0,774115102	---
17290072	7,650742	7,408024	8,056801	7,289066	7,291708	7,426759	7,705189	7,335844333	-0,36934467	0,774134061	---
17357837	6,440989	6,725947	5,997269	6,397906	5,71481	5,94381	6,3880683	6,018842	-0,36922633	0,77419756	Glyat
17204201	5,127959	4,641033	4,107904	4,303559	5,027123	3,438784	4,625632	4,256488667	-0,36914333	0,774242102	---
17308764	6,563469	6,693074	6,596771	6,335786	5,929193	6,481646	6,6177713	6,248875	-0,36889633	0,77437467	Serp2
17200543	6,019519	6,450466	7,2669	6,199002	6,1603	6,272256	6,5789617	6,210519333	-0,36844233	0,774618395	---
17305908	6,787352	6,316979	5,949435	6,287906	5,541052	6,119534	6,3512553	5,982830667	-0,36842467	0,774627881	---
17357861	9,508906	9,746198	9,484417	9,542974	9,090283	9,00115	9,5798403	9,211469	-0,36837133	0,774656517	Keg1
17539649	8,677666	8,597521	8,648516	7,882746	8,542164	8,394005	8,6412343	8,272971667	-0,36826267	0,774714868	Gm19528
17296128	6,726083	6,157687	6,035448	5,993361	5,718098	6,103676	6,306406	5,938378333	-0,36802767	0,774841072	Gapt
17210525	5,66489	6,180354	5,655264	5,901207	5,214182	5,281416	5,8335027	5,465601667	-0,367901	0,774909105	---
17220593	4,704239	5,052984	5,443275	4,556004	5,133466	4,40743	5,0668327	4,698966667	-0,367866	0,774927904	---
17207693	7,366115	7,337487	6,711575	6,882759	7,20973	6,219435	7,1383923	6,770641333	-0,367751	0,774989678	---
17202637	6,238915	6,29843	6,910849	6,080426	6,371268	5,893339	6,4827313	6,115011	-0,36772033	0,775006151	---
17233384	5,389231	5,359144	5,80968	4,966306	5,189837	5,298946	5,5193517	5,151696333	-0,36765533	0,77504107	Fabp7
17227826	3,951196	3,062015	3,450996	2,955564	3,3884	3,017546	3,488069	3,120503333	-0,36756567	0,775089242	---
17460261	5,158282	5,029392	5,579168	4,712417	5,112051	4,840786	5,255614	4,888418	-0,367196	0,775287871	Emx1
17526086	4,39431	3,666222	4,090211	3,704358	3,746407	3,598806	4,0502477	3,683190333	-0,36705733	0,775362392	Pou2f3

17243525	5,143478	5,682071	5,203698	4,902399	4,933115	5,092938	5,3430823	4,976150667	-0,36693167	0,775429934	Slc41a2
17293348	6,350048	6,394001	5,917602	6,01998	5,935583	5,605643	6,2205503	5,853735333	-0,366815	0,775492643	Ctla2b
17284650	3,980807	4,199025	4,741417	3,684354	3,811086	4,325766	4,307083	3,940402	-0,366681	0,775564676	---
17459693	5,792472	5,956557	6,021659	5,263844	5,769671	5,637169	5,9235627	5,556894667	-0,366668	0,775571664	Tgoln1 // Tgoln1
17414913	4,678932	4,741429	4,890603	4,56708	4,322007	4,322007	4,7703213	4,403698	-0,36662333	0,775595677	Frmd3
17202295	6,179875	6,53134	6,847641	6,065852	6,182008	6,2115	6,5196187	6,15312	-0,36649867	0,775662701	---
17428473	8,082404	8,432513	7,974856	7,705455	8,072612	7,61228	8,1632577	7,796782333	-0,36647533	0,775675246	Gm12839
17438940	8,164758	7,872272	8,22917	7,764645	7,634445	7,767719	8,0887333	7,722269667	-0,36646367	0,775681519	5830473C10Rik
17548603	4,617821	4,431583	4,537863	4,269826	4,05318	4,165556	4,529089	4,162854	-0,366235	0,775804474	Gm4884 // Gm4884
17203411	5,526877	5,206703	5,700701	5,474575	4,771781	5,089677	5,4780937	5,112011	-0,36608267	0,775886395	---
17387997	3,739529	4,248006	3,807555	3,48742	3,70025	3,509381	3,9316967	3,565683667	-0,366013	0,775923862	Olfr1254
17329772	4,74978	3,815725	4,567965	4,300152	3,759293	3,975996	4,3778233	4,011813667	-0,36600967	0,775925655	---
17469537	4,261245	3,964849	4,078238	3,797626	3,728833	3,680551	4,101444	3,73567	-0,365774	0,776052414	---
17209117	3,91745	4,425885	3,973131	3,705873	4,358708	3,154663	4,1054887	3,739748	-0,36574067	0,776070345	---
17203281	4,628229	4,610266	5,16924	4,65171	4,975473	3,683665	4,8025783	4,436949333	-0,365629	0,776130416	---
17368853	3,632706	3,754518	3,843734	3,550696	3,285125	3,298699	3,7436527	3,378173333	-0,36547933	0,776210937	---
17203167	4,921192	3,975578	4,412466	4,042188	3,990153	4,180678	4,436412	4,071006333	-0,36540567	0,776250573	---
17549376	6,823935	6,897491	6,904439	6,544745	6,486382	6,498663	6,8752883	6,50993	-0,36535833	0,776276041	---
17549752	6,823935	6,897491	6,904439	6,544745	6,486382	6,498663	6,8752883	6,50993	-0,36535833	0,776276041	---
17286585	4,339009	4,013367	4,197408	3,961952	3,6384	3,853813	4,1832613	3,818055	-0,36520633	0,776357833	---
17507888	3,347625	3,890323	3,888293	3,274656	3,396516	3,359477	3,708747	3,343549667	-0,36519733	0,776362676	Gm6040
17207279	6,145213	6,076478	6,815697	5,439961	6,408196	6,093663	6,345796	5,980606667	-0,36518933	0,776366981	---
17521256	4,6745	4,392764	4,325778	4,217283	4,046626	4,033828	4,4643473	4,099245667	-0,36510167	0,776414159	Iqcf5
17546232	4,308333	4,451962	4,667132	4,353345	4,292319	3,686496	4,475809	4,11072	-0,365089	0,776420976	Zf12
17206323	4,187224	4,031204	4,27542	4,102722	3,620571	3,675833	4,164616	3,799708667	-0,36490733	0,77651875	---
17299703	5,041551	5,283149	5,277216	4,639385	4,68611	5,182667	5,2006387	4,836054	-0,36458467	0,776692443	G630016D24Rik
17293793	5,097	4,937838	4,275326	4,63925	3,96904	4,608516	4,7700547	4,405602	-0,36445267	0,77676351	Gm7762 // Gm7762
17549814	5,565309	5,696683	5,693827	5,398181	5,230196	5,234191	5,6519397	5,287522667	-0,364417	0,776782713	---
17201151	5,572439	7,572928	5,24378	5,305888	5,960168	6,029957	6,1297157	5,765337667	-0,364378	0,776803712	---
17313860	4,027037	3,473719	4,120412	3,289673	3,628716	3,609703	3,8737227	3,509364	-0,36435867	0,776814122	Mirlet7b
17239501	6,859052	7,030082	6,941779	6,53601	6,353497	6,849843	6,9436377	6,579783333	-0,36385433	0,777085726	3110003A17Rik
17246065	6,005412	5,872856	5,69768	5,29288	5,575966	5,615742	5,8586493	5,494862667	-0,36378667	0,777122174	Ptges3
17202773	3,310284	3,153102	3,609703	3,255649	2,915716	2,812132	3,3576963	2,994499	-0,36319733	0,77743969	---
17470625	4,437759	4,331575	5,011458	4,462921	4,048983	4,179765	4,5935973	4,230556333	-0,363041	0,777523939	---
17210719	4,737647	5,007382	5,410403	5,284024	4,860053	3,922327	5,0518107	4,688801333	-0,36300933	0,777541006	---
17207041	6,28886	6,333254	6,285777	5,954101	6,125073	5,739874	6,3026303	5,939682667	-0,36294767	0,777574242	---
17481632	3,878448	4,325435	3,650602	4,052915	3,210009	3,503244	3,951495	3,588722667	-0,36277233	0,777668747	Olfr510
17208879	6,296407	6,704655	6,679018	6,425925	6,085185	6,080811	6,5600267	6,197307	-0,36271967	0,777697137	---
17209729	3,514597	4,083532	4,581234	3,865617	3,447539	3,778249	4,0597877	3,697135	-0,36265267	0,777733255	---
17207883	3,795131	3,824038	3,766628	3,91022	3,141834	3,245885	3,7952657	3,432646333	-0,36261933	0,777751225	---
17432470	4,284867	3,985919	4,159636	3,735244	3,887796	3,720264	4,143474	3,781101333	-0,36237267	0,777884213	Gm13089
17244085	6,487049	7,185349	7,40871	7,055808	6,518203	6,419991	7,027036	6,664667333	-0,36236867	0,77788637	Slc17a8
17204147	5,805973	6,091962	6,026604	5,519024	6,06886	5,249714	5,9748463	5,612532667	-0,36231367	0,777916026	---
17511748	8,642002	7,91409	7,251556	6,854311	7,199489	8,667045	7,9358827	7,573615	-0,36226767	0,77794083	Ces1g
17358132	7,880586	8,044009	7,874272	7,322202	7,682208	7,70832	7,9329557	7,57091	-0,36204567	0,778060548	Fam108b
17235743	4,416635	3,79044	4,417996	3,917816	3,854292	3,767583	4,208357	3,846563667	-0,36179333	0,778196646	Celf5 // Celf5
17206131	3,989276	4,199339	4,527143	4,000453	3,917915	3,713535	4,238586	3,877301	-0,361285	0,778470891	---
17205677	4,990038	5,685715	6,088903	4,629197	6,121695	4,930001	5,5882187	5,226964333	-0,36125433	0,778487439	---
17257049	7,085925	6,401258	7,035625	6,439035	6,409767	6,59029	6,840936	6,479697333	-0,36123867	0,778495893	---
17460773	4,091471	3,494044	3,773683	3,288955	3,541413	3,446025	3,7863993	3,425464333	-0,360935	0,778659772	Vmn1r54
17537520	4,263799	4,22962	4,560742	4,004397	4,023417	3,944407	4,351387	3,990740333	-0,36064667	0,778815409	Gm382
17532489	9,867499	10,28184	9,607319	9,384124	9,504348	9,786707	9,918886	9,558393	-0,360493	0,778898368	Gm19491
17206047	3,079727	2,882516	3,152509	2,708583	2,877178	2,447624	3,0382507	2,677795	-0,36045567	0,778918524	---
17538060	5,779159	6,108335	6,031461	5,527057	5,875022	5,435662	5,972985	5,612580333	-0,36040467	0,77894606	Mum1l1
17339852	3,971106	4,327268	4,417437	3,459524	4,237126	3,937989	4,2386037	3,878213	-0,36039067	0,778953619	---
17319968	5,160462	5,49034	5,031636	4,891596	4,713735	4,996035	5,2274793	4,867122	-0,36035733	0,778971616	Pnpla5
17422215	5,355773	4,859691	4,844522	4,715545	4,800422	4,463783	5,0199953	4,659916667	-0,36007867	0,779122095	---
17232509	3,363384	3,734173	3,365531	2,912247	3,401083	3,069716	3,487696	3,127682	-0,360014	0,779157019	---
17544826	4,349982	4,08351	4,375927	3,775226	4,015671	3,938828	4,2698063	3,909908333	-0,359898	0,779219669	---
17205865	6,644101	6,633565	7,496023	6,595944	6,628385	6,470128	6,924563	6,564819	-0,359744	0,779302851	---
17448744	8,079601	7,860188	8,003875	6,679787	8,206307	7,978358	7,9812213	7,621484	-0,35973733	0,779306452	---
17482943	7,679468	8,099983	7,942829	7,597194	7,566914	7,480087	7,9074267	7,548065	-0,35936167	0,779509404	Il4ra
17498551	5,075606	4,767696	4,941391	4,667663	4,399633	4,639709	4,928231	4,569001667	-0,35922933	0,779580909	---
17326799	5,133561	5,164009	4,265417	4,205884	4,639754	4,639754	4,854329	4,495130667	-0,35919833	0,779597661	---
17423191	4,628063	3,961172	4,31565	4,025052	3,956276	3,846033	4,3016283	3,942453667	-0,35917467	0,77961045	Naip3 // Naip3
17499622	4,302362	4,185813	4,249534	3,581943	3,87674	4,201794	4,245903	3,886825667	-0,35907733	0,779663049	Defb7
17208757	5,311628	5,711609	5,95745	5,902941	4,809559	5,191259	5,660229	5,301253	-0,358976	0,779717814	---
17356339	7,194003	7,040211	7,084036	6,693512	6,748902	6,80048	7,1060833	6,747631333	-0,358452	0,780001066	B3gnt1
17288309	5,103631	4,791663	4,698709	4,465417	5,001252	4,052051	4,8646677	4,50624	-0,35842767	0,780014222	Zfp455
17515084	7,424109	7,70422	7,835937	7,356478	7,263971	7,268551	7,6547553	7,296333333	-0,358422	0,780017285	Icam4
17257041	3,300214	3,783552	4,579696	4,239092	2,913058	3,436135	3,8878207	3,529428333	-0,35839233	0,780033325	---
17448798	7,467783	7,420846	7,08946	7,108752	6,526515	7,268551	7,3260297	6,967939333	-0,35809033	0,780196627	---
17200411	6,174067	4,764122	6,803808	5,282175	5,716454	5,669172	5,913999	5,555933667	-0,35806533	0,780210147	---
17268098	3,507063	3,662758	4,29006	3,614238	3,435894	3,336516	3,8199603	3,462216	-0,35774433	0,780383763	---
17225259	3,985804	3,709186	3,468009	3,288145	3,494499	3,307219	3,7209997	3,363287667	-0,357712	0,780401253	---
17210341	6,734681	6,255811	5,734449	6,171768	5,795587	5,685966	6,241647	5,884440333	-0,35720667	0,780674653	---
17357930	5,729551	5,785739	5,5438	5,185611	5,172624	5,629483	5,6863633	5,329239333	-0,357124	0,780719387	Olfr1487
17204843	5,55685	5,397799	5,989798	5,555008	5,089181	5,22894	5,648149	5,291043	-0,357106	0,780729128	---
17294559	6,070652	6,017591	6,005463	5,81106	5,662535	5,549048	6,0312353	5,674214333	-0,357021	0,780775127	1700023H06Rik
17204497	6,616101	5,294297	5,199592	6,085321	5,245146	4,708467	5,70333	5,346311333	-0,35701867	0,78077639	---
17207995	3,022586	2,784982	3,535102	2,588704	2,883439	2,799537	3,1142233	2,757226667	-0,35699667	0,780788297	---
17201801	4,877327	3,560618	4,489148	4,193872	3,600013	4,062256	4,309031	3,952047	-0,356984	0,780795152	---
17201977	4,877327	3,560618	4,489148	4,193872	3,600013	4,062256	4,309031	3,952047	-0,356984	0,780795152	---
17204169	4,16083	4,505518	3,888815	3,46979	3,960695	4,054984	4,1850543	3,828489667	-0,35656467	0,781022131	---
17204367	6,988834	7,274287	7,122896	6,995646	6,472276	6,848408	7,1286723	6,77211	-0,35656233	0,781023394	---
17201831	5,576264	5,041945	5,680354	5,301699	4,797565	5,130687	5,4328543	5,076650333	-0,356204	0,781217407	---
17297885	4,866769	4,482747	4,832481	4,345891	4,378123	4,389479	4,7273323	4,371164333	-0,356168	0,781236901	---
17219662	6,418021	8,118077	6,58264	7,191379	6,00541	6,8535	7,0395793	6,683429667	-0,35614967	0,781246829	Pyhin1
17265526	3,866425	3,669214	3,87581	3,496031	3,453652	3,393389	3,8038163	3,447690667	-0,35612567	0,781259825	Nlrp1c
17207301	5,141205	5,626237	5,017129	5,144206	4,502758	5,069306	5,2615237	4,905423333	-0,35610033	0,781273544	---
17208493	6,115016	6,319887	6,081088	6,024674	5,386973	6,0372	6,171997	5,816282333	-0,35571467	0,781482425	---
17257061	10,10375	10,05305	9,515414	9,874247	8,687743	10,04351	9,890738	9,535166667	-0,35557133	0,78156007	---
17466919	7,668899	8,25198	7,313354	6,76184	7,59441	7,811516	7,7447443	7,389255333	-0,355489	0,781604674	---
17508938	10,33335	10,55532	10,04378	10,59951	9,529497	9,737032	10,310817	9,955346333	-0,35547033	0,781614787	Fgl1
17203935	6,932024	6,840704	6,915249	6,605453	6,327099	6,689022	6,8959923	6,540524667	-0,35546767	0,781616232	---
17209171	3,608958	4,198337	4,09365	3,453762	3,838648	3,54223	3,9669817	3,611546667	-0,355435	0,78163393	---
17414038	3,335608	3,340139	2,962991	2,860514	2,892304	2,819802	3,2129127	2,85754	-0,35537267	0,781667702	---
17347234	8,194646	8,218426	8,224831	7,637336	8,142127	7,792568	8,2126343	7,857343667	-0,35529067	0,781712132	Gm9349
17495300	9,162814	8,893935	8,82756	8,603198	8,844961	8,371347	8,9614363	8,606502	-0,35493433	0,781905232	---
17547454	5,736159	6,595337	6,182303	5,363538	6,364805	5,721358	6,1712663	5,816567	-0,35469933	0,782032607	---
17233271	4,175529	3,695136	3,715779	3,598226	3,489725	3,434531	3,862148	3,507494	-0,354654	0,782057181	---
17408336	10,63572	10,65242	10,5387	10,37784	10,02142	10,36396	10,608947	10,25440667	-0,35454	0,78211898	Hsd3b3
17459309	3,822787	3,453524	4,570569	3,85568	3,432178	3,496	3,94896	3,594619333	-0,35434067	0,782227051	---
17550458	7,547723	7,556657	7,548079	7,361213	7,048973	7,179331	7,5508197	7,196505667	-0,354314	0,78224151	---
17288316	5,220904	5,357563	5,243992	5,028765	4,957366	4,773558	5,274153	4,919896333	-0,35425667	0,782272597	F630042J09Rik
17205845	5,871147	6,039822	5,982178	5,768469	5,956845	5,105202	5,9643823	5,610172	-0,35421033	0,782297721	---
17547203	4,64473	5,763834	5,232589	4,572996	5,585044	4,420712	5,2137177	4,859584	-0,35413367	0,782339294	LOC434960
17549798	6,830794	6,999408	6,717873	6,465245	6,375119	6,645797	6,8493583	6,495387	-0,35397133	0,782427329	---
17202401	6,29702	6,866237	7,303358	6,510157	6,245087	6,650439	6,822205	6,468561	-0,353644	0,782604874	---
17326453	4,371801	4,542119	4,335805	4,028665	4,109353	4,050815	4,416575	4,062944333	-0,35363067	0,782612107	Gabrr3
17483840	6,983303	7,004982	6,959457	6,678243	6,500966	6,708331	6,9825807	6,62918	-0,35340067	0,782736884	---
17257046	5,872575	5,888697	5,545981	4,824097	5,06623	6,356772	5,7690843	5,415699667	-0,35338467	0,782745564	---
17201799	5,712327	5,445876	5,37928	5,496984	4,576435	5,404404	5,5124943	5,159274333	-0,35322	0,782834911	---
17201975	5,712327	5,445876	5,37928	5,496984	4,576435	5,404404	5,5124943	5,159274333	-0,35322	0,782834911	---
17205621	4,389138	5,307143	5,249224	3,941115	5,107585	4,837191	4,981835	4,628630333	-0,35320467	0,782843231	---
17513974	8,508965	9,267122	8,958247	8,084066	8,734219	8,856577	8,9114447	8,558287333	-0,35315733	0,782868916	---
17202311	5,001829	4,9617	4,594927	4,206025	4,879584	4,413891	4,8528187	4,499833333	-0,35298533	0,782962256	---
17364403	12,15337	12,34355	12,44497	12,00122	11,93191	11,95041	12,313963	11,96118	-0,35278333	0,783071891	Cyp2c67
17372773	3,879949	4,432453	3,907265	3,818197	3,630863	3,713281	4,0732223	3,720780333	-0,352442	0,783257183	Olfr1023
17472056	4,476213	4,285463	4,313907	3,910363	3,923857	4,184566	4,3585277	4,006262	-0,35226567	0,783352922	Gprc5d
17548508	4,90663	5,573162	4,781526	4,51322	4,707816	4,983969	5,087106	4,735001667	-0,35210433	0,783440528	LOC100505136
17200941	3,751161	3,843123	3,680748	3,732809	2,820453	3,666219	3,758344	3,406493667	-0,35185033	0,783578472	---
17539611	6,706803	6,908151	6,788727	6,376481	6,462602	6,509252	6,801227	6,449445	-0,351782	0,783615587	Gpm6b
17205235	4,343791	6,084641	6,038531	4,978824	5,393098	5,040439	5,4889877	5,137453667	-0,351534	0,783750303	---
17209425	4,343791	6,084641	6,038531	4,978824	5,393098	5,040439	5,4889877	5,137453667	-0,351534	0,783750303	---
17416756	5,429326	5,152545	4,741261	5,224442	4,003945	5,040438	5,1077107	4,756275	-0,35143567	0,783803724	---
17481256	5,301044	4,79051	4,703128	4,541625	4,447451	4,75167	4,9315607	4,580248667	-0,351312	0,783870914	Olfr641
17239473	6,06669	5,860209	6,204113	5,835484	5,968136	5,273692	6,0436707	5,692437333	-0,35123333	0,783913658	---
17483058	4,834748	4,866275	5,297643	4,692148	4,635744	4,617122	4,9995553	4,648338	-0,35121733	0,783922352	Gm20367
17288246	4,090499	3,837767	3,804519	3,632602	3,644072	3,402483	3,9109283	3,559719	-0,35120933	0,783926699	LOC100861707 // LOC100861707
17387975	3,385272	3,469317	3,326248	2,979912	3,367236	2,78055	3,3936123	3,042566	-0,35104633	0,784015274	Olfr1248
17357788	3,591717	3,57983	3,343364	3,209145	3,055012	3,197803	3,5049703	3,153986667	-0,35098367	0,784049331	Olfr1431
17546995	3,507142	4,528894	3,551465	3,064258	4,07333	3,397015	3,8625003	3,511534333	-0,350966	0,784058932	---
17448760	3,994832	4,510143	4,930833	5,649445	3,744872	2,988973	4,4786027	4,127763333	-0,35083933	0,784127774	---

17248847	5,804302	5,114207	5,568102	4,989076	5,418772	5,026427	5,495537	5,144758333	-0,35077867	0,784160748	Havcr1
17200389	3,76852	3,829554	3,542536	3,232523	3,476403	3,379622	3,7135367	3,362849333	-0,35068733	0,784210393	---
17346344	6,063274	6,083364	6,281519	6,019934	5,569031	5,787562	6,142719	5,792175667	-0,35054333	0,784288671	Nrtn
17394878	3,753542	3,494044	3,591419	3,314621	3,160509	3,312878	3,6130017	3,262669333	-0,35033233	0,784403385	---
17399008	6,664071	6,577813	6,539662	6,284355	6,259997	6,186335	6,5938487	6,243562333	-0,35028633	0,784428396	Mir3093
17288872	6,166556	6,169992	5,851057	5,457736	5,683645	5,995896	6,062535	5,712425667	-0,35010933	0,784524641	---
17336944	5,071432	4,426515	5,371119	4,391096	4,263869	5,164009	4,9563553	4,606324667	-0,35003067	0,784567421	---
17344835	5,408911	4,306741	4,307804	4,28954	4,138806	4,545197	4,6744853	4,324514333	-0,349971	0,784599869	Olfr92
17203243	5,661734	7,003394	5,996445	5,597783	5,983031	6,031121	6,2205243	5,870645	-0,34987933	0,784649723	---
17299893	4,262377	4,45778	4,532857	4,154536	3,659742	4,389343	4,4176713	4,067873667	-0,34979767	0,784694141	Trav14d-3-dv8
17294685	5,367204	4,822056	5,275166	4,673485	4,760034	4,982645	5,1548087	4,805388	-0,34942067	0,784899221	---
17548671	5,373801	4,925963	4,874051	4,746816	4,643777	4,735726	5,0579383	4,708773	-0,34916533	0,785038148	Gm19274
17531701	7,845726	8,241259	7,930922	7,509273	7,955292	7,506091	8,005969	7,656885333	-0,34908367	0,785082588	Gm20317
17338353	6,601449	7,127206	7,153835	6,550127	6,545209	6,739929	6,96083	6,611755	-0,349075	0,785087304	---
17204361	8,053018	7,890823	7,892388	7,882035	7,489276	7,418393	7,9454097	7,596568	-0,34884167	0,78521429	---
17348458	10,21976	10,34891	10,12988	10,05227	9,419979	10,17991	10,23285	9,884053	-0,348797	0,785238601	---
17374316	4,832698	5,131185	4,491467	4,56165	4,522367	4,325189	4,81845	4,469735333	-0,34871467	0,785283415	---
17428876	6,059676	6,944724	6,415411	6,360875	6,386966	5,625984	6,4732703	6,124608333	-0,348662	0,785312083	---
17543742	4,143964	4,419941	4,328732	3,906591	3,955552	3,984603	4,2975457	3,948915333	-0,34863033	0,785329321	---
17475205	7,699434	8,206307	7,991645	7,866383	7,631942	7,353339	7,9657953	7,617221333	-0,348574	0,785359986	---
17221434	4,652743	4,241774	5,196337	4,20642	4,177034	4,661709	4,6969513	4,348387667	-0,34856367	0,785365611	---
17474916	6,368467	5,63641	6,417356	6,12513	5,377029	5,874453	6,1407443	5,792204	-0,34854033	0,785378313	Zfp108
17318105	8,279142	6,076181	6,038506	7,953752	5,87125	5,523754	6,797943	6,449585333	-0,34835767	0,78547776	I830127L07Rik
17547608	8,866884	9,352812	8,843674	8,246699	8,765496	9,006634	9,0211233	8,672943	-0,34818033	0,785574316	Actg1 // Actg1
17364426	8,289187	8,809635	8,705538	8,203789	8,385572	8,170773	8,6014533	8,253378	-0,34807533	0,785631492	Cyp2c40
17342827	5,624017	6,133173	6,059307	5,605484	5,646245	5,520979	5,9388323	5,590902667	-0,34792967	0,78571082	Scube3
17277685	4,8963	4,306604	3,766351	3,934104	3,901957	4,090763	4,323085	3,975608	-0,347477	0,785957387	Oog1
17520722	6,12509	6,159383	6,409621	5,917715	5,921289	5,812877	6,2313647	5,883960333	-0,34740433	0,785996976	Dbr1
17372269	3,90613	4,023417	3,977294	3,816982	3,329445	3,718661	3,968947	3,621696	-0,347251	0,786080518	---
17549398	4,667208	4,640377	4,286558	4,14756	4,291937	4,113029	4,531381	4,184175333	-0,34720567	0,786105219	---
17549784	4,667208	4,640377	4,286558	4,14756	4,291937	4,113029	4,531381	4,184175333	-0,34720567	0,786105219	---
17210687	5,321312	4,99151	5,083676	4,982644	4,165556	5,206965	5,132166	4,785055	-0,347111	0,786156803	---
17202475	3,094144	2,986575	3,466261	2,710862	2,870067	2,925659	3,1823267	2,835529333	-0,34679733	0,786327746	---
17332166	7,881868	7,992144	8,085311	7,667343	7,479679	7,771946	7,986441	7,639656	-0,346785	0,786334468	Donson
17465620	7,336318	7,982867	7,553016	7,443387	7,063529	7,325321	7,624067	7,277412333	-0,34665467	0,786405509	Podxl
17446285	6,024143	5,907541	6,024512	5,661956	5,576184	5,678132	5,9853987	5,638757333	-0,34664133	0,786412777	Nub1
17363429	6,849098	6,251809	6,311318	6,013274	6,119477	6,240396	6,4707417	6,124382333	-0,34635933	0,78656651	Aldh1a7
17200361	5,058295	4,625798	4,699818	4,503368	4,239974	4,601976	4,794637	4,448439333	-0,34619767	0,786654657	---
17249921	8,45259	8,345105	8,663396	8,086148	7,841138	8,49586	8,4870303	8,141048667	-0,34598167	0,786772443	---
17317046	7,807382	7,93675	7,631747	7,494627	7,322526	7,520859	7,7919597	7,446004	-0,34595567	0,786786623	Tnfrsf11b
17205679	4,759291	4,866898	5,088841	4,513649	4,385339	4,778601	4,90501	4,559196333	-0,34581367	0,786864067	---
17476943	4,677302	4,525954	4,529571	4,336957	4,24654	4,113008	4,577609	4,232168333	-0,34544067	0,787067533	Gm6820
17513856	8,343876	8,485271	8,397912	8,094755	8,136294	7,960146	8,4090197	8,063731667	-0,345288	0,787150825	Rpl13 // Rpl13
17359966	8,200597	8,692692	8,865285	8,060814	8,393126	8,269229	8,5861913	8,241056333	-0,345135	0,787234308	---
17489558	3,87585	3,789016	3,419624	3,487833	3,238052	3,323647	3,69483	3,349844	-0,344986	0,787315617	Abpz
17366744	7,975274	7,268551	7,634708	7,695526	7,018228	7,130896	7,6261777	7,28155	-0,34462767	0,787511193	Mir669a-3 // Mir669a-3
17503116	5,469497	4,778176	4,98223	4,769619	4,552163	4,874456	5,0766343	4,732079333	-0,344555	0,78755086	Mir23a
17206545	6,614793	6,447314	7,111644	6,794991	6,159309	6,186085	6,7245837	6,380128333	-0,34445533	0,787605268	---
17205371	6,381358	6,548941	6,612298	6,125457	6,13064	6,253691	6,514199	6,169929333	-0,34426967	0,787706635	---
17507571	4,451073	4,15933	4,735223	4,108672	4,124203	4,080595	4,448542	4,10449	-0,344052	0,787825489	Mir1968
17547475	5,872152	6,80997	7,059585	5,905975	6,923976	5,880751	6,580569	6,236900667	-0,34366833	0,78803503	LOC100039675
17200989	7,794554	7,715694	7,365753	6,984056	7,330641	7,530392	7,6253337	7,281696333	-0,34363733	0,788051963	---
17285240	6,783478	5,80086	6,422791	6,014428	6,043053	5,919	6,3357097	5,992160333	-0,34354933	0,788100033	---
17285944	8,890628	9,00944	8,615224	8,593926	8,244204	8,646725	8,8384307	8,494951667	-0,343479	0,788138455	Cmah
17442534	6,375741	6,336184	6,535981	6,064031	6,166472	5,987057	6,4159687	6,07252	-0,34344867	0,788155026	Ddx55
17368096	5,523774	5,649509	5,605958	5,344962	5,199761	5,204394	5,5930803	5,249705667	-0,34337467	0,788195454	Edf1
17204425	6,05917	5,934653	4,999248	4,889047	6,265994	4,807989	5,664357	5,32101	-0,343347	0,788210569	---
17203257	5,668638	5,546233	5,736159	5,771633	5,209925	4,939492	5,6503433	5,307016667	-0,34332667	0,788221678	---
17207949	4,349611	3,865736	4,346052	4,142323	4,078713	3,310524	4,187133	3,843853333	-0,34327967	0,788247357	---
17494940	6,067382	5,676972	5,927615	5,742959	5,605176	5,294482	5,8906563	5,547539	-0,34311733	0,788336057	Nrip3
17457983	5,207921	4,877472	5,295537	4,847539	4,610119	4,894416	5,1269767	4,784024667	-0,342952	0,788426405	Olfr447
17301920	4,747343	5,15926	4,93292	4,177444	4,842048	4,792137	4,9465077	4,603876333	-0,34263133	0,788601668	4933402J15Rik
17208347	6,902984	6,920951	7,151119	6,995333	6,773084	6,179036	6,9916847	6,649151	-0,34253367	0,788655056	---
17467298	5,489077	5,47483	5,45787	5,173948	4,917508	5,302735	5,4739257	5,131397	-0,34252867	0,788657789	Vmn1r34
17339908	3,399019	3,341798	3,426329	2,841087	3,0855	3,213126	3,3890487	3,046571	-0,34247767	0,788685669	---
17519952	4,541769	4,247465	4,439207	3,877448	4,205776	4,118444	4,4094803	4,067222667	-0,34225767	0,788805947	---
17349549	7,992907	8,448233	8,045582	7,774163	7,809976	7,876061	8,1622407	7,820066667	-0,342174	0,788851694	Mir1949
17414533	4,042883	4,189877	3,570858	3,524597	3,531302	3,721197	3,9345393	3,592365333	-0,342174	0,788851694	---
17547178	3,96208	4,602255	5,216337	4,178652	4,553637	4,02216	4,5935573	4,251483	-0,34207433	0,788906192	---
17202791	5,92822	5,736159	5,276218	5,241523	6,048852	4,624	5,6468657	5,304791667	-0,342074	0,788906375	---
17424468	5,433463	4,577622	5,377866	5,205987	4,467958	4,688884	5,1296503	4,787609667	-0,34204067	0,788924602	---
17473852	4,151585	3,998739	3,743591	3,757085	3,367237	3,743591	3,9646383	3,622637667	-0,34200067	0,788946476	Vmn1r88
17546563	5,741649	6,665719	6,792527	5,678843	6,753435	5,741811	6,399965	6,058029667	-0,34193533	0,788982205	LOC100862046
17546635	5,741649	6,665719	6,792527	5,678843	6,753435	5,741811	6,399965	6,058029667	-0,34193533	0,788982205	---
17344990	8,039632	8,104448	7,857948	7,487532	7,822615	7,666463	8,000676	7,65887	-0,341806	0,789052938	Cenpq
17344287	10,08478	10,15421	9,507099	9,663262	9,59819	9,461046	9,915363	9,574166	-0,341197	0,789386089	---
17205003	4,211115	5,15966	4,718209	4,363456	4,082286	4,619659	4,696328	4,355133667	-0,34119433	0,789387548	---
17239464	5,891215	6,674886	6,570187	5,809862	6,37525	5,927727	6,3787627	6,037613	-0,34114967	0,789411988	---
17203697	6,433782	4,990346	5,71664	5,350201	5,537616	5,229667	5,7135893	5,372494667	-0,34109467	0,789442083	---
17536233	4,916741	4,819882	5,004572	4,456454	4,764421	4,497418	4,9137317	4,572764333	-0,34096733	0,789511763	Gm5941 // Gm5941
17201745	5,27785	4,964174	4,889867	4,478314	4,765137	4,866202	5,0439637	4,703217667	-0,340746	0,789632897	---
17201921	5,27785	4,964174	4,889867	4,478314	4,765137	4,866202	5,0439637	4,703217667	-0,340746	0,789632897	---
17306123	5,771152	5,881772	5,852318	5,584812	5,656955	5,241523	5,8350807	5,49443	-0,34065067	0,789685077	---
17548589	6,232214	6,140333	5,808242	5,836099	5,544584	5,778229	6,060263	5,719637333	-0,34062567	0,789698762	Gm19288
17201285	6,033165	5,833049	6,314314	5,774744	5,687778	5,697066	6,060176	5,719862667	-0,34031333	0,789869744	---
17536665	5,352719	5,379966	5,168669	5,113263	4,901548	4,86578	5,3004513	4,960197	-0,34025433	0,789902047	Gm19763
17209713	5,475402	5,910081	6,178626	5,512707	5,66399	5,3668	5,854703	5,514499	-0,340204	0,789929606	---
17207215	5,268176	5,343771	5,673406	5,241523	4,948703	5,074634	5,428451	5,088286667	-0,34016433	0,789951326	---
17495320	4,868553	4,917215	5,04896	4,686826	4,397794	4,729761	4,9449093	4,604793667	-0,34011567	0,789977974	Cyp2r1
17415796	3,853168	3,734679	4,306569	3,642532	3,814064	3,417726	3,9648053	3,624774	-0,34003133	0,790024153	---
17200785	4,896333	4,009559	5,127446	4,4753	3,744872	4,794018	4,6777793	4,338063333	-0,339716	0,79019685	---
17265796	4,0409	3,648351	3,906828	3,569602	3,488064	3,51964	3,8653597	3,525768667	-0,339591	0,790265318	Olfr393
17448133	4,150578	3,518406	3,580798	3,276698	3,652288	3,302023	3,7499273	3,410336333	-0,339591	0,790265318	---
17547191	5,067517	5,838879	5,629042	4,787853	5,904693	4,824123	5,5118127	5,172223	-0,33958967	0,790266048	---
17226127	3,930489	3,657953	3,874862	3,526118	3,385422	3,533047	3,8211013	3,481529	-0,33957233	0,790275543	Cdh19
17459929	5,26367	4,955991	5,259908	4,788288	4,868121	4,805486	5,1598563	4,820631667	-0,33922467	0,79046601	Lbx2
17547141	5,75801	6,850803	7,072972	5,844037	6,991787	5,828448	6,560595	6,221424	-0,339171	0,790495415	LOC100862046
17473399	8,873563	9,047739	9,030037	8,418545	8,667865	8,847467	8,9837797	8,644625667	-0,339154	0,79050473	---
17506322	5,624467	5,686661	5,711806	5,217802	5,243231	5,544703	5,6743113	5,335245333	-0,339066	0,79055295	Klhdc4
17200305	5,290533	5,346574	5,46117	5,158664	5,214894	4,707663	5,3660923	5,027073667	-0,33901867	0,790578887	---
17209827	3,758643	3,576563	3,143593	3,013556	3,52345	2,924781	3,492933	3,153929	-0,339004	0,790586925	---
17409688	6,461979	5,89938	6,380157	5,983663	5,879547	5,861393	6,247172	5,908201	-0,338971	0,790605009	---
17288227	3,816697	3,403685	3,414408	3,324011	3,176887	3,117007	3,54493	3,205968333	-0,33896167	0,790610123	---
17208999	3,978065	4,419751	4,146762	3,435677	3,86404	4,228007	4,181526	3,842574667	-0,33895133	0,790615786	---
17208825	4,916502	4,133896	4,282413	3,752614	4,349786	4,213677	4,4442703	4,105359	-0,33891133	0,790637707	---
17323063	6,90314	6,248388	5,997268	6,248388	6,448824	5,435662	6,382932	6,044291333	-0,33864067	0,790786054	---
17207603	4,456587	5,628623	5,237361	5,510605	3,859538	4,936735	5,1075237	4,768959333	-0,33856433	0,790827896	---
17209003	6,916395	6,758341	7,645782	7,524345	5,690275	7,090607	7,1068393	6,768409	-0,33843033	0,790901353	---
17217619	6,881087	6,651556	7,057325	6,759373	6,107682	6,707659	6,8633227	6,524904667	-0,338418	0,790908114	Phlda3
17282117	8,921251	9,391249	9,206374	8,823892	8,779044	8,90073	9,172958	8,834555333	-0,33840267	0,79091652	Plek2
17205081	5,725026	5,103883	5,425834	5,164264	5,13731	4,938834	5,4182477	5,080136	-0,33811167	0,791076068	---
17209271	5,725026	5,103883	5,425834	5,164264	5,13731	4,938834	5,4182477	5,080136	-0,33811167	0,791076068	---
17207813	4,477979	4,280478	4,192734	4,343143	3,580627	4,013295	4,3170637	3,979021667	-0,338042	0,79111427	---
17414647	3,628222	3,737355	3,476503	3,623384	3,18189	3,023961	3,6140267	3,276411667	-0,337615	0,791348454	---
17249990	8,975336	10,04789	8,738088	8,843466	8,886326	9,019295	9,2537713	8,916362333	-0,337409	0,791461457	Irgm2
17440719	5,618834	5,652202	5,933402	5,408072	5,310509	5,474012	5,7348127	5,397531	-0,33728167	0,791531315	Tmem211
17208929	7,310109	6,950644	7,398318	6,948834	6,262346	7,436603	7,2196903	6,882594333	-0,337096	0,791633187	---
17471464	4,798052	4,852719	4,809618	4,577389	4,536808	4,335325	4,8201297	4,483174	-0,33695567	0,791710194	Klrb1b
17203525	6,631489	6,628807	5,591803	6,481901	5,482987	5,877139	6,284033	5,947342333	-0,33669067	0,791855632	---
17337948	5,300032	6,271054	5,400462	5,224754	5,152709	5,58429	5,6571827	5,320584333	-0,33659833	0,791906313	---
17511548	4,652001	4,162959	4,311554	4,216405	3,800605	4,099709	4,3755047	4,038906333	-0,33659833	0,791906313	---
17366728	5,00851	4,766254	4,345538	4,554576	4,214612	4,34142	4,7067673	4,370202667	-0,33656467	0,791924793	---
17258573	5,312817	4,914426	5,405866	4,634841	5,039568	4,949108	5,2110363	4,874505667	-0,33653067	0,791943457	Fam100b
17227892	4,611147	4,686151	4,779788	4,228396	4,355389	4,483763	4,692362	4,355849333	-0,33651267	0,791953338	Prg4
17481135	5,511044	5,673234	5,762114	5,442018	5,269454	5,22596	5,6487973	5,312477333	-0,33632	0,792059107	Olfr554
17207743	5,286471	4,146787	4,360623	4,716473	4,021402	4,047083	4,5979603	4,261652667	-0,33630767	0,792065878	---
17200745	4,35734	4,897812	4,708899	4,987284	4,207695	3,760497	4,6546837	4,318492	-0,33619167	0,792129567	---
17398653	5,152496	5,21717	5,437471	4,872296	4,967282	4,959021	5,2690457	4,932866333	-0,33617933	0,792136339	---
17404513	5,810418	6,22504	5,899924	5,435661	5,970034	5,52217	5,9784607	5,642621667	-0,335839	0,792323227	1600017P15Rik
17395053	4,86711	4,181606	4,553457	4,2864	4,390672	3,917615	4,5340577	4,198229	-0,33582867	0,792328902	LOC100862165
17286190	4,820622	4,529478	4,640336	4,370371	4,238616	4,373971	4,6634787	4,327652667	-0,335826	0,792330366	Prl2a1
17458016	3,814742	3,270892	3,250921	3,170646	3,170646	2,988973	3,4455183	3,110088333	-0,33543	0,79254788	Olfr47
17291062	7,5205	7,848322	8,172071	7,750397	7,25199	7,53223	7,8469643	7,511539	-0,33542533	0,792550444	---
17209163	6,751997	6,394389	6,413263	6,408709	6,045202	6,100322	6,519883	6,184744333	-0,33513867	0,792707941	---

17204391	7,201166	7,165446	7,274277	6,977565	6,655749	7,002555	7,2136297	6,878623	-0,33500667	0,792780473	---
17475195	5,372999	5,195484	5,146496	5,117509	5,01295	4,579712	5,2383263	4,903390333	-0,334936	0,792819306	---
17442126	4,602407	4,462621	5,000423	4,540685	4,38179	4,138355	4,6884837	4,35361	-0,33487367	0,792853562	---
17546753	5,335177	6,058814	6,238562	5,199297	6,04869	5,380472	5,8775177	5,542819667	-0,334698	0,792950108	LOC100862046
17366734	7,561543	7,138454	7,219055	7,165726	6,294198	7,455145	7,3063507	6,971689667	-0,334661	0,792970444	---
17416690	7,10349	7,190041	7,076065	6,730838	6,786339	6,848912	7,1231987	6,788696333	-0,33450233	0,79305766	Orc1
17229036	9,018724	8,344725	9,05149	8,163733	8,898758	8,34926	8,8049797	8,470583667	-0,334396	0,793116114	Fmo2
17549004	5,619618	5,526217	5,885042	5,451286	5,235344	5,341141	5,676959	5,342590333	-0,33436867	0,79313114	Pycr2 // Pycr2
17463443	4,322239	6,679472	4,779645	5,836493	4,373362	4,56868	5,260452	4,926178333	-0,33427367	0,793183369	Clec2h
17209773	5,731452	5,755494	5,494003	5,098935	5,420209	5,458987	5,6603163	5,326043667	-0,33427267	0,793183919	---
17208465	5,554784	5,70467	4,926826	5,456831	5,012421	4,714401	5,3954267	5,061217667	-0,334209	0,793218923	---
17387950	4,15472	3,45324	3,433953	3,491082	3,316758	3,2318	3,6806377	3,346546667	-0,334091	0,793283804	Olfr1242
17547000	2,910515	3,783953	3,48284	3,009286	2,996281	3,169673	3,392436	3,058413333	-0,33402267	0,793321379	---
17201817	5,631964	5,862197	5,811311	5,318676	5,43965	5,545409	5,7684907	5,434578333	-0,33391233	0,793382052	---
17201993	5,631964	5,862197	5,811311	5,318676	5,43965	5,545409	5,7684907	5,434578333	-0,33391233	0,793382052	---
17466294	4,252118	4,110395	4,353197	3,997719	3,880622	3,835842	4,23857	3,904727667	-0,33384233	0,793420548	Try5
17201363	6,372433	6,725023	6,483278	6,288741	6,252493	6,038334	6,5269113	6,193189333	-0,333722	0,793486729	---
17333729	4,000776	3,791257	4,151163	3,618785	3,570858	3,753015	3,9810653	3,647552667	-0,33351267	0,793601872	Mir125a
17546219	3,550775	3,954358	4,204461	3,726926	3,415993	3,566178	3,903198	3,569699	-0,333499	0,793609389	---
17516383	9,508026	9,618184	9,500239	9,357196	9,076	9,192818	9,5421497	9,208671333	-0,33347833	0,793620758	Snord14e // Snord14e
17548340	6,47949	6,473745	6,348558	6,051728	6,015136	6,234766	6,433931	6,100543333	-0,33338767	0,793670635	Atp6v1g1 // Atp6v1g1
17208015	5,69053	5,268215	5,571021	5,372929	4,43438	5,72286	5,509922	5,176723	-0,333199	0,793774433	---
17213293	4,398077	5,002304	4,380132	4,117363	4,109477	4,554307	4,5935043	4,260382333	-0,333122	0,7938168	---
17429285	6,914814	5,697062	5,643588	5,736241	5,585392	5,934598	6,0851547	5,752077	-0,33307767	0,793841194	Olfr1333
17446440	5,431922	5,324453	4,982178	5,313848	4,525824	4,900072	5,2461843	4,913248	-0,33293633	0,793918966	---
17366525	5,299082	5,491817	5,561969	5,145083	4,802113	5,407016	5,450956	5,118070667	-0,33288533	0,793947032	Ucma
17364365	6,89571	6,050136	6,432484	5,665692	6,620144	6,093898	6,4594433	6,126578	-0,33286533	0,793958039	---
17206355	3,229026	3,221847	3,864031	2,989578	3,08242	3,244642	3,4383013	3,105546667	-0,33275467	0,794018944	---
17548145	6,301506	6,420426	6,136932	5,842687	5,840605	6,177458	6,286288	5,953583333	-0,33270467	0,794046463	Atp6v1g1 // Atp6v1g1
17201853	6,623997	5,93353	5,85573	6,060925	5,188621	6,165787	6,1377523	5,805111	-0,33264133	0,794081322	---
17491691	3,525945	3,191943	3,247128	3,198688	2,963337	2,805071	3,321672	2,989032	-0,33264	0,794082056	---
17215065	6,567006	7,272602	6,881728	6,360875	6,687614	6,675088	6,907112	6,574525667	-0,33258633	0,794111595	---
17289977	5,579174	5,68123	5,71474	5,311759	5,368309	5,298062	5,6583813	5,326043333	-0,332338	0,794248299	Gm6320
17533282	7,698599	7,702342	7,805583	7,378417	7,149591	7,68154	7,735508	7,403182667	-0,33232533	0,794255272	Mid1ip1
17210333	7,56379	7,163387	7,349828	7,234177	7,210064	6,6358	7,3590017	7,026680333	-0,33232133	0,794257474	---
17506945	4,133866	3,318773	4,327174	3,480516	3,457696	3,844942	3,9266043	3,594384667	-0,33221967	0,794313448	---
17319638	3,854102	3,796132	3,652094	3,359103	3,594176	3,352681	3,7674427	3,43532	-0,33212267	0,794366855	Cyp2d11
17442398	5,41665	5,689532	5,602036	5,518581	5,150288	5,043435	5,569406	5,237434667	-0,33197133	0,794450186	Gm19833
17248911	6,818522	7,785521	6,324942	6,501438	6,642242	6,790361	6,9763283	6,644680333	-0,331648	0,794628256	Olfr56
17520820	4,896333	4,756244	4,111949	3,934644	4,598012	4,238118	4,5881753	4,256924667	-0,33125067	0,794847135	---
17305599	6,105353	5,958322	6,129011	5,854506	5,734343	5,610497	6,0642287	5,733115333	-0,33111333	0,794922802	---
17207499	4,711097	4,971019	5,157391	4,156862	5,077808	4,611632	4,9465023	4,615434	-0,33106833	0,794947597	---
17219643	6,383989	6,66811	6,492586	5,961582	6,391158	6,198792	6,514895	6,183844	-0,331051	0,794957148	Aim2
17206915	4,717966	4,313657	5,152211	4,325931	4,367998	4,497093	4,7279447	4,397007333	-0,33093733	0,795019784	---
17398557	5,571495	5,789833	5,564868	5,131617	5,383893	5,417888	5,6420653	5,311132667	-0,33093267	0,795022355	Sfrp2
17285938	6,060364	5,393219	5,46603	5,08955	5,591976	5,245426	5,639871	5,308984	-0,330887	0,795047521	Hist1h2aa
17337612	3,966451	4,003098	4,250133	3,570622	3,853908	3,802995	4,0732273	3,742508333	-0,330719	0,795140109	Olfr126
17291076	4,68366	3,928143	3,817887	4,136684	3,543541	3,757356	4,14323	3,812527	-0,330703	0,795148927	Vmn1r202
17203523	6,996337	6,960271	6,528664	6,535365	6,87572	6,082512	6,828424	6,497865667	-0,33055833	0,795228665	---
17274184	10,10246	10,36915	10,12847	10,01545	9,66398	9,929062	10,200027	9,869497333	-0,33052933	0,79524465	Socs2 // Socs2
17305163	6,128382	5,968362	6,55086	5,90109	5,749332	6,006307	6,215868	5,885576333	-0,33029167	0,795375668	2610528A11Rik
17408985	6,879823	6,738881	7,048633	6,61366	6,573959	6,489025	6,8891123	6,558881333	-0,330231	0,795409115	Prok1
17203691	7,725755	6,874917	7,933961	7,38108	6,914148	7,249061	7,5115443	7,181429667	-0,33011467	0,795473256	---
17417749	7,771035	7,65951	7,62516	7,40149	7,406359	7,257836	7,685235	7,355228333	-0,33000667	0,795532808	Med8
17260633	8,223503	8,132977	8,256254	7,790458	8,00222	7,830444	8,2042447	7,874374	-0,32987067	0,795607804	Fignl1
17278321	9,444721	10,55205	9,851748	9,832966	9,513474	9,512599	9,9495063	9,619679667	-0,32982667	0,79563207	Serpina3m
17528532	6,34421	6,782128	6,97579	6,451466	6,456192	6,205046	6,7007093	6,370901333	-0,329808	0,795642364	C2cd4a
17209083	4,582031	4,549804	4,288215	4,139482	4,020182	4,271257	4,47335	4,143640333	-0,32970967	0,795696597	---
17207293	7,198637	6,381659	6,433735	6,471052	6,338558	6,215355	6,6713437	6,341655	-0,32968867	0,795708179	---
17312945	8,577933	8,91862	8,819472	8,543117	8,296488	8,48746	8,7720083	8,442355	-0,32965333	0,795727667	---
17479644	3,325987	3,504283	3,238632	2,927728	3,108222	3,044136	3,3563007	3,026695333	-0,32960533	0,795754142	---
17204729	7,887573	8,957973	8,652912	8,099605	8,080542	8,329526	8,499486	8,169891	-0,329595	0,795759842	---
17420439	8,519067	8,447862	8,288582	8,146467	8,289351	7,830917	8,4185037	8,088911667	-0,329592	0,795761497	---
17207769	4,568116	5,017443	4,481475	4,319771	4,36875	4,390335	4,6890113	4,359618667	-0,32939267	0,795871452	---
17240087	4,330408	4,649861	5,582179	4,311755	4,754296	4,508422	4,8541493	4,524824333	-0,329325	0,795908782	---
17207275	6,249451	5,634802	6,166661	6,122127	5,328804	5,612013	6,0169713	5,687648	-0,32932333	0,795909701	---
17208363	6,067382	5,689725	5,667641	5,57637	5,403512	5,457183	5,8082493	5,479021667	-0,32922767	0,795962481	---
17206133	2,783963	3,220278	3,2214	2,816021	2,657721	2,764274	3,0752137	2,746005333	-0,32920833	0,795973147	---
17209923	3,692284	4,121735	3,157696	3,665663	2,954351	3,364256	3,6572383	3,32809	-0,32914833	0,796006252	---
17370139	3,322358	3,32101	3,493561	2,944503	3,160885	3,044342	3,3789763	3,04991	-0,32906633	0,796051496	---
17200195	5,319103	6,082158	5,780245	5,230865	5,38711	5,576643	5,7271687	5,398206	-0,32896267	0,7961087	---
17427003	6,108744	5,772117	5,928497	5,761506	5,695874	5,365418	5,9364527	5,607599333	-0,32885333	0,796169034	Rps6
17274385	6,517529	6,747423	6,600581	6,442564	6,489165	5,947536	6,6218443	6,293088333	-0,328756	0,796222751	Gm3993 // Gm3993
17337133	5,205309	5,44544	4,871885	4,751373	4,891627	4,894014	5,1742113	4,845471333	-0,32874	0,796231581	H2-Q7
17249977	5,337555	8,069791	5,371119	5,929992	6,220218	5,642325	6,2594883	5,930845	-0,32864333	0,796284934	Gm12250
17547775	6,305187	6,218582	6,380672	5,931845	5,99296	5,993847	6,3014803	5,972884	-0,32859633	0,796310876	Spin1 // Spin1
17202623	4,504057	4,836505	4,324111	4,44592	3,806531	4,427355	4,554891	4,226602	-0,328289	0,79648053	---
17335624	3,991643	3,802784	4,122869	3,693815	3,544719	3,69467	3,972432	3,644401333	-0,32803067	0,796623163	---
17402705	7,399018	6,858086	6,718699	6,597101	6,577483	6,817143	6,9919343	6,663909	-0,32802533	0,796626108	Gm5549
17200403	5,813922	6,112654	5,812085	4,97888	6,126469	5,649445	5,912887	5,584931333	-0,32795567	0,796664577	---
17207701	5,773202	6,235526	5,8484	4,985752	5,983308	5,904266	5,952376	5,624442	-0,327934	0,796676542	---
17515521	4,173392	4,078577	3,865547	3,62922	3,813994	3,690598	4,039172	3,711270667	-0,32790133	0,796694581	---
17204223	7,334299	7,536525	7,418497	7,269339	6,966408	7,070356	7,4297737	7,102034333	-0,32773933	0,796784047	---
17251898	7,677281	7,436574	7,467758	7,09131	7,178013	7,320198	7,5272043	7,199507	-0,32769733	0,796807243	Mir324
17300372	10,68108	10,57475	10,66133	10,39832	10,22473	10,31109	10,639053	10,31138	-0,32767333	0,796820498	---
17547265	3,136464	4,527263	3,757073	3,23711	4,197324	3,003372	3,8069333	3,479268667	-0,32766467	0,796825285	---
17525673	4,817016	4,479976	4,778398	4,458528	4,536355	4,098642	4,6917967	4,364508333	-0,32728833	0,797033168	Panx3
17314773	5,549139	5,034837	5,480864	4,736715	5,202153	5,144361	5,3549467	5,027743	-0,32720367	0,797079944	1700120C14Rik // 1700120C14Rik
17306676	4,412343	4,138247	4,189735	4,033738	3,646581	4,079082	4,246775	3,919800333	-0,32697467	0,797206475	---
17547503	4,614034	4,32776	4,464828	4,233302	3,995992	4,196844	4,468874	4,142046	-0,326828	0,797287525	Gm19386
17333844	5,569214	5,618372	5,76637	5,133845	5,329362	5,510504	5,6513187	5,324570333	-0,32674833	0,797331553	---
17309374	5,486907	5,289662	5,182885	4,992838	5,143636	4,843216	5,319818	4,99323	-0,326588	0,797420169	BC005512 // BC005512
17404073	7,570969	8,255087	8,087303	7,554316	7,588879	7,790642	7,9711197	7,644612333	-0,32650733	0,797464757	Fabp5 // Fabp5
17405955	3,987709	3,729967	3,542356	3,480007	3,476241	3,324355	3,753344	3,426867667	-0,32647633	0,797481892	Serpini2
17208593	6,983242	6,998366	7,069763	7,044632	6,373273	6,654111	7,0171237	6,690672	-0,32645167	0,797495528	---
17392047	4,84273	4,664176	5,024038	4,585055	4,488637	4,47858	4,843648	4,517424	-0,326224	0,797621388	Lrrn4
17210785	6,098351	4,641359	4,987183	5,771268	4,588721	4,388405	5,2422977	4,916131333	-0,32616633	0,79765327	---
17210019	7,149252	6,316491	6,908683	6,611272	6,874161	5,910501	6,7914753	6,465311333	-0,326164	0,79765456	---
17512544	6,126786	5,933527	6,079837	5,922777	5,553698	5,685263	6,0467167	5,720579333	-0,32613733	0,797669304	Ctrl
17513641	9,10532	9,427211	9,198808	8,883276	8,839717	9,030285	9,2437797	8,917759333	-0,32602033	0,797733996	Slc7a5
17357700	5,276218	5,495993	5,43955	5,174257	4,814892	5,244987	5,4039203	5,078045333	-0,325875	0,797814362	Ms4a4d
17200201	4,656007	5,147181	4,226862	4,548148	4,699366	3,80517	4,6766833	4,350894667	-0,32578667	0,797862106	---
17210233	6,180997	5,871162	5,147725	5,202944	5,349043	5,670571	5,7332947	5,407519333	-0,32577533	0,79786948	---
17208371	6,840766	6,19152	6,624015	5,932158	6,607759	6,139321	6,5521003	6,226412667	-0,32568767	0,797917965	---
17548593	8,927812	9,044851	8,808126	8,445581	8,683767	8,674973	8,9269297	8,601440333	-0,32548933	0,798027665	3110003A17Rik // 3110003A17Rik
17288221	4,622626	4,081336	3,535139	3,584356	3,839189	3,839189	4,0797003	3,754244667	-0,32545567	0,798046288	---
17425878	4,856727	3,454437	3,543073	3,212228	3,504403	4,161527	3,9514123	3,626052667	-0,32535967	0,798099394	Mup4
17547094	5,447565	6,186358	6,270918	5,307041	6,202823	5,419083	5,9682803	5,642982333	-0,325298	0,798133509	---
17547110	5,447565	6,186358	6,270918	5,307041	6,202823	5,419083	5,9682803	5,642982333	-0,325298	0,798133509	LOC100862076
17243845	4,037844	4,152143	3,789363	3,513872	3,841028	3,64882	3,9931167	3,667906667	-0,32521	0,798182194	---
17541060	4,691681	4,843482	4,623004	4,377497	4,112196	4,692882	4,719389	4,394191667	-0,32519733	0,798189202	Rhox3g-ps
17467359	5,053429	5,69876	5,381383	5,064883	5,042104	5,051272	5,3778573	5,052753	-0,32510433	0,798240657	Tacstd2
17210717	6,008184	6,100868	6,427144	6,563424	5,169196	5,828473	6,178732	5,853697667	-0,32503433	0,798279389	---
17501502	4,726564	4,712595	4,615993	4,508436	4,520528	4,051626	4,6850507	4,360196667	-0,324854	0,798379178	---
17372812	4,229592	3,928216	4,128307	3,764726	3,908092	3,638778	4,0953717	3,770532	-0,32483967	0,79838711	Olfr1093
17337618	5,900168	5,489582	5,553459	5,685397	5,155959	5,127391	5,6477363	5,322915667	-0,32482067	0,798397625	Olfr128
17262193	7,776706	8,509612	8,583952	8,25811	7,612372	8,02593	8,29009	7,965470667	-0,32461933	0,798509052	Cdk2ap1
17548068	6,777004	7,245543	7,0289	6,665587	6,485158	6,927245	7,017149	6,692663333	-0,32448567	0,798583037	LOC100504825
17206715	3,95015	2,828941	3,016733	2,873788	2,904553	3,044718	3,2652747	2,941019667	-0,324255	0,79871073	---
17270558	4,898208	4,740435	4,790097	4,641817	4,411237	4,402978	4,80958	4,485344	-0,324236	0,798721249	Lyzl6
17538812	8,603707	8,131969	7,608724	7,898985	7,580109	7,892928	8,1148	7,790674	-0,324126	0,798782151	---
17524423	4,325842	4,2802	4,310009	4,052649	4,038152	3,852948	4,3053503	3,981249667	-0,32410067	0,798796177	---
17529227	3,46374	3,585812	3,788274	3,418746	3,193831	3,253006	3,6126087	3,288527667	-0,324081	0,798807066	---
17291335	4,697708	4,650213	4,837125	4,158008	4,598643	4,456226	4,7283487	4,404292333	-0,32405633	0,798820724	---
17548783	4,489549	4,489549	4,512968	3,877755	4,134525	4,507725	4,4973553	4,173335	-0,32402033	0,798840658	9430032J07Rik
17202063	8,169607	7,988428	8,220819	8,526399	6,906761	7,973754	8,1262847	7,802304667	-0,32398	0,798862991	---
17205971	4,882318	5,914257	5,88651	5,360535	5,558099	4,792933	5,5610283	5,237189	-0,32383933	0,798940886	---
17450856	3,467578	3,8412	3,695239	3,186938	3,415506	3,430143	3,6680057	3,344195667	-0,32381	0,798957131	Vmn2r8
17457969	5,650599	5,222403	5,625757	5,781141	4,830288	4,916084	5,4995863	5,175837667	-0,32374867	0,798991097	Olfr38
17201353	8,826618	8,2801	8,315577	7,911998	8,288801	8,250402	8,4740983	8,150400333	-0,323698	0,799019158	---
17348679	4,051755	4,211267	4,000421	3,746746	3,721075	3,824823	4,0878143	3,764214667	-0,32359967	0,799073621	---
17206947	5,510383	5,464403	5,684525	5,62701	4,667079	5,394484	5,5531037	5,229524333	-0,32357933	0,799084883	---
17516120	4,752954	4,530678	4,488994	4,347558	4,225533	4,228808	4,5908753	4,267299667	-0,32357567	0,799086914	Olfr145
17207311	4,210738	3,212476	3,51964	3,226818	3,413828	3,332629	3,647618	3,324425	-0,323193	0,799298895	---

17547530	3,297404	3,48444	3,618332	2,921236	3,235446	3,274409	3,4667253	3,143697	-0,32302833	0,799390131	LOC100862148 // LOC100862148
17205057	7,008066	6,869067	6,893383	6,827378	6,241591	6,732527	6,9235053	6,600498667	-0,32300667	0,799402136	---
17209247	7,008066	6,869067	6,893383	6,827378	6,241591	6,732527	6,9235053	6,600498667	-0,32300667	0,799402136	---
17240606	11,13348	11,39084	11,3518	10,9715	10,91676	11,01888	11,29204	10,96904667	-0,32299333	0,799409524	Cd24a // Cd24a
17545908	4,255931	3,663426	3,912175	3,607986	3,499334	3,755295	3,943844	3,620871667	-0,32297233	0,799421161	---
17548149	5,006423	5,722436	5,096814	5,081569	4,931547	4,843754	5,2752243	4,95229	-0,32293433	0,799442217	Rhoa // Rhoa
17325884	4,33642	4,604186	4,373878	4,263043	3,869233	4,213808	4,4381613	4,115361333	-0,3228	0,799516659	Cd200r2
17205923	3,300187	3,666319	3,446679	3,430219	3,02489	2,990149	3,4710617	3,148419333	-0,32264233	0,79960404	---
17504372	5,283798	4,602578	5,151925	4,799539	4,393815	4,877192	5,012767	4,690182	-0,322585	0,799635817	---
17205887	4,10645	4,754871	4,62938	4,261008	4,141485	4,120692	4,4969003	4,174395	-0,32250533	0,799679975	---
17549388	6,452696	6,67641	6,322393	6,059709	6,080161	6,344525	6,483833	6,161465	-0,322368	0,799756102	---
17347903	8,247641	8,521976	8,274024	8,134234	7,941792	8,001263	8,3478803	8,025763	-0,32211733	0,799895071	Mcfd2
17549566	9,576941	9,999592	9,932028	9,56504	9,543242	9,434019	9,836187	9,514100333	-0,32208667	0,799912074	---
17203551	4,902381	5,097974	5,193355	5,147205	4,465776	4,614713	5,06457	4,742564667	-0,32200533	0,799957171	---
17201565	2,694176	3,340138	3,713327	2,925824	2,76504	3,091555	3,2492137	2,927473	-0,32174067	0,800103939	---
17433528	4,835024	5,07006	4,696619	4,388601	4,555945	4,692088	4,8672343	4,545544667	-0,32168967	0,800132224	Ajap1
17274556	5,673901	5,374599	6,158746	5,57057	5,342303	5,329549	5,7357487	5,414140667	-0,321608	0,800177518	---
17204959	5,341413	4,809648	4,626297	4,962024	4,267149	4,58341	4,925786	4,604194333	-0,32159167	0,800186577	---
17259527	10,13092	10,38193	10,43274	10,05847	9,864607	10,0578	10,315197	9,993625667	-0,321571	0,80019804	---
17543085	5,887328	5,587852	5,831668	5,988232	5,024589	5,32961	5,7689493	5,447477	-0,32147233	0,800252768	---
17252880	4,318653	3,953321	4,326826	3,728933	3,659481	4,246218	4,1996	3,878210667	-0,32138933	0,800298809	Mir132
17220748	5,974134	6,082759	6,249064	5,900863	5,716896	5,724165	6,1019857	5,780641333	-0,32134433	0,800323772	---
17372779	4,064754	3,842512	3,745469	3,614336	3,501299	3,573359	3,884245	3,562998	-0,321247	0,800377768	Olfr1028
17534139	5,692644	5,993727	6,078992	5,588726	5,491735	5,721186	5,9217877	5,600549	-0,32123867	0,800382392	Gm19899
17203991	4,580826	4,715763	4,624	3,717234	5,243152	3,996591	4,6401963	4,318992333	-0,321204	0,800401624	---
17547101	5,57871	6,436992	6,578839	5,504731	6,568866	5,557522	6,1981803	5,877039667	-0,32114067	0,800436762	LOC100862046
17399160	5,834312	6,285836	6,308199	5,927395	5,452025	6,08564	6,1427823	5,821686667	-0,32109567	0,800461729	---
17218040	4,212701	4,453496	4,212355	3,850061	4,119663	3,945949	4,2928507	3,971891	-0,32095967	0,800537191	---
17210617	3,475062	3,211997	3,544654	3,196784	2,997239	3,075026	3,410571	3,089683	-0,320888	0,800576959	---
17498059	5,541278	5,49069	5,274877	4,948713	4,859491	5,53616	5,435615	5,114788	-0,320827	0,80061081	Krtap5-2
17202153	3,158014	3,547946	4,429914	3,205762	3,684278	3,283519	3,711958	3,391186333	-0,32077167	0,800641517	---
17407400	5,371172	5,236288	4,638123	4,977527	4,050007	5,256146	5,081861	4,761226667	-0,32063433	0,800717736	Sprr1b
17290078	4,615792	4,2253	4,608633	4,158064	3,923923	4,406009	4,4832417	4,162665333	-0,32057633	0,800749927	Gm6416
17367909	4,724812	5,862521	5,650052	5,339942	5,22875	4,706982	5,4124617	5,091891333	-0,32057033	0,800753257	---
17366336	6,609863	6,239649	6,403041	6,037397	5,849446	6,404265	6,4175177	6,097036	-0,32048167	0,800802472	---
17200901	3,59435	3,601555	3,4488	3,144955	3,189692	3,348948	3,548235	3,227865	-0,32037	0,800864458	---
17210605	4,737576	5,508095	5,550244	4,772076	5,211815	4,850992	5,265305	4,944961	-0,320344	0,800878891	---
17495299	11,81992	11,82447	11,51333	11,28897	11,42893	11,47935	11,71924	11,39908333	-0,32015667	0,800982892	---
17375083	9,346057	9,505404	9,229769	9,023534	8,961995	9,135337	9,36041	9,040288667	-0,32012133	0,801002509	Snap23
17204491	4,378502	3,559386	3,363223	3,312216	3,350126	3,678552	3,767037	3,446964667	-0,32007233	0,801029715	---
17256338	5,236433	5,326687	5,385168	5,188814	4,88592	4,91393	5,316096	4,996221333	-0,31987467	0,801139473	---
17206899	5,998531	6,229296	5,337272	5,467115	5,805723	5,332845	5,855033	5,535227667	-0,31980533	0,801177975	---
17204037	6,617496	6,138415	6,889057	5,934411	6,64467	6,107269	6,5483227	6,228783333	-0,31953933	0,801325708	---
17300131	4,225708	3,845482	4,245271	3,637804	3,901793	3,818568	4,105487	3,786055	-0,319432	0,801385327	---
17402350	8,475007	8,165082	7,679457	7,619695	8,022591	7,719001	8,1065153	7,787095667	-0,31941967	0,801392178	Fabp2 // Fabp2
17458379	6,35781	6,385466	6,453152	5,879909	6,196073	6,162273	6,3988093	6,079418333	-0,319391	0,801408102	Gimap1
17312070	2,910193	2,789403	3,503754	2,569561	2,869593	2,806227	3,0677833	2,748460333	-0,319323	0,801445876	---
17418263	8,248524	8,690743	8,492542	7,647893	8,311474	8,514522	8,4772697	8,157963	-0,31930667	0,80145495	---
17406482	6,116193	6,525542	6,526591	6,073793	6,150321	5,987279	6,3894427	6,070464333	-0,31897833	0,801637368	Cd1d1
17209757	5,006496	4,789789	4,509163	4,128462	4,530109	4,690644	4,7684827	4,449738333	-0,31874433	0,801767402	---
17200269	6,181931	5,209874	6,275772	6,356448	5,024913	5,330245	5,8891923	5,570535333	-0,318657	0,801815938	---
17267617	4,864821	4,592507	4,488605	4,146416	4,720378	4,123284	4,6486443	4,330026	-0,31861833	0,801837428	Gm15698 // Gm15698
17202591	6,14072	5,590368	5,825924	5,477631	5,728734	5,394851	5,8523373	5,533738667	-0,31859867	0,801848359	---
17504019	6,945562	7,852273	6,917367	6,760541	6,580357	7,418914	7,2384007	6,919937333	-0,31846333	0,801923581	---
17544162	4,363941	4,321925	4,347336	4,076229	3,87335	4,128384	4,3444007	4,025987667	-0,318413	0,801951559	---
17209167	7,195493	6,922544	7,459639	6,796112	6,931927	6,894533	7,1925587	6,874190667	-0,318368	0,801976574	---
17328567	8,965804	8,527886	8,549301	8,049958	8,360378	8,677794	8,680997	8,36271	-0,318287	0,802021602	Igll1
17204191	3,747533	4,319158	3,986565	3,737126	3,623094	3,738637	4,017752	3,699619	-0,318133	0,802107218	---
17203091	5,231258	4,854259	5,236584	5,038268	4,710035	4,619619	5,107367	4,789307333	-0,31805967	0,802147991	---
17205377	4,635164	4,200119	4,237736	4,249553	3,835576	4,033886	4,357673	4,039671667	-0,31800133	0,802180425	---
17546620	5,978733	6,603991	6,810772	5,733208	6,825527	5,880921	6,4644987	6,146552	-0,31794667	0,802210822	LOC100862076
17208529	5,31665	4,042009	4,688121	4,443372	4,239588	4,40999	4,68226	4,364316667	-0,31794333	0,802212675	---
17219846	3,3158	3,693468	3,203409	3,165853	3,025433	3,067694	3,4042257	3,086326667	-0,317899	0,802237327	---
17549574	6,153963	6,491373	6,435849	5,878153	6,167199	6,082148	6,360395	6,0425	-0,317895	0,802239552	---
17549730	6,153963	6,491373	6,435849	5,878153	6,167199	6,082148	6,360395	6,0425	-0,317895	0,802239552	---
17201493	7,006099	6,947441	6,76524	6,147646	6,800474	6,817082	6,90626	6,588400667	-0,31785933	0,802259385	---
17342890	7,211953	6,117132	6,920897	6,462315	6,363606	6,470519	6,749994	6,432146667	-0,31784733	0,802266058	Clps
17378116	5,122485	5,106699	5,205368	4,777501	4,800285	4,903938	5,1448507	4,827241333	-0,31760933	0,802398418	Bpifa3
17206681	4,856435	6,041424	5,972196	5,266076	5,276342	5,37485	5,6233517	5,305756	-0,31759567	0,802406019	---
17254668	3,84113	3,34266	3,453508	3,171847	3,282693	3,229982	3,545766	3,228174	-0,317592	0,802408059	---
17323071	10,31054	10,1194	9,850741	9,601192	10,04442	9,682302	10,09356	9,775971333	-0,317589	0,802409727	---
17532574	3,991589	3,773644	4,18451	3,64966	3,581695	3,765854	3,9832477	3,665736333	-0,31751133	0,802452926	Ccr1l1
17315705	6,26785	6,284963	6,467963	6,212313	5,745151	6,110971	6,3402587	6,022811667	-0,317447	0,80248871	Card6
17247939	5,954438	6,191628	6,181835	5,997268	5,504071	5,874756	6,1093003	5,792031667	-0,31726867	0,802587912	---
17269212	7,494568	6,951582	7,250155	6,940348	6,624206	7,180219	7,2321017	6,914924333	-0,31717733	0,802638724	---
17524139	3,712785	3,881084	3,333465	3,060337	3,662895	3,25269	3,6424447	3,325307333	-0,31713733	0,802660978	---
17300630	5,138658	5,286094	5,180316	4,851292	4,776022	5,026366	5,2016893	4,88456	-0,31712933	0,802665429	Tssk4
17202301	5,559238	4,890953	5,762376	5,204999	4,75662	5,299587	5,404189	5,087068667	-0,31712033	0,802670436	---
17351841	8,196069	8,211183	8,049979	7,207598	8,30606	7,99249	8,1524103	7,835382667	-0,31702767	0,802721995	---
17224730	5,232573	5,048854	5,105124	4,89178	4,585545	4,958698	5,1288503	4,812007667	-0,31684267	0,802824936	Scg2
17507938	8,323761	7,596769	7,831147	7,75392	7,357089	7,691489	7,9172257	7,600832667	-0,316393	0,803075204	---
17540935	3,715841	3,418097	3,506216	3,250107	3,246936	3,194202	3,546718	3,230415	-0,316303	0,803125304	---
17312940	7,391364	7,572865	7,681046	7,339734	7,587511	6,769984	7,548425	7,232409667	-0,31601533	0,803285459	---
17210585	3,465399	3,970157	3,852751	3,684116	3,567564	3,088697	3,762769	3,446792333	-0,31597667	0,803306989	---
17390876	3,896252	3,796694	3,708684	3,524587	3,591817	3,337684	3,8005433	3,484696	-0,31584733	0,803379006	---
17258935	4,592661	4,449585	4,428906	4,263868	4,037327	4,222535	4,490384	4,174576667	-0,31580733	0,803401281	Enpp7
17203925	4,797761	4,956728	4,205645	4,330062	3,791675	4,891622	4,653378	4,337786333	-0,31559167	0,803521389	---
17208537	4,141575	4,08275	3,892794	3,106282	4,187078	3,877791	4,0390397	3,723717	-0,31532267	0,803671225	---
17205197	4,615785	4,520338	4,022763	4,288983	4,420611	3,503841	4,3862953	4,071145	-0,31515033	0,803767231	---
17209387	4,615785	4,520338	4,022763	4,288983	4,420611	3,503841	4,3862953	4,071145	-0,31515033	0,803767231	---
17207517	6,999749	6,165899	6,856799	5,884763	6,858269	6,334718	6,674149	6,35925	-0,314899	0,803907269	---
17466596	5,309246	5,006122	5,038525	4,826069	4,754529	4,82862	5,1179643	4,803072667	-0,31489167	0,803911355	1700026J14Rik
17439235	5,635814	5,126635	5,199988	5,126635	4,682522	5,208943	5,3208123	5,006033333	-0,314779	0,803974139	LOC100862180 // LOC100862166 // Gm7
17418213	9,606221	9,400215	9,706162	9,315577	9,310663	9,142061	9,570866	9,256100333	-0,31476567	0,80398156	---
17205791	4,807091	4,117064	4,316187	3,591705	4,600118	4,104263	4,4134473	4,098695333	-0,314752	0,803989185	---
17323135	6,167318	5,845418	5,94629	5,69627	5,655048	5,663684	5,986342	5,671667333	-0,31467467	0,804032283	---
17202713	5,554629	5,792529	6,213974	5,666511	5,166943	5,783726	5,8537107	5,53906	-0,31465067	0,804045658	---
17226867	5,708271	5,357436	5,85865	5,214911	5,503883	5,26193	5,6414523	5,326908	-0,31454433	0,804104922	1700037F24Rik
17210117	5,64009	5,704021	5,941853	5,756062	5,395438	5,190996	5,761988	5,447498667	-0,31448933	0,804135578	---
17473554	5,82917	5,898367	6,176531	5,83167	5,371855	5,757269	5,9680227	5,653598	-0,31442467	0,804171623	Galp
17202463	3,972796	3,266864	3,721577	3,378875	3,119505	3,51967	3,6537457	3,33935	-0,31439567	0,804187788	---
17481616	4,868829	3,640481	3,867395	3,823694	4,199803	3,410061	4,1255683	3,811186	-0,31438233	0,80419522	Olfr495
17319674	5,537233	5,579511	5,532051	5,459537	5,029369	5,217075	5,5495983	5,235327	-0,31427133	0,804257097	Cyp2d13
17370136	5,393693	5,464468	5,467115	5,325255	5,201447	4,855829	5,4417587	5,127510333	-0,31424833	0,804269919	---
17541400	4,478171	4,331643	4,368156	4,114662	4,093918	4,026728	4,3926567	4,078436	-0,31422067	0,804285342	9530027J09Rik
17201195	7,268551	6,977022	7,601313	6,878828	7,170357	6,856177	7,2822953	6,968454	-0,31384133	0,804496844	---
17397394	3,923855	3,677898	3,42981	3,385375	3,285473	3,41928	3,6771877	3,363376	-0,31381167	0,804513387	---
17317983	6,65818	6,454552	6,910954	6,445371	6,299006	6,338338	6,674562	6,360905	-0,313657	0,804599641	Arc
17501346	5,698232	5,624645	5,72341	5,270984	5,324882	5,509483	5,6820957	5,368449667	-0,313646	0,804605776	Mfap3l
17432496	3,671519	3,363665	3,601008	3,123451	3,591101	2,980806	3,5453973	3,231786	-0,31361133	0,80462511	Gm13109
17387355	5,073936	4,471712	4,932398	4,26755	4,9674	4,302455	4,8260153	4,512468333	-0,313547	0,804660991	---
17508164	8,815987	9,175396	9,284681	8,662449	8,40736	9,265666	9,0920213	8,778491667	-0,31352967	0,804670659	---
17505851	6,87368	7,286144	6,857353	6,609828	6,671716	6,795297	7,0057257	6,692280333	-0,31344533	0,804717698	Nudt7
17238141	7,54305	7,088307	7,500525	6,906396	7,049284	7,23628	7,377294	7,063986667	-0,31330733	0,804794676	Tac2
17547769	9,048226	9,152568	9,14182	8,619077	8,990559	8,793316	9,1142047	8,800984	-0,31322067	0,804843024	Rps18 // Rps18
17429685	4,887599	4,590331	4,879435	4,716579	4,702429	3,998905	4,7857883	4,472637667	-0,31315067	0,804882076	Oxct2a
17432783	7,076061	6,752405	6,862594	6,674016	6,379762	6,698002	6,89702	6,583926667	-0,31309333	0,804914063	Zfp933
17206223	5,817223	5,531903	5,385657	5,57089	4,748451	5,476836	5,578261	5,265392333	-0,31286867	0,80503942	---
17289511	4,539343	4,501742	4,293573	4,219235	4,106268	4,070673	4,444886	4,132058667	-0,31282733	0,805062484	5930438M14
17284527	4,842629	4,537559	5,029315	4,704021	4,314656	4,452515	4,8031677	4,490397333	-0,31277033	0,805094293	---
17233659	6,132844	5,974602	6,166615	5,684502	6,041107	5,610286	6,0913537	5,778631667	-0,312722	0,805121265	Gm17455
17326982	8,400473	8,645228	8,30464	7,922188	8,13991	8,350298	8,4501137	8,137465333	-0,31264833	0,805162377	---
17415359	5,249137	5,595204	5,202537	4,731221	4,752708	5,625033	5,3489593	5,036320667	-0,31263867	0,805167772	Ifna7
17267553	7,445725	7,572031	7,585126	7,131868	7,13061	7,4025	7,534294	7,221659333	-0,31263467	0,805170005	Srsf1 // Srsf1
17445605	3,242182	3,583885	3,232199	2,985284	3,073638	3,061955	3,3527553	3,040292333	-0,312463	0,805265818	---
17205301	7,133868	7,266684	7,316437	7,287437	6,892257	6,600424	7,2389963	6,926706	-0,31229033	0,805362201	---
17485185	5,204471	5,303067	5,121266	4,959342	5,026252	4,706596	5,2096013	4,897396667	-0,31220467	0,805410024	---
17386315	4,683301	4,854267	4,404256	4,362704	4,29328	4,349408	4,6472747	4,335130667	-0,312144	0,805443893	4930578N16Rik
17489759	7,033449	7,376712	7,217298	6,910104	6,909353	6,872567	7,209153	6,897341333	-0,31181167	0,805629453	C230052I12Rik
17549286	13,21223	13,43769	13,42836	13,18684	12,92942	13,02696	13,359427	13,04774	-0,31168667	0,805699259	---
17541646	4,289237	4,184691	4,513391	3,891734	4,175389	3,985298	4,3291063	4,017473667	-0,31163267	0,805729417	---
17414059	6,705314	6,85498	7,162457	6,698712	6,45667	6,63262	6,9075837	6,596000667	-0,311583	0,805757155	---
17343801	5,724457	5,94417	5,860611	5,560133	5,503221	5,53168	5,8430793	5,531678	-0,31140133	0,805858624	---
17208047	4,88071	4,860322	4,791239	4,316598	4,302472	4,978997	4,8440903	4,532689	-0,31140133	0,805858624	---

17366862	3,500107	3,906822	3,652449	3,129502	3,78624	3,209528	3,6864593	3,37509	-0,31136933	0,805876499	---
17464251	4,702437	4,750581	4,644392	4,402668	4,607162	4,1538	4,6991367	4,387876667	-0,31126	0,805937574	2010013B24Rik
17448746	8,889824	8,749384	9,17498	8,255132	8,663635	8,961995	8,9380627	8,626920667	-0,311142	0,806003495	---
17207839	6,492164	6,420048	7,305326	6,595057	6,983721	5,705701	6,7391793	6,428159667	-0,31101967	0,806071843	---
17208917	7,010046	6,947183	5,383166	6,81572	4,794018	6,797616	6,4467983	6,135784667	-0,31101367	0,806075195	---
17344289	6,733149	6,805667	6,605507	6,370743	6,455505	6,385878	6,7147743	6,404042	-0,31073233	0,8062324	Aif1
17418261	9,394613	9,53669	9,171156	8,974741	8,949281	9,24637	9,3674863	9,056797333	-0,310689	0,806256617	---
17284463	5,798574	5,769799	4,94382	5,452461	4,922101	5,205708	5,5040643	5,193423333	-0,310641	0,806283442	Igh-VJ558 // Igh-VJ558
17205109	4,194788	4,673743	4,813654	4,45491	4,504758	3,790718	4,5607283	4,250128667	-0,31059967	0,806306542	---
17209299	4,194788	4,673743	4,813654	4,45491	4,504758	3,790718	4,5607283	4,250128667	-0,31059967	0,806306542	---
17467502	5,243124	4,991034	5,358405	4,819412	4,590709	5,250899	5,197521	4,887006667	-0,31051433	0,806354236	---
17206119	5,716158	5,387529	6,09504	5,868043	5,062644	5,336982	5,732909	5,422556333	-0,31035267	0,8064446	---
17227797	8,992176	9,16442	8,983294	8,761884	8,878649	8,568492	9,04663	8,736341667	-0,31028833	0,806480562	Rgs18
17489555	4,046846	3,88802	3,968248	3,57328	3,618331	3,78072	3,9677047	3,657443667	-0,310261	0,806495842	Scgb2b1-ps // Scgb2b1-ps
17205737	7,211396	6,570566	7,084362	6,335671	6,711467	6,888626	6,9554413	6,645254667	-0,31018667	0,806537397	---
17284411	6,463425	6,229068	5,499326	5,509529	5,462816	6,290276	6,0639397	5,754207	-0,30973267	0,806791245	---
17357680	4,747024	4,523605	4,733556	4,296109	4,546607	4,23231	4,6680617	4,358342	-0,30971967	0,806798515	Gm8369
17244860	3,611525	3,99621	3,929246	3,705127	3,593032	3,309863	3,8456603	3,536007333	-0,309653	0,806835798	---
17205443	3,724742	3,627602	3,558016	3,34528	3,542093	3,09408	3,6367867	3,327151	-0,30963567	0,806845492	---
17207289	4,627148	5,083029	5,091183	4,593102	4,372673	4,9076	4,9337867	4,624458333	-0,30932833	0,80701739	---
17204697	5,015103	5,79037	5,001952	4,74457	5,373366	4,761813	5,2691417	4,959916333	-0,30922533	0,807075008	---
17204761	6,205714	6,503094	6,537774	6,314539	6,115853	5,888695	6,4155273	6,106362333	-0,309165	0,807108761	---
17284919	9,706764	9,687798	9,841956	9,805108	9,203694	9,301305	9,745506	9,436702333	-0,30880367	0,807310932	Akr1c14
17201523	3,8433	4,357528	3,767819	3,360522	3,975114	3,706789	3,989549	3,680808333	-0,30874067	0,807346187	---
17514961	4,429517	4,326866	4,797364	3,921898	4,336857	4,369087	4,5179157	4,209280667	-0,308635	0,807405321	Olfr869
17317391	3,707442	3,571268	3,552428	3,40886	3,305011	3,19145	3,6103793	3,301773667	-0,30860567	0,807421738	---
17339876	9,129975	9,294453	9,477123	9,488817	8,858384	8,629055	9,300517	8,992085333	-0,30843167	0,807519125	Qpct
17453126	7,91163	8,336558	8,825547	8,340782	8,090127	7,718455	8,3579117	8,049788	-0,30812367	0,80769154	---
17326709	4,901884	4,938778	5,109612	4,663864	4,603536	4,758703	4,9834247	4,675367667	-0,308057	0,807728864	---
17491718	4,451229	3,859652	3,608998	3,978989	3,507513	3,509252	3,973293	3,665251333	-0,30804167	0,807737449	---
17491720	4,451229	3,859652	3,608998	3,978989	3,507513	3,509252	3,973293	3,665251333	-0,30804167	0,807737449	---
17511731	4,503179	4,078658	4,656888	4,039824	4,180583	4,094663	4,4129083	4,105023333	-0,307885	0,807825168	Ces1f
17220752	3,351358	3,565052	3,63998	3,249766	2,987012	3,396669	3,5187967	3,211149	-0,30764767	0,807958072	---
17457498	5,503294	5,58821	5,237031	5,361312	4,844477	5,199987	5,442845	5,135258667	-0,30758633	0,807992422	Jhdm1d
17205215	4,012966	4,788794	4,233926	4,00215	4,217917	3,893413	4,3452287	4,037826667	-0,307402	0,808095665	---
17209405	4,012966	4,788794	4,233926	4,00215	4,217917	3,893413	4,3452287	4,037826667	-0,307402	0,808095665	---
17230902	5,867317	5,683973	5,562952	5,566383	5,014573	5,611649	5,7047473	5,397535	-0,30721233	0,80820191	Kcnk2
17255819	7,338355	6,952142	7,272216	6,770708	7,024807	6,846044	7,187571	6,880519667	-0,30705133	0,808292108	---
17238697	6,078	5,401156	5,987918	5,453428	5,678216	5,414915	5,822358	5,515519667	-0,30683833	0,808411453	Olfr820
17204335	3,630605	3,204726	4,37055	3,269186	3,106032	3,910199	3,7352937	3,428472333	-0,30682133	0,808420979	---
17550843	4,032338	3,788274	4,233658	3,744872	3,530249	3,858811	4,01809	3,711310667	-0,30677933	0,808444515	---
17319554	7,130737	7,260553	7,211836	7,028498	6,837049	6,817492	7,201042	6,894346333	-0,30669567	0,8084914	Pmm1
17290913	4,402086	4,562866	4,308697	4,418063	4,046965	3,889437	4,4245497	4,118155	-0,30639467	0,808660099	Gpx5
17300223	4,230173	3,843317	3,845043	3,729384	3,469339	3,800789	3,9728443	3,666504	-0,30634033	0,808690555	---
17547298	5,451185	6,183045	6,28402	5,373881	6,208147	5,418237	5,97275	5,666755	-0,305995	0,808884152	LOC100862076
17488501	7,474188	7,604182	7,663881	7,176734	7,243908	7,403794	7,5807503	7,274812	-0,30593833	0,808915924	Pak4
17549776	8,31004	8,610934	8,654617	8,08319	8,141509	8,433463	8,525197	8,219387333	-0,30580967	0,80898807	---
17248104	8,053333	8,304913	7,798412	7,737548	7,73969	7,76247	8,0522193	7,746569333	-0,30565	0,809077608	Rtn4
17239360	4,348387	4,463142	4,285847	4,079605	4,14497	3,956177	4,365792	4,060250667	-0,30554133	0,809138552	Plagl1 // Plagl1
17261150	4,813399	4,568256	5,154144	4,459908	4,723088	4,436941	4,8452663	4,539979	-0,30528733	0,809281021	---
17277060	8,052661	8,043188	8,044475	7,44957	7,97082	7,804256	8,0467747	7,741548667	-0,305226	0,809315426	---
17203215	5,856702	5,872238	5,474861	5,97028	5,160268	5,157741	5,7346003	5,429429667	-0,30517067	0,809346468	---
17332974	6,814076	7,198988	6,633998	6,618083	6,690995	6,423038	6,882354	6,577372	-0,304982	0,809452316	Rps6ka2
17255755	7,602038	7,799521	7,73145	7,275014	7,556897	7,38622	7,711003	7,406043667	-0,30495933	0,809465033	Kpnb1 // Kpnb1
17209857	5,754855	6,006419	5,859235	5,69888	5,143488	5,863744	5,873503	5,568704	-0,304799	0,809554998	---
17324692	7,522426	7,944595	7,175143	7,570021	6,744006	7,413812	7,547388	7,242613	-0,304775	0,809568465	---
17341995	7,277877	8,074258	8,300071	7,747221	8,200597	6,790361	7,8840687	7,579393	-0,30467567	0,809624208	---
17200771	5,72344	5,088157	5,377508	5,901879	4,502378	4,871101	5,3963683	5,091786	-0,30458233	0,809676588	---
17486948	5,14446	4,582289	4,681564	4,445864	4,394578	4,654189	4,802771	4,498210333	-0,30456067	0,809688748	Ceacam15
17421452	4,513196	4,809041	4,341156	4,53706	3,853813	4,359097	4,5544643	4,24999	-0,30447433	0,809737202	---
17305527	5,400268	5,239784	5,22082	4,990182	4,817297	5,140397	5,2869573	4,982625333	-0,304332	0,809817093	Ang6
17262297	5,653691	5,562573	5,833132	5,476234	5,528452	5,131748	5,683132	5,378811333	-0,30432067	0,809823455	---
17207249	4,876492	5,484386	5,125145	5,229646	4,498087	4,84541	5,1620077	4,857714333	-0,30429333	0,809838798	---
17334030	5,733851	4,607804	5,058883	4,734524	4,571608	5,18223	5,1335127	4,829454	-0,30405867	0,809970536	Vmn2r113
17457664	3,842944	4,087729	3,791654	3,838394	3,458718	3,513151	3,9074423	3,603421	-0,30402133	0,809991496	1810009J06Rik
17359707	4,012523	4,010994	4,226506	3,714417	3,933072	3,690698	4,083341	3,779395667	-0,30394533	0,810034167	Wnt8b
17394087	8,208782	8,208114	7,95697	7,63621	7,933319	7,8928	8,124622	7,820776333	-0,30384567	0,810090129	Tomm34
17316019	5,816173	5,877573	5,78049	5,714222	5,296707	5,552325	5,8247453	5,521084667	-0,30366067	0,810194015	---
17249593	7,479435	8,531398	7,364117	7,483045	7,488298	7,492647	7,79165	7,487996667	-0,30365333	0,810198134	Irf1
17207341	5,087922	5,541808	5,6344	5,135882	5,342267	4,875057	5,4213767	5,117735333	-0,30364133	0,810204873	---
17451645	6,571173	6,581913	6,703827	6,209012	6,366454	6,370766	6,618971	6,315410667	-0,30356033	0,810250363	---
17297637	4,393648	3,786642	4,118004	3,504287	4,024948	3,859608	4,0994313	3,796281	-0,30315033	0,810480661	---
17551107	5,381749	5,430228	5,989766	5,164009	5,359731	5,368687	5,600581	5,297475667	-0,30310533	0,810505941	---
17254948	6,08671	6,004102	6,07856	5,532704	6,085796	5,641822	6,0564573	5,753440667	-0,30301667	0,810555756	Cuedc1
17397932	4,854323	4,676103	4,640914	4,454631	4,579048	4,228662	4,72378	4,420780333	-0,30299967	0,810565307	Sucnr1
17209893	5,085366	5,012738	4,377513	4,274402	4,204494	5,087776	4,8252057	4,522224	-0,30298167	0,81057542	---
17475704	5,135826	5,823304	5,327561	5,088819	5,248684	5,040438	5,428897	5,125980333	-0,30291667	0,810611941	---
17202525	5,560242	6,01533	6,316305	5,556485	5,785095	5,642041	5,963959	5,661207	-0,302752	0,810704468	---
17284466	6,673062	6,499023	6,296526	5,750937	6,260017	6,549957	6,489537	6,186970333	-0,30256667	0,810808621	---
17449960	4,078409	3,954577	4,381789	3,907321	3,845215	3,754995	4,1382583	3,835843667	-0,30241467	0,810894051	---
17209093	4,0045	4,118479	4,42103	3,836285	3,995268	3,805763	4,1813363	3,879105333	-0,302231	0,810997291	---
17393307	3,826383	3,559417	3,446023	3,444223	3,160914	3,32081	3,6106077	3,308649	-0,30195867	0,811150395	---
17337832	4,752242	4,92753	4,623262	4,342752	4,70184	4,352596	4,767678	4,465729333	-0,30194867	0,811156017	---
17544288	4,732103	5,493071	5,530823	4,887087	5,2403	4,72279	5,251999	4,950059	-0,30194	0,81116089	---
17208525	4,652416	4,827999	4,690534	5,635249	3,580628	4,049286	4,7236497	4,421721	-0,30192867	0,811167262	---
17469682	4,113482	4,82854	4,634299	4,057691	4,324602	4,288342	4,5254403	4,223545	-0,30189533	0,811186005	---
17206081	3,891546	3,393824	3,617956	3,327305	3,189367	3,481602	3,634442	3,332758	-0,301684	0,81130484	---
17547182	5,615407	6,100623	5,365373	5,551431	5,450212	5,174981	5,693801	5,392208	-0,301593	0,811356016	LOC100504515
17270094	8,762732	8,338462	8,929322	8,215234	8,45747	8,45336	8,6768387	8,375354667	-0,301484	0,811417318	---
17200281	5,59457	3,976984	4,178333	4,264377	4,210313	4,37097	4,5832957	4,281886667	-0,301409	0,811459502	---
17551115	6,175463	6,493507	6,545045	5,747022	6,178259	6,385332	6,4046717	6,103537667	-0,301134	0,811614193	---
17209533	3,703643	3,651108	3,457564	3,181647	3,428227	3,2993	3,604105	3,303058	-0,301047	0,811663138	---
17377382	4,759485	4,732838	4,779982	4,582757	4,333468	4,453036	4,757435	4,456420333	-0,30101467	0,811681329	8030411F24Rik
17237541	5,771398	5,880379	6,167752	5,526019	5,760617	5,629951	5,939843	5,638862333	-0,30098067	0,811700458	LOC100862026
17527188	5,555764	5,325828	5,407092	5,322635	5,062407	5,000782	5,4295613	5,128608	-0,30095333	0,811715837	Tnfaip8l3
17412965	5,928511	6,082889	6,157402	6,061239	5,376134	5,828614	6,0562673	5,755329	-0,30093833	0,811724277	Ccl21b
17413025	5,928511	6,082889	6,157402	6,061239	5,376134	5,828614	6,0562673	5,755329	-0,30093833	0,811724277	Ccl21b
17413123	5,928511	6,082889	6,157402	6,061239	5,376134	5,828614	6,0562673	5,755329	-0,30093833	0,811724277	Ccl21b
17434150	5,928511	6,082889	6,157402	6,061239	5,376134	5,828614	6,0562673	5,755329	-0,30093833	0,811724277	Ccl21b
17359918	4,783978	5,102367	4,791173	4,599633	4,40639	4,76886	4,892506	4,591627667	-0,30087833	0,811758036	Elovl3
17467185	3,754712	3,642731	3,951526	3,61022	3,156682	3,680075	3,7829897	3,482325667	-0,300664	0,811878643	---
17494712	3,721623	3,589284	3,469734	3,290314	3,247017	3,341355	3,593547	3,292895333	-0,30065167	0,811885584	---
17210415	3,614654	3,666958	3,091328	2,688436	3,894787	2,887934	3,4576467	3,157052333	-0,30059433	0,811917849	---
17495851	7,551117	7,763021	7,728378	7,489295	7,347106	7,304748	7,6808387	7,380383	-0,30045567	0,811995892	Usp31
17548141	7,526424	7,427941	7,46967	7,025702	7,228575	7,268507	7,4746783	7,174261333	-0,300417	0,812017655	Bri3 // Bri3
17491816	7,464365	7,624938	7,545808	7,140261	7,323101	7,270502	7,545037	7,244621333	-0,30041567	0,812018405	Mphosph10
17397886	5,36541	4,822878	4,991095	4,666316	4,794018	4,817911	5,0597943	4,759415	-0,30037933	0,812038856	---
17402402	5,901145	5,63146	5,616801	5,505874	5,244007	5,498551	5,7164687	5,416144	-0,30032467	0,812069626	Tram1l1
17547656	5,232548	5,071856	5,014818	4,900982	4,805472	4,711821	5,1064073	4,806091667	-0,30031567	0,812074692	LOC100862018
17341020	6,093857	5,226356	5,894058	5,225871	5,53161	5,555886	5,7380903	5,437789	-0,30030133	0,81208276	---
17457765	3,913815	3,331213	4,109128	3,349426	3,616938	3,486946	3,7847187	3,484436667	-0,300282	0,812093643	Prss2
17379750	4,451795	4,582989	4,4362	4,402758	3,865193	4,302543	4,490328	4,190164667	-0,30016333	0,812160443	5031425F14Rik
17210167	4,80539	4,790875	5,451389	5,193101	4,672523	4,281721	5,0158847	4,715781667	-0,300103	0,812194408	---
17206005	4,122323	4,228573	3,692194	3,473843	3,628277	4,041531	4,0143633	3,714550333	-0,299813	0,812357686	---
17260306	5,418273	5,264016	5,286762	4,934831	4,969209	5,165843	5,323017	5,023294333	-0,29972267	0,812408553	Rps15a-ps6
17516172	3,985089	3,320926	3,601388	3,216081	3,386014	3,406454	3,635801	3,336183	-0,299618	0,812467495	Olfr905
17231558	7,170483	7,239845	7,424323	6,728752	7,247878	6,959197	7,278217	6,978609	-0,299608	0,812473126	Katna1
17304141	5,715221	5,982174	6,05864	5,74402	5,688793	5,42473	5,9186783	5,619181	-0,29949733	0,812535452	Ppifos
17200433	4,88893	4,7993	5,547667	4,519017	4,577745	5,240737	5,0786323	4,779166333	-0,299466	0,8125531	---
17462092	6,910429	6,940341	6,730964	6,701628	6,610893	6,371462	6,860578	6,561327667	-0,29925033	0,812674576	Zfand4
17458355	6,172661	6,453943	5,967858	5,675961	5,862426	6,158337	6,198154	5,898908	-0,299246	0,812677017	Gimap9
17231522	5,739933	5,517921	5,552525	5,167695	5,38545	5,35956	5,6034597	5,304235	-0,29922467	0,812689034	Lrp11
17391476	5,736159	6,30116	6,600237	5,997268	5,115634	6,627244	6,2125187	5,913382	-0,29913667	0,812738608	---
17392925	6,329618	5,82495	6,070797	5,82097	5,902684	5,60443	6,0751217	5,776028	-0,29909367	0,812762832	Trib3
17381262	4,097404	3,639097	4,16384	3,539761	3,607482	3,855844	3,9667803	3,667695667	-0,29908467	0,812767902	1700080N15Rik
17518500	7,744786	7,909869	8,013105	7,864183	7,454115	7,452656	7,8892533	7,590318	-0,29893533	0,812852036	Parp16
17203555	4,236002	4,714555	4,962718	4,024869	4,201399	4,790638	4,6377583	4,338968667	-0,29878967	0,812934113	---
17543331	3,828004	3,80691	3,77513	3,283725	3,813364	3,416681	3,803348	3,50459	-0,298758	0,812951956	1700010D01Rik
17207085	4,056928	3,944131	4,362746	3,593846	3,374073	4,49995	4,1212683	3,822623	-0,29864533	0,813015446	---
17350166	6,852573	6,995255	6,608001	6,5046	6,443573	6,611783	6,8186097	6,519985333	-0,29862433	0,81302728	C330007P06Rik
17403723	4,334644	4,636771	4,712737	4,412776	4,050391	4,325251	4,561384	4,262806	-0,298578	0,813053392	---
17204695	6,891737	6,520412	5,993531	6,33393	5,830263	6,346214	6,46856	6,170135667	-0,29842433	0,813139998	---
17493432	5,887912	6,355295	6,146997	5,434108	5,73028	6,330609	6,130068	5,831665667	-0,29840233	0,813152398	Thrsp
17354327	6,136833	6,246658	5,829267	5,794253	5,725229	5,798742	6,0709193	5,772741333	-0,298178	0,813278849	Dtwd2

17207821	4,761291	4,741537	5,78264	4,717408	5,098599	4,575002	5,095156	4,797003	-0,298153	0,813292943	---
17202379	6,121724	6,070164	6,118149	6,015938	5,47429	5,925413	6,1033457	5,805213667	-0,298132	0,813304781	---
17208745	4,494131	3,450484	3,763411	3,049883	3,713278	4,0506	3,9026753	3,604587	-0,29808833	0,813329398	---
17202119	7,037739	6,441803	6,902103	6,602379	6,569269	6,315832	6,7938817	6,495826667	-0,298055	0,81333819	---
17208157	5,786926	6,14201	5,35293	5,837564	5,552175	4,997966	5,760622	5,462568333	-0,29805367	0,813348942	---
17204105	3,334491	4,375749	3,126356	3,428902	3,383859	3,129751	3,6121987	3,314170667	-0,298028	0,813363412	---
17249483	6,997171	7,221625	7,123356	6,738954	6,795885	6,913256	7,1140507	6,816031667	-0,298019	0,813368486	Zcchc10
17517298	3,415633	3,06178	3,688862	2,998154	3,068892	3,20545	3,3887583	3,090832	-0,29792633	0,813420732	---
17549772	6,350611	6,686841	6,351652	6,211313	6,027229	6,257113	6,4630347	6,165218333	-0,29781633	0,813482754	---
17475200	6,790361	7,77822	7,972627	6,952509	7,699434	6,996484	7,513736	7,216142333	-0,29759367	0,813608318	---
17464099	4,67037	3,787613	4,022563	3,972953	3,89543	3,719438	4,160182	3,862607	-0,297575	0,813618845	---
17464476	6,049739	6,566692	6,042879	5,951691	5,758176	6,057033	6,21977	5,9223	-0,29747	0,813678063	Rps4y2
17471707	3,469689	3,5431	3,637059	3,162971	3,290759	3,303748	3,5499493	3,252492667	-0,29745667	0,813685583	Klra6
17206095	3,453524	3,859212	4,548837	3,689955	3,656325	3,623205	3,9538577	3,656495	-0,29736267	0,813738601	---
17532363	6,026464	5,354742	5,507944	5,329226	5,528117	5,140253	5,6297167	5,332532	-0,29718467	0,813839006	E530011L22Rik
17302953	3,56049	4,293352	3,657623	3,617253	3,499873	3,5029	3,837155	3,540008667	-0,29714633	0,813860631	---
17509425	6,386498	6,073219	6,588148	6,287605	5,73011	6,138844	6,3492883	6,052186333	-0,297102	0,81388564	Gm7385
17248074	8,858049	9,080432	8,167527	8,759406	8,159981	8,295486	8,7020027	8,404957667	-0,297045	0,813917797	---
17203121	5,138131	5,169018	5,216238	4,937291	4,6496	5,045698	5,1744623	4,877529667	-0,29693267	0,813981174	---
17509691	4,288631	4,45275	4,048102	4,210992	3,743327	3,944441	4,263161	3,966253333	-0,29690767	0,81399528	---
17207783	5,555245	5,277282	5,967017	5,265344	5,408885	5,234617	5,599848	5,302948667	-0,29689933	0,813999981	---
17346150	7,510284	8,364984	7,252854	7,306367	7,438539	7,49298	7,709374	7,412628667	-0,29674533	0,814086876	Lrg1
17206155	3,91192	3,856845	4,015909	3,574277	3,560093	3,760141	3,9282247	3,631503667	-0,296721	0,814100607	---
17200703	4,011835	4,526907	4,263421	4,15568	3,486893	4,270035	4,2673877	3,970869333	-0,29651833	0,814214978	---
17298929	3,609059	3,655184	3,685151	3,323716	3,277874	3,458378	3,649798	3,353322667	-0,29647533	0,814239247	4930529F24Rik
17208077	6,109526	6,254331	7,060384	6,439756	6,495939	5,599501	6,474747	6,178398667	-0,29634833	0,814310927	---
17391439	9,570213	10,08594	9,540816	9,420708	8,948917	9,938877	9,732323	9,436167333	-0,29615567	0,814419683	---
17417654	6,833165	6,653442	6,871411	5,876899	6,796531	6,796452	6,786006	6,489960667	-0,29604533	0,814481969	---
17278101	4,8224	4,477677	4,540364	4,177578	4,344246	4,430534	4,6134803	4,317452667	-0,29602767	0,814491943	Gm20069
17206307	6,584786	7,135889	7,031709	6,577261	6,550901	6,736206	6,9174613	6,621456	-0,29600533	0,814504552	---
17201137	6,565152	7,554242	6,681649	6,106084	6,524061	7,283061	6,933681	6,637735333	-0,29594567	0,814538239	---
17208209	6,443494	6,502504	6,431901	5,591594	6,455353	6,44328	6,4592997	6,163409	-0,29589067	0,814569292	---
17324678	9,534669	9,44437	9,956529	9,687414	9,0252	9,335439	9,6451893	9,349351	-0,29583833	0,814598841	---
17381190	3,273046	3,407134	3,565672	3,25111	2,996007	3,111315	3,415284	3,119477333	-0,29580667	0,814616721	Gm14496
17468707	12,14705	11,80702	12,02232	11,9731	10,92012	12,19604	11,99213	11,69642	-0,29571	0,814671306	---
17362986	5,814254	5,838547	5,773526	5,636175	5,498475	5,404924	5,8087757	5,513191333	-0,29558433	0,814742271	Ms4a2
17515371	6,215713	6,195512	5,506028	5,11646	5,678018	6,236146	5,9724177	5,676874667	-0,295543	0,814765614	2310047B19Rik
17320811	7,572316	6,944352	7,033257	7,128098	6,612338	6,922976	7,1833083	6,887804	-0,29550433	0,814787451	---
17549416	6,828805	6,931661	6,699513	6,538209	6,395093	6,640586	6,819993	6,524629333	-0,29536367	0,814866899	---
17549472	6,750987	6,84875	6,612961	6,193915	6,639468	6,493346	6,737566	6,442243	-0,295323	0,814889869	---
17549616	6,750987	6,84875	6,612961	6,193915	6,639468	6,493346	6,737566	6,442243	-0,295323	0,814889869	---
17290961	4,142817	3,783996	4,24387	3,804894	3,73497	3,74497	4,0568943	3,761611333	-0,295283	0,814912463	Zkscan4 // Zkscan4
17398336	3,655653	3,947698	3,609703	3,384699	3,722705	3,219907	3,7376847	3,442437	-0,29524767	0,814932421	---
17297072	7,657183	7,858128	7,663472	7,556942	7,216939	7,519249	7,726261	7,431043333	-0,29521767	0,814949368	Rpp14
17467690	7,613177	7,698248	7,534852	7,43678	7,543582	6,980692	7,6154257	7,320351333	-0,29507433	0,815030338	Ptcd3
17291408	4,816833	4,713922	4,990523	4,687298	4,381047	4,567767	4,840426	4,545370667	-0,29505533	0,815041072	Hdgfl1
17300159	4,321425	4,706363	4,781925	4,546992	4,178327	4,199318	4,6032377	4,308212333	-0,29502533	0,81505802	---
17214039	11,82173	12,18426	11,95222	11,76346	11,6919	11,61799	11,98607	11,69111667	-0,29495333	0,815098698	---
17214041	11,82173	12,18426	11,95222	11,76346	11,6919	11,61799	11,98607	11,69111667	-0,29495333	0,815098698	---
17214043	11,82173	12,18426	11,95222	11,76346	11,6919	11,61799	11,98607	11,69111667	-0,29495333	0,815098698	---
17254200	11,82173	12,18426	11,95222	11,76346	11,6919	11,61799	11,98607	11,69111667	-0,29495333	0,815098698	---
17533187	5,360108	5,270128	5,491265	5,362072	5,255424	4,619802	5,3738337	5,079099333	-0,29473433	0,815222439	Fthl17
17233955	3,675038	3,809008	3,738253	3,403051	3,630469	3,304752	3,7407663	3,446090667	-0,29467567	0,81525559	---
17547702	6,060527	6,734013	6,856715	6,036427	6,385496	6,345555	6,5504183	6,255826	-0,29459233	0,815302682	Tmx1 // Tmx1
17204889	4,583781	4,19484	4,396495	4,495619	4,010293	3,785521	4,3917053	4,097144333	-0,294561	0,81532039	---
17379755	5,619567	5,262975	5,527431	5,069933	5,255947	5,200442	5,469991	5,175440667	-0,29455033	0,815326418	---
17209107	4,103476	5,348781	4,252412	4,311048	4,666258	3,843766	4,568223	4,273690667	-0,29453233	0,815336591	---
17204229	4,126858	4,165556	3,393883	3,508988	3,171456	4,12254	3,8954323	3,600994667	-0,29443767	0,815390093	---
17291801	6,733951	6,63048	6,613302	6,327488	6,279006	6,487969	6,6592443	6,364821	-0,29442333	0,815398194	---
17257157	4,556401	4,273783	4,545965	4,089251	4,245923	4,157937	4,4587163	4,164370333	-0,294346	0,815441903	Wnt3
17291001	5,180678	5,362123	5,208409	5,045314	4,887573	4,935299	5,2504033	4,956062	-0,29434133	0,815444541	Hist1h2ak
17297707	3,874268	4,534427	4,474065	4,371422	4,037295	3,591334	4,2942533	4,000017	-0,29423633	0,815503892	Gm10398
17360770	7,954621	6,370074	8,602786	8,096727	7,201235	6,74686	7,6424937	7,348274	-0,29421967	0,815513313	Pnliprp1
17354212	7,871737	8,552839	7,93794	7,727318	7,666578	8,086124	8,1208387	7,826673333	-0,29416533	0,815544026	Sumo2 // Sumo2
17483301	7,871737	8,552839	7,93794	7,727318	7,666578	8,086124	8,1208387	7,826673333	-0,29416533	0,815544026	Sumo2 // Sumo2
17206613	4,776988	4,474808	4,353226	4,036147	3,618626	5,067842	4,5350073	4,240871667	-0,29413567	0,815560797	---
17271695	3,830976	3,58198	3,790237	3,563841	3,360709	3,396396	3,7343977	3,440315333	-0,29408233	0,815590947	4932435O22Rik
17277146	7,160452	6,657915	6,617739	6,587713	6,385033	6,581388	6,8120353	6,518044667	-0,29399067	0,81564277	Acot4
17228021	5,541862	5,068218	5,800257	4,855181	5,487424	5,185872	5,4701123	5,176159	-0,29395333	0,815663877	---
17255008	5,21912	5,762462	5,522078	5,223436	5,308512	5,090108	5,50122	5,207352	-0,293868	0,815712124	Coil
17230752	4,049535	4,124816	4,033717	3,609894	3,598463	4,118467	4,069356	3,775608	-0,293748	0,815779976	---
17212518	9,13161	9,16206	9,068143	8,790495	8,867365	8,822836	9,1206043	8,826898667	-0,29370567	0,815803914	Wdr75
17202285	5,925274	6,196027	6,075904	6,033954	5,361897	5,920302	6,065735	5,772051	-0,293684	0,815816166	---
17288068	7,367868	7,045897	7,330423	7,017123	7,179663	6,666667	7,2480627	6,954484333	-0,29357833	0,81587592	---
17208331	5,35022	5,469186	5,388319	5,412036	4,74676	5,168294	5,402575	5,10903	-0,293545	0,815894771	---
17234718	5,21164	5,254144	5,127406	4,77999	4,83613	5,096467	5,19773	4,904195667	-0,29353433	0,815900804	Gm2696
17354078	4,781614	4,373905	4,603604	4,380435	4,12126	4,377071	4,5863743	4,292922	-0,29345233	0,815947179	Ppp2r2b
17464316	6,320487	6,673879	6,503302	6,187782	5,941349	6,488434	6,4992227	6,205855	-0,29336767	0,815995066	Stk38l
17271572	5,465	5,247693	5,216352	5,229151	4,932456	4,887411	5,3096817	5,016339333	-0,29334233	0,816009394	2610035D17Rik
17277140	7,190041	6,703052	6,790363	6,898653	6,319854	6,585407	6,8944853	6,601304667	-0,29318067	0,816100841	Acot1
17434467	4,051207	3,555412	3,395264	3,410097	3,236742	3,4756	3,6672943	3,374146333	-0,293148	0,81611932	Gm9897
17253215	7,737271	7,4422	7,337918	7,284443	7,173748	7,180183	7,5057963	7,212791333	-0,293005	0,816200217	Slc6a4
17495665	6,978606	7,56513	7,029577	6,825217	7,028764	6,840595	7,1911043	6,898192	-0,29291233	0,816252645	---
17210367	7,109322	7,96022	7,408597	7,342384	7,064452	7,192606	7,492713	7,199814	-0,292899	0,816260189	---
17494126	4,015394	3,154581	3,188212	3,113429	3,03077	3,335855	3,452729	3,160018	-0,292711	0,816366564	Olfr33
17362397	9,59725	9,874919	9,891981	9,640783	9,362814	9,482586	9,78805	9,495394333	-0,29265567	0,816397876	---
17448793	8,628221	8,844774	8,559779	7,942642	8,372234	8,840322	8,6775913	8,385066	-0,29252533	0,816471632	---
17516141	3,938271	3,884454	3,734884	3,620578	3,490263	3,569305	3,8525363	3,560048667	-0,29248767	0,81649295	Olfr890
17428276	7,101809	6,354963	7,333579	6,659173	6,579268	6,674554	6,930117	6,637665	-0,292452	0,816513135	---
17439239	3,770824	4,06368	4,147257	3,425286	3,783229	3,89613	3,9939203	3,701548333	-0,292372	0,816558414	LOC100862150 // AA792892
17248075	7,989081	8,388114	8,364115	7,978767	8,127334	7,758094	8,2471033	7,954731667	-0,29237167	0,816558602	---
17403268	7,608084	8,604105	6,832762	7,059231	7,601826	7,507339	7,6816503	7,389465333	-0,292185	0,816664262	Gbp2
17547541	7,272909	7,287298	7,198265	6,90054	6,822153	7,159872	7,252824	6,960855	-0,291969	0,816786542	Smpdl3a // Smpdl3a
17545283	5,898894	5,365011	6,192803	5,477619	5,477619	5,625867	5,8189027	5,527035	-0,29186767	0,816843914	Gm5946
17200455	5,241523	4,051371	5,164009	4,610855	4,210738	4,760218	4,8189677	4,527270333	-0,29169733	0,816940361	---
17304512	5,520345	6,438168	6,28586	6,304863	5,474046	5,590679	6,0814577	5,789862667	-0,291595	0,81699831	---
17332690	5,573492	5,615763	5,407347	5,26991	5,148563	5,303517	5,5322007	5,240663333	-0,29153733	0,817030968	C530047H08Rik
17324835	11,64981	11,89955	11,79432	11,49783	11,38788	11,5834	11,781227	11,48970333	-0,29152333	0,817038896	Tfrc
17405478	7,818935	8,134015	7,653607	7,533377	7,59757	7,601535	7,8688523	7,577494	-0,29135833	0,817132346	P2ry13
17465597	6,28212	6,454931	6,655624	6,341754	6,093247	6,083728	6,464225	6,172909667	-0,29131533	0,817156701	Klf14
17410770	4,789454	5,215711	4,790069	4,652417	4,689472	4,579401	4,9317447	4,64043	-0,29131467	0,817157079	---
17257308	3,812437	3,778536	3,682415	3,459047	3,458496	3,482206	3,757796	3,466583	-0,291213	0,817214666	---
17248965	4,999211	4,883276	4,962824	4,718025	4,784539	4,469236	4,948437	4,657266667	-0,29117033	0,817238835	Olfr1390
17216458	6,547321	6,284771	6,225009	6,030245	6,115291	6,038251	6,352367	6,061262333	-0,29110467	0,817276033	Serpinb2
17200869	3,962399	4,152909	3,929618	3,638382	3,803283	3,730073	4,0149753	3,723912667	-0,29106267	0,817299826	---
17488883	3,71308	3,994236	4,042551	3,870731	3,603919	3,402061	3,9166223	3,625570333	-0,291052	0,817305869	Gm6579
17445308	9,573689	9,701186	9,673165	9,497858	9,286239	9,291237	9,6493467	9,358444667	-0,290902	0,817390851	Cyp51
17218377	7,272905	7,765017	7,439378	7,336833	7,092258	7,176293	7,4924333	7,201794667	-0,29063867	0,817540062	Acbd6 // Acbd6
17545655	3,762559	3,43629	3,221973	3,145777	3,152129	3,251266	3,4736073	3,183057333	-0,29055	0,817590308	---
17204009	7,467785	7,259436	7,525753	6,857964	7,308663	7,214866	7,417658	7,127164333	-0,29049367	0,817622234	---
17365030	8,974903	8,991075	8,863996	8,744625	8,616946	8,597043	8,9433247	8,652871333	-0,29045333	0,817645092	Chuk
17359961	7,559862	7,929881	7,375229	7,74049	7,094748	7,158409	7,6216573	7,331215667	-0,29044167	0,817651704	---
17440791	5,974051	5,308759	5,726474	5,425586	5,231492	5,482683	5,6697613	5,379920333	-0,289841	0,817992205	Svop
17338101	6,01929	6,24473	5,892731	5,581225	5,686797	6,01929	6,0522503	5,762437333	-0,289813	0,818008081	---
17460643	4,19431	4,661117	4,409919	3,993325	4,34819	4,054764	4,421782	4,132093	-0,289689	0,818078392	Dnajb8
17546556	3,431034	5,442002	4,886333	3,761294	5,598394	3,530738	4,5864563	4,296808667	-0,28964767	0,81810183	---
17547117	3,431034	5,442002	4,886333	3,761294	5,598394	3,530738	4,5864563	4,296808667	-0,28964767	0,81810183	---
17547193	3,431034	5,442002	4,886333	3,761294	5,598394	3,530738	4,5864563	4,296808667	-0,28964767	0,81810183	---
17547305	3,431034	5,442002	4,886333	3,761294	5,598394	3,530738	4,5864563	4,296808667	-0,28964767	0,81810183	---
17235358	3,846224	3,718569	3,554121	3,326245	3,471552	3,452476	3,7063047	3,416757667	-0,289547	0,818158917	---
17209767	4,322712	4,817066	3,983799	3,962707	4,452224	3,840483	4,3745257	4,085138	-0,28938767	0,81824928	---
17539824	5,285639	5,362394	5,333401	5,057498	5,270339	4,785571	5,3271447	5,037802667	-0,289342	0,818275181	Mir188
17495301	11,13766	11,03543	10,95266	10,65122	10,59678	11,01019	11,041917	10,75273	-0,28918667	0,818363289	---
17440748	5,529683	5,190235	5,404782	5,037851	4,98908	5,230219	5,3749	5,085716667	-0,28918333	0,81836518	Ficd
17530298	6,343955	5,785678	6,95706	5,708998	6,007403	6,502926	6,362231	6,073109	-0,289122	0,818399972	---
17333001	5,283826	5,212098	4,310493	4,670179	4,576556	4,692379	4,9354723	4,646371333	-0,289101	0,818411884	Tcp10a
17363923	4,332596	4,286577	4,286577	4,092439	3,930036	4,016324	4,3019167	4,012933	-0,28898367	0,818478448	Gm6642
17360557	4,982544	4,836799	4,810595	4,528279	4,732471	4,503016	4,876646	4,587922	-0,288724	0,818625777	---
17208843	4,475536	3,696008	4,930479	3,778395	4,289795	4,16785	4,367341	4,07868	-0,288661	0,818661526	---
17549884	4,956228	4,307858	4,4232	4,366511	3,949099	4,505784	4,5624287	4,273798	-0,28863067	0,818678738	---
17394911	5,696314	5,360911	5,698229	5,203068	5,206901	5,479803	5,5851513	5,296590667	-0,28856067	0,818718462	Tshz2 // Tshz2
17497320	5,27819	4,863712	5,061802	4,903631	4,735935	4,698543	5,0679013	4,779369667	-0,28853167	0,818734919	Clrn3
17334468	7,475954	7,237057	7,076881	6,895917	6,994856	7,034239	7,2632973	6,975004	-0,28829333	0,818870186	Igfals
17374184	3,554291	3,133115	3,2642	2,923963	3,16123	3,00157	3,317202	3,028921	-0,288281	0,818877186	Olfr1284

17396339	6,786716	7,280054	7,299408	6,747705	6,978274	6,775993	7,1220593	6,833990667	-0,28806867	0,818997716	---
17200307	4,041855	4,207539	3,965351	3,326097	4,279888	3,744595	4,0715817	3,783526667	-0,288055	0,819005474	---
17434614	8,989773	8,991982	8,738703	8,628046	8,591791	8,638287	8,9068193	8,619374667	-0,28744467	0,819352028	4930420K17Rik
17297537	6,883727	6,687884	6,868975	6,381446	6,659481	6,537329	6,8135287	6,526085333	-0,28744333	0,819352786	Plau
17535241	6,441562	6,294709	6,506759	6,126905	5,969165	6,285219	6,4143433	6,127096333	-0,287247	0,819464297	Gm14698 // Styx
17331258	3,704322	4,138455	4,078417	3,479082	3,683135	3,897678	3,9737313	3,686631667	-0,28709967	0,819547988	Olfr197
17210041	3,716997	4,664007	4,32321	3,566845	3,764139	4,512531	4,234738	3,947838333	-0,28689967	0,81966161	---
17427917	7,268656	7,202648	7,039365	6,87739	6,884854	6,888201	7,170223	6,883481667	-0,28674133	0,819751571	Lrrc42
17335790	7,788691	7,374094	7,376759	7,092346	7,24092	7,3462	7,5131813	7,226488667	-0,28669267	0,819779224	---
17352660	3,993967	3,886616	4,452522	3,692347	4,026258	3,754791	4,111035	3,824465333	-0,28656967	0,819849119	---
17204145	5,360274	4,690788	5,39921	4,877399	5,13571	4,577525	5,1500907	4,863544667	-0,286546	0,819862569	---
17551137	6,124844	6,281075	6,559011	5,830361	5,948112	6,326855	6,3216433	6,035109333	-0,286534	0,819869388	---
17272619	6,631808	6,982772	6,845722	6,692476	6,344227	6,564253	6,8201007	6,533652	-0,28644867	0,819917884	Socs3
17432320	6,683641	6,281294	7,044436	6,471859	6,469366	6,208812	6,6697903	6,383345667	-0,28644467	0,819920157	Cela2a
17202565	5,880214	4,591092	4,296705	4,748384	5,113096	4,047248	4,9226703	4,636242667	-0,28642767	0,819929818	---
17503972	4,337678	3,834954	4,379107	3,725342	3,815167	4,151973	4,183913	3,897494	-0,286419	0,819934744	Mir138-2
17228055	4,721271	4,04917	4,079353	4,0918	3,879184	4,019629	4,2832647	3,996871	-0,28639367	0,819949142	---
17430875	5,166723	5,054255	5,264729	5,217536	4,621195	4,787938	5,1619023	4,875556333	-0,286346	0,819976234	---
17538477	4,002837	3,298917	3,300415	3,459967	3,048411	3,234829	3,5340563	3,247735667	-0,28632067	0,819990632	Gm15114
17285863	10,4293	10,33969	10,637	10,11592	10,30614	10,12501	10,468663	10,18235667	-0,28630667	0,81999859	Hist1h2bb
17214025	11,77564	12,15783	12,01178	11,80411	11,69508	11,58716	11,98175	11,69545	-0,2863	0,820002379	---
17205751	5,014859	4,01068	4,06778	4,25377	4,03908	3,941957	4,3644397	4,078269	-0,28617067	0,820075893	---
17309150	5,136349	4,807008	5,26885	4,633667	4,679279	5,041048	5,0707357	4,784664667	-0,286071	0,820132549	---
17522846	4,224198	3,856807	3,767189	3,61583	3,94897	3,425282	3,949398	3,663360667	-0,28603733	0,820151687	---
17221430	3,927929	3,993796	4,020503	3,824565	3,694776	3,56488	3,9807427	3,694740333	-0,28600233	0,820171585	---
17365165	7,076799	7,102405	7,445776	6,694777	6,845382	7,22706	7,2083267	6,922406333	-0,28592033	0,820218203	---
17548775	5,126035	4,636714	5,005438	4,616858	4,581137	4,712501	4,922729	4,636832	-0,285897	0,820231469	Gm20326
17203225	6,366714	6,893061	6,019597	6,267233	5,33219	6,822583	6,4264573	6,140668667	-0,28578867	0,820293063	---
17299663	5,794665	5,221788	5,402421	5,12644	5,302006	5,133076	5,472958	5,187174	-0,285784	0,820295717	Slc39a2
17339392	8,822178	9,148216	8,830917	8,495843	8,729749	8,718735	8,9337703	8,648109	-0,28566133	0,820365466	Myl12a // Myl12a
17318822	8,356915	8,883728	8,721696	8,334774	8,154056	8,616725	8,654113	8,368518333	-0,28559467	0,820403376	Apol8
17293968	4,82364	4,477304	4,526514	4,497066	4,249586	4,22516	4,6091527	4,323937333	-0,28521533	0,820619116	Zfp459
17494146	5,95792	5,608035	5,903383	5,998065	5,402952	5,212838	5,8231127	5,537951667	-0,285161	0,820650022	Olfr571
17200063	5,732003	6,127577	5,86107	5,458226	6,419361	4,987658	5,9068833	5,621748333	-0,285135	0,820664812	---
17333819	3,344107	3,141584	3,259909	3,078021	2,869779	2,943053	3,2485333	2,963617667	-0,28491567	0,820789587	Vmn2r102
17350194	4,446922	4,183682	4,554382	4,114276	4,359462	3,856735	4,3949953	4,110157667	-0,28483767	0,820833965	---
17485475	5,181611	5,145191	5,988037	5,368246	4,91966	5,172426	5,4382797	5,153444	-0,28483567	0,820835103	---
17443833	6,723192	6,167305	6,414341	5,98746	6,328486	6,134519	6,434946	6,150155	-0,284791	0,820860516	Stag3
17334846	6,6884	6,629386	6,474737	6,106822	6,458492	6,37314	6,5975077	6,312818	-0,28468967	0,820918175	Tmem8
17433234	5,607018	5,744751	5,98838	5,416743	5,810663	5,25869	5,7800497	5,495365333	-0,28468433	0,82092121	---
17220204	8,11726	8,354109	8,330081	7,834036	8,06334	8,05057	8,26715	7,982648667	-0,28450133	0,821025347	Pycr2
17420011	6,814184	7,339545	7,132461	6,750718	7,065332	6,616737	7,0953967	6,810929	-0,28446767	0,821044506	---
17290687	3,211442	2,985085	3,67795	3,053408	3,072446	2,89547	3,2914923	3,007108	-0,28438433	0,821091933	---
17549418	3,520602	3,617995	3,685277	3,382242	3,312499	3,276281	3,607958	3,323674	-0,284284	0,821149039	---
17549800	3,520602	3,617995	3,685277	3,382242	3,312499	3,276281	3,607958	3,323674	-0,284284	0,821149039	---
17261840	9,587418	9,456557	9,570933	9,22545	9,207458	9,329186	9,5383027	9,254031333	-0,28427133	0,821156248	Hmmr
17451943	5,847529	5,480934	5,556934	5,363594	5,387322	5,281867	5,6284657	5,344261	-0,28420467	0,821194194	LOC433944
17206699	6,698871	6,236518	6,4778	6,257565	6,426225	5,876977	6,471063	6,186922333	-0,28414067	0,821230625	---
17550016	6,694483	6,905008	6,936689	6,57408	6,612017	6,49772	6,8453933	6,561272333	-0,284121	0,82124182	---
17206665	3,270892	4,032043	3,694433	3,340138	3,226818	3,578131	3,6657893	3,381695667	-0,28409367	0,821257379	---
17346704	5,452989	5,874473	5,354224	5,217913	5,109815	5,501756	5,560562	5,276494667	-0,28406733	0,82127237	---
17409500	7,20206	6,497338	6,596323	6,764495	5,922805	6,756454	6,7652403	6,481251333	-0,283989	0,821316963	---
17344757	4,344413	3,963817	3,955403	3,644864	3,581013	4,186702	4,0878777	3,804193	-0,28368467	0,821490236	H2-M1
17274054	9,503237	9,740484	9,71193	9,346037	9,366102	9,393005	9,6518837	9,368381333	-0,28350233	0,821594066	Smc6
17372775	4,723599	4,894148	4,090447	4,470062	3,918638	4,469186	4,569398	4,285962	-0,283436	0,821631843	---
17466639	6,208767	5,514246	5,58531	5,654772	5,349903	5,453556	5,769441	5,486077	-0,283364	0,821672849	---
17475397	4,480273	3,728287	4,245669	3,415829	4,144564	4,043867	4,1514097	3,868086667	-0,283323	0,8216962	Vmn1r184
17549104	4,502569	4,36287	4,598774	4,155656	4,249549	4,20924	4,488071	4,204815	-0,283256	0,821734361	---
17475081	7,608724	7,641607	7,091415	7,32416	7,188824	6,979235	7,4472487	7,164073	-0,28317567	0,821780119	Ceacam10
17283086	3,425978	3,649234	3,562511	3,227452	3,227162	3,333756	3,5459077	3,26279	-0,28311767	0,821813157	---
17269161	4,722233	4,216373	4,383616	4,111069	4,277917	4,084107	4,4407407	4,157697667	-0,283043	0,821855691	Gm11562
17331669	10,26708	10,54927	10,59556	10,43769	10,15058	9,974831	10,470637	10,18770033	-0,28293633	0,821916458	App
17439333	5,515033	5,125634	5,334368	5,153106	4,874356	5,098955	5,3250117	5,042139	-0,28287267	0,82195273	LOC100862180 // LOC100862166 // Gm7
17405482	7,411277	6,763249	7,033373	6,628942	6,774456	6,956004	7,0692997	6,786467333	-0,28283233	0,82197571	P2ry12
17549450	5,80862	6,082642	5,896901	5,635035	5,748254	5,556428	5,9293877	5,646572333	-0,28281533	0,821985396	---
17549844	5,80862	6,082642	5,896901	5,635035	5,748254	5,556428	5,9293877	5,646572333	-0,28281533	0,821985396	---
17500093	3,81263	3,654417	3,904843	3,371876	3,595839	3,556174	3,79063	3,507963	-0,282667	0,822069914	---
17399266	10,18054	10,4799	10,53794	10,38308	9,962758	10,00458	10,39946	10,116806	-0,282654	0,822077322	Pklr
17445361	3,499334	3,629586	3,460514	3,141926	3,369074	3,230486	3,5298113	3,247162	-0,28264933	0,822079981	1700015F17Rik
17549808	6,24758	6,0503	6,519142	6,028726	6,199124	5,741403	6,2723407	5,989751	-0,28258967	0,822113981	---
17206141	3,498395	2,929048	3,062766	2,908833	2,99204	2,741869	3,163403	2,880914	-0,282489	0,822171347	---
17491763	6,853117	6,649696	6,956575	6,665215	6,543158	6,404047	6,819796	6,537473333	-0,28232267	0,822266144	Chrna7
17305015	8,09897	8,175481	8,090767	8,011862	7,802999	7,703646	8,1217393	7,839502333	-0,282237	0,822314971	Fam35a
17283937	8,805277	8,927595	8,775408	8,250401	8,475821	8,93572	8,8360933	8,553980667	-0,28211267	0,822385842	---
17283659	4,272362	4,380488	4,769512	4,192424	4,119297	4,264435	4,4741207	4,192052	-0,28206867	0,822410924	Serpina9
17501787	9,109324	8,958291	8,741282	8,574019	8,371034	9,01784	8,936299	8,654297667	-0,28200133	0,822449309	Tm6sf2
17481622	4,224828	3,976663	4,347584	3,847447	3,783496	4,072142	4,183025	3,901028333	-0,28199667	0,822451969	Olfr503
17324522	8,620872	8,71073	8,767612	8,448166	8,424984	8,38012	8,699738	8,417756667	-0,28198133	0,82246071	Il1rap
17205769	2,984045	3,364908	3,418808	3,018262	3,018262	2,885363	3,2559203	2,973962333	-0,281958	0,822474012	---
17213244	3,695488	3,102353	3,062342	3,101547	2,973938	2,939152	3,2867277	3,004879	-0,28184867	0,822536345	---
17331311	5,507449	6,258734	5,749689	5,588867	5,497119	5,58459	5,838624	5,556858667	-0,28176533	0,822583858	Arl6
17549372	7,383432	7,71372	7,459064	6,998841	7,209864	7,502512	7,5187387	7,237072333	-0,28166633	0,822640307	---
17549756	7,383432	7,71372	7,459064	6,998841	7,209864	7,502512	7,5187387	7,237072333	-0,28166633	0,822640307	---
17202709	2,708583	2,821051	3,013081	2,447624	2,900901	2,349405	2,8475717	2,565976667	-0,281595	0,822680983	---
17376988	4,861592	4,835321	5,376161	4,693276	4,690371	4,844752	5,024358	4,742799667	-0,28155833	0,822701892	Otor
17202283	7,007739	7,506646	7,366513	6,843671	7,107009	7,085925	7,2936327	7,012201667	-0,281431	0,822774508	---
17209121	4,507498	3,206895	3,385781	3,559219	3,297863	3,398861	3,700058	3,418647667	-0,28141033	0,822786294	---
17477695	7,223588	7,291673	7,605932	7,081324	7,195959	6,999838	7,373731	7,092373667	-0,28135733	0,822816521	Zfp526
17290983	4,482075	4,255931	3,966962	4,081957	3,99095	3,788274	4,2349893	3,953727	-0,28126233	0,822870704	Olfr1535
17549236	8,129227	8,216081	7,933801	7,775282	7,839624	7,821026	8,0930363	7,811977333	-0,281059	0,822986688	---
17217169	5,165606	4,378227	4,843232	4,559986	4,324431	4,659667	4,7956883	4,514694667	-0,28099367	0,823023958	Mir135b
17288604	3,823067	3,935239	4,158772	3,716295	3,657251	3,700634	3,9723593	3,691393333	-0,280966	0,823039742	Zdhhc11 // Zdhhc11
17481334	4,44828	4,278662	4,768002	4,429624	4,163723	4,058905	4,4983147	4,217417333	-0,28089733	0,823078916	Dub1
17304336	3,881525	4,413352	3,956986	3,767435	3,881525	3,760711	4,0839543	3,803223667	-0,28073067	0,823174007	---
17200817	7,28085	7,299343	6,868594	6,773751	7,049014	6,783885	7,1495957	6,868883333	-0,28071233	0,823184468	---
17283725	7,87354	7,942955	7,365512	7,355257	7,475444	7,509577	7,7273357	7,446759333	-0,28057633	0,823262072	Clmn
17276664	7,346903	6,703051	7,256222	7,108434	6,696112	6,659925	7,1020587	6,821490333	-0,28056833	0,823266637	---
17428441	6,110123	5,866303	5,664662	5,570429	5,729863	5,499108	5,8803627	5,5998	-0,28056267	0,82326987	Cyp4x1
17408008	4,925094	5,137546	5,329479	4,867341	4,831993	4,85116	5,1307063	4,850164667	-0,28054167	0,823281854	Hist2h2ab // Hist2h2ab
17230055	5,021976	5,627894	5,218164	5,021955	4,975815	5,029049	5,2893447	5,008939667	-0,280405	0,823359847	---
17320571	5,197578	5,323676	5,429221	4,893147	5,180269	5,035893	5,316825	5,036436333	-0,28038867	0,823369169	Syt10
17313100	9,217257	9,464824	9,414299	9,139729	8,923222	9,192383	9,36546	9,085111333	-0,28034867	0,823391998	Atf4
17548786	5,502531	6,293988	6,262715	5,91326	6,299655	5,005645	6,0197447	5,73952	-0,28022467	0,823462772	LOC100862051 // LOC100862051
17545078	8,877929	8,90016	8,713315	8,55387	8,26063	8,836381	8,830468	8,550293667	-0,28017433	0,823491502	Ammecr1
17537112	7,335499	7,258668	7,099173	7,017354	6,882853	6,952766	7,2311133	6,950991	-0,28012233	0,823521184	Lpar4
17381774	5,99789	5,92283	6,108082	5,585703	5,839881	5,763129	6,0096007	5,729571	-0,28002967	0,823574082	C1ql3
17399952	4,847075	5,01361	5,060682	4,894221	4,537129	4,649988	4,973789	4,693779333	-0,28000967	0,823585499	Flg
17284593	4,100472	4,133684	4,219723	3,629627	3,783996	4,200266	4,151293	3,871296333	-0,27999667	0,82359292	---
17261710	7,953076	7,936642	8,092293	7,613745	7,664174	7,864628	7,9940037	7,714182333	-0,27982133	0,823693019	Ccdc99
17299962	3,419894	3,453525	3,161893	3,018262	3,150428	3,027549	3,345104	3,065413	-0,279691	0,823767435	---
17289466	7,75875	7,609533	7,716532	7,2102	7,420179	7,615566	7,6949383	7,415315	-0,27962333	0,823806073	Smn1
17201431	3,773955	3,848274	3,616863	3,248674	3,656024	3,495791	3,746364	3,466829667	-0,27953433	0,823856895	---
17307428	4,901779	5,018218	5,239129	4,567326	4,887949	4,865323	5,053042	4,773532667	-0,27950933	0,823871172	---
17257960	4,428437	3,773413	3,469921	3,597926	3,408998	3,826631	3,8905903	3,611185	-0,27940533	0,823930564	---
17325367	3,859775	3,671596	4,02512	3,683867	3,483624	3,551031	3,8521637	3,572840667	-0,279323	0,823977587	---
17470636	4,233253	3,961887	3,91309	3,694433	4,218961	3,356874	4,0360767	3,756756	-0,27932067	0,823978919	Vmn2r20
17370124	7,446131	7,283947	7,232042	7,186699	6,848658	7,089172	7,3207067	7,041509667	-0,279197	0,824049553	D730039F16Rik
17548230	5,5617	5,817993	5,61955	5,245234	5,44661	5,469935	5,6664143	5,387259667	-0,27915467	0,824073734	Srsf6 // Srsf6
17205463	4,559391	5,103765	4,671082	4,437134	4,408352	4,651496	4,7780793	4,498994	-0,27908533	0,824113338	---
17314452	3,722749	4,065391	3,731504	3,726579	3,536398	3,419565	3,8398813	3,560847333	-0,279034	0,824142662	Gm19442
17333889	4,363694	4,373572	4,320157	3,994031	4,298566	3,928432	4,3524743	4,073676333	-0,278798	0,824277489	Gm7732 // 4930488N24Rik
17507036	3,082192	3,134526	2,992565	2,788879	2,830029	2,754255	3,069761	2,791054333	-0,27870667	0,824329673	---
17270189	6,42341	6,119378	6,152638	5,723215	6,111916	6,024306	6,2318087	5,953145667	-0,278663	0,824354624	---
17519778	6,884656	7,27494	6,935384	6,474628	7,065073	6,719867	7,03166	6,753189333	-0,27847067	0,82446453	LOC100862063
17209703	6,363768	6,87154	6,189026	6,233036	5,791483	6,564496	6,474778	6,196338333	-0,27843967	0,824482246	---
17203755	4,870193	4,462122	4,482978	4,187627	4,799638	3,992737	4,6050977	4,326667333	-0,27843033	0,82448758	---
17255312	3,438883	4,287643	3,853615	3,559089	3,556921	3,629144	3,860047	3,581718	-0,278329	0,824545493	---
17494111	5,111319	4,977272	5,151917	4,654041	4,707684	5,044301	5,0801693	4,802008667	-0,27816067	0,824641707	Olfr551
17316369	4,116345	4,539979	4,184713	3,914845	3,981106	4,111252	4,2803457	4,002401	-0,27794467	0,824765181	---
17208123	3,687766	3,786876	3,622019	3,152561	3,60012	3,510374	3,698887	3,421018333	-0,27786867	0,82480863	---
17391432	9,135337	9,305352	8,74154	8,623352	8,796651	8,928829	9,060743	8,782944	-0,277799	0,824848461	---
17459769	4,642496	3,984261	4,473543	3,980316	3,900576	4,386159	4,3667667	4,089017	-0,27774967	0,824876667	Reg1
17267499	3,553979	3,483257	3,362922	3,075639	3,331956	3,159368	3,4667193	3,188987667	-0,27773167	0,824886959	---
17206515	6,40336	6,984237	7,089283	6,444354	6,466343	6,733019	6,8256267	6,547905333	-0,27772133	0,824892867	---

17394940	5,481442	5,216248	5,297445	5,218704	5,234473	4,708835	5,3317117	5,054004	-0,27770767	0,824900681	---
17247944	3,855311	4,027538	3,675882	3,547431	3,475262	3,703132	3,8529103	3,575275	-0,27763533	0,824942041	Mir216a
17519782	7,534582	8,050509	7,886111	7,464319	7,541828	7,632304	7,823734	7,546150333	-0,27758367	0,824971585	LOC100862068
17487964	6,085253	5,97945	6,10459	6,147036	5,566226	5,623443	6,056431	5,778901667	-0,27752933	0,825002655	Ceacam2
17462602	3,632524	4,725449	3,788273	4,405427	3,309125	3,599331	4,0487487	3,771294333	-0,27745433	0,825045544	Gm10319
17354164	5,549242	5,337951	5,080717	4,934786	5,313027	4,88785	5,3226367	5,045221	-0,27741567	0,825067657	Gm94
17340135	9,591567	9,735294	9,765466	9,864664	8,960948	9,434565	9,6974423	9,420059	-0,27738333	0,825086149	Slc3a1
17203521	5,35216	5,636202	5,271638	5,617418	5,015786	4,795042	5,42	5,142748667	-0,27725133	0,825161644	---
17547484	5,369155	6,136497	6,216041	5,346992	6,19536	5,347776	5,907231	5,630042667	-0,27718833	0,825197678	Gm17361
17208571	4,651879	3,659773	3,536486	3,32912	3,556054	4,13168	3,9493793	3,672284667	-0,27709467	0,825251255	---
17548279	7,254299	7,363413	7,334335	6,836697	6,91601	7,368103	7,317349	7,04027	-0,277079	0,825260217	Mrps16 // Mrps16
17318794	5,92822	5,687066	5,874533	5,69358	5,561219	5,40379	5,8299397	5,552863	-0,27707667	0,825261552	Apol7c
17428477	6,657928	6,819212	6,606667	6,26054	6,473758	6,51867	6,6946023	6,417656	-0,27694633	0,82533611	Cyp4b1
17550484	5,389433	5,267478	5,637729	5,286518	5,082364	5,094955	5,4315467	5,154612333	-0,27693433	0,825342975	---
17376672	4,153017	4,237621	4,299502	3,773549	4,150521	3,935471	4,2300467	3,953180333	-0,27686633	0,825381877	---
17322733	3,594168	3,360934	3,330238	3,400247	2,910352	3,144481	3,4284467	3,151693333	-0,27675333	0,825446528	---
17341080	7,939518	8,12726	7,421704	7,572309	7,587016	7,499151	7,829494	7,552825333	-0,27666867	0,825494972	Thbs2
17299804	8,140915	8,228424	8,03653	7,762445	8,023306	7,790218	8,1352897	7,858656333	-0,27663333	0,82551519	---
17202573	4,237507	3,819161	4,280604	3,854655	3,693726	3,959151	4,112424	3,835844	-0,27658	0,825545708	---
17536195	3,85881	3,164356	3,492473	3,212464	3,239093	3,234392	3,505213	3,228649667	-0,27656333	0,825555245	Samt3
17384442	10,1676	10,2143	9,976976	9,764841	9,938572	9,825994	10,119625	9,843135667	-0,27648967	0,8255974	---
17374984	5,270056	5,213404	5,038085	4,623286	4,969973	5,098835	5,1738483	4,897364667	-0,27648367	0,825600834	Pla2g4b
17202541	5,223701	6,589738	6,420228	5,565279	5,778597	6,060525	6,077889	5,801467	-0,276422	0,825636124	---
17392801	4,764913	4,392345	5,179756	4,541117	4,349486	4,617186	4,7790047	4,502596333	-0,27640833	0,825643946	---
17257756	3,8197	3,911651	3,746617	3,636175	3,57798	3,434838	3,8259893	3,549664333	-0,276325	0,825691638	---
17391329	5,191952	5,330061	5,41343	5,14484	4,966841	4,995001	5,3118143	5,035560667	-0,27625367	0,825732465	Mrps5 // Mrps5
17291005	9,977407	10,18799	10,33525	9,939813	9,877061	9,85533	10,166882	9,890734667	-0,27614767	0,825793137	Hist1h1b
17434623	5,709793	5,400239	5,084588	5,147936	5,274193	4,944068	5,3982067	5,122065667	-0,276141	0,825796953	9330182L06Rik
17333342	6,91968	7,189996	6,790496	6,727841	6,593243	6,751059	6,966724	6,690714333	-0,27600967	0,825872131	Tcp1
17340397	8,511135	8,478722	8,241899	7,884424	8,107339	8,411982	8,4105853	8,134581667	-0,27600367	0,825875566	Nanp
17299849	5,985623	5,876589	5,980342	5,715539	5,681677	5,617524	5,947518	5,67158	-0,275938	0,825913158	Trav12-2 // Trav12-2
17299926	5,985623	5,876589	5,980342	5,715539	5,681677	5,617524	5,947518	5,67158	-0,275938	0,825913158	Trav12-2 // Trav12-2
17201277	4,095555	4,197604	4,329174	4,083145	3,952867	3,758521	4,2074443	3,931511	-0,27593333	0,825915829	---
17400987	3,175358	3,108679	3,325449	2,945755	2,919485	2,916531	3,203162	2,927257	-0,275905	0,82593205	---
17210021	3,768525	2,989217	4,113689	3,373332	3,376552	3,29393	3,6238103	3,347938	-0,27587233	0,825950752	---
17427414	10,87032	10,79323	10,88073	10,68009	10,46607	10,57114	10,848093	10,57243333	-0,27566	0,826072322	Cyp2j5 // Cyp2j5
17438319	7,526827	6,697197	7,367889	7,055909	6,799585	6,909667	7,1973043	6,921720333	-0,275584	0,82611584	---
17334158	5,150805	5,036168	5,024778	4,813396	4,74478	4,826947	5,0705837	4,795041	-0,27554267	0,826139509	Prss21
17274294	10,38705	10,79713	10,72268	10,30537	10,31754	10,45758	10,63562	10,36016333	-0,27545667	0,826188757	Odc1
17393192	7,389136	7,512188	7,726011	7,197654	7,386223	7,217468	7,542445	7,267115	-0,27533	0,826261299	Eif2s2
17277382	5,58211	5,612191	5,904988	5,149411	5,530298	5,593862	5,699763	5,424523667	-0,27523933	0,826313227	Fam164c
17493145	4,601169	4,528559	4,744454	4,290939	4,54288	4,214781	4,6247273	4,349533333	-0,275194	0,826339192	Folh1
17540366	5,143085	4,72545	4,978078	5,274639	3,834164	4,912524	4,948871	4,673775667	-0,27509533	0,826395708	---
17494499	5,303232	5,039142	5,469099	5,55424	4,463277	4,968761	5,270491	4,995426	-0,275065	0,826413084	Gm4972
17347440	5,616939	5,192814	5,704479	5,193701	5,280585	5,215354	5,504744	5,22988	-0,274864	0,82652823	4930429F11Rik
17249851	7,224223	7,355288	7,443506	6,863671	7,02731	7,327846	7,3410057	7,066275667	-0,27473	0,826605003	Gm12238
17287891	6,640654	7,065793	6,627301	6,466368	6,489697	6,553597	6,777916	6,503220667	-0,27469533	0,826624865	Hnrnpk // Hnrnpk
17207137	4,835042	5,082942	5,366038	5,021255	4,959107	4,4798	5,094674	4,820054	-0,27462	0,82666803	---
17489997	4,398685	3,939047	3,710173	3,801639	3,578637	3,843915	4,0159683	3,741397	-0,27457133	0,826695917	Vmn2r57
17206167	5,974613	5,745445	6,220136	5,762691	6,021453	5,332388	5,9800647	5,705510667	-0,274554	0,82670585	---
17208271	5,990473	4,993658	6,196961	4,927314	5,984823	5,445307	5,7270307	5,452481333	-0,27454933	0,826708524	---
17260236	4,902089	4,735437	5,172318	4,661215	4,743081	4,582003	4,9366147	4,662099667	-0,274515	0,826728198	Myl7
17454043	6,256419	6,071621	6,344664	5,74652	6,100727	6,001925	6,2242347	5,949724	-0,27451067	0,826730681	Pop7
17546570	3,802287	4,421134	4,124928	3,588368	4,259945	3,676668	4,1161163	3,841660333	-0,274456	0,826762008	---
17392366	4,654294	4,630002	4,754441	4,453501	4,324789	4,437197	4,679579	4,405162333	-0,27441667	0,826784549	---
17514623	7,015756	6,825503	6,861762	6,587708	6,731783	6,560593	6,901007	6,626694667	-0,27431233	0,826844343	Trpc6
17439045	7,418775	7,982315	7,987964	7,698384	7,425979	7,441968	7,7963513	7,522110333	-0,274241	0,826885227	Thap6
17505907	5,182311	4,643996	4,71297	4,593295	4,638401	4,485877	4,8464257	4,572188333	-0,27423733	0,826887329	Dynlrb2
17517105	8,119194	8,331863	7,793736	7,577952	7,834918	8,009345	8,0815977	7,807405	-0,27419267	0,82691293	Il18
17291057	10,43133	10,4994	10,6285	10,42495	10,25528	10,05651	10,519743	10,24558	-0,27416333	0,826929743	Hist1h2ag
17200711	5,468902	5,428938	5,961455	4,960885	4,687237	6,38877	5,619765	5,345630667	-0,27413433	0,826946366	---
17207785	4,940738	5,049675	5,56387	4,987084	5,292118	4,452746	5,184761	4,910649333	-0,27411167	0,826959358	---
17358103	8,096688	7,785378	7,738977	7,639213	7,66094	7,49869	7,873681	7,599614333	-0,27406667	0,826985153	Aldh1a1
17521141	3,94631	3,937842	3,764715	3,406588	3,659495	3,760722	3,8829557	3,608935	-0,27402067	0,827011521	Glyctk // Glyctk
17464303	8,311641	7,869287	7,824749	7,695767	7,71682	7,771121	8,0018923	7,727902667	-0,27398967	0,827029292	Med21
17209551	5,91927	5,209177	5,195775	5,23655	4,625123	5,642781	5,4420073	5,168151333	-0,273856	0,82710592	---
17324675	10,13979	10,38499	10,29254	10,20113	9,794451	10,0005	10,27244	9,998693667	-0,27374633	0,827168795	---
17348677	3,71285	3,349446	3,898626	3,259196	3,401217	3,479319	3,6536407	3,379910667	-0,27373	0,82717816	---
17207643	5,150602	4,933217	5,461838	4,871519	4,841772	5,011242	5,1818857	4,908177667	-0,273708	0,827190774	---
17202917	4,795821	4,513563	4,200225	4,356897	3,661384	4,670237	4,503203	4,229506	-0,273697	0,827197081	---
17320322	5,959567	5,970691	5,871818	5,576061	5,610104	5,794884	5,9340253	5,660349667	-0,27367567	0,827209313	Mapk11
17507904	6,197981	5,990962	6,38533	5,803613	6,04399	5,905857	6,1914243	5,91782	-0,27360433	0,827250215	---
17207805	4,722287	4,416424	4,691306	4,382552	4,358117	4,269024	4,6100057	4,336564333	-0,27344133	0,827343686	---
17219758	4,233065	4,635499	4,353919	3,95241	4,209474	4,240345	4,4074943	4,134076333	-0,273418	0,827357067	Olfr248
17392644	4,812156	5,014485	4,687582	4,534985	4,654436	4,504649	4,8380743	4,56469	-0,27338433	0,827376374	Zfp937 // Zfp937
17424929	5,838023	5,794291	5,614493	5,333428	5,686551	5,406984	5,7489357	5,475654333	-0,27328133	0,827435446	---
17547678	5,239981	5,043752	5,22183	4,732695	5,133937	4,819232	5,168521	4,895288	-0,273233	0,827463167	BC106175
17331792	4,0594	3,963729	3,94652	3,7779	3,679919	3,692837	3,989883	3,716885333	-0,27299767	0,827598155	---
17446349	4,282653	4,014207	4,024135	3,821515	3,990001	3,690526	4,1069983	3,834014	-0,27298433	0,827605803	---
17201631	3,235475	3,617119	3,343412	2,98727	3,259727	3,130107	3,3986687	3,125701333	-0,27296733	0,827615555	---
17208725	4,047015	3,600963	4,010626	4,143036	3,407313	3,289561	3,8862013	3,613303333	-0,272898	0,82765533	---
17397740	4,42356	4,467243	4,709542	4,177861	4,32151	4,282647	4,5334483	4,260672667	-0,27277567	0,827725514	Mab21l1
17300177	3,978822	3,273018	3,995196	3,174551	3,602498	3,651848	3,749012	3,476299	-0,272713	0,827761469	---
17432582	4,149579	4,299635	3,968465	4,110416	3,811214	3,678123	4,1392263	3,866584333	-0,272642	0,827802207	Gm13178
17205741	6,772494	6,847279	7,020037	7,246345	5,855071	6,72056	6,8799367	6,607325333	-0,27261133	0,827819803	---
17457967	4,772723	4,645208	4,848221	4,657489	4,469808	4,321215	4,755384	4,482837333	-0,27254667	0,82785691	Olfr453
17333913	4,984587	4,965661	5,15288	4,698142	4,696802	4,89074	5,034376	4,761894667	-0,27248133	0,827894401	Zfp54
17397419	3,202485	3,401897	3,520609	3,056612	3,161713	3,089334	3,374997	3,102553	-0,272444	0,827915825	---
17205875	4,842217	5,541871	5,380659	4,760246	5,387797	4,799435	5,2549157	4,982492667	-0,272423	0,827927876	---
17357906	4,130001	3,52803	3,335827	3,555096	3,322743	3,298867	3,6646193	3,392235333	-0,272384	0,827950258	Olfr1461
17248472	3,753623	3,717539	4,042583	3,568312	3,461821	3,666566	3,837915	3,565566333	-0,27234867	0,827970535	---
17357969	5,868973	5,979105	5,894109	5,785841	5,472443	5,666938	5,9140623	5,641740667	-0,27232167	0,827986031	E030024N20Rik
17475211	7,119167	7,623431	7,132938	7,243382	6,526515	7,288682	7,2918453	7,019526333	-0,272319	0,827987561	---
17449800	7,565047	7,525761	7,208355	7,007064	7,153708	7,321727	7,4330543	7,160833	-0,27222133	0,828043616	Sowahb
17285438	8,335641	8,652486	8,403941	8,2577	8,122192	8,195668	8,4640227	8,191853333	-0,27216933	0,828073462	Inhba
17530292	9,073044	9,188985	8,858799	8,892267	8,600506	8,811756	9,040276	8,768176333	-0,27209967	0,82811345	---
17345134	5,563173	5,205867	5,827152	5,050826	5,503761	5,225855	5,532064	5,260147333	-0,27191667	0,8282185	Runx2
17336036	5,94222	6,101653	6,287532	5,60626	5,876706	6,032759	6,1104683	5,838575	-0,27189333	0,828231895	Zfp472
17455127	11,92287	12,10896	12,17562	11,87829	11,66842	11,84552	12,06915	11,79741	-0,27174	0,828319926	Cyp3a41b
17255558	6,111089	6,078799	6,338685	5,773063	5,902894	6,037633	6,176191	5,90453	-0,271661	0,828365285	Hoxb9
17202247	6,503492	5,844683	6,624807	6,420323	5,61459	6,123152	6,3243273	6,052688333	-0,271639	0,828377917	---
17203531	4,699902	4,792152	4,893477	4,874188	4,58676	4,109747	4,795177	4,523565	-0,271612	0,82839342	---
17233323	5,700515	5,885	5,66414	5,411466	5,142548	5,880968	5,749885	5,478327333	-0,27155767	0,828424619	Pln
17208057	4,807067	6,443876	5,706357	5,224879	5,630226	5,287623	5,6524333	5,380909333	-0,2715245	0,828443951	---
17213490	5,886201	5,91959	5,964336	5,699763	5,642243	5,613572	5,9233757	5,651859333	-0,27151633	0,828448354	Icos
17291026	4,359013	4,218905	3,95737	3,749326	3,952929	4,018849	4,1784293	3,907034667	-0,27139467	0,828518222	Mir1983 // Mir1983
17544618	4,712062	4,205183	4,323629	4,121412	4,290154	4,015331	4,4136247	4,142299	-0,27132567	0,828557849	Tceal6
17405985	8,67878	8,665322	8,365859	8,16373	8,364108	8,368281	8,569987	8,298706333	-0,27128067	0,828583693	Pdcd10
17283863	4,880362	4,741439	5,165891	4,509313	4,703611	4,760979	4,9292307	4,657967667	-0,271263	0,82859384	4930465M20Rik
17434324	6,403969	6,285439	6,3108	5,968584	5,953912	6,264012	6,3334027	6,062169333	-0,27123333	0,828610879	1700109H08Rik
17550621	5,791364	5,96484	5,904947	5,32567	5,863275	5,658908	5,8870503	5,615951	-0,27109933	0,828687845	---
17350916	9,751191	10,22406	9,567876	9,408643	9,647429	9,67392	9,847709	9,576664	-0,271045	0,828719055	Gm4951
17386442	3,998891	3,464716	3,807964	3,364864	3,698988	3,394702	3,7571903	3,486184667	-0,27100567	0,828741649	---
17466530	11,11496	11,37363	11,35788	10,93204	11,08943	11,01202	11,282157	11,01116333	-0,27099333	0,828748734	Rny1 // Rny1
17544269	3,75411	4,054801	3,485393	3,573776	3,454185	3,453525	3,764768	3,493828667	-0,27093933	0,828779755	---
17207285	4,842718	4,182851	5,316826	4,529495	4,39497	4,605155	4,7807983	4,509873333	-0,270925	0,828787989	---
17427759	4,01159	4,224054	4,117119	3,936534	3,724452	3,879099	4,1175877	3,846695	-0,27089267	0,828806564	---
17259515	6,712792	7,390926	7,202326	6,730617	6,920061	6,842772	7,1020147	6,83115	-0,27086467	0,828822649	---
17341998	8,017242	7,324142	7,049972	6,778168	7,127874	7,672805	7,4637853	7,192949	-0,27083633	0,828838927	---
17441622	6,549297	6,381527	6,586192	5,901093	6,419565	6,384107	6,505672	6,234921667	-0,27075033	0,828888336	---
17517344	6,814466	6,797552	6,936501	6,520792	6,859731	6,356409	6,8495063	6,578977333	-0,270529	0,829015511	---
17514914	3,152396	3,368155	2,968614	2,782912	2,968525	2,926314	3,163055	2,892583667	-0,27047133	0,829048649	Olfr834
17218618	7,049682	7,243836	7,359516	6,775074	7,02509	7,041724	7,217678	6,947296	-0,270382	0,829099986	Gm6177
17207155	4,218333	4,351281	4,835903	3,889671	4,08531	4,619398	4,4685057	4,198126333	-0,27037933	0,829101518	---
17414266	3,664326	3,405808	3,597536	3,317567	3,308085	3,230885	3,55589	3,285512333	-0,27037767	0,829102476	---
17283565	10,846	11,18619	10,93713	10,70744	10,754	10,69681	10,989773	10,71941667	-0,27035667	0,829114545	Serpina10
17330945	7,199886	7,557419	7,414974	6,723674	7,265862	7,371742	7,3907597	7,120426	-0,27033367	0,829127763	---
17516278	4,687494	3,819617	3,698507	4,000247	3,738053	3,656621	4,0685393	3,798307	-0,27023233	0,829186002	Olfr965
17327479	5,817614	5,438899	5,614362	5,368026	5,348572	5,343696	5,623625	5,353431333	-0,27019367	0,829208226	Gm15340
17328185	5,58828	5,830503	5,845039	5,983567	5,139681	5,33011	5,7546073	5,484452667	-0,27015467	0,829230642	---
17429307	6,346289	6,341325	6,670691	6,159192	6,156483	6,23221	6,4527683	6,182628333	-0,27014	0,829239072	Slc2a1
17201421	3,193337	3,202352	3,883587	3,237293	3,028871	3,202731	3,4264253	3,156298333	-0,270127	0,829246544	---
17319205	6,804591	6,928925	6,628846	6,616137	6,186626	6,749349	6,787454	6,517370667	-0,27008333	0,829271644	Mir1943
17550042	6,804591	6,928925	6,628846	6,616137	6,186626	6,749349	6,787454	6,517370667	-0,27008333	0,829271644	---

17293813	6,00452	6,62488	5,809685	5,962276	5,95051	5,716174	6,1463617	5,87632	-0,27004167	0,829295594	LOC100861733
17451618	5,108981	5,215307	5,305694	4,670573	5,243858	4,905697	5,209994	4,940042667	-0,26995133	0,829347522	---
17494785	6,952807	6,54083	6,415411	6,51229	6,118422	6,468805	6,6363493	6,366505667	-0,26984367	0,829409417	Olfr484
17207343	6,218045	6,467831	6,713406	6,159918	6,124532	6,305417	6,4664273	6,196622333	-0,269805	0,829431647	---
17494148	3,423369	3,407177	3,818205	3,111659	3,396308	3,331595	3,5495837	3,279854	-0,26972967	0,829474959	---
17210073	5,372655	6,440574	5,514146	5,628888	5,438252	5,451104	5,7757917	5,506081333	-0,26971033	0,829486075	---
17460583	9,257833	9,618731	9,448505	9,027966	9,218818	9,269177	9,4416897	9,171987	-0,26970267	0,829490483	---
17264036	10,56634	10,8043	10,78661	10,30631	10,51536	10,52648	10,719083	10,44938333	-0,2697	0,829492016	Ubb // Ubb
17543623	3,064013	2,906423	3,119306	2,719427	2,781774	2,779487	3,029914	2,760229333	-0,26968467	0,829500832	---
17265765	5,879323	5,592123	5,613785	5,468689	5,69797	5,109548	5,695077	5,425402333	-0,26967467	0,829506582	Olfr75-ps1
17205975	6,014828	6,118996	5,449462	5,440839	5,572718	5,760978	5,8610953	5,591511667	-0,26958367	0,829558906	---
17255157	7,94756	7,680013	7,515957	7,499937	7,307518	7,527583	7,71451	7,445012667	-0,26949733	0,829608549	Nme1 // Nme1
17205263	5,036091	5,482902	5,471807	5,25453	5,132448	4,79541	5,3302667	5,060796	-0,26947067	0,829623884	---
17209453	5,036091	5,482902	5,471807	5,25453	5,132448	4,79541	5,3302667	5,060796	-0,26947067	0,829623884	---
17387908	3,604136	3,4626	3,491512	3,000274	3,380821	3,368853	3,519416	3,249982667	-0,26943333	0,829645353	Olfr1225
17481258	4,886461	5,00375	4,856326	4,530252	4,818966	4,589323	4,9155123	4,646180333	-0,269332	0,829703628	Olfr646
17343065	3,998892	4,115716	3,998853	4,010492	3,460702	3,834724	4,0378203	3,768639333	-0,269181	0,829790474	Tmem217
17202753	6,051266	5,678718	5,889287	5,619815	5,881774	5,310492	5,8730903	5,604027	-0,26906333	0,829858155	---
17477774	5,824582	5,67393	5,740112	5,4814	5,453618	5,496715	5,746208	5,477244333	-0,26896367	0,829915486	Hrc
17470663	4,496997	4,626136	4,672802	4,146957	4,452805	4,38942	4,598645	4,329727333	-0,26891767	0,829941948	---
17469599	5,445192	5,186106	5,214495	5,026127	4,940777	5,072189	5,281931	5,013031	-0,2689	0,829952111	Arl8b // Arl8b
17331278	4,229183	3,915893	3,794601	3,633276	4,089642	3,410121	3,9798923	3,711013	-0,26887933	0,829964001	Olfr209
17408011	9,955184	10,10163	10,28218	9,859817	9,878599	9,794257	10,112998	9,844224333	-0,26877367	0,830024792	Hist2h2ac
17387826	4,107018	4,086456	3,965359	3,751383	3,551184	4,049964	4,0529443	3,784177	-0,26876733	0,830028435	---
17207501	4,688224	4,304318	4,439323	4,139199	4,058067	4,428494	4,4772883	4,208586667	-0,26870167	0,830066216	---
17210593	5,109779	4,117345	4,023289	4,495572	3,882602	4,066145	4,4168043	4,148106333	-0,268698	0,830068326	---
17317278	5,368343	5,424301	5,630075	5,03385	5,373813	5,209134	5,4742397	5,205599	-0,26864067	0,830101314	Klhl38
17466140	5,467815	5,199988	4,962824	4,694064	4,830341	5,300303	5,210209	4,941569333	-0,26863967	0,830101889	---
17520171	4,687747	4,761858	4,399642	4,392014	4,328706	4,322609	4,6164157	4,347776333	-0,26863933	0,830102081	Bcl2a1b
17431746	4,278763	3,995118	4,350665	4,031023	3,855352	3,932531	4,208182	3,939635333	-0,26854667	0,830155402	---
17355556	5,103646	4,873885	5,13503	4,820963	4,661455	4,824508	5,0375203	4,768975333	-0,268545	0,830156361	---
17276251	10,19579	10,3162	10,41145	9,938921	10,28788	9,891043	10,307813	10,03928133	-0,268532	0,830163841	---
17366213	3,533592	3,747169	3,590167	3,428194	3,257876	3,379503	3,6236427	3,355191	-0,26845167	0,830210068	---
17277504	5,572522	5,616361	5,416677	5,353401	5,092154	5,354788	5,5351867	5,266781	-0,26840567	0,83023654	Esrrb
17487737	6,729063	6,632448	6,783566	6,554524	6,519874	6,265808	6,7150257	6,446735333	-0,26829033	0,830302914	Lypd3
17201407	2,9792	3,504163	3,489032	2,997851	3,206417	2,963271	3,3241317	3,055846333	-0,26828533	0,830305792	---
17486549	5,870368	5,666222	5,606525	5,546197	5,183641	5,608604	5,7143717	5,446147333	-0,26822433	0,830340899	Vmn2r55
17278496	3,834382	4,05817	4,011623	3,560673	3,917973	3,621061	3,9680583	3,699902333	-0,268156	0,830380229	1700013N06Rik
17537333	4,343513	4,106837	4,476766	4,106255	3,927929	4,088724	4,3090387	4,040969333	-0,26806933	0,830430114	4930555B12Rik
17467986	5,240189	4,902711	4,511091	4,864761	4,535858	4,44945	4,8846637	4,616689667	-0,267974	0,830484991	---
17208087	6,030604	4,694382	5,547553	5,699203	5,207028	4,562646	5,4241797	5,156292333	-0,26788733	0,830534882	---
17383369	5,570166	5,298652	5,437859	5,16716	4,988748	5,347184	5,435559	5,167697333	-0,26786167	0,830549658	---
17337966	4,772182	4,811443	5,208262	4,657077	4,585196	4,74603	4,930629	4,662767667	-0,26786133	0,83054985	F630040K05Rik
17508984	4,579871	5,056297	4,472958	4,512867	4,533493	4,259206	4,703042	4,435188667	-0,26785333	0,830554455	---
17293244	8,48785	8,467905	7,997296	7,86906	8,15401	8,126847	8,3176837	8,049972333	-0,26771133	0,830636208	---
17380838	3,565978	3,595242	3,653473	3,069905	3,497076	3,444689	3,6048977	3,337223333	-0,26767433	0,830657511	---
17209901	5,598002	4,315033	4,537863	4,327482	5,063751	4,256677	4,816966	4,549303333	-0,26766267	0,830664229	---
17366012	7,141336	6,89054	6,940151	6,64043	6,761716	6,767763	6,9906757	6,723303	-0,26737267	0,830831219	4930506M07Rik
17204449	4,144273	4,614703	4,015289	4,257343	4,122986	3,591909	4,2580883	3,990746	-0,26734233	0,830848688	---
17290418	4,871888	4,921501	5,066998	4,382913	4,988061	4,687548	4,9534623	4,686174	-0,26728833	0,830879787	---
17362915	4,603234	4,239536	4,622909	4,124025	4,155724	4,384067	4,4885597	4,221272	-0,26728767	0,830880171	---
17200127	7,031338	6,643262	7,357047	7,2864	6,354492	6,588932	7,010549	6,743274667	-0,26727433	0,83088785	---
17202465	5,530803	5,623369	6,16909	5,097383	6,029415	5,394713	5,7744207	5,507170333	-0,26725033	0,830901673	---
17209697	6,217612	6,015467	6,022138	5,494682	5,940059	6,018795	6,0850723	5,817845333	-0,267227	0,830915111	---
17445458	5,738526	5,929027	5,845753	5,462355	5,820357	5,429102	5,8377687	5,570604667	-0,267164	0,830951397	---
17384567	4,032725	3,618201	3,717898	3,418746	3,62916	3,51964	3,789608	3,522515333	-0,26709267	0,830992484	Olfr355
17504361	4,779549	4,25709	4,499324	4,441251	4,242843	4,050618	4,5119877	4,244904	-0,26708367	0,830997668	4930513N10Rik
17411399	3,337027	3,237811	3,068393	2,973238	2,866648	3,002278	3,2144103	2,947388	-0,26702233	0,831032997	---
17334869	7,773373	7,677261	7,662341	7,254615	7,403752	7,653695	7,704325	7,437354	-0,266971	0,831062567	Axin1
17265806	4,969567	4,916949	5,399491	4,989106	4,585805	4,910305	5,0953357	4,828405333	-0,26693033	0,831085993	Olfr139
17224195	3,985016	3,64239	3,942236	3,642152	3,55301	3,573887	3,8565473	3,589683	-0,26686433	0,831124014	Pinc
17207047	7,130866	6,565552	7,197942	6,518817	6,94261	6,63238	6,9647867	6,697935667	-0,266851	0,831131696	---
17343733	6,47385	6,239107	6,20453	6,134898	6,089967	5,892099	6,305829	6,038988	-0,266841	0,831137457	Mir219-1
17451626	5,067943	5,28372	5,518193	5,199987	5,079043	4,790358	5,289952	5,023129333	-0,26682267	0,831148018	---
17206105	2,921324	3,372592	3,552274	2,883651	2,996264	3,166232	3,2820633	3,015382333	-0,266681	0,831229638	---
17201749	5,078034	5,148441	4,626567	4,478333	4,777692	4,797036	4,951014	4,684353667	-0,26666033	0,831241545	---
17201925	5,078034	5,148441	4,626567	4,478333	4,777692	4,797036	4,951014	4,684353667	-0,26666033	0,831241545	---
17529288	4,175577	3,863939	3,722729	3,635233	3,735492	3,591764	3,9207483	3,654163	-0,26658533	0,831284759	---
17205873	3,998368	3,980788	4,320906	4,04721	3,103977	4,349168	4,1000207	3,833451667	-0,266569	0,831294171	---
17506882	3,997155	3,372462	2,923815	3,262304	3,23445	2,996972	3,431144	3,164575333	-0,26656867	0,831294363	---
17427456	4,276242	4,348178	3,719812	3,754267	3,889416	3,901103	4,114744	3,848262	-0,266482	0,831344302	---
17205825	5,138972	5,331752	4,942384	4,403768	5,093329	5,116778	5,1377027	4,871291667	-0,266411	0,831385217	---
17200991	6,557253	6,803646	7,059279	6,59348	6,622381	6,405109	6,806726	6,540323333	-0,26640267	0,831390019	---
17206885	4,039233	3,656091	4,566916	3,78747	3,518646	4,156936	4,0874133	3,821017333	-0,266396	0,831393861	---
17201253	5,973299	6,675172	6,748675	6,451133	5,809888	6,336994	6,4657153	6,199338333	-0,266377	0,83140481	---
17403806	4,978257	5,073181	4,809271	4,503009	4,704741	4,85409	4,9535697	4,68728	-0,26628967	0,831455141	4922501L14Rik
17531256	3,195807	3,050373	3,559314	3,137245	2,905975	2,963834	3,268498	3,002351333	-0,26614667	0,831537559	---
17353921	3,8891	3,967143	3,829163	3,559413	3,610268	3,71767	3,8951353	3,629117	-0,26601833	0,83161153	---
17431895	7,919743	8,149226	8,130661	7,75589	7,69736	7,948365	8,0665433	7,800538333	-0,266005	0,831619216	Mrto4
17206925	4,510143	3,669679	4,308971	4,059829	4,111412	3,51964	4,162931	3,896960333	-0,26597067	0,831639007	---
17328623	5,61378	5,552387	6,028647	5,524989	5,430331	5,441801	5,7316047	5,465707	-0,26589767	0,831681089	Mir301b
17534480	4,001584	3,73887	3,711791	3,552221	3,446597	3,655847	3,817415	3,551555	-0,26586	0,831702803	Odz1
17226164	3,958925	3,60745	3,820165	3,338865	3,680486	3,56963	3,7955133	3,529660333	-0,265853	0,831706839	---
17324680	8,942089	9,149802	8,945508	8,850776	8,714892	8,674269	9,0124663	8,746645667	-0,26582067	0,831725479	---
17334638	5,918677	5,864749	6,085864	5,983239	5,840611	5,248079	5,95643	5,690643	-0,265787	0,831744888	Prss34
17421791	5,812199	5,428677	6,081326	5,407959	5,696877	5,420266	5,7740673	5,508367333	-0,2657	0,831795047	Ube4b // Ube4b
17391888	6,135922	6,660345	6,030159	5,72305	6,195798	6,110535	6,2754753	6,009794333	-0,265681	0,831806002	Rnf24
17350134	4,80187	5,521722	5,668384	5,091232	5,08036	5,023358	5,3306587	5,064983333	-0,26567533	0,831809269	Pou4f3
17207369	5,760762	5,495258	5,654121	5,320381	5,23194	5,56082	5,6367137	5,371047	-0,26566667	0,831814266	---
17204923	5,002137	4,623902	4,546387	5,071745	3,99065	4,313174	4,724142	4,458523	-0,265619	0,83184175	---
17389277	3,436137	2,967276	3,325073	2,914285	3,03961	2,977949	3,2428287	2,977281333	-0,26554733	0,831883073	Olfr1295
17289516	5,843367	5,876905	6,25466	5,659156	5,562541	5,95715	5,991644	5,726282333	-0,26536167	0,831990138	4933416M06Rik
17201479	7,280429	6,965769	7,430975	6,982384	7,23504	6,663696	7,2257243	6,960373333	-0,265351	0,83199629	---
17474067	5,1586	5,336134	5,282792	5,030287	5,080271	4,871159	5,2591753	4,993905667	-0,26526967	0,832043196	Slc8a2
17299770	6,227988	6,045203	6,372041	6,071146	5,874918	5,903459	6,2150773	5,949841	-0,26523633	0,83206242	---
17210715	3,230292	3,362208	2,879339	2,984408	3,040581	2,651407	3,1572797	2,892132	-0,26514767	0,83211356	---
17210379	6,322928	6,557389	6,401592	6,314855	6,137919	6,033753	6,427303	6,162175667	-0,26512733	0,832125287	---
17548547	5,62424	5,666535	5,536511	5,33449	5,152386	5,545031	5,6090953	5,343969	-0,26512633	0,832125864	Gm19475
17452552	5,886338	5,962782	5,701847	5,502065	5,617338	5,636676	5,8503223	5,585359667	-0,26496267	0,83222027	Rhof
17314619	6,171336	5,736159	5,805712	5,599983	5,387855	5,930505	5,9044023	5,639447667	-0,26495467	0,832224885	Olfr282
17207237	8,299122	8,165035	8,480125	7,836843	8,183331	8,129395	8,3147607	8,049856333	-0,26490433	0,832253921	---
17549222	5,962542	5,655678	5,773102	5,436095	5,490411	5,670346	5,7971073	5,532284	-0,26482333	0,832300649	---
17549310	5,962542	5,655678	5,773102	5,436095	5,490411	5,670346	5,7971073	5,532284	-0,26482333	0,832300649	---
17271133	3,356105	3,807793	3,427287	3,200765	3,23826	3,35769	3,530395	3,265571667	-0,26482333	0,832300649	C330019F10Rik
17209157	4,905806	3,563975	4,300386	4,353762	3,781342	3,84083	4,2567223	3,991978	-0,26474433	0,832346226	---
17548446	10,00345	10,01366	10,13788	9,792333	9,778853	9,789727	10,051663	9,786971	-0,26469233	0,832376227	Ormdl2 // Ormdl2
17387708	3,363821	3,263721	3,476107	2,942738	3,446576	2,920291	3,367883	3,103201667	-0,26468133	0,832382573	Olfr1090
17205947	3,087918	3,346694	3,09489	2,552462	3,17939	3,003618	3,1765007	2,911823333	-0,26467733	0,832384881	---
17206741	4,056411	4,87632	4,704517	4,17685	4,251987	4,415079	4,5457493	4,281305333	-0,264444	0,832519517	---
17467483	5,241522	4,221402	3,788273	4,284509	4,058739	4,114748	4,4170657	4,152665333	-0,26440033	0,832544716	Igkv5-43
17202127	5,375646	4,387482	5,576132	4,547413	4,726563	5,272151	5,1130867	4,848709	-0,26437767	0,832557797	---
17343652	6,738462	6,798849	6,838326	6,301205	6,68771	6,593768	6,791879	6,527561	-0,264318	0,83259223	Kifc1
17548561	10,98759	10,92177	11,17764	10,52826	10,9946	10,77127	11,029	10,76471	-0,26429	0,832608389	Hist4h4
17275330	3,479396	3,951692	3,344756	3,272128	3,381372	3,329571	3,591948	3,327690333	-0,26425767	0,83262705	---
17491626	4,675053	4,450325	4,156835	4,377683	3,88842	4,22439	4,4274043	4,163497667	-0,26390667	0,832829648	---
17254591	6,151423	6,35439	6,35044	6,151131	5,797728	6,115691	6,2854177	6,021516667	-0,263901	0,832832919	Tbx2
17364851	6,28579	6,326338	6,386882	6,109921	6,020686	6,0768	6,3330033	6,069135667	-0,26386767	0,832852162	Loxl4
17544623	6,857063	6,992768	6,745056	6,819145	6,633368	6,350822	6,8649623	6,601111667	-0,26385067	0,832861976	---
17202043	4,72025	5,060313	4,933918	4,801465	4,303781	4,817999	4,904827	4,641081667	-0,26374533	0,832922787	---
17305254	7,409926	7,264217	7,564596	7,123326	7,099008	7,225214	7,412913	7,149182667	-0,26373033	0,832931447	---
17551149	5,093751	4,557323	4,605971	4,346232	4,634176	4,485786	4,7523483	4,488731333	-0,263617	0,832996882	---
17550713	4,741154	4,545364	4,698846	4,443474	4,362803	4,388548	4,661788	4,398275	-0,263513	0,833056932	---
17509357	10,05031	10,46346	10,28499	9,91813	9,949007	10,14113	10,266253	10,00275567	-0,26349767	0,833065786	---
17391453	10,15822	10,40924	10,02863	10,09389	9,66152	10,05033	10,198697	9,935246667	-0,26345	0,833093311	---
17455143	11,81181	12,01033	12,08295	11,79888	11,59526	11,72153	11,968363	11,70522333	-0,26314	0,833272342	Cyp3a41a
17544149	6,906445	7,070744	7,082469	6,63286	6,72503	6,912519	7,019886	6,756803	-0,263083	0,833305265	Hmgn5
17209025	4,632242	5,082669	4,611649	4,27675	4,426515	4,834091	4,77552	4,512452	-0,263068	0,833313929	---
17205563	4,183779	4,826624	4,318992	3,946771	4,528858	4,064892	4,4431317	4,180173667	-0,262958	0,833377468	---
17387353	8,015757	8,66381	8,084977	7,932419	7,795218	8,248507	8,254848	7,992048	-0,2628	0,833468742	Mir684-1
17428184	5,41495	5,41746	5,204255	5,237514	5,005998	5,004933	5,345555	5,082815	-0,26274	0,833503406	---
17291193	6,516314	6,086165	6,958254	6,324606	6,626074	5,82204	6,5202443	6,257573333	-0,262671	0,833543271	---
17494457	3,656197	3,720973	3,511212	3,453757	3,288454	3,358461	3,6294607	3,366890667	-0,26257	0,833601628	Olfr675
17422276	7,131708	7,141238	7,124699	6,86843	6,914928	6,826742	7,1325483	6,870033333	-0,262515	0,833633408	BC046331
17417658	3,219539	3,530945	3,386981	3,123178	3,048771	3,177975	3,379155	3,116641333	-0,26251367	0,833634178	---

17204183	4,090345	4,674049	3,947503	4,36712	3,532455	4,0249	4,237299	3,974825	-0,262474	0,833657099	---
17207867	5,197813	4,27458	4,839664	4,992651	4,257906	4,27424	4,7706857	4,508265667	-0,26242	0,833688304	---
17389283	5,702022	5,393318	5,78362	5,684981	4,922761	5,483982	5,62632	5,363908	-0,262412	0,833692927	Olfr1298
17252847	4,881195	4,854825	5,598245	4,562393	5,111486	4,873169	5,1114217	4,849016	-0,26240567	0,833696586	---
17233920	8,063332	8,889742	8,145493	8,211378	8,100447	7,999616	8,366189	8,103813667	-0,26237533	0,833714116	Dnajc12
17514399	6,480965	6,265606	6,3399	6,180186	5,892026	6,227171	6,362157	6,099794333	-0,26236267	0,833721435	Gucy1a2
17220721	5,21964	5,223471	5,198286	4,949337	4,962425	4,94263	5,213799	4,951464	-0,262335	0,833737424	A430027H14Rik
17354581	3,66115	3,535062	3,364162	3,012276	3,297528	3,46365	3,5201247	3,257818	-0,26230667	0,833753798	---
17202853	4,987889	5,467115	5,001981	5,677307	4,165556	4,827296	5,1523283	4,890053	-0,26227533	0,833771906	---
17549656	5,560167	5,558844	5,087996	5,119337	5,020309	5,280662	5,4023357	5,140102667	-0,262233	0,833796372	---
17269096	7,319879	7,510281	7,293236	7,053184	7,044552	7,239404	7,3744653	7,11238	-0,26208533	0,833881719	Krt23
17210823	3,835734	3,251509	4,411266	3,401908	3,289918	4,020456	3,8328363	3,570760667	-0,26207567	0,833887307	---
17321939	4,94037	5,29907	5,209682	4,671274	5,04831	4,94348	5,1497073	4,887688	-0,26201933	0,833919869	Krt6b
17328044	7,272278	7,218213	7,514996	7,213216	7,068117	6,938282	7,3351623	7,073205	-0,26195733	0,833955707	---
17252875	4,246747	4,238329	4,924969	4,568216	3,957933	4,098376	4,470015	4,208175	-0,26184	0,834023535	Ovca2 // Ovca2
17302271	4,159794	4,245845	4,100254	3,910868	4,135276	3,674268	4,168631	3,906804	-0,261827	0,83403105	Mir759
17373594	7,984649	6,575485	7,487166	7,541494	6,672933	7,047457	7,3491	7,087294667	-0,26180533	0,834043576	---
17466827	5,849296	6,078507	6,335226	5,833065	5,939616	5,704966	6,0876763	5,825882333	-0,261794	0,834050128	Hoxa4
17281094	4,015949	4,093882	4,407966	3,956241	3,950089	3,826143	4,172599	3,910824333	-0,26177467	0,834061305	Gm7550
17507116	5,787126	5,735335	5,792907	5,511829	5,324102	5,694148	5,7717893	5,510026333	-0,261763	0,83406805	Pcp2
17411749	7,495735	8,205133	7,599589	7,415977	7,361132	7,738351	7,766819	7,505153333	-0,26166567	0,834124323	Sumo2 // Sumo2
17495338	5,830593	5,938381	5,678816	5,273749	6,018234	5,370828	5,81593	5,554270333	-0,26165967	0,834127792	Gm6816
17370369	4,107291	3,339332	3,836025	3,535753	3,306483	3,655556	3,7608827	3,499264	-0,26161867	0,834151498	Olfr365
17549594	5,637751	5,540709	5,598865	4,826247	5,648465	5,517897	5,5924417	5,330869667	-0,261572	0,83417848	---
17269181	4,468724	4,477476	4,594516	4,431158	4,047523	4,277331	4,513572	4,252004	-0,261568	0,834180793	Gm11563
17459332	5,405995	5,466555	5,53914	5,214944	5,199988	5,21218	5,4705633	5,209037333	-0,261526	0,834205078	---
17234713	3,951381	3,676794	3,624771	3,408113	3,460969	3,599459	3,750982	3,489513667	-0,26146833	0,834238423	Krtap12-1
17463164	4,201532	3,918843	4,473231	3,99526	3,925689	3,888309	4,1978687	3,936419333	-0,26144933	0,83424941	4930417O13Rik
17391433	7,19004	7,944595	7,967833	7,961093	7,681977	6,675088	7,7008227	7,439386	-0,26143667	0,834256735	---
17311807	9,738412	9,845217	9,832436	9,592678	9,594393	9,444895	9,805355	9,543988667	-0,26136633	0,834297407	Sqle
17420442	9,380869	8,949404	9,051722	8,995829	8,244864	9,357233	9,1273317	8,865975333	-0,26135633	0,83430319	---
17548769	6,729121	6,317306	6,690594	6,449657	6,225376	6,278081	6,579007	6,317704667	-0,26130233	0,834334418	---
17319887	6,173968	6,38062	6,342018	6,061645	5,91683	6,134431	6,2988687	6,037635333	-0,26123333	0,834374323	Scube1
17209811	4,157254	4,33358	3,966952	4,289447	3,771735	3,612951	4,1525953	3,891377667	-0,26121767	0,834383384	---
17204485	4,543373	3,717271	3,772908	4,479174	3,480487	3,290315	4,011184	3,749992	-0,261192	0,834398228	---
17377578	5,492933	5,747184	5,832033	5,529704	5,505284	5,253732	5,6907167	5,429573333	-0,26114333	0,834426376	Gm19332
17298789	3,600017	3,299874	3,550673	3,077448	3,347816	3,24189	3,4835213	3,222384667	-0,26113667	0,834430232	---
17322478	5,939897	5,578094	6,173803	6,045242	5,336935	5,526212	5,8972647	5,636129667	-0,261135	0,834431196	---
17205861	6,695174	6,259095	6,695449	6,199365	6,456345	6,210773	6,549906	6,288827667	-0,26107833	0,834463971	---
17534233	5,762898	5,641233	5,527022	5,22402	5,476504	5,447488	5,6437177	5,382670667	-0,261047	0,834482095	Rhox4c
17540240	6,149732	6,048284	6,316484	5,981848	5,799376	5,950966	6,1715	5,91073	-0,26077	0,834642332	Mid1ip1 // Mid1ip1
17411274	6,307521	6,464464	6,397304	6,015426	6,074964	6,296642	6,389763	6,129010667	-0,26075233	0,834652553	2510003D18Rik
17247898	7,570772	7,65414	7,622637	7,363128	7,273874	7,428659	7,6158497	7,355220333	-0,26062933	0,834723716	Fancl
17503557	5,948292	5,917654	5,970761	5,729718	5,558232	5,766989	5,945569	5,684979667	-0,26058933	0,83474686	---
17288697	3,673553	3,599598	3,672496	3,410201	3,549808	3,203878	3,648549	3,387962333	-0,26058667	0,834748403	---
17547664	5,653524	6,129026	5,865615	5,311887	5,757707	5,796894	5,8827217	5,622162667	-0,260559	0,834764411	Sgpp1 // Sgpp1
17329036	4,046615	4,069885	4,168718	3,744872	3,766265	3,992422	4,0950727	3,834519667	-0,260553	0,834767883	Olfr168
17272107	6,687612	6,878181	6,658436	6,358914	6,52035	6,563416	6,7414097	6,480893333	-0,26051633	0,834789099	Unc13d
17369256	5,786793	5,595191	5,620857	5,571354	5,130198	5,51984	5,6676137	5,407130667	-0,260483	0,834808387	Cstad
17526271	7,390908	8,191499	7,897169	7,55294	7,381503	7,763825	7,8265253	7,566089333	-0,260436	0,834835584	---
17202989	6,599955	6,263963	6,559348	6,22161	5,976152	6,444324	6,474422	6,214028667	-0,26039333	0,834860274	---
17541125	6,129892	6,199266	6,429778	6,109175	6,223402	5,645379	6,2529787	5,992652	-0,26032667	0,834898853	Rhox9
17222332	6,305115	6,577152	6,203681	6,318809	5,996674	5,989664	6,3619827	6,101715667	-0,260267	0,834933384	Mgat4a
17201299	5,340281	5,201283	5,584182	5,198033	5,086474	5,060796	5,3752487	5,115101	-0,26014767	0,835002449	---
17205413	5,260072	4,932705	4,3743	4,64934	4,003752	5,13357	4,8556923	4,595554	-0,26013833	0,835007851	---
17237074	6,404099	6,268488	6,416667	6,078994	6,183566	6,046497	6,3630847	6,103019	-0,26006567	0,83504991	Gm4340
17205037	3,105647	3,626319	4,063026	3,21334	2,945126	3,856329	3,5983307	3,338265	-0,26006567	0,83504991	---
17209227	3,105647	3,626319	4,063026	3,21334	2,945126	3,856329	3,5983307	3,338265	-0,26006567	0,83504991	---
17514375	5,126401	5,715989	5,231744	5,410526	4,935122	4,94832	5,3580447	5,097989333	-0,26005533	0,835055891	---
17285680	6,150724	6,492205	6,36229	6,057958	6,0079	6,159668	6,335073	6,075175333	-0,25989767	0,835147156	Hist1h3h
17207387	3,55084	3,931209	3,488982	2,938333	3,126055	4,126965	3,6570103	3,397117667	-0,25989267	0,83515005	---
17520162	6,072378	5,609444	5,262162	5,200412	5,722129	5,241778	5,6479947	5,388106333	-0,25988833	0,835152559	Bcl2a1a
17543474	6,755692	6,906303	6,75305	6,738044	6,189252	6,708202	6,805015	6,545166	-0,259849	0,835175329	P2ry4
17421105	4,081783	3,996995	4,25458	3,851325	3,649329	4,053164	4,1111193	3,851272667	-0,25984667	0,835176679	AI507597
17482376	8,121932	7,91569	7,917415	7,89753	7,468908	7,80906	7,9850123	7,725166	-0,25984633	0,835176872	Anks4b
17255254	3,796495	3,523825	3,7005	3,335796	3,577965	3,327574	3,6736067	3,413778333	-0,25982833	0,835187293	---
17318967	6,118221	6,614418	6,399529	5,910966	6,29726	6,144614	6,3773893	6,117613333	-0,259776	0,835217589	Gm20024
17470794	4,155193	4,137178	4,403063	3,849249	3,855119	4,211819	4,2318113	3,972062333	-0,259749	0,835233221	Mir200c
17206241	6,513399	5,727132	6,051437	6,059793	5,65215	5,800867	6,0973227	5,837603333	-0,25971933	0,835250396	---
17326180	4,146392	3,800823	4,049389	3,89663	3,651848	3,668972	3,998868	3,73915	-0,259718	0,835251168	---
17244911	3,574543	3,420459	3,376416	3,147124	3,280336	3,165066	3,4571393	3,197508667	-0,25963067	0,835301731	---
17411783	5,271616	5,349399	4,996359	4,987894	4,989284	4,861385	5,2057913	4,946187667	-0,25960367	0,835317364	Gxylt1
17315495	4,976669	4,962822	4,866097	4,518811	4,531992	4,976087	4,935196	4,67563	-0,259566	0,835339173	Hoxc11
17490061	5,44673	4,804991	5,043838	4,842052	4,735694	4,93915	5,0985197	4,838965333	-0,25955433	0,835345928	Ckap4
17312223	9,968389	10,55154	10,01709	9,75363	9,958961	10,04615	10,179006	9,919580333	-0,259426	0,835420239	BC025446
17250698	3,797718	3,889548	3,984143	3,92484	3,533249	3,435207	3,8904697	3,631098667	-0,259371	0,835452088	---
17205705	5,271256	5,480408	5,519154	4,928214	4,618869	5,945772	5,423606	5,164285	-0,259321	0,835481044	---
17349220	5,247657	4,695082	5,209489	4,821238	4,939722	4,613338	5,0507427	4,791432667	-0,25931	0,835487414	---
17549586	3,887367	3,95045	3,938339	3,699156	3,680571	3,618509	3,9253853	3,666078667	-0,25930667	0,835489344	---
17549856	3,887367	3,95045	3,938339	3,699156	3,680571	3,618509	3,9253853	3,666078667	-0,25930667	0,835489344	---
17491741	5,148887	3,859212	4,27383	4,080434	4,287658	4,136076	4,4273097	4,168056	-0,25925367	0,835520038	---
17386514	7,760699	7,679577	7,413704	7,334879	7,13191	7,609684	7,6179933	7,358824333	-0,259169	0,835569073	Gpr155
17348634	9,230777	9,092017	8,892662	8,786943	8,756107	8,894966	9,0718187	8,812672	-0,25914667	0,835582008	Rsl24d1
17533093	8,081675	8,13266	8,129285	7,689882	7,743857	8,132588	8,11454	7,855442333	-0,25909767	0,835610388	Slc38a5
17263174	4,704929	4,285113	4,164141	4,133272	4,166337	4,077432	4,3847277	4,125680333	-0,25904733	0,835639542	2210407C18Rik
17215376	5,095916	4,889553	5,024064	4,620443	4,850931	4,761446	5,0031777	4,744273333	-0,25890433	0,835722375	Sag
17524296	5,14787	5,467637	4,89105	4,917857	4,845523	4,966615	5,1688523	4,909998333	-0,258854	0,835751532	Zfp558
17204327	5,848118	5,90358	6,150392	6,664863	6,158581	4,302248	5,9673633	5,708564	-0,25879933	0,835783201	---
17210157	4,713359	4,038775	5,439075	4,760756	4,532478	4,1218	4,730403	4,471678	-0,258725	0,835826265	---
17537934	3,856377	3,78718	3,818626	3,486936	3,723422	3,475692	3,8207277	3,562016667	-0,258711	0,835834376	---
17200171	6,069372	6,145594	6,270276	5,843574	6,22826	5,637396	6,1617473	5,903076667	-0,25867067	0,835857744	---
17206941	4,220446	4,779165	5,079451	4,001702	4,279926	5,021758	4,6930207	4,434462	-0,25855867	0,835922636	---
17500561	4,700541	4,580825	4,7386	4,41519	4,348369	4,480813	4,673322	4,414790667	-0,25853133	0,835938474	Gm19410
17421172	5,472372	5,456172	5,540095	5,268831	5,082262	5,342018	5,4895463	5,231037	-0,25850933	0,835951221	---
17262032	6,953966	7,036722	6,756778	6,633507	6,602687	6,735749	6,915822	6,657314333	-0,25850767	0,835952187	Thg1l
17423735	3,788274	3,714821	3,972896	3,745603	3,613051	3,34226	3,8253303	3,566971333	-0,258359	0,836038334	---
17550304	5,238317	5,718246	5,417555	5,32264	4,957582	5,319037	5,4580393	5,199753	-0,25828633	0,836080446	---
17293495	7,493485	7,350144	7,110984	7,063318	6,945657	7,170903	7,3182043	7,059959333	-0,258245	0,8361044	Zfp935
17285616	3,395684	2,961395	3,182422	3,057233	2,890353	2,817314	3,1798337	2,921633333	-0,25820033	0,836130286	Olfr263
17370068	5,175021	4,969668	5,395727	4,987329	4,786269	4,992379	5,1801387	4,921992333	-0,25814633	0,836161583	Nron
17280842	8,146421	8,17828	8,053468	7,891395	7,763377	7,948994	8,1260563	7,867922	-0,25813433	0,836168538	Ifrd1
17202687	3,403268	4,178576	4,262013	3,33107	4,116148	3,622329	3,9479523	3,689849	-0,25810333	0,836186506	---
17257043	6,996484	5,859921	5,276218	5,587687	5,387981	6,383004	6,0442077	5,786224	-0,25798367	0,836255867	---
17239985	6,58516	6,035185	6,192322	6,11744	5,927167	5,994188	6,270889	6,012931667	-0,25795733	0,836271132	---
17341996	8,574361	8,530166	8,580027	8,160822	8,489266	8,26063	8,561518	8,303572667	-0,25794533	0,836278088	---
17206199	4,209843	4,699298	4,24931	4,098502	4,116207	4,170072	4,3861503	4,128260333	-0,25789	0,836310163	---
17386689	3,830521	4,476635	3,716267	3,788274	3,73668	3,724802	4,0078077	3,749918667	-0,257889	0,836310743	A330043C09Rik
17269210	8,929615	8,299717	8,373103	8,55708	8,043085	8,228634	8,534145	8,276266333	-0,25787867	0,836316733	---
17514447	4,822728	4,74186	4,57698	4,534544	4,357779	4,475803	4,713856	4,456042	-0,257814	0,83635422	Casp12
17429304	4,916317	4,872383	5,160456	4,677703	4,783223	4,715166	4,983052	4,725364	-0,257688	0,836427268	---
17207911	4,649293	4,148211	4,709053	3,420447	3,925302	5,387805	4,5021857	4,244518	-0,25766767	0,836439056	---
17203495	5,511565	5,694209	6,461543	5,876163	5,043095	5,975136	5,8891057	5,631464667	-0,257641	0,836454517	---
17483146	5,526106	5,287003	5,448737	5,165401	5,391914	4,93173	5,4206153	5,163015	-0,25760033	0,836478096	Doc2a
17550871	4,16504	3,884574	3,942549	3,735133	3,741338	3,743431	3,9973877	3,739967333	-0,25742033	0,836582466	---
17337100	6,912266	6,402041	6,373009	6,225267	6,389324	6,300536	6,5624387	6,305042333	-0,25739633	0,836596384	H2-Q4
17273418	4,479089	3,886024	4,006688	3,986726	3,741373	3,871522	4,1239337	3,866540333	-0,25739333	0,836598123	---
17540709	5,460854	4,753323	4,12778	4,625072	4,138225	4,806773	4,7806523	4,523356667	-0,25729567	0,836654761	E330010L02Rik
17401562	6,502315	7,667793	6,852344	6,613364	6,571856	7,065385	7,007484	6,750201667	-0,25728233	0,836662493	---
17269758	7,243708	7,271498	6,882977	6,818063	6,856327	6,952001	7,1327277	6,875463667	-0,257264	0,836673125	Psmc3ip
17533312	6,478251	6,133763	6,402093	6,129395	6,315866	5,797473	6,3380357	6,080911333	-0,25712433	0,836754127	Gm14475 // Gm14478 // Gm14482 // G
17203041	5,428036	5,100904	5,839297	5,405557	5,476769	4,714944	5,456079	5,19909	-0,256989	0,836832623	---
17446618	3,433267	3,291543	3,385417	3,268902	3,15148	2,918979	3,3700757	3,113120333	-0,25695533	0,836852152	---
17324404	3,870336	4,049008	4,103876	3,694432	3,792576	3,765601	4,00774	3,750869667	-0,25687033	0,836901458	Adipoq
17523434	4,571402	4,278431	4,562125	4,15875	4,257776	4,224859	4,4706527	4,213795	-0,25685767	0,836908806	Mir138-1
17373484	7,397264	7,353795	7,78737	7,370059	7,287926	7,10999	7,5128097	7,255991667	-0,256818	0,836931817	Pex16
17329016	4,38361	4,257744	4,804847	4,308422	4,139269	4,228765	4,482067	4,225485333	-0,25658167	0,83706893	---
17353520	7,660563	7,491646	7,566142	7,344114	7,171165	7,433368	7,5727837	7,316215667	-0,256568	0,837076859	Cdc25c
17278268	5,571921	6,740154	5,690194	5,799431	5,696031	5,737421	6,0007563	5,744294333	-0,256462	0,837138364	Serpina3f
17208539	3,750235	3,877114	3,100448	3,321608	3,027612	3,609224	3,5759323	3,319481333	-0,256451	0,837144747	---
17353239	4,91989	4,894576	4,947635	4,800868	4,742578	4,449352	4,9207003	4,664266	-0,25643433	0,837154418	---
17208165	4,921639	3,717224	4,33445	4,285893	4,341615	3,576547	4,3244377	4,068018333	-0,25641933	0,837163123	---
17398052	5,347837	5,543262	5,29924	5,122472	5,197826	5,100797	5,3967797	5,140365	-0,25641467	0,837165831	Kcnab1
17222318	5,314582	5,055939	5,161419	4,518351	5,011234	5,233159	5,1773133	4,920914667	-0,25639867	0,837175115	---
17204527	4,298872	4,376283	3,057966	3,992991	3,272016	3,698988	3,9110403	3,654665	-0,25637533	0,837188655	---

17340748	4,495431	4,646321	4,759747	4,460111	4,400162	4,272159	4,633833	4,377477333	-0,25635567	0,837200068	Pabpc6
17267011	6,02042	5,67815	5,551808	5,60418	5,533375	5,343758	5,750126	5,493771	-0,256355	0,837200455	Gm11437
17201445	4,615801	4,279224	3,869967	4,30521	4,096636	3,594117	4,2549973	3,998654333	-0,256343	0,837207418	---
17208405	4,897079	4,409905	4,657223	4,022142	4,86479	4,308708	4,6547357	4,398546667	-0,256189	0,83729679	---
17548438	5,899601	5,602109	5,793478	5,48452	5,59476	5,447355	5,7650627	5,508878333	-0,25618433	0,837299499	Rpl31-ps21
17367902	4,907097	5,055987	5,00854	4,043013	5,736903	4,42319	4,9905413	4,734368667	-0,25617267	0,83730627	---
17273860	6,13298	5,974606	6,081096	5,806522	5,841054	5,772795	6,062894	5,806790333	-0,25610367	0,837346317	Gm9548
17524014	8,261057	8,204967	8,359945	7,928149	8,088028	8,041526	8,275323	8,019234333	-0,25608867	0,837355023	Endod1
17284910	4,951236	4,932504	4,789637	4,715688	4,749449	4,44018	4,8911257	4,635105667	-0,25602	0,837394879	Calm5
17518021	5,962886	5,970294	5,596288	5,582413	5,524474	5,654776	5,843156	5,587221	-0,255935	0,837444217	Gramd2
17213313	9,980039	10,32146	10,13411	9,788256	9,901596	9,97815	10,145203	9,889334	-0,255869	0,837482529	Snord70
17200297	4,4517	3,639737	4,546481	3,883777	3,958836	4,028293	4,2126393	3,956968667	-0,25567067	0,83759767	---
17404237	5,407741	4,783803	5,275558	4,610854	5,025876	5,063693	5,1557007	4,900141	-0,25555967	0,837662116	---
17208259	6,179629	5,432933	5,800571	5,332181	5,36701	5,947299	5,8043777	5,54883	-0,25554767	0,837669084	---
17525304	6,069011	5,768292	5,783434	5,518339	5,701071	5,635291	5,873579	5,618233667	-0,25534533	0,837786572	Kcnj5
17457019	5,427541	4,931871	5,130042	4,7795	5,017818	4,926102	5,1631513	4,907806667	-0,25534467	0,837786959	Gm6117
17202717	3,634938	4,145027	4,561498	4,223877	3,863437	3,488469	4,113821	3,858594333	-0,25522667	0,837855486	---
17415439	6,057961	6,505892	6,223868	5,94431	5,963464	6,114276	6,2625737	6,00735	-0,25522367	0,837857228	Ift74
17251656	5,344561	5,184604	5,360116	5,01996	5,168269	4,935613	5,296427	5,041280667	-0,25514633	0,837902141	Dnahc2 // Dnahc2
17205949	5,731785	5,432601	5,672194	5,51203	5,336287	5,222857	5,6121933	5,357058	-0,25513533	0,83790853	---
17465495	4,564416	4,027033	4,071107	4,07992	3,944799	3,872555	4,220852	3,965758	-0,255094	0,837932537	---
17418211	9,527743	9,710381	9,816742	9,427466	9,453019	9,409177	9,6849553	9,429887333	-0,255068	0,837947638	---
17548916	5,181112	5,396249	5,189674	4,906061	5,14975	4,94628	5,2556783	5,000697	-0,25498133	0,837997977	LOC100862127
17205253	4,882144	4,718653	5,020457	4,840822	4,767256	4,248374	4,8737513	4,618817333	-0,254934	0,838025472	---
17209443	4,882144	4,718653	5,020457	4,840822	4,767256	4,248374	4,8737513	4,618817333	-0,254934	0,838025472	---
17494777	3,830368	4,548429	3,905709	3,998866	3,828968	3,69188	4,0948353	3,839904667	-0,25493067	0,838027408	Olfr478
17548178	9,709845	9,984037	9,770323	9,511137	9,462126	9,726255	9,8214017	9,566506	-0,25489567	0,838047739	Snrpd1 // Snrpd1
17344953	4,555728	4,473977	4,762714	4,216555	4,358121	4,453282	4,597473	4,342652667	-0,25482033	0,8380915	Crisp1
17318020	7,509802	6,835064	7,087128	7,355955	6,756756	6,554924	7,143998	6,889211667	-0,25478633	0,838111252	Ly6d
17486324	6,067822	5,594834	5,728548	5,851962	5,263874	5,511496	5,797068	5,542444	-0,254624	0,838205562	Vmn2r39 // Vmn2r50
17481600	4,05368	3,41642	3,345572	3,484495	3,233888	3,333573	3,605224	3,350652	-0,254572	0,838235775	Olfr477
17544277	4,544692	4,497148	4,634993	4,247334	4,076813	4,589034	4,5589443	4,304393667	-0,25455067	0,83824817	---
17297225	3,953904	3,710424	3,621779	3,598148	3,393236	3,531516	3,7620357	3,507633333	-0,25440233	0,83833436	---
17272337	3,919821	4,180895	3,854498	3,748801	3,683069	3,760224	3,9850713	3,730698	-0,25437333	0,838351212	---
17473889	6,247779	6,529311	6,183992	6,118513	6,000328	6,079179	6,3203607	6,066006667	-0,254354	0,838362447	Pla2g4c
17353973	5,841733	5,709178	6,229836	5,588605	5,780025	5,64917	5,9269157	5,6726	-0,25431567	0,838384723	---
17364067	3,498362	3,273156	3,612386	3,197239	3,216968	3,206814	3,4613013	3,207007	-0,25429433	0,83839712	Gm8978
17503531	5,035882	5,958188	5,40452	4,976927	5,141517	5,517302	5,4661967	5,211915333	-0,25428133	0,838404675	Gm15483 // Gm6834 // Rps13
17495005	8,811646	8,730811	8,781607	8,557158	8,568301	8,436261	8,774688	8,520573333	-0,25411467	0,838501537	Tmem41b
17221960	8,841848	9,024131	9,057457	8,811531	8,706896	8,64271	8,9744787	8,720379	-0,25409967	0,838510255	Ptp4a1
17200275	3,549291	3,01977	4,089684	3,294731	3,211992	3,390008	3,552915	3,298910333	-0,25400467	0,838565472	---
17461312	7,984427	8,21136	8,133105	7,780634	7,858682	7,927675	8,1096307	7,855663667	-0,253967	0,838587366	Trnt1
17512664	5,244585	5,204975	5,143346	4,932052	4,904193	4,994991	5,1976353	4,943745333	-0,25389	0,838632125	Zfp90 // Zfp90
17355058	6,076033	5,803413	5,769163	5,566299	5,640052	5,680597	5,8828697	5,628982667	-0,253887	0,838633868	---
17379247	5,464578	5,448864	5,516144	5,161073	5,305373	5,201737	5,4765287	5,222727667	-0,253801	0,838683861	Gdap1l1
17231003	7,013291	7,281988	7,114006	6,998863	6,641066	7,008046	7,1364283	6,882658333	-0,25377	0,838701883	Mfsd7b
17362998	8,724459	8,645028	8,766304	8,32428	8,688293	8,362098	8,7119303	8,458223667	-0,25370667	0,838738702	Ms4a3
17210453	5,035093	4,6442	5,348527	4,802445	4,49391	4,970431	5,0092733	4,755595333	-0,253678	0,838755368	---
17208243	4,224666	3,803509	4,443906	4,824358	3,395514	3,491278	4,1573603	3,903716667	-0,25364367	0,838775329	---
17238683	3,487528	3,581272	3,166121	3,277661	3,185835	3,010726	3,4116403	3,158074	-0,25356633	0,838820292	Olfr800
17550382	7,149941	7,135542	7,534985	7,085342	7,214953	6,759643	7,2734893	7,019979333	-0,25351	0,838853046	---
17204713	4,283261	4,958331	5,092615	4,480107	4,858694	4,234879	4,778069	4,52456	-0,253509	0,838853627	---
17206887	6,45314	6,874583	6,492192	6,821838	6,136632	6,100938	6,6066383	6,353136	-0,25350233	0,838857504	---
17316159	4,756268	4,681742	4,664667	4,46943	4,366148	4,506727	4,7008923	4,447435	-0,25345733	0,838883669	---
17548342	4,564713	3,967272	4,970807	4,555509	4,121603	4,065456	4,5009307	4,247522667	-0,253408	0,838912356	---
17493772	4,697045	4,629957	4,962824	4,62955	4,403474	4,496808	4,7632753	4,509944	-0,25333133	0,838956938	Dnajb13
17551091	7,771599	8,253116	7,86146	7,361465	7,756757	8,008162	7,9620583	7,708794667	-0,25326367	0,838996288	---
17447128	10,00153	10,25751	10,19343	9,864494	9,898525	9,929833	10,150823	9,897617333	-0,253206	0,839029825	Slbp
17293952	5,472759	5,460949	5,338237	5,215595	5,226131	5,070635	5,4239817	5,170787	-0,25319467	0,839036416	Zfp456
17551095	8,756187	8,966877	8,90267	8,44475	8,543665	8,877879	8,8752447	8,622098	-0,25314667	0,839064332	---
17462661	7,112468	8,213227	7,403903	6,982517	7,39212	7,595783	7,5765327	7,323473333	-0,25305933	0,839115126	---
17201213	5,976488	6,910195	5,748206	5,838109	5,842444	6,195274	6,2116297	5,958609	-0,25302067	0,839137616	---
17391459	9,78358	10,02748	9,933722	9,463909	9,725688	9,796346	9,9149273	9,661981	-0,25294633	0,839180853	---
17548852	11,24724	11,45379	11,41049	11,08676	11,09066	11,17531	11,370507	11,11757667	-0,25293	0,839190354	Npm1 // Npm1
17275750	8,753637	9,134849	8,883306	8,532178	8,655852	8,825318	8,9239307	8,671116	-0,25281467	0,839257444	---
17204085	3,881237	3,928409	3,271159	3,826294	3,246773	3,249409	3,6936017	3,440825333	-0,25277633	0,839279744	---
17264980	5,236124	5,470269	5,351442	5,137891	5,061914	5,099792	5,3526117	5,099865667	-0,252746	0,83929739	Spem1
17547857	5,367089	5,591519	5,500437	5,13669	5,365411	5,199074	5,4863483	5,233725	-0,25262333	0,839368755	Gm7686
17532710	4,147491	4,084204	3,928928	3,706631	3,572121	4,12418	4,053541	3,800977333	-0,25256367	0,839403471	Gm14347
17535774	3,806624	4,660543	4,357697	3,775633	4,415654	3,876293	4,2749547	4,022526667	-0,252428	0,839482409	Snora70
17277521	6,381503	6,33143	6,242923	6,164394	6,0832	5,950993	6,3186187	6,066195667	-0,252423	0,839485319	Vash1
17366077	4,344663	4,542837	4,454201	4,076249	4,458258	4,050462	4,4472337	4,194989667	-0,252244	0,839589483	Prlhr
17210583	6,494873	6,539776	5,833016	6,100221	5,271518	6,739512	6,2892217	6,037083667	-0,252138	0,839651173	---
17449176	8,573096	8,748059	8,525309	8,405363	8,321811	8,363073	8,615488	8,363415667	-0,25207233	0,839689392	Uba6
17336942	7,73824	8,552752	7,552196	7,561136	7,816056	7,709808	7,9477293	7,695666667	-0,25206267	0,839695018	---
17415012	3,809866	3,828386	4,076456	3,752676	3,56771	3,638296	3,9049027	3,652894	-0,25200867	0,839726448	---
17227259	4,860785	4,309619	5,310339	4,87613	4,000248	4,84844	4,8269143	4,574939333	-0,251975	0,839746045	---
17482958	5,326704	5,259635	4,767145	5,010428	4,775007	4,812527	5,117828	4,865987333	-0,25184067	0,839824239	Il21r
17215507	4,975654	4,946729	5,088694	4,856566	4,90601	4,493061	5,0036923	4,751879	-0,25181333	0,839840151	Trpm8
17203107	6,316354	6,143881	5,856754	6,439469	4,870202	6,251985	6,105663	5,853885333	-0,25177767	0,839860914	---
17230812	4,26169	4,196123	4,209017	4,030878	3,83587	4,044804	4,2222767	3,970517333	-0,25175933	0,839871586	4930532G15Rik
17392322	5,555735	5,089725	5,530155	5,158876	5,22318	5,038384	5,3918717	5,140146667	-0,251725	0,839891574	Bfsp1
17357916	3,495117	3,535062	3,189596	3,120223	3,141637	3,202962	3,4065917	3,154940667	-0,251651	0,839934656	Olfr1469
17204521	6,321397	6,677134	6,847494	6,887256	6,212008	5,992011	6,6153417	6,363758333	-0,25158333	0,839974052	---
17470029	8,005711	6,976807	7,794563	7,823259	7,071874	7,127463	7,5923603	7,340865333	-0,251495	0,840025483	---
17537328	3,278144	3,082788	3,324377	2,944046	3,008331	2,978619	3,2284363	2,976998667	-0,25143767	0,840058867	Ube2dnl2
17324161	6,1964	6,439648	6,54649	6,213194	5,985384	6,229747	6,3941793	6,142775	-0,25140433	0,840078277	---
17517883	5,183843	4,734819	5,142313	4,745085	4,628663	4,933015	5,020325	4,768921	-0,251404	0,840078471	1600029O15Rik
17523095	6,475125	6,51457	6,673347	6,263385	6,209988	6,436138	6,5543473	6,303170333	-0,251177	0,840210663	Exog
17208921	6,194006	5,443267	5,890755	5,697668	5,550117	5,52674	5,842676	5,591508333	-0,25116767	0,840216099	---
17510584	8,515733	8,389893	8,044269	8,089344	8,087543	8,019608	8,3166317	8,065498333	-0,25113333	0,840236094	---
17460366	8,53032	8,667221	8,74764	8,37805	8,369455	8,444567	8,6483937	8,397357333	-0,25103633	0,84029259	Add2
17371097	3,163108	3,06092	2,906852	2,681615	2,814638	2,881554	3,0436267	2,792602333	-0,25102433	0,840299579	---
17469919	7,810205	7,908475	7,993396	7,617494	7,756024	7,585545	7,9040253	7,653021	-0,25100433	0,840311228	Tmem40
17406323	4,921269	5,418945	5,696537	5,034083	4,957542	5,292228	5,3455837	5,094617667	-0,250966	0,840333556	Mnd1
17510622	5,052949	4,597743	4,983297	4,354183	4,537862	4,989256	4,8779963	4,627100333	-0,250896	0,84037433	1700007B14Rik // 1700007B14Rik
17280817	4,648077	4,237817	4,333243	4,112602	4,09349	4,260367	4,406379	4,155486333	-0,25089267	0,840376272	Scin
17201305	3,990892	3,676132	3,44197	3,181064	3,577787	3,597496	3,702998	3,452115667	-0,25088233	0,840382291	---
17229917	5,723474	5,693397	5,37489	5,296824	5,332538	5,410016	5,5972537	5,346459333	-0,25079433	0,840433554	Kcnj9
17449129	4,711978	4,065564	4,093549	4,170569	4,030103	3,918106	4,2903637	4,039592667	-0,250771	0,840447146	Epha5
17316066	5,285441	4,385087	4,847322	4,374496	4,95132	4,439846	4,8392833	4,588554	-0,25072933	0,84047142	---
17499753	6,580689	6,400684	6,983592	6,532077	6,467617	6,21332	6,6549883	6,404338	-0,25065033	0,840517444	Gm15292
17204031	5,307276	5,135328	5,020195	5,054204	4,695904	4,960766	5,1542663	4,903624667	-0,25064167	0,840522493	---
17530339	4,395125	4,392739	4,13255	4,082704	4,111078	3,974713	4,3068047	4,056165	-0,25063967	0,840523659	Ccrl1
17375418	5,576998	5,19879	5,483098	5,119884	5,207398	5,179722	5,4196287	5,169001333	-0,25062733	0,840530844	Duox1
17491743	4,388126	4,367872	4,110126	4,391096	3,619381	4,103771	4,288708	4,038082667	-0,25062533	0,840532009	---
17457731	7,513242	7,028267	7,902631	7,292474	7,274666	7,125279	7,48138	7,230806333	-0,25057367	0,840562112	Try4
17514482	4,142861	4,692843	4,124787	4,108291	4,017551	4,083046	4,3201637	4,069629333	-0,25053433	0,840585029	Mmp13
17201389	3,363629	3,247895	3,411385	3,099987	3,026534	3,145358	3,3409697	3,090626333	-0,25034333	0,840696322	---
17288992	4,61155	4,372237	4,466529	4,108543	4,121664	4,469634	4,4834387	4,233280333	-0,25015833	0,840804133	Edil3
17442944	6,88017	7,102565	7,12808	6,981374	6,46142	6,917552	7,0369383	6,786782	-0,25015633	0,840805299	Sumf2
17227867	4,218624	3,725125	4,222198	3,954882	3,749939	3,710691	4,0553157	3,805170667	-0,250145	0,840811904	2310030A07Rik // 2310030A07Rik
17208545	5,442715	6,367636	5,464479	5,548283	5,509507	5,467187	5,7582767	5,508325667	-0,249951	0,840924976	---
17201343	6,078106	5,709596	4,975495	5,220659	5,168958	5,623747	5,5877323	5,337788	-0,24994433	0,840928862	---
17518331	9,159954	9,554205	9,385495	9,086567	9,081244	9,182454	9,3665513	9,116755	-0,24979633	0,841015134	Tipin
17534110	3,669222	3,651848	3,940747	3,608424	3,556914	3,347241	3,753939	3,504193	-0,249746	0,841044476	---
17495549	6,006589	6,027441	5,827638	6,037236	5,681017	5,394232	5,9538893	5,704161667	-0,24972767	0,841055164	Itpripl2
17203651	5,951573	7,135002	6,577038	5,999209	6,251851	6,663424	6,5545377	6,304828	-0,24970967	0,841065657	---
17306575	3,379092	3,25949	3,530981	3,160903	3,160286	3,099482	3,3898543	3,140223667	-0,24963067	0,841111714	Mir208a
17525329	8,477453	8,851789	8,481564	8,413313	8,303068	8,345659	8,603602	8,354013333	-0,24958867	0,841136201	St3gal4
17480598	4,159044	4,132298	4,445481	4,285804	3,854241	3,848069	4,2456077	3,996038	-0,24956967	0,841147279	Olfr521
17200469	6,358569	5,372499	5,688902	5,671554	5,517027	5,482725	5,8066567	5,557102	-0,24955467	0,841156025	---
17204941	3,946896	4,241121	4,246565	3,659043	3,873707	4,153233	4,1448607	3,895327667	-0,249533	0,841168657	---
17284515	5,812444	5,360843	5,543863	5,123198	5,384181	5,461431	5,5723833	5,322936667	-0,24944667	0,841218996	---
17443519	6,078247	6,324999	6,243781	6,026081	6,001163	5,871469	6,2156757	5,966237667	-0,249438	0,841224049	Vgf
17414321	4,386693	4,223656	4,128762	3,912654	3,823915	4,254235	4,2463703	3,996934667	-0,24943567	0,84122541	---
17516118	4,103771	3,798211	3,715224	3,929657	3,520083	3,41926	3,872402	3,623	-0,249402	0,841245041	Olfr877
17209629	6,016384	5,473125	5,113684	5,544972	4,883263	5,426781	5,5343977	5,285005333	-0,24939233	0,841250677	---
17537034	3,67254	3,747119	4,086929	3,673891	3,569795	3,514984	3,8355293	3,586223333	-0,249306	0,841301021	Cypt2
17382350	7,600303	7,503184	7,66788	7,266349	7,457752	7,299429	7,5904557	7,341176667	-0,249279	0,841316766	Mrpl41
17299987	4,689539	4,389614	4,53302	4,317986	4,442646	4,103771	4,537391	4,288134333	-0,24925667	0,84132979	---
17548006	3,939491	3,652398	3,917799	3,658908	3,472038	3,630979	3,8365627	3,587308333	-0,24925433	0,841331151	4932414J04Rik // 4932414J04Rik
17500756	4,713902	4,284573	4,561121	4,018925	4,361562	4,431349	4,5198653	4,270612	-0,24925333	0,841331734	---
17548595	4,270096	4,331158	4,627749	4,230816	3,993623	4,257012	4,4096677	4,160483667	-0,249184	0,841372168	E330027M22Rik // E330027M22Rik

17203179	6,743193	6,474185	5,896478	6,006757	5,858388	6,501807	6,3712853	6,122317333	-0,248968	0,841498147	---
17345802	8,353017	8,578563	8,100492	7,872184	8,41162	8,001694	8,344024	8,095166	-0,248858	0,841562311	Tspo2
17205201	5,699337	5,550507	6,262158	5,630948	5,99763	5,136912	5,837334	5,588496667	-0,24883733	0,841574366	---
17209391	5,699337	5,550507	6,262158	5,630948	5,99763	5,136912	5,837334	5,588496667	-0,24883733	0,841574366	---
17211416	4,462037	4,288148	4,514443	4,474697	4,012451	4,030991	4,4215427	4,172713	-0,24882967	0,841578838	Khdc1a
17366647	4,856784	4,662864	4,847904	4,588504	4,60736	4,425223	4,789184	4,540362333	-0,24882167	0,841583505	4930412O13Rik
17200161	4,429003	4,787567	5,151296	4,640587	4,091246	4,889587	4,7892887	4,540473333	-0,24881533	0,8415872	---
17201169	4,80187	5,997268	6,150321	4,861149	4,789005	6,552869	5,6498197	5,401007667	-0,248812	0,841589144	---
17417238	4,414293	4,563177	4,351767	4,325305	4,35107	3,906616	4,443079	4,194330333	-0,24874867	0,84162609	---
17550172	4,414293	4,563177	4,351767	4,325305	4,35107	3,906616	4,443079	4,194330333	-0,24874867	0,84162609	---
17401552	4,850863	4,742236	4,830682	4,298994	4,673085	4,705569	4,807927	4,559216	-0,248711	0,841648064	A630076J17Rik
17550831	4,073342	3,97581	3,76751	3,784875	3,736331	3,549343	3,9388873	3,690183	-0,24870433	0,841651953	---
17473734	4,682691	4,277254	4,38043	4,784308	4,109169	3,700789	4,4467917	4,198088667	-0,248703	0,841652731	Vmn1r79
17306098	4,731449	4,106925	4,830681	4,387737	4,260402	4,275084	4,5563517	4,307741	-0,24861067	0,841706599	Rnase1
17259956	5,73256	5,72791	5,855865	5,495859	5,566069	5,508631	5,7721117	5,523519667	-0,248592	0,84171749	Ccdc157
17232135	6,758232	6,189524	6,508299	6,320062	6,176253	6,214214	6,4853517	6,236843	-0,24850867	0,841766111	1110021L09Rik
17513681	7,646111	7,700671	7,871523	7,639994	7,211071	7,621852	7,739435	7,490972333	-0,24846267	0,841792951	Mvd
17547006	6,368285	6,454365	6,418578	5,839908	6,548105	6,107913	6,4137427	6,165308667	-0,248434	0,841809677	LOC100040022 // LOC100040022
17547018	6,368285	6,454365	6,418578	5,839908	6,548105	6,107913	6,4137427	6,165308667	-0,248434	0,841809677	LOC100040022 // LOC100040022
17210095	3,103977	3,872743	3,84241	3,709497	3,055827	3,308658	3,6063767	3,357994	-0,24838267	0,841839631	---
17549344	6,075953	6,699915	6,125299	6,001853	6,193914	5,960277	6,300389	6,052014667	-0,24837433	0,841844493	---
17417702	7,636102	7,650241	7,696462	7,341078	7,372838	7,523895	7,660935	7,412603667	-0,24833133	0,841869585	Slc6a9
17301440	7,912385	8,419163	7,945687	7,842768	7,926519	7,76324	8,0924117	7,844175667	-0,248236	0,841925218	Ccdc25
17387678	3,76082	3,628624	3,351053	3,271955	3,325558	3,398418	3,5801657	3,331977	-0,24818867	0,841952841	Olfr1065
17522649	4,987772	4,088345	4,628929	4,540311	4,290144	4,130106	4,5683487	4,320187	-0,24816167	0,841968598	---
17357460	10,71189	10,84891	10,68347	10,67487	10,27765	10,5475	10,74809	10,50000667	-0,24808333	0,842014316	Fads1
17298908	4,550448	5,321703	4,558482	4,60342	4,736794	4,346232	4,810211	4,562148667	-0,24806233	0,842026572	---
17285783	6,296312	5,809521	6,210334	6,111647	5,889296	5,571419	6,105389	5,857454	-0,247935	0,842100893	Vmn1r213
17547881	4,100347	4,297317	4,253498	3,913997	4,047131	3,946247	4,217054	3,969125	-0,247929	0,842104395	LOC100861767
17480673	3,980237	4,130812	4,669552	3,87633	4,090569	4,069972	4,2602003	4,012290333	-0,24791	0,842115486	Gpx2-ps1
17547676	6,632454	6,478787	6,679047	6,448197	6,214076	6,384287	6,5967627	6,348853333	-0,24790933	0,842115875	LOC100862592
17382432	5,118894	5,352546	5,212921	5,004309	4,975796	4,960607	5,2281203	4,980237333	-0,247883	0,842131246	Slc34a3
17216937	8,652247	8,878441	8,682852	8,260531	8,621439	8,588007	8,7378467	8,489992333	-0,24785433	0,84214798	Yod1
17275858	4,936153	4,587017	4,548979	4,369399	4,696799	4,262436	4,6907163	4,442878	-0,24783833	0,842157319	Wdr20b
17432861	5,229212	5,190452	5,67442	5,453053	4,958675	4,939054	5,3646947	5,116927333	-0,24776733	0,842198766	---
17205871	4,007326	3,551664	3,872498	3,294614	3,590665	3,802999	3,810496	3,562759333	-0,24773667	0,842216668	---
17430928	6,328512	6,73514	6,528962	6,245128	6,242735	6,361549	6,5308713	6,283137333	-0,247734	0,842218225	---
17324685	8,549548	9,066596	8,854417	8,48256	8,726293	8,518739	8,8235203	8,575864	-0,24765633	0,842263567	---
17550482	7,687866	8,203992	8,166586	7,918852	7,860181	7,536593	8,0194813	7,771875333	-0,247606	0,842292952	---
17287247	6,709478	5,860794	6,646351	6,208179	6,109245	6,156383	6,405541	6,157935667	-0,24760533	0,842293342	2310081J21Rik
17329844	7,689945	7,880116	7,914739	7,514369	7,486753	7,741035	7,8282667	7,580719	-0,24754767	0,84232701	Gm19316
17406908	9,858426	10,03193	9,980124	9,740846	9,600513	9,786574	9,9568267	9,709311	-0,24751567	0,842345694	Fdps
17207561	4,593172	4,504327	4,260824	4,06489	4,440639	4,110581	4,4527743	4,20537	-0,24740433	0,8424107	---
17549858	6,389275	6,609607	6,692292	6,472652	6,224862	6,25157	6,5637247	6,316361333	-0,24736333	0,842434641	---
17389481	9,069777	9,088444	8,915968	8,682808	8,77621	8,873126	9,0247297	8,777381333	-0,24734833	0,8424434	Arhgap11a
17549226	5,235996	4,968979	5,129273	5,019943	4,879179	4,693149	5,111416	4,864090333	-0,24732567	0,842456636	---
17269839	8,89074	8,981922	9,045818	8,684824	8,749548	8,742223	8,9728267	8,725531667	-0,247295	0,842474544	Ccdc56
17499715	6,922816	6,849167	6,874813	6,643484	6,681413	6,580235	6,8822653	6,635044	-0,24722133	0,842517563	Gm15299
17457753	6,254202	6,250575	6,448393	6,251449	6,02053	5,9396	6,3177233	6,070526333	-0,247197	0,842531774	Gm5409
17213747	4,450041	5,288444	4,467158	4,647613	4,557074	4,259438	4,7352143	4,488041667	-0,24717267	0,842545985	Pth2r
17399792	3,07022	3,901928	3,182934	3,105527	3,005392	3,30269	3,3850273	3,137869667	-0,24715767	0,842554745	---
17204775	4,792655	4,614358	4,379121	4,265236	4,14479	4,684717	4,6120313	4,364914333	-0,247117	0,842578495	---
17208739	3,806447	4,386374	3,29143	3,389935	3,665522	3,687512	3,8280837	3,580989667	-0,247094	0,842591928	---
17204059	6,910002	6,640103	7,002084	6,040918	6,975255	6,794739	6,8507297	6,603637333	-0,24709233	0,842592901	---
17299976	3,507208	3,299682	3,226701	3,183864	3,003392	3,105126	3,3445303	3,097460667	-0,24706967	0,84260614	---
17544631	4,573495	4,497567	4,839961	4,400775	4,449985	4,31811	4,6366843	4,389623333	-0,247061	0,842611201	Nxf7
17463169	5,907457	5,963969	5,946273	5,520047	5,833498	5,72321	5,939233	5,692251667	-0,24698133	0,842657732	Scnn1a
17214656	5,435875	5,050049	5,44266	4,960778	5,226812	5,00025	5,309528	5,062613333	-0,24691467	0,842696672	Gm17751
17293484	4,199631	4,555913	4,110093	4,028957	3,970986	4,125096	4,2885457	4,041679667	-0,246866	0,8427251	Gm3604
17223279	3,558128	3,58978	3,377428	3,3142	3,227521	3,243055	3,5084453	3,261592	-0,24685333	0,842732499	Hsfy2
17266196	4,872674	4,629346	4,3949	4,298759	4,663245	4,194761	4,6323067	4,385588333	-0,24671833	0,842811361	Mir423
17447900	6,140638	5,569081	5,830303	5,856215	5,509609	5,434389	5,846674	5,600071	-0,246603	0,84287874	---
17373634	9,678249	9,715619	8,75813	9,474981	9,130846	8,806477	9,3839993	9,137434667	-0,24656467	0,842901137	---
17477424	7,146319	7,143913	7,158885	7,265117	6,797359	6,647102	7,1497057	6,903192667	-0,246513	0,842931324	Snord88c
17246412	4,03337	3,76167	3,714177	3,766707	3,628288	3,374688	3,8364057	3,589894333	-0,24651133	0,842932297	Olfr773
17546978	3,829972	4,913378	4,596384	3,903755	4,46761	4,22885	4,446578	4,200071667	-0,24650633	0,842935219	LOC100504792
17391428	7,83724	7,822444	7,794558	7,505323	7,75392	7,455506	7,8180807	7,571583	-0,24649767	0,842940283	---
17550302	3,519216	3,926889	3,630329	3,385212	3,394082	3,557687	3,6921447	3,445660333	-0,24648433	0,842948073	---
17204551	4,116519	4,158614	4,729473	3,784282	4,45823	4,022655	4,3348687	4,088389	-0,24647967	0,8429508	---
17453124	7,16591	7,409396	7,414308	7,193417	6,971354	7,085506	7,3298713	7,083425667	-0,24644567	0,842970666	Psph
17367234	5,550205	5,398242	5,451416	5,125748	5,358975	5,176032	5,466621	5,220251667	-0,24636933	0,843015269	---
17203135	6,730124	6,979079	7,237156	6,888848	7,092042	6,226391	6,9821197	6,735760333	-0,24635933	0,843021112	---
17547511	6,558072	6,131495	6,361244	6,072539	6,30722	5,932068	6,3502703	6,103942333	-0,246328	0,843039422	Gm19629
17464789	4,694891	4,392323	4,568697	4,182525	4,40977	4,324682	4,5519703	4,305659	-0,24631133	0,843049161	A430035B10Rik
17538295	3,673685	3,366317	3,696271	3,417102	3,325747	3,25457	3,5787577	3,332473	-0,24628467	0,843064744	Gm5643
17548967	5,652411	5,515957	5,762578	5,553118	5,374032	5,264957	5,6436487	5,397369	-0,24627967	0,843067666	Klhl30 // Klhl30
17288079	8,717155	9,188541	8,531349	8,393051	8,4221	8,883108	8,8123483	8,566086333	-0,246262	0,84307799	---
17206057	6,59569	5,864906	6,355297	6,119499	5,296102	6,661655	6,2719643	6,025752	-0,24621233	0,843107014	---
17525782	5,133839	4,813567	5,104098	4,877469	4,677968	4,757564	5,017168	4,771000333	-0,24616767	0,843133118	Olfr975
17526270	8,991584	9,436893	9,169979	9,007498	9,076019	8,776505	9,1994853	8,953340667	-0,24614467	0,843146559	---
17405916	3,71595	3,700312	3,854547	3,45599	3,453775	3,622686	3,7569363	3,510817	-0,24611933	0,843161365	Gm20356
17465042	3,886405	4,199978	3,640165	3,767466	3,682482	3,538327	3,9088493	3,662758333	-0,246091	0,843177924	---
17374045	3,791183	4,28938	4,630162	4,547797	3,599253	3,825422	4,2369083	3,990824	-0,24608433	0,84318182	---
17202245	2,783593	3,729402	3,569378	3,015697	2,800788	3,528192	3,360791	3,114892333	-0,24589867	0,84329034	---
17266672	6,073897	5,979402	6,021551	5,533109	5,805862	5,998183	6,02495	5,779051333	-0,24589867	0,84329034	1110002N22Rik
17310669	7,253608	7,060525	7,148872	6,880512	7,111554	6,733378	7,154335	6,908481333	-0,24585367	0,843316644	---
17537713	6,640671	6,871685	6,67711	6,290592	6,538911	6,622557	6,729822	6,48402	-0,245802	0,843346846	Timm8a1 // Timm8a1
17237359	3,742531	3,93688	3,777539	3,572921	3,788561	3,358111	3,8189833	3,573197667	-0,24578567	0,843356394	---
17540148	6,807381	7,029463	6,972233	6,37561	6,829175	6,867389	6,936359	6,690724667	-0,24563433	0,843444863	Lancl3
17221197	8,888769	8,943101	9,041188	8,826748	8,551133	8,758317	8,9576863	8,712066	-0,24562033	0,843453048	Tcf24
17467534	3,212868	3,041736	3,280967	3,001371	2,824556	2,97295	3,1785237	2,932959	-0,24556467	0,843485594	Igkv6-23 // Igkv6-23
17445041	4,348317	4,613559	4,491062	4,563831	4,04871	4,103771	4,4843127	4,238770667	-0,245542	0,843498846	---
17391905	5,808016	5,132988	5,323094	5,229763	5,062498	5,235287	5,421366	5,175849333	-0,24551667	0,843513658	---
17526674	9,407256	9,535685	9,497356	9,127474	9,284061	9,29231	9,480099	9,234615	-0,245484	0,843532758	Rexo2
17512688	5,976363	5,841822	6,063575	5,733023	5,597236	5,815175	5,9605867	5,715144667	-0,245442	0,843557315	---
17287827	9,24642	9,360226	9,023161	9,025167	9,017265	8,851105	9,2099357	8,964512333	-0,24542333	0,84356823	Tgfbi
17514435	6,229616	6,945282	6,166361	6,158886	6,154302	6,291867	6,4470863	6,201685	-0,24540133	0,843581094	Casp4
17312229	4,295412	4,214237	4,425872	4,128516	3,899025	4,171778	4,3118403	4,066439667	-0,24540067	0,843581483	Ly6f
17361424	7,190185	6,853067	7,159495	6,919693	6,986381	6,560705	7,0675823	6,822259667	-0,24532267	0,843627093	Peli3
17539700	6,037985	5,970129	5,980505	5,898148	5,604373	5,750322	5,9962063	5,750947667	-0,24525867	0,843664519	G530011O06Rik
17508111	6,14572	6,466726	6,28046	5,810278	6,150321	6,196642	6,2976353	6,052413667	-0,24522167	0,843686156	Ap3m2
17413211	7,173331	6,882348	7,274223	6,778965	6,922843	6,893061	7,1099673	6,864956333	-0,245011	0,843809362	Vcp // Vcp
17246416	3,172019	3,015235	3,101682	2,91226	2,957492	2,684294	3,096312	2,851348667	-0,24496333	0,843837242	Olfr777
17247078	3,362695	3,271194	3,489826	3,027829	3,330936	3,030104	3,3745717	3,129623	-0,24494867	0,843845821	---
17300798	3,748432	3,574645	3,768064	3,479743	3,518019	3,358825	3,697047	3,452195667	-0,24485133	0,843902754	---
17401228	6,015285	6,166364	6,056664	5,670674	5,906558	5,926736	6,0794377	5,834656	-0,24478167	0,843943506	Hipk1 // Hipk1
17205187	4,442914	4,686731	4,324529	3,903131	4,550816	4,265926	4,4847247	4,239957667	-0,244767	0,843952086	---
17209377	4,442914	4,686731	4,324529	3,903131	4,550816	4,265926	4,4847247	4,239957667	-0,244767	0,843952086	---
17208253	6,091901	6,006292	5,93669	5,81494	5,775929	5,710063	6,0116277	5,766977333	-0,24465033	0,844020337	---
17538755	9,449538	9,346746	9,230474	9,477128	8,45747	9,358333	9,3422527	9,097643667	-0,244609	0,844044519	---
17467398	5,569987	5,233845	5,591133	5,403378	5,129313	5,12848	5,4649883	5,220390333	-0,244598	0,844050954	Igkv4-91 // Igkv4-91
17372759	5,406239	4,982283	5,016829	5,203531	4,752627	4,717605	5,135117	4,890587667	-0,24452933	0,844091129	Olfr1014
17547969	8,767676	9,214461	8,825141	8,734544	8,640304	8,698872	8,9357593	8,69124	-0,24451933	0,844096979	---
17273536	6,737225	7,022278	6,921865	6,770635	6,703516	6,473782	6,8937893	6,649311	-0,24447833	0,844120968	Zfp750
17249922	8,019441	7,591077	7,794558	7,509098	7,441741	7,720911	7,801692	7,55725	-0,244442	0,844142227	---
17231314	4,264831	4,486719	4,469658	4,136093	4,250175	4,10173	4,4070693	4,162666	-0,24440333	0,844164852	Cr2
17274955	4,249774	4,209076	4,420883	3,950118	4,055953	4,140514	4,2932443	4,048861667	-0,24438267	0,844176945	Ferd3l
17202533	7,437266	7,446081	7,570873	7,352218	7,091844	7,277154	7,48474	7,240405333	-0,24433467	0,844205032	---
17493824	6,793566	6,879319	6,75283	6,40077	6,657909	6,634353	6,8085717	6,564344	-0,24422767	0,844267646	Relt
17474744	4,150582	3,926946	4,139596	3,926176	3,550101	4,008174	4,0723747	3,828150333	-0,24422433	0,844269597	Vmn1r116
17506567	5,540302	5,41162	5,4898	4,978801	5,528792	5,201553	5,480574	5,236382	-0,244192	0,844288518	4933417D19Rik
17551141	5,596359	5,44242	6,673838	5,71141	5,805417	5,463438	5,9042057	5,660088333	-0,24411733	0,844332216	---
17479758	5,38126	5,096067	4,861378	5,007293	4,723493	4,875608	5,1129017	4,868798	-0,24410367	0,844340214	Sh3gl3
17494144	5,129162	4,628385	5,380115	4,904699	4,716008	4,784669	5,0458873	4,801792	-0,24409533	0,844345091	Olfr569
17521178	6,822014	6,829783	7,11474	6,627151	6,739367	6,667794	6,922179	6,678104	-0,244075	0,844356992	Poc1a
17388548	7,380794	7,146099	7,491157	7,143267	7,11004	7,032734	7,33935	7,095347	-0,244003	0,844399132	Gm13889
17204879	6,278709	6,128772	5,808009	5,763459	5,8206	5,899621	6,07183	5,827893333	-0,24393667	0,844437957	---
17548662	5,105906	4,842648	5,072223	4,792087	4,760022	4,736887	5,0069257	4,762998667	-0,243927	0,844443615	Gm5606
17389307	3,602498	3,318629	3,38538	3,219705	3,165339	3,189692	3,4355023	3,191578667	-0,24392367	0,844455566	Olfr1312
17474865	3,847511	3,489899	3,765224	3,418908	3,581303	3,370769	3,700878	3,456993333	-0,24388467	0,844468394	Vmn1r178
17204387	4,11054	4,572283	5,031047	4,472872	4,30111	4,208297	4,57129	4,327426333	-0,24386367	0,844480686	---
17201513	4,401484	4,671439	4,291497	4,496563	4,53549	3,601066	4,4548067	4,211039667	-0,243767	0,844537272	---
17202307	3,640773	5,078637	4,206629	3,805659	4,294728	4,094525	4,3086797	4,064970667	-0,243709	0,844571225	---
17284574	4,409429	4,591291	4,059305	4,200862	4,033037	4,095048	4,3533417	4,109649	-0,24369267	0,844580787	---

17377525	7,75392	7,864716	7,756502	7,47312	7,570022	7,600997	7,7917127	7,548046333	-0,24366633	0,844596203	Gins1
17204647	4,969471	5,292685	4,947551	4,325817	4,721239	5,431682	5,0699023	4,826246	-0,24365633	0,844602057	---
17284321	5,165693	4,597382	4,353364	4,378062	4,417887	4,589612	4,7054797	4,461853667	-0,243626	0,844619816	---
17529930	4,982724	4,68953	5,101786	4,677606	4,820874	4,545005	4,92468	4,681161667	-0,24351833	0,844682851	Clstn2
17369103	6,793218	6,813177	6,822496	6,741541	6,336909	6,619995	6,8096303	6,566148333	-0,243482	0,844704124	Tbc1d13
17276814	6,592497	6,941187	6,53076	6,519588	6,376607	6,437847	6,688148	6,444680667	-0,24346733	0,844712712	Zfp36l1 // Zfp36l1
17267174	4,567327	4,662988	4,744009	4,354948	4,246755	4,642232	4,658108	4,414645	-0,243463	0,844715249	8430403D17Rik
17528255	4,681749	5,366639	5,396165	4,905267	5,078063	4,730994	5,1481843	4,904774667	-0,24340967	0,844746477	Ankdd1a
17258604	5,866302	5,21904	5,246304	5,14402	5,232871	5,224638	5,443882	5,200509667	-0,24337233	0,844768337	Aanat
17235026	6,621979	6,698095	6,591762	6,578784	6,168196	6,434829	6,6372787	6,393936333	-0,24334233	0,844785904	LOC100862144
17449628	9,458562	9,658962	9,228165	9,016756	9,276984	9,322003	9,448563	9,205247667	-0,24331533	0,844801714	---
17201529	6,687603	6,216536	7,344793	7,008352	6,07334	6,437352	6,749644	6,506348	-0,243296	0,844813035	---
17371964	6,931355	6,841049	6,921294	6,610944	6,63494	6,718189	6,8978993	6,654691	-0,24320833	0,844864372	1700011J10Rik
17206207	5,869145	5,366481	6,070675	5,23837	5,630558	5,707834	5,768767	5,525587333	-0,24317967	0,84488116	---
17292256	8,94476	9,208838	9,069496	8,883988	8,783628	8,826073	9,0743647	8,831229667	-0,243135	0,844907319	---
17490038	8,774704	9,087001	8,779125	8,563024	8,61824	8,730174	8,8802767	8,637146	-0,24313067	0,844909856	---
17387979	4,097229	3,693149	3,998669	3,831603	3,314236	3,913912	3,9296823	3,686583667	-0,24309867	0,844928597	Olfr1249
17208847	3,584432	3,920279	3,135597	3,506157	3,340171	3,064833	3,5467693	3,303720333	-0,243049	0,844957686	---
17532135	3,651521	3,587744	3,802204	3,499317	3,324181	3,488863	3,6804897	3,437453667	-0,243036	0,844965299	---
17528388	5,070137	4,962153	5,106417	4,69483	4,893658	4,821136	5,0462357	4,803208	-0,24302767	0,84497018	---
17494170	4,806875	4,102871	3,714612	3,903348	4,049311	3,942627	4,2081193	3,965095333	-0,243024	0,844972328	Olfr603
17246284	8,63129	8,416233	8,27072	7,887477	8,227531	8,474171	8,4394143	8,196393	-0,24302133	0,84497389	Suox
17203485	4,643751	3,981833	4,785642	4,190087	4,230044	4,262185	4,4704087	4,227438667	-0,24297	0,845003956	---
17414729	7,323794	7,828804	7,370075	7,343216	7,124153	7,326482	7,5075577	7,264617	-0,24294067	0,845021137	Orm1
17219682	4,649345	4,273939	4,480224	4,370501	4,203749	4,100611	4,467836	4,224953667	-0,24288233	0,845055305	Olfr430
17203443	5,541054	4,962399	5,65901	5,178035	4,80572	5,450095	5,3874877	5,144616667	-0,242871	0,845061943	---
17206173	4,302487	5,755513	5,670953	5,181564	4,557998	5,260877	5,2429843	5,000146333	-0,242838	0,845081273	---
17543396	4,503541	4,321578	4,391664	4,181828	4,119193	4,187815	4,4055943	4,162945333	-0,242649	0,84519199	Eda2r
17353663	7,478774	8,030306	7,383893	7,442646	7,311338	7,411218	7,630991	7,388400667	-0,24259033	0,84522636	Tmem173
17358258	4,261824	4,28473	4,689333	4,210969	4,237609	4,059827	4,4119623	4,169468333	-0,242494	0,845282801	1700021P04Rik
17232977	5,749429	5,710319	5,735881	5,507185	5,306399	5,654593	5,7318763	5,489392333	-0,242484	0,84528866	Scml4
17431717	4,906146	4,913384	5,129292	4,892679	4,460638	4,86833	4,9829407	4,740549	-0,24239167	0,84534276	---
17202885	3,461674	4,205996	2,859907	3,226925	3,057518	3,516056	3,5091923	3,266833	-0,24235933	0,845361706	---
17410809	8,318769	8,276937	8,406269	8,033699	8,078825	8,162405	8,3339917	8,091643	-0,24234867	0,845367956	Lmo4
17209113	4,770839	5,217896	5,426749	4,788308	5,196361	4,70395	5,1384947	4,896206333	-0,24228833	0,84540331	---
17206655	6,657515	6,60795	6,248727	5,911905	6,440875	6,434672	6,5047307	6,262484	-0,24224667	0,845427727	---
17378168	5,220591	5,462149	5,416785	5,156861	4,881152	5,334878	5,3665083	5,124297	-0,24221133	0,845448433	Bpifb5
17275776	3,804044	3,347386	3,456769	3,228214	3,096022	3,557349	3,5360663	3,293861667	-0,24220467	0,84545234	---
17408123	4,717103	4,76439	4,522052	4,377151	4,450776	4,449352	4,6678483	4,425759667	-0,24208867	0,845520321	Cd160
17473666	3,509542	3,581378	3,577635	3,246056	3,384242	3,312007	3,556185	3,314101667	-0,24208333	0,845523447	Vmn1r67
17399864	6,516791	6,561399	6,423113	6,325509	6,464653	5,985165	6,5004343	6,258442333	-0,241992	0,845576976	Sprr2b
17219382	5,09041	4,971477	5,005918	4,94208	4,773903	4,626087	5,0226017	4,78069	-0,24191167	0,845624062	Cd244
17284551	5,087161	5,194539	5,023397	4,928671	4,616431	5,034277	5,101699	4,859793	-0,241906	0,845627383	---
17370794	4,393378	4,590284	4,446702	4,169556	4,526503	4,008612	4,476788	4,234890333	-0,24189767	0,845632268	---
17467418	6,137733	6,182336	5,673542	6,321989	5,242172	5,704056	5,9978703	5,756072333	-0,241798	0,845690689	---
17209537	3,63631	2,749477	3,554197	2,996663	3,075318	3,142783	3,313328	3,071588	-0,24174	0,845724689	---
17210791	4,299166	3,947618	4,331835	4,16222	3,936696	3,754582	4,192873	3,951166	-0,241707	0,845744034	---
17310816	9,219791	9,030535	8,906495	8,841194	8,593536	8,997002	9,0522737	8,810577333	-0,24169633	0,845750287	Cmbl
17250533	9,340012	9,457722	9,417907	9,270985	8,996297	9,223453	9,4052137	9,163578333	-0,24163533	0,845786048	Map2k3
17476294	6,382498	6,319383	6,347702	5,997634	6,280567	6,046751	6,349861	6,108317333	-0,24154367	0,845839789	---
17508591	9,734029	10,0718	9,693441	9,450788	9,663089	9,660777	9,83309	9,591551333	-0,24153867	0,845842721	Snord13 // Snord13
17209887	7,126781	7,257388	7,293914	6,891237	6,819828	7,242517	7,2260277	6,984527333	-0,24150033	0,845865196	---
17200963	6,886014	6,754688	6,57472	6,449864	5,926245	7,115008	6,738474	6,497039	-0,241435	0,845903502	---
17201649	8,26063	8,060631	7,879129	7,906965	7,816375	7,752831	8,0667967	7,825390333	-0,24140633	0,845920311	---
17344905	4,0729	4,098709	4,775772	4,190382	4,110332	3,922651	4,3157937	4,074455	-0,24133867	0,845959988	Olfr116
17473912	4,080408	3,937403	3,862811	3,579585	3,792636	3,784395	3,9602073	3,718872	-0,24133533	0,845961942	---
17347666	5,652586	5,394253	5,421008	5,288185	5,144322	5,311469	5,4892823	5,247992	-0,24129033	0,84598833	Gm19696
17389788	4,432245	3,912954	4,165305	3,749949	4,043582	3,993189	4,170168	3,928906667	-0,24126133	0,846005335	Phgr1 // Phgr1
17450838	5,238889	5,035961	4,863989	4,811214	4,7585	4,845499	5,0462797	4,805071	-0,24120867	0,84603622	Rnf212 // Rnf212
17208701	6,312639	6,209352	6,266881	6,228449	5,810456	6,026407	6,2629573	6,021770667	-0,24118667	0,846049121	---
17286893	5,02304	4,881452	5,289199	4,716729	4,979839	4,773684	5,0645637	4,823417333	-0,24114633	0,846072775	Rnf182
17379864	8,484985	9,074326	8,856592	8,754092	8,71172	8,22669	8,805301	8,564167333	-0,24113367	0,846080203	1500012F01Rik
17461625	6,260548	6,525397	6,633953	6,250422	6,09616	6,350096	6,4732993	6,232226	-0,24107333	0,846115587	Ogg1
17203433	3,70407	4,414773	3,3615	3,528019	3,672474	3,556877	3,826781	3,58579	-0,240991	0,846163875	---
17200569	5,908914	6,049691	5,166188	5,544082	5,426566	5,431395	5,7082643	5,467347667	-0,24091667	0,846207474	---
17359858	6,163177	6,112389	5,817624	5,866804	5,917383	5,586323	6,0310633	5,79017	-0,24089333	0,84622116	Dpcd
17478386	6,013515	5,515447	5,717122	5,477665	5,477665	5,568091	5,7486947	5,507807	-0,24088767	0,846224484	Slc6a5
17515517	5,429086	5,546071	5,637254	5,364221	5,110362	5,415411	5,5374703	5,296664667	-0,24080567	0,846272583	Cypt4
17495677	7,590037	7,875243	7,723726	7,604322	7,454298	7,408024	7,7296687	7,488881333	-0,24078733	0,846283337	Eri2
17299785	5,374248	4,893493	4,878071	5,189352	4,764946	4,469182	5,048604	4,807826667	-0,24077733	0,846289203	---
17223717	4,168203	4,225373	4,441421	4,111033	3,941743	4,059978	4,2783323	4,037584667	-0,24074767	0,846306606	---
17351424	5,08234	4,750264	4,616491	4,863742	4,414675	4,44847	4,816365	4,575629	-0,240736	0,84631345	---
17201333	3,233192	2,797198	3,080417	2,720601	2,814627	2,853606	3,0369357	2,796278	-0,24065767	0,846359403	---
17536887	4,539853	4,21329	4,902429	4,29157	4,287223	4,354812	4,5518573	4,311201667	-0,24065567	0,846360576	LOC434825
17378993	4,398159	4,436115	4,458193	4,203981	4,088035	4,278486	4,4308223	4,190167333	-0,240655	0,846360968	---
17307260	4,144142	4,493281	4,173673	4,039721	3,934634	4,114852	4,2703653	4,029735667	-0,24062967	0,84637583	Gm6904
17488057	5,537191	5,491481	5,828499	5,541655	5,307053	5,286604	5,619057	5,378437333	-0,24061967	0,846381696	Cyp2s1
17504558	4,427216	3,998843	4,402833	4,128608	4,25127	3,727334	4,2762973	4,035737333	-0,24056	0,846416701	Ces2f
17200235	5,681417	5,863087	6,225498	5,31645	6,050817	5,68113	5,923334	5,682799	-0,240535	0,846431369	---
17204997	7,612864	7,165138	7,291383	7,379691	6,619459	7,348805	7,3564617	7,115985	-0,24047667	0,846465594	---
17209587	5,588578	5,113638	5,039956	4,756816	5,278327	4,985639	5,2473907	5,006927333	-0,24046333	0,846473417	---
17207881	3,392521	3,32192	3,900214	3,504533	3,225382	3,163379	3,5382183	3,297764667	-0,24045367	0,846479089	---
17470829	3,72006	3,839563	3,925822	3,914086	3,243172	3,606835	3,8284817	3,588031	-0,24045067	0,846480849	Rnu7 // Rnu7
17467499	3,103462	3,456612	3,21248	2,994666	3,226278	2,83087	3,257518	3,017271333	-0,24024667	0,846600551	---
17302327	5,032018	5,67232	4,70974	4,898182	4,933366	4,862364	5,138026	4,897970667	-0,24005533	0,846712837	---
17378232	6,636256	6,557925	7,166641	6,321575	6,63666	6,682423	6,7869407	6,546886	-0,24005467	0,846713228	1700003F12Rik
17200261	5,503767	4,283348	4,012809	4,773048	4,165101	4,141699	4,5999747	4,359949333	-0,24002533	0,846730444	---
17481651	4,968285	5,062627	5,296388	4,816047	4,962786	4,828526	5,1091	4,869119667	-0,23998033	0,846756855	Tub
17445629	3,405927	3,566461	3,611678	3,216431	3,387383	3,260617	3,528022	3,288143667	-0,23987833	0,846816724	---
17202341	4,918676	4,847867	5,311197	4,274309	4,989906	5,093917	5,0259133	4,786044	-0,23986933	0,846822007	---
17204115	4,473479	5,399346	4,714139	4,726044	4,722425	4,418902	4,8623213	4,622457	-0,23986433	0,846824941	---
17370341	3,603315	3,344012	3,609007	3,37069	3,441055	3,025075	3,518778	3,27894	-0,239838	0,846840399	Olfr346
17212931	8,112079	7,901722	7,947791	7,712459	7,740228	7,789454	7,9871973	7,747380333	-0,239817	0,846852725	1700066M21Rik
17204627	5,860656	5,055357	5,736406	5,2909	4,763395	5,878673	5,5508063	5,310989333	-0,239817	0,846852725	---
17256924	5,948345	5,73531	5,893302	5,686589	5,764769	5,406207	5,8589857	5,619188333	-0,23979733	0,84686427	Asb16
17319576	6,516386	6,991022	6,710444	6,536404	6,570738	6,391353	6,739284	6,499498333	-0,23978567	0,846871118	Nhp2l1
17234809	4,27142	4,244155	3,971123	4,011662	3,852403	3,903316	4,1622327	3,922460333	-0,23977233	0,846878945	Olfr1358
17308598	5,624802	5,250707	5,534776	5,137106	5,164175	5,389906	5,470095	5,230395667	-0,23969933	0,846921798	Mir687
17345318	7,294045	7,534367	7,864013	7,413902	6,891212	7,668236	7,5641417	7,32445	-0,23969167	0,846926298	---
17551085	4,130404	3,807593	4,484828	4,0827	3,904078	3,717062	4,1409417	3,90128	-0,23966167	0,84694391	---
17431811	4,550979	4,247958	4,715365	4,430262	4,07579	4,289367	4,5047673	4,265139667	-0,23962767	0,84696387	Pla2g2f
17550046	3,578582	3,76884	4,588456	3,475903	4,05874	3,683017	3,978626	3,73922	-0,239406	0,847094014	---
17205051	5,921898	5,448704	5,77719	5,874436	5,725787	4,829412	5,7159307	5,476545	-0,23938567	0,847105953	---
17209241	5,921898	5,448704	5,77719	5,874436	5,725787	4,829412	5,7159307	5,476545	-0,23938567	0,847105953	---
17451734	5,5434	5,807951	5,734432	5,703305	5,600227	5,064295	5,695261	5,455942333	-0,23931867	0,847145294	---
17507908	4,049389	3,418081	3,440274	3,605156	3,370027	3,214611	3,6359147	3,396598	-0,23931667	0,847146469	---
17550524	6,236413	6,576238	6,595509	6,110669	6,308368	6,271379	6,4693867	6,230138667	-0,239248	0,847186791	---
17517175	8,687589	8,852716	8,594901	8,558043	8,398062	8,461576	8,7117353	8,472560333	-0,239175	0,847229659	Ppp2r1b
17280138	5,800159	6,18813	6,011025	5,700263	5,714272	5,867262	5,9997713	5,760599	-0,23917233	0,847231225	2410004P03Rik
17237784	6,108253	6,250646	6,295187	6,107045	6,000885	5,828664	6,2180287	5,978864667	-0,239164	0,847236119	B930054O08
17212514	6,330913	6,5149	6,755525	6,354395	5,933549	6,596016	6,5337793	6,294653333	-0,239126	0,847258435	---
17210549	5,734861	5,155145	5,534184	5,286299	5,133613	5,286991	5,47473	5,235634333	-0,23909567	0,847276249	---
17381772	3,420402	3,143906	3,218205	3,154009	3,077595	2,83411	3,2608377	3,021904667	-0,238933	0,847371787	---
17325329	8,772131	9,176536	8,590961	8,531141	8,807961	8,483904	8,8465427	8,607668667	-0,238874	0,847406441	Gm5483
17530728	4,511835	4,73711	4,688068	4,448236	4,338834	4,433536	4,645671	4,406868667	-0,23880233	0,847448538	Iqcf4 // Iqcf4
17548557	3,327339	2,949765	3,116898	2,921838	2,934441	2,821387	3,131334	2,892555333	-0,23877867	0,84746244	LOC674392 // LOC674392 // LOC674392
17278239	7,588311	8,381355	8,735892	7,958423	7,639414	8,391643	8,235186	7,996493333	-0,23869267	0,847512959	Serpina4-ps1
17471268	5,710816	5,152194	5,221941	5,054424	5,186168	5,128299	5,3616503	5,122963667	-0,23868667	0,847516484	---
17237694	6,250932	6,121902	6,451573	5,975931	6,098383	6,034041	6,2748023	6,036118333	-0,238684	0,84751805	Gm15910
17201023	8,828979	8,788837	8,917846	8,415071	8,717964	8,686627	8,8452207	8,606554	-0,23866667	0,847528233	---
17440022	4,14402	4,191148	4,078695	4,039721	3,834641	3,824135	4,1379543	3,899499	-0,23845533	0,847652392	---
17411101	3,233192	3,200762	3,889484	3,5424	3,046318	3,019465	3,441146	3,202727667	-0,23841833	0,847674132	---
17226655	9,526913	9,990951	9,630364	9,48531	9,443007	9,505203	9,716076	9,47784	-0,238236	0,847781271	C4bp
17536252	3,174359	2,941433	3,110977	2,802273	2,965582	2,744347	3,0755897	2,837400667	-0,238189	0,84780889	---
17305542	3,942567	4,08802	4,306545	3,945549	3,765492	3,911573	4,1123773	3,874204667	-0,23817267	0,847818489	4930503E14Rik
17411761	9,688919	9,615916	9,432699	9,216831	9,311558	9,494908	9,579178	9,341099	-0,238079	0,847873535	Ccne2
17201701	4,893562	5,258871	4,590667	4,533134	4,768326	4,727414	4,9143667	4,676291333	-0,23807533	0,84787569	---
17421491	6,769658	6,953853	7,078559	6,828617	6,61164	6,647593	6,9340233	6,69595	-0,23807333	0,847876865	Gm13152 // Gm13152
17445627	5,65659	5,644493	5,728378	5,937937	4,562415	5,814889	5,676487	5,438413667	-0,23807333	0,847876865	---
17424848	4,973059	5,535654	5,111074	5,200671	4,800168	4,904909	5,2065957	4,968582667	-0,238013	0,847912324	---
17497399	4,548356	4,47258	4,704099	4,31992	4,244141	4,447236	4,5750117	4,337099	-0,23791267	0,847971295	Tcerg1l
17208877	7,310053	6,888681	6,748486	6,398411	6,790453	7,04486	6,9824067	6,744574667	-0,237832	0,84801871	---
17210039	4,257555	4,230696	4,267588	4,109601	3,896756	4,036006	4,2519463	4,014121	-0,23782533	0,848022628	---
17537207	5,909625	5,582738	5,698043	5,231998	5,767066	5,477888	5,7301353	5,492317333	-0,237818	0,848026939	Gm7134

17467278	5,645548	5,868664	5,953412	5,517835	5,715767	5,520824	5,8225413	5,584808667	-0,23773267	0,8480771	Gm7144
17285532	4,925495	6,197801	5,587691	5,713712	4,840683	5,44345	5,570329	5,332615	-0,237714	0,848088073	---
17209809	7,422273	6,942593	6,790328	6,429636	7,362523	6,650318	7,0517313	6,814159	-0,23757233	0,848171356	---
17494435	4,417015	4,262823	3,989031	4,042931	3,955167	3,958228	4,2229563	3,985442	-0,23751433	0,848205455	Gm19934
17455744	4,377454	4,197277	4,218661	4,311204	3,965263	3,804488	4,264464	4,026985	-0,237479	0,848226229	---
17208369	4,729094	5,122001	5,380706	5,065195	4,629047	4,825171	5,077267	4,839804333	-0,23746267	0,848235832	---
17311213	4,902185	5,052496	5,199987	5,109007	4,835312	4,498652	5,051556	4,814323667	-0,23723233	0,848371268	Rims2
17534567	3,524052	3,241707	3,125055	3,130237	3,162363	2,886556	3,296938	3,059718667	-0,23721933	0,848378913	---
17474689	4,01657	3,631702	4,26846	3,566998	3,782753	3,855404	3,972244	3,735051667	-0,23719233	0,84839479	---
17338532	5,660568	5,784219	6,015223	5,509287	5,529533	5,709628	5,8200033	5,582816	-0,23718733	0,84839773	Gm6934
17322507	5,65096	5,828013	5,599942	5,385177	5,385861	5,596543	5,6929717	5,455860333	-0,23711133	0,848442425	Dnase1
17202483	3,492721	3,317802	2,955394	2,991059	3,040118	3,023752	3,2553057	3,018309667	-0,236996	0,848510254	---
17203079	6,762428	6,749076	6,712797	6,152236	6,651656	6,70951	6,7414337	6,504467333	-0,23696633	0,848527703	---
17299250	4,755542	4,605861	4,955696	4,662983	4,500041	4,443277	4,7723663	4,535433667	-0,23693267	0,848547504	Gm15217
17207141	4,087977	4,677905	4,264567	4,419086	3,852339	4,048657	4,343483	4,106694	-0,236789	0,848632009	---
17250742	10,33067	10,42886	10,37512	10,12458	10,20497	10,09474	10,378217	10,14143	-0,23678667	0,848633381	Snord49a
17386725	5,656063	5,735485	5,405339	5,318751	5,209484	5,558328	5,5989623	5,362187667	-0,23677467	0,84864044	Pde11a
17283475	4,16244	4,384936	4,35746	4,052159	4,214712	3,927875	4,301612	4,064915333	-0,23669667	0,848686323	Mir1936
17331217	3,904957	4,105422	4,118444	3,778572	3,682485	3,957787	4,042941	3,806281333	-0,23665967	0,848708089	Olfr172
17507945	3,160802	2,958644	3,658746	3,037029	3,04627	2,984988	3,2593973	3,022762333	-0,236635	0,8487226	Defb11
17402039	6,0033	5,699485	6,339707	5,876993	5,692201	5,763418	6,014164	5,777537333	-0,23662667	0,848727503	Lrrc39
17200701	3,018452	2,821051	2,914063	2,516188	2,642163	2,885363	2,9178553	2,681238	-0,23661733	0,848732993	---
17274045	8,003378	7,866965	7,77171	7,524316	7,715227	7,69283	7,8806843	7,644124333	-0,23656	0,848766723	Rdh14
17232998	5,784931	5,802515	6,045077	5,724657	5,53066	5,667626	5,8775077	5,640981	-0,23652667	0,848786334	9030612E09Rik
17277679	6,999835	6,413394	6,19675	6,853732	5,6568	6,389907	6,5366597	6,300146333	-0,23651333	0,848794179	Gm2022
17438969	9,028925	9,107594	8,952253	8,817107	8,791164	8,771276	9,0295907	8,793182333	-0,23640833	0,848855956	Pf4
17407496	4,199087	3,718719	4,365368	4,053225	3,701152	3,81971	4,0943913	3,858029	-0,23636233	0,848883022	Crct1
17242328	6,051176	5,750145	5,855344	5,759872	5,307054	5,880984	5,885555	5,649303333	-0,23625167	0,848948141	Krtap10-4
17404195	6,250678	6,027661	6,261213	5,884595	6,00451	5,941871	6,1798507	5,943658667	-0,236192	0,848983253	Gm9733
17541719	3,740187	3,652095	3,983654	3,86069	3,264071	3,542794	3,7919787	3,555851667	-0,236127	0,849021504	Mir450-2
17514933	4,488712	4,083563	4,491501	4,209449	4,21649	3,929719	4,354592	4,118552667	-0,23603933	0,849073097	Olfr855
17200829	4,814709	4,056253	4,882981	4,240507	4,403367	4,402215	4,5846477	4,348696333	-0,23595133	0,84912489	---
17465476	3,508655	3,337367	3,4261	2,95342	3,200363	3,410692	3,4240407	3,188158333	-0,23588233	0,849165502	---
17509018	3,551908	3,830821	3,618491	3,596092	3,325496	3,372049	3,6670733	3,431212333	-0,235861	0,849178059	Gm5347
17331254	4,556902	4,632854	4,59202	4,453457	4,196896	4,424003	4,5939253	4,358118667	-0,23580667	0,84921004	Olfr194
17207367	3,444215	3,77491	3,222027	3,506438	3,05764	3,169655	3,480384	3,244577667	-0,23580633	0,849210236	---
17443874	6,450355	6,234136	6,528426	5,987854	6,351646	6,166013	6,4043057	6,168504333	-0,23580133	0,84921318	Got2
17281773	6,978564	7,096709	7,092763	6,679787	6,863456	6,917521	7,056012	6,820254667	-0,23575733	0,84923908	Timm9
17361580	5,536241	5,203073	5,628058	5,187726	5,190294	5,282111	5,4557907	5,220043667	-0,235747	0,849245162	Tsga10ip
17207537	8,088419	7,817382	8,248734	7,889643	7,611429	7,946588	8,0515117	7,815886667	-0,235625	0,849316981	---
17209201	4,891809	4,933513	4,720836	4,296547	4,582367	4,960807	4,8487193	4,613240333	-0,235479	0,849402936	---
17201625	6,087246	5,446037	6,165894	5,604626	5,885558	5,502562	5,8997257	5,664248667	-0,235477	0,849404113	---
17204337	3,136564	3,277887	3,309791	2,999852	3,075669	2,942354	3,241414	3,005958333	-0,23545567	0,849416674	---
17466838	6,038455	5,690135	5,879434	5,547455	5,786818	5,567448	5,8693413	5,633907	-0,23543433	0,849429234	Hoxa6
17349866	4,34954	4,462514	4,371273	4,249125	4,095345	4,132563	4,3944423	4,159011	-0,23543133	0,849431	Pcdhb1
17403043	6,919415	6,925071	7,108589	6,647635	6,77767	6,821572	6,9843583	6,748959	-0,23539933	0,849449842	Rg9mtd2
17203061	5,514448	5,854236	5,02727	4,948092	5,184708	5,557043	5,465318	5,229947667	-0,23537033	0,849466917	---
17201315	5,413243	4,341628	5,07202	5,249517	3,930138	4,941294	4,942297	4,706983	-0,235314	0,849500087	---
17548812	4,915128	4,483563	4,562692	4,402254	4,370233	4,483511	4,6537943	4,418666	-0,23512833	0,84960942	---
17548856	4,915128	4,483563	4,562692	4,402254	4,370233	4,483511	4,6537943	4,418666	-0,23512833	0,84960942	---
17305334	6,623608	6,407801	6,601083	6,418739	6,552169	5,956202	6,544164	6,309036667	-0,23512733	0,849610009	1700091H14Rik
17397988	3,174915	3,005976	3,397564	2,851993	3,089995	2,9311	3,1928183	2,957696	-0,23512233	0,849612953	---
17459900	8,874971	9,233233	8,9107	8,77843	8,615975	8,919188	9,0063013	8,771197667	-0,23510367	0,849623946	---
17491352	3,724842	3,608191	3,350225	3,243733	3,359213	3,37503	3,561086	3,325992	-0,235094	0,849629639	Mrgprb4
17209825	8,168039	7,57298	7,9553	8,030659	7,641089	7,319328	7,898773	7,663692	-0,235081	0,849637295	---
17249980	9,397696	10,87754	9,038003	9,427978	9,536827	9,643391	9,7710797	9,536065333	-0,23501433	0,849676557	Igtp
17504391	4,812451	4,517561	4,849352	4,377588	4,568241	4,528656	4,7264547	4,491495	-0,23495967	0,849708754	---
17427117	7,017	6,744998	6,893935	6,52457	6,790825	6,635722	6,885311	6,650372333	-0,23493867	0,849721123	---
17427686	7,944825	8,245277	8,115758	8,040272	7,770585	7,790195	8,1019533	7,867017333	-0,234936	0,849722693	Slc35d1
17201317	3,595583	3,835203	3,355596	3,507297	3,073171	3,501186	3,5954607	3,360551333	-0,23490933	0,8497384	---
17386328	7,513956	7,65564	7,300782	7,284398	7,153904	7,327459	7,490126	7,255253667	-0,23487233	0,849760193	Ssb // Ssb
17547877	8,694247	8,57667	8,573164	8,591053	8,315478	8,233036	8,6146937	8,379855667	-0,234838	0,849780415	Deptor
17349540	3,933582	4,125184	3,980412	3,522079	3,909527	3,903203	4,0130593	3,778269667	-0,23478967	0,849808885	---
17266851	7,807827	8,198143	7,648814	7,768795	7,57218	7,609459	7,884928	7,650144667	-0,23478333	0,849812616	Slfn9
17550130	4,496302	4,23602	4,533197	4,470176	3,997084	4,093999	4,4218397	4,187086333	-0,23475333	0,849830288	---
17202953	6,248917	6,377985	5,812904	5,539201	5,892856	6,303567	6,146602	5,911874667	-0,23472733	0,849845603	---
17361006	3,923227	3,897973	3,889484	3,744872	3,537371	3,724322	3,9035613	3,668855	-0,23470633	0,849857974	---
17475202	7,244967	7,382452	7,338559	7,077048	7,681977	6,502848	7,3219927	7,087291	-0,23470167	0,849860723	---
17204781	3,591701	3,815353	3,32369	3,189692	3,317316	3,51964	3,5769147	3,342216	-0,23469867	0,84986249	---
17200095	6,234951	5,920172	6,368279	5,987096	5,753212	6,079072	6,1744673	5,939793333	-0,234674	0,849877021	---
17411304	8,366041	8,757753	8,410317	8,211212	8,348006	8,27125	8,5113703	8,276822667	-0,23454767	0,849951446	Rabggtb
17505769	5,001844	4,957366	5,428479	4,905646	4,970635	4,807924	5,1292297	4,894735	-0,23449467	0,849982671	LOC100861890
17209491	5,367668	4,816724	5,659654	5,358822	4,559881	5,221868	5,2813487	5,046857	-0,23449167	0,849984438	---
17259689	4,860378	5,057277	5,38315	4,590462	5,008071	4,999037	5,1002683	4,865856667	-0,23441167	0,850031573	---
17493272	6,654993	6,964989	6,813234	6,552603	6,593399	6,584011	6,811072	6,576671	-0,234401	0,850037857	Picalm // Picalm
17272080	6,003461	5,838305	6,239794	5,711842	6,031992	5,634574	6,0271867	5,792802667	-0,234384	0,850047874	Itgb4
17469455	5,222825	5,033209	5,049459	4,948903	4,875296	4,778248	5,101831	4,867482333	-0,23434867	0,850068693	Prok2
17283084	3,235654	2,879572	3,171745	2,86978	2,952983	2,76122	3,095657	2,861327667	-0,23432933	0,850080085	---
17300127	5,054227	4,57788	4,796234	4,485264	4,793597	4,446901	4,809447	4,575254	-0,234193	0,85016042	A630038E17Rik
17440653	4,61217	4,377899	4,563162	4,403202	4,08261	4,364845	4,5177437	4,283552333	-0,23419133	0,850161402	1700028D13Rik
17261033	3,914812	4,489657	3,873993	3,791001	4,293118	3,491811	4,0928207	3,858643333	-0,23417733	0,850169652	---
17286198	4,507327	4,667704	4,818121	4,776052	4,069017	4,445602	4,664384	4,430223667	-0,23416033	0,85017967	Prl2c1
17531801	4,068411	4,556921	4,162362	4,025271	3,872157	4,187947	4,2625647	4,028458333	-0,23410633	0,850211493	---
17497877	5,168978	4,969283	5,274457	4,453968	5,325356	4,931152	5,1375727	4,903492	-0,23408067	0,850226619	B230206H07Rik
17227606	6,739445	6,708521	6,782349	6,477338	6,414939	6,635946	6,7434383	6,509407667	-0,23403067	0,850256086	2310009B15Rik
17246381	4,609615	4,378986	4,742558	4,469197	4,257059	4,303018	4,577053	4,343091333	-0,23396167	0,850296753	Mettl7b
17347729	8,8237	8,760949	8,544736	8,527338	8,19238	8,708001	8,709795	8,475906333	-0,23388867	0,850339779	Abcg5
17510127	5,81377	6,057995	5,968764	5,644109	5,551617	5,943285	5,946843	5,713003667	-0,23383933	0,850368857	Jund // Jund
17374683	6,697406	6,53733	6,513369	6,480315	6,161729	6,404671	6,5827017	6,348905	-0,23379667	0,850394006	---
17469739	6,718325	6,60631	6,68651	6,52343	6,560492	6,226037	6,6703817	6,436653	-0,23372867	0,85043409	Rpusd3
17201675	2,601993	3,123178	2,939525	2,537088	2,487218	2,939525	2,888232	2,654610333	-0,23362167	0,850497166	---
17411174	6,901435	6,426807	6,410489	6,348843	6,236125	6,452996	6,579577	6,345988	-0,233589	0,850516424	Gipc2
17545574	3,421144	3,274015	3,696756	3,324052	3,232164	3,135256	3,4639717	3,230490667	-0,233481	0,850580096	Magea5
17209523	6,382597	6,865364	6,608119	6,467532	6,113777	6,574442	6,6186933	6,385250333	-0,233443	0,8506025	---
17249861	4,244969	3,815353	3,828214	3,520393	3,897973	3,769888	3,9628453	3,729418	-0,23342733	0,850611737	---
17549770	10,34636	10,44939	10,58967	10,18454	10,29839	10,20223	10,461807	10,22838667	-0,23342	0,850616061	---
17248496	4,641304	4,758657	4,717746	4,414237	4,499858	4,503492	4,7059023	4,472529	-0,23337333	0,850643576	Gm12128
17473635	4,957511	5,078604	4,70615	4,831351	4,793591	4,417281	4,9140883	4,680741	-0,23334733	0,850658906	Olfr1350
17351328	6,881679	6,914801	6,805084	6,622532	6,644949	6,634044	6,867188	6,633841667	-0,23334633	0,850659496	---
17337650	3,10022	3,136549	2,954654	2,880042	2,772912	2,83863	3,0638077	2,830528	-0,23327967	0,850698805	Gm20516
17370691	4,978708	4,589813	4,449388	4,275777	4,454925	4,587368	4,6726363	4,439356667	-0,23327967	0,850698805	Lypd6b
17207951	4,254347	3,975182	3,928353	4,437312	3,559093	3,46174	4,0526273	3,819381667	-0,23324567	0,850718854	---
17203515	4,987189	5,249014	5,679502	5,54859	4,706526	4,960875	5,305235	5,071997	-0,233238	0,850723375	---
17401890	6,455854	6,306512	6,499451	6,63865	6,026161	5,897629	6,4206057	6,18748	-0,23312567	0,850789618	Amy2a5 // Amy2a5
17401893	6,455854	6,306512	6,499451	6,63865	6,026161	5,897629	6,4206057	6,18748	-0,23312567	0,850789618	Amy2a5 // Amy2a5
17401896	6,455854	6,306512	6,499451	6,63865	6,026161	5,897629	6,4206057	6,18748	-0,23312567	0,850789618	Amy2a5 // Amy2a5
17401899	6,455854	6,306512	6,499451	6,63865	6,026161	5,897629	6,4206057	6,18748	-0,23312567	0,850789618	Amy2a5 // Amy2a5
17425276	9,98691	10,06619	10,03414	9,624599	9,892209	9,871075	10,02908	9,795961	-0,233119	0,850793549	Amd1 // Amd1
17298895	6,025012	5,977435	6,479147	6,063562	5,484224	6,234684	6,1605313	5,92749	-0,23304133	0,850839353	Mir346
17291651	3,737972	3,126438	3,365622	3,097055	3,229846	3,204171	3,4100107	3,177024	-0,23298667	0,850871593	---
17200985	7,361168	7,207276	7,450657	6,346942	7,75059	7,222663	7,3397003	7,106731667	-0,23296867	0,850882209	---
17548612	6,681028	5,924829	6,465476	6,242789	6,058174	6,071473	6,357111	6,124145333	-0,23296567	0,850883979	Gm7710
17467531	3,80278	4,520707	4,098231	4,046291	3,6055	4,071032	4,1405727	3,907607667	-0,232965	0,850884372	---
17206449	4,274301	5,449815	4,669092	4,464497	4,670279	4,559622	4,797736	4,564799333	-0,23293667	0,850901083	---
17414966	4,330268	3,918448	4,101308	3,821136	3,977275	3,85296	4,1166747	3,883790333	-0,23288433	0,850931949	B230208B08Rik
17203427	6,152531	5,276765	4,658056	5,371118	5,135257	4,882334	5,3624507	5,129569667	-0,232881	0,850933915	---
17465164	3,912996	3,979872	4,08986	3,849603	3,620139	3,814367	3,9942427	3,761369667	-0,232873	0,850938634	Rnf148
17205329	3,076368	3,309126	3,401449	2,694316	2,840939	3,553077	3,2623143	3,029444	-0,23287033	0,850940207	---
17333986	6,389078	6,779707	6,054502	6,062334	6,211027	6,251318	6,4077623	6,174893	-0,23286933	0,850940797	Zfp943
17280296	4,530114	4,696278	4,475135	4,531009	3,92864	4,543302	4,5671757	4,334317	-0,23285867	0,850947088	Cys1
17550721	4,926727	4,459721	4,989352	4,50914	4,916491	4,251709	4,7919333	4,559113333	-0,23282	0,850969895	---
17291854	7,277241	7,377294	6,993139	7,117864	6,860003	6,971504	7,2158913	6,983123667	-0,23276767	0,851000765	Rpp40
17425401	6,268556	6,326782	6,650948	6,243613	5,846197	6,45833	6,4154287	6,182713333	-0,23271533	0,851031635	Klf4
17202593	4,94423	5,36541	5,451189	5,444702	4,790835	4,827296	5,2536097	5,020944333	-0,23266533	0,85106113	---
17345862	5,882346	5,645377	5,600907	5,450165	5,338171	5,642299	5,7095433	5,476878333	-0,232665	0,851061327	---
17409960	4,241165	3,921641	4,129259	3,8179	3,826332	3,949863	4,097355	3,864698333	-0,23265667	0,851066243	Gm4610
17548088	3,694432	3,716447	3,664943	3,453524	3,586291	3,338086	3,6919407	3,459300333	-0,23264033	0,851075878	Seh1l // Seh1l
17210247	3,348138	4,159914	4,138418	3,159277	3,592175	4,19722	3,8821567	3,649557333	-0,23259933	0,851100065	---
17366922	7,701011	7,399063	7,477243	7,392404	6,951015	7,536124	7,5257723	7,293181	-0,23259133	0,851104785	Mir669m-1 // Mir669m-1
17391332	5,333464	5,476149	6,726196	5,717009	5,53808	5,583021	5,8452697	5,612703333	-0,23256633	0,851119533	Mal
17208837	3,553077	4,077024	4,580497	3,810213	3,914659	3,788274	4,0701993	3,837715333	-0,232484	0,851168107	---
17546047	6,635309	6,471269	6,564212	6,321593	6,242945	6,40887	6,55693	6,324469333	-0,23246067	0,851181874	Tceanc
17470592	5,501622	5,976578	5,953614	5,539903	5,485547	5,709189	5,8106047	5,578213	-0,23239167	0,851222584	Gdf3
17429562	6,834421	6,955906	6,556295	6,107399	6,834421	6,707659	6,7822073	6,549826333	-0,232381	0,851228878	---

17529928	3,257552	3,320907	3,399925	3,203664	3,079527	2,998278	3,326128	3,093823	-0,232305	0,851273721 ---
17325371	3,164694	3,095245	3,407759	3,064671	3,027545	2,878581	3,222566	2,990265667	-0,23230033	0,851276475 ---
17352415	4,557214	4,703879	4,646078	4,399549	4,465716	4,345103	4,6357237	4,403481	-0,23224267	0,851310502 ---
17442751	4,902563	4,984832	5,093394	4,836358	4,712093	4,73562	4,9935963	4,761357	-0,23223933	0,851312469 4933438B17Rik
17202497	5,942023	6,242813	6,814929	6,119015	6,024295	6,159751	6,333255	6,101020333	-0,23223467	0,851315223 ---
17204587	5,355229	5,982563	5,67638	4,478018	5,853251	5,986222	5,6713907	5,439163667	-0,232227	0,851319747 ---
17535767	7,527876	7,863766	7,589324	7,457942	7,603634	7,222742	7,660322	7,428106	-0,232216	0,851326238 Rpl10
17293370	4,208131	3,652197	4,386097	3,781075	3,924714	3,844078	4,0821417	3,849955667	-0,232186	0,851343941 Tpbpa
17396047	4,653153	4,61159	4,361127	4,515853	4,369709	4,04385	4,5419567	4,309804	-0,23215267	0,851363611 Zfp704
17401530	6,577137	6,577457	6,401978	6,34313	6,237103	6,279896	6,5188573	6,286709667	-0,23214767	0,851366562 AI504432
17209863	6,423729	4,555941	4,549095	4,943341	5,164009	4,725449	5,176255	4,944266333	-0,23198867	0,851460397 ---
17221980	3,869367	3,706212	4,257979	3,716725	3,714746	3,706212	3,9445193	3,712561	-0,23195833	0,851478299 Lgsn
17398651	7,269605	7,450016	7,200252	7,141597	6,863633	7,218806	7,3066243	7,074678667	-0,23194567	0,851485775 ---
17391486	7,886846	7,961093	7,249715	6,938855	7,83724	7,625868	7,699218	7,467321	-0,231897	0,851514499 ---
17263448	4,922757	4,91239	5,035227	4,717006	4,575298	4,882392	4,9567913	4,724898667	-0,23189267	0,851517056 1700007J10Rik
17339934	6,803337	6,941648	6,750996	6,677552	6,174766	6,948091	6,8319937	6,600136333	-0,23185733	0,851537911 Morn2
17345404	8,880273	9,63383	9,307695	9,123708	8,872175	9,130351	9,2739327	9,042078	-0,23185467	0,851539485 Gm5093
17458573	8,487724	8,71982	8,33085	8,217957	8,37788	8,246997	8,512798	8,280944667	-0,23185333	0,851540272 Snx10
17285777	4,961028	5,177113	5,785156	4,689375	5,161513	5,376869	5,3077657	5,075919	-0,23184667	0,851544207 ---
17456460	3,705186	3,990816	3,620393	3,605219	3,54974	3,466023	3,7721317	3,540327333	-0,23180433	0,851569195 Hyal5
17484061	5,062213	4,839415	4,928716	4,725814	4,704516	4,704641	4,943448	4,711657	-0,231791	0,851577065 Gm10584
17534589	3,214071	3,137157	3,158852	2,779313	3,018666	3,01676	3,1700267	2,938246333	-0,23178033	0,851583361 ---
17262589	7,361886	7,35066	7,379666	7,042258	7,252164	7,102541	7,3640707	7,132321	-0,23174967	0,851601463 Ppp2ca // Ppp2ca
17288856	3,906637	3,772834	4,16992	3,746532	3,690194	3,71748	3,949797	3,718068667	-0,23172833	0,851614056 3110006O06Rik // 3110006O06Rik
17377851	4,927124	4,795125	5,233922	4,74416	4,644702	4,872262	4,9853903	4,753708	-0,23168233	0,85164121 Xkr7
17289928	9,427914	9,641355	9,586001	9,345223	9,344466	9,270865	9,5517567	9,320184667	-0,231572	0,851706343 Ppap2a
17208533	7,269053	6,371771	7,102567	6,801913	6,324494	6,922294	6,9144637	6,682900333	-0,23156333	0,85171146 ---
17260474	9,049075	9,016159	8,832497	8,855318	8,686112	8,661613	8,9659103	8,734347667	-0,23156267	0,851711853 Igfbp3
17299540	5,703629	5,381838	5,370961	5,323445	5,001491	5,436862	5,485476	5,253932667	-0,23154333	0,851723267 ---
17368367	3,789683	3,494549	3,490715	3,196149	3,398439	3,485913	3,591649	3,360167	-0,231482	0,851759477 Gm2240
17548158	4,733232	4,390616	4,515395	4,415615	4,468926	4,060353	4,5464143	4,314964667	-0,23144967	0,851778567 C130023O10Rik
17402744	4,23226	4,363187	4,257873	3,97492	4,000162	4,183955	4,28444	4,053012333	-0,23142767	0,851791556 ---
17210347	4,762059	4,006747	4,711343	4,327509	3,699496	4,758905	4,493383	4,26197	-0,231413	0,851800216 ---
17482678	5,982683	5,691957	5,983172	5,709742	5,718783	5,535072	5,8859373	5,654532333	-0,231405	0,851804939 ---
17239182	7,237114	7,377918	7,375381	6,940212	7,132115	7,223952	7,3301377	7,098759667	-0,231378	0,851820881 ---
17327191	5,802319	5,446765	5,903858	5,480093	5,511014	5,467877	5,7176473	5,486328	-0,23131933	0,85185552 ---
17203189	5,384834	4,986717	4,719485	4,685061	4,646996	5,065156	5,0303453	4,799071	-0,23127433	0,851882091 ---
17229433	10,12494	10,23759	10,21005	9,967653	9,8507	10,06093	10,19086	9,959761	-0,231099	0,851985628 Nuf2
17208815	5,715323	4,657808	4,255931	4,483939	4,794018	4,657808	4,876354	4,645255	-0,231099	0,851985628 ---
17293386	3,85648	3,673569	4,030237	3,548273	3,632911	3,685863	3,8534287	3,622349	-0,23107967	0,851997046 Ctsq
17525712	4,316555	3,783142	4,15259	3,655545	4,034609	3,869058	4,0840957	3,853070667	-0,231025	0,85202933 Olfr937
17269149	4,313673	4,490983	4,517889	4,041857	4,334437	4,253211	4,4408483	4,209835	-0,23101333	0,852036221 Krtap9-3
17207159	5,219432	5,450266	4,98285	5,694323	4,770577	4,49463	5,217516	4,98651	-0,231006	0,852040552 ---
17291620	5,897724	5,256123	5,658804	5,705925	5,254736	5,15906	5,604217	5,373240333	-0,23097667	0,852057876 ---
17271001	5,287153	5,368751	5,229979	4,686017	5,143119	5,364009	5,2952943	5,064381667	-0,23091267	0,852095675 ---
17357908	4,096635	3,886993	3,572017	3,650754	3,565395	3,647033	3,8518817	3,621060667	-0,230821	0,852149818 Olfr1462
17537220	3,680728	3,42293	3,398775	3,222422	3,373277	3,214334	3,500811	3,270011	-0,2308	0,852162222 Hdx // Hdx
17353370	9,309606	9,262539	9,34898	9,131301	9,04321	9,054231	9,3070417	9,076247333	-0,23079433	0,852165569 D0H4S114
17439029	5,462628	5,300552	5,527353	5,291992	5,22168	5,084481	5,4301777	5,199384333	-0,23079333	0,852166159 Areg
17372742	9,15945	8,110344	8,459799	8,717649	8,222658	8,096956	8,576531	8,345754333	-0,23077667	0,852176004 ---
17315763	5,188631	5,19523	5,082076	4,811668	5,133029	4,828945	5,1553123	4,924547333	-0,230765	0,852182895 Egflam
17236892	4,652314	4,981543	4,370052	4,58401	4,26734	4,46032	4,6679697	4,437223333	-0,23074633	0,852193922 ---
17499739	5,381755	5,2774	4,941087	4,652043	5,493601	4,762463	5,2000807	4,969369	-0,23071167	0,852214399 Defa22
17203267	4,251544	4,326756	4,407565	4,064926	4,1802	4,048815	4,3286217	4,097980333	-0,23064133	0,852255947 ---
17200609	4,082957	4,198265	4,284527	3,776428	4,589379	3,508131	4,188583	3,957979333	-0,23060367	0,852278199 ---
17548888	7,865158	7,848143	8,194093	7,726916	7,704161	7,784576	7,9691313	7,738551	-0,23058033	0,852291983 ---
17200819	2,97489	2,853286	3,532758	3,015634	2,891199	2,762698	3,1203113	2,889843667	-0,23046767	0,852358545 ---
17394114	5,613053	5,401936	5,432451	5,350516	5,061344	5,344225	5,48248	5,252028333	-0,23045167	0,852367998 Wfdc12
17202755	5,067676	5,603624	5,149384	5,214114	4,902611	5,01264	5,2735613	5,043121667	-0,23043967	0,852375088 ---
17420561	6,068029	6,601713	6,235336	6,093725	5,921983	6,198162	6,3016927	6,07129	-0,23040267	0,852396948 Tmco4
17342740	7,030223	6,965531	6,850156	6,471366	6,987671	6,695814	6,9486367	6,718283667	-0,230353	0,852426294 AI413582
17212211	7,064888	7,156662	7,032048	6,859383	6,823267	6,88005	7,0845327	6,854233333	-0,23029933	0,852458004 Il1rl1
17255495	5,528498	5,712646	5,834357	5,37266	5,455899	5,556301	5,6918337	5,46162	-0,23021367	0,852508624 ---
17298823	8,653172	8,77538	8,885637	8,599856	8,442576	8,58112	8,7713963	8,541184	-0,23021233	0,852509412 Gm3219
17218565	5,931329	6,206049	6,307159	5,963263	5,839519	5,951276	6,148179	5,918019333	-0,23015967	0,852540534 Scarna3a
17366910	3,670556	3,868619	3,398734	3,405028	3,249518	3,59308	3,6459697	3,415875333	-0,23009433	0,852579142 ---
17246402	4,397355	4,048156	4,357955	3,943021	4,001341	4,168962	4,267822	4,037774667	-0,23004733	0,852606918 Olfr770
17202339	4,46677	3,804598	4,538875	3,969384	4,496336	3,654613	4,270081	4,040111	-0,22997	0,852652622 ---
17305980	6,632089	6,767951	6,550059	6,696072	6,408187	6,155987	6,650033	6,420082	-0,229951	0,852663851 Ccnb1ip1
17549288	3,433534	3,21773	3,404076	3,208277	3,14854	3,008714	3,35178	3,121843667	-0,22993633	0,85267252 ---
17547591	6,628211	6,028753	6,338434	6,139568	6,020213	6,145884	6,3317993	6,101888333	-0,229911	0,852687493 Kansl1 // Kansl1
17284599	3,554013	3,551335	3,568092	3,46698	3,207062	3,309705	3,5578133	3,327915667	-0,22989767	0,852695373 ---
17430004	3,672617	4,011465	4,348565	4,007362	3,846023	3,489617	4,0108823	3,781000667	-0,22988167	0,85270483 ---
17408821	4,74406	4,608003	4,945165	4,433153	4,634363	4,540151	4,7657427	4,535889	-0,22985367	0,852721379 1700095B22Rik
17370592	3,574038	3,362052	3,513931	3,300744	3,307661	3,152382	3,4833403	3,253595667	-0,22974467	0,852785807 ---
17229255	5,049314	4,827841	4,905242	4,939956	4,473008	4,617011	4,9063827	4,676658333	-0,22972433	0,852797827 ---
17325734	3,391997	3,205643	3,475328	3,118356	3,234125	3,031374	3,357656	3,127951667	-0,22970433	0,852809649 ---
17209139	6,49801	5,875776	5,834197	5,838396	5,730423	5,95008	6,0693277	5,839633	-0,22969467	0,852815363 ---
17207227	4,795128	5,148775	4,900741	4,367508	5,284379	4,503798	4,9482147	4,718561667	-0,229653	0,852839994 ---
17206869	4,517476	3,724976	4,01908	3,657472	3,653668	4,261608	4,0871773	3,857582667	-0,22959467	0,852874478 ---
17362369	5,785373	5,647693	5,982496	5,50616	5,412987	5,807698	5,8051873	5,575615	-0,22957233	0,852887681 Slc22a26
17400486	6,309507	6,476744	6,200356	6,230956	5,887199	6,180013	6,328869	6,099389333	-0,22947967	0,852942465 Mtmr11
17471073	4,909373	4,723915	5,020625	4,440274	4,826696	4,6988	4,8846377	4,655256667	-0,229381	0,8530008 Cd27
17404230	5,637597	5,677276	5,677563	5,583035	5,271069	5,450417	5,6641453	5,434840333	-0,229305	0,853045737 Gm5150
17200777	5,367772	6,179344	6,526035	5,545222	6,170619	5,669403	6,0243837	5,795081333	-0,22930233	0,853047314 ---
17203545	9,450855	8,673324	9,129256	8,790726	8,654283	9,120618	9,0844783	8,855209	-0,22926933	0,853066826 ---
17457056	5,141912	4,540269	5,305835	4,713437	4,872669	4,714394	4,9960053	4,766833333	-0,229172	0,853124381 B230378P21Rik
17550548	10,56292	11,09338	10,83553	10,64517	10,61806	10,54128	10,83061	10,60150333	-0,22910667	0,853163017 ---
17238642	3,997832	3,694432	3,863158	3,634662	3,632288	3,601159	3,8518073	3,622703	-0,22910433	0,853164396 Olfr763
17352330	7,206549	6,919191	6,724067	6,625092	6,738204	6,799294	6,9499357	6,720863333	-0,22907233	0,85318332 Cd226
17478256	4,852522	5,254592	4,842739	4,708925	4,706918	4,846912	4,9832843	4,754251667	-0,22903267	0,853206779 Gm9999
17454500	6,675159	7,231787	7,589112	7,367948	6,628965	6,812193	7,1653527	6,936368667	-0,228984	0,853235561 Tmem184a
17373721	7,388151	7,621824	7,360027	7,300915	7,033906	7,348277	7,4566673	7,227699333	-0,228968	0,853245023 Apip
17216165	3,258996	3,905092	3,184677	3,207742	3,405366	3,048784	3,4495883	3,220630667	-0,22895767	0,853251135 ---
17288344	4,688148	4,011986	4,011118	3,621618	4,37278	4,030025	4,237084	4,008141	-0,228943	0,853259809 Zfp273
17373855	3,412427	3,704773	3,740769	3,418641	3,465952	3,286685	3,619323	3,390426	-0,228897	0,853287016 ---
17320666	6,363671	6,006285	6,566124	6,072261	6,308223	5,86907	6,3120267	6,083184667	-0,228842	0,853319546 Slc2a13
17289329	5,918204	5,389927	5,806977	5,423391	5,245308	5,759891	5,705036	5,476196667	-0,22883933	0,853321123 Fam169a
17233767	4,103772	4,436691	4,210738	4,113511	3,594226	4,357047	4,2504003	4,021594667	-0,22880567	0,853341037 Mir5108
17335842	7,191505	7,071542	6,598106	6,420732	6,795169	6,959066	6,9537177	6,724989	-0,22872867	0,853386583 Pde9a
17358271	10,50917	10,88532	10,53141	10,1419	10,37138	10,72659	10,641967	10,41329	-0,22867667	0,853417343 Cycs
17321951	3,996043	3,730739	4,112451	3,741353	3,490128	3,921745	3,946411	3,717742	-0,228669	0,853421878 Krt6a
17200473	5,079444	5,732654	4,227798	4,922423	5,025336	4,406494	5,0132987	4,784751	-0,22854767	0,853493655 ---
17237510	5,869243	6,029163	5,739804	5,570948	5,786188	5,595758	5,8794033	5,650964667	-0,22843867	0,853558142 4933412E12Rik
17427162	4,883944	4,70893	4,838143	4,42178	4,862743	4,461246	4,810339	4,581923	-0,228416	0,853571552 ---
17436872	5,233346	5,103491	5,278294	5,001773	4,898596	5,029714	5,2050437	4,976694333	-0,22834933	0,853610996 Sh3tc1
17502049	6,551971	6,772058	6,747215	6,260073	6,688481	6,437656	6,6904147	6,46207	-0,22834467	0,853613758 2010320M18Rik
17221284	5,162054	4,886014	4,990992	4,822382	4,794168	4,737624	5,01302	4,784724667	-0,22829533	0,853642948 ---
17444494	5,469224	5,141301	5,072201	4,810242	5,585775	4,601945	5,2275753	4,999320667	-0,22825467	0,853667011 Gm15708
17208265	6,994102	6,267499	6,728879	6,51039	6,410558	6,385138	6,6634933	6,435362	-0,22813133	0,853739992 ---
17429277	3,673522	3,453524	3,50379	3,357935	3,275247	3,313287	3,543612	3,315489667	-0,22812233	0,853745318 Olfr1338
17205819	6,872033	6,305827	6,759689	6,725423	5,432458	7,095304	6,6458497	6,417728333	-0,22812133	0,85374591 ---
17443106	3,153102	3,494078	3,801958	3,139356	3,496907	3,128692	3,483046	3,254985	-0,228061	0,853781614 ---
17448821	5,767362	6,138916	6,036558	5,837747	5,65844	5,762642	5,9809453	5,752943	-0,22800233	0,853816333 Ociad2
17283855	4,711657	4,929637	4,916517	4,619422	4,592348	4,662168	4,8526037	4,624646	-0,22795767	0,853842769 ---
17321578	6,150891	6,479677	6,452045	6,21651	6,176783	6,005581	6,360871	6,132958	-0,227913	0,853869204 Bcdin3d
17496698	6,90459	6,457348	6,675282	6,250772	6,536572	6,566324	6,6790733	6,451222667	-0,22785067	0,853906098 Zfp688
17466055	4,472969	4,827841	4,533739	4,476187	4,310479	4,364576	4,6115163	4,383747333	-0,227769	0,853954436 1700025N23Rik
17469244	6,896514	6,594915	6,606994	6,490941	6,561804	6,362494	6,6994743	6,471746333	-0,227728	0,853978705 ---
17424243	7,050935	6,657298	6,494459	6,127444	6,579159	6,813157	6,7342307	6,506586667	-0,227644	0,854028429 AI464131
17400334	4,178727	3,957767	4,063515	3,846554	3,345629	4,324895	4,0666697	3,839026	-0,22764367	0,854028626 Gm4349
17550244	5,164009	6,173804	5,403666	5,310339	5,58268	5,165584	5,580493	5,352867667	-0,22762533	0,854039479 ---
17358737	3,793144	3,735646	3,398148	3,491746	3,404516	3,347891	3,6423127	3,414717667	-0,227595	0,854057436 Lipk
17208387	5,240652	5,414211	5,333368	5,092599	4,716875	5,496091	5,3294103	5,101855	-0,22755533	0,854080918 ---
17473443	5,855187	5,690375	5,754146	5,592604	5,455884	5,56875	5,7665693	5,539079333	-0,22749	0,854119597 Zfp865
17308731	10,04403	10,29441	10,3223	9,958167	10,0417	9,978485	10,220247	9,992784	-0,22746267	0,854135779 Tpt1 // Tpt1
17210673	3,440851	3,000852	2,966452	2,779487	3,094266	2,852332	3,1360517	2,908695	-0,22735667	0,854198538 ---
17284577	7,069125	6,802738	6,007766	6,256648	6,458295	6,48266	6,626543	6,399201	-0,227342	0,854207222 Ighv1-53 // Ighv1-53
17354428	3,960772	3,895997	3,806421	3,655088	3,540948	3,785237	3,88773	3,660424333	-0,22730567	0,854228735 ---
17358043	7,080328	7,319652	7,210551	6,934903	7,134081	6,859695	7,2035103	6,976226333	-0,227284	0,854241564 D030056L22Rik
17248076	8,982105	8,883728	8,68424	8,866831	7,872552	9,12891	8,8500243	8,622764333	-0,22726	0,854255775 ---
17455550	3,131431	2,943245	3,362455	2,804961	3,169432	2,781121	3,1457103	2,918504667	-0,22720567	0,854287947 ---
17396053	4,548701	4,234055	4,752591	4,361712	4,246259	4,245769	4,5117823	4,28458	-0,22720233	0,854289921 ---
17309832	6,514496	6,600867	6,147667	5,944361	6,346176	6,290936	6,42101	6,193824333	-0,22718567	0,85429979 C6 // C6

17257852	7,28691	7,180012	7,330009	7,035132	7,093704	6,986891	7,2656437	7,038575667	-0,227068	0,85436947	Arsg
17456051	5,186376	4,899323	5,088541	4,937404	4,801136	4,754683	5,05808	4,831074333	-0,22700567	0,854406385	---
17231850	5,394048	5,033523	5,142302	4,937862	5,058216	4,892885	5,1899577	4,962987667	-0,22697	0,854427508	Gm20139
17381937	4,901605	4,784784	4,783222	4,495048	4,864636	4,429316	4,8232037	4,596333333	-0,22687033	0,854486537	---
17270048	6,64241	6,416239	6,368723	6,232989	6,15646	6,357505	6,4757907	6,248984667	-0,226806	0,854524642	Ppy
17231135	6,228185	6,003169	6,017572	5,964041	5,860535	5,744059	6,0829753	5,856211667	-0,22676367	0,854549716	Hhat
17398779	6,401625	6,565117	6,451573	6,064012	6,307182	6,366907	6,4727717	6,246033667	-0,226738	0,85456492	Etv3l
17229162	6,464683	6,531572	6,736908	6,3793	6,343908	6,329764	6,577721	6,350990667	-0,22673033	0,854569461	Gm16548
17366722	3,900884	3,184777	3,470272	3,505522	3,24358	3,126785	3,5186443	3,291962333	-0,226682	0,854598091	Mir669e // Mir669e
17549250	5,573061	5,465089	5,307575	5,028727	5,162735	5,474318	5,448575	5,221926667	-0,22664833	0,854618034	---
17549324	5,573061	5,465089	5,307575	5,028727	5,162735	5,474318	5,448575	5,221926667	-0,22664833	0,854618034	---
17209497	3,146729	3,208287	3,528906	3,122218	3,017634	3,06422	3,2946407	3,068024	-0,22661667	0,854636793	---
17391490	5,934265	6,139029	6,070387	5,7441	5,885954	5,833816	6,0478937	5,82129	-0,22660367	0,854644494	Zc3h8
17501122	4,540089	4,089337	4,386341	4,189358	4,220752	3,925994	4,338589	4,112034667	-0,22655433	0,85467372	---
17388599	9,059806	9,207212	9,046811	8,841862	8,963983	8,828401	9,1046097	8,878082	-0,22652767	0,854689517	Ttc17
17471840	6,679787	6,989743	6,7594	6,328875	6,719697	6,700972	6,8096433	6,583181333	-0,226462	0,854728421	Magohb
17204313	6,524314	6,472839	6,715	6,769817	6,248733	6,01441	6,5707177	6,34432	-0,22639767	0,854766536	---
17213179	7,061981	7,322752	6,962126	6,891794	6,868081	6,908016	7,1156197	6,889297	-0,22632267	0,854810973	Ndufb3
17369787	3,050789	3,084763	3,268621	2,7768	2,90765	3,040817	3,1347243	2,908422333	-0,226302	0,854823219	Mir199b
17208759	5,452255	5,066794	5,331056	4,610457	5,265434	5,295356	5,2833683	5,057082333	-0,226286	0,854832699	---
17288112	6,366714	5,999117	5,893764	5,554735	6,015222	6,01079	6,0865317	5,860249	-0,22628267	0,854834674	Mir24-1
17317167	7,408172	7,250621	7,106016	7,196237	6,76912	7,120659	7,2549363	7,028672	-0,22626433	0,854845537	Has2
17206149	4,076418	4,001651	3,693479	3,824946	3,559058	3,709117	3,9238493	3,697707	-0,22614233	0,854917829	---
17424762	5,949862	6,142623	6,024247	6,013518	5,685642	5,739239	6,0389107	5,812799667	-0,226111	0,854936397	Rgp1 // Rgp1
17538841	9,352459	9,586881	9,90542	9,869114	8,352508	9,944988	9,61492	9,38887	-0,22605	0,854972546	---
17240089	6,085672	6,095247	6,243894	5,93248	6,012086	5,80217	6,1416043	5,915578667	-0,22602567	0,854986967	Tpd52l1
17478201	9,318561	9,390205	9,223239	8,997932	9,079511	9,176716	9,3106683	9,084719667	-0,22594867	0,855032601	Gtf2h1
17478793	4,961435	4,881839	5,026909	4,555039	4,821184	4,816339	4,9567277	4,730854	-0,22587367	0,855077052	Magel2
17201735	7,850682	7,2048	7,769292	7,505649	7,294629	7,347039	7,608258	7,382439	-0,225819	0,855109453	---
17201911	7,850682	7,2048	7,769292	7,505649	7,294629	7,347039	7,608258	7,382439	-0,225819	0,855109453	---
17548790	4,788789	5,179266	5,089255	4,654041	4,812123	4,913724	5,0191033	4,793296	-0,22580733	0,855116368	LOC100861683
17548793	4,788789	5,179266	5,089255	4,654041	4,812123	4,913724	5,0191033	4,793296	-0,22580733	0,855116368	LOC100861683
17548799	4,788789	5,179266	5,089255	4,654041	4,812123	4,913724	5,0191033	4,793296	-0,22580733	0,855116368	LOC100861683
17215372	9,96345	10,14486	9,945959	9,91627	9,825126	9,635455	10,01809	9,792283667	-0,225806	0,855117158	Scarna6
17519394	6,636647	6,832238	6,387392	6,318285	6,353645	6,506984	6,618759	6,392971333	-0,22578767	0,855128025	Myo5a
17234845	4,250055	4,240047	4,646212	3,991865	4,214331	4,25279	4,3787713	4,152995333	-0,225776	0,85513494	Olfr1355
17453771	6,075605	5,162359	6,032208	6,001332	5,166875	5,42466	5,756724	5,530955667	-0,22576833	0,855139484	Mir721
17341365	5,596327	5,55019	5,612951	5,286052	5,504053	5,292225	5,5864893	5,360776667	-0,22571267	0,855172481	Zfp820 // Zfp820
17387852	3,683499	3,456966	3,681852	3,403427	3,204275	3,537482	3,607439	3,381728	-0,225711	0,855173469	---
17200967	7,883653	8,397782	8,400614	7,349587	8,262352	8,393028	8,2273497	8,001655667	-0,225694	0,855183546	---
17376889	4,949017	4,447143	4,710006	4,588513	4,500896	4,339714	4,7020553	4,476374333	-0,225681	0,855191252	Mir1952
17381200	7,57661	7,720325	7,528299	7,19179	7,391731	7,564723	7,6084113	7,382748	-0,22566333	0,855201724	Rpp38
17408897	9,850816	10,36661	10,17817	9,928797	10,01572	9,774242	10,131865	9,906253	-0,22561233	0,855231956	Chi3l3
17351902	3,968287	3,645678	3,811416	3,526926	3,553454	3,668512	3,8084603	3,582964	-0,22549633	0,855300724	Skor2
17424904	5,764575	4,581336	5,149345	4,988422	4,90502	4,9255	5,1650853	4,939647333	-0,225438	0,855335308	---
17387802	3,721571	3,897973	3,728887	3,684813	3,094266	3,893121	3,7828103	3,5574	-0,22541033	0,855351711	Olfr1163
17239354	6,958365	7,019592	7,117173	6,682872	6,983989	6,752059	7,03171	6,806306667	-0,22540333	0,855355861	Stx11
17204075	5,366644	4,054958	4,31651	4,532253	4,536633	3,993241	4,5793707	4,354042333	-0,22532833	0,855400329	---
17241614	4,095576	4,440171	4,627339	4,022785	4,381985	4,082606	4,3876953	4,162458667	-0,22523667	0,855454681	1700023F02Rik // 1700023F02Rik
17347978	3,811601	3,718248	3,806501	3,599575	3,536682	3,52447	3,7787833	3,553575667	-0,22520767	0,855471877	---
17477031	5,557097	4,778487	5,079364	5,077769	4,639882	5,021841	5,138316	4,913164	-0,225152	0,855504886	Gm9268
17219895	3,577787	3,805283	3,139585	3,159837	3,398091	3,289371	3,5075517	3,282433	-0,22511867	0,855524653	---
17370594	3,355747	3,432161	3,266589	3,004649	3,279974	3,094759	3,351499	3,126460667	-0,22503833	0,855572292	---
17203393	4,919363	4,795733	5,913642	4,632559	5,285893	5,035234	5,2095793	4,984562	-0,22501733	0,855584746	---
17384983	3,105625	3,169335	3,379369	2,964063	3,138828	2,876459	3,2181097	2,993116667	-0,224993	0,855599177	---
17201423	3,435401	3,32388	3,590138	3,269264	3,271292	3,134516	3,4498063	3,225024	-0,22478233	0,855724123	---
17301256	5,581311	5,595376	5,784609	5,341074	5,660812	5,285103	5,6537653	5,428996333	-0,224769	0,855732032	Sox7
17319527	3,386372	3,467279	3,23017	3,284373	2,986159	3,13899	3,3612737	3,136507333	-0,22476633	0,855733614	---
17359855	4,198182	4,198182	4,710007	4,099689	4,042282	4,290138	4,3687903	4,144036333	-0,224754	0,855740929	LOC100861983
17201325	4,177191	3,309675	4,163574	3,908195	3,387687	3,680407	3,88348	3,658763	-0,224717	0,855762876	---
17430231	4,885059	4,852691	4,67979	4,718117	4,589379	4,435929	4,8058467	4,581141667	-0,224705	0,855769994	Gm12947
17225984	3,974238	4,800143	4,430903	3,941068	4,234782	4,355451	4,4017613	4,177100333	-0,224661	0,855796094	---
17548246	4,457687	4,436346	4,504794	4,164966	4,322886	4,237106	4,4662757	4,241652667	-0,224623	0,855818636	Gm10723
17376862	5,409839	5,219529	5,373877	5,123181	5,245717	4,960683	5,334415	5,109860333	-0,22455467	0,855859173	---
17205291	2,998697	3,243094	3,097833	2,776052	3,024387	2,865644	3,113208	2,888694333	-0,22451367	0,855883496	---
17209481	2,998697	3,243094	3,097833	2,776052	3,024387	2,865644	3,113208	2,888694333	-0,22451367	0,855883496	---
17423049	4,433446	3,740214	4,569188	4,368071	3,911291	3,789976	4,247616	4,023112667	-0,22450333	0,855889626	---
17202445	3,414343	3,553885	3,381781	3,596535	3,231679	2,848322	3,450003	3,225512	-0,224491	0,855896943	---
17511885	4,591474	4,662896	4,773468	4,436068	4,556717	4,361586	4,675946	4,451457	-0,224489	0,85589813	---
17450119	4,082744	4,02792	4,040419	3,70459	3,72528	4,047882	4,050361	3,825917333	-0,22444367	0,855925025	---
17351831	11,00298	11,51987	11,23302	10,97379	11,09092	11,01789	11,251957	11,02753333	-0,22442333	0,855937088	Snord58b
17504112	8,427773	8,526762	8,384873	8,139306	8,298391	8,228502	8,4464693	8,222066333	-0,224403	0,855949152	Arl2bp
17538571	4,704012	4,407308	4,683213	4,459814	4,34273	4,31878	4,5981777	4,373774667	-0,224403	0,855949152	Tmem29 // Tmem29
17418462	4,549084	4,744357	5,336534	4,479879	4,70062	4,776341	4,8766583	4,65228	-0,22437833	0,855963787	1700041M05Rik
17547931	10,78955	11,02844	10,80547	10,6163	10,57049	10,76369	10,874487	10,65016	-0,22432667	0,855994441	Set // Set
17407826	5,807924	5,982544	6,038095	5,470093	5,879585	5,806047	5,9428543	5,718575	-0,22427933	0,856022526	Mcl1 // Mcl1
17336071	4,80187	4,678967	4,326497	4,489629	4,144887	4,500272	4,6024447	4,378262667	-0,224182	0,856080281	Olfr55
17398115	4,78772	4,437165	4,785537	4,547551	4,399381	4,39095	4,6701407	4,445960667	-0,22418	0,856081468	Ptx3
17343789	5,961828	6,844094	5,632368	5,865826	5,954312	5,945727	6,1460967	5,921955	-0,22414167	0,856104215	Psmb9
17209735	5,627995	5,564349	5,66837	6,332351	4,537863	5,318147	5,620238	5,396120333	-0,22411767	0,856118456	---
17398441	6,135649	5,844133	5,913542	5,612403	5,574929	6,033726	5,9644413	5,740352667	-0,22408867	0,856135666	Accn5
17455736	5,29213	4,794019	4,980061	4,846463	4,618721	4,928771	5,02207	4,797985	-0,224085	0,856137842	LOC100862180 // LOC100862166 // Gm7
17229389	5,20619	5,36439	5,340467	5,071329	5,102543	5,065079	5,3036823	5,079650333	-0,224032	0,856169294	Uck2
17516227	3,968182	3,56317	3,892963	3,702514	3,415221	3,634487	3,808105	3,584074	-0,224031	0,856169887	Olfr922
17263705	6,790728	6,561185	6,456553	6,381135	6,252792	6,502809	6,602822	6,378912	-0,22391	0,856241698	Gm16516
17374610	7,486196	7,455873	7,416526	7,223978	7,204877	7,258065	7,452865	7,228973333	-0,22389167	0,856252579	5430417L22Rik
17524077	5,252528	5,338366	5,414175	5,024413	5,268913	5,04012	5,335023	5,111148667	-0,22387433	0,856262866	Fut4
17290823	7,391846	7,428077	7,007426	6,921337	7,057326	7,177162	7,275783	7,051941667	-0,22384133	0,856282453	1600012F09Rik
17386090	4,282392	4,59181	4,837004	4,533871	4,252998	4,252995	4,570402	4,346621333	-0,22378067	0,856318461	Scn7a
17548494	3,382272	3,914413	2,931293	3,177715	3,432396	2,946587	3,409326	3,185566	-0,22376	0,856330728	Vmn2r122
17395141	8,900433	8,982516	9,01053	8,629005	8,900756	8,692469	8,964493	8,740743333	-0,22374967	0,856336861	---
17416714	6,556358	6,690729	6,705998	6,558475	6,295922	6,427442	6,6510283	6,427279667	-0,22374867	0,856337455	Cc2d1b
17486764	4,36446	4,340546	4,461851	4,178641	4,154587	4,162422	4,3889523	4,165216667	-0,22373567	0,856345171	Obox6
17528772	7,866602	8,226941	8,172568	7,778922	8,005304	7,810712	8,0887037	7,864979333	-0,22372433	0,856351899	LOC100861984
17535642	3,536984	2,96153	3,426786	3,162875	3,101929	2,989332	3,3084333	3,084712	-0,22372133	0,856353679	---
17450501	8,790777	10,35132	7,986391	8,671318	8,872492	8,913635	9,0428293	8,819148333	-0,223681	0,856377621	Gbp10
17201547	5,934948	6,0234	6,221427	5,635684	5,963191	5,909914	6,059925	5,836263	-0,223662	0,856388899	---
17523630	5,686711	5,379007	5,76998	5,331787	5,632612	5,200412	5,6118993	5,388270333	-0,223629	0,856408488	---
17423739	3,203131	3,246006	3,030635	2,996805	2,903018	2,909274	3,159924	2,936365667	-0,22355833	0,856450438	---
17439013	4,580802	3,832814	4,034961	3,935809	3,915675	3,926437	4,1495257	3,925973667	-0,223552	0,856454198	Epgn
17285780	4,477019	4,90503	4,55416	4,745332	4,234172	4,286157	4,645403	4,421887	-0,223516	0,85647557	Vmn1r208 // Vmn1r208
17548328	5,591028	5,640759	5,763194	5,471894	5,291475	5,561402	5,6649937	5,441557	-0,22343667	0,856522668	Ube4b // Ube4b
17351838	8,532601	8,237899	8,362319	8,234131	8,019202	8,209291	8,3776063	8,154208	-0,22339833	0,856545427	---
17292640	6,222097	5,961777	6,108587	5,753416	5,873776	5,995162	6,097487	5,874118	-0,223369	0,856562843	Ror2
17435048	6,801449	6,874267	6,732567	6,383822	6,719239	6,635356	6,802761	6,579472333	-0,22328867	0,85661054	Tmem60
17534074	5,958249	6,425209	5,996189	5,796692	6,013518	5,899591	6,126549	5,903267	-0,223282	0,856614498	Lonrf3
17211568	6,887954	6,828898	7,135143	6,609684	6,841641	6,730902	6,950665	6,727409	-0,223256	0,856629936	1700001G17Rik
17205733	6,620133	6,119764	5,681714	5,500183	5,881991	6,369709	6,140537	5,917294333	-0,22324267	0,856637853	---
17462219	5,869337	5,904535	5,810006	5,782007	5,638118	5,494364	5,8612927	5,638163	-0,22312967	0,856704952	Bms1 // Bms1
17404811	3,787952	3,816206	3,782388	3,628913	3,377755	3,710561	3,7955153	3,572409667	-0,22310567	0,856719204	E330009D11
17236096	9,463626	9,799486	9,559301	9,353946	9,365365	9,433917	9,607471	9,384409333	-0,22306167	0,856745333	---
17353962	7,762515	7,732509	7,595521	7,744469	7,255274	7,421645	7,6968483	7,473796	-0,22305233	0,856750876	Fgf1
17201531	7,305398	7,030556	7,294649	7,522101	6,398108	7,041289	7,210201	6,987166	-0,223035	0,856761169	---
17357815	8,803176	9,19166	8,823148	8,756692	8,713261	8,679015	8,939328	8,716322667	-0,22300533	0,856778787	Fam111a
17476424	9,25376	9,177617	9,215766	9,435464	8,782482	8,760432	9,2157143	8,992792667	-0,22292167	0,856828476	Prodh2
17279107	6,090267	5,877425	6,242341	5,717223	5,815865	6,008215	6,070011	5,847101	-0,22291	0,856835405	A230065H16Rik
17207377	5,80473	6,392499	6,207554	6,050685	6,057084	5,628534	6,1349277	5,912101	-0,22282667	0,856884899	---
17476609	4,637566	4,492094	4,503625	4,699906	4,14167	4,123326	4,5444283	4,321634	-0,22279433	0,856904104	Gm5325
17457609	8,823084	9,083935	8,864271	8,812029	8,532859	8,758197	8,9237633	8,701028333	-0,222735	0,856939346	Mgam
17342538	4,997732	4,95061	5,054021	4,813194	4,557496	4,963603	5,0007877	4,778097667	-0,22269	0,856966076	A930017K11Rik
17417078	4,303915	3,639186	3,685458	3,530791	3,775807	3,653935	3,8761863	3,653511	-0,22267533	0,856974788	---
17394060	6,061859	6,213821	6,32061	5,988799	5,969666	5,969927	6,1987633	5,976130667	-0,22263267	0,857000133	---
17511565	3,80802	3,778152	3,655524	3,548116	3,540574	3,485263	3,747232	3,524651	-0,222581	0,857030825	---
17204411	5,813539	4,899521	4,326848	5,186303	4,835091	4,350882	5,0133027	4,790758667	-0,222544	0,857052805	---
17428419	4,587755	4,37411	4,830308	4,370378	4,226053	4,528111	4,597391	4,374847333	-0,22254367	0,857053003	---
17427207	4,71096	4,474003	4,882591	4,888864	4,160205	4,350921	4,6891847	4,466663333	-0,22252133	0,857066271	---
17206599	5,473101	4,912503	5,928225	5,147458	5,94805	4,550768	5,437943	5,215425333	-0,22251767	0,857068449	---
17215697	6,706765	6,464786	6,735622	6,399809	6,520246	6,319583	6,6357243	6,413212667	-0,22251167	0,857072013	Ramp1
17523457	6,948109	6,868979	6,764043	6,473023	6,804103	6,636712	6,860377	6,637946	-0,222431	0,857119937	D9Ertd402e
17285803	3,897973	3,583719	3,46952	3,23069	3,461926	3,591324	3,650404	3,42798	-0,222424	0,857124096	Vmn1r223
17372039	4,965466	4,686732	5,039468	4,605829	4,589379	4,829436	4,897222	4,674881333	-0,22234067	0,857173606	Hoxd12
17200249	5,719263	4,73366	5,610102	5,062698	4,916656	5,416669	5,3543417	5,132007667	-0,222334	0,857177567	---
17528768	6,329746	6,055956	6,032014	5,859204	5,908699	5,982815	6,1392387	5,916906	-0,22233267	0,85717836	4930509E16Rik
17220937	3,664313	3,658198	3,778732	3,50894	3,487164	3,438348	3,7004143	3,478150667	-0,22226367	0,857219357	---

17467239	6,070806	5,344115	6,091422	5,928225	5,197132	5,714272	5,8354477	5,613209667	-0,222238	0,857234608	---
17498323	5,888394	5,637236	5,899199	5,754137	5,52654	5,477545	5,8082763	5,586074	-0,22220233	0,857255801	Tnfrsf23
17415172	3,266746	3,270892	2,944799	3,15971	2,758317	2,897863	3,1608123	2,93863	-0,22218233	0,857267685	---
17206349	6,887668	6,81406	6,735004	5,809862	7,298991	6,661397	6,812244	6,590083333	-0,22216067	0,85728056	---
17201335	4,102222	4,780201	4,252894	3,680495	4,258605	4,529775	4,378439	4,156291667	-0,22214733	0,857288483	---
17303589	4,572781	4,025068	4,211829	3,96545	3,937007	4,240984	4,2698427	4,047813667	-0,222079	0,857329089	Olfr720
17245675	7,130924	6,953641	6,901939	6,74542	6,845074	6,730047	6,9955013	6,773513667	-0,22198767	0,857383366	Tsfm
17308734	8,366778	8,502588	8,185795	8,015721	8,160542	8,21298	8,3517203	8,129747667	-0,22197267	0,857392281	Gtf2f2
17478864	8,550764	8,715841	8,679611	8,286913	8,491638	8,501952	8,6487387	8,426834333	-0,22190433	0,857432892	Mtmr10
17203013	6,045864	4,844294	5,096572	4,896065	5,073768	5,351259	5,32891	5,107030667	-0,22187933	0,85744775	---
17446616	3,48937	3,557357	3,544976	3,499116	3,271671	3,155467	3,5305677	3,308751333	-0,22181633	0,857485194	---
17231491	4,933087	5,223134	4,855891	4,749084	4,68055	4,91725	5,0040373	4,782294667	-0,22174267	0,85752898	Gm3213
17481205	4,490521	4,231842	4,550708	4,166704	4,273924	4,167407	4,424357	4,202678333	-0,22167867	0,857567022	Olfr606
17330152	4,223897	4,973823	4,559141	4,475005	4,225952	4,390911	4,5856203	4,363956	-0,22166433	0,857575542	---
17399845	5,538309	5,310056	5,3678	5,241523	5,289733	5,019971	5,4053883	5,183742333	-0,221646	0,85758644	Pglyrp3
17299268	8,311747	8,439754	8,527491	8,179944	8,113854	8,320268	8,4263307	8,204688667	-0,221642	0,857588818	Cdkn3
17459801	7,009777	6,718801	6,904644	6,519845	6,848288	6,600674	6,8777407	6,656269	-0,22147167	0,857690076	AW146020
17420445	8,850776	8,206307	8,389545	8,639118	7,699434	8,443725	8,4822093	8,260759	-0,22145033	0,857702759	---
17242306	5,239321	5,430603	5,652472	5,194111	5,30233	5,161629	5,4407987	5,219356667	-0,221442	0,857707713	LOC100862482
17213317	8,581232	9,089728	8,74291	8,656816	8,618618	8,474127	8,8046233	8,583187	-0,22143633	0,857711082	---
17399794	5,355667	5,33665	5,462674	4,898375	5,424092	5,168362	5,384997	5,163609667	-0,22138733	0,857740214	S100a2
17447038	7,59187	7,523327	7,380137	6,891006	7,301005	7,639241	7,4984447	7,277084	-0,22136067	0,857756069	Ppp1cb // Ppp1cb
17200133	7,928985	7,471892	8,156171	7,800215	7,709991	7,382949	7,8523493	7,631051667	-0,22129767	0,857793526	---
17497514	5,083737	4,723726	4,950706	4,670556	4,645779	4,778253	4,9193897	4,698196	-0,22119367	0,857855365	6430531B16Rik
17325093	7,181489	7,279546	6,896881	6,692328	6,937273	7,064775	7,1193053	6,898125333	-0,22118	0,857863491	Muc13
17355304	6,068461	6,758341	6,322093	6,534014	5,878968	6,07254	6,382965	6,161840667	-0,22112433	0,857896593	4930503L19Rik
17550452	7,790277	7,694193	7,645669	7,443669	7,46983	7,553298	7,7100463	7,488932333	-0,221114	0,857902737	---
17344568	8,354718	8,50336	8,254443	8,079358	8,142132	8,228059	8,3708403	8,149849667	-0,22099067	0,857976081	H2-T23
17427792	5,017085	4,846398	4,894593	4,825875	4,530612	4,738656	4,9193587	4,698381	-0,22097767	0,857983812	Tmem61
17231421	4,558515	3,941137	4,241045	4,099021	3,97019	4,008661	4,246899	4,025957333	-0,22094167	0,858005222	---
17257054	9,380487	9,497282	9,671251	9,083908	9,401674	9,400614	9,51634	9,295398667	-0,22094133	0,85800542	---
17540574	4,039774	4,581026	4,07366	4,038082	4,015827	3,977808	4,2314867	4,010572333	-0,22091433	0,858021478	4930453H23Rik
17491406	4,74531	4,662376	4,748977	4,353744	4,522205	4,617998	4,7188877	4,497982333	-0,22090533	0,858026831	1700081H22Rik
17257055	10,71485	10,43149	10,62782	10,44039	10,45376	10,21796	10,591387	10,37070333	-0,22068333	0,858158873	---
17414364	6,450924	6,278757	6,334725	6,254762	6,17145	5,976176	6,354802	6,134129333	-0,22067267	0,858165218	Akap2
17341470	4,048205	3,938719	4,159731	4,010903	3,790722	3,683107	4,048885	3,828244	-0,220641	0,858184054	Vmn2r114
17302919	6,331107	6,467876	6,685662	6,309732	6,322491	6,190724	6,4948817	6,274315667	-0,220566	0,858228669	4930594M22Rik
17490180	5,355002	4,5432	5,155964	5,186175	4,429355	4,776988	5,0180553	4,797506	-0,22054933	0,858238584	---
17300408	5,299658	5,174331	5,585965	5,477873	5,035509	4,884927	5,353318	5,132769667	-0,22054833	0,858239179	Zfhx2 // Zfhx2
17400006	6,554728	6,592439	6,534375	6,755163	6,072242	6,192628	6,560514	6,340011	-0,220503	0,858266147	Tdpoz2 // Tdpoz2
17418854	6,15752	6,091831	5,92498	5,901627	5,615106	5,996307	6,0581103	5,83768	-0,22043033	0,858309378	---
17468816	5,699362	5,556832	5,422318	5,285727	5,334023	5,397498	5,559504	5,339082667	-0,22042133	0,858314732	---
17447326	6,630201	5,989182	6,625074	5,95832	6,230316	6,394791	6,414819	6,194475667	-0,22034333	0,858361139	---
17261439	5,360247	5,624745	5,467884	5,106361	5,27564	5,409855	5,484292	5,263952	-0,22034	0,858363122	Bod1
17224154	4,195956	4,747412	4,444607	4,070064	4,340013	4,316997	4,4626583	4,242358	-0,22030033	0,858386723	Ankar
17288239	5,544752	5,828722	4,678112	5,473465	5,258587	4,658654	5,3505287	5,130235333	-0,22029333	0,858390888	Gm10324 // Gm10324
17232591	3,243112	3,050085	3,050514	2,856042	2,931742	2,895055	3,1145703	2,894279667	-0,22029067	0,858392475	---
17476535	5,103462	5,061538	5,287949	4,84928	5,054112	4,888695	5,150983	4,930695667	-0,22028733	0,858394458	Dmkn
17426940	3,514125	3,741991	3,401563	3,339756	3,301949	3,355232	3,5525597	3,332312333	-0,22024733	0,858418258	---
17208609	4,962665	5,745379	5,735923	5,626596	4,907869	5,248996	5,4813223	5,261153667	-0,22016867	0,858465067	---
17305592	5,759737	5,615924	5,705534	5,793797	5,256977	5,370263	5,6937317	5,473679	-0,22005267	0,858534094	4930431P03Rik
17268784	3,274996	3,2726	3,194546	3,07198	2,983706	3,026428	3,2473807	3,027371333	-0,22000933	0,858559882	---
17432458	4,101969	4,127079	4,038408	4,126953	3,709031	3,771502	4,089152	3,869162	-0,21999	0,858571388	---
17455554	8,082539	8,119015	8,231883	7,907761	8,029903	7,83601	8,144479	7,924558	-0,219921	0,858612452	N4bp2l1
17492327	4,338254	3,977942	4,601832	3,887855	3,909117	4,461294	4,3060093	4,086088667	-0,21992067	0,85861265	---
17518793	5,049211	4,993742	5,158957	4,982644	4,736273	4,823281	5,0673033	4,847399333	-0,219904	0,858622569	---
17204817	7,579548	7,795827	7,614131	7,616016	7,694818	7,019003	7,6631687	7,443279	-0,21988967	0,8586311	---
17298620	5,620533	5,55517	5,661786	5,254981	5,424342	5,498561	5,6124963	5,392628	-0,21986833	0,858643796	1810011H11Rik
17390596	7,963023	8,340304	8,090414	7,70556	7,911733	8,116858	8,131247	7,911383667	-0,21986333	0,858646772	Serf2 // Serf2
17380464	8,781712	9,077237	8,915012	8,544785	8,646091	8,923643	8,9246537	8,704839667	-0,219814	0,858676134	2210418O10Rik
17367726	4,885325	4,472653	4,308971	4,358377	4,401057	4,248201	4,5556497	4,335878333	-0,21977133	0,85870153	---
17499956	3,413254	3,583197	3,599582	3,282203	3,303099	3,351504	3,532011	3,312268667	-0,21974233	0,858718791	---
17302548	3,498934	3,488174	3,093203	2,906405	3,171048	3,343766	3,3601037	3,140406333	-0,21969733	0,858745576	---
17291490	3,470659	3,398063	3,367116	2,937584	3,304569	3,334607	3,411946	3,192253333	-0,21969267	0,858748354	Prl7d1
17209837	5,520802	5,225176	5,438217	5,505032	4,679616	5,34051	5,3947317	5,175052667	-0,219679	0,858756489	---
17206683	5,549414	5,345059	5,602084	5,155141	5,267297	5,415165	5,4988523	5,279201	-0,21965133	0,858772957	---
17530881	6,462017	6,678699	6,706519	6,275323	6,453182	6,459812	6,615745	6,396105667	-0,21963933	0,8587801	Cish // Cish
17210731	5,171987	5,530552	5,52396	5,842094	4,998801	4,72676	5,408833	5,189218333	-0,21961467	0,858794784	---
17204359	3,991905	4,694499	4,829631	4,83096	3,698006	4,328228	4,505345	4,285731333	-0,21961367	0,858795379	---
17549922	5,492846	5,079921	5,329365	5,067192	5,076385	5,099748	5,3007107	5,081108333	-0,21960233	0,858802125	---
17251653	5,319772	5,230309	5,403666	5,262176	4,691483	5,341349	5,3179157	5,098336	-0,21957967	0,858815618	Kdm6b
17331019	6,354482	6,42098	6,349656	5,893719	6,271377	6,30137	6,3750393	6,155488667	-0,21955067	0,858832882	2310061J03Rik
17422678	5,4254	5,473324	5,78965	5,345531	5,232135	5,452086	5,5627913	5,343250667	-0,21954067	0,858838835	Vwa1
17395844	6,54359	6,260546	6,460136	6,070146	6,316887	6,218658	6,421424	6,201897	-0,219527	0,858846971	Stmn3
17350203	5,049016	4,779597	5,284451	4,813187	4,884378	4,756933	5,037688	4,818166	-0,219522	0,858849947	Spink5
17432503	6,745127	6,127608	6,85186	6,65488	6,149932	6,261882	6,574865	6,355564667	-0,21930033	0,858981918	---
17368163	3,724853	3,93337	3,791702	3,571231	3,779198	3,441665	3,8166417	3,597364667	-0,219277	0,85899581	Lcn13
17365623	6,436329	6,500407	6,147589	5,963917	6,154143	6,308578	6,3614417	6,142212667	-0,219229	0,859024391	---
17534247	3,707207	3,465126	3,927741	3,425928	3,32249	3,694027	3,7000247	3,480815	-0,21920967	0,859035902	---
17359940	8,766048	9,20666	9,378703	8,998181	9,012686	8,683071	9,117137	8,897979333	-0,21915767	0,859066866	---
17531482	10,46935	10,89147	10,88863	10,50696	10,636	10,44906	10,749817	10,53067333	-0,21914333	0,859075401	Camp
17516124	4,042325	3,748953	3,710719	3,6388	3,445708	3,760326	3,833999	3,614911333	-0,21908767	0,859108549	Olfr878
17548683	7,442478	7,679321	7,593752	7,217539	7,292593	7,548429	7,5718503	7,352853667	-0,21899667	0,85916274	Rab8a // Rab8a
17541108	4,850333	4,700604	5,147017	4,634521	4,922763	4,483686	4,899318	4,680323333	-0,21899467	0,859163931	Rhox8
17273304	5,602948	5,351073	5,837576	5,230345	5,343096	5,561225	5,597199	5,378222	-0,218977	0,859174452	Cbr2
17205059	5,605546	5,825562	6,10774	5,450692	5,701813	5,729536	5,8462827	5,627347	-0,21893567	0,859199068	---
17209249	5,605546	5,825562	6,10774	5,450692	5,701813	5,729536	5,8462827	5,627347	-0,21893567	0,859199068	---
17358018	3,899787	3,43112	3,993208	3,733325	3,422203	3,511906	3,774705	3,555811333	-0,21889367	0,859224081	---
17519789	4,238678	4,071415	4,187472	3,972848	3,961047	3,907311	4,165855	3,947068667	-0,21878633	0,859288008	---
17360078	6,292903	6,12431	6,046695	5,72792	6,000998	6,078643	6,154636	5,935853667	-0,21878233	0,859290391	2010012O05Rik
17208959	5,407363	5,245724	5,431633	5,353047	4,98339	5,092262	5,3615733	5,142899667	-0,21867367	0,859355117	---
17202987	4,669446	4,468444	3,671804	3,827807	3,821243	4,504634	4,269898	4,051228	-0,21867	0,859357301	---
17453106	5,933719	6,204231	6,386792	5,806663	6,231064	5,831038	6,174914	5,956255	-0,218659	0,859363853	Zfp11
17268640	3,994832	6,267233	6,129892	4,861149	4,320177	6,554924	5,4639857	5,245416667	-0,218569	0,859417465	---
17337193	5,235874	5,557343	5,331761	5,118289	5,119707	5,231639	5,3749927	5,156545	-0,21844767	0,859489746	Psors1c2
17405168	4,659667	4,418959	4,709742	4,470831	4,527205	4,13544	4,5961227	4,377825333	-0,21829733	0,859579312	LOC622124
17487471	6,089315	6,419953	6,288821	5,976652	6,115928	6,050624	6,2660297	6,047734667	-0,218295	0,859580703	Ceacam16
17355634	5,756906	5,98388	5,614691	5,395556	5,711379	5,593686	5,785159	5,566873667	-0,21828533	0,859586462	Hsbp1l1
17378315	9,484195	9,828964	9,254292	9,013151	9,324605	9,57495	9,5224837	9,304235333	-0,21824833	0,859608508	---
17299351	3,088061	3,309125	3,084785	2,828365	2,871208	3,127682	3,160657	2,942418333	-0,21823867	0,859614268	---
17240957	5,481872	5,428328	5,514335	5,677308	5,137711	4,955097	5,474845	5,256705333	-0,21813967	0,859673258	---
17299493	3,416449	3,413514	3,295663	3,050296	3,159327	3,261607	3,3752087	3,157076667	-0,218132	0,859677826	Olfr742
17300061	4,559912	4,117609	4,709686	4,409277	4,094185	4,229516	4,4624023	4,244326	-0,21807633	0,859710998	---
17203881	4,701343	5,156477	4,643016	4,37869	4,842819	4,625212	4,833612	4,615573667	-0,21803833	0,859733642	---
17333962	5,151433	5,389811	4,94347	4,733171	4,964381	5,133098	5,1615713	4,94355	-0,21802133	0,859743773	Gm10509 // Gm10509
17458006	4,209467	3,955922	3,749363	3,650534	3,751596	3,858672	3,971584	3,753600667	-0,21798333	0,859766419	Olfr13
17409513	10,44761	10,42891	10,74692	10,9681	10,11485	9,886648	10,541147	10,32319933	-0,21794733	0,859787873	Amy2a5
17201311	3,105647	2,744061	4,067469	3,226817	3,018262	3,018262	3,3057257	3,087780333	-0,21794533	0,859789065	---
17349552	7,060331	7,350744	7,566083	7,121591	7,311442	6,89051	7,3257193	7,107847667	-0,21787167	0,859832968	Snora74a
17305910	4,936025	4,742043	4,722834	4,373187	4,750082	4,624057	4,8003007	4,582442	-0,21785867	0,859840716	3632451O06Rik
17408345	7,991308	8,290206	8,749088	8,634853	7,599052	8,143227	8,343534	8,125710667	-0,21782333	0,859861775	Hsd3b6 // Hsd3b6
17363078	4,751534	4,664274	4,522861	4,421051	4,686337	4,177886	4,646223	4,428424667	-0,21779833	0,859876675	Olfr262
17203843	5,217458	5,065495	5,885576	4,90296	5,255376	5,356954	5,3895097	5,171763333	-0,21774633	0,859907669	---
17455507	8,704689	8,780489	8,433548	8,322002	8,343317	8,600334	8,6395753	8,421884333	-0,217691	0,859940651	Hsph1
17201855	6,476089	6,79036	7,366512	6,988023	6,668965	6,323029	6,8776537	6,660005667	-0,217648	0,859966282	---
17489933	5,060619	4,802451	5,113034	4,699493	4,78839	4,83553	4,9920347	4,774471	-0,21756367	0,860016553	---
17345391	5,497841	4,967185	4,731839	4,910054	4,878036	4,75611	5,0656217	4,848066667	-0,217555	0,860021719	Slc22a7
17406452	10,61848	11,01621	10,99345	10,77466	10,75183	10,449	10,876047	10,65849667	-0,21755	0,8600247	Rnu73b
17550178	3,377985	3,354555	3,200618	3,10528	3,141089	3,034335	3,3110527	3,093568	-0,21748467	0,860063648	---
17305585	4,509693	4,418873	4,429707	4,165149	4,230491	4,310429	4,4527577	4,235356333	-0,21740133	0,860113328	Ptgdr
17480641	5,48811	5,571456	5,833842	5,447852	5,470261	5,323092	5,631136	5,413735	-0,217401	0,860113527	Kcne3
17471881	4,555305	4,626575	4,494452	4,086078	4,521062	4,417082	4,5587773	4,341407333	-0,21737	0,860132009	Tas2r130
17205123	4,17732	3,971634	5,236205	3,859212	4,602653	4,271186	4,4617197	4,244350333	-0,21736933	0,860132406	---
17209313	4,17732	3,971634	5,236205	3,859212	4,602653	4,271186	4,4617197	4,244350333	-0,21736933	0,860132406	---
17538830	9,141737	9,226464	9,030727	8,151549	9,308389	9,286993	9,132976	8,915643667	-0,21733233	0,860154466	---
17531395	4,752242	4,381766	5,133665	4,368816	4,737612	4,509361	4,755891	4,538596333	-0,21729467	0,860176924	Fbxw23
17202171	5,686255	5,631177	5,814138	5,85992	4,835312	5,784457	5,7105233	5,493229667	-0,21729367	0,86017752	---
17223390	8,497339	8,703178	8,620857	8,350368	8,367073	8,452085	8,6071247	8,389842	-0,21728267	0,860184079	Orc2
17390997	7,936817	7,861358	7,840768	7,632261	7,738813	7,616024	7,8796477	7,662366	-0,21728167	0,860184675	Secisbp2l
17361090	11,30933	11,36733	11,59737	11,21808	11,18369	11,22045	11,424677	11,20740667	-0,21727	0,860191631	BC021614
17363171	3,498356	3,186485	3,473269	3,480255	2,827918	3,198222	3,3860367	3,168798333	-0,21723833	0,860210512	Olfr1501
17415813	4,097625	3,62943	3,524457	3,418747	3,586029	3,59504	3,750504	3,533272	-0,217232	0,860214288	---
17545977	3,742074	3,569928	3,291222	3,226622	3,391559	3,333383	3,534408	3,317188	-0,21722	0,860221443	---
17248209	4,235469	4,105505	4,246564	3,989077	3,771259	4,175733	4,195846	3,978689667	-0,21715633	0,860259406	---

17200311	3,211997	2,985439	3,290582	2,70765	3,262549	2,866364	3,1626727	2,945521	-0,21715167	0,860262189	---
17414097	3,360174	3,312074	3,289438	3,066507	3,357916	2,885854	3,320562	3,103425667	-0,21713633	0,860271332	---
17202347	4,705082	3,87488	4,199275	4,328793	3,973302	3,825812	4,2597457	4,042635667	-0,21711	0,860287035	---
17280254	5,352182	5,428185	5,00221	4,848343	4,821138	5,461788	5,260859	5,043756333	-0,21710267	0,860291407	Gm5433
17265724	5,713627	5,955352	5,508615	5,244945	5,592659	5,688758	5,7258647	5,508787333	-0,21707733	0,860306514	Gsg2
17351840	8,722261	9,468328	9,011652	8,719143	8,719309	9,112663	9,0674137	8,850371667	-0,217042	0,860327584	---
17455401	7,052492	7,524492	7,155277	7,110878	6,813522	7,156765	7,244087	7,027055	-0,217032	0,860333548	Slc7a1
17312066	3,819045	3,670017	3,732044	3,576596	3,31804	3,675413	3,7403687	3,523349667	-0,217019	0,8603413	---
17508125	6,453208	6,703854	6,747037	6,381208	6,360165	6,512096	6,6346997	6,417823	-0,21687667	0,860426184	Ank1 // Ank1
17549436	7,334335	7,438916	7,306535	7,089958	7,15662	7,183455	7,3599287	7,143344333	-0,21658433	0,86060055	---
17295757	9,936415	9,984938	10,02518	9,685046	9,855414	9,75649	9,9821777	9,76565	-0,21652767	0,860634353	Ccnb1
17260775	8,750811	8,705424	8,482363	8,293237	8,494899	8,501176	8,6461993	8,429770667	-0,21642867	0,860693413	Pno1
17373937	4,750622	4,960531	4,952663	4,590948	4,700839	4,722917	4,8879387	4,671568	-0,21637067	0,860728016	LOC100862167
17363101	6,751658	6,850348	6,369684	6,597873	6,272553	6,452329	6,65723	6,440918333	-0,21631167	0,860763217	Keg1
17378337	6,806279	7,052763	7,022552	6,710766	6,808649	6,713336	6,9605313	6,744250333	-0,216281	0,860781514	Map1lc3a
17451356	5,541641	5,370595	5,754601	5,408213	5,244399	5,36541	5,5556123	5,339340667	-0,21627167	0,860787083	Sgsm1
17281919	3,809289	3,620721	4,06345	3,795846	3,480486	3,568381	3,8311533	3,614904333	-0,216249	0,860800607	---
17308299	9,152119	9,351207	9,130935	9,113249	8,982473	8,890182	9,2114203	8,995301333	-0,216119	0,860878176	Slc39a14
17509498	6,423847	5,955798	6,162338	6,259759	5,620186	6,013752	6,180661	5,964565667	-0,21609533	0,860892299	Gm15881
17352549	7,165126	7,641946	7,063667	7,226206	7,090921	6,905378	7,2902463	7,074168333	-0,216078	0,860902642	Mpp7
17382098	4,208116	3,862777	3,99666	3,625026	4,032634	3,761785	4,0225177	3,806481667	-0,216036	0,860927705	---
17501504	6,813843	6,443746	6,100789	6,292185	6,030998	6,387306	6,4527927	6,236829667	-0,215963	0,860971269	---
17311428	8,459283	8,37433	8,224982	8,195217	8,02282	8,192848	8,352865	8,136961667	-0,21590333	0,861006878	Ebag9
17206651	3,327971	2,816391	3,135597	2,78638	2,78638	3,05954	3,0933197	2,877433333	-0,21588633	0,861017023	---
17464080	11,40205	11,24727	11,27797	10,94247	11,18443	11,1528	11,309097	11,09323333	-0,21586333	0,86103075	Slco1b2
17489965	4,813343	5,186856	5,198501	4,772658	4,910697	4,86782	5,0662333	4,850391667	-0,21584167	0,861043681	Gm6124
17202025	4,425797	5,128468	4,767479	4,781246	4,167336	4,726242	4,7739147	4,558274667	-0,21564	0,86116405	---
17403533	7,806873	7,729641	7,595381	7,293284	7,556732	7,63499	7,7106317	7,495002	-0,21562967	0,861170218	Ctbs
17491454	7,63744	7,843199	7,715632	7,588298	7,367611	7,593554	7,7320903	7,516487667	-0,21560267	0,861186335	Nipa1
17536950	6,218782	6,016908	6,694921	5,909606	6,310001	6,064248	6,3102037	6,094618333	-0,21558533	0,861196682	1700018G05Rik
17210793	3,119588	3,430924	2,939109	3,209539	2,718995	2,914351	3,163207	2,947628333	-0,21557867	0,861200662	---
17402535	4,48339	4,02787	3,998537	3,9121	3,93705	4,013922	4,1699323	3,954357333	-0,215575	0,861202851	---
17445596	4,420089	4,413713	4,815438	4,235146	4,332652	4,434834	4,5497467	4,334210667	-0,215536	0,861226132	Grm3
17473477	7,886533	7,924166	8,141776	7,6199	7,910918	7,775084	7,9841583	7,768634	-0,21552433	0,861233096	U2af2
17442719	6,931726	7,457588	7,039219	7,039704	6,674812	7,067459	7,1428443	6,927325	-0,21551933	0,861236081	Aacs
17238675	3,639452	3,882225	3,560215	3,86514	3,28891	3,281325	3,693964	3,478458333	-0,21550567	0,861244239	Olfr791
17207241	4,71344	3,617488	4,490758	3,911873	4,237303	4,026325	4,2738953	4,058500333	-0,215395	0,861310307	---
17378450	6,216123	5,98739	6,275811	5,805073	6,008678	6,019606	6,1597747	5,944452333	-0,21532233	0,861353691	Mmp24
17457683	3,793942	3,888949	4,533059	3,947164	3,807988	3,814962	4,0719833	3,856704667	-0,21527867	0,861379762	---
17380360	8,767359	8,58882	8,44297	8,203417	8,563141	8,386843	8,5997163	8,384467	-0,21524933	0,861397276	Tubb1
17265126	9,33175	9,500964	9,445431	9,06992	9,259437	9,303197	9,4260483	9,210851333	-0,215197	0,861428524	Gabarap // Gabarap
17214981	4,919424	5,283067	5,0812	5,052615	4,85794	4,727658	5,0945637	4,879404333	-0,21515933	0,861451015	---
17319801	4,263692	4,655207	4,802136	3,973759	4,473743	4,628127	4,5736783	4,358543	-0,21513533	0,861465345	A4galt
17303360	6,586448	6,905386	5,490187	6,56454	5,733553	6,038532	6,3273403	6,112208333	-0,215132	0,861467336	Gm10338
17328201	9,349295	9,603984	9,388229	9,215121	9,130908	9,350226	9,4471693	9,232085	-0,21508433	0,861495799	Gspt1
17336458	5,398071	5,816973	5,469018	5,499942	5,339756	5,199137	5,561354	5,346278333	-0,21507567	0,861500974	Tap2
17201281	4,957609	4,623403	4,588957	3,84512	4,771066	4,908575	4,723323	4,508253667	-0,21506933	0,861504756	---
17278296	3,838965	4,312799	3,93068	3,817153	3,824041	3,796235	4,0274813	3,812476333	-0,215005	0,861543174	Serpina3i
17244563	4,492158	4,642436	5,127447	4,227347	4,875814	4,513903	4,7540137	4,539021333	-0,21499233	0,861550738	Csl
17262404	4,967087	5,269722	5,154421	4,715574	5,012043	5,018655	5,13041	4,915424	-0,214986	0,86155452	Zfp879
17397142	7,295	7,592486	7,151796	7,036046	7,059927	7,298494	7,3464273	7,131489	-0,21493833	0,861582986	Spata5
17444836	5,372218	4,622832	4,887745	4,739758	4,856291	4,641973	4,9609317	4,746007333	-0,21492433	0,861591347	Gsx1
17225085	4,17471	3,913627	4,020078	3,832565	3,777611	3,853813	4,0361383	3,821329667	-0,21480867	0,861660427	---
17545553	4,734448	3,846132	4,414006	4,477519	4,113278	3,759844	4,3315287	4,116880333	-0,21464833	0,861756193	Magea3
17249829	6,966672	7,339661	7,066482	6,92482	6,787674	7,016791	7,1242717	6,909761667	-0,21451	0,861838827	LOC100862216
17362839	4,839532	5,001168	5,154851	5,063306	4,576142	4,713025	4,998517	4,784157667	-0,21435933	0,861928837	Ms4a10
17437923	7,834532	7,876067	7,570656	7,425919	7,677369	7,53514	7,7604183	7,546142667	-0,21427567	0,861978824	Tmem33
17294688	3,4563	3,378054	3,545119	3,238956	3,354249	3,143515	3,4598243	3,245573333	-0,214251	0,861993562	Gm17750
17225578	3,173687	3,245746	3,584233	3,014061	3,303093	3,043766	3,3345553	3,120306667	-0,21424867	0,861994957	Olfr1416
17264314	3,037897	3,089748	3,528414	3,085682	2,931599	2,996042	3,2186863	3,004441	-0,21424533	0,861996948	---
17208819	4,938167	4,579649	3,866308	4,303847	3,88429	4,553301	4,4613747	4,247146	-0,21422867	0,862006906	---
17338378	7,549771	7,613588	7,347195	7,195558	7,423629	7,248709	7,503518	7,289298667	-0,21421933	0,862012483	Treml4
17534157	6,113493	5,816356	6,014204	5,636713	5,767877	5,896924	5,981351	5,767171333	-0,21417967	0,862036184	Rnf113a1
17210713	5,70442	4,914911	5,168084	5,175085	4,907707	5,062426	5,2624717	5,048406	-0,21406567	0,862104304	---
17455061	8,747545	8,6369	8,621326	8,330622	8,558912	8,47415	8,6685903	8,454561333	-0,214029	0,862126215	Pdap1
17366768	4,188158	3,409476	3,372015	3,564316	3,282899	3,480425	3,6565497	3,442546667	-0,214003	0,862141752	---
17466253	4,740974	3,968103	4,340424	4,189605	4,26623	3,951859	4,3498337	4,135898	-0,21393567	0,862181991	Prss58
17376977	8,069865	8,171156	8,089538	7,748228	7,986118	7,954429	8,1101863	7,896258333	-0,213928	0,862186573	Snrpb2
17386481	8,351936	8,401702	8,198436	8,032413	8,053975	8,22391	8,317358	8,103432667	-0,21392533	0,862188166	Ola1
17210179	6,686659	6,422734	6,186775	6,433724	6,527226	5,693487	6,432056	6,218145667	-0,21391033	0,862197131	---
17536653	3,179302	3,063546	2,949843	2,935043	2,732345	2,8836	3,0642303	2,850329333	-0,213901	0,862202709	---
17376096	6,786459	6,968211	6,882647	6,584925	6,707582	6,703144	6,8791057	6,665217	-0,21388867	0,86221008	Polr1b
17284696	6,18983	6,362158	6,018561	6,012286	5,989139	5,927706	6,190183	5,976377	-0,213806	0,862259486	D430020J02Rik
17202643	6,224238	6,042884	5,444815	5,73948	5,965829	5,365218	5,903979	5,690175667	-0,21380333	0,86226108	---
17501025	7,339241	7,459461	7,372063	7,21483	7,071065	7,243461	7,390255	7,176452	-0,213803	0,862261279	Mlf1ip
17207051	3,983762	4,695504	4,082019	3,869804	3,986413	4,263673	4,2537617	4,039963333	-0,21379833	0,862264068	---
17243212	7,635418	7,616319	7,630718	7,30713	7,512125	7,421877	7,627485	7,413710667	-0,21377433	0,862278412	---
17548070	6,644007	6,898634	6,596918	6,508094	6,492683	6,497595	6,7131863	6,499457333	-0,213729	0,862305508	Gm19848
17296537	8,858644	9,039105	8,973777	8,760118	8,784013	8,68626	8,9571753	8,743463667	-0,21371167	0,862315868	Zfp131
17506938	6,962832	6,730843	6,613018	6,371499	6,710742	6,583345	6,7688977	6,555195333	-0,21370233	0,862321447	Irf2bp2
17309347	6,805889	6,797144	7,073775	6,780599	6,811063	6,444329	6,8922693	6,678663667	-0,21360567	0,862379228	---
17431478	5,670326	5,668809	5,948451	5,55411	5,692542	5,40017	5,7625287	5,548940667	-0,213588	0,862389788	Grhl3
17202061	3,874473	4,34069	4,373692	3,716075	3,939457	4,29263	4,196285	3,982720667	-0,21356433	0,862403935	---
17208221	7,36098	7,17319	7,089175	7,322096	6,582662	7,078121	7,2077817	6,994293	-0,21348867	0,862449168	---
17354128	9,350821	9,37384	9,274586	9,092672	9,012226	9,253899	9,3330823	9,119599	-0,21348333	0,862452356	Gm9781
17209495	4,039515	3,467454	3,711775	3,541121	3,286796	3,750379	3,7395813	3,526098667	-0,21348267	0,862452755	---
17241436	9,103186	9,323524	9,164424	8,926476	9,079181	8,945127	9,1970447	8,983594667	-0,21345	0,862472283	Ddx21
17354170	3,757056	3,682961	3,448822	3,432773	3,370318	3,445509	3,629613	3,4162	-0,213413	0,862494403	Gm10267
17443742	6,927011	6,849435	6,792561	6,256675	6,836006	6,836094	6,8563357	6,642925	-0,21341067	0,862495798	Zscan21
17547238	3,043057	4,460095	3,970835	3,176898	4,467309	3,189692	3,8246623	3,611299667	-0,21336267	0,862524495	---
17344120	6,612522	6,750082	6,554537	6,412591	6,570745	6,29374	6,639047	6,425692	-0,213355	0,862529078	Snord52
17215462	5,32134	5,548501	5,722435	5,467115	5,082414	5,402828	5,5307587	5,317452333	-0,21330633	0,862558175	Heatr7b1
17432577	5,08015	4,451169	4,907867	4,676221	4,491303	4,631775	4,813062	4,599766333	-0,21329567	0,862564552	Gm436
17505309	3,615436	3,632639	3,986368	3,371146	3,706327	3,517115	3,7448143	3,531529333	-0,213285	0,862570929	Mir140
17292157	7,90203	7,940219	7,846135	7,549557	7,708015	7,791019	7,896128	7,682863667	-0,21326433	0,862583286	Dtnbp1
17481755	7,504099	7,458626	7,233386	7,155235	7,233117	7,168276	7,3987037	7,185542667	-0,213161	0,862645071	Swap70
17518636	8,314909	8,502491	8,247732	8,249869	8,032699	8,143081	8,355044	8,141883	-0,213161	0,862645071	2810417H13Rik
17230120	4,449729	4,519695	4,489555	4,549915	4,002313	4,267517	4,4863263	4,273248333	-0,213078	0,862694701	Olfr231
17215879	5,582243	5,84556	5,297921	5,576492	5,076618	5,433386	5,5752413	5,362165333	-0,213076	0,862695897	Aqp12
17265552	4,561885	4,760587	4,845084	4,79475	4,529129	4,204452	4,7225187	4,509443667	-0,213075	0,862696495	---
17266287	7,705722	7,39491	7,725464	7,461083	7,009658	7,716146	7,6086987	7,395629	-0,21306967	0,862699684	---
17287066	8,634577	8,868918	8,796484	8,681714	8,649964	8,329261	8,7666597	8,553646333	-0,21301333	0,862733371	Rnf144b
17268401	5,060801	5,182396	5,34015	4,780118	5,11753	5,046695	5,194449	4,981447667	-0,21300133	0,862740547	---
17533223	6,894564	6,940924	6,66506	6,68286	6,54822	6,630667	6,833516	6,620582333	-0,21293367	0,862781013	LOC100862652
17518596	6,577496	6,682754	6,711092	6,440898	6,336142	6,555525	6,657114	6,444188333	-0,21292567	0,862785798	Pif1
17200131	5,40314	5,443549	6,070027	5,322453	5,489521	5,466103	5,6389053	5,426025667	-0,21287967	0,862813308	---
17406423	4,787959	5,016537	5,14723	4,682048	4,728729	4,902398	4,9839087	4,771058333	-0,21285033	0,862830851	Glt28d2
17523416	9,270282	9,66246	9,468075	9,207055	9,26991	9,285371	9,466939	9,254112	-0,212827	0,862844806	Abhd5
17462694	5,07369	4,994026	4,762786	4,720492	4,668438	4,803285	4,9435007	4,730738333	-0,21276233	0,862883483	Nanog
17415411	4,004771	3,463959	4,247595	3,752748	3,463971	3,861423	3,9054417	3,692714	-0,21272767	0,862904217	Zfp352
17296971	6,455169	6,57848	6,193223	6,051086	6,286199	6,251445	6,4089573	6,196243333	-0,212714	0,862912391	---
17270096	7,986914	7,997303	8,249612	8,477846	7,441741	7,676111	8,077943	7,865232667	-0,21271033	0,862914585	---
17549666	6,329838	6,435411	6,511239	6,088162	6,194105	6,356097	6,425496	6,212788	-0,212708	0,86291598	---
17475352	5,451893	5,592834	5,583989	5,19016	5,378456	5,422019	5,5429053	5,330211667	-0,21269367	0,862924553	Hnrnpul1
17481628	5,068549	4,191739	4,291431	4,401407	4,27891	4,233356	4,5172397	4,304557667	-0,212682	0,862931532	Olfr507
17208225	7,035233	6,840846	7,491081	7,029674	7,068386	6,631081	7,1223867	6,909713667	-0,212673	0,862936915	---
17507319	3,706409	3,816097	3,724084	3,535389	3,544468	3,528769	3,7488633	3,536208667	-0,21265467	0,862947881	---
17395789	6,447077	6,338695	6,841256	6,442885	6,334803	6,211417	6,5423427	6,329701667	-0,212641	0,862956056	Srms
17409994	6,707077	6,890303	6,780128	6,675047	6,496239	6,568369	6,7925027	6,579885	-0,21261767	0,862970013	1810037I17Rik // 1810037I17Rik
17460770	4,231065	3,436039	3,648273	3,693028	3,482539	3,502069	3,7717923	3,559212	-0,21258033	0,862992345	V1ra8
17234736	4,231109	4,456773	4,762602	4,346232	4,053658	4,412917	4,4834947	4,270935667	-0,212559	0,863005106	Krtap10-10
17305388	4,324503	4,082156	4,287875	3,995014	3,9783	4,083817	4,2315113	4,019043667	-0,21246767	0,863059742	---
17214907	6,06508	5,855923	5,700364	5,274103	5,73576	5,974113	5,873789	5,661325333	-0,21246367	0,863062135	Csprs // Csprs
17446253	8,988272	9,061097	9,01049	8,706361	8,855143	8,861032	9,019953	8,807512	-0,212441	0,863075695	Abcf2
17524944	6,750773	7,144319	6,862669	6,732317	6,536822	6,851348	6,9192537	6,706829	-0,21242467	0,863085466	Zfp599
17417138	8,000198	8,135703	8,158516	7,784237	7,887184	7,986015	8,098139	7,885812	-0,212327	0,863143897	Tal1
17293242	9,505369	9,378822	9,301527	9,123201	9,103761	9,321859	9,3952393	9,182940333	-0,212299	0,863160649	---
17368523	5,940443	6,262578	6,26274	5,816987	6,135832	5,876065	6,1552537	5,942961333	-0,21229233	0,863164638	D2Bwg1423e
17350693	6,053353	5,764834	5,652877	5,554344	5,740505	5,539447	5,823688	5,611432	-0,212256	0,863186376	---
17395705	6,062566	6,135953	6,635943	5,959847	6,293028	5,944929	6,278154	6,065934667	-0,21221933	0,863208315	---
17205545	7,365367	7,022128	6,92014	7,367791	6,15688	7,146443	7,102545	6,890371333	-0,21217367	0,863235639	---
17413770	8,829187	8,834288	8,761115	8,52397	8,513441	8,750723	8,8081967	8,596044667	-0,212152	0,863248603	Tmod1
17235831	7,486348	7,596754	7,239953	7,303473	6,924078	7,459053	7,4410183	7,228868	-0,21215033	0,863249601	Gm10778 // Gm10778
17486342	3,557016	3,552576	3,471637	3,21794	3,334547	3,392568	3,5270763	3,315018333	-0,212058	0,863304851	Vmn2r37

17367036	10,13229	10,1668	10,16003	9,940228	9,93539	9,947431	10,15304	9,941016333	-0,21202367	0,863325396	Pter
17252712	3,401767	3,55795	2,956646	2,946398	3,25667	3,077281	3,3054543	3,093449667	-0,21200467	0,863336766	Zfp735
17355844	4,770976	4,346232	4,172538	3,858461	4,413641	4,381765	4,4299153	4,217955667	-0,21195967	0,863363695	---
17344208	6,202534	5,962781	6,053219	5,949275	5,879211	5,754651	6,0728447	5,861045667	-0,211799	0,86345985	Ly6g6f
17535143	3,842667	2,906405	3,462265	3,004396	3,1723	3,399295	3,403779	3,191997	-0,211782	0,863470024	C030023E24Rik
17403309	7,208068	6,722178	7,154401	7,036738	6,934293	6,478323	7,0282157	6,816451333	-0,21176433	0,863480598	---
17330705	4,836015	4,58186	4,479829	4,238634	4,505579	4,518199	4,632568	4,420804	-0,211764	0,863480798	---
17500707	2,962901	3,189692	3,123703	2,87714	2,818645	2,94529	3,0920987	2,880358333	-0,21174033	0,863494963	4930529F22Rik
17210217	7,364925	7,131423	7,46828	7,44646	7,171131	6,711925	7,3215427	7,109838667	-0,211704	0,863516709	---
17202143	3,175614	3,442378	3,42679	2,907078	3,328249	3,17438	3,3482607	3,136569	-0,21169167	0,863524092	---
17312661	4,578285	4,365726	4,509374	4,172843	4,197143	4,448422	4,4844617	4,272802667	-0,211659	0,863543644	Apol10a
17548219	6,792108	6,413687	6,354375	6,049876	6,307242	6,568295	6,5200567	6,308471	-0,21158567	0,86358754	Sord // Sord
17280742	4,174567	4,394643	4,075546	3,91621	4,139087	3,954755	4,2149187	4,003350667	-0,211568	0,863598115	9130015A21Rik
17439303	5,443515	5,749681	6,087771	5,397417	5,820958	5,427919	5,7603223	5,548764667	-0,21155767	0,863604301	---
17411869	3,787848	3,306249	3,603624	3,461702	3,329037	3,272535	3,565907	3,354424667	-0,21148233	0,863649397	---
17206433	3,724179	4,138939	4,903072	4,434062	3,554259	4,143458	4,2553967	4,043926333	-0,21147033	0,863656581	---
17520167	9,753941	10,1644	9,907317	9,702931	9,728126	9,760201	9,941886	9,730419333	-0,21146667	0,863658776	---
17529555	9,753941	10,1644	9,907317	9,702931	9,728126	9,760201	9,941886	9,730419333	-0,21146667	0,863658776	---
17448880	5,238832	5,481261	5,577317	5,205997	5,404084	5,052985	5,43247	5,221022	-0,211448	0,86366995	Lnx1
17337974	7,440631	7,749834	7,627654	7,2798	7,408473	7,495649	7,6060397	7,394640667	-0,211399	0,863699285	Mrps18a
17449636	9,475741	9,842978	9,447385	9,138771	9,524712	9,468459	9,5887013	9,377314	-0,21138733	0,863706269	---
17204877	4,323967	4,154079	5,294123	4,741345	4,28117	4,115548	4,590723	4,379354333	-0,21136867	0,863717445	---
17205717	6,282105	6,204127	5,954995	5,844186	5,303429	6,659531	6,1470757	5,935715333	-0,21136033	0,863722434	---
17471850	5,578945	5,484523	5,554462	5,319432	5,265734	5,398737	5,53931	5,327967667	-0,21134233	0,86373321	Styk1
17239874	5,820813	5,429937	5,696066	5,527875	5,348029	5,436962	5,6489387	5,437622	-0,21131667	0,863748577	---
17333755	3,72183	3,77254	3,676993	3,565381	3,486714	3,485405	3,7237877	3,5125	-0,21128767	0,863765939	Vmn2r92
17285056	9,396582	9,544119	9,533376	9,416214	9,182865	9,241373	9,491359	9,280150667	-0,21120833	0,863813439	Idi1
17312746	6,565117	6,510315	6,328765	6,404852	6,036207	6,329555	6,4680657	6,256871333	-0,21119433	0,863821821	Kctd17
17551099	5,066198	5,438526	5,538364	4,981109	4,656251	5,772251	5,347696	5,136537	-0,211159	0,863842978	---
17203065	6,567693	6,340255	7,062173	6,411035	6,645022	6,28063	6,656707	6,445562333	-0,21114467	0,86385156	---
17544814	5,286528	5,123073	4,919288	4,784591	4,936042	4,974884	5,1096297	4,898505667	-0,211124	0,863863935	---
17216554	4,93135	4,792922	4,005573	4,317244	4,413589	4,365911	4,576615	4,365581333	-0,21103367	0,863918027	---
17474748	3,479136	3,466628	3,125909	3,120795	3,19525	3,12254	3,3572243	3,146195	-0,21102933	0,863920622	Vmn1r125
17204155	4,818656	5,028524	5,781799	4,78861	4,949223	5,258137	5,2096597	4,998656667	-0,211003	0,863936391	---
17498673	6,421069	6,502742	6,345551	6,133804	6,122467	6,380122	6,4231207	6,212131	-0,21098967	0,863944375	Camsap3
17341709	2,65059	2,708214	4,19484	2,787575	2,962985	3,170196	3,184548	2,973585333	-0,21096267	0,863960544	---
17270617	4,920647	4,762329	4,828316	4,562511	4,65555	4,660488	4,8370973	4,626183	-0,21091433	0,863989489	Gm10844
17548180	4,196518	4,220066	4,208836	3,961101	4,205211	3,826488	4,2084733	3,9976	-0,21087333	0,864014043	LOC100861963
17326386	5,435606	5,592883	5,552336	5,43376	5,230272	5,284189	5,5269417	5,316073667	-0,210868	0,864017237	Gm16892
17417869	4,46869	4,426212	4,397601	4,280413	4,249172	4,130353	4,4308343	4,219979333	-0,210855	0,864025023	AU022252 // AU022252
17336738	8,156713	8,146466	8,136393	7,759521	8,11291	7,934652	8,146524	7,935694333	-0,21082967	0,864040195	Rdbp
17319980	3,979658	3,982593	4,184668	3,822195	3,74758	3,944838	4,048973	3,838204333	-0,21076867	0,864076729	---
17450641	5,257764	4,948883	5,039325	4,708225	4,902564	5,003121	5,0819907	4,871303333	-0,21068733	0,864125444	Lpcat2b
17201413	6,919214	6,815122	7,01369	7,029063	6,627199	6,459754	6,9160087	6,705338667	-0,21067	0,864135826	---
17324762	9,380367	9,435282	9,357172	9,166563	9,172302	9,20197	9,3909403	9,180278333	-0,210662	0,864140618	Rnf168
17458514	7,812433	7,658641	7,251686	7,509141	7,339088	7,24266	7,5742533	7,363629667	-0,21062367	0,864163579	Npy
17269516	6,505491	6,495829	6,274491	6,178782	6,318542	6,146648	6,4252703	6,214657333	-0,210613	0,864169968	Klhl11
17442491	6,294876	6,125118	5,959686	6,021622	5,810736	5,915569	6,12656	5,915975667	-0,21058433	0,86418714	4930568K20Rik
17330150	5,961715	5,762474	6,178812	5,749805	5,780025	5,741431	5,967667	5,757087	-0,21058	0,864189735	---
17205275	6,994203	6,369503	6,940225	6,647735	6,64804	6,376417	6,767977	6,557397333	-0,21057967	0,864189935	---
17209465	6,994203	6,369503	6,940225	6,647735	6,64804	6,376417	6,767977	6,557397333	-0,21057967	0,864189935	---
17229556	9,570206	9,585413	9,557155	9,488873	9,4977	9,094596	9,5709247	9,360389667	-0,210535	0,864216691	Atf6
17546333	3,877629	4,750164	4,32559	3,871572	4,576631	3,873655	4,3177943	4,107286	-0,21050833	0,864232665	Rbmy1a1
17332334	6,980776	7,304229	7,020663	6,305504	7,150981	7,217707	7,1018893	6,891397333	-0,210492	0,86424245	---
17408052	9,159168	9,180348	9,196021	8,912172	9,024925	8,967136	9,1785123	8,968077667	-0,21043467	0,864276796	Terc
17459709	3,86011	3,45971	4,166038	3,630742	3,575272	3,648632	3,8286193	3,618215333	-0,210404	0,864295168	---
17482626	9,508876	9,405745	9,274549	9,078996	9,252329	9,226851	9,39639	9,186058667	-0,21033133	0,864338702	Mettl9
17549468	4,223601	4,074771	4,153682	3,957947	3,748744	4,114488	4,1506847	3,940393	-0,21029167	0,864362467	---
17395928	5,878625	6,389348	6,22217	5,916369	5,883444	6,059675	6,163381	5,953162667	-0,21021833	0,864406405	Sox18
17215291	5,753153	5,600305	5,879846	5,324511	5,596119	5,682153	5,7444347	5,534261	-0,21017367	0,864433168	3110079O15Rik
17262174	9,617322	9,709723	9,816758	9,471975	9,521659	9,519774	9,714601	9,504469333	-0,21013167	0,864458333	Gnb2l1 // Gnb2l1
17453406	6,576519	6,312238	6,683907	6,228291	6,433668	6,280324	6,5242213	6,314094333	-0,210127	0,86446113	Lat2
17489547	2,876383	2,897119	3,05858	2,797616	2,719369	2,684838	2,9440273	2,733941	-0,21008633	0,864485497	Gm6725
17448099	5,593719	5,493699	5,708333	5,472544	5,341297	5,351686	5,5985837	5,388509	-0,21007467	0,864492488	5033403H07Rik
17233916	5,403831	5,410769	5,293126	5,168786	5,164357	5,144399	5,369242	5,159180667	-0,21006133	0,864500478	---
17200677	4,676839	5,049684	4,506328	4,911534	4,199418	4,491888	4,7442837	4,53428	-0,21000367	0,864535034	---
17408289	5,084926	5,592495	5,255861	5,323526	4,619689	5,360338	5,311094	5,101184333	-0,20990967	0,864591365	Gm4450
17248017	3,169005	3,216461	3,059019	2,764713	2,964013	3,086085	3,1481617	2,938270333	-0,20989133	0,864602352	---
17449125	3,393071	3,123178	3,189692	3,021422	3,168997	2,885962	3,2353137	3,025460333	-0,20985333	0,864625126	---
17412402	5,007098	4,833003	5,070888	4,588782	4,847548	4,845102	4,9703297	4,760477333	-0,20985233	0,864625725	---
17299261	5,142363	5,014211	5,030765	4,857256	4,603803	5,096898	5,0624463	4,852652333	-0,209794	0,864660686	---
17202095	5,297414	5,699203	5,135428	5,542022	5,232384	4,728362	5,3773483	5,167589333	-0,209759	0,864681663	---
17357799	4,129247	3,540527	4,072701	3,996473	3,364066	3,752729	3,9141583	3,704422667	-0,20973567	0,864695648	Olfr1440
17528833	4,975341	5,087075	5,448482	4,91634	4,987824	4,977712	5,1702993	4,960625333	-0,209674	0,864732609	---
17430413	6,163705	5,786183	5,946558	5,820068	5,766013	5,681435	5,965482	5,755838667	-0,20964333	0,864750991	Lck
17317533	4,731556	4,625914	4,757356	4,53417	4,518233	4,433496	4,704942	4,495299667	-0,20964233	0,86475159	Adcy8
17550062	6,529623	5,85814	6,338992	6,288595	5,698715	6,110666	6,2422517	6,032658667	-0,209593	0,864781161	---
17509359	11,14818	11,85447	11,43845	11,244	11,22801	11,34042	11,480367	11,27081	-0,20955667	0,86480294	---
17207997	6,74568	6,267233	6,569584	6,562576	6,339075	6,052274	6,527499	6,317975	-0,209524	0,864822522	---
17207321	5,920055	6,026038	6,242171	6,258795	5,786872	5,514026	6,0627547	5,853231	-0,20952367	0,864822722	---
17448756	7,429241	7,737113	8,274673	7,577593	7,580109	7,654792	7,8136757	7,604164667	-0,209511	0,864830315	---
17372906	5,197635	5,189586	4,85353	4,85417	4,819595	4,938643	5,0802503	4,870802667	-0,20944767	0,864868281	Olfr1184
17497633	6,083602	6,286464	6,307971	6,160204	5,853228	6,036363	6,2260123	6,016598333	-0,209414	0,864888464	Olfr522
17498015	7,470422	7,370729	7,474259	7,201435	7,258294	7,227486	7,43847	7,229071667	-0,20939833	0,864897856	Tollip
17331225	3,625397	3,414036	3,229408	3,330365	3,106638	3,203648	3,422947	3,213550333	-0,20939667	0,864898855	Olfr175-ps1
17342009	8,049589	8,254206	8,210485	7,878394	8,267693	7,74006	8,1714267	7,962049	-0,20937767	0,864910246	---
17468438	3,896514	4,003558	3,742741	3,750928	3,556942	3,707082	3,8809377	3,671650667	-0,209287	0,864964603	Nat8
17331245	3,070189	2,972967	2,87934	2,681323	2,86802	2,745321	2,9741653	2,764888	-0,20927733	0,864970399	Olfr190
17456886	5,108337	5,258871	5,332	5,337735	4,730712	5,003032	5,2330693	5,023826333	-0,209243	0,864990984	Cpa4
17323822	3,820991	3,627349	4,419836	3,710977	4,01494	3,514701	3,9560587	3,746872667	-0,209186	0,86502516	---
17525498	3,566895	3,502781	3,709463	3,397631	3,553077	3,201034	3,5930463	3,383914	-0,20913233	0,865057338	1700027I24Rik
17550456	5,841504	6,175655	5,865636	5,662831	5,863975	5,72865	5,9609317	5,751818667	-0,209113	0,865068931	---
17413706	5,395911	5,395496	5,564972	5,251476	5,185144	5,29244	5,4521263	5,24302	-0,20910633	0,865072928	E230008N13Rik
17206353	4,203918	4,875958	4,075818	4,138901	4,122449	4,267048	4,3852313	4,176132667	-0,20909867	0,865077525	---
17375538	6,892563	6,926733	6,846464	6,592033	6,409579	7,036862	6,8885867	6,679491333	-0,20909533	0,865079524	Sqrdl
17350349	3,703109	3,33796	3,687117	3,517631	3,305491	3,277975	3,576062	3,367032333	-0,20902967	0,865118901	---
17402517	6,921122	6,677439	7,042809	6,70425	6,281869	7,028312	6,8804567	6,671477	-0,20897967	0,865148884	---
17207403	5,346596	5,658405	5,197633	5,148767	5,239249	5,187745	5,400878	5,191920333	-0,20895767	0,865162077	---
17519957	3,602896	3,382745	3,783988	3,494044	3,281174	3,367635	3,5898763	3,380951	-0,20892533	0,865181467	---
17509500	3,237764	3,195112	3,456593	3,066146	3,180363	3,016311	3,2964897	3,087606667	-0,208883	0,865206854	---
17448800	9,276563	9,224858	9,189055	8,847648	9,254292	8,961995	9,2301587	9,021311667	-0,208847	0,865228445	---
17495816	5,227924	5,209053	5,428119	5,157054	5,037028	5,044477	5,2883653	5,079519667	-0,20884567	0,865229244	---
17416540	7,383914	7,475306	7,603484	7,25447	7,37037	7,211328	7,487568	7,278722667	-0,20884533	0,865229444	Lrp8
17205863	8,134336	8,284394	8,256401	7,998938	7,893305	8,156368	8,2250437	8,016203667	-0,20884	0,865232643	---
17362024	5,451139	5,592494	5,551175	5,478687	4,933741	5,556057	5,5316027	5,322828333	-0,20877433	0,865272026	---
17200889	6,173803	5,553486	6,140286	5,812176	5,369499	6,059675	5,9558583	5,747116667	-0,20874167	0,865291618	---
17449816	4,02233	3,87816	4,146667	3,677423	4,029839	3,713853	4,015719	3,807038333	-0,20868067	0,865328205	Gm7081
17261959	3,951221	4,089983	3,995241	3,947768	3,94301	3,51964	4,0121483	3,803472667	-0,20867567	0,865331204	---
17482482	4,77698	4,719198	5,091156	4,560138	4,786828	4,614364	4,8624447	4,653776667	-0,208668	0,865335803	E130201H02Rik
17486859	7,164456	7,374304	6,90824	7,154225	6,82767	6,839197	7,149	6,940364	-0,208636	0,865354997	Gpr77
17458946	3,58264	3,493262	3,620339	3,128346	3,422571	3,51964	3,5654137	3,356852333	-0,20856133	0,865399785	Vmn1r11
17285879	5,345315	5,49718	5,502902	5,534302	4,991055	5,194378	5,4484657	5,239911667	-0,208554	0,865404183	Hist1h1a
17202077	5,347059	5,239141	5,69635	5,788826	4,790472	5,077632	5,4275167	5,218976667	-0,20854	0,865412581	---
17299792	3,863521	3,835792	4,03008	3,735119	3,821593	3,547214	3,9097977	3,701308667	-0,208489	0,865443175	---
17402020	4,592451	5,091265	4,765572	4,936868	4,348689	4,538339	4,8164293	4,607965333	-0,208464	0,865458172	---
17348410	7,979616	8,177022	8,058433	7,74722	7,89036	7,952159	8,0716903	7,863246333	-0,208444	0,86547017	Rbbp8
17432058	5,124189	5,660712	5,792875	5,12745	5,635038	5,190121	5,5259253	5,317536333	-0,208389	0,865503165	Padi1
17511359	8,193359	8,114625	8,112082	7,861362	7,995335	7,938551	8,140022	7,931749333	-0,20827267	0,865572958	4921524J17Rik
17510247	5,771141	6,018582	5,79082	5,785419	5,659243	5,511133	5,860181	5,651931667	-0,20824933	0,865586958	Rpl18a
17548479	6,51569	6,567639	6,664955	6,33706	6,373164	6,413423	6,5827613	6,374549	-0,20821233	0,865609157	Mrps17 // Mrps17
17298977	9,41631	9,412465	9,156569	9,100638	9,135582	9,124618	9,328448	9,120279333	-0,20816867	0,865635357	Mbl1
17551103	4,575161	4,716516	4,883667	4,21198	4,62632	4,712608	4,7251147	4,516969333	-0,20814533	0,865649358	---
17207201	3,33824	3,869812	4,325194	3,455605	3,555486	3,897773	3,8444153	3,636288	-0,20812733	0,865660158	---
17268107	5,478673	5,003735	5,444605	5,120829	5,112221	5,069831	5,3090043	5,100960333	-0,208044	0,865710162	Sgca
17492078	7,165247	7,148812	7,096431	7,066035	6,878829	6,841576	7,13683	6,928813333	-0,20801667	0,865726564	Mctp2
17460974	6,25826	6,089541	6,304523	5,991448	6,248361	5,788468	6,2174413	6,009425667	-0,20801567	0,865727164	4930590J08Rik
17365625	4,721328	4,841022	4,862471	4,525119	4,62035	4,655361	4,8082737	4,600276667	-0,207997	0,865738366	Sorcs1
17341361	4,492468	4,448277	4,690391	4,36238	4,44021	4,204754	4,543712	4,335781333	-0,20793067	0,865778172	9330136K24Rik
17320854	4,652134	4,571831	4,731952	4,346616	4,356993	4,628547	4,6519723	4,444052	-0,20792033	0,865784373	---
17375080	4,968153	4,899654	5,297412	5,054664	4,724364	4,762453	5,055073	4,847160333	-0,20791267	0,865788974	Zfp106 // Zfp106
17489124	6,710394	6,718172	6,66939	6,605367	6,404242	6,464802	6,6993187	6,491470333	-0,20784833	0,865827583	BC053749
17203585	5,089762	4,639956	4,77613	4,074561	5,011877	4,795871	4,8352827	4,627436333	-0,20784633	0,865828783	---
17381143	5,668375	5,483038	5,456254	5,327662	5,361024	5,295488	5,535889	5,328058	-0,207831	0,865837985	Myt1
17308794	3,71815	3,684005	3,853216	3,368134	3,675977	3,587825	3,7517903	3,543978667	-0,20781167	0,865849589	D5Ertd579e // D5Ertd579e

17456855	4,975951	4,837284	5,16775	4,845863	4,949095	4,562637	4,9936617	4,785865	-0,20779667	0,865858591	---
17305787	3,877981	3,927584	4,206851	3,599198	3,826031	3,964057	4,0041387	3,796428667	-0,20771	0,865910607	---
17475342	8,703656	8,479338	8,618673	8,444388	8,368646	8,365637	8,6005557	8,392890333	-0,20766533	0,865937417	Tgfb1
17546345	3,849337	4,773998	4,322121	3,871572	4,585753	3,865177	4,315152	4,107500667	-0,20765133	0,86594582	Rbmy1a1
17461076	5,149053	4,832702	4,754857	4,865767	4,968378	4,279563	4,912204	4,704569333	-0,20763467	0,865955824	---
17406029	10,37045	10,50602	10,25763	10,08192	10,11883	10,31048	10,378033	10,17041	-0,20762333	0,865962626	Ube2d2a // Ube2d2a
17400539	9,220622	9,300858	9,157241	8,931592	9,146759	8,977568	9,2262403	9,018639667	-0,20760067	0,865976232	Hist2h2ab
17306425	7,946985	7,790843	8,166757	7,615938	7,818303	7,847554	7,968195	7,760598333	-0,20759667	0,865978633	---
17327035	5,58767	5,314475	5,355337	5,097764	5,093571	5,443438	5,4191607	5,211591	-0,20756967	0,86599484	Olig1
17204649	4,64841	4,554986	5,008893	4,424517	4,513859	4,65127	4,7374297	4,529882	-0,20754767	0,866008046	---
17200789	5,450367	5,721805	5,748961	5,560543	5,42027	5,317729	5,6403777	5,432847333	-0,20753033	0,86601845	---
17320068	7,031972	7,041005	7,426935	6,927396	6,85184	7,098138	7,1666373	6,959124667	-0,20751267	0,866029055	LOC100861793
17209129	3,869974	4,566364	5,098947	4,750039	3,852578	4,310267	4,5117617	4,304294667	-0,207467	0,866056469	---
17326752	3,361666	3,43426	3,158664	3,073442	2,958572	3,300214	3,3181967	3,110742667	-0,207454	0,866064273	---
17385022	3,131128	3,271268	2,784333	2,765201	2,922217	2,87711	3,062243	2,854842667	-0,20740033	0,86609649	---
17471528	5,377076	5,196981	5,332557	5,15905	5,015165	5,110227	5,3022047	5,094814	-0,20739067	0,866102293	Clec1a
17547704	7,237474	7,197945	7,366513	7,056844	7,098512	7,024457	7,2673107	7,059937667	-0,207373	0,866112899	Ppm1a // Ppm1a
17372771	3,456369	3,401726	3,450959	3,112446	3,265953	3,308583	3,4363513	3,228994	-0,20735733	0,866122305	Olfr1022
17208967	6,195055	5,436718	6,717203	6,247712	5,667603	5,81182	6,1163253	5,909045	-0,20728033	0,866168533	---
17301145	3,15845	3,316931	3,143986	2,995056	3,045982	2,956673	3,2064557	2,999237	-0,20721867	0,866205557	---
17467108	5,916409	5,940178	5,210292	5,206582	5,60707	5,631772	5,6889597	5,481808	-0,20715167	0,866245786	Lsm5
17248065	7,24043	7,037549	7,415329	7,699142	6,865687	6,507084	7,2311027	7,023971	-0,20713167	0,866257794	---
17449522	8,183331	8,118409	8,459505	7,811015	8,183331	8,145529	8,2537483	8,046625	-0,20712333	0,866262798	---
17456830	4,634898	4,530057	5,180432	4,287875	4,863759	4,572479	4,7817957	4,574704333	-0,20709133	0,866282013	BB283400
17200537	4,517678	5,247952	4,442359	4,178034	4,602825	4,805922	4,7359963	4,528927	-0,20706933	0,866295223	---
17494455	3,485685	3,53127	3,854012	3,433951	3,472931	3,342991	3,6236557	3,416624333	-0,20703133	0,866318041	Olfr672
17217838	2,80932	3,024084	3,008678	2,727533	2,781158	2,71233	2,9473607	2,740340333	-0,20702033	0,866324646	---
17311437	4,273438	4,419072	4,857147	4,477758	4,196974	4,253916	4,5165523	4,309549333	-0,207003	0,866335055	2310069G16Rik
17204683	4,417176	5,17797	4,543811	4,880208	4,32764	4,310109	4,7129857	4,505985667	-0,207	0,866336856	---
17449531	7,808568	7,057325	7,338559	7,175143	7,402488	7,005869	7,401484	7,1945	-0,206984	0,866346464	---
17339497	6,367314	6,614455	6,543163	6,484543	6,31861	6,101017	6,5083107	6,30139	-0,20692067	0,866384497	Snord92
17431166	6,129948	5,970299	6,150321	5,771485	5,889629	5,969032	6,0835227	5,876715333	-0,20680733	0,86645256	Lin28a
17202829	5,952133	5,714105	5,836952	5,747835	5,534084	5,600866	5,8343967	5,627595	-0,20680167	0,866455964	---
17538437	5,248847	5,304737	5,099168	5,023818	5,030665	4,977933	5,217584	5,010805333	-0,20677867	0,866469777	---
17425384	4,959042	5,025672	4,963343	4,921392	4,740025	4,66631	4,9826857	4,775909	-0,20677667	0,866470978	---
17209777	5,592349	5,946709	5,074967	4,785854	5,579206	5,628641	5,5380083	5,331233667	-0,20677467	0,866472179	---
17398404	3,319679	3,059643	2,916528	2,982892	2,760538	2,932115	3,0986167	2,891848333	-0,20676833	0,866475983	---
17362874	4,514835	4,379118	4,687249	4,39607	4,127802	4,43718	4,5270673	4,320350667	-0,20671667	0,866507014	Ms4a8a
17257871	3,674723	4,063286	3,78406	3,683434	3,544259	3,674259	3,8406897	3,633984	-0,20670567	0,866513621	---
17531749	5,512712	5,230544	4,948225	5,020164	5,103884	4,947417	5,2304937	5,023821667	-0,206672	0,866533842	---
17399899	4,581449	3,767465	4,036392	4,027502	3,749306	3,988534	4,1284353	3,921780667	-0,20665467	0,866544254	Lce1c
17200427	4,223606	4,621434	3,737954	4,560175	3,629245	3,773611	4,1943313	3,987677	-0,20665433	0,866544454	---
17476726	3,994832	3,565188	4,025272	3,677045	3,735472	3,553077	3,861764	3,655198	-0,206566	0,866597512	---
17318114	3,368125	3,153103	3,124764	2,923818	3,094017	3,008614	3,2153307	3,008816333	-0,20651433	0,866628548	Gm3142
17210001	4,7068	3,200484	4,439363	3,889383	3,863437	3,974365	4,115549	3,909061667	-0,20648733	0,866644767	---
17201339	5,089967	4,32314	4,244864	3,991434	4,666852	4,380332	4,552657	4,346206	-0,206451	0,866666593	---
17419543	6,159866	6,372256	6,213686	5,962624	6,117022	6,047039	6,2486027	6,042228333	-0,20637433	0,86671265	Cd164l2
17486942	4,753048	4,791516	4,774518	4,64631	4,51065	4,543052	4,7730273	4,566670667	-0,20635667	0,866723264	---
17294793	4,83679	4,630337	4,438988	4,383644	4,567395	4,336084	4,6353717	4,429041	-0,20633067	0,866738884	A830009L08Rik
17494462	5,014532	4,727459	4,929789	4,811052	4,782811	4,459219	4,8905933	4,684360667	-0,20623267	0,866797762	---
17269074	6,206991	5,977124	6,166247	5,824746	5,977015	5,930067	6,1167873	5,910609333	-0,206178	0,866830607	Krt12
17366055	7,307727	7,107164	6,705523	6,613031	6,733676	7,155219	7,040138	6,833975333	-0,20616267	0,86683982	Rab11fip2
17418357	7,123488	7,288787	7,164396	6,916214	6,85502	7,187049	7,1922237	6,986094333	-0,20612933	0,866859849	Yrdc
17483420	8,172537	8,095016	8,295886	8,104441	7,925227	7,915392	8,187813	7,981686667	-0,20612633	0,866861651	Ctf1
17206193	7,037271	7,26152	6,919327	6,571525	6,947042	7,08118	7,072706	6,866582333	-0,20612367	0,866863254	---
17299838	6,193021	5,512735	5,661364	5,707798	5,686006	5,355237	5,78904	5,583013667	-0,20602633	0,86692174	---
17275830	6,656394	7,314641	6,612279	6,481481	6,867828	6,616073	6,8611047	6,655127333	-0,20597733	0,866951184	---
17299013	4,436051	4,599754	4,919313	4,434738	4,331465	4,57112	4,651706	4,445774333	-0,20593167	0,866978627	---
17428192	8,166567	8,055644	8,001025	7,842314	7,845571	7,91766	8,074412	7,868515	-0,205897	0,86699946	Rnf11
17533177	4,757973	4,360614	4,630033	4,633067	4,209577	4,288298	4,5828733	4,376980667	-0,20589267	0,867002064	Gm5751
17388999	6,168705	6,233621	6,255901	5,894355	6,023188	6,123074	6,219409	6,013539	-0,20587	0,867015686	Prrg4
17462970	4,514117	4,813158	4,411764	4,519005	4,343704	4,258826	4,5796797	4,373845	-0,20583467	0,867036921	A230083G16Rik
17225888	8,856528	8,792913	8,593552	8,437446	8,573246	8,614812	8,7476643	8,541834667	-0,20582967	0,867039925	D1Ertd622e
17240965	5,022376	5,113212	4,966159	4,750536	4,953356	4,78048	5,0339157	4,828124	-0,20579167	0,867062763	Gm4981
17205095	5,155524	4,464501	4,732864	5,276215	4,829663	3,62967	4,7842963	4,578516	-0,20578033	0,867069575	---
17209285	5,155524	4,464501	4,732864	5,276215	4,829663	3,62967	4,7842963	4,578516	-0,20578033	0,867069575	---
17417480	6,22128	6,170158	6,185145	6,034318	5,940696	5,984316	6,1921943	5,986443333	-0,205751	0,867087204	Prdx1
17401357	8,319183	8,488597	8,172106	8,000916	8,274432	8,087341	8,3266287	8,120896333	-0,20573233	0,867098423	Rhoc
17459902	8,665231	8,762584	9,136967	8,472268	8,896448	8,578899	8,8549273	8,649205	-0,20572233	0,867104434	---
17509758	5,14278	5,326344	5,44709	4,814701	5,215091	5,269266	5,3054047	5,099686	-0,20571867	0,867106638	Csgalnact1
17326794	5,001169	4,466862	4,868336	4,581534	4,46385	4,673887	4,778789	4,573090333	-0,20569867	0,867118658	Mir155
17244022	4,075066	4,408241	3,961313	4,049248	3,870397	3,907921	4,1482067	3,942522	-0,20568467	0,867127073	Ano4
17248512	9,052741	9,228774	8,897798	8,890093	8,880395	8,791813	9,059771	8,854100333	-0,20567067	0,867135488	Nudcd2
17425813	5,142794	4,002066	4,463116	4,384077	4,217094	4,389823	4,535992	4,330331333	-0,20566067	0,867141498	---
17459258	4,799594	4,94141	4,8319	4,747249	4,436647	4,772114	4,8576347	4,652003333	-0,20563133	0,867159129	---
17414112	5,034409	5,304472	4,715169	4,677293	4,777287	4,982644	5,0180167	4,812408	-0,20560867	0,867172754	---
17534869	3,023343	3,280655	3,165004	2,939525	2,939525	2,973156	3,156334	2,950735333	-0,20559867	0,867178764	Gm14623
17206387	3,725321	4,752242	4,530323	3,978235	4,206479	4,206479	4,335962	4,130397667	-0,20556433	0,867199402	---
17303730	4,202533	4,369783	4,197175	4,015623	3,962903	4,174284	4,256497	4,050936667	-0,20556033	0,867201806	---
17200855	6,90314	6,565117	7,124347	6,856178	6,76184	6,357947	6,8642013	6,658655	-0,20554633	0,867210222	---
17338941	5,359283	5,219567	5,374644	5,260525	4,877192	5,199343	5,3178313	5,112353333	-0,205478	0,867251298	---
17379482	6,513221	6,279785	6,488725	6,311706	6,050996	6,302622	6,4272437	6,221774667	-0,205469	0,867256708	Wfdc10
17263218	4,343398	4,272226	4,373186	4,107397	4,182676	4,082364	4,3296033	4,124145667	-0,20545767	0,867263521	Hist3h2bb-ps
17488078	3,267848	3,481913	3,485844	3,133654	3,252433	3,233192	3,4118683	3,206426333	-0,205442	0,867272939	Nlrp9c
17207529	3,27729	2,8643	3,838879	3,057232	2,795373	3,511695	3,326823	3,121433333	-0,20538967	0,8673044	---
17484477	6,231899	5,968326	6,112676	6,010453	5,695896	5,990401	6,1043003	5,898916667	-0,20538367	0,867308007	Prap1
17496664	7,366199	6,937121	7,325831	6,850222	7,196127	6,966668	7,209717	7,004339	-0,205378	0,867311413	Zfp747
17254065	4,145587	4,032943	4,615364	4,087709	4,101653	3,988443	4,2646313	4,059268333	-0,205363	0,867320431	Ccl8
17344694	5,621269	6,031535	5,939923	5,535283	5,912994	5,528437	5,8642423	5,658904667	-0,20533767	0,867335661	---
17202951	5,373898	5,659496	5,92981	5,826201	5,33881	5,182183	5,6544013	5,449064667	-0,20533667	0,867336262	---
17202169	6,798389	7,162237	7,414681	7,134316	6,756588	6,868419	7,1251023	6,919774333	-0,205328	0,867341473	---
17230331	5,652375	4,80265	5,302172	5,746828	4,769485	4,624935	5,252399	5,047082667	-0,20531633	0,867348487	Tfb2m
17242726	5,288179	5,477156	5,541208	5,198329	5,14708	5,345295	5,4355143	5,230234667	-0,20527967	0,867370531	---
17202855	7,07852	6,769436	6,50472	7,418215	5,939565	6,379212	6,7842253	6,578997333	-0,205228	0,867401594	---
17287245	4,230262	4,124417	4,041255	3,853953	3,757014	4,169285	4,131978	3,926750667	-0,20522733	0,867401995	---
17338545	4,39288	4,331806	4,454722	4,4025	3,816578	4,344682	4,393136	4,18792	-0,205216	0,867408809	Kcnh8
17447504	6,470074	6,771798	6,527497	6,317933	6,457067	6,378733	6,5897897	6,384577667	-0,205212	0,867411214	---
17382139	3,452827	3,406778	3,240827	3,264209	2,971992	3,248662	3,3668107	3,161621	-0,20518967	0,867424642	Enkur
17460933	8,059584	8,464505	8,06096	7,928078	8,016648	8,024896	8,1950163	7,989874	-0,20514233	0,867453102	Lsm3
17374172	3,995419	3,722495	3,791989	3,587727	3,937421	3,36957	3,8366343	3,631572667	-0,20506167	0,867501606	Olfr1279
17260869	8,605417	8,804242	8,742159	8,672173	8,472735	8,391993	8,7172727	8,512300333	-0,20497233	0,867555324	Slc1a4
17323074	8,853151	9,197449	8,858194	8,665304	8,718447	8,910194	8,969598	8,764648333	-0,20494967	0,867568955	---
17427441	5,702696	5,297956	5,413334	5,340921	5,200514	5,257705	5,4713287	5,26638	-0,20494867	0,867569556	---
17348455	9,549304	10,28548	9,980253	9,395753	9,657291	10,14723	9,9383457	9,733424667	-0,204921	0,867586194	---
17285620	8,498951	8,678384	8,693719	8,338462	8,423076	8,494823	8,6236847	8,418787	-0,20489767	0,867600226	Trim27
17320686	4,10381	4,313371	4,128678	4,166229	3,888219	3,876788	4,181953	3,977078667	-0,20487433	0,867614258	---
17377551	4,056314	3,856138	4,110835	3,951016	3,562409	3,895455	4,0077623	3,80296	-0,20480233	0,867657559	---
17257066	5,839297	6,478787	5,403666	4,861149	5,805723	6,440543	5,90725	5,702471667	-0,20477833	0,867671993	---
17267203	4,582283	3,920331	3,908595	4,222617	3,811589	3,762746	4,1370697	3,932317333	-0,20475233	0,86768763	LOC629393
17267629	4,355354	4,572881	4,654476	4,161033	4,426515	4,381016	4,5275703	4,322854667	-0,20471567	0,867709683	---
17481165	4,771729	4,773644	4,39649	4,705771	4,360777	4,261235	4,6472877	4,442594333	-0,20469333	0,867723115	Olfr576
17333850	3,603196	3,881538	3,774522	3,66233	3,410156	3,572756	3,7530853	3,548414	-0,20467133	0,867736347	Vmn1r229
17548156	7,596556	7,327248	7,251032	7,211553	7,097034	7,252445	7,391612	7,187010667	-0,20460133	0,867778451	2310047B19Rik // 2310047B19Rik
17214027	5,632893	5,762533	5,919128	5,479775	5,65601	5,565173	5,771518	5,566986	-0,204532	0,867820156	B930094L07Rik
17285536	8,055898	7,75749	8,304972	8,150554	7,406773	7,947506	8,0394533	7,834944333	-0,204509	0,867833991	---
17478273	3,486244	3,363826	3,083924	3,169049	3,054649	3,09699	3,3113313	3,106896	-0,20443533	0,867878306	Mrgprb8
17284706	4,673447	4,493174	4,603473	4,45431	4,287618	4,415063	4,5900313	4,385663667	-0,20436767	0,867919013	Gm5441
17495847	5,096813	4,934398	4,719902	4,643211	4,660035	4,834888	4,9170377	4,712711333	-0,20432633	0,867943879	4933432K03Rik
17263142	4,238321	3,941536	4,276924	3,983675	4,021909	3,83826	4,1522603	3,947948	-0,20431233	0,867952302	4930504O13Rik
17341405	7,360908	7,818112	7,45653	7,118647	7,564367	7,339669	7,5451833	7,340894333	-0,204289	0,86796634	Gm7072
17342010	7,402452	6,981515	7,098254	6,903726	7,037792	6,927891	7,1607403	6,956469667	-0,20427067	0,867977369	---
17357636	5,455766	4,788934	5,072176	4,633287	4,995998	5,074803	5,1056253	4,901362667	-0,20426267	0,867982183	4930526L06Rik // 4930526L06Rik
17204885	4,092016	5,322739	5,579826	4,588475	5,265607	4,527756	4,9981937	4,793946	-0,20424767	0,867991207	---
17534892	5,9121	6,323222	5,4863	5,669299	5,648418	5,791265	5,9072073	5,702994	-0,20421333	0,868011864	Zfp449
17370348	4,05538	3,257684	3,414429	3,403273	3,342907	3,368964	3,575831	3,371714667	-0,20411633	0,868070227	Olfr50
17225969	5,287344	5,315053	4,932529	5,010277	4,893365	5,019001	5,1783087	4,974214333	-0,20409433	0,868083464	B230216N24Rik
17287185	8,38365	8,619553	8,16416	8,124622	8,198084	8,232375	8,389121	8,185027	-0,204094	0,868083665	Nol8
17201841	3,832131	3,580801	3,421447	3,604575	3,159187	3,458644	3,6114597	3,407468667	-0,203991	0,868145643	---
17493687	5,149208	4,680468	4,997171	5,307683	4,250163	4,657057	4,9422823	4,738301	-0,20398133	0,86815146	---
17323076	9,06963	9,060031	8,931809	8,818721	8,893508	8,737314	9,02049	8,816514333	-0,20397567	0,86815487	---
17429844	3,664654	4,095672	4,177415	3,827298	3,786778	3,711744	3,979247	3,775273333	-0,20397367	0,868156074	---
17290852	6,298851	6,439744	6,491917	6,404111	5,974602	6,240102	6,4101707	6,206271667	-0,203899	0,868201006	---
17546963	4,001677	4,685966	4,434804	3,968806	4,576835	3,965323	4,374149	4,170321333	-0,20382767	0,868243935	Rbmy1a1
17233694	6,627945	6,252769	6,424952	6,298681	6,187315	6,208581	6,435222	6,231525667	-0,20369633	0,868322978	Prf1

17207551	6,69306	6,289706	6,439426	6,051558	6,525944	6,233751	6,474064	6,270417667	-0,20364633	0,868353072	---
17347435	8,398518	8,471865	8,167469	8,096767	8,205966	8,124245	8,3459507	8,142326	-0,20362467	0,868366113	Cdc42ep3
17490391	10,12427	10,16211	10,33064	10,09224	10,10426	9,80974	10,205673	10,00208	-0,20359333	0,868384973	Atf5
17201639	3,233192	3,815353	4,065765	3,735817	3,270892	3,496833	3,70477	3,501180667	-0,20358933	0,868387381	---
17314691	10,82536	11,188	11,06001	10,79716	10,90858	10,75694	11,024457	10,82089333	-0,20356333	0,868403031	---
17539400	7,068864	6,820653	7,283852	6,711096	7,064775	6,786873	7,0577897	6,854248	-0,20354167	0,868416073	---
17465270	3,761837	4,175149	3,976839	3,870274	3,452954	3,980048	3,971275	3,767758667	-0,20351633	0,868431322	---
17200279	3,626109	3,402475	3,467089	2,96154	3,234893	3,688723	3,4985577	3,295052	-0,20350567	0,868437743	---
17516978	7,479823	7,87936	7,392432	7,60899	7,236239	7,296451	7,5838717	7,38056	-0,20331167	0,86855453	Fam55d
17262355	3,319602	3,447491	3,786403	3,330683	3,151585	3,461362	3,517832	3,314543333	-0,20328867	0,868568377	Mir804
17339499	7,922049	7,668969	7,817214	7,85216	7,61837	7,327837	7,802744	7,599455667	-0,20328833	0,868568578	Snord53
17240575	3,499532	2,935966	3,314175	3,033667	3,038676	3,067478	3,249891	3,046607	-0,203284	0,868571187	---
17481344	4,512692	4,323103	4,411998	4,334266	4,042264	4,261706	4,415931	4,212745333	-0,20318567	0,86863039	Olfr676
17239506	5,199128	5,240536	4,889417	4,829981	4,89579	4,993774	5,1096937	4,906515	-0,20317867	0,868634605	Ect2l
17308345	4,097056	4,03695	4,529497	4,037419	4,074957	3,941622	4,2211677	4,017999333	-0,20316833	0,868640826	---
17250517	3,872334	3,693547	3,682073	3,32267	3,695774	3,620139	3,749318	3,546194333	-0,20312367	0,86866772	---
17229192	4,742904	4,618418	4,222375	4,172784	4,455621	4,345943	4,527899	4,324782667	-0,20311633	0,868672136	4930568G15Rik
17525955	10,95025	10,9225	10,78677	10,77225	10,44634	10,83174	10,886507	10,68344333	-0,20306333	0,868704049	Sc5d
17370373	3,941688	4,070078	4,091093	3,93085	3,682506	3,880529	4,0342863	3,831295	-0,20299133	0,868747404	Olfr367-ps
17519279	7,37333	7,502458	7,444804	7,290818	7,284237	7,136884	7,4401973	7,237313	-0,20288433	0,868811838	2310009A05Rik
17329380	9,819496	9,852411	9,744119	9,525108	9,583815	9,698655	9,805342	9,602526	-0,202816	0,868852991	Rfc4
17326003	5,09982	4,671571	4,753882	4,84472	4,675982	4,396317	4,8417577	4,639006333	-0,20275133	0,868891937	---
17410239	8,400811	8,496237	8,387537	8,288986	8,126423	8,260975	8,428195	8,225461333	-0,20273367	0,868902577	5730508B09Rik
17333893	3,739704	3,647338	4,117317	3,744435	3,548435	3,603423	3,8347863	3,632097667	-0,20268867	0,86892968	Vmn1r234
17458150	6,12256	6,301509	5,807647	5,824285	6,116	5,683366	6,0772387	5,874550333	-0,20268833	0,868929881	Zfp956
17217399	8,324455	8,291481	8,308074	7,881948	8,363482	8,070629	8,3080033	8,105353	-0,20265033	0,868952768	Chi3l1
17201159	7,903395	7,907708	7,99864	6,978724	8,028069	8,195168	7,936581	7,733987	-0,202594	0,868986699	---
17300149	6,203207	5,810148	5,818693	5,864592	5,470407	5,889334	5,944016	5,741444333	-0,20257167	0,869000151	---
17549272	5,756535	5,986273	5,858736	5,565622	5,591753	5,836527	5,8671813	5,664634	-0,20254733	0,869014808	---
17483696	6,784821	6,852791	6,628503	6,723833	6,221099	6,713756	6,7553717	6,552896	-0,20247567	0,869057978	Tgfb1i1
17240940	4,643289	3,796448	4,241489	4,196969	4,239345	3,637586	4,2270753	4,024633333	-0,202442	0,869078259	4930467K11Rik
17521603	6,146376	6,781333	6,434787	6,123746	6,412865	6,218698	6,4541653	6,251769667	-0,20239567	0,86910617	Gmppb
17228743	9,630915	9,802204	9,548223	9,366634	9,441677	9,565908	9,6604473	9,458073	-0,20237433	0,869119022	Cacybp
17206531	8,06881	7,242521	7,840389	7,672219	7,469476	7,40291	7,71724	7,514868333	-0,20237167	0,869120629	---
17454692	8,647584	8,211392	8,416538	8,194487	8,17083	8,303119	8,4251713	8,222812	-0,20235933	0,869128059	Mmd2
17236792	4,247432	4,104169	4,130743	3,953087	3,953173	3,969093	4,1607813	3,958451	-0,20233033	0,869145529	4930556N09Rik
17237805	3,635457	3,450695	3,642725	3,252418	3,381249	3,488247	3,5762923	3,373971333	-0,202321	0,869151152	---
17538002	4,642014	4,510142	4,270566	4,134639	4,516156	4,164978	4,4742407	4,271924333	-0,20231633	0,869153964	---
17277760	4,163517	4,118672	4,003324	3,906162	3,944858	3,827557	4,095171	3,892859	-0,202312	0,869156574	5430427M07Rik
17212968	4,029807	3,842691	4,043724	3,819107	3,773407	3,716791	3,972074	3,769768333	-0,20230567	0,86916039	---
17473854	4,225383	3,651848	3,346358	3,578711	3,694433	3,343632	3,7411963	3,538925333	-0,202271	0,869181275	Vmn1r89
17548287	7,554692	7,494697	7,391204	7,435122	7,09777	7,300933	7,4801977	7,277941667	-0,202256	0,869190312	Oaz2
17220792	4,732179	4,816761	4,612839	4,616569	4,379873	4,558666	4,720593	4,518369333	-0,20222367	0,869209793	---
17309345	6,519055	6,549628	6,415277	6,365343	6,448029	6,064118	6,4946533	6,292496667	-0,20215667	0,86925016	Gm4666 // Gm6219
17367458	4,168078	4,262858	4,545435	4,19876	3,940417	4,230757	4,325457	4,123311333	-0,20214567	0,869256788	Gpr158
17548422	6,585726	6,998138	6,562191	6,363094	6,544991	6,631567	6,7153517	6,513217333	-0,20213433	0,869263617	---
17476490	4,215682	4,052121	4,264275	3,826362	4,176731	3,922595	4,1773593	3,975229333	-0,20213	0,869266228	2200002J24Rik
17361099	7,340044	7,10341	7,259351	7,001358	6,982491	7,112624	7,2342683	7,032157667	-0,20211067	0,869277877	Aip
17337601	3,886575	3,496374	3,62118	3,446281	3,503864	3,447744	3,668043	3,465963	-0,20208	0,869296355	---
17283944	5,790874	6,009919	6,728717	5,69135	6,25746	5,974489	6,1765033	5,974433	-0,20207033	0,869302179	---
17544424	4,721962	4,46891	4,90836	4,479176	4,502986	4,510905	4,699744	4,497689	-0,202055	0,869311419	Nox1
17204373	3,128399	3,48921	3,272498	2,637997	3,525434	3,120535	3,2967023	3,094655333	-0,202047	0,869316239	---
17322026	4,699047	4,42758	4,609467	4,426034	4,297387	4,406544	4,578698	4,376655	-0,202043	0,869318649	Krt2
17387636	3,633107	3,651848	3,806628	3,586995	3,392642	3,506073	3,6971943	3,495236667	-0,20195767	0,86937007	Olfr1040
17324412	5,45753	5,648643	5,957923	5,387319	5,473655	5,597332	5,688032	5,486102	-0,20193	0,869386742	BC106179
17508605	4,264654	4,507299	4,182947	4,004766	4,268704	4,075691	4,3183	4,116387	-0,201913	0,869396986	---
17360731	6,72031	6,254736	6,821297	6,572637	6,334308	6,283802	6,598781	6,396915667	-0,20186533	0,869425712	Gfra1
17269139	4,112283	3,982026	4,170291	3,823493	3,992073	3,843464	4,0882	3,886343333	-0,20185667	0,869430935	Krtap1-5
17275069	10,15288	9,904222	10,04504	10,08768	9,697702	9,711348	10,034047	9,832243333	-0,201804	0,869462675	Agmo
17224736	3,03688	3,257726	3,127009	2,941418	2,884159	2,990634	3,1405383	2,938737	-0,20180133	0,869464282	---
17309542	4,322299	4,189391	4,535007	4,227718	3,868191	4,345438	4,348899	4,147115667	-0,20178333	0,86947513	B930095G15Rik // B930095G15Rik
17210805	5,184406	5,060063	4,963014	4,870219	4,647902	5,08423	5,069161	4,867450333	-0,20171067	0,869518925	---
17276881	4,453526	4,489795	4,982548	4,476024	4,451975	4,392892	4,6419563	4,440297	-0,20165933	0,869549865	---
17308083	6,062605	5,85071	5,700585	5,65169	5,76942	5,587842	5,8713	5,669650667	-0,20164933	0,869555892	Adamdec1
17511366	10,32328	10,29684	10,26155	9,999008	10,10365	10,17425	10,29389	10,09230267	-0,20158733	0,869593262	Dnaja2
17351837	8,956633	9,057611	8,94962	8,644276	8,863428	8,851464	8,9879547	8,786389333	-0,20156533	0,869606523	BC031181
17202211	3,130631	3,167349	3,123178	2,948291	2,948291	2,919897	3,140386	2,938826333	-0,20155967	0,869609938	---
17248249	6,943531	6,694505	6,970805	6,553118	6,760189	6,690968	6,8696137	6,668091667	-0,201522	0,869632643	Mpg
17200019	3,632055	3,608295	3,818329	3,566845	3,496596	3,390831	3,6862263	3,484757333	-0,201469	0,869664591	---
17286212	3,848416	3,89457	3,651848	3,710464	3,477963	3,602002	3,798278	3,596809667	-0,20146833	0,869664993	Prl4a1
17348396	5,685255	5,279529	5,668092	5,506235	5,388849	5,133512	5,544292	5,342865333	-0,20142667	0,86969011	---
17366780	6,560051	6,803337	6,552938	6,772697	6,048852	6,490538	6,6387753	6,437362333	-0,201413	0,869698349	---
17462139	5,185302	4,764935	4,744645	4,717407	4,84077	4,532547	4,898294	4,696908	-0,201386	0,869714625	Olfr213
17292800	7,211771	7,284529	7,183171	7,057948	6,952035	7,0654	7,2264903	7,025127667	-0,20136267	0,869728692	Uimc1
17366748	4,210197	3,939132	4,131294	3,761639	4,169473	3,745446	4,093541	3,892186	-0,201355	0,869733313	---
17206759	4,066516	5,168011	4,036402	4,244514	4,345553	4,076856	4,423643	4,222307667	-0,20133533	0,86974517	---
17275875	5,840006	5,774519	5,898533	5,591958	5,606924	5,710413	5,837686	5,636431667	-0,20125433	0,869794003	Lrr1
17448811	8,708009	7,849802	8,016002	7,955292	8,065309	7,949467	8,191271	7,990022667	-0,20124833	0,86979762	---
17258004	4,092058	3,832456	4,038494	3,850737	3,928193	3,580399	3,9876693	3,786443	-0,20122633	0,869810884	1700025D23Rik
17209631	5,799179	4,875833	6,105231	5,562357	4,921432	5,692802	5,5934143	5,392197	-0,20121733	0,86981631	---
17429873	2,864549	3,027118	3,08871	2,863161	2,743727	2,769921	2,993459	2,792269667	-0,20118933	0,869833192	---
17203577	3,033063	3,451477	3,582637	3,215321	3,016619	3,231682	3,3557257	3,154540667	-0,201185	0,869835805	---
17205991	5,720489	6,006909	6,413512	6,196632	5,811493	5,529237	6,04697	5,845787333	-0,20118267	0,869837211	---
17462764	6,231349	6,377163	6,321934	6,034742	6,23198	6,060181	6,3101487	6,108967667	-0,201181	0,869838216	Clec4a2
17487712	5,639787	5,2782	5,888403	5,435405	5,347023	5,420618	5,60213	5,401015333	-0,20111467	0,869878211	1700008P20Rik
17204091	5,764025	5,948685	5,411318	5,712655	5,381591	5,426529	5,7080093	5,506925	-0,20108433	0,869896501	---
17288234	5,475619	5,571678	5,279528	5,436648	4,968207	5,318798	5,442275	5,241217667	-0,20105733	0,869912781	Gm10324 // Gm10324 // Gm10324
17526347	4,20514	4,381313	4,150811	4,114782	4,035696	3,983767	4,2457547	4,044748333	-0,20100633	0,869943534	---
17384046	5,943024	5,657141	5,959777	5,71169	5,515695	5,729711	5,853314	5,652365333	-0,20094867	0,869978307	---
17548442	5,572518	5,882906	5,725621	5,530831	5,564415	5,48296	5,727015	5,526068667	-0,20094633	0,869979714	Gm6745
17476773	6,61361	6,709517	6,404166	6,280168	6,439041	6,40528	6,5757643	6,374829667	-0,20093467	0,86998675	Ccdc123
17471794	3,744872	3,543656	3,601099	3,571568	3,406265	3,309126	3,6298757	3,428986333	-0,20088933	0,870014087	Gm6590 // Gm6590
17371101	7,436659	7,585648	7,359694	7,18962	7,162402	7,427324	7,460667	7,259782	-0,200885	0,870016701	Tank
17246386	4,028472	3,190089	3,926276	3,448551	3,359862	3,73384	3,7149457	3,514084333	-0,20086133	0,870030973	Olfr764 // Olfr766
17532879	5,908686	5,739828	5,86567	5,667778	5,504591	5,739247	5,8380613	5,637205333	-0,200856	0,870034189	Syp
17247946	3,327119	3,280294	3,396137	3,118134	3,168075	3,114792	3,3345167	3,133667	-0,20084967	0,870038009	Mir217
17428217	7,760897	7,902659	7,826702	7,622602	7,623531	7,641696	7,830086	7,629276333	-0,20080967	0,870062132	Cdkn2c
17287814	4,854325	4,455681	4,83446	4,572742	4,532716	4,436621	4,714822	4,514026333	-0,20079567	0,870070575	Fbxl21
17403992	3,576248	3,312757	3,794998	3,329432	3,340961	3,411244	3,5613343	3,360545667	-0,20078867	0,870074796	---
17436659	4,360082	4,497274	4,917034	4,568637	4,191929	4,411461	4,5914633	4,390675667	-0,20078767	0,870075399	Grk4
17417223	9,737694	9,826504	8,837434	10,01284	8,647245	9,13921	9,4672107	9,266431667	-0,200779	0,870080626	Cyp4a10
17207329	5,839332	5,357491	5,906082	5,502561	5,845732	5,152345	5,7009683	5,500212667	-0,20075567	0,870094699	---
17276252	10,41334	10,6317	10,37208	10,09614	10,15613	10,56274	10,472373	10,27167	-0,20070333	0,870126262	---
17314624	7,036499	6,083952	6,74467	6,465878	6,438409	6,358758	6,621707	6,421015	-0,200692	0,870133097	Olfr279
17543363	3,793351	3,410705	3,578682	3,36151	3,535348	3,28383	3,594246	3,393562667	-0,20068333	0,870138324	Msn // Msn
17247373	4,930923	4,180632	4,758218	4,678161	4,163197	4,426514	4,6232577	4,422624	-0,20063367	0,87016828	---
17546082	3,592208	3,659532	3,921722	3,593265	3,511127	3,467235	3,7244873	3,523875667	-0,20061167	0,87018155	Gm8817
17461467	3,436021	3,726244	3,761437	3,522181	3,348442	3,451369	3,641234	3,440664	-0,20057	0,870206682	---
17489707	9,118918	9,276485	9,254238	9,139399	8,96939	8,939274	9,216547	9,016021	-0,200526	0,870233222	Cebpg
17471898	3,504884	3,491159	3,748859	3,387513	3,331836	3,424029	3,581634	3,381126	-0,200508	0,87024408	Gm4736
17501550	6,700749	7,19904	6,844371	6,766115	6,569572	6,806951	6,91472	6,714212667	-0,20050733	0,870244482	Naf1
17463332	4,899388	4,982133	5,141637	4,572208	4,994372	4,85516	5,0077193	4,807246667	-0,20047267	0,870265394	Fgf6
17344686	5,236532	5,669055	4,673534	4,978212	4,989226	5,010777	5,1930403	4,992738333	-0,200302	0,87036835	---
17481695	10,59753	10,68675	10,65828	10,54302	10,38795	10,41069	10,64752	10,44722	-0,2003	0,870369556	Ipo7
17249898	6,894139	7,138584	6,876706	6,744202	6,709291	6,855082	6,9698097	6,769525	-0,20028467	0,870378807	Galnt10
17424461	6,272297	6,53142	6,556769	6,489781	5,937735	6,332218	6,4534953	6,253244667	-0,20025067	0,870399319	Ccl21a
17324996	3,774464	3,865654	4,252525	3,535831	4,154312	3,601822	3,9642143	3,763988333	-0,200226	0,870414201	Mir1947
17473410	6,006637	5,936013	6,225534	5,911038	5,859189	5,797299	6,0560613	5,855842	-0,20021933	0,870418223	Zfp628
17204753	4,807295	5,038773	5,561665	5,12031	4,696356	4,99045	5,135911	4,935705333	-0,20020567	0,870426469	---
17315124	5,886668	5,448539	5,834464	5,616485	5,404542	5,548231	5,7232237	5,523086	-0,20013767	0,870467496	Ankrd33
17285706	7,843684	7,878941	8,163012	7,808747	7,72427	7,752307	7,961879	7,761774667	-0,20010433	0,870487609	Hist1h4m
17350171	4,728162	4,782358	4,562332	4,403993	4,51739	4,551221	4,6909507	4,490868	-0,20008267	0,870500682	Stk32a
17542913	4,160667	3,691063	3,68167	3,562652	3,636242	3,734343	3,8444667	3,644412333	-0,20005433	0,870517778	---
17445253	3,18062	3,21134	3,295615	2,931308	3,056481	3,099642	3,2291917	3,029143667	-0,200048	0,8705216	---
17320684	4,611874	4,429464	4,738235	4,367305	4,356458	4,455905	4,593191	4,393222667	-0,19996833	0,870569672	---
17514871	9,055571	9,370581	9,112909	8,912523	8,857905	9,168866	9,179687	8,979764667	-0,19992233	0,87059743	Chordc1
17301131	8,732967	8,989325	8,915773	8,679693	8,579128	8,779479	8,879355	8,679433333	-0,19992167	0,870597832	Rnaseh2b
17209609	3,840522	3,628391	4,353742	3,815353	3,492751	3,91483	3,940885	3,740978	-0,199907	0,870606683	---
17231963	6,786559	7,213782	7,187857	6,976411	6,708833	6,903233	7,0627327	6,862825667	-0,199907	0,870606683	Fam54a
17206963	6,434165	6,753902	5,986903	6,542599	6,051848	5,980805	6,3916567	6,191750667	-0,199906	0,870607287	---
17294664	7,461617	7,750022	7,657329	7,505992	7,209758	7,553509	7,6229893	7,423086333	-0,199903	0,870609097	Polr3g
17291852	3,565899	3,344619	3,123178	3,066066	2,981813	3,386231	3,3445653	3,144703333	-0,199862	0,870633839	---
17522803	7,091377	7,327911	7,512239	6,750841	7,324012	7,257174	7,310509	7,110675667	-0,19983333	0,870651139	---
17428797	8,545413	9,248999	8,949249	8,806014	8,701095	8,637071	8,9145537	8,714726667	-0,199827	0,870654961	Snord38a

17448543	6,104681	6,457428	6,134212	5,934673	6,059574	6,102868	6,232107	6,032371667	-0,19973533	0,870710283	Gnpda2
17295735	4,705606	4,376184	4,614892	4,450622	4,015901	4,630991	4,5655607	4,365838	-0,19972267	0,870717928	Mrps36
17483615	5,299962	5,220653	4,84868	4,860941	5,170954	4,738419	5,1230983	4,923438	-0,19966033	0,870755549	Itgax
17335204	6,532	6,63567	6,765346	6,525812	6,435752	6,37268	6,6443387	6,444748	-0,19959067	0,870797598	Anks1
17516365	12,70415	12,86589	12,74848	12,6188	12,49817	12,60285	12,77284	12,57327333	-0,19956667	0,870812084	Hspa8
17534569	7,753027	8,019998	7,893891	7,702495	7,606371	7,759365	7,888972	7,689410333	-0,19956167	0,870815102	Utp14a
17396890	3,381651	3,398531	3,145102	2,967901	3,206961	3,151808	3,308428	3,10889	-0,199538	0,870829388	---
17389802	7,558983	7,717049	7,534364	7,343103	7,375289	7,493459	7,6034653	7,403950333	-0,199515	0,870843271	Fam82a2
17397120	6,699131	6,742611	6,901754	6,56585	6,752937	6,426303	6,7811653	6,581696667	-0,19946867	0,870871239	Bbs12
17210447	2,858603	3,293091	2,815713	3,002228	2,778477	2,588442	2,9891357	2,789715667	-0,19942	0,870900617	---
17440954	7,088911	7,291765	7,104096	7,023669	6,999496	6,8634	7,1615907	6,962188333	-0,19940233	0,870911282	Mvk
17543529	3,875698	4,216114	4,666686	3,902419	4,106955	4,150986	4,2528327	4,053453333	-0,19937933	0,870925166	Slc7a3
17209691	4,865414	5,108599	4,855394	4,585675	4,575974	5,069735	4,9431357	4,743794667	-0,199341	0,870948308	---
17272537	5,329844	4,603145	5,226218	4,670664	4,926631	4,963906	5,053069	4,853733667	-0,19933533	0,870951729	---
17241530	3,278477	3,417567	3,381021	3,021513	3,272674	3,184881	3,3590217	3,159689333	-0,19933233	0,87095354	---
17447148	8,01693	7,434788	7,761952	7,356277	7,641606	7,617894	7,73789	7,538592333	-0,19929767	0,870974468	---
17210803	4,476424	4,564605	5,1267	5,125818	4,343821	4,100214	4,7225763	4,523284333	-0,199292	0,870977889	---
17501354	8,869973	9,136638	8,88705	8,811383	8,659345	8,825061	8,9645537	8,765263	-0,19929067	0,870978694	2700029M09Rik
17304514	6,111951	6,651803	6,201281	6,268328	5,956286	6,142564	6,3216783	6,122392667	-0,19928567	0,870981713	Snord19
17424042	6,691586	7,009644	6,695401	6,449356	6,536602	6,812823	6,798877	6,599593667	-0,19928333	0,870983121	Aptx
17354589	4,413828	5,131736	4,475417	4,33338	4,504613	4,58522	4,6736603	4,474404333	-0,199256	0,870999623	Gm4841
17425389	7,411842	7,489511	7,346162	7,038245	7,222603	7,388988	7,4158383	7,216612	-0,19922633	0,871017534	---
17505501	6,454935	6,674525	6,845763	6,295571	6,486876	6,595415	6,6584077	6,459287333	-0,19912033	0,871081533	Ftsjd1
17203861	3,148722	4,421291	4,035029	3,773044	3,570911	3,663738	3,8683473	3,669231	-0,19911633	0,871083948	---
17203271	6,103684	5,43118	4,817168	5,985807	4,884322	4,884571	5,4506773	5,251566667	-0,19911067	0,87108737	---
17221234	10,07974	10,01428	10,28022	9,896365	9,963409	9,917156	10,124747	9,925643333	-0,19910333	0,871091798	---
17400809	4,896333	5,134367	5,271349	5,164008	5,769107	3,771686	5,100683	4,901600333	-0,19908267	0,871104276	---
17343158	3,851859	3,633134	3,425788	3,446482	3,453524	3,41357	3,636927	3,437858667	-0,19906833	0,871112931	---
17361829	5,735383	5,537947	5,440953	5,528643	5,259973	5,328564	5,5714277	5,372393333	-0,19903433	0,871133461	Tigd3
17231100	5,794005	5,657497	5,531851	5,379779	5,554247	5,45224	5,6611177	5,462088667	-0,199029	0,871136681	Traf5
17244705	6,580764	6,805257	6,266769	6,200174	6,221997	6,633646	6,55093	6,351939	-0,198991	0,871159627	Acss3
17274212	9,495087	9,516952	9,391747	9,216176	9,31432	9,276334	9,4679287	9,268943333	-0,19898533	0,871163048	Rock2
17399776	5,671344	5,236925	6,474051	5,75422	5,567827	5,463421	5,7941067	5,595156	-0,19895067	0,871183982	S100a14
17289971	4,822329	5,016436	4,890534	4,637512	4,891165	4,603921	4,9097663	4,710866	-0,19890033	0,871214377	Ccno
17521130	5,907398	6,129673	6,056043	5,822719	5,739725	5,934044	6,031038	5,832162667	-0,19887533	0,871229474	Glyctk
17372888	3,510248	3,617332	4,032941	3,7381	3,510003	3,315965	3,7201737	3,521356	-0,19881767	0,871264299	Olfr1151
17228656	5,345775	5,04641	5,442042	5,066494	5,11	5,061412	5,2780757	5,079302	-0,19877367	0,871290872	Fam5b
17373657	4,170537	3,984812	4,236211	4,027397	3,808931	3,958951	4,13052	3,931759667	-0,19876033	0,871298924	---
17207029	6,872405	5,920715	6,438523	6,097013	6,128225	6,410182	6,4105477	6,211806667	-0,198741	0,8713106	---
17235947	7,694759	7,732286	7,63825	7,476036	7,410317	7,583024	7,6884317	7,489792333	-0,19863933	0,871372004	D10Wsu102e
17312079	6,789545	7,084415	6,668221	6,504294	6,790594	6,651389	6,8473937	6,648759	-0,19863467	0,871374822	---
17290957	5,110578	5,100966	5,22064	4,853968	5,128522	4,854033	5,1440613	4,945507667	-0,19855367	0,871423747	Nkapl
17307470	2,980942	3,059093	3,033705	2,798825	2,815326	2,863969	3,02458	2,82604	-0,19854	0,871432002	---
17541232	3,618823	4,142681	3,596983	4,083863	3,230567	3,44849	3,7861623	3,58764	-0,19852233	0,871442673	---
17372850	4,499508	3,905137	4,067323	4,398329	3,922548	3,555545	4,1573227	3,958807333	-0,19851533	0,871446901	Olfr1126
17506947	5,852434	5,836396	6,267232	5,772048	5,972451	5,616025	5,985354	5,786841333	-0,19851267	0,871448512	---
17208007	6,091251	5,669642	5,296374	5,831594	5,378392	5,251781	5,6857557	5,487255667	-0,1985	0,871456164	---
17402357	8,427885	8,656506	8,375552	8,398776	8,299334	8,166358	8,4866477	8,288156	-0,19849167	0,871461197	1810037I17Rik
17494950	5,313941	5,764782	5,711844	5,49768	5,358979	5,311439	5,5968557	5,398366	-0,19848967	0,871462405	Scube2
17372498	6,835976	7,045636	7,00613	6,773795	6,622645	6,895855	6,9625807	6,764098333	-0,19848233	0,871466835	Zc3h15
17330649	4,592941	4,179548	4,671928	3,997512	4,186907	4,664591	4,4814723	4,283003333	-0,198469	0,871474889	Gm17783
17214753	5,293566	5,168962	5,013948	4,938652	4,963256	4,979231	5,1588253	4,960379667	-0,19844567	0,871488984	Irs1 // Irs1
17537173	5,577903	5,628552	5,416654	5,419151	5,270739	5,33792	5,5410363	5,342603333	-0,198433	0,871496636	2810403D21Rik
17211564	4,064523	4,403592	4,220796	4,292313	4,362326	3,438135	4,229637	4,031224667	-0,19841233	0,87150912	---
17469339	5,624892	6,374553	5,862548	5,788005	5,920516	5,558316	5,9539977	5,755612333	-0,19838533	0,87152543	---
17550136	4,594137	6,088146	4,775227	4,195594	4,956179	5,710682	5,1525033	4,954151667	-0,19835167	0,871545768	---
17449656	4,292856	4,668839	4,025181	4,09332	4,239918	4,058621	4,3289587	4,130619667	-0,198339	0,871553421	Ppef2
17550468	6,530279	6,76444	6,728096	6,338794	6,698479	6,390543	6,6742717	6,475938667	-0,198333	0,871557045	---
17473932	3,950151	3,652068	4,042281	3,609703	3,86681	3,573091	3,8815	3,683201333	-0,19829867	0,871577787	Sult2a5
17331241	3,836688	3,805514	3,779694	3,401585	3,64326	3,782295	3,8072987	3,609046667	-0,198252	0,87160598	Olfr187
17463833	6,376268	6,415431	6,379576	6,192522	6,267936	6,116089	6,390425	6,192182333	-0,19824267	0,871611619	H2afj
17204065	4,051123	4,965191	4,537435	4,439413	4,099621	4,420117	4,5179163	4,319717	-0,19819933	0,871637799	---
17490067	5,959677	5,721886	6,14185	5,921721	6,075356	5,231784	5,9411377	5,742953667	-0,198184	0,871647063	---
17347112	3,730152	3,857463	3,76869	3,6217	3,515825	3,624285	3,785435	3,58727	-0,198165	0,871658543	---
17202419	4,410852	4,736008	5,299836	4,524848	4,919044	4,40831	4,8155653	4,617400667	-0,19816467	0,871658744	---
17255335	7,471889	7,665702	7,582977	7,379013	7,216838	7,530251	7,5735227	7,375367333	-0,19815533	0,871664383	Samd14
17205603	4,324191	4,2965	4,482371	4,228334	4,054737	4,225632	4,3676873	4,169567667	-0,19811967	0,871685933	---
17232800	4,718062	4,593719	4,514923	4,553998	4,205665	4,472686	4,6089013	4,410783	-0,19811833	0,871686739	---
17204553	3,783134	2,937488	3,540235	3,196009	3,302219	3,168281	3,4202857	3,222169667	-0,198116	0,871688149	---
17406403	5,348422	5,557954	5,495657	5,253815	5,323112	5,231203	5,4673443	5,269376667	-0,19796767	0,871777777	Fam160a1
17243761	5,607914	5,575651	5,081015	5,095114	5,133675	5,441955	5,4215267	5,223581333	-0,19794533	0,871791273	---
17209665	5,853298	5,968906	5,464671	5,633598	5,524509	5,535406	5,7622917	5,564504333	-0,19778733	0,871886754	---
17549558	4,350686	4,342475	4,284024	4,11842	4,150419	4,115012	4,3257283	4,127950333	-0,197778	0,871892395	---
17549716	4,350686	4,342475	4,284024	4,11842	4,150419	4,115012	4,3257283	4,127950333	-0,197778	0,871892395	---
17257165	7,084157	7,00426	6,836771	6,654283	6,698475	6,979157	6,9750627	6,777305	-0,19775767	0,871904683	Arf2
17239363	3,679029	3,763425	3,772905	3,5065	3,586401	3,529359	3,738453	3,540753333	-0,19769967	0,871939737	Fam164b
17250149	7,414187	7,557976	7,248893	7,138024	7,117594	7,372425	7,4070187	7,209347667	-0,197671	0,871957063	Btnl10
17459822	5,212361	4,937974	5,343009	5,043409	5,054023	4,802923	5,164448	4,966785	-0,197663	0,871961898	---
17283767	7,262561	7,503074	7,196579	6,978685	7,197286	7,19328	7,320738	7,123083667	-0,19765433	0,871967136	Scarna13
17228941	3,481603	3,520382	3,41317	3,367838	3,109431	3,345021	3,4717183	3,274096667	-0,19762167	0,87198688	---
17336953	7,268551	6,64897	7,392504	6,856178	6,893061	6,967957	7,1033417	6,905732	-0,19760967	0,871994133	---
17387464	6,790113	7,186232	6,844405	6,76106	6,796288	6,670575	6,94025	6,742641	-0,197609	0,871994536	2700094K13Rik
17210437	6,147949	5,894164	5,837523	5,710605	5,75984	5,816481	5,9598787	5,762308667	-0,19757	0,872018109	---
17502440	7,306436	7,251379	7,39482	7,119515	7,242774	6,997637	7,317545	7,119975333	-0,19756967	0,87201831	Klf2
17542893	4,023835	3,631969	3,95215	3,512363	3,725724	3,777343	3,869318	3,67181	-0,197508	0,872055585	---
17446981	6,972857	7,016733	7,006316	6,869872	6,744397	6,789331	6,9986353	6,8012	-0,19743533	0,87209951	Fndc4
17359689	11,82716	11,78976	11,44828	11,33883	11,25197	11,88215	11,6884	11,49098333	-0,19741667	0,872110794	Scd2
17205799	4,400627	4,085346	3,938582	3,826928	3,91428	4,091136	4,1415183	3,944114667	-0,19740367	0,872118652	---
17512177	4,523097	4,094533	4,452466	4,137694	4,049874	4,290508	4,3566987	4,159358667	-0,19734	0,87215714	---
17220760	2,909402	4,005008	3,688297	2,991589	3,230292	3,788879	3,5342357	3,33692	-0,19731567	0,872171851	---
17399402	8,434719	7,910998	7,86885	7,909493	7,943778	7,769431	8,0715223	7,874234	-0,19728833	0,872188375	---
17474485	6,506991	6,256677	6,264928	6,021823	6,36304	6,051978	6,3428653	6,145613667	-0,19725167	0,872210542	Fbxo46
17432477	3,903181	3,829908	4,36904	3,712837	4,006052	3,791499	4,034043	3,836796	-0,197247	0,872213364	Gm13084
17531734	4,946088	4,655354	4,704103	4,519359	4,686963	4,507515	4,768515	4,571279	-0,197236	0,872220014	Stac
17289452	4,252327	4,281742	4,539236	4,138941	4,119248	4,223659	4,3577683	4,160616	-0,19715233	0,872270598	---
17300888	3,862001	3,693354	3,856991	3,680452	3,664531	3,476042	3,8041153	3,607008333	-0,197107	0,872298008	B020004C17Rik
17349949	4,862107	5,025335	4,870969	5,021173	4,549469	4,596503	4,9194703	4,722381667	-0,19708867	0,872309093	Pcdhb22
17528428	3,490262	3,644545	3,304295	3,213208	3,193891	3,440763	3,4797007	3,282620667	-0,19708	0,872314333	---
17201359	4,081957	3,776757	3,358162	3,539194	3,191058	3,895443	3,7389587	3,541898333	-0,19706033	0,872326224	---
17340772	6,845324	6,308662	6,751594	6,196973	6,654966	6,462518	6,6351933	6,438152333	-0,197041	0,872337914	Qk
17505252	9,355314	9,684906	9,406833	9,322737	9,245531	9,287721	9,482351	9,285329667	-0,19702133	0,872349806	Cyb5b
17547801	6,79164	6,765271	6,843398	6,498072	6,705047	6,606154	6,800103	6,603091	-0,197012	0,87235545	Nisch // Nisch
17529871	7,12035	7,067416	7,432882	7,038103	7,006613	6,98501	7,2068827	7,009908667	-0,196974	0,872378427	Acpl2
17203709	4,297219	3,439332	3,671451	3,422097	3,389504	4,00554	3,8026673	3,605713667	-0,19695367	0,872390723	---
17548334	10,92937	11,04927	10,8132	10,67811	10,63604	10,88689	10,930613	10,73368	-0,19693333	0,872403018	Ptges3 // Ptges3
17305524	3,808587	3,372462	3,097804	3,170179	3,15974	3,358254	3,4262843	3,229391	-0,19689333	0,872427207	Ear10
17200885	5,455307	4,283234	4,929725	5,172111	3,79824	5,107358	4,889422	4,692569667	-0,19685233	0,872452001	---
17533280	4,333257	4,619263	4,5945	4,236537	4,237687	4,482265	4,5156733	4,318829667	-0,19684367	0,872457242	---
17344642	4,944894	5,388999	5,361465	4,866805	5,028506	5,209751	5,231786	5,035020667	-0,19676533	0,872504615	H2-T10
17432757	5,649582	5,128978	5,231217	5,197069	5,166338	5,056202	5,3365923	5,139869667	-0,19672267	0,872530419	---
17374612	5,345872	5,282751	5,503285	5,193541	5,185653	5,162584	5,3773027	5,180592667	-0,19671	0,872538079	Phgr1
17401224	4,697341	4,321074	4,370509	4,334276	4,120804	4,343912	4,4629747	4,266330667	-0,196644	0,872577997	---
17406708	5,04743	4,677175	5,014596	4,546117	4,769314	4,833896	4,913067	4,716442333	-0,19662467	0,87258969	Tmem79
17398581	6,634261	6,757646	6,635034	6,570784	6,385584	6,48074	6,675647	6,479036	-0,196611	0,872597956	Fbxw7
17543639	4,643063	3,762252	4,254151	4,167345	3,955627	3,946704	4,219822	4,023225333	-0,19659667	0,872606626	---
17233737	9,826217	10,1037	9,828515	9,400495	9,933326	9,834859	9,9194773	9,722893333	-0,196584	0,872614287	---
17398888	7,803699	7,887701	7,799044	7,669337	7,549152	7,682403	7,830148	7,633630667	-0,19651733	0,872654611	Isg20l2
17200087	4,847071	4,93772	4,830259	4,944291	4,472023	4,60922	4,8716833	4,675178	-0,19650533	0,87266187	---
17315392	10,10185	10,27725	9,991879	9,577012	10,11276	10,09181	10,12366	9,927194	-0,19646567	0,872685864	---
17507532	5,600451	5,32586	5,726911	5,173255	5,508072	5,382722	5,551074	5,354683	-0,196391	0,872731031	---
17438530	4,880533	5,057462	4,57773	4,816554	4,476158	4,633921	4,838575	4,642211	-0,196364	0,872747364	Lphn3
17497953	5,405762	5,568113	5,598477	5,641205	5,043356	5,298757	5,5241173	5,327772667	-0,19634467	0,87275906	Polr2l
17502954	8,48902	8,730719	8,524826	8,292332	8,390154	8,47325	8,5815217	8,385245333	-0,19627633	0,872800399	Dnajb1
17350364	8,290712	8,420206	8,050191	7,847261	8,178091	8,146944	8,253703	8,057432	-0,196271	0,872803626	LOC100861902
17391463	10,22262	10,24489	10,28195	10,02378	10,02008	10,11681	10,24982	10,05355667	-0,19626333	0,872808264	---
17433909	7,145687	7,230137	7,417511	7,191686	6,907551	7,105418	7,264445	7,068218333	-0,19622667	0,872830447	Gltpd1
17310466	7,877888	7,913876	7,97755	7,713532	7,585976	7,881142	7,9231047	7,726883333	-0,19622133	0,872833674	Drosha
17203007	6,332439	5,874553	5,749914	5,771186	5,873235	5,723862	5,9856353	5,789427667	-0,19620767	0,872841942	---
17547642	4,728101	4,554978	4,580497	4,75663	4,209418	4,308971	4,621192	4,425006333	-0,19618567	0,872855252	Gm10838
17349065	4,204324	4,176454	4,233107	3,932258	4,067361	4,025828	4,2046283	4,008482333	-0,196146	0,872879252	---
17396132	7,779113	7,89372	7,632616	7,564219	7,59461	7,558262	7,768483	7,572363667	-0,19611933	0,872895386	E2f5
17280919	4,218733	4,61091	4,420654	4,457455	4,120976	4,083564	4,4167657	4,220665	-0,19610067	0,87290668	---
17253448	5,759811	6,407913	6,085003	4,954052	6,172765	6,537664	6,0842423	5,888160333	-0,196082	0,872917975	Mir144
17212945	5,962362	5,177544	5,635338	5,381772	5,527923	5,27739	5,591748	5,395695	-0,196053	0,872935522	---

17305683	7,004064	6,84488	6,691009	6,899086	6,63136	6,421358	6,846651	6,650601333	-0,19604967	0,872937539	---
17200687	4,636233	5,189812	4,63487	4,890059	4,180428	4,802289	4,820305	4,624258667	-0,19604633	0,872939556	---
17271151	6,82922	6,216665	6,917338	6,418361	6,367961	6,588849	6,6544077	6,458390333	-0,19601733	0,872957103	Cacng4
17532280	5,098114	5,267304	5,311221	4,93786	4,890184	5,260692	5,2255463	5,029578667	-0,19596767	0,872987156	1700020M21Rik // 1700020M21Rik
17331533	7,943871	7,940568	7,704888	7,656293	7,786496	7,558738	7,863109	7,667175667	-0,19593333	0,873007932	Hspa13
17451482	5,380052	5,313377	5,715709	5,304651	5,435906	5,080898	5,4697127	5,273818333	-0,19589433	0,873031532	Svop
17222564	8,317756	8,283068	8,339685	8,055143	8,218233	8,079601	8,313503	8,117659	-0,195844	0,873061991	Snord89
17490935	6,690807	6,28263	6,687559	6,312703	6,361413	6,39935	6,5536653	6,357822	-0,19584333	0,873062395	Spaca4
17207825	4,491777	4,685489	4,452773	3,899879	4,523025	4,619664	4,5433463	4,347522667	-0,19582367	0,873074296	---
17507236	4,891194	4,773602	4,954751	4,683831	4,68113	4,667182	4,8731823	4,677381	-0,19580133	0,873087812	---
17232172	5,173577	4,988746	4,967331	4,770858	4,859492	4,912004	5,043218	4,847451333	-0,19576667	0,873108791	Taar1
17277919	5,103509	5,435662	5,298115	4,99872	5,210694	5,040579	5,2790953	5,083331	-0,19576433	0,873110204	Gm10433
17430192	7,64269	7,62708	7,526089	7,348126	7,394312	7,466312	7,5986197	7,402916667	-0,195703	0,873147323	Zmym1
17221751	5,4465	4,986809	5,260046	4,942241	5,034641	5,129445	5,2311183	5,035442333	-0,195676	0,873163664	4933415F23Rik
17403565	5,719486	5,231211	5,453109	5,310916	5,368256	5,137656	5,4679353	5,272276	-0,19565933	0,873173751	Gm16325
17393854	4,499561	4,263144	4,497409	4,245704	4,095074	4,332409	4,420038	4,224395667	-0,19564233	0,87318404	4933416E03Rik
17435041	2,779973	3,061977	3,18925	2,824687	2,815714	2,803898	3,0104	2,814766333	-0,19563367	0,873189286	---
17275873	3,68675	3,522269	3,871697	3,504899	3,442075	3,547013	3,693572	3,497995667	-0,19557633	0,873223987	---
17340244	7,398115	6,938192	6,995063	6,921351	6,840941	6,982384	7,1104567	6,914892	-0,19556467	0,873231049	Ttc7
17549454	3,709755	3,683751	3,707044	3,508745	3,571515	3,434156	3,7001833	3,504805333	-0,195378	0,873344041	---
17531455	3,588398	3,386335	4,241693	3,719251	3,238584	3,672489	3,7388087	3,543441333	-0,19536733	0,873350499	Fbxw26
17547869	9,170939	9,158586	8,921699	8,55444	9,084913	9,025784	9,0837413	8,888379	-0,19536233	0,873353525	Ywhaz
17456131	7,606899	7,493223	7,361881	7,284412	7,342724	7,248841	7,4873343	7,291992333	-0,195342	0,873365834	Tes
17365721	8,349936	8,478533	8,284225	8,224622	8,055199	8,246872	8,370898	8,175564333	-0,19533367	0,873370879	Bbip1
17300112	3,556018	3,312831	3,62772	3,314289	3,335401	3,260975	3,4988563	3,303555	-0,19530133	0,873390453	---
17448148	2,875259	2,960163	3,107935	2,654623	2,997345	2,70551	2,981119	2,785826	-0,195293	0,873395498	---
17292671	4,773253	4,689309	5,151923	4,547232	4,955659	4,525795	4,871495	4,676228667	-0,19526633	0,873411642	Msx2
17208055	4,288063	4,652902	5,19712	4,449698	4,932151	4,170457	4,712695	4,517435333	-0,19525967	0,873415678	---
17304510	10,89171	11,16049	11,24906	10,96599	11,05172	10,69787	11,10042	10,90519333	-0,19522667	0,873435657	Snord69
17293362	7,951903	8,403138	7,901816	7,850461	7,931487	7,889376	8,085619	7,890441333	-0,19517767	0,873465323	Ctla2a
17221869	4,731613	4,256681	4,966979	4,671696	4,413484	4,284718	4,6517577	4,456632667	-0,195125	0,87349721	---
17531181	6,535105	6,742095	6,866769	6,479742	6,367125	6,711751	6,7146563	6,519539333	-0,195117	0,873502054	Rhoa // Rhoa
17285546	4,802131	4,78779	4,668893	4,445469	4,491457	4,73659	4,752938	4,557838667	-0,19509933	0,87351275	Sfrp4
17442970	9,147513	9,331138	9,155739	8,862991	9,130444	9,05569	9,2114633	9,016375	-0,19508833	0,87351941	Vkorc1l1
17300316	4,922044	4,570976	4,845411	4,738581	4,34376	4,6711	4,779477	4,584480333	-0,19499667	0,873574914	Psmb11
17293376	4,265517	3,833337	4,127004	3,816441	4,112363	3,712343	4,075286	3,880382333	-0,19490367	0,873631229	Ctsj
17305487	5,846202	5,775406	5,313574	5,56249	5,340399	5,447724	5,6450607	5,450204333	-0,19485633	0,873659893	---
17206373	6,12922	6,753325	7,096497	6,62096	6,431045	6,342547	6,6596807	6,464850667	-0,19483	0,873675839	---
17352859	5,827705	5,841607	5,879497	5,485353	5,774839	5,704217	5,849603	5,654803	-0,1948	0,873694007	E430002N23Rik
17523486	6,625935	7,056394	6,609477	6,471139	6,549897	6,68649	6,7639353	6,569175333	-0,19476	0,873718232	Zfp105
17544300	3,511346	3,541274	4,075506	3,018262	3,972586	3,553077	3,7093753	3,514641667	-0,19473367	0,87373418	---
17437667	4,755687	4,645082	4,738512	4,539359	4,533297	4,482436	4,7130937	4,518364	-0,19472967	0,873736602	C030018K13Rik
17448565	4,245118	4,185256	4,349221	4,031855	4,228727	3,934829	4,259865	4,065137	-0,194728	0,873737611	Gabrg1
17429875	5,463078	5,548026	5,542755	5,500221	5,277945	5,19156	5,517953	5,323242	-0,194711	0,873747907	---
17247367	3,65669	3,521406	3,81936	3,631682	3,436763	3,345561	3,6658187	3,471335333	-0,19448333	0,873885801	---
17547002	4,272391	4,511446	4,147798	4,074788	3,70497	4,568432	4,310545	4,116063333	-0,19448167	0,873886811	Rmi1
17465728	3,667563	3,705355	3,803391	3,422068	3,658594	3,512299	3,7254363	3,530987	-0,19444933	0,873906396	Gm13861
17304240	7,054312	7,21397	7,064435	6,76847	6,863846	7,117254	7,1109057	6,916523333	-0,19438233	0,873946982	Pde12
17387674	3,782326	3,461324	4,045917	3,578502	3,518299	3,609679	3,763189	3,568826667	-0,19436233	0,873959098	Olfr1061
17208829	6,359656	6,337661	6,445221	6,748106	5,999052	5,812459	6,380846	6,186539	-0,194307	0,873992618	---
17546355	3,602497	4,477646	4,063727	3,606956	4,353984	3,600312	4,0479567	3,853750667	-0,194206	0,874053807	Rbmy1a1
17384741	4,763904	4,84498	5,074766	4,63264	4,623455	4,84498	4,89455	4,700358333	-0,19419167	0,874062491	---
17327972	3,368395	3,419537	3,235021	3,083105	3,080415	3,276938	3,3409843	3,146819333	-0,194165	0,874078647	---
17525432	4,813571	4,670749	4,773185	4,736643	4,425517	4,512851	4,7525017	4,558337	-0,19416467	0,874078849	Gm17365
17425379	3,581736	3,896393	3,773019	3,584667	3,610968	3,473077	3,7503827	3,556237333	-0,19414533	0,874090562	Rps15a-ps8
17334666	5,417402	5,117416	5,565184	4,986077	5,325512	5,206051	5,3666673	5,172546667	-0,19412067	0,874105507	Tpsb2
17257848	7,591373	7,307712	7,741836	7,556118	7,19321	7,309531	7,5469737	7,352953	-0,19402067	0,874166098	LOC100503496
17320494	6,341043	6,120025	6,389673	6,033701	6,225163	6,009842	6,2835803	6,089568667	-0,19401167	0,874171551	Syce3
17350730	4,854997	4,680928	5,003849	4,694254	4,611523	4,651993	4,8465913	4,65259	-0,19400133	0,874177812	1700065I17Rik
17534982	4,881511	4,922796	4,696644	4,666137	4,585782	4,667127	4,8336503	4,639682	-0,19396833	0,874197809	Gpr112
17502525	6,910025	6,109726	6,238976	6,616271	5,892995	6,167562	6,4195757	6,225609333	-0,19396633	0,87419902	Mir28b
17202383	4,260075	3,884283	4,319853	4,235163	4,054355	3,592861	4,154737	3,960793	-0,193944	0,874212553	---
17264519	5,061419	4,751378	5,126247	4,783567	4,782285	4,791486	4,9796813	4,785779333	-0,193902	0,874238004	Hes7
17407354	7,23567	7,086728	7,038061	6,917225	6,887121	6,974415	7,120153	6,926253667	-0,19389933	0,87423962	S100a1
17330916	5,272229	5,541863	5,499166	5,37392	5,228662	5,129004	5,4377527	5,243862	-0,19389067	0,874244872	---
17221653	4,147887	4,471068	4,399443	4,362692	4,147887	3,926276	4,339466	4,145618333	-0,19384767	0,874270929	---
17235347	4,989069	4,574869	5,030309	4,761869	4,738605	4,51224	4,864749	4,670904667	-0,19384433	0,874272949	---
17259525	9,489046	9,907726	9,828506	9,418616	9,639569	9,585692	9,7417593	9,547959	-0,19380033	0,874299614	---
17225829	5,923487	5,986329	6,308827	5,976852	5,890835	5,769761	6,072881	5,879149333	-0,19373167	0,874341228	1700063A18Rik
17204061	5,771903	6,325605	5,875492	5,90372	5,754069	5,734203	5,991	5,797330667	-0,19366933	0,874379006	---
17353215	9,460501	9,436194	9,522338	9,176472	9,202165	9,459397	9,473011	9,279344667	-0,19366633	0,874380824	---
17215192	5,100039	4,960565	5,148762	5,096822	4,74478	4,786787	5,0697887	4,876129667	-0,193659	0,874385268	Chrnd
17359195	7,872308	7,79276	7,809639	7,648698	7,54567	7,699434	7,8249023	7,631267333	-0,193635	0,874399814	Tbc1d12
17254347	8,265985	8,370883	8,437	8,071092	8,252637	8,169315	8,357956	8,164348	-0,193608	0,874416179	Ddx52
17506692	4,850469	4,887804	5,2305	4,638001	4,953822	4,796213	4,989591	4,796012	-0,193579	0,874433756	---
17321474	6,49136	6,871222	6,401258	6,268951	6,510299	6,404065	6,5879467	6,394438333	-0,19350833	0,874476589	Tuba1a
17363058	3,713364	3,37441	3,815887	3,515232	3,44505	3,36286	3,6345537	3,441047333	-0,19350633	0,874477801	Olfr1424
17427401	6,642023	6,975677	6,857578	6,711459	6,515464	6,667847	6,8250927	6,63159	-0,19350267	0,874480024	Cyp2j9
17546974	3,657068	3,961271	3,365606	3,258634	3,510005	3,634925	3,661315	3,467854667	-0,19346033	0,874505684	Gm7066
17469939	7,237008	7,75744	7,196612	7,209037	7,263836	7,137849	7,39702	7,203574	-0,193446	0,874514373	Snora7a
17249700	7,756197	7,743837	7,674445	7,360425	7,663752	7,570031	7,7248263	7,531402667	-0,19342367	0,87452791	3230401D17Rik // 3230401D17Rik
17432781	6,621963	7,065428	7,226069	6,750059	6,795432	6,787703	6,9711533	6,777731333	-0,193422	0,874528921	Rps19-ps3
17236227	6,536247	6,696214	6,552001	6,543722	6,645862	6,0147	6,5948207	6,401428	-0,19339267	0,874546702	Nt5dc3
17314182	5,043778	4,627521	4,707027	4,800052	4,60623	4,391932	4,7927753	4,599404667	-0,19337067	0,874560038	---
17547732	5,151972	5,097513	4,881666	4,652064	4,932205	4,966777	5,043717	4,850348667	-0,19336833	0,874561453	Exoc2 // Exoc2
17361878	5,578724	5,209062	5,196807	5,069458	5,259389	5,075704	5,3281977	5,134850333	-0,19334733	0,874574183	Slc22a20
17219356	7,930846	8,71379	8,481782	8,526389	7,947046	8,072994	8,3754727	8,182143	-0,19332967	0,874584893	Tstd1
17363559	5,729017	5,841814	5,830184	5,558428	5,647818	5,61507	5,8003383	5,607105333	-0,193233	0,874643496	Fam189a2
17276375	4,171952	4,41923	4,347739	4,160562	4,127887	4,070836	4,3129737	4,119761667	-0,193212	0,874656227	1700086L19Rik
17547949	6,420805	6,352252	6,138385	6,120413	6,12375	6,08766	6,303814	6,110607667	-0,19320633	0,874659663	Gm10808
17549358	6,439628	6,818492	6,601999	6,350024	6,546522	6,384652	6,6200397	6,427066	-0,19297367	0,874800732	---
17549744	6,439628	6,818492	6,601999	6,350024	6,546522	6,384652	6,6200397	6,427066	-0,19297367	0,874800732	---
17218790	7,664141	7,823802	7,759032	7,588078	7,649278	7,430708	7,7489917	7,556021333	-0,19297033	0,874802754	Scyl3
17200247	6,247676	6,268964	6,155082	6,016158	5,654864	6,421824	6,2239073	6,030948667	-0,19295867	0,874809828	---
17473806	5,664285	5,788221	5,782745	5,475012	5,680237	5,501173	5,7450837	5,552140667	-0,192943	0,874819328	2310014L17Rik
17367310	4,856682	4,127551	4,700469	4,194065	4,656221	4,255647	4,5615673	4,368644333	-0,192923	0,874831455	BC061194
17285527	7,77822	7,406366	7,518496	7,471852	7,378739	7,273726	7,567694	7,374772333	-0,19292167	0,874832264	---
17358454	4,564886	4,344052	4,63752	4,256099	3,966363	4,745273	4,515486	4,322578333	-0,19290767	0,874840753	C030016D13Rik
17548338	3,511121	3,261157	3,27962	3,135449	3,040424	3,297915	3,3506327	3,157929333	-0,19270333	0,874964669	Tmeff1 // Tmeff1
17285761	3,72682	3,30068	3,200507	3,00421	3,368989	3,276739	3,4093357	3,216646	-0,19268967	0,874972957	Vmn1r198
17220759	8,826617	9,070626	8,354712	8,739854	8,59565	8,338462	8,7506517	8,557988667	-0,192663	0,87498913	---
17377069	4,850195	4,831165	4,729993	4,514899	4,659111	4,659667	4,8037843	4,611225667	-0,19255867	0,87505241	LOC171588
17250221	4,190331	4,411546	4,356052	4,106704	4,053263	4,220333	4,3193097	4,126766667	-0,192543	0,875061913	---
17538974	4,701216	4,827117	5,06496	4,682609	4,663465	4,669665	4,864431	4,671913	-0,192518	0,875077077	Usp51
17372896	3,782139	3,916979	3,955374	4,185232	3,403161	3,488569	3,8848307	3,692320667	-0,19251	0,875081929	Olfr1161
17401203	3,333866	2,945024	3,195191	2,865015	3,137878	2,893722	3,158027	2,965538333	-0,19248867	0,875094869	---
17340570	6,120819	5,709298	6,239576	5,910567	5,767292	5,814591	6,023231	5,830816667	-0,19241433	0,875139959	Gm2808
17369126	8,403737	8,508548	8,284129	8,247306	8,218412	8,153458	8,3988047	8,206392	-0,19241267	0,87514097	Lrrc8a
17414851	5,351039	4,881184	5,014688	4,991129	4,851213	4,827429	5,0823037	4,889923667	-0,19238	0,875160786	---
17337633	3,139687	3,180184	3,231114	2,988251	3,071052	2,91455	3,1836617	2,991284333	-0,19237733	0,875162403	Olfr135
17260912	4,658178	4,851788	4,739467	4,598126	4,594741	4,479446	4,749811	4,557437667	-0,19237333	0,87516483	---
17505689	8,362823	8,502779	8,504968	8,151549	8,289586	8,35235	8,4568567	8,264495	-0,19236167	0,875171907	Aars
17279986	4,097563	4,029364	3,685614	3,788274	3,62232	3,824869	3,9375137	3,745154333	-0,19235933	0,875173322	---
17444804	9,183757	9,602034	9,243166	9,064236	9,171722	9,21608	9,3429857	9,150679333	-0,19230633	0,875205474	LOC100505283 // Gm15682 // Gm10045
17526923	6,078525	6,016299	6,086774	5,90971	5,983065	5,711939	6,0605327	5,868238	-0,19229467	0,875212552	Hspb2
17439893	8,168496	8,111636	8,24427	8,012645	7,747647	8,187382	8,1748007	7,982558	-0,19224267	0,875244098	---
17265727	8,339353	8,126245	8,266766	8,063025	8,094147	7,99852	8,2441213	8,051897333	-0,192224	0,875255423	Tmem93
17463883	10,29618	10,3278	10,30685	10,00441	10,24141	10,10835	10,310277	10,11805667	-0,19222	0,875257849	Strap
17507218	5,674285	5,610491	5,681665	5,281198	5,649081	5,459539	5,6554803	5,463272667	-0,19220767	0,875265332	---
17528932	4,414608	3,961053	3,805673	3,964748	3,893063	3,747085	4,0604447	3,868298667	-0,192146	0,875302745	---
17309264	4,55612	4,440615	4,68362	4,434822	4,300303	4,368917	4,5601183	4,368014	-0,19210433	0,875328025	4921525B02Rik
17355730	3,074052	3,067845	3,013081	3,050447	2,745835	2,782396	3,0516593	2,859559333	-0,1921	0,875330654	---
17466729	7,296786	7,06523	7,323606	6,916029	7,245953	6,947647	7,2285407	7,036543	-0,19199767	0,875392746	4921507P07Rik
17421462	5,669762	5,889089	5,255702	5,619144	5,276218	5,343208	5,604851	5,412856667	-0,19199433	0,875394768	LOC100861620 // Gm13235
17462778	6,177631	5,795186	6,349164	6,189222	5,886066	5,670829	6,107327	5,915372333	-0,19195467	0,875418837	Clec4b2
17377723	4,940295	4,833662	4,963172	4,887176	4,52291	4,751332	4,9123763	4,720472667	-0,19190367	0,875449784	Defb20
17317634	4,382714	4,16042	4,505203	4,191019	4,188718	4,092891	4,3494457	4,157542667	-0,191903	0,875450189	---
17201535	4,529222	4,590586	4,994037	4,391194	4,539906	4,607042	4,704615	4,512714	-0,191901	0,875451402	---
17365243	8,913616	8,662773	8,823824	8,815618	8,487768	8,52115	8,800071	8,608178667	-0,19189233	0,875456662	Npm3
17550773	3,120243	3,173329	3,094773	3,005266	2,946402	2,86118	3,1294483	2,937616	-0,19183233	0,875493072	---
17474292	4,255408	4,127723	4,10507	3,772483	4,091821	4,048509	4,1627337	3,970937667	-0,191796	0,875515121	Ceacam14
17219456	7,486675	7,667858	7,392504	6,577758	7,62465	7,769431	7,515679	7,323946333	-0,19173267	0,875553556	---
17225171	5,626441	5,910809	5,658157	5,555324	5,547563	5,517364	5,7318023	5,540083667	-0,19171867	0,875562052	---
17467543	4,544658	4,088153	4,509105	4,149642	4,132366	4,284775	4,3806387	4,188927667	-0,191711	0,875566705	Igkv8-19 // Igkv8-19

17203181	5,766938	5,691006	5,585142	5,603811	5,70667	5,157556	5,6810287	5,489345667	-0,191683	0,875583699	---
17543625	6,224382	6,008747	6,196779	5,881398	5,893735	6,079906	6,1433027	5,951679667	-0,191623	0,875620114	Cxcr3
17222094	4,11527	4,072567	4,181873	3,874243	4,207105	3,713506	4,1232367	3,931618	-0,19161867	0,875622744	AA619741
17280420	3,40247	3,460414	3,41665	3,372119	3,137741	3,194824	3,4265113	3,234894667	-0,19161667	0,875623958	---
17279833	7,37712	7,267248	7,782542	7,214108	7,493834	7,14424	7,4756367	7,284060667	-0,191576	0,87564864	---
17444055	5,363087	5,196409	4,831857	4,990196	4,764038	5,062422	5,130451	4,938885333	-0,19156567	0,875654912	---
17388033	4,017752	3,153395	3,723337	3,751412	3,331788	3,236967	3,6314947	3,440055667	-0,191439	0,875731797	Olfr142
17246345	7,132019	7,487687	7,270259	6,899167	7,062707	7,353817	7,296655	7,105230333	-0,19142467	0,875740497	Dnajc14
17297627	8,228871	8,17412	8,265874	7,944006	8,156998	7,993599	8,222955	8,031534333	-0,19142067	0,875742925	Samd8
17229059	4,483359	4,594127	4,392069	4,552051	3,87372	4,469714	4,4898517	4,298495	-0,19135667	0,875781775	Fmo3
17206537	3,039947	4,040911	3,470654	2,998483	3,478693	3,500296	3,5171707	3,325824	-0,19134667	0,875787846	---
17232189	4,220344	4,105563	3,943354	4,014762	3,941681	3,738816	4,0897537	3,898419667	-0,191334	0,875795535	Taar7d
17383825	6,781455	6,821478	6,790361	6,328852	6,725947	6,764528	6,7977647	6,606442333	-0,19132233	0,875802617	Fam78a
17291204	6,753962	6,614676	6,851275	6,475229	6,433529	6,737229	6,739971	6,548662333	-0,19130867	0,875810914	Hist1h2ac
17277287	5,35256	5,077442	5,338126	4,999257	5,198668	4,99632	5,2560427	5,064748333	-0,19129433	0,875819615	Vrtn
17352824	6,071534	6,135949	5,955774	5,98908	5,658958	5,94135	6,054419	5,863129333	-0,19128967	0,875822448	Zfp521
17483233	4,456742	4,467774	4,770976	4,372335	4,308256	4,441036	4,565164	4,373875667	-0,19128833	0,875823258	---
17375866	4,893167	5,174882	4,946156	4,761883	4,925397	4,753161	5,004735	4,813480333	-0,19125467	0,875843696	Astl
17209525	5,355633	4,845362	6,201153	5,509042	5,142236	5,177107	5,4673827	5,276128333	-0,19125433	0,875843899	---
17313799	6,891333	6,60699	6,895267	6,615727	6,589996	6,614178	6,7978633	6,606633667	-0,19122967	0,875858874	Gm10923
17333050	7,094497	7,218233	7,131339	6,729759	7,001343	7,139609	7,148023	6,956903667	-0,19111933	0,875925859	Fgfr1op
17504712	4,793643	5,094896	4,917348	4,74148	4,644704	4,8465	4,9352957	4,744228	-0,19106767	0,875957229	Exoc3l // Exoc3l
17501484	4,879338	4,920348	5,061926	4,570718	4,831468	4,886271	4,9538707	4,762819	-0,19105167	0,875966944	Spock3
17393032	5,172103	5,191338	5,85008	5,343812	5,086203	5,210384	5,404507	5,213466333	-0,19104067	0,875973623	Tspyl3
17201461	3,057006	3,344082	2,808009	2,941849	2,587796	3,106412	3,069699	2,878685667	-0,19101333	0,875990219	---
17260469	3,429817	3,597267	3,626446	3,445301	3,281248	3,353961	3,5511767	3,36017	-0,19100667	0,875994267	Gm11985
17260192	5,595575	5,609612	5,511694	5,388213	5,33219	5,423528	5,5722937	5,381310333	-0,19098333	0,876008435	Pgam2
17344658	3,697928	3,702304	3,834343	3,398439	3,714012	3,549255	3,7448583	3,553902	-0,19095633	0,87602483	Gm8909
17221193	7,864847	8,11323	7,910149	7,935077	7,73135	7,648944	7,962742	7,771790333	-0,19095167	0,876027663	---
17324439	3,081573	3,185862	3,242757	3,057326	2,852686	3,027378	3,170064	2,97913	-0,190934	0,876038391	---
17548510	4,720111	4,2953	4,565179	4,2981	4,302135	4,407592	4,5268633	4,335942333	-0,190921	0,876046285	---
17471990	4,918827	4,679317	5,020571	4,668058	4,645232	4,732763	4,872905	4,682017667	-0,19088733	0,876066728	---
17551131	6,789164	7,102051	7,130697	6,727039	6,939023	6,783422	7,007304	6,816494667	-0,19080933	0,876114095	---
17466878	4,443003	4,592734	4,754478	4,318761	4,590442	4,308971	4,5967383	4,406058	-0,19068033	0,876192437	Hoxa10
17405352	5,442864	5,164389	5,239332	5,076838	5,146119	5,05159	5,282195	5,091515667	-0,19067933	0,876193044	---
17338434	8,197459	8,313968	7,959062	7,85673	8,039164	8,002794	8,1568297	7,966229333	-0,19060033	0,876241025	AI314976
17537630	4,486987	4,523026	4,576134	4,417095	4,337607	4,259672	4,5287157	4,338124667	-0,190591	0,876246693	Cstf2
17203831	3,105647	3,315033	3,56083	3,189692	3,030359	3,189692	3,32717	3,136581	-0,190589	0,876247908	---
17496338	7,300565	7,092041	7,310654	7,124488	6,938633	7,068556	7,23442	7,043892333	-0,19052767	0,876285161	Il27
17463587	3,043026	2,795071	2,871754	2,934967	2,602407	2,601068	2,9032837	2,712814	-0,19046967	0,87632039	---
17460569	10,58171	10,43061	10,48031	10,23175	10,42546	10,26404	10,497543	10,30708333	-0,19046	0,876326262	Gp9
17333707	4,639909	4,519595	4,363312	4,358595	4,255844	4,337113	4,5076053	4,317184	-0,19042133	0,876349749	Mirlet7e
17209619	5,097753	4,189613	5,050464	5,148898	4,325193	4,292483	4,7792767	4,588858	-0,19041867	0,876351369	---
17274949	8,970551	9,115505	9,084208	8,696423	8,932279	8,970313	9,0567547	8,866338333	-0,19041633	0,876352787	Twistnb
17458932	2,887103	2,984701	3,129214	2,76476	2,842227	2,822851	3,0003393	2,809946	-0,19039333	0,876366758	Vmn1r6
17205195	3,683431	3,117991	3,674552	3,617414	2,984373	3,30304	3,4919913	3,301609	-0,19038233	0,87637344	---
17209385	3,683431	3,117991	3,674552	3,617414	2,984373	3,30304	3,4919913	3,301609	-0,19038233	0,87637344	---
17417821	5,808645	5,010942	5,236094	5,488615	4,867324	5,128597	5,3518937	5,161512	-0,19038167	0,876373845	Olfr1331
17525503	8,107342	8,091614	7,961401	7,788116	7,860595	7,94057	8,0534523	7,863093667	-0,19035867	0,876387817	Chek1
17341350	4,645306	4,245184	4,770315	4,620284	4,431671	4,0378	4,5536017	4,363251667	-0,19035	0,876393081	Vmn1r232
17298112	4,998135	4,824996	5,052949	4,817108	4,608865	4,879066	4,9586933	4,768346333	-0,190347	0,876394904	Wnt5a
17439786	5,28339	5,333737	5,440142	4,96881	5,263935	5,253601	5,352423	5,162115333	-0,19030767	0,876418798	A830049A06
17253258	5,862417	5,807849	5,863353	5,739954	5,727282	5,495475	5,8445397	5,654237	-0,19030267	0,876421835	Coro6
17481217	3,870918	4,047453	4,196462	4,035952	3,624184	3,883911	4,0382777	3,848015667	-0,190262	0,87644654	Olfr618
17547692	4,111977	3,432445	3,509285	3,386313	3,580628	3,515996	3,684569	3,494312333	-0,19025667	0,87644978	Gm20245
17330625	5,947797	5,642823	5,974399	5,736602	5,867505	5,39015	5,8550063	5,664752333	-0,190254	0,8764514	Cd200
17318179	5,880012	5,770424	5,97885	5,649142	5,521084	5,888317	5,8764287	5,686181	-0,19024767	0,876455248	Gm19570
17248127	6,482904	6,336945	7,130564	6,43101	6,73663	6,212065	6,6501377	6,459901667	-0,190236	0,876462335	---
17350925	11,14268	12,14334	10,9787	11,4492	11,18139	11,06343	11,421573	11,23134	-0,19023333	0,876463956	Iigp1
17285793	3,316151	3,277279	3,138055	3,094084	2,99784	3,068915	3,2438283	3,053613	-0,19021533	0,876474891	Vmn1r216
17240725	3,269554	3,165121	3,61379	3,143937	3,14829	3,185675	3,3494883	3,159300667	-0,19018767	0,876491699	---
17228792	7,174928	7,753919	7,18341	6,836751	7,245628	7,45961	7,3707523	7,180663	-0,19008933	0,876551443	---
17533533	3,610079	3,568838	3,347416	3,34321	3,243232	3,369732	3,5087777	3,318724667	-0,190053	0,876573518	---
17496901	8,631195	8,703131	8,603247	8,53867	8,376444	8,452313	8,6458577	8,455809	-0,19004867	0,876576151	Tial1
17542068	3,578434	3,237079	3,308962	3,130298	3,249684	3,174757	3,374825	3,184913	-0,189912	0,876659193	Mir883a
17337098	7,203681	7,406666	6,888308	7,047179	6,801768	7,080067	7,1662183	6,976338	-0,18988033	0,876678436	---
17426397	4,021727	3,786888	3,823642	3,624439	3,595804	3,842443	3,877419	3,687562	-0,189857	0,876692615	---
17533743	5,435662	5,222947	5,237295	5,503294	5,180266	4,642781	5,2986347	5,108780333	-0,18985433	0,876694235	Gm5124
17314575	5,901108	5,381975	5,951485	5,480387	5,610961	5,573665	5,744856	5,555004333	-0,18985167	0,876695856	---
17204129	5,879735	6,636219	5,677927	5,74582	6,307432	5,571098	6,064627	5,874783333	-0,18984367	0,876700717	---
17523847	3,78714	3,691147	4,04141	3,753312	3,450035	3,746938	3,839899	3,650095	-0,189804	0,876724822	Mmp7
17473856	8,695984	8,963228	8,849847	8,673101	8,591129	8,675447	8,836353	8,646559	-0,189794	0,876730899	Lig1
17530579	5,24583	5,226051	5,313587	5,002349	5,042371	5,171369	5,2618227	5,072029667	-0,189793	0,876731507	Col6a4
17216556	3,877661	3,419065	3,415313	3,379407	3,536768	3,226618	3,5706797	3,380931	-0,18974867	0,876758449	Gm7145 // Gm7145
17289584	7,365569	7,503826	7,418594	7,026741	7,321329	7,370675	7,4293297	7,239581667	-0,189748	0,876758854	Cenpk
17317205	8,527796	8,911005	8,797856	8,743258	8,625921	8,298374	8,7455523	8,555851	-0,18970133	0,876787215	---
17202813	3,788362	4,554284	4,154463	4,239576	3,925286	3,763278	4,165703	3,976046667	-0,18965633	0,876814564	---
17312139	6,843167	6,548234	6,679071	6,494089	6,42834	6,579109	6,6901573	6,500512667	-0,18964467	0,876821654	---
17549476	4,539319	4,781071	4,799706	4,420639	4,340045	4,790699	4,7066987	4,517127667	-0,189571	0,876866428	---
17549620	4,539319	4,781071	4,799706	4,420639	4,340045	4,790699	4,7066987	4,517127667	-0,189571	0,876866428	---
17391427	3,270892	3,189692	4,067469	2,873788	4,112525	2,973156	3,509351	3,319823	-0,189528	0,876892563	---
17286958	4,994687	4,806079	4,778152	4,701138	4,587248	4,721953	4,8596393	4,670113	-0,18952633	0,876893576	Hspb1 // Hspb1
17292069	5,282985	5,549222	5,553371	5,282431	5,471648	5,062987	5,4618593	5,272355333	-0,189504	0,876907151	BC024659 // BC024659
17520233	10,80003	10,70094	10,67395	10,5064	10,54437	10,55564	10,724973	10,53547	-0,18950333	0,876907556	Ctsh
17537336	5,93697	6,305515	6,155937	6,012512	5,886464	5,931035	6,1328073	5,943337	-0,18947033	0,876927615	---
17207935	4,874523	5,936907	4,285659	5,166499	5,107584	4,254705	5,032363	4,842929333	-0,18943367	0,876949903	---
17208655	5,901437	5,170101	5,561155	5,139872	5,407343	5,517185	5,544231	5,3548	-0,189431	0,876951523	---
17204897	5,983222	6,597636	5,40211	5,740763	5,237923	6,43617	5,9943227	5,804952	-0,18937067	0,876988198	---
17336082	4,73801	4,372883	4,485716	4,119958	4,621767	4,287002	4,532203	4,342909	-0,189294	0,877034804	Actl9
17475870	5,878538	6,139054	5,997268	5,687427	5,817963	5,941634	6,0049533	5,815674667	-0,18927867	0,877044125	Sycn
17265524	5,405889	5,13937	5,211144	5,040476	4,942732	5,205522	5,2521343	5,06291	-0,18922433	0,877077156	---
17405789	9,95014	9,85393	9,739307	9,505614	9,681354	9,788758	9,8477923	9,658575333	-0,189217	0,877081614	Trim59
17380659	5,368071	4,908756	5,446761	5,190712	5,16238	4,802905	5,241196	5,051999	-0,189197	0,877093773	Cdh4
17406066	8,303793	8,413644	8,362748	8,164576	8,069755	8,278296	8,3600617	8,170875667	-0,189186	0,877100461	Fnip2
17255002	4,958768	4,980956	4,780911	4,637977	4,971924	4,543271	4,9068783	4,717724	-0,18915433	0,877119713	4930405P13Rik
17445357	3,831453	3,428413	3,322897	3,349104	3,345976	3,32026	3,5275877	3,338446667	-0,189141	0,87712782	---
17270388	6,021432	5,733491	6,163735	5,693652	6,029622	5,628058	5,972886	5,783777333	-0,18910867	0,877147478	C1ql1
17275306	4,271142	4,553943	4,347485	4,121968	4,25643	4,226952	4,3908567	4,201783333	-0,18907333	0,87716896	Foxg1
17219067	8,441583	8,351682	8,324735	8,16299	8,191377	8,196581	8,3726667	8,183649333	-0,18901733	0,877203009	Tada1
17204623	6,206268	6,194208	6,150742	6,352198	5,807576	5,82442	6,1837393	5,994731333	-0,189008	0,877208684	---
17226597	4,35788	4,509153	4,77073	4,560733	4,05221	4,457815	4,545921	4,356919333	-0,18900167	0,877212535	---
17242703	6,117867	6,395489	6,319386	6,137232	6,179229	5,949312	6,2775807	6,088591	-0,18898967	0,877219832	E130317F20Rik
17207151	4,443044	4,275269	4,882106	3,986474	4,503524	4,543458	4,533473	4,344485333	-0,18898767	0,877221048	---
17345977	7,920737	8,133332	7,997303	7,807219	7,843638	7,833735	8,017124	7,828197333	-0,18892667	0,877258139	Sgol1
17477968	6,080608	5,873454	6,009176	5,666664	5,857973	5,871845	5,987746	5,798827333	-0,18891867	0,877263004	Car11
17329840	3,327933	3,222045	3,260287	3,151432	3,035763	3,056317	3,2700883	3,081170667	-0,18891767	0,877263612	---
17479252	3,255605	3,449817	2,942633	3,457944	2,731252	2,892271	3,2160183	3,027155667	-0,18886267	0,877297057	---
17352399	3,675822	4,209154	3,908352	3,762175	3,720927	3,743648	3,9311093	3,74225	-0,18885933	0,877299084	---
17311199	8,813762	8,801994	8,734618	8,5407	8,665769	8,577335	8,783458	8,594601333	-0,18885667	0,877300705	Dcaf13
17531529	3,566182	3,152251	2,95263	3,050725	3,089486	2,964329	3,2236877	3,034846667	-0,188841	0,877310232	---
17200025	5,908386	5,840887	6,783504	6,415818	5,698508	5,851954	6,1775923	5,98876	-0,18883233	0,877315502	---
17363148	3,73448	4,240104	4,376414	4,263869	3,202615	4,318088	4,1169993	3,928190667	-0,18880867	0,877329894	---
17258455	4,222936	4,371026	4,440019	4,007203	4,360742	4,099669	4,3446603	4,155871333	-0,188789	0,877341854	---
17319661	5,022828	5,048846	5,216213	4,842131	5,018598	4,860794	5,0959623	4,907174333	-0,188788	0,877342462	Cyp2d34
17423699	3,776447	4,117866	3,657066	3,639107	3,801104	3,544876	3,8504597	3,661695667	-0,188764	0,877357057	---
17288467	9,238379	9,292456	9,435277	9,010073	9,186172	9,203655	9,3220373	9,1333	-0,18873733	0,877373275	Lpcat1
17460357	5,391563	5,316272	4,886351	5,036592	4,883929	5,107459	5,198062	5,009326667	-0,18873533	0,877374491	Gm19265
17238681	3,388395	3,280507	4,01398	3,007333	3,809079	3,300304	3,5609607	3,372238667	-0,188722	0,8773826	Olfr799
17309041	5,899536	5,845741	5,841717	5,688369	5,825716	5,506833	5,8623313	5,673639333	-0,188692	0,877400844	Dach1
17207443	7,121353	6,812738	7,410252	6,938324	6,606564	7,233468	7,114781	6,926118667	-0,18866233	0,877418887	---
17356427	4,825355	4,715847	4,87951	4,518317	4,671408	4,665001	4,806904	4,618242	-0,188662	0,87741909	Ccdc85b // Ccdc85b
17477785	4,163599	3,883628	4,277287	3,764568	4,19174	3,802222	4,1081713	3,91951	-0,18866133	0,877419495	Mtag2
17376538	5,709815	5,305405	5,38	5,592733	5,249407	4,987251	5,4650733	5,276463667	-0,18860967	0,877450918	---
17473631	4,601495	4,605304	4,216574	4,260475	4,199255	4,397917	4,4744577	4,285882333	-0,18857533	0,8774718	Olfr1346
17508885	5,490858	5,714571	5,267755	5,115194	5,266731	5,525566	5,4910613	5,302497	-0,18856433	0,877478491	Mtmr7
17209657	4,83765	4,3504	5,290158	4,871142	4,351968	4,689509	4,8260693	4,637539667	-0,18852967	0,877499576	---
17202333	5,620741	5,385118	6,3821	5,768137	5,456644	5,597608	5,7959863	5,607463	-0,18852333	0,877503428	---
17248476	9,60111	9,668459	9,548799	9,438546	9,340147	9,474197	9,6061227	9,41763	-0,18849267	0,877522081	Pank3
17317472	3,642961	3,648412	3,764323	3,429468	3,498014	3,562871	3,685232	3,496784333	-0,18844767	0,877549453	Gsdmc4
17545379	5,80659	5,323074	5,179849	5,35538	5,196792	5,192051	5,4365043	5,248074333	-0,18843	0,877560199	---
17294477	8,589489	8,54261	8,427539	8,215715	8,367815	8,410869	8,5198793	8,331466333	-0,188413	0,87757054	Rfesd
17414159	3,548347	3,519547	3,741257	3,434562	3,54231	3,267089	3,6030503	3,414653667	-0,18839667	0,877580475	Olfr270
17269358	4,558673	4,312956	5,473763	4,684218	4,777178	4,31885	4,7817973	4,593415333	-0,188382	0,877589397	Krt15
17211529	2,939525	3,020309	2,9252	2,754444	2,815713	2,749743	2,961678	2,7733	-0,188378	0,87759183	---

17343176	6,262028	6,060098	6,369186	5,990895	5,937982	6,19736	6,2304373	6,042079	-0,18835833	0,877603793	Tff1
17334674	5,071858	5,074643	5,056226	4,823084	5,041712	4,772968	5,0675757	4,879254667	-0,188321	0,877626504	Tpsg1
17387824	5,387359	5,367501	5,560557	5,350998	5,139827	5,259702	5,4384723	5,250175667	-0,18829667	0,877641306	---
17512990	6,876754	6,921078	6,52592	6,474973	6,607887	6,676038	6,774584	6,586299333	-0,18828467	0,877648607	Ddx19b
17259524	8,569938	8,61049	8,53795	8,338462	8,357648	8,45747	8,5727927	8,384526667	-0,188266	0,877659962	---
17225110	4,572618	5,205462	4,733323	4,909827	4,826182	4,210647	4,8371343	4,648885333	-0,188249	0,877670304	---
17463642	3,957208	3,536692	3,449661	3,369337	3,581333	3,42822	3,6478537	3,45963	-0,18822367	0,877685716	Tas2r113
17277754	4,576264	3,804736	3,867338	4,324358	3,823916	3,535394	4,0827793	3,894556	-0,18822333	0,877685919	---
17548709	3,591457	3,508337	3,187912	3,130254	3,40238	3,190405	3,4292353	3,241013	-0,18822233	0,877686527	LOC100862152
17467302	5,350195	4,984009	6,086945	5,250499	5,403666	5,202364	5,4737163	5,285509667	-0,18820667	0,877696058	Vmn1r36
17385363	5,68987	5,736159	5,534205	5,437324	5,323401	5,634997	5,6534113	5,465240667	-0,18817067	0,87771796	---
17210779	4,301729	4,056282	3,778328	4,550587	3,734999	3,28625	4,0454463	3,857278667	-0,18816767	0,877719785	---
17531563	6,371716	6,444013	6,527605	6,307211	6,117167	6,354502	6,447778	6,259626667	-0,18815133	0,877729722	Klhl18
17366740	8,073533	7,876034	7,815963	8,226315	7,191343	7,783421	7,9218433	7,733693	-0,18815033	0,877730331	Mir467c // Mir467c
17411626	3,773604	3,367502	3,49898	3,511504	3,210039	3,354103	3,5466953	3,358548667	-0,18814667	0,877732561	8430436N08Rik
17216278	4,266739	4,260322	4,374712	4,038957	4,160371	4,138051	4,300591	4,112459667	-0,18813133	0,87774189	---
17386117	4,74832	4,707715	4,563307	4,514067	4,473504	4,467591	4,673114	4,485054	-0,18806	0,877785291	---
17549550	4,382486	4,603959	4,716663	4,127703	4,359186	4,652061	4,5677027	4,37965	-0,18805267	0,877789753	---
17549708	4,382486	4,603959	4,716663	4,127703	4,359186	4,652061	4,5677027	4,37965	-0,18805267	0,877789753	---
17220757	8,749943	8,688326	8,982105	8,512497	8,683656	8,660073	8,8067913	8,618742	-0,18804933	0,877791781	---
17404509	3,925554	3,889016	3,729373	3,737273	3,798037	3,444491	3,847981	3,659933667	-0,18804733	0,877792998	---
17269381	5,621314	5,582909	5,342506	5,036285	5,477314	5,469185	5,5155763	5,327594667	-0,18798167	0,877832953	Krt9
17342617	5,422711	5,737709	5,563844	5,645989	5,303934	5,210446	5,5747547	5,386789667	-0,187965	0,877843094	Arhgdig
17360240	5,583673	5,362022	5,480551	5,202602	5,465644	5,194152	5,4754153	5,287466	-0,18794933	0,877852627	Ccdc147
17459897	9,439641	9,46116	9,012544	9,118644	8,940973	9,290124	9,3044483	9,116580333	-0,187868	0,877902118	---
17449578	5,471632	5,712492	5,468867	5,425465	5,282301	5,3818	5,550997	5,363188667	-0,18780833	0,877938427	Gm19619
17200471	6,693726	6,799682	7,040709	6,812096	6,64347	6,515152	6,8447057	6,656906	-0,18779967	0,877943701	---
17503431	8,916623	9,019709	8,944188	8,695869	8,772001	8,849483	8,9601733	8,772451	-0,18772233	0,877990763	Orc6
17358544	7,633153	8,571203	7,440524	7,83673	7,479234	7,765831	7,8816267	7,693931667	-0,187695	0,878007397	Cd274
17265821	4,742841	4,640846	4,875154	4,600796	4,563448	4,531683	4,752947	4,565309	-0,187638	0,878042088	Olfr411
17205093	5,027137	4,382917	4,796997	4,680308	4,063892	4,900036	4,7356837	4,548078667	-0,187605	0,878062172	---
17209283	5,027137	4,382917	4,796997	4,680308	4,063892	4,900036	4,7356837	4,548078667	-0,187605	0,878062172	---
17270315	4,704348	4,595894	4,943107	4,743145	4,57579	4,361625	4,747783	4,560186667	-0,18759633	0,878067447	Gjc1
17388435	8,187725	8,096786	8,04452	8,069927	7,725616	7,970766	8,109677	7,922103	-0,187574	0,87808104	Tspan18
17420437	9,878298	10,06416	9,546792	9,417624	9,701579	9,807511	9,82975	9,642238	-0,187512	0,878118776	---
17482826	3,836612	3,913248	4,332077	3,943021	3,598601	3,977967	4,0273123	3,839863	-0,18744933	0,87815692	---
17331168	7,26577	7,290284	7,270729	7,079198	7,047095	7,138425	7,2755943	7,088239333	-0,187355	0,878214342	---
17356517	5,606438	5,567698	5,893509	5,372054	5,43231	5,701218	5,689215	5,501860667	-0,18735433	0,878214748	Kcnk7
17547819	5,275058	5,685565	5,242597	5,318021	5,244639	5,078591	5,4010733	5,213750333	-0,187323	0,878233821	9430077A04Rik
17548896	3,525041	3,878659	3,873628	3,710898	3,605234	3,399375	3,7591093	3,571835667	-0,18727367	0,878263853	Gm10568
17542486	4,831235	4,707988	4,342601	4,700999	4,424554	4,194454	4,6272747	4,440002333	-0,18727233	0,878264665	Naa10
17533474	7,98096	7,782316	7,826012	7,498957	7,932619	7,596184	7,863096	7,67592	-0,187176	0,878323312	Maoa
17528778	6,414982	6,496516	6,396096	6,252645	6,305258	6,188274	6,4358647	6,248725667	-0,187139	0,878345838	Ccpg1
17467456	8,234985	7,495579	7,79338	7,73296	7,763809	7,465944	7,8413147	7,654231	-0,18708367	0,878379527	---
17300036	4,300811	4,689229	4,729279	4,638421	4,184639	4,335029	4,5731063	4,386029667	-0,18707667	0,878383789	---
17467171	7,656356	7,641263	7,685452	7,509933	7,352088	7,55983	7,6610237	7,473950333	-0,18707333	0,878385818	Vopp1
17517218	4,10385	3,689348	4,073476	3,739586	3,879519	3,686401	3,955558	3,768502	-0,187056	0,878396372	---
17207171	3,640183	3,868843	4,494563	3,863861	3,853844	3,724802	4,0011963	3,814169	-0,18702733	0,878413826	---
17285641	3,509718	3,608896	3,884953	3,5888	3,395396	3,45832	3,6678557	3,480838667	-0,187017	0,878420117	Olfr1367
17318058	4,687629	4,801978	4,974484	4,414072	4,904565	4,584468	4,8213637	4,634368333	-0,18699533	0,87843331	Cyp11b2
17340356	5,780914	5,223408	5,568192	5,513238	5,394094	5,104264	5,5241713	5,337198667	-0,18697267	0,878447111	Ston1
17524206	9,855138	10,08609	9,924644	9,812481	9,641216	9,851327	9,9552907	9,768341333	-0,18694933	0,878461319	---
17454816	5,187968	5,170781	5,462195	5,001266	5,0401	5,218794	5,273648	5,08672	-0,186928	0,878474309	Zfp316
17205023	5,384389	4,600078	5,320035	5,722898	4,527081	4,493779	5,1015007	4,914586	-0,18691467	0,878482428	---
17209213	5,384389	4,600078	5,320035	5,722898	4,527081	4,493779	5,1015007	4,914586	-0,18691467	0,878482428	---
17549904	7,094418	6,478768	6,728998	6,513415	6,67212	6,555915	6,7673947	6,580483333	-0,18691133	0,878484457	---
17296317	4,627271	4,580487	4,493021	4,40179	4,319145	4,419131	4,5669263	4,380022	-0,18690433	0,87848872	Hspb3
17396097	3,731432	3,887463	3,634371	3,762751	3,530221	3,399591	3,7510887	3,564187667	-0,186901	0,87849075	Slc7a12
17221520	4,170467	3,790495	3,543386	3,755827	3,678565	3,509314	3,8347827	3,647902	-0,18688067	0,878503131	Crisp4
17394535	5,169077	4,327408	4,709777	4,523928	4,547565	4,57414	4,7354207	4,548544333	-0,18687633	0,87850577	---
17233351	4,709217	4,617192	4,779797	4,572045	4,656588	4,3171	4,7020687	4,515244333	-0,18682433	0,878537435	---
17209487	5,07835	4,189047	4,105118	4,31903	4,291542	4,201488	4,457505	4,270686667	-0,18681833	0,878541089	---
17438113	4,460416	4,411644	4,632771	4,372464	4,263868	4,308206	4,5016103	4,314846	-0,18676433	0,878573973	Cwh43
17489903	3,52296	3,431803	3,50566	3,039755	3,476914	3,383585	3,4868077	3,300084667	-0,186723	0,878599145	---
17414184	4,610199	4,765093	4,716484	4,440054	4,54852	4,543043	4,6972587	4,510539	-0,18671967	0,878601175	4930522O17Rik
17445705	5,93952	5,790246	6,15024	5,654806	6,028936	5,636115	5,960002	5,773285667	-0,18671633	0,878603205	Gm3510
17385964	4,019029	3,716224	3,995109	3,559362	3,850948	3,759995	3,9101207	3,723435	-0,18668567	0,878621881	---
17208775	4,233572	4,535392	4,52324	4,347613	3,990057	4,394534	4,4307347	4,244068	-0,18666667	0,878633452	---
17549568	5,477135	5,491213	5,654227	5,476502	5,254547	5,331556	5,5408583	5,354201667	-0,18665667	0,878639542	---
17549722	5,477135	5,491213	5,654227	5,476502	5,254547	5,331556	5,5408583	5,354201667	-0,18665667	0,878639542	---
17330861	5,026711	4,775873	5,053432	4,351768	4,720794	5,223514	4,9520053	4,765358667	-0,18664667	0,878645633	---
17519349	6,723852	6,790843	6,982279	6,726301	6,681516	6,529359	6,8323247	6,645725333	-0,18659933	0,878674461	Gm16551
17375641	9,104366	8,994888	8,926053	8,79706	8,746646	8,921818	9,0084357	8,821841333	-0,18659433	0,878677506	Dut
17402662	6,870899	6,562208	6,44822	6,264432	6,451989	6,605238	6,627109	6,440553	-0,186556	0,878700853	Lef1
17243509	8,695634	8,638301	8,763385	8,526065	8,510259	8,501336	8,6991067	8,512553333	-0,18655333	0,878702477	Nfyb
17207261	6,569649	6,620524	7,08176	6,695471	6,836625	6,180208	6,757311	6,570768	-0,186543	0,878708771	---
17420443	9,181207	9,068613	8,476498	8,850776	8,785796	8,530166	8,9087727	8,722246	-0,18652667	0,878718719	---
17316775	4,338303	4,243138	4,215628	4,090731	4,29047	3,85637	4,2656897	4,079190333	-0,18649933	0,878735368	Abra
17385028	3,305083	2,90559	3,197071	3,109302	2,873595	2,865404	3,1359147	2,949433667	-0,186481	0,878746535	---
17308145	5,204911	5,053551	5,389483	4,97124	4,927406	5,190136	5,2159817	5,029594	-0,18638767	0,878803386	Synb
17531370	4,216861	4,089667	3,777981	4,054889	3,631089	3,839424	4,0281697	3,841800667	-0,186369	0,878814757	Fbxw16
17417898	6,349295	6,104962	6,049324	6,022951	6,035795	5,885775	6,1678603	5,981507	-0,18635333	0,8788243	Cldn19
17440717	4,828997	4,640357	5,195149	4,254521	4,755764	5,095161	4,8881677	4,701815333	-0,18635233	0,878824909	---
17515035	7,519169	7,470193	7,391547	7,532825	7,210551	7,078625	7,460303	7,274000333	-0,18630267	0,878855164	A230050P20Rik
17240100	6,944723	7,170753	7,173834	7,108262	6,712745	6,90946	7,0964367	6,910155667	-0,186281	0,878868363	Rnf217
17421103	5,291892	5,512465	5,368033	5,220335	5,166145	5,227086	5,3907967	5,204522	-0,18627467	0,878872221	B330016D10Rik
17420014	4,803183	4,835312	5,237461	4,818967	4,772594	4,725684	4,958652	4,772415	-0,186237	0,878895168	---
17210495	5,427022	5,723642	4,602287	5,083869	5,13176	4,978617	5,2509837	5,064748667	-0,186235	0,878896386	---
17371289	4,232311	4,11454	4,136179	4,128784	4,157327	3,638368	4,16101	3,974826333	-0,18618367	0,878927659	---
17517588	3,7588	3,823976	4,016602	3,671794	3,879587	3,48956	3,8664593	3,680313667	-0,18614567	0,87895081	---
17363098	5,728445	5,788668	5,799592	5,845542	5,348377	5,564358	5,772235	5,586092333	-0,18614267	0,878952638	A330040F15Rik
17205685	6,74674	6,538963	6,448161	6,723606	6,082611	6,369234	6,5779547	6,391817	-0,18613767	0,878955684	---
17418355	3,486519	4,315131	3,896078	3,573194	3,828635	3,73752	3,8992427	3,713116333	-0,18612633	0,878962589	---
17220370	6,245449	6,38552	5,984105	6,221482	5,794179	6,04104	6,2050247	6,018900333	-0,18612433	0,878963807	1700047M11Rik
17201079	7,590718	7,629926	7,889503	7,0559	7,552066	7,943841	7,7033823	7,517269	-0,18611333	0,878970509	---
17326685	6,11131	6,392958	6,058387	6,254005	5,828252	5,922112	6,1875517	6,001456333	-0,18609533	0,878981476	2810055G20Rik
17400386	9,029877	9,250383	9,228365	8,922404	9,003242	9,024817	9,1695417	8,983487667	-0,186054	0,879006659	Hormad1
17206597	3,75977	3,869212	3,789712	4,174237	3,173111	3,513219	3,8062313	3,620189	-0,18604233	0,879013767	---
17344126	5,364	4,652241	4,72526	4,632742	4,819221	4,731475	4,9138337	4,727812667	-0,186021	0,879026766	Hspa1b
17507355	7,086191	6,966043	6,921238	6,796848	6,939133	6,679432	6,9911573	6,805137667	-0,18601967	0,879027578	Lig4
17302974	5,997416	5,768448	5,97457	5,605507	5,722093	5,854871	5,913478	5,727490333	-0,18598767	0,879047076	---
17237252	10,14981	10,23119	10,1496	9,971068	10,00016	10,00142	10,176867	9,990882667	-0,185984	0,87904931	Nap1l1
17492305	5,739446	5,675081	5,65042	5,360447	5,715451	5,431108	5,6883157	5,502335333	-0,18598033	0,879051544	Hapln3
17204911	7,328875	7,716886	7,773216	7,590566	7,22016	7,450346	7,6063257	7,420357333	-0,18596833	0,879058856	---
17330183	11,56423	11,70783	11,61951	11,36018	11,55611	11,41745	11,630523	11,44458	-0,18594333	0,879074089	Stfa3
17515074	7,524371	7,946927	7,223499	7,415923	7,342529	7,378538	7,5649323	7,378996667	-0,18593567	0,87907876	Icam1
17288021	3,779139	3,9863	4,038314	3,667282	3,694432	3,884243	3,9345843	3,748652333	-0,185932	0,879080995	Gm20599
17280343	5,837139	5,522278	5,641485	5,462199	5,408401	5,57265	5,6669673	5,481083333	-0,185884	0,879110243	Gm9866
17384450	10,24384	10,78136	10,45403	10,12833	10,3455	10,44786	10,493077	10,30723	-0,18584667	0,879132992	---
17200405	2,870163	3,383432	3,919529	3,07244	3,467282	3,075886	3,3910413	3,205202667	-0,18583867	0,879137867	---
17264971	6,206717	6,431828	6,284563	6,028767	6,077297	6,25954	6,3077027	6,121868	-0,18583467	0,879140305	G630025P09Rik // G630025P09Rik
17526663	10,5579	10,67125	10,46433	10,31705	10,33846	10,48059	10,564493	10,3787	-0,18579333	0,879165493	Fam55b
17203387	3,249046	3,896002	4,102458	3,269817	3,692358	3,727997	3,7491687	3,563390667	-0,185778	0,879174837	---
17282963	7,063071	7,175144	7,066661	6,771078	6,868293	7,108177	7,1016253	6,915849333	-0,185776	0,879176056	---
17219477	9,289301	9,560374	9,270736	8,909459	9,159085	9,494566	9,3734703	9,187703333	-0,185767	0,87918154	---
17448589	3,960965	3,475156	3,736916	3,411593	3,574301	3,629876	3,7243457	3,53859	-0,18575567	0,879188447	Cox7b2
17221252	9,263321	9,530774	10,2201	9,81248	8,932279	9,712277	9,6713983	9,485678667	-0,18571967	0,879210386	---
17513699	5,526491	5,510213	5,563126	5,225243	5,489105	5,328408	5,5332767	5,347585333	-0,18569133	0,879227653	Snai3
17292491	6,609215	7,154305	6,977725	6,905148	6,49792	6,781293	6,9137483	6,728120333	-0,185628	0,879266251	Gm8672
17254300	8,994997	9,130734	9,183062	8,960607	8,975971	8,815421	9,102931	8,917333	-0,185598	0,879284535	1100001G20Rik
17342403	5,835306	5,992082	6,151018	5,758151	5,481008	6,182487	5,992802	5,807215333	-0,18558667	0,879291443	Rhbdl1
17263200	7,402974	7,421385	7,509385	7,251476	7,121077	7,404505	7,4445813	7,259019333	-0,185562	0,879306476	Zfp39
17524590	4,962345	4,771774	4,805538	4,738694	4,804233	4,440285	4,8465523	4,661070667	-0,18548167	0,87935544	Zglp1
17380119	5,929258	5,977653	5,997234	5,961343	5,407588	5,978903	5,9680483	5,782611333	-0,185437	0,879382666	Cass4
17334262	8,895815	8,761609	8,920965	8,662514	8,703114	8,656465	8,859463	8,674031	-0,185432	0,879385714	Pgp
17207563	7,559078	7,480089	7,623523	7,572529	7,202139	7,331743	7,55423	7,368803667	-0,18542633	0,879389168	---
17207625	5,006941	5,367955	5,173034	5,61378	5,045065	4,332814	5,1826433	4,997219667	-0,18542367	0,879390793	---
17204369	6,438897	6,812324	6,376597	6,086935	6,691938	6,292723	6,542606	6,357198667	-0,18540733	0,879400749	---
17413942	3,862434	3,500651	3,450346	3,405271	3,541309	3,310656	3,604477	3,419078667	-0,18539833	0,879406235	---
17292685	5,157464	4,850726	5,151892	4,978133	4,75093	4,874949	5,0533607	4,868004	-0,18535667	0,879431634	---
17294682	6,327188	5,844929	5,995835	5,665116	6,074022	5,872768	6,055984	5,870635333	-0,18534867	0,87943651	Mef2c // Mef2c
17207203	4,024494	3,845322	3,380715	3,360431	4,038455	3,295686	3,750177	3,564857333	-0,18531967	0,879454188	---
17206581	4,747314	5,473983	4,96617	4,585924	5,187355	4,858242	5,062489	4,877173667	-0,18531533	0,87945683	---
17521469	6,061277	6,208511	5,938025	5,972674	5,950816	5,72839	6,069271	5,88396	-0,185311	0,879459471	LOC100862215

17222951	7,603648	7,51169	7,092965	7,372855	7,10797	7,17164	7,4027677	7,217488333	-0,18527933	0,879478776	1700019D03Rik
17209951	5,794395	5,835875	4,687484	5,595392	5,067567	5,098979	5,4392513	5,253979333	-0,185272	0,879483246	---
17301849	5,678377	5,505245	5,325011	5,357379	5,412114	5,18337	5,5028777	5,317621	-0,18525667	0,879492593	Fndc3a // Fndc3a
17201763	7,3105	7,504274	7,53669	6,923828	7,246957	7,625049	7,450488	7,265278	-0,18521	0,879521043	---
17201939	7,3105	7,504274	7,53669	6,923828	7,246957	7,625049	7,450488	7,265278	-0,18521	0,879521043	---
17357897	4,747497	4,344632	4,122342	4,203091	4,300472	4,155312	4,4048237	4,219625	-0,18519867	0,879527952	Olfr1449
17249513	6,927435	7,576024	7,02368	7,045784	6,571815	7,354053	7,175713	6,990550667	-0,18516233	0,879550103	---
17288434	5,979523	5,613443	5,96589	5,88207	5,533219	5,588162	5,852952	5,667817	-0,185135	0,879566767	8030423J24Rik
17548715	7,990222	8,263701	7,944603	7,742303	8,042754	7,858068	8,0661753	7,881041667	-0,18513367	0,87956758	LOC100861831
17231686	3,033747	3,004262	3,02573	2,780854	2,969753	2,757752	3,0212463	2,836119667	-0,18512667	0,879571847	---
17206415	5,099449	4,958935	5,222222	5,109173	4,841387	4,7747	5,0935353	4,90842	-0,18511533	0,879578757	---
17398734	8,524023	8,491179	7,978255	7,566987	8,452173	8,419147	8,3311523	8,146102333	-0,18505	0,87961859	Cd5l
17274813	4,601227	4,456019	4,342547	4,404498	4,140565	4,299582	4,4665977	4,281548333	-0,18504933	0,879618997	Slc26a3
17289695	9,181668	9,228365	9,056674	8,876966	8,991843	9,042807	9,155569	8,970538667	-0,18503033	0,879630581	2810008M24Rik
17395718	5,745922	5,559844	5,90651	5,624309	5,537618	5,495356	5,7374253	5,552427667	-0,18499767	0,879650499	Chrna4
17239420	7,076387	7,08222	7,044004	6,945314	6,815991	6,886365	7,067537	6,882556667	-0,18498033	0,879661067	Aig1
17299097	3,538234	3,5031	3,325688	3,308008	3,254687	3,249397	3,455674	3,270697333	-0,18497667	0,879663303	---
17299099	3,538234	3,5031	3,325688	3,308008	3,254687	3,249397	3,455674	3,270697333	-0,18497667	0,879663303	---
17420811	5,575719	5,800135	5,687668	5,377118	5,418672	5,712938	5,6878407	5,502909333	-0,18493133	0,879690945	---
17351196	5,78077	5,382034	5,704208	5,586445	5,656953	5,068869	5,6223373	5,437422333	-0,184915	0,879700904	Apcdd1
17219756	6,383896	5,890034	6,725115	5,896146	6,499271	6,048939	6,333015	6,148118667	-0,18489633	0,879712286	Olfr417
17283832	5,701613	5,539523	5,905543	5,329954	5,666003	5,596162	5,7155597	5,530706333	-0,18485333	0,879738507	1700121N20Rik
17500662	7,256826	7,548917	7,43517	7,279357	7,371743	7,035311	7,4136377	7,228803667	-0,184834	0,879750296	Zdhhc2
17398632	3,190742	3,277282	3,492517	3,092419	3,225924	3,087735	3,3201803	3,135359333	-0,184821	0,879758224	---
17248191	6,441852	6,310003	6,422214	6,436165	6,073292	6,110165	6,3913563	6,206540667	-0,18481567	0,879761476	Gpr75
17325335	7,834391	7,819356	7,309548	6,906262	8,101652	7,401066	7,6544317	7,46966	-0,18477167	0,879788308	Gm5416
17398323	3,862184	4,085948	4,106791	3,949205	3,85691	3,694515	4,0183077	3,833543333	-0,18476433	0,87979278	Serpini1
17526574	5,159413	5,48195	5,002894	5,190876	5,016719	4,882404	5,2147523	5,029999667	-0,18475267	0,879799894	---
17222072	7,345935	7,770462	7,402405	7,464228	7,254447	7,24589	7,5062673	7,321521667	-0,18474567	0,879804163	Ccdc115
17207055	6,909632	6,578185	6,719388	6,612141	6,644627	6,396276	6,735735	6,551014667	-0,18472033	0,879819613	---
17467926	4,360936	4,026143	3,59987	3,487081	4,033206	3,912767	3,9956497	3,811018	-0,18463167	0,879873687	Gm4374
17284536	3,845593	3,58489	3,504011	3,408929	3,474276	3,497514	3,6448313	3,460239667	-0,18459167	0,879898083	---
17210145	3,135694	3,092297	3,350636	3,15416	2,875906	2,994869	3,1928757	3,008311667	-0,184564	0,879914957	---
17407188	8,222465	8,464619	8,536983	8,081346	8,430877	8,158218	8,4080223	8,223480333	-0,184542	0,879928375	---
17546251	5,619334	5,633485	5,559535	5,647722	4,959412	5,651666	5,604118	5,4196	-0,184518	0,879943013	LOC100862032 // LOC100862032
17537373	4,052606	3,943667	3,843385	3,744872	3,897973	3,643267	3,9465527	3,762037333	-0,18451533	0,87994464	---
17230249	4,882557	4,706358	5,175272	4,656791	4,651947	4,901905	4,9213957	4,736881	-0,18451467	0,879945046	---
17479923	3,171923	3,26579	3,456511	3,190188	3,117435	3,033171	3,2980747	3,113598	-0,18447667	0,879968224	Olfr291
17548096	4,551428	4,483039	4,217208	3,996247	4,294161	4,407966	4,417225	4,232791333	-0,18443367	0,879994452	Sf3b2 // Sf3b2
17205785	5,243813	5,33287	4,606791	5,015363	5,025448	4,589396	5,061158	4,876735667	-0,18442233	0,880001365	---
17362121	4,77813	4,524427	4,748719	4,584187	4,374609	4,539242	4,6837587	4,499346	-0,18441267	0,880007261	1700019N12Rik
17230819	5,936328	5,886724	5,828016	5,735267	5,771144	5,591471	5,8836893	5,699294	-0,18439533	0,880017834	Gm2061
17415345	6,773348	6,520621	6,952378	6,801764	6,435061	6,456623	6,7487823	6,564482667	-0,18429967	0,880076191	Gm13286 // Gm13287 // Gm13285 // G[illegible]
17263193	4,479003	4,337562	4,53257	4,421985	3,954385	4,419871	4,4497117	4,265413667	-0,184298	0,880077208	Fam183b
17482634	4,254299	4,312579	4,40155	4,134749	4,071536	4,209406	4,3228093	4,138563667	-0,18424567	0,880109133	Otoa
17316607	4,72441	4,71157	4,672671	4,65691	4,487915	4,411266	4,7028837	4,518697	-0,18418667	0,880145126	---
17240342	8,93192	9,12242	8,854024	8,7156	8,573027	9,06725	8,9694547	8,785292333	-0,18416233	0,880159972	Rpf2
17363995	3,342579	3,562302	3,420051	3,389984	3,341271	3,041191	3,441644	3,257482	-0,184162	0,880160175	---
17419840	5,930578	5,946635	6,198023	5,567069	6,003372	5,952348	6,0250787	5,840929667	-0,184149	0,880168106	Runx3
17490995	5,314002	5,412855	5,630621	5,08301	5,373868	5,348743	5,4524927	5,268540333	-0,18395233	0,880288098	Grin2d
17463617	6,612463	7,00071	6,75104	6,376538	6,738977	6,696875	6,788071	6,60413	-0,183941	0,880295013	---
17205015	9,251873	9,535851	9,081092	9,055256	8,915429	9,346477	9,2896053	9,105720667	-0,18388467	0,880329387	---
17392253	3,544697	3,452543	3,51089	3,374024	3,1773	3,4052	3,50271	3,318841333	-0,18386867	0,88033915	---
17401727	4,340385	4,043786	4,553643	3,954798	4,264129	4,167339	4,3126047	4,128755333	-0,18384933	0,880350947	---
17507901	5,138672	5,119001	5,125038	4,996405	4,981853	4,852969	5,1275703	4,943742333	-0,183828	0,880363965	Gm14851
17483793	5,648304	5,799011	5,960972	5,690176	5,567374	5,599257	5,8027623	5,618935667	-0,18382667	0,880364779	Ppapdc1a
17481982	9,484545	9,630264	9,536643	9,278109	9,433845	9,388176	9,550484	9,36671	-0,183774	0,880396918	Far1
17203745	5,64586	6,21055	5,684487	5,967997	5,459421	5,562284	5,8469657	5,663234	-0,18373167	0,880422752	---
17487291	7,072275	6,933673	6,885017	6,910881	6,631798	6,797323	6,963655	6,780000667	-0,18365433	0,880469947	Gemin7
17549882	3,24334	3,411038	3,1323	3,024098	3,070956	3,140661	3,262226	3,078571667	-0,18365433	0,880469947	---
17236727	3,965107	4,103194	4,057171	3,717392	3,710875	4,146257	4,041824	3,858174667	-0,18364933	0,880472999	---
17528319	7,473272	7,620378	7,541251	7,355043	7,303962	7,425004	7,544967	7,361336333	-0,18363067	0,880484391	Snx22
17474872	3,414338	3,819129	3,646723	3,340138	3,61645	3,372718	3,62673	3,443102	-0,183628	0,880486018	Vmn1r181
17220794	4,552541	4,355914	4,640751	4,398747	4,084088	4,515533	4,516402	4,332789333	-0,18361267	0,880495376	D730003I15Rik
17411751	9,309452	9,569954	9,8447	9,60572	9,364285	9,20342	9,574702	9,391141667	-0,18356033	0,880527317	Trp53inp1
17484140	8,981833	8,780802	8,895894	8,68646	8,582083	8,839396	8,8861763	8,702646333	-0,18353	0,88054583	Bccip
17370551	7,769757	7,786692	7,844313	7,681647	7,626614	7,541924	7,800254	7,616728333	-0,18352567	0,880548475	Arhgap15
17494187	4,298689	4,346886	4,356977	4,18622	4,162272	4,103555	4,334184	4,150682333	-0,18350167	0,880563124	Olfr620
17415233	4,189389	4,493787	4,413215	4,363992	4,107377	4,074603	4,3654637	4,181990667	-0,183473	0,880580621	---
17448806	11,58313	11,45568	11,65662	11,4932	11,52306	11,12889	11,565143	11,38171667	-0,18342667	0,880608902	---
17262141	8,384748	8,130953	8,098166	7,941547	7,973145	8,149076	8,2046223	8,021256	-0,18336633	0,88064573	Timd2
17321422	5,053557	5,019996	4,918307	4,804521	4,724995	4,912543	4,9972867	4,814019667	-0,183267	0,880706366	Rhebl1
17549516	6,269958	6,406088	6,47041	6,124165	6,164783	6,307734	6,382152	6,198894	-0,183258	0,880711861	---
17272817	6,245665	6,454951	6,470676	6,29045	6,177139	6,153938	6,3904307	6,207175667	-0,183255	0,880713692	Rbfox3
17216469	8,589185	8,49907	8,505022	8,315796	8,582485	8,145308	8,5310923	8,347863	-0,18322933	0,880729361	Serpinb10
17220485	4,19524	5,011704	4,886978	4,55703	4,537863	4,449352	4,697974	4,514748333	-0,18322567	0,880731599	4930488B22Rik
17333320	4,300768	4,282706	4,699442	4,36544	4,378332	3,989866	4,4276387	4,244546	-0,18309267	0,880812796	Mrgprh
17540977	6,871879	7,245943	7,263963	6,848178	6,8938	7,09054	7,1272617	6,944172667	-0,183089	0,880815035	Nkrf
17403138	9,749716	9,830267	9,709632	9,63398	9,477465	9,628941	9,763205	9,580128667	-0,18307633	0,880822768	Eif4e
17457715	4,130734	3,875067	3,613369	3,879584	3,624988	3,565378	3,8730567	3,689983333	-0,18307333	0,8808246	---
17534376	4,101133	3,878466	3,907279	4,005177	3,528013	3,804628	3,9622927	3,779272667	-0,18302	0,880857163	---
17379680	4,470793	4,442123	4,25142	4,251413	4,268969	4,094924	4,388112	4,205102	-0,18301	0,880863268	---
17268907	6,931683	6,624039	6,999486	6,509235	6,770131	6,726848	6,851736	6,668738	-0,182998	0,880870595	Gjd3
17219185	8,317356	8,265647	8,186822	8,081719	8,37413	7,765007	8,2566083	8,073618667	-0,18298967	0,880875683	Olfml2b
17395389	4,81613	4,446952	4,39715	4,305115	4,429571	4,376619	4,5534107	4,370435	-0,18297567	0,880884232	Gm14406
17450848	3,452084	3,340902	3,456355	3,166866	3,43562	3,097964	3,416447	3,233483333	-0,18296367	0,880891559	Tmed11
17391467	9,502543	9,455029	9,112496	9,153607	8,87914	9,488553	9,3566893	9,173766667	-0,18292267	0,880916593	---
17384190	7,055554	6,820189	7,032377	6,881713	6,648397	6,829262	6,9693733	6,786457333	-0,182916	0,880920664	Lmx1b
17530686	6,350128	6,172018	6,32344	6,038065	6,313572	5,945334	6,281862	6,098990333	-0,18287167	0,880947734	Abhd14a
17363316	6,462093	6,259397	6,462793	6,123445	6,573717	5,938879	6,394761	6,212013667	-0,18274733	0,881023659	Foxb2
17491797	6,217154	6,451896	6,249956	6,160506	6,311381	5,898968	6,3063353	6,123618333	-0,182717	0,881042183	---
17412013	5,424683	5,435662	5,352815	5,203392	5,191913	5,269726	5,4043867	5,221677	-0,18270967	0,881046661	Mmp16
17369864	7,691541	7,161105	7,964176	7,895914	6,899011	7,473779	7,6056073	7,422901333	-0,182706	0,881048901	---
17304872	3,825372	3,424849	3,240518	3,391243	3,341413	3,210047	3,496913	3,314234333	-0,18267867	0,881065593	---
17543928	4,023714	3,920159	4,134019	3,658925	3,739302	4,131674	4,025964	3,843300333	-0,18266367	0,881074754	Mir325 // Mir325
17379759	5,97986	5,694135	6,11821	5,706421	5,703324	5,834515	5,930735	5,748086667	-0,18264833	0,881084118	Trp53rk
17352412	4,041813	4,034707	4,064055	3,882622	3,940985	3,769088	4,0468583	3,864231667	-0,18262667	0,881097351	Mtpap
17200713	5,228683	5,392754	5,933018	5,043322	5,595843	5,367451	5,5181517	5,335538667	-0,182613	0,881105697	---
17446580	5,526773	5,61474	6,086792	5,956456	5,192604	5,531415	5,7427683	5,560158333	-0,18261	0,881107529	Shh
17428861	9,636966	9,88573	9,639078	9,242035	9,403769	9,968218	9,7205913	9,538007333	-0,182584	0,881123409	---
17330168	5,430855	5,223479	4,857213	4,749273	5,478153	4,736573	5,1705157	4,987999667	-0,182516	0,881164941	BC100530
17359852	5,63877	5,60121	5,599481	5,387021	5,532361	5,372618	5,6131537	5,430666667	-0,182487	0,881182653	---
17287792	4,310392	3,862939	3,682334	3,733763	3,778559	3,795896	3,9518883	3,769406	-0,18248233	0,881185504	---
17335926	5,145251	5,518032	5,234211	5,086414	5,086334	5,177327	5,2991647	5,116691667	-0,182473	0,881191204	---
17516164	6,045244	5,405492	5,78829	5,645669	5,66992	5,376113	5,746342	5,563900667	-0,18244133	0,881210546	Olfr901
17415349	6,673943	6,328598	6,734582	6,443551	6,281997	6,464252	6,579041	6,3966	-0,182441	0,88121075	Gm13287
17206359	3,270892	4,141255	3,609703	3,609703	3,651848	3,213015	3,67395	3,491522	-0,182428	0,881218691	---
17519775	3,797048	3,518997	3,508284	3,371816	3,396453	3,508783	3,6081097	3,425684	-0,18242567	0,881220116	---
17204571	6,469435	7,591023	7,425852	7,079229	6,975686	6,884211	7,1621033	6,979708667	-0,18239467	0,881239051	---
17495308	7,199739	6,891591	7,143489	7,369342	6,568025	6,750291	7,078273	6,895886	-0,182387	0,881243734	---
17354979	3,023222	3,297465	2,723068	2,789926	2,99036	2,716342	3,014585	2,832209333	-0,18237567	0,881250657	---
17538134	5,693876	5,351837	4,964937	4,914066	5,239389	5,310099	5,3368833	5,154518	-0,18236533	0,881256969	Cldn2
17207073	6,40341	6,617005	6,490863	7,04472	5,676599	6,242976	6,5037593	6,321431667	-0,18232767	0,881279978	---
17203211	4,663058	4,778204	4,005323	4,952355	3,89476	4,052866	4,482195	4,299993667	-0,18220133	0,881357153	---
17472985	3,936663	3,601887	4,269604	3,650839	3,703025	3,907745	3,9360513	3,753869667	-0,18218167	0,881369167	4930528G23Rik
17240858	4,95236	4,772489	4,97749	4,840591	4,776682	4,538637	4,9007797	4,718636667	-0,182143	0,88139279	Fam184a
17229391	9,965873	9,815415	9,924387	9,516944	9,868251	9,774052	9,9018917	9,719749	-0,18214267	0,881392994	Uck2
17341993	7,484809	7,413232	7,384524	7,271348	7,272487	7,192318	7,4275217	7,245384333	-0,18213733	0,881396252	Tbl3
17498032	8,165451	8,261988	8,327121	8,020255	8,065009	8,122964	8,25152	8,069409333	-0,18211067	0,881412544	Mob2
17302251	8,653528	8,924776	8,626904	8,397885	8,469944	8,791058	8,7350693	8,552962333	-0,182107	0,881414784	Sugt1
17503389	4,429446	4,690912	4,656266	4,397043	4,368032	4,465267	4,592208	4,410114	-0,182094	0,881422726	BC056474
17300050	3,318796	3,119471	3,602091	3,246389	3,198334	3,049361	3,346786	3,164694667	-0,18209133	0,881424355	A430107P09Rik
17232330	2,98022	2,992494	2,894845	2,771372	2,866475	2,683496	2,955853	2,773781	-0,182072	0,881436167	---
17548475	5,077105	5,196412	5,046845	4,761239	4,986075	5,026902	5,1067873	4,924738667	-0,18204867	0,881450423	Gm19566
17476175	8,40406	8,507788	8,386953	8,220657	8,194154	8,338005	8,4329337	8,250938667	-0,181995	0,881483213	Zfp84
17548846	10,24266	10,12449	10,19986	10,01544	10,00799	9,997631	10,189003	10,00702033	-0,181983	0,881490545	Atf1 // Atf1
17419658	5,189794	5,16491	5,610775	5,09026	5,342391	4,986965	5,3218263	5,139872	-0,18195433	0,88150806	---
17515027	5,237802	4,978883	5,137722	4,759375	5,087337	4,961832	5,1181357	4,936181333	-0,18195433	0,88150806	Rdh8
17501317	4,471526	4,409637	4,641769	4,478904	4,320177	4,178031	4,507644	4,325704	-0,18194	0,881516818	BC030500
17454114	5,912704	5,717213	5,837785	5,630336	5,611376	5,680195	5,8225673	5,640635667	-0,18193167	0,88152191	Irs3
17449542	10,20297	9,86757	9,674231	9,742626	9,724266	9,732204	9,9149237	9,733032	-0,18189167	0,881546352	---
17518312	11,66586	11,96836	11,95795	11,75084	11,78034	11,51537	11,864057	11,68218333	-0,18187333	0,881557554	Snord16a
17285883	5,130091	5,492774	5,373017	5,23282	5,096581	5,120866	5,3319607	5,150089	-0,18187167	0,881558572	Trim38
17427958	4,60316	4,347297	5,048877	4,476495	4,39105	4,586446	4,6664447	4,484663667	-0,181781	0,881613976	---

17208279	4,214661	4,570093	4,294382	4,258578	4,03732	4,238109	4,359712	4,178002333	-0,18170967	0,881657568	---
17476930	3,53495	3,453959	2,979869	3,099857	3,349174	2,974685	3,322926	3,141238667	-0,18168733	0,881671217	Gm4884
17293534	5,132664	5,309068	5,545174	5,828408	4,544036	5,069487	5,3289687	5,147310333	-0,18165833	0,881688939	Gm5665
17469698	3,692619	3,400979	3,654729	3,306059	3,538209	3,359096	3,5827757	3,401121333	-0,18165433	0,881691384	---
17281506	8,49849	8,524781	8,322278	8,190282	8,322928	8,287376	8,4485163	8,266862	-0,18165433	0,881691384	Pole2
17330175	9,070157	8,924907	8,533732	8,504904	8,93205	8,546885	8,842932	8,661279667	-0,18165233	0,881692606	Stfa2
17299502	3,417308	3,289187	3,408698	3,1852	3,28208	3,102994	3,371731	3,190091333	-0,18163967	0,881700347	Olfr744
17532737	6,069214	5,701908	5,601139	5,601992	5,641393	5,584166	5,7907537	5,609183667	-0,18157	0,881742925	---
17210413	3,689193	3,927075	4,02417	3,625285	3,884596	3,585879	3,880146	3,698586667	-0,18155933	0,881749444	---
17453917	6,512827	6,534994	6,635648	6,418639	6,403935	6,316396	6,5611563	6,379656667	-0,18149967	0,881785912	Slc12a9
17204953	5,297414	5,714043	5,908645	5,413857	5,449023	5,512751	5,640034	5,458543667	-0,18149033	0,881791617	---
17402144	7,228858	7,545889	7,356753	7,082481	7,189532	7,31515	7,3771667	7,195721	-0,18144567	0,881818918	Usp12 // Usp12
17549662	6,562759	6,256386	6,797719	6,351005	6,443563	6,278081	6,5389547	6,357549667	-0,181405	0,881843775	---
17210827	3,767017	3,076977	2,86764	2,956279	3,266911	2,94427	3,2372113	3,05582	-0,18139133	0,881852129	---
17332219	3,944602	4,409678	4,872232	4,215888	4,247104	4,219377	4,4088373	4,227456333	-0,181381	0,881858445	4930563D23Rik
17287576	7,476364	7,358225	7,621861	7,260278	7,264242	7,387832	7,4854833	7,304117333	-0,181366	0,881867614	Uimc1 // Uimc1
17316804	9,992164	10,14238	9,952054	9,759806	9,839235	9,943547	10,028866	9,847529333	-0,18133667	0,881885545	Eif3e
17476465	7,368964	7,224676	7,215139	7,179673	6,941776	7,143388	7,269593	7,088279	-0,181314	0,8818994	U2af1l4
17322825	3,727893	3,483106	3,747004	3,50407	3,550469	3,359522	3,6526677	3,471353667	-0,181314	0,8818994	---
17206051	4,327058	3,816728	4,48943	4,024216	4,193473	3,871781	4,211072	4,029823333	-0,18124867	0,881939339	---
17541695	3,664335	3,713455	3,865275	3,536249	3,571276	3,592005	3,7476883	3,56651	-0,18117833	0,881982336	Mir363
17442165	8,650512	8,496133	8,485811	8,249671	8,441247	8,398008	8,544152	8,362975333	-0,18117667	0,881983354	Rnf34
17449047	4,616086	4,267994	4,364242	4,213587	4,383879	4,107383	4,4161073	4,234949667	-0,18115767	0,88199497	Arl9
17206821	3,411502	3,966412	3,5717	3,536578	3,612688	3,25706	3,6498713	3,468775333	-0,181096	0,882032671	---
17409526	10,45174	10,40799	10,69475	10,91772	10,17872	9,914841	10,51816	10,33709367	-0,18106633	0,882050809	Amy2a5
17400562	8,965315	9,164205	9,137907	8,800737	8,996092	8,927427	9,0891423	8,908085333	-0,181057	0,882056515	Hist2h2bb
17341231	6,196852	6,247723	6,187255	6,272719	5,860024	5,956118	6,21061	6,029620333	-0,18098967	0,882097683	Rgmb
17537936	6,292763	5,996439	6,251933	6,008516	5,949013	6,040783	6,1803783	5,999437333	-0,180941	0,88212744	Tceal1
17540587	2,922425	2,987004	3,039829	2,820961	2,919747	2,66641	2,983086	2,802372667	-0,18071333	0,882266656	---
17545955	5,451901	5,817108	5,762579	5,549622	5,536235	5,403666	5,677196	5,496507667	-0,18068833	0,882281945	Bmx
17205795	4,739644	4,587295	4,494812	4,682773	4,773694	3,823376	4,6072503	4,426614333	-0,180636	0,88231395	---
17354506	4,158063	4,102331	3,942675	3,900534	3,925464	3,835178	4,0676897	3,887058667	-0,180631	0,882317008	---
17550420	4,717371	4,921372	5,09681	4,664478	4,85221	4,677061	4,911851	4,731249667	-0,18060133	0,882335151	---
17347981	3,27206	3,189692	3,463705	3,01548	3,141397	3,226791	3,3084857	3,127889333	-0,18059633	0,882338209	---
17496308	2,998609	2,916036	2,941171	2,803839	2,795198	2,714999	2,9519387	2,771345333	-0,18059333	0,882340044	---
17427213	4,308971	4,582093	4,346232	4,205373	4,444862	4,045287	4,412432	4,231840667	-0,18059133	0,882341267	---
17338103	4,984715	4,905595	5,029846	4,832303	4,741931	4,80427	4,9733853	4,792834667	-0,18055067	0,882366139	Crip3
17203781	4,521664	3,792909	3,9795	4,174346	3,568568	4,00956	4,0980243	3,917491333	-0,180533	0,882376944	---
17250141	6,203107	6,452645	6,376115	6,128542	6,120328	6,241472	6,3439557	6,163447333	-0,18050833	0,882392031	Zfp39 // Zfp39
17470681	4,457675	3,906185	4,601877	3,99904	4,20433	4,220844	4,3219123	4,141404667	-0,18050767	0,882392439	---
17344909	5,092237	5,035599	5,267193	5,000978	4,90905	4,943509	5,1316763	4,951179	-0,18049733	0,882398759	Olfr761
17207809	4,138247	4,037153	3,348516	3,746622	3,623117	3,612819	3,8413053	3,660852667	-0,18045267	0,882426079	---
17367740	7,506157	7,561441	7,776254	7,449882	7,442563	7,410083	7,6146173	7,434176	-0,18044133	0,882433011	Zmynd19
17203023	5,914495	5,653335	6,38634	6,411427	5,148631	5,852827	5,9847233	5,804295	-0,18042833	0,882440962	---
17204379	3,364458	3,916144	3,920976	3,400585	3,571472	3,688297	3,7338593	3,553451333	-0,180408	0,8824534	---
17212371	3,723789	3,065336	3,443061	3,24161	3,171166	3,278199	3,4107287	3,230325	-0,18040367	0,88245605	---
17202901	5,264709	4,539416	4,047549	4,167554	5,033069	4,109871	4,6172247	4,436831333	-0,18039333	0,882462371	---
17489886	8,690095	8,916742	8,732172	8,600825	8,561788	8,635216	8,7796697	8,599276333	-0,18039333	0,882462371	Ccne1
17421300	3,792199	3,597524	3,884514	3,349543	3,528553	3,855129	3,758079	3,577741667	-0,18033733	0,882496625	9430007A20Rik
17203519	4,607708	3,817704	4,299746	4,267105	4,11611	3,801123	4,2417193	4,061446	-0,18027333	0,882535775	---
17543728	4,683175	4,668434	4,703371	4,581635	4,760314	4,172557	4,6849933	4,504835333	-0,180158	0,88260633	Gm9112
17369194	3,375666	3,050641	3,311579	3,072559	3,130311	2,994875	3,245962	3,065915	-0,180047	0,88267424	---
17526978	3,720001	3,357111	3,621075	3,401031	3,357041	3,400035	3,5660623	3,386035667	-0,18002667	0,882686681	Mir34c
17551161	6,846785	6,568846	7,168373	6,538093	6,874325	6,631625	6,8613347	6,681347667	-0,179987	0,88271095	---
17204593	4,292295	4,733852	4,586151	4,395582	4,186589	4,49039	4,5374327	4,357520333	-0,17991233	0,882756636	---
17209037	6,358291	6,561509	6,174221	6,03465	6,27443	6,245231	6,3646737	6,184770333	-0,17990333	0,882762143	---
17310259	8,987888	8,762694	8,785981	8,854511	8,430388	8,711993	8,845521	8,665630667	-0,17989033	0,882770098	Prlr
17262050	4,055538	4,136733	3,902458	4,092596	3,722352	3,740166	4,0315763	3,851704667	-0,17987167	0,88278152	Adam19 // Adam19
17549406	4,589016	4,970016	4,618241	4,335989	4,692257	4,609505	4,7257577	4,545917	-0,17984067	0,882800489	---
17549804	4,589016	4,970016	4,618241	4,335989	4,692257	4,609505	4,7257577	4,545917	-0,17984067	0,882800489	---
17389851	6,06941	5,814468	6,136842	5,904584	5,886301	5,690531	6,0069067	5,827138667	-0,179768	0,882844955	Vps18 // Vps18
17494706	4,659372	3,932856	4,219853	3,98436	3,915582	4,37291	4,2706937	4,090950667	-0,179743	0,882860254	Olfr705
17318728	6,729056	6,471865	6,687771	6,550828	6,54424	6,254701	6,629564	6,449923	-0,179641	0,882922675	Zfp251
17502039	5,634112	5,738238	5,946661	5,473081	5,839927	5,467115	5,7730037	5,593374333	-0,17962933	0,882929815	Rab3a
17239682	4,390259	4,482722	4,532089	4,385084	4,259084	4,222068	4,4683567	4,288745333	-0,17961133	0,882940831	4930455C13Rik
17211256	3,800148	3,692403	3,627137	3,488439	3,627137	3,465361	3,7065627	3,526979	-0,17958367	0,882957764	---
17535529	5,538586	5,381112	5,326084	5,170749	5,231961	5,304405	5,4152607	5,235705	-0,17955567	0,8829749	F8a
17549600	6,136764	5,789691	5,554572	5,703651	5,674096	5,564653	5,827009	5,647466667	-0,17954233	0,882983061	---
17399474	8,718422	8,916748	8,976398	8,89252	8,441792	8,738647	8,8705227	8,690986333	-0,17953633	0,882986733	Pmvk
17254629	5,231046	4,701381	5,088206	4,881872	4,787806	4,812682	5,0068777	4,827453333	-0,17942433	0,883055284	---
17313788	6,819658	6,863037	6,91136	6,690506	6,690178	6,675106	6,864685	6,685263333	-0,17942167	0,883056916	Fam118a
17418952	8,944805	9,246486	9,157663	9,037582	8,922725	8,850456	9,116318	8,936921	-0,179397	0,883072015	Yars
17548975	4,599358	4,791871	4,58112	4,517138	4,479215	4,43782	4,6574497	4,478057667	-0,179392	0,883075075	Capza1 // Capza1
17204961	3,1604	3,437281	3,6661	3,686994	3,019761	3,018903	3,4212603	3,241886	-0,17937433	0,883085889	---
17220877	4,83296	5,03101	5,11685	4,689569	4,794436	4,958763	4,9936067	4,814256	-0,17935067	0,883100376	Gm10516
17450005	5,292865	5,350209	5,443563	5,13467	5,138521	5,275431	5,3622123	5,182874	-0,17933833	0,883107925	A930011G23Rik
17387829	4,35731	3,627569	4,17309	3,721554	3,735834	4,162595	4,0526563	3,873327667	-0,17932867	0,883113843	Olfr1179
17209741	5,477832	5,687572	6,337205	5,141471	6,201437	5,621763	5,834203	5,654890333	-0,17931267	0,883123637	---
17428659	9,16334	9,274832	9,167215	8,923675	9,059008	9,084805	9,2017957	9,022496	-0,17929967	0,883131594	Nasp
17530094	5,020601	4,964518	5,032275	4,88027	4,736226	4,863047	5,005798	4,826514333	-0,17928367	0,883141389	Cldn18
17542501	6,506033	6,742766	6,613367	6,602729	6,587902	6,13374	6,620722	6,441457	-0,179265	0,883152816	Renbp
17202567	3,868943	4,260561	4,011998	3,90545	3,668465	4,029792	4,0471673	3,867902333	-0,179265	0,883152816	---
17209165	6,221086	5,973955	6,186775	6,998998	5,7324	5,112664	6,127272	5,948020667	-0,17925133	0,883161182	---
17442942	10,50765	10,71153	10,60553	10,35491	10,45617	10,476	10,608237	10,42902667	-0,17921	0,883186485	Snora15
17306835	7,163526	7,410354	7,385721	7,272221	7,005121	7,144658	7,319867	7,140666667	-0,17920033	0,883192402	Rabggta
17477884	5,169494	4,971331	5,511235	4,937706	5,062239	5,114563	5,2173533	5,038169333	-0,179184	0,883202401	Hsd17b14
17311649	6,921435	7,437539	6,982872	6,816943	6,924632	7,062791	7,1139487	6,934788667	-0,17916	0,883217094	---
17508174	6,184075	6,089875	5,64236	5,541409	5,915408	5,922093	5,9721033	5,79297	-0,17913333	0,88323342	Ido2
17461112	4,368701	4,162675	4,08149	4,552554	3,667484	3,855462	4,2042887	4,025166667	-0,179122	0,883240358	---
17438227	4,078335	4,01396	3,999099	4,055707	3,654632	3,843847	4,0304647	3,851395333	-0,17906933	0,883272602	Gm6116
17417336	5,749784	5,541616	5,630589	5,479546	5,255794	5,649445	5,640663	5,461595	-0,179068	0,883273418	Kncn
17530540	4,652984	5,183599	4,925824	4,600318	4,975999	4,648918	4,9208023	4,741745	-0,17905733	0,883279949	Col6a5
17234856	4,631187	4,259729	3,794924	4,124805	4,10928	3,914985	4,2286133	4,04969	-0,17892333	0,883361993	Olfr1352
17491697	3,780365	3,514811	3,79059	3,680006	3,416019	3,45304	3,6952553	3,516355	-0,17890033	0,883376076	Mir344d-1
17498271	7,815217	8,037402	8,164577	7,859213	7,89066	7,730701	8,005732	7,826858	-0,178874	0,883392201	Cars
17432708	3,645595	3,436739	3,293873	3,261618	3,356927	3,221303	3,4587357	3,279949333	-0,17878633	0,883445882	---
17363129	3,728412	3,650378	3,582829	3,404829	3,499097	3,521478	3,653873	3,475134667	-0,17873833	0,883475276	Olfr1447
17519347	3,261344	3,621113	3,613277	3,418348	3,276352	3,264895	3,498578	3,319865	-0,178713	0,88349079	---
17357899	2,827314	3,18537	3,178833	2,84847	2,851235	2,955959	3,063839	2,885221333	-0,17861767	0,883549173	Olfr1450
17447698	5,098706	4,815403	4,586544	4,845648	4,45303	4,666161	4,833551	4,654946333	-0,17860467	0,883557134	Clnk
17487670	4,666263	4,329275	4,263624	4,289832	4,295376	4,138431	4,4197207	4,241213	-0,17850767	0,883616543	Vmn1r177
17431693	4,540896	3,957282	4,730366	4,065889	4,618293	4,009287	4,4095147	4,231156333	-0,17835833	0,88370801	Cela3b
17268088	3,442405	3,790514	4,197428	3,910659	3,40722	3,577412	3,8101157	3,631763667	-0,178352	0,88371189	---
17507790	4,358531	4,32695	4,802737	4,238327	4,356624	4,358212	4,4960727	4,317721	-0,17835167	0,883712094	---
17351839	9,236066	9,134074	9,28564	8,966854	9,027162	9,126731	9,2185933	9,040249	-0,17834433	0,883716586	---
17314626	6,15306	5,986728	6,187521	5,822119	6,05987	5,910307	6,109103	5,930765333	-0,17833767	0,88372067	9330020H09Rik
17481232	10,49498	10,65228	10,74163	10,38562	10,50225	10,46604	10,62963	10,45130333	-0,17832667	0,883727408	Hbb-b1
17216351	3,576495	3,436766	3,710977	3,433946	3,408163	3,347278	3,574746	3,396462333	-0,17828367	0,883753748	---
17300362	6,105704	6,580274	7,007739	6,893061	6,026959	6,238874	6,5645723	6,386298	-0,17827433	0,883759465	---
17326914	4,36637	4,394166	4,971738	4,537619	4,296099	4,363769	4,5774247	4,399162333	-0,17826233	0,883766816	Krtap13-1
17503254	9,6981	10,0522	9,90462	9,675438	9,581308	9,863399	9,8849733	9,706715	-0,17825833	0,883769266	Klf1
17541919	6,906681	6,310201	6,719406	6,445269	6,420145	6,536122	6,6454293	6,467178667	-0,17825067	0,883773963	Gpr101
17432554	5,200202	4,646104	4,923366	4,603581	4,589379	5,042017	4,923224	4,744992333	-0,17823167	0,883785602	1700012P22Rik
17334059	5,842876	5,233129	5,756916	5,425226	5,550179	5,32285	5,6109737	5,432751667	-0,178222	0,883791524	6330415G19Rik
17202995	6,90266	6,685359	6,536946	6,771399	6,319258	6,499679	6,7083217	6,530112	-0,17820967	0,883799079	---
17205149	5,625706	5,591362	5,556663	5,620656	5,817466	4,800998	5,5912437	5,41304	-0,17820367	0,883802755	---
17209339	5,625706	5,591362	5,556663	5,620656	5,817466	4,800998	5,5912437	5,41304	-0,17820367	0,883802755	---
17323548	8,513686	8,357272	8,204874	8,163683	8,093009	8,284732	8,3586107	8,180474667	-0,178136	0,883844209	Smpd4
17374283	8,041901	8,158717	8,01326	7,770472	7,878474	8,030535	8,0712927	7,893160333	-0,17813233	0,883846455	2410042D21Rik
17481342	4,604782	4,471068	4,14194	4,078742	4,266541	4,338115	4,40593	4,227799333	-0,17813067	0,883847476	Olfr669
17326062	4,868458	4,69086	4,587689	4,672152	4,559196	4,38131	4,715669	4,537552667	-0,17811633	0,883856257	Retnlb
17240027	7,523446	7,665862	7,483656	7,387686	7,293879	7,457062	7,5576547	7,379542333	-0,17811233	0,883858708	Trmt11
17549050	3,810043	3,999223	4,117427	3,868335	3,871459	3,652626	3,9755643	3,797473333	-0,178091	0,883871778	---
17549056	3,810043	3,999223	4,117427	3,868335	3,871459	3,652626	3,9755643	3,797473333	-0,178091	0,883871778	---
17475431	4,762717	4,841825	4,844714	4,632284	4,769842	4,512879	4,8164187	4,638335	-0,17808367	0,883876271	Nlrp9a
17370897	7,572131	7,26043	7,320971	7,239713	7,272858	7,106712	7,3845107	7,206427667	-0,178083	0,883876679	Gpd2
17462889	5,849245	6,127069	5,886458	5,612046	5,814618	5,902028	5,9542573	5,776230667	-0,17802667	0,883911193	C1s // C1s
17388010	4,737496	4,40819	4,246313	4,383202	4,293276	4,181446	4,4639997	4,285974667	-0,178025	0,883912214	Olfr1264
17550737	3,73133	3,335147	3,322506	3,335147	3,302498	3,217331	3,4629943	3,284992	-0,17800233	0,883926101	---
17371993	5,653716	5,311161	5,736159	5,60195	5,323779	5,241523	5,567012	5,389084	-0,177928	0,883971646	Gpr155
17372354	7,463636	7,401459	7,358298	7,324635	7,174704	7,190442	7,4077977	7,229927	-0,17787067	0,884006776	Ssfa2
17266335	8,154255	8,321173	8,195642	8,171887	7,937008	8,028579	8,22369	8,045824667	-0,17786533	0,884010044	Traf4
17330046	7,337005	7,179594	7,146671	6,990161	7,107797	7,031756	7,22109	7,043238	-0,177852	0,884018214	Sec22a
17211517	4,135228	4,002962	3,795509	3,759966	3,655677	3,984574	3,9778997	3,800072333	-0,17782733	0,884033329	---

17279553	6,143639	6,47718	6,418249	6,04059	6,15736	6,307692	6,346356	6,168547333	-0,17780867	0,884044767	Zfp386 // Zfp386
17240098	4,357758	4,571842	4,028376	4,272973	4,133554	4,018087	4,3193253	4,141538	-0,17778733	0,88405784	Gm3258
17210061	5,030793	5,008187	4,860856	4,733076	5,462831	4,170576	4,966612	4,788827667	-0,17778433	0,884059678	---
17465733	4,494851	4,312421	4,529715	4,224736	4,392015	4,187076	4,4456623	4,267942333	-0,17772	0,884099101	Npn2
17389117	3,708953	3,493595	3,667752	3,391705	3,433491	3,51196	3,6234333	3,445718667	-0,17771467	0,88410237	---
17450979	7,60159	7,711641	7,462678	7,41026	7,505105	7,327421	7,5919697	7,414262	-0,17770767	0,884106659	Pgam5
17423207	5,750117	5,359838	5,135121	6,045808	4,990574	4,67558	5,4150253	5,237320667	-0,17770467	0,884108498	Mir3471-1 // Mir3471-1
17201115	6,85242	7,191585	6,9408	6,798733	6,646734	7,00645	6,994935	6,817305667	-0,17762933	0,884154665	---
17414649	4,840115	4,596787	4,810887	4,801942	4,405222	4,507859	4,749263	4,571674333	-0,17758867	0,884179588	---
17487078	3,467009	3,08166	3,485206	3,002371	3,14904	3,349746	3,344625	3,167052333	-0,17757267	0,884189394	Psg23
17549090	3,599174	3,263715	3,231808	3,281502	3,200428	3,080063	3,364899	3,187331	-0,177568	0,884192254	---
17383588	8,15663	8,316304	8,503572	8,47757	8,03554	7,930724	8,325502	8,147944667	-0,17755733	0,884198791	Ccbl1
17249256	3,84539	3,317113	3,485844	3,395722	3,083596	3,636362	3,549449	3,371893333	-0,17755567	0,884199812	Olfr51
17421394	8,88271	9,435926	8,9277	8,736118	8,892862	9,084887	9,082112	8,904622333	-0,17748967	0,884240263	Gm13034
17204035	4,559132	5,175706	3,796094	4,586312	4,628913	3,783264	4,5103107	4,332829667	-0,177481	0,884245575	---
17446539	8,393688	8,505697	8,454851	8,321917	8,176465	8,323481	8,451412	8,273954333	-0,17745767	0,884259877	Paxip1
17210755	3,785306	4,828513	4,290172	3,987154	4,122902	4,261586	4,3013303	4,123880667	-0,17744967	0,88426478	---
17431282	5,11162	5,018207	5,288455	5,237725	4,908519	4,739828	5,1394273	4,962024	-0,17740333	0,884293179	Extl1
17225189	5,723059	5,392612	5,320955	5,384523	5,321029	5,198881	5,4788753	5,301477667	-0,17739767	0,884296653	---
17379126	4,523226	4,385716	3,983934	3,910301	4,168506	4,281879	4,2976253	4,120228667	-0,17739667	0,884297266	---
17514343	12,26462	12,27012	11,83623	12,04024	11,89149	11,90708	12,123657	11,94627	-0,17738667	0,884303395	---
17272810	5,616483	5,629804	5,628778	5,529424	5,419861	5,393624	5,6250217	5,447636333	-0,17738533	0,884304213	C1qtnf1
17409502	5,014939	4,291369	4,121709	4,264283	3,972746	4,658965	4,4760057	4,298664667	-0,177341	0,884331387	Amy2b
17221265	9,315577	9,098089	9,448505	9,227267	8,799892	9,303069	9,2873903	9,110076	-0,17731433	0,884347733	---
17206883	6,662459	6,899187	6,193102	6,770205	6,543443	5,909294	6,584916	6,407647333	-0,17726867	0,884375727	---
17546905	3,585644	3,453224	4,305694	3,614443	3,716854	3,48152	3,7815207	3,604272333	-0,17724833	0,884388191	Zfy2
17405838	5,753437	5,790896	5,555936	5,581165	5,462496	5,524891	5,7000897	5,522850667	-0,177239	0,884393913	Gm6634
17435114	9,489729	10,2797	9,899643	9,709744	9,554456	9,87324	9,8896907	9,71248	-0,17721067	0,884411282	---
17250028	6,092997	6,39701	6,047903	5,762773	6,107048	6,136504	6,1793033	6,002108333	-0,177195	0,884420886	Sh3bp5l
17454428	6,964754	6,828419	7,149736	6,621175	6,821681	6,968533	6,9809697	6,803796333	-0,17717333	0,884434168	Micall2
17289455	6,445514	6,268492	5,840594	5,81871	5,981881	6,222779	6,1848667	6,00779	-0,17707667	0,884493431	Serf1
17535206	3,251248	3,182099	3,264307	3,052545	3,104778	3,009116	3,2325513	3,055479667	-0,17707167	0,884496496	---
17480214	4,091672	3,77229	3,93647	3,361973	3,765092	4,142239	3,9334773	3,756434667	-0,17704267	0,884514276	---
17235399	5,897525	5,802545	5,965123	5,633922	5,702591	5,797562	5,8883977	5,711358333	-0,17703933	0,88451632	Izumo4
17370951	4,782103	4,283991	4,391096	4,228786	4,54597	4,151374	4,48573	4,30871	-0,17702	0,884528173	---
17269333	5,63082	5,024252	5,611462	5,200372	4,948236	5,587029	5,422178	5,245212333	-0,17696567	0,884561486	Krt36
17317428	3,841821	3,895814	4,173196	3,749552	3,685792	3,944605	3,970277	3,793316333	-0,17696067	0,884564552	Gsdmc
17449546	9,423162	9,394867	9,451257	8,776505	9,622397	9,339527	9,4230953	9,246143	-0,17695233	0,884569661	---
17214593	5,950639	6,116887	6,144707	5,964138	5,697486	6,019779	6,0707443	5,893801	-0,17694333	0,884575179	Stk11ip
17201049	7,423147	7,825084	7,575734	6,892962	7,496015	7,90427	7,6079883	7,431082333	-0,176906	0,88459807	---
17504452	3,128474	3,047415	3,130972	2,994547	2,883324	2,898337	3,102287	2,925402667	-0,17688433	0,884611355	---
17518134	5,168781	5,09831	5,267727	5,025366	5,01173	4,967106	5,1782727	5,001400667	-0,176872	0,884618918	Gm5122
17445440	4,060609	4,216368	4,061248	3,991263	3,870704	3,945813	4,1127417	3,935926667	-0,176815	0,884653869	Steap1
17238525	4,99402	5,109987	5,378835	4,991636	5,043752	4,917022	5,1609473	4,984136667	-0,17681067	0,884656526	---
17513401	5,646611	5,500098	5,951419	5,579756	5,333867	5,654107	5,699376	5,522576667	-0,17679933	0,884663476	Slc38a8
17434163	3,657161	3,376289	3,879199	4,000346	3,099987	3,281954	3,6375497	3,460762333	-0,17678733	0,884670834	---
17545514	4,986216	3,650762	3,923706	4,070681	3,690002	4,269691	4,1868947	4,010124667	-0,17677	0,884681463	---
17532799	4,896333	4,554817	4,849745	4,609523	4,641033	4,520111	4,766965	4,590222333	-0,17674267	0,884698225	Gm14379
17391285	4,332275	4,29332	4,173518	3,913196	4,288649	4,067469	4,266371	4,089771333	-0,17659967	0,88478592	---
17266372	10,24765	10,40298	10,42465	10,15483	10,35228	10,03843	10,358427	10,18184667	-0,17658	0,884797982	Snord42b
17230918	8,827437	8,977194	8,983122	8,533682	8,819525	8,904811	8,929251	8,752672667	-0,17657833	0,884799004	Cenpf
17201609	6,833166	7,301723	7,518497	6,828942	7,151995	7,142737	7,2177953	7,041224667	-0,17657067	0,884803706	---
17366448	5,424756	5,31911	5,180805	5,30949	5,041214	5,044273	5,3082237	5,131659	-0,17656467	0,884807386	Gm13180 // Gm13180
17318695	6,941513	7,175143	7,056624	6,742159	6,874235	7,027269	7,05776	6,881221	-0,176539	0,884823127	Lrrc24
17548000	9,337912	9,34256	9,372522	9,066919	9,240345	9,216242	9,350998	9,174502	-0,176496	0,8848495	Tubb5 // Tubb5
17240834	6,353376	6,212544	6,180399	6,3038	5,682182	6,230877	6,248773	6,072286333	-0,17648667	0,884855225	---
17547279	3,642535	4,107462	3,566132	3,536207	3,894553	3,356113	3,772043	3,595624333	-0,17641867	0,884896932	---
17547398	3,642535	4,107462	3,566132	3,536207	3,894553	3,356113	3,772043	3,595624333	-0,17641867	0,884896932	---
17547439	3,642535	4,107462	3,566132	3,536207	3,894553	3,356113	3,772043	3,595624333	-0,17641867	0,884896932	---
17547452	3,642535	4,107462	3,566132	3,536207	3,894553	3,356113	3,772043	3,595624333	-0,17641867	0,884896932	---
17547460	3,642535	4,107462	3,566132	3,536207	3,894553	3,356113	3,772043	3,595624333	-0,17641867	0,884896932	---
17200525	3,893417	3,208255	3,435014	3,571307	3,174906	3,261251	3,5122287	3,335821333	-0,17640733	0,884903884	---
17336894	4,959665	4,878897	4,744984	4,597453	4,662973	4,793962	4,861182	4,684796	-0,176386	0,884916969	---
17288252	3,211997	3,279294	3,374575	3,244362	3,213019	2,879363	3,288622	3,112248	-0,176374	0,88492433	---
17547519	4,641517	4,927535	4,696977	4,481832	4,589214	4,665867	4,755343	4,578971	-0,176372	0,884925556	Tbc1d15 // Tbc1d15
17542749	3,292909	3,431227	3,393883	3,122888	3,441432	3,024604	3,372673	3,196308	-0,176365	0,88492985	---
17299867	5,170581	5,266846	5,298055	5,063916	4,925414	5,217095	5,2451607	5,068808333	-0,17635233	0,88493762	---
17299944	5,170581	5,266846	5,298055	5,063916	4,925414	5,217095	5,2451607	5,068808333	-0,17635233	0,88493762	---
17205159	3,653863	3,870665	3,818146	3,712658	3,546508	3,554459	3,7808913	3,604541667	-0,17634967	0,884939255	---
17209349	3,653863	3,870665	3,818146	3,712658	3,546508	3,554459	3,7808913	3,604541667	-0,17634967	0,884939255	---
17471151	5,045836	4,937795	4,848103	4,677123	4,662756	4,962809	4,9439113	4,767562667	-0,17634867	0,884939869	Kcna5
17351833	12,35736	12,67105	12,47452	12,2255	12,35872	12,38968	12,500977	12,32463333	-0,17634333	0,88494314	---
17380366	8,108528	7,762789	7,964787	7,87538	7,79055	7,641152	7,945368	7,769027333	-0,17634067	0,884944776	Atp5e // Atp5e
17221012	3,747051	3,634945	3,468966	3,5062	3,461658	3,354113	3,6169873	3,440657	-0,17633033	0,884951114	---
17517282	3,964119	3,825051	4,422942	3,95387	3,888412	3,840919	4,070704	3,894400333	-0,17630367	0,884967472	---
17393876	5,418561	5,790306	5,547404	5,382909	5,256672	5,587928	5,5854237	5,409169667	-0,176254	0,884997939	LOC100861957
17230005	4,124928	4,741741	4,275998	3,985941	4,534462	4,093511	4,380889	4,204638	-0,176251	0,884999779	---
17365331	5,531032	5,434088	5,707367	5,281135	5,45143	5,411285	5,5574957	5,381283333	-0,17621233	0,885023499	Pitx3
17308827	4,746566	3,98011	4,271741	4,253533	3,965218	4,25111	4,3328057	4,156620333	-0,17618533	0,885040062	---
17444682	4,434234	4,946376	4,885776	4,815426	4,465626	4,456782	4,755462	4,579278	-0,176184	0,88504088	Cyp3a57
17353054	4,292213	3,937808	4,058608	3,952039	3,931762	3,876434	4,0962097	3,920078333	-0,17613133	0,88507319	4921528I01Rik
17226748	5,41297	4,930179	5,222386	5,185524	4,810135	5,041516	5,1885117	5,012391667	-0,17612	0,885080143	---
17549576	5,535881	5,392004	5,665767	5,499421	5,470362	5,095649	5,5312173	5,355144	-0,17607333	0,885108773	---
17409617	7,235425	7,38869	7,203094	6,806126	7,243382	7,249507	7,2757363	7,099671667	-0,17606467	0,88511409	Gm19420
17466932	4,891634	5,281657	4,937104	4,782192	4,670667	5,129358	5,0367983	4,860739	-0,17605933	0,885117362	Tril
17206303	6,978487	7,203485	6,791721	6,970056	6,587645	6,887821	6,991231	6,815174	-0,176057	0,885118793	---
17206041	5,231722	5,451163	5,379026	5,20079	5,123108	5,209871	5,3539703	5,177923	-0,17604733	0,885124724	---
17206787	4,210738	3,153102	4,794018	3,494044	4,000248	4,13544	4,0526193	3,876577333	-0,176042	0,885127996	---
17330158	4,815865	5,287253	5,231823	4,816784	4,982092	5,008061	5,111647	4,935645667	-0,17600133	0,885152947	---
17311492	6,864905	6,903442	6,650452	6,446014	6,714283	6,730542	6,8062663	6,630279667	-0,17598667	0,885161945	Med30
17328104	5,930585	6,172267	6,05336	5,869195	5,890628	5,868517	6,0520707	5,876113333	-0,17595733	0,885179943	Socs1
17408856	8,487388	8,562686	8,264799	8,207397	8,157344	8,422268	8,438291	8,262336333	-0,17595467	0,885181579	I830077J02Rik
17200415	6,355897	6,958729	6,502729	6,859502	6,316919	6,113251	6,605785	6,429890667	-0,17589433	0,885218598	---
17201861	6,387781	6,709656	6,930348	6,309043	6,803337	6,387781	6,6759283	6,500053667	-0,17587467	0,885230665	---
17491896	4,94686	4,699356	4,246411	4,472649	4,496044	4,396337	4,6308757	4,45501	-0,17586567	0,885236188	---
17535434	11,13737	11,08006	11,03513	11,0647	10,68566	10,97487	11,084187	10,90841	-0,17577667	0,8852908	Nsdhl
17532619	12,14973	12,47605	12,26405	12,13455	12,0148	12,21315	12,29661	12,12083333	-0,17577667	0,8852908	---
17550564	12,14973	12,47605	12,26405	12,13455	12,0148	12,21315	12,29661	12,12083333	-0,17577667	0,8852908	---
17406752	8,077615	8,101212	8,0419	7,96936	7,856843	7,867201	8,0735757	7,897801333	-0,17577433	0,885292231	Slc25a44
17380082	3,988723	3,467055	3,606557	3,452002	3,787969	3,295093	3,687445	3,511688	-0,175757	0,885302868	1700007M16Rik // 1700007M16Rik
17331876	3,529584	3,788968	3,50721	3,391418	3,501116	3,405965	3,6085873	3,432833	-0,17575433	0,885304504	Krtap8-2
17516408	3,516971	3,159378	3,280236	3,06057	3,211371	3,157429	3,3188617	3,143123333	-0,17573833	0,885314323	Mir100
17383832	5,823528	6,027375	5,940627	5,586545	5,565091	6,112745	5,93051	5,754793667	-0,17571633	0,885327823	Prrc2b // Prrc2b
17438920	10,99888	10,94392	10,83289	10,74198	10,6699	10,83691	10,92523	10,74959667	-0,17563333	0,885378759	Afm
17429581	4,14892	4,217029	4,312816	4,383748	3,870173	3,897973	4,226255	4,050631333	-0,17562367	0,885384691	Gm12888
17357715	5,241523	5,203991	5,033045	5,047139	4,835312	5,069285	5,1595197	4,983912	-0,17560767	0,88539451	---
17438599	3,722773	3,213799	3,632053	3,270565	3,342182	3,429321	3,522875	3,347356	-0,175519	0,885448927	---
17200055	4,789452	5,489507	6,156971	5,663316	5,145658	5,10048	5,4786433	5,303151333	-0,175492	0,885465499	---
17440350	4,054818	5,06664	4,764067	5,060863	4,264306	4,033913	4,6285083	4,453027333	-0,175481	0,88547225	Zfp932
17480489	6,400346	6,225543	6,497097	6,227038	6,259494	6,11012	6,3743287	6,198884	-0,17544467	0,88549455	---
17468879	7,200871	6,983435	7,039664	6,831267	6,932633	6,933776	7,0746567	6,899225333	-0,17543133	0,885502734	Vmn1r43
17403514	8,65747	8,72687	8,711512	8,385826	8,645002	8,538829	8,6986173	8,523219	-0,17539833	0,885522989	Ssx2ip
17211367	4,917627	4,920765	5,102425	4,838139	4,902092	4,674488	4,9802723	4,804906333	-0,175366	0,885542836	Mir133b
17547515	3,671961	3,789248	3,863363	3,441525	3,509029	3,847943	3,7748573	3,599499	-0,17535833	0,885547541	Gm19894
17337063	7,807416	8,240562	7,965617	7,827136	7,76504	7,895346	8,0045317	7,829174	-0,17535767	0,885547951	---
17283685	6,763031	6,706513	6,857826	6,65881	6,549175	6,593369	6,77579	6,600451333	-0,17533867	0,885559613	Gsc
17405746	8,50588	8,488262	8,309988	8,066347	8,260586	8,451288	8,43471	8,259407	-0,175303	0,885581507	Rarres1
17479301	4,730899	4,168875	4,237264	4,392206	3,802063	4,416893	4,3790127	4,203720667	-0,175292	0,885588259	Mir7-2
17487752	5,232764	4,776188	4,929799	4,823058	4,731617	4,858307	4,9795837	4,804327333	-0,17525633	0,885610153	Gm4763
17496857	6,58328	6,254894	6,516346	6,07995	6,43419	6,31476	6,4515067	6,2763	-0,17520667	0,885640642	Cox6a2
17208903	4,13967	3,186575	3,382055	3,512772	3,080678	3,589434	3,5694333	3,394294667	-0,17513867	0,885682386	---
17306865	10,06516	9,901811	10,12537	10,07652	9,51263	9,977787	10,03078	9,855645667	-0,17513467	0,885684842	---
17547468	3,175944	3,855273	3,300228	2,904571	3,977901	2,923788	3,443815	3,268753333	-0,17506167	0,885729659	1700040F15Rik
17460360	4,541442	4,458432	4,827455	4,288993	4,613148	4,400052	4,6091097	4,434064333	-0,17504533	0,885739686	Figla
17472345	3,48862	3,375644	4,022563	3,421115	3,497154	3,443514	3,6289423	3,453927667	-0,17501467	0,885758514	Gm5724
17471480	5,381207	5,448782	5,414264	5,028855	5,488585	5,201846	5,414751	5,239762	-0,174989	0,885774273	Gm15987
17515019	6,268147	6,336852	6,364798	6,730544	5,787012	5,927279	6,3232657	6,148278333	-0,17498733	0,885775296	Pin1
17358962	9,2284	9,44782	9,2869	9,189356	9,124368	9,124441	9,32104	9,146055	-0,174985	0,885776729	Btaf1
17424937	8,583795	8,300071	8,285293	8,018566	8,193117	8,432944	8,3897197	8,214875667	-0,174844	0,885863303	Exosc3
17292960	5,098827	4,915617	4,988389	4,686458	4,88191	4,909997	5,0009443	4,826121667	-0,17482267	0,885876403	---
17314115	6,035811	6,289405	6,952785	6,388231	6,137686	6,227781	6,4260003	6,251232667	-0,17476767	0,885910176	Adm2
17321966	6,183142	6,061227	6,493142	6,151911	6,01424	6,047256	6,245837	6,071135667	-0,17470133	0,88595091	Krt5
17248971	5,431743	4,987889	5,040439	4,785855	5,267506	4,882616	5,153357	4,978659	-0,174698	0,885952957	Olfr1388
17421145	4,658448	4,496103	5,106596	4,462166	4,909551	4,365451	4,7537157	4,579056	-0,17465967	0,885976497	LOC545691

17309661	3,983932	4,169708	4,148743	3,749012	4,00086	4,028571	4,1007943	3,926147667	-0,17464667	0,885984481 1700108J01Rik
17499736	4,695477	4,428317	4,745539	4,381191	4,478665	4,485538	4,623111	4,448464667	-0,17464633	0,885984685 Defa25
17267466	4,98429	5,07939	4,842601	4,751093	4,836855	4,794441	4,9687603	4,794129667	-0,17463067	0,885994307 Fam33a
17344833	4,690381	4,559931	4,91339	4,546323	4,464108	4,629379	4,721234	4,546603333	-0,17463067	0,885994307 Olfr91
17330850	7,455908	6,796813	7,394002	7,087481	7,068928	6,966442	7,2155743	7,040950333	-0,174624	0,885998401 ---
17454049	8,141134	8,509303	8,44535	8,234313	8,152131	8,185493	8,3652623	8,190645667	-0,17461667	0,886002904 Gnb2
17549494	7,224999	7,296288	7,238346	7,013502	6,759871	7,462433	7,253211	7,078602	-0,174609	0,886007613 ---
17544164	6,47661	6,24822	5,845549	6,154352	5,847148	6,045073	6,1901263	6,015524333	-0,174602	0,886011912 Rps6ka6
17245895	8,127379	8,173444	8,050527	7,815945	7,890099	8,121542	8,1171167	7,942528667	-0,174588	0,88602051 Inhbc
17460756	4,147254	3,952017	3,724356	3,651848	3,779847	3,868312	3,941209	3,766669	-0,17454	0,886049989 Vmn1r46
17491564	6,853848	6,670741	6,993413	6,682031	6,611066	6,701427	6,839334	6,664841333	-0,17449267	0,88607906 ---
17286278	4,334822	4,016747	4,119738	4,083702	3,856668	4,00756	4,1571023	3,982643333	-0,174459	0,886099738 4930519D14Rik
17350801	4,521317	3,991451	4,46174	4,253257	4,023402	4,174516	4,324836	4,150391667	-0,17444433	0,886108746 Gm19500
17203773	5,393953	5,203255	4,718209	5,043461	4,496231	5,252637	5,105139	4,930776333	-0,17436267	0,886158907 ---
17483192	5,521259	4,855196	5,021746	4,747772	4,925206	5,202365	5,1327337	4,958447667	-0,174286	0,886206 Sez6l2
17549538	6,681762	6,813325	6,877746	6,539443	6,550955	6,759631	6,7909443	6,616676333	-0,174268	0,886217057 ---
17549690	6,681762	6,813325	6,877746	6,539443	6,550955	6,759631	6,7909443	6,616676333	-0,174268	0,886217057 ---
17537507	5,336461	6,259987	5,708618	5,674818	5,417254	5,690221	5,7683553	5,594097667	-0,17425767	0,886223405 ---
17296080	5,280529	5,157809	4,854395	4,74478	4,97692	5,048358	5,0975777	4,923352667	-0,174225	0,886243472 ---
17315946	8,673283	8,835344	8,632152	8,490085	8,524465	8,603554	8,713593	8,539368	-0,174225	0,886243472 Dnajc21
17341987	7,678583	7,891631	7,739144	7,621827	7,592855	7,572118	7,769786	7,5956	-0,174186	0,886267429 Gfer
17206347	4,629065	5,470372	4,355717	5,264641	4,126224	4,541845	4,8183847	4,644236667	-0,174148	0,886290774 ---
17204787	3,818609	4,114026	3,92459	4,056997	3,611327	3,66646	3,9524083	3,778261333	-0,174147	0,886291388 ---
17291078	3,858147	3,408237	3,175794	3,145702	3,546317	3,227767	3,480726	3,306595333	-0,17413067	0,886301422 ---
17306866	8,130165	7,243382	7,829861	6,957011	7,785364	7,93874	7,7344693	7,560371667	-0,17409767	0,886321696 ---
17431476	2,899583	3,018115	2,991851	2,821094	2,755379	2,810837	2,9698497	2,79577	-0,17407967	0,886332754 ---
17370273	7,827816	7,806613	7,807328	7,554193	7,645596	7,719758	7,813919	7,639849	-0,17407	0,886338693 Ndufa8 // Ndufa8
17318140	6,456769	6,208491	6,147738	5,899375	6,231419	6,160159	6,2709993	6,096984333	-0,174015	0,886372483 ---
17370376	5,372604	5,088458	4,959506	5,29983	4,785748	4,813028	5,1401893	4,966202	-0,17398733	0,886389482 Olfr368
17209921	4,896333	4,483787	5,360408	4,690091	4,488058	5,040438	4,9135093	4,739529	-0,17398033	0,886393782 ---
17402013	4,570077	4,417921	4,493655	4,514464	4,220572	4,224689	4,4938843	4,319908333	-0,173976	0,886396445 ---
17335330	10,85315	10,84292	10,86721	10,68417	10,70965	10,64764	10,854427	10,68048667	-0,17394	0,886418564 Rpl10a
17402477	3,2868	3,204118	3,297086	2,913646	3,196705	3,1559	3,262668	3,088750333	-0,17391767	0,886432286 Mir302c
17514826	6,10608	6,245605	6,404125	6,122519	6,158935	5,95262	6,2519367	6,078024667	-0,173912	0,886435767 Taf1d
17333731	7,859511	8,071594	7,84924	7,758098	7,881473	7,619071	7,9267817	7,752880667	-0,173901	0,886442526 Fpr2
17403811	5,460131	5,408662	5,196669	5,236531	5,124563	5,1827	5,355154	5,181264667	-0,17388933	0,886449695 Lrriq3
17415424	4,076686	3,814821	3,897717	3,82786	3,749605	3,690213	3,9297413	3,755892667	-0,17384867	0,886474682 ---
17387626	3,826098	3,330803	3,890741	3,441332	3,581354	3,503466	3,6825473	3,508717333	-0,17383	0,886486152 ---
17417569	5,60751	5,247183	5,496861	5,458862	5,09602	5,275209	5,450518	5,276697	-0,173821	0,886491682 ---
17402979	4,936219	5,004853	4,877025	4,586586	4,732338	4,977785	4,9393657	4,765569667	-0,173796	0,886507044 ---
17504374	7,851731	7,836227	8,036501	7,790193	7,707672	7,705206	7,908153	7,734357	-0,173796	0,886507044 ---
17532649	11,10381	11,58822	11,40783	11,30192	11,18703	11,08972	11,36662	11,19289	-0,17373	0,886547601 ---
17206929	6,164065	5,796471	6,505086	5,724298	5,857356	6,362843	6,1552073	5,981499	-0,17370833	0,886560915 ---
17549362	6,00864	5,641753	5,505424	5,414562	5,639675	5,58046	5,7186057	5,544899	-0,17370667	0,886561939 ---
17549754	6,00864	5,641753	5,505424	5,414562	5,639675	5,58046	5,7186057	5,544899	-0,17370667	0,886561939 ---
17429918	4,199048	4,096483	4,386205	4,028535	4,134376	3,99777	4,2272453	4,053560333	-0,173685	0,886575254 ---
17401883	2,712228	3,023008	2,948605	2,720371	2,732012	2,710436	2,8946137	2,720939667	-0,173674	0,886582014 ---
17294135	7,42153	7,03129	6,904232	6,971468	6,836815	7,025962	7,1190173	6,945348333	-0,173669	0,886585087 Srd5a1
17338612	4,646601	4,869072	4,959244	4,663422	4,834659	4,45592	4,8249723	4,651333667	-0,17363867	0,886603728 4930571B16Rik // 4930571B16Rik
17314693	8,108424	8,172525	8,147136	7,933273	8,005542	7,968363	8,142695	7,969059333	-0,17363567	0,886605571 Tuba1c
17405082	4,259322	4,179165	4,36597	3,932727	4,380196	3,970832	4,2681523	4,094585	-0,17356733	0,886647566 Slc7a11
17461153	6,939476	7,319808	7,843928	7,145266	7,184805	7,25246	7,3677373	7,194177	-0,17356033	0,886651868 ---
17377324	6,90314	6,659048	6,994998	6,796535	6,629674	6,610355	6,8523953	6,678854667	-0,17354067	0,886663955 ---
17415428	4,608452	4,707559	4,484348	4,367893	4,490787	4,421266	4,6001197	4,426648667	-0,173471	0,886706773 ---
17538186	4,354065	4,340115	4,487884	3,994626	4,340647	4,326405	4,3940213	4,220559333	-0,173462	0,886712304 ---
17549262	4,651635	4,43138	5,032897	4,595876	4,5014	4,49834	4,705304	4,531872	-0,173432	0,886730743 ---
17549302	4,651635	4,43138	5,032897	4,595876	4,5014	4,49834	4,705304	4,531872	-0,173432	0,886730743 ---
17319226	5,244071	5,19593	5,270024	5,006214	5,019884	5,163761	5,236675	5,063286333	-0,17338867	0,886757378 Kcnj4
17487149	8,587277	8,696862	8,83314	8,498394	8,559403	8,539323	8,7057597	8,532373333	-0,17338633	0,886758812 Foxa3
17450328	5,278974	4,953372	5,210009	4,869408	4,948623	5,104248	5,1474517	4,974093	-0,17335867	0,886775817 1700016H13Rik
17535048	4,042289	4,095173	4,022955	3,684284	3,904351	4,051762	4,0534723	3,880132333	-0,17334	0,886787291 Cd40lg
17414318	4,884538	4,763885	4,91426	4,587752	4,628714	4,82625	4,8542277	4,680905333	-0,17332233	0,886798151 Gm12511
17295027	5,251141	4,942093	5,019656	4,899484	4,841876	4,951584	5,0709633	4,897648	-0,17331533	0,886802453 AW495222
17206249	5,066683	4,94703	5,758187	5,60957	4,898898	4,743652	5,2573	5,08404	-0,17326	0,886836467 ---
17456485	8,932693	9,258755	9,218317	8,857075	8,936319	9,096658	9,1365883	8,963350667	-0,17323767	0,886850195 Arf5
17463611	7,794777	7,579607	7,476261	7,276146	7,231954	7,822857	7,6168817	7,443652333	-0,17322933	0,886855318 Prh1
17447098	9,294074	9,511181	9,395665	9,226116	9,398713	9,056458	9,4003067	9,227095667	-0,173211	0,886866588 ---
17286695	5,156888	5,081596	4,917167	5,182909	4,753992	4,699308	5,0518837	4,878736333	-0,17314733	0,886905726 5033403F01Rik
17359956	7,793474	7,892928	8,146467	7,985966	7,828804	7,498691	7,9442897	7,771153667	-0,173136	0,886912694 ---
17332251	4,732521	4,749208	4,378777	4,348607	4,604757	4,387751	4,6201687	4,447038333	-0,17313033	0,886916177 1810053B23Rik
17331585	3,896925	4,293172	4,244824	3,953304	4,060244	3,901982	4,1449737	3,971843333	-0,17313033	0,886916177 E330011O21Rik
17430960	5,659574	5,895952	6,106208	5,723988	5,635484	5,782894	5,8872447	5,714122	-0,17312267	0,886920891 ---
17277801	5,57336	5,811692	5,420127	5,377618	5,47399	5,434269	5,6017263	5,428625667	-0,17310067	0,886934416 Spata7
17208149	4,575756	5,328951	4,053812	3,892632	4,653107	4,893557	4,6528397	4,479765333	-0,17307433	0,886950605 ---
17227464	5,730855	5,767102	5,903564	5,625948	5,62629	5,6301	5,800507	5,627446	-0,173061	0,886958802 5730559C18Rik
17357928	4,208111	3,672313	3,681837	3,645676	3,650935	3,746496	3,854087	3,681035667	-0,17305133	0,886964745 Olfr1484
17549194	3,46856	3,409804	3,478467	3,301931	3,231009	3,304806	3,452277	3,279248667	-0,17302833	0,886978885 ---
17205295	3,294092	3,243041	4,182651	3,463586	3,214686	3,52258	3,5732613	3,400284	-0,17297733	0,887010241 ---
17209485	3,294092	3,243041	4,182651	3,463586	3,214686	3,52258	3,5732613	3,400284	-0,17297733	0,887010241 ---
17363407	11,92253	11,94529	11,93069	11,75715	11,80918	11,71331	11,932837	11,75988	-0,17295667	0,887022948 Anxa1
17331173	8,14867	8,166647	8,156138	8,090618	7,902203	7,960043	8,1571517	7,984288	-0,17286367	0,887080129 St3gal6
17466303	3,531312	3,929382	3,761862	3,570504	3,673548	3,459918	3,740852	3,56799	-0,172862	0,887081154 Prss3
17258555	4,876676	4,626969	4,960115	4,384048	4,927119	4,634222	4,8212533	4,648463	-0,17279033	0,887125222 Cdk3-ps
17211857	5,463519	4,855729	5,502817	5,273487	5,186327	4,84394	5,2740217	5,101251333	-0,17277033	0,88713752 4933424G06Rik
17536165	5,548048	5,691279	5,47075	5,381523	5,420897	5,389396	5,5700257	5,397272	-0,17275367	0,887147768 1600014K23Rik
17305556	3,545016	3,338943	3,180454	3,222989	3,131724	3,191447	3,3548043	3,182053333	-0,172751	0,887149408 ---
17414747	4,75647	4,576856	4,803207	4,320838	4,50359	4,794018	4,7121777	4,539482	-0,17269567	0,887183435 Orm2
17204561	3,96525	3,994832	3,720061	3,583635	3,651848	3,926619	3,893381	3,720700667	-0,17268033	0,887192864 ---
17487982	9,20585	9,165661	9,364203	9,136271	9,128421	8,952995	9,245238	9,072562333	-0,17267567	0,887195734 Bckdha
17275686	3,555307	3,29844	3,311032	3,21854	3,202862	3,22548	3,3882597	3,215627333	-0,17263233	0,887222382 ---
17479142	4,654447	4,667248	4,732617	4,500086	4,507906	4,528467	4,6847707	4,512153	-0,17261767	0,887231402 Gm5334
17236049	4,939436	4,798913	4,990026	4,628157	4,772622	4,809748	4,9094583	4,736842333	-0,172616	0,887232427 Rfx4
17299983	6,633845	5,994515	6,026031	5,95597	5,97799	6,202598	6,2181303	6,045519333	-0,172611	0,887235502 ---
17431907	6,167366	5,647555	6,08698	5,887858	5,599207	5,897229	5,9673003	5,794764667	-0,17253567	0,887281832 ---
17210481	5,367812	4,541869	5,46536	5,069232	4,627578	5,160755	5,1250137	4,952521667	-0,172492	0,887308688 ---
17495368	6,9893	7,239315	6,573627	6,549759	6,954957	6,780083	6,9340807	6,761599667	-0,172481	0,887315454 Nup35 // Nup35
17544290	3,347197	3,61103	3,923209	3,171738	3,838924	3,353367	3,6271453	3,454676333	-0,172469	0,887322834 ---
17332656	7,073098	7,414119	6,996484	7,068117	6,662851	7,235381	7,1612337	6,988783	-0,17245067	0,88733411 A630089N07Rik // A630089N07Rik
17523676	6,515557	6,79961	6,98759	6,377287	6,458349	6,949972	6,7675857	6,595202667	-0,172383	0,88737573 ---
17423939	4,214012	4,352835	4,093843	3,939409	4,144628	4,059531	4,22023	4,047856	-0,172374	0,887381265 Lingo2
17345007	4,811391	4,86139	4,781276	4,977438	4,309659	4,650121	4,818019	4,645672667	-0,17234633	0,887398283 Opn5
17311094	3,492445	3,84645	3,499101	3,500491	3,353998	3,466714	3,6126653	3,440401	-0,17226433	0,887448722 ---
17344336	8,61407	8,705056	8,729751	8,574056	8,434677	8,523477	8,682959	8,510736667	-0,17222233	0,887474558 Tcf19
17279582	2,890514	3,087593	3,203526	2,709239	3,111972	2,84385	3,0605443	2,888353667	-0,17219067	0,887494038 ---
17378348	7,658419	7,539878	7,663597	7,469845	7,442427	7,433061	7,6206313	7,448444333	-0,172187	0,887496294 Trp53inp2
17274247	10,13237	10,23701	9,913334	9,674345	9,997278	10,09457	10,094238	9,922064333	-0,17217367	0,887504496 Top2a // Top2a
17299082	3,651848	3,152213	3,411633	3,235129	3,386483	3,077606	3,4052313	3,233072667	-0,17215867	0,887513724 ---
17366589	5,276218	4,69749	5,195277	4,785854	5,035163	4,831503	5,0563283	4,884173333	-0,172155	0,887515979 A230108P19Rik
17294289	6,015861	5,874837	6,143205	5,753648	5,722436	6,041378	6,011301	5,839154	-0,172147	0,887520901 Nkd2
17380102	9,41688	9,45737	9,767461	9,280495	9,429357	9,415503	9,547237	9,375118333	-0,17211867	0,887538331 2010011I20Rik
17432587	4,291668	4,038478	4,724141	4,012085	4,545986	3,979927	4,351429	4,179332667	-0,17209633	0,887552071 ---
17275524	7,044435	7,400171	7,282868	7,024784	6,97635	7,21006	7,2424913	7,070398	-0,17209333	0,887553916 Srp54c
17303989	6,4683	6,347603	6,366714	6,091637	6,214775	6,360016	6,3942057	6,222142667	-0,172063	0,887572578 Zfp503
17207581	6,752687	5,665614	6,62192	6,060537	6,522912	5,940762	6,3467403	6,174737	-0,17200333	0,887609286 ---
17424771	4,686691	4,649715	5,248703	4,72682	4,681545	4,660786	4,861703	4,689717	-0,171986	0,887619951 Spag8
17204399	6,247608	5,510417	5,001631	5,525443	5,237683	5,480622	5,586552	5,414582667	-0,17196933	0,887630205 ---
17419032	11,17472	11,1465	11,08991	11,07809	10,85173	10,96551	11,137043	10,96511	-0,17193333	0,887652355 ---
17325135	4,500389	4,353628	4,605448	4,303663	4,348113	4,291914	4,4864883	4,314563333	-0,171925	0,887657482 Ropn1 // Ropn1
17364090	7,236696	7,227102	7,021259	6,951352	6,983059	7,034897	7,1616857	6,989769333	-0,17191633	0,887662814 Ankrd22
17341076	5,181674	5,007879	4,886657	4,718437	5,042195	4,799831	5,0254033	4,853487667	-0,17191567	0,887663224 4930474M22Rik
17503217	7,069908	6,728736	6,852845	6,604026	6,700737	6,831047	6,8838297	6,711936667	-0,171893	0,887677171 Gadd45gip1
17297621	5,132071	4,864612	5,152655	4,800762	4,637001	5,195964	5,0497793	4,877909	-0,17187033	0,887691118 ---
17318483	7,838313	7,855934	7,975049	7,681228	7,835973	7,636489	7,8897653	7,717896667	-0,17186867	0,887692143 Bop1
17374517	3,331741	3,232538	3,373602	3,286652	2,860408	3,275264	3,312627	3,140774667	-0,17185233	0,887702193 ---
17236305	4,872698	3,994832	4,601532	4,292402	4,23601	4,425144	4,4896873	4,317852	-0,17183533	0,887712653 Tyms-ps // Tyms-ps // Tyms-ps // Tyms-
17458003	4,991756	4,698821	5,03903	4,476276	4,816735	4,921115	4,909869	4,738042	-0,171827	0,887717781 Olfr437
17280923	8,271315	8,440837	8,18749	7,871123	8,183711	8,32934	8,2998807	8,128058	-0,17182267	0,887720447 Tfb2m
17211087	5,459508	5,446515	5,273058	5,144559	5,322725	5,196341	5,393027	5,221208333	-0,17181867	0,887722909 ---
17547598	3,289379	3,539396	3,491432	3,39238	3,226369	3,186018	3,440069	3,268255667	-0,17181333	0,88772619 ---
17370843	8,42527	8,390704	8,308545	8,136347	8,171805	8,301015	8,3748397	8,203055667	-0,171784	0,88774424 Arl6ip6
17409026	4,694369	4,630709	4,896239	4,436428	4,693756	4,575828	4,740439	4,568670667	-0,17176833	0,887753881 Ubl4b
17406843	4,653653	3,775229	4,190492	3,946408	4,091525	4,066202	4,206458	4,034711667	-0,17174633	0,887767418 5830417I10Rik
17311633	4,559497	4,462907	4,239284	4,267696	4,263198	4,215609	4,4205627	4,248834333	-0,17172833	0,887778495 Has2
17368887	5,243724	4,892941	5,30457	4,853366	5,035443	5,037395	5,1470783	4,975401333	-0,171677	0,887810084 Gm13547

17251756	7,126966	7,158955	6,827832	6,994651	6,643165	6,96111	7,0379177	6,866308667	-0,171609	0,887851931	Plscr3
17539084	4,079883	3,884342	4,395276	4,010867	3,988646	3,845234	4,1198337	3,948249	-0,17158467	0,887866906	---
17363980	7,168806	7,234827	7,393178	7,189883	7,101736	6,990655	7,2656037	7,094091333	-0,17151233	0,887911423	Minpp1 // Minpp1
17547610	7,15254	6,987039	7,195409	6,883818	6,921108	7,015531	7,1116627	6,940152333	-0,17151033	0,887912654	LOC100861787
17330497	7,958646	8,574742	8,452049	8,195044	8,200621	8,075285	8,328479	8,156983333	-0,17149567	0,88792168	---
17249423	4,255931	4,517433	4,228661	4,01902	4,396434	4,072183	4,3340083	4,162545667	-0,17146267	0,887941991	Ppp2ca
17227636	9,887129	9,871532	9,431365	9,680352	9,308235	9,687099	9,7300087	9,558562	-0,17144667	0,887951838	Cfhr1
17223760	5,408874	5,264706	5,395131	5,414336	5,165181	4,97496	5,356237	5,184825667	-0,17141133	0,887973586	4933402D24Rik
17362383	4,347592	4,030346	3,829406	4,112381	3,770194	3,810612	4,0691147	3,897729	-0,17138567	0,887989383	Slc22a27
17204949	8,50589	7,941362	8,472819	8,022321	8,280558	8,103083	8,3066903	8,135320667	-0,17136967	0,887999232	---
17512677	5,554386	5,431266	5,183553	5,09445	5,176693	5,384089	5,389735	5,218410667	-0,17132433	0,888027135	Cdh1 // Cdh1
17302928	3,542432	3,720282	3,381453	3,292272	3,475478	3,362448	3,5480557	3,376732667	-0,171323	0,888027956	AA536875
17457295	3,685727	3,104048	3,243621	3,373053	3,019999	3,126403	3,3444653	3,173151667	-0,17131367	0,888033701	---
17326917	3,602222	3,411627	3,788339	3,273354	3,737312	3,277609	3,6007293	3,429425	-0,17130433	0,888039446	2310034C09Rik
17232041	4,259197	3,88717	4,274372	4,127548	3,829433	3,949899	4,1402463	3,96896	-0,17128633	0,888050526	---
17267454	8,246967	8,141163	8,156929	8,209048	7,82249	7,99971	8,1816863	8,010416	-0,17127033	0,888060375	Prr11
17288898	9,304594	9,436063	9,160872	8,99164	9,180453	9,215659	9,3005097	9,129250667	-0,171259	0,888067351	Lysmd3
17464783	4,828051	4,648144	4,422049	4,537699	4,315643	4,531217	4,632748	4,461519667	-0,17122833	0,888086229	Gm16055
17446351	3,688551	3,419396	3,65377	3,257185	3,323851	3,667058	3,587239	3,416031333	-0,17120767	0,888098951	E130116L18Rik
17484569	3,886672	3,95367	3,699753	3,791309	3,643146	3,592204	3,8466983	3,675553	-0,17114533	0,888137323	Olfr61
17202705	2,807494	3,106761	3,046314	2,953028	2,819882	2,674278	2,9868563	2,815729333	-0,171127	0,888148609	---
17273749	5,270595	5,233217	5,762109	5,146984	5,293766	5,31179	5,4219737	5,250846667	-0,171127	0,888148609	---
17514971	3,512065	3,336742	3,270892	3,220717	3,046023	3,339691	3,373233	3,202143667	-0,17108933	0,888171798	Olfr872
17248102	3,870246	3,830848	3,555252	3,646734	3,454213	3,642188	3,7521153	3,581045	-0,17107033	0,888183495	---
17207425	8,484822	8,139868	8,626397	8,304663	8,328108	8,105162	8,417029	8,245977667	-0,17105133	0,888195192	---
17425396	3,775359	4,240232	4,136894	3,882946	3,917859	3,838539	4,0508283	3,879781333	-0,171047	0,88819786	---
17525794	3,259308	3,153102	3,307059	2,968004	2,974768	3,263558	3,239823	3,068776667	-0,17104633	0,88819827	Olfr983
17457792	4,143659	4,31812	4,154417	3,867976	4,238859	3,996483	4,2053987	4,034439333	-0,17095933	0,888251834	---
17457691	5,959516	5,731359	5,969363	5,782626	5,867537	5,497231	5,886746	5,715798	-0,170948	0,888258811	---
17482381	4,308877	4,258037	4,463909	4,306269	3,951448	4,260303	4,3436077	4,172673333	-0,17093433	0,888267226	Abca14
17284455	4,667012	4,703485	4,877084	4,775945	4,933887	4,02498	4,7491937	4,578270667	-0,170923	0,888274204	---
17396199	3,680845	3,784451	3,669056	3,575498	3,448481	3,597671	3,7114507	3,54055	-0,17090067	0,888287955	---
17496643	5,986474	5,871792	6,268877	5,949092	5,859252	5,806121	6,042381	5,871488333	-0,17089267	0,88829288	Gm4532
17467711	9,252191	9,337734	9,129159	9,097785	9,020848	9,087781	9,2396947	9,068804667	-0,17089	0,888294522	Usp39
17548989	4,14512	4,365479	4,272665	4,101782	4,217301	3,951518	4,261088	4,090200333	-0,17088767	0,888295959	Tmco1 // Tmco1
17518254	3,242009	3,30995	3,278516	3,10735	3,196058	3,014425	3,276825	3,105944333	-0,17088067	0,888300269	---
17219516	4,699681	5,024551	4,851634	4,766877	4,540097	4,756306	4,858622	4,68776	-0,170862	0,888311763	---
17494770	5,936796	5,651926	5,79908	5,681752	5,628072	5,565486	5,795934	5,625103333	-0,17083067	0,888331056	Olfr469
17421179	5,348243	4,842597	5,051893	4,898562	5,089021	4,742733	5,080911	4,910105333	-0,17080567	0,888346449	Pramel1
17467265	2,839624	2,914952	3,173267	2,761095	2,759503	2,894878	2,9759477	2,805158667	-0,170789	0,888356712	---
17539411	5,312098	5,141301	5,190025	4,978067	4,936306	5,216691	5,2144747	5,043688	-0,17078667	0,888358149	---
17376300	3,882072	3,829487	4,044602	3,6205	3,889874	3,733536	3,9187203	3,74797	-0,17075033	0,888380522	1700020A23Rik
17549504	4,491903	5,050561	4,138891	4,249112	4,418248	4,501914	4,5604517	4,389758	-0,17069367	0,888415417	---
17549660	4,491903	5,050561	4,138891	4,249112	4,418248	4,501914	4,5604517	4,389758	-0,17069367	0,888415417	---
17549610	5,08105	5,37261	5,201554	5,090024	4,903676	5,149439	5,2184047	5,047713	-0,17069167	0,888416648	---
17208391	4,332112	4,899378	4,849959	4,600179	4,457171	4,512094	4,6938163	4,523148	-0,17066833	0,888431017	---
17345670	4,940179	4,584421	5,200158	4,652432	4,67669	4,883651	4,9082527	4,737591	-0,17066167	0,888435123	Guca1a
17210245	5,039169	5,277861	4,972607	4,817956	4,993151	4,966549	5,0965457	4,925885333	-0,17066033	0,888435944	---
17303197	4,732128	5,239237	4,523178	4,497813	4,501124	4,983709	4,8315143	4,660882	-0,17063233	0,888453187	---
17203195	5,596314	5,362829	5,572031	5,411291	5,693507	4,914573	5,5103913	5,339790333	-0,170601	0,888472483	---
17412853	9,944744	10,08925	9,860085	9,597006	9,944988	9,840389	9,964693	9,794127667	-0,17056533	0,888494448	---
17316831	8,554638	8,635966	8,587549	8,45686	8,416384	8,393298	8,5927177	8,422180667	-0,170537	0,888511898	Nudcd1
17214018	10,26737	10,28019	10,41632	10,32645	10,1106	10,01526	10,321293	10,15077	-0,17052333	0,888520315	Gm8883
17516131	6,339153	6,737182	6,578162	6,41342	6,461926	6,267863	6,551499	6,381069667	-0,17042933	0,888578209	Olfr884
17233534	7,120585	7,001416	6,864739	6,756802	6,785688	6,932964	6,99558	6,825151333	-0,17042867	0,888578619	Sowahc
17534621	5,793613	5,899358	5,843167	5,622976	5,643924	5,757994	5,8453793	5,674964667	-0,17041467	0,888587242	Slc25a14
17346713	3,916082	3,69775	3,739471	3,225981	4,029106	3,586991	3,7844343	3,614026	-0,17040833	0,888591143	---
17516192	6,214283	6,600841	6,589041	6,197001	6,54208	6,153922	6,468055	6,297667667	-0,17038733	0,888604078	Olfr914
17349910	4,967144	4,525182	4,73196	4,523136	4,532295	4,657734	4,7414287	4,571055	-0,17037367	0,888612495	Pcdhb13
17270322	8,306551	8,582125	8,49681	8,143118	8,355268	8,376045	8,4618287	8,291477	-0,17035167	0,888626046	Eftud2
17247656	10,25622	10,45891	10,3592	10,24537	10,19035	10,12757	10,35811	10,18776333	-0,17034667	0,888629126	Cct4
17443710	11,12266	12,24925	12,23567	12,32152	11,36278	11,41228	11,869193	11,69886	-0,17033333	0,888637339	Azgp1
17467477	6,360529	5,913856	6,201797	5,885084	6,021073	6,059086	6,1587273	5,988414333	-0,170313	0,888649863	---
17216274	3,48123	3,132727	3,586355	3,203212	3,297049	3,189208	3,400104	3,229823	-0,170281	0,888669574	Gm20268
17412119	4,23671	4,110568	4,328371	4,05179	3,986756	4,126354	4,2252163	4,054966667	-0,17024967	0,888688875	---
17449064	4,835719	4,768752	5,296244	4,666985	4,90801	4,815117	4,966905	4,796704	-0,170201	0,888718854	Spink2
17486058	3,213759	3,127186	2,950865	3,098477	2,852553	2,830217	3,09727	2,927082333	-0,17018767	0,888727067	Vmn1r61
17411981	8,218925	8,194929	8,201005	8,007169	8,061727	8,035414	8,204953	8,03477	-0,170183	0,888729942	Nbn
17205481	6,110571	5,297764	5,510209	5,679555	4,870591	5,857988	5,6395147	5,469378	-0,17013667	0,888758485	---
17364917	5,303811	5,607119	5,601779	5,257345	5,578634	5,166345	5,5042363	5,334108	-0,17012833	0,888763619	Hpse2
17462180	5,559551	5,774498	5,656648	5,12922	5,743136	5,607967	5,6635657	5,493441	-0,17012467	0,888765878	Zfp239
17481634	4,022909	3,692853	3,458169	3,572943	3,586901	3,503717	3,7246437	3,554520333	-0,17012333	0,888766699	Olfr512
17432760	5,201419	5,16385	5,044425	5,05859	4,78937	5,051431	5,1365647	4,966463667	-0,170101	0,888780457	Zfp534
17234213	4,477508	4,387867	4,624454	4,294695	4,249623	4,435221	4,4966097	4,326513	-0,17009667	0,888783127	Pcdh15
17205211	5,709966	6,260301	6,129618	6,022291	5,855793	5,711535	6,033295	5,863206333	-0,17008867	0,888788055	---
17209401	5,709966	6,260301	6,129618	6,022291	5,855793	5,711535	6,033295	5,863206333	-0,17008867	0,888788055	---
17258282	6,258989	5,894213	5,985682	5,872786	5,901406	5,854513	6,0462947	5,876235	-0,17005967	0,888805921	---
17370721	3,821341	3,687464	4,022563	3,707863	3,617887	3,695441	3,8437893	3,673730333	-0,170059	0,888806332	---
17467144	4,609098	4,545653	4,29264	4,411755	4,270267	4,255201	4,4824637	4,312407667	-0,170056	0,88880818	Vmn1r7
17491378	9,124582	9,14317	9,237329	9,081187	8,802794	9,111135	9,1683603	8,998372	-0,16998833	0,888849869	E2f8
17462657	5,559027	5,258223	5,371119	5,285555	5,101127	5,291722	5,396123	5,226134667	-0,16998833	0,888849869	1700063H04Rik
17324110	8,752348	8,980386	9,115464	8,701217	9,040806	8,596217	8,9493993	8,779413333	-0,169986	0,888851307	Snord66
17500270	5,542893	5,064284	5,564338	5,258739	5,183091	5,219789	5,390505	5,220539667	-0,16996533	0,88886404	Zfp703
17454254	5,615568	5,548572	5,822304	5,636099	5,279858	5,560715	5,662148	5,492224	-0,169924	0,888889506	Mir106b
17266903	5,791588	5,578546	6,083973	5,857487	5,445719	5,641162	5,8180357	5,648122667	-0,169913	0,888896283	Gas2l2
17364945	6,040844	5,629424	5,971122	5,643188	5,745846	5,742622	5,8804633	5,710552	-0,16991133	0,88889731	---
17516263	3,502248	3,617543	3,786721	3,404455	3,580166	3,412316	3,635504	3,465645667	-0,16985833	0,888929966	Olfr951
17468865	6,686899	6,47332	6,573865	6,456157	6,314786	6,453679	6,578028	6,408207333	-0,16982067	0,888953175	Chchd6
17244961	3,508893	3,444724	3,269961	3,083777	3,456348	3,174034	3,4078593	3,238053	-0,16980633	0,888962007	Glipr1l3
17334381	5,91533	5,879612	6,229963	5,91533	5,864055	5,736159	6,0083017	5,838514667	-0,169787	0,88897392	Tbl3 // Tbl3
17248957	3,257215	3,246469	3,006951	3,001055	3,11312	2,887147	3,1702117	3,000440667	-0,169771	0,888983779	Olfr1392
17274412	4,601068	4,576578	4,086996	3,89843	4,238836	4,618111	4,4215473	4,251792333	-0,169755	0,888993638	Gm19960
17376510	8,264754	8,258157	8,377518	8,150964	8,054413	8,185814	8,300143	8,130397	-0,169746	0,888999184	Smox
17313577	7,131305	7,134907	7,227387	6,935304	7,071981	6,977103	7,164533	6,994796	-0,169737	0,88900473	---
17321626	6,74523	6,679197	6,773869	6,507613	6,453766	6,727737	6,7327653	6,563038667	-0,16972667	0,889011097	Gpd1 // Gpd1
17304271	5,024917	4,529052	4,433219	4,279916	4,310041	4,888056	4,662396	4,492671	-0,169725	0,889012124	---
17300396	8,30347	8,516191	8,340276	8,298842	8,134233	8,217703	8,3866457	8,216926	-0,16971967	0,889015411	Ngdn
17288261	6,683112	5,914503	5,687095	6,223825	5,871913	5,679973	6,0949033	5,925237	-0,16966633	0,889048277	Vmn2r85
17455053	4,245987	3,859573	4,622272	4,141481	3,758839	4,318532	4,2426107	4,072950667	-0,16966	0,889052179	Gm4871
17389795	7,830917	7,934685	7,759473	7,564056	7,774386	7,677675	7,8416917	7,672039	-0,16965267	0,889056699	Ccdc32
17308954	4,4506	4,570331	4,724754	4,316762	4,465973	4,454069	4,581895	4,412268	-0,169627	0,889072516	---
17474663	4,925057	5,11691	5,3167	5,004674	4,849326	4,995789	5,1195557	4,949929667	-0,169626	0,889073132	Zfp296
17412269	5,371974	5,257185	5,474869	5,110771	5,302732	5,181654	5,3680093	5,198385667	-0,16962367	0,88907457	Klhl32
17550486	6,490317	6,066998	6,230334	6,044154	6,155853	6,078792	6,2625497	6,092933	-0,16961667	0,889078884	---
17210887	7,81336	7,903797	7,759796	7,704227	7,677084	7,586821	7,825651	7,656044	-0,169607	0,889084841	Atp6v1h
17467768	5,056813	4,95858	5,287692	4,923375	4,881961	4,988959	5,1010283	4,931431667	-0,16959667	0,889091209	Sh2d6
17371839	5,078976	4,879347	4,917329	4,923331	4,897454	4,546341	4,9585507	4,789042	-0,16950867	0,889145443	Gm13662
17549492	4,380958	4,301417	4,480052	4,183048	4,21016	4,26077	4,3874757	4,217992667	-0,169483	0,889161261	---
17372035	4,997156	4,956352	5,240658	4,844355	4,807087	5,034298	5,064722	4,895246667	-0,16947533	0,889165986	Hoxd13
17229094	3,246214	3,150794	3,289369	3,081364	3,107631	2,989073	3,2287923	3,059356	-0,16943633	0,889190023	---
17279881	8,935443	9,125244	8,936357	8,836936	8,584101	9,06777	8,9990147	8,829602333	-0,16941233	0,889204816	Ubxn2a
17442096	9,171924	9,338014	9,246967	9,007965	9,186493	9,054242	9,2523017	9,0829	-0,16940167	0,88921139	Arpc3
17204811	3,029101	3,349007	3,274974	3,063129	3,161947	2,91986	3,217694	3,048312	-0,169382	0,889223512	---
17394984	4,833322	4,977017	4,896784	4,722116	4,812027	4,664947	4,9023743	4,73303	-0,16934433	0,889246728	4930470P17Rik
17291258	2,913913	2,952442	3,270892	2,948089	2,767282	2,913913	3,045749	2,876428	-0,169321	0,889261111	Hist1h2ba
17271244	8,192089	8,368538	8,312347	8,097432	8,042995	8,22465	8,2909913	8,121692333	-0,169299	0,889274671	Slc16a6
17520922	5,154107	5,381043	5,179934	4,929288	4,992952	5,28512	5,2383613	5,06912	-0,16924133	0,889310218	Rab6b
17454310	6,02382	5,801235	6,41208	5,874109	5,801447	6,053864	6,079045	5,909806667	-0,16923833	0,889312067	Gpc2
17275294	3,467217	3,483162	3,081503	3,367665	3,259971	2,896577	3,3439607	3,174737667	-0,169223	0,889321519	---
17358876	8,310056	8,246287	8,123017	7,938738	8,091757	8,141353	8,2264533	8,057282667	-0,16917067	0,889353779	Rpp30
17409690	7,995757	8,038462	7,815891	7,704538	7,794897	7,843169	7,9500367	7,780868	-0,16916867	0,889355012	Rtcd1
17223982	5,030471	5,105241	5,637486	5,045529	5,271887	4,948278	5,2577327	5,088564667	-0,169168	0,889355423	---
17445689	6,770495	5,869563	6,451772	6,363033	6,261627	5,95968	6,3639433	6,19478	-0,16916333	0,8893583	LOC100861621 // Speer4c
17383520	5,807028	5,872606	6,120801	5,67777	5,844331	5,770849	5,9334783	5,764316667	-0,16916167	0,889359327	Mir219-2
17226080	3,224691	3,187628	3,206854	3,05719	3,102618	2,951985	3,206391	3,037264333	-0,16912667	0,889380904	Serpinb3a
17421174	4,84715	4,786954	4,780636	4,655792	4,614755	4,636828	4,8049133	4,635791667	-0,16912167	0,889383986	Lrrc38
17400612	6,695979	6,578922	6,791478	6,551844	6,583444	6,42379	6,688793	6,519692667	-0,16910033	0,889397138	---
17202739	3,947293	4,732821	3,363223	3,994022	3,759611	3,782778	4,0144457	3,845470333	-0,16897533	0,889474201	---
17284105	5,951986	5,808501	6,0417	5,616543	5,58606	6,092738	5,9340623	5,765113667	-0,16894867	0,889490642	1200009I06Rik // 1200009I06Rik
17206163	5,989877	6,940526	6,694599	6,443337	6,390404	6,284418	6,5416673	6,372719667	-0,16894767	0,889491259	---
17276153	8,940478	8,956787	8,617987	8,811471	8,460563	8,73638	8,8384173	8,669471333	-0,168946	0,889492287	Daam1
17201515	3,015055	3,606339	3,387592	3,56963	2,830556	3,102065	3,3363287	3,167417	-0,16891167	0,889513455	---
17252333	4,829677	4,740908	5,303604	4,786681	4,737807	4,843014	4,958063	4,789167333	-0,16889567	0,88952332	1700051A21Rik
17398993	5,977068	5,885091	6,133192	5,893592	5,779645	5,815437	5,9984503	5,829558	-0,16889233	0,889525375	Gm3764

17201607	4,555193	4,086643	4,50779	4,233368	4,261398	4,148256	4,3832087	4,214340667	-0,168868	0,889540379	---
17506356	8,777122	9,033756	9,132468	8,821499	8,790472	8,824802	8,9811153	8,812257667	-0,16885767	0,88954675	Zfpm1
17429421	6,164664	5,896215	6,227662	5,987566	5,903501	5,891087	6,0961803	5,927384667	-0,16879567	0,889584979	Rimkla
17485698	5,37512	5,499066	5,380843	5,275031	5,396103	5,077516	5,418343	5,24955	-0,168793	0,889586624	D030047H15Rik
17367666	4,646278	4,641033	4,556841	4,459268	4,537863	4,340652	4,6147173	4,445927667	-0,16878967	0,889588679	Il1f5
17201189	8,220576	8,397655	8,419256	7,954986	8,114561	8,46165	8,345829	8,177065667	-0,16876333	0,889604917	---
17476648	6,917135	7,219425	7,12044	6,876759	6,86708	7,006873	7,0856667	6,916904	-0,16876267	0,889605328	Gm6003
17243644	7,453043	7,474786	7,444396	7,282385	7,348053	7,23551	7,4574083	7,288649333	-0,168759	0,889607589	Cry1
17505903	5,522142	5,794767	5,621946	5,609944	5,265059	5,557679	5,646285	5,477560667	-0,16872433	0,889628965	Maf // Maf
17404429	5,023707	5,086496	5,068709	4,755229	4,915066	5,002451	5,0596373	4,890915333	-0,168722	0,889630404	Nceh1 // Nceh1
17288014	5,095387	5,608665	5,655631	5,046553	5,502045	5,304997	5,4532277	5,284531667	-0,168696	0,889646437	---
17498171	5,522062	5,438672	5,407931	5,316077	5,257608	5,288909	5,4562217	5,287531333	-0,16869033	0,889649932	Th
17546743	5,36541	5,966433	6,285836	5,332026	5,915583	5,864012	5,8725597	5,703873667	-0,168686	0,889652604	---
17286020	5,279094	4,975031	5,414436	5,168716	5,074247	4,919596	5,2228537	5,054186333	-0,16866733	0,889664115	D130043K22Rik
17507194	5,059212	4,741068	4,919681	4,519507	4,838232	4,856223	4,9066537	4,737987333	-0,16866633	0,889664732	Cd209b
17375935	5,791879	5,695272	5,909929	5,762293	5,701187	5,427762	5,7990267	5,630414	-0,16861267	0,889697827	Acoxl
17399549	5,802035	6,012897	6,439652	6,023922	5,947865	5,776962	6,0848613	5,916249667	-0,16861167	0,889698443	4933434E20Rik
17486581	8,784639	8,965717	9,225517	8,841739	8,914542	8,713822	8,9919577	8,823367667	-0,16859	0,889711805	Rps5 // Rps5
17372823	3,816518	3,801378	3,763068	3,658714	3,623091	3,593545	3,7936547	3,625116667	-0,168538	0,889743874	Olfr1112
17433582	5,631733	5,435691	5,636631	5,359506	5,40636	5,432639	5,5680183	5,399501667	-0,16851667	0,889757031	Trp73
17284638	3,367236	3,381007	3,291513	3,102356	3,270892	3,160971	3,3465853	3,178073	-0,16851233	0,889759704	---
17223473	3,943206	3,896927	3,683854	3,546486	3,493746	3,978253	3,841329	3,672828333	-0,16850067	0,889766899	Als2cr11
17525576	3,650386	3,585993	3,857269	3,581026	3,487935	3,519275	3,6978827	3,529412	-0,16847067	0,889785401	---
17447257	3,236988	3,026954	3,413591	3,060564	3,087031	3,024527	3,2258443	3,057374	-0,16847033	0,889785607	---
17366930	3,452223	3,347032	3,609703	3,493719	3,176762	3,233192	3,4696527	3,301224333	-0,16842833	0,889811511	Mir466g // Mir466g
17278470	9,388848	9,393959	9,332879	9,135787	9,259807	9,214923	9,3718953	9,203505667	-0,16838967	0,88983536	Vrk1
17353693	7,82954	7,936873	7,751617	7,772341	7,696741	7,543805	7,8393433	7,670962333	-0,168381	0,889840705	Pfdn1
17549260	3,604124	4,205561	3,952897	3,811003	3,580271	3,866194	3,9208607	3,752489333	-0,16837133	0,889846667	---
17223646	4,283913	4,347108	4,23021	4,166841	4,086474	4,102969	4,287077	4,118761333	-0,16831567	0,889881003	4930587A21Rik
17396971	5,572006	5,813062	5,700316	5,407522	5,639599	5,533381	5,695128	5,526834	-0,168294	0,889894367	LOC100862007
17483264	7,375831	7,431026	7,265285	7,088411	7,182263	7,296703	7,3573807	7,189125667	-0,168255	0,889918424	Itgal
17419646	4,735266	4,601842	4,51002	4,285939	4,568131	4,488392	4,6157093	4,447487333	-0,168222	0,88993878	---
17407448	3,061702	3,473424	3,377826	3,134613	3,154414	3,119266	3,3043173	3,136097667	-0,16821967	0,889940219	Lce1e
17214823	3,901426	3,895497	4,534925	3,981327	4,123436	3,72243	4,110616	3,942397667	-0,16821833	0,889941042	---
17207495	5,252947	5,354647	6,119004	5,731855	5,440863	5,049248	5,5755327	5,407322	-0,16821067	0,889945771	---
17201697	4,039024	3,795285	3,455726	3,608578	3,646956	3,529997	3,763345	3,595177	-0,168168	0,889972091	---
17202447	3,449333	3,72213	3,612336	4,000248	2,939525	3,339539	3,5945997	3,426437333	-0,16816233	0,889975587	---
17427077	4,31505	4,261306	4,431208	4,104763	4,131892	4,26656	4,3358547	4,167738333	-0,16811633	0,890003964	Ifna12
17393592	8,837723	8,846436	8,745782	8,68493	8,531452	8,709486	8,8099803	8,641956	-0,16802433	0,890060721	Ndrg3
17250750	4,995333	4,969592	4,759014	4,819193	4,411037	4,917654	4,9079797	4,739961333	-0,16801833	0,890064423	---
17206521	3,750235	3,893717	3,970209	3,572946	4,173638	3,363618	3,871387	3,703400667	-0,16798633	0,890084165	---
17437385	7,318289	7,676322	7,432632	7,381002	7,224277	7,318031	7,4757477	7,30777	-0,16797767	0,890089512	Sod3
17366121	5,973934	6,054945	5,889858	5,628851	6,056245	5,729715	5,9729123	5,804937	-0,16797533	0,890090952	---
17351822	5,704501	5,390508	5,417184	5,172741	5,519138	5,316584	5,5040643	5,336154333	-0,16791	0,890131261	Rpl17
17286641	8,377926	8,815967	8,461575	8,430506	8,418614	8,302691	8,5518227	8,383937	-0,16788567	0,890146274	Dsp
17500510	5,900468	5,77569	6,041596	5,721722	5,917887	5,574515	5,905918	5,738041333	-0,16787667	0,890151827	---
17201083	6,169126	6,886992	6,779664	6,772986	5,912248	6,646925	6,6119273	6,444053	-0,16787433	0,890153267	---
17549392	7,646596	7,494337	7,718705	7,43981	7,585124	7,331112	7,6198793	7,452015333	-0,167864	0,890159643	---
17358032	8,926394	8,935184	8,878428	8,591526	8,788527	8,856402	8,9133353	8,745485	-0,16785033	0,890168075	2410127L17Rik
17248589	6,647255	6,137197	6,534365	6,380299	6,278728	6,156251	6,4396057	6,271759333	-0,16784633	0,890170543	---
17415850	5,220581	4,296747	5,146235	5,351002	4,386857	4,422285	4,8878543	4,720048	-0,16780633	0,890195225	Ube2u
17208887	5,847945	5,429122	5,911244	5,649446	4,696655	6,338872	5,729437	5,561657667	-0,16777933	0,890211885	---
17404400	3,154405	3,169892	3,132193	3,074064	2,993266	2,886075	3,1521633	2,984468333	-0,167695	0,890263924	---
17551169	5,457571	6,084767	5,689449	5,705468	5,428994	5,594303	5,743929	5,576255	-0,167674	0,890276883	---
17398315	4,002794	3,767136	3,949912	3,878631	3,92614	3,412067	3,906614	3,738946	-0,167668	0,890280585	LOC626082
17290384	5,140126	5,281548	5,304733	4,768937	5,445153	5,009349	5,2421357	5,074479667	-0,167656	0,890287991	---
17211305	4,483614	4,994767	3,97097	4,64568	4,11559	4,185298	4,483117	4,315522667	-0,16759433	0,890326046	Pi15
17539004	5,002193	4,60883	4,663938	4,71782	4,569965	4,484406	4,7583203	4,590730333	-0,16759	0,89032872	Klf8
17530227	7,124991	6,945403	7,85043	7,363298	7,188313	6,866518	7,3069413	7,139376333	-0,167565	0,890344148	---
17546953	3,816446	4,658532	4,287383	3,872397	4,651996	3,735295	4,2541203	4,086562667	-0,16755767	0,890348674	Rbmy1a1
17480596	3,644882	3,691152	3,480971	3,572278	3,308266	3,433886	3,6056683	3,438143333	-0,167525	0,890368834	Olfr520
17538803	5,276218	7,292522	5,083676	5,908494	5,805723	5,435662	5,8841387	5,716626333	-0,16751233	0,890376652	---
17508593	8,397411	8,492205	8,275537	8,087442	8,25186	8,32336	8,3883843	8,220887333	-0,167497	0,890386115	Mak16
17262662	5,23357	5,421393	5,355899	4,98945	5,171227	5,347757	5,336954	5,169478	-0,167476	0,890399076	Shroom1 // Shroom1
17413300	6,449519	6,287965	6,736205	6,412729	6,193367	6,365209	6,4912297	6,323768333	-0,16746133	0,890408128	Rusc2
17292603	6,270237	6,319383	6,719792	6,529773	5,936147	6,341147	6,4364707	6,269022333	-0,16744833	0,890416151	Gm2848
17526195	7,149826	7,268968	7,073737	6,995104	6,929964	7,065226	7,164177	6,996764667	-0,16741233	0,89043837	Hinfp
17542895	3,492552	3,754598	3,292883	3,256865	3,411306	3,369678	3,5133443	3,345949667	-0,16739467	0,890449274	---
17533492	3,627397	3,885813	4,098636	4,256958	3,485549	3,367236	3,8706153	3,703247667	-0,16736767	0,890465939	---
17512772	4,829975	4,578071	5,165992	4,69588	4,769359	4,606897	4,8580127	4,690712	-0,16730067	0,890507294	---
17447092	8,440959	8,571203	8,36126	8,008906	8,424703	8,438132	8,4578073	8,290580333	-0,167227	0,890552766	---
17348052	3,003624	2,95437	2,882319	2,811047	2,831918	2,695693	2,946771	2,779552667	-0,16721833	0,890558116	---
17495306	11,15836	10,96742	11,13754	11,03218	10,95875	10,77082	11,087773	10,92058333	-0,16719	0,890575606	---
17260482	3,84539	3,686665	3,712645	3,618739	3,469353	3,655065	3,7482333	3,581052333	-0,167181	0,890581162	---
17283675	5,340369	5,566625	5,3595	5,319549	5,199988	5,245422	5,4221647	5,254986333	-0,16717833	0,890582808	Serpina3c
17322486	6,093199	6,118316	5,810691	5,631694	5,63188	6,257173	6,007402	5,840249	-0,167153	0,890598446	---
17322487	6,093199	6,118316	5,810691	5,631694	5,63188	6,257173	6,007402	5,840249	-0,167153	0,890598446	---
17357904	4,066061	3,906066	3,648229	4,176429	3,485626	3,45694	3,873452	3,706331667	-0,16712033	0,890618612	Olfr1454
17311805	4,413031	4,288883	4,391098	4,071677	4,091228	4,428819	4,3643373	4,197241333	-0,167096	0,890633634	Zfp572
17506400	7,12972	7,25207	7,339951	7,019747	7,088524	7,112232	7,2405803	7,073501	-0,16707933	0,890643923	Ctu2
17367868	5,699014	5,580132	6,090604	5,618719	5,812887	5,436996	5,7899167	5,622867333	-0,16704933	0,890662444	Rnf208
17221932	3,700679	3,375503	2,990211	3,158743	3,163965	3,242547	3,3554643	3,188418333	-0,167046	0,890664502	---
17250317	4,547688	4,523431	4,652603	4,286374	4,533936	4,402357	4,574574	4,407555667	-0,16701833	0,890681582	---
17331418	7,800356	7,771203	7,80268	7,779528	7,457109	7,636585	7,791413	7,624407333	-0,16700567	0,890689402	Zfp654
17533961	6,958229	6,745804	6,619749	6,846728	6,314666	6,661502	6,774594	6,607632	-0,166962	0,890716361	---
17494480	5,110174	5,021299	4,990163	4,992095	4,874999	4,753689	5,0405453	4,873594333	-0,166951	0,890723153	Olfr691
17203027	6,405195	5,789602	5,803406	6,131601	5,362729	6,003082	5,999401	5,832470667	-0,16693033	0,890735913	---
17292855	11,14668	10,70567	10,72516	10,76298	10,69675	10,61703	10,85917	10,69225333	-0,16691667	0,890744351	F12
17493182	9,64023	9,961149	9,711561	9,514793	9,616458	9,680972	9,77098	9,604074333	-0,16690567	0,890751142	Rps13 // Rps13
17500830	3,150028	3,00409	3,34693	2,989989	3,046788	2,96357	3,167016	3,000115667	-0,16690033	0,890754435	---
17285799	3,453524	3,158758	3,270892	3,124018	3,067315	3,191144	3,2943913	3,127492333	-0,166899	0,890755258	Vmn1r219
17311263	5,516362	5,29751	5,4871	5,341213	5,029472	5,429608	5,4336573	5,266764333	-0,166893	0,890758963	AU022793
17543495	4,0064	3,91992	3,975152	3,619041	4,019728	3,762073	3,9671573	3,800280667	-0,16687667	0,890769048	Tex11
17203165	6,35042	5,864787	6,496054	6,396399	5,641889	6,172409	6,237087	6,070232333	-0,16685467	0,890782631	---
17517752	6,419823	5,973753	6,401566	6,178381	6,163677	5,952688	6,2650473	6,098248667	-0,16679867	0,890817209	Ulk3
17387565	4,803146	4,896007	4,850524	4,823586	4,73754	4,488194	4,8498923	4,683106667	-0,16678567	0,890825236	Lrrc55
17386633	3,961792	3,997776	3,787775	3,927443	3,630001	3,689617	3,915781	3,749020333	-0,16676067	0,890840673	4930441J16Rik
17395613	5,233724	5,011477	4,916959	4,865064	4,79545	5,001413	5,0540533	4,887309	-0,16674433	0,890850758	BC066135
17419409	7,936624	7,627247	7,887578	7,522545	7,759003	7,669836	7,8171497	7,650461333	-0,16668833	0,890885339	Snora61
17309338	3,796203	3,435739	3,767539	3,474529	3,24353	3,781383	3,6664937	3,499814	-0,16667967	0,89089069	---
17474300	3,295508	3,651848	3,462017	3,225573	3,369586	3,314258	3,469791	3,303139	-0,166652	0,890907775	Gm5155
17454414	8,261115	7,483398	7,952723	8,127853	7,517177	7,552288	7,8990787	7,732439333	-0,16663933	0,890915597	Mir339
17382755	4,379409	4,113233	4,429788	4,34185	4,0969	3,983822	4,3074767	4,140857333	-0,16661933	0,890927948	Glt6d1
17549792	6,178746	6,036561	6,151133	6,03714	5,97004	5,859452	6,1221467	5,955544	-0,16660267	0,890938241	---
17419371	6,045559	6,282603	6,43459	5,983192	6,198271	6,081516	6,2542507	6,087659667	-0,166591	0,890945445	Epb4.1
17534287	3,757465	3,121353	3,20838	3,337791	3,145486	3,104172	3,3623993	3,195816333	-0,166583	0,890950386	Rhox3f
17210533	6,423838	6,313632	6,129892	5,19296	6,570567	6,604097	6,2891207	6,122541333	-0,16657933	0,89095265	---
17361310	6,726855	7,115778	7,074211	6,723847	6,770497	6,922844	6,9722813	6,805729333	-0,166552	0,89096953	Rce1
17340473	4,130049	3,852247	4,213189	3,976358	3,863743	3,855941	4,0651617	3,898680667	-0,166481	0,891013379	4930470H14Rik // 4930470H14Rik
17403035	5,024884	5,006419	5,152747	4,946966	5,029274	4,7084	5,06135	4,89488	-0,16647	0,891020173	Gm19708
17408360	10,20715	10,1414	10,04288	10,04136	9,832149	10,01855	10,130477	9,964019667	-0,166457	0,891028202	Hao2
17324441	6,144472	5,653598	5,897168	5,739531	5,645991	5,810351	5,8984127	5,731957667	-0,166455	0,891029437	Rtp1
17444236	8,096994	8,247175	8,336228	7,920222	8,280047	7,980838	8,226799	8,060369	-0,16643	0,891044877	Wipi2
17476219	5,253696	4,912603	5,139576	4,785594	5,092974	4,92804	5,1019583	4,935536	-0,16642233	0,891049613	Zfp420
17468067	6,500195	6,305758	6,411619	6,227453	6,354574	6,136353	6,4058573	6,23946	-0,16639733	0,891065053	Dok1
17203681	5,418565	6,196992	6,167015	6,000848	5,384079	5,898456	5,927524	5,761127667	-0,16639633	0,891065671	---
17530291	8,440274	8,642307	8,455029	8,304074	8,367372	8,367069	8,5125367	8,346171667	-0,166365	0,891085024	Cdv3
17532526	8,228924	8,474949	8,262419	8,108582	8,16927	8,189428	8,3220973	8,15576	-0,16633733	0,891102113	Lztfl1
17291935	6,078347	6,022605	6,033874	5,811322	5,938868	5,885745	6,044942	5,878645	-0,166297	0,891127025	---
17374058	9,043733	8,935976	8,798613	8,606129	8,817965	8,855382	8,9261073	8,759825333	-0,166282	0,891136291	Kif18a
17482047	6,951805	6,678387	6,846375	6,672719	6,680239	6,624794	6,8255223	6,659250667	-0,16627167	0,891142673	Insc
17247924	4,112325	3,805636	3,925426	3,815174	3,903068	3,626348	3,9477957	3,78153	-0,16626567	0,89114638	Gm12070
17496726	5,056211	5,122023	5,090426	4,569669	5,198185	5,002049	5,0895533	4,923301	-0,16625233	0,891154616	1700120K04Rik
17340229	5,723412	5,361851	5,891952	5,794624	5,234828	5,449056	5,6590717	5,492836	-0,16623567	0,891164911	Gm19773
17421588	5,618118	5,735567	5,746536	5,459524	5,590483	5,551534	5,7000737	5,533847	-0,16622667	0,89117047	Fbxo2
17533776	4,563735	4,210738	4,039277	4,236014	4,07246	4,006656	4,27125	4,105043333	-0,16620667	0,891182824	Ppp1r2-ps7
17367738	3,462641	3,261193	3,256429	3,314151	3,066485	3,101079	3,3267543	3,160571667	-0,16618267	0,89119765	---
17499576	3,494846	3,884708	3,609703	3,432468	3,694433	3,36382	3,6630857	3,496907	-0,16617867	0,891200121	Defb34
17494168	5,425376	4,842005	5,123962	4,92224	5,015391	4,955188	5,1304477	4,964273	-0,16617467	0,891202592	Olfr601
17348965	9,048309	9,122669	9,021744	8,827705	8,912456	8,954042	9,0642407	8,898067667	-0,166173	0,891203621	Galnt1
17216373	7,625247	7,53342	7,596539	7,47616	7,35208	7,428478	7,5850687	7,418906	-0,16616267	0,891210005	Phlpp1
17503245	8,526708	8,685543	8,646459	8,357272	8,472843	8,53014	8,61957	8,453418333	-0,16615167	0,8912168	Syce2
17445697	3,652343	3,91222	3,688137	3,571268	3,729049	3,453971	3,7509	3,584762667	-0,16613733	0,891225654	---
17387357	4,186708	4,109513	4,267016	4,117232	4,067882	3,879827	4,1877457	4,021647	-0,16609867	0,891249541	---

17419031	7,299407	7,087694	6,109175	5,997268	7,351868	6,64897	6,832092	6,666035333	-0,16605667	0,891275487	---
17536329	5,604547	5,855457	5,881763	5,417729	6,036756	5,389323	5,780589	5,614602667	-0,16598633	0,891318939	Gspt2
17295373	6,280559	5,865419	6,450446	5,956065	6,114995	6,027463	6,198808	6,032841	-0,165967	0,891330884	2310005E17Rik
17404906	4,720843	4,702904	4,540915	4,638701	4,463461	4,364629	4,6548873	4,488930333	-0,165957	0,891337062	Gm12541
17410422	3,689533	3,405175	3,398686	3,324363	3,264229	3,407182	3,497798	3,331924667	-0,16587333	0,891388755	---
17547955	3,591074	3,609339	3,768558	3,533957	3,51866	3,418746	3,6563237	3,490454333	-0,16586933	0,891391227	4930553J12Rik
17261913	3,791557	3,453524	3,609703	3,518249	3,494597	3,344379	3,6182613	3,452408333	-0,165853	0,891401318	Gabra6
17497017	4,959974	4,681962	4,798385	4,66223	4,588958	4,691595	4,8134403	4,647594333	-0,165846	0,891405644	Cuzd1
17362885	3,435661	3,409307	3,508897	3,349315	3,223063	3,283969	3,4512883	3,285449	-0,16583933	0,891409763	1700017D01Rik
17208415	3,525566	3,723414	3,932544	3,746003	3,573978	3,364045	3,7271747	3,561342	-0,16583267	0,891413882	---
17388886	7,158372	7,526485	7,004432	7,062159	7,123553	7,006171	7,229763	7,063961	-0,165802	0,891432831	---
17261055	4,138478	4,314549	4,554796	4,159822	4,24044	4,110155	4,335941	4,170139	-0,165802	0,891432831	1700093K21Rik
17357537	10,89799	11,06476	11,35775	10,97252	10,9479	10,90289	11,106833	10,94110333	-0,16573	0,89147732	---
17545287	3,57304	3,187414	3,03118	3,247102	3,016391	3,030991	3,263878	3,098161333	-0,16571667	0,891485559	---
17278341	4,118917	3,972755	4,130559	4,090926	3,928591	3,705619	4,074077	3,908378667	-0,16569833	0,891496888	---
17548324	12,15268	12,15368	12,22207	12,02899	11,96269	12,03979	12,176143	12,01049	-0,16565333	0,891524696	Anp32b // Anp32b
17201611	5,300384	5,040332	4,977439	4,988101	5,031016	4,802144	5,1060517	4,940420333	-0,16563133	0,891538291	---
17351137	3,164307	3,130888	3,150528	3,003547	2,983386	2,961997	3,1485743	2,982976667	-0,16559767	0,891559096	Il17b
17348220	4,657	4,656544	4,675032	4,149529	4,519111	4,823167	4,6628587	4,497269	-0,16558967	0,89156404	Kif5b
17285907	8,843098	8,861301	8,914347	8,943647	8,564749	8,613646	8,8729153	8,707347333	-0,165568	0,89157743	Slc17a3
17200659	5,531994	5,30606	5,355921	5,07395	5,519917	5,103431	5,3979917	5,232432667	-0,165559	0,891582991	---
17302692	3,550648	3,344571	3,489333	3,330035	3,430412	3,127527	3,4615173	3,295991333	-0,165526	0,891603386	Gm9376
17496438	6,557224	6,772182	6,622477	6,567128	6,305106	6,583107	6,6506277	6,485113667	-0,165514	0,891610802	Ino80e
17547614	7,845129	7,92489	7,533975	7,753098	7,36487	7,689528	7,767998	7,602498667	-0,16549933	0,891619866	Spin1 // Spin1
17435497	3,435576	3,482425	3,112717	3,20256	3,115707	3,21598	3,3435727	3,178082333	-0,16549033	0,891625428	---
17439197	3,080677	2,862805	3,233458	2,810437	2,867598	3,002481	3,05898	2,893505333	-0,16547467	0,891635111	Gm7792
17444593	9,005177	9,101975	9,270207	8,813383	8,978362	8,989199	9,1257863	8,960314667	-0,16547167	0,891636965	Arpc1a
17222593	4,213601	4,29332	4,132438	4,005687	4,199846	3,937454	4,2131197	4,047662333	-0,16545733	0,891645824	Gm3646
17223787	4,752999	4,717631	4,64652	4,652202	4,555655	4,413085	4,7057167	4,540314	-0,16540267	0,89167961	Ccnyl1
17375603	4,458147	4,2056	4,034108	4,20075	3,986942	4,013995	4,2326183	4,067229	-0,16538933	0,891687851	Ctxn2
17206633	3,548956	4,137048	5,373533	4,197251	3,323671	5,04255	4,353179	4,187824	-0,165355	0,891709072	---
17225986	6,959627	6,867457	7,045396	6,79374	6,711841	6,871048	6,9574933	6,792209667	-0,16528367	0,891753163	---
17350250	3,881134	3,836004	4,111364	3,789157	3,714304	3,829207	3,942834	3,777556	-0,165278	0,891756666	Spink12
17428731	4,444987	4,738791	4,805866	4,49623	4,573168	4,424601	4,6632147	4,497999667	-0,165215	0,891795608	Hpdl
17548898	6,255615	6,769694	6,46679	6,08334	6,377581	6,535534	6,4973663	6,332151667	-0,16521467	0,891795814	Gm7634
17444924	5,750359	5,634065	5,650174	5,255479	5,669151	5,614331	5,6781993	5,512987	-0,16521233	0,891797257	8430423G03Rik
17326954	4,102113	4,265238	4,505315	4,026218	4,073337	4,277542	4,2908887	4,125699	-0,16518967	0,891811268	Gm10789
17210555	5,37495	5,141316	6,147968	4,74478	5,735616	5,688292	5,5547447	5,389562667	-0,165182	0,891816007	---
17454069	7,217146	7,131783	7,151062	7,05142	6,904105	7,048984	7,1666637	7,001503	-0,16516067	0,891829195	Mospd3
17349799	5,486571	5,509083	5,726648	5,192779	5,834208	5,199869	5,5741007	5,408952	-0,16514867	0,891836613	Vaultrc5
17513978	11,72771	12,11891	12,09564	11,59626	11,99604	11,85466	11,980753	11,81565333	-0,1651	0,891866698	---
17513980	11,72771	12,11891	12,09564	11,59626	11,99604	11,85466	11,980753	11,81565333	-0,1651	0,891866698	---
17298956	12,79575	12,80897	12,77037	12,73278	12,54498	12,60204	12,791697	12,6266	-0,16509667	0,891868758	Mat1a
17207943	3,791225	3,182407	2,963566	3,152195	3,07995	3,209767	3,3123993	3,147304	-0,16509533	0,891869583	---
17333790	4,010606	3,382102	3,630811	3,750436	3,423578	3,354323	3,6745063	3,509445667	-0,16506067	0,891891014	Vmn2r98
17403969	5,3668	5,195642	5,410607	5,087366	5,176807	5,213828	5,3243497	5,159333667	-0,165016	0,891918628	2700069I18Rik // 2700069I18Rik
17478956	9,952346	10,66728	10,04952	9,791142	9,860347	10,52277	10,223049	10,05808633	-0,16496233	0,891951807	---
17274202	8,256655	8,233121	8,108511	7,97788	8,092801	8,032872	8,199429	8,034517667	-0,16491133	0,891983338	E2f6
17334065	5,348002	5,227564	5,386685	5,176171	5,260058	5,031302	5,3207503	5,155843667	-0,16490667	0,891986223	Zscan10
17367637	5,95176	5,856674	5,965252	5,845179	5,673649	5,760293	5,924562	5,759707	-0,164855	0,892018168	Nxph2
17246440	3,364227	3,3266	3,320032	3,236401	3,089881	3,190046	3,336953	3,172109333	-0,16484367	0,892025176	Olfr812
17379995	6,315766	5,591197	5,975993	5,706014	5,780442	5,901989	5,9609853	5,796148333	-0,164837	0,892029298	1700017J07Rik
17396045	4,862324	4,588071	5,040304	4,750276	4,641569	4,604359	4,830233	4,665401333	-0,16483167	0,892032596	---
17372744	3,129156	3,182384	3,215104	3,055644	3,092319	2,884214	3,175548	3,010725667	-0,16482233	0,892038366	Olfr996
17372261	6,096346	5,885951	6,115512	5,880944	5,822754	5,899804	6,032603	5,867834	-0,164769	0,892071344	---
17480491	8,863872	8,983714	8,872455	8,693	8,681003	8,851777	8,9066803	8,741926667	-0,16475367	0,892080825	Prkrir
17371123	8,159436	8,32185	8,094643	8,067944	7,917307	8,096457	8,1919763	8,027236	-0,16474033	0,89208907	Psmd14
17486586	3,661101	4,371666	4,215406	3,92276	3,92276	3,908443	4,0827243	3,917987667	-0,16473667	0,892091337	1810019N24Rik
17242653	7,121288	7,070983	7,100325	6,874103	7,030012	6,894303	7,097532	6,932806	-0,164726	0,892097933	Polrmt
17256215	6,963841	6,722752	6,738994	6,635571	6,430006	6,86585	6,808529	6,643809	-0,16472	0,892101643	Wipf2
17290275	5,978009	6,081437	5,8412	6,058706	5,661406	5,686449	5,966882	5,802187	-0,164695	0,892117102	Akr1c12
17443689	6,438286	6,457376	6,135728	6,073125	6,308657	6,155539	6,3437967	6,179107	-0,16468967	0,8921204	Zcwpw1
17486704	4,373929	4,288954	4,691652	4,404141	4,198805	4,257544	4,4515117	4,28683	-0,16468167	0,892125347	Gm5585
17225150	5,541296	5,437933	6,244875	5,635315	5,395494	5,699287	5,741368	5,576698667	-0,16466933	0,892132973	B3gnt7
17214841	10,76985	10,89068	10,84375	10,66277	10,60955	10,73807	10,83476	10,67013	-0,16463	0,892157297	Agfg1
17210607	5,809721	5,528557	4,986658	5,277321	5,304787	5,249	5,4416453	5,277036	-0,16460933	0,892170077	---
17340983	7,509991	7,507225	7,235514	7,309077	7,203278	7,246584	7,4175767	7,252979667	-0,164597	0,892177704	Wtap
17544069	4,060252	4,378469	4,092572	4,165519	3,92313	3,948983	4,1770977	4,012544	-0,16455367	0,892204502	---
17219460	10,44521	11,06425	10,84184	10,54047	10,56639	10,7508	10,783767	10,61922	-0,16454667	0,892208831	---
17547380	4,423306	5,983669	5,350613	4,518588	5,734431	5,010944	5,2525293	5,087987667	-0,16454167	0,892211923	---
17209799	5,335057	5,904634	5,828759	5,636028	5,53523	5,403611	5,6894833	5,524956333	-0,164527	0,892220994	---
17541736	4,258711	4,57353	4,369635	4,250763	4,251313	4,206226	4,4006253	4,236100667	-0,16452467	0,892222437	Plac1
17240137	3,423076	3,403072	3,339591	3,272805	3,258434	3,141157	3,3885797	3,224132	-0,16444767	0,892270058	Gm4794
17271091	8,345602	8,352342	8,102402	7,929166	8,194427	8,183536	8,266782	8,102376333	-0,16440567	0,892296034	Nol11
17379389	3,556545	3,626908	3,465378	3,401173	3,354544	3,399903	3,5496103	3,385206667	-0,16440367	0,892297271	Svs6
17331903	3,936024	3,909487	3,877363	3,813336	3,71171	3,704629	3,9076247	3,743225	-0,16439967	0,892299745	Krtap6-2
17532732	4,791343	4,434425	4,585716	4,357034	4,52683	4,434425	4,603828	4,439429667	-0,16439833	0,89230057	Nudt10 // Nudt11
17311878	7,188745	6,649305	7,396303	7,072816	6,721649	6,946751	7,0781177	6,913738667	-0,164379	0,892312528	Gsdmcl1
17470674	3,756718	3,337892	3,832722	3,614915	3,425475	3,393892	3,642444	3,478094	-0,16435	0,892330464	Vmn2r27
17320928	10,42883	10,50321	10,40073	10,33971	10,24143	10,25867	10,444257	10,27993667	-0,16432	0,89234902	Slc38a2
17463308	4,339484	4,244646	4,558719	4,219219	4,290134	4,140642	4,3809497	4,216665	-0,16428467	0,892370875	Akap3
17325347	12,06228	12,15969	12,07376	11,85225	12,00384	11,94686	12,098577	11,93431667	-0,16426	0,892386133	Stfa1
17469402	4,590301	4,658889	4,825351	4,443786	4,694276	4,443786	4,6915137	4,527282667	-0,164231	0,892404071	---
17326790	3,688183	3,790623	3,807684	3,586096	3,706706	3,501012	3,7621633	3,597938	-0,16422533	0,892407576	4930529L06Rik // 4930529L06Rik
17525868	7,013916	7,078171	6,700936	6,805073	6,854747	6,640604	6,9310077	6,766808	-0,16419967	0,892423453	Ubash3b
17299946	3,902251	4,225225	4,563348	4,224678	4,285168	3,688389	4,2302747	4,066078333	-0,16419633	0,892425515	---
17457722	3,760903	3,718841	3,521112	3,519163	3,542652	3,446454	3,666952	3,502756333	-0,16419567	0,892425927	---
17399544	4,0655	3,952443	3,78809	3,78067	3,795428	3,737397	3,9353443	3,771165	-0,16417933	0,892436031	Gm19710
17512479	7,190474	7,121138	7,359726	7,098035	6,999872	7,080928	7,2237793	7,059611667	-0,16416767	0,892443248	Acd
17415542	4,195572	3,922394	4,139163	3,971115	4,015567	3,777999	4,0857097	3,921560333	-0,16414933	0,892454589	---
17449585	5,728737	5,92306	5,796945	5,725324	5,755675	5,475353	5,8162473	5,652117333	-0,16413	0,892466548	Btc
17498115	5,134049	4,875775	5,211128	4,762198	5,068508	4,897884	5,0736507	4,90953	-0,16412067	0,892472322	Gm14492
17406273	3,97109	3,894999	3,799069	3,830198	3,604624	3,738241	3,888386	3,724354333	-0,16403167	0,892527381	---
17329395	5,405607	4,736258	5,333863	4,935994	5,035362	5,01229	5,158576	4,994548667	-0,16402733	0,892530061	St6gal1
17207045	7,746254	7,998696	7,703581	7,507897	7,796037	7,652541	7,816177	7,652158333	-0,16401867	0,892535423	---
17252406	7,305475	7,951871	7,018569	7,325451	7,017252	7,441159	7,425305	7,261287333	-0,16401767	0,892536042	---
17533462	3,390211	3,262313	3,455252	3,076739	3,327878	3,211388	3,3692587	3,205335	-0,16392367	0,892594198	Gm5382
17383667	5,188805	4,965196	5,181482	4,937837	5,151963	4,753944	5,1118277	4,947914667	-0,163913	0,892600797	Gm14486
17363495	4,090556	3,686452	4,549393	3,91374	4,035772	3,885197	4,1088003	3,944903	-0,16389733	0,89261049	---
17211375	5,413957	5,469978	4,741548	4,892848	5,184868	5,056094	5,2084943	5,044603333	-0,163891	0,892614409	Paqr8
17439573	4,046403	3,931247	4,079668	3,942803	3,676413	3,946654	4,019106	3,85529	-0,163816	0,892660813	5830403M04Rik
17517573	3,855663	3,806727	4,137016	3,774601	3,59183	3,941576	3,9331353	3,769335667	-0,16379967	0,89267092	---
17238150	6,370788	6,454839	6,417462	6,222112	6,155248	6,374354	6,414363	6,250571333	-0,16379167	0,89267587	Zbtb39
17527767	7,643881	8,211046	8,096753	7,903433	7,535444	8,0215	7,9838933	7,820125667	-0,16376767	0,89269072	Gm7589
17474692	3,618897	3,808247	3,474091	3,585533	3,342848	3,481673	3,633745	3,470018	-0,163727	0,892715883	---
17209141	10,49263	10,09521	10,69321	10,24802	10,36867	10,17318	10,427017	10,26329	-0,16372667	0,89271609	---
17475222	6,744006	6,465245	7,412401	7,401066	6,102208	6,627244	6,873884	6,710172667	-0,16371133	0,892725578	---
17306999	3,886142	3,732396	3,807034	3,744524	3,773898	3,416042	3,808524	3,644821333	-0,16370267	0,892730941	Gzmg
17262452	4,464131	4,037306	4,307376	4,176516	4,137594	4,003622	4,2696043	4,105910667	-0,16369367	0,89273651	---
17233357	8,877386	8,931921	8,742685	8,782627	8,638333	8,640023	8,850664	8,686994333	-0,16366967	0,892751361	Hsf2
17203361	5,332937	5,656233	5,473038	5,783244	5,388259	4,799877	5,4874027	5,323793333	-0,16360933	0,892788697	---
17494868	4,319568	4,454382	4,677877	4,317174	4,296153	4,347699	4,4839423	4,320342	-0,16360033	0,892794266	Trim66
17202931	4,494368	5,414148	3,979416	4,712419	4,655262	4,029517	4,6293107	4,465732667	-0,163578	0,892808087	---
17363068	3,751964	3,429838	3,395525	3,546236	3,429838	3,110534	3,5257757	3,362202667	-0,163573	0,892811181	Olfr1427
17205879	3,978404	3,675387	3,184127	3,402312	3,835501	3,109428	3,6126393	3,449080333	-0,163559	0,892819845	---
17512709	4,083143	4,091663	4,211046	4,026171	4,030216	3,838971	4,1286173	3,965119333	-0,163498	0,892857596	Tmed6
17476260	3,746016	3,741367	3,924035	3,965184	3,173198	3,782582	3,803806	3,640321333	-0,16348467	0,892865848	---
17365796	5,171701	4,991076	5,313777	5,033923	5,033186	4,919038	5,1588513	4,995382333	-0,163469	0,892875544	4930552P12Rik
17357416	4,800761	4,399415	4,936965	4,759646	4,58007	4,307033	4,7123803	4,548916333	-0,163464	0,892878638	---
17448784	7,429241	7,026304	7,19825	7,153351	6,026959	7,983118	7,2179317	7,054476	-0,16345567	0,892883796	---
17247506	6,168758	5,458008	5,857914	5,463601	5,716613	5,814138	5,8282267	5,664784	-0,16344267	0,892891841	---
17250769	3,811419	3,865715	4,018517	3,629098	3,828918	3,747308	3,8985503	3,735108	-0,16344233	0,892892048	---
17304431	4,446096	4,505942	4,646232	4,453398	4,408125	4,246542	4,5327567	4,369355	-0,16340167	0,892917217	---
17550811	3,815434	3,745314	3,77568	3,452704	3,634923	3,758666	3,7788093	3,615431	-0,16337833	0,892931659	---
17364337	4,114417	3,656577	4,034791	3,893359	3,680089	3,742325	3,9352617	3,771924333	-0,16333733	0,892957035	---
17547935	4,928476	4,650996	4,926267	4,729486	4,62722	4,659026	4,8352463	4,671910667	-0,16333567	0,892958067	AA066038
17538835	8,449239	8,578769	7,44864	8,19978	8,154928	7,631942	8,1588827	7,99555	-0,16333267	0,892959924	---
17504454	6,113614	6,394743	6,349998	6,069911	6,15	6,148489	6,2861183	6,1228	-0,16331833	0,892968795	Car7
17208663	4,617223	5,488977	5,085645	4,721252	4,952378	5,028265	5,0639483	4,900631667	-0,16331667	0,892969827	---
17347925	5,852101	5,523273	5,882792	5,351259	5,686674	5,730368	5,752722	5,589433667	-0,16328833	0,892987364	---
17291340	5,622303	5,596258	5,391648	5,316403	5,318042	5,485911	5,5367363	5,373452	-0,16328433	0,89298984	C530050E15Rik
17212947	7,184983	7,321435	7,158614	6,987287	6,968503	7,219444	7,2216773	7,058411333	-0,163266	0,893001188	Spats2l
17204609	4,696613	4,890475	4,492764	4,916487	4,546635	4,126956	4,693284	4,530026	-0,163258	0,89300614	---
17207441	7,831464	7,66957	8,041222	7,583117	7,752543	7,716845	7,8474187	7,684168333	-0,16325033	0,893010886	---

17391434	7,992589	8,788518	8,040866	7,849802	8,289351	8,193222	8,273991	8,110791667	-0,16319933	0,893042455 ---
17494095	3,951913	3,666436	3,978066	3,470142	3,84971	3,787019	3,8654717	3,702290333	-0,16318133	0,893053597 Olfr545
17496015	4,497192	4,358295	4,667606	4,331354	4,358295	4,344167	4,5076977	4,344605333	-0,16309233	0,893108691 Zkscan2
17287126	6,664954	7,050512	6,536569	6,654594	6,677229	6,431262	6,7506783	6,587695	-0,16298333	0,893176171 Ippk
17402296	8,42745	8,66764	8,422938	8,33405	8,254559	8,440523	8,5060093	8,343044	-0,16296533	0,893187315 Dnttip2
17470050	4,444337	4,421938	4,617895	4,338209	4,553434	4,103771	4,4947233	4,331804667	-0,16291867	0,893216207 Olfr215
17257562	7,960637	8,238896	8,410907	7,888256	8,220849	8,012584	8,20348	8,040563	-0,162917	0,893217239 ---
17462839	5,69182	5,594861	5,366116	5,408628	5,306294	5,449179	5,5509323	5,388033667	-0,16289867	0,89322859 ---
17203459	5,477466	4,469141	4,628602	4,696663	4,634316	4,755596	4,858403	4,695525	-0,162878	0,893241385 ---
17501117	5,108564	5,207191	5,276072	5,042922	4,994895	5,065385	5,1972757	5,034400667	-0,162875	0,893243243 Gm19744
17486554	4,752906	4,418569	4,605477	4,536551	4,204281	4,54754	4,5923173	4,429457333	-0,16286	0,89325253 Vmn2r56
17292023	5,575277	5,822123	5,932145	5,548171	5,381852	5,911122	5,776515	5,613715	-0,1628	0,89328968 Gcnt2
17234175	9,08743	8,894627	8,883942	8,845081	8,740148	8,792412	8,955333	8,792547	-0,162786	0,893298349 Ipmk
17372870	3,265234	3,086922	3,201767	3,187979	2,917205	2,960418	3,184641	3,021867333	-0,16277367	0,893305985 Olfr1130
17326594	3,605147	3,632627	3,853067	3,477114	3,500681	3,624733	3,696947	3,534176	-0,162771	0,893307636 4931420L22Rik
17299613	4,300737	3,9046	4,243629	4,170074	3,953183	3,837409	4,1496553	3,986888667	-0,16276667	0,89331032 Vmn2r88
17457603	3,969368	3,845775	3,796681	3,683904	3,64961	3,790096	3,870608	3,70787	-0,162738	0,89332807 Tas2r108
17406233	4,38762	4,469061	4,197485	4,201972	4,326605	4,03739	4,3513887	4,188655667	-0,162733	0,893331166 Npy2r
17291353	10,83908	10,83985	10,65037	10,43609	10,67863	10,72645	10,776433	10,61372333	-0,16271	0,893345408 Top2a // Top2a
17317173	3,648974	4,00458	3,935274	3,731076	3,552859	3,816838	3,8629427	3,700257667	-0,162685	0,893360889 ---
17361494	7,5229	7,216583	7,380836	7,303733	7,081105	7,247428	7,3734397	7,210755333	-0,16268433	0,893361302 Pacs1
17549330	4,791134	4,764974	4,933084	4,644352	4,775061	4,581814	4,8297307	4,667075667	-0,162655	0,893379466 ---
17441072	5,296038	4,921711	5,083675	5,057304	5,027541	4,728632	5,1004747	4,937825667	-0,162649	0,893383181 ---
17458043	5,2417	5,324067	5,2098	5,00728	5,159786	5,120578	5,2585223	5,095881333	-0,162641	0,893388135 Cntnap2
17473417	4,963188	4,575496	4,743266	4,606532	4,676682	4,51104	4,76065	4,598084667	-0,16256533	0,893434993 Nat14
17237400	4,608031	4,386577	4,755253	4,470446	4,417161	4,374698	4,583287	4,420768333	-0,16251867	0,893463893 Lgr5 // Lgr5
17449528	8,762732	9,168309	9,030727	8,995829	8,892861	8,58567	8,987256	8,824786667	-0,16246933	0,893494446 ---
17457773	5,579792	5,419446	6,191226	5,471654	5,297099	5,934451	5,7301547	5,567734667	-0,16242	0,893525 ---
17349357	5,883346	5,45986	6,288345	5,776379	5,717108	5,650924	5,8771837	5,714803667	-0,16238	0,893549774 Gm10549
17283770	8,327356	8,308889	8,39372	7,916577	8,275184	8,351135	8,3433217	8,180965333	-0,16235633	0,893564432 Glrx5 // Glrx5
17253304	4,671329	4,844225	4,976677	4,571565	4,52135	4,912314	4,8307437	4,668409667	-0,162334	0,893578265 Taok1 // Taok1
17528271	5,909671	5,707238	5,848026	5,968206	5,332021	5,677809	5,821645	5,659345333	-0,16229967	0,893599531 Gm17749
17240584	6,083013	5,555339	5,859179	5,427813	5,432971	6,14986	5,8325103	5,670214667	-0,16229567	0,893602008 ---
17527016	7,044902	7,212095	6,74538	6,751865	6,629447	7,134192	7,0007923	6,838501333	-0,162291	0,893604899 Zc3h12c
17217768	4,783954	4,346334	4,696615	4,58389	4,336305	4,419949	4,6089677	4,446714667	-0,162253	0,893628436 ---
17488336	6,712253	6,715153	6,199419	6,111159	6,639416	6,389917	6,542275	6,380164	-0,162111	0,893716398 Zfp780b
17407476	5,64356	5,370027	5,15101	5,260695	5,157051	5,260525	5,388199	5,226090333	-0,16210867	0,893717843 Kprp
17469605	5,626493	5,942537	5,418133	5,378336	5,525855	5,596678	5,6623877	5,500289667	-0,162098	0,893724451 ---
17460591	9,200135	9,034401	9,130627	8,991584	9,082651	8,804739	9,121721	8,959658	-0,162063	0,893746133 ---
17300116	5,864144	5,941325	5,950426	5,537124	5,970281	5,762317	5,9186317	5,756574	-0,16205767	0,893749437 ---
17279427	7,333574	7,483583	7,329857	7,170455	7,363335	7,127153	7,382338	7,220314333	-0,16202367	0,8937705 Btbd6
17299910	7,054503	6,574009	6,73966	6,644746	6,527665	6,70973	6,7893907	6,627380333	-0,16201033	0,893778761 ---
17428801	11,37109	11,50769	11,57037	11,36006	11,39701	11,20617	11,48305	11,32108	-0,16197	0,893803748 Snord55
17509694	4,542845	3,984038	4,44736	4,385916	4,024702	4,077786	4,3247477	4,162801333	-0,16194633	0,893818411 ---
17340307	7,873529	8,10844	7,914082	7,679872	7,782275	7,948082	7,9653503	7,803409667	-0,16194067	0,893821922 Msh6
17494806	3,900269	3,76627	3,687003	3,832807	3,45233	3,582633	3,784514	3,62259	-0,161924	0,893832247 Olfr504
17206577	3,602498	3,667886	4,677482	3,189692	3,712992	4,559496	3,982622	3,820726667	-0,16189533	0,893850008 ---
17507794	4,369169	4,481166	4,616014	4,401369	4,289372	4,290112	4,488783	4,326951	-0,161832	0,893889249 ---
17242879	6,553368	6,400414	6,53863	6,322094	6,239408	6,445537	6,4974707	6,335679667	-0,161791	0,893914652 Adamtsl5
17434105	6,594375	6,75948	6,785424	6,734256	6,306102	6,613563	6,713093	6,551307	-0,161786	0,89391775 Ccl21c
17341065	4,621374	4,373712	4,675063	4,642437	4,090119	4,452243	4,5567163	4,394933	-0,16178333	0,893919403 4930488N24Rik
17202089	3,231958	3,605671	3,571414	3,163656	3,29884	3,461229	3,469681	3,307908333	-0,16177267	0,893926012 ---
17226602	3,935048	4,018861	4,299466	3,961169	3,853723	3,953221	4,0844583	3,922704333	-0,161754	0,893937578 ---
17334833	5,669919	6,102568	5,817734	5,759575	5,431436	5,913955	5,863407	5,701655333	-0,16175167	0,893939024 Pigq // Pigq
17206691	4,431901	4,57517	5,096511	4,720919	4,261114	4,636297	4,701194	4,539443333	-0,16175067	0,893939644 ---
17353453	4,812915	4,574115	4,534903	4,549758	4,449574	4,437464	4,6406443	4,478932	-0,16171233	0,893963397 4933408B17Rik
17208511	4,126155	5,362024	6,15641	4,69326	5,017701	5,448508	5,214863	5,053156333	-0,16170667	0,893966908 ---
17548360	4,67948	4,528645	4,65175	4,484629	4,583668	4,306566	4,6199583	4,458287667	-0,16167067	0,893989216 Mul1 // Mul1
17350301	7,878235	8,117912	7,676668	7,720739	7,639016	7,828079	7,8909383	7,729278	-0,16166033	0,893995619 Ythdc2
17315463	4,432039	4,644306	4,646992	4,284326	4,44412	4,509911	4,5744457	4,412785667	-0,16166	0,893995826 ---
17205673	3,981869	3,926543	3,914759	3,226549	4,237303	3,874341	3,941057	3,779397667	-0,16165933	0,893996239 ---
17467031	6,291433	6,443602	6,337952	6,305483	6,023999	6,258536	6,3576623	6,196006	-0,16165633	0,893998098 Ggct
17453703	6,690381	7,010619	6,891411	6,834953	6,703243	6,569287	6,864137	6,702494333	-0,16164267	0,894006567 Prkrip1
17431298	5,751576	5,626602	5,556623	5,476298	5,510684	5,462931	5,6449337	5,483304333	-0,16162933	0,894014829 1700021N21Rik
17248986	6,482963	6,307733	6,378424	6,135951	6,381187	6,167107	6,3897067	6,228081667	-0,161625	0,894017514 Olfr1382
17274893	8,676292	8,619692	8,604301	8,399186	8,563729	8,452515	8,6334283	8,47181	-0,16161833	0,894021646 Sypl
17357036	3,679643	3,843304	3,714024	3,612742	3,484574	3,655111	3,745657	3,584142333	-0,16151467	0,894085889 ---
17491403	4,659667	5,108617	4,790632	4,736563	4,731059	4,606835	4,852972	4,691485667	-0,16148633	0,894103448 ---
17389936	6,264108	5,84498	6,224696	6,109175	6,075407	5,664855	6,1112613	5,949812333	-0,161449	0,894126586 ---
17285067	5,435636	5,106281	4,860807	4,795826	5,155521	4,96707	5,1342413	4,972805667	-0,16143567	0,894134849 Idi2
17328432	9,683022	9,84788	9,674873	9,517622	9,602897	9,601006	9,7352583	9,573841667	-0,16141667	0,894146625 Mcm4
17431841	6,69761	6,492204	6,592333	6,482435	6,590196	6,225398	6,594049	6,432676333	-0,16137267	0,894173895 Otud3
17374455	8,017416	8,32765	8,068139	8,086696	7,918571	7,923826	8,137735	7,976364333	-0,16137067	0,894175135 Spred1
17430824	8,208384	8,169156	8,248479	7,966034	8,159056	8,016883	8,208673	8,047324333	-0,16134867	0,894188771 Snhg3
17279153	10,67077	11,11665	10,8842	10,6763	10,52403	10,98725	10,89054	10,72919333	-0,16134667	0,89419001 ---
17517605	9,296108	9,385265	9,506283	9,322236	9,247691	9,133741	9,3958853	9,234556	-0,16132933	0,894200753 Imp3
17543034	4,100086	3,541795	3,414708	3,335531	3,62805	3,609093	3,6855297	3,524224667	-0,161305	0,894215836 ---
17356482	7,426873	7,549967	7,5764	7,557044	7,187699	7,324618	7,5177467	7,356453667	-0,161293	0,894223274 Rnaseh2c
17529679	4,469557	4,332417	4,76819	4,242231	4,462627	4,381465	4,523388	4,362107667	-0,16128033	0,894231125 Zic1
17203879	6,390146	6,701202	6,378474	6,103608	6,413303	6,469126	6,4899407	6,328679	-0,16126167	0,894242695 ---
17540950	5,804994	5,856317	5,45888	5,461231	5,569685	5,605507	5,7067303	5,545474333	-0,161256	0,894246208 Akap17b
17441723	4,918075	4,703238	4,906187	4,666471	4,5874	4,790149	4,8425	4,681273333	-0,16122667	0,89426439 ---
17528355	8,660872	8,684209	8,552692	8,458638	8,496532	8,458946	8,632591	8,471372	-0,161219	0,894269142 Usp3
17326237	2,991924	2,826638	3,104917	2,821798	2,735292	2,882751	2,974493	2,813280333	-0,16121267	0,894273068 ---
17473155	6,017019	6,061763	6,194998	5,781775	6,062612	5,94578	6,09126	5,930055667	-0,16120433	0,894278233 Cacng7
17407386	4,478667	4,13525	4,256362	4,167939	4,25358	3,965194	4,290093	4,128904333	-0,16118867	0,894287945 Lelp1
17464571	4,067551	3,792015	3,686706	3,816723	3,527782	3,718207	3,8487573	3,687570667	-0,16118667	0,894289184 Mir489
17203207	3,936648	3,231255	3,908281	3,539426	2,831468	4,221761	3,6920613	3,530885	-0,16117633	0,89429559 ---
17533065	3,502536	3,973676	4,179826	3,717554	3,517159	3,937799	3,885346	3,724170667	-0,16117533	0,89429621 ---
17449443	2,892952	3,361422	3,334029	3,175474	2,991822	2,93766	3,1961343	3,034985333	-0,161149	0,894312533 ---
17371078	4,14015	4,280137	4,087814	4,021237	4,243503	3,759938	4,169367	4,008226	-0,161141	0,894317493 ---
17246459	4,942322	4,952943	4,833617	4,89722	4,549192	4,799084	4,9096273	4,748498667	-0,16112867	0,894325138 Olfr824
17244189	7,371085	7,790782	7,724672	7,585184	7,49914	7,318886	7,6288463	7,467736667	-0,16110967	0,894336916 ---
17245367	3,658184	3,569824	3,671673	3,322873	3,784935	3,308623	3,633227	3,472143667	-0,16108333	0,89435324 ---
17328621	4,364412	4,340598	4,41922	4,026335	4,29306	4,321602	4,3747433	4,213665667	-0,16107767	0,894356753 Mir130b
17516784	5,273257	5,276219	4,863919	5,010637	4,960847	4,958723	5,1377983	4,976735667	-0,16106267	0,894366052 Fxyd2
17306424	9,998523	10,2827	10,11219	10,05177	9,884974	9,973485	10,131138	9,970076333	-0,16106133	0,894366879 ---
17524698	9,255301	9,007925	9,025578	8,820899	8,982553	9,002208	9,096268	8,93522	-0,161048	0,894375144 Cdkn2d
17237130	3,24711	3,409209	3,204413	3,113722	3,237534	3,02638	3,2869107	3,125878667	-0,161032	0,894385063 ---
17550779	3,252679	3,021021	3,080153	2,977757	3,046984	2,84604	3,117951	2,956927	-0,161024	0,894390023 ---
17437850	5,378396	5,553225	5,452376	5,306271	5,325387	5,269396	5,4613323	5,300351333	-0,160981	0,894416681 ---
17300944	7,325624	7,495487	7,365192	7,16411	7,317075	7,222197	7,3954343	7,234460667	-0,16097367	0,894421227 Mrp63
17510212	6,200442	6,175659	6,132624	5,881336	6,154265	5,990252	6,169575	6,008617667	-0,16095733	0,894431354 Kcnn1
17200971	6,561518	6,998091	6,54557	6,746325	6,472519	6,403502	6,7017263	6,540782	-0,16094433	0,894439413 ---
17486701	3,605672	3,869382	3,500234	3,579973	3,337711	3,574824	3,6584293	3,497502667	-0,16092667	0,894450366 Gm19482
17334495	8,445934	8,538634	8,696463	8,336054	8,368685	8,493542	8,5603437	8,399427	-0,16091667	0,894456566 Nme3
17226531	5,229821	5,22074	5,267412	5,120633	4,993963	5,120633	5,2393243	5,078409667	-0,16091467	0,894457806 Lct
17287296	4,285643	4,242439	4,167661	3,918615	4,024483	4,269937	4,2319143	4,071011667	-0,16090267	0,894465246 Gm8777
17475187	7,40558	7,332246	7,416733	7,285577	7,092555	7,293751	7,384853	7,223961	-0,160892	0,894471859 9130221H12Rik
17494429	3,893989	4,047257	4,058851	4,217448	3,893989	3,406008	4,0000323	3,839148333	-0,160884	0,894476819 ---
17206437	4,721881	4,733321	4,729782	4,904246	4,483037	4,315183	4,728328	4,567488667	-0,16083933	0,894504513 ---
17354730	6,829121	7,036922	6,987485	6,724969	6,810756	6,835312	6,951176	6,790345667	-0,16083033	0,894510094 Slc26a2
17413528	7,84725	7,979286	8,009459	7,794832	7,636312	7,922369	7,9453317	7,784504333	-0,16082733	0,894511954 Melk
17321217	6,303873	6,093709	6,413723	5,947289	6,146825	6,234777	6,270435	6,109630333	-0,16080467	0,894526008 Olfr283
17206377	3,782776	3,104306	3,286886	3,04607	3,22985	3,415691	3,3913227	3,230537	-0,16078567	0,894537789 ---
17322227	5,839297	6,045217	5,794977	5,781806	5,726003	5,689411	5,8931637	5,732406667	-0,160757	0,894555563 ---
17219848	7,816553	8,130696	7,801368	7,719784	7,689853	7,856711	7,9162057	7,755449333	-0,16075633	0,894555977 Exo1
17481161	4,877733	4,387209	4,049793	4,640335	4,027747	4,164464	4,438245	4,277515333	-0,16072967	0,894572512 Olfr572
17445898	4,493845	4,147978	4,458202	4,170806	4,322243	4,124817	4,366675	4,205955333	-0,16071967	0,894578713 Slc26a5
17286150	4,085049	4,097799	4,062322	3,785398	4,253666	3,723969	4,0817233	3,921011	-0,16071233	0,89458326 Prl3b1
17536381	3,667503	3,497956	4,346511	3,726757	3,482378	3,820782	3,8373233	3,676639	-0,16068433	0,894600622 ---
17493972	4,460833	4,249869	4,216827	4,182317	3,964265	4,298896	4,3091763	4,148492667	-0,16068367	0,894601035 3200002M19Rik // 3200002M19Rik
17467104	3,221926	3,184374	3,445772	2,982854	3,144221	3,243138	3,284024	3,123404333	-0,16061967	0,894640722 ---
17210045	6,043237	6,111249	5,662259	5,894253	5,744318	5,696352	5,938915	5,778307667	-0,16060733	0,89464837 ---
17547612	5,195914	5,127019	5,621787	5,191847	5,240497	5,030645	5,3149067	5,154329667	-0,160577	0,894667181 Gm10278
17369838	4,521993	4,189236	4,169387	3,997849	4,156183	4,244901	4,2935387	4,132977667	-0,160561	0,894677103 ---
17349361	7,725787	8,142601	7,93565	7,79675	7,921231	7,604377	7,9346793	7,774119333	-0,16056	0,894677723 2410004N09Rik
17294016	8,205056	8,450614	8,609933	8,329903	8,333012	8,121143	8,4218677	8,261352667	-0,160515	0,89470563 A530054K11Rik
17321823	5,467306	5,573766	6,063401	5,347078	5,582115	5,693803	5,701491	5,540998667	-0,16049233	0,894719687 Krt80
17233532	3,092203	3,081098	3,118135	2,978894	3,055734	2,775387	3,0971453	2,936671667	-0,16047367	0,894731264 ---
17203449	6,468116	6,374133	5,970798	5,923526	6,740281	5,668074	6,2710157	6,110627	-0,16038867	0,894783981 ---
17316780	6,826921	7,075036	6,500442	6,547033	6,594882	6,779323	6,8007997	6,640412667	-0,160387	0,894785014 Angpt1
17359394	7,166866	7,093678	7,070308	6,985112	6,920615	6,943997	7,110284	6,949908	-0,160376	0,894791837 Ccnj
17367443	4,559497	4,329397	4,327743	4,208442	4,297175	4,22999	4,4055457	4,245202333	-0,16034333	0,894812098 ---

17206697	3,641625	3,776661	4,094137	3,638289	3,794809	3,59831	3,8374743	3,677136	-0,16033833	0,894815199	---
17392209	8,52892	8,899356	8,566159	8,455985	8,466038	8,591636	8,6648117	8,504553	-0,16025867	0,894864613	Esf1
17305856	9,103263	9,29952	9,116831	9,017776	8,999496	9,021581	9,1732047	9,012951	-0,16025367	0,894867714	Exoc5
17337566	3,174084	3,524737	3,268494	2,984306	3,258284	3,243993	3,3224383	3,162194333	-0,160244	0,89487371	Olfr107
17307403	9,955105	10,16086	10,06738	9,833094	9,985869	9,883664	10,061115	9,900875667	-0,16023933	0,894876605	Nupl1
17509676	4,833166	4,616037	4,616649	4,59784	4,552489	4,434832	4,6886173	4,528387	-0,16023033	0,894882187	Gm10664
17313654	7,831923	7,95599	8,201906	7,863878	7,795524	7,849739	7,9966063	7,836380333	-0,160226	0,894884875	Tspo
17540237	5,968116	5,889179	6,108312	5,874025	5,83529	5,775656	5,9885357	5,828323667	-0,160212	0,894893559	Gm10489
17264538	4,736817	4,828137	4,89902	4,691711	4,55107	4,740706	4,8213247	4,661162333	-0,16016233	0,894924368	Gucy2e
17201843	3,605428	3,162144	4,393432	3,444534	3,674273	3,56172	3,7203347	3,560175667	-0,160159	0,894926435	---
17335431	5,068117	4,680926	4,949248	4,682485	4,792123	4,743209	4,8994303	4,739272333	-0,160158	0,894927056	1700030A11Rik
17220464	4,752008	4,698491	4,952711	4,441126	4,739705	4,742048	4,80107	4,640959667	-0,16011033	0,894956624	Hhipl2
17422978	3,526206	3,256954	3,359094	3,381065	3,021179	3,259685	3,3807513	3,220643	-0,16010833	0,894957865	---
17389994	4,114688	4,265385	4,438285	4,128696	4,108957	4,100445	4,272786	4,112699333	-0,16008667	0,894971306	Mapkbp1 // Mapkbp1
17327331	8,396025	8,455918	8,481421	8,260075	8,331079	8,261953	8,4444547	8,284369	-0,16008567	0,894971926	Chaf1b
17359965	9,609065	9,385909	9,459818	9,205847	9,601657	9,167054	9,4849307	9,324852667	-0,160078	0,894976682	---
17204977	3,425875	3,413514	3,56083	3,151805	3,053003	3,715233	3,4667397	3,306680333	-0,16005933	0,894988262	---
17252400	9,7843	9,406406	9,561144	9,394867	9,430513	9,44644	9,58395	9,42394	-0,16001	0,895018867	---
17525379	7,378239	7,429241	7,558563	7,256237	7,405015	7,22479	7,4553477	7,295347333	-0,16000033	0,895024864	Fam118b
17455159	9,954081	9,766905	10,03695	9,786158	9,645879	9,846028	9,919312	9,759355	-0,159957	0,895051748	Cyp3a44
17261166	3,878463	3,600988	3,693565	3,321009	3,729025	3,643126	3,7243387	3,564386667	-0,159952	0,89505485	---
17262679	6,382248	6,304667	6,434736	6,261871	6,055155	6,324898	6,3738837	6,213974667	-0,159909	0,895081528	A430108G06Rik
17268071	5,467116	4,774066	5,69661	5,021389	5,592553	4,844124	5,3125973	5,152688667	-0,15990867	0,895081734	Tmem92
17493405	7,743833	7,242106	7,577904	7,343941	7,325775	7,414435	7,521281	7,361383667	-0,15989733	0,895088766	---
17489682	5,732049	5,447575	5,912954	5,517014	5,918797	5,177079	5,697526	5,53763	-0,159896	0,895089593	Kctd15
17497303	5,056146	4,977923	5,372512	4,857476	5,1834	4,886264	5,135527	4,975713333	-0,15981367	0,895140677	Fam196a
17531357	3,661706	4,059053	3,44928	3,468678	3,491985	3,729982	3,7233463	3,563548333	-0,159798	0,895150397	Fbxw22
17548640	3,724676	3,497201	3,432041	3,473355	3,438614	3,26271	3,551306	3,391559667	-0,15974633	0,895182456	---
17321221	4,149824	3,993668	4,212714	3,990443	4,034039	3,852541	4,1187353	3,959007667	-0,15972767	0,895194038	Lalba
17202501	6,913948	7,823025	7,097004	7,456307	6,587914	7,310652	7,2779923	7,118291	-0,15970133	0,895210378	---
17277267	6,837512	7,0343	6,692657	6,691026	6,492663	6,901857	6,854823	6,695182	-0,159641	0,895247817	Aldh6a1 // Aldh6a1
17364707	8,352553	8,330036	8,458998	8,157343	8,34065	8,164758	8,380529	8,220917	-0,159612	0,895265812	Arhgap19
17440194	8,78221	8,629625	8,726333	8,492392	8,60673	8,560368	8,7127227	8,553163333	-0,15955933	0,895298495	Dr1
17201375	3,756519	3,82134	3,612178	3,429802	3,996842	3,284769	3,7300123	3,570471	-0,15954133	0,895309666	---
17409540	10,64744	10,61342	10,89129	11,11064	10,4401	10,12281	10,717383	10,55785	-0,15953333	0,89531463	Amy2a5
17281895	8,055402	8,221657	7,900911	7,802119	7,850868	8,046427	8,0593233	7,899804667	-0,15951867	0,895323732	Trmt5
17289239	8,664282	8,540462	8,398294	8,22255	8,520238	8,38171	8,534346	8,374832667	-0,15951333	0,895327042	Tbca
17386639	7,749594	7,724546	7,461806	7,481986	7,336988	7,638528	7,6453153	7,485834	-0,15948133	0,895346901	Lnp
17517379	4,838103	4,604384	5,118298	4,633793	4,801063	4,647698	4,853595	4,694184667	-0,15941033	0,895390966	Sh2d7
17233702	4,819159	4,848953	4,917533	4,912769	4,536959	4,657719	4,8618817	4,702482333	-0,15939933	0,895397793	Nodal
17286916	3,889142	3,980263	4,190884	4,047985	3,801384	3,73289	4,0200963	3,860753	-0,15934333	0,895432549	Gm5083
17496352	7,279759	6,905648	6,733375	6,859684	6,713838	6,867256	6,9729273	6,813592667	-0,15933467	0,895437928	Ccdc101 // Ccdc101
17203465	6,040153	5,868506	6,042804	5,856272	5,727028	5,890193	5,983821	5,824497667	-0,15932333	0,895444963	---
17245463	4,501802	4,803865	4,582711	4,658644	4,413335	4,338491	4,6294593	4,470156667	-0,15930267	0,89545779	---
17364986	8,929778	8,61799	8,688326	8,776505	8,470406	8,51132	8,7453647	8,586077	-0,15928767	0,8954671	---
17224617	6,048964	5,724909	5,985196	5,574247	5,815892	5,89111	5,9196897	5,760416333	-0,15927333	0,895475997	LOC100862164
17437278	9,523389	9,66259	9,621742	9,412897	9,337669	9,579397	9,6025737	9,443321	-0,15925267	0,895488825	Ncapg
17200949	3,752891	4,173892	4,090927	3,625546	4,073814	3,840697	4,0059033	3,846685667	-0,15921767	0,89551055	---
17503866	4,923491	4,822942	4,722647	4,567081	4,66817	4,756191	4,8230267	4,663814	-0,15921267	0,895513653	Slc6a2
17472756	4,558686	4,652302	4,576455	4,170611	4,652238	4,486973	4,5958143	4,436607333	-0,159207	0,895517171	---
17207917	5,164009	4,58199	6,083699	5,127447	5,145728	5,07897	5,276566	5,117381667	-0,15918433	0,895531241	---
17441949	7,310053	7,580588	7,429241	7,304484	7,403806	7,134043	7,4399607	7,280777667	-0,159183	0,895532068	Aldh2 // Aldh2
17548336	3,555048	3,781075	3,354862	3,493508	3,427852	3,292084	3,5636617	3,404481333	-0,15918033	0,895533724	LOC636187
17238695	3,010978	2,991066	2,87545	2,840482	2,800934	2,758567	2,9591647	2,799994333	-0,15917033	0,895539931	Olfr813
17548906	7,138634	6,977185	7,005609	6,843596	6,932033	6,868355	7,040476	6,881328	-0,159148	0,895553794	D1Ertd448e
17542897	4,027103	3,937458	3,941779	3,763699	4,008042	3,65721	3,96878	3,809650333	-0,15912967	0,895565175	Gm6927
17206009	6,423838	6,580274	3,788274	5,784219	4,691483	5,839297	5,597462	5,438333	-0,159129	0,895565589	---
17547076	3,833211	5,456873	4,834887	4,100209	5,39702	4,150367	4,7083237	4,549198667	-0,159125	0,895568072	LOC100039675
17479069	6,079883	6,118201	6,600237	6,035626	6,235928	6,049548	6,266107	6,107034	-0,159073	0,895600352	Lysmd4
17457971	6,710461	6,215526	6,344429	6,213764	6,321262	6,258193	6,423472	6,264406333	-0,15906567	0,895604904	Olfr452
17303433	5,822807	5,960298	5,937054	5,87663	5,624848	5,741579	5,9067197	5,747685667	-0,159034	0,895624563	Fam107a
17285687	5,669599	6,00936	6,082056	5,864369	5,601641	5,818102	5,9203383	5,761370667	-0,15896767	0,895665743	Hist1h4m
17528583	4,724851	4,674437	4,832145	4,755837	4,53094	4,467763	4,743811	4,584846667	-0,15896433	0,895667813	---
17366484	6,945322	7,196106	7,06166	6,894553	6,961877	6,869879	7,067696	6,908769667	-0,15892633	0,895691405	Bend7
17500957	4,950301	5,167684	5,24245	5,070158	4,979178	4,834352	5,120145	4,961229333	-0,15891567	0,895698027	Lrp2bp
17551119	5,173528	5,284367	5,065893	4,831939	5,108038	5,107179	5,174596	5,015718667	-0,15887733	0,895721827	---
17439960	3,314497	3,501966	3,440123	3,260337	3,223949	3,295763	3,418862	3,260016333	-0,15884567	0,895741488	LOC100503112 // LOC100503112
17299773	7,894021	7,343112	7,89392	7,536629	7,504581	7,613362	7,710351	7,551524	-0,158827	0,895753077	---
17317801	4,984618	5,266396	5,35101	4,931783	5,131918	5,061904	5,2006747	5,041868333	-0,15880633	0,895765909	Kcnk9
17547883	4,511153	4,615139	4,903115	4,489961	4,644277	4,41886	4,676469	4,517699333	-0,15876967	0,895788676	Ptk2 // Ptk2
17425028	10,91648	11,01731	11,08683	10,73143	10,87166	10,94123	11,006873	10,84810667	-0,15876667	0,895790538	Hemgn
17370804	5,115634	5,513033	5,579493	5,491622	5,006437	5,233827	5,40272	5,243962	-0,158758	0,89579592	Gm13540 // Bloc1s2
17472233	4,287914	3,9124	4,340818	3,928379	4,085632	4,050886	4,1803773	4,021632333	-0,158745	0,895803992	---
17354101	6,803682	6,631678	6,907321	6,686986	6,780446	6,399082	6,7808937	6,622171333	-0,15872233	0,895818066	Dpysl3
17317044	3,435254	3,472517	3,37998	3,295018	3,148105	3,368675	3,4292503	3,270599333	-0,158651	0,89586236	Mir28c // Mir28c
17477679	7,349788	7,169633	7,135079	6,794016	7,446363	6,938169	7,2181667	7,059516	-0,15865067	0,895862567	Nosip
17398509	10,82046	11,11317	11,12065	10,81823	10,86508	10,89511	11,018093	10,85947333	-0,15862	0,895881611	LOC100862155
17333184	5,926055	6,088211	5,984185	5,692732	5,949106	5,880882	5,9994837	5,840906667	-0,158577	0,895908313	---
17455494	9,22812	9,30329	9,568223	9,261271	9,227304	9,135378	9,3665443	9,207984333	-0,15856	0,89591887	---
17308361	9,040244	8,984975	9,164829	8,937106	8,848063	8,929237	9,0633493	8,904802	-0,15854733	0,895926736	Bmp1
17524904	6,521297	6,484028	6,555074	6,528387	6,276523	6,280115	6,520133	6,361675	-0,158458	0,895982215	Zfp653
17436927	4,956158	4,92782	5,307907	4,822054	5,288487	4,605977	5,0639617	4,905506	-0,15845567	0,895983664	Psapl1
17204675	5,441532	6,225595	5,945347	6,016351	5,108429	6,012352	5,8708247	5,712377333	-0,15844733	0,895988839	---
17206211	3,044846	3,137095	3,065585	3,034678	2,950986	2,786772	3,0825087	2,924145333	-0,15836333	0,896041009	---
17232786	5,379281	4,94338	5,061743	5,162843	4,820032	4,926574	5,1281347	4,969816333	-0,15831833	0,896068958	Wasf1
17379960	6,920504	7,117899	6,875709	6,880778	6,714133	6,844269	6,9713707	6,81306	-0,15831067	0,89607372	Ptpn1
17286412	4,479184	4,293821	4,38798	4,078614	4,415594	4,191949	4,386995	4,228719	-0,158276	0,896095252	Serpinb9f
17357018	5,712855	5,252332	5,261516	5,28276	5,154336	5,314794	5,408901	5,25063	-0,158271	0,896098358	---
17250909	5,631856	5,962401	5,81728	5,692743	5,695872	5,548159	5,8038457	5,645591333	-0,15825433	0,89610871	Zkscan6
17345696	7,252718	7,659281	7,376466	7,134767	7,24854	7,430396	7,4294883	7,271234333	-0,158254	0,896108917	Bysl
17251220	6,28884	5,898368	6,072824	6,002542	5,703988	6,07876	6,0866773	5,92843	-0,15824733	0,896113058	---
17547554	7,016603	6,791161	6,813429	6,478668	6,725376	6,94248	6,873731	6,715508	-0,158223	0,896128173	Ndufa12 // Ndufa12
17385838	7,232514	7,469675	6,99295	6,712179	7,076856	7,431566	7,231713	7,073533667	-0,15817933	0,896155297	---
17231387	8,08551	8,239689	8,181983	7,988225	8,034364	8,010137	8,1690607	8,010908667	-0,158152	0,896172275	Mthfd1l
17378438	3,873398	3,609703	4,010139	3,709562	3,542242	3,767044	3,83108	3,672949333	-0,15813067	0,896185527	Mir499
17308999	9,127224	9,675239	9,41197	9,2472	9,117679	9,375186	9,404811	9,246688333	-0,15812267	0,896190497	---
17508684	3,929938	3,945172	3,90519	3,695627	3,710394	3,899929	3,9267667	3,76865	-0,15811667	0,896194224	---
17536961	5,371561	5,949644	5,147738	5,315274	5,366244	5,313097	5,4896477	5,331538333	-0,15810933	0,896198779	Chic1
17537241	4,873206	4,868878	4,838596	4,612679	4,836471	4,657237	4,8602267	4,702129	-0,15809767	0,896206027	4933403O08Rik
17550837	4,174264	4,136544	4,290826	3,928227	4,358126	3,841037	4,2005447	4,042463333	-0,15808133	0,896216173	---
17277193	3,304644	3,136693	2,978837	2,858703	3,024117	3,06322	3,140058	2,982013333	-0,15804467	0,896238951	---
17250205	9,139627	9,151675	9,163631	9,043728	8,98964	8,947523	9,1516443	8,993630333	-0,158014	0,896258002	2310033P09Rik
17322426	4,465799	4,356989	4,100959	4,359903	4,030746	4,059121	4,3079157	4,149923333	-0,15799233	0,896271462	---
17285612	3,199505	3,601882	3,537759	2,941316	3,291214	3,63267	3,446382	3,2884	-0,157982	0,896277882	---
17332345	4,034836	4,983486	4,998517	4,615885	4,324059	4,602997	4,6722797	4,514313667	-0,157966	0,896287822	---
17470088	4,65804	4,753409	4,712092	4,186762	5,050227	4,412673	4,707847	4,549887333	-0,15795967	0,896291757	1700030F04Rik
17356401	8,033913	8,344187	7,986501	7,935511	7,933609	8,021704	8,1215337	7,963608	-0,15792567	0,89631288	Eif1ad
17547871	5,438895	5,315446	5,258464	5,13295	5,154736	5,251408	5,3376017	5,179698	-0,15790367	0,896326548	Zfp706 // Zfp706
17299998	6,820558	6,777404	6,996775	6,947344	6,547498	6,626187	6,8649123	6,707009667	-0,15790267	0,896327169	---
17506279	6,796656	6,970391	6,676076	6,708638	6,589133	6,671661	6,8143743	6,656477333	-0,157897	0,89633069	Irf8
17326445	3,291558	3,397682	3,145593	2,943443	3,147515	3,27023	3,2782777	3,120396	-0,15788167	0,896340217	Olfr183
17281453	4,053931	4,027286	4,277157	4,020249	3,979072	3,885476	4,119458	3,961599	-0,157859	0,896354299	---
17262702	7,960618	8,325465	8,180959	8,020392	7,934912	8,038228	8,1556807	7,997844	-0,15783667	0,896368175	Rad50
17493683	9,338938	9,536305	9,624055	9,4154	9,451645	9,158745	9,499766	9,34193	-0,157836	0,896368589	Snord15a
17427939	5,453847	5,327361	5,404275	5,230855	5,072167	5,409136	5,395161	5,237386	-0,157775	0,896406491	Dmrtb1
17551165	5,427999	6,509699	5,653541	5,84769	5,948707	5,321553	5,8637463	5,705983333	-0,157763	0,896413947	---
17475322	4,789625	4,288763	4,703792	4,392453	4,571071	4,345368	4,59406	4,436297333	-0,15776267	0,896414154	B3gnt8
17319524	5,681783	5,51128	5,91726	5,183613	5,645721	5,807946	5,703441	5,54576	-0,157681	0,896464899	Tef // Tef
17235939	9,266015	9,572428	9,452537	9,13056	9,450655	9,236818	9,4303267	9,272677667	-0,157649	0,896484783	---
17341608	6,44997	6,220953	6,791819	6,211058	6,397032	6,381931	6,4875807	6,330007	-0,15757367	0,896531596	Flywch1
17335467	8,851458	8,980539	9,022912	8,769957	8,828778	8,783494	8,9516363	8,794076333	-0,15756	0,896540089	Cdkn1a
17205479	5,461948	5,266078	5,717957	5,662218	5,576964	4,734133	5,4819943	5,324438333	-0,157556	0,896542575	---
17201337	3,3778	3,157548	3,708888	3,124372	3,256957	3,390286	3,4147453	3,257205	-0,15754033	0,896552311	---
17278894	9,077425	8,936111	9,014509	8,77587	8,866571	8,913027	9,0093483	8,851822667	-0,15752567	0,896561425	Ppp2r5c
17363025	6,348052	6,189959	6,267832	6,075619	6,066481	6,191189	6,2686143	6,111096333	-0,157518	0,89656619	Stx3
17310893	9,663819	9,6943	9,623259	9,507999	9,444137	9,556718	9,6604593	9,502951333	-0,157508	0,896572404	Mtdh
17208585	4,880862	4,875131	4,697188	4,65323	4,569513	4,758005	4,817727	4,660249333	-0,15747767	0,896591255	---
17204837	7,035756	7,633376	7,48302	7,370936	7,201405	7,107403	7,3840507	7,226581333	-0,15746933	0,896596434	---
17209723	4,6642	4,47722	4,430196	4,123644	4,888331	4,087239	4,523872	4,366404667	-0,15746733	0,896597677	---
17204605	6,474744	6,276592	7,111765	6,446069	6,778557	6,166078	6,6210337	6,463568	-0,15746567	0,896598713	---
17526729	4,883865	4,638371	4,767807	4,58132	4,585334	4,651143	4,7633477	4,605932333	-0,15741533	0,896629994	Htr3b
17457022	5,704445	5,430646	5,600427	5,47076	5,451698	5,340837	5,578506	5,421098333	-0,15740767	0,896634759	---
17342002	8,295486	7,866383	7,772733	7,455506	7,843534	8,163341	7,9782007	7,820793667	-0,157407	0,896635173	---
17479188	5,054031	4,950049	4,7347	5,24714	4,526951	4,492568	4,9129267	4,755553	-0,15737367	0,89665589	LOC100861851

17370511	7,7166	7,739029	7,560976	7,385269	7,663608	7,495659	7,6722017	7,514845333	-0,15735633	0,896666663	Arpc5l
17380441	7,279374	7,280278	7,045174	7,033416	6,753181	7,34635	7,2016087	7,044315667	-0,157293	0,896706027	---
17512938	8,766901	9,151662	9,242905	8,939916	8,931866	8,817823	9,0538227	8,896535	-0,15728767	0,896709342	Snord111
17463806	6,412601	6,220758	6,506654	6,333864	6,163651	6,170684	6,3800043	6,222733	-0,15727133	0,896719494	Gm8994
17355009	10,17318	10,30831	10,3468	10,04282	10,2279	10,08577	10,276097	10,11883	-0,15726667	0,896722395	Nars
17299916	6,479305	5,772868	5,966714	5,932918	5,91182	5,902364	6,0729623	5,915700667	-0,15726167	0,896725503	---
17316802	3,880409	3,86738	3,771286	3,690685	3,648056	3,708608	3,8396917	3,682449667	-0,157242	0,896737727	---
17321663	3,987906	3,649476	4,159608	3,860679	3,753487	3,711147	3,93233	3,775104333	-0,15722567	0,896747879	Gm9763
17546790	7,891466	8,098319	8,443531	8,28124	7,852745	7,827778	8,1444387	7,987254333	-0,15718433	0,896773571	---
17300650	7,594583	7,95448	7,913063	7,630091	7,721029	7,639475	7,8207087	7,663531667	-0,157177	0,89677813	2610027L16Rik
17238689	4,015686	4,02524	3,561666	4,113739	3,774991	3,242365	3,8675307	3,710365	-0,15716567	0,896785175	Olfr808
17240727	3,060319	3,488163	2,939525	2,973156	2,931065	3,112321	3,162669	3,005514	-0,157155	0,896791805	---
17345057	3,877194	4,127178	3,838586	3,780097	3,733764	3,857706	3,9476527	3,790522333	-0,15713033	0,896807138	E130008D07Rik
17476728	9,412036	9,527976	9,648807	9,271072	9,474098	9,372307	9,5296063	9,372492333	-0,157114	0,896817291	Cebpa
17444585	3,865834	3,892241	3,703569	3,801812	3,584777	3,603716	3,820548	3,663435	-0,157113	0,896817913	1700018F24Rik
17279102	6,977623	7,068594	7,041927	6,795503	6,925695	6,89567	7,0293813	6,872289333	-0,157092	0,896830967	Cdc42bpb
17547947	10,10904	10,33196	10,15303	10,05929	10,05454	10,00893	10,19801	10,04092	-0,15709	0,896832211	1500003O03Rik // 1500003O03Rik
17363488	8,187866	8,317357	8,263906	8,064783	8,179584	8,053635	8,2563763	8,099334	-0,15704233	0,896861842	1110059E24Rik
17524348	3,460149	3,058971	3,024402	3,112085	3,079613	2,880739	3,181174	3,024145667	-0,15702833	0,896870546	Olfr862
17445543	11,33279	11,05518	10,95505	10,83802	11,06492	10,96908	11,11434	10,95734	-0,157	0,89688816	Crot
17463434	6,73963	6,830752	6,953378	6,740077	6,753346	6,559702	6,8412533	6,684375	-0,15687833	0,8969638	Gm10069
17341704	9,027996	9,089499	8,382212	8,893024	7,978943	9,157167	8,8332357	8,676378	-0,15685767	0,896976649	---
17272780	5,410877	5,696829	5,867746	5,486588	5,452712	5,565654	5,658484	5,501651333	-0,15683267	0,896992193	BC100451
17205227	4,486202	4,385407	4,56906	4,048557	4,090555	4,831068	4,480223	4,323393333	-0,15682967	0,896994058	---
17209417	4,486202	4,385407	4,56906	4,048557	4,090555	4,831068	4,480223	4,323393333	-0,15682967	0,896994058	---
17323465	8,818541	8,94552	8,415326	8,332961	8,600167	8,775813	8,7264623	8,569647	-0,15681533	0,89700297	Snap29
17407706	5,852075	6,168194	6,662994	6,178658	6,465281	5,56899	6,2277543	6,070976333	-0,156778	0,897026182	---
17234711	6,596922	6,5392	6,476034	6,390312	6,4168	6,334812	6,5373853	6,380641333	-0,156744	0,897047323	Gm10142
17361996	7,235544	7,189918	6,905629	7,005465	7,205572	6,649967	7,1103637	6,953668	-0,15669567	0,897077376	Ppp2r5b
17237564	6,274617	6,405741	6,434996	6,336854	6,268034	6,040411	6,3717847	6,215099667	-0,156685	0,897084009	Mdm1
17316977	4,605639	4,551086	4,692618	4,691941	4,210598	4,476751	4,6164477	4,459763333	-0,15668433	0,897084423	Mir1907
17544196	4,982557	4,770263	4,842607	4,765393	4,641033	4,719071	4,8651423	4,708499	-0,15664333	0,897109918	Hdx
17362399	4,514174	4,580198	4,584455	4,393752	4,383013	4,432297	4,559609	4,403020667	-0,15658833	0,897144119	Slc22a28
17387736	2,991702	2,862619	3,097378	2,894671	2,864098	2,723228	2,9838997	2,827332333	-0,15656733	0,897157178	Olfr1107
17232722	4,099769	4,195184	4,521742	4,087124	3,665691	4,594253	4,2722317	4,115689333	-0,15654233	0,897172725	---
17433231	4,999775	5,395557	5,769327	5,756422	5,36473	4,573882	5,3882197	5,231678	-0,15654167	0,89717314	---
17271905	5,984561	5,61697	6,188512	5,682387	5,878487	5,759706	5,9300143	5,773526667	-0,15648767	0,897206721	4933422H20Rik // 4933422H20Rik
17302331	7,592531	7,641606	7,804094	7,413105	7,448996	7,706692	7,6794103	7,522931	-0,15647933	0,897211904	Gm5088
17448050	6,636503	6,403821	6,507348	6,465658	6,324118	6,288473	6,5158907	6,359416333	-0,15647433	0,897215013	Ccdc149
17204731	4,965052	4,71735	5,175298	5,391599	4,196024	4,800708	4,9525667	4,796126333	-0,15644033	0,897236158	---
17255258	6,446068	6,435954	6,473236	6,363872	6,375134	6,146943	6,4517527	6,295316333	-0,15643633	0,897238646	---
17450942	3,962718	3,971126	3,837287	3,794608	3,761317	3,745921	3,9237103	3,767282	-0,15642833	0,897243621	Gm10416
17431032	3,901471	3,989371	4,191411	3,95304	3,920585	3,739357	4,0274177	3,870994	-0,15642367	0,897246524	1810019J16Rik
17525706	3,380501	3,129589	3,190819	3,016211	3,071932	3,143505	3,2336363	3,077216	-0,15642033	0,897248597	Olfr146
17463387	6,327255	5,981498	6,371326	6,231153	5,907571	6,072156	6,226693	6,070293333	-0,15639967	0,89726145	Tspan11
17229865	4,709242	4,476901	5,024583	4,529053	4,743026	4,619147	4,7369087	4,580557333	-0,15635133	0,897291511	Atp1a4
17509118	4,068871	3,99159	3,734881	3,949244	3,750333	3,626775	3,9317807	3,775450667	-0,15633	0,897304779	D330022K07Rik
17394980	4,856183	4,660779	4,715635	4,527512	4,659091	4,577126	4,744199	4,587909667	-0,15628933	0,897330073	Gm16796
17209823	5,978632	6,403636	7,132668	6,647799	6,069395	6,328882	6,5049787	6,348692	-0,15628667	0,897331731	---
17266466	7,063743	7,165652	6,89373	6,817984	6,935842	6,900486	7,0410417	6,884770667	-0,156271	0,897341476	Tmem199
17330437	4,670978	4,731528	4,835856	4,832341	4,502401	4,434871	4,7461207	4,589871	-0,15624967	0,897354745	4930435E12Rik
17355953	7,521399	7,328242	7,849148	7,637137	7,281714	7,3112	7,566263	7,410017	-0,156246	0,897357026	---
17479415	4,137809	4,171049	4,611982	4,228603	4,089511	4,134009	4,3069467	4,150707667	-0,156239	0,89736138	AI854517
17407382	3,439405	3,417786	3,417409	3,209402	3,169758	3,426807	3,4248667	3,268655667	-0,156211	0,897378796	Prr9
17239589	5,364727	4,794019	5,015192	4,922221	4,839506	4,94362	5,0579793	4,901782333	-0,156197	0,897387504	4930444F02Rik
17401586	6,034202	6,07746	6,347501	5,811078	6,126889	6,052639	6,1530543	5,996868667	-0,15618567	0,897394554	---
17366623	5,892324	5,437087	5,991141	5,722284	5,465205	5,664524	5,7735173	5,617337667	-0,15617967	0,897398286	---
17333465	6,168483	6,290397	6,357028	5,753208	6,369867	6,224295	6,2719693	6,11579	-0,15617933	0,897398493	Smoc2
17447032	5,422275	5,205897	5,383235	5,229313	5,096681	5,216959	5,3371357	5,180984333	-0,15615133	0,89741591	---
17371146	6,592893	6,663139	6,585489	6,296295	6,627365	6,45308	6,6138403	6,457713333	-0,156127	0,897431047	---
17284334	4,169835	3,935018	4,107833	3,664582	4,162948	3,916781	4,0708953	3,914770333	-0,156125	0,897432291	Ighg
17256862	3,874884	3,823733	3,75322	3,567678	3,694432	3,721355	3,817279	3,661155	-0,156124	0,897432913	1700006E09Rik
17550733	4,425193	4,315589	4,431483	3,933789	4,516874	4,253233	4,390755	4,234632	-0,156123	0,897433535	---
17496589	8,765095	8,729889	8,788424	8,499621	8,643581	8,671865	8,761136	8,605022333	-0,15611367	0,897439341	Qprt
17538801	9,552071	9,591407	9,639078	9,29773	9,772076	9,244415	9,5941853	9,438073667	-0,15611167	0,897440585	---
17391447	8,420753	8,881181	8,653521	8,239305	8,655907	8,591915	8,6518183	8,495709	-0,15610933	0,897442036	---
17517222	4,801714	4,686448	4,652964	4,523148	4,66811	4,481637	4,7137087	4,557631667	-0,156077	0,89746215	Arhgap20
17234264	6,973187	6,874678	6,686843	6,528832	6,756901	6,780813	6,8449027	6,688848667	-0,156054	0,897476458	Gnaz
17201149	9,094146	9,410866	9,237707	8,910544	9,204887	9,159201	9,247573	9,091544	-0,156029	0,89749201	---
17208071	4,865878	5,278041	4,351156	4,808017	4,415371	4,803636	4,8316917	4,675674667	-0,156017	0,897499475	---
17391458	6,663275	8,688326	7,561136	7,505323	6,696112	8,243278	7,637579	7,481571	-0,156008	0,897505074	---
17253505	7,693849	7,678128	7,842163	7,622205	7,638413	7,485608	7,7380467	7,582075333	-0,15597133	0,897527885	Sdf2
17497366	6,030787	5,866846	6,228396	5,98627	5,922906	5,748939	6,0420097	5,886038333	-0,15597133	0,897527885	Ebf3
17344114	9,281072	9,597783	9,473173	9,382479	9,243394	9,258368	9,450676	9,294747	-0,155929	0,897554221	1110038B12Rik
17506065	3,405914	3,218485	2,866516	2,787801	3,043588	3,19178	3,1636383	3,007723	-0,15591533	0,897562724	---
17482918	4,827479	4,167233	4,47678	4,610036	4,128039	4,265673	4,4904973	4,334582667	-0,15591467	0,897563139	---
17544282	3,447255	3,697215	3,655117	3,358142	3,528242	3,445581	3,5998623	3,443988333	-0,155874	0,89758844	---
17474941	8,486695	8,729176	8,755188	8,54971	8,327726	8,626144	8,6570197	8,501193333	-0,15582633	0,897618096	Kcnn4
17419036	11,20983	11,36216	11,40673	11,13975	11,10027	11,27123	11,32624	11,17041667	-0,15582333	0,897619963	---
17281280	7,232292	7,156511	7,354065	7,155979	7,343603	6,775972	7,2476227	7,091851333	-0,15577133	0,897652317	Mbip
17260666	4,766919	4,49148	4,33407	4,371299	4,323443	4,430415	4,530823	4,375052333	-0,15577067	0,897652732	---
17548398	6,404853	6,467738	6,10461	5,967154	6,241148	6,301882	6,3257337	6,170061333	-0,15567233	0,897713917	LOC100861912
17294066	7,317086	7,706576	7,311837	7,404642	7,27497	7,188923	7,4451663	7,289511667	-0,15565467	0,897724911	Mtrr
17541846	8,511485	8,425182	8,204911	8,234696	8,1668	8,273198	8,380526	8,224898	-0,155628	0,897741504	Mmgt1
17220833	9,540633	9,70917	9,558768	9,407881	9,500201	9,433649	9,602857	9,447243667	-0,15561333	0,897750631	Lpgat1
17302138	9,278704	9,581877	9,107632	9,018904	9,189304	9,221583	9,2988743	9,143263667	-0,15561067	0,89775229	D5Ertd579e // D5Ertd579e
17409480	4,458751	4,277859	4,680961	4,304214	4,3322	4,314334	4,4725237	4,316916	-0,15560767	0,897754157	Ntng1
17291617	6,110884	5,65439	5,797018	5,602902	5,742352	5,750242	5,8540973	5,698498667	-0,15559867	0,897759757	---
17207523	7,022227	7,038009	6,865094	6,77547	6,883672	6,79944	6,97511	6,819527333	-0,15558267	0,897769714	---
17280184	7,784962	8,244056	7,537354	7,66122	7,520312	7,918124	7,8554573	7,699885333	-0,155572	0,897776352	Zfp125
17356425	8,576632	8,54604	8,584938	8,540798	8,214319	8,485909	8,5692033	8,413675333	-0,155528	0,897803733	---
17378905	5,389142	5,460664	5,440545	5,13853	5,098846	5,586435	5,430117	5,274603667	-0,15551333	0,89781286	Arhgap40
17319588	7,841115	7,976869	7,675647	7,826451	7,425565	7,775146	7,8312103	7,675720667	-0,15548967	0,897827589	Tnfrsf13c
17349876	3,720073	3,945585	3,957505	3,779778	3,713014	3,663929	3,8743877	3,718907	-0,15548067	0,897833189	Pcdhb4
17323424	6,534252	6,885244	6,51172	6,359626	6,514408	6,590785	6,6437387	6,488273	-0,15546567	0,897842525	Ube2l3 // Ube2l3
17511643	5,390695	5,560789	5,772053	5,283057	5,36268	5,611495	5,5745123	5,419077333	-0,155435	0,89786161	Ces1a
17206251	5,98867	6,072568	5,921711	6,277216	5,276218	5,963245	5,9943163	5,838893	-0,15542333	0,89786887	---
17268637	7,641821	6,47437	6,688883	6,787889	6,657323	6,893594	6,9350247	6,779602	-0,15542267	0,897869285	---
17208301	5,105666	4,210737	5,083675	3,874636	4,623611	5,435661	4,800026	4,644636	-0,15539	0,897889616	---
17379401	6,223695	6,073136	6,592975	6,034326	6,334783	6,054554	6,296602	6,141221	-0,155381	0,897895217	Rbpjl
17208905	3,449194	4,75738	3,727535	3,73188	4,109045	3,627105	3,9780363	3,822676667	-0,15535967	0,897908495	---
17300067	4,4578	4,459211	5,010191	4,048959	4,440627	4,971541	4,6424007	4,487042333	-0,15535833	0,897909324	---
17205633	5,039548	4,693068	4,230781	3,829101	5,174026	4,494233	4,6544657	4,49912	-0,15534567	0,897917208	---
17535054	3,411793	3,358178	3,541567	3,301334	3,276396	3,267787	3,4371793	3,281839	-0,15534033	0,897920527	Gm364
17203595	5,214797	5,315041	4,692224	4,5463	4,831613	5,378149	5,0740207	4,918687333	-0,15533333	0,897924884	---
17515478	5,203431	5,484457	5,230463	5,253129	5,086278	5,11304	5,306117	5,150815667	-0,15530133	0,897944801	Bmper
17218965	4,164443	4,113379	4,042342	4,050445	3,883535	3,920339	4,1067213	3,951439667	-0,15528167	0,897957042	Adcy10
17390490	6,520445	6,183919	6,538152	6,259485	6,241072	6,276154	6,414172	6,258903667	-0,15526833	0,897965341	Ppip5k1
17364585	3,450258	3,21861	3,417038	3,198017	3,077984	3,344138	3,3619687	3,206713	-0,15525567	0,897973225	C330026H20Rik
17468522	8,809034	8,872998	8,499269	8,324518	8,707434	8,683653	8,7271003	8,571868333	-0,155232	0,897987956	---
17489565	3,531605	3,689588	3,695402	3,54108	3,513845	3,396028	3,638865	3,483651	-0,155214	0,89799916	Abpg
17245850	8,837713	8,951031	9,060458	8,886673	8,867892	8,629196	8,949734	8,794587	-0,155147	0,898040864	Mars
17210421	3,429722	3,521623	3,759326	3,553006	3,222519	3,469725	3,5702237	3,415083333	-0,15514033	0,898045014	---
17399828	4,050624	4,116728	3,943253	3,886462	3,910339	3,848396	4,0368683	3,881732333	-0,155136	0,898047712	Pglyrp4
17457981	4,215565	4,17824	3,997671	4,177551	3,718753	4,029822	4,130492	3,975375333	-0,15511667	0,898059746	Olfr448
17505069	6,771589	6,782133	6,577209	6,501662	6,585103	6,579083	6,7103103	6,555282667	-0,15502767	0,898115149	Slc7a6
17236366	4,654285	4,577715	4,423061	4,390123	4,581232	4,218663	4,551687	4,396672667	-0,15501433	0,89812345	Sycp3
17548816	4,897073	4,861392	4,820851	4,692071	4,620965	4,801305	4,859772	4,704780333	-0,15499167	0,898137561	---
17348705	3,37432	3,444191	3,286793	3,434767	3,097735	3,107872	3,3684347	3,213458	-0,15497667	0,898146899	---
17204321	5,876247	5,628163	5,564324	5,535692	5,617166	5,450979	5,689578	5,534612333	-0,15496567	0,898153747	---
17330870	5,087047	4,883277	5,040439	4,734665	5,020513	4,790705	5,0035877	4,848627667	-0,15496	0,898157275	Gm4827 // Gm4827
17202711	5,978419	5,888474	6,339282	6,161143	5,955919	5,624326	6,068725	5,913796	-0,154929	0,898176574	---
17266368	12,56343	12,73494	12,62663	12,4516	12,53298	12,47567	12,641667	12,48675	-0,15491667	0,898184253	Tlcd1 // Tlcd1
17539966	9,211554	9,335679	9,344544	9,100953	9,113267	9,212881	9,297259	9,142367	-0,154892	0,898199609	Gata1
17528894	3,706075	3,910912	3,514242	3,542083	3,621239	3,503311	3,7104097	3,555544333	-0,15486533	0,898216212	Hmgcll1
17202353	6,15133	4,421061	4,783948	5,097838	5,006662	4,787294	5,1187797	4,963931333	-0,15484833	0,898226796	---
17306937	5,314421	5,576016	5,576383	5,304955	5,179032	5,518368	5,48894	5,334118333	-0,15482167	0,898243399	Cma1
17260177	8,62324	8,681466	8,704936	8,475755	8,464787	8,604641	8,6698807	8,515061	-0,15481967	0,898244644	Mrps24
17499874	6,991858	6,873432	6,913527	6,762983	6,709706	6,841691	6,9262723	6,77146	-0,15481233	0,89824921	Thsd1
17239876	8,96015	8,884928	8,865286	8,621178	8,79709	8,827677	8,9034547	8,748648333	-0,15480633	0,898252946	2010003K15Rik
17371340	4,452994	4,563871	4,351037	4,328315	4,376654	4,198556	4,4559673	4,301175	-0,15479233	0,898261663	4932414N04Rik
17312939	8,511743	8,541183	8,667202	8,467417	8,419445	8,368958	8,573376	8,418606667	-0,15476933	0,898275983	Polr2f // Polr2f
17548723	5,026809	5,206793	5,202256	4,915446	5,018672	5,037454	5,145286	4,990524	-0,154762	0,898280549	Glce // Glce
17547672	3,63672	4,09601	3,662291	3,660239	3,729237	3,541311	3,7983403	3,643595667	-0,15474467	0,898291342	---

17496845	3,794461	3,525895	4,062057	3,822876	3,591912	3,503417	3,7941377	3,639401667	-0,154736	0,898296738	---
17251354	6,161066	6,224669	6,271074	6,184551	5,950565	6,057496	6,2189363	6,064204	-0,15473233	0,898299021	Mfsd6l
17444743	5,668018	5,04928	5,414511	5,203997	5,241201	5,222504	5,3772697	5,222567333	-0,15470233	0,898317701	Gm6408
17414045	9,552134	9,518641	9,417895	9,450627	9,210725	9,363249	9,4962233	9,341533667	-0,15468967	0,898325588	E130309F12Rik
17376649	9,4838	9,543118	9,584399	9,349536	9,51816	9,279577	9,5371057	9,382424333	-0,15468133	0,898330777	Crls1
17311191	6,508264	6,291285	6,249447	6,145143	6,329451	6,110545	6,3496653	6,195046333	-0,154619	0,898369591	Cthrc1
17443825	6,215466	6,178109	6,262004	6,051864	6,171788	5,968112	6,2185263	6,063921333	-0,154605	0,898378309	Gal3st4 // Gal3st4
17415434	4,054087	4,113343	4,017498	3,843132	3,815096	4,062996	4,0616427	3,907074667	-0,154568	0,89840135	5830433M19Rik
17282272	4,51256	4,175037	3,949419	4,198973	4,042962	3,931395	4,2123387	4,057776667	-0,154562	0,898405086	---
17471062	7,452234	7,654078	7,244364	7,450049	7,138876	7,298065	7,4502253	7,295663333	-0,154562	0,898405086	Tapbpl
17273253	5,747128	5,430837	5,792066	5,460699	5,658981	5,386775	5,656677	5,502151667	-0,15452533	0,89842792	Myadml2
17433164	5,225122	5,137127	5,248913	5,238449	4,907535	5,001629	5,2037207	5,049204333	-0,15451633	0,898433524	---
17506456	3,415058	3,986763	3,412607	3,40579	3,665408	3,279713	3,6048093	3,450303667	-0,15450567	0,898440167	---
17484100	9,603048	9,438145	9,636966	9,513935	9,352827	9,348039	9,5593863	9,404933667	-0,15445267	0,898473173	---
17459185	6,634001	6,298573	6,611346	6,504422	6,253732	6,322472	6,51464	6,360208667	-0,15443133	0,898486459	C130060K24Rik
17326844	8,249792	8,401264	8,185316	8,404281	7,898273	8,070581	8,2787907	8,124378333	-0,15441233	0,898498292	---
17275785	7,942792	8,149546	7,88138	7,796606	7,828467	7,885449	7,9912393	7,836840667	-0,15439867	0,898506804	Fam179b
17311173	4,956215	4,783535	4,969457	4,614621	4,816398	4,815247	4,903069	4,748755333	-0,15431367	0,898559743	Baalc
17368799	3,565584	3,17532	3,213554	3,323572	3,133449	3,034535	3,3181527	3,163852	-0,15430067	0,89856784	---
17514316	3,14207	3,029683	3,367088	2,997798	3,133075	2,945186	3,1796137	3,025353	-0,15426067	0,898592754	---
17516112	4,90367	4,7765	4,72775	4,878816	4,73328	4,333076	4,80264	4,648390667	-0,15424933	0,898599813	Olfr875
17210153	3,204632	3,069369	3,34235	3,119867	3,160106	2,873634	3,2054503	3,051202333	-0,154248	0,898600643	---
17474858	3,263949	3,338785	2,782639	3,059227	2,934267	2,929145	3,1284577	2,974213	-0,15424467	0,89860272	Vmn1r173
17382423	4,371896	4,594306	4,92314	4,425392	4,439211	4,562034	4,6297807	4,475545667	-0,154235	0,898608741	Tubb4b
17200993	6,807144	7,232158	7,209096	6,960181	6,919617	6,905976	7,0827993	6,928591333	-0,154208	0,898625558	---
17466237	4,891651	4,627275	4,69646	4,704568	4,533528	4,514772	4,738462	4,584289333	-0,15417267	0,898647567	Tas2r138
17201805	5,706178	5,821115	5,858527	5,748187	5,691535	5,48362	5,7952733	5,641114	-0,15415933	0,898655872	---
17201981	5,706178	5,821115	5,858527	5,748187	5,691535	5,48362	5,7952733	5,641114	-0,15415933	0,898655872	---
17414351	5,279136	5,198069	5,29712	5,03764	5,068625	5,205667	5,2581083	5,103977333	-0,154131	0,898673521	Palm2
17484484	8,351129	8,266231	8,072115	7,800563	8,118799	8,307812	8,229825	8,075724667	-0,15410033	0,898692624	Paox
17281526	3,679622	3,771144	4,275822	4,016505	3,453525	3,794382	3,9088627	3,754804	-0,15405867	0,89871858	---
17548938	6,282724	5,747815	5,877792	5,593652	5,781207	6,07131	5,9694437	5,815389667	-0,154054	0,898721487	3110040M04Rik
17550825	3,196408	3,299162	3,220748	3,102867	3,075121	3,076242	3,2387727	3,084743333	-0,15402933	0,898736853	---
17320879	5,019691	4,287948	4,495707	4,598423	4,262454	4,480425	4,6011153	4,447100667	-0,15401467	0,89874599	4833422M21Rik
17362765	5,903133	5,741941	5,892049	5,604864	5,683187	5,787036	5,8457077	5,691695667	-0,154012	0,898747651	Cd6
17359160	6,54733	6,688135	6,335358	6,223482	6,403444	6,481884	6,5236077	6,369603333	-0,15400433	0,898752427	Plce1
17421797	5,499116	5,09105	5,337348	5,144058	5,023475	5,298022	5,3091713	5,155185	-0,15398633	0,898763641	LOC100861760
17308388	4,964115	4,720504	5,068202	4,822088	4,709134	4,759781	4,917607	4,763667667	-0,15393933	0,898792921	Sftpc
17542670	6,193378	6,4755	6,310248	6,09298	6,311605	6,112765	6,3263753	6,17245	-0,15392533	0,898801643	Slc10a3
17202205	5,176761	4,301509	4,424457	4,718443	4,179709	4,542808	4,6342423	4,48032	-0,15392233	0,898803512	---
17316725	8,245695	8,318512	8,233906	8,199496	8,214537	7,922349	8,2660377	8,112127333	-0,15391033	0,898810988	Slc25a32
17484892	4,782641	4,694329	4,740786	4,868657	4,268399	4,619	4,739252	4,585352	-0,1539	0,898817426	---
17350009	4,386457	4,644154	4,78596	4,469692	3,86587	5,01942	4,6055237	4,451660667	-0,153863	0,898840477	---
17427761	3,941816	3,536367	3,66058	3,652099	3,468993	3,556089	3,712921	3,559060333	-0,15386067	0,898841931	---
17399604	5,042345	4,869315	5,237097	4,854813	4,873	4,959386	5,0495857	4,895733	-0,15385267	0,898846915	Nup210l
17387780	3,326825	3,562198	3,311607	3,131464	3,411242	3,196368	3,40021	3,246358	-0,153852	0,898847331	Olfr1154
17545712	4,871695	4,701525	4,560802	4,618728	4,556765	4,497052	4,7113407	4,557515	-0,15382567	0,898863738	Cnksr2
17520425	8,447906	8,52759	8,763868	8,472835	8,405925	8,399134	8,579788	8,425964667	-0,15382333	0,898865191	Pcolce2
17526008	5,578393	5,612789	5,758224	5,387781	5,679782	5,420382	5,649802	5,495981667	-0,15382033	0,89886706	Grik4
17256502	7,979375	8,398729	8,290347	8,065411	8,21961	7,92197	8,222817	8,068997	-0,15382	0,898867268	Naglu
17477426	5,922206	6,248388	6,487864	6,247162	6,206326	5,743575	6,219486	6,065687667	-0,15379833	0,898880768	Snord88a
17244524	7,77927	7,937409	7,766047	7,600687	7,572373	7,84828	7,8275753	7,67378	-0,15379533	0,898882637	---
17548961	4,150038	3,918852	4,007277	4,029541	3,838388	3,746945	4,025389	3,871624667	-0,15376433	0,898901952	Gm19335
17200041	5,657225	5,286619	5,633823	5,33992	5,968335	4,808138	5,525889	5,372131	-0,153758	0,898905898	---
17442498	4,490731	4,551867	4,706637	4,19494	4,455821	4,637304	4,5830783	4,429355	-0,15372333	0,898927498	---
17529897	4,214552	4,384614	4,789356	4,14672	4,372449	4,408189	4,4628407	4,309119333	-0,15372133	0,898928744	Gm19325
17211235	4,978883	4,966969	5,085214	5,014767	4,704602	4,850654	5,0103553	4,856674333	-0,153681	0,898953876	Kcnb2
17209719	6,396675	6,686733	6,421391	5,552206	6,601082	6,890472	6,5015997	6,34792	-0,15367967	0,898954707	---
17509687	3,753741	3,813905	4,799316	3,882174	4,15939	3,864359	4,1223207	3,968641	-0,15367967	0,898954707	---
17200337	3,677797	3,2932	3,666074	2,864171	3,733525	3,578387	3,5456903	3,392027667	-0,15366267	0,8989653	---
17368741	5,989484	5,782564	5,882346	5,844146	5,625835	5,723449	5,884798	5,731143333	-0,15365467	0,898970285	Ak8
17441768	4,192233	3,853302	4,086546	3,823894	4,042415	3,804981	4,044027	3,89043	-0,153597	0,899006218	Oas1f
17485713	7,928782	7,86565	7,817385	7,753367	7,633031	7,764732	7,8706057	7,717043333	-0,15356233	0,899027821	Gp6
17273107	5,237205	4,884475	5,122803	4,870667	4,88017	5,033066	5,0814943	4,927967667	-0,15352667	0,899050047	Pde6g
17204771	6,621558	6,549349	6,78217	6,487493	6,359501	6,645532	6,6510257	6,497508667	-0,153517	0,899056071	---
17213645	7,487638	7,608777	7,405285	7,297182	7,416589	7,327502	7,5005667	7,347091	-0,15347567	0,89908183	Fastkd2
17285739	5,040145	4,979871	5,068965	5,043721	4,881433	4,70346	5,0296603	4,876204667	-0,15345567	0,899094294	Gm11292
17484832	5,589311	5,567691	5,735993	5,584293	5,455812	5,392593	5,6309983	5,477566	-0,15343233	0,899108835	Drd4
17249769	3,699233	3,659838	4,070746	3,676839	3,46915	3,823534	3,809939	3,656507667	-0,15343133	0,899109458	---
17209913	5,940868	5,911306	5,809731	6,017378	5,787802	5,396489	5,8873017	5,733889667	-0,153412	0,899121507	---
17463797	3,619716	3,493089	3,246843	3,261196	3,360537	3,277867	3,453216	3,299866667	-0,15334933	0,899160564	---
17500826	2,922965	2,921034	2,936082	2,712023	2,799995	2,808016	2,9266937	2,773344667	-0,153349	0,899160771	---
17545073	3,024036	3,268354	2,740571	2,828286	2,891199	2,853438	3,010987	2,857641	-0,153346	0,899162641	---
17379986	6,015656	5,894837	5,959128	5,697732	5,890996	5,820867	5,9565403	5,803198333	-0,153342	0,899165134	Mocs3
17548215	6,458731	6,642087	6,084891	6,164554	6,164554	6,396594	6,3952363	6,241900667	-0,15333567	0,899169081	LOC552890
17387748	3,744872	3,390394	3,59271	3,412521	3,307409	3,54835	3,575992	3,42276	-0,153232	0,899233695	Olfr1111
17206369	3,625127	3,217202	3,442009	3,390441	3,304867	3,129382	3,4281127	3,274896667	-0,153216	0,899243667	---
17221110	4,816753	4,992904	4,665835	4,679608	4,632241	4,704105	4,825164	4,671984667	-0,15317933	0,899266522	Npbwr1
17225683	4,759213	4,900388	4,586565	4,427648	4,756307	4,60269	4,748722	4,595548333	-0,15317367	0,899270055	2310007B03Rik
17454209	6,386391	6,36104	6,419271	6,440077	6,114707	6,152437	6,3889007	6,235740333	-0,15316033	0,899278366	Gjc3
17269404	4,123802	4,362621	4,260856	4,101979	4,027298	4,158537	4,249093	4,095938	-0,153155	0,89928169	Krt16
17203629	3,527873	3,782469	3,204429	2,752846	3,306908	3,995615	3,5049237	3,351789667	-0,153134	0,89929478	---
17523650	6,867834	6,622867	6,567583	6,351487	6,768909	6,478697	6,6860947	6,533031	-0,15306367	0,899338623	Ccr2
17241612	3,356699	3,781765	3,979359	3,438976	3,644837	3,574864	3,705941	3,552892333	-0,15304867	0,899347974	---
17329043	3,013167	2,922541	3,14261	2,869853	2,851015	2,898369	3,026106	2,873079	-0,153027	0,89936148	Olfr171
17457712	4,359438	4,533554	4,597206	4,33631	4,275122	4,419727	4,4967327	4,343719667	-0,153013	0,899370208	---
17356604	7,492899	7,310689	7,544911	7,387554	7,451631	7,050444	7,4494997	7,296543	-0,15295667	0,899405327	Fau
17516170	4,09212	3,688786	3,504129	3,9303	3,529036	3,366975	3,7616783	3,608770333	-0,152908	0,899435667	Olfr904
17410251	8,90736	8,951037	8,844513	8,645681	8,768924	8,82977	8,90097	8,748125	-0,152845	0,899474945	Enpep
17202207	6,530444	6,453108	6,389441	7,035321	5,83643	6,042709	6,4576643	6,30482	-0,15284433	0,89947536	---
17283540	8,622143	9,112243	8,54665	8,527485	8,357641	8,93738	8,7603453	8,607502	-0,15284333	0,899475984	---
17549278	3,561395	3,560831	3,465402	3,356498	3,407322	3,365303	3,5292093	3,376374333	-0,152835	0,899481179	---
17210375	5,467903	4,855319	4,911058	5,031109	5,078043	4,666633	5,0780933	4,925261667	-0,15283167	0,899483258	---
17299135	5,424617	5,462878	5,295518	5,383111	5,297496	5,043944	5,3943377	5,241517	-0,15282067	0,899490116	---
17214729	6,396536	5,972607	5,715541	5,994172	6,000898	5,631292	6,028228	5,875454	-0,152774	0,899519212	LOC100862198
17318772	8,290039	7,938042	7,291262	7,212394	7,54531	8,303456	7,839781	7,687053333	-0,15272767	0,899548101	Apol7a
17416566	9,380318	9,192064	9,001268	8,787857	9,101642	9,226094	9,1912167	9,038531	-0,15268567	0,899574289	Magoh
17210079	2,82288	3,090494	3,466262	3,03596	2,980541	2,905094	3,1265453	2,973865	-0,15268033	0,899577615	---
17246466	4,175888	3,697427	3,67837	3,837152	3,523969	3,732647	3,8505617	3,697922667	-0,152639	0,899603388	Olfr827
17498722	5,302215	4,921162	5,398714	5,201667	5,010704	4,951847	5,2073637	5,054739333	-0,15262433	0,899612534	Retn // Retn
17266221	3,149643	3,42008	3,205111	3,119498	3,118599	3,078886	3,258278	3,105661	-0,152617	0,899617107	---
17548042	9,590223	9,587545	9,526144	9,400965	9,518499	9,326608	9,5679707	9,415357333	-0,15261333	0,899619393	Galnt1
17530780	3,101051	2,773582	2,934838	2,773582	2,813214	2,764845	2,9364903	2,783880333	-0,15261	0,899621472	---
17419792	6,441083	6,325127	6,587265	6,206059	6,28944	6,400161	6,4511583	6,298553333	-0,152605	0,899624589	2610002D18Rik
17204015	3,320103	3,397911	3,434346	3,21283	3,016762	3,464957	3,38412	3,231516333	-0,15260367	0,899625421	---
17400875	6,63152	6,417602	6,746256	6,385075	6,657601	6,294993	6,5984593	6,445889667	-0,15256967	0,899646623	Zfp697
17457318	3,484285	3,492991	3,310359	3,189692	3,322432	3,317843	3,4292117	3,276655667	-0,152556	0,899655145	Mir490
17486065	3,306469	3,249501	3,065792	3,155986	3,101723	2,906405	3,207254	3,054704667	-0,15254933	0,899659302	Vmn1r64
17205641	5,019575	5,053583	4,686536	4,610855	4,587624	5,103667	4,919898	4,767382	-0,152516	0,899680089	---
17259522	8,850776	9,015012	8,829791	8,733654	8,807961	8,696464	8,8985263	8,746026333	-0,1525	0,899690067	---
17448172	2,963681	3,219425	3,27501	2,997979	3,18538	2,817531	3,1527053	3,000296667	-0,15240867	0,899747026	---
17326534	3,927455	3,822694	3,841727	3,684819	3,770834	3,679004	3,8639587	3,711552333	-0,15240633	0,899748481	Pou1f1
17546935	3,86897	4,495499	4,132232	3,88398	4,595615	3,559949	4,165567	4,013181333	-0,15238567	0,89976137	Rbmy1a1
17546944	3,86897	4,495499	4,132232	3,88398	4,595615	3,559949	4,165567	4,013181333	-0,15238567	0,89976137	Rbmy1a1
17529095	4,220579	4,403118	4,406483	4,572425	4,119208	3,881394	4,3433933	4,191009	-0,15238433	0,899762202	Gm10635
17334126	5,290918	4,790364	4,828433	4,896334	4,617029	4,939292	4,969905	4,817551667	-0,15235333	0,899781536	D930048G16Rik
17321326	7,836805	7,886987	7,724904	7,710257	7,688593	7,592824	7,816232	7,663891333	-0,15234067	0,899789436	Arf3
17531001	7,235559	7,182952	7,181517	7,07348	7,032128	7,037435	7,2000093	7,047681	-0,15232833	0,899797128	Sema3f
17281075	3,332639	3,336818	3,27559	3,172118	3,157032	3,158984	3,3150157	3,162711333	-0,15230433	0,899812096	1700031P21Rik // 1700031P21Rik
17203319	6,352521	5,996326	6,919543	6,462026	6,021452	6,328172	6,4227967	6,27055	-0,15224667	0,899848064	---
17200935	5,536693	4,809713	5,985396	5,996557	5,244965	4,63358	5,443934	5,291700667	-0,15223333	0,89985638	---
17333198	4,708867	4,394999	4,519155	4,597324	4,455951	4,113126	4,541007	4,388800333	-0,15220667	0,899873013	---
17358613	4,698618	4,830711	4,547273	4,401013	4,492944	4,726334	4,6922007	4,540097	-0,15210367	0,899937261	Trpd52l3
17201659	6,561133	6,299448	6,834617	6,518651	6,884545	5,835692	6,565066	6,412962667	-0,15210333	0,899937469	---
17257085	8,554415	8,646509	8,28709	8,135114	8,353938	8,542654	8,4960047	8,343902	-0,15210267	0,899937885	Hexim1
17484102	10,11133	9,970226	9,987846	10,14684	9,366283	10,10024	10,023134	9,871121	-0,152013	0,89999382	---
17356209	5,915102	6,012615	6,001057	6,118194	5,602344	5,752261	5,976258	5,824266333	-0,15199167	0,900007128	A930001C03Rik
17204081	4,353987	5,104341	5,04216	4,265544	5,142614	4,63641	4,833496	4,681522667	-0,15197333	0,900018566	---
17532151	5,795399	5,592841	6,061586	5,666421	5,620266	5,707224	5,8166087	5,664637	-0,15197167	0,900019605	Scn5a
17213315	7,224463	7,660964	7,44798	7,365134	7,146687	7,365771	7,444469	7,292530667	-0,15193833	0,9000404	---
17387730	4,562901	4,031389	3,675521	3,823015	4,137876	3,85312	4,089937	3,938003667	-0,15193333	0,90004352	Olfr1104
17503222	4,492072	4,451768	4,504144	4,378036	4,254859	4,359322	4,4826613	4,330739	-0,15192233	0,900050382	---
17375767	7,793474	7,854058	7,891104	7,786749	7,698838	7,597301	7,846212	7,694296	-0,151916	0,900054333	Blvra // Blvra
17246091	4,624805	4,592535	5,061452	4,847501	4,492428	4,483207	4,7595973	4,607712	-0,15188533	0,900073466	Il23a

17360299	3,113183	3,031733	2,927277	2,638022	2,928858	3,049714	3,0240643	2,872198	-0,15186633	0,900085319	---
17455664	5,442781	4,806972	4,893807	5,082889	4,48949	5,115592	5,0478533	4,895990333	-0,151863	0,900087399	LOC100862180 // LOC100862166 // Gm7
17237356	5,704705	5,638243	5,699032	5,64246	5,395719	5,548362	5,68066	5,528847	-0,151813	0,900118594	---
17277666	5,926863	5,807486	6,476472	5,840005	5,988374	5,92702	6,0702737	5,918466333	-0,15180733	0,90012213	Gm1995
17259172	4,266586	4,151861	4,567648	4,034728	4,134096	4,361959	4,3286983	4,176927667	-0,15177067	0,900145007	Gm11762
17359929	9,792826	9,590316	9,513112	9,487214	9,492564	9,461184	9,6320847	9,480320667	-0,151764	0,900149167	---
17217364	3,460708	3,172808	3,13326	3,014158	3,395037	2,902315	3,255592	3,103836667	-0,15175533	0,900154574	---
17208869	4,764757	4,513019	4,667135	4,719266	4,367205	4,403378	4,6483037	4,496616333	-0,15168733	0,900197003	---
17265625	7,681431	7,61419	7,513413	7,290636	7,583985	7,479358	7,6030113	7,451326333	-0,151685	0,900198459	Med31
17430393	5,646854	5,933778	5,947699	5,568806	5,778449	5,726091	5,842777	5,691115333	-0,15166167	0,900213018	---
17468281	3,601091	3,742467	3,970945	3,459163	3,585085	3,81528	3,771501	3,619842667	-0,15165833	0,900215098	Gm10445
17383464	5,070417	5,035453	4,734085	4,956008	4,62297	4,806246	4,9466517	4,795074667	-0,151577	0,90026585	---
17464184	4,437604	4,212954	4,303971	4,105899	4,157339	4,236597	4,3181763	4,166611667	-0,15156467	0,900273546	---
17427784	5,242727	5,405687	5,41427	5,043005	5,263332	5,301658	5,354228	5,202665	-0,151563	0,900274586	Bsnd
17503903	6,077319	6,058013	6,064756	5,798213	5,97611	5,971084	6,066696	5,915135667	-0,15156033	0,900276251	Amfr // Amfr
17204971	4,255931	4,163518	5,23265	4,615547	4,05874	4,523135	4,5506997	4,399140667	-0,151559	0,900277083	---
17495297	10,01008	9,971815	9,82526	9,800911	9,810923	9,740663	9,9357183	9,784165667	-0,15155267	0,900281035	Psma1
17406525	2,96346	3,053003	3,078013	2,846406	2,887075	2,906405	3,031492	2,879962	-0,15153	0,900295179	---
17449521	8,605477	8,854083	8,31673	8,780129	7,841107	8,700538	8,5920967	8,440591333	-0,15150533	0,900310572	---
17405407	5,090969	4,749307	4,682677	4,752841	4,738466	4,577158	4,8409843	4,689488333	-0,151496	0,900316397	1700007F19Rik
17475916	6,336802	5,825107	5,994966	5,971614	5,972651	5,758152	6,0522917	5,900805667	-0,151486	0,900322637	Gm6537
17261335	4,82762	4,657671	4,68744	4,755018	4,222285	4,741056	4,7242437	4,572786333	-0,15145733	0,900340527	4931440F15Rik
17246426	3,48301	3,621983	3,524137	3,221299	3,357233	3,596371	3,5430433	3,391634333	-0,151409	0,900370691	Olfr803
17492062	9,780778	10,54943	10,20109	9,986315	9,810874	10,27996	10,177099	10,02571633	-0,151383	0,900386918	---
17531705	7,384515	7,516271	7,724349	7,143909	7,509827	7,517318	7,5417117	7,390351333	-0,15136033	0,900401064	Ccrl2
17310912	8,492229	8,305448	8,377498	8,560107	8,147332	8,013676	8,391725	8,240371667	-0,15135333	0,900405433	Laptm4b
17237247	6,415986	6,063082	6,541358	6,261584	6,350218	5,954672	6,340142	6,188824667	-0,15131733	0,900427901	Bbs10
17541648	5,104257	4,850975	4,919451	4,998838	4,71805	4,703856	4,9582277	4,806914667	-0,151313	0,900430606	Usp26
17335788	4,105861	4,701745	4,301738	4,011192	4,150936	4,493738	4,3697813	4,218501333	-0,15128	0,900451202	---
17208889	6,754519	6,58255	5,977434	6,182839	6,503448	6,174406	6,4381677	6,286897667	-0,15127	0,900457444	---
17430831	8,549245	8,697111	8,687724	8,368137	8,649333	8,46284	8,6446933	8,493436667	-0,15125667	0,900465766	Snora73b // Snora73b
17493133	3,273128	3,144916	3,449221	3,047238	3,27501	3,091279	3,2890883	3,137842333	-0,151246	0,900472423	Olfr305
17396143	7,040275	7,203933	6,975395	7,108752	6,904884	6,752346	7,073201	6,921994	-0,151207	0,900496766	Car13
17297258	4,191566	4,073791	3,915491	3,968009	3,896751	3,862478	4,0602827	3,909079333	-0,15120333	0,900499055	Nek10
17269256	6,191437	6,234546	6,296308	5,95795	5,977731	6,333191	6,2407637	6,089624	-0,15113967	0,900538795	Krtap4-16
17549466	5,488531	5,414534	5,36135	5,451838	5,068768	5,290396	5,4214717	5,270334	-0,15113767	0,900540043	---
17549618	5,488531	5,414534	5,36135	5,451838	5,068768	5,290396	5,4214717	5,270334	-0,15113767	0,900540043	---
17490624	9,255115	9,398348	9,461689	9,11619	9,301077	9,244623	9,3717173	9,22063	-0,15108733	0,900571462	Snord34
17277605	4,44561	4,0419	4,215133	3,987187	4,060402	4,201829	4,2342143	4,083139333	-0,151075	0,900579161	Samd15
17509642	7,572491	7,670091	7,697273	7,722855	7,496488	7,267323	7,6466183	7,495555333	-0,151063	0,900586652	Klhl2
17300169	5,846914	5,771928	5,709186	5,65378	5,633599	5,587462	5,7760093	5,624947	-0,15106233	0,900587068	---
17321817	5,648882	5,073504	5,234513	5,071976	5,193006	5,238744	5,3189663	5,167908667	-0,15105767	0,900589981	A330009N23Rik
17472847	9,679859	9,687685	9,584547	9,360193	9,603173	9,53558	9,650697	9,499648667	-0,15104833	0,900595807	4933424B01Rik
17531327	3,582479	3,758152	3,849873	3,535062	3,611233	3,591161	3,730168	3,579152	-0,151016	0,900615992	Fbxw14
17423709	2,939525	3,085708	3,47021	3,08037	2,917415	3,044734	3,1651477	3,014173	-0,15097467	0,900641795	---
17477897	7,025136	7,020748	7,09833	6,895545	6,913838	6,881927	7,0480713	6,897103333	-0,150968	0,900645957	Bcat2
17290193	4,659668	4,598248	4,410549	4,567081	4,319268	4,32941	4,556155	4,405253	-0,150902	0,90068716	---
17367446	3,145183	3,301362	3,17089	3,226818	3,141637	2,7963	3,2058117	3,054918333	-0,15089333	0,900692571	---
17209177	5,168714	5,387849	4,806439	5,227545	4,756073	4,926809	5,1210007	4,970142333	-0,15085833	0,900714422	---
17545756	3,7855	3,499993	3,15668	3,280591	3,263543	3,445548	3,4807243	3,329894	-0,15083033	0,900731903	---
17548530	4,48279	4,760092	4,647556	4,401115	4,426187	4,610694	4,630146	4,479332	-0,150814	0,900742101	LOC100862384
17317386	3,643261	3,365627	3,735196	3,325194	3,73968	3,226817	3,5813613	3,430563667	-0,15079767	0,900752299	---
17422975	6,004928	5,820798	5,879467	5,818933	5,784824	5,649065	5,901731	5,750940667	-0,15079033	0,900756877	---
17544061	5,626476	5,457386	5,282974	5,175151	5,292389	5,446958	5,455612	5,304832667	-0,15077933	0,900763745	Cysltr1
17522377	4,468119	4,445717	4,605688	4,391385	4,367866	4,307953	4,506508	4,355734667	-0,15077333	0,900767491	Kif9
17508123	4,861126	4,533844	4,73571	4,488971	4,476922	4,71254	4,7102267	4,559477667	-0,150749	0,900782684	---
17526265	7,119728	7,602262	7,535557	7,241989	7,38514	7,178235	7,4191823	7,268454667	-0,15072767	0,900796004	Trappc4
17495610	5,847526	6,023555	6,122519	5,84412	5,950998	5,746304	5,9978667	5,847140667	-0,150726	0,900797045	Gprc5b
17281880	5,80276	5,829973	6,202604	5,730002	5,757539	5,895666	5,9451123	5,794402333	-0,15071	0,900807035	Six1
17302356	6,651297	6,356666	6,524559	6,8275	5,964419	6,28848	6,5108407	6,360133	-0,15070767	0,900808492	---
17367905	7,478406	7,259067	6,918005	7,274992	6,619618	7,308792	7,2184927	7,067800667	-0,150692	0,900818274	---
17238822	4,943341	4,438934	4,867077	4,346232	4,540064	4,911019	4,749784	4,599105	-0,150679	0,900826392	---
17529784	4,547378	4,587968	4,75967	4,402069	4,53013	4,510788	4,631672	4,480995667	-0,15067633	0,900828057	1700065D16Rik
17523597	8,248432	8,128197	7,921165	7,814936	7,895633	8,135204	8,0992647	7,948591	-0,15067367	0,900829722	Limd1
17209839	4,413626	4,846832	4,90502	5,021809	4,364452	4,327245	4,721826	4,571168667	-0,15065733	0,900839921	---
17401556	6,362741	6,465276	6,677691	6,311396	6,199891	6,542524	6,5019027	6,351270333	-0,15063233	0,900855531	Hbxip
17412636	4,305135	4,535607	5,013854	4,458003	4,636744	4,308155	4,6181987	4,467634	-0,15056467	0,900897785	---
17245727	3,191174	3,306783	3,154585	3,207814	3,032367	2,960743	3,217514	3,066974667	-0,15053933	0,900913604	---
17239463	8,318293	8,408777	8,070292	8,08404	8,06531	8,196459	8,2657873	8,115269667	-0,15051767	0,900927135	---
17319545	7,404904	7,508765	7,464231	7,24475	7,347095	7,334536	7,4593	7,308793667	-0,15050633	0,900934212	Polr3h
17200683	4,208705	3,334544	3,842214	3,727419	3,614112	3,592572	3,7951543	3,644701	-0,15045333	0,90096731	---
17454187	4,651821	4,807595	4,682897	4,369746	4,74478	4,57656	4,7141043	4,563695333	-0,150409	0,900994997	Pilrb2
17221186	9,485559	9,735312	9,67212	9,556263	9,575377	9,310132	9,630997	9,480590667	-0,15040633	0,900996662	Snhg6
17514745	9,321961	9,369056	9,069849	9,011813	9,149154	9,148818	9,253622	9,103261667	-0,15036033	0,901025391	Cwc15
17227198	6,10352	6,017766	6,179291	5,926406	5,951183	5,971984	6,1001923	5,949857667	-0,15033467	0,901041421	Lgr6
17481102	10,96379	11,05341	11,15722	10,90671	10,92904	10,88779	11,05814	10,90784667	-0,15029333	0,901067236	Rrm1
17250740	11,58015	11,67504	11,77107	11,55386	11,6484	11,37313	11,67542	11,52513	-0,15029	0,901069318	Snord49b
17210831	5,753559	6,054643	6,329	6,104546	5,990842	5,591005	6,045734	5,895464333	-0,15026967	0,901082018	---
17390668	4,218661	4,322946	4,253191	4,239293	4,079924	4,024773	4,2649327	4,114663333	-0,15026933	0,901082226	---
17391544	7,863714	7,817757	7,782141	7,504601	7,669806	7,838411	7,821204	7,670939333	-0,15026467	0,901085141	Ckap2l
17504160	9,665877	9,522986	9,542049	9,450425	9,33753	9,492169	9,5769707	9,426708	-0,15026267	0,90108639	Polr2c
17336949	8,416538	8,45336	8,696464	8,662449	7,922961	8,530166	8,5221207	8,371858667	-0,150262	0,901086806	---
17380001	4,091062	4,041563	4,041542	4,003924	3,654915	4,0647	4,0580557	3,907846333	-0,15020933	0,901119702	---
17315407	3,056974	2,999703	3,291394	3,001312	2,900901	2,995242	3,1160237	2,965818333	-0,15020533	0,9011222	---
17390296	7,365511	7,600727	7,535147	7,339948	7,355624	7,35524	7,5004617	7,350270667	-0,150191	0,901131153	Ttbk2
17410124	4,353987	4,045603	4,017908	3,888175	4,061759	4,017009	4,139166	3,988981	-0,150185	0,901134901	Arsj // Arsj
17439764	5,622592	5,970422	6,37466	5,542437	5,910542	6,064163	5,9892247	5,839047333	-0,15017733	0,901139689	---
17414853	3,573177	4,070883	3,703945	4,11847	3,281692	3,497316	3,7826683	3,632492667	-0,15017567	0,90114073	---
17204507	3,363163	3,969231	4,350034	4,371528	3,435894	3,42448	3,8941427	3,743967333	-0,15017533	0,901140939	---
17321202	6,334103	6,076053	6,324629	6,258972	6,029442	5,995969	6,2449283	6,094794333	-0,150134	0,901166757	Zfp641
17205379	3,3778	3,079215	3,622548	3,437772	3,249243	2,942152	3,3598543	3,209722333	-0,150132	0,901168006	---
17455269	6,082259	5,537353	6,007107	5,762464	5,837323	5,576621	5,875573	5,725469333	-0,15010367	0,901185704	---
17290901	5,189868	5,651332	5,183457	5,080132	5,030895	5,463568	5,3415523	5,191531667	-0,15002067	0,901237552	Srsf10
17245539	5,685545	5,929652	5,706128	5,748442	5,725578	5,397305	5,773775	5,623775	-0,15	0,901250463	Srgap1
17295796	9,597031	9,659371	9,457632	9,372337	9,39855	9,493156	9,5713447	9,421347667	-0,149997	0,901252337	Pik3r1
17363070	4,387177	3,610352	4,227921	3,81778	3,761674	4,196026	4,07515	3,92516	-0,14999	0,90125671	Olfr1428
17536324	4,648505	4,874249	4,625285	4,394039	4,543122	4,760927	4,716013	4,566029333	-0,14998367	0,901260666	Gm14827
17311529	5,446103	5,285311	5,689552	5,478245	5,32739	5,165398	5,4736553	5,323677667	-0,14997767	0,901264414	---
17206789	4,4962	4,582147	4,565021	3,856414	5,080474	4,256674	4,5477893	4,397854	-0,14993533	0,901290861	---
17294408	5,464762	5,518304	5,314674	5,167842	5,361219	5,318914	5,43258	5,282658333	-0,14992167	0,901299399	---
17210515	7,736406	7,148609	7,214949	7,241225	7,043611	7,365489	7,3666547	7,216775	-0,14987967	0,901325638	---
17233355	3,086303	3,153102	2,876304	2,949213	3,004861	2,712062	3,0385697	2,888712	-0,14985767	0,901339383	---
17419377	8,757343	8,877355	8,672065	8,57972	8,63423	8,643248	8,768921	8,619066	-0,149855	0,901341049	Taf12
17281123	10,45313	10,88313	10,50469	10,30672	10,42882	10,656	10,61365	10,46384667	-0,14980333	0,901373328	---
17286998	5,976508	5,710328	5,728469	5,724553	5,865586	5,375766	5,8051017	5,655301667	-0,1498	0,901375411	Rbm24
17305933	3,131652	3,177248	3,365011	2,891867	3,348423	2,984362	3,224637	3,074884	-0,149753	0,901404777	Olfr725
17268079	3,245431	3,550694	3,397624	3,318488	3,03823	3,387803	3,3979163	3,248173667	-0,14974267	0,901411233	---
17210703	6,920453	6,638381	6,960225	6,774219	6,792192	6,503459	6,8396863	6,689956667	-0,14972967	0,901419355	---
17440998	9,960592	9,827618	9,919746	9,595302	9,818521	9,845019	9,902652	9,752947333	-0,14970467	0,901434976	Ankrd13a
17388212	5,773265	5,447279	6,028772	5,574043	5,542408	5,683782	5,749772	5,600077667	-0,14969433	0,901441433	---
17541986	8,957241	8,903113	8,60158	8,748293	8,60398	8,66064	8,8206447	8,670971	-0,14967367	0,901454346	Atp11c
17392894	6,679787	6,582517	6,676438	6,433524	6,565252	6,490987	6,6462473	6,496587667	-0,14965967	0,901463094	Fam110a
17209099	6,254476	5,896475	5,519051	6,16226	5,386892	5,671905	5,8900007	5,740352333	-0,14964833	0,901470175	---
17451970	4,259726	4,292374	4,093604	3,794399	4,116471	4,285967	4,2152347	4,065612333	-0,14962233	0,901486422	Sds // Sds
17225391	5,093211	5,007294	5,315414	5,128942	5,021049	4,817077	5,1386397	4,989022667	-0,149617	0,901489754	Iqca
17537397	3,228839	3,291462	3,015375	2,993394	3,073308	3,020193	3,1785587	3,028965	-0,14959367	0,901504334	Gm5945 // Gm5167
17224702	8,781565	8,972763	8,823961	8,661135	8,739923	8,728535	8,8594297	8,709864333	-0,14956533	0,901522039	Farsb
17250624	6,162925	6,129312	6,191667	5,898804	6,007462	6,128952	6,1613013	6,011739333	-0,149562	0,901524122	Fam83g
17207503	4,065001	3,615739	4,458358	3,70515	4,412117	3,573176	4,046366	3,896814333	-0,14955167	0,90153058	---
17549500	3,437378	3,580716	3,425686	3,290531	3,285082	3,419552	3,48126	3,331721667	-0,14953833	0,901538912	---
17549648	3,437378	3,580716	3,425686	3,290531	3,285082	3,419552	3,48126	3,331721667	-0,14953833	0,901538912	---
17219623	12,63426	12,72044	12,66734	12,41038	12,61896	12,54411	12,674013	12,52448333	-0,14953	0,901544119	Crp
17543744	5,592481	5,542491	5,807199	5,352588	5,396936	5,744079	5,6473903	5,497867667	-0,14952267	0,901548702	1700031F05Rik
17409277	5,980645	6,200734	6,102585	6,029977	6,029977	5,775468	6,0946547	5,945140667	-0,149514	0,901554118	Sort1
17448449	3,405827	3,371232	3,510595	3,257289	3,179509	3,402319	3,429218	3,279705667	-0,14951233	0,901555159	---
17250710	6,261076	6,31661	6,403594	6,205191	6,048787	6,278766	6,3270933	6,177581333	-0,149512	0,901555367	Trpv2
17485528	7,14082	7,310948	7,185495	6,909877	7,161536	7,117384	7,212421	7,062932333	-0,14948867	0,901569949	Tfpt
17452201	4,008995	3,716764	3,985236	3,848481	3,805036	3,609064	3,903665	3,754193667	-0,14947133	0,901580781	Adam1b
17260579	8,165494	8,184379	8,166612	7,854459	8,021029	8,192635	8,1721617	8,022707667	-0,149454	0,901591613	Hus1
17239787	7,452645	7,38025	7,366306	7,215647	7,367929	7,167264	7,3997337	7,25028	-0,14945367	0,901591821	Enpp1
17238226	11,96447	11,93654	11,93808	11,80015	11,82985	11,76073	11,946363	11,79691	-0,14945333	0,90159203	Naca
17204957	4,066699	4,644188	4,474928	4,3128	4,114557	4,310108	4,3952717	4,245821667	-0,14945	0,901594113	---
17468461	7,496325	7,378428	7,447727	7,232963	7,298318	7,342895	7,4408267	7,291392	-0,14943467	0,901603695	Cml1
17321248	8,270404	8,635738	8,345072	8,163038	8,276261	8,363659	8,4170713	8,267652667	-0,14941867	0,901613694	Ccnt1

17427641	4,7236	4,821999	4,994815	4,750965	4,64193	4,699273	4,8468047	4,697389333	-0,14941533	0,901615777	---
17526917	7,877365	8,005186	7,726985	7,583612	7,867073	7,710614	7,8698453	7,720433	-0,14941233	0,901617652	2310030G06Rik
17511410	4,794944	4,881277	5,181009	4,752216	4,819036	4,83778	4,95241	4,803010667	-0,14939933	0,901625777	Abcc12
17200529	5,239481	5,030354	5,344495	4,791349	4,981476	5,393417	5,2047767	5,055414	-0,14936267	0,901648692	---
17240376	4,766877	4,58209	4,851216	4,47395	4,700937	4,577241	4,7333943	4,584042667	-0,14935167	0,901655567	---
17404139	7,232732	7,434431	7,218273	6,974046	7,198874	7,264469	7,2951453	7,145796333	-0,149349	0,901657234	Snx16
17371912	7,374079	7,612202	7,48176	7,080911	7,33589	7,603198	7,489347	7,339999667	-0,14934733	0,901658275	B230120H23Rik
17548525	9,413558	9,611575	9,438592	9,269277	9,341719	9,40478	9,4879083	9,338592	-0,14931633	0,90167765	Hnrnpf
17248539	4,64787	4,883481	4,51992	4,653522	4,387845	4,561969	4,683757	4,534445333	-0,14931167	0,901680567	Gabrb2
17203191	3,527822	3,233259	3,627631	3,081424	3,19974	3,659647	3,462904	3,313603667	-0,14930033	0,90168765	---
17282100	3,967968	3,721234	3,787611	3,5137	3,987918	3,527335	3,8256043	3,676317667	-0,14928667	0,901696192	---
17290388	5,29293	5,272464	5,082961	4,863756	5,058586	5,278246	5,2161183	5,066862667	-0,14925567	0,901715567	Idi2 // Idi2
17354426	3,675091	3,824464	3,661669	3,576596	3,582454	3,554434	3,720408	3,571161333	-0,14924667	0,901721192	---
17344150	6,027201	5,893148	6,108557	5,97448	5,959588	5,647145	6,0096353	5,860404333	-0,149231	0,901730984	Ng23
17271769	5,633751	5,556111	5,289612	5,388611	5,239879	5,403292	5,493158	5,343927333	-0,14923067	0,901731193	Cd300e
17284222	6,438227	6,256655	6,505476	6,329607	6,177736	6,245327	6,4001193	6,25089	-0,14922933	0,901732026	Ahnak2
17281402	6,072637	6,323476	5,672116	5,915208	5,825131	5,880363	6,022743	5,873567333	-0,14917567	0,90176557	---
17464037	3,437929	3,570188	3,237823	3,378922	3,253702	3,165805	3,4153133	3,266143	-0,14917033	0,901768904	---
17482969	7,1629	7,040457	7,009039	6,935838	6,909276	6,919796	7,0707987	6,921636667	-0,149162	0,901774113	D430042O09Rik
17222596	4,025085	3,870111	3,755309	3,708996	3,675921	3,81811	3,8835017	3,734342333	-0,14915933	0,901775779	1700066B17Rik
17463509	9,612037	9,566822	9,495973	9,376867	9,527057	9,323484	9,5582773	9,409136	-0,14914133	0,901787031	Clec12a
17527810	3,87393	3,839624	4,073163	3,744514	3,819036	3,7758	3,9289057	3,779783333	-0,14912233	0,901798907	Tmem202
17475358	3,297618	3,003951	3,081278	2,833303	3,270892	2,831301	3,1276157	2,978498667	-0,149117	0,901802241	---
17271030	3,764615	3,477873	3,604834	3,368398	3,404874	3,626721	3,615774	3,466664333	-0,14910967	0,901806825	---
17502011	5,860507	5,844542	5,639315	5,635965	5,767871	5,493238	5,7814547	5,632358	-0,14909667	0,901814951	Gm3336
17475170	6,126833	5,909303	6,027754	6,049007	5,738862	5,828746	6,0212967	5,872205	-0,14909167	0,901818076	Zfp574
17267501	8,313363	8,642129	8,706515	8,26667	8,691221	8,256989	8,5540023	8,40496	-0,14904233	0,901848915	---
17456832	4,183077	4,376629	4,494036	4,219658	4,342105	4,044868	4,3512473	4,202210333	-0,149037	0,901852249	1700095J07Rik
17353541	10,95724	11,03602	10,90833	10,80353	10,79736	10,85359	10,967197	10,81816	-0,14903667	0,901852457	Etf1
17248242	8,004547	8,053944	8,097649	7,881875	7,906236	7,921023	8,0520467	7,903044667	-0,149002	0,901874128	Snrnp25
17422317	3,544809	3,249225	3,444971	3,237256	3,518858	3,035913	3,4130017	3,264009	-0,14899267	0,901879963	---
17278378	5,428244	5,195244	5,172272	5,096877	5,331652	4,920394	5,2652533	5,116307667	-0,14894567	0,901909345	Tcl1b4
17200895	5,17968	4,394858	5,133159	4,763088	4,534448	4,9634	4,9025657	4,753645333	-0,14892033	0,901925182	---
17320705	8,79796	8,621311	8,637733	8,408236	8,436584	8,765497	8,685668	8,536772333	-0,14889567	0,901940603	Yaf2
17412681	5,869288	5,75516	5,825507	5,464855	5,753079	5,785375	5,8166517	5,667769667	-0,148882	0,901949147	Cga
17550797	3,465408	3,253064	3,270892	3,285891	3,120687	3,136168	3,329788	3,180915333	-0,14887267	0,901954982	---
17253667	4,97204	4,909142	5,237321	4,904918	4,810318	4,956726	5,039501	4,890654	-0,148847	0,901971029	Sebox
17289851	5,731776	5,928321	6,155764	5,826526	5,671128	5,871714	5,9386203	5,789789333	-0,148831	0,901981032	---
17427064	3,487969	3,631433	3,446797	3,23274	3,262663	3,624342	3,5220663	3,373248333	-0,148818	0,90198916	Ifnb1
17426356	4,467956	4,370212	4,249779	4,182959	4,375193	4,083355	4,362649	4,213835667	-0,14881333	0,901992077	Tnfsf8
17468895	7,510743	7,348014	7,413014	7,379405	7,196042	7,249924	7,4239237	7,275123667	-0,1488	0,902000414	Chst13
17538796	8,356915	8,916442	8,668962	8,517098	8,451301	8,527562	8,6474397	8,498653667	-0,148786	0,902009167	---
17548383	6,891829	6,575415	6,756472	6,534806	6,607142	6,635427	6,7412387	6,592458333	-0,14878033	0,90201271	Rrs1 // Rrs1
17256537	8,368818	8,336015	8,145229	7,978133	8,245627	8,179972	8,283354	8,134577333	-0,14877667	0,902015002	Mlx
17395684	5,046917	4,795543	4,929154	4,72989	4,847679	4,747747	4,9238713	4,775105333	-0,148766	0,902021671	Bhlhe23
17225253	4,905517	5,080589	4,944759	4,852817	4,863544	4,768253	4,976955	4,828204667	-0,14875033	0,902031467	Kcnj13
17409491	6,92912	6,581108	6,691115	6,701753	6,456483	6,596925	6,733781	6,585053667	-0,14872733	0,902045847	---
17462812	3,99906	3,865841	4,124868	3,909916	3,6724	3,961298	3,9965897	3,847871333	-0,14871833	0,902051475	---
17462814	3,99906	3,865841	4,124868	3,909916	3,6724	3,961298	3,9965897	3,847871333	-0,14871833	0,902051475	---
17377568	5,02098	4,931071	5,041421	4,706747	4,955284	4,885289	4,997824	4,849106667	-0,14871733	0,9020521	4930442J19Rik
17422940	3,9663	4,383188	3,789635	4,035718	3,821282	3,835981	4,0463743	3,897660333	-0,148714	0,902054184	LOC670059
17278612	4,726935	4,772434	4,746193	4,337099	5,013986	4,448454	4,7485207	4,599846333	-0,14867433	0,902078986	Mir342 // Mir342
17306136	3,0682	2,919163	2,957965	2,869823	2,887421	2,742102	2,981776	2,833115333	-0,14866067	0,902087532	Gm5800
17276235	6,124051	6,201041	6,083959	5,974602	5,975164	6,01335	6,1363503	5,987705333	-0,148645	0,902097328	1810048J11Rik
17224540	10,23051	10,46228	10,26221	10,12409	10,25406	10,13094	10,318333	10,16969667	-0,14863667	0,902102538	Tuba4a
17459248	3,432367	3,492044	3,510074	3,244428	3,321259	3,422947	3,4781617	3,329544667	-0,148617	0,902114836	Vmn1r37
17309280	5,501539	5,035935	4,990035	4,985293	5,038278	5,058233	5,1758363	5,027268	-0,14856833	0,902145268	4930432J09Rik
17273240	5,611742	5,596447	5,971419	5,271126	5,774922	5,687922	5,726536	5,57799	-0,148546	0,902159233	Pycr1
17232444	6,938872	7,087694	6,916286	6,881118	6,759288	6,856818	6,9809507	6,832408	-0,14854267	0,902161318	Hddc2
17237273	8,618526	8,613157	8,619375	8,496134	8,354914	8,554413	8,6170193	8,468487	-0,14853233	0,902167779	---
17420539	4,91595	4,944474	4,977877	4,791512	4,753499	4,847775	4,9461003	4,797595333	-0,148505	0,902184872	Pla2g2a
17325339	3,881525	4,576402	3,888317	3,507546	4,35604	4,037218	4,1154147	3,966934667	-0,14848	0,902200506	2010005H15Rik // 2010005H15Rik
17209977	3,941876	3,314061	4,042764	3,778212	3,698653	3,376416	3,7662337	3,617760333	-0,14847333	0,902204675	---
17503043	7,014898	7,083446	7,067435	6,79416	7,141813	6,784414	7,0552597	6,906795667	-0,148464	0,902210512	Samd1
17247927	4,900362	5,160772	4,940899	4,715451	4,868895	4,972367	5,0006777	4,852237667	-0,14844	0,90222552	---
17242578	8,966573	8,873171	8,763146	8,722592	8,805898	8,629149	8,86763	8,719213	-0,148417	0,902239904	Ppap2c
17550500	4,255931	3,8749	4,013209	3,98918	3,840159	3,869511	4,0480133	3,899616667	-0,14839667	0,90225262	---
17231759	9,71079	10,09228	9,721378	9,427663	9,729277	9,922439	9,8414827	9,693126333	-0,14835633	0,902277845	Gm10335
17355842	6,270545	6,291876	6,608868	5,948368	6,410842	6,367061	6,3904297	6,242090333	-0,14833933	0,902288477	---
17441580	5,531483	5,262232	5,264139	5,337952	5,133386	5,141618	5,352618	5,204318667	-0,14829933	0,902313494	1700081H04Rik
17435451	5,958786	6,247558	6,228076	5,753049	6,166919	6,069609	6,1448067	5,996525667	-0,148281	0,902324961	1500035N22Rik
17284002	8,55352	8,741953	8,614261	8,487936	8,421869	8,555206	8,636578	8,488337	-0,148241	0,902349979	Hsp90aa1
17362966	4,1071	3,867799	3,895796	3,850615	3,567133	4,00823	3,9568983	3,808659333	-0,148239	0,90235123	BE692007
17538828	7,046453	6,565117	6,803337	6,925071	6,744006	6,30116	6,804969	6,656745667	-0,14822333	0,902361029	---
17270615	4,526615	5,147709	4,792654	5,029474	4,135147	4,857709	4,822326	4,67411	-0,148216	0,902365615	---
17428752	5,615011	5,477688	5,642724	5,390039	5,349575	5,551174	5,5784743	5,430262667	-0,14821167	0,902368326	Btbd19
17234742	6,558609	6,193597	6,384021	6,196957	6,367369	6,127346	6,3787423	6,230557333	-0,148185	0,902385005	Gm3285
17455962	10,19716	10,21627	10,03418	9,917114	9,954172	10,13194	10,149203	10,00107533	-0,148128	0,902420659	C1galt1
17549564	7,321378	7,108351	7,246538	7,08946	7,075253	7,067172	7,2254223	7,077295	-0,14812733	0,902421076	---
17549720	7,321378	7,108351	7,246538	7,08946	7,075253	7,067172	7,2254223	7,077295	-0,14812733	0,902421076	---
17252032	7,910514	7,721487	7,715259	7,488395	7,611334	7,803189	7,78242	7,634306	-0,148114	0,902429416	Med11
17535515	3,768694	3,00321	3,258669	3,264983	2,97769	3,343664	3,3435243	3,195445667	-0,14807867	0,902451518	Xlr4b
17537029	4,91721	4,885617	5,099198	4,775697	4,754496	4,927904	4,9673417	4,819365667	-0,147976	0,902515741	Magee1
17291091	4,162724	3,999894	4,112876	4,098057	3,799912	3,933626	4,0918313	3,943865	-0,14796633	0,902521788	Vmn1r208
17253858	8,080315	8,449714	8,202271	8,084661	8,134478	8,069266	8,2441	8,096135	-0,147965	0,902522623	Atad5
17499792	5,225907	5,139582	5,285447	4,983593	5,168425	5,055024	5,2169787	5,069014	-0,14796467	0,902522831	Defa17
17392676	8,753712	8,874881	8,658224	8,592687	8,71304	8,537401	8,7622723	8,614376	-0,14789633	0,90256558	2310001A20Rik
17334192	6,641614	6,5943	6,787662	6,344359	6,726963	6,508601	6,6745253	6,526634333	-0,147891	0,902568917	Prss27
17245167	3,687524	3,320481	3,176198	3,439696	3,134743	3,16625	3,3947343	3,246896333	-0,147838	0,902602075	---
17360785	5,088402	5,103835	5,292639	5,078569	4,795842	5,166991	5,1616253	5,013800667	-0,14782467	0,902610417	Pnliprp2
17547777	4,210139	4,499987	4,54752	4,165319	4,332108	4,316792	4,4192153	4,271406333	-0,147809	0,902620218	Gm19281
17285157	8,419486	8,611572	8,319186	8,252644	8,409198	8,244978	8,4500813	8,302273333	-0,147808	0,902620844	Heatr1
17472129	7,732396	7,600467	7,335913	7,097128	7,580414	7,54785	7,5562587	7,408464	-0,14779467	0,902629186	Gucy2c
17470513	3,08988	3,084477	3,353972	3,275343	2,93498	2,874694	3,1761097	3,028339	-0,14777067	0,902644202	---
17244679	6,216571	6,720171	6,439568	5,844957	6,661873	6,426207	6,45877	6,311012333	-0,14775767	0,902652336	BC067068
17200643	4,019428	4,037098	4,245294	4,093133	3,719344	4,046081	4,1006067	3,952852667	-0,147754	0,90265463	---
17368422	6,14714	5,969656	5,757121	5,860086	5,667277	5,903349	5,9579723	5,810237333	-0,147735	0,902666518	Adamts13
17291964	10,06189	10,41324	10,01309	10,05307	9,968623	10,02334	10,16274	10,015011	-0,147729	0,902670272	Eef1e1
17419028	9,174322	9,143133	9,155664	9,350639	9,029793	8,649666	9,1577063	9,010032667	-0,14767367	0,902704894	---
17306858	10,68925	10,77792	10,85717	10,6479	10,69742	10,53606	10,77478	10,62712667	-0,14765333	0,902717616	---
17209007	6,143991	6,849457	6,244975	5,842207	6,639026	6,314256	6,4128077	6,265163	-0,14764467	0,902723039	---
17356061	6,682973	6,710108	6,824371	6,422563	6,797931	6,554038	6,7391507	6,591510667	-0,14764	0,902725959	Nudt8
17288967	4,800874	4,54106	4,597809	4,479805	4,497095	4,519947	4,646581	4,498949	-0,147632	0,902730965	---
17499979	4,308261	4,436587	4,543743	4,493251	3,977539	4,375011	4,4295303	4,281933667	-0,14759667	0,902753074	---
17205715	4,672843	4,353945	4,635231	4,48294	4,383716	4,352617	4,5540063	4,406424333	-0,147582	0,902762252	---
17285036	4,438915	4,80443	4,731214	4,355201	4,807852	4,368779	4,6581863	4,510610667	-0,14757567	0,902766215	Adarb2
17330684	5,190534	5,040508	5,261064	4,98958	5,132716	4,927106	5,1640353	5,016467333	-0,147568	0,902771012	Tagln3
17266074	7,784488	7,792962	7,915497	7,693056	7,663902	7,693399	7,8309823	7,683452333	-0,14753	0,902794791	Gemin4
17340099	8,773138	8,61162	8,576088	8,578552	8,27288	8,667032	8,6536153	8,506154667	-0,14746067	0,902838179	Abcg8
17301117	5,677207	5,37344	5,516126	5,302616	5,378371	5,443519	5,5222577	5,374835333	-0,14742233	0,902862168	Trim13
17255565	4,661228	3,980633	4,210515	3,975035	4,205725	4,229431	4,2841253	4,136730333	-0,147395	0,902879274	Hoxb8
17354992	10,5283	10,61524	10,52662	10,28747	10,42828	10,51238	10,55672	10,40937667	-0,14734333	0,902911609	Amd1 // Amd1
17457872	4,779413	3,984303	3,752422	3,935113	4,155593	3,983504	4,172046	4,024736667	-0,14730933	0,902932888	Tas2r139
17387886	4,072516	3,974303	4,131899	3,987267	3,84552	3,904052	4,0595727	3,912279667	-0,147293	0,902943111	Olfr1218
17417860	7,983223	7,98921	7,985966	7,797315	7,868899	7,850364	7,986133	7,838859333	-0,14727367	0,902955211	Ccdc23
17372019	2,901383	2,966922	3,041606	2,922505	2,805165	2,740431	2,9699703	2,822700333	-0,14727	0,902957506	---
17278626	6,594757	6,429289	6,738713	6,823004	6,242739	6,25522	6,5875863	6,440321	-0,14726533	0,902960427	Mir345
17207431	6,848831	6,869913	7,107177	7,1386	6,650656	6,594881	6,9419737	6,794712333	-0,14726133	0,90296293	---
17386479	8,522602	8,663336	8,835642	8,302443	8,577328	8,70014	8,67386	8,526637	-0,147223	0,902986923	Mir5115 // Mir5115
17339390	9,191987	9,630419	9,340843	9,20392	9,129882	9,387819	9,3877497	9,240540333	-0,14720933	0,902995477	Gm16519
17359098	7,263568	7,160316	6,99225	6,844958	7,099056	7,030576	7,1387113	6,99153	-0,14718133	0,903013003	Cep55
17378125	3,888606	3,841221	3,96432	3,682447	3,876081	3,694103	3,898049	3,750877	-0,147172	0,903018845	Bpifa1
17414836	6,989322	7,382258	6,998785	6,708759	7,185012	7,0351	7,123455	6,976290333	-0,14716467	0,903023435	Tlr4
17475336	6,285836	6,414018	6,284245	6,103946	6,216431	6,222249	6,328033	6,180875333	-0,14715767	0,903027816	B9d2
17536074	6,01356	5,955279	5,876938	5,814082	5,938454	5,65179	5,9485923	5,801442	-0,14715033	0,903032406	---
17469461	5,178598	5,242692	5,313178	5,08525	5,33219	4,875604	5,2448227	5,097681333	-0,14714133	0,90303804	---
17479959	4,283632	3,693344	3,651417	3,629388	3,871209	3,686524	3,8761643	3,729040333	-0,147124	0,90304889	Vmn2r78
17334040	3,320334	3,255229	3,570995	3,304282	3,180471	3,220481	3,382186	3,235078	-0,147108	0,903058905	Vmn2r-ps129
17224395	6,980291	6,740429	7,193584	6,916695	6,806212	6,750093	6,9714347	6,824333333	-0,14710133	0,903063078	Mir375
17548080	7,390248	7,605245	7,529717	7,46262	7,372262	7,249038	7,5084033	7,361306667	-0,14709667	0,903065999	Polr2k // Polr2k
17441357	4,636511	4,491551	4,598008	4,50027	4,45958	4,324971	4,5753567	4,428273667	-0,147083	0,903074554	---
17548168	3,869256	3,882852	3,639191	3,712818	3,717664	3,51964	3,7970997	3,650040667	-0,147059	0,903089577	Gm10765
17547535	5,178391	5,312496	5,100873	5,130084	4,957006	5,063498	5,1972533	5,050196	-0,14705733	0,90309062	Gm20276
17404098	6,286922	6,10375	6,371182	6,244703	5,742929	6,333057	6,2539513	6,106896333	-0,147055	0,903092081	Fabp12

17501692	7,655457	7,640551	7,661465	7,484703	7,39851	7,633193	7,652491	7,505468667	-0,14702233	0,90311253	Atp13a1
17352227	5,08783	5,140502	5,164906	5,244258	4,64539	5,062529	5,1310793	4,984059	-0,14702033	0,903113782	---
17418024	4,448185	4,704855	4,517512	4,382427	4,33557	4,511567	4,5568507	4,409854667	-0,146996	0,903129014	Gm8439
17549240	4,00373	3,951795	3,599271	3,967137	3,60376	3,543054	3,8515987	3,704650333	-0,14694833	0,903158854	---
17246497	4,318026	4,363897	4,038108	4,02407	4,168417	4,086705	4,2400103	4,093064	-0,14694633	0,903160106	Vmn2r87
17270673	4,012946	4,070778	4,223012	3,957618	4,011725	3,896581	4,1022453	3,955308	-0,14693733	0,90316574	---
17492619	9,547961	9,759263	9,656301	9,495657	9,521598	9,505493	9,6545083	9,507582667	-0,14692567	0,903173044	Blm
17491788	6,765242	6,776299	7,017388	6,652868	6,714088	6,751244	6,8529763	6,706066667	-0,14690967	0,903183061	Klf13
17337118	5,468562	5,022645	4,851606	5,335852	4,654987	4,911261	5,114271	4,967366667	-0,14690433	0,903186399	---
17206443	4,823205	4,148752	4,198946	4,290033	4,633389	3,806827	4,390301	4,243416333	-0,14688467	0,903198712	---
17422662	8,463848	8,506623	8,45064	8,214005	8,39851	8,367955	8,4737037	8,326823333	-0,14688033	0,903201425	Ssu72
17542084	3,745331	3,463717	3,339661	3,232751	3,348977	3,526397	3,5162363	3,369375	-0,14686133	0,90321332	Mir871
17206213	4,761418	4,388702	4,701714	4,458685	4,836999	4,115727	4,617278	4,470470333	-0,14680767	0,903246919	---
17292170	4,655169	4,552031	4,254398	4,198741	4,278492	4,543965	4,4871993	4,340399333	-0,1468	0,903251719	---
17271143	5,197878	5,56551	5,658875	5,374235	5,337481	5,270171	5,4740877	5,327295667	-0,146792	0,903256727	Cacng1
17459706	4,938721	4,759484	4,881102	4,68136	4,64651	4,811199	4,859769	4,713023	-0,146746	0,903285528	---
17284450	4,134907	3,493741	3,51964	3,053003	3,644699	4,010443	3,716096	3,569381667	-0,14671433	0,903305355	---
17463967	4,004884	4,016096	4,196021	4,157194	3,852213	3,767533	4,0723337	3,925646667	-0,146687	0,903322469	Capza3
17322125	7,468772	7,825138	7,799351	7,451657	7,512471	7,689161	7,6977537	7,551096333	-0,14665733	0,903341045	Krt18 // Krt18
17368526	8,41839	8,47998	8,618738	8,199587	8,455563	8,422014	8,5057027	8,359054667	-0,146648	0,903346889	Wdr5
17211000	7,765433	7,530323	7,680037	7,598201	7,886988	7,050952	7,6585977	7,512047	-0,14655067	0,903407836	Rrs1
17550749	3,400263	3,217866	3,232461	3,105398	3,207674	3,097889	3,28353	3,136987	-0,146543	0,903412637	---
17503446	8,175781	8,202647	7,952037	7,817421	8,071815	8,001619	8,110155	7,963618333	-0,14653667	0,903416603	Gpt2
17282958	3,461124	4,032043	3,13333	3,409941	3,545478	3,231514	3,5421657	3,395644333	-0,14652133	0,903426205	Gm5039
17351835	10,0891	10,17429	10,21273	10,02815	9,954454	10,054	10,158707	10,01220133	-0,14650533	0,903436224	---
17205615	4,282362	4,188608	4,478082	3,904363	4,304848	4,30033	4,3163507	4,169847	-0,14650367	0,903437268	---
17250126	3,387135	3,707709	3,669789	3,459569	3,473044	3,392545	3,588211	3,441719333	-0,14649167	0,903444783	---
17200153	5,642903	6,050076	5,985402	5,29815	6,001968	5,938931	5,8927937	5,746349667	-0,146444	0,903474633	---
17548238	11,63336	11,82028	11,63723	11,6416	11,40398	11,60605	11,696957	11,55054333	-0,14641333	0,903493838	Fabp5
17326527	9,315416	9,411242	9,274426	9,133504	9,172596	9,255829	9,3336947	9,187309667	-0,146385	0,903511582	Cggbp1
17438134	6,99779	7,27615	7,095009	7,021809	7,033002	6,874991	7,122983	6,976600667	-0,14638233	0,903513252	Dcun1d4
17479839	2,944595	2,983467	3,066217	2,937224	2,813412	2,804559	2,998093	2,851731667	-0,14636133	0,903526404	---
17403994	4,333706	4,296975	3,991838	4,08372	4,053175	4,046546	4,2075063	4,061147	-0,14635933	0,903527656	Il7
17278236	4,5513	4,650673	4,822856	4,566185	4,522603	4,497002	4,674943	4,528596667	-0,14634633	0,903535798	---
17461919	7,813565	7,187709	7,681074	7,6708	7,186557	7,385969	7,5607827	7,414442	-0,14634067	0,903539347	---
17491739	3,055596	3,118503	3,398254	3,081399	3,067342	2,984606	3,1907843	3,044449	-0,14633533	0,903542687	Mir344-2
17430669	4,739786	4,67509	5,209339	4,80228	4,747183	4,63581	4,8747383	4,728424333	-0,146314	0,903556048	---
17289422	4,556035	4,875203	4,710784	4,553645	4,628619	4,520817	4,7140073	4,567693667	-0,14631367	0,903556257	Foxd1
17393878	5,037756	5,162925	5,069131	5,038209	4,834685	4,957986	5,0899373	4,943626667	-0,14631067	0,903558136	Plcg1 // Plcg1
17211867	5,31582	5,208839	5,456657	5,033054	5,153062	5,356285	5,3271053	5,180800333	-0,146305	0,903561685	Zap70
17265229	7,554926	7,607952	7,92852	7,327644	7,744438	7,580431	7,6971327	7,550837667	-0,146295	0,903567948	Alox15
17289660	8,45336	8,488734	8,415975	8,161626	8,365381	8,392259	8,4526897	8,306422	-0,14626767	0,903585067	Dimt1
17495250	4,846558	4,778259	4,990141	4,585348	4,636125	4,95476	4,8716527	4,725411	-0,14624167	0,903601351	---
17550807	3,362192	3,505057	3,52936	3,26357	3,355992	3,338332	3,4655363	3,319298	-0,14623833	0,903603439	---
17294485	5,257464	4,872938	5,205377	4,987888	5,063758	4,845434	5,1119263	4,965693333	-0,146233	0,903606779	Gpr150
17252574	5,037438	4,599469	4,8676	4,562333	4,940428	4,563062	4,8348357	4,688607667	-0,146228	0,903609911	Itgae
17549526	4,561614	4,760574	4,896836	4,570932	4,719258	4,490152	4,7396747	4,593447333	-0,14622733	0,903610329	---
17549678	4,561614	4,760574	4,896836	4,570932	4,719258	4,490152	4,7396747	4,593447333	-0,14622733	0,903610329	---
17498958	5,494745	5,252707	5,619696	5,312211	5,414929	5,201341	5,455716	5,309493667	-0,14622233	0,90361346	---
17207709	3,239712	3,053078	3,128822	2,808741	2,886596	3,28774	3,1405373	2,994359	-0,14617833	0,90364102	---
17208679	6,577453	7,465613	6,915496	6,883945	6,953845	6,682277	6,9861873	6,840022333	-0,146165	0,903649371	---
17508833	4,149372	4,595806	4,124036	4,391096	3,972162	4,06747	4,289738	4,143576	-0,146162	0,90365125	---
17431053	6,63244	6,625703	6,731062	6,418412	6,591137	6,541173	6,6630683	6,516907333	-0,146161	0,903651877	Zdhhc18
17379224	4,899426	4,604129	5,310534	4,887784	4,624008	4,863937	4,9380297	4,791909667	-0,14612	0,903677558	---
17472364	4,826156	4,728162	4,662867	4,624336	4,474974	4,679613	4,7390617	4,592974333	-0,14608733	0,90369802	Slco1a4
17297999	4,80622	4,673441	4,800279	4,813035	4,354918	4,673761	4,75998	4,613904667	-0,14607533	0,903705537	Asb14
17399972	3,743164	3,843843	3,743164	3,452238	3,686696	3,7531	3,7767237	3,630678	-0,14604567	0,90372412	Hrnr
17389093	7,486168	7,454803	7,471446	7,356394	7,37858	7,239338	7,4708057	7,324770667	-0,146035	0,903730802	2700007P21Rik
17414991	5,067602	4,763469	4,991835	4,896799	4,811416	4,676677	4,9409687	4,794964	-0,14600467	0,903749803	Gm11412
17269717	9,372488	9,800393	9,519543	9,391749	9,441918	9,420745	9,5641413	9,418137333	-0,146004	0,903750221	Stat3
17204825	4,945794	4,19408	3,892204	3,958092	4,00437	4,631647	4,344026	4,198036333	-0,14598967	0,9037592	---
17341285	3,389726	3,109655	3,445814	3,074228	3,231245	3,201989	3,315065	3,169154	-0,145911	0,903808481	Vmn2r94
17348648	6,315418	6,371771	6,235703	6,090758	6,139383	6,255028	6,3076307	6,161723	-0,14590767	0,903810569	Taf4b
17201129	8,380094	8,118495	8,076116	8,073371	7,620439	8,443268	8,1915683	8,045692667	-0,14587567	0,903830617	---
17329624	4,099146	3,937855	4,035663	3,7965	4,053714	3,784839	4,0242213	3,878351	-0,14587033	0,903833958	---
17534673	9,147976	8,97371	9,015673	8,57623	9,121655	9,0019	9,0457863	8,899928333	-0,145858	0,903841685	2610018G03Rik
17384177	5,45723	5,550379	5,285175	4,945774	5,346453	5,563008	5,430928	5,285078333	-0,14584967	0,903846906	Zbtb34
17427096	3,136156	3,317734	3,052189	2,990808	3,137377	2,940358	3,168693	3,022847667	-0,14584533	0,90384962	Gm13280
17544740	3,604301	3,521976	3,699079	3,570403	3,498534	3,318925	3,608452	3,462620667	-0,14583133	0,903858391	Kir3dl2
17529196	4,14232	3,900714	4,201399	4,137272	3,736978	3,932733	4,0814777	3,935661	-0,14581667	0,90386758	---
17296545	4,36071	4,498844	4,44427	4,377289	4,358178	4,130921	4,434608	4,288796	-0,145812	0,903870504	AF067063 // AF067063
17523188	8,120258	8,384	8,450172	8,186146	8,219125	8,11175	8,3181433	8,172340333	-0,145803	0,903876143	---
17472735	4,423229	3,875768	3,534948	3,857568	3,838931	3,700101	3,9446483	3,798866667	-0,14578167	0,903889508	---
17293142	9,38916	9,199555	9,16295	8,912955	9,374681	9,026685	9,250555	9,104773667	-0,14578133	0,903889717	Hnrnpk
17307690	5,295115	5,296336	5,350694	5,166208	5,085938	5,252664	5,3140483	5,16827	-0,14577833	0,903891597	---
17457802	5,316153	5,2355	5,814138	5,364736	5,259308	5,304472	5,4552637	5,309505333	-0,14575833	0,903904128	---
17273694	5,637228	5,274502	5,518739	5,505383	5,189856	5,288889	5,476823	5,331076	-0,145747	0,903911228	Pomc
17244726	5,704621	5,830331	6,002538	5,983732	5,730579	5,385952	5,84583	5,700087667	-0,14574233	0,903914152	Myf5
17329023	6,191625	5,545977	6,122545	6,010261	5,565777	5,846928	5,9533823	5,807655333	-0,145727	0,903923759	Iglv1 // Iglv1
17548717	11,61756	11,8143	11,61672	11,63507	11,38302	11,59332	11,68286	11,53713667	-0,14572333	0,903926057	Fabp5 // Fabp5
17486771	4,490239	4,656211	4,164145	4,562216	4,046777	4,264472	4,436865	4,291155	-0,14571	0,903934411	Crx
17545150	5,16579	4,942242	4,943164	4,975596	4,81575	4,822901	5,0170653	4,871415667	-0,14564967	0,903972214	Lhfpl1
17284493	3,058721	3,192453	2,930465	2,837905	2,922368	2,984456	3,0605463	2,914909667	-0,14563667	0,90398036	Igh-VJ558 // Igh-VJ558
17236745	3,583774	4,120158	3,749476	3,799347	3,317288	3,899867	3,8178027	3,672167333	-0,14563533	0,903981195	---
17203987	7,138014	6,258246	6,03508	6,386433	5,881242	6,726862	6,4771133	6,331512333	-0,145601	0,904002708	---
17200059	6,389581	6,970099	6,771	6,187062	6,715337	6,791503	6,7102267	6,564634	-0,14559267	0,90400793	---
17401725	3,727779	3,601895	4,103111	3,731202	3,857494	3,40732	3,8109283	3,665338667	-0,14558967	0,90400981	---
17549220	3,258793	3,56672	3,51964	3,316553	3,365092	3,226817	3,4483843	3,302820667	-0,14556367	0,904026102	---
17470515	5,058808	5,064071	5,342026	5,011801	5,047282	4,969273	5,1549683	5,009452	-0,14551633	0,904055763	Iqsec3
17427655	4,380489	4,506704	4,19879	4,215429	4,215678	4,218341	4,3619943	4,216482667	-0,14551167	0,904058687	Insl5
17381521	3,254937	3,449736	3,482803	3,275587	3,286846	3,188527	3,3958253	3,25032	-0,14550533	0,904062656	---
17201167	6,935733	6,930648	6,383141	6,325341	6,60988	6,877819	6,7498407	6,604346667	-0,145494	0,904069758	---
17316272	3,246828	2,850835	2,977462	2,900401	2,869996	2,868386	3,0250417	2,879594333	-0,14544733	0,904099002	---
17217048	5,208719	5,383393	5,265242	4,809233	5,218841	5,392991	5,2857847	5,140355	-0,14542967	0,904110073	5430435G22Rik
17324297	4,673495	4,636905	4,634207	4,542217	4,554139	4,412001	4,6482023	4,502785667	-0,14541667	0,90411822	9230117E06Rik
17446466	3,374291	4,116879	3,876946	3,768938	3,577032	3,586024	3,789372	3,643998	-0,145374	0,904144959	Gm5862
17275950	6,110993	6,117738	6,224425	6,256976	5,862598	5,89755	6,151052	6,005708	-0,145344	0,904163761	4931403G20Rik
17291325	5,493804	5,510471	5,382986	5,28961	5,508417	5,153319	5,4624203	5,317115333	-0,145305	0,904188203	---
17357207	8,756729	8,802468	9,01712	8,83608	8,69426	8,610071	8,8587723	8,713470333	-0,145302	0,904190083	Tmem223
17400881	4,854008	4,670962	4,819635	4,714317	4,583063	4,611378	4,781535	4,636252667	-0,14528233	0,904202409	---
17502781	8,018766	8,320878	7,73338	7,830251	7,7215	8,085447	8,0243413	7,879066	-0,14527533	0,904206796	Anapc10 // Anapc10
17278090	6,126884	6,044444	6,081286	5,673822	6,112535	6,030469	6,0842047	5,938942	-0,14526267	0,904214735	Chga
17373508	5,738537	5,509828	6,075105	5,613623	5,705625	5,568454	5,77449	5,629234	-0,145256	0,904218914	D930015M05Rik
17328249	3,212335	3,561735	3,721054	3,246081	3,388542	3,424742	3,4983747	3,353121667	-0,145253	0,904220794	---
17429826	5,491053	5,403666	5,779993	5,491394	5,589171	5,158575	5,5582373	5,413046667	-0,14519067	0,904259863	---
17403457	6,305736	6,36878	6,222838	5,994829	6,274883	6,192133	6,299118	6,153948333	-0,14516967	0,904273025	2410004B18Rik
17481594	4,177799	3,718621	3,54316	3,933041	3,357552	3,713546	3,8131933	3,668046333	-0,145147	0,904287233	Olfr473
17473365	3,238625	3,359573	3,339694	2,994053	3,359795	3,148612	3,3126307	3,167486667	-0,145144	0,904289113	---
17391563	7,434717	7,06901	7,946974	7,314046	7,513874	7,187425	7,483567	7,338448333	-0,14511867	0,904304992	---
17298131	7,645696	7,677643	7,795373	7,606976	7,527134	7,549338	7,7062373	7,561149333	-0,145088	0,904324215	Selk
17489518	2,7776	2,745832	3,4629	2,911593	2,754151	2,885363	2,995444	2,850369	-0,145075	0,904332364	---
17392543	4,520699	4,243533	4,35427	4,109491	4,352906	4,220881	4,372834	4,227759333	-0,14507467	0,904332573	---
17203199	2,845176	2,730611	3,17311	2,88413	2,822814	2,606781	2,916299	2,771241667	-0,14505733	0,904343438	---
17404731	6,536972	6,337034	6,449736	6,423927	6,124347	6,340322	6,4412473	6,296198667	-0,14504867	0,90434887	Dcun1d1
17285510	3,502847	3,796206	3,671005	3,587121	3,441822	3,505985	3,656686	3,511642667	-0,14504333	0,904352214	---
17358266	4,675466	4,14524	4,624555	4,307069	4,466443	4,236641	4,4817537	4,336717667	-0,145036	0,904356811	Pgm5
17344238	5,87302	5,883923	6,132798	5,690537	5,85048	5,913664	5,963247	5,818227	-0,14502	0,90436684	Gpank1
17289699	6,724553	6,974711	6,648034	6,614733	6,616122	6,681383	6,7824327	6,637412667	-0,14502	0,90436684	Ercc8
17491511	3,471663	3,053003	3,033189	2,944422	3,102156	3,076242	3,1859517	3,04094	-0,14501167	0,904372064	---
17246424	3,503326	3,35472	3,577027	3,358716	3,314988	3,326402	3,4783577	3,333368667	-0,144989	0,904386273	Olfr802
17449718	7,382467	8,649576	6,884626	7,366348	7,359099	7,756341	7,6388897	7,493929333	-0,14496033	0,904404244	Cxcl10
17540743	4,210649	4,7793	4,021111	4,394301	4,123462	4,05842	4,33702	4,192061	-0,144959	0,90440508	Slx
17387363	10,49915	10,94833	10,80047	10,71047	10,47892	10,62385	10,749317	10,60441333	-0,14490333	0,904439977	---
17438697	4,137838	4,55899	4,093682	4,097822	4,098953	4,159235	4,2635033	4,11867	-0,14483333	0,904483862	Csn3
17444309	5,631674	5,36898	5,509218	5,219397	5,619438	5,236607	5,5032907	5,358480667	-0,14481	0,90449849	Grid2ip
17207879	4,93365	4,941309	5,054095	4,3665	5,501358	4,62678	4,9763513	4,831546	-0,14480533	0,904501416	---
17318734	6,108389	5,652753	6,16479	6,152458	5,740364	5,598701	5,9753107	5,830507667	-0,144803	0,904502879	Zfp647
17361683	8,072586	8,257857	8,110821	8,071212	7,945023	7,990809	8,147088	8,002348	-0,14474	0,904542378	Sipa1
17489928	3,371703	3,3892	3,400299	3,224704	3,332335	3,169953	3,3870673	3,242330667	-0,14473667	0,904544468	Gm5591
17252689	8,258842	8,135967	7,966024	7,960431	7,942452	8,023804	8,1202777	7,975562333	-0,14471533	0,904557844	Spata22
17337368	6,431069	5,904969	6,521224	6,27634	6,2828	5,864055	6,285754	6,141065	-0,144689	0,904574355	A930015D03Rik
17251282	7,303146	7,167073	7,402323	7,186666	7,228912	7,022934	7,2908473	7,146170667	-0,14467667	0,904582088	Stx8
17299279	7,696835	7,691665	7,524193	7,46984	7,495314	7,513535	7,6375643	7,492896333	-0,144668	0,904587522	Cgrrf1
17407435	5,500578	4,867864	4,843294	4,739529	5,394886	4,64341	5,0705787	4,925941667	-0,144637	0,904606959	Lce1b

17491642	3,128061	3,194874	2,907881	2,891901	2,946959	2,958099	3,0769387	2,932319667	-0,144619	0,904618246	---
17232197	3,44688	3,147887	3,4142	3,183684	3,258639	3,132792	3,3363223	3,191705	-0,14461733	0,904619291	Taar8a
17258783	5,018491	4,426418	5,175094	4,45576	4,772622	4,957864	4,8733343	4,728748667	-0,14458567	0,904639147	---
17280913	5,658384	5,616716	5,835464	5,501881	5,565396	5,609538	5,7035213	5,558938333	-0,144583	0,904640819	Nova1
17263388	5,209355	5,279627	5,666087	5,200706	5,155225	5,36541	5,385023	5,240447	-0,144576	0,904645209	Wnt3a
17485891	5,654627	5,488053	5,735084	5,388912	5,443201	5,611956	5,6259213	5,481356333	-0,144565	0,904652106	Tmem150b
17525393	4,642896	4,738885	4,69251	4,515873	4,522992	4,60175	4,6914303	4,546871667	-0,14455867	0,904656078	---
17316021	9,914279	10,06939	9,978236	9,80969	9,957534	9,761129	9,9873017	9,842784333	-0,14451733	0,904681996	Tars
17231287	6,472976	6,227031	6,541308	6,489054	5,983517	6,335272	6,4137717	6,269281	-0,14449067	0,904698719	Cd46
17340673	5,344532	5,36761	5,527698	5,211555	5,295141	5,299692	5,41328	5,268796	-0,144484	0,904702899	Fndc1
17237603	3,674317	3,610678	3,885324	3,673266	3,677123	3,386499	3,7234397	3,578962667	-0,144477	0,904707289	1700025F24Rik
17322788	5,652948	5,583766	5,618049	5,615834	5,30501	5,500521	5,6182543	5,473788333	-0,144466	0,904714187	---
17503092	3,928646	4,425885	4,083035	3,899684	4,195415	3,909086	4,1458553	4,001395	-0,14446033	0,904717741	Mir709
17206625	4,686366	6,136818	5,459672	5,180941	5,404752	5,263825	5,4276187	5,283172667	-0,144446	0,904726729	---
17358825	4,342712	4,354075	3,861104	4,096356	3,92245	4,105779	4,1859637	4,041528333	-0,14443533	0,904733418	I830012O16Rik
17503703	6,69365	7,022977	6,858758	6,481987	6,802843	6,857319	6,8584617	6,714049667	-0,144412	0,904748051	---
17486513	3,409007	3,743027	3,351153	3,153102	3,460395	3,456462	3,5010623	3,356653	-0,14440933	0,904749723	Vmn1r72
17438322	10,05441	10,01865	10,05192	9,893081	9,804045	9,994667	10,04166	9,897264333	-0,14439567	0,904758294	---
17485963	5,254961	5,501808	5,52313	5,24495	5,529997	5,071795	5,426633	5,282247333	-0,14438567	0,904764565	Sbk2
17353626	6,389501	6,403213	6,177499	6,174571	6,223582	6,139063	6,3234043	6,179072	-0,14433233	0,904798013	Gm1614
17336634	8,280249	8,308327	8,211859	7,951503	8,127012	8,28893	8,2668117	8,122481667	-0,14433	0,904799477	---
17341975	6,391637	6,229296	6,510702	6,245975	6,267053	6,185635	6,3772117	6,232887667	-0,144324	0,90480324	Npw
17546504	3,320297	3,379181	3,327656	3,090529	3,51632	2,987443	3,342378	3,198097333	-0,14428067	0,904830417	LOC100042494
17308815	4,261897	4,120176	4,160611	4,093719	3,962734	4,053425	4,1808947	4,036626	-0,14426867	0,904837943	Mir1971
17336432	7,420845	8,629023	7,413172	7,802118	7,515803	7,712314	7,8210133	7,676745	-0,14426833	0,904838152	Tap1
17435769	5,035949	5,164635	5,379327	5,195839	5,197586	4,753723	5,1933037	5,049049333	-0,14425433	0,904846933	Mir1960
17317115	6,233002	6,753644	6,540971	6,27552	6,466418	6,352921	6,5092057	6,364953	-0,14425267	0,904847978	Dscc1
17548468	8,748746	8,754601	8,3995	8,42662	8,475822	8,567708	8,6342823	8,49005	-0,14423233	0,904860731	Rbpms2 // Rbpms2
17438814	9,383282	9,343961	9,216459	9,1158	9,084667	9,31059	9,3145673	9,170352333	-0,144215	0,904871603	Mob1b
17262478	11,92179	11,89033	11,90182	11,77531	11,6988	11,80719	11,904647	11,76043333	-0,14421333	0,904872648	Hnrnpab
17514424	7,191772	7,945899	6,941124	7,048347	7,233289	7,364635	7,3595983	7,215423667	-0,14417467	0,904896901	Casp1
17424077	9,87634	10,00337	9,915409	9,868715	9,66483	9,829182	9,9317063	9,787575667	-0,14413067	0,904924499	B4galt1
17348631	5,623675	5,35323	5,276218	5,273612	5,18882	5,358469	5,4177077	5,273633667	-0,144074	0,904960044	---
17514320	7,363381	7,29253	7,319116	7,175809	7,07209	7,294923	7,325009	7,180940667	-0,14406833	0,904963598	2610044O15Rik
17363470	7,886869	8,222937	7,705256	7,729962	7,980719	7,67218	7,938354	7,794287	-0,144067	0,904964435	Gda
17461471	4,588137	4,755769	4,213851	4,482285	4,294461	4,348873	4,5192523	4,375206333	-0,144046	0,904977607	---
17417824	4,290814	3,600794	3,81909	3,81051	3,923901	3,54424	3,903566	3,759550333	-0,14401567	0,904996635	Olfr1330
17454360	6,066144	6,126871	6,45024	6,138575	5,959108	6,113604	6,2144183	6,070429	-0,14398933	0,905013154	Prkar1b
17405489	5,769576	5,905416	5,60034	5,51183	5,416446	5,915107	5,758444	5,614461	-0,143983	0,905017127	Igsf10
17205297	5,898325	5,602919	5,337834	5,802667	5,096962	5,50752	5,613026	5,469049667	-0,14397633	0,905021309	---
17390844	7,467099	7,901554	7,560484	7,666569	7,274757	7,555949	7,6430457	7,499091667	-0,143954	0,905035319	Myef2
17507922	7,156571	6,805743	6,757334	6,718946	6,719636	6,849205	6,9065493	6,762595667	-0,14395367	0,905035528	---
17507931	7,156571	6,805743	6,757334	6,718946	6,719636	6,849205	6,9065493	6,762595667	-0,14395367	0,905035528	---
17210729	5,99253	6,239002	6,091934	5,992999	5,845261	6,053413	6,107822	5,963891	-0,143931	0,905049748	---
17288354	6,42766	5,495567	5,942037	5,923315	5,835958	5,674318	5,955088	5,811197	-0,143891	0,905074841	BC048507
17298944	3,04156	3,226817	3,029924	3,106279	3,063044	2,697349	3,0994337	2,955557333	-0,14387633	0,905084043	---
17200673	4,252688	3,646091	4,935031	3,571139	4,795557	4,035497	4,2779367	4,134064333	-0,14387233	0,905086552	---
17248213	9,901287	10,10637	9,848666	9,734578	9,666472	10,02369	9,9521077	9,808246667	-0,143861	0,905093662	Cpeb4
17239755	8,657601	9,001373	8,757259	8,580639	8,798857	8,605174	8,805411	8,661556667	-0,14385433	0,905097845	---
17289031	7,19004	7,000252	6,880291	7,048354	6,807867	6,782827	7,0235277	6,879682667	-0,143845	0,9051037	Rps23
17214719	5,081963	4,924701	5,226711	4,763352	5,007129	5,031383	5,0777917	4,933954667	-0,143837	0,905108719	LOC100862182
17385281	4,824443	4,675376	4,890782	4,70435	4,61895	4,635877	4,796867	4,653059	-0,143808	0,905126913	Cacnb4
17266617	2,96652	3,097228	3,01212	2,685686	2,985651	2,97319	3,0252893	2,881509	-0,14378033	0,905144271	---
17208255	3,925049	4,505739	4,308706	4,571731	4,053	3,683601	4,246498	4,102777333	-0,14372067	0,905181706	---
17304012	4,585968	4,293473	4,655914	4,388497	4,265297	4,450424	4,511785	4,368072667	-0,14371233	0,905186935	Kcnma1
17519875	5,199747	5,172027	5,193144	5,098756	5,080141	4,954926	5,188306	5,044607667	-0,14369833	0,905195719	Mei4
17296639	6,051179	5,793362	5,836411	5,786599	5,697105	5,766159	5,8936507	5,749954333	-0,14369633	0,905196974	Gm10408
17397899	3,380198	3,117007	3,199847	3,344246	2,864126	3,057606	3,2323507	3,088659333	-0,14369133	0,905200111	C130079G13Rik
17519734	8,560257	8,76362	8,839684	8,540263	8,656982	8,535288	8,721187	8,577511	-0,143676	0,905209732	Gm19592
17383816	7,104159	6,909805	7,063087	6,693362	7,102409	6,850338	7,0256837	6,882036333	-0,14364733	0,905227719	---
17205139	4,92689	5,121592	5,223315	4,779643	5,094779	4,966525	5,090599	4,946982333	-0,14361667	0,905246961	---
17209329	4,92689	5,121592	5,223315	4,779643	5,094779	4,966525	5,090599	4,946982333	-0,14361667	0,905246961	---
17497924	6,596736	6,558683	6,643408	6,595382	6,369625	6,402991	6,599609	6,455999333	-0,14360967	0,905251353	Lrdd
17284963	6,048982	5,818517	6,23442	5,88906	5,714841	6,067205	6,033973	5,890368667	-0,14360433	0,9052547	Marcksl1-ps4
17322455	6,84678	7,180221	6,967563	6,737586	6,784503	7,041804	6,998188	6,854631	-0,143557	0,905284401	Zfp263
17207545	4,236816	4,189877	3,648661	4,006983	3,867656	3,770067	4,025118	3,881568667	-0,14354933	0,905289211	---
17514867	11,60943	11,93482	11,76102	11,57151	11,58096	11,72222	11,768423	11,62489667	-0,14352667	0,905303435	---
17448082	8,626628	8,563947	8,493619	8,463267	8,418563	8,371796	8,561398	8,417875333	-0,14352267	0,905305945	Sepsecs
17422565	5,951024	6,025066	5,935193	5,729955	5,659205	6,091632	5,9704277	5,826930667	-0,143497	0,905322051	Tmem52
17203541	4,794277	3,381076	4,466212	3,824753	4,17953	4,206878	4,213855	4,070387	-0,143468	0,905340249	---
17423395	4,673018	4,592418	4,986723	4,668845	4,481961	4,67095	4,7507197	4,607252	-0,14346767	0,905340459	Slc26a7
17208979	2,846239	3,270892	3,098664	3,001431	2,877619	2,906405	3,0719317	2,928485	-0,14344667	0,905353637	---
17519667	3,85759	3,667641	4,364465	3,510494	3,683823	4,26511	3,963232	3,819809	-0,143423	0,905368489	---
17281108	7,159734	7,568917	7,186326	7,32129	6,993158	7,170377	7,3049923	7,161608333	-0,143384	0,905392964	Eapp
17269134	4,43479	4,583773	4,710103	4,442812	4,165556	4,690202	4,576222	4,432856667	-0,14336533	0,905404679	---
17315051	5,92194	5,496034	5,830943	5,559289	5,741844	5,517773	5,749639	5,606302	-0,143337	0,90542246	---
17290828	3,590129	3,609703	3,651001	3,337865	3,651848	3,43123	3,6169443	3,473647667	-0,14329667	0,905447773	4930448F12Rik
17507584	7,389676	7,441725	7,411856	7,350032	7,172412	7,290973	7,414419	7,271139	-0,14328	0,905458234	Dcun1d2
17331407	8,318871	8,25503	8,126402	8,058536	8,151686	8,060377	8,2334343	8,090199667	-0,14323467	0,905486686	4930453N24Rik
17548197	5,603415	5,448745	5,424236	5,56054	5,073949	5,412271	5,492132	5,34892	-0,143212	0,905500912	Gm20093
17205817	7,575672	6,934973	6,635011	7,175612	6,33451	7,20592	7,048552	6,905347333	-0,14320467	0,905505515	---
17399981	4,749293	4,687222	5,064243	4,816035	4,527033	4,728099	4,833586	4,690389	-0,143197	0,905510327	Rptn
17359113	6,449384	6,755883	6,853046	7,030855	6,481737	6,116167	6,6861043	6,542919667	-0,14318467	0,905518068	O3far1
17377443	3,577693	3,728928	3,545558	3,473292	3,363702	3,585643	3,617393	3,474212333	-0,14318067	0,905520579	Gm14139
17290846	6,120512	6,513141	6,409524	6,653178	6,038923	5,921608	6,3477257	6,204569667	-0,143156	0,905536061	---
17377199	7,589397	7,964484	7,857749	7,729004	7,661871	7,591337	7,8038767	7,660737333	-0,14313933	0,905546522	Naa20
17442823	7,818355	7,808602	7,947108	7,851005	7,470497	7,82319	7,8580217	7,714897333	-0,14312433	0,905555938	Ran
17361053	5,562508	5,44951	5,572966	5,213894	5,431627	5,510098	5,528328	5,385206333	-0,14312167	0,905557611	Nudt8 // Nudt8
17368760	5,954692	6,120316	6,254291	5,931335	5,986228	5,982457	6,1097663	5,966673333	-0,143093	0,905575605	Mir3088
17264863	10,56537	10,83265	10,78671	10,58328	10,65364	10,51858	10,728243	10,58516667	-0,14307667	0,905585858	---
17477930	6,390171	6,370247	6,533413	6,439218	6,247358	6,178069	6,431277	6,288215	-0,143062	0,905595064	Rasip1
17203499	5,496538	5,807353	6,224164	5,765942	5,925643	5,407422	5,842685	5,699669	-0,143016	0,905623939	---
17514247	8,603437	8,570523	8,70177	8,544336	8,563383	8,339047	8,6252433	8,482255333	-0,142988	0,905641516	Tomm20
17457807	5,43681	5,487321	5,460732	5,345142	5,346498	5,264261	5,461621	5,318633667	-0,14298733	0,905641934	Ephb6
17316274	3,394612	3,078247	3,321781	2,939546	3,047029	3,379126	3,26488	3,121900333	-0,14297967	0,905646747	---
17290924	7,383832	7,589541	7,807457	7,362961	7,174088	7,814886	7,59361	7,450645	-0,142965	0,905655954	---
17358797	9,08425	9,032851	8,749876	8,535583	8,911263	8,991241	8,955659	8,812695667	-0,14296333	0,905657	Fas
17421550	7,274889	7,597486	7,673822	7,302973	7,621738	7,192689	7,515399	7,372466667	-0,14293233	0,905676461	Mthfr
17350138	10,64765	10,93835	10,7739	10,62751	10,59617	10,70744	10,786633	10,64370667	-0,14292667	0,905680018	---
17350295	4,412449	4,214517	4,283834	4,23753	4,326374	3,91814	4,3036	4,160681333	-0,14291867	0,90568504	Mcc
17319494	6,00474	5,850868	5,575008	5,362097	6,015603	5,624161	5,8102053	5,667287	-0,14291833	0,90568525	Chadl
17201823	6,752958	6,481083	7,105589	6,495815	6,531421	6,883754	6,7798767	6,636996667	-0,14288	0,905709315	---
17201999	6,752958	6,481083	7,105589	6,495815	6,531421	6,883754	6,7798767	6,636996667	-0,14288	0,905709315	---
17475214	6,582784	7,13636	6,570187	6,497536	6,150321	7,212911	6,7631103	6,620256	-0,14285433	0,905725428	---
17278543	5,746319	5,513512	5,756352	5,487879	5,493521	5,606343	5,672061	5,529247667	-0,14281333	0,905751168	Cyp46a1
17390410	5,247997	5,220899	5,16887	4,962264	5,310742	4,936361	5,2125887	5,069789	-0,14279967	0,905759749	Tgm7
17239760	3,233192	3,271072	3,040043	2,935128	3,090419	3,090419	3,1814357	3,038655333	-0,14278033	0,905771887	Taar7a
17421170	7,333456	7,85851	7,199667	7,169448	7,370573	7,423451	7,4638777	7,321157333	-0,14272033	0,905809557	Hnrnph2 // Hnrnph2
17257047	5,357649	5,697088	5,162416	5,357037	5,373344	5,058773	5,4057177	5,263051333	-0,14266633	0,905843462	---
17301605	5,849009	5,746732	5,757919	5,706879	5,559729	5,65907	5,7845533	5,641892667	-0,14266067	0,90584702	Nkx2-6
17284636	4,519986	4,627885	4,753701	4,661024	4,355923	4,456659	4,6338573	4,491202	-0,14265533	0,905850369	---
17311602	8,002349	8,128992	7,958495	7,941201	7,798857	7,921813	8,0299453	7,887290333	-0,142655	0,905850578	Mtbp
17227646	6,530092	6,315995	6,90185	6,370961	6,423983	6,52503	6,5826457	6,439991333	-0,14265433	0,905850997	Gm4788
17366185	4,891669	5,354466	5,244917	4,928154	4,887181	5,247801	5,163684	5,021045333	-0,14263867	0,905860834	---
17246154	5,11872	5,199766	5,161165	4,890048	5,108683	5,053067	5,1598837	5,017266	-0,14261767	0,90587402	Myl6b
17494811	3,83918	3,517666	3,541636	3,724808	3,422278	3,323609	3,6328273	3,490231667	-0,14259567	0,905887834	Olfr514
17462756	3,266653	3,477982	3,627556	3,565087	3,204057	3,17529	3,457397	3,314811333	-0,14258567	0,905894113	Clec4b1
17527993	6,935597	6,867321	7,206081	6,971395	6,679239	6,930636	7,0029997	6,860423333	-0,14257633	0,905899974	---
17414380	3,4112	3,067245	3,208673	2,984005	3,078909	3,1965	3,2290393	3,086471333	-0,142568	0,905905206	---
17495836	5,006427	4,892492	5,540006	4,933216	5,142425	4,935817	5,1463083	5,003819333	-0,142489	0,905954814	Mettl9
17332457	4,518806	4,831535	4,660784	4,518835	4,387951	4,676973	4,670375	4,527919667	-0,14245533	0,905975955	Lca5l
17300733	4,603911	4,417457	4,245916	4,27311	4,128849	4,438096	4,422428	4,280018333	-0,14240967	0,906004633	---
17541721	5,955962	5,571368	5,799229	5,74153	5,613965	5,544012	5,7755197	5,633169	-0,14235067	0,906041686	Mir542
17202617	5,870401	6,056215	5,5256	5,845011	5,053573	6,126807	5,8174053	5,675130333	-0,142275	0,906089207	---
17423867	4,821535	4,490349	4,75595	4,524341	4,651312	4,465412	4,689278	4,547021667	-0,14225633	0,906100931	1700003M02Rik
17200589	3,737925	3,668063	4,340415	3,835031	3,65244	3,832259	3,9154677	3,773243333	-0,14222433	0,906121029	---
17404880	4,047624	3,642458	3,959814	3,735146	3,555851	3,932394	3,8832987	3,741130333	-0,14216833	0,906156202	Il2
17368193	5,077971	4,861461	4,897127	4,884775	4,711077	4,814273	4,9455197	4,803375	-0,14214467	0,906171067	Lcn11
17263185	5,566768	5,071432	5,013679	5,236612	4,855745	5,13311	5,217293	5,075155667	-0,14213733	0,906175673	Olfr323
17322254	6,288908	6,161877	6,33422	6,115162	6,139846	6,103706	6,2616683	6,119571333	-0,142097	0,906201008	Npff
17208433	6,603193	6,705028	6,167667	6,38389	6,294391	6,371316	6,4919627	6,349865667	-0,142097	0,906201008	---
17454831	6,170053	6,229295	6,266639	6,30898	5,798667	6,132119	6,2219957	6,079922	-0,14207367	0,906215664	Zfp853
17395079	6,573147	7,87429	5,909074	6,550438	6,510258	6,869643	6,7855037	6,643446333	-0,14205733	0,906225924	Zbp1
17232215	4,127437	4,193434	3,797554	3,855948	4,026451	3,809915	4,039475	3,897438	-0,142037	0,906238696	Moxd1

17548102	9,433769	9,579599	9,132905	8,918683	9,176118	9,62542	9,382091	9,240073667	-0,14201733	0,90625105	Cycs // Cycs
17548541	9,433769	9,579599	9,132905	8,918683	9,176118	9,62542	9,382091	9,240073667	-0,14201733	0,90625105	Cycs
17541322	4,706297	4,545946	4,817044	4,629005	4,385247	4,629005	4,6897623	4,547752333	-0,14201	0,906255657	Dcaf12l2
17227795	2,984045	2,795071	2,808834	2,674052	2,741736	2,746164	2,86265	2,720650667	-0,14199933	0,906262357	---
17307463	8,41819	8,301988	8,305193	8,236355	8,156956	8,206097	8,3417903	8,199802667	-0,14198767	0,906269686	Ebpl
17306816	7,065484	7,688092	7,249715	7,166101	7,086758	7,324481	7,3344303	7,192446667	-0,14198367	0,906272198	Tgm1
17365167	5,003071	4,977742	5,319993	4,954888	4,933729	4,986244	5,1002687	4,958287	-0,14198167	0,906273455	Pdzd7
17476695	5,163321	4,813631	4,963714	4,906672	4,854772	4,753315	4,980222	4,838253	-0,141969	0,906281412	---
17424161	3,058873	3,005027	3,127795	3,048976	2,965267	2,751547	3,0638983	2,92193	-0,14196833	0,906281831	---
17203467	5,360586	4,652576	3,843465	4,291693	4,356588	4,782509	4,6188757	4,47693	-0,14194567	0,90629607	---
17548854	3,490751	3,616694	3,922773	3,547695	3,705754	3,350935	3,6767393	3,534794667	-0,14194467	0,906296698	---
17406182	4,638569	4,954704	4,767019	4,628475	4,65927	4,646715	4,786764	4,64482	-0,141944	0,906297117	---
17300370	10,00235	10,07598	10,21999	9,897323	9,848469	10,1267	10,09944	9,957497333	-0,14194267	0,906297954	---
17543262	3,695792	3,699425	3,651848	3,228394	3,734729	3,658163	3,682355	3,540428667	-0,14192633	0,906308215	---
17499800	4,628652	4,188518	4,287852	4,458108	4,272225	3,948915	4,3683407	4,226416	-0,14192467	0,906309262	Defa-ps1
17347619	5,56174	5,847224	5,458873	5,55281	4,982644	5,906613	5,6226123	5,480689	-0,14192333	0,906310099	Slc8a1
17449052	5,831767	6,286383	6,700452	6,368675	5,999157	6,025053	6,2728673	6,130961667	-0,14190567	0,906321198	Hopx
17281826	4,984091	4,84232	5,031281	4,803745	4,815005	4,813313	4,952564	4,810687667	-0,14187633	0,906339626	Rtn1
17437111	5,238746	4,965843	5,334924	5,075753	4,921829	5,11642	5,1798377	5,038000667	-0,141837	0,906364336	4930487D11Rik // 4930487D11Rik
17486299	5,42992	5,522463	5,000945	5,369847	4,959737	5,198242	5,317776	5,175942	-0,141834	0,906366221	Vmn2r45
17515641	4,349605	4,522851	4,364317	4,239781	4,30161	4,269946	4,4122577	4,270445667	-0,141812	0,906380042	AI414108
17304493	9,623322	9,927868	9,682559	9,582221	9,66922	9,55689	9,744583	9,602777	-0,141806	0,906383812	Gnl3
17474863	3,99075	3,808265	3,838835	3,855107	3,710859	3,646501	3,8792833	3,737489	-0,14179433	0,906391142	Vmn1r174
17448577	5,269177	5,018591	5,056846	4,980722	4,868684	5,069883	5,1148713	4,973096333	-0,141775	0,906403288	Gabra2
17532692	6,399283	6,350913	6,158298	6,436462	5,798311	6,248425	6,3028313	6,161066	-0,14176533	0,906409361	---
17527581	6,832277	6,52009	6,258957	6,28017	6,087507	6,818401	6,537108	6,395359333	-0,14174867	0,906419833	Cyp1a2
17213945	3,721563	3,765708	3,683721	3,638012	3,535645	3,572356	3,723664	3,582004333	-0,14165967	0,906475752	---
17368292	6,724187	6,320001	6,481335	6,280302	6,359931	6,460323	6,5085077	6,366852	-0,14165567	0,906478265	Sdccag3
17549040	4,738028	4,413483	4,661836	4,658197	4,507086	4,223296	4,604449	4,462859667	-0,14158933	0,906519945	---
17549046	4,738028	4,413483	4,661836	4,658197	4,507086	4,223296	4,604449	4,462859667	-0,14158933	0,906519945	---
17549052	4,738028	4,413483	4,661836	4,658197	4,507086	4,223296	4,604449	4,462859667	-0,14158933	0,906519945	---
17549058	4,738028	4,413483	4,661836	4,658197	4,507086	4,223296	4,604449	4,462859667	-0,14158933	0,906519945	---
17549064	4,738028	4,413483	4,661836	4,658197	4,507086	4,223296	4,604449	4,462859667	-0,14158933	0,906519945	---
17549074	4,738028	4,413483	4,661836	4,658197	4,507086	4,223296	4,604449	4,462859667	-0,14158933	0,906519945	---
17549142	4,738028	4,413483	4,661836	4,658197	4,507086	4,223296	4,604449	4,462859667	-0,14158933	0,906519945	---
17549146	4,738028	4,413483	4,661836	4,658197	4,507086	4,223296	4,604449	4,462859667	-0,14158933	0,906519945	---
17549154	4,738028	4,413483	4,661836	4,658197	4,507086	4,223296	4,604449	4,462859667	-0,14158933	0,906519945	---
17233632	10,16403	10,03307	10,413	10,19717	9,916245	10,07192	10,203367	10,06177833	-0,14158833	0,906520573	---
17499765	4,86797	4,717489	4,747144	4,528605	4,631596	4,747706	4,7775343	4,635969	-0,14156533	0,906535025	Gm14850
17341708	2,984045	3,053003	4,067469	3,859212	2,487218	3,333494	3,3681723	3,226641333	-0,141531	0,906556599	---
17440726	5,837072	6,122014	5,977795	5,907976	5,755049	5,849267	5,9789603	5,837430667	-0,14152967	0,906557437	---
17255949	6,070317	5,734661	6,045026	5,923963	5,759619	5,741914	5,9500013	5,808498667	-0,14150267	0,906574403	Ppp1r1b
17474880	7,534777	7,824987	7,75676	7,637991	7,605396	7,448641	7,705508	7,564009333	-0,14149867	0,906576917	Zfp180
17323198	3,824177	3,544459	3,625224	3,6801	3,460754	3,428516	3,66462	3,523123333	-0,14149667	0,906578174	Efcab1
17304901	4,662478	4,613382	4,975949	4,501163	4,862315	4,463851	4,750603	4,609109667	-0,14149333	0,906580268	3425401B19Rik
17451186	4,370196	4,397892	4,276577	4,262161	4,111807	4,246243	4,3482217	4,206737	-0,14148467	0,906585715	Miat
17379873	9,640278	10,02739	9,870636	9,682674	9,744949	9,686247	9,8461013	9,704623333	-0,141478	0,906589904	---
17550126	7,035571	7,031623	6,531565	6,942039	6,307243	6,925071	6,866253	6,724784333	-0,14146867	0,906595769	---
17260885	8,289321	8,28441	8,390551	8,195664	8,263569	8,080667	8,3214273	8,179966667	-0,14146067	0,906600796	Aftph
17284654	4,177482	4,255931	3,957501	4,42283	3,75331	3,790435	4,1303047	3,988858333	-0,14144633	0,906609803	---
17401696	4,107479	3,782585	4,030646	3,660436	3,972857	3,863185	3,97357	3,832159333	-0,14141067	0,906632217	---
17320711	9,573913	9,854678	9,752603	9,665916	9,42412	9,666952	9,7270647	9,585662667	-0,141402	0,906637664	Zcrb1
17343170	4,641388	5,013722	5,321764	5,101446	4,6499	4,801346	4,9922913	4,850897333	-0,141394	0,906642691	Tff2
17418757	4,219604	4,140867	4,053782	3,955553	4,036065	3,998454	4,1380843	3,996690667	-0,14139367	0,9066429	1700080G11Rik
17353786	4,305211	4,359907	4,392622	4,243683	4,038389	4,351511	4,35258	4,211194333	-0,14138567	0,906647928	Gm10544
17358721	6,709608	6,875521	7,204557	6,937872	6,721846	6,705812	6,9298953	6,78851	-0,14138533	0,906648137	---
17203265	5,761102	4,910893	5,535262	5,129775	5,039023	5,614388	5,402419	5,261062	-0,141357	0,906665943	---
17491730	4,409022	4,156069	4,446722	4,284634	4,02879	4,274322	4,337271	4,195915333	-0,14135567	0,906666781	A330076H08Rik
17299776	3,404632	3,150537	3,130673	3,028784	3,129202	3,103797	3,228614	3,087261	-0,141353	0,906668457	---
17548538	4,987889	4,82963	4,895667	4,778031	4,667124	4,844013	4,9043953	4,763056	-0,14133933	0,906677046	Cpa2 // Cpa2
17506923	6,376998	5,886106	6,36103	6,189172	6,057147	5,953846	6,2080447	6,066721667	-0,141323	0,906687311	Slc35f3
17277978	4,322382	4,150099	4,336967	3,972124	4,101348	4,312062	4,269816	4,128511333	-0,14130467	0,906698833	Catsperb
17375927	5,887534	5,051665	5,51441	5,741501	5,383556	4,904642	5,4845363	5,343233	-0,14130333	0,906699671	---
17298825	7,665059	7,954091	7,674592	7,592424	7,680883	7,596541	7,7645807	7,623282667	-0,141298	0,906703023	Mmrn2
17309899	6,722198	6,916792	6,95585	6,611731	6,690901	6,868327	6,8649467	6,723653	-0,14129367	0,906705746	Rpl37
17339340	7,315307	7,206779	7,433067	7,1272	7,185271	7,218872	7,3183843	7,177114333	-0,14127	0,906720621	Zfp161
17326506	3,803889	3,817625	3,781856	3,647728	3,706761	3,625318	3,8011233	3,659935667	-0,14118767	0,906772368	Stx19
17489499	3,352946	3,280013	3,150221	3,068467	3,060179	3,231006	3,26106	3,119884	-0,141176	0,906779701	Gm19607
17496839	6,546472	6,750714	6,932896	6,421433	6,792119	6,593049	6,7433607	6,602200333	-0,14116033	0,906789548	Pycard
17315087	4,671245	5,001405	4,687109	4,648865	4,732164	4,555258	4,7865863	4,645429	-0,14115733	0,906791433	Scn8a
17488485	7,879079	7,809194	8,099748	7,580621	8,041077	7,742921	7,9293403	7,788206333	-0,141134	0,906806099	Il28a
17320974	6,896739	7,078358	7,120718	6,681311	7,060164	6,930956	7,0319383	6,890810333	-0,141128	0,906809871	Rpap3
17524895	5,695582	5,5844	5,627821	5,515192	5,448411	5,520928	5,6359343	5,494843667	-0,14109067	0,906833337	Elavl3
17248125	3,370322	3,604861	3,636412	3,548582	3,461236	3,17858	3,5371983	3,396132667	-0,14106567	0,906849051	---
17537046	4,741516	4,237242	3,953387	4,052951	4,338699	4,117381	4,310715	4,169677	-0,141038	0,906866442	---
17462244	5,12981	5,275415	5,163306	5,0032	4,983637	5,158607	5,1895103	5,048481333	-0,141029	0,906872099	Cacna2d4
17307685	4,45583	4,582025	4,420581	4,274168	4,258821	4,502434	4,4861453	4,345141	-0,14100433	0,906887605	4930471C04Rik
17548514	5,471177	5,527586	5,260373	4,98946	5,531187	5,515606	5,4863787	5,345417667	-0,140961	0,906914845	Suclg2 // Suclg2
17322541	6,405443	6,478741	6,503407	6,276966	6,502561	6,185188	6,4625303	6,321571667	-0,14095867	0,906916312	Dnaja3
17421150	6,565486	6,750497	6,739293	6,252764	6,780672	6,598979	6,685092	6,544145	-0,140947	0,906923646	4930455G09Rik
17284596	3,010198	3,184482	3,120717	3,119358	2,915927	2,85736	3,1051323	2,964215	-0,14091733	0,906942295	---
17200467	6,839497	6,836103	6,879673	6,668831	6,819515	6,644217	6,8517577	6,710854333	-0,14090333	0,906951096	---
17343710	7,946108	8,050857	7,211722	7,51892	7,938144	7,328981	7,736229	7,595348333	-0,14088067	0,906965346	H2-K1
17285530	3,204818	3,112569	2,973151	2,95356	3,018262	2,896142	3,096846	2,955988	-0,140858	0,906979596	---
17495454	4,316888	3,570703	4,300071	3,943014	4,132113	3,68999	4,062554	3,921705667	-0,14084833	0,906985673	---
17548914	5,992687	6,101498	6,2608	5,984782	6,068612	5,879075	6,1183283	5,977489667	-0,14083867	0,90699175	Akr1cl // Akr1cl // Akr1cl // Akr1cl
17418217	4,308971	4,215517	4,394407	4,096812	4,286826	4,112982	4,3062983	4,16554	-0,14075833	0,907042255	---
17318256	6,481812	6,390711	6,54199	6,300224	6,393897	6,298137	6,4715043	6,330752667	-0,14075167	0,907046447	Scrib
17491660	3,582837	3,847589	3,624838	3,201664	3,704651	3,72679	3,685088	3,544368333	-0,14071967	0,907066566	A230006K03Rik
17297692	7,201383	7,310262	6,9295	7,205405	6,793873	7,019732	7,1470483	7,006336667	-0,14071167	0,907071596	Rps24
17287969	5,317307	5,253242	5,242336	4,921254	5,020127	5,449498	5,2709617	5,130293	-0,14066867	0,907098632	Etohd2
17282129	6,318234	6,213541	6,109176	5,864216	6,346176	6,008592	6,2136503	6,072994667	-0,14065567	0,907106806	Tmem229b
17340646	4,819108	4,379822	4,772604	4,763741	4,72997	4,055886	4,657178	4,516532333	-0,14064567	0,907113093	Tagap1
17283683	5,164009	5,098598	3,9433	4,588344	4,80392	4,391868	4,7353023	4,594710667	-0,14059167	0,907147047	---
17229984	7,046453	6,901507	6,98	6,700029	6,793081	7,013097	6,9759867	6,835402333	-0,14058433	0,907151658	Darc
17290655	4,992271	5,274977	5,114058	4,955518	4,740473	5,263577	5,127102	4,986522667	-0,14057933	0,907154802	Gpr137b-ps
17368786	6,757658	6,791691	6,637866	6,772392	6,34023	6,652871	6,7290717	6,588497667	-0,140574	0,907158156	Ttf1
17341695	10,63918	10,68908	10,66398	10,43526	10,44903	10,68641	10,66408	10,52356667	-0,14051333	0,907196303	---
17511069	5,815398	5,314971	5,397666	5,409214	5,444602	5,252867	5,509345	5,368894333	-0,14045067	0,90723571	Nanos3
17315255	10,82446	11,04355	10,99132	10,82481	10,84084	10,77246	10,95311	10,81270333	-0,14040667	0,90726338	Eif4b
17474258	5,435413	4,779003	4,977877	4,8456	4,737844	5,187644	5,0640977	4,923696	-0,14040167	0,907266524	Psg29
17462863	9,74133	9,924705	9,734891	9,756421	9,63295	9,590423	9,8003087	9,659931333	-0,14037733	0,907281827	C1rl
17426062	6,34632	5,848459	5,621916	5,865199	5,604383	5,926061	5,9388983	5,798547667	-0,14035067	0,907298597	Mup7
17548232	4,67046	4,356823	4,65525	4,384575	4,364814	4,512137	4,5608443	4,420508667	-0,14033567	0,907308031	Gm10724
17200995	6,635383	6,838416	6,220437	6,111316	6,378537	6,783428	6,5647453	6,424427	-0,14031833	0,907318932	---
17278826	6,95358	6,798839	7,16906	6,820087	6,913242	6,767223	6,9738263	6,833517333	-0,140309	0,907324801	Mir666
17205853	3,006026	4,004177	3,430348	2,846157	3,239383	3,934311	3,4801837	3,339950333	-0,14023333	0,90737239	---
17297817	4,847848	4,640532	4,748777	4,737168	4,459988	4,619313	4,745719	4,605489667	-0,14022933	0,907374906	Gm10394
17289037	8,784706	8,773734	8,590219	8,552619	8,526665	8,648708	8,7162197	8,575997333	-0,14022233	0,907379309	Ssbp2
17520043	7,342583	7,506316	7,343591	7,150347	7,238207	7,383334	7,3974967	7,257296	-0,14020067	0,907392936	Cyb5r4
17548296	3,59028	3,863003	3,648017	3,532767	3,499334	3,648628	3,7004333	3,560243	-0,14019033	0,907399435	LOC100503320
17202137	4,223654	3,981475	3,672763	4,073734	3,395165	3,988456	3,9592973	3,819118333	-0,140179	0,907406563	---
17299487	3,78775	3,446294	3,811766	3,560659	3,556852	3,507778	3,6819367	3,541763	-0,14017367	0,907409918	Olfr740
17283940	9,02108	9,276824	9,38731	9,086357	9,147541	9,030799	9,2284047	9,088232333	-0,14017233	0,907410756	---
17214009	3,955452	4,02258	4,033785	4,022252	3,924106	3,644971	4,003939	3,863776333	-0,14016267	0,907416837	Apol7d
17442995	6,65661	6,436762	6,706754	6,477993	6,384995	6,516695	6,600042	6,459894333	-0,14014767	0,907426271	Kctd7
17437611	8,561291	8,842464	8,438325	8,569446	8,458805	8,393391	8,6140267	8,473880667	-0,140146	0,907427319	Pgm1
17359002	5,081954	5,081954	5,211548	4,806314	4,979571	5,169169	5,125152	4,985018	-0,140134	0,907434867	A330032B11Rik
17483383	7,092034	7,8822	7,159353	7,282712	7,27668	7,153799	7,3778623	7,237730333	-0,140132	0,907436125	Snora30
17353765	9,796877	10,02084	9,850753	9,800001	9,758234	9,689925	9,88949	9,749386667	-0,14010333	0,907454156	Hars
17530703	6,021821	6,154747	5,92397	5,783753	5,886823	6,009665	6,0335127	5,893413667	-0,140099	0,907456882	Parp3
17534130	3,873079	3,611046	3,469367	3,449961	3,571037	3,51223	3,651164	3,511076	-0,140088	0,907463801	Gm15008
17202039	4,582129	4,127097	4,879908	4,581881	4,471931	4,115066	4,5297113	4,389626	-0,14008533	0,907465478	---
17434933	8,598609	8,799141	8,62128	8,675401	8,562475	8,360913	8,67301	8,532929667	-0,14008033	0,907468623	Hgf
17239266	4,816246	4,131818	4,893329	4,395103	4,36194	4,664194	4,6137977	4,473745667	-0,140052	0,907486446	---
17535900	3,367236	3,144522	3,329064	3,103259	3,114134	3,203358	3,280274	3,140250333	-0,14002367	0,907504268	---
17408507	6,392712	6,404782	6,356344	6,286268	6,194062	6,253483	6,3846127	6,244604333	-0,14000833	0,907513913	---
17244126	8,073801	7,85409	7,562195	7,53826	7,821637	7,71026	7,8300287	7,690052333	-0,13997633	0,907534043	Actr6
17245422	6,760181	6,8752	6,831993	6,5044	6,668174	6,874952	6,822458	6,682508667	-0,13994933	0,907551027	Hmga2
17374304	6,302144	6,453328	6,404099	6,31034	6,237057	6,192369	6,3865237	6,246588667	-0,139935	0,907560044	Aven
17343682	9,54565	10,10558	10,02863	9,803038	9,700407	9,756628	9,8932867	9,753357667	-0,139929	0,907563819	---
17536931	6,627054	6,595272	6,789247	6,610395	6,559357	6,422063	6,6705243	6,530605	-0,13991933	0,9075699	---
17536943	6,627054	6,595272	6,789247	6,610395	6,559357	6,422063	6,6705243	6,530605	-0,13991933	0,9075699	---
17369235	8,870304	8,847478	9,021409	8,849031	8,677987	8,792495	8,9130637	8,773171	-0,13989267	0,907586675	Ppp2r4

17457601	4,057971	4,027343	3,88578	3,896001	3,762634	3,892803	3,9903647	3,850479333	-0,13988533	0,907591289	Tas2r137
17383522	5,602774	6,045928	6,136926	5,792015	5,992496	5,581525	5,9285427	5,788678667	-0,139864	0,907604709	Cercam
17430521	7,304358	6,998428	7,220934	6,877728	7,111916	7,114527	7,1745733	7,034723667	-0,13984967	0,907613727	Tmem39b
17548669	6,465199	6,440203	6,353886	6,377127	6,18705	6,275618	6,4197627	6,279931667	-0,139831	0,90762547	Tmem66 // Tmem66
17535989	4,633289	4,508217	4,714489	4,350003	4,649252	4,437278	4,618665	4,478844333	-0,13982067	0,907631971	---
17241053	4,654138	4,457069	4,597705	4,388815	4,419875	4,480774	4,5696373	4,429821333	-0,139816	0,907634907	Cdh23
17305291	7,209249	6,711333	6,896133	6,750536	6,788173	6,858574	6,938905	6,799094333	-0,13981067	0,907638262	---
17506720	8,589329	8,766278	8,538323	8,37326	8,631736	8,469564	8,63131	8,49152	-0,13979	0,907651264	Urb2
17317214	6,950479	7,696352	7,233011	6,678436	7,508037	7,274027	7,2932807	7,1535	-0,13978067	0,907657136	9130401M01Rik
17469684	7,022776	7,047113	7,053821	6,955036	6,958797	6,790559	7,0412367	6,901464	-0,13977267	0,907662169	Gt(ROSA)26Sor
17535972	8,360742	8,50025	8,147042	8,151531	8,188014	8,249188	8,3360113	8,196244333	-0,139767	0,907665734	Vbp1
17203509	4,089198	4,515646	4,542193	4,463582	4,391567	3,872649	4,3823457	4,242599333	-0,13974633	0,907678737	---
17202073	3,331443	4,404907	2,850187	3,226817	3,236017	3,704504	3,5288457	3,389112667	-0,139733	0,907687126	---
17210127	3,779106	3,190543	4,6204	3,71436	3,441147	4,015391	3,8633497	3,723632667	-0,139717	0,907697192	---
17371739	9,709225	9,693785	9,719026	9,499147	9,638702	9,565095	9,7073453	9,567648	-0,13969733	0,907709566	Hat1
17526114	4,072118	4,00828	3,91823	3,45961	4,354923	3,765046	3,9995427	3,859859667	-0,139683	0,907718584	Rnf26
17359874	5,715432	5,779998	5,851361	5,635242	5,590539	5,702033	5,7822637	5,642604667	-0,139659	0,907733685	Hps6
17202027	3,503079	3,235439	3,768309	3,435615	3,546623	3,105647	3,5022757	3,362628333	-0,13964733	0,907741025	---
17510867	7,916388	7,638511	7,687934	7,771393	7,54669	7,505825	7,747611	7,607969333	-0,13964167	0,907744591	Zfp330
17229417	8,201166	8,15556	8,109845	8,086434	7,914659	8,046567	8,1555237	8,015886667	-0,139637	0,907747527	Pbx1
17417736	3,674452	3,32721	3,848569	3,224984	3,645198	3,561182	3,6167437	3,477121333	-0,13962233	0,907756755	---
17209687	2,981277	3,459479	2,879339	3,189692	2,85296	2,858579	3,1066983	2,967077	-0,13962133	0,907757385	---
17406960	7,703709	7,524244	7,811601	7,694832	7,356331	7,569571	7,6798513	7,540244667	-0,13960667	0,907766613	Mir92b
17400449	8,930986	9,050362	8,942348	8,80435	8,868582	8,832006	8,9745653	8,834979333	-0,139586	0,907779617	Anp32e
17547767	9,377557	9,592046	9,428983	9,26177	9,297968	9,420188	9,4661953	9,326642	-0,13955333	0,907800172	Hnrnpf // Hnrnpf
17323616	5,772424	5,447266	5,327904	5,445636	5,457374	5,225949	5,5158647	5,376319667	-0,139545	0,907805416	B830017H08Rik
17296969	6,776364	6,592323	6,265965	6,522219	6,336903	6,356912	6,544884	6,405344667	-0,13953933	0,907808981	---
17328419	6,306034	6,101379	6,32974	6,152343	6,129169	6,037057	6,2457177	6,106189667	-0,139528	0,907816113	---
17203807	4,610855	3,826293	3,832037	4,532403	3,726194	3,592041	4,0897283	3,950212667	-0,13951567	0,907823874	---
17537013	3,102768	3,146421	3,091746	2,944614	2,918217	3,059619	3,113645	2,97415	-0,139495	0,907836878	---
17347210	9,416218	9,399584	9,311838	9,130177	9,277525	9,301455	9,37588	9,236385667	-0,13949433	0,907837298	Memo1
17368658	5,473745	5,538236	5,521145	5,287048	5,480937	5,34672	5,511042	5,371568333	-0,13947367	0,907850303	Ppp1r26
17523695	9,792328	9,554756	9,531821	9,430531	9,514919	9,515041	9,6263017	9,486830333	-0,13947133	0,907851771	Aasdhppt
17210137	3,401015	3,666763	3,224403	3,482794	3,063954	3,327058	3,430727	3,291268667	-0,13945833	0,907859952	---
17546792	3,718787	3,47318	3,504054	3,373892	3,707322	3,196492	3,5653403	3,425902	-0,13943833	0,907872537	Tspy-ps
17281924	3,989551	4,474377	4,281701	4,050651	4,010697	4,266027	4,248543	4,109125	-0,139418	0,907885333	---
17206317	4,901153	3,947714	3,510574	3,630381	3,778832	4,531985	4,1198137	3,980399333	-0,13941433	0,90788764	---
17216620	6,578477	6,503308	6,144723	5,994603	6,316126	6,497543	6,408836	6,269424	-0,139412	0,907889109	Tfcp2l1
17471819	4,544175	3,84991	3,865841	3,910119	4,035946	3,895672	4,086642	3,947245667	-0,13939633	0,907898968	Gm5581 // Gm5581
17363663	5,961136	6,041945	5,854097	5,820356	5,893977	5,724711	5,9523927	5,813014667	-0,139378	0,907910505	Snora19
17366118	5,961136	6,041945	5,854097	5,820356	5,893977	5,724711	5,9523927	5,813014667	-0,139378	0,907910505	Snora19
17265386	5,169752	5,335353	4,704431	4,672138	4,956134	5,163137	5,0698453	4,930469667	-0,13937567	0,907911974	A430084P05Rik
17237284	8,94984	9,10125	8,931833	8,766485	8,913871	8,884562	8,9943077	8,854972667	-0,139335	0,907937566	Krr1
17217497	5,572433	5,374922	4,812981	5,112493	4,791412	5,438458	5,2534453	5,114121	-0,13932433	0,907944279	LOC100503055
17345306	5,619979	5,859642	6,161745	5,80931	5,698457	5,715634	5,8804553	5,741133667	-0,13932167	0,907945957	Rsph9
17354432	4,440938	4,072478	4,234681	4,135042	3,980078	4,215028	4,2493657	4,110049333	-0,13931633	0,907949314	---
17542090	3,490626	3,369373	3,516679	3,254657	3,402883	3,301203	3,4588927	3,319581	-0,13931167	0,907952251	Mir465b-1
17542094	3,490626	3,369373	3,516679	3,254657	3,402883	3,301203	3,4588927	3,319581	-0,13931167	0,907952251	Mir465b-1
17240899	2,880542	2,820959	2,994065	2,711121	2,894823	2,671741	2,898522	2,759228333	-0,13929367	0,907963579	---
17234494	4,60163	4,538574	4,709071	4,488602	4,379181	4,563676	4,616425	4,477153	-0,139272	0,907977215	S100b
17253450	5,784219	5,950218	5,533505	5,445297	6,041953	5,362906	5,7559807	5,616718667	-0,139262	0,907983509	Mir451
17201623	3,091242	2,893808	3,052956	3,019681	2,611503	2,989087	3,0126687	2,873423667	-0,139245	0,907994208	---
17207913	5,971392	5,443609	5,541299	5,591872	5,592606	5,354166	5,6521	5,512881333	-0,13921867	0,908010782	---
17361320	5,430805	5,394995	5,646039	5,509489	5,201878	5,342825	5,490613	5,351397333	-0,13921567	0,90801267	Gm960
17490628	6,206958	6,444858	6,36487	6,27039	6,214901	6,113799	6,3388953	6,199696667	-0,13919867	0,90802337	Flt3l // Flt3l
17210817	6,680112	6,246572	6,668549	6,571742	6,14125	6,464757	6,5317443	6,392583	-0,13916133	0,908046867	---
17280897	8,673215	8,635024	8,418445	8,331649	8,553698	8,423897	8,5755613	8,436414667	-0,13914667	0,908056099	Stxbp6
17421853	5,782119	5,644551	5,818474	5,83163	5,529139	5,466936	5,7483813	5,609235	-0,13914633	0,908056308	---
17474878	4,273385	4,005426	3,931798	3,88483	4,00883	3,899649	4,070203	3,931103	-0,1391	0,908085472	Vmn1r183
17550294	3,346105	3,390606	3,27507	3,229498	3,282919	3,082103	3,3372603	3,198173333	-0,139087	0,908093655	---
17435725	3,697601	3,944642	4,076745	3,872977	3,743644	3,685256	3,9063293	3,767292333	-0,139037	0,908125127	Il6
17343289	5,7889	5,79756	6,215096	5,777132	5,607996	5,999344	5,933852	5,794824	-0,139028	0,908130792	Pdxk-ps
17493880	7,029858	6,965675	6,950397	6,798198	6,97924	6,751465	6,9819767	6,842967667	-0,139009	0,908142752	Atg16l2
17541697	3,252108	3,283481	3,007094	3,125492	3,05485	2,945386	3,1808943	3,041909333	-0,138985	0,90815786	Mir92-2
17321434	5,469945	5,72189	5,512408	5,542557	5,23792	5,50687	5,568081	5,429115667	-0,13896533	0,90817024	Dhh
17317223	6,030605	6,295912	6,180889	6,114356	5,982723	5,993499	6,1691353	6,030192667	-0,13894267	0,908184509	---
17288908	8,356261	8,606764	8,489367	8,231806	8,263308	8,540535	8,4841307	8,345216333	-0,13891433	0,908202345	Cetn3
17201557	2,993617	3,000335	3,131196	2,648301	3,128618	2,931503	3,041716	2,902807333	-0,13890867	0,908205912	---
17290795	4,139063	3,994832	3,9025	3,861499	3,905813	3,852408	4,0121317	3,87324	-0,13889167	0,908216614	---
17407531	5,588374	5,312596	5,56245	5,699923	5,101863	5,245049	5,4878067	5,348945	-0,13886167	0,9082355	Gm10696
17370206	5,625202	5,819667	5,736595	5,524338	5,71049	5,530112	5,7271547	5,588313333	-0,13884133	0,908248301	---
17338416	6,440834	6,160197	6,236022	6,108689	6,383564	5,928286	6,2790177	6,140179667	-0,138838	0,908250399	Trem2
17536060	4,068079	4,128194	4,177437	4,172036	3,865337	3,919858	4,12457	3,985743667	-0,13882633	0,908257744	4930480E11Rik
17214865	3,915051	4,006645	3,866539	3,703747	3,863552	3,804461	3,9294117	3,790586667	-0,138825	0,908258583	Wdr69
17350966	5,488038	5,599483	5,407072	5,400569	5,237806	5,439799	5,4981977	5,359391333	-0,13880633	0,908270335	G630071F17
17486048	3,386055	3,123812	3,086394	2,912305	3,021132	3,24641	3,1987537	3,059949	-0,13880467	0,908271385	Vmn2r28
17253307	8,166697	8,474019	8,243299	8,122019	8,189855	8,155781	8,2946717	8,155885	-0,13878667	0,908282717	Nufip2
17301428	8,659564	8,52717	8,488539	8,323457	8,382468	8,553024	8,5584243	8,419649667	-0,13877467	0,908290272	Pbk
17200221	5,773304	6,352797	5,934042	5,70812	5,890779	6,044969	6,0200477	5,881289333	-0,13875833	0,908300555	---
17444884	5,784742	5,933813	5,586465	5,587817	5,632525	5,668462	5,76834	5,629601333	-0,13873867	0,908312937	Mtus2
17208881	6,267418	5,914115	6,045943	5,827103	5,826046	6,158116	6,0758253	5,937088333	-0,138737	0,908313986	---
17387806	3,315003	3,340521	3,289939	3,119686	3,25243	3,157176	3,3151543	3,176430667	-0,13872367	0,908322381	Olfr1164
17285516	4,409014	4,46742	4,533915	4,439203	4,292732	4,262284	4,4701163	4,331406333	-0,13871	0,908330985	Tcrg-V6
17334474	7,089794	7,300548	7,292849	6,959017	7,15277	7,155287	7,2277303	7,089024667	-0,13870567	0,908333714	Spsb3
17330056	2,845315	2,991009	2,894374	2,830811	2,840988	2,6429	2,9102327	2,771566333	-0,13866633	0,908358479	---
17434655	5,39171	5,431782	5,469236	5,145816	5,355627	5,37538	5,4309093	5,292274333	-0,138635	0,908378207	---
17514926	5,408976	5,35076	5,54056	5,508669	5,315003	5,060743	5,433432	5,294805	-0,138627	0,908383244	---
17213892	4,006672	3,600396	4,349736	3,767348	3,894215	3,879438	3,9856013	3,847000333	-0,138601	0,908399615	---
17495412	7,496729	7,661072	7,839722	7,558108	7,634228	7,389443	7,665841	7,527259667	-0,13858133	0,908411998	Snord14a // Snord14a
17531932	9,078209	8,815196	8,755882	8,777349	8,708986	8,747209	8,8830957	8,744514667	-0,138581	0,908412208	Cmtm8
17480205	7,532976	7,39509	7,208388	7,425615	7,266973	7,028219	7,378818	7,240269	-0,138549	0,908432358	Rab30
17548863	3,930653	3,939132	4,037923	3,723637	3,949727	3,818711	3,969236	3,830691667	-0,13854433	0,908435296	Fundc2 // Fundc2
17291190	4,949654	5,120682	5,140481	4,894207	4,913863	4,987117	5,0702723	4,931729	-0,13854333	0,908435926	Hist1h2bn // Hist1h2bl // Hist1h2bj // Hi
17550496	4,314329	4,14054	3,986661	4,011096	3,892075	4,122752	4,1471767	4,008641	-0,13853567	0,908440754	---
17360890	7,377367	7,620497	7,197382	7,121491	7,403172	7,255024	7,3984153	7,259895667	-0,13851967	0,908450829	Grk5
17204925	6,257184	5,927072	6,026889	6,065866	6,083816	5,645911	6,0703817	5,931864333	-0,13851733	0,908452298	---
17299485	4,145613	4,102432	3,734642	3,952802	3,550859	4,063514	3,994229	3,855725	-0,138504	0,908460694	Olfr739
17468432	3,394962	3,367288	3,402353	3,233167	3,241421	3,274513	3,388201	3,249700333	-0,13850067	0,908462793	Cml5
17350447	9,56957	9,601764	9,515036	9,444654	9,37784	9,44838	9,5621233	9,423624667	-0,13849867	0,908464052	Dmxl1
17499387	7,112592	7,311732	7,221862	6,806959	7,205606	7,218253	7,2153953	7,076939333	-0,138456	0,90849092	Zfp828
17534877	4,397437	4,308872	4,19985	3,659304	4,506481	4,325009	4,302053	4,163598	-0,138455	0,908491549	3830403N18Rik
17539479	6,531119	6,493962	6,56601	6,416622	6,457253	6,302053	6,5303637	6,391976	-0,13838767	0,908533951	Tmem27
17429600	3,719652	3,50651	3,183674	3,229932	3,47296	3,291787	3,4699453	3,331559667	-0,13838567	0,908535211	Gm12887
17239693	6,494766	6,160142	6,385496	6,330954	6,134864	6,159491	6,3468013	6,208436333	-0,138365	0,908548226	---
17437479	4,543672	4,614367	4,487963	4,843136	4,105719	4,282153	4,5486673	4,410336	-0,13833133	0,908569428	---
17231431	6,573019	6,575744	6,473901	6,33868	6,220199	6,64897	6,540888	6,402616333	-0,13827167	0,908607005	1700052N19Rik
17355189	9,006994	9,109745	9,036425	8,853042	8,863394	9,021944	9,0510547	8,912793333	-0,13826133	0,908613513	Spire1
17350673	9,928466	9,308726	10,06549	8,966529	9,979038	9,942338	9,7675607	9,629301667	-0,138259	0,908614983	9430076G02Rik
17424928	6,30695	6,275415	6,141077	6,030826	6,248483	6,029387	6,2411473	6,102898667	-0,13824867	0,908621491	Tomm5
17389066	6,977774	7,15831	7,001143	6,89905	6,889349	6,934093	7,0457423	6,907497333	-0,138245	0,9086238	Dnajc24
17453137	7,413275	7,9092	7,673854	7,651426	7,415323	7,514875	7,665443	7,527208	-0,138235	0,908630098	Cct6a // Cct6a
17547897	4,743031	4,852099	4,588689	4,6879	4,424668	4,656565	4,7279397	4,589711	-0,13822867	0,908634087	---
17272163	6,689933	6,31731	6,549149	6,432028	6,292582	6,41714	6,5187973	6,380583333	-0,138214	0,908643324	Trim47
17284039	9,2643	9,412436	9,344862	9,185148	9,124296	9,297551	9,3405327	9,202331667	-0,138201	0,908651512	Cinp
17315240	3,515295	3,586964	3,480515	3,35675	3,344374	3,467066	3,5275913	3,389396667	-0,13819467	0,908655501	Gm5477
17217232	5,205355	5,156495	5,25058	5,24925	4,982502	4,966198	5,2041433	5,065983333	-0,13816	0,908677335	Tmem81
17393562	7,701374	7,625289	7,986893	7,821782	7,685975	7,391459	7,7711853	7,633072	-0,13811333	0,908706729	---
17548687	5,642221	5,732236	6,032019	5,843234	5,467222	5,681716	5,8021587	5,664057333	-0,13810133	0,908714287	Nod2 // Nod2
17392785	3,016675	2,906579	3,139003	3,024669	2,814126	2,80925	3,0207523	2,882681667	-0,13807067	0,908733603	---
17540075	9,014679	9,026686	9,055719	8,950975	8,790258	8,941736	9,0323613	8,894323	-0,13803833	0,90875397	Ftsj1
17457207	5,655642	5,438056	5,603488	5,484976	5,245112	5,552988	5,5657287	5,427692	-0,13803667	0,90875502	Agbl3
17315538	4,737416	4,657073	4,755205	4,685564	4,623935	4,426155	4,7165647	4,578551333	-0,13801333	0,908769718	Hoxc4
17285386	10,11805	10,31033	10,04033	10,0441	9,975931	10,03467	10,156237	10,01823367	-0,138003	0,908776227	Psma2
17510635	6,045902	5,668071	6,129825	5,788235	5,855809	5,785749	5,9479327	5,809931	-0,13800167	0,908777067	Nr3c2 // Nr3c2
17323427	4,274972	4,40739	4,989158	4,224983	4,419525	4,613016	4,5571733	4,419174667	-0,13799867	0,908778956	Rimbp3
17330636	4,926865	5,192194	4,894612	4,828329	4,919403	4,851955	5,004557	4,866562333	-0,13799467	0,908781476	Gm609 // Gm609
17341132	9,86308	9,928905	10,03903	9,827641	9,697816	9,891593	9,9436717	9,805683333	-0,13798833	0,908785466	Phf10
17523224	8,615793	8,679771	8,581994	8,478735	8,451121	8,533751	8,6258527	8,487869	-0,13798367	0,908788405	Eif1b
17355213	8,326439	8,453537	8,233057	8,130152	8,211566	8,257426	8,3376777	8,199714667	-0,137963	0,908801424	Cep76
17527308	4,089791	3,86404	4,044736	3,703883	3,945025	3,935908	3,9995223	3,861605333	-0,137917	0,908830401	Psma4
17522488	5,978158	5,624961	5,961257	5,827942	5,887914	5,434798	5,854792	5,716884667	-0,13790733	0,908836491	Prss46
17200565	5,322117	6,00547	6,10925	5,761166	5,874036	5,387926	5,812279	5,674376	-0,137903	0,90883922	---
17484644	8,279085	8,253162	8,356018	8,173102	8,127391	8,174063	8,2960883	8,158185333	-0,137903	0,90883922	Psmd13
17519718	7,978299	8,034095	7,961191	7,686436	7,816046	8,057467	7,991195	7,853316333	-0,13787867	0,90885455	Mto1

17539762	5,622548	5,743602	5,544177	5,267575	5,649445	5,57971	5,6367757	5,49891	-0,13786567	0,908862739	AU022751
17489856	3,974715	4,082484	3,730323	3,883103	3,905348	3,585501	3,929174	3,791317333	-0,13785667	0,908868409	---
17251179	4,52543	4,110071	4,015064	3,959271	4,170256	4,107477	4,216855	4,079001333	-0,13785367	0,908870299	Myh13
17339077	4,000233	3,989921	4,183495	3,973177	3,770908	4,01609	4,057883	3,920058333	-0,13782467	0,908888569	---
17423490	7,546349	7,87247	7,131809	7,075234	7,419907	7,642045	7,516876	7,379062	-0,137814	0,908895289	Ripk2
17202727	3,369377	3,720822	3,424461	3,380428	3,602439	3,118356	3,5048867	3,367074333	-0,13781233	0,908896339	---
17259507	9,768005	9,576349	9,759682	9,539433	9,64225	9,508918	9,7013453	9,563533667	-0,13781167	0,908896759	Hmga1-rs1
17475580	9,687038	9,756338	9,892161	9,588424	9,714526	9,619165	9,7785123	9,640705	-0,13780733	0,908899489	Blvrb
17309118	6,015535	6,050745	5,888935	5,931151	5,627598	5,983061	5,9850717	5,84727	-0,13780167	0,908903059	Tbc1d4
17497203	8,346231	8,501401	8,379716	8,233862	8,139367	8,440949	8,409116	8,271392667	-0,13772333	0,90895241	Uros
17532641	11,16492	11,51877	11,30553	11,15852	11,2509	11,16667	11,32974	11,19203	-0,13771	0,908960811	---
17379456	5,317946	5,281467	5,413941	5,391899	4,987768	5,220572	5,3377847	5,200079667	-0,137705	0,908963961	Gm14317
17292312	5,305261	4,87533	5,342335	4,870175	5,471845	4,76799	5,1743087	5,03667	-0,13763867	0,909005755	A330048O09Rik
17550855	5,048321	4,852857	5,337799	4,896091	4,934943	4,995079	5,079659	4,942037667	-0,13762133	0,909016676	---
17481684	7,09566	7,292741	7,126472	6,979774	7,096267	7,026012	7,1716243	7,034017667	-0,13760667	0,909025918	D930014E17Rik
17221923	6,20789	6,609772	6,65624	6,307279	6,429842	6,323975	6,4913007	6,353698667	-0,137602	0,909028858	---
17488076	4,20794	4,8306	5,385139	4,612204	4,995512	4,403162	4,807893	4,670292667	-0,13760033	0,909029908	---
17260761	10,4041	10,44733	10,27036	10,19101	10,3596	10,15838	10,37393	10,23633	-0,1376	0,909030118	Plek
17424476	4,071252	4,301827	4,281664	4,11458	4,003375	4,124037	4,2182477	4,080664	-0,13758367	0,90904041	---
17201499	7,204013	5,723963	5,982599	5,975982	6,160424	6,361423	6,303525	6,165943	-0,137582	0,90904146	---
17207799	3,469896	4,220063	4,082013	4,669092	3,297027	3,393135	3,9239907	3,786418	-0,13757267	0,909047341	---
17285742	8,395655	8,70927	8,909784	8,727537	8,234395	8,640133	8,6715697	8,534021667	-0,137548	0,909062884	Hist1h2bk
17420514	8,04991	8,141313	8,023643	7,900652	8,029144	7,87244	8,071622	7,934078667	-0,13754333	0,909065824	Camk2n1
17448240	6,57084	6,260594	6,445078	6,663849	6,033236	6,16686	6,425504	6,287981667	-0,13752233	0,909079057	---
17548066	6,000818	6,029894	5,974689	5,917025	5,772204	5,903684	6,0018003	5,864304333	-0,137496	0,90909565	LOC100861752
17481531	5,202094	5,189583	5,221978	5,080338	5,103571	5,017268	5,2045517	5,067059	-0,13749267	0,909097751	Rbmxl2
17246113	9,346112	9,308624	9,525555	9,409344	9,084438	9,274099	9,3934303	9,255960333	-0,13747	0,909112034	Slc39a5
17547624	3,342911	3,27669	3,116578	3,00666	3,2414	3,07572	3,245393	3,107926667	-0,13746633	0,909114344	Gm19984
17310304	6,834262	7,000718	6,608243	6,613848	6,751545	6,665472	6,8144077	6,676955	-0,13745267	0,909122956	Rad1
17382169	7,542232	7,488741	7,459257	7,194634	7,511513	7,371767	7,4967433	7,359304667	-0,13743867	0,909131779	Mastl
17549390	4,897317	5,088731	4,907702	4,792528	4,753373	4,935573	4,9645833	4,827158	-0,13742533	0,909140181	---
17549774	4,897317	5,088731	4,907702	4,792528	4,753373	4,935573	4,9645833	4,827158	-0,13742533	0,909140181	---
17431992	4,743229	4,470198	4,706079	4,578093	4,43835	4,490822	4,6398353	4,502421667	-0,13741367	0,909147533	Padi6
17449355	3,655981	3,395577	3,385394	3,323495	3,273214	3,428044	3,478984	3,341584333	-0,13739967	0,909156355	Ugt2b37
17262338	11,99672	11,96614	11,82407	11,67461	11,79706	11,90308	11,928977	11,79158333	-0,13739333	0,909160346	---
17374482	3,744872	3,824728	3,756408	3,750572	3,579669	3,583673	3,775336	3,637971333	-0,13736467	0,909178412	4930412B13Rik // 4930412B13Rik
17372805	4,547636	3,929389	3,996239	4,047863	4,274284	3,739033	4,1577547	4,020393333	-0,13736133	0,909180512	Olfr1038-ps
17320583	6,371639	7,019355	6,907717	6,565664	6,595439	6,725542	6,766237	6,628881667	-0,13735533	0,909184294	Cpne8
17208151	4,162413	4,81443	4,147498	4,757881	4,085269	3,869163	4,3747803	4,237437667	-0,13734267	0,909192276	---
17342386	6,902184	6,895175	6,951707	6,692088	6,982347	6,662675	6,9163553	6,779036667	-0,13731867	0,909207401	Metrn
17420024	8,735714	8,863085	8,836357	8,564454	8,679315	8,779519	8,8117187	8,674429333	-0,13728933	0,909225888	E2f2
17428904	6,234781	6,357569	6,187264	5,993873	6,172784	6,20123	6,2598713	6,122629	-0,13724233	0,909255509	Klf17
17519692	3,566047	3,074612	3,261335	3,352975	3,175338	2,962	3,3006647	3,163437667	-0,137227	0,909265173	Omt2b
17395969	3,922817	3,61322	2,875353	3,590564	3,057396	3,351752	3,4704633	3,333237333	-0,137226	0,909265803	---
17550158	3,922817	3,61322	2,875353	3,590564	3,057396	3,351752	3,4704633	3,333237333	-0,137226	0,909265803	---
17343164	10,62039	10,56982	9,619868	10,05217	10,18883	10,1575	10,270026	10,13283333	-0,13719267	0,909286812	Tff3
17426340	5,400002	5,307625	5,47917	5,372087	5,203169	5,200003	5,395599	5,258419667	-0,13717933	0,909295215	1700018C11Rik
17481200	3,091314	3,176039	3,188082	2,887108	3,21262	2,944214	3,1518117	3,014647333	-0,13716433	0,90930467	Usp17l5
17397878	3,215087	3,174139	3,258419	3,025262	3,197347	3,013681	3,2158817	3,078763333	-0,13711833	0,909333663	Gm5538
17439546	4,731925	5,041923	4,874975	4,765273	4,782258	4,689975	4,882941	4,745835333	-0,13710567	0,909341647	---
17311519	6,111864	5,651657	5,654226	5,473325	5,753133	5,780025	5,8059157	5,668827667	-0,137088	0,909352782	Nov
17262316	5,900469	5,814138	5,914295	5,919378	5,514135	5,784129	5,8763007	5,739214	-0,13708667	0,909353623	Ltc4s
17247319	4,774695	4,968698	4,945065	4,72387	4,805478	4,747864	4,8961527	4,759070667	-0,137082	0,909356564	4930415F15Rik
17427202	3,745211	3,798919	4,004593	3,857359	3,701056	3,579082	3,8495743	3,712499	-0,13707533	0,909360766	---
17423333	3,554637	3,922135	4,163613	3,855333	3,649189	3,724661	3,8801283	3,743061	-0,13706733	0,909365809	---
17267538	3,221031	3,30761	3,53675	3,037649	3,383552	3,233008	3,3551303	3,218069667	-0,13706067	0,909370011	Olfr463
17449855	6,015257	5,924458	6,078567	5,965815	5,770628	5,87067	6,006094	5,869037667	-0,13705633	0,909372743	A430089I19Rik
17449873	6,015257	5,924458	6,078567	5,965815	5,770628	5,87067	6,006094	5,869037667	-0,13705633	0,909372743	A430089I19Rik
17296052	8,946099	8,929193	8,839293	8,582602	8,808553	8,912262	8,9048617	8,767805667	-0,137056	0,909372953	Kif2a
17513228	3,135699	3,790272	3,822645	3,381325	3,386534	3,569942	3,582872	3,445933667	-0,13693833	0,909447124	Gm6793 // Gm6793
17242910	8,1009	7,960788	8,122272	7,912386	7,98668	7,874101	8,06132	7,924389	-0,136931	0,909451747	Mbd3
17548804	3,783225	3,535062	3,792897	3,623934	3,507694	3,568808	3,703728	3,566812	-0,136916	0,909461203	---
17358014	10,08711	10,37666	9,958704	9,516867	9,98539	10,5098	10,140825	10,004019	-0,13680567	0,909530759	---
17541563	3,762106	3,434897	3,434897	3,417895	3,306698	3,496911	3,5439667	3,407168	-0,13679867	0,909535172	Olfr1324
17204581	5,561499	6,083297	6,060991	6,125977	5,809102	5,360316	5,901929	5,765131667	-0,13679733	0,909536012	---
17273580	6,066708	5,999168	6,210453	5,994688	6,031657	5,8396	6,0921097	5,955315	-0,13679467	0,909537694	1700012B15Rik
17403919	8,920516	9,037151	8,942666	8,644344	8,839825	9,005815	8,9667777	8,829994667	-0,136783	0,909545049	Depdc1a
17205453	3,744872	3,45013	3,424988	3,052068	3,955553	3,202088	3,5399967	3,403236333	-0,13676033	0,909559339	---
17242326	5,436649	5,223469	5,442866	5,197209	5,346845	5,148777	5,3676613	5,230943667	-0,13671767	0,909586239	Gm19668
17397439	10,07181	10,20718	9,898694	9,87118	9,89467	10,00174	10,059228	9,92253	-0,136698	0,909598639	Naa15
17534122	7,790027	8,000766	7,723128	7,62134	7,638454	7,84408	7,8379737	7,701291333	-0,13668233	0,909608516	Ube2a
17498053	4,376893	4,591406	4,726627	4,331911	4,62185	4,331136	4,5649753	4,428299	-0,13667633	0,909612299	Gm9340
17445507	8,420475	8,662492	8,59748	8,397931	8,42943	8,443136	8,560149	8,423499	-0,13665	0,909628902	Dbf4
17314675	6,366714	6,580274	6,801682	6,243097	6,446143	6,649485	6,58289	6,446241667	-0,13664833	0,909629953	---
17429655	4,979945	5,247734	5,193726	4,780104	4,922689	5,308685	5,1404683	5,003826	-0,13664233	0,909633736	Oxct2b
17274875	9,577492	9,943941	9,434272	9,367518	9,614551	9,563862	9,6519017	9,515310333	-0,13659133	0,909665893	Nampt
17471578	2,892968	2,864885	2,953663	2,686635	2,762148	2,853017	2,9038387	2,767266667	-0,136572	0,909678083	Klrc3
17348850	7,169094	7,157272	6,828969	6,844363	6,843238	7,058047	7,0517783	6,915216	-0,13656233	0,909684178	Rnf138
17548305	9,566036	9,772026	9,790211	9,587089	9,517939	9,613578	9,7094243	9,572868667	-0,13655567	0,909688382	Tmem234
17397875	4,981391	5,204177	5,13613	4,983236	5,070938	4,858001	5,1072327	4,970738333	-0,13649433	0,909727057	Gpr171 // Gpr171
17423228	8,622473	8,684815	8,635395	8,505613	8,480286	8,547312	8,647561	8,511070333	-0,13649067	0,909729369	Ints8
17482547	4,901225	4,619884	5,21025	4,872686	4,780744	4,668519	4,910453	4,773983	-0,13647	0,909742401	Gm5737
17381930	3,170185	3,287704	3,184264	3,156618	3,136644	2,939625	3,214051	3,077629	-0,136422	0,909772669	4930515L03Rik
17462608	4,465801	5,131958	4,062818	5,081098	3,905742	4,264572	4,5535257	4,417137333	-0,13638833	0,9097939	Mug-ps1
17503333	8,623629	8,923933	8,77262	8,659451	8,630285	8,621283	8,773394	8,637006333	-0,13638767	0,90979432	Tnpo2
17432790	6,721377	6,993108	6,848515	6,749631	6,708594	6,69563	6,8543333	6,717951667	-0,13638167	0,909798104	Miip
17516133	3,501296	3,414444	3,477392	3,414444	3,31566	3,253976	3,4643773	3,328026667	-0,13635067	0,909817654	Olfr885
17439940	3,102818	3,069698	3,165188	2,970169	3,05992	2,898609	3,112568	2,976232667	-0,13633533	0,909827323	---
17495932	6,872515	6,740816	6,626852	6,699167	6,564561	6,567462	6,7467277	6,610396667	-0,136331	0,909830056	Palb2
17545627	3,152925	3,238169	3,006545	2,965216	2,887856	3,135596	3,1325463	2,996222667	-0,13632367	0,909834681	---
17382089	4,313589	4,179044	4,55141	4,288187	4,148768	4,198124	4,3480143	4,211693	-0,13632133	0,909836153	---
17286914	3,777279	3,734743	4,29459	3,742501	3,755298	3,899863	3,9355373	3,799220667	-0,13631667	0,909839096	---
17492493	3,54705	3,734285	3,725938	3,592818	3,433496	3,572136	3,669091	3,532816667	-0,13627433	0,909865794	Mir1965 // Mir1965
17326940	5,122699	4,927976	4,886585	4,814272	4,944264	4,769941	4,9790867	4,842825667	-0,136261	0,909874203	---
17391018	10,40578	10,58665	10,54313	10,3417	10,37799	10,4071	10,511853	10,37559667	-0,13625667	0,909876935	Cops2
17512193	4,328426	4,122181	4,219752	4,126728	4,18007	3,954837	4,223453	4,087211667	-0,13624133	0,909886606	Cmtm2a
17209143	3,681034	4,963331	3,626319	3,383144	4,852507	3,626319	4,090228	3,95399	-0,136238	0,909888708	---
17434995	3,875243	3,484935	3,827589	3,655854	3,411504	3,711705	3,7292557	3,593021	-0,13623467	0,909890811	Gnat3
17207469	4,491183	4,635309	4,584003	4,698355	4,366218	4,237265	4,570165	4,433946	-0,136219	0,909900691	---
17548352	11,32306	11,33327	11,24586	11,1867	11,1694	11,13746	11,30073	11,16452	-0,13621	0,909906368	---
17433525	4,805995	4,728737	4,901817	4,770451	4,65615	4,601337	4,812183	4,675979333	-0,13620367	0,909910362	---
17358658	4,530063	4,901534	4,228847	4,355528	4,303134	4,593223	4,5534813	4,417295	-0,13618633	0,909921294	---
17477399	3,527167	4,021649	3,469955	3,591145	3,623512	3,395645	3,6729237	3,536767333	-0,13615633	0,909940216	---
17465603	3,018262	3,023387	3,188424	2,989716	2,900901	2,931108	3,076691	2,940575	-0,136116	0,909965655	Mir29b-1
17209075	4,899795	5,317205	5,108201	5,147715	5,050555	4,718637	5,1084003	4,972302333	-0,136098	0,909977009	---
17289085	8,789441	8,971917	8,771061	8,798783	8,555374	8,769972	8,8441397	8,708043	-0,13609667	0,90997785	Dhfr
17427185	4,540586	4,672834	4,930506	4,438341	4,662311	4,6353	4,714642	4,578650667	-0,13599133	0,910044291	4930577H14Rik
17259871	3,390998	3,592311	3,645046	3,367382	3,374889	3,478266	3,542785	3,406845667	-0,13593933	0,910077093	---
17467573	3,428421	3,426671	3,405948	3,104322	3,294137	3,45482	3,4203467	3,284426333	-0,13592033	0,910089078	---
17452887	3,167036	3,034507	3,213483	2,963471	3,039207	3,004676	3,138342	3,002451333	-0,13589067	0,910107793	---
17377425	3,716011	3,789598	4,026925	3,747731	3,690248	3,686885	3,844178	3,708288	-0,13589	0,910108214	Cst10
17200387	5,016851	4,501745	4,445661	4,657721	4,49473	4,404243	4,6547523	4,518898	-0,13585433	0,910130714	---
17282290	4,655628	4,697813	4,564936	4,429003	4,698442	4,383407	4,639459	4,503617333	-0,13584167	0,910138705	---
17243493	4,885673	4,93144	4,656573	4,762252	4,516845	4,787122	4,824562	4,688739667	-0,13582233	0,910150901	Glt8d2
17202037	4,702593	4,66298	4,852872	4,559525	4,417347	4,834121	4,7394817	4,603664333	-0,13581733	0,910154056	---
17363220	7,39555	7,565247	7,521286	7,194348	7,446613	7,433736	7,4940277	7,358232333	-0,13579533	0,910167935	Cep78
17422117	7,026312	6,695796	6,736016	6,646079	6,664707	6,740016	6,8193747	6,683600667	-0,135774	0,910181394	Acot7
17250468	5,980836	6,598267	5,974602	6,211536	5,771633	6,163236	6,1845683	6,048801667	-0,13576667	0,910186021	---
17425603	4,920614	4,385981	4,509027	4,423041	4,629922	4,355415	4,6052073	4,469459333	-0,135748	0,910197797	Gm12538
17231680	6,633381	6,690899	6,574763	6,424999	6,521017	6,545788	6,6330143	6,497268	-0,13574633	0,910198849	---
17471970	3,111845	3,302033	3,393883	3,134533	3,28966	2,976356	3,2692537	3,133516333	-0,13573733	0,910204527	Tas2r140
17209605	6,019991	6,217813	6,6321	6,090895	6,019991	6,351823	6,289968	6,154236333	-0,13573167	0,910208102	---
17208341	7,538787	7,237802	7,175727	7,201645	7,250751	7,092734	7,3174387	7,18171	-0,13572867	0,910209995	---
17289415	5,220115	5,4093	5,24859	5,101972	5,278448	5,090455	5,2926683	5,156958333	-0,13571	0,910221772	---
17268332	4,247329	3,91795	4,198945	4,136052	3,881231	3,939813	4,121408	3,985698667	-0,13570933	0,910222192	2010300F17Rik
17486519	3,428907	3,146069	2,994622	2,895848	3,090271	3,176355	3,189866	3,054158	-0,135708	0,910223034	Vmn1r81
17349514	7,041858	7,1958	6,834262	6,557308	7,090001	7,017608	7,0239733	6,888305667	-0,13566767	0,910248481	Egr1
17209581	6,195055	5,537078	6,193101	5,854266	5,531928	6,132061	5,975078	5,839418333	-0,13565967	0,910253529	---
17432197	4,318606	4,299574	4,327048	4,179282	4,167624	4,191495	4,315076	4,179467	-0,135609	0,910285497	Gm694
17212316	4,232403	4,359739	4,063478	4,093795	4,005514	4,149489	4,21854	4,082932667	-0,13560733	0,910286548	2900092D14Rik
17445238	4,948943	4,810295	5,16727	4,666748	4,88064	4,972367	4,9755027	4,839918333	-0,13558433	0,910301061	Kl
17232129	4,358529	4,178762	4,400108	4,063452	4,282411	4,184793	4,3124663	4,176885333	-0,135581	0,910303164	---
17227791	4,211873	4,032796	4,122165	3,836733	4,126203	3,997192	4,122278	3,986709333	-0,13556867	0,910310946	Rgs21
17209859	5,611753	5,697608	6,18055	5,695029	5,234344	6,15391	5,8299703	5,694427667	-0,13554267	0,910327351	---

17317555	6,046545	5,815399	5,861928	5,897994	5,798265	5,621019	5,9079573	5,772426	-0,13553133	0,910334503	Oc90
17370520	3,296439	3,240051	3,348549	3,340925	3,131805	3,005742	3,295013	3,159490667	-0,13552233	0,910340182	---
17237180	10,40883	10,38347	10,21596	10,21084	10,2269	10,16431	10,336087	10,20068333	-0,13540333	0,910415274	---
17310652	8,251554	8,284528	8,338928	8,210833	8,168171	8,089804	8,29167	8,156269333	-0,13540067	0,910416957	Zfp622
17389449	4,572718	4,807846	4,495262	4,433037	4,640421	4,396227	4,6252753	4,489895	-0,13538033	0,910429788	Fmn1
17336086	6,861598	6,664788	6,75377	6,810791	6,385691	6,677557	6,760052	6,624679667	-0,13537233	0,910434837	Adamts10
17464573	10,02905	10,00825	9,797721	9,834743	9,622766	9,971415	9,945007	9,809641333	-0,13536567	0,910439044	Tfpi2
17206993	3,027354	3,48328	3,292803	3,18996	2,971009	3,236442	3,2678123	3,132470333	-0,135342	0,910453979	---
17550522	3,819079	4,136392	3,982824	3,804995	3,861254	3,866047	3,9794317	3,844098667	-0,135333	0,910459659	---
17463795	3,412851	3,27799	3,275258	3,100705	3,233451	3,225946	3,322033	3,186700667	-0,13533233	0,91046008	---
17302352	3,164176	3,093122	3,172022	3,007519	3,155727	2,860139	3,1431067	3,007795	-0,13531167	0,910473122	---
17370499	5,581179	5,723756	5,905952	5,628841	5,721308	5,454857	5,7369623	5,601668667	-0,13529367	0,910484482	Wdr38
17366904	6,612864	5,953881	6,155672	6,039392	5,602075	6,675079	6,2408057	6,105515333	-0,13529033	0,910486585	---
17229533	5,28088	5,893926	5,651595	5,54353	5,46124	5,415761	5,6088003	5,473510333	-0,13529	0,910486796	Gm7694
17520971	5,267649	4,889922	5,406441	5,157292	5,068088	4,932778	5,188004	5,052719333	-0,13528467	0,910490162	---
17541917	10,79261	11,04596	11,01551	10,90067	10,89727	10,65035	10,95136	10,81609667	-0,13526333	0,910503625	Snord61
17531939	7,712784	7,431982	7,175539	7,414041	7,20311	7,297372	7,4401017	7,304841	-0,13526067	0,910505308	Gpd1l
17383232	5,317005	5,387843	5,493616	5,248482	5,233443	5,31078	5,399488	5,264235	-0,135253	0,910510147	Tmem8c
17500517	4,731793	4,704266	4,689128	4,612145	4,633347	4,473967	4,7083957	4,573153	-0,13524267	0,910516668	Mboat4
17453133	4,861753	6,035233	3,585267	4,646607	4,192953	5,237144	4,8274177	4,692234667	-0,135183	0,910554326	---
17549506	6,967931	7,293006	6,829939	6,719258	6,868831	7,097239	7,030292	6,895109333	-0,13518267	0,910554537	---
17549658	6,967931	7,293006	6,829939	6,719258	6,868831	7,097239	7,030292	6,895109333	-0,13518267	0,910554537	---
17524925	6,855168	7,279307	7,258416	6,833572	7,262098	6,891686	7,1309637	6,995785333	-0,13517833	0,910557272	Elof1
17206861	5,620424	6,564061	5,978175	5,819284	6,378851	5,559083	6,05422	5,919072667	-0,13514733	0,910576837	---
17238679	4,364254	4,619744	4,563995	4,534071	4,630395	3,978088	4,5159977	4,380851333	-0,13514633	0,910577469	Olfr794
17499959	4,471437	4,473683	4,559122	4,352762	4,431672	4,31438	4,501414	4,366271333	-0,13514267	0,910579783	---
17313456	5,402593	4,809987	5,053078	4,910759	4,968336	4,981182	5,0885527	4,953425667	-0,135127	0,910589671	4930407I10Rik
17355026	9,606974	9,88942	9,399968	9,53161	9,407668	9,55176	9,6321207	9,497012667	-0,135108	0,910601663	Atp8b1
17414428	8,021727	7,928698	7,846899	7,837912	7,757105	7,797073	7,9324413	7,797363333	-0,135078	0,910620599	Gng10
17320071	3,219316	3,06005	3,222763	3,124205	3,003616	2,969086	3,1673763	3,032302333	-0,135074	0,910623124	---
17315878	8,544938	8,593333	8,443123	8,299087	8,428176	8,44892	8,5271313	8,392061	-0,13507033	0,910625438	Skp2
17464010	8,392653	8,502621	8,28525	8,299069	8,3304	8,145897	8,393508	8,258455333	-0,13505267	0,91063659	Aebp2
17530496	5,94924	5,725886	5,748958	5,716702	5,601407	5,700978	5,808028	5,673029	-0,134999	0,910670465	Gm19687
17315346	4,474026	4,354832	4,393492	4,344897	4,164169	4,308421	4,40745	4,272495667	-0,13495433	0,91069866	Gm9918
17222429	7,18357	7,362032	7,013922	6,880327	7,150297	7,124041	7,186508	7,051555	-0,134953	0,910699502	Txndc9
17410311	4,274434	3,919537	4,115407	3,978623	3,916679	4,00923	4,103126	3,968177333	-0,13494867	0,910702237	Rrh
17545199	5,784218	6,096028	6,071013	5,961707	5,742296	5,842425	5,983753	5,848809333	-0,13494367	0,910705393	Lrch2
17344309	6,41403	6,387299	6,125497	6,11141	6,10805	6,302622	6,308942	6,174027333	-0,13491467	0,9107237	Tnf
17512275	8,286527	8,551475	8,184972	8,137937	8,239883	8,240451	8,3409913	8,206090333	-0,134901	0,910732327	Nae1
17230031	3,977011	3,734457	3,740595	3,734457	3,527807	3,785117	3,8173543	3,682460333	-0,134894	0,910736746	Gm20134
17244699	3,402709	3,185098	3,097188	3,138449	3,203673	2,9382	3,2283317	3,093440667	-0,134891	0,91073864	Rps15a-ps1
17510365	6,551263	6,019166	6,187872	5,836051	6,308868	6,208787	6,252767	6,117902	-0,134865	0,910755053	Pgls
17513581	5,747112	5,548099	6,048291	5,709276	5,699818	5,529843	5,7811673	5,646312333	-0,134855	0,910761366	1700018B08Rik
17238646	4,143219	3,665332	3,63758	3,775528	3,92578	3,340266	3,815377	3,680524667	-0,13485233	0,91076305	Olfr774
17390980	6,326846	6,181982	6,195883	6,080258	6,146275	6,073678	6,2349037	6,100070333	-0,13483333	0,910775044	Shc4
17532063	7,590874	7,170591	7,13939	7,101556	7,137772	7,25703	7,300285	7,165452667	-0,13483233	0,910775676	Acaa1b
17454915	5,385243	5,811211	5,465914	5,139659	5,647499	5,470763	5,5541227	5,419307	-0,13481567	0,910786197	---
17340745	3,3974	3,449031	3,288981	3,270892	3,242208	3,217889	3,3784707	3,243663	-0,13480767	0,910791248	---
17226829	6,186337	6,157982	6,216409	5,88342	6,094521	6,178428	6,1869093	6,052123	-0,13478633	0,910804716	Mfsd4
17430722	5,197804	5,390521	5,424782	5,060737	5,161287	5,386772	5,3377023	5,202932	-0,13477033	0,910814817	---
17419027	9,789037	9,758478	9,737901	9,508828	9,756314	9,615976	9,7618053	9,627039333	-0,134766	0,910817553	---
17510368	5,471287	5,317086	5,631916	5,365469	5,268734	5,381797	5,4734297	5,338666667	-0,134763	0,910819447	Unc13a
17541335	3,637271	3,587852	3,697895	3,494751	3,422422	3,601557	3,641006	3,506243333	-0,13476267	0,910819657	4930515L19Rik
17439532	4,940643	5,393133	5,818736	5,15569	5,151934	5,440684	5,3841707	5,249436	-0,13473467	0,910837335	---
17316268	2,997583	3,100456	3,270792	2,886879	3,104549	2,973211	3,1229437	2,988213	-0,13473067	0,91083986	Mir3964 // Mir3964
17466476	4,546523	4,769675	4,537863	4,762712	4,199872	4,487305	4,6180203	4,483296333	-0,134724	0,910844069	Nobox
17493723	6,377007	6,564159	6,39721	6,233051	6,634321	6,066839	6,4461253	6,311403667	-0,13472167	0,910845542	Spcs2
17431362	8,409887	8,613695	8,499388	8,362646	8,336996	8,419281	8,5076567	8,372974333	-0,13468233	0,910870376	Tmem57
17379716	4,734663	4,842634	4,663064	4,763838	4,423441	4,64904	4,746787	4,612106333	-0,13468067	0,910871428	---
17323067	9,104711	9,052728	9,00213	8,951392	8,876738	8,927412	9,0531897	8,918514	-0,13467567	0,910874585	---
17268955	5,502568	5,005829	5,24912	4,94107	5,01958	5,392872	5,2525057	5,117840667	-0,134665	0,91088132	Tns4
17297810	5,213741	5,029526	5,127853	4,988422	4,936917	5,041865	5,1237067	4,989068	-0,13463867	0,910897946	Cphx
17297842	5,213741	5,029526	5,127853	4,988422	4,936917	5,041865	5,1237067	4,989068	-0,13463867	0,910897946	Cphx
17466228	8,33278	8,31516	8,304578	8,055375	8,292728	8,200523	8,317506	8,182875333	-0,13463067	0,910902997	Clec5a
17509433	2,915787	3,134845	3,035839	2,987846	3,006927	2,687923	3,0288237	2,894232	-0,13459167	0,910927622	---
17511500	5,124277	5,303351	5,124538	4,982014	5,213298	4,953084	5,1840553	5,049465333	-0,13459	0,910928674	Gm2716 // Gm2716
17275298	3,859308	3,838263	3,490492	3,682907	3,627005	3,47439	3,7293543	3,594767333	-0,134587	0,910930568	---
17202405	6,290696	5,692223	6,284983	6,417089	6,010635	5,436423	6,0893007	5,954715667	-0,134585	0,910931831	---
17411566	3,135524	2,888827	3,164107	3,038798	2,900901	2,845129	3,0628193	2,928276	-0,13454333	0,91095814	---
17347061	5,895787	5,829537	6,401526	5,741422	5,78418	6,197655	6,0422833	5,907752333	-0,134531	0,910965928	---
17391915	4,606554	4,479419	5,022373	4,627834	4,484822	4,592156	4,702782	4,568270667	-0,13451133	0,910978346	Erv3
17249234	4,888714	4,802552	4,914486	4,834145	4,572524	4,795553	4,868584	4,734074	-0,13451	0,910979188	Grm6
17301247	7,991416	7,962957	7,934035	7,799812	7,786923	7,898183	7,9628027	7,828306	-0,13449667	0,910987607	Pinx1
17495020	5,193937	5,400945	5,228458	5,309074	4,926201	5,184606	5,2744467	5,139960333	-0,13448633	0,910994132	1600010M07Rik
17396978	9,296341	9,329144	9,170199	9,194963	9,045541	9,151726	9,265228	9,130743333	-0,13448467	0,910995185	Exosc9
17331264	3,355404	3,089993	2,940012	2,910188	3,148104	2,923732	3,1284697	2,994008	-0,13446167	0,911009708	Olfr199
17454787	5,531877	5,669324	5,435662	5,611955	5,274456	5,347091	5,545621	5,411167333	-0,13445367	0,91101476	---
17514666	7,755525	7,781231	7,713139	7,716535	7,55998	7,570091	7,749965	7,615535333	-0,13442967	0,911029915	Ccdc82
17273910	7,22789	7,057234	7,174258	6,903762	7,153471	6,998903	7,1531273	7,018712	-0,13441533	0,911038966	Hs1bp3
17221250	7,999653	9,066596	7,474559	7,991645	6,629674	9,516399	8,1802693	8,045906	-0,13436333	0,911071804	---
17207689	7,941936	8,04363	7,884851	7,964356	7,740456	7,762628	7,9568057	7,82248	-0,13432567	0,911095591	---
17259924	6,178026	6,372172	6,249012	6,00832	6,352938	6,034985	6,2664033	6,132081	-0,13432233	0,911097696	4930556J24Rik
17372927	4,146265	4,021251	3,745326	3,918495	3,781266	3,810148	3,9709473	3,836636333	-0,134311	0,911104854	Olfr1204
17229988	6,014071	6,293896	5,982217	6,08396	5,898375	5,904922	6,096728	5,962419	-0,134309	0,911106117	Cadm3
17376560	7,342938	7,306965	6,980857	7,024419	7,206406	6,997015	7,2102533	7,075946667	-0,13430667	0,91110759	---
17466659	9,628821	9,685048	9,395095	9,408582	9,423346	9,474148	9,5696547	9,435358667	-0,134296	0,911114327	Tra2a
17296881	6,708217	5,923645	5,96758	6,373824	6,088786	5,733954	6,199814	6,065521333	-0,13429267	0,911116432	Gm9602
17307472	10,19807	10,3678	10,24527	10,11816	10,08569	10,20468	10,27038	10,13617667	-0,13420333	0,911172851	Kpna3
17491596	7,866602	7,500398	7,133722	7,789011	6,881822	7,42732	7,5002407	7,366051	-0,13418967	0,911181483	Snord116
17491598	7,866602	7,500398	7,133722	7,789011	6,881822	7,42732	7,5002407	7,366051	-0,13418967	0,911181483	Snord116 // Snord116
17491602	7,866602	7,500398	7,133722	7,789011	6,881822	7,42732	7,5002407	7,366051	-0,13418967	0,911181483	Snord116
17491604	7,866602	7,500398	7,133722	7,789011	6,881822	7,42732	7,5002407	7,366051	-0,13418967	0,911181483	Snord116
17491608	7,866602	7,500398	7,133722	7,789011	6,881822	7,42732	7,5002407	7,366051	-0,13418967	0,911181483	Snord116 // Snord116
17491610	7,866602	7,500398	7,133722	7,789011	6,881822	7,42732	7,5002407	7,366051	-0,13418967	0,911181483	Snord116 // Snord116
17491612	7,866602	7,500398	7,133722	7,789011	6,881822	7,42732	7,5002407	7,366051	-0,13418967	0,911181483	Snord116
17491614	7,866602	7,500398	7,133722	7,789011	6,881822	7,42732	7,5002407	7,366051	-0,13418967	0,911181483	Snord116
17491616	7,866602	7,500398	7,133722	7,789011	6,881822	7,42732	7,5002407	7,366051	-0,13418967	0,911181483	Snord116 // Snord116
17491618	7,866602	7,500398	7,133722	7,789011	6,881822	7,42732	7,5002407	7,366051	-0,13418967	0,911181483	Snord116 // Snord116
17491620	7,866602	7,500398	7,133722	7,789011	6,881822	7,42732	7,5002407	7,366051	-0,13418967	0,911181483	Snord116 // Snord116
17491622	7,866602	7,500398	7,133722	7,789011	6,881822	7,42732	7,5002407	7,366051	-0,13418967	0,911181483	Snord116
17491624	7,866602	7,500398	7,133722	7,789011	6,881822	7,42732	7,5002407	7,366051	-0,13418967	0,911181483	Snord116 // Snord116
17491628	7,866602	7,500398	7,133722	7,789011	6,881822	7,42732	7,5002407	7,366051	-0,13418967	0,911181483	Snord116 // Snord116
17491630	7,866602	7,500398	7,133722	7,789011	6,881822	7,42732	7,5002407	7,366051	-0,13418967	0,911181483	Snord116 // Snord116
17333386	4,106349	4,470156	5,2014	4,439233	4,210738	4,725449	4,592635	4,458473333	-0,13416167	0,911199167	Tcp10c
17236196	6,2706	6,44626	6,216124	6,245011	6,017425	6,268092	6,3109947	6,176842667	-0,134152	0,911205272	1810014B01Rik
17374121	8,010326	7,955774	8,015656	7,781321	7,748124	8,04991	7,9939187	7,859785	-0,13413367	0,911216852	Ccdc34
17233799	8,486435	8,589564	8,450026	8,427118	8,307322	8,389244	8,508675	8,374561333	-0,13411367	0,911229484	Slc25a16
17206559	4,884824	5,529953	4,062483	5,022655	4,866499	4,185865	4,8257533	4,691673	-0,13408033	0,911250538	---
17205655	6,227658	6,684812	5,279607	6,463889	5,620966	5,704996	6,0640257	5,929950333	-0,13407533	0,911253696	---
17468798	9,492129	9,659431	9,54924	9,461874	9,381371	9,455347	9,5669333	9,432864	-0,13406933	0,911257486	Mcm2
17396765	4,412724	4,27368	4,439312	4,188573	4,354849	4,180129	4,3752387	4,241183667	-0,134055	0,91126654	Usp13
17203801	6,77335	6,481336	6,528886	7,024183	6,099414	6,257943	6,594524	6,460513333	-0,13401067	0,911294543	---
17516174	3,204569	3,125373	3,144413	3,023269	3,149836	2,899274	3,1581183	3,024126333	-0,133992	0,911306334	Olfr906
17293489	6,737433	6,816672	7,050626	6,770481	6,993663	6,438616	6,8682437	6,734253333	-0,13399033	0,911307387	Gm3609
17425990	5,80535	5,477771	5,287922	5,525966	5,201503	5,441693	5,523681	5,389720667	-0,13396033	0,911326337	Mup7
17520032	4,030534	3,523893	3,645148	3,450036	3,859496	3,488197	3,7331917	3,599243	-0,13394867	0,911333707	---
17478521	7,142691	7,113007	7,121382	6,991971	6,988214	6,99508	7,1256933	6,991755	-0,13393833	0,911340234	Tubgcp5
17205343	4,582829	3,932414	4,90661	3,867016	4,39548	4,75757	4,473951	4,340022	-0,133929	0,91134613	---
17439766	7,530189	7,540611	7,41854	7,089673	7,484189	7,513755	7,4964467	7,362539	-0,13390767	0,911359606	LOC100861848
17326702	4,16875	4,412935	4,242367	3,783221	4,445619	4,193515	4,274684	4,140785	-0,133899	0,911365081	Mirlet7c-1
17449548	8,310712	8,23532	8,124234	8,429847	7,057325	8,781431	8,223422	8,089534333	-0,13388767	0,911372241	---
17427799	6,989344	7,132512	6,934978	6,588942	7,056184	7,010127	7,0189447	6,885084333	-0,13386033	0,911389508	Dhcr24 // Dhcr24
17210535	4,827383	4,904329	4,544389	4,298542	4,209148	5,366889	4,7587003	4,624859667	-0,13384067	0,911401932	---
17490521	7,062104	6,675287	6,941705	6,599667	6,905819	6,772115	6,893032	6,759200333	-0,13383167	0,911407617	Bcl2l12
17314222	5,719511	5,824099	5,975288	5,684095	5,610263	5,823156	5,8396327	5,705838	-0,13379467	0,911430992	Acr
17221551	7,942467	6,531128	7,365104	7,335646	7,220795	6,880883	7,2795663	7,145774667	-0,13379167	0,911432887	---
17210777	6,954933	6,343476	6,411328	6,138272	6,336556	6,833551	6,5699123	6,436126333	-0,133786	0,911436467	---
17504376	3,371482	3,214151	3,382338	3,18497	3,229349	3,152388	3,322657	3,188902333	-0,13375467	0,911456263	Gm15679
17307493	7,917385	7,721355	7,821783	7,700996	7,614044	7,74425	7,8201743	7,68643	-0,13374433	0,911462791	6330409N04Rik
17203729	5,148144	5,659725	5,419372	4,993613	5,173638	5,658919	5,4090803	5,27539	-0,13369033	0,911496908	---
17206859	6,577495	6,727562	6,671406	6,81029	6,343688	6,421468	6,658821	6,525148667	-0,13367233	0,91150828	---
17246262	4,525647	3,953666	3,963886	3,954494	3,974544	4,113198	4,147733	4,014078667	-0,13365433	0,911519653	Rps26
17414342	5,263534	5,236762	5,295161	5,09031	5,206714	5,097512	5,2651523	5,131512	-0,13364033	0,911528498	Gm12530
17272487	4,635186	4,499484	4,996629	4,486701	4,706304	4,537433	4,710433	4,576812667	-0,13362033	0,911541135	St6galnac1

17273984	6,652027	7,108753	6,677089	6,814149	6,67332	6,549563	6,812623	6,679010667	-0,13361233	0,911546189	Wdr35
17517323	8,962305	8,990026	8,938689	8,839234	8,74926	8,901699	8,9636733	8,830064333	-0,133609	0,911548295	Npat
17307666	5,239408	5,123492	5,044726	4,846819	5,171837	4,988306	5,1358753	5,002320667	-0,13355467	0,911582626	4930578I06Rik
17202965	5,238691	5,090205	5,746202	4,951121	5,521221	5,202092	5,358366	5,224811333	-0,13355467	0,911582626	---
17367436	10,43684	10,70839	10,38208	10,3538	10,29862	10,47423	10,509103	10,37555	-0,13355333	0,911583468	Gm13363 // Gm13363 // Gm13363 // Gı
17548176	5,26212	5,769916	5,401962	5,233802	5,379926	5,419669	5,4779993	5,344465667	-0,13353367	0,911595895	Acss1 // Acss1
17266607	3,266115	3,043959	3,015198	3,00003	2,944758	2,979894	3,108424	2,974894	-0,13353	0,911598212	---
17291108	3,780971	3,392988	3,386581	3,392988	3,346797	3,420208	3,52018	3,386664333	-0,13351567	0,911607269	---
17309293	7,660217	7,635726	7,578954	7,453001	7,579452	7,441931	7,6249657	7,491461333	-0,13350433	0,91161443	Rnf219
17405845	4,037645	3,810424	3,836154	3,742376	3,802384	3,739087	3,894741	3,761282333	-0,13345867	0,911643287	Sis
17368401	5,035792	4,90428	5,143199	4,943692	4,630338	5,108882	5,027757	4,894304	-0,133453	0,911646867	Gm711
17477595	7,627686	7,180246	7,332483	7,30678	7,27503	7,15834	7,3801383	7,246716667	-0,13342167	0,911666667	Mir707
17303717	5,940624	6,036457	6,379975	5,969367	5,917073	6,070377	6,1190187	5,985605667	-0,133413	0,911672144	1810063B07Rik
17536052	4,289979	4,45716	3,74321	4,519054	4,111411	3,459647	4,1634497	4,030037333	-0,13341233	0,911672565	Obp1b
17297497	6,809697	7,070527	6,844328	6,772867	6,803144	6,748326	6,908184	6,774779	-0,133405	0,911677199	Chchd1
17489726	4,145356	4,237143	4,451503	4,090143	4,256493	4,0873	4,2780007	4,144645333	-0,13335533	0,911708586	Wdr88
17521204	6,312028	6,37988	6,305305	6,204558	6,156309	6,236362	6,3324043	6,199076333	-0,133328	0,911725859	Rpl29
17429414	6,438144	6,30768	6,777146	6,348844	6,544759	6,229466	6,5076567	6,374356333	-0,13330033	0,911743343	Ppcs
17272866	5,994413	5,526133	5,62066	5,717405	5,566126	5,457813	5,7137353	5,580448	-0,13328733	0,911751559	---
17462133	4,751339	4,735114	4,929074	4,599081	4,729706	4,68689	4,8051757	4,671892333	-0,13328333	0,911754087	Olfr212
17373776	10,66671	10,93829	11,01509	10,57796	10,85697	10,78534	10,873363	10,74009	-0,13327333	0,911760407	---
17445682	5,5647	5,661699	5,657805	5,543312	5,382881	5,558222	5,628068	5,494805	-0,133263	0,911766937	4930572O03Rik
17547779	8,966903	9,363004	9,172558	9,123112	8,780402	9,199244	9,1674883	9,034252667	-0,13323567	0,911784212	Rps24 // Rps24 // Rps24 // Rps24 // Rp
17385961	3,932941	3,850948	4,065428	3,918065	3,761076	3,770559	3,9497723	3,816566667	-0,13320567	0,911803172	---
17267773	8,151709	6,992932	7,993066	7,816056	7,559606	7,362469	7,712569	7,579377	-0,133192	0,91181181	---
17532811	5,164009	5,026613	5,44584	4,836987	5,328889	5,071021	5,212154	5,078965667	-0,13318833	0,911814127	Foxp3
17284981	3,470849	3,437779	3,436004	3,276291	3,32329	3,345507	3,4482107	3,315029333	-0,13318133	0,911818551	Akr1c21
17326081	9,669126	9,738724	9,622193	9,533855	9,430178	9,666537	9,676681	9,543523333	-0,13315767	0,911833509	C330027C09Rik
17247317	3,41799	3,35306	3,277601	3,230116	3,245622	3,173485	3,3495503	3,216407667	-0,13314267	0,91184299	---
17259693	4,166609	3,922845	3,904651	3,836625	3,901045	3,857026	3,998035	3,864898667	-0,13313633	0,911846993	Ptchd3
17527818	6,128129	5,514149	5,792906	5,942348	5,42064	5,672859	5,811728	5,678615667	-0,13311233	0,911862162	Senp8
17378670	6,566104	6,767176	6,902038	6,488636	6,721408	6,62595	6,745106	6,611998	-0,133108	0,911864901	Tgif2
17398321	5,910068	5,936882	5,329843	5,781747	5,20681	5,789023	5,7255977	5,592526667	-0,133071	0,911888287	---
17328070	4,432935	4,573096	4,660846	4,405662	4,580828	4,281179	4,5556257	4,422556333	-0,13306933	0,911889341	Tekt5
17402181	5,714198	5,739195	5,667599	5,798216	5,425507	5,498075	5,7069973	5,573932667	-0,13306467	0,91189229	F3
17382802	8,944401	9,310928	9,350843	9,057745	9,020313	9,128927	9,2020573	9,068995	-0,13306233	0,911893765	Ubac1
17515062	7,706008	7,739521	7,850122	7,494121	7,785723	7,61662	7,765217	7,632154667	-0,13306233	0,911893765	Mrpl4
17205549	5,387927	5,297824	5,227113	6,205372	4,270522	5,037788	5,304288	5,171227333	-0,13306067	0,911894819	---
17234812	5,32881	4,872993	5,234261	5,222196	4,936244	4,878443	5,1453547	5,012294333	-0,13306033	0,911895029	---
17240233	3,012941	3,034571	2,944695	2,935962	2,848838	2,808278	2,9974023	2,864359333	-0,133043	0,911905985	---
17501672	6,837051	6,987944	7,010716	6,698376	6,71106	7,027165	6,945237	6,812200333	-0,13303667	0,911909989	Zfp930
17203193	5,137904	5,266196	4,414266	4,869172	5,486264	4,06382	4,9394553	4,806418667	-0,13303667	0,911909989	---
17256436	6,019965	6,064904	6,022784	5,575029	5,950082	6,183485	6,0358843	5,902865333	-0,133019	0,911921156	Hspb9
17227480	5,496024	5,341319	5,732224	5,250196	5,390441	5,529919	5,523189	5,390185333	-0,13300367	0,911930848	Gpr25
17339889	7,702116	7,691959	7,408835	7,345592	7,455417	7,602892	7,60097	7,467967	-0,133003	0,911931269	Fam82a1
17523058	7,714168	7,85568	7,750283	7,733655	7,590365	7,597168	7,773377	7,640396	-0,132981	0,911945176	Xylb
17479927	3,696753	4,005663	3,456057	3,685268	3,438088	3,636199	3,719491	3,586518333	-0,13297267	0,911950443	Olfr290
17268834	6,854546	7,339342	6,768224	6,904191	6,765994	6,893086	6,9873707	6,854423667	-0,132947	0,911966668	Ormdl3
17344436	6,979645	7,020077	7,312227	6,94847	7,031746	6,933025	7,103983	6,971080333	-0,13290267	0,911994692	---
17550342	3,960967	4,309914	3,928644	3,75197	4,10309	3,945789	4,0665083	3,933616333	-0,132892	0,912001435	---
17354784	7,107382	7,260717	7,228684	7,195882	7,042476	6,959758	7,1989277	7,066038667	-0,132889	0,912003332	E330013P06
17376723	5,421302	5,294625	5,141815	5,362096	5,094215	5,002871	5,285914	5,153060667	-0,13285333	0,912025879	---
17385058	5,045896	4,980079	4,862581	4,797936	4,839542	4,852615	4,962852	4,830031	-0,132821	0,912046319	1700057H21Rik
17448738	3,31292	2,970321	3,153102	2,99624	3,293048	2,748616	3,1454477	3,012634667	-0,132813	0,912051377	---
17535108	3,273631	3,380176	3,550518	3,592363	3,201135	3,012422	3,4014417	3,26864	-0,13280167	0,912058541	---
17425280	5,025172	4,687266	4,553826	4,432339	4,789259	4,646295	4,7554213	4,622631	-0,13279033	0,912065706	C630028M04Rik
17312054	7,042016	6,741427	6,551768	6,357216	6,74808	6,831553	6,7784037	6,645616333	-0,13278733	0,912067603	Khdrbs3
17544535	5,971971	6,271501	5,748233	5,743083	5,931819	5,918518	5,997235	5,864473333	-0,13276167	0,912083829	Armcx6
17430915	7,376851	7,219387	7,359415	7,220849	7,358769	6,977878	7,318551	7,185832	-0,132719	0,912110804	Ppp1r8
17291402	4,081383	3,783793	4,116677	3,894767	3,722325	3,966655	3,993951	3,861249	-0,132702	0,912121552	1700092E19Rik
17486956	7,184855	6,844925	7,288683	6,718039	7,029802	7,172579	7,1061543	6,973473333	-0,132681	0,912134829	Slc1a5 // Slc1a5
17549370	4,628304	4,215187	4,685897	4,500116	4,2076	4,423636	4,509796	4,377117333	-0,13267867	0,912136304	---
17549748	4,628304	4,215187	4,685897	4,500116	4,2076	4,423636	4,509796	4,377117333	-0,13267867	0,912136304	---
17439643	5,92713	6,114098	5,600737	5,688279	5,781301	5,774386	5,880655	5,747988667	-0,13266633	0,912144102	---
17543988	7,100844	6,994818	6,868577	6,829527	6,905148	6,831606	6,9880797	6,855427	-0,13265267	0,912152743	Taf9b
17230062	3,415724	3,460728	3,582697	3,226817	3,478138	3,35625	3,486383	3,353735	-0,132648	0,912155693	Gm7897
17423972	6,108952	6,145709	6,111811	6,06098	5,749512	6,158052	6,1221573	5,989514667	-0,13264267	0,912159065	---
17306623	3,123283	2,99262	3,210158	3,026649	3,112753	2,788755	3,108687	2,976052333	-0,13263467	0,912164123	Mir208b
17331403	3,835113	3,827829	3,772923	3,627552	3,756809	3,653682	3,811955	3,679347667	-0,13260733	0,912181405	Csnka2ip
17407518	4,963273	4,936481	5,095242	4,907866	4,901483	4,788033	4,998332	4,865794	-0,132538	0,912225244	Gm9125
17274267	7,441721	7,664393	7,623588	7,395514	7,452729	7,483846	7,5765673	7,444029667	-0,13253767	0,912225455	Nol10
17200913	5,708529	6,035487	5,868541	6,203413	5,557829	5,453736	5,8708523	5,738326	-0,13252633	0,912232621	---
17331796	5,740713	5,657068	5,865816	5,559627	5,135011	6,171381	5,7545323	5,622006333	-0,132526	0,912232832	---
17473341	5,414006	5,343833	5,226482	5,387999	5,076536	5,122287	5,328107	5,195607333	-0,13249967	0,912249483	Brsk1
17214115	5,066149	4,803748	5,08503	4,913447	4,937863	4,706127	4,9849757	4,852479	-0,13249667	0,91225138	1700027A15Rik
17225356	3,970206	3,859125	3,785006	3,586246	3,656045	3,974576	3,8714457	3,738955667	-0,13249	0,912255595	Glrp1
17200101	3,748934	4,629729	4,308971	4,039721	4,246667	4,003792	4,2292113	4,096726667	-0,13248467	0,912258968	---
17291629	4,210738	3,762081	4,333335	3,736786	4,335978	3,835962	4,1020513	3,969575333	-0,132476	0,912264448	G630018N14Rik
17208441	4,934487	5,317023	5,955327	4,837219	5,468308	5,50401	5,402279	5,269845667	-0,13243333	0,912291428	---
17252404	11,1419	10,79967	10,80868	10,64325	10,81229	10,89751	10,91675	10,78435	-0,1324	0,912312507	---
17200287	2,901298	3,268346	2,558208	2,955559	2,894435	2,480782	2,909284	2,776925333	-0,13235867	0,912338645	---
17231613	3,952171	3,772438	3,905404	3,661958	3,887498	3,683484	3,876671	3,744313333	-0,13235767	0,912339277	Gm5177
17275378	4,946131	4,809512	4,976936	4,882721	4,693924	4,758911	4,9108597	4,778518667	-0,132341	0,912349817	Coch
17396069	6,786226	6,644163	6,925719	6,381044	6,719975	6,858125	6,7853693	6,653048	-0,13232133	0,912362254	Chmp4c
17392594	3,417762	3,397074	3,612293	3,267741	3,50799	3,254452	3,4757097	3,343394333	-0,13231533	0,912366049	Cst11
17238669	4,3344	3,845204	3,886041	4,207589	3,956312	3,504801	4,0218817	3,889567333	-0,13231433	0,912366681	Olfr788
17263024	5,044058	5,108208	4,869036	4,932193	4,788707	4,903478	5,0071007	4,874792667	-0,132308	0,912370686	Nmur2
17281497	7,011079	7,313391	7,225517	7,075567	6,969869	7,10765	7,183329	7,051028667	-0,13230033	0,912375535	1110034A24Rik
17334419	8,573319	8,669852	8,741808	8,604532	8,442818	8,540777	8,6616597	8,529375667	-0,132284	0,912385864	Sepx1
17350639	5,523465	5,274742	5,565665	5,393358	5,226687	5,347066	5,454624	5,322370333	-0,13225367	0,912405048	Prdm6
17477832	8,964189	9,684684	9,460983	9,028349	9,350744	9,334031	9,369952	9,237708	-0,132244	0,912411161	Ftl1 // Ftl1
17549002	8,437599	8,679174	8,483973	8,415066	8,316751	8,472246	8,533582	8,401354333	-0,13222767	0,912421491	Polr2k // Polr2k
17350032	4,456701	4,451294	4,728363	4,361441	4,262159	4,616141	4,5454527	4,413247	-0,13220567	0,912435405	Gm5820
17355316	7,756319	7,903312	7,574456	7,546067	7,653438	7,637984	7,7446957	7,612496333	-0,13219933	0,912439411	Poli
17222959	3,157759	3,185722	3,246139	3,10941	3,002563	3,08106	3,19654	3,064344333	-0,13219567	0,91244173	1700019A02Rik
17321241	11,70699	11,80121	11,74377	11,64455	11,6366	11,57427	11,750657	11,61847333	-0,13218333	0,91244953	Snora34
17405069	4,186701	3,839149	3,845136	4,058391	3,895611	3,520461	3,9569953	3,824821	-0,13217433	0,912455222	1700018B24Rik
17357883	4,063314	3,910381	4,57319	4,440642	3,766524	3,943242	4,182295	4,050136	-0,132159	0,91246492	---
17474682	5,97958	5,655123	5,662673	5,607436	5,554502	5,739019	5,765792	5,633652333	-0,13213967	0,912477148	---
17518543	5,762357	5,843847	5,623706	5,671801	5,580359	5,581336	5,7433033	5,611165333	-0,132138	0,912478202	---
17460958	7,375078	7,586041	7,405684	7,279095	7,336631	7,354682	7,455601	7,323469333	-0,13213167	0,912482208	C130022K22Rik
17375503	6,091889	6,091401	5,789556	5,688116	6,067194	5,821177	5,9909487	5,858829	-0,13211967	0,912489797	AA467197
17548844	4,823246	4,527497	4,693202	4,644073	4,310509	4,693009	4,681315	4,549197	-0,132118	0,912490852	Gm20289
17508162	9,205311	9,597277	8,95467	9,001463	9,073797	9,285646	9,2524193	9,120302	-0,13211733	0,912491273	---
17458133	6,958436	6,677861	6,889593	6,742109	6,78648	6,600961	6,8419633	6,70985	-0,13211333	0,912493803	Zfp212
17418560	6,852821	6,696116	6,907413	6,592371	6,716173	6,751473	6,8187833	6,686672333	-0,132111	0,912495279	Fam176b
17459901	8,997903	9,128064	9,235382	8,859911	9,092104	9,013062	9,1204497	8,988359	-0,13209067	0,91250814	---
17255482	4,002097	4,202368	4,247701	4,009428	3,960864	4,085654	4,150722	4,018648667	-0,13207333	0,912519103	---
17228943	5,862595	5,997269	5,907651	5,758152	5,850045	5,76312	5,922505	5,790439	-0,132066	0,912523742	Mettl13
17430762	5,805509	5,917254	5,897068	5,770763	5,728025	5,724847	5,873277	5,741211667	-0,13206533	0,912524163	Oprd1
17252726	3,440518	3,186749	3,300755	3,33919	3,118945	3,073717	3,3093407	3,177284	-0,13205667	0,912529645	Olfr397
17535710	6,151515	6,340838	6,397662	6,396205	5,927347	6,170335	6,2966717	6,164629	-0,13204267	0,9125385	---
17466279	3,774719	4,168114	3,791932	3,702198	3,425745	4,210738	3,9115883	3,779560333	-0,132028	0,912547778	Gm2663
17212882	8,130731	8,277073	8,12823	7,856955	7,954971	8,328085	8,178678	8,046670333	-0,13200767	0,912560639	Hspe1
17400281	7,469378	7,502084	7,451133	7,23636	7,338006	7,452246	7,4741983	7,342204	-0,13199433	0,912569073	Cdc42se1
17216498	4,326661	4,518229	4,3578	4,108231	4,341894	4,356629	4,4008967	4,268918	-0,13197867	0,912578983	Cdh7
17430833	8,38189	8,461569	8,527743	8,194906	8,523232	8,257223	8,4570673	8,325120333	-0,131947	0,912599014	Snora73a // Snora73a
17399649	6,647062	6,806441	6,390217	6,553531	6,484319	6,410116	6,6145733	6,482655333	-0,131918	0,912617358	Rab13
17207405	3,302441	3,544635	3,433131	3,587482	3,022651	3,274424	3,4267357	3,294852333	-0,13188333	0,912639288	---
17516621	6,708344	6,541564	6,698775	6,69446	6,263083	6,595555	6,649561	6,517699333	-0,13186167	0,912652994	Bcl9l
17204143	5,018916	4,895962	4,371916	4,58992	5,320494	3,980814	4,7622647	4,630409333	-0,13185533	0,912657001	---
17437087	7,580762	8,46785	7,783952	8,052631	7,748398	7,636001	7,944188	7,812343333	-0,13184467	0,912663749	Tmem128 // Tmem128
17323659	7,496228	7,747684	7,506164	7,516197	7,391496	7,446975	7,5833587	7,451556	-0,13180267	0,912690319	Trmt2a
17442307	5,167707	5,576221	5,425834	5,163068	5,336548	5,27478	5,3899207	5,258132	-0,13178867	0,912699176	Lrrc43
17409555	10,41417	10,36913	10,64908	10,87551	10,21727	9,944352	10,47746	10,34571067	-0,13174933	0,91272406	Amy2a5
17392561	8,135081	8,060035	8,04468	7,869699	8,091448	7,883465	8,079932	7,948204	-0,131728	0,912737556	Foxa2
17245171	11,21059	11,35957	11,32714	11,20601	11,15446	11,14171	11,2991	11,16739333	-0,13170667	0,912751053	Cct2
17278487	5,923464	6,152191	5,896999	6,078094	5,494712	6,004752	5,9908847	5,859186	-0,13169867	0,912756114	---
17234136	6,186509	6,149322	6,142399	5,936349	5,969755	6,177056	6,15941	6,02772	-0,13169	0,912761598	Slc16a9
17468627	7,00179	7,023405	7,327394	6,838995	7,142172	6,976363	7,1175297	6,985843333	-0,13168633	0,912763918	Prokr1
17235354	6,357393	5,963362	6,502332	6,090108	6,042698	6,295376	6,2743623	6,142727333	-0,131635	0,912796396	Gm19876
17286719	9,534728	9,63224	9,646009	9,401445	9,539947	9,47675	9,6043257	9,472714	-0,13161167	0,912811159	Pak1ip1
17492425	4,964556	4,847447	5,374075	4,971214	4,798597	5,021511	5,062026	4,930440667	-0,13158533	0,91282782	Mesp1
17550310	4,770925	4,580168	4,412049	4,631025	4,343258	4,394125	4,587714	4,456136	-0,131578	0,91283246	---

17255775	6,356508	6,354437	6,38444	6,170987	6,186846	6,342889	6,3651283	6,233574	-0,13155433	0,912847435	Socs7
17208105	4,61057	4,540795	4,310758	4,432288	4,488157	4,147034	4,4873743	4,355826333	-0,131548	0,912851442	---
17238210	9,349451	9,330702	9,210274	9,100051	9,18996	9,205827	9,296809	9,165279333	-0,13152967	0,912863043	Prim1
17236708	8,506113	8,562982	8,570026	8,367805	8,427639	8,449097	8,5463737	8,414847	-0,13152667	0,912864941	Ccdc41
17286472	9,003813	9,179578	9,04101	8,855639	9,00572	8,968523	9,0748003	8,943294	-0,13150633	0,912877807	Ripk1
17548677	7,968649	8,049072	8,020621	7,960886	7,999748	7,683275	8,0127807	7,881303	-0,13147767	0,912895946	Atp6v1b2 // Atp6v1b2
17448526	4,225492	4,059714	4,070814	4,146475	3,969603	3,845549	4,1186733	3,987209	-0,13146433	0,912904383	Kctd8
17488957	10,43933	10,44822	10,55759	10,42515	10,27424	10,35139	10,481713	10,35026	-0,13145333	0,912911344	Capns1
17444851	5,966315	5,905438	5,836486	6,038844	5,593156	5,681892	5,9027463	5,771297333	-0,131449	0,912914086	D5Ertd605e // D5Ertd605e
17343762	4,238727	4,290204	4,347972	4,211663	4,311726	3,959171	4,292301	4,160853333	-0,13144767	0,91291493	H2-Oa
17237795	3,243699	3,047175	3,277502	3,035398	3,156193	2,982467	3,1894587	3,058019333	-0,13143933	0,912920203	---
17494699	6,031888	5,496229	6,221057	5,734394	5,808504	5,812018	5,9163913	5,784972	-0,13141933	0,912932859	Olfr700
17503974	5,523417	5,575787	5,753015	5,6475	5,284046	5,526419	5,6174063	5,485988333	-0,131418	0,912933702	Slc12a3
17219466	10,94497	11,18801	11,26162	10,93837	11,17083	10,89117	11,131533	11,00012333	-0,13141	0,912938765	---
17329720	5,536801	5,233076	5,257114	5,467878	5,002753	5,162138	5,3423303	5,210923	-0,13140733	0,912940452	---
17208547	5,53544	6,392499	5,410702	5,877904	5,920523	5,146005	5,779547	5,648144	-0,131403	0,912943194	---
17331436	2,805938	3,076548	2,829864	2,823983	2,835242	2,658939	2,9041167	2,772721333	-0,13139533	0,912948046	---
17349944	3,677201	3,890363	3,721557	3,735376	3,712211	3,447357	3,7630403	3,631648	-0,13139233	0,912949944	Pcdhb21
17539060	3,591446	3,26503	3,473638	3,321615	3,361764	3,252601	3,4433713	3,311993333	-0,131378	0,912959015	---
17449519	5,820049	4,798677	5,890919	4,500975	5,939031	5,675579	5,503215	5,371861667	-0,13135333	0,912974624	---
17239371	8,731332	8,857756	8,756222	8,593991	8,715003	8,642299	8,78177	8,650431	-0,131339	0,912983695	Ltv1
17226109	3,218997	3,06328	3,206775	3,135671	3,021026	2,938381	3,1630173	3,031692667	-0,13132467	0,912992765	Serpinb3c
17291992	5,259048	5,0557	5,171596	5,148375	4,924165	5,019907	5,1621147	5,030815667	-0,131299	0,913009008	Ofcc1
17293961	4,553452	4,762084	4,524479	4,593725	4,262843	4,589589	4,6133383	4,482052333	-0,131286	0,913017235	Zfp429
17446515	4,261757	4,080557	4,258847	4,132483	4,031809	4,043028	4,200387	4,069106667	-0,13128033	0,913020822	4930584F24Rik
17274635	3,337893	3,535062	3,712456	3,361138	3,73863	3,091864	3,5284703	3,397210667	-0,13125967	0,913033901	---
17474956	7,051394	7,159823	7,295482	6,8933	7,171312	7,048357	7,1688997	7,037656333	-0,13124333	0,913044238	1500002O20Rik
17299397	3,776958	3,650323	3,737102	3,697306	3,632347	3,441016	3,721461	3,590223	-0,131238	0,913047613	4930447J18Rik
17502813	5,581427	5,343869	5,219034	5,244393	5,180832	5,325508	5,3814433	5,250244333	-0,131199	0,913072296	---
17433658	4,631711	5,059895	5,168336	4,916075	4,735021	4,815272	4,953314	4,822122667	-0,13119133	0,913077148	---
17372923	3,072937	2,912457	3,118544	2,885906	2,865589	2,958872	3,034646	2,903455667	-0,13119033	0,913077781	Olfr1202
17252011	4,865665	4,285407	4,585601	4,357498	4,477298	4,508442	4,578891	4,447746	-0,131145	0,913106472	Mir195
17465296	4,089398	4,101578	4,50236	4,05957	4,13532	4,105021	4,231112	4,099970333	-0,13114167	0,913108582	Mir592
17204569	6,088042	5,881998	6,433833	6,029568	6,209882	5,77104	6,1346243	6,003496667	-0,13112767	0,913117443	---
17280590	9,578491	9,670769	9,56164	9,391983	9,49198	9,533589	9,6036333	9,472517333	-0,131116	0,913124827	Prkar2b
17405142	6,406039	6,727185	6,590877	6,460976	6,542862	6,326928	6,5747003	6,443588667	-0,13111167	0,91312757	Setd7
17350684	4,620637	4,461176	4,020995	4,405707	4,032118	4,271733	4,3676027	4,236519333	-0,13108333	0,913145503	Gm4221
17470788	5,29437	4,923528	5,305068	5,071771	4,943898	5,114058	5,174322	5,043242333	-0,13107967	0,913147824	---
17471635	5,567039	5,84182	5,975484	5,567085	5,987856	5,436183	5,794781	5,663708	-0,131073	0,913152044	Klra17
17410061	5,068441	5,454317	5,434474	5,13938	5,529597	4,895049	5,3190773	5,188008667	-0,13106867	0,913154786	---
17337679	3,740047	3,649717	3,676423	3,390461	3,720872	3,561714	3,688729	3,557682333	-0,13104667	0,913168711	Gm17495
17535416	3,66471	3,376758	3,541754	3,43141	3,387544	3,371139	3,5277407	3,396697667	-0,131043	0,913171032	Magea4
17453269	5,271492	5,272661	5,137233	5,162013	5,2155	4,910778	5,2271287	5,096097	-0,13103167	0,913178206	---
17312339	6,656489	6,5302	6,535141	6,57993	6,267233	6,481601	6,5739433	6,442921333	-0,131022	0,913184325	---
17249481	7,531251	7,740602	7,253514	7,485445	7,208075	7,43879	7,5084557	7,377436667	-0,131019	0,913186223	---
17335419	5,199621	4,644247	4,721623	4,861764	4,672062	4,638625	4,8551637	4,724150333	-0,13101333	0,91318981	Pnpla1
17306863	9,314856	9,893587	9,924167	9,730293	9,551737	9,457555	9,71087	9,579861667	-0,13100833	0,913192975	---
17385486	5,987133	6,06749	5,722975	5,739764	5,94361	5,701217	5,925866	5,794863667	-0,13100233	0,913196773	Wdsub1
17334200	5,26815	4,898605	5,649292	5,423417	5,049102	4,950716	5,2720157	5,141078333	-0,13093733	0,913237918	BC028777
17374722	7,659725	7,642222	7,601313	7,522336	7,45139	7,53674	7,63442	7,503488667	-0,13093133	0,913241716	Rad51
17245686	5,146946	4,8639	5,153218	4,719582	4,906226	5,145637	5,054688	4,923815	-0,130873	0,913278642	LOC100504608
17255853	7,132122	7,018353	6,975644	6,964201	6,841614	6,927687	7,0420397	6,911167333	-0,13087233	0,913279064	Cisd3
17317212	11,02857	11,23251	11,17358	10,9782	11,13217	10,93168	11,144887	11,01401667	-0,13087	0,913280541	---
17316428	5,211733	5,06181	5,098768	5,037412	4,929551	5,012777	5,1241037	4,993246667	-0,130857	0,913288771	BC048602 // BC048602
17544373	5,05773	5,026255	5,270159	4,759843	5,143562	5,05819	5,118048	4,987198333	-0,13084967	0,913293413	Pcdh19
17203847	3,13078	2,957658	2,91975	2,951128	2,772349	2,892196	3,0027293	2,871891	-0,13083833	0,913300588	---
17367237	4,613227	4,117736	4,805258	4,4847	4,107602	4,551413	4,5120737	4,381238333	-0,13083533	0,913302487	A930004D18Rik
17338946	7,79897	7,59461	7,402616	7,315705	7,702303	7,385767	7,598732	7,467925	-0,130807	0,913320423	Crb3
17211883	3,243429	3,580437	3,508421	3,476561	3,12983	3,333494	3,4440957	3,313295	-0,13080067	0,913324433	---
17376588	4,70694	4,77315	4,973629	4,867866	4,645169	4,548288	4,8179063	4,687107667	-0,13079867	0,913325699	1700026D11Rik
17225809	5,231937	5,417103	5,174529	5,191011	5,068215	5,171954	5,274523	5,143726667	-0,13079633	0,913327176	---
17378958	6,649811	6,70149	6,938596	6,721029	6,740766	6,43575	6,763299	6,632515	-0,130784	0,913334984	Fam83d
17362240	10,03711	10,15163	9,924331	9,88331	9,830708	10,00676	10,03769	9,906926	-0,13076433	0,913347435	Stip1
17337202	5,115459	4,908585	4,986751	5,071485	4,653901	4,893126	5,0035983	4,872837333	-0,130761	0,913349545	2300002M23Rik
17280483	10,06577	9,896233	9,996243	9,836311	9,786062	9,943649	9,986082	9,855340667	-0,13074133	0,913361996	Acp1
17543082	4,69684	4,473492	4,390918	4,210737	4,423656	4,534639	4,5204167	4,389677333	-0,13073933	0,913363262	Gm41
17474078	5,154707	5,297001	5,267666	4,9045	5,194191	5,228528	5,2397913	5,109073	-0,13071833	0,913376557	Meis3
17247356	4,380944	4,043782	4,363331	4,084911	4,145442	4,165556	4,2626857	4,131969667	-0,130716	0,913378034	---
17372614	7,400794	7,582381	7,410574	7,482399	7,202433	7,316838	7,464583	7,33389	-0,130693	0,913392596	Timm10
17341305	3,742438	3,522781	3,69139	3,479589	3,661859	3,423083	3,652203	3,521510333	-0,13069267	0,913392807	Vmn2r105
17261177	4,365967	4,124714	4,665555	4,315527	4,219152	4,229524	4,385412	4,254734333	-0,13067767	0,913402304	---
17520063	4,797978	4,8497	5,183409	4,5703	5,170998	4,697798	4,9436957	4,813032	-0,13066367	0,913411167	Mrap2
17549188	5,1114	5,645527	5,310719	5,464494	5,040658	5,170534	5,355882	5,225228667	-0,13065333	0,91341771	---
17543139	3,077757	3,172132	3,398219	3,032793	2,795429	3,427933	3,216036	3,085385	-0,130651	0,913419187	Mageb1
17310398	3,755532	3,981219	4,03913	3,623223	4,176133	3,584606	3,9252937	3,794654	-0,13063967	0,913426363	---
17308413	8,487254	8,588198	8,615176	8,245129	8,45657	8,597044	8,5635427	8,432914333	-0,13062833	0,913433538	Epb4.9
17372959	3,776881	3,560169	3,406323	3,559987	3,289996	3,501575	3,5811243	3,450519333	-0,130605	0,913448312	Olfr1262
17500702	3,200019	3,165384	3,359732	3,137311	3,099005	3,097037	3,2417117	3,111117667	-0,130594	0,913455276	Adam25
17248793	4,412443	4,378942	4,673818	4,361349	4,278105	4,433969	4,488401	4,357807667	-0,13059333	0,913455698	Fam71b
17360001	3,647485	3,566366	3,551532	3,565152	3,395339	3,413116	3,588461	3,457869	-0,130592	0,913456543	---
17551135	9,856631	9,838724	9,696346	9,577934	9,737208	9,684839	9,7972337	9,666660333	-0,13057333	0,913468362	---
17283463	7,069907	7,154937	7,037032	6,743676	7,00801	7,118488	7,087292	6,956724667	-0,13056733	0,913472161	Itpk1
17360115	7,748247	8,112103	7,830606	7,772302	7,802433	7,724525	7,8969853	7,76642	-0,13056533	0,913473427	Taf5
17211458	5,74749	5,754508	5,686619	5,580379	5,636727	5,579825	5,729539	5,598977	-0,130562	0,913475538	Col9a1
17516189	2,980598	3,017323	3,0451	2,897889	2,873281	2,880169	3,0143403	2,883779667	-0,13056067	0,913476382	Olfr913
17427883	4,235921	4,551652	4,313832	4,061263	4,12544	4,523091	4,367135	4,236598	-0,130537	0,913491367	---
17233331	9,219178	9,259401	9,236589	9,054177	9,130984	9,138432	9,2383893	9,107864333	-0,130525	0,913498965	Asf1a
17536624	6,745951	6,468403	6,356958	6,473042	6,271368	6,435336	6,5237707	6,393248667	-0,130522	0,913500865	Dlg3
17438875	5,829298	5,399771	5,395241	5,340318	5,274148	5,61828	5,5414367	5,410915333	-0,13052133	0,913501287	Gm7056
17315225	6,48002	6,961481	6,542123	5,940455	6,39079	6,260855	6,3278747	6,197366667	-0,130508	0,91350973	Krt86
17357276	7,400479	7,571822	7,418333	7,370839	7,19107	7,437252	7,4635447	7,333053667	-0,130491	0,913520494	Ints5
17304794	11,36652	11,30027	11,2789	11,25681	11,11495	11,18246	11,31523	11,18474	-0,13049	0,913521127	Hacl1 // Hacl1
17212049	4,838681	4,497307	4,686476	4,49234	4,447233	4,691483	4,6741547	4,543685333	-0,13046933	0,913534214	---
17502789	12,76771	12,85599	12,68938	12,51013	12,60442	12,80713	12,771027	12,64056	-0,13046667	0,913535902	Gypa
17235714	6,715199	6,719026	6,760514	6,692626	6,556256	6,554493	6,7315797	6,601125	-0,13045467	0,913543501	Dohh
17421168	4,243431	4,986766	4,408346	4,511551	4,129324	4,606353	4,546181	4,415742667	-0,13043833	0,913553843	---
17549640	4,382212	4,29475	4,368721	4,163973	4,211415	4,278981	4,348561	4,218123	-0,130438	0,913554055	---
17459843	5,667578	5,457877	5,583836	5,515588	5,344993	5,4574	5,5697637	5,439327	-0,13043667	0,913554899	Tacr1
17447150	7,695095	7,857295	7,784646	7,674423	7,68317	7,588209	7,779012	7,648600667	-0,13041133	0,913570941	Letm1
17363160	3,214332	3,20014	3,200937	2,974193	3,172861	3,07714	3,2051363	3,074731333	-0,130405	0,913574951	---
17447960	4,390803	4,537461	4,674871	4,410931	4,508271	4,292724	4,5343783	4,403975333	-0,130403	0,913576218	---
17241655	3,502687	3,519539	3,6691	3,554708	3,34819	3,397221	3,5637753	3,433373	-0,13040233	0,91357664	Rtkn2
17380953	7,120692	7,121116	6,974095	6,709196	6,97374	7,141765	7,0719677	6,941567	-0,13040067	0,913577695	Ppdpf
17256342	6,946305	6,893061	6,9497	6,810408	6,720952	6,866518	6,9296887	6,799292667	-0,130396	0,91358065	---
17243091	5,714598	5,944462	6,387127	5,837276	5,951067	5,866735	6,0153957	5,885026	-0,13036967	0,913597326	---
17209061	4,682921	3,439882	3,678575	3,410987	3,840975	4,15832	3,9337927	3,803427333	-0,13036533	0,91360007	---
17318942	5,885733	5,498567	5,778769	5,425372	5,622207	5,724491	5,721023	5,59069	-0,130333	0,913620546	Pvalb
17350581	4,416091	4,291101	4,389	4,263031	4,130307	4,311891	4,3653973	4,235076333	-0,130321	0,913628145	1700034E13Rik
17208001	2,954173	3,000852	2,911283	3,001975	2,769621	2,703882	2,955436	2,825159333	-0,13027667	0,913656221	---
17275237	8,706523	8,63299	8,556663	8,558072	8,416576	8,530699	8,6320587	8,501782333	-0,13027633	0,913656432	Pnpla8
17456772	4,999389	5,070336	5,180535	4,837375	5,108916	4,913165	5,08342	4,953152	-0,130268	0,91366171	Fam40b
17253145	5,192132	5,054336	4,985776	4,984061	4,945215	4,912252	5,0774147	4,947176	-0,13023867	0,913680287	Dbil5
17513503	7,591374	7,528511	7,402488	7,379566	7,234267	7,517923	7,5074577	7,377252	-0,13020567	0,913701186	Zdhhc7
17206125	6,460904	5,796992	5,433184	5,788768	5,599813	5,911893	5,8970267	5,766824667	-0,130202	0,913703508	---
17465227	2,882504	3,000852	3,202659	2,815714	3,018263	2,861462	3,0286717	2,898479667	-0,130192	0,913709842	---
17524326	3,80433	3,553593	3,811077	3,607541	3,630206	3,540741	3,723	3,592829333	-0,13017067	0,913723353	Olfr828
17243839	4,329585	4,095739	4,406892	4,145193	4,286592	4,009931	4,2774053	4,147238667	-0,13016667	0,913725886	Ascl1
17205041	3,296958	3,461027	3,278305	3,163656	2,932075	3,550088	3,34543	3,215273	-0,130157	0,913732009	---
17209231	3,296958	3,461027	3,278305	3,163656	2,932075	3,550088	3,34543	3,215273	-0,130157	0,913732009	---
17539064	5,099742	5,515756	5,443594	5,447272	5,135161	5,086272	5,3530307	5,222901667	-0,130129	0,913749743	Acot9
17529705	5,919864	5,550607	6,072741	5,792043	5,761112	5,599681	5,8477373	5,717612	-0,13012533	0,913752065	---
17295788	7,659557	8,074258	7,930367	7,778105	7,724488	7,771217	7,8880607	7,757936667	-0,130124	0,91375291	Gm10257
17214400	5,949743	5,659108	5,680664	5,550039	5,828822	5,520317	5,7631717	5,633059333	-0,13011233	0,913760299	Cdk5r2
17350086	3,915976	4,223015	3,880136	3,859212	3,871157	3,898422	4,0063757	3,876263667	-0,130112	0,91376051	---
17495303	10,18791	10,23259	10,13264	10,06325	10,11751	9,982095	10,18438	10,054285	-0,130095	0,913771277	---
17280187	4,997572	4,928235	5,039125	4,955971	4,860029	4,758684	4,9883107	4,858228	-0,13008267	0,913779089	Itgb1bp1 // Itgb1bp1
17516617	6,019146	6,049334	6,128657	5,979423	5,878311	5,949235	6,0657123	5,935656333	-0,130056	0,913795979	C030014I23Rik
17548010	9,257759	9,655718	9,519334	9,377604	9,275955	9,389097	9,4776037	9,347552	-0,13005167	0,913798724	Gm19399
17298968	5,786291	5,934608	5,86011	5,747429	5,418768	6,024702	5,8603363	5,730299667	-0,13003667	0,913808225	Sftpa1
17481133	4,733354	4,687794	4,727108	4,485825	4,676025	4,596384	4,7160853	4,586078	-0,13000733	0,913826805	Olfr552
17497146	5,958246	6,120423	6,199891	5,947079	6,06069	5,880791	6,0928533	5,962853333	-0,13	0,91383145	Zranb1
17323653	4,733163	4,856191	4,905815	4,749299	4,548334	4,807587	4,831723	4,70174	-0,129983	0,913842218	---
17396823	10,08544	10,23215	10,06987	9,961189	10,03932	9,997082	10,129153	9,999197	-0,12995633	0,91385911	Fxr1
17431502	8,6406	8,596033	8,88684	8,528805	8,575417	8,629406	8,7078243	8,577876	-0,12994833	0,913864177	Lypla2

17305432	5,390811	5,270228	5,289155	5,598689	5,024969	4,936749	5,3167313	5,186802333	-0,129929	0,913876424	Gm10377
17219546	5,84968	6,044183	6,134047	5,954504	5,91853	5,765095	6,0093033	5,879376333	-0,129927	0,913877691	Slamf9
17260618	4,587818	4,425877	4,663692	4,407871	4,54806	4,331723	4,559129	4,429218	-0,129911	0,913887826	---
17337089	5,556976	5,565242	5,751729	5,549088	5,406589	5,528583	5,624649	5,494753333	-0,12989567	0,913897539	H2-Q1 // H2-Q1
17392110	4,594269	4,50132	4,701872	4,73854	4,253013	4,416247	4,5991537	4,469266667	-0,129887	0,913903029	9630028H03Rik
17499530	4,474524	4,455027	4,666543	4,328788	4,541426	4,33623	4,5320313	4,402148	-0,12988333	0,913905352	---
17455492	5,556971	7,684678	4,945648	7,420846	5,076602	5,3002	6,0624323	5,932549333	-0,129883	0,913905563	Gm8615
17346760	8,411885	8,64815	8,450854	8,283978	8,361856	8,475407	8,5036297	8,373747	-0,12988267	0,913905774	Ralbp1
17451568	8,090178	8,22395	8,350034	8,19689	7,837515	8,240294	8,2213873	8,091566333	-0,129821	0,913944839	Gltp
17308881	8,646877	8,694719	8,703403	8,340548	8,634226	8,680762	8,6816663	8,551845333	-0,129821	0,913944839	1190002H23Rik
17486203	4,98299	4,949919	5,233851	4,843019	5,024571	4,909787	5,0555867	4,925792333	-0,12979433	0,913961733	---
17496728	5,963212	5,808894	5,748508	5,836332	5,786679	5,508255	5,8402047	5,710422	-0,12978267	0,913969124	Zfp629
17201869	7,803663	7,593111	7,617321	7,80964	7,463355	7,351827	7,671365	7,541607333	-0,12975767	0,913984962	---
17371636	5,781058	6,010671	6,108696	5,566129	5,967406	5,977688	5,9668083	5,837074333	-0,129734	0,913999955	Gad1
17513525	7,364348	7,294456	7,214934	7,136724	7,125014	7,222883	7,291246	7,161540333	-0,12970567	0,914017906	Gins2
17401036	5,171392	4,928868	5,058106	4,990331	4,95507	4,823884	5,0527887	4,923095	-0,12969367	0,914025508	Nhlh2
17482290	4,351804	4,059022	4,418612	4,053716	4,246848	4,139856	4,2764793	4,146806667	-0,12967267	0,914038813	Acsm4
17334636	5,081735	4,868165	5,109266	5,100219	4,81854	4,751437	5,019722	4,890065333	-0,12965667	0,91404895	Gm17801
17537260	3,858344	3,821154	3,824305	3,7376	3,742712	3,63457	3,834601	3,704960667	-0,12964033	0,914059299	2010106E10Rik
17293475	4,730604	4,950552	5,111772	4,797585	4,832272	4,774166	4,930976	4,801341	-0,129635	0,914062678	Gm19792
17251430	6,065906	6,152378	5,880299	5,933926	5,985175	5,790579	6,032861	5,903226667	-0,12963433	0,9140631	Rpl26
17401180	8,26997	8,477153	8,141397	8,157245	8,111507	8,230892	8,2961733	8,166548	-0,12962533	0,914068802	Trim33
17403848	8,943198	9,123507	9,102314	8,90573	8,882073	8,992344	9,0563397	8,926715667	-0,129624	0,914069647	Zranb2
17403809	3,296865	3,061017	3,02367	3,004847	2,931816	3,056029	3,127184	2,997564	-0,12962	0,914072181	---
17375161	6,6204	6,822017	6,660191	6,537186	6,681643	6,494953	6,7008693	6,571260667	-0,12960867	0,914079362	Tmem62
17249347	8,397482	8,529457	8,430725	8,300852	8,374067	8,293939	8,4525547	8,322952667	-0,129602	0,914083586	Nhp2
17250183	7,331702	7,136362	7,156931	7,00301	7,181888	7,051329	7,2083317	7,078742333	-0,12958933	0,914091612	Trim11
17218004	3,356495	2,979766	3,310095	3,191159	2,924753	3,14169	3,215452	3,085867333	-0,12958467	0,914094568	---
17417347	6,159095	5,796869	5,855747	5,834872	5,687763	5,900349	5,937237	5,807661333	-0,12957567	0,914100271	---
17254616	5,273736	5,326356	5,330205	5,174183	5,073216	5,294175	5,310099	5,180524667	-0,12957433	0,914101116	---
17357475	5,826262	6,213194	6,18417	5,518232	6,024557	6,292115	6,074542	5,944968	-0,129574	0,914101327	Fen1 // Fen1
17315606	5,445934	5,574059	5,881681	5,502652	5,469915	5,540413	5,6338913	5,504326667	-0,12956467	0,914107241	Pde1b
17421788	5,55685	5,874881	5,669949	5,587474	5,577064	5,548473	5,70056	5,571003667	-0,12955633	0,914112521	---
17312219	9,172504	9,90784	9,539506	9,04587	9,693753	9,491565	9,53995	9,410396	-0,129554	0,914113999	Ly6g
17363670	4,290203	3,921565	4,322303	4,216066	3,944892	3,984492	4,1780237	4,048483333	-0,12954033	0,914122659	2610016A17Rik
17291821	5,391105	5,605354	5,485761	5,194439	5,503699	5,395474	5,4940733	5,364537333	-0,129536	0,914125404	Eci3
17365382	4,825026	4,917773	4,779312	4,757464	4,680516	4,695561	4,8407037	4,711180333	-0,12952333	0,91413343	2310034G01Rik
17548482	4,619174	4,475623	4,51312	4,544452	4,244442	4,430497	4,5359723	4,406463667	-0,12950867	0,914142723	Gm20382
17492179	4,909222	4,974339	4,748034	4,615555	5,050588	4,576993	4,8771983	4,747712	-0,12948633	0,914156875	---
17441130	6,781928	6,916757	6,942708	6,656931	6,871672	6,724398	6,8804643	6,751000333	-0,129464	0,914171026	Triap1
17362356	4,001684	3,912364	4,048448	3,950749	3,823191	3,800186	3,9874987	3,858042	-0,12945667	0,914175673	Slc22a19
17440633	5,81537	5,716958	5,74597	5,414065	5,64728	5,828643	5,7594327	5,629996	-0,12943667	0,914188346	Mn1
17537455	5,289701	5,950479	5,772795	5,278022	5,848024	5,498645	5,6709917	5,541563667	-0,129428	0,914193838	Gm17361
17544297	5,289701	5,950479	5,772795	5,278022	5,848024	5,498645	5,6709917	5,541563667	-0,129428	0,914193838	---
17544305	5,289701	5,950479	5,772795	5,278022	5,848024	5,498645	5,6709917	5,541563667	-0,129428	0,914193838	---
17544313	5,289701	5,950479	5,772795	5,278022	5,848024	5,498645	5,6709917	5,541563667	-0,129428	0,914193838	---
17544321	5,289701	5,950479	5,772795	5,278022	5,848024	5,498645	5,6709917	5,541563667	-0,129428	0,914193838	Gm17361
17248925	6,095599	5,477898	5,186512	5,638731	4,896245	5,836821	5,5866697	5,457265667	-0,129404	0,914209046	Olfr1395
17459347	6,460091	5,957265	6,028203	6,004913	5,937815	6,114625	6,1485197	6,019117667	-0,129402	0,914210314	Igkv15-103
17371099	3,809711	4,236797	4,252876	3,927093	3,91813	4,065957	4,0997947	3,970393333	-0,12940133	0,914210736	---
17337580	3,38101	3,68573	2,955851	3,183199	3,393334	3,057926	3,3408637	3,211486333	-0,12937733	0,914225945	Olfr111
17548690	10,71884	11,09879	10,8711	10,59013	10,71469	10,99582	10,896243	10,76688	-0,12936333	0,914234816	Gm6625
17309022	6,624074	6,572106	6,384228	5,526347	6,805635	6,860352	6,5268027	6,397444667	-0,129358	0,914238196	---
17388650	3,806414	4,030458	3,614371	3,691842	3,825804	3,545689	3,817081	3,687778333	-0,12930267	0,914273262	---
17469121	6,244564	6,102822	6,319178	5,914196	6,106418	6,258042	6,222188	6,092885333	-0,12930267	0,914273262	Prickle2
17264634	8,778797	8,976601	9,291664	9,068342	8,87182	8,719017	9,0156873	8,886393	-0,12929433	0,914278543	---
17441755	5,189694	5,219256	5,037232	4,703628	5,240428	5,114256	5,1487273	5,019437333	-0,12929	0,914281289	Dtx1
17355825	7,701644	7,908232	7,632352	7,601392	7,682124	7,570844	7,7474093	7,61812	-0,12928933	0,914281711	Cndp2
17481535	3,844256	3,852671	3,863921	3,631705	3,871872	3,669481	3,853616	3,724352667	-0,12926333	0,914298189	Gm15514
17304850	6,613993	6,659864	6,873023	6,55971	6,609062	6,59036	6,7156267	6,586377333	-0,12924933	0,914307061	Oxnad1 // Oxnad1
17459398	3,534675	3,679227	3,497067	3,603787	3,473138	3,246359	3,570323	3,441094667	-0,12922833	0,91432037	---
17523279	5,950552	6,005377	6,183083	6,049861	5,934664	5,766872	6,0463373	5,917132333	-0,129205	0,914335158	---
17235682	7,286176	7,156048	7,386563	7,138695	7,096532	7,205959	7,2762623	7,147062	-0,12920033	0,914338115	2510012J08Rik
17356412	5,226201	5,237287	5,373872	5,069171	5,182844	5,197879	5,27912	5,149964667	-0,12915533	0,914366635	4930481A15Rik
17462224	6,547523	5,609616	6,558518	5,812785	6,389061	6,126355	6,2385523	6,109400333	-0,129152	0,914368748	---
17298183	8,983943	9,129163	9,23401	8,999805	9,033013	8,926899	9,1157053	8,986572333	-0,129133	0,91438079	Tkt
17516852	6,822075	6,808016	6,597047	6,492844	6,617592	6,729344	6,7423793	6,61326	-0,12911933	0,914389452	Pcsk7
17201211	8,30868	7,978572	7,620691	7,709406	7,782328	8,028868	7,9693143	7,840200667	-0,12911367	0,914393044	---
17473941	6,33746	6,471724	6,105716	5,821532	6,185629	6,520435	6,3049667	6,175865333	-0,12910133	0,914400861	Sult2a2
17204083	3,122408	2,861063	2,861059	2,735125	3,002438	2,720004	2,9481767	2,819189	-0,12898767	0,914472907	---
17548266	5,751182	6,005639	5,563357	5,493618	5,571881	5,867795	5,7733927	5,644431333	-0,12896133	0,914489599	Mrpl47
17206215	4,420093	3,115842	4,108797	3,809151	3,851957	3,596816	3,8815773	3,752641333	-0,128936	0,914505657	---
17391470	9,809267	9,505524	9,506516	9,29773	9,681863	9,455029	9,6071023	9,478207333	-0,128895	0,914531647	---
17218923	4,449352	4,158754	4,367561	4,357482	4,000248	4,231295	4,3252223	4,196341667	-0,12888067	0,914540733	---
17439479	3,308237	2,937498	3,021195	3,078625	2,81196	2,989717	3,0889767	2,960100667	-0,128876	0,914543691	---
17291500	4,279179	4,063273	3,95895	3,967642	3,838237	4,109025	4,1004673	3,971634667	-0,12883267	0,914571161	Prl7c1
17235408	7,808568	7,746744	7,802397	7,673609	7,616714	7,680907	7,785903	7,657076667	-0,12882633	0,914575176	Dot1l
17208711	4,096281	3,889328	4,285151	3,959307	4,06089	3,864097	4,0902533	3,961431333	-0,128822	0,914577923	---
17252359	5,579278	5,443523	5,82628	5,56185	5,507491	5,393321	5,6163603	5,487554	-0,12880633	0,914587855	Ggt6
17551109	9,233493	9,253261	9,209105	9,085604	9,073611	9,15027	9,231953	9,103161667	-0,12879133	0,914597364	---
17427073	6,276276	6,006334	6,357207	6,270058	6,017161	5,966241	6,2132723	6,084486667	-0,12878567	0,914600957	Ifna14
17374807	5,640349	5,573403	5,723162	5,497867	5,4695	5,583232	5,645638	5,516866333	-0,12877167	0,914609832	Chac1
17389928	7,897152	8,030787	7,843727	7,567337	7,891524	7,926551	7,9238887	7,795137333	-0,12875133	0,914622723	Ndufaf1
17438963	12,33516	12,10741	12,14815	11,98063	12,09642	12,12743	12,196907	12,06816	-0,12874667	0,914625681	Ppbp
17458771	9,998229	9,971945	10,19317	9,894064	9,987297	9,895799	10,054448	9,92572	-0,128728	0,914637515	Gars
17502992	6,967681	6,628091	6,81408	6,809395	6,740285	6,474028	6,803284	6,674569333	-0,12871467	0,914645968	---
17477960	6,44888	5,954129	6,04972	6,345765	5,731089	5,989841	6,1509097	6,022231667	-0,128678	0,914669215	Ntn5
17516261	4,214839	3,716016	4,170636	4,060319	3,790336	3,864882	4,0338303	3,905179	-0,12865133	0,914686122	Olfr944
17287081	5,893307	5,930412	6,043533	5,739892	5,757868	5,983582	5,9557507	5,827114	-0,12863667	0,914695421	Id4
17266586	4,954373	4,625492	4,633042	4,622727	4,672697	4,531583	4,7376357	4,609002333	-0,12863333	0,914697534	Omg
17387316	9,975625	10,01777	9,83884	9,840534	9,78387	9,821958	9,9440783	9,815454	-0,12862433	0,91470324	Nckap1
17418753	5,118335	4,930423	4,992142	5,099526	4,779019	4,776488	5,0136333	4,885011	-0,12862233	0,914704508	4930471C06Rik
17508025	8,11059	8,211181	8,214221	8,008039	7,974976	8,167165	8,178664	8,05006	-0,128604	0,914716132	Ckap2
17205569	3,15996	4,270257	3,284928	3,702573	2,987716	3,639045	3,571715	3,443111333	-0,12860367	0,914716343	---
17226089	4,067986	3,672373	3,555587	3,624959	3,66291	3,622299	3,7653153	3,636722667	-0,12859267	0,914723318	Serpinb3d
17538425	5,912022	5,813353	5,708655	5,705819	5,630583	5,711852	5,8113433	5,682751333	-0,128592	0,914723741	Snora35
17321336	5,33471	4,969406	5,279266	4,992865	5,051929	5,152871	5,1944607	5,065888333	-0,12857233	0,91473621	Wnt10b
17534135	5,731134	5,897757	6,577322	5,790167	6,196794	5,833607	6,0687377	5,940189333	-0,12854833	0,914751427	Sowahd
17493275	4,78969	4,912669	5,170137	4,683728	4,859429	4,943767	4,9574987	4,828974667	-0,128524	0,914766856	Ccdc83
17472288	4,400628	4,506488	4,206663	4,252462	4,087989	4,387777	4,3712597	4,242742667	-0,128517	0,914771295	Lmo3
17252272	7,951169	8,059359	8,051546	7,804662	7,925974	7,945898	8,0206913	7,892178	-0,12851333	0,91477362	Mis12
17282689	6,971641	6,973909	6,994608	6,949284	6,805302	6,80004	6,9800527	6,851542	-0,12851067	0,91477531	Acyp1
17324394	8,996613	9,39501	9,143238	8,985142	9,118382	9,045832	9,178287	9,049785333	-0,12850167	0,914781017	Snord2
17211973	7,936214	8,240408	8,185671	8,017553	8,120799	7,838454	8,1207643	7,992268667	-0,12849567	0,914784822	Unc50
17397240	8,2371	8,382989	8,320848	8,217209	8,063477	8,274805	8,3136457	8,185163667	-0,128482	0,914793487	Hspa4l
17303568	9,624511	9,622672	9,62856	9,547404	9,443355	9,49955	9,6252477	9,496769667	-0,128478	0,914796024	Thoc7
17285660	5,416383	5,386605	5,330836	5,121278	5,261727	5,365404	5,3779413	5,249469667	-0,12847167	0,91480004	Zkscan4
17323576	5,444824	5,128633	5,754405	5,272483	5,366091	5,303965	5,4426207	5,314179667	-0,128441	0,914819485	Ccdc74a
17397511	6,699348	6,996041	6,846087	6,682812	6,701377	6,772018	6,8471587	6,718735667	-0,128423	0,914830899	Lhfp
17239040	6,751775	6,792369	6,550244	6,466423	6,409729	6,832968	6,6981293	6,569706667	-0,12842267	0,914831111	Ulbp1
17501574	4,071084	3,707682	4,291627	3,781138	4,087	3,816994	4,0234643	3,895044	-0,12842033	0,91483259	Nat3
17420996	5,190099	5,12557	5,333002	4,861198	5,251609	5,150615	5,2162237	5,087807333	-0,12841633	0,914835127	6330545A04Rik
17498075	4,157165	4,256265	4,452884	4,358087	4,029749	4,093265	4,2887713	4,160367	-0,12840433	0,914842736	---
17270730	7,238413	7,306387	7,180827	7,157352	7,084349	7,098765	7,2418757	7,113488667	-0,128387	0,914853728	Limd2
17265473	3,538036	3,621045	3,506346	3,406167	3,40476	3,469446	3,5551423	3,426791	-0,12835133	0,914876345	Nlrp1a
17432393	5,918894	5,835785	6,098315	5,81456	5,754726	5,898658	5,950998	5,822648	-0,12835	0,914877191	---
17518340	3,11044	3,101449	3,180571	2,819972	3,093657	3,093784	3,13082	3,002471	-0,128349	0,914877825	---
17463670	5,709258	5,805239	5,87681	5,496478	5,760933	5,74887	5,7971023	5,668760333	-0,128342	0,914882264	LOC100862607
17261231	7,48752	7,60948	7,391273	7,298203	7,421576	7,383618	7,496091	7,367799	-0,128292	0,914913972	---
17221995	4,861117	4,811715	4,885271	4,811715	4,657104	4,704458	4,852701	4,724425667	-0,12827533	0,914924541	Gm5415
17216723	3,145921	3,572626	3,370353	3,30558	3,190518	3,208034	3,3629667	3,234710667	-0,128256	0,914936802	---
17483165	6,304574	6,413333	6,409161	6,215047	6,324343	6,202962	6,3756893	6,247450667	-0,12823867	0,914947795	Hirip3
17303472	12,63426	12,56123	12,46013	12,42405	12,3892	12,45767	12,551873	12,42364	-0,12823333	0,914951177	Ptma
17388499	5,660871	5,571643	5,499127	5,529785	5,314116	5,503074	5,5772137	5,448991667	-0,128222	0,914958365	Accs
17349624	4,523933	4,385842	4,461515	4,503258	4,263869	4,219505	4,4570967	4,328877333	-0,12821933	0,914960056	4930471G03Rik
17354473	6,466757	6,829788	6,314685	6,41702	6,356459	6,45312	6,5370767	6,408866333	-0,12821033	0,914965764	C330018D20Rik
17468425	4,80665	4,856036	5,204824	4,69566	4,812057	4,975227	4,9558367	4,827648	-0,12818867	0,914979505	Gm11128
17406718	5,386982	5,246784	5,503867	5,459877	5,263396	5,029819	5,379211	5,251030667	-0,12818033	0,91498479	Bglap-rs1 // Bglap-rs1
17237596	5,689569	5,470393	5,695137	5,435037	5,672017	5,363533	5,6183663	5,490195667	-0,12817067	0,914990921	Dyrk2
17322473	5,710558	5,904578	6,092569	5,729611	5,710586	5,883047	5,9025683	5,774414667	-0,12815367	0,915001703	Zfp597 // Zfp597
17413789	9,04319	9,0573	8,934111	8,775236	8,914419	8,960527	9,0115337	8,883394	-0,12813967	0,915010582	Ncbp1
17281927	5,202997	4,987053	4,799849	5,01997	4,752436	4,833162	4,996633	4,868522667	-0,12811033	0,915029187	Kcnh5
17544625	7,096632	6,801862	7,170576	6,992025	6,837516	6,855336	7,0230233	6,894959	-0,12806433	0,915058363	---
17544627	7,096632	6,801862	7,170576	6,992025	6,837516	6,855336	7,0230233	6,894959	-0,12806433	0,915058363	---

17284522	3,203531	3,283623	2,910357	2,956084	3,058296	2,998973	3,1325037	3,004451	-0,12805267	0,915065762	---
17236150	4,449353	4,772312	4,769297	4,790875	4,123817	4,692123	4,663654	4,535605	-0,128049	0,915068088	Ascl4
17469917	3,388485	3,475765	3,728369	3,289136	3,689354	3,229993	3,530873	3,402827667	-0,12804533	0,915070414	---
17533207	8,789001	9,03318	8,876984	8,801058	8,574802	8,939202	8,8997217	8,771687333	-0,12803433	0,915077391	Xk
17284876	3,894396	4,114282	3,798627	3,602144	3,984677	3,836483	3,9357683	3,807768	-0,12800033	0,915098957	1700024F13Rik
17345607	6,357115	5,956146	6,309355	6,104144	6,1362	5,998281	6,2075387	6,079541667	-0,127997	0,915101071	---
17200925	6,368141	5,983921	6,288869	6,341126	6,02188	5,894231	6,2136437	6,085745667	-0,127898	0,915163869	---
17326654	3,697176	3,379611	3,789401	3,380891	3,470396	3,631358	3,6220627	3,494215	-0,12784767	0,915195798	4930578N18Rik
17461177	9,295124	9,474593	9,317459	9,178136	9,286351	9,239162	9,362392	9,234549667	-0,12784233	0,915199181	Ppp4r2
17497357	4,045149	3,601201	3,400919	3,612351	3,406698	3,644765	3,682423	3,554604667	-0,12781833	0,915214406	---
17408970	3,977149	3,774055	3,746619	3,788294	3,634059	3,692032	3,8326077	3,704795	-0,12781267	0,915218001	Olfr266
17295386	4,72993	4,748837	4,926279	4,843172	4,585869	4,592598	4,801682	4,673879667	-0,12780233	0,915224556	Tmem171
17208901	4,958234	5,531792	4,757092	4,848291	4,497004	5,518495	5,0823727	4,954596667	-0,127776	0,915241262	---
17473177	8,68405	8,841356	8,722356	8,640441	8,635993	8,588037	8,749254	8,621490333	-0,12776367	0,915249086	Prpf31
17325988	5,771093	5,387609	5,986973	5,827682	5,416874	5,517856	5,715225	5,587470667	-0,12775433	0,915255007	Abhd10
17231780	3,066581	3,04917	2,940812	2,902466	2,864032	2,90682	3,0188543	2,891106	-0,12774833	0,915258814	---
17260016	3,754163	3,918247	3,625031	3,64699	3,487144	3,780259	3,7658137	3,638131	-0,12768267	0,915300474	Gm11961 // Gm11961
17330148	6,378321	6,182734	6,3243	6,07087	6,030875	6,400577	6,2951183	6,167440667	-0,12767767	0,915303646	---
17200441	4,903936	5,42072	4,783633	4,930084	4,866759	4,928487	5,0360963	4,908443333	-0,127653	0,915319296	---
17482669	4,487221	4,433546	4,490246	4,386773	4,392245	4,249084	4,4703377	4,342700667	-0,127637	0,915329447	Hs3st2
17293239	8,380093	7,78306	8,188522	7,79637	7,951553	8,220857	8,117225	7,989593333	-0,12763167	0,915332831	---
17429516	6,847748	6,918468	6,851852	6,578705	6,747644	6,908826	6,8726893	6,745058333	-0,127631	0,915333254	Dem1
17490606	8,660097	8,875546	8,955102	8,661119	8,878556	8,568187	8,8302483	8,702620667	-0,12762767	0,915335369	Snord35b
17352656	11,48625	11,88319	11,51922	11,5347	11,37424	11,59689	11,629553	11,50194333	-0,12761	0,915346578	Gm9386 // Gm10349 // Nutf2
17509659	3,526254	3,671012	3,44809	3,469113	3,47719	3,316252	3,548452	3,420851667	-0,12760033	0,915352711	Trim75
17482133	5,876294	5,468533	5,619419	5,503572	5,523694	5,554187	5,6547487	5,527151	-0,12759767	0,915354403	Tmc5
17280275	3,05855	3,202409	2,891049	2,811846	3,029611	2,927794	3,0506693	2,923083667	-0,12758567	0,915362017	---
17474181	7,401878	7,405748	7,443352	7,061392	7,418945	7,387965	7,4169927	7,289434	-0,12755867	0,915379148	Prkd2
17493220	4,725183	4,675675	4,858113	4,557521	4,588793	4,729985	4,7529903	4,625433	-0,12755733	0,915379994	Ccdc81
17494228	7,813572	7,932342	8,021959	7,650594	7,878827	7,855816	7,9226243	7,795079	-0,12754533	0,915387608	Beta-s // Hbb-b2 // Hbb-b1
17225066	8,202997	8,107115	8,1174	8,076853	7,970251	7,997782	8,142504	8,014962	-0,127542	0,915389723	Sp140
17226591	3,798612	3,749946	4,110956	3,635534	3,803382	3,838122	3,8865047	3,759012667	-0,127492	0,915421448	---
17237351	3,633331	3,831272	3,66098	3,451409	3,758356	3,533356	3,7085277	3,581040333	-0,12748733	0,91542441	---
17302490	4,027614	3,682136	4,308476	3,889908	3,968532	3,777449	4,0060753	3,878629667	-0,12744567	0,915450848	Scel
17469627	5,73041	5,68444	5,600262	5,37801	5,608106	5,64674	5,671704	5,544285333	-0,12741867	0,915467981	Oxtr
17493571	7,470215	7,293801	7,565886	7,00616	7,526839	7,414699	7,4433007	7,315899333	-0,12740133	0,91547898	Tsku
17243520	4,305301	4,257514	4,34416	4,005859	4,331353	4,187564	4,302325	4,174925333	-0,12739967	0,915480038	1700025N21Rik
17366884	7,036182	6,657485	6,271422	6,961069	6,088272	6,53357	6,6550297	6,527637	-0,12739267	0,91548448	---
17419853	4,52178	4,311631	4,561666	4,645425	4,308889	4,058594	4,4650257	4,337636	-0,12738967	0,915486383	---
17507148	9,467194	9,579628	9,510481	9,470753	9,29962	9,404767	9,519101	9,391713333	-0,12738767	0,915487653	Clec4g
17222420	3,60126	3,614259	3,81987	3,62125	3,380561	3,651482	3,678463	3,551097667	-0,12736533	0,915501825	Lyg1
17437072	8,179031	8,426785	8,323583	8,171299	8,11577	8,260349	8,3097997	8,182472667	-0,127327	0,915526151	Lyar
17475231	6,124372	6,132023	6,330839	6,053336	5,942252	6,20967	6,1957447	6,068419333	-0,12732533	0,915527208	Tmem145
17377989	9,125254	9,167234	9,099689	8,886218	8,967439	9,156552	9,1307257	9,003403	-0,12732267	0,9155289	Mapre1
17459733	3,208782	3,252807	3,081503	3,152237	3,141637	2,867279	3,1810307	3,053717667	-0,127313	0,915535035	---
17353141	6,93725	7,450066	7,877269	7,50516	6,901051	7,476563	7,4215283	7,294258	-0,12727033	0,915562112	Mir187
17261550	6,769907	7,206922	7,028418	6,811906	6,99811	6,813464	7,001749	6,874493333	-0,12725567	0,915571419	Npm1
17200981	7,082012	7,506121	6,897563	7,045494	7,281248	6,777193	7,1618987	7,034645	-0,12725367	0,915572689	---
17292058	11,80579	11,63817	11,56333	11,45901	11,54367	11,62296	11,669097	11,54188	-0,12721667	0,91559617	Elovl2
17297299	3,818847	3,941838	3,970835	3,718569	3,940401	3,690916	3,9105067	3,783295333	-0,12721133	0,915599555	B230110C06Rik
17517544	7,743941	7,93283	7,810309	7,546815	7,827974	7,730707	7,8290267	7,701832	-0,12719467	0,915610132	Rcn2
17306775	7,668049	7,70437	7,410356	7,472923	7,427403	7,500902	7,5942583	7,467076	-0,12718233	0,91561796	Mdp1
17489713	6,101665	5,948197	6,01047	5,758044	6,123531	5,797219	6,0201107	5,892931333	-0,12717933	0,915619864	Lrp3
17283556	4,021488	3,915336	4,399034	4,165556	3,897973	3,890824	4,1119527	3,984784333	-0,12716833	0,915626845	Ifi27l2b
17202051	3,646611	4,722816	4,786681	5,121895	3,888134	3,764601	4,3853693	4,25821	-0,12715933	0,915632557	---
17366988	5,252251	5,113652	5,335952	5,103572	5,160577	5,056275	5,2339517	5,106808	-0,12714367	0,9156425	Gm10851
17230573	6,4952	6,584836	6,135562	6,229738	6,441448	6,163078	6,4051993	6,278088	-0,12711133	0,915663022	Enah
17404373	6,54691	6,385839	6,267085	6,341521	6,078946	6,39811	6,3999447	6,272859	-0,12708567	0,915679312	Naaladl2
17380263	5,214369	5,235351	5,207429	5,210895	5,006796	5,058304	5,2190497	5,091998333	-0,12705133	0,915701104	1700010B08Rik
17259529	9,250798	8,938101	9,453786	9,12783	9,032803	9,100999	9,2142283	9,087210667	-0,12701767	0,915722473	---
17294696	9,083618	9,085414	9,065104	8,945648	8,873781	9,033666	9,0780453	8,951031667	-0,12701367	0,915725012	Rasa1
17252721	4,728708	4,591921	4,506545	4,637582	4,462661	4,345899	4,609058	4,482047333	-0,12701067	0,915726916	Olfr390
17263698	8,233935	8,079016	8,152887	7,962565	8,029067	8,093186	8,1552793	8,028272667	-0,12700667	0,915729455	Gm16515
17532660	12,17727	12,1947	12,10703	11,97316	12,05263	12,0722	12,159667	12,03266333	-0,12700333	0,915731571	---
17481682	8,96477	9,497999	9,378422	9,168276	9,293983	8,998012	9,280397	9,153423667	-0,12697333	0,915750613	---
17296647	6,19136	5,859822	5,881332	5,926067	5,652137	5,973413	5,9775047	5,850539	-0,12696567	0,915755479	1700110I01Rik
17321645	8,326221	8,491571	8,39825	8,269836	8,25651	8,308799	8,4053473	8,278381667	-0,12696567	0,915755479	Lima1
17313895	7,144602	7,200385	7,020054	6,95863	7,024634	7,000901	7,1216803	6,994721667	-0,12695867	0,915759923	Gtse1
17484103	8,177315	8,085983	8,484067	8,217152	8,308135	7,841276	8,2491217	8,122187667	-0,126934	0,91577558	---
17475226	4,650073	4,587349	4,534085	4,493505	4,372231	4,524976	4,5905023	4,463570667	-0,12693167	0,915777061	Prr19
17542021	7,222603	7,584421	7,081882	7,207324	7,117384	7,183438	7,296302	7,169382	-0,12692	0,915784467	Mir505
17302345	3,33117	3,429384	3,563618	3,293631	3,253951	3,395885	3,4413907	3,314489	-0,12690167	0,915796104	---
17467011	4,874093	4,577484	4,687178	4,787467	4,68158	4,289062	4,7129183	4,586036333	-0,126882	0,915808589	Plekha8 // Plekha8
17414855	4,174133	4,180336	4,347402	4,156195	3,958529	4,206519	4,233957	4,107081	-0,126876	0,915812397	---
17429896	8,323753	8,429596	8,520377	8,284667	8,233049	8,375484	8,4245753	8,297733333	-0,126842	0,915833981	Cdca8
17285654	5,656795	5,603462	5,682105	5,453603	5,506998	5,601282	5,647454	5,520627667	-0,12682633	0,915843926	Zfp187 // Zfp187
17238890	6,47724	6,524127	6,768738	6,57492	6,701293	6,113459	6,590035	6,463224	-0,126811	0,91585366	Syne1
17520422	4,056991	4,232599	3,894684	4,066811	3,922443	3,814828	4,0614247	3,934694	-0,12673067	0,915904659	---
17393264	5,228312	4,967936	5,433827	5,196895	4,821868	5,231259	5,210025	5,083340667	-0,12668433	0,915934074	Ggt7
17292026	5,269228	4,977567	5,196878	4,941433	5,057391	5,064801	5,147891	5,021208333	-0,12668267	0,915935132	Gcnt2 // Gcnt2
17221756	8,398792	8,570171	8,511642	8,302286	8,331877	8,466675	8,493535	8,366946	-0,126589	0,915994601	Ogfrl1
17202487	3,39639	4,155162	3,876495	3,709102	4,081881	3,257326	3,809349	3,682769667	-0,12657933	0,916000739	---
17324733	6,789931	6,790361	6,562253	6,621118	6,670701	6,471033	6,7141817	6,587617333	-0,12656433	0,916010262	1500031L02Rik
17534142	7,656154	7,661963	7,596814	7,458876	7,556176	7,520198	7,6383103	7,51175	-0,12656033	0,916012802	Nkap
17205487	6,917134	6,834121	6,926468	6,566445	6,696112	7,035522	6,8925743	6,766026333	-0,126548	0,916020633	---
17430475	6,692199	6,720978	6,978776	6,700137	6,651992	6,660194	6,7973177	6,670774333	-0,12654333	0,916023596	Ccdc28b
17247562	8,064833	8,248094	8,074818	7,968813	8,049335	7,990093	8,1292483	8,002747	-0,12650133	0,916050264	Vps54
17336929	5,58219	5,740664	5,808016	5,480873	5,727076	5,543456	5,71029	5,583801667	-0,12648833	0,916058518	Ly6g6e
17329833	7,633182	7,914606	7,631224	7,454343	7,707181	7,638059	7,7263373	7,599861	-0,12647633	0,916066138	Fbxo45
17328000	10,14361	10,38219	10,22191	10,08643	10,10417	10,17772	10,249237	10,12277333	-0,12646333	0,916074393	Usp7
17304294	5,034351	5,217534	5,270449	5,123162	5,013922	5,005989	5,1741113	5,047691	-0,12642033	0,916101697	---
17401545	5,27751	4,783546	4,985878	4,991546	4,808709	4,867425	5,0156447	4,889226667	-0,126418	0,916103179	Kcna10
17509527	4,36938	4,512843	4,982358	4,437763	4,55451	4,493111	4,621527	4,495128	-0,126399	0,916115244	---
17327907	3,662876	3,153102	3,420982	3,286048	3,250371	3,321386	3,41232	3,285935	-0,126385	0,916124134	---
17224724	5,306166	5,725978	5,385358	5,160594	5,3907	5,487113	5,4725007	5,346135667	-0,126365	0,916136834	BC035947 // BC035947
17537643	5,856586	5,613644	5,782756	5,666101	5,594306	5,613556	5,7509953	5,624654333	-0,126341	0,916152074	Tmem35
17239470	3,820005	3,123178	3,573308	3,694432	3,694432	2,748616	3,505497	3,37916	-0,126337	0,916154615	---
17404337	8,705015	8,420052	8,744146	8,407601	8,359567	8,723085	8,623071	8,496751	-0,12632	0,91616541	Cpa3
17487898	6,224124	6,11485	5,974164	6,014194	5,586986	6,333063	6,1043793	5,978081	-0,12629833	0,916179169	Erf
17343521	4,447785	4,451098	4,623238	4,530801	4,304264	4,308199	4,5073737	4,381088	-0,12628567	0,916187213	Cyp4f41-ps
17262823	4,34334	4,069501	4,209295	4,104524	4,151659	3,987097	4,2073787	4,081093333	-0,12628533	0,916187425	Il3
17212199	6,25957	6,751803	6,425791	6,550734	6,214411	6,293199	6,4790547	6,352781333	-0,12627333	0,916195046	Il1rl2
17542546	7,88681	7,789165	7,797398	7,847813	7,394093	7,852664	7,8244577	7,69819	-0,12626767	0,916198644	Irak1
17458403	5,821864	5,064688	5,633526	5,437681	5,357445	5,346159	5,5066927	5,380428333	-0,12626433	0,916200761	---
17240365	10,45815	10,62471	10,49925	10,32152	10,38628	10,49554	10,52737	10,40111333	-0,12625667	0,91620563	Amd1
17204141	3,466491	4,140587	3,321247	3,563029	3,538371	3,448301	3,642775	3,516567	-0,126208	0,916236537	---
17504727	6,853215	6,998275	6,837018	6,671294	6,819921	6,818712	6,8961693	6,769975667	-0,12619367	0,91624564	Elmo3
17431951	6,518014	6,373419	6,632652	6,352438	6,46358	6,329518	6,5080283	6,381845333	-0,126183	0,916252414	---
17539818	3,939316	3,728077	3,987996	3,91261	3,757524	3,606748	3,8851297	3,758960667	-0,126169	0,916261306	Mir500
17206319	4,117683	4,171628	4,21153	3,90729	3,892104	4,322969	4,166947	4,040787667	-0,12615933	0,916267445	---
17202505	7,501257	7,611894	7,560105	7,711308	7,18232	7,401209	7,557752	7,431612333	-0,12613967	0,916279936	---
17269464	7,44224	7,359582	7,494248	7,254405	7,407783	7,255486	7,4320233	7,305891333	-0,126132	0,916284805	Jup
17420108	5,850912	5,612469	5,880608	5,68614	5,502048	5,777589	5,7813297	5,655259	-0,12607067	0,91632376	9130020K20Rik
17354681	6,386191	5,935222	5,944619	5,967831	5,952993	5,96701	6,0886773	5,962611333	-0,126066	0,916326724	Slc6a7
17434061	8,412302	8,597168	8,729256	8,2524	8,521709	8,586443	8,5795753	8,453517333	-0,126058	0,916331805	Samd11
17449593	5,027089	5,027351	4,799121	4,979366	4,845335	4,65088	4,951187	4,825193667	-0,12599333	0,916372879	1700063O14Rik
17449931	6,212049	5,917262	6,233419	6,356465	5,863185	5,765116	6,12091	5,994922	-0,125988	0,916376267	Naa11
17512221	8,143362	7,801745	7,870465	7,809207	7,862877	7,765584	7,938524	7,812556	-0,125968	0,916388971	Cmtm4
17350996	5,953242	6,009867	5,781649	5,851866	5,886313	5,628753	5,9149193	5,788977333	-0,125942	0,916405486	Arsi
17260747	5,288243	5,898751	5,797727	5,577858	5,43262	5,596432	5,6615737	5,535636667	-0,125937	0,916408662	2810442I21Rik
17240164	7,936491	8,111237	7,940721	7,721357	8,013955	7,875342	7,9961497	7,870218	-0,12593167	0,91641205	Rwdd1
17209175	5,349697	5,183246	5,274271	5,41849	4,573522	5,437458	5,2690713	5,143156667	-0,12591467	0,916422848	---
17370073	4,832969	5,018688	5,037438	4,871347	4,8512	4,788809	4,9630317	4,837118667	-0,125913	0,916423907	---
17476864	11,8961	11,63725	11,97452	11,71787	11,72265	11,68962	11,835957	11,71004667	-0,12591	0,916425813	---
17407905	8,155511	8,247383	8,31341	7,965709	8,2516	8,121299	8,238768	8,112869333	-0,12589867	0,916433012	Prpf3
17207125	7,412972	7,120252	6,801771	7,40553	6,818414	6,733366	7,111665	6,98577	-0,125895	0,916435341	---
17226489	5,447548	5,471941	5,471609	5,276749	5,357687	5,378993	5,4636993	5,337809667	-0,12588967	0,916438729	Ysk4
17278290	6,664862	7,161863	6,976053	6,631505	6,80537	6,988253	6,9342593	6,808376	-0,12588333	0,916442752	Serpina3h
17243544	5,162793	5,342706	5,625571	5,045114	5,555253	5,15317	5,3770233	5,251179	-0,12584433	0,916467526	Aldh1l2
17502976	7,761168	7,85389	7,788074	7,631942	7,751049	7,642666	7,801044	7,675219	-0,125825	0,916479808	Ddx39
17525198	5,765921	5,784743	5,720011	5,651781	5,648957	5,592715	5,7568917	5,631151	-0,12574067	0,916533382	Ntm
17305111	3,567484	3,442742	3,35205	3,374723	3,263179	3,347174	3,454092	3,328358667	-0,12573333	0,916538041	---
17307255	3,884958	3,51091	3,450246	3,49004	3,518367	3,460674	3,6153713	3,489693667	-0,12567767	0,916573407	Gm5142
17449866	6,057007	5,85496	6,09148	5,978728	5,777841	5,869889	6,001149	5,875486	-0,125663	0,916582725	A430089I19Rik

17458630	5,071854	4,944343	4,996973	4,916729	4,870416	4,849068	5,00439	4,878737667	-0,12565233	0,916589502	Evx1
17447093	8,058295	8,059121	8,137864	7,820185	8,217195	7,840954	8,0850933	7,959444667	-0,12564867	0,916591831	---
17258736	4,514182	4,502373	4,663154	4,51288	4,650369	4,139529	4,559903	4,434259333	-0,12564367	0,916595008	---
17550454	6,797485	7,155497	6,981696	6,919044	6,966727	6,671999	6,978226	6,85259	-0,125636	0,916599879	---
17521175	6,002711	5,950116	5,827045	5,875741	5,721606	5,805685	5,926624	5,801010667	-0,12561333	0,91661428	4930500F10Rik
17377471	4,182047	3,957272	4,590207	4,078816	4,272688	4,001244	4,2431753	4,117582667	-0,12559267	0,916627411	Gm14123
17415771	6,479483	6,27702	6,745208	6,356182	6,446942	6,321889	6,5005703	6,375004333	-0,125566	0,916644354	---
17200507	5,082003	5,312913	4,650684	5,116135	4,842243	4,710524	5,0152	4,889634	-0,125566	0,916644354	---
17223863	10,82429	10,69675	10,70116	10,63173	10,49146	10,72234	10,740733	10,61517667	-0,12555667	0,916650284	Idh1
17503926	4,005208	4,086408	4,537863	4,130349	3,955553	4,166939	4,2098263	4,084280333	-0,125546	0,916657061	Mt4
17275637	4,357888	3,836515	3,941362	3,910425	3,844445	4,004278	4,045255	3,919716	-0,125539	0,916661509	Gm20016
17463542	5,489846	5,281229	5,386325	5,116554	5,33406	5,330177	5,3858	5,260263667	-0,12553633	0,916663203	D630042F21Rik
17493116	7,502015	7,333215	7,568069	7,437539	6,991012	7,598139	7,4677663	7,34223	-0,12553633	0,916663203	Olfr310
17474547	5,706649	5,673746	5,643611	5,903278	5,899576	4,844551	5,6746687	5,549135	-0,12553367	0,916664897	Rtn2
17283930	8,068308	8,239529	8,171144	7,896052	8,19046	8,015872	8,1596603	8,034128	-0,12553233	0,916665745	Wars
17389313	3,89909	4,368628	3,546516	3,825138	4,246804	3,365698	3,938078	3,812546667	-0,12553133	0,91666638	Olfr1316
17322337	7,090634	7,317271	7,420438	7,157822	7,163972	7,129979	7,2761143	7,150591	-0,12552333	0,916671463	Nfe2
17468602	4,42464	4,297707	4,473855	4,414748	4,163411	4,241502	4,398734	4,273220333	-0,12551367	0,916677605	Gkn3
17232731	7,569426	7,85579	7,947262	7,510787	7,864842	7,62041	7,790826	7,665346333	-0,12547967	0,916699209	Rnu3a // Rnu3a
17419799	10,52471	10,69558	10,69729	10,39616	10,59103	10,55415	10,639193	10,51378	-0,12541333	0,916741358	Rhd
17251719	3,732962	3,606872	3,51144	3,559674	3,471217	3,444165	3,6170913	3,491685333	-0,125406	0,916746018	Mir1934
17201267	4,40684	4,922025	4,25403	3,945247	4,928179	4,333257	4,5276317	4,402227667	-0,125404	0,916747289	---
17344963	4,742695	4,765417	4,676875	4,819461	4,654649	4,334681	4,728329	4,602930333	-0,12539867	0,916750678	Crisp2
17506846	7,308065	7,470593	7,307697	7,132193	7,269679	7,3083	7,3621183	7,236724	-0,12539433	0,916753432	Gm505
17445615	4,324862	4,603731	4,178463	4,176775	4,357576	4,196574	4,3690187	4,243641667	-0,125377	0,916764446	1700003C15Rik // 1700003C15Rik // 170
17296528	6,204652	6,210178	6,390459	6,163881	6,158298	6,107098	6,2684297	6,143092333	-0,12533733	0,916789653	Gm7120
17267695	4,939297	4,389699	4,71967	4,372474	4,632272	4,667944	4,6828887	4,557563333	-0,12532533	0,916797279	---
17362579	4,506127	4,741604	4,708955	4,610637	4,558342	4,411753	4,6522287	4,526910667	-0,125318	0,916801939	Best1
17485339	8,140732	8,031449	7,783503	7,853754	7,709311	8,016799	7,985228	7,859954667	-0,12527333	0,916830324	Slc22a18
17406027	6,804586	6,237973	6,370858	6,264175	6,473249	6,300231	6,471139	6,345885	-0,125254	0,91684261	---
17242760	9,089679	9,213066	9,373851	9,102575	9,12642	9,07184	9,225532	9,100278333	-0,12525367	0,916842822	ORF61
17434653	3,346399	3,146462	3,228605	3,105727	3,128506	3,11151	3,2404887	3,115247667	-0,125241	0,916850872	Mir879
17206153	3,36796	3,439821	3,081503	3,132662	3,317955	3,06307	3,296428	3,171229	-0,125199	0,916877564	---
17365270	4,008063	4,082036	4,293959	4,027511	3,886882	4,094134	4,1280193	4,002842333	-0,125177	0,916891546	Kcnip2
17495452	3,239034	3,111026	2,878933	2,997559	3,066875	2,789093	3,076331	2,951175667	-0,12515533	0,916905316	---
17232843	6,529342	6,506543	6,289363	6,219045	6,126911	6,603874	6,4417493	6,31661	-0,12513933	0,916915485	Zbtb24
17418279	9,359824	9,289105	9,257427	9,113098	9,214733	9,203209	9,3021187	9,177013333	-0,12510533	0,916937094	Sf3a3
17231281	3,997135	3,788127	3,955982	3,679756	3,892492	3,793687	3,913748	3,788645	-0,125103	0,916938577	Cd34
17456798	3,203872	2,986644	3,784867	3,018262	3,406698	3,175129	3,3251277	3,200029667	-0,125098	0,916941755	---
17200587	4,743263	5,562332	5,029489	5,206611	5,436769	4,31645	5,1116947	4,98661	-0,12508467	0,916950229	---
17302773	9,883961	10,0797	9,969069	9,769636	9,926979	9,860867	9,9775767	9,852494	-0,12508267	0,9169515	Ipo5
17468222	6,068815	5,856849	6,206287	5,688008	6,011727	6,057131	6,0439837	5,918955333	-0,12502833	0,916986034	Cd207
17270453	4,596788	4,856316	5,263696	4,851172	4,904493	4,58609	4,9056	4,780585	-0,125015	0,916994509	Map3k14
17306864	10,34625	10,59069	10,22309	10,25604	10,30376	10,22519	10,386677	10,26166333	-0,12501333	0,916995568	---
17217283	9,608877	9,439332	9,587626	9,457048	9,378593	9,425162	9,5452783	9,420267667	-0,12501067	0,916997263	Ppp1r15b
17342719	5,915839	6,276003	6,011846	5,862728	5,951588	6,014369	6,067896	5,942895	-0,125001	0,917003408	Grm4
17306201	10,39989	10,49163	10,34922	10,22303	10,30265	10,34007	10,41358	10,28858333	-0,12499667	0,917006162	Supt16h
17202609	4,022102	4,558475	3,863241	3,733645	4,117329	4,21788	4,1479393	4,022951333	-0,124988	0,917011671	---
17457758	4,393472	4,198904	4,548605	4,012839	4,540878	4,212314	4,380327	4,255343667	-0,12498333	0,917014637	Prss1
17262172	3,160854	2,943625	3,218074	3,064555	2,993533	2,889591	3,1075177	2,982559667	-0,124958	0,91703074	---
17476557	5,779483	5,694544	6,492961	5,6717	5,928979	5,991454	5,988996	5,864044333	-0,12495167	0,917034765	Krtdap
17534593	7,376015	7,174172	7,606263	7,34085	7,241507	7,199239	7,3854833	7,260532	-0,12495133	0,917034977	Bcorl1
17377857	6,513347	6,319377	6,350078	6,429626	6,087806	6,290524	6,3942673	6,269318667	-0,12494867	0,917036672	BC020535
17397801	10,3869	10,45914	10,43011	10,25261	10,36448	10,28427	10,425383	10,30045333	-0,12493	0,917048538	2810407C02Rik
17288225	4,756735	4,479433	3,732137	4,677404	4,053538	3,862631	4,3227683	4,197857667	-0,12491067	0,917060827	Zfp640 // Zfp640
17209035	4,431314	4,405938	4,820879	4,678529	4,086068	4,518825	4,5527103	4,427807333	-0,124903	0,9170657	---
17334045	3,152567	3,357135	3,107079	3,207199	2,829569	3,205316	3,2055937	3,080694667	-0,124899	0,917068243	Vmn2r115
17299834	3,908327	3,737936	4,132339	4,051082	3,523557	3,829384	3,9262007	3,801341	-0,12485967	0,917093246	---
17549230	4,500129	3,788274	4,531315	4,152839	4,08776	4,204572	4,2732393	4,148390333	-0,124849	0,917100027	---
17372979	5,156323	5,2879	5,317957	5,074533	5,128349	5,184757	5,25406	5,129213	-0,124847	0,917101298	---
17342182	6,55553	6,486318	6,635086	6,47592	6,375028	6,451464	6,558978	6,434137333	-0,12484067	0,917105324	Baiap3
17472063	9,772451	9,899445	9,741003	9,798423	9,605549	9,634474	9,8042997	9,679482	-0,12481767	0,917119945	Hebp1
17487031	3,381197	3,567973	3,41997	3,259932	3,447098	3,287717	3,45638	3,331582333	-0,12479767	0,917132659	---
17458734	6,838691	6,721686	6,685359	6,571692	6,694894	6,604774	6,7485787	6,623786667	-0,124792	0,917136261	Plekha8
17525482	3,651848	3,40663	3,3582	3,198162	3,436506	3,407648	3,472226	3,347438667	-0,12478733	0,917139228	Gm5615
17248831	4,007758	3,718802	3,582656	3,438155	3,666592	3,830136	3,7697387	3,644961	-0,12477767	0,917145373	Gm12171
17200185	6,116903	6,161904	6,444081	5,878943	6,030032	6,439626	6,2409627	6,116200333	-0,12476233	0,917155121	---
17267520	8,049475	8,35233	8,441293	7,922437	8,307837	8,238574	8,2810327	8,156282667	-0,12475	0,917162962	Epx
17551157	4,683632	4,698493	4,877244	4,494716	4,682267	4,708188	4,753123	4,628390333	-0,12473267	0,917173981	---
17378524	5,74858	5,465042	5,642182	5,305309	5,6713	5,505017	5,6186013	5,493875333	-0,124726	0,917178219	Spag4
17374514	4,849961	4,771492	5,158733	4,784831	4,664426	4,956751	4,9267287	4,802002667	-0,124726	0,917178219	1700054M17Rik
17408131	4,997077	5,157413	5,243306	5,087842	4,893586	5,042294	5,1325987	5,007907333	-0,12469133	0,917200259	4930442L01Rik
17549342	11,57119	11,39807	11,03574	11,09115	10,95597	11,58382	11,335	11,21031333	-0,12468667	0,917203225	---
17333846	3,569307	3,372462	3,259178	3,262511	3,223835	3,340545	3,4003157	3,275630333	-0,12468533	0,917204073	Vmn1r226
17332734	4,95313	5,150319	5,37422	4,969758	5,083235	5,050621	5,159223	5,034538	-0,124685	0,917204285	Gm19404
17481163	5,138768	4,839736	4,877737	5,082081	4,675371	4,724897	4,9520803	4,827449667	-0,12463067	0,917238828	Olfr574
17338599	4,675832	4,854508	5,057837	4,632812	4,832878	4,748622	4,8627257	4,738104	-0,12462167	0,917244551	St6gal2
17204189	3,86084	3,584832	3,925552	3,792424	3,612777	3,592182	3,790408	3,665794333	-0,12461367	0,917249637	---
17261865	9,711077	9,712611	9,578193	9,477132	9,612379	9,53862	9,6672937	9,542710333	-0,12458333	0,917268923	Ccng1
17266516	9,658829	9,510496	9,441642	9,23949	9,636377	9,361378	9,536989	9,412415	-0,124574	0,917274857	Fam58b
17345066	4,456035	4,845208	4,859089	4,572901	4,60321	4,610535	4,7201107	4,595548667	-0,124562	0,917282486	Mep1a
17538846	3,0202	2,775883	2,898663	2,787537	2,788613	2,744958	2,8982487	2,773702667	-0,124546	0,91729266	Mirlet7f-2
17538847	3,0202	2,775883	2,898663	2,787537	2,788613	2,744958	2,8982487	2,773702667	-0,124546	0,91729266	---
17332117	3,786544	3,830667	4,110368	3,567592	3,944883	3,841472	3,909193	3,784649	-0,124544	0,917293931	---
17307337	7,473244	7,484648	7,325666	7,132671	7,376774	7,400573	7,4278527	7,303339333	-0,12451333	0,91731343	Cdadc1
17266424	5,412778	5,480187	5,730566	5,270637	5,408625	5,570736	5,541177	5,416666	-0,124511	0,917314913	Foxn1
17285672	2,960505	2,971244	3,257675	2,992066	2,805593	3,018263	3,0631413	2,938640667	-0,12450067	0,917321484	---
17492847	10,08419	10,16556	9,979267	9,941385	9,878254	10,03595	10,076339	9,951863	-0,124476	0,917337168	Btbd1
17365471	6,399531	6,145945	6,413116	6,146362	6,11573	6,323165	6,3195307	6,195085667	-0,124445	0,917356879	Calhm1
17545947	6,857787	6,933198	7,065512	6,938487	6,529157	7,015567	6,9521657	6,827737	-0,12442867	0,917367265	Siah1b
17206225	4,029337	4,094774	3,237874	3,363209	3,364512	4,26101	3,7873283	3,662910333	-0,124418	0,917374048	---
17291122	8,185374	8,326197	8,001132	8,060039	8,040329	8,03912	8,170901	8,046496	-0,124405	0,917382314	Zfp322a
17542074	2,996602	2,931836	3,05592	2,818858	2,896417	2,895926	2,994786	2,870400333	-0,12438567	0,917394608	Mir741
17521043	4,271008	4,204072	4,150944	4,127497	4,02936	4,096032	4,2086747	4,084296333	-0,12437833	0,917399271	Cpne4
17328411	9,03348	9,071207	8,952316	8,848043	8,927674	8,908252	9,019001	8,894656333	-0,12434467	0,91742068	0610037P05Rik
17441337	4,16973	4,246247	4,081704	4,091333	3,942789	4,090542	4,1658937	4,041554667	-0,124339	0,917424283	---
17277039	3,700402	4,061806	4,008311	4,071256	3,542345	3,783916	3,9235063	3,799172333	-0,124334	0,917427463	---
17265329	7,55382	7,73541	7,443039	7,224604	7,660542	7,474234	7,577423	7,453126667	-0,12429633	0,917451416	Spag7
17244244	7,766263	7,822174	8,254099	7,772121	7,793348	7,90418	7,947512	7,823216333	-0,12429567	0,91745184	Nedd1
17308888	8,15023	8,402395	8,046334	7,892628	8,154126	8,179431	8,199653	8,075395	-0,124258	0,917475794	Naa16
17422302	7,352953	7,722605	7,571673	7,192914	7,532411	7,54929	7,549077	7,424871667	-0,12420533	0,917509287	Lrrc47
17321618	3,527338	3,293744	3,209612	3,465991	3,040581	3,15153	3,3435647	3,219367333	-0,12419733	0,917514375	---
17479198	4,479865	3,978265	4,176284	4,235177	3,885424	4,141273	4,2114713	4,087291333	-0,12418	0,917525399	---
17550789	4,012974	4,022657	4,331486	4,000248	4,018025	3,97632	4,1223723	3,998197667	-0,12417467	0,917528791	---
17332315	4,281404	3,939172	4,020691	4,251935	3,865031	3,751782	4,0804223	3,956249333	-0,124173	0,917529851	---
17464205	5,089535	4,437804	5,007598	4,725934	4,76318	4,673374	4,844979	4,720829333	-0,12414967	0,91754469	---
17439541	4,667994	4,322206	4,294253	4,372544	4,211919	4,327592	4,428151	4,304018333	-0,12413267	0,917555502	---
17207979	4,071876	4,516574	3,414383	4,152071	3,793024	3,685369	4,0009443	3,876821333	-0,124123	0,91756165	---
17348688	5,305025	4,983297	4,983016	5,013851	4,892656	4,992472	5,090446	4,966326333	-0,12411967	0,91756377	1700001G01Rik
17200091	3,575398	2,935287	3,430856	3,255168	3,179672	3,134619	3,313847	3,189819667	-0,12402733	0,917622497	---
17405627	3,251773	3,518827	3,532468	3,430012	3,38167	3,119307	3,434356	3,310329667	-0,12402633	0,917623133	Vmn2r5
17345951	5,183626	5,543834	5,610282	5,462895	5,201113	5,30172	5,445914	5,321909333	-0,12400467	0,917636914	Gm5094 // Gm5094
17201219	9,526843	9,419049	9,172151	8,706896	9,530935	9,508208	9,372681	9,248679667	-0,12400133	0,917639034	---
17445394	4,397971	4,839987	4,633126	4,5683	4,456182	4,474616	4,6236947	4,499699333	-0,12399533	0,917642851	A330021E22Rik
17503536	5,371338	5,338356	5,44309	5,230002	5,157948	5,392948	5,3842613	5,260299333	-0,123962	0,917664053	Gm2694
17376480	9,645939	9,530774	9,546752	9,322106	9,537261	9,492254	9,5744883	9,450540333	-0,123948	0,917672958	Mavs
17548928	8,327417	8,546782	8,326248	8,216407	8,383986	8,228218	8,400149	8,276203667	-0,12394533	0,917674654	Zfp330 // Zfp330
17387662	3,206155	3,273775	3,399464	3,145957	3,090683	3,270946	3,2931313	3,169195333	-0,123936	0,917680591	Olfr1054
17320064	6,117027	5,451381	5,907963	5,780649	5,753067	5,570858	5,825457	5,701524667	-0,12393233	0,917682923	Gm19277
17535503	3,162655	3,761866	3,477506	3,180278	3,38239	3,46757	3,4673423	3,343412667	-0,12392967	0,91768462	Xlr3b
17487664	2,95362	3,353489	3,084666	2,946405	3,128417	2,945263	3,1305917	3,006695	-0,12389667	0,917705611	Vmn1r167
17406454	4,839804	4,683425	4,966399	4,729309	4,543581	4,845067	4,829876	4,705985667	-0,12389033	0,917709639	---
17549384	5,295447	5,002259	5,195575	5,077774	5,143924	4,899972	5,164427	5,040556667	-0,12387033	0,917722362	---
17549768	5,295447	5,002259	5,195575	5,077774	5,143924	4,899972	5,164427	5,040556667	-0,12387033	0,917722362	---
17367438	3,457742	3,308892	3,738414	3,473831	3,302649	3,356975	3,5016827	3,377818333	-0,12386433	0,917726178	---
17322979	5,981552	6,171599	6,008812	5,955123	5,790024	6,045242	6,0539877	5,930129667	-0,123858	0,917730207	Gm9961
17338059	6,326072	5,967479	6,269473	6,127555	6,102749	5,961163	6,1876747	6,063822333	-0,12385233	0,917733812	Gm88
17396095	3,352242	3,648285	3,20937	3,362276	2,956524	3,519636	3,403299	3,279478667	-0,12382033	0,917754168	---
17238836	3,530565	3,423191	4,051492	3,73272	3,335741	3,565351	3,668416	3,544604	-0,123812	0,917759469	Syne1
17250093	5,072797	5,37665	5,608396	4,914769	5,303935	5,46773	5,3526143	5,228811333	-0,123803	0,917765195	Olfr320
17521652	8,373885	8,235856	8,331628	8,358793	8,143991	8,067182	8,3137897	8,189988667	-0,123801	0,917766467	Nicn1
17305947	3,003517	2,939525	2,761565	2,893503	2,837708	2,601993	2,9015357	2,777734667	-0,123801	0,917766467	Olfr732
17420820	5,723317	5,120702	5,71158	5,857812	4,990265	5,336124	5,518533	5,394733667	-0,12379933	0,917767527	---
17260369	6,83153	6,896594	7,016573	6,662855	6,943433	6,767082	6,914899	6,791123333	-0,12377567	0,917782583	Myo1g

17263189	5,03616	4,595573	5,063349	4,860472	4,829459	4,633849	4,8983607	4,774593333	-0,12376733	0,917787884	Olfr317
17402410	4,777043	3,907852	4,204078	4,252634	4,240915	4,024131	4,2963243	4,17256	-0,12376433	0,917789793	---
17297114	3,526658	3,436548	3,693642	3,517847	3,318556	3,449158	3,5522827	3,428520333	-0,12376233	0,917791065	---
17430628	4,936222	5,347946	5,174348	5,016714	5,003871	5,06669	5,1528387	5,029091667	-0,123747	0,917800819	Fabp3
17331589	7,082319	7,236114	7,28309	7,133276	7,024765	7,072248	7,2005077	7,076763	-0,12374467	0,917802304	Btg3
17331882	4,091334	3,886465	4,158662	3,705357	3,899273	4,160613	4,045487	3,921747667	-0,12373933	0,917805697	Krtap16-10b
17274744	5,701342	5,718606	5,921685	5,540499	5,800153	5,629831	5,7805443	5,656827667	-0,12371667	0,917820117	Fam150b
17293721	5,369158	5,398128	5,66475	5,46564	5,144297	5,450968	5,4773453	5,353635	-0,12371033	0,917824146	Cntnap3
17222321	4,160792	4,085966	3,776113	3,908224	3,807545	3,936004	4,0076237	3,883924333	-0,12369933	0,917831144	---
17419741	5,977345	5,84886	5,99196	5,927618	5,77087	5,748721	5,9393883	5,815736333	-0,123652	0,917861258	Slc30a2
17326783	3,902342	3,804767	3,889035	3,886397	3,528753	3,810044	3,8653813	3,741731333	-0,12365	0,91786253	4930553E22Rik // 4930553E22Rik
17402097	5,843534	5,437208	5,924268	5,698714	5,529988	5,605389	5,7350033	5,611363667	-0,12363967	0,917869104	---
17365990	3,479658	3,486678	3,638083	3,437594	3,312272	3,483674	3,5348063	3,41118	-0,12362633	0,917877587	---
17303079	3,526217	3,490137	3,071501	3,266247	3,055971	3,394789	3,3626183	3,239002333	-0,123616	0,917884162	Gm6337
17205031	4,200747	3,867862	4,273844	4,126776	4,159054	3,685784	4,114151	3,990538	-0,123613	0,91788607	---
17209221	4,200747	3,867862	4,273844	4,126776	4,159054	3,685784	4,114151	3,990538	-0,123613	0,91788607	---
17489917	3,910986	3,387941	3,339732	3,423074	3,469141	3,375618	3,5462197	3,422611	-0,12360867	0,917888827	Gm6818
17491699	6,415414	5,766941	6,44822	5,962325	6,167157	6,130338	6,2101917	6,086606667	-0,123585	0,917903885	Mir344d-2
17325761	9,979249	10,02424	9,906165	9,87308	9,817085	9,848771	9,9698847	9,846312	-0,12357267	0,917911732	Naa50
17514093	8,37214	8,534983	8,358776	8,275743	8,265228	8,35433	8,4219663	8,298433667	-0,12353267	0,917937182	Ttc13
17238002	8,633303	8,50561	8,48596	8,385464	8,410123	8,458715	8,5416243	8,418100667	-0,12352367	0,917942909	R3hdm2
17221230	9,333577	9,835163	9,738974	9,54565	9,703076	9,288532	9,6359047	9,512419333	-0,12348533	0,917967299	---
17459014	9,851614	9,953394	9,711395	9,619825	9,69434	9,831974	9,838801	9,715379667	-0,12342133	0,918008023	Abcg2
17406657	4,360075	4,970944	4,776006	4,869746	4,123647	4,743376	4,7023417	4,578923	-0,12341867	0,918009719	---
17477101	7,356382	7,348019	7,479204	7,336527	7,239802	7,237189	7,394535	7,271172667	-0,12336233	0,918045566	Nkg7
17262600	6,295694	6,331808	6,403808	6,181927	6,193444	6,285906	6,34377	6,220425667	-0,12334433	0,91805702	Tcf7
17523633	4,40459	4,237818	4,386073	4,323033	4,143979	4,19149	4,342827	4,219500667	-0,12332633	0,918068474	Ccr9
17510715	12,59752	12,80513	12,61288	12,49867	12,49636	12,65056	12,671843	12,54853	-0,12331333	0,918076747	---
17202689	3,136523	2,870814	2,787253	3,002147	2,757736	2,664796	2,93153	2,808226333	-0,12330367	0,918082899	---
17309024	5,206764	5,037148	5,009142	5,023704	4,943027	4,916421	5,0843513	4,961050667	-0,12330067	0,918084808	Klhl1
17263379	8,567745	8,561299	8,477054	8,247859	8,529119	8,459282	8,535366	8,412086667	-0,12327933	0,918098384	Arf1
17428251	4,451313	4,407366	4,264602	4,158353	4,309779	4,285404	4,374427	4,251178667	-0,12324833	0,918118112	---
17309001	5,276746	5,025557	4,988922	5,043158	5,218792	4,659562	5,097075	4,973837333	-0,12323767	0,9181249	Pcdh20
17379395	4,145962	4,085978	4,229443	4,024031	4,136896	3,930755	4,1537943	4,030560667	-0,12323367	0,918127445	Svs5
17200697	5,840397	6,301009	5,986218	5,941826	6,027605	5,788525	6,0425413	5,919318667	-0,12322267	0,918134446	---
17396439	6,324554	6,05308	5,962348	5,983248	6,093348	5,893751	6,1133273	5,990115667	-0,12321167	0,918141446	Tnik
17341508	6,432549	6,342853	6,3066	6,362159	6,244833	6,105454	6,3606673	6,237482	-0,12318533	0,918158205	Mmp25
17494162	8,507867	8,000309	8,624675	8,298046	8,323035	8,142219	8,377617	8,254433333	-0,12318367	0,918159266	Dub2a
17246803	5,56724	5,813579	5,55262	5,536383	5,4859	5,541615	5,6444797	5,521299333	-0,12318033	0,918161387	Osm
17542573	6,937353	6,992246	6,951198	6,720438	6,850444	6,940384	6,9602657	6,837088667	-0,123177	0,918163509	Mecp2
17210043	5,779521	6,592791	6,234483	6,330696	6,275842	5,630729	6,202265	6,079089	-0,123176	0,918164145	---
17390823	7,418223	7,461577	7,32107	7,286138	7,175143	7,370133	7,40029	7,277138	-0,123152	0,918179419	Slc30a4
17508633	9,205857	9,32991	9,299461	9,151462	9,156748	9,157564	9,2784093	9,155258	-0,12315133	0,918179844	Wrn
17439307	4,028617	3,672441	3,736709	3,694434	3,634152	3,739849	3,812589	3,689478333	-0,12311067	0,918205726	Gm3259
17377140	4,763219	4,197959	4,394943	4,365364	4,259312	4,362203	4,4520403	4,328959667	-0,12308067	0,918224819	1700010M22Rik
17524170	8,161145	8,401373	8,347656	8,183118	8,166879	8,190936	8,3033913	8,180311	-0,12308033	0,918225032	5830418K08Rik
17368633	5,789444	5,935494	5,99714	5,931425	5,529356	5,892087	5,9073593	5,784289333	-0,12307	0,918231608	F730016J06Rik
17203413	7,225335	6,30116	6,98872	7,098261	6,506795	6,541018	6,838405	6,715358	-0,123047	0,918246247	---
17533040	5,860888	6,062129	6,043119	5,821364	5,81043	5,965228	5,988712	5,865674	-0,123038	0,918251976	Slc35a2
17516247	2,894225	3,31757	3,134341	2,959243	2,974907	3,042956	3,1153787	2,992368667	-0,12301	0,918269797	Olfr933
17423433	9,531003	9,681763	9,526608	9,391813	9,513493	9,465097	9,5797913	9,456801	-0,12299033	0,918282315	Otud6b
17444628	7,973167	8,110469	8,126326	7,883662	8,02231	7,935065	8,0699873	7,947012333	-0,122975	0,918292075	Bud31
17266563	8,507584	8,492613	8,5333	8,327288	8,459339	8,377966	8,5111657	8,388197667	-0,122968	0,918296531	Wsb1
17337863	4,201131	4,020284	4,304525	4,037233	3,91234	4,207511	4,1753133	4,052361333	-0,122952	0,918306715	---
17242651	3,265916	3,232486	2,921035	2,854576	2,957698	3,238333	3,1398123	3,016869	-0,12294333	0,918312231	---
17347987	4,348667	4,233618	4,593511	4,583296	4,272879	3,950797	4,391932	4,268990667	-0,12294133	0,918313504	Gm19783
17202235	5,890425	6,527731	6,174394	6,461543	6,377586	5,384621	6,1975167	6,074583333	-0,12293333	0,918318597	---
17476924	5,142019	4,706229	4,391095	4,450546	4,851141	4,568876	4,7464477	4,623521	-0,12292667	0,91832284	4930433I11Rik
17204415	6,369301	6,502991	6,341825	6,226622	6,26824	6,350592	6,4047057	6,281818	-0,12288767	0,918347665	---
17283617	3,491932	3,658035	3,712227	3,364159	3,564338	3,565125	3,6207313	3,497874	-0,12285733	0,918366974	Serpina1d
17397390	4,233133	4,866719	4,112525	4,645195	3,816864	4,381765	4,4041257	4,281274667	-0,122851	0,918371006	---
17413078	5,837852	6,105876	6,448119	6,23323	5,843669	5,946481	6,1306157	6,007793333	-0,12282233	0,918389254	Ccl21b
17434230	5,837852	6,105876	6,448119	6,23323	5,843669	5,946481	6,1306157	6,007793333	-0,12282233	0,918389254	Ccl21b
17286973	7,891829	8,476326	8,515785	8,19978	8,165796	8,149904	8,2946467	8,171826667	-0,12282	0,91839074	Gmpr
17439997	5,018417	4,781785	4,944419	4,823525	4,729218	4,823525	4,9148737	4,792089333	-0,12278433	0,918413445	Brdt
17433029	6,312565	6,223426	6,56839	6,236801	6,043257	6,456074	6,368127	6,245377333	-0,12274967	0,918435513	Apitd1
17255727	6,513297	6,730041	6,820433	6,591853	6,524648	6,579057	6,6879237	6,565186	-0,12273767	0,918443153	Osbpl7
17387688	3,256692	3,194245	3,203749	3,191987	3,121015	2,973477	3,2182287	3,095493	-0,12273567	0,918444426	Olfr1079
17359967	7,295528	7,4073	7,327359	7,224355	7,167811	7,269839	7,3433957	7,220668333	-0,12272733	0,918449731	Nfkb2
17206529	3,795095	3,403468	3,281153	3,195377	3,302518	3,613641	3,4932387	3,370512	-0,12272667	0,918450156	---
17285432	3,676297	4,019889	3,732347	3,940557	3,605258	3,514574	3,809511	3,686796333	-0,12271467	0,918457795	A530046M15
17222053	4,705399	4,899589	5,317391	4,809169	5,025173	4,719894	4,9741263	4,851412	-0,12271433	0,918458007	Bend6
17405893	4,093462	4,109516	4,577108	3,928575	4,255709	4,227676	4,2600287	4,13732	-0,12270867	0,918461615	Slitrk3
17504122	5,317185	4,992763	5,130643	5,276089	4,915549	4,880971	5,1468637	5,024203	-0,12266067	0,918492174	Ccl22
17476162	3,869457	4,040851	3,916883	3,860767	3,841308	3,757134	3,942397	3,819736333	-0,12266067	0,918492174	---
17467232	7,41747	7,7836	7,764217	7,786917	7,444591	7,365884	7,6550957	7,532464	-0,12263167	0,918510637	Gprin3
17372751	3,857915	3,681198	3,432923	3,389319	3,486891	3,72796	3,6573453	3,534723333	-0,122622	0,918516791	Olfr1008
17277561	5,142542	5,02238	5,161908	5,115066	4,93676	4,907139	5,1089433	4,986321667	-0,12262167	0,918517003	Tmem63c
17479647	5,712896	5,614098	5,701912	5,381176	5,742013	5,537867	5,676302	5,553685333	-0,12261667	0,918520187	Slc28a1
17516950	3,129375	3,273723	3,13888	3,122519	2,995127	3,056527	3,1806593	3,058057667	-0,12260167	0,918529737	---
17515045	7,96077	8,026031	8,031073	7,695733	8,02401	7,930341	8,005958	7,883361333	-0,12259667	0,91853292	Ppan
17203001	5,981731	6,005053	5,314965	5,436194	5,801376	5,69641	5,7672497	5,64466	-0,12258967	0,918537377	---
17486496	3,788173	3,915169	3,699561	3,768826	3,657855	3,608507	3,8009677	3,678396	-0,12257167	0,918548837	Zscan4e
17401343	6,269798	6,310144	6,526675	6,136891	6,172341	6,429754	6,3688723	6,246328667	-0,12254367	0,918566665	---
17255246	6,014636	6,1643	6,310431	6,371424	5,78042	5,97009	6,1631223	6,040644667	-0,12247767	0,918608688	---
17326198	4,133718	4,157529	4,224177	4,09296	4,21078	3,844263	4,171808	4,049334333	-0,12247367	0,918611235	Ccdc54
17550466	4,83601	4,47783	4,919739	4,466483	4,700105	4,699584	4,7445263	4,622057333	-0,122469	0,918614206	---
17501800	5,280969	5,249852	5,105081	5,229624	5,102397	4,936483	5,2119673	5,089501333	-0,122466	0,918616116	Hapln4
17200319	5,13984	4,744272	4,885886	4,825943	4,722498	4,854224	4,9233327	4,800888333	-0,12244433	0,918629913	---
17372264	4,609938	4,292443	4,578463	4,489739	4,222789	4,401019	4,4936147	4,371182333	-0,12243233	0,918637554	Gm13944
17411732	7,137005	6,910879	6,944453	6,838555	6,819661	6,966855	6,9974457	6,875023667	-0,122422	0,918644133	2610301B20Rik
17300374	10,22141	10,26954	10,48669	10,40037	10,1789	10,03116	10,32588	10,20347667	-0,12240333	0,918656019	---
17372644	10,61489	10,74485	10,73192	10,52145	10,59052	10,6125	10,69722	10,57482333	-0,12239667	0,918660265	Slc43a3
17399408	6,290416	6,36768	6,465337	6,262691	6,280508	6,213194	6,3744777	6,252131	-0,12234667	0,918692103	Adam15
17548350	6,466073	6,772883	6,65697	6,551855	6,43383	6,543225	6,6319753	6,509636667	-0,12233867	0,918697198	---
17321978	4,555231	4,483532	4,678664	4,142131	4,700539	4,507782	4,5724757	4,450150667	-0,122325	0,918705901	Krt71
17331583	3,088649	3,043606	3,049085	2,864744	3,049254	2,90038	3,0604467	2,938126	-0,12232067	0,91870866	---
17520071	3,593288	3,568289	3,421541	3,600321	3,468901	3,147014	3,527706	3,405412	-0,122294	0,918725642	---
17515982	3,131622	3,695421	2,86056	3,179981	3,003249	3,137493	3,229201	3,106907667	-0,12229333	0,918726066	---
17550376	4,138659	3,948325	4,139364	3,713866	4,317662	3,827982	4,0754493	3,95317	-0,12227933	0,918734982	---
17207901	5,377567	5,878375	4,934589	5,464892	6,013533	4,345404	5,3968437	5,274609667	-0,122234	0,918763851	---
17284339	4,431673	4,364637	4,562417	4,324032	4,176112	4,491899	4,452909	4,330681	-0,122228	0,918767672	---
17206673	4,917724	5,002834	5,959698	5,23871	5,249298	5,025632	5,2934187	5,171213333	-0,12220533	0,918782107	---
17422959	7,558351	7,703139	7,319908	7,508949	7,297272	7,408596	7,5271327	7,404939	-0,12219367	0,918789537	Tmem68
17315507	5,104623	4,744188	5,198575	4,831146	4,987988	4,861687	5,0157953	4,893607	-0,12218833	0,918792934	Hoxc9
17276002	4,490589	4,168188	4,790525	4,60536	4,213558	4,263824	4,4831007	4,360914	-0,12218667	0,918793995	F730035M05Rik
17212341	2,658198	2,769903	2,711723	2,646818	2,593351	2,53314	2,7132747	2,591103	-0,12217167	0,918803548	---
17428303	3,063742	3,402123	3,03223	3,028127	3,118193	2,985316	3,1660317	3,043878667	-0,122153	0,918815437	Skint6
17232560	9,649724	9,583526	9,52437	9,542055	9,360462	9,488712	9,5858733	9,463743	-0,12213033	0,918829872	Hdac2
17550771	3,452034	3,469657	3,634996	3,382648	3,452304	3,355385	3,5188957	3,396779	-0,12211667	0,918838577	---
17379229	6,184636	5,986699	6,48457	5,970331	6,113033	6,206196	6,218635	6,09652	-0,122115	0,918839638	Tox2
17515272	7,351839	7,226168	7,43293	7,24955	7,150585	7,244459	7,336979	7,214864667	-0,12211433	0,918840063	1810026J23Rik
17205485	3,640632	3,401884	3,574944	3,094728	3,726533	3,429959	3,5391533	3,417073333	-0,12208	0,91886193	---
17404877	3,687872	3,512877	3,783913	3,632827	3,441876	3,543748	3,661554	3,539483667	-0,12207033	0,918868086	---
17283837	4,40199	4,514695	4,648088	4,397197	4,447241	4,354145	4,521591	4,399527667	-0,12206333	0,918872545	4933406K04Rik
17405456	3,608144	3,467536	3,361432	3,347855	3,197717	3,525375	3,4790373	3,356982333	-0,122055	0,918877852	---
17259600	8,025025	8,310861	8,125957	7,98981	8,005128	8,100744	8,1539477	8,031894	-0,12205367	0,918878702	Foxk2
17200165	3,23055	3,000852	4,027285	3,117303	3,496119	3,279127	3,4195623	3,297516333	-0,122046	0,918883585	---
17232183	5,151522	4,956644	5,226597	4,956557	4,903387	5,108716	5,1115877	4,989553333	-0,12203433	0,918891015	Taar5
17216299	5,730824	6,04683	5,657431	5,553168	5,809235	5,706588	5,811695	5,689663667	-0,12203133	0,918892926	---
17413817	6,148146	6,258813	6,339851	6,128963	6,295371	5,956405	6,2489367	6,126913	-0,12202367	0,918897809	Foxe1
17279150	10,17758	9,947747	10,15789	10,05153	9,759776	10,10586	10,094406	9,972388667	-0,122017	0,918902056	---
17287320	5,221016	5,471171	5,449201	5,268964	5,428748	5,077667	5,3804627	5,258459667	-0,122003	0,918910973	Nxnl2
17290937	5,901762	6,902604	6,825756	6,078457	7,217078	5,968599	6,543374	6,421378	-0,121996	0,918915431	---
17448174	7,915193	8,317313	7,85494	8,207598	7,927534	7,586373	8,0291487	7,907168333	-0,12198033	0,91892541	Arap2
17378287	5,115027	5,218328	5,232866	5,023659	5,236149	4,940511	5,1887403	5,066773	-0,12196733	0,91893369	a
17363150	4,447845	4,327337	4,32335	4,425692	4,212839	4,094117	4,3661773	4,244216	-0,12196133	0,918937512	Olfr1465
17548063	5,849073	5,878196	6,120873	5,971687	5,717703	5,792878	5,9493807	5,827422667	-0,121958	0,918939635	Fam53c // Fam53c
17206043	6,661125	5,842766	6,750252	6,261925	6,418078	6,2083	6,4180477	6,296101	-0,12194667	0,918946854	---
17325661	3,657939	3,730898	3,580425	3,608631	3,564301	3,430492	3,6564207	3,534474667	-0,121946	0,918947279	---
17446915	9,779187	9,883986	9,900702	9,748149	9,766046	9,683871	9,854625	9,732688667	-0,12193633	0,918953436	Ppm1g
17221542	3,095651	3,164706	3,242568	2,983016	3,106447	3,047661	3,1676417	3,045708	-0,12193367	0,918955135	---
17395016	8,992626	9,236465	9,157491	8,967875	8,95109	9,101887	9,1288607	9,006950667	-0,12191	0,91897021	Aurka
17412045	3,78436	3,059794	3,368951	3,338538	3,175621	3,333328	3,4043683	3,282495667	-0,12187267	0,918993991	---

17247428	5,99171	5,967997	5,785319	5,854779	5,662547	5,862095	5,9150087	5,793140333	-0,12186833	0,918996751	Fbxo48
17428589	3,711621	3,355587	4,032089	3,598706	3,636621	3,498458	3,6997657	3,577928333	-0,12183733	0,919016499	Tspan1
17342993	5,06669	5,126259	5,288846	5,121807	4,827478	5,16706	5,1605983	5,038781667	-0,12181667	0,919029664	---
17379487	5,740572	5,719762	5,836244	5,750005	5,544638	5,636789	5,765526	5,643810667	-0,12171533	0,919094217	Wfdc13
17345919	3,48429	3,224824	3,349414	3,441316	3,269361	2,98272	3,3528427	3,231132333	-0,12171033	0,919097403	Tbc1d5
17503023	9,369017	9,291882	9,277282	9,199222	9,131907	9,241983	9,312727	9,191037333	-0,12168967	0,919110569	Asf1b
17204207	5,257694	5,123006	4,985618	5,017394	5,21752	4,766393	5,122106	5,000435667	-0,12167033	0,919122886	---
17346975	7,163658	7,300201	7,452966	7,108752	7,275196	7,167937	7,3056083	7,183961667	-0,12164667	0,919137964	Emilin2
17224551	5,116194	5,099366	5,164008	4,816195	5,002912	5,19553	5,1265227	5,004879	-0,12164367	0,919139875	Ptprn
17514471	9,371089	9,610483	9,390119	9,2718	9,322879	9,41212	9,4572303	9,335599667	-0,12163067	0,919148157	Dcun1d5
17462663	5,623064	6,619404	6,18324	5,690337	6,041486	6,329	6,1419027	6,020274333	-0,12162833	0,919149644	Mfap5
17548713	6,711176	6,504624	6,63973	6,377193	6,589296	6,524186	6,61851	6,496891667	-0,12161833	0,919156015	LOC100862285
17528554	5,093891	5,138605	5,797605	5,682676	5,188751	4,793879	5,343367	5,221768667	-0,12159833	0,919168757	Gm16144
17294320	6,847682	6,566871	6,58732	6,661731	6,499134	6,476219	6,667291	6,545694667	-0,12159633	0,919170032	---
17383965	7,438284	7,486516	7,50612	7,213799	7,477504	7,374851	7,4769733	7,355384667	-0,12158867	0,919174916	Cdk9
17268220	5,601682	5,451098	5,6706	5,292377	5,611997	5,454298	5,57446	5,452890667	-0,12156933	0,919187234	---
17272895	6,091434	5,80091	6,041597	6,150689	5,536624	5,881967	5,9779803	5,856426667	-0,12155367	0,919197216	Eif4a3
17549352	3,80568	3,945311	3,481866	3,966151	3,571854	3,330203	3,7442857	3,622736	-0,12154967	0,919199764	---
17549740	3,80568	3,945311	3,481866	3,966151	3,571854	3,330203	3,7442857	3,622736	-0,12154967	0,919199764	---
17321849	6,863368	6,416085	6,611253	6,650599	6,307242	6,568349	6,6302353	6,50873	-0,12150533	0,919228011	Krt81
17438299	4,490355	4,583067	4,109951	4,347565	4,031951	4,439342	4,3944577	4,272952667	-0,121505	0,919228224	---
17548585	9,787384	9,925112	9,735029	9,592172	9,699033	9,791807	9,8158417	9,694337333	-0,12150433	0,919228649	Twf1 // Twf1
17250095	2,958923	2,974133	3,122276	2,897594	2,897594	2,8957	3,018444	2,896962667	-0,12148133	0,919243303	Olfr319
17246707	8,398686	8,321244	7,968748	7,900551	8,001245	8,422457	8,2295593	8,108084333	-0,121475	0,919247339	Sec14l4
17417598	7,683295	7,754392	7,660718	7,559467	7,596696	7,577884	7,6994683	7,578015667	-0,12145267	0,919261569	Eif2b3
17463781	7,531117	7,515546	8,060063	7,5712	7,502525	7,668643	7,702242	7,580789333	-0,12145267	0,919261569	Emp1
17346030	4,792611	4,896131	4,890092	4,849979	4,507679	4,856848	4,8596113	4,738168667	-0,12144267	0,919267941	1700025K24Rik // 1700025K24Rik
17438334	4,472378	4,42472	4,471245	4,211213	4,416646	4,376179	4,4561143	4,334679333	-0,121435	0,919272826	Gm7271
17473650	5,46699	4,963927	5,540333	5,229875	5,328946	5,048142	5,32375	5,202321	-0,121429	0,919276649	Aurkc
17422404	6,62038	6,450035	6,566608	6,227428	6,433445	6,611864	6,5456743	6,424245667	-0,12142867	0,919276862	Ttc34
17305012	7,228986	6,929138	7,259163	7,074302	7,049471	6,929405	7,1390957	7,017726	-0,12136967	0,919314457	Gprin2
17201373	3,17054	3,144402	4,202234	3,279245	3,299589	3,574237	3,5057253	3,384357	-0,12136833	0,919315307	---
17235990	6,30463	6,059154	6,139494	5,973124	6,080456	6,085768	6,1677593	6,046449333	-0,12131	0,919352478	1500009L16Rik
17395029	6,193878	6,313664	6,274587	6,200793	6,180441	6,036979	6,2607097	6,139404333	-0,12130533	0,919355452	Cass4
17535984	3,742525	3,742525	3,635613	3,617155	3,757574	3,382059	3,7068877	3,585596	-0,12129167	0,919364161	Rab39b // Rab39b
17467556	8,244929	8,5825	8,470851	8,282332	8,348647	8,303481	8,43276	8,311486667	-0,12127333	0,919375844	Rpia
17248132	3,642829	3,605428	3,710338	3,500229	3,559591	3,53497	3,652865	3,531596667	-0,12126833	0,919379031	Gm12100
17204277	7,053475	5,885189	6,391965	5,622112	7,120673	6,224202	6,443543	6,322329	-0,121214	0,919413656	---
17265973	4,182689	4,606086	4,424161	4,232605	4,496545	4,120231	4,404312	4,283127	-0,121185	0,919432138	---
17324135	5,568054	5,71813	5,689863	5,546579	5,556836	5,509153	5,6586823	5,537522667	-0,12115967	0,919448283	Chrd
17491537	7,840038	7,492102	7,148043	7,798498	6,894365	7,423843	7,4933943	7,372235333	-0,121159	0,919448708	Snord116
17491539	7,840038	7,492102	7,148043	7,798498	6,894365	7,423843	7,4933943	7,372235333	-0,121159	0,919448708	Snord116
17491566	7,840038	7,492102	7,148043	7,798498	6,894365	7,423843	7,4933943	7,372235333	-0,121159	0,919448708	Snord116
17491570	7,840038	7,492102	7,148043	7,798498	6,894365	7,423843	7,4933943	7,372235333	-0,121159	0,919448708	Snord116
17491572	7,840038	7,492102	7,148043	7,798498	6,894365	7,423843	7,4933943	7,372235333	-0,121159	0,919448708	Snord116
17491574	7,840038	7,492102	7,148043	7,798498	6,894365	7,423843	7,4933943	7,372235333	-0,121159	0,919448708	Snord116
17491576	7,840038	7,492102	7,148043	7,798498	6,894365	7,423843	7,4933943	7,372235333	-0,121159	0,919448708	Snord116
17491578	7,840038	7,492102	7,148043	7,798498	6,894365	7,423843	7,4933943	7,372235333	-0,121159	0,919448708	Snord116
17491580	7,840038	7,492102	7,148043	7,798498	6,894365	7,423843	7,4933943	7,372235333	-0,121159	0,919448708	Snord116
17491582	7,840038	7,492102	7,148043	7,798498	6,894365	7,423843	7,4933943	7,372235333	-0,121159	0,919448708	Snord116
17491584	7,840038	7,492102	7,148043	7,798498	6,894365	7,423843	7,4933943	7,372235333	-0,121159	0,919448708	Snord116
17491586	7,840038	7,492102	7,148043	7,798498	6,894365	7,423843	7,4933943	7,372235333	-0,121159	0,919448708	Snord116
17491588	7,840038	7,492102	7,148043	7,798498	6,894365	7,423843	7,4933943	7,372235333	-0,121159	0,919448708	Snord116
17491590	7,840038	7,492102	7,148043	7,798498	6,894365	7,423843	7,4933943	7,372235333	-0,121159	0,919448708	Snord116
17491592	7,840038	7,492102	7,148043	7,798498	6,894365	7,423843	7,4933943	7,372235333	-0,121159	0,919448708	Snord116
17491594	7,840038	7,492102	7,148043	7,798498	6,894365	7,423843	7,4933943	7,372235333	-0,121159	0,919448708	Snord116
17291186	4,499847	4,493905	4,97252	4,478379	4,489888	4,634529	4,655424	4,534265333	-0,12115867	0,91944892	Hist1h2ae
17337120	7,262631	7,434979	6,731906	7,028704	7,202346	6,835073	7,143172	7,022041	-0,121131	0,919466553	H2-Q7 // H2-Q7
17336660	7,519503	7,676302	7,709825	7,572814	7,439315	7,530132	7,63521	7,514087	-0,121123	0,919471651	Atf6b
17516908	7,447574	8,202598	8,075262	7,91555	7,767704	7,678825	7,908478	7,787359667	-0,12111833	0,919474626	Apoa4
17230327	8,87176	8,956032	8,873316	8,795645	8,839933	8,702229	8,9003693	8,779269	-0,12110033	0,919486098	---
17549982	2,931083	3,118096	3,037888	2,840649	2,999747	2,88341	3,0290223	2,907935333	-0,121087	0,919494595	---
17301738	5,699506	5,508761	5,846465	5,515703	5,662493	5,513288	5,6849107	5,563828	-0,12108267	0,919497357	Phyhip
17410043	7,774434	7,643765	7,721294	7,596633	7,480015	7,699636	7,7131643	7,592094667	-0,12106967	0,919505643	Mettl14
17221093	8,29291	8,412063	8,248283	7,971979	8,191204	8,426886	8,317752	8,196689667	-0,12106233	0,919510317	---
17540713	6,399554	5,720027	5,086476	5,974401	5,177826	5,690658	5,7353523	5,614295	-0,12105733	0,919513504	Gm6121
17531130	9,027978	9,318336	9,296122	9,062752	9,142764	9,073753	9,2141453	9,093089667	-0,12105567	0,919514566	Apeh
17288544	5,184061	4,667871	5,237867	5,176276	4,766771	4,783601	5,029933	4,908882667	-0,12105033	0,919517965	5430425J12Rik
17207991	3,642579	3,461829	3,699416	3,657612	3,417031	3,366053	3,6012747	3,480232	-0,12104267	0,919522852	---
17415302	5,183206	4,969398	5,474384	4,813342	5,323253	5,127407	5,208996	5,088000667	-0,12099533	0,919553021	---
17219282	6,208721	6,221599	6,256331	6,136456	6,077539	6,109819	6,2288837	6,107938	-0,12094567	0,919584678	LOC100862110
17471321	5,45516	5,338914	5,691105	5,416205	5,280263	5,425937	5,4950597	5,374135	-0,12092467	0,919598064	---
17225368	4,117899	4,034346	4,29328	4,078104	4,106737	3,897973	4,1485083	4,027604667	-0,12090367	0,919611449	4933400F21Rik
17550504	4,092593	3,954858	4,241591	3,932076	3,988678	4,00563	4,0963473	3,975461333	-0,120886	0,919622711	---
17324673	10,91471	11,07237	10,95433	10,79199	11,0085	10,7783	10,98047	10,85959667	-0,12087333	0,919630785	---
17236832	5,389086	5,370328	5,568666	5,241559	5,363995	5,359931	5,4426933	5,321828333	-0,120865	0,919636097	4921510H08Rik
17412852	8,030787	8,010417	8,166658	7,923952	7,961745	7,959575	8,0692873	7,948424	-0,12086333	0,919637159	---
17440728	7,86191	7,769646	7,997806	7,849386	7,620347	7,797041	7,876454	7,755591333	-0,12086267	0,919637584	Aym1
17434475	9,751186	9,693494	9,924333	9,408156	9,667578	9,930758	9,789671	9,668830667	-0,12084033	0,919651821	Hspa8 // Hspa8
17245302	11,16286	11,20637	11,06148	10,92199	11,07329	11,07293	11,14357	11,02273667	-0,12083333	0,919656283	Rap1b
17548667	4,874759	4,968011	5,000471	4,725525	4,833726	4,921509	4,947747	4,82692	-0,120827	0,91966032	Gm19515
17257235	9,165623	9,162332	9,02492	8,921777	9,127795	8,940999	9,117625	8,996857	-0,120768	0,919697931	Itgb3
17332851	9,110254	9,031277	9,026418	8,928954	9,070128	8,806599	9,055983	8,935227	-0,120756	0,919705581	Gtf2h5
17520305	3,064919	3,126205	3,051191	3,087334	2,868474	2,924273	3,0807717	2,960027	-0,12074467	0,919712806	---
17500195	5,924699	6,377534	6,194316	6,170154	6,042927	5,921237	6,1655163	6,044772667	-0,12074367	0,919713443	Star
17202007	2,845369	2,85383	3,311569	2,928529	2,787968	2,932085	3,0035893	2,882860667	-0,12072867	0,919723006	---
17344620	5,554436	5,809909	6,089001	5,621605	5,699161	5,770417	5,817782	5,697061	-0,120721	0,919727893	Gm11127
17313047	4,975665	5,167945	4,988115	5,093037	5,008746	4,66778	5,0439083	4,923187667	-0,12072067	0,919728106	Gm10856
17251721	4,386485	4,451303	4,500754	4,497217	4,132315	4,346875	4,4461807	4,325469	-0,12071167	0,919733843	---
17549232	3,522911	3,397157	4,255212	3,545012	3,655258	3,61296	3,7250933	3,60441	-0,12068333	0,919751906	---
17277519	3,089659	3,199754	2,964541	2,985018	2,917209	2,989817	3,0846513	2,964014667	-0,12063667	0,919781658	---
17389638	4,574697	4,580755	4,861429	4,369861	4,78189	4,503242	4,6722937	4,551664333	-0,12062933	0,919786333	2810405F15Rik
17252310	5,959289	5,822263	5,938675	5,669476	6,009447	5,679429	5,9067423	5,786117333	-0,120625	0,919789096	Fam64a
17254827	9,267374	9,533602	9,470674	9,173303	9,488125	9,248431	9,4238833	9,303286333	-0,120597	0,919806948	Supt4h1
17441018	4,517554	4,758667	4,767443	4,605711	4,66384	4,412337	4,6812213	4,560629333	-0,120592	0,919810035	4930519G04Rik
17513534	5,364538	5,405916	5,351856	5,149168	5,291786	5,319609	5,3741033	5,253521	-0,12058233	0,919816299	1190005I06Rik
17214904	7,73444	7,358315	7,279673	7,55234	7,197309	7,261069	7,457476	7,336906	-0,12057	0,919824162	C130026I21Rik
17365981	4,482521	4,194561	4,265842	4,250966	4,308771	4,021489	4,314308	4,193742	-0,120566	0,919826712	1700019N19Rik // 1700019N19Rik
17257560	9,90238	9,539639	9,641496	9,650334	9,60669	9,464853	9,694505	9,573959	-0,120546	0,919839464	---
17429274	4,028415	4,194709	3,989966	3,787577	4,13381	3,930097	4,07103	3,950494667	-0,12053533	0,919846265	Olfr1342
17550781	3,750895	3,875763	3,669407	3,515651	3,706578	3,712319	3,765355	3,644849333	-0,12050567	0,91986518	---
17549498	3,938056	3,880366	3,920873	3,85115	3,784607	3,742062	3,9130983	3,792606333	-0,120492	0,919873894	---
17403004	6,730663	7,205667	6,738372	6,704876	6,586807	7,021557	6,8915673	6,77108	-0,12048733	0,91987687	H2afz
17474844	5,010952	4,54728	4,390265	4,463832	4,359723	4,763483	4,649499	4,529012667	-0,12048633	0,919877507	Vmn1r169
17549512	6,771981	6,803051	7,124462	6,689054	6,94229	6,706751	6,8998313	6,779365	-0,12046633	0,919890259	---
17549664	6,771981	6,803051	7,124462	6,689054	6,94229	6,706751	6,8998313	6,779365	-0,12046633	0,919890259	---
17378637	4,531458	4,575943	4,815593	4,643642	4,477833	4,440156	4,640998	4,520543667	-0,12045433	0,919897911	Gm14168
17366790	5,683606	5,52109	5,287574	5,540963	5,264019	5,325933	5,4974233	5,376971667	-0,12045167	0,919899611	---
17366826	5,683606	5,52109	5,287574	5,540963	5,264019	5,325933	5,4974233	5,376971667	-0,12045167	0,919899611	Mir669k
17488221	7,674934	7,854975	7,941426	7,653595	7,822262	7,634144	7,8237783	7,703333667	-0,12044467	0,919904075	Shkbp1
17445525	4,548399	4,52511	4,69633	4,345678	4,398222	4,664672	4,5899463	4,469524	-0,12042233	0,919918315	Rundc3b
17277622	5,046224	4,906279	4,846638	5,052188	4,792411	4,593324	4,933047	4,812641	-0,120406	0,91992873	---
17511660	7,650291	8,087929	7,897309	7,829838	7,671085	7,773612	7,8785097	7,758178333	-0,12033133	0,919976342	Ces1b
17402513	6,590522	6,532699	6,606103	6,539843	6,414884	6,41363	6,5764413	6,456119	-0,12032233	0,919982081	Neurog2
17210657	7,569335	7,201077	7,682997	7,202734	7,387548	7,502459	7,4844697	7,364247	-0,12022267	0,920045639	---
17403875	5,979071	6,068922	6,426908	6,100398	6,090966	5,922897	6,1583003	6,038087	-0,12021333	0,920051591	---
17284648	6,154865	6,269786	6,19632	6,090116	6,070558	6,099683	6,2069903	6,086785667	-0,12020467	0,920057118	Ighm // Ighm
17405817	9,827051	10,13609	10,20443	10,22528	9,919745	9,661942	10,055857	9,935655667	-0,12020133	0,920059244	---
17285867	12,07292	12,11681	12,26758	12,11636	12,13299	11,8474	12,152437	12,03225	-0,12018667	0,920068598	Hist1h2ab
17425870	4,908611	5,078131	5,070797	4,85202	5,037564	4,807425	5,0191797	4,899003	-0,12017667	0,920074975	Slc46a2
17200397	3,75433	4,14102	3,928906	3,618593	3,930929	3,914208	3,9414187	3,821243333	-0,12017533	0,920075825	---
17300133	2,963377	2,97791	3,086246	2,930257	2,874377	2,862421	3,0091777	2,889018333	-0,12015933	0,920086029	---
17259792	3,574821	3,494044	3,706122	3,577685	3,394631	3,442258	3,5916623	3,471524667	-0,12013767	0,920099848	---
17299823	3,393874	3,292391	3,136259	3,146558	3,202116	3,1135	3,2741747	3,154058	-0,12011667	0,920113241	---
17512755	10,55156	10,69739	10,47611	10,40121	10,41613	10,54747	10,57502	10,45493667	-0,12008333	0,9201345	Psmd7
17349089	3,904617	3,81438	3,811255	3,93078	3,636362	3,602897	3,8434173	3,723346333	-0,120071	0,920142366	4930474G06Rik
17266891	7,1814	7,284839	7,105255	7,166728	6,960316	7,084302	7,190498	7,070448667	-0,12004933	0,920156185	Pex12
17449920	6,305129	6,26668	6,04482	5,906907	6,177892	6,171703	6,205543	6,085500667	-0,12004233	0,92016065	Paqr3
17300328	8,493748	8,533266	8,470902	8,260711	8,430976	8,446116	8,4993053	8,379267667	-0,12003767	0,920163626	1700123O20Rik
17372738	3,910877	4,107579	4,110593	3,863538	3,850736	4,054679	4,0430163	3,922984333	-0,120032	0,92016724	---
17387778	5,195704	4,29332	4,763389	4,978361	4,835312	4,078687	4,7508043	4,630786667	-0,12001767	0,920176382	Olfr1141
17387720	4,192293	3,878365	3,903768	3,737528	4,094521	3,782378	3,9914753	3,871475667	-0,11999967	0,920187863	Olfr1098
17405097	3,172541	3,220998	3,366862	2,879913	3,344183	3,176357	3,253467	3,133484333	-0,11998267	0,920198706	---
17473720	4,587776	4,757246	4,597149	4,617874	4,65679	4,307587	4,6473903	4,527417	-0,11997333	0,920204659	---

17333402	6,887305	6,295876	5,868501	6,088162	6,330894	6,272761	6,3505607	6,230605667	-0,119955	0,920216353	Gm16050
17401590	4,867679	4,727856	4,781136	4,782244	4,555576	4,679019	4,7922237	4,672279667	-0,119944	0,92022337	Eps8l3
17236816	4,882022	4,801355	4,890949	4,538611	5,029418	4,646593	4,8581087	4,738207333	-0,11990133	0,920250585	Kera
17269638	9,136394	9,245509	9,197675	9,10995	9,052075	9,057858	9,1931927	9,073294333	-0,11989833	0,920252498	Rab5c
17208041	3,869964	3,458512	4,43287	3,808999	3,469652	4,123022	3,9204487	3,800557667	-0,119891	0,920257176	---
17370855	3,999271	3,987746	3,904905	3,820076	4,030267	3,681939	3,963974	3,844094	-0,11988	0,920264193	Galnt13
17490233	9,148026	9,008386	9,101168	8,816302	9,082955	8,998685	9,08586	8,965980667	-0,11987933	0,920264618	2310044H10Rik
17311459	2,942133	3,180318	2,83544	2,908266	2,891436	2,798559	2,9859637	2,866087	-0,11987667	0,920266319	---
17324637	7,333013	7,35102	7,152988	7,241627	7,136248	7,099619	7,279007	7,159164667	-0,11984233	0,92028822	Fam43a
17342996	6,045565	6,354626	6,609499	6,041487	6,089792	6,518893	6,3365633	6,216724	-0,11983933	0,920290134	Gm16197
17237280	7,660825	7,616195	7,627501	7,423182	7,548189	7,573653	7,6348403	7,515008	-0,11983233	0,920294599	Phlda1
17219378	6,445176	5,555359	6,184966	5,912973	5,68634	6,226699	6,0618337	5,942004	-0,11982967	0,9202963	---
17255531	4,440871	4,376341	4,752242	4,162566	4,738445	4,308971	4,5231513	4,403327333	-0,119824	0,920299915	---
17246098	3,022409	2,852448	3,040063	2,90081	2,796061	2,858603	2,97164	2,851824667	-0,11981533	0,920305443	---
17204371	5,337436	3,544411	5,17046	5,212527	4,479618	4,000756	4,6841023	4,564300333	-0,119802	0,920313949	---
17455536	4,895837	4,782983	5,021108	4,696834	4,880476	4,763247	4,899976	4,780185667	-0,11979033	0,920321391	LOC100861794
17542096	3,139306	2,872583	2,894924	2,994249	2,694059	2,859144	2,9689377	2,849150667	-0,119787	0,920323517	Mir465
17407246	5,150826	4,902583	5,08796	4,987957	4,702653	5,091424	5,047123	4,927344667	-0,11977833	0,920329046	Creb3l4
17482131	2,911181	3,292704	3,02646	3,023962	2,885833	2,961238	3,0767817	2,957011	-0,11977067	0,920333937	---
17499831	4,21365	4,355456	4,254719	4,422564	3,960088	4,08187	4,2746083	4,154840667	-0,11976767	0,920335851	Defb13
17258457	7,767258	7,581245	7,574428	7,274333	7,706677	7,582622	7,640977	7,521210667	-0,11976633	0,920336701	Sap30bp
17478191	3,839903	3,966552	3,923542	3,797104	3,682015	3,891707	3,909999	3,790275333	-0,11972367	0,92036392	---
17358874	3,023262	3,052285	3,006511	2,871281	3,040228	2,811399	3,0273527	2,907636	-0,11971667	0,920368386	---
17210439	4,802898	4,510862	5,474726	4,569301	4,937405	4,922664	4,9294953	4,80979	-0,11970533	0,920375616	---
17468930	4,46724	4,597743	4,52747	4,473725	4,567802	4,191876	4,5308177	4,411134333	-0,11968333	0,920389651	---
17538742	4,246467	3,908258	4,271547	3,819718	4,215019	4,032609	4,1420907	4,022448667	-0,119642	0,92041602	---
17202323	6,091073	6,449831	6,190056	6,509857	6,253685	5,608562	6,2436533	6,124034667	-0,11961867	0,920430907	---
17202949	5,274141	6,193253	6,433261	6,170405	5,266088	6,10544	5,966885	5,847311	-0,119574	0,920459404	---
17245511	8,657764	8,698469	8,67472	8,639441	8,533625	8,499166	8,6769843	8,557410667	-0,11957367	0,920459617	Xpot
17437977	5,517593	5,33379	5,433843	5,291917	5,359909	5,274748	5,4284087	5,308858	-0,11955067	0,920474292	---
17206137	3,855036	3,193505	2,957878	3,112652	2,941026	3,59409	3,335473	3,215922667	-0,11955033	0,920474504	---
17407621	5,229172	5,425077	5,355158	5,111427	5,338651	5,200733	5,336469	5,216937	-0,119532	0,920486201	BC021767
17233740	11,01798	11,07382	11,03522	10,9496	10,84065	10,9782	11,04234	10,92281667	-0,11952333	0,920491731	---
17444770	5,804996	5,469393	5,367421	5,414167	5,42453	5,444553	5,54727	5,42775	-0,11952	0,920493858	Gm3404
17547771	3,797477	3,845516	3,793614	3,729705	3,693569	3,654797	3,8122023	3,692690333	-0,119512	0,920498962	Serpinb9b // Serpinb9b
17371503	10,61872	10,8082	10,69838	10,61592	10,51701	10,63387	10,708433	10,58893333	-0,1195	0,920506619	Ssb
17436832	5,454633	5,279607	5,4535	5,251431	5,518062	5,059776	5,3959133	5,276423	-0,11949033	0,920512786	Hmx1
17480566	9,981406	10,18061	10,15014	9,859316	10,04358	10,05086	10,104052	9,984585333	-0,11946667	0,920527887	---
17302354	4,9781	5,229928	5,20255	5,046697	4,969071	5,036419	5,1368593	5,017395667	-0,11946367	0,920529801	---
17469320	9,077499	9,193578	9,068728	8,918768	9,00808	9,054593	9,1132683	8,993813667	-0,11945467	0,920535544	Tmf1
17539105	6,302508	6,404538	6,263342	6,559678	6,236589	5,815814	6,3234627	6,204027	-0,11943567	0,920547667	---
17486790	9,043833	9,178974	9,371098	9,050282	9,040567	9,144788	9,1979683	9,078545667	-0,11942267	0,920555962	Gltscr2
17305906	4,569067	3,974617	4,311006	4,291526	4,235256	3,96965	4,2848967	4,165477333	-0,11941933	0,920558089	---
17354653	7,065063	7,031952	7,076538	7,03658	6,783003	6,995814	7,057851	6,938465667	-0,11938533	0,920579784	Tcof1
17210237	5,435328	5,75378	5,847651	5,819151	5,344875	5,514592	5,6789197	5,559539333	-0,11938033	0,920582975	---
17534315	3,811553	3,602809	3,73404	3,680719	3,514108	3,595467	3,716134	3,596764667	-0,11936933	0,920589994	Rhox10
17526820	6,207937	6,180719	5,703227	6,280807	5,795355	5,657647	6,0306277	5,911269667	-0,119358	0,920597226	Bco2
17550787	3,23253	3,227663	3,096924	2,956986	3,152372	3,089796	3,1857057	3,066384667	-0,119321	0,920620836	---
17207207	5,65511	5,845338	6,049909	6,071713	5,352585	5,768195	5,850119	5,730831	-0,119288	0,920641894	---
17212284	3,153102	3,419537	3,221124	3,173911	3,139295	3,122702	3,2645877	3,145302667	-0,119285	0,920643809	---
17468662	10,23071	10,42386	10,45041	10,21755	10,29718	10,23244	10,368327	10,24905667	-0,11927	0,920653381	---
17543106	4,776039	4,517487	4,631183	4,486257	4,565485	4,515224	4,6415697	4,522322	-0,11924767	0,920667633	Pet2
17464915	8,480251	8,368714	8,396656	8,184043	8,370571	8,33331	8,415207	8,295974667	-0,11923233	0,920677418	B630005N14Rik
17545824	5,502344	5,435001	5,712044	5,454808	5,367558	5,46933	5,5497963	5,430565333	-0,119231	0,920678269	Nhs
17441895	8,857089	9,099058	8,88431	8,874475	8,842911	8,765392	8,946819	8,827592667	-0,11922633	0,920681247	Naa25
17272544	4,906165	4,849146	4,824477	4,800941	4,784715	4,636512	4,8599293	4,740722667	-0,11920667	0,920693798	---
17245665	6,887371	6,568134	6,680798	6,531965	6,744006	6,502848	6,712101	6,592939667	-0,11916133	0,920722729	Xrcc6bp1
17443535	6,929784	7,126384	7,118118	7,021612	6,809926	6,985273	7,0580953	6,938937	-0,11915833	0,920724644	Ufsp1
17478116	5,567588	5,436302	5,563648	5,389182	5,253979	5,566944	5,5225127	5,403368333	-0,11914433	0,920733579	Otog
17222876	8,411682	8,767929	8,425468	8,295485	8,414303	8,537868	8,5350263	8,415885333	-0,119141	0,920735706	---
17376159	5,124509	4,873549	4,734919	4,773855	4,921869	4,679846	4,9109923	4,791856667	-0,11913567	0,92073911	Gm14047 // Gm14047
17239469	10,59642	10,65567	10,9207	10,6666	10,46262	10,68632	10,724263	10,60518	-0,11908333	0,92077251	---
17202259	6,076383	5,405302	6,031922	5,822065	5,59287	5,741497	5,837869	5,718810667	-0,11905833	0,920788466	---
17298757	4,310697	4,030072	4,360632	4,020116	4,073387	4,250726	4,2338003	4,114743	-0,11905733	0,920789104	Antxrl
17549008	3,757092	3,895586	3,783073	3,674061	3,6965	3,708038	3,811917	3,692866333	-0,11905067	0,920793359	---
17543885	4,896306	4,646637	4,760218	4,502961	4,575303	4,867822	4,7677203	4,648695333	-0,119025	0,920809741	Magee2
17425521	3,58221	3,278147	3,748879	3,346411	3,55207	3,353767	3,536412	3,417416	-0,118996	0,92082825	Mir32
17233064	7,873011	8,450665	8,141247	7,961792	7,950867	8,195277	8,1549743	8,035978667	-0,11899567	0,920828463	---
17454252	3,663169	3,576639	3,704953	3,670871	3,567369	3,349547	3,6482537	3,529262333	-0,11899133	0,920831229	Mir93
17232191	3,161094	3,361327	3,325972	3,130312	3,208731	3,15241	3,2827977	3,163817667	-0,11898	0,920838463	Taar7e
17414935	8,175244	7,942822	8,252131	8,18575	7,919838	7,9077	8,123399	8,004429333	-0,11896967	0,920845058	Kdm4c
17308319	4,771322	4,705223	4,871386	4,684508	4,733393	4,573129	4,7826437	4,663676667	-0,118967	0,92084676	Piwil2
17454794	5,867808	5,523464	5,681116	5,607118	5,73131	5,377088	5,690796	5,571838667	-0,11895733	0,92085293	Rbak
17491890	4,150017	4,83171	5,072635	4,380879	4,616599	4,700288	4,6847873	4,565854	-0,11893333	0,920868249	H47 // H47
17438246	7,954051	8,435102	7,991642	7,99299	8,042625	7,988406	8,1269317	8,008007	-0,11892467	0,920873781	Pdgfra
17210852	3,409788	3,189542	3,081464	2,973156	3,233534	3,117365	3,2269313	3,108018333	-0,118913	0,920881228	---
17550460	9,223548	9,511557	9,235929	9,231659	9,256302	9,126354	9,323678	9,204771667	-0,11890633	0,920885484	---
17412602	8,318988	8,301734	8,116823	8,13845	8,035258	8,207189	8,2458483	8,126965667	-0,11888267	0,92090059	Rngtt
17549590	7,544847	7,56744	7,905937	7,978755	6,979614	7,703228	7,6727413	7,553865667	-0,11887567	0,920905059	---
17381277	4,488098	4,578872	4,868247	4,592081	4,409445	4,577101	4,6450723	4,526209	-0,11886333	0,920912931	---
17448039	4,424332	4,277845	4,284978	4,424685	4,201111	4,00484	4,3290517	4,210212	-0,11883967	0,920928038	C130083M11Rik
17512151	8,798781	8,789411	8,404501	8,942867	8,136559	8,556777	8,664231	8,545401	-0,11883	0,920934209	---
17358905	6,236408	6,492745	6,23397	6,179738	6,42465	6,002284	6,321041	6,202224	-0,118817	0,920942508	Hectd2
17464963	5,283097	5,303684	5,544076	5,237607	5,243935	5,292882	5,3769523	5,258141333	-0,118811	0,920946338	---
17538960	4,881289	4,628362	4,524194	4,673817	4,567116	4,43649	4,6779483	4,559141	-0,11880733	0,920948678	Gpr143
17495414	8,722741	8,942713	8,497534	8,79297	8,544375	8,46929	8,720996	8,602211667	-0,11878433	0,920963361	Pik3c2a
17209745	4,143332	4,373606	3,888774	3,948802	3,981404	4,119332	4,1352373	4,016512667	-0,11872467	0,92100145	---
17204693	7,972316	8,026632	7,884966	7,74944	7,806368	7,971941	7,9613047	7,842583	-0,11872167	0,921003366	---
17310335	3,52306	3,667412	3,584878	3,714815	3,460293	3,244108	3,5917833	3,473072	-0,11871133	0,921009962	Gm19276
17450968	6,466657	6,493217	6,611798	6,437542	6,433828	6,3442	6,5238907	6,40519	-0,11870067	0,921016772	Gm15787
17450199	6,044659	5,926194	6,119606	5,914967	5,94599	5,873415	6,030153	5,911457333	-0,11869567	0,921019964	Nkx6-1
17321491	5,121532	5,087631	5,513175	5,390993	4,859322	5,115967	5,2407793	5,122094	-0,11868533	0,921026561	---
17292967	4,72168	4,693702	4,902222	4,484215	4,683242	4,794097	4,7725347	4,653851333	-0,11868333	0,921027838	Pitx1
17208171	3,182467	3,924979	3,324365	3,494162	3,310199	3,271405	3,4772703	3,358588667	-0,11868167	0,921028902	---
17352621	10,3879	10,54995	10,37652	10,30044	10,32021	10,33771	10,438123	10,31945333	-0,11867	0,92103635	Rock1
17201387	4,462097	4,344321	4,801381	4,527255	4,291329	4,433208	4,535933	4,417264	-0,118669	0,921036988	---
17499526	4,195861	3,642827	3,483538	3,738576	3,547588	3,680079	3,7740753	3,655414333	-0,118661	0,921042095	---
17437115	3,696819	3,625275	3,720485	3,644144	3,376724	3,665751	3,6808597	3,562206333	-0,11865333	0,92104699	---
17300231	6,491055	6,465245	6,777349	6,566277	6,341222	6,470199	6,577883	6,459232667	-0,11865033	0,921048905	---
17249375	10,20323	10,13993	10,14552	9,977722	10,12803	10,02698	10,162893	10,044244	-0,11864933	0,921049544	Cdkn2aipnl
17223308	5,520782	5,549752	5,41444	5,249619	5,519106	5,360452	5,4949913	5,376392333	-0,118599	0,921081678	4930558J18Rik
17335818	6,604605	6,740431	6,361382	6,427433	6,506313	6,416896	6,568806	6,450214	-0,118592	0,921086147	Slc37a1
17451251	5,632761	5,518511	5,817627	5,48038	5,58268	5,550225	5,6562997	5,537761667	-0,118538	0,921120624	Myo18b
17314777	6,942846	6,720347	7,0617	6,783722	6,776046	6,759531	6,9082977	6,789766333	-0,11853133	0,921124881	Prpf40b
17319109	6,807185	6,896627	7,038047	6,824355	6,928443	6,6335	6,913953	6,795432667	-0,11852033	0,921131904	Ankrd54
17448212	7,336714	7,107303	7,759383	7,231081	7,443049	7,173744	7,4011333	7,282624667	-0,11850867	0,921139353	3110047P20Rik // 3110047P20Rik
17510080	6,516499	6,617729	6,626782	6,46143	6,430717	6,513348	6,5870033	6,468498333	-0,118505	0,921141694	2810422J05Rik
17442032	7,628664	7,752237	7,792673	7,696292	7,526344	7,595486	7,7245247	7,606040667	-0,118484	0,921155102	Ppp1cc
17274633	2,826171	3,07469	3,015646	2,783878	2,907786	2,869402	2,972169	2,853688667	-0,11848033	0,921157443	---
17201427	5,034486	5,184799	5,886561	5,189268	5,700722	4,860466	5,3686153	5,250152	-0,11846333	0,921168298	---
17417422	7,666145	7,33633	7,244697	7,310205	7,420259	7,161382	7,415724	7,297282	-0,118442	0,921181919	Ipp
17331794	3,37868	3,483595	3,531487	3,335854	3,351297	3,351297	3,4645873	3,346149333	-0,118438	0,921184474	---
17284253	5,601674	5,587008	5,860643	5,689396	5,524519	5,480121	5,6831083	5,564678667	-0,11842967	0,921189795	Jag2
17221676	4,586132	4,776566	4,696836	4,517093	4,716809	4,470358	4,6865113	4,568086667	-0,11842467	0,921192987	Gm4956
17448595	4,171669	3,926543	4,224429	4,008889	4,020719	3,937797	4,107547	3,989135	-0,118412	0,921201075	Gabra4
17367949	6,420245	6,575168	6,688687	6,455312	6,628162	6,492009	6,5613667	6,442981	-0,11838567	0,92121789	2010317E24Rik
17360877	9,272688	9,219791	9,071696	9,053663	8,963615	9,19178	9,1880583	9,069686	-0,11837233	0,921226404	Fam45a
17229463	3,888784	3,936117	4,001668	3,965119	3,765903	3,740442	3,9421897	3,823821333	-0,11836833	0,921228958	1700084C01Rik
17293774	3,786912	3,57736	3,491901	3,511315	3,549009	3,440747	3,6187243	3,500357	-0,11836733	0,921229596	Nlrp4f
17450727	8,321542	8,582783	8,339252	8,38702	8,169241	8,332262	8,4145257	8,296174333	-0,11835133	0,921239813	Tmed5
17243523	4,566882	4,60348	5,083665	4,53046	4,739945	4,628603	4,7513423	4,633002667	-0,11833967	0,921247263	---
17464934	7,831536	7,712941	7,672393	7,524071	7,664452	7,673361	7,7389567	7,620628	-0,11832867	0,921254287	2610001J05Rik
17339585	8,216084	8,349748	8,155218	8,023059	8,175438	8,167584	8,24035	8,122027	-0,118323	0,921257906	Spast
17210047	3,724528	3,635158	3,333062	3,268775	3,671659	3,397359	3,5642493	3,445931	-0,11831833	0,921260886	---
17250089	5,527497	5,228666	5,440555	5,199987	5,195037	5,446784	5,398906	5,280602667	-0,11830333	0,921270464	Olfr179
17203757	5,507063	5,681515	5,197452	5,336178	5,602354	5,092609	5,46201	5,343713667	-0,11829633	0,921274934	---
17458037	4,253741	4,018753	4,388078	4,021952	4,133118	4,150644	4,2201907	4,101904667	-0,118286	0,921281533	9430018G01Rik
17356633	5,932214	6,318857	5,911892	5,945173	6,031782	5,831185	6,054321	5,936046667	-0,11827433	0,921288983	Gm550
17430791	10,66536	10,30699	10,50264	10,44559	10,39524	10,27934	10,491663	10,37339	-0,11827333	0,921289622	Rnu11
17352695	3,028353	3,107414	2,924446	2,8327	3,099817	2,772989	3,020071	2,901835333	-0,11823567	0,921313676	Mir1b // Mir1b
17200437	6,766691	6,639484	6,445217	6,582448	6,71313	6,201145	6,6171307	6,498907667	-0,118223	0,921321765	---
17377464	7,405081	7,317532	7,451382	7,182575	7,573676	7,063096	7,3913317	7,273115667	-0,118216	0,921326235	Cst7
17514349	12,76392	12,74525	12,36626	12,6899	12,43218	12,39871	12,625143	12,50693	-0,11821333	0,921327938	---
17440130	9,019604	9,292628	9,053662	8,935744	9,031589	9,043936	9,1219647	9,003756333	-0,11820833	0,921331131	Rpl5 // Gm14217

17509388	4,170874	4,29636	4,243239	4,21474	4,067086	4,074055	4,2368243	4,118627	-0,11819733	0,921338156	Wdr17
17501104	7,76312	7,816082	7,654747	7,577908	7,633944	7,667541	7,7446497	7,626464333	-0,11818533	0,921345819	Dctd
17369832	6,624599	6,771665	6,745775	6,418556	6,689511	6,679462	6,714013	6,595843	-0,11817	0,921355612	Naif1
17400510	4,406421	4,675322	4,640025	4,355518	4,497354	4,514413	4,5739227	4,455761667	-0,118161	0,92136136	Sf3b4
17322991	4,409557	4,356653	4,575467	4,393215	4,418024	4,176032	4,4472257	4,329090333	-0,11813533	0,921377751	1700003L19Rik
17274868	8,30448	8,588584	8,316703	8,290051	8,328772	8,23657	8,4032557	8,285131	-0,11812467	0,921384564	2010109K11Rik
17246432	3,663897	3,52538	3,393883	3,373093	3,520818	3,33494	3,52772	3,409617	-0,118103	0,921398401	Olfr807
17291970	3,66819	4,275924	4,092171	3,949904	4,019089	3,712989	4,012095	3,893994	-0,118101	0,921399679	---
17510494	8,562853	8,867033	8,527745	8,499436	8,546765	8,557146	8,6525437	8,534449	-0,11809467	0,921403724	Hmgn2
17518090	6,096152	5,518032	6,144946	5,853773	6,004421	5,546658	5,91971	5,801617333	-0,11809267	0,921405001	---
17219299	7,747409	7,803924	7,779577	7,681977	7,664217	7,630466	7,77697	7,658886667	-0,11808333	0,921410962	Pfdn2 // Pfdn2
17412102	7,189683	7,276584	6,893555	6,900184	7,022154	7,083262	7,1199407	7,001866667	-0,118074	0,921416923	Ggh
17245053	6,996484	7,099499	6,909573	6,885013	6,893061	6,873279	7,001852	6,883784333	-0,11806767	0,921420968	Thap2
17371436	6,202891	6,122094	5,953792	6,053128	5,893218	5,978261	6,0929257	5,974869	-0,11805667	0,921427993	Bbs5
17437529	8,315151	8,37048	8,347795	8,111277	8,157469	8,410612	8,3444753	8,226452667	-0,11802267	0,921449709	Stim2
17335344	4,453062	4,529834	4,651548	4,493796	4,399089	4,38755	4,5448147	4,426811667	-0,118003	0,92146227	4930511I11Rik
17221792	8,200637	8,165969	8,124394	8,113751	7,985673	8,037576	8,1636667	8,045666667	-0,118	0,921464186	Fam135a
17233665	3,946509	3,840742	4,066239	3,917018	3,687726	3,894789	3,9511633	3,833177667	-0,11798567	0,921473341	4930565A17
17482914	3,947328	4,0276	4,176889	3,908682	3,892237	3,996986	4,0506057	3,932635	-0,11797067	0,921482922	4930551E15Rik
17457737	3,800746	3,891515	4,08168	3,841116	3,576752	4,002199	3,924647	3,806689	-0,117958	0,921491012	Try10
17265708	7,712574	7,707633	7,385223	7,559127	7,368058	7,52445	7,60181	7,483878333	-0,11793167	0,921507832	---
17510099	7,541413	7,734446	7,833479	7,596021	7,634991	7,524726	7,7031127	7,585246	-0,11786667	0,921549352	Ssbp4
17548264	11,48595	11,37659	11,02979	11,12397	11,06319	11,35163	11,297443	11,17959667	-0,11784667	0,921562127	Wbp5 // Wbp5
17344811	6,242054	5,897773	5,757752	5,736905	6,071475	5,73569	5,9658597	5,848023333	-0,11783633	0,921568728	H2-M5
17265548	5,301785	4,923194	5,175184	5,062675	4,927714	5,056354	5,1333877	5,015581	-0,11780667	0,921587678	---
17364890	6,232419	6,227031	6,151003	6,016676	6,290121	5,950262	6,2034843	6,085686333	-0,117798	0,921593215	Hps1
17292769	4,620231	4,754225	4,646316	4,539031	4,503942	4,624429	4,6735907	4,555800667	-0,11779	0,921598325	---
17380840	8,496482	8,68027	8,671148	8,456558	8,540215	8,497822	8,6159667	8,498198333	-0,11776833	0,921612166	2310003C23Rik
17349774	7,319544	7,447266	7,276052	7,308303	7,199886	7,181494	7,3476207	7,229894333	-0,11772633	0,921638996	Hars2
17509355	3,913476	4,062772	4,008497	3,848985	3,998431	3,784176	3,994915	3,877197333	-0,11771767	0,921644533	---
17236754	8,582571	8,546115	8,635595	8,513575	8,459583	8,437982	8,5880937	8,47038	-0,11771367	0,921647088	Eea1
17206957	4,446063	4,260101	3,958234	4,128503	4,112148	4,070745	4,221466	4,103798667	-0,11766733	0,921676688	---
17268404	10,57613	10,65821	10,78674	10,4085	10,6483	10,61128	10,673693	10,55602667	-0,11766667	0,921677114	Pnpo
17532637	8,303452	8,763504	8,219674	8,314487	8,313623	8,30563	8,4288767	8,311246667	-0,11763	0,921700539	---
17549170	9,50239	9,588381	10,11098	9,373438	9,767456	9,708002	9,733917	9,616298667	-0,11761833	0,921707993	---
17469556	8,921166	8,917484	8,969077	8,734809	8,821205	8,89887	8,935909	8,818294667	-0,11761433	0,921710548	Crbn
17345335	8,894993	9,118329	8,887215	8,778198	9,098738	8,670762	8,9668457	8,849232667	-0,117613	0,9217114	Polr1c
17474130	5,708336	5,183769	5,40241	5,393632	5,413828	5,134233	5,431505	5,313897667	-0,11760733	0,921715021	Ceacam9
17203407	4,930298	5,005618	4,813539	5,184001	4,136513	5,076153	4,916485	4,798889	-0,117596	0,921722261	---
17377744	5,931152	6,081392	6,176399	5,981742	5,958167	5,896251	6,062981	5,945386667	-0,11759433	0,921723326	Rem1
17272461	7,042723	7,072835	6,946253	6,968446	6,945981	6,794611	7,0206037	6,903012667	-0,117591	0,921725456	Cygb
17294805	8,020249	8,088246	8,041574	7,914413	7,990555	7,892328	8,050023	7,932432	-0,117591	0,921725456	Zcchc9
17235300	6,227159	5,946908	6,359228	6,062671	6,120635	5,997233	6,177765	6,060179667	-0,11758533	0,921729076	Apc2
17322731	7,745187	7,429624	7,952061	7,442942	7,888089	7,443137	7,7089573	7,591389333	-0,117568	0,92174015	---
17351668	10,37835	10,30354	10,27742	10,0992	10,19541	10,31205	10,31977	10,20222	-0,11755	0,921751651	Mbd2
17351143	6,868355	6,938718	6,966808	6,763951	6,874938	6,782462	6,924627	6,807117	-0,11751	0,921777207	Sh3tc2
17491276	8,529718	8,689544	8,576544	8,418582	8,518519	8,506263	8,598602	8,481121333	-0,11748067	0,921795949	Spty2d1
17517828	5,059482	4,777812	4,835153	4,610855	5,022482	4,686669	4,8908157	4,773335333	-0,11748033	0,921796162	Cyp11a1
17506479	7,104098	7,16981	7,293305	7,068397	6,979931	7,166452	7,189071	7,071593333	-0,11747767	0,921797866	Spg7
17461132	6,193192	5,921344	6,126958	5,902694	5,974602	6,011775	6,080498	5,963023667	-0,11747433	0,921799996	Mitf
17459236	9,078056	9,188054	9,087916	9,013756	8,961453	9,026452	9,1180087	9,000553667	-0,117455	0,921812349	Mad2l1
17272814	4,646025	4,530747	4,790169	4,675192	4,359483	4,579968	4,655647	4,538214333	-0,11743267	0,921826619	---
17437959	4,344418	4,332121	4,478174	4,21308	4,174257	4,415241	4,3849043	4,267526	-0,11737833	0,921861337	Bend4
17507247	4,568861	4,379835	4,669597	4,483521	4,375178	4,407462	4,539431	4,422053667	-0,11737733	0,921861976	BC068157
17382638	5,84488	5,654585	6,062828	5,909934	5,679438	5,620827	5,8540977	5,736733	-0,11736467	0,92187007	Mamdc4
17326930	3,748585	4,058897	3,648253	3,654889	3,802932	3,645838	3,8185783	3,701219667	-0,11735867	0,921873903	Krtap6-1
17203749	6,097798	5,749907	5,862584	6,13166	5,188214	6,038367	5,9034297	5,786080333	-0,11734933	0,921879867	---
17261122	7,339428	7,821627	7,54879	7,467911	7,350922	7,538998	7,5699483	7,452610333	-0,117338	0,921887109	Papolg
17416763	8,627016	8,891109	8,744103	8,461979	8,660797	8,787472	8,754076	8,636749333	-0,11732667	0,921894352	---
17214184	4,614289	4,716986	5,215159	4,544622	4,869308	4,780556	4,8488113	4,731495333	-0,117316	0,921901168	Gm216
17338934	7,251857	7,322245	7,10249	6,861511	7,329198	7,133964	7,2255307	7,108224333	-0,11730633	0,921907345	Alkbh7
17361552	9,058483	9,109014	9,105034	9,048576	8,815964	9,056211	9,0908437	8,973583667	-0,11726	0,921936953	Banf1
17400810	3,695484	3,687544	3,661745	3,656603	3,722775	3,31365	3,681591	3,564342667	-0,11724833	0,921944409	Gm5544
17419873	4,761298	4,616255	4,702295	4,579873	4,544101	4,604145	4,6932827	4,576039667	-0,117243	0,921947817	---
17464480	5,978672	6,198032	6,058972	5,839297	5,863085	6,181571	6,0785587	5,961317667	-0,117241	0,921949095	3010003L21Rik
17422488	5,835579	5,992904	5,867979	5,678622	5,774227	5,891898	5,8988207	5,781582333	-0,11723833	0,921950799	Morn1
17498084	6,512434	6,139833	6,514554	6,204626	6,315911	6,294576	6,3889403	6,271704333	-0,117236	0,92195229	Krtap5-1
17474396	6,824212	6,930039	6,744255	6,61675	6,694125	6,83602	6,8328353	6,715631667	-0,11720367	0,921972953	Irf2bp1
17415627	3,839075	3,7301	3,889247	3,636035	3,694432	3,776384	3,819474	3,702283667	-0,11719033	0,921981474	---
17227828	7,993628	7,754621	7,608469	7,474765	7,823167	7,70722	7,7855727	7,668384	-0,11718867	0,921982539	Pla2g4a
17271033	9,164205	9,165971	9,149028	8,928401	9,073529	9,125759	9,1597347	9,042563	-0,11717167	0,921993403	Kpna2
17207117	4,590566	4,771328	5,393064	5,633131	4,377468	4,392888	4,9183193	4,801162333	-0,117157	0,922002776	---
17305950	5,389296	5,281959	5,03245	5,03245	5,403553	4,916425	5,2345683	5,117476	-0,11709233	0,922044105	Olfr733
17283380	7,415533	7,680639	7,087095	7,193854	7,264589	7,37363	7,3944223	7,277357667	-0,11706467	0,922061787	Fbln5
17526988	5,910651	6,05702	6,173803	5,874326	5,861351	6,054614	6,047158	5,930097	-0,117061	0,92206413	1810046K07Rik
17216888	4,784891	4,657341	4,907224	4,773959	4,478052	4,746264	4,783152	4,666091667	-0,11706033	0,922064556	Thsd7b
17437380	6,349954	6,764634	6,555418	6,500946	6,13997	6,677944	6,5566687	6,43962	-0,11704867	0,922072013	9230114K14Rik
17222940	7,288682	7,429927	7,286757	7,373005	7,145364	7,135892	7,335122	7,218087	-0,117035	0,922080748	Inpp1
17532455	7,433758	7,70827	7,132448	6,97472	7,564813	7,383848	7,4248253	7,307793667	-0,11703167	0,922082878	1110059G10Rik
17344720	4,333123	4,570582	4,732137	4,355577	4,622069	4,307159	4,5452807	4,428268333	-0,11701233	0,922095235	H2-M10.2
17542254	5,072651	5,249485	5,032298	4,744349	5,168315	5,090906	5,1181447	5,00119	-0,11695467	0,922132093	Xlr4a
17289675	4,378399	4,344243	4,275968	4,466338	4,137316	4,044163	4,33287	4,215939	-0,116931	0,922147221	3830408C21Rik
17289497	8,998392	9,041508	9,000572	8,908632	8,935195	8,845858	9,0134907	8,896561667	-0,116929	0,922148499	Ccdc125
17214387	5,997507	5,809873	5,998318	5,703331	5,802997	5,948637	5,9352327	5,818321667	-0,116911	0,922160004	Wnt10a
17272262	5,899235	6,037158	6,185877	6,019837	5,939183	5,81258	6,0407567	5,923866667	-0,11689	0,922173427	Evpl
17528924	5,239793	5,073924	4,68354	4,710371	5,1934	4,74289	4,9990857	4,882220333	-0,11686533	0,922189195	Fam83b
17244559	4,400168	4,165556	4,157442	4,074516	4,149048	4,149048	4,2410553	4,124204	-0,11685133	0,922198144	Gm18409
17293748	4,925371	5,043337	5,045774	4,83015	5,036962	4,796855	5,0048273	4,887989	-0,11683833	0,922206453	Prss47
17535712	5,601639	5,453522	5,617795	5,55703	5,285695	5,479741	5,557652	5,440822	-0,11683	0,92221178	Avpr2
17362216	7,033277	7,142173	7,281599	7,061709	6,997348	7,047518	7,1523497	7,035525	-0,11682467	0,92221519	Nudt22
17548044	3,548088	3,696447	3,712435	3,590672	3,438847	3,577013	3,6523233	3,535510667	-0,11681267	0,92222286	Syt4 // Syt4
17215720	8,818165	8,888793	8,860982	8,795528	8,595564	8,826427	8,85598	8,739173	-0,116807	0,922226483	Scly
17258961	6,085021	6,139573	6,217085	6,097565	6,057829	5,935873	6,1472263	6,030422333	-0,116804	0,9222284	---
17262733	3,260714	3,13005	3,292532	3,120409	3,082508	3,130106	3,2277653	3,111007667	-0,11675767	0,922258019	---
17494214	3,694461	3,801767	3,19128	3,824565	3,189692	3,322986	3,5625027	3,445747667	-0,116755	0,922259724	Olfr68
17415535	4,807432	4,667085	5,07341	4,712602	4,844557	4,640543	4,849309	4,732567333	-0,11674167	0,922268247	Gm3831
17457060	4,833587	4,325356	4,569067	4,301311	4,387772	4,688741	4,5760033	4,459274667	-0,11672867	0,922276558	---
17546244	4,057646	3,406764	3,028877	3,687335	3,19457	3,261307	3,4977623	3,381070667	-0,11669167	0,922300211	---
17244608	5,019904	5,297721	5,198775	5,05835	5,142508	4,965497	5,1721333	5,055451667	-0,11668167	0,922306604	Alx1
17200391	5,619278	5,534659	5,61607	5,000154	5,162435	6,257399	5,5900023	5,473329333	-0,116673	0,922312145	---
17254970	5,552024	4,724342	5,299555	4,863597	5,339636	5,022686	5,1919737	5,075306333	-0,11666733	0,922315767	1700106J16Rik
17270109	8,26063	8,264545	8,412309	8,499269	7,949467	8,13881	8,3124947	8,195848667	-0,116646	0,922329406	---
17338906	3,9336	4,144052	3,816323	3,852702	3,787604	3,903733	3,9646583	3,848013	-0,11664533	0,922329832	1700061G19Rik
17383633	9,203917	9,142953	9,198557	8,962982	9,045507	9,187011	9,181809	9,065166667	-0,11664233	0,92233175	Crat
17298946	4,691819	4,801013	4,759166	4,665912	4,685657	4,550541	4,750666	4,634036667	-0,11662933	0,922340061	Dydc1
17388683	7,499243	7,063276	6,913504	7,054732	7,106731	6,964703	7,1586743	7,042055333	-0,116619	0,922346667	Rag1
17549598	4,290634	4,265559	4,667132	4,129991	4,434169	4,309333	4,407775	4,291164333	-0,11661067	0,922351995	---
17240527	8,653788	8,529935	8,562987	8,439251	8,471086	8,486588	8,5822367	8,465641667	-0,116595	0,922362011	Gm9855
17349868	3,393408	3,52581	3,555829	3,343925	3,331877	3,449608	3,4916823	3,375136667	-0,11654567	0,922393552	Pcdhb2
17387646	3,789963	3,511984	3,797155	3,706326	3,281718	3,761471	3,6997007	3,583171667	-0,116529	0,922404208	Olfr52
17324570	3,963926	4,090237	4,15778	4,016102	3,915093	3,931173	4,0706477	3,954122667	-0,116525	0,922406766	Gm10823
17543572	7,490863	7,434927	7,174656	7,262019	7,231145	7,257762	7,3668153	7,250308667	-0,11650667	0,922418487	Il2rg
17270303	4,041414	4,610044	4,295534	4,065947	4,430813	4,100741	4,315664	4,199167	-0,116497	0,922424668	---
17542583	4,04418	4,019424	4,12673	4,009402	3,887327	3,944169	4,0634447	3,946966	-0,11647867	0,92243639	Tex28
17232176	3,650229	3,934494	3,626395	3,651848	3,732794	3,477136	3,7370393	3,620592667	-0,11644667	0,922456851	Taar2
17491074	5,386023	5,363906	5,420951	5,338977	5,164009	5,318589	5,3902933	5,273858333	-0,116435	0,92246431	Abcc8
17269486	5,999881	6,007365	6,018258	5,834742	5,927401	5,914069	6,0085013	5,892070667	-0,11643067	0,922467081	Leprel4
17220907	8,815922	8,841227	8,905066	8,93405	8,684786	8,594105	8,8540717	8,737647	-0,11642467	0,922470917	Irf6
17446832	7,09001	6,978688	7,147796	6,964332	6,905468	6,997447	7,0721647	6,955749	-0,11641567	0,922476672	Ucn
17466075	9,570697	9,726274	9,574882	9,39693	9,577185	9,548611	9,623951	9,507575333	-0,11637567	0,922502249	Jhdm1d
17212900	4,046	4,09923	4,153011	3,945076	3,834815	4,16933	4,0994137	3,983073667	-0,11634	0,922525056	Rftn2
17330751	4,255565	4,112525	4,508533	4,41902	3,955553	4,15305	4,2922077	4,175874333	-0,11633333	0,922529318	Cd96
17350563	8,255273	8,557657	8,185943	8,256954	8,157835	8,235191	8,3329577	8,21666	-0,11629767	0,922552126	Srfbp1
17275303	5,73143	6,102077	5,953449	5,888071	5,377882	6,172113	5,9289853	5,812688667	-0,11629667	0,922552765	Tfb2m // Tfb2m
17295770	9,377801	9,447406	9,273922	9,168609	9,289074	9,292628	9,3663763	9,250103667	-0,11627267	0,922568113	Slc30a5
17479012	6,936605	6,957221	6,578535	6,520794	6,984601	6,618164	6,8241203	6,707853	-0,11626733	0,922571523	Lins // Lins
17463316	8,026547	8,182061	8,08365	8,000197	8,021436	7,921848	8,0974193	7,981160333	-0,116259	0,922576852	D6Wsu163e
17307575	4,398971	4,228857	4,455984	4,050465	4,265981	4,418597	4,3612707	4,245014333	-0,11625633	0,922578557	Defb48
17444027	6,382944	6,040114	6,369639	6,024475	6,304555	6,114909	6,2642323	6,147979667	-0,11625267	0,922580902	Uncx
17293020	9,733208	9,801758	9,690865	9,79519	9,408821	9,673075	9,7419437	9,625695333	-0,11624833	0,922583673	Lect2
17330156	4,522507	5,105825	4,193549	4,538706	4,479922	4,454538	4,6072937	4,491055333	-0,11623833	0,922590068	---
17415774	6,514404	6,627147	6,836184	6,599932	6,663895	6,365204	6,659245	6,543010333	-0,11623467	0,922592413	Foxd3
17501989	5,89639	6,129962	6,270339	5,842561	6,271436	5,834006	6,098897	5,982667667	-0,11622933	0,922595824	Lrrc25
17347265	4,517488	4,516644	4,897054	4,12803	4,985729	4,468794	4,6437287	4,527517667	-0,116211	0,922607548	---

17459656	6,002097	5,997586	6,114758	5,960419	6,026959	5,778486	6,038147	5,921954667	-0,11619233	0,922619485	Capg
17327889	9,131156	9,023525	9,021692	9,029852	8,934031	8,863916	9,058791	8,942599667	-0,11619133	0,922620125	Glyr1
17316954	4,035954	3,946265	3,858016	3,671899	3,936342	3,883427	3,946745	3,830556	-0,116189	0,922621617	---
17398180	10,16996	10,09859	10,02813	9,979188	10,00479	9,964158	10,098893	9,982712	-0,11618133	0,92262652	Mfsd1
17371708	6,435803	6,705883	6,597646	6,575233	6,152193	6,663365	6,5797773	6,463597	-0,11618033	0,922627159	Cybrd1
17495985	5,454571	5,933098	5,831526	5,573842	5,500977	5,795893	5,7397317	5,623570667	-0,116161	0,922639523	Gm20299
17216886	6,221781	5,614658	5,926469	6,117777	5,635262	5,6614	5,9209693	5,804813	-0,11615633	0,922642508	---
17337663	3,381855	3,020199	2,864691	2,678652	3,397705	2,841943	3,088915	2,972766667	-0,11614833	0,922647624	Gm4345
17423697	2,996265	2,818261	2,998022	2,89093	2,772034	2,801163	2,937516	2,821375667	-0,11614033	0,92265274	---
17362934	4,642753	3,889592	4,360716	4,136893	4,30581	4,101966	4,297687	4,181556333	-0,11613067	0,922658923	Ms4a5
17326500	2,933409	2,925401	2,830472	2,854566	2,712928	2,773398	2,8964273	2,780297333	-0,11613	0,922659349	---
17413369	7,940747	7,83587	7,849788	7,705188	7,879742	7,693153	7,8754683	7,759361	-0,11610733	0,922673845	Creb3
17276056	10,6717	10,62763	10,54298	10,41375	10,51947	10,56088	10,614103	10,49803333	-0,11607	0,922697722	Psma3
17402778	4,881758	4,691596	4,551377	4,654213	4,776426	4,345883	4,7082437	4,592174	-0,11606967	0,922697935	Cxxc4
17344911	4,11588	3,856198	3,898359	3,769887	3,903263	3,849156	3,9568123	3,840768667	-0,11604367	0,922714564	Olfr129
17287160	5,585516	6,810784	5,863381	5,565804	6,168905	6,17687	6,0865603	5,970526333	-0,116034	0,922720747	Aspn
17237492	5,441264	5,373068	5,618489	5,411918	5,426392	5,246446	5,477607	5,361585333	-0,11602167	0,922728635	1700058G18Rik
17213319	9,620279	9,961844	10,03345	9,807339	9,8288	9,631386	9,8718577	9,755841667	-0,116016	0,922732259	Snord11
17204237	3,602498	4,510143	3,558265	4,132946	4,000248	3,189692	3,890302	3,774295333	-0,11600667	0,922738229	---
17442521	6,681687	6,72525	6,698528	6,486735	6,584728	6,68601	6,7018217	6,585824333	-0,11599733	0,922744198	Snrnp35
17412272	8,653734	8,631155	8,508113	8,322315	8,515271	8,607494	8,5976673	8,481693333	-0,115974	0,922759122	Ndufaf4
17245581	5,508241	5,245821	5,462638	5,403666	5,296378	5,168743	5,4055667	5,289595667	-0,115971	0,922761041	A130077B15Rik // A130077B15Rik
17543491	5,993607	6,070809	6,260695	5,999114	5,923948	6,054136	6,1083703	5,992399333	-0,115971	0,922761041	Gm14866
17341387	3,395025	3,551396	3,40308	3,183861	3,539676	3,278065	3,4498337	3,333867333	-0,11596633	0,922764026	---
17516151	3,592504	3,633586	3,546924	3,344363	3,466911	3,613905	3,5910047	3,475059667	-0,115945	0,922777671	Olfr25
17385398	3,765928	3,660595	3,404303	3,583253	3,366483	3,533262	3,6102753	3,494332667	-0,11594267	0,922779164	Ermn
17357741	8,590109	8,555104	8,49499	8,325921	8,518563	8,447931	8,5467343	8,430805	-0,11592933	0,922787692	Mrpl16
17328046	4,7897	4,652658	4,874432	4,489424	4,653112	4,826478	4,7722633	4,656338	-0,11592533	0,92279025	Grin2a
17207103	5,720657	5,926032	5,374985	5,988152	5,559102	5,126695	5,6738913	5,557983	-0,11590833	0,922801124	---
17467453	3,66507	4,130496	3,047374	3,711897	3,1649	3,618506	3,6143133	3,498434333	-0,115879	0,922819887	---
17274114	5,172572	4,777894	4,749699	4,868526	4,900972	4,583056	4,900055	4,784184667	-0,11587033	0,922825431	---
17206139	2,949382	2,973156	2,832213	2,802382	2,802382	2,802382	2,9182503	2,802382	-0,11586833	0,92282671	---
17362524	6,32132	6,857138	6,781881	6,454758	6,555575	6,602416	6,6534463	6,537583	-0,11586333	0,922829908	LOC100861648
17428857	7,947521	8,607411	8,264545	7,942642	8,604325	7,924942	8,273159	8,157303	-0,115856	0,922834599	---
17206037	6,019795	6,278339	6,189975	6,509729	6,016366	5,614447	6,162703	6,046847333	-0,11585567	0,922834812	---
17384979	4,769255	4,549231	4,728456	4,576487	4,523985	4,598973	4,682314	4,566481667	-0,11583233	0,922849738	---
17435282	6,335153	6,687753	6,490846	6,503117	6,406143	6,257067	6,504584	6,388775667	-0,11580833	0,92286509	Nos3
17293845	2,980679	2,990008	3,000746	2,920905	2,873883	2,82925	2,9904777	2,874679333	-0,11579833	0,922871487	---
17489157	11,0814	10,9294	10,84747	10,95608	10,79067	10,76419	10,952757	10,83698	-0,11577667	0,922885347	---
17322230	6,184241	6,104503	5,98776	6,248231	5,921032	5,759919	6,092168	5,976394	-0,115774	0,922887053	Map3k12
17252157	5,627141	5,595809	5,702675	5,578457	5,573433	5,426455	5,641875	5,526115	-0,11576	0,922896009	4930544D05Rik
17238111	4,563185	4,500873	4,620124	4,200128	4,553416	4,583367	4,561394	4,445637	-0,115757	0,922897928	Myo1a
17549926	4,121127	4,43524	4,386537	4,076596	4,204123	4,314965	4,3143013	4,198561333	-0,11574	0,922908803	---
17284060	5,967106	5,912361	6,022321	5,866258	5,888008	5,800329	5,9672627	5,851531667	-0,115731	0,92291456	4930595D18Rik // 4930595D18Rik
17206021	5,361974	5,280665	5,452995	5,52936	5,446523	4,772594	5,3652113	5,249492333	-0,115719	0,922922237	---
17453776	6,164363	5,915122	6,037517	5,72927	6,006506	6,034075	6,0390007	5,923283667	-0,115717	0,922923516	Emid2
17258964	5,730176	5,903109	5,708108	5,630645	5,72357	5,640126	5,7804643	5,664780333	-0,115684	0,922944627	Ccdc40
17237751	6,089949	6,105061	6,034287	5,939216	5,938834	6,004195	6,0764323	5,960748333	-0,115684	0,922944627	BC048403
17251503	5,285476	5,230752	5,144516	5,193807	5,078279	5,041655	5,220248	5,104580333	-0,11566767	0,922955076	Tmem107
17534730	3,889531	3,760615	3,82139	3,685606	3,688707	3,750247	3,8238453	3,708186667	-0,11565867	0,922960834	Fam122c
17251866	7,628047	7,609605	7,567432	7,501156	7,467065	7,490012	7,6016947	7,486077667	-0,115617	0,922987491	Phf23
17298998	5,837055	5,544763	5,28957	5,542357	5,328502	5,453715	5,5571293	5,441524667	-0,11560467	0,922995381	---
17550448	3,203795	3,210126	3,474078	3,318211	3,15574	3,067247	3,2959997	3,180399333	-0,11560033	0,922998153	---
17338821	10,61592	10,71587	10,77014	10,58414	10,67342	10,4976	10,700643	10,58505333	-0,11559	0,923004764	Rpl36
17207753	6,741095	6,848236	6,873044	7,025127	6,822962	6,267594	6,8207917	6,705227667	-0,115564	0,923021399	---
17279737	10,4323	10,57207	10,34573	10,27782	10,22764	10,49795	10,450033	10,33447	-0,11556333	0,923021825	Rab10
17499072	4,187874	4,368493	4,657083	4,256686	4,081493	4,528608	4,4044833	4,288929	-0,11555433	0,923027583	1700018L24Rik
17242449	7,852828	7,828384	7,826801	7,629742	7,802757	7,728868	7,8360043	7,720455667	-0,11554867	0,923031209	Trappc10
17366166	2,956893	3,412266	3,07854	2,91796	3,048631	3,13467	3,149233	3,033753667	-0,11547933	0,923075569	---
17388681	4,210297	3,73266	3,819884	3,823017	3,554976	4,038433	3,920947	3,805475333	-0,11547167	0,923080475	Rag2 // Rag2
17314907	4,884851	4,384803	4,786618	4,538979	4,703554	4,467358	4,685424	4,569963667	-0,11546033	0,923087726	---
17501994	8,570155	8,678322	8,676409	8,485012	8,489654	8,60388	8,6416287	8,526182	-0,11544667	0,923096471	Lsm4
17447940	7,428785	7,42294	7,417295	7,337909	7,205534	7,37938	7,4230067	7,307607667	-0,115399	0,92312697	Lcorl
17432718	3,315696	3,5666	3,11274	3,373591	3,198335	3,076921	3,3316787	3,216282333	-0,11539633	0,923128676	---
17460333	3,50825	3,881918	3,392533	3,469149	3,573821	3,393571	3,5942337	3,478847	-0,11538667	0,923134862	Alms1-ps2
17538816	9,001	8,947945	9,344714	8,819716	8,959099	9,168727	9,0978863	8,982514	-0,11537233	0,923144033	---
17238236	8,309395	8,235033	8,179222	8,09639	8,139946	8,141203	8,2412167	8,125846333	-0,11537033	0,923145313	Ptges3
17549906	12,62019	12,6897	12,71147	12,41493	12,55331	12,70702	12,673787	12,55842	-0,11536667	0,923147659	---
17308481	11,88495	11,93035	11,78463	11,52369	11,81022	11,92006	11,866643	11,75132333	-0,11532	0,923177521	---
17469965	6,88869	6,442441	6,833318	7,02041	6,199977	6,598183	6,721483	6,60619	-0,115293	0,923194798	---
17256323	4,432049	4,220225	4,254623	4,272683	4,120162	4,168217	4,302299	4,187020667	-0,11527833	0,923204183	Krtap31-1
17218341	3,886914	3,66849	3,807909	3,699779	3,765121	3,552598	3,787771	3,672499333	-0,11527167	0,92320845	Gm5531
17313016	9,188604	9,304588	9,163244	9,030704	9,099009	9,180944	9,218812	9,103552333	-0,11525967	0,923216129	Tomm22
17492985	5,452452	5,33939	5,688878	5,701589	5,353826	5,079535	5,4935733	5,378316667	-0,11525667	0,923218048	Arnt2
17232937	6,96564	6,843228	7,193766	6,997499	6,728908	6,930496	7,000878	6,885634333	-0,11524367	0,923226368	Ostm1
17449685	8,586408	8,72419	8,508183	8,399808	8,654081	8,419164	8,6062603	8,491017667	-0,11524267	0,923227007	Sdad1
17243117	8,188098	8,267776	8,146504	8,096391	8,168594	7,991714	8,2007927	8,085566333	-0,11522633	0,92323746	Lsm7
17534637	6,967955	6,933321	6,833388	6,662351	6,934931	6,791728	6,9115547	6,796336667	-0,115218	0,923242793	Rbmx2
17536245	3,854676	3,732566	3,948751	3,719638	3,804237	3,666468	3,845331	3,730114333	-0,11521667	0,923243646	---
17350824	7,212924	7,209245	6,89303	6,870259	7,051225	7,048075	7,1050663	6,989853	-0,11521333	0,923245779	Slc12a2
17527369	3,999821	3,842825	4,083929	3,86262	3,869746	3,848657	3,975525	3,860341	-0,115184	0,923264551	---
17398176	6,422367	6,395049	6,406005	6,378712	6,387716	6,111512	6,407807	6,292646667	-0,11516033	0,923279697	LOC100861992
17314904	3,29388	3,286764	3,365781	3,235925	3,277049	3,088006	3,315475	3,200326667	-0,11514833	0,923287376	---
17523990	7,549381	7,621519	7,37575	7,365755	7,56449	7,27097	7,51555	7,400405	-0,115145	0,92328951	Jrkl
17231459	4,502909	4,113801	4,946889	4,05831	4,690734	4,469136	4,5211997	4,40606	-0,11513967	0,923292923	---
17247896	6,912269	6,812912	6,862722	6,816195	6,712254	6,714059	6,8626343	6,747502667	-0,11513167	0,923298043	---
17292828	6,829037	6,785511	7,134649	6,67414	6,847047	6,882619	6,916399	6,801268667	-0,11513033	0,923298896	Mxd3
17365686	5,030792	5,128376	5,116988	4,965648	4,983661	4,981493	5,092052	4,976934	-0,115118	0,923306789	1700001K23Rik
17463520	9,237513	9,173489	9,145741	8,9665	9,137688	9,107227	9,185581	9,070471667	-0,11510933	0,923312336	Clec1b
17419636	6,603953	6,82385	6,81613	6,666039	6,605497	6,627134	6,7479777	6,63289	-0,11508767	0,923326202	Gpn2
17483770	3,218089	2,943563	3,160336	2,967922	2,946719	3,062199	3,1073293	2,99228	-0,11504933	0,923350736	---
17261393	5,995334	5,813284	5,989637	5,602146	6,007048	5,84398	5,9327517	5,817724667	-0,115027	0,92336503	Acyp2
17550765	4,138051	4,192295	4,337492	3,984131	4,243639	4,095088	4,2226127	4,107619333	-0,11499333	0,923386578	---
17220797	6,64782	7,014528	6,89196	6,804687	6,740025	6,664671	6,851436	6,736461	-0,114975	0,923398312	Tmem206
17419718	5,68226	5,489265	5,740006	5,523254	5,435006	5,608348	5,637177	5,522202667	-0,11497433	0,923398739	Zfp593 // Zfp593
17211545	3,871411	3,272019	3,770505	3,663571	3,561769	3,343673	3,6379783	3,523004333	-0,114974	0,923398952	Gm4850 // Gm4850
17547907	10,845	11,08829	11,04678	10,87762	10,86215	10,89546	10,993357	10,87841	-0,11494667	0,923416447	---
17215605	7,157355	7,148246	7,172114	6,851617	7,227694	7,053573	7,1592383	7,044294667	-0,11494367	0,923418367	Cxcr7
17201775	2,784333	3,580725	3,815825	3,147788	3,3499	3,338369	3,3936277	3,278685667	-0,114942	0,923419434	---
17201951	2,784333	3,580725	3,815825	3,147788	3,3499	3,338369	3,3936277	3,278685667	-0,114942	0,923419434	---
17327609	6,558254	6,316379	6,504979	6,206103	6,481852	6,346838	6,4598707	6,344931	-0,11493967	0,923420927	---
17459936	5,998137	5,915088	6,211136	5,977221	5,820439	5,98196	6,0414537	5,92654	-0,11491367	0,923437569	Ccdc142
17549224	6,393806	6,226655	6,322324	6,157743	6,252825	6,187576	6,3142617	6,199381333	-0,11488033	0,923458905	---
17273806	9,671756	9,717365	9,556985	9,420966	9,536327	9,644252	9,648702	9,533848333	-0,11485367	0,923475975	0610009D07Rik
17252323	10,18325	10,31286	10,10222	10,11456	10,00918	10,13003	10,199443	10,08459	-0,11485333	0,923476188	Txndc17
17278806	3,235866	3,516028	3,494973	3,402422	3,28669	3,2132	3,4156223	3,300770667	-0,11485167	0,923477255	Mir411
17518102	5,054424	5,109521	5,356708	5,119129	4,913633	5,143337	5,173551	5,058699667	-0,11485133	0,923477468	---
17535183	5,344586	4,886762	4,983339	4,985589	5,018618	4,865935	5,0715623	4,956714	-0,11484833	0,923479389	Slitrk2
17217083	8,903297	8,518372	8,86602	8,665617	8,772417	8,50517	8,762563	8,647734667	-0,11482833	0,923492191	Pm20d1
17307522	4,455136	4,429361	4,255051	4,441189	4,130727	4,223154	4,3798493	4,265023333	-0,114826	0,923493684	Dleu7
17243850	8,218107	8,303839	8,172814	8,1208	8,034148	8,19544	8,2315867	8,116796	-0,11479067	0,923516302	4930547N16Rik
17256378	5,453685	5,283051	5,24801	5,348747	5,250315	5,041345	5,3282487	5,213469	-0,11477967	0,923523344	Klhl10
17428882	3,453524	5,304472	4,391096	4,346232	4,426515	4,032043	4,3830307	4,268263333	-0,11476733	0,923531239	---
17482928	5,389077	5,11954	5,418822	5,09731	5,326987	5,158906	5,3091463	5,194401	-0,11474533	0,923545322	4930571K23Rik
17517097	7,207289	6,506904	6,934333	6,676067	6,759714	6,868521	6,882842	6,768100667	-0,11474133	0,923547883	1600029D21Rik
17393369	5,248386	5,116125	5,218707	5,031897	5,068772	5,138334	5,194406	5,079667667	-0,11473833	0,923549803	Fam83c
17453154	7,625175	7,846036	7,82791	7,79256	7,732206	7,430149	7,7663737	7,651638333	-0,11473533	0,923551723	Chchd2
17469035	5,642687	5,672924	5,879223	5,506489	5,695901	5,648273	5,7316113	5,616887667	-0,11472367	0,923559192	LOC100861999
17240969	3,40547	3,311062	2,653696	3,045521	2,940309	3,040244	3,1234093	3,008691333	-0,114718	0,92356282	---
17345180	5,698358	5,608981	6,078458	5,632538	5,809919	5,599357	5,7952657	5,680604667	-0,114661	0,92359931	Tcte1 // Tcte1
17297181	3,928823	3,814469	4,446768	3,998329	3,910256	3,937625	4,0633533	3,948736667	-0,11461667	0,923627692	Sntn
17278395	6,399613	6,387908	6,622715	6,308251	6,539309	6,218936	6,4700787	6,355498667	-0,11458	0,923651167	D430019H16Rik
17423009	4,578953	4,599855	4,214371	4,505574	4,38073	4,163325	4,464393	4,349876333	-0,11451667	0,923691715	Sdr16c5
17429371	6,322826	6,127427	6,081204	6,348275	5,470011	6,369626	6,1771523	6,062637333	-0,114515	0,923692782	Ppih
17549276	3,513192	3,423792	3,598522	3,358568	3,394199	3,439202	3,5118353	3,397323	-0,11451233	0,92369449	---
17517924	3,776948	3,594692	3,986218	3,747448	3,702112	3,564779	3,7859527	3,671446333	-0,11450633	0,923698331	Hcn4
17203043	4,998366	6,153702	4,847409	5,676112	4,871335	5,108529	5,333159	5,218658667	-0,11450033	0,923702173	---
17550793	3,332071	3,282172	3,269409	3,118455	3,258542	3,163176	3,2945507	3,180057667	-0,114493	0,923706868	---
17512776	6,318035	6,526544	6,480577	6,34908	6,210854	6,421826	6,4417187	6,327253333	-0,11446533	0,923724582	Gm1943 // Gm1943
17248309	8,069929	7,920536	8,164815	7,768244	7,953226	8,090423	8,05176	7,937297667	-0,11446233	0,923726503	Stk10
17408684	8,455363	8,546091	8,510426	8,358442	8,479452	8,330638	8,50396	8,389510667	-0,11444933	0,923734827	Dclre1b
17459095	10,27613	10,40216	10,49543	10,08627	10,31033	10,43378	10,39124	10,27679333	-0,11444667	0,923736534	---
17499089	5,433416	5,38249	5,695191	5,335332	5,206123	5,626322	5,503699	5,389259	-0,11444	0,923740803	Gm5607
17331215	3,237144	3,417826	3,649126	3,333217	3,576393	3,051181	3,4346987	3,320263667	-0,114435	0,923744004	---

17435107	6,326311	6,710814	6,16638	6,394187	6,238592	6,227448	6,4011683	6,286742333	-0,114426	0,923749767	Lrrc17
17341631	5,715462	5,288799	5,629709	5,407247	5,561929	5,321529	5,5446567	5,430235	-0,11442167	0,923752541	Prss33
17409379	9,517976	9,625228	9,533422	9,498914	9,459142	9,375324	9,5588753	9,44446	-0,11441533	0,923756597	Tmem167b
17494296	5,424285	5,105763	5,163953	5,202334	5,258783	4,889736	5,2313337	5,116951	-0,11438267	0,923777513	---
17428957	6,144929	5,836409	6,460226	6,219267	5,775412	6,103745	6,147188	6,032808	-0,11438	0,923779221	Dph2
17433968	5,505037	5,163942	5,630213	5,25763	5,412229	5,286409	5,433064	5,318756	-0,114308	0,923825325	Mir429
17522816	4,93546	4,623879	4,7473	4,544572	4,5747	4,844475	4,7688797	4,654582333	-0,11429733	0,923832155	---
17362070	6,358802	6,269098	6,327361	6,141598	6,351602	6,119175	6,3184203	6,204125	-0,11429533	0,923833436	Ccdc88b
17300275	8,659472	8,781061	8,70801	8,420753	8,781431	8,603708	8,716181	8,601964	-0,114217	0,923883598	---
17365072	6,729488	7,147995	7,171787	6,689069	7,099597	6,917995	7,0164233	6,902220333	-0,114203	0,923892563	Bloc1s2
17358651	4,571387	4,195009	4,24984	4,218557	4,263868	4,191209	4,3387453	4,224544667	-0,11420067	0,923894058	Gm19602
17547833	6,731901	6,436795	6,808578	6,440374	6,663275	6,531033	6,6590913	6,544894	-0,11419733	0,923896192	Stmn4 // Stmn4
17360260	5,733687	5,524732	5,632151	5,551213	5,489964	5,506828	5,63019	5,516001667	-0,11418833	0,923901956	Sorcs3
17208605	6,022098	5,881637	5,44674	5,573779	5,542141	5,891994	5,7834917	5,669304667	-0,114187	0,92390281	---
17200583	8,055401	7,079469	7,709808	7,608724	7,458586	7,43481	7,6148927	7,500706667	-0,114186	0,92390345	---
17242133	5,981828	5,834421	6,021532	5,720291	5,844189	5,93086	5,945927	5,83178	-0,114147	0,923928426	Ybey
17547668	6,395196	6,79453	6,558766	6,244536	6,591379	6,570174	6,5828307	6,468696333	-0,11413433	0,923936538	Tmed10 // Tmed10
17295697	8,598013	8,59211	8,528073	8,319314	8,543798	8,5127	8,572732	8,458604	-0,114128	0,923940594	Rad17
17357630	5,723482	5,081476	5,415671	5,472932	5,273102	5,132247	5,4068763	5,292760333	-0,114116	0,923948279	Gpr44
17508835	4,785657	5,158427	5,064603	4,939507	4,934705	4,792152	5,0028957	4,888788	-0,11410767	0,923953616	Sgcz
17471960	3,368326	3,268527	3,443625	3,267312	3,335278	3,135641	3,3601593	3,246077	-0,11408233	0,923969841	Tas2r136
17277659	4,045149	3,068741	3,406734	3,393501	3,367236	3,417645	3,5068747	3,392794	-0,11408067	0,923970908	BB287469
17331078	6,74253	6,800777	6,418916	6,492946	6,627102	6,499984	6,6540743	6,540010667	-0,11406367	0,923981796	Tmem45a
17548569	4,128698	4,231553	4,194615	4,139456	4,001572	4,071837	4,1849553	4,070955	-0,11400033	0,924022359	LOC100862361
17548571	4,128698	4,231553	4,194615	4,139456	4,001572	4,071837	4,1849553	4,070955	-0,11400033	0,924022359	LOC100862361
17375984	5,995086	5,707725	5,951666	5,672653	5,801568	5,83831	5,8848257	5,770843667	-0,113982	0,924034101	Gm355
17481477	6,373472	6,218059	6,424081	6,26779	6,359466	6,046496	6,3385373	6,224584	-0,11395333	0,924052462	Gm15645
17283435	8,733685	8,872561	8,665901	8,567107	8,687733	8,675449	8,7573823	8,643429667	-0,11395267	0,924052889	Gm2695
17201163	5,768872	6,200245	5,790595	5,400666	5,998888	6,018333	5,919904	5,805962333	-0,11394167	0,924059935	---
17244555	4,019525	3,951518	4,404521	4,04508	4,108545	3,880133	4,125188	4,011252667	-0,11393533	0,924063991	---
17210099	4,986934	4,968329	4,331241	5,471707	3,524347	4,948702	4,762168	4,648252	-0,113916	0,924076375	---
17469764	5,071603	4,916088	4,979727	4,701174	5,003021	4,921484	4,9891393	4,875226333	-0,113913	0,924078296	Prrt3
17360861	3,692452	3,937047	3,95371	3,653927	3,773341	3,81432	3,8610697	3,747196	-0,11387367	0,92410349	---
17404071	3,233192	2,810954	2,967321	2,906405	2,952616	2,810837	3,0038223	2,889952667	-0,11386967	0,924106053	---
17353796	7,410195	7,562415	7,214489	7,186633	7,292206	7,366662	7,3956997	7,281833667	-0,113866	0,924108401	Taf7
17299489	3,764805	3,483929	3,541281	3,540129	3,487496	3,420816	3,5966717	3,482813667	-0,113858	0,924113526	Olfr741
17442800	4,468646	4,58889	4,557157	4,37752	4,449859	4,445808	4,538231	4,424395667	-0,11383533	0,924128045	Piwil1
17276250	8,139776	7,807033	8,329008	7,836999	8,164602	7,933623	8,092239	7,978408	-0,113831	0,924130821	---
17500089	4,051777	3,955754	4,189476	3,800608	3,979172	4,075747	4,065669	3,951842333	-0,11382667	0,924133596	1810011O10Rik
17391623	5,480714	5,715988	5,898168	5,499398	5,703213	5,550879	5,69829	5,584496667	-0,11379333	0,924154949	Cpxm1
17533067	6,040642	5,626763	5,950052	5,606866	5,748827	5,920409	5,8724857	5,758700667	-0,113785	0,924160287	Pcsk1n
17415538	4,40571	4,570389	4,95286	4,569173	4,330143	4,688348	4,6429863	4,529221333	-0,113765	0,924173098	---
17357926	4,231385	4,080531	4,022353	4,039491	4,015604	3,937887	4,111423	3,997660667	-0,11376233	0,924174807	Olfr1480
17311452	4,137449	4,335685	4,394009	4,054957	4,167721	4,303193	4,2890477	4,175290333	-0,11375733	0,92417801	4930548G14Rik // 4930548G14Rik
17425287	6,582784	6,692203	6,443588	6,491477	6,456269	6,429584	6,5728583	6,45911	-0,11374833	0,924183775	---
17475112	5,94719	6,160008	6,612223	6,08114	6,152436	6,144681	6,239807	6,126085667	-0,11372133	0,924201071	Rps19
17353420	8,942154	9,178715	9,257462	8,861014	9,097313	9,07885	9,1261103	9,012392333	-0,113718	0,924203206	Fam13b
17350110	9,260571	9,262201	9,257544	9,142588	9,068836	9,227772	9,2601053	9,146398667	-0,11370667	0,924210467	Rbm27
17459406	3,169815	3,198789	3,252819	2,996264	3,096192	3,187848	3,207141	3,093434667	-0,11370633	0,92421068	---
17201425	5,013285	5,093677	5,183754	4,704021	5,094068	5,15158	5,0969053	4,983223	-0,11368233	0,924226055	---
17205283	3,087918	3,610276	4,180574	3,599198	3,664426	3,274184	3,626256	3,512602667	-0,11365333	0,924244633	---
17209473	3,087918	3,610276	4,180574	3,599198	3,664426	3,274184	3,626256	3,512602667	-0,11365333	0,924244633	---
17403150	6,335834	6,42423	6,269307	6,087764	6,410347	6,190413	6,3431237	6,229508	-0,11361567	0,924268764	Tspan5
17342699	5,924521	5,836995	5,663966	5,623381	5,844215	5,617043	5,808494	5,694879667	-0,11361433	0,924269619	Ip6k3
17467407	5,622216	5,318381	5,669061	5,117476	5,414817	5,736524	5,5365527	5,422939	-0,11361367	0,924270046	---
17318405	3,083023	3,186714	2,888333	2,995221	2,822278	2,999772	3,05269	2,939090333	-0,11359967	0,924279015	Mir1942
17290997	13,16941	13,1698	13,20876	13,07971	13,07003	13,05745	13,182657	13,06906333	-0,11359333	0,924283073	Hist1h2bl
17331102	10,02828	10,16749	10,04238	9,999689	9,840713	10,05698	10,079383	9,965794	-0,11358933	0,924285635	Nit2
17240659	3,262753	3,246582	2,823271	2,963547	3,0214	3,00703	3,1108687	2,997325667	-0,113543	0,92431532	---
17508850	8,247043	8,757973	8,051074	8,107292	8,206816	8,40139	8,35203	8,238499333	-0,11353067	0,924323222	Msr1
17322278	3,423033	4,106093	3,42703	3,472418	3,508268	3,634881	3,652052	3,538522333	-0,11352967	0,924323862	---
17521211	8,635961	8,35814	8,340637	8,530526	8,17153	8,292174	8,4449127	8,33141	-0,11350267	0,924341161	Abhd14b
17431876	5,03606	5,157272	5,022706	4,973114	4,999302	4,903171	5,0720127	4,958529	-0,11348367	0,924353335	Gm16287
17385719	9,631615	9,307305	9,222741	9,285527	9,168939	9,366782	9,3872203	9,273749333	-0,113471	0,92436145	Dpp4
17241692	9,36264	9,358324	9,426128	9,220577	9,332991	9,253112	9,382364	9,268893333	-0,11347067	0,924361664	Cdk1
17457685	4,648573	4,660137	5,045104	4,7188	4,847987	4,446652	4,7846047	4,671146333	-0,11345833	0,924369566	---
17229020	10,4987	10,51302	10,43841	10,4376	10,21993	10,45223	10,483377	10,36992	-0,11345667	0,924370634	Fmo1
17415367	7,538007	7,202972	7,512013	7,270257	7,203027	7,439361	7,417664	7,304215	-0,113449	0,924375546	Ifna5
17219156	5,579123	5,515491	5,150419	5,460299	5,372769	5,071666	5,415011	5,301578	-0,113433	0,924385798	3110045C21Rik
17357072	6,105586	6,096693	6,151876	5,967169	6,046028	6,000783	6,1180517	6,00466	-0,11339167	0,924412282	Pla2g16
17494315	4,128819	3,92941	4,116136	4,222743	3,842124	3,769389	4,0581217	3,944752	-0,11336967	0,924426379	Olfr648
17311831	7,029381	7,375121	7,233895	7,121802	7,028168	7,148386	7,212799	7,099452	-0,113347	0,924440903	Trib1
17353781	5,808207	5,897532	5,818034	5,862788	5,631393	5,689635	5,8412577	5,727938667	-0,113319	0,924458845	Pcdhac2 // Pcdhac1 // Pcdha2 // Pcdha1
17544345	3,460505	3,353851	3,362176	3,233183	3,217612	3,385847	3,3921773	3,278880667	-0,11329667	0,924473156	4932411N23Rik
17457430	5,60804	6,111775	5,942402	5,727247	5,784747	5,810474	5,8874057	5,774156	-0,11324967	0,924503274	1110001J03Rik
17328823	5,557616	5,357759	5,904428	5,379186	5,536844	5,564042	5,606601	5,493357333	-0,11324367	0,924507119	Gsc2
17275161	5,541292	5,165012	5,825119	5,573342	5,330606	5,28785	5,5104743	5,397266	-0,11320833	0,924529761	Gm7008
17346107	8,167964	8,164435	8,183846	8,02272	8,058466	8,095484	8,1720817	8,05889	-0,11319167	0,924540442	Ubxn6
17204823	3,545046	4,331687	4,269978	3,607688	4,354763	3,844691	4,0489037	3,935714	-0,11318967	0,924541723	---
17289817	3,507603	3,75197	3,485844	3,535062	3,32694	3,543895	3,5818057	3,468632333	-0,11317333	0,924552191	---
17233022	12,96873	13,00506	12,96756	12,81043	12,82494	12,96647	12,98045	12,86728	-0,11317	0,924554327	Cd24a
17450830	8,929978	8,859937	8,71863	8,811251	8,545984	8,811857	8,8361817	8,723030667	-0,113151	0,924566503	Slc26a1
17204937	3,322899	3,693182	3,43968	3,188232	3,51943	3,408651	3,4852537	3,372104333	-0,11314933	0,924567571	---
17512854	7,260212	7,4554	7,203755	6,847559	7,192378	7,540044	7,3064557	7,193327	-0,11312867	0,924580816	---
17517276	4,007589	3,829093	3,817976	3,686472	3,79917	3,829767	3,884886	3,771803	-0,113083	0,924610083	Gm1715
17463650	9,48506	9,507677	9,4772	9,41051	9,284521	9,435658	9,489979	9,376896333	-0,11308267	0,924610296	2700089E24Rik
17338767	6,716883	6,593533	6,971794	6,858944	6,50491	6,579164	6,7607367	6,647672667	-0,113064	0,92462226	Kdm4b
17422257	4,985224	5,332933	5,239516	5,095233	5,117602	5,005675	5,185891	5,072836667	-0,11305433	0,924628455	Gm833
17423133	4,966729	4,629829	4,55243	4,796268	4,676448	4,337112	4,7163293	4,603276	-0,11305333	0,924629096	---
17391426	8,255132	8,475146	8,861671	8,546335	8,255919	8,450614	8,5306497	8,417622667	-0,113027	0,924645973	---
17470654	3,392308	2,95268	3,663158	3,115114	3,556383	2,997602	3,3360487	3,223033	-0,11301567	0,924653237	Vmn2r22
17547690	4,490075	4,789037	4,875538	4,494332	4,53658	4,78473	4,7182167	4,605214	-0,11300267	0,924661569	Rrm2 // Rrm2
17410424	8,886002	9,051735	8,941599	8,731219	8,957998	8,851141	8,9597787	8,846786	-0,11299267	0,924667978	Aimp1
17541115	4,003152	3,818776	3,948619	3,935359	3,66124	3,835011	3,9235157	3,810536667	-0,112979	0,924676738	---
17379480	3,499317	3,445871	3,646605	3,436079	3,602498	3,214285	3,5305977	3,417620667	-0,112977	0,92467802	---
17222925	8,104692	8,059826	7,95481	7,845765	7,948716	7,98602	8,039776	7,926833667	-0,11294233	0,924700239	Mfsd6
17481149	3,541816	3,500727	3,584543	3,417958	3,529191	3,341117	3,542362	3,429422	-0,11294	0,924701735	Olfr561
17363605	8,486477	8,416993	8,385819	8,279631	8,255738	8,415101	8,429763	8,316823333	-0,11293967	0,924701948	Pip5k1b
17284702	3,542414	3,757246	3,564757	3,623338	3,444563	3,457749	3,6214723	3,50855	-0,11292233	0,924713058	Gm10421
17256806	6,168455	6,28879	6,507084	6,136303	6,232382	6,256994	6,321443	6,208559667	-0,11288333	0,924738056	Rdm1
17382681	5,041641	4,947117	5,014095	4,959635	4,90629	4,798306	5,000951	4,888077	-0,112874	0,924744039	Gm996
17400901	4,789884	4,939398	4,987302	4,852888	4,701851	4,823261	4,905528	4,792666667	-0,11286133	0,924752158	Tbx15
17472635	4,941299	4,821703	4,972324	4,798819	4,733075	4,86486	4,9117753	4,798918	-0,11285733	0,924754722	1700060C16Rik
17407473	3,164066	3,268903	3,328561	3,072908	3,167665	3,182432	3,2538433	3,141001667	-0,11284167	0,924764764	Lce1k
17287304	3,315771	3,077371	3,559347	3,202516	3,22975	3,181761	3,3174963	3,204675667	-0,11282067	0,924778225	---
17377349	4,440802	4,490613	4,783107	4,332317	4,574814	4,470014	4,5715073	4,458715	-0,11279233	0,924796387	---
17381215	4,063908	4,033797	3,969774	3,934829	4,009986	3,784369	4,022493	3,909728	-0,112765	0,924813908	Meig1
17320410	4,487558	4,470809	4,671626	4,331747	4,415184	4,54478	4,543331	4,430570333	-0,11276067	0,924816686	---
17353639	7,874472	7,569754	7,535069	7,617857	7,719706	7,303532	7,659765	7,547031667	-0,11273333	0,924834208	Dnajc18
17346950	9,296394	9,509027	9,692039	9,39714	9,222906	9,539244	9,4991533	9,38643	-0,11272333	0,924840618	Mir1195 // Mir1195
17549206	9,296394	9,509027	9,692039	9,39714	9,222906	9,539244	9,4991533	9,38643	-0,11272333	0,924840618	---
17550080	9,296394	9,509027	9,692039	9,39714	9,222906	9,539244	9,4991533	9,38643	-0,11272333	0,924840618	---
17221057	2,820484	3,162045	3,047793	2,873788	2,829404	2,988973	3,0101073	2,897388333	-0,112719	0,924843396	---
17250567	5,000865	4,854087	5,191456	4,815433	4,795114	5,097707	5,0154693	4,902751333	-0,112718	0,924844037	Aldh3a1
17524969	9,36862	9,40868	9,427445	9,245715	9,293839	9,327044	9,4015817	9,288866	-0,11271567	0,924845533	Anln
17201617	4,271077	4,365731	4,963258	4,414618	4,449491	4,397812	4,5333553	4,420640333	-0,112715	0,924845961	---
17439178	4,193738	4,138967	4,003331	3,950669	3,942042	4,105206	4,112012	3,999305667	-0,11270633	0,924851516	2010109A12Rik
17310515	4,221733	4,171869	3,89606	3,885565	4,01575	4,050269	4,096554	3,983861333	-0,11269267	0,924860278	Cdh9
17351444	7,907182	8,195773	7,976082	7,981781	7,790155	7,969046	8,0263457	7,913660667	-0,112685	0,924865192	Chmp1b
17352173	2,929199	2,981502	3,183781	2,880442	3,076164	2,799848	3,031494	2,918818	-0,112676	0,924870962	---
17487619	4,683952	5,186353	4,094056	4,226902	4,883013	4,51649	4,654787	4,542135	-0,112652	0,924886348	Vmn1r149
17481248	4,692739	4,068467	4,508193	4,490368	4,216	4,225104	4,423133	4,310490667	-0,11264233	0,924892545	Olfr632
17439021	6,223058	6,109673	6,207578	5,973228	6,091202	6,138014	6,180103	6,067481333	-0,11262167	0,924905794	Ereg
17458718	6,165293	6,334952	6,366714	6,142375	6,205146	6,181642	6,2889863	6,176387667	-0,11259867	0,92492054	Prr15
17543024	4,955945	4,927001	4,748946	4,919121	4,560472	4,814522	4,8772973	4,764705	-0,11259233	0,9249246	Tmem47
17374594	6,021362	5,802989	6,240611	5,830055	5,892771	6,004447	6,021654	5,909091	-0,112563	0,924943406	Pak6
17217901	8,162806	8,237079	8,054736	7,892497	8,103461	8,121027	8,1515403	8,038995	-0,11254533	0,924954733	Zbtb41
17291692	7,601313	7,209665	7,568715	7,409378	7,378654	7,254067	7,4598977	7,347366333	-0,11253133	0,924963708	Wrnip1 // Wrnip1
17426043	6,157756	5,642198	5,601299	5,880792	5,542462	5,640429	5,8004177	5,687894333	-0,11252333	0,924968837	Gm2083 // Mup17
17351226	3,481527	3,299476	3,498718	3,192111	3,247112	3,502979	3,4265737	3,314067333	-0,11250633	0,924979737	---
17516257	4,035256	3,855707	4,447397	4,001184	4,081707	3,918088	4,1127867	4,000326333	-0,11246033	0,92500923	Olfr943
17548734	4,109022	4,188039	4,296208	4,235218	4,120719	3,899979	4,1977563	4,085305333	-0,112451	0,925015214	---
17336190	7,155039	7,263538	6,945786	6,99005	7,153351	6,883722	7,1214543	7,009041	-0,11241333	0,925039366	Ndufa7
17200141	3,521539	3,206467	3,694394	3,575703	2,815195	3,694291	3,4741333	3,361729667	-0,11240367	0,925045564	---

17373527	5,508452	5,457947	5,466335	5,358094	5,286389	5,451044	5,477578	5,365175667	-0,11240233	0,925046419	---	
17330162	7,631607	7,874203	7,67278	7,682569	7,775738	7,383143	7,7261967	7,613816667	-0,11238	0,925060739	BC117090	
17417437	8,948967	8,963431	8,886163	8,833171	8,76769	8,860567	8,9328537	8,820476	-0,11237767	0,925062235	Gpbp1l1	
17365444	7,571759	7,826961	7,362421	7,291777	7,688919	7,443332	7,587047	7,474676	-0,112371	0,92506651	Pcgf6	
17267186	5,597026	5,202403	5,788751	5,356256	5,42862	5,466269	5,5293933	5,417048333	-0,112345	0,925083181	9530048J24Rik	
17221215	9,877813	9,969409	9,848855	9,807354	9,773146	9,77862	9,8986923	9,786373333	-0,112319	0,925099853	Cops5	
17221989	4,257349	4,439662	4,092076	4,076179	4,299055	4,07693	4,263029	4,150721333	-0,11230767	0,92510712	---	
17458940	6,439953	6,592134	6,550348	6,495102	6,134242	6,616171	6,5274783	6,415171667	-0,11230667	0,925107761	Vmn1r9	
17242494	5,26108	5,243549	5,485738	5,385527	5,003419	5,264589	5,3301223	5,217845	-0,11227733	0,925126571	---	
17403168	4,65027	4,716766	4,723775	4,467917	4,751147	4,535022	4,696937	4,584695333	-0,11224167	0,925149443	B930007M17Rik	
17435089	7,362252	7,540112	7,342875	7,282062	7,45139	7,175143	7,4150797	7,302865	-0,11221467	0,925166757	Fgl2	
17471146	4,043409	4,093042	4,400746	4,020238	4,167296	4,013033	4,1790657	4,066855667	-0,11221	0,92516975	Gm10415	
17544220	4,247588	4,381711	4,584734	4,363412	4,440195	4,073829	4,4046777	4,292478667	-0,112199	0,925176804	Pof1b	
17392370	4,914797	4,948257	5,000764	4,836553	4,704199	4,986539	4,954606	4,842430333	-0,11217567	0,925191767	Gm5535	
17220533	7,650314	7,729634	7,720251	7,564673	7,633687	7,565329	7,7000663	7,587896333	-0,11217	0,925195401	Bpnt1	
17428786	8,296622	8,235655	8,406815	8,163596	8,243479	8,195523	8,3130307	8,200866	-0,11216467	0,925198821	Rps8	
17362922	7,113815	6,966389	6,615302	7,023964	6,504022	6,831029	6,898502	6,786338333	-0,11216367	0,925199463	Ms4a1	
17317486	9,503174	9,56443	9,369394	9,202001	9,466484	9,432038	9,4789993	9,366841	-0,11215833	0,925202883	Fam49b	
17366597	8,166134	8,072298	7,985891	7,974333	7,940639	7,972899	8,0747743	7,962623667	-0,11215067	0,9252078	Usp6nl	
17517142	6,043111	6,174384	6,242812	5,967028	6,107347	6,04954	6,1534357	6,041305	-0,11213067	0,925220626	Fdxacb1	
17461842	6,517057	6,852447	6,911741	6,597913	6,74338	6,603665	6,760415	6,648319333	-0,11209567	0,925243072	LOC100504971	
17550400	3,658072	3,638407	3,784216	3,558932	3,686276	3,499315	3,693565	3,581507667	-0,11205733	0,925267657	---	
17360570	4,427951	4,317636	4,015515	4,071035	4,524994	3,828921	4,2537007	4,14165	-0,11205067	0,925271932	9930023K05Rik	
17480349	5,452224	5,198356	5,380898	4,962321	5,124382	5,608623	5,343826	5,231775333	-0,11205067	0,925271932	Kctd14	
17200203	4,470236	4,301817	4,938485	4,085079	4,79145	4,497995	4,5701793	4,458174667	-0,11200467	0,925301435	---	
17502521	5,777136	5,889519	5,803315	5,691607	5,711586	5,730774	5,8233233	5,711322333	-0,112001	0,925303787	F2rl3	
17367807	6,189094	6,158884	6,34071	6,101353	6,036912	6,214449	6,2295627	6,117571333	-0,11199133	0,925309987	Nelf	
17208565	5,490748	5,356115	6,056707	5,677278	5,919662	4,970734	5,6345233	5,522558	-0,11196533	0,925326663	---	
17208969	4,198002	4,141517	4,490353	4,393727	4,098183	4,002185	4,276624	4,164698333	-0,11192567	0,925352105	---	
17239751	8,121509	8,668754	8,23812	7,980459	8,41822	8,293932	8,3427943	8,230870333	-0,111924	0,925353174	Snora33	
17411172	3,351596	3,166705	3,110411	3,170384	3,13055	2,99206	3,2095707	3,097664667	-0,111906	0,925364719	---	
17283764	7,132808	7,11403	7,273292	7,038932	7,135425	7,010073	7,1733767	7,061476667	-0,1119	0,925368567	Snhg10	
17377963	6,759242	6,715925	6,75232	6,429852	6,785702	6,676285	6,7424957	6,630613	-0,11188267	0,925379685	Dnmt3b	
17449523	7,467783	8,204122	7,48821	7,646682	7,430635	7,747221	7,7200383	7,608179333	-0,111859	0,925394866	---	
17527984	6,154518	6,227594	6,326814	6,154816	6,075254	6,143294	6,2363087	6,124454667	-0,111854	0,925398073	A730043L09	
17401205	5,967517	5,985576	6,111812	5,815184	5,903217	6,010944	6,021635	5,909781667	-0,11185333	0,925398501	Syt6	
17507665	3,237802	3,467232	4,008012	3,576777	3,200071	3,600651	3,5710153	3,459166333	-0,111849	0,92540128	AF366264	
17387750	4,706863	4,77347	4,869315	4,585351	4,632332	4,796479	4,783216	4,671387333	-0,11182867	0,925414323	Pramel7	
17528395	7,660707	7,515221	7,640419	7,607132	7,487185	7,386594	7,605449	7,493637	-0,111812	0,925425014	Aph1c	
17206389	5,390962	4,991778	5,160417	4,995498	4,915585	5,296639	5,1810523	5,069240667	-0,11181167	0,925425228	---	
17264097	5,378091	4,988101	4,90001	4,997404	5,147756	4,785609	5,088734	4,976923	-0,111811	0,925425655	---	
17425038	6,7456	7,038503	6,705424	6,505272	6,97852	6,670305	6,8298423	6,718032333	-0,11181	0,925426297	Nans // Nans	
17450414	6,523079	6,862419	6,817791	6,586714	6,649629	6,631528	6,7344297	6,622623667	-0,111806	0,925428863	Abcg3	
17437146	2,882196	2,896372	3,096131	2,907878	2,793586	2,837882	2,958233	2,846448667	-0,11178433	0,925442761	---	
17359008	10,36146	10,32218	10,37736	10,29704	10,19466	10,23397	10,353667	10,24189	-0,11177667	0,925447679		03/06/17
17494181	3,103816	3,21493	3,415416	2,902509	3,308478	3,187911	3,2447207	3,132966	-0,11175467	0,925461791	Olfr611	
17516300	3,936491	4,429363	3,955194	4,16461	3,899784	3,921508	4,107016	3,995300667	-0,11171533	0,925487023	Olfr981	
17254244	6,481047	6,323622	6,349991	6,184623	6,553419	6,081498	6,3848867	6,27318	-0,11170667	0,925492583	1700020L24Rik	
17219208	5,625214	5,164009	5,36543	5,36543	5,176253	5,277872	5,3848843	5,273185	-0,11169933	0,925497287	1700009P17Rik	
17333354	3,443601	3,521881	3,40057	3,337329	3,250237	3,44341	3,4553507	3,343658667	-0,111692	0,925501992	Tcp10b	
17302301	3,613419	3,434568	3,521547	3,322071	3,431592	3,480852	3,523178	3,411505	-0,111673	0,925514181	---	
17533577	7,717443	8,037045	7,825086	7,575029	7,907251	7,76233	7,859858	7,748203333	-0,11165467	0,925525942	Rbm10	
17342038	8,05351	8,110023	7,878327	7,714316	8,026324	7,966258	8,0139533	7,902299333	-0,111654	0,925526369	Fahd1	
17288430	4,346587	4,210738	4,526776	4,275802	4,362987	4,11036	4,361367	4,249716333	-0,11165067	0,925528508	1700100L14Rik // 1700100L14Rik	
17206097	2,966786	2,776676	3,108436	3,02806	2,759477	2,729419	2,9506327	2,838985333	-0,11164733	0,925530646	---	
17504367	11,79263	11,85019	11,87514	11,69114	11,78025	11,71168	11,83932	11,72769	-0,11163	0,925541766	Sap18 // Sap18	
17509031	4,132527	4,043473	4,257071	4,153269	4,013107	3,931818	4,144357	4,032731333	-0,11162567	0,925544546	Gm6329	
17203161	5,081999	4,051572	4,858431	4,233006	5,217132	4,20699	4,6640007	4,552376	-0,11162467	0,925545188	---	
17357226	11,60323	11,56262	11,74194	11,54204	11,59848	11,43247	11,63593	11,52433	-0,1116	0,925561012	---	
17202983	3,930894	4,144757	4,712909	4,830494	3,683742	3,939557	4,2628533	4,151264333	-0,111589	0,92556807	---	
17299968	4,373723	3,991023	4,074695	4,059093	3,991031	4,054565	4,1464803	4,034896333	-0,111584	0,925571277	---	
17412350	8,374787	8,459401	8,397682	8,236127	8,284361	8,376631	8,4106233	8,299039667	-0,11158367	0,925571491	Map3k7	
17230480	5,563414	5,530277	5,668387	5,517975	5,521531	5,387868	5,5873593	5,475791333	-0,111568	0,925581542	---	
17550741	3,674069	3,688806	3,852189	3,588439	3,573964	3,717961	3,7383547	3,626788	-0,11156667	0,925582398	---	
17333642	9,028224	9,334032	8,951999	8,8447	8,833472	9,301392	9,1047517	8,993188	-0,11156367	0,925584322	Zfp960 // Zfp960	
17548858	4,444018	4,182232	3,95115	4,127761	3,934027	4,180938	4,1924667	4,080908667	-0,111558	0,925587958	Xlr4a	
17210141	4,286231	5,400536	4,521689	4,78579	3,996079	5,091921	4,736152	4,624596667	-0,11155533	0,925589669	---	
17405621	3,243699	3,415491	2,834672	2,884238	3,223893	3,051074	3,1646207	3,053068333	-0,11155233	0,925591594	Vmn2r4	
17546192	8,587954	8,425225	8,226787	8,180505	8,393126	8,331718	8,413322	8,301783	-0,111539	0,925600148	Hccs	
17520251	3,393631	3,315325	3,183351	3,159789	3,351183	3,046746	3,2974357	3,185906	-0,11152967	0,925606136	---	
17319103	6,70227	6,657376	6,576096	6,30156	6,552038	6,747571	6,6452473	6,533723	-0,11152433	0,925609558	Gm10865 // Gm10865	
17379523	10,43671	10,55521	10,42951	10,29932	10,25348	10,53407	10,47381	10,36229	-0,11152	0,925612338	Ube2c	
17497729	7,259302	7,18274	7,337344	7,126097	7,182176	7,136564	7,2597953	7,148279	-0,11151633	0,92561469	Sigirr	
17514541	5,23645	5,395666	5,278295	5,231341	5,20306	5,141481	5,3034703	5,191960667	-0,11150967	0,925618968	Mmp10	
17233066	8,420753	9,278378	8,807491	8,18911	8,734219	9,24877	8,8355407	8,724033	-0,11150767	0,925620251	---	
17489241	7,066517	7,913569	7,762283	7,170038	7,783977	7,453901	7,5807897	7,469305333	-0,11148433	0,925635221	---	
17314824	6,120996	5,732059	5,863523	5,587364	6,008975	5,785796	5,905526	5,794045	-0,111481	0,92563736	---	
17281193	7,82216	7,973945	7,593647	7,593401	7,58567	7,876257	7,796584	7,685109333	-0,11147467	0,925641424	Ppp2r3c // Ppp2r3c	
17274050	5,506814	4,840987	5,211	4,847619	5,164393	5,212443	5,186267	5,074818333	-0,11144867	0,925658105	7420701I03Rik	
17305235	3,976163	3,91337	4,218772	3,837608	3,851263	4,085103	4,0361017	3,924658	-0,11144367	0,925661314	Dydc2	
17418403	7,34933	7,215011	7,28166	7,094665	7,126706	7,29032	7,2820003	7,170563667	-0,11143667	0,925665805	Rspo1	
17202191	3,348613	3,053003	3,148491	2,973156	3,053003	3,189692	3,183369	3,071950333	-0,11141867	0,925677354	---	
17267053	6,784582	6,255325	6,61263	6,311378	6,580274	6,426636	6,5508457	6,439429333	-0,11141633	0,925678851	---	
17201405	4,210737	4,662764	3,956654	4,039721	4,381765	4,074444	4,2767183	4,16531	-0,11140833	0,925683984	---	
17550166	5,406466	5,105631	6,130409	5,52744	5,179617	5,601349	5,547502	5,436135333	-0,11136667	0,92571072	---	
17420963	5,763801	5,719284	5,631067	5,811162	5,508667	5,460341	5,7047173	5,59339	-0,11132733	0,925735958	Spata21	
17482307	10,46112	10,54032	10,4053	10,36671	10,31861	10,38745	10,468913	10,35759	-0,11132333	0,925738525	Ppp1cc	
17475839	5,697152	5,752644	5,787678	5,434274	5,802208	5,667124	5,7458247	5,634535333	-0,11128933	0,925760342	Gmfg	
17294228	5,155461	5,188115	5,105368	5,054372	4,955515	5,105195	5,149648	5,038360667	-0,11128733	0,925761626	Gm20554	
17453064	4,494411	4,390411	4,607637	4,426362	4,454988	4,27726	4,4974863	4,386203333	-0,111283	0,925764406	Rimbp2	
17384927	7,677721	7,723397	7,546131	7,487691	7,548954	7,576793	7,649083	7,537812667	-0,11127033	0,925772534	Gtdc1	
17508394	3,596269	3,703055	3,902666	3,770522	3,5059	3,591789	3,7339967	3,622737	-0,11125967	0,925779379	---	
17522547	6,119968	6,544224	5,943182	6,019448	6,082752	6,171402	6,202458	6,091200667	-0,11125733	0,925780876	Rtp3 // Rtp3	
17434285	6,42382	6,63434	6,893717	6,715707	6,403249	6,499161	6,6506257	6,539372333	-0,11125333	0,925783443	Ccl21a	
17302031	5,555398	5,21176	5,288771	5,17646	5,186504	5,359241	5,3519763	5,240735	-0,11124133	0,925791144	Siah3	
17336062	3,547879	3,665669	3,568791	3,549812	3,427814	3,471019	3,594113	3,482881667	-0,11123133	0,925797561	LOC100861596	
17225173	12,47916	12,7505	12,61294	12,47121	12,49324	12,54451	12,6142	12,50298667	-0,11121333	0,925809112	Snord82	
17284860	3,620023	3,602731	3,735232	3,376152	3,744872	3,503349	3,652662	3,541457667	-0,11120433	0,925814887	Speer6-ps1	
17236638	7,368377	7,330749	7,294047	7,254649	7,086639	7,318332	7,3310577	7,219873333	-0,11118433	0,925827722	Fgd6	
17483716	5,739042	5,832863	6,117171	5,581182	5,93746	5,83691	5,8963587	5,785184	-0,11117467	0,925833925	Slc5a2	
17255042	5,55724	5,303802	5,577361	5,476178	5,401917	5,226797	5,4794677	5,368297333	-0,11117033	0,925836706	Dgke	
17210059	4,171213	4,464853	4,289684	4,094298	3,887419	4,610542	4,3085833	4,197419667	-0,11116367	0,925840984	---	
17290980	3,70662	3,614044	3,598563	3,54179	3,320879	3,723069	3,6397423	3,528579333	-0,111163	0,925841412	Olfr1366	
17330093	3,085086	3,651848	3,417145	3,340138	3,264814	3,215642	3,384693	3,273531333	-0,11116167	0,925842268	---	
17494717	4,100578	4,214056	4,414929	4,470179	3,909668	4,016306	4,2431877	4,132051	-0,11113667	0,925858312	Olfr6	
17413384	7,0405	7,13386	7,037977	7,138567	6,752785	6,987621	7,070779	6,959657667	-0,11112133	0,925868152	Rgp1	
17372942	3,397111	3,311486	3,575579	3,365682	3,251407	3,333744	3,4280587	3,316944333	-0,11111433	0,925872644	Olfr1258	
17402408	5,192216	4,969073	4,779785	5,683936	3,764235	5,159646	4,980358	4,869272333	-0,11108567	0,925891042	---	
17503910	8,203704	8,186564	8,144257	8,077279	8,090431	8,033576	8,178175	8,067095333	-0,11107967	0,925894893	Ogfod1	
17323068	9,437798	9,466631	9,622091	9,266056	9,332272	9,594987	9,50884	9,397771667	-0,11106833	0,925902166	---	
17267581	4,366917	4,184245	4,611588	4,474538	4,203826	4,15122	4,3875833	4,276528	-0,11105533	0,925910509	---	
17353333	5,817918	5,602188	6,16868	5,960384	5,710143	5,58513	5,8629287	5,751885667	-0,111043	0,925918425	A830052D11Rik	
17206983	6,843762	6,988954	7,036152	6,835019	6,930162	6,770588	6,9562893	6,845256333	-0,111033	0,925924843	---	
17499813	4,055387	3,818549	3,75068	3,885931	3,683698	3,721891	3,874872	3,76384	-0,111032	0,925925485	Defb50	
17212016	5,132215	4,734244	4,68571	4,741392	4,837733	4,63995	4,850723	4,739691667	-0,11103133	0,925925912	---	
17245202	9,264698	9,351641	9,2757	9,15	9,191109	9,217944	9,2973463	9,186351	-0,11099533	0,925949018	Yeats4	
17292168	3,153574	2,976487	3,449571	3,007154	3,150165	3,089381	3,1932107	3,082233333	-0,11097733	0,92596057	---	
17516993	10,2765	10,39596	10,27334	10,21274	10,23863	10,16154	10,315267	10,20430333	-0,11096333	0,925969556	Nhp2l1	
17500919	5,562304	5,546681	5,629741	5,491171	5,351708	5,563013	5,5795753	5,468630667	-0,11094467	0,925981537	Sorbs2	
17484000	3,914949	3,965897	4,041747	3,918132	3,929994	3,741674	3,9741977	3,863266667	-0,110931	0,925990309	Fam24a	
17414173	8,358926	8,585309	8,380555	8,378312	8,339759	8,274001	8,4415967	8,330690667	-0,110906	0,926006355	Nipsnap3b	
17286566	5,181231	5,27061	5,268761	4,880421	5,152275	5,355246	5,2402007	5,129314	-0,11088667	0,926018765	---	
17455740	3,600753	3,260427	2,961319	3,136616	3,262568	3,090671	3,2741663	3,163285	-0,11088133	0,926022188	---	
17386685	4,97719	5,062771	5,026245	4,950103	4,858141	4,925358	5,0220687	4,911200667	-0,110868	0,926030746	---	
17368685	6,441434	6,461822	6,372991	6,315944	6,282797	6,344903	6,4254157	6,314548	-0,11086767	0,92603096	Ralgds	
17310582	2,984045	3,053003	3,305652	2,980289	3,018262	3,011639	3,1142333	3,003396667	-0,11083667	0,926050859	---	
17235239	5,474271	5,495478	5,570691	5,437778	5,429749	5,340432	5,51348	5,402653	-0,110827	0,926057064	1600002K03Rik	
17483220	9,368208	9,506473	9,306246	9,309327	9,360644	9,178513	9,3936423	9,282828	-0,11081433	0,926065194	Cdipt	
17272019	6,293198	6,417446	6,386396	6,047867	6,290072	6,426704	6,36568	6,254881	-0,110799	0,926075037	Caskin2	
17547634	10,44362	10,59637	10,4496	10,33563	10,40521	10,41637	10,49653	10,38573667	-0,11079333	0,926078674	Kpnb1	
17285143	4,559068	4,547161	4,905754	4,769013	4,517924	4,392701	4,670661	4,559879333	-0,11078167	0,926086163	---	
17270508	7,608846	7,71615	7,464566	7,433734	7,499839	7,523725	7,5965207	7,485766	-0,11075467	0,926103495	Plekhm1	
17206183	5,212512	5,534633	4,921131	5,097084	5,086599	5,152361	5,2227587	5,112014667	-0,110744	0,926110342	---	
17456560	5,714972	5,626711	6,037062	5,6016	5,879017	5,565931	5,792915	5,682182667	-0,11073233	0,926117831	Fam71f2	

17525088	3,311822	3,331477	3,442609	3,202933	3,217708	3,333123	3,3619693	3,251254667	-0,11071467	0,926129172	---
17343117	3,312895	4,136012	3,259315	3,538801	3,534135	3,303232	3,5694073	3,458722667	-0,11068467	0,926148431	---
17376780	4,402804	4,405635	4,450189	4,265171	4,442692	4,21873	4,4195427	4,308864333	-0,11067833	0,926152497	---
17467306	3,62067	3,702031	3,748561	3,733608	3,487459	3,518206	3,6904207	3,579757667	-0,110663	0,92616234	Vmn1r39
17488975	7,324259	7,53332	7,532754	7,336228	7,419057	7,303124	7,4634443	7,352803	-0,11064133	0,926176249	Tbcb
17489936	3,59885	3,50926	3,818003	3,477012	3,607999	3,50926	3,6420377	3,531423667	-0,110614	0,926193797	---
17351082	4,241643	4,064649	4,173025	4,283549	3,942032	3,92196	4,1597723	4,049180333	-0,110592	0,926207921	Pde6a
17475597	6,488648	6,641328	6,616425	6,38081	6,585901	6,447945	6,5821337	6,471552	-0,11058167	0,926214555	Sertad1
17299449	3,921986	3,888402	3,771961	3,788274	3,540412	3,921929	3,860783	3,750205	-0,110578	0,926216909	4933429O19Rik
17205805	3,777905	3,442638	3,999506	3,45321	3,857708	3,577402	3,7400163	3,62944	-0,11057633	0,926217979	---
17387571	5,134875	4,880538	5,073639	4,952128	4,913505	4,891691	5,029684	4,919108	-0,110576	0,926218193	Olfr987
17509944	6,023995	5,732121	6,004258	5,944495	5,81204	5,672121	5,9201247	5,809552	-0,11057267	0,926220333	Ncan
17498057	5,37809	4,81311	5,337296	4,982644	5,07401	5,140126	5,1761653	5,065593333	-0,110572	0,926220761	Gm4553
17537730	5,712711	5,985425	5,834729	5,782019	5,699217	5,719991	5,8442883	5,733742333	-0,110546	0,926237453	Armcx4
17456545	5,199987	4,994887	5,016057	4,972993	4,861217	5,045083	5,0703103	4,959764333	-0,110546	0,926237453	Lep
17459010	3,046442	2,998012	2,977503	2,850041	2,920171	2,920127	3,007319	2,896779667	-0,11053933	0,926241733	Vmn1r29
17303695	10,91901	10,88937	10,75474	10,64814	10,77882	10,8046	10,854373	10,74385333	-0,11052	0,926254146	2700060E02Rik
17498892	7,891275	7,917285	7,893396	7,73995	7,734044	7,896425	7,900652	7,790139667	-0,11051233	0,926259068	Abhd13
17380093	4,267355	4,097864	4,148247	3,879897	4,362325	3,939759	4,1711553	4,060660333	-0,110495	0,926270197	---
17508775	6,384317	6,248134	6,302212	6,139398	6,255735	6,20806	6,3115543	6,201064333	-0,11049	0,926273407	Mfhas1
17443430	8,740708	8,588638	8,729969	8,526206	8,634434	8,567341	8,6864383	8,575993667	-0,11044467	0,926302513	Polr2j
17213295	10,46777	10,64658	10,57241	10,50934	10,43546	10,41065	10,562253	10,45181667	-0,11043667	0,92630765	Nop58
17337142	6,537861	6,869142	6,790361	6,85096	6,434842	6,580274	6,7324547	6,622025333	-0,11042933	0,926312358	H2-Q10
17399802	4,753723	4,475518	4,598657	4,661102	4,467721	4,367801	4,6092993	4,498874667	-0,11042467	0,926315355	S100a4
17376664	3,188023	2,934185	2,988647	3,060479	3,029922	2,689211	3,0369517	2,926537333	-0,11041433	0,92632199	---
17508994	4,817334	4,728158	4,84385	4,703393	4,75357	4,601145	4,7964473	4,686036	-0,11041133	0,926323916	Zfp42
17448256	4,970431	4,60992	5,243229	4,914414	4,756395	4,821666	4,9411933	4,830825	-0,11036833	0,926351526	---
17257067	6,609951	6,756195	6,677645	6,668283	6,487647	6,556825	6,6812637	6,570918333	-0,11034533	0,926366294	Acbd4
17389291	4,133889	4,003225	3,916348	3,931466	3,951227	3,839761	4,0178207	3,907484667	-0,110336	0,926372287	Olfr1305
17426011	6,018926	5,617553	5,386971	5,636378	5,357343	5,698785	5,6744833	5,564168667	-0,11031467	0,926385986	Mup8
17499748	3,766245	3,742232	3,652611	3,656148	3,480082	3,693979	3,7203627	3,610069667	-0,110293	0,926399898	Gm15293
17302287	3,508822	3,28851	3,034883	3,106981	3,179027	3,21545	3,277405	3,167152667	-0,11025233	0,926426012	---
17547462	2,904436	3,46986	3,265349	3,286526	3,093125	2,929259	3,213215	3,10297	-0,110245	0,926430721	---
17312280	7,777328	7,972073	7,755497	7,841062	7,84347	7,489694	7,834966	7,724742	-0,110224	0,926444206	Gsdmd
17284591	7,132005	6,834163	6,906627	7,161839	6,460219	6,920145	6,9575983	6,847401	-0,11019733	0,926461331	---
17266724	5,22286	4,87843	4,898829	4,737401	4,897709	5,034462	5,0000397	4,889857333	-0,11018233	0,926470964	C030013C21Rik
17311551	9,262732	9,433846	8,944517	9,132541	9,114108	9,064014	9,2136983	9,103554333	-0,110144	0,926495581	Col14a1
17518563	5,116571	4,740066	5,139423	5,066826	4,906121	4,692757	4,9986867	4,888568	-0,11011867	0,92651185	Rasl12
17384202	5,532374	5,359378	5,732239	5,560601	5,31728	5,415832	5,5413303	5,431237667	-0,11009267	0,926528548	9430024E24Rik
17372377	4,710433	4,327509	4,498129	4,296805	4,463586	4,445454	4,5120237	4,401948333	-0,11007533	0,926539679	Ppp1r1c
17336874	5,680165	5,451988	5,564302	5,410192	5,655575	5,300528	5,565485	5,455431667	-0,11005333	0,926553809	D17H6S56E-3
17439861	7,417189	7,729884	7,694709	7,731493	7,332315	7,447879	7,6139273	7,503895667	-0,11003167	0,926567724	BC005561
17548201	5,684244	5,661013	6,070498	5,647219	5,793749	5,644693	5,8052517	5,695220333	-0,11003133	0,926567938	Fbxw5 // Fbxw5
17474797	4,773354	4,859175	4,794221	4,941578	4,605686	4,549512	4,8089167	4,698925333	-0,10999133	0,926593628	Vmn1r157
17547717	4,649478	4,708717	5,121968	4,645859	4,826996	4,677368	4,826721	4,716741	-0,10998	0,926600907	---
17243406	5,444552	4,947434	5,463739	5,281273	5,233654	5,010922	5,2852417	5,175283	-0,10995867	0,926614609	Tle6
17539519	7,578836	7,55585	7,538572	7,532219	7,274158	7,537051	7,5577527	7,447809333	-0,10994333	0,926624457	Pir
17328884	8,473284	8,59148	8,434424	8,424723	8,364088	8,380553	8,4997293	8,389788	-0,10994133	0,926625742	Ranbp1
17281529	8,852977	8,902118	8,778697	8,807648	8,729145	8,667324	8,8445973	8,734705667	-0,10989167	0,926657643	Nemf
17504005	11,24406	11,30342	11,34699	11,13498	11,24543	11,18441	11,298157	11,18827333	-0,10988333	0,926662995	Herpud1
17221502	5,783737	5,902085	6,011413	5,632134	5,985669	5,749793	5,8990783	5,789198667	-0,10987967	0,926665351	Jph1
17411716	5,460861	5,464007	5,390434	5,200094	5,305347	5,48026	5,438434	5,328567	-0,109867	0,926673487	4930412C18Rik
17419420	7,003713	7,245569	7,429554	6,979605	7,12628	7,243382	7,2262787	7,116422333	-0,10985633	0,926680338	---
17252754	10,6452	10,97267	10,96648	10,53738	10,92229	10,79523	10,86145	10,75163333	-0,10981667	0,926705817	---
17504572	8,192358	8,59565	8,202454	8,359427	7,940301	8,361287	8,330154	8,220338333	-0,10981567	0,92670646	Ces2g
17208175	6,065308	5,221043	6,138883	6,01889	5,112375	5,964533	5,8084113	5,698599333	-0,109812	0,926708815	---
17231252	5,951303	5,800332	6,050754	5,975618	5,713859	5,783555	5,9341297	5,824344	-0,10978567	0,92672573	Camk1g
17240022	5,656457	5,676258	5,767779	5,460855	5,648714	5,661586	5,7001647	5,590385	-0,10977967	0,926729584	Cenpw
17547606	5,905484	5,717551	6,005167	5,731761	5,762521	5,8046	5,8760673	5,766294	-0,10977333	0,926733653	Gm9734
17275151	7,630539	7,326474	7,402966	7,602497	7,241971	7,186193	7,4533263	7,343553667	-0,10977267	0,926734081	---
17383430	7,535739	7,696548	7,601163	7,54641	7,451937	7,505792	7,61115	7,501379667	-0,10977033	0,92673558	Gtf3c4
17384422	11,6538	11,52417	11,54219	11,49023	11,45319	11,44744	11,573387	11,46362	-0,10976667	0,926737935	AI182371
17455673	5,516439	5,56585	5,03939	4,977642	5,492047	5,322718	5,373893	5,264135667	-0,10975733	0,926743931	---
17447735	3,274113	3,489318	3,156721	3,512484	3,103461	2,974998	3,3067173	3,196981	-0,10973633	0,92675742	---
17548211	6,644129	7,020603	7,006835	6,552932	6,927443	6,862006	6,8905223	6,780793667	-0,10972867	0,926762345	---
17421858	4,80187	4,71244	4,476871	4,513216	4,473464	4,675365	4,663727	4,554015	-0,109712	0,926773052	Spsb1
17289289	7,054728	7,186509	7,271988	6,988739	7,193315	7,002057	7,171075	7,061370333	-0,10970467	0,926777763	Poc5
17223300	5,029406	4,744893	5,092401	4,774817	5,106461	4,656333	4,9555667	4,845870333	-0,10969633	0,926783116	BC055402
17548040	5,231718	4,929171	5,186768	4,81502	5,024837	5,178745	5,1158857	5,006200667	-0,109685	0,926790396	Gm10554
17500332	5,144938	4,461096	4,865499	4,861149	4,605655	4,675703	4,8238443	4,714169	-0,10967533	0,926796606	Gm9731
17278124	3,997279	4,008112	3,581455	3,651922	3,813061	3,792847	3,862282	3,75261	-0,109672	0,926798748	Cox8c
17487208	4,654897	4,384192	4,426548	4,765	4,114558	4,257129	4,4885457	4,378895667	-0,10965	0,926812881	---
17520582	4,307918	4,064638	4,783814	4,425677	3,937401	4,464351	4,3854567	4,275809667	-0,109647	0,926814808	C430002N11Rik
17368184	3,889456	3,777074	4,041051	3,793085	3,949835	3,635749	3,902527	3,792889667	-0,10963733	0,926821018	Lcn3
17520416	10,09054	9,940409	10,17775	10,05921	9,948657	9,871922	10,069566	9,959929667	-0,10963667	0,926821446	Paqr9
17274389	6,98231	6,646598	6,824357	6,584279	6,993319	6,546775	6,817755	6,708124333	-0,10963067	0,926825301	---
17538194	4,330656	4,143352	4,516142	4,374771	4,181985	4,104525	4,33005	4,220427	-0,109623	0,926830226	Vsig1
17463654	5,503044	5,382849	5,289867	5,55204	5,135396	5,159542	5,39192	5,282326	-0,109594	0,926848857	---
17464638	8,857323	8,892194	8,794316	8,674815	8,811271	8,728993	8,8479443	8,738359667	-0,10958467	0,926854853	Pon2
17247332	3,872028	3,524831	3,673162	3,852573	3,431555	3,457157	3,690007	3,580428333	-0,10957867	0,926858708	---
17378640	6,357565	6,339128	6,428355	6,285836	6,130205	6,380287	6,3750193	6,265442667	-0,10957667	0,926859993	Dlgap4
17537190	10,94993	10,81048	10,81001	10,80244	10,6842	10,75505	10,856807	10,74723	-0,10957667	0,926859993	Sh3bgrl
17306638	6,878954	7,034279	6,80847	6,676153	6,891443	6,8254	6,9072343	6,797665333	-0,109569	0,926864918	Ap1g2
17246636	7,649412	7,867809	7,734353	7,838697	7,437162	7,647014	7,7505247	7,640957667	-0,109567	0,926866203	Morc2a
17282281	5,724861	5,540174	5,768289	5,438405	5,508661	5,757568	5,6777747	5,568211333	-0,10956333	0,926868559	0610009B14Rik
17538079	5,907722	5,467115	5,43677	5,518283	5,50717	5,457496	5,603869	5,494316333	-0,10955267	0,926875412	D330045A20Rik
17315530	3,687214	3,740114	3,777343	3,550725	3,715627	3,609703	3,7348903	3,625351667	-0,10953867	0,926884406	Hoxc5
17536026	6,144671	6,199246	6,400295	6,59037	6,106295	5,718951	6,2480707	6,138538667	-0,109532	0,926888689	---
17344555	3,96067	3,886083	3,966021	3,881645	3,827236	3,775306	3,9375913	3,828062333	-0,109529	0,926890617	---
17334780	6,003527	5,847776	5,973129	5,80198	5,947493	5,746431	5,9414773	5,831968	-0,10950933	0,926903252	Metrn // Metrn
17458960	3,79282	3,551477	4,252568	3,780117	3,812314	3,675927	3,8656217	3,756119333	-0,10950233	0,926907749	Vmn1r20
17304186	7,038548	6,817694	7,158484	6,950133	6,765236	6,970852	7,0049087	6,895407	-0,10950167	0,926908178	Plac9
17341319	3,723058	3,645543	3,672543	3,680681	3,6076	3,424402	3,6803813	3,570894333	-0,109487	0,926917601	Vmn2r108
17310770	5,161294	5,00458	4,710007	5,304711	4,566566	4,676162	4,958627	4,849146333	-0,10948067	0,92692167	---
17550032	5,161294	5,00458	4,710007	5,304711	4,566566	4,676162	4,958627	4,849146333	-0,10948067	0,92692167	---
17221245	11,76593	11,91078	11,86468	11,6842	11,78426	11,7445	11,84713	11,73765333	-0,10947667	0,92692424	---
17519509	7,26739	7,598291	7,310718	7,108954	7,375104	7,363914	7,392133	7,282657333	-0,10947567	0,926924882	Leo1
17448787	9,058952	8,982105	9,259794	8,579398	8,621521	9,771525	9,1002837	8,990814667	-0,109469	0,926929166	---
17479897	4,834873	5,103462	4,861232	4,798388	4,819829	4,852948	4,933189	4,823721667	-0,10946733	0,926930237	Gm20077
17498592	4,320054	4,34707	4,295618	4,246615	4,241539	4,146228	4,320914	4,211460667	-0,10945333	0,926939232	1700019B03Rik
17433858	4,485355	4,171216	4,438841	4,423171	4,320232	4,02374	4,3651373	4,255714333	-0,109423	0,926958721	---
17355565	9,467902	9,332758	9,233798	9,148447	9,211373	9,346425	9,3448193	9,235415	-0,10940433	0,926970715	Slc14a1
17433803	4,987598	4,474772	4,539462	4,491984	4,635059	4,54661	4,6672773	4,557884333	-0,109393	0,926977997	5830444B04Rik
17336245	6,818582	6,987575	7,080422	7,028754	6,746107	6,783662	6,962193	6,852841	-0,109352	0,927004341	Rgl2
17516135	4,394364	4,911597	4,739696	4,291572	4,791329	4,634723	4,6818857	4,572541333	-0,10934433	0,927009267	Olfr887
17439481	10,55786	10,59169	10,55872	10,40134	10,43768	10,54125	10,569423	10,46009	-0,10933333	0,927016335	Bmp2k
17347839	4,20681	4,396391	4,87126	4,269952	4,40593	4,470643	4,491487	4,382175	-0,109312	0,927030043	Six2
17433353	8,123872	8,166097	8,150723	7,922319	8,09688	8,093583	8,1468973	8,037594	-0,10930333	0,927035612	Vamp3
17208475	7,245585	6,83207	6,867762	6,904586	7,111226	6,6017	6,9818057	6,872504	-0,10930167	0,927036683	---
17316867	3,674163	3,692858	3,644832	3,65149	3,642447	3,390043	3,6706177	3,561326667	-0,109291	0,927043537	4930523O13Rik
17263444	3,484602	3,534135	3,666859	3,529789	3,421065	3,406877	3,5618653	3,452577	-0,10928833	0,927045251	Olfr223
17464718	10,23621	10,39399	10,46746	10,42201	10,2464	10,10143	10,365887	10,25661333	-0,10927333	0,92705489	Asns
17344456	7,784501	7,810627	7,806387	7,769398	7,699372	7,605145	7,800505	7,691305	-0,1092	0,927102014	2310061I04Rik
17325991	3,948703	3,966796	3,897295	4,055606	3,666353	3,763264	3,937598	3,828407667	-0,10919033	0,927108226	---
17453087	9,102921	9,094113	9,149055	8,973902	9,077621	8,966999	9,115363	9,006174	-0,109189	0,927109083	Stx2
17202943	3,919673	3,343488	3,676833	3,275395	3,127676	4,209361	3,6466647	3,537477333	-0,10918733	0,927110154	---
17224977	4,807096	4,479072	4,851249	4,785144	4,4642	4,560517	4,7124723	4,603287	-0,10918533	0,927111439	Dner
17214220	8,453549	8,094117	8,26125	7,980008	8,277526	8,223946	8,2696387	8,160493333	-0,10914533	0,927137144	Ctdsp1
17334449	9,313319	9,358158	9,384522	9,338057	9,16222	9,228325	9,3519997	9,242867333	-0,10913233	0,927145499	Hagh
17429008	8,571502	8,394569	8,437343	8,388802	8,285373	8,401888	8,4678047	8,358687667	-0,109117	0,927155353	St3gal3
17434167	3,788095	3,896949	3,53288	3,649651	3,73321	3,507727	3,739308	3,630196	-0,109112	0,927158566	Vmn2r122
17281783	5,271008	5,155781	5,244469	5,109659	5,083127	5,151145	5,2237527	5,114643667	-0,109109	0,927160494	Dact1 // Dact1
17549070	6,226285	6,307932	6,502848	6,035333	6,361259	6,313164	6,3456883	6,236585333	-0,109103	0,92716435	---
17205259	3,511687	3,720249	3,249862	3,170158	3,511668	3,472668	3,4939327	3,384831333	-0,10910133	0,927165421	---
17209449	3,511687	3,720249	3,249862	3,170158	3,511668	3,472668	3,4939327	3,384831333	-0,10910133	0,927165421	---
17415130	5,080867	4,940807	5,022601	4,876903	4,925971	4,914136	5,0147583	4,90567	-0,10908833	0,927173776	Gm12680
17418260	9,668629	9,418251	9,049002	9,184104	9,23869	9,38591	9,3786273	9,269568	-0,10905933	0,927192413	---
17319700	6,761198	7,093987	6,798358	6,650017	6,440935	7,235473	6,8845143	6,775475	-0,10903933	0,927205267	---
17465298	8,810434	8,832855	8,736586	8,559909	8,729584	8,763332	8,7932917	8,684275	-0,10901667	0,927219835	Zfp800
17387700	5,380427	4,878272	4,484036	4,769629	4,995839	4,650226	4,914245	4,805231333	-0,10901367	0,927221763	Olfr1085
17236339	7,429286	7,519679	7,187374	7,311965	7,218024	7,279522	7,3787797	7,269837	-0,10894267	0,927267396	Gnptab
17396521	5,190764	5,034448	5,094931	5,074275	5,050221	4,868819	5,1067143	4,997771667	-0,10894267	0,927267396	Egfem1
17203035	4,041594	3,566289	3,919512	3,922017	3,58198	3,696594	3,842465	3,733530333	-0,10893467	0,927272538	---
17279608	8,817999	9,082537	9,19591	8,948295	8,915693	8,905684	9,0321487	8,923224	-0,10892467	0,927278965	Ncapg2

17389009	9,749416	9,999244	9,81279	9,786913	9,664628	9,783144	9,8538167	9,744895	-0,10892167	0,927280893	Eif3m
17232202	8,871064	8,654202	8,568116	8,447091	8,599778	8,719751	8,697794	8,588873333	-0,10892067	0,927281536	Stx7
17209947	7,575074	7,25173	7,425105	7,420846	7,094748	7,409576	7,417303	7,30839	-0,108913	0,927286464	---
17205105	4,323597	3,752135	4,339259	4,190877	4,516726	3,380715	4,1383303	4,029439333	-0,108891	0,927300604	---
17209295	4,323597	3,752135	4,339259	4,190877	4,516726	3,380715	4,1383303	4,029439333	-0,108891	0,927300604	---
17227675	8,618447	8,900791	8,782072	8,566588	8,680604	8,727575	8,7671033	8,658255667	-0,10884767	0,927328457	Cfhr2
17548730	4,619948	4,615957	4,617911	4,455618	4,55056	4,521117	4,6179387	4,509098333	-0,10884033	0,927333171	---
17418945	7,230802	6,942641	7,215548	6,918199	7,044506	7,099809	7,1296637	7,020838	-0,10882567	0,927342599	Fndc5
17469280	4,037058	3,359447	3,529671	3,819382	3,381381	3,398947	3,6420587	3,533236667	-0,108822	0,927344955	---
17391233	8,363381	8,560973	8,430912	8,262239	8,524122	8,242455	8,4517553	8,342938667	-0,10881667	0,927348384	Itpripl1
17345926	6,240228	6,124465	6,177514	6,084538	6,02567	6,105551	6,1807357	6,071919667	-0,108816	0,927348812	Satb1
17294680	6,109194	6,135231	5,939923	6,01221	6,071578	5,774119	6,0614493	5,952635667	-0,10881367	0,927350312	Mir3961 // Mir3961
17505239	8,437447	8,475127	8,440687	8,283002	8,42162	8,322249	8,451087	8,342290333	-0,10879667	0,927361239	Nip7
17305133	5,395744	5,197969	5,120732	5,164862	5,213549	5,009706	5,2381483	5,129372333	-0,108776	0,927374524	Rgr
17281202	8,073922	7,995978	7,852165	7,627829	7,956509	8,011412	7,9740217	7,86525	-0,10877167	0,92737731	Ppp2r3c
17414866	3,981426	3,356858	3,548304	3,488358	3,709026	3,362971	3,6288627	3,520118333	-0,10874433	0,92739488	---
17345186	10,3743	10,41869	10,30281	10,18035	10,33709	10,25217	10,365267	10,25653667	-0,10873	0,927404094	Hsp90ab1
17220604	7,74143	7,997303	7,985825	7,82451	7,837116	7,73686	7,908186	7,799495333	-0,10869067	0,927429379	1700007P06Rik // 1700007P06Rik
17324667	6,790361	6,285836	5,814138	7,08946	4,982644	6,492204	6,2967783	6,188102667	-0,10867567	0,927439021	---
17341205	8,082956	8,131391	7,951023	7,862612	8,007977	7,968788	8,0551233	7,946459	-0,10866433	0,927446307	Tbp // Tbp
17316493	8,037975	8,460511	8,523414	8,230037	8,17706	8,288816	8,3406333	8,231971	-0,10866233	0,927447593	---
17349764	8,712723	8,8368	8,556347	8,607509	8,60351	8,568864	8,7019567	8,593294333	-0,10866233	0,927447593	Wdr55
17493912	3,189363	3,223498	3,386153	3,164709	2,904524	3,403799	3,266338	3,157677333	-0,10866067	0,927448664	Art2b
17334123	4,319926	4,043418	4,480456	4,343854	4,059297	4,114762	4,2812667	4,172637667	-0,108629	0,927469021	---
17308916	8,631882	8,569329	8,450158	8,440475	8,337834	8,547207	8,5504563	8,441838667	-0,10861767	0,927476307	Wbp4
17387788	3,952084	3,556171	3,722015	3,620083	3,676684	3,607658	3,7434233	3,634808333	-0,108615	0,927478022	Olfr1157
17365198	9,431396	9,902525	9,624354	9,538507	9,479033	9,614911	9,6527583	9,544150333	-0,108608	0,927482522	---
17287372	4,673696	4,778067	5,109263	4,93063	4,63188	4,672703	4,8536753	4,745071	-0,10860433	0,927484879	4921525O09Rik
17218694	5,021377	4,930493	5,204397	5,034336	4,817632	4,97853	5,052089	4,943499333	-0,10858967	0,927494308	Ankrd45
17397679	4,757139	5,057436	4,986031	4,888436	4,768344	4,81813	4,9335353	4,82497	-0,10856533	0,927509952	A730037C10Rik
17237077	2,971101	3,034415	3,196425	3,135672	2,938844	2,80176	3,0673137	2,958758667	-0,108555	0,927516595	---
17427462	8,173336	8,142965	7,871622	7,984751	8,043967	7,833543	8,062641	7,954087	-0,108554	0,927517238	Tm2d1
17479006	8,932319	8,993419	9,09339	8,967031	8,994408	8,732079	9,006376	8,897839333	-0,10853667	0,927528382	Chsy1
17440117	6,762073	6,751312	6,496271	6,652812	6,699833	6,331447	6,6698853	6,561364	-0,10852133	0,92753824	Rpl5
17309802	8,61653	8,39316	8,513271	8,292243	8,476851	8,428337	8,5076537	8,399143667	-0,10851	0,927545526	Oxct1
17415421	3,661905	3,522982	3,619815	3,459036	3,556257	3,46388	3,6015673	3,493057667	-0,10850967	0,927545741	---
17509851	7,083729	6,924792	7,28789	6,937757	7,255517	6,77762	7,0988037	6,990298	-0,10850567	0,927548312	Zfp869
17498360	5,57306	5,673537	6,096439	5,937396	5,447949	5,632229	5,781012	5,672524667	-0,10848733	0,9275601	Mrgprg
17445869	8,826301	9,043066	9,00239	8,885442	8,78034	8,880525	8,9572523	8,848769	-0,10848333	0,927562671	Dnajc2
17372833	2,797203	2,930218	2,874088	2,820965	2,815713	2,639422	2,8671697	2,7587	-0,10846967	0,927571458	Olfr1118
17508168	4,0724	3,796944	4,236798	4,050642	3,742797	3,98735	4,0353807	3,926929667	-0,108451	0,92758346	---
17377344	6,070531	5,89725	6,475003	6,075198	6,097581	5,944737	6,1475947	6,039172	-0,10842267	0,927601677	Sstr4
17366629	2,924045	2,888172	3,03124	2,883016	2,806699	2,828477	2,947819	2,839397333	-0,10842167	0,92760232	---
17511677	11,21255	11,54613	11,26015	11,33417	11,10755	11,25188	11,33961	11,2312	-0,10841	0,927609821	Ces1c
17381302	7,737327	7,533357	7,04559	6,994958	7,238992	7,757107	7,438758	7,330352333	-0,10840567	0,927612607	Phyh // Phyh
17252405	11,28268	11,22569	11,19967	11,1344	10,91529	11,33315	11,236013	11,12761333	-0,1084	0,927616251	---
17537962	5,925373	5,900127	6,28719	6,034656	5,98723	5,765631	6,0375633	5,929172333	-0,108391	0,927622038	Plp1
17201633	4,135568	3,98424	3,616798	3,697683	3,842751	3,871048	3,912202	3,803827333	-0,10837467	0,92763254	---
17372294	8,458736	8,379961	8,317333	8,201614	8,202566	8,426794	8,3853433	8,276991333	-0,108352	0,927647114	Ube2e3
17386444	3,991832	4,064626	4,183804	3,936479	4,00877	3,969959	4,0800873	3,971736	-0,10835133	0,927647543	---
17448335	10,38828	10,496	10,40761	10,33271	10,29667	10,33747	10,43063	10,32228333	-0,10834667	0,927650544	Pds5a
17523659	5,284374	5,43469	5,162848	4,978212	5,1854	5,393303	5,2939707	5,185638333	-0,10833233	0,92765976	Ccr5
17450950	6,030605	5,821033	5,859201	5,73124	5,839297	5,815318	5,903613	5,795285	-0,108328	0,927662546	AB010352 // AB010352
17265277	5,626417	5,257062	5,695973	5,451326	5,448574	5,354628	5,526484	5,418176	-0,108308	0,927675407	Vmo1
17205155	5,866257	6,593151	6,516613	6,635403	5,963193	6,052551	6,3253403	6,217049	-0,10829133	0,927686124	---
17209345	5,866257	6,593151	6,516613	6,635403	5,963193	6,052551	6,3253403	6,217049	-0,10829133	0,927686124	---
17527988	6,855896	6,684398	6,826934	6,722238	6,69397	6,626163	6,789076	6,680790333	-0,10828567	0,927689767	Spesp1
17376599	4,158224	4,205305	4,123828	3,991484	4,060739	4,110693	4,1624523	4,054172	-0,10828033	0,927693197	AU019990 // AU019990
17281106	3,03427	3,458537	3,145827	3,141406	3,043437	3,128995	3,212878	3,104612667	-0,10826533	0,927702842	---
17534752	3,892619	3,806405	3,970835	3,590747	4,034732	3,719653	3,889953	3,781710667	-0,10824233	0,927717632	4933416I08Rik
17402146	9,089615	9,257749	9,039251	8,948764	9,149789	8,963344	9,1288717	9,020632333	-0,10823933	0,927719561	Alg14
17374743	6,039168	5,810074	5,832081	5,778111	5,908907	5,669669	5,8937743	5,785562333	-0,108212	0,927737138	Gm14137
17300155	3,14584	2,907622	3,005938	2,848976	2,97514	2,91068	3,0198	2,911598667	-0,10820133	0,927743997	Trav7-4 // Trav7-4
17248747	5,290232	5,238038	5,269558	5,023507	5,288813	5,160915	5,2659427	5,157745	-0,10819767	0,927746355	Sox30
17207957	5,918914	5,798348	6,311194	6,072492	5,821217	5,810259	6,0094853	5,901322667	-0,10816267	0,927768863	---
17260603	4,413597	4,611941	4,397758	4,489853	4,333478	4,275571	4,474432	4,366300667	-0,10813133	0,927789013	Zpbp
17342011	8,124828	8,570572	8,680307	8,560317	8,185151	8,305919	8,458569	8,350462333	-0,10810667	0,927804876	---
17487562	3,978309	3,981643	3,904094	3,849324	3,635073	4,055331	3,954682	3,846576	-0,108106	0,927805305	Vmn1r121
17388974	7,958276	8,095807	7,750125	7,8131	7,618827	8,048063	7,934736	7,826663333	-0,10807267	0,927826742	Depdc7
17444806	5,541722	5,396961	5,388428	5,44844	5,192005	5,362451	5,4423703	5,334298667	-0,10807167	0,927827385	Rasl11a
17413570	4,066091	4,483144	4,357869	4,015921	4,278426	4,288548	4,302368	4,194298333	-0,10806967	0,927828671	---
17311839	2,910545	2,952328	2,973156	2,699018	2,902277	2,910545	2,945343	2,83728	-0,108063	0,927832959	---
17434310	9,32509	9,357965	9,288147	9,237432	9,153606	9,255983	9,323734	9,215673667	-0,10806033	0,927834674	Fam133b
17425010	5,428666	5,407444	5,468585	5,348171	5,270427	5,36193	5,4348983	5,326842667	-0,10805567	0,927837675	Foxe1
17239102	5,249835	5,462746	5,187831	5,050769	5,21895	5,306676	5,3001373	5,192131667	-0,10800567	0,927869832	Ust
17534615	5,287552	5,405519	5,209913	5,446325	5,012091	5,120679	5,3009947	5,193031667	-0,107963	0,927897273	Rab33a
17229277	5,839811	5,527291	5,84271	5,519636	5,36541	6,000905	5,736604	5,628650333	-0,10795367	0,927903276	Rcsd1
17421764	4,161294	3,449471	3,747909	3,579344	4,056084	3,399397	3,7862247	3,678275	-0,10794967	0,927905849	---
17361703	6,851391	6,753542	6,847321	6,749631	6,546748	6,832334	6,817418	6,709571	-0,107847	0,927971884	Pcnxl3
17240661	4,111008	3,676845	3,701436	3,705522	3,752798	3,707447	3,829763	3,721922333	-0,10784067	0,927975958	---
17233708	6,873529	6,910423	6,909154	6,803337	6,994453	6,571852	6,897702	6,789880667	-0,10782133	0,927988394	Lrrc20
17397936	11,20673	11,17707	11,15991	11,05204	11,10698	11,06125	11,181237	11,07342333	-0,10781333	0,927993539	Mbnl1
17312675	5,316313	5,402437	5,154324	5,51079	4,759041	5,279807	5,2910247	5,183212667	-0,107812	0,927994397	Gm8221
17355391	8,533136	8,727394	8,589711	8,474833	8,547842	8,504165	8,616747	8,508946667	-0,10780033	0,928001902	Me2
17324859	6,206485	6,09139	6,140062	6,074443	6,001966	6,038159	6,145979	6,038189333	-0,10778967	0,928008763	Tnk2
17541705	4,504254	4,075754	4,318178	4,071261	4,229939	4,273619	4,2993953	4,191606333	-0,107789	0,928009192	Kis2
17462905	10,95622	10,84157	10,9174	10,79405	10,7473	10,85053	10,905063	10,79729333	-0,10777	0,928021413	Lpcat3
17213473	4,503623	4,346652	4,699487	4,71753	4,202916	4,306008	4,5165873	4,408818	-0,10776933	0,928021842	---
17410905	3,253162	3,480968	3,440889	3,255645	3,066822	3,529259	3,391673	3,283908667	-0,10776433	0,928025059	AI747448
17477766	5,095069	5,285119	5,21703	5,108408	5,018953	5,146569	5,1990727	5,09131	-0,10776267	0,928026131	1700039E15Rik
17510578	8,329525	8,483315	8,266486	8,265306	8,239305	8,251436	8,3597753	8,252015667	-0,10775967	0,92802806	9130011J15Rik
17414032	4,164105	4,587949	4,554169	4,297466	4,607682	4,077806	4,4354077	4,327651333	-0,10775633	0,928030205	Murc
17308965	8,661598	8,718593	8,660992	8,624948	8,431966	8,661027	8,6803943	8,572647	-0,10774733	0,928035994	Diap3
17532078	4,988701	4,60103	4,936494	4,73737	4,759042	4,706592	4,842075	4,734334667	-0,10774033	0,928040497	Slc22a14
17202927	4,498181	4,970591	4,401837	4,406516	4,76909	4,371809	4,6235363	4,515805	-0,10773133	0,928046286	---
17400177	7,532641	7,488311	7,444204	7,316751	7,449611	7,375617	7,4883853	7,380659667	-0,10772567	0,928049932	Rfx5
17372021	4,06424	4,154315	4,246659	4,1135	4,107152	3,921404	4,1550713	4,047352	-0,10771933	0,928054006	Chn1 // Chn1
17428749	6,291837	5,825529	6,196754	5,804162	6,201761	6,175101	6,1047067	5,997008	-0,10769867	0,9280673	1700021J08Rik
17353985	9,699246	9,710896	9,710454	9,664188	9,558807	9,57453	9,7068653	9,599175	-0,10769033	0,928072661	Nr3c1
17402406	4,080904	4,166285	4,369619	4,18205	4,112525	3,999182	4,2056027	4,097919	-0,10768367	0,928076949	---
17534746	5,567452	5,477956	5,435962	5,279182	5,442228	5,436938	5,49379	5,386116	-0,107674	0,928083168	Cxx1c
17444756	3,626761	3,869228	3,416054	3,599711	3,599711	3,389632	3,6373477	3,529684667	-0,107663	0,928090244	4930449I24Rik
17215982	3,918926	3,838372	4,950632	4,256736	4,129606	3,998619	4,2359767	4,128320333	-0,10765633	0,928094533	---
17514087	7,38885	7,280287	7,193981	7,293357	7,088925	7,157909	7,287706	7,180063667	-0,10764233	0,928103539	2310022B05Rik
17273973	5,056571	5,007532	5,612539	5,034284	5,140825	5,178758	5,2255473	5,117955667	-0,10759167	0,928136134	Matn3
17376212	5,351264	5,314012	5,393782	5,272747	5,238675	5,224904	5,3530193	5,245442	-0,10757733	0,928145356	Tgm6
17340737	4,204302	4,168159	4,156435	3,84513	4,356314	4,004745	4,1762987	4,068729667	-0,107569	0,928150717	Gm16702
17283216	6,36733	6,435887	6,335536	6,276739	6,17099	6,368342	6,3795843	6,272023667	-0,10756067	0,928156078	2610021K21Rik
17329904	4,597298	4,4424	4,076334	4,22581	4,3716	4,195994	4,3720107	4,264468	-0,10754267	0,928167658	---
17244572	8,183331	8,264774	8,112521	7,992717	8,039798	8,205492	8,1868753	8,079335667	-0,10753967	0,928169588	Tmtc3
17217846	4,859235	4,308594	3,919355	3,766502	4,694662	4,303425	4,3623947	4,254863	-0,10753167	0,928174735	Mir181b-1
17380612	5,252695	5,231911	5,571716	5,134238	5,323324	5,276218	5,3521073	5,244593333	-0,107514	0,928186101	Cdh26
17219171	5,330173	5,368896	4,793486	5,163302	5,177566	4,829165	5,164185	5,056677667	-0,10750733	0,928190391	Sh2d1b1
17334052	3,024926	3,090239	2,942413	2,822377	2,973156	2,939525	3,0191927	2,911686	-0,10750667	0,928190819	Vmn2r116
17438528	2,984808	3,088166	3,01939	2,997401	2,666326	3,106149	3,030788	2,923292	-0,107496	0,928197682	---
17241294	6,213885	6,127339	6,423947	6,256812	6,016369	6,169524	6,255057	6,147568333	-0,10748867	0,9282024	2010107G23Rik
17389844	4,871225	4,94616	5,218637	4,622585	5,121243	4,969738	5,0120073	4,904522	-0,10748533	0,928204545	Rhov
17387670	5,068824	5,09705	4,607865	5,063402	4,488059	4,899888	4,9245797	4,817116333	-0,10746333	0,928218699	Olfr1057
17495307	8,35778	8,704162	8,347087	8,422009	8,271404	8,393318	8,4696763	8,362243667	-0,10743267	0,92823843	---
17497895	5,883429	5,756662	5,947854	5,840867	5,72624	5,698553	5,8626483	5,75522	-0,10742833	0,928241218	Cend1
17397129	5,143714	5,042513	5,183977	5,046911	4,89662	5,104393	5,1234013	5,015974667	-0,10742667	0,928242291	Fgf2
17226194	8,60119	8,676705	8,652276	8,531273	8,48448	8,592256	8,6433903	8,536003	-0,10738733	0,928267598	Ralb
17342529	4,869648	4,962565	4,605374	4,8003	4,696424	4,618712	4,812529	4,705145333	-0,10738367	0,928269958	Nhlrc4
17257035	6,168033	6,050077	6,104945	6,010078	5,994249	5,9966	6,107685	6,000309	-0,107376	0,928274891	Eftud2
17257036	6,168033	6,050077	6,104945	6,010078	5,994249	5,9966	6,107685	6,000309	-0,107376	0,928274891	---
17293089	9,900075	9,893149	9,826169	9,717699	9,768425	9,811141	9,873131	9,765755	-0,107376	0,928274891	Ubqln1
17468523	5,290425	5,103727	5,402457	5,116279	5,974602	4,383676	5,2655363	5,158185667	-0,10735067	0,928291191	---
17326725	5,896562	5,613625	5,635813	5,532105	5,650034	5,641852	5,7153333	5,607997	-0,10733633	0,928300414	4930478L05Rik
17536933	6,664476	6,448824	6,67744	6,613554	6,43476	6,420421	6,5969133	6,489578333	-0,107335	0,928301272	Dmrtc1c1
17543732	6,664476	6,448824	6,67744	6,613554	6,43476	6,420421	6,5969133	6,489578333	-0,107335	0,928301272	Dmrtc1c1
17405672	11,13956	11,17293	11,13431	11,16277	10,69534	11,26673	11,148933	11,04161333	-0,10732	0,928310924	---
17351446	6,863667	6,947371	6,873719	6,79705	6,837403	6,728379	6,894919	6,787610667	-0,10730833	0,928318431	Impa2
17205629	4,737378	3,96008	4,28364	3,871773	4,701957	4,085459	4,3270327	4,219729667	-0,107303	0,928321862	---
17443065	3,941253	4,122285	3,901571	3,824565	4,000542	3,818099	3,9883697	3,881068667	-0,107301	0,928323149	---

17258765	5,07332	4,963397	5,222051	5,067591	4,750846	5,118512	5,086256	4,978983	-0,107273	0,928341166	---
17407481	4,378758	4,440678	4,694817	4,458738	4,442893	4,290832	4,504751	4,397487667	-0,10726333	0,928347387	Lce1l
17505478	12,18406	12,04919	11,96177	12,10713	11,7349	12,03122	12,065007	11,95775	-0,10725667	0,928351677	Tat
17339039	3,023598	3,067397	2,960146	2,762011	2,971627	2,995746	3,017047	2,909794667	-0,10725233	0,928354465	---
17475175	6,648971	6,135862	6,275995	6,229296	6,439066	6,070749	6,3536093	6,246370333	-0,107239	0,928363045	D930028M14Rik
17292172	3,188881	3,108148	3,106457	3,051563	2,96614	3,06409	3,1344953	3,027264333	-0,107231	0,928368193	---
17231477	7,891895	7,63713	7,476908	7,57588	7,621955	7,486427	7,6686443	7,561420667	-0,10722367	0,928372912	Myct1
17281963	6,418668	6,35277	6,43024	6,259404	6,406604	6,214046	6,4005593	6,293351333	-0,107208	0,928382993	Wdr89
17275832	7,815221	7,913047	7,782632	7,744021	7,700475	7,744836	7,8369667	7,729777333	-0,10718933	0,928395006	Fancm
17202243	7,027603	6,941258	6,601082	6,843035	6,710984	6,694377	6,8566477	6,749465333	-0,10718233	0,92839951	---
17528541	5,37046	5,408458	5,620943	5,477058	5,169853	5,431418	5,4666203	5,359443	-0,10717733	0,928402728	4930502A04Rik
17414061	7,675835	7,54276	7,349338	7,34492	7,491951	7,409548	7,5226443	7,415473	-0,10717133	0,928406589	Zfp189
17478785	5,443295	5,029801	4,923024	5,146382	5,07875	4,849545	5,13204	5,024892333	-0,10714767	0,928421819	Gm7367
17298460	7,482125	7,653518	7,566168	7,534597	7,501327	7,344562	7,5672703	7,460162	-0,10710833	0,928447132	Btd
17283641	7,266572	7,72791	7,574898	7,340917	7,573636	7,333539	7,5231267	7,416030667	-0,107096	0,928455069	Serpina1e
17468891	3,642571	3,433177	3,276226	3,471828	3,14384	3,415033	3,450658	3,343567	-0,107091	0,928458287	Vmn1r53
17220762	9,497205	9,626645	9,78136	9,691525	9,20765	9,68481	9,63507	9,527995	-0,107075	0,928468584	---
17311504	9,775613	9,670056	9,581786	9,622773	9,467353	9,616204	9,6758183	9,568776667	-0,10704167	0,928490036	Colec10
17471440	3,613855	3,86176	4,291613	3,683324	3,868343	3,894473	3,9224093	3,81538	-0,10702933	0,928497974	---
17221244	12,16329	12,10632	12,13988	12,19069	11,94078	11,95695	12,136497	12,02947333	-0,10702333	0,928501835	---
17549548	6,80777	6,995454	7,07765	6,526237	6,711923	7,321671	6,9602913	6,853277	-0,10701433	0,928507627	---
17549704	6,80777	6,995454	7,07765	6,526237	6,711923	7,321671	6,9602913	6,853277	-0,10701433	0,928507627	---
17498041	6,500498	6,702759	6,476084	6,183599	6,410049	6,764805	6,5597803	6,452817667	-0,10696267	0,92854088	Dusp8
17342761	8,220084	8,153233	8,401001	8,227641	8,206113	8,019689	8,258106	8,151147667	-0,10695833	0,928543669	Rps10
17399010	4,951177	4,984193	5,090901	4,940432	5,101926	4,663083	5,008757	4,901813667	-0,10694333	0,928553324	Mir9-1
17446022	3,187317	3,150026	3,277964	3,071916	3,277512	2,94505	3,2051023	3,098159333	-0,106943	0,928553538	---
17547789	5,843602	5,432921	5,45616	5,481999	5,396281	5,53361	5,577561	5,47063	-0,106931	0,928561262	---
17285486	4,211052	4,205415	4,210738	4,443655	3,697587	4,165242	4,2090683	4,102161333	-0,106907	0,928576709	---
17339501	6,139823	6,173175	6,205312	6,017485	6,159374	6,020738	6,17277	6,065865667	-0,10690433	0,928578425	---
17306186	4,25385	4,061129	4,46966	3,807994	4,441361	4,214606	4,2615463	4,154653667	-0,10689267	0,928585934	Olfr221
17356672	4,977567	4,814224	5,092801	4,749156	4,828264	4,986514	4,9615307	4,854644667	-0,106886	0,928590225	1700123I01Rik
17295054	6,231712	5,939481	6,198839	5,844727	6,014713	6,189958	6,123344	6,016466	-0,106878	0,928595375	Gm9776
17545618	5,778775	5,611373	5,672624	5,488678	5,48531	5,768179	5,6875907	5,580722333	-0,10686833	0,928601597	Ptchd1
17548771	5,468776	4,928658	5,25291	5,177948	5,052361	5,099432	5,2167813	5,109913667	-0,10686767	0,928602026	Gm19654
17403735	5,390718	5,018376	5,162792	4,961348	5,212882	5,077076	5,1906287	5,083768667	-0,10686	0,928606961	Asb17
17311733	4,696775	4,768513	4,491595	4,537478	4,582955	4,515878	4,6522943	4,545437	-0,10685733	0,928608677	Fer1l6
17207635	6,43289	6,660059	6,825987	6,458887	6,860933	6,278575	6,6396453	6,532798333	-0,106847	0,928615328	---
17388590	3,078364	3,070695	3,010071	3,09949	3,018916	2,720233	3,0530433	2,946213	-0,10683033	0,928626056	---
17205957	5,174446	4,692897	4,678152	4,872213	4,707318	4,645483	4,8484983	4,741671333	-0,106827	0,928628202	---
17269974	5,018852	4,907724	5,207127	4,91762	4,956366	4,939264	5,0445677	4,93775	-0,10681767	0,928634209	Meox1
17292507	3,403546	3,267359	3,306624	3,109683	3,21581	3,331656	3,325843	3,219049667	-0,10679333	0,928649872	---
17488073	8,607582	9,150546	8,90946	8,897609	8,793134	8,656466	8,889196	8,782403	-0,106793	0,928650087	---
17459504	7,473437	7,344314	7,532805	7,230254	7,455611	7,344314	7,4501853	7,343393	-0,10679233	0,928650516	Chmp3
17317056	10,1481	10,53121	10,51187	10,53027	10,20857	10,13198	10,39706	10,29027333	-0,10678667	0,928654164	Enpp2
17496945	4,163197	4,18953	4,147909	4,067748	4,336076	3,776546	4,1668787	4,060123333	-0,10675533	0,928674333	---
17324959	10,34175	10,78493	10,69547	10,26861	10,64188	10,59148	10,607383	10,50065667	-0,10672667	0,928692786	---
17290999	12,89066	12,9418	12,99783	12,85776	12,80341	12,84898	12,94343	12,83671667	-0,10671333	0,928701369	Hist1h4k
17203151	3,3283	3,390981	3,270892	3,398798	3,208211	3,063027	3,3300577	3,223345333	-0,10671233	0,928702013	---
17278765	9,062342	9,305419	9,274508	9,022691	9,240896	9,058603	9,2140897	9,107396667	-0,106693	0,928714458	AF357426
17300071	4,38705	4,126593	5,010204	4,993151	4,317179	3,893457	4,507949	4,401262333	-0,10668667	0,928718535	---
17522235	8,1907	8,518823	8,361367	8,223328	8,267832	8,259686	8,3569633	8,250282	-0,10668133	0,928721968	Cdc25a
17292190	9,773199	9,899065	9,810597	9,652605	9,737408	9,772812	9,8276203	9,720941667	-0,10667867	0,928723685	Nup153
17425374	5,267437	4,953407	5,543405	5,287476	5,057168	5,099589	5,2547497	5,148077667	-0,106672	0,928727977	---
17491354	4,543987	3,956797	4,240764	4,214221	4,049227	4,158099	4,2471827	4,140515667	-0,106667	0,928731195	Mrgprb1
17288420	4,299799	4,155374	4,269866	4,23877	4,051318	4,115041	4,2416797	4,135043	-0,10663667	0,928750723	4933416O17Rik
17465856	7,111761	7,162343	7,266298	6,945922	7,092301	7,182428	7,180134	7,073550333	-0,10658367	0,928784843	Ptn
17344329	7,759893	7,250512	7,776947	7,571861	7,492977	7,402884	7,595784	7,489240667	-0,10654333	0,928810809	---
17295346	8,074482	8,392407	7,935282	7,821823	8,160846	8,099898	8,134057	8,027522333	-0,10653467	0,928816389	Utp15
17303830	9,391417	9,357889	9,306266	9,289326	9,136532	9,310132	9,3518573	9,24533	-0,10652733	0,92882111	Ppp3cb
17536339	6,563489	6,059676	5,623309	6,149982	6,018762	5,758152	6,082158	5,975632	-0,106526	0,928821968	Zxdb
17411145	2,8956	3,031758	3,078692	2,779874	2,981775	2,924832	3,0020167	2,895493667	-0,106523	0,9288239	Mir3963 // Mir3963
17354430	2,90082	3,641202	3,084473	3,041631	3,386051	2,879249	3,2088317	3,102310333	-0,10652133	0,928824973	---
17292390	5,590988	5,452378	5,911025	5,520267	5,515883	5,598689	5,6514637	5,544946333	-0,10651733	0,928827548	Wnk2
17428851	7,033663	7,699434	7,501348	7,866383	7,080606	6,967957	7,4114817	7,304982	-0,10649967	0,928838922	---
17339218	4,064679	3,949525	4,236004	4,016946	3,903086	4,010713	4,0834027	3,976915	-0,10648767	0,928846648	Gm9984
17288030	3,277915	3,313125	3,291275	3,26672	3,025286	3,270892	3,294105	3,187632667	-0,10647233	0,92885652	BC052688
17269652	5,331041	5,209099	5,436446	5,155176	5,34622	5,155796	5,3255287	5,219064	-0,10646467	0,928861456	Kcnh4
17221269	4,295041	4,077024	4,464422	3,921426	4,322167	4,273568	4,278829	4,172387	-0,106442	0,92887605	Cpa6
17525454	4,056541	4,015299	3,675822	3,914995	3,936276	3,577096	3,9158873	3,809455667	-0,10643167	0,928882703	9230110F15Rik
17307269	6,583332	6,548022	6,477376	6,530515	6,371288	6,387638	6,5362433	6,429813667	-0,10642967	0,928883991	Phf11
17206049	5,807251	5,32697	5,552238	5,106179	5,835508	5,425487	5,562153	5,455724667	-0,10642833	0,928884849	---
17448763	7,409576	6,415411	5,001981	6,285836	6,285836	5,936022	6,275656	6,169231333	-0,10642467	0,92888721	---
17308914	4,953001	4,920878	5,388349	4,953537	4,940986	5,048438	5,0874093	4,980987	-0,10642233	0,928888712	---
17346185	8,328796	8,42668	8,391702	8,210014	8,29684	8,321197	8,3823927	8,276017	-0,10637567	0,928918759	Dpp9
17280552	8,808399	8,939061	8,781486	8,606986	8,826653	8,776208	8,842982	8,736615667	-0,10636633	0,928924769	Bcap29
17407190	9,644179	9,938225	9,746387	9,715465	9,703514	9,590724	9,7762637	9,669901	-0,10636267	0,92892713	Hax1
17231733	7,375015	7,44684	7,318582	7,235433	7,359235	7,2267	7,3801457	7,273789333	-0,10635633	0,928931208	Adat2
17229923	4,523659	4,754322	4,898534	4,434517	4,802027	4,620973	4,725505	4,619172333	-0,10633267	0,928946447	4933439K11Rik
17234477	5,39326	5,512709	6,027914	5,672304	5,463351	5,479244	5,6446277	5,538299667	-0,106328	0,928949451	Slc5a4a
17341589	7,038384	6,911769	7,109211	6,739802	7,18161	6,819	7,019788	6,913470667	-0,10631733	0,92895632	Paqr4
17274190	5,13533	4,940223	5,252577	4,909853	5,124655	4,974793	5,1093767	5,003100333	-0,10627633	0,92898272	Gm5432
17304516	3,253861	3,148738	3,245415	3,138017	3,007149	3,184055	3,2160047	3,109740333	-0,10626433	0,928990447	---
17213880	8,942857	9,038313	8,984584	8,831058	8,970565	8,845392	8,9885847	8,882338333	-0,10624633	0,929002038	Rpe
17307046	6,962857	7,09454	6,776131	6,838339	6,850297	6,826225	6,9445093	6,838287	-0,10622233	0,929017493	Cenpj
17384533	7,173916	7,259884	7,060314	7,074411	7,110642	6,990436	7,1647047	7,058496333	-0,10620833	0,929026508	Lhx6
17206365	2,858603	3,088089	2,877658	3,000513	2,880181	2,625041	2,94145	2,835245	-0,106205	0,929028654	---
17355455	4,995413	4,910424	5,227943	4,89393	4,957533	4,963735	5,0445933	4,938399333	-0,106194	0,929035738	2010010A06Rik
17248821	3,860061	3,996096	3,951893	3,791	3,932766	3,765747	3,9360167	3,829837667	-0,106179	0,929045397	Gm12169
17239702	8,260183	8,191851	7,884762	7,858011	8,093257	8,067052	8,1122653	8,006106667	-0,10615867	0,929058491	Tbpl1
17255904	4,588496	4,770113	4,710753	4,560532	4,449961	4,740473	4,6897873	4,583655333	-0,106132	0,929075664	---
17208697	4,90828	5,221177	5,839297	5,310339	5,176016	5,164009	5,322918	5,216788	-0,10613	0,929076952	---
17343076	8,37892	8,750362	8,498596	8,041474	8,747896	8,520135	8,542626	8,436501667	-0,10612433	0,929080601	---
17277307	6,854613	6,954163	7,077254	6,863842	6,854555	6,849272	6,96201	6,855889667	-0,10612033	0,929083177	Fcf1
17303117	6,657763	6,383644	5,882008	6,270554	5,881545	6,452982	6,307805	6,201693667	-0,10611133	0,929088973	Gm3696
17209019	5,979878	6,401258	6,182482	6,209071	6,316387	5,719832	6,1878727	6,081763333	-0,10610933	0,929090261	---
17418223	4,203819	4,200871	4,307641	4,054204	4,22379	4,116084	4,2374437	4,131359333	-0,10608433	0,929106361	Rhbdl2
17229958	3,05327	3,240702	3,157765	2,918827	3,078619	3,136184	3,150579	3,044543333	-0,10603567	0,929137704	---
17417924	7,0738	6,76184	6,502848	7,049397	6,450257	6,520744	6,779496	6,673466	-0,10603	0,929141353	---
17397774	8,093657	8,294289	8,157418	8,072103	8,041745	8,113449	8,181788	8,075765667	-0,10602233	0,929146291	Tsc22d2
17240085	2,926757	2,850195	2,852469	2,778934	2,754791	2,777681	2,8764737	2,770468667	-0,106005	0,929157454	---
17549964	2,926757	2,850195	2,852469	2,778934	2,754791	2,777681	2,8764737	2,770468667	-0,106005	0,929157454	---
17443801	5,645142	6,565896	5,619384	5,513305	5,62755	6,371588	5,943474	5,837481	-0,105993	0,929165183	---
17346990	9,195205	9,278861	9,101325	9,006912	9,090347	9,160179	9,191797	9,085812667	-0,10598433	0,929170764	Smchd1
17529803	3,740397	4,014081	4,004862	3,914112	3,614303	3,913053	3,91978	3,813822667	-0,10595733	0,929188154	BC043934
17425770	7,478565	7,411079	7,24632	7,265856	7,24127	7,310992	7,3786547	7,272706	-0,10594867	0,929193736	Ptgr1
17400622	7,139822	7,15894	7,223825	7,123001	7,04281	7,038999	7,1741957	7,06827	-0,10592567	0,92920855	Lix1l
17262591	4,709001	4,777101	4,949472	4,761617	4,680805	4,675389	4,811858	4,705937	-0,105921	0,929211555	Ppp2ca // Ppp2ca
17414834	3,696884	3,694433	3,620902	3,319802	4,061192	3,313555	3,6707397	3,564849667	-0,10589	0,929231522	---
17547899	3,164682	3,235749	3,315839	3,160607	3,140945	3,097091	3,2387567	3,132881	-0,10587567	0,929240754	Gm17756
17237537	5,678521	5,289263	5,957445	5,856207	5,548185	5,203215	5,641743	5,535869	-0,105874	0,929241827	Lrrc10
17285446	8,567541	7,983063	8,17051	7,950802	8,107561	8,345276	8,2403713	8,134546333	-0,105825	0,929273389	---
17208295	7,364147	6,812769	7,603729	7,625805	7,10978	6,727586	7,260215	7,154390333	-0,10582467	0,929273604	---
17459196	4,367887	4,483196	4,661308	4,415349	4,413601	4,366025	4,5041303	4,398325	-0,10580533	0,929286057	Tnip3
17529793	9,64131	9,446516	9,36198	9,309785	9,313855	9,508757	9,4832687	9,377465667	-0,105803	0,92928756	Atp1b3
17321989	4,41446	4,693369	4,552935	4,154122	4,613177	4,576063	4,553588	4,447787333	-0,10580067	0,929289063	Krt74
17396056	10,1796	10,23496	10,21886	10,19943	10,02284	10,09375	10,21114	10,10534	-0,1058	0,929289492	Fabp5
17457709	4,470063	4,513021	4,712655	4,373277	4,401295	4,603773	4,5652463	4,459448333	-0,105798	0,92929078	---
17255485	5,882466	5,509145	5,935216	5,689547	5,711541	5,608365	5,775609	5,669817667	-0,10579133	0,929295075	---
17526968	3,507556	3,304893	3,409142	3,241734	3,192478	3,470053	3,407197	3,301421667	-0,10577533	0,929305381	4833427G06Rik
17226653	2,772282	3,029389	2,901003	2,843583	2,823622	2,718164	2,9008913	2,795123	-0,10576833	0,92930989	---
17519895	11,90062	12,02848	11,92293	11,85048	11,81142	11,87295	11,950677	11,84495	-0,10572667	0,92933673	Rps27a // Rps27a
17298057	9,128982	9,165482	9,090418	8,959249	8,994253	9,114237	9,128294	9,022579667	-0,10571433	0,929344675	D14Abb1e
17426979	5,65847	6,00954	5,514851	5,779305	5,533854	5,552561	5,7276203	5,621906667	-0,10571367	0,929345104	Scarna8
17476980	3,818706	3,241173	3,784548	3,486812	3,447057	3,593473	3,614809	3,509114	-0,105695	0,929357129	Vmn2r-ps54 // Vmn2r-ps54
17512023	6,735036	6,686345	6,910072	6,795835	6,5808	6,637734	6,777151	6,671456333	-0,10569467	0,929357343	Prss54
17209539	8,41492	8,695569	9,142987	8,608503	8,578368	8,749569	8,7511587	8,64548	-0,10567867	0,92936765	---
17289840	5,289989	5,181684	5,682289	5,153405	5,351808	5,331799	5,384654	5,279004	-0,10565	0,929386117	---
17358207	8,3692	8,582713	8,094861	7,940018	8,36746	8,422352	8,3489247	8,243276667	-0,105648	0,929387406	Gm19425
17291469	3,644483	3,682692	3,645848	3,529813	3,613721	3,512591	3,6576743	3,552041667	-0,10563267	0,929397284	Prl7a1
17298014	3,947312	4,018437	3,816176	3,94314	3,686464	3,835454	3,9273083	3,821686	-0,10562233	0,92940394	Hesx1
17344696	4,449078	4,473253	4,254553	4,343452	4,222286	4,294345	4,3922947	4,286694333	-0,10560033	0,929418113	---
17445268	10,24467	10,20424	10,15607	10,01724	10,12206	10,14896	10,20166	10,09608667	-0,10557333	0,929435507	Gatad1
17467316	8,843989	9,089605	8,897016	8,564787	9,063138	8,886017	8,9435367	8,837980667	-0,105556	0,929446674	Gadd45a
17449964	3,921018	3,897694	4,010257	3,858212	3,781209	3,872909	3,9429897	3,837443333	-0,10554633	0,929452902	Prkg2

17346932	5,610013	5,410775	5,554987	5,556035	5,403445	5,299664	5,5252583	5,419714667	-0,10554367	0,92945462	5031415H12Rik
17549780	5,242	4,434201	5,402471	5,150706	4,82937	4,782028	5,026224	4,920701333	-0,10552267	0,929468149	---
17491999	7,396276	7,392601	7,458939	7,280838	7,392704	7,257737	7,4159387	7,310426333	-0,10551233	0,929474807	Lrrc28
17466812	4,215365	4,435125	4,546287	4,477646	4,243151	4,159447	4,3989257	4,293414667	-0,105511	0,929475666	Hoxa3
17487560	3,640416	3,998782	3,404404	3,531103	3,570139	3,625832	3,6812007	3,575691333	-0,10550933	0,929476739	Vmn1r120
17203461	3,478914	3,405662	4,030622	3,345802	3,463333	3,789539	3,6383993	3,532891333	-0,105508	0,929477598	---
17313767	8,925223	9,052768	8,903984	8,788692	8,861017	8,915751	8,9606583	8,855153333	-0,105505	0,929479531	Nup50
17359562	2,945817	3,092518	3,16028	2,950284	3,08812	2,843719	3,066205	2,960707667	-0,10549733	0,929484471	---
17207855	4,298483	4,735309	4,674705	4,904302	4,213678	4,274052	4,569499	4,464010667	-0,10548833	0,929490269	---
17470846	5,819932	5,626829	5,863011	5,58089	5,690161	5,722276	5,769924	5,664442333	-0,10548167	0,929494564	Eno2
17425355	4,052003	4,188989	4,490662	4,021399	4,127689	4,266167	4,2438847	4,138418333	-0,10546633	0,929504443	4930412L05Rik
17323072	9,314619	9,280658	9,459521	9,358435	8,763449	9,616533	9,3515993	9,246139	-0,10546033	0,929508309	---
17348079	9,150943	9,330188	9,25958	9,092678	9,203539	9,128124	9,2469037	9,141447	-0,10545667	0,929510671	Cul2
17414408	4,539884	4,672178	4,66495	4,505831	4,484978	4,569881	4,6256707	4,52023	-0,10544067	0,92952098	Zkscan16
17507882	3,743124	4,066649	3,904146	3,58579	3,915811	3,896029	3,9046397	3,79921	-0,10542967	0,929528067	Gm15056
17285753	3,486654	3,356354	3,388719	3,319407	3,302419	3,293628	3,4105757	3,305151333	-0,10542433	0,929531504	Vmn1r195
17548250	5,079142	5,093735	5,235002	5,021011	5,056916	5,013715	5,1359597	5,030547333	-0,10541233	0,929539235	Gm19343
17498867	6,383504	6,383241	6,42797	6,201664	6,298716	6,37825	6,3982383	6,292876667	-0,10536167	0,929571881	Gm6410
17435455	5,269317	5,088382	5,33321	5,106634	5,163276	5,104938	5,230303	5,124949333	-0,10535367	0,929577035	2900005J15Rik
17310044	10,39236	10,35366	10,12975	10,24794	10,15668	10,15511	10,291923	10,18657667	-0,10534667	0,929581546	Lifr
17211365	3,007697	3,321683	2,949586	2,836167	3,186397	2,940379	3,0929887	2,987647667	-0,105341	0,929585197	Mir206
17550715	3,527459	3,332479	3,497678	3,227233	3,471741	3,342659	3,4525387	3,347211	-0,10532767	0,929593788	---
17384173	7,114248	7,322525	7,408638	6,658044	7,419899	7,451503	7,2818037	7,176482	-0,10532167	0,929597654	---
17448762	7,893939	8,461569	7,779315	7,593865	8,040866	8,184157	8,044941	7,939629333	-0,10531167	0,929604098	---
17290939	5,638633	5,805545	5,391397	5,323168	5,624167	5,572314	5,6118583	5,506549667	-0,10530867	0,929606031	Pgbd1
17218543	9,937636	9,975808	9,923123	9,82653	9,736061	9,958059	9,9455223	9,840216667	-0,10530567	0,929607964	Rfwd2
17219431	4,069645	3,683035	3,887695	3,971159	3,675895	3,677409	3,880125	3,774821	-0,105304	0,929609038	---
17358810	5,139402	5,112397	5,23376	4,852612	5,202819	5,114292	5,161853	5,056574333	-0,10527867	0,929625362	Ch25h
17541618	8,289884	8,628178	8,393601	8,303391	8,365221	8,327252	8,437221	8,331954667	-0,10526633	0,929633309	Mbnl3
17455608	7,440453	7,753129	7,665281	7,400398	7,675481	7,467227	7,619621	7,514368667	-0,10525233	0,92964233	Rfc3
17399823	11,86281	12,05602	11,91888	11,83951	11,91868	11,76381	11,945903	11,84066667	-0,10523667	0,929652425	S100a8
17212018	8,961382	9,188959	8,93662	8,895389	8,991571	8,884321	9,028987	8,923760333	-0,10522667	0,929658869	Eif5b
17345085	4,573885	4,523785	4,685696	4,613388	4,246777	4,607522	4,5944553	4,489229	-0,10522633	0,929659084	D730048J04Rik
17493358	7,965484	8,055282	7,713121	7,641957	7,890333	7,885937	7,9112957	7,806075667	-0,10522	0,929663165	4632434I11Rik
17295792	4,286531	3,795729	4,047757	4,094342	4,000248	3,71978	4,043339	3,938123333	-0,10521567	0,929665958	4922502H24Rik
17501686	3,918339	4,313985	3,947614	3,780133	4,027872	4,056342	4,0599793	3,954782333	-0,105197	0,929677986	---
17287667	5,459428	5,222204	5,286342	5,154649	5,04027	5,457472	5,322658	5,217463667	-0,10519433	0,929679705	Slc34a1
17410693	4,481371	4,05005	4,384467	4,124501	4,214063	4,26176	4,305296	4,200108	-0,105188	0,929683786	4930425O10Rik
17202203	3,267365	3,476068	3,068129	2,877619	3,439165	3,179247	3,2705207	3,165343667	-0,105177	0,929690875	---
17399645	6,227713	6,854794	6,366714	6,109516	6,346175	6,678075	6,4830737	6,377922	-0,10515167	0,9297072	---
17312374	7,829364	7,825422	7,943987	7,874155	7,661987	7,747229	7,8662577	7,761123667	-0,105134	0,929718585	Gpaa1
17478511	5,867309	5,77224	6,073271	5,893303	5,876335	5,627788	5,9042733	5,799142	-0,10513133	0,929720303	Siglech // Siglech
17532137	8,139696	8,466197	8,256468	8,052674	8,205013	8,289429	8,2874537	8,182372	-0,10508167	0,929752311	Myd88
17405675	10,22563	10,30514	10,49104	10,03437	10,55541	10,11681	10,340603	10,23553	-0,10507333	0,929757681	---
17539758	4,609766	4,471067	4,549392	4,184662	4,744779	4,385593	4,5434083	4,438344667	-0,10506367	0,929763911	Mycs
17529481	4,754674	4,451822	4,731837	4,688283	4,511011	4,423909	4,646111	4,541067667	-0,10504333	0,929777015	Tbx18
17284034	4,380761	4,174554	4,230205	3,931903	4,281364	4,257131	4,26184	4,156799333	-0,10504067	0,929778734	---
17389335	4,586685	4,918532	4,663659	4,777797	4,624262	4,451754	4,7229587	4,617937667	-0,105021	0,929791408	Chrm5
17454214	8,17675	8,25253	7,775902	7,971282	7,790555	8,128285	8,068394	7,963374	-0,10502	0,929792053	Gm4963
17254602	5,537791	5,804288	5,611819	5,44693	5,565447	5,626494	5,6512993	5,546290333	-0,105009	0,929799142	Tbx4
17429886	5,673791	5,614164	5,611411	5,57548	5,546034	5,462863	5,633122	5,528125667	-0,10499633	0,929807306	Maneal
17318907	10,20161	10,40356	10,42852	10,29108	10,24479	10,18294	10,344563	10,23960333	-0,10496	0,929830723	Eif3d
17354036	9,120055	9,312605	9,309301	9,18672	9,219653	9,020746	9,2473203	9,142373	-0,10494733	0,929838886	Lars
17212346	7,522426	7,69339	7,432259	7,336533	7,546942	7,44976	7,5493583	7,444411667	-0,10494667	0,929839316	AI597479
17352603	9,087078	9,268927	9,081642	8,931689	9,028143	9,163002	9,1458823	9,040944667	-0,10493767	0,929845117	Usp14
17253396	8,239727	8,181049	8,313569	8,208649	7,928226	8,282672	8,2447817	8,139849	-0,10493267	0,929848339	Phf12
17317388	6,451276	6,385168	6,804123	6,485493	6,380296	6,460149	6,5468557	6,441979333	-0,10487633	0,929884648	4933412E24Rik
17345859	4,256561	4,443102	4,2038	4,125665	4,275862	4,187364	4,3011543	4,196297	-0,10485733	0,929896895	---
17494216	3,427882	3,321555	3,348914	3,242239	3,257088	3,284467	3,366117	3,261264667	-0,10485233	0,929900117	Olfr67
17206565	3,395135	3,478801	3,866772	3,594048	3,464555	3,367604	3,580236	3,475402333	-0,10483367	0,929912149	---
17464175	4,353745	4,310643	4,433051	4,364225	4,148144	4,2706	4,365813	4,260989667	-0,10482333	0,92991881	D6Ertd474e
17391506	6,714496	6,565117	6,833967	6,389666	6,657082	6,752388	6,7045267	6,599712	-0,10481467	0,929924396	Zc3h6
17336396	5,715549	5,52052	5,51026	5,501894	5,525477	5,404622	5,5821097	5,477331	-0,10477867	0,929947601	H2-Oa
17340708	3,631144	3,523223	3,785887	3,526479	3,41031	3,689143	3,6467513	3,541977333	-0,104774	0,929950609	Gm8457 // Gm8457
17212724	6,023078	5,871241	5,960503	6,24305	5,753954	5,543542	5,9516073	5,846848667	-0,10475867	0,929960493	Stat4
17322793	8,308457	8,416538	8,216432	8,24757	8,187826	8,191778	8,313809	8,209058	-0,104751	0,929965435	1810013L24Rik
17376378	6,312313	6,457019	6,469301	6,365471	6,391876	6,16706	6,4128777	6,308135667	-0,104742	0,929971236	Itpa
17496989	9,433033	9,411617	9,130926	9,03546	9,36752	9,25839	9,325192	9,220456667	-0,10473533	0,929975534	Nsmce4a
17506571	6,208179	6,245684	6,207524	6,266076	5,937934	6,143197	6,2204623	6,115735667	-0,10472667	0,92998112	Zfp276
17326011	5,314891	4,845905	5,565915	4,883084	5,564598	4,964861	5,242237	5,137514333	-0,10472267	0,929983699	Dppa4
17236926	8,069671	8,009717	8,005103	7,911737	7,820243	8,038353	8,0281637	7,923444333	-0,10471933	0,929985848	Gm4301 // Gm4301
17549456	5,370783	5,523105	5,417554	5,385569	5,319243	5,292484	5,4371473	5,332432	-0,10471533	0,929988426	---
17467387	3,096915	3,021335	2,960983	2,859181	2,911892	2,994021	3,026411	2,921698	-0,104713	0,92998993	---
17230550	11,49106	11,39509	11,47287	11,22013	11,34229	11,48247	11,453007	11,34829667	-0,10471	0,929991864	Lbr
17416212	6,834697	6,868853	6,191796	6,294639	6,710443	6,57616	6,631782	6,527080667	-0,10470133	0,929997451	1700024P16Rik
17436457	9,094898	9,093636	9,084639	9,004571	8,878706	9,075812	9,0910577	8,986363	-0,10469467	0,930001748	Tacc3
17346812	9,706892	9,828338	9,395282	9,665191	9,363863	9,58743	9,643504	9,538828	-0,104676	0,930013781	Gm10495
17201293	5,365211	4,365458	4,951777	4,684021	4,884563	4,799852	4,8941487	4,789478667	-0,10467	0,930017649	---
17457836	4,083239	3,992699	4,189843	3,993848	3,867076	4,090875	4,0885937	3,983933	-0,10466067	0,930023666	Pip
17401563	4,798133	4,814289	4,850045	4,835969	4,859038	4,453555	4,8208223	4,716187333	-0,104635	0,930040212	Slc16a4
17459080	7,723193	7,489736	7,568398	7,517198	7,440637	7,509625	7,5937757	7,489153333	-0,10462233	0,930048377	Tigd2
17313645	6,646086	7,131722	6,636907	6,519001	6,740119	6,841766	6,804905	6,700295333	-0,10460967	0,930056543	---
17550036	6,646086	7,131722	6,636907	6,519001	6,740119	6,841766	6,804905	6,700295333	-0,10460967	0,930056543	---
17415006	5,15228	5,274873	5,572767	5,230752	5,420967	5,03441	5,3333067	5,228709667	-0,104597	0,930064709	---
17240581	5,153804	4,888589	5,116652	4,905135	4,932225	5,007939	5,053015	4,948433	-0,104582	0,930074379	---
17311157	9,29928	9,370813	9,206868	9,148301	9,155583	9,259377	9,2923203	9,187753667	-0,10456667	0,930084264	Atp6v1c1
17203099	7,739152	7,74774	7,659309	7,477134	7,558989	7,796448	7,7154003	7,610857	-0,10454333	0,930099307	---
17250460	8,699938	8,846331	8,884865	8,809857	8,629386	8,678286	8,810378	8,705843	-0,104535	0,93010468	Alkbh5
17328187	10,42973	10,60715	10,36963	10,31518	10,40591	10,37182	10,468837	10,36430333	-0,10453333	0,930105754	Rsl1d1
17409406	8,994074	8,888081	8,802029	8,764688	8,775802	8,830151	8,894728	8,790213667	-0,10451433	0,930118003	Stxbp3a
17252609	6,346176	6,119679	6,353508	6,15367	6,274049	6,078103	6,273121	6,168607333	-0,10451367	0,930118433	P2rx5
17319565	8,077559	8,074079	8,104091	8,00596	7,905859	8,030499	8,085243	7,980772667	-0,10447033	0,930146371	Pppde2
17485462	5,803145	5,502879	5,770919	5,793382	5,231255	5,738952	5,6923143	5,587863	-0,10445133	0,930158621	Mrgprf
17267626	5,357437	5,334416	5,241505	5,135987	5,23963	5,244417	5,3111193	5,206678	-0,10444133	0,930165068	Coil // Coil
17473744	6,986583	6,718989	6,94052	6,891009	6,820607	6,621222	6,8820307	6,777612667	-0,104418	0,930180112	Zfp606
17477266	4,563727	4,523294	4,984137	4,76498	4,615836	4,377107	4,690386	4,585974333	-0,10441167	0,930184196	Klk5
17366912	3,227726	3,420053	3,431875	3,25989	3,313407	3,193134	3,3598847	3,255477	-0,10440767	0,930186775	---
17367031	5,217365	5,067286	5,138751	5,122931	4,958947	5,028303	5,141134	5,036727	-0,104407	0,930187205	E030013I19Rik
17209193	6,613811	5,823962	6,587049	6,487426	5,789761	6,434427	6,3416073	6,237204667	-0,10440267	0,930189999	---
17278886	5,642334	5,684505	5,687571	5,674612	5,200919	5,825675	5,67147	5,567068667	-0,10440133	0,930190858	Mir409
17354157	9,107474	8,776815	7,837023	7,965771	8,539197	8,903214	8,5737707	8,469394	-0,10437667	0,930206763	Spink3
17395702	8,634568	8,871284	8,494827	8,476265	8,557373	8,653934	8,666893	8,562524	-0,104369	0,930211706	---
17428712	4,29752	4,104326	3,809623	3,959524	3,955553	3,983286	4,0704897	3,966121	-0,10436867	0,930211921	---
17436235	5,921963	5,882163	5,819391	5,964947	5,686507	5,659002	5,8745057	5,770152	-0,10435367	0,930221592	---
17331886	3,265833	3,184862	3,349724	3,17912	3,254437	3,05383	3,2668063	3,162462333	-0,104344	0,930227825	Krtap16-3
17368359	5,116817	5,061399	5,413452	4,88096	5,228582	5,169243	5,1972227	5,092928333	-0,10429433	0,93025985	---
17389216	3,269289	3,019189	3,237188	3,13752	2,953722	3,121567	3,175222	3,070936333	-0,10428567	0,930265438	Gm15130
17351404	4,147774	4,182883	4,445547	4,134051	4,192918	4,136405	4,2587347	4,154458	-0,10427667	0,930271242	Raxos1
17229007	8,654943	7,985492	7,950404	7,774998	8,263395	8,239623	8,1969463	8,092672	-0,10427433	0,930272746	Fmo4
17237522	4,11689	3,843372	4,035363	3,816613	4,006169	3,860046	3,9985417	3,894276	-0,10426567	0,930278335	Best3
17202783	4,437213	4,476918	4,608818	3,83761	4,407483	4,965117	4,5076497	4,403403333	-0,10424633	0,930290801	---
17487035	4,271259	4,267998	4,105472	4,007887	4,064236	4,259985	4,2149097	4,110702667	-0,104207	0,930316165	Psg18
17210393	7,282013	7,110059	7,109372	7,390373	6,557281	7,241198	7,167148	7,062950667	-0,10419733	0,930322399	---
17374198	4,080365	4,273994	4,478285	4,105808	4,094344	4,319942	4,277548	4,173364667	-0,10418333	0,930331426	Olfr1299
17329401	4,507201	4,663333	4,508241	4,403055	4,416858	4,546371	4,5595917	4,455428	-0,10416367	0,930344109	---
17225153	10,75694	11,04975	10,85352	10,76187	10,7748	10,81105	10,886737	10,78257333	-0,10416333	0,930344324	Ncl
17494643	6,685502	7,516817	6,464704	6,9275	6,680414	6,746629	6,8890077	6,784847667	-0,10416	0,930346473	Gvin1 // Gvin1
17548806	8,387222	8,610874	8,47449	8,417254	8,391125	8,351732	8,490862	8,386703667	-0,10415833	0,930347548	Polr2k // Polr2k
17358517	9,540421	9,62331	9,584537	9,385621	9,478229	9,572012	9,582756	9,478620667	-0,10413533	0,93036238	Jak2
17479454	4,510142	4,605342	4,581121	4,234941	4,543172	4,606126	4,565535	4,461413	-0,104122	0,930370979	Wdr93
17345666	9,961186	10,12236	9,922985	9,694923	9,9246	10,07465	10,002177	9,898057667	-0,10411933	0,930372698	---
17435639	3,562747	3,158399	3,211612	3,135862	3,141637	3,34295	3,3109193	3,206816333	-0,104103	0,930383231	---
17213185	10,38618	10,33886	10,23782	10,1585	10,23516	10,25699	10,320953	10,21688333	-0,10407	0,930404513	Cbx3 // Cbx3
17506040	4,020092	3,911655	4,229692	4,053227	3,869195	3,926828	4,053813	3,94975	-0,104063	0,930409027	---
17224146	4,423026	4,424719	4,646913	4,548554	4,460252	4,173665	4,4982193	4,394157	-0,10406233	0,930409457	
17525391	11,50093	11,68478	11,6966	11,54392	11,61735	11,40887	11,627437	11,52338	-0,10405667	0,930413112	Gm5614
17412335	3,713347	3,658449	3,578509	3,608323	3,481122	3,548721	3,6501017	3,546055333	-0,10404633	0,930419776	4930556G01Rik
17265908	3,141828	3,133177	2,99659	2,886386	3,216053	2,857136	3,0905317	2,986525	-0,10400667	0,930445358	---
17241801	4,127359	3,723348	3,842093	3,625305	4,109121	3,646377	3,8976	3,793601	-0,103999	0,930450303	A330049N07Rik
17503810	6,143482	6,325965	6,25282	6,146169	6,192313	6,07184	6,2407557	6,136774	-0,10398167	0,930461482	Irx5
17365461	5,227562	5,022491	5,266539	5,203247	5,020189	4,981217	5,1721973	5,068217667	-0,10397967	0,930462772	Calhm2
17367163	5,778974	5,967798	5,714551	5,688848	5,780746	5,67988	5,820441	5,716491333	-0,10394967	0,93048212	Cacnb2
17535902	4,229912	4,173177	4,0467	4,013668	4,186257	3,93802	4,1499297	4,045981667	-0,103948	0,930483195	---
17284566	3,198663	3,357805	3,018838	3,015576	3,224103	3,0238	3,1917687	3,087826333	-0,10394233	0,93048685	---

03/05/17

17513472	4,583029	4,55994	4,631073	4,526712	4,382324	4,553181	4,5913473	4,487405667	-0,10394167	0,93048728	Kcng4	
17510854	2,779803	2,905539	3,121994	2,80297	2,832775	2,859769	2,9357787	2,831838	-0,10394067	0,930487925	---	
17384499	4,394104	4,46021	4,697223	4,417305	4,289012	4,533451	4,517179	4,413256	-0,103923	0,930499319	---	
17294518	7,300543	7,538516	7,287136	7,359671	7,23223	7,222534	7,3753983	7,271478333	-0,10392	0,930501254	Ankrd32	
17259521	10,87647	11,25204	11,23323	10,87449	11,1176	11,05789	11,12058	11,01666	-0,10392	0,930501254	---	
17323816	3,499334	3,204322	3,313037	3,39272	3,094266	3,217973	3,3388977	3,234986333	-0,10391133	0,930506844	Gm10088	
17540555	4,356513	4,80003	4,949692	4,842481	4,401094	4,550947	4,7020783	4,598174	-0,10390433	0,930511359	Zfp182	
17524087	9,629712	9,841653	9,639184	9,585052	9,622303	9,591536	9,7035163	9,599630333	-0,103886	0,930523184	Ankrd49	
17437350	4,432772	4,25145	4,539226	4,326944	4,263012	4,321908	4,407816	4,303954667	-0,10386133	0,930539093	---	
17523427	4,517929	4,566323	4,483177	4,315988	4,428301	4,511559	4,5224763	4,418616	-0,10386033	0,930539738	9530059O14Rik	
17455656	4,367167	4,040339	4,142974	4,36598	3,999873	3,873099	4,1834933	4,079650667	-0,10384267	0,930551134	C87414 // C87414	
17302582	4,293993	4,382749	4,391091	4,426058	4,287548	4,042726	4,3559443	4,252110667	-0,10383367	0,930556939	Trim52	
17378799	6,692566	6,788297	6,51934	6,487334	6,661887	6,539523	6,6667343	6,562914667	-0,10381967	0,930565969	---	
17201063	9,570822	9,790889	9,326042	9,343595	9,096601	9,9361	9,5625843	9,458765333	-0,103819	0,930566399	---	
17456800	5,11709	5,001558	5,301396	4,945595	5,119349	5,043682	5,1400147	5,036208667	-0,103806	0,930574784	1700023L04Rik	
17227806	5,460344	5,045546	5,120239	5,009557	5,299373	5,005798	5,2087097	5,104909333	-0,10380033	0,930578439	---	
17453773	4,239894	4,162737	4,28522	4,213222	4,085812	4,07751	4,2292837	4,125514667	-0,103769	0,93059865	---	
17466606	7,149951	7,308577	7,070447	7,080295	6,965213	7,17217	7,176325	7,072559333	-0,10376567	0,930600801	Gimap6	
17384234	7,426178	7,445425	7,496857	7,465561	7,300159	7,291479	7,4561533	7,352399667	-0,10375367	0,930608541	Pbx3	
17218305	6,179559	6,062066	6,311911	6,00441	6,030564	6,207303	6,184512	6,080759	-0,103753	0,930608971	Rgs8	
17530107	6,053106	6,173133	6,359683	6,091998	5,943258	6,239441	6,1953073	6,091565667	-0,10374167	0,930616282	Sox14	
17357148	12,89926	13,09763	13,09852	12,98591	12,95212	12,84623	13,031803	12,92808667	-0,10371667	0,930632408	Snord22	
17534907	3,91904	4,216114	4,415287	4,233166	3,981006	4,02512	4,1834803	4,079764	-0,10371633	0,930632623	---	
17297777	5,347242	5,187124	5,299646	5,128352	5,151959	5,242597	5,278004	5,174302667	-0,10370133	0,930642299	Cphx	
17457044	5,382622	5,143934	5,24572	5,03259	5,151944	5,27669	5,2574253	5,153741333	-0,103684	0,930653481	---	
17202663	3,797064	3,705879	3,606823	3,673069	3,647717	3,477949	3,7032553	3,599578333	-0,103677	0,930657996	---	
17422138	6,266284	6,39064	6,319275	6,217864	6,204773	6,242535	6,3253997	6,221724	-0,10367567	0,930658856	Gpr153	
17311841	4,611984	4,45141	4,692233	4,411003	4,365657	4,667955	4,585209	4,481538333	-0,10367067	0,930662082	Fam84b	
17374470	7,378591	7,411598	7,250536	7,150167	7,282473	7,297149	7,3469083	7,243263	-0,10364533	0,930678424	Fam98b	
17261417	10,44816	10,59921	10,49272	10,30378	10,38381	10,54159	10,513363	10,40972667	-0,10363667	0,930684015	Chac2	
17298387	6,265592	6,007059	6,229094	5,995202	6,13034	6,065365	6,1672483	6,063635667	-0,10361267	0,930699497	Sema3g	
17348897	3,403979	3,538829	3,313358	3,359836	3,330875	3,254656	3,418722	3,315122333	-0,10359967	0,930707884	---	
17296943	6,556108	6,407523	5,71452	6,12173	5,780175	6,465502	6,2260503	6,122469	-0,10358133	0,930719711	---	
17291648	5,306769	5,197621	5,219002	5,130551	5,106547	5,175561	5,2411307	5,137553	-0,10357767	0,930722077	1700018A04Rik	
17312738	7,570947	7,64786	7,619729	7,511708	7,51086	7,505235	7,6128453	7,509267667	-0,10357767	0,930722077	Mpst	
17203025	6,632796	6,583869	5,996278	6,767313	6,554746	5,58025	6,4043143	6,300769667	-0,10354467	0,930743366	---	
17529175	10,84929	11,05766	10,9008	10,80503	10,81893	10,87329	10,935917	10,83241667	-0,1035	0,930772183	Tmem30a	
17373842	3,2826	3,129188	3,319604	3,2135	3,104744	3,102654	3,2437973	3,140299333	-0,103498	0,930773473	---	
17536519	9,863038	9,894338	9,887877	9,822387	9,786377	9,726022	9,881751	9,778262	-0,103489	0,93077928	Igbp1	
17506126	4,97197	4,670418	5,27765	4,821984	4,82434	4,963322	4,973346	4,869882	-0,103464	0,930795409	Adad2	
17282783	4,766371	4,395041	4,548585	4,562483	4,414045	4,423118	4,569999	4,466548667	-0,10345033	0,930804226	Zdhhc22	
17209993	3,460875	3,636889	3,920629	3,512155	3,665076	3,530835	3,6727977	3,569355333	-0,10344233	0,930809388	---	
17439560	5,733597	5,835045	5,592699	5,590036	5,621961	5,63908	5,720447	5,617025667	-0,10342133	0,930822937	---	
17218722	3,920649	4,148821	4,105948	4,22687	3,766322	3,871995	4,0584727	3,955062333	-0,10341033	0,930830034	2810442N19Rik	
17403224	9,263999	10,42299	9,015988	9,544012	9,444653	9,404099	9,567659	9,464254667	-0,10340433	0,930833905	Gbp7	
17298262	8,393228	8,692036	8,328861	8,149636	8,465343	8,488985	8,471375	8,367988	-0,103387	0,930845089	Mustn1	
17474361	3,253963	3,413514	3,848225	3,070963	3,64059	3,494044	3,505234	3,401865667	-0,10336833	0,930857133	Sycp1-ps1	
17204687	5,495785	5,930931	6,977017	5,927099	6,188177	5,978368	6,1345777	6,031214667	-0,103363	0,930860574	---	
17250471	7,583738	7,373779	7,951305	7,597501	7,469621	7,531612	7,636274	7,532911333	-0,10336267	0,930860789	---	
17294058	3,686229	3,53949	3,508362	3,548275	3,376436	3,499291	3,578027	3,474667333	-0,10335967	0,930862725	4930525G20Rik	
17547971	6,314292	6,371837	6,474432	6,323256	6,326781	6,200506	6,3868537	6,283514333	-0,10333933	0,930875845	Vps8 // Vps8	
17206259	6,035027	5,830373	6,265619	5,597919	5,709057	6,514077	6,043673	5,940351	-0,103322	0,930887029	---	
17206615	2,98194	2,909094	3,04468	2,855563	2,895955	2,874235	2,9785713	2,875251	-0,10332033	0,930888104	---	
17305590	4,545353	4,292486	4,30806	4,288003	4,280015	4,267956	4,3819663	4,278658	-0,10330833	0,930895847	Mir327 // Mir327	
17210027	6,095098	6,590835	5,922502	6,206333	6,004205	6,087986	6,2028117	6,099508	-0,10330367	0,930898858	---	
17397957	7,501148	7,540673	7,430314	7,350748	7,432833	7,378647	7,4907117	7,387409333	-0,10330233	0,930899719	P2ry1	
17223378	7,545445	7,681668	7,764475	7,462961	7,676579	7,542154	7,6638627	7,560564667	-0,103298	0,930902515	Ppil3	
17500694	5,896228	5,656346	5,684846	5,661458	5,577527	5,688557	5,7458067	5,642514	-0,10329267	0,930905956	Gm2085	
17210277	8,364128	7,627192	8,418537	8,246414	7,611563	8,242012	8,136619	8,033329667	-0,10328933	0,930908107	---	
17210299	8,364128	7,627192	8,418537	8,246414	7,611563	8,242012	8,136619	8,033329667	-0,10328933	0,930908107	---	
17357949	3,35198	3,433897	3,321272	3,225614	3,231149	3,340541	3,3690497	3,265768	-0,10328167	0,930913054	Olfr1505	
17394142	3,616783	3,71706	3,382853	3,380731	3,580243	3,445913	3,572232	3,468962333	-0,10326967	0,930920797	Svs3b	
17284803	4,004567	3,89786	3,64359	3,744689	3,863537	3,62804	3,8486723	3,745422	-0,10325033	0,930933272	D230030E09Rik	
17225980	3,347414	3,045002	3,330783	3,260672	3,291246	2,861593	3,2410663	3,137837	-0,10322933	0,930946823	---	
17394012	4,778184	4,834719	4,767178	4,725918	4,672941	4,671561	4,7933603	4,69014	-0,10322033	0,930952631	---	
17293808	6,374889	5,949672	5,739421	6,289825	5,849302	5,615233	6,0213273	5,91812	-0,10320733	0,930961019	---	
17358015	11,36419	11,09263	11,16233	10,93451	11,0556	11,31942	11,206383	11,10317667	-0,10320667	0,93096145	---	
17315975	5,047281	5,023429	4,95329	5,006488	4,627833	5,080075	5,008	4,904798667	-0,10320133	0,930964891	Rad1 // Rad1	
17408352	10,50675	10,60374	10,46061	10,46828	10,37834	10,4149	10,5237	10,42050667	-0,10319333	0,930970054	Hsd3b1	
17290394	5,595992	5,704894	5,595103	5,55718	5,531488	5,49783	5,6319963	5,528832667	-0,10316367	0,930989198	---	
17290396	5,595992	5,704894	5,595103	5,55718	5,531488	5,49783	5,6319963	5,528832667	-0,10316367	0,930989198	---	
17475197	3,744872	2,774894	3,694432	3,270892	3,694432	2,939525	3,4047327	3,301616333	-0,10311633	0,931019743	---	
17548573	6,400867	6,383378	6,398568	6,270854	6,323701	6,27894	6,394271	6,291165	-0,103106	0,931026411	4931406P16Rik // 4931406P16Rik	
17525680	4,21353	3,942901	4,06633	4,216799	3,911461	3,785184	4,0742537	3,971148	-0,10310567	0,931026627	Olfr151 // Olfr160	
17330773	7,816137	7,807566	7,68649	7,648148	7,656111	7,696707	7,7700643	7,666988667	-0,10307567	0,931045987	Pvrl3	
17522550	7,832367	8,010638	7,729806	7,86764	7,705057	7,690924	7,8576037	7,754540333	-0,10306333	0,931053946	---	
17226974	7,181143	7,136484	7,316338	7,035307	7,103713	7,18577	7,2113217	7,108263333	-0,10305833	0,931057173	Sox13	
17378808	4,446475	4,278553	4,680001	4,380171	4,42546	4,290323	4,468343	4,365318	-0,103025	0,931078685	Bpi	
17459455	11,82047	11,71125	11,81692	11,70109	11,6978	11,64069	11,78288	11,67986	-0,10302	0,931081912	Fabp1	
17417842	3,220229	2,993375	3,032318	2,935068	3,06961	2,932185	3,081974	2,978954333	-0,10301967	0,931082127	---	
17392138	7,043289	6,958846	7,048651	6,858439	6,882443	7,000862	7,0169287	6,913914667	-0,103014	0,931085784	Mkks	
17434675	4,756387	4,525972	4,91188	4,672564	4,857609	4,355032	4,731413	4,628401667	-0,10301133	0,931087505	---	
17380153	4,797826	4,847396	4,824086	4,610855	4,699131	4,850331	4,8231027	4,720105667	-0,102997	0,931096756	Tfap2c	
17371941	8,62588	8,723296	8,646482	8,557413	8,571742	8,557557	8,6652193	8,562237333	-0,102982	0,931106437	Cdca7	
17448813	5,479816	4,75062	4,396504	4,51472	5,224702	4,578575	4,8756467	4,772665667	-0,102981	0,931107082	---	
17379933	5,209654	5,32043	5,073331	5,149313	4,847892	5,297313	5,2011383	5,098172667	-0,10296567	0,931116978	---	
17463761	7,431815	7,537339	7,301471	7,575041	7,176394	7,210294	7,4235417	7,320576333	-0,10296533	0,931117193	8430419L09Rik	
17247148	4,726241	4,770438	4,791464	4,709339	4,672442	4,5975	4,7627143	4,659760333	-0,102954	0,931124508	---	
17276328	8,678284	8,544058	8,554236	8,460684	8,497948	8,509092	8,5921927	8,489241333	-0,10295133	0,931126229	Hif1a	
17307113	5,397482	5,15954	5,409951	5,31425	5,149914	5,193981	5,3223243	5,219381667	-0,10294267	0,931131823	Gja3	
17350740	9,011537	8,961992	8,904991	8,818187	8,852152	8,899355	8,9595067	8,856564667	-0,102942	0,931132253	Lmnb1	
17338701	7,152421	7,446508	7,442936	7,075565	7,304161	7,353339	7,3472883	7,244355	-0,10293333	0,931137847	Chaf1a	
17208153	3,521172	3,657048	3,754321	3,457966	3,744226	3,421568	3,6441803	3,541253333	-0,102927	0,931141934	---	
17471094	8,441748	8,318155	8,549446	8,207079	8,473	8,320518	8,4364497	8,333532333	-0,10291733	0,931148173	Ltbr	
17435660	7,793322	8,097797	8,014493	7,876235	7,817399	7,903285	7,9685373	7,865639667	-0,10289767	0,931160867	Nom1	
17547521	10,79788	11,0411	11,04467	10,91498	10,82861	10,83139	10,961217	10,85832667	-0,10289	0,931165815	Rps24 // Rps24	
17283345	9,398677	9,556867	9,383623	9,301558	9,28267	9,446297	9,446389	9,343508333	-0,10288067	0,931171839	Smek1	
17308714	6,767609	7,30028	6,744625	6,794836	6,876962	6,832075	6,9375047	6,834624333	-0,10288033	0,931172054	Slc25a30	
17459752	3,607683	3,854034	3,765472	3,700768	3,718778	3,499034	3,7423963	3,639526667	-0,10286967	0,931178939	Reg3a	
17240357	8,237321	8,480327	8,090898	8,062548	8,219574	8,217896	8,2695153	8,166672667	-0,10284267	0,931196366	Gtf3c6	
17550801	3,232663	3,173757	3,371756	3,10567	3,175861	3,188143	3,259392	3,156558	-0,102834	0,93120196	---	
17231742	6,515944	6,35321	6,208598	6,10429	6,290557	6,374546	6,3592507	6,256464333	-0,10278633	0,931232727	Hivep2	
17332344	7,36797	7,039158	7,441921	7,006842	7,414841	7,119167	7,2830163	7,180283333	-0,102733	0,931267154	---	
17469533	3,335212	3,309126	3,305176	3,226817	3,173676	3,240891	3,3165047	3,213794667	-0,10271	0,931282001	Gm19757	
17428424	4,685831	4,862688	5,123063	4,953405	4,714932	4,695235	4,8905273	4,787857333	-0,10267	0,931307821	Foxd2	
17465532	4,987889	4,458282	4,604255	4,672417	4,381765	4,688268	4,6834753	4,580816667	-0,10265867	0,931315138	---	
17412123	8,567287	8,70352	8,411241	8,293886	8,53012	8,550104	8,5606827	8,458036667	-0,102646	0,931323314	Ccnc	
17412294	2,956549	3,080174	2,96677	2,867223	2,950907	2,877434	3,0011643	2,898521333	-0,102643	0,931325251	---	
17424633	6,772218	6,950888	7,115116	6,856984	6,819127	6,854253	6,946074	6,843454667	-0,10261933	0,931340529	E130306D19Rik	
17278244	6,409768	5,926376	6,1426	5,749459	6,151644	6,269906	6,1595813	6,057003	-0,10257833	0,931366997	Serpina5	
17428354	3,328754	3,148316	3,036607	3,222224	3,092311	2,891426	3,1712257	3,068653667	-0,102572	0,931371086	Skint5	
17414114	10,088	10,10452	10,14909	9,994267	9,958918	10,08072	10,11387	10,01130167	-0,10256833	0,931373453	Smc2	
17535145	3,496977	3,551113	3,424541	3,423937	3,292254	3,448801	3,490877	3,388330667	-0,10254633	0,931387656	Gm4988	
17209105	6,076017	6,149662	6,491997	6,457307	5,810266	6,14248	6,2392253	6,136684333	-0,102541	0,931391099	---	
17323782	7,950678	7,990427	8,06739	7,861139	7,858476	7,981367	8,0028317	7,900327333	-0,10250433	0,931414771	Hira	
17548890	6,757521	6,90208	6,827375	6,589417	6,916851	6,67322	6,828992	6,726496	-0,102496	0,931420151	---	
17550268	2,964429	2,927092	2,832925	2,647731	2,984776	2,784539	2,9081487	2,805682	-0,10246667	0,931439089	---	
17300203	5,129606	4,554176	4,825083	4,705209	4,811112	4,685157	4,8362883	4,733826	-0,10246233	0,931441887	---	
17513270	5,757387	5,643238	5,868582	5,632208	5,751242	5,578373	5,7564023	5,653941	-0,10246133	0,931442533	4933408N05Rik	
17483252	7,007739	7,034359	6,76863	6,987038	6,693784	6,822583	6,9369093	6,834468333	-0,102441	0,93145566	Zfp553	
17423314	4,651196	4,663001	4,725654	4,588198	4,619136	4,525221	4,6799503	4,577518333	-0,102432	0,931461471	---	
17493681	11,69997	11,87423	12,01794	11,88413	11,84239	11,55837	11,864047	11,76163	-0,10241667	0,931471371	Snord15b	
17267106	6,908628	7,047956	6,843671	6,742149	6,864604	6,88626	6,9334183	6,831004333	-0,102414	0,931473093	Znhit3	
17203503	6,96195	6,273657	6,608129	6,589042	6,469605	6,477858	6,6145787	6,512168333	-0,10241033	0,93147546	---	
17223655	3,556851	3,849496	3,666157	3,554091	3,561975	3,649215	3,6908347	3,588427	-0,10240767	0,931477182	---	
17379260	5,732106	5,368988	5,594815	5,530191	5,332053	5,526467	5,565303	5,462903667	-0,10239933	0,931482562	R3hdml	
17239767	4,580417	4,263179	4,161849	4,245704	4,349412	4,103135	4,3351483	4,232750333	-0,102398	0,931483423	Taar8c	
17487743	5,140093	5,066029	5,016537	4,739015	5,127238	5,049229	5,0742197	4,971827333	-0,10239233	0,931487082	Tex101	
17270434	6,315195	6,00831	6,486095	6,15656	6,156682	6,189278	6,2698667	6,167506667	-0,10236	0,931507958	1700023F06Rik	
17462113	8,887995	8,949191	8,849052	8,636738	8,761347	8,981086	8,8954127	8,793057	-0,10235567	0,931510756		03/09/17
17387734	4,095225	4,045189	4,192945	3,935437	3,994179	4,096704	4,1111197	4,008773333	-0,10234633	0,931516783	Olfr1106	
17351163	4,989138	5,026257	5,138265	4,829941	5,092301	4,924404	5,05122	4,948882	-0,102338	0,931522163	---	
17260023	6,502465	6,704987	6,776189	6,530951	6,388887	6,756797	6,6612137	6,558878333	-0,10233533	0,931523885	Uqcr10	
17274905	4,29608	4,405547	4,536184	3,95743	4,767204	4,206179	4,4126037	4,310271	-0,10233267	0,931525607	---	

17340741	4,037715	4,255225	4,271259	4,080194	4,139152	4,03788	4,1880663	4,085742	-0,10232433	0,931530988	6530411M01Rik
17226123	3,024792	3,324076	3,148726	3,081033	2,924449	3,185149	3,1658647	3,063543667	-0,102321	0,93153314	---
17289824	9,42483	9,423667	9,269098	9,241538	9,243708	9,325525	9,3725317	9,270257	-0,10227467	0,931563057	Mier3
17340197	8,802517	8,785904	8,798413	8,785529	8,677326	8,617218	8,7956113	8,693357667	-0,10225367	0,931576617	Epas1
17206187	5,287143	4,452376	4,712141	5,517264	3,600366	5,027277	4,81722	4,714969	-0,102251	0,931578339	---
17368154	5,763406	5,7647	5,886122	5,586967	5,766449	5,754207	5,8047427	5,702541	-0,10220167	0,931610195	Lcn10
17246672	6,626724	6,626835	6,859831	6,570625	6,465902	6,770318	6,7044633	6,602281667	-0,10218167	0,93162311	---
17286233	5,457341	5,151993	5,276466	5,082945	5,205484	5,29083	5,2952667	5,193086333	-0,10218033	0,931623971	A330102I10Rik
17200873	5,638763	4,779111	5,701096	5,565931	4,864018	5,382493	5,37299	5,270814	-0,102176	0,93162677	---
17441340	4,287213	4,181708	3,868118	3,884415	4,291626	3,854532	4,1123463	4,010191	-0,10215533	0,931640115	---
17336758	7,895555	7,978981	7,923409	7,927877	7,876921	7,686695	7,9326483	7,830497667	-0,10215067	0,931643129	Ehmt2
17208287	4,331869	4,317669	4,542464	4,218938	4,426509	4,240116	4,397334	4,295187667	-0,10214633	0,931645927	---
17539626	6,382916	6,470438	6,186049	6,150254	6,242117	6,34062	6,3464677	6,244330333	-0,10213733	0,931651739	Trappc2
17413915	7,582034	7,545493	7,589317	7,469109	7,539165	7,402226	7,5722813	7,470166667	-0,10211467	0,931666377	Tgfbr1
17207287	5,016391	5,38707	5,059844	5,333234	5,164009	4,659723	5,154435	5,052322	-0,102113	0,931667453	---
17307774	8,25651	8,314085	8,310919	8,215337	8,111471	8,248394	8,293838	8,191734	-0,102104	0,931673265	Esco2
17344511	8,830818	8,89699	8,997875	8,781647	8,835081	8,802646	8,908561	8,806458	-0,102103	0,931673911	Abcf1
17257599	6,692513	6,748783	6,771784	6,588278	6,659007	6,659516	6,7376933	6,635600333	-0,102093	0,931680369	Gm885
17312358	5,224954	4,902942	5,017402	4,928917	5,282334	4,627789	5,0484327	4,946346667	-0,102086	0,931684889	Gm10345
17201365	4,509642	4,781699	4,596116	4,539538	4,823125	4,218564	4,6291523	4,527075667	-0,10207667	0,931690917	---
17490551	6,760321	6,489414	6,600616	6,519293	6,318419	6,706442	6,6167837	6,514718	-0,10206567	0,931698021	Prr12
17414434	9,026122	9,069969	9,098389	8,951691	8,910789	9,025821	9,0648267	8,962767	-0,10205967	0,931701895	Ugcg
17323675	6,678854	6,602725	6,920374	6,483125	6,66623	6,746437	6,7339843	6,631930667	-0,10205367	0,93170577	Arvcf
17394806	6,207168	5,932708	5,981277	6,153309	5,772365	5,889348	6,0403843	5,938340667	-0,10204367	0,931712228	Nfatc2
17328324	3,675431	3,965107	3,735754	3,572299	3,820283	3,677621	3,7920973	3,690067667	-0,10202967	0,93172127	---
17373915	6,193992	5,921959	6,196729	6,12042	5,983337	5,902836	6,1042267	6,002197667	-0,102029	0,9317217	Wt1
17459409	2,998693	3,152566	3,055707	2,905177	3,084899	2,910813	3,0689887	2,966963	-0,10202567	0,931723853	---
17224954	4,775681	4,55047	4,73426	4,54128	4,550729	4,662327	4,6868037	4,584778667	-0,102025	0,931724284	Sphkap
17279962	8,535869	8,668517	8,501051	8,353284	8,480622	8,565475	8,568479	8,466460333	-0,10201867	0,931728374	Gen1
17300187	4,748044	4,716634	4,791446	4,653294	4,620016	4,676759	4,7520413	4,650023	-0,10201833	0,931728589	---
17323776	7,225958	7,084436	7,137643	7,204431	7,036741	6,900935	7,1493457	7,047369	-0,10197667	0,931755499	2510002D24Rik
17258867	8,189282	8,389211	8,353165	8,1301	8,168156	8,327505	8,3105527	8,208587	-0,10196567	0,931762603	Birc5
17468594	5,803349	5,781784	5,870526	5,830941	5,665154	5,653754	5,818553	5,716616333	-0,10193667	0,931781333	Gkn1
17267584	8,654698	8,538888	8,531824	8,437984	8,563494	8,418132	8,5751367	8,473203333	-0,10193333	0,931783486	Akap1
17268817	6,746229	6,61395	6,677176	6,441532	6,568884	6,721147	6,6791183	6,577187667	-0,10193067	0,931785208	LOC100862101
17327019	5,066484	4,843002	4,96071	4,884667	4,648745	5,031125	4,956732	4,854845667	-0,10188633	0,931813842	4930404I05Rik
17379938	7,248199	7,155976	7,196973	6,864166	7,220895	7,210432	7,2003827	7,098497667	-0,101885	0,931814703	Cebpb
17329034	4,01092	3,739094	3,551933	4,055495	3,474371	3,466479	3,7673157	3,665448333	-0,10186733	0,931826114	Olfr167
17413821	10,03335	10,1538	10,25524	10,07854	9,973987	10,0843	10,147463	10,045609	-0,10185433	0,93183451	Anp32b // Anp32b
17322729	3,749435	3,497561	3,586435	3,589522	3,468285	3,470073	3,6111437	3,509293333	-0,10185033	0,931837094	---
17359935	8,391695	8,295486	7,693639	8,19978	7,902004	7,973584	8,12694	8,025122667	-0,10181733	0,931858409	---
17444828	7,947423	8,141651	7,95687	7,881717	7,918921	7,939863	8,0153147	7,913500333	-0,10181433	0,931860347	Polr1d
17206569	6,639235	6,069664	6,160471	6,011828	6,346018	6,206094	6,28979	6,18798	-0,10181	0,931863146	---
17201847	6,176902	5,484648	5,522772	5,679118	5,601487	5,598294	5,7281073	5,626299667	-0,10180767	0,931864653	---
17529307	5,113595	4,94776	5,107762	4,910662	4,938363	5,014769	5,0563723	4,954598	-0,10177433	0,931886184	Elovl4
17362941	4,12525	3,892283	3,856412	3,90289	3,751247	3,914506	3,9579817	3,856214333	-0,10176733	0,931890705	Ms4a14
17249232	3,680229	3,704419	3,816829	3,470843	3,922711	3,502654	3,7338257	3,632069333	-0,10175633	0,931897811	---
17502157	7,55146	7,471461	7,67834	7,405442	7,430821	7,559741	7,567087	7,465334667	-0,10175233	0,931900394	Ocel1
17303067	3,967955	3,864982	4,023857	3,948339	3,782929	3,820287	3,9522647	3,850518333	-0,10174633	0,93190427	---
17200209	5,059249	3,596562	5,025995	4,325315	3,889605	5,161712	4,560602	4,458877333	-0,10172467	0,931918266	---
17548579	4,304759	4,286323	4,215058	4,243824	4,099994	4,157149	4,2687133	4,166989	-0,10172433	0,931918481	Mrgprb13
17514333	13,49375	13,39394	13,14362	13,39894	13,17655	13,15075	13,34377	13,24208	-0,10169	0,931940659	---
17374453	3,939849	3,899601	3,869288	3,564389	3,906068	3,933213	3,9029127	3,801223333	-0,10168933	0,93194109	---
17283935	9,152048	9,447076	9,605709	8,971908	9,690969	9,236989	9,401611	9,299955333	-0,10165567	0,931962838	---
17519571	3,94963	4,331028	3,595382	3,576596	3,895766	4,098711	3,95868	3,857024333	-0,10165567	0,931962838	---
17449629	9,767282	10,06501	9,611062	9,478287	9,815641	9,844474	9,8144513	9,712800667	-0,10165067	0,931966068	---
17367676	5,986779	5,92388	6,288371	5,886271	6,126345	5,881484	6,0663433	5,9647	-0,10164333	0,931970805	Il1f10
17543648	6,604681	6,558981	6,355375	6,397841	6,46645	6,34985	6,5063457	6,404713667	-0,101632	0,931978126	Ercc6l
17284358	4,845495	4,975226	3,925477	4,815461	3,981797	4,644049	4,582066	4,480435667	-0,10163033	0,931979203	---
17237984	6,399662	6,468874	6,635157	6,308242	6,485717	6,404872	6,501231	6,399610333	-0,10162067	0,931985448	Arhgap9
17549428	6,068212	6,273907	6,097218	6,035341	6,148085	5,951112	6,1464457	6,044846	-0,10159967	0,931999014	---
17549818	6,068212	6,273907	6,097218	6,035341	6,148085	5,951112	6,1464457	6,044846	-0,10159967	0,931999014	---
17375516	3,930154	3,91525	3,957089	3,868176	3,813331	3,816199	3,9341643	3,832568667	-0,10159567	0,932001598	4930417H01Rik
17507917	5,426126	5,357578	5,433814	5,375215	5,237743	5,299856	5,4058393	5,304271333	-0,101568	0,932019471	Gm7861 // Gm7849
17507926	5,426126	5,357578	5,433814	5,375215	5,237743	5,299856	5,4058393	5,304271333	-0,101568	0,932019471	Gm7861 // Gm7849
17549092	3,776482	4,008335	3,852046	3,783951	3,610993	3,937252	3,8789543	3,777398667	-0,10155567	0,932027439	---
17287033	6,619312	6,49649	6,76834	6,392606	6,621771	6,565117	6,6280473	6,526498	-0,10154933	0,93203153	---
17466741	5,711429	5,902639	5,800703	5,655657	5,792615	5,66188	5,8049237	5,703384	-0,10153967	0,932037775	---
17433683	9,512706	9,71027	9,76903	9,453173	9,612558	9,621679	9,664002	9,56247	-0,101532	0,932042728	Tnfrsf14
17285000	8,840525	8,699077	8,747188	8,60514	8,80127	8,575833	8,7622633	8,660747667	-0,10151567	0,93205328	Klf6
17526695	5,008565	5,123119	5,205442	4,908165	5,240786	4,883641	5,1123753	5,010864	-0,10151133	0,93205608	Nnmt
17463792	4,13415	3,889486	4,031229	3,985528	3,861841	3,902976	4,0182883	3,916781667	-0,10150667	0,932059095	---
17415943	6,216431	6,159605	6,154651	6,124349	5,927064	6,174763	6,1768957	6,075392	-0,10150367	0,932061033	Dnajc6
17297882	4,02563	3,712641	4,166335	3,75174	3,837095	4,011299	3,968202	3,866711333	-0,10149067	0,932069432	---
17204899	2,972202	3,267957	3,193147	3,071	2,736187	3,321652	3,1444353	3,042946333	-0,101489	0,932070509	---
17219542	7,200855	7,153442	7,424978	7,143492	7,065612	7,265705	7,2597583	7,158269667	-0,10148867	0,932070724	Pigm
17255862	6,232627	6,337447	6,538927	6,194549	6,247412	6,362585	6,369667	6,268182	-0,101485	0,932073093	Psmb3
17208437	8,976765	8,046814	9,256506	9,085779	8,520464	8,369412	8,7600283	8,658551667	-0,10147667	0,932078477	---
17330742	8,185102	8,262563	8,099803	8,104949	8,141156	7,99696	8,1824893	8,081021667	-0,10146767	0,932084291	Plcxd2
17536044	3,380055	3,08655	3,365542	3,356397	3,05451	3,116901	3,2773823	3,175936	-0,10144633	0,932098074	Gm5938
17516749	6,058283	5,780542	6,125259	6,045183	5,915735	5,698857	5,988028	5,886591667	-0,10143633	0,932104535	---
17495651	5,14569	5,039701	4,948856	5,057469	4,813421	4,95905	5,044749	4,943313333	-0,10143567	0,932104966	Pdilt
17299200	10,51135	10,6666	10,5134	10,38344	10,48432	10,5193	10,563783	10,46235333	-0,10143	0,932108627	Psmc6
17355280	6,362442	5,951653	5,355771	6,01295	5,555399	5,797258	5,8899553	5,788535667	-0,10141967	0,932115303	---
17480192	6,907154	7,111197	7,131175	6,823723	6,8611	7,160496	7,049842	6,948439667	-0,10140233	0,932126502	Ccdc90b
17255572	4,145261	4,106555	4,239195	4,003638	4,133938	4,049258	4,1636703	4,062278	-0,10139233	0,932132963	Hoxb7
17389287	3,843123	3,961586	3,496085	3,841889	3,805373	3,349358	3,7669313	3,66554	-0,10139133	0,932133609	Gm13931
17507431	5,292578	5,130718	5,376016	5,128613	5,131528	5,235014	5,2664373	5,165051667	-0,10138567	0,932137271	---
17374692	9,947205	10,00601	10,03011	9,864506	9,883948	9,930762	9,9944417	9,893072	-0,10136967	0,932147608	Casc5
17550334	4,049856	4,413054	4,116155	3,987619	4,266171	4,021174	4,1930217	4,091654667	-0,101367	0,932149331	---
17550336	4,049856	4,413054	4,116155	3,987619	4,266171	4,021174	4,1930217	4,091654667	-0,101367	0,932149331	---
17550338	4,049856	4,413054	4,116155	3,987619	4,266171	4,021174	4,1930217	4,091654667	-0,101367	0,932149331	---
17550340	4,049856	4,413054	4,116155	3,987619	4,266171	4,021174	4,1930217	4,091654667	-0,101367	0,932149331	---
17550344	4,049856	4,413054	4,116155	3,987619	4,266171	4,021174	4,1930217	4,091654667	-0,101367	0,932149331	---
17550352	4,049856	4,413054	4,116155	3,987619	4,266171	4,021174	4,1930217	4,091654667	-0,101367	0,932149331	---
17550354	4,049856	4,413054	4,116155	3,987619	4,266171	4,021174	4,1930217	4,091654667	-0,101367	0,932149331	---
17550356	4,049856	4,413054	4,116155	3,987619	4,266171	4,021174	4,1930217	4,091654667	-0,101367	0,932149331	---
17550358	4,049856	4,413054	4,116155	3,987619	4,266171	4,021174	4,1930217	4,091654667	-0,101367	0,932149331	---
17550464	3,500705	3,593346	3,435268	3,470291	3,389115	3,365865	3,509773	3,408423667	-0,10134933	0,932160746	---
17368762	6,971852	7,215843	7,036313	6,94624	7,042599	6,931136	7,0746693	6,973325	-0,10134433	0,932163977	Ddx31
17308058	4,023631	3,985417	3,879253	3,871682	3,787307	3,925354	3,962767	3,861447667	-0,10131933	0,93218013	Adam7
17258704	4,953166	4,850046	5,152771	5,03839	4,678763	4,934883	4,9853277	4,884012	-0,10131567	0,932182499	---
17381306	8,088327	8,10907	8,012215	7,917073	8,028809	7,959911	8,0698707	7,968597667	-0,101273	0,932210068	Mcm10
17320737	4,752461	4,574543	4,757516	4,406538	4,744064	4,630135	4,69484	4,593579	-0,101261	0,932217822	Adamts20
17457677	6,12029	6,032283	6,123081	6,035647	5,841193	6,095075	6,0918847	5,990638333	-0,10124633	0,932227299	---
17238447	4,236644	3,853669	4,790644	4,156428	4,19667	4,224159	4,2936523	4,192419	-0,10123333	0,9322357	---
17516649	4,512187	4,758915	4,81982	4,539925	4,707508	4,539925	4,696974	4,595786	-0,101188	0,932264994	Treh
17541715	2,998608	3,124937	3,063996	2,95532	3,025578	2,90314	3,0625137	2,961346	-0,10116767	0,932278133	Mir450b
17338071	6,229906	5,86712	5,912846	5,781415	5,891862	6,033118	6,0032907	5,902131667	-0,101159	0,932283733	Dlk2
17347896	4,260048	3,651848	4,121149	3,970377	3,93465	3,824565	4,011015	3,909864	-0,101151	0,932288903	---
17202601	3,401604	3,396003	3,221703	3,271541	3,284947	3,159434	3,33977	3,238640667	-0,10112933	0,932302904	---
17494781	4,973685	4,208295	4,812371	4,921543	4,211832	4,557606	4,6647837	4,563660333	-0,10112333	0,932306782	Olfr482
17323888	7,504753	7,6081	7,752189	7,542174	7,456758	7,562785	7,6216807	7,520572333	-0,10110833	0,932316475	Eif2b5
17363164	3,58547	3,487731	3,681939	3,499616	3,410837	3,541384	3,5850467	3,483945667	-0,101101	0,932321214	Olfr1499
17531981	6,262522	6,248388	6,456645	6,168124	6,386966	6,109175	6,3225183	6,221421667	-0,10109667	0,932324015	---
17370733	4,731704	4,476728	4,717125	4,702137	4,39184	4,528322	4,6418523	4,540766333	-0,101086	0,932330908	Tas2r134
17506585	6,311954	6,109432	6,303729	6,029669	6,217931	6,174271	6,241705	6,140623667	-0,10108133	0,932333924	Spire2
17523186	6,570723	6,871414	6,424937	6,73522	6,540083	6,288582	6,622358	6,521295	-0,101063	0,932345772	---
17537399	3,140042	3,214456	3,102417	3,10234	2,94827	3,103174	3,152305	3,051261333	-0,10104367	0,932358266	Gm5945
17539986	7,452847	7,522426	7,453733	7,331345	7,145212	7,649482	7,4763353	7,375346333	-0,100989	0,932393596	Suv39h1
17480078	11,70537	11,85044	11,82753	11,69799	11,61627	11,76616	11,794447	11,69347333	-0,10097333	0,932403721	Picalm
17522328	5,733369	5,743475	5,966979	5,790333	5,691743	5,658878	5,8146077	5,713651333	-0,10095633	0,932414708	2610002I17Rik
17528775	9,222346	9,27022	9,086168	8,999826	9,188298	9,087763	9,1929113	9,091962333	-0,100949	0,932419447	Gm5918
17456710	9,569994	9,791101	9,842894	9,61011	9,643925	9,647114	9,734663	9,633716333	-0,10094667	0,932420955	Tspan33
17272555	6,637842	6,611596	6,781734	6,632719	6,538713	6,556906	6,6770573	6,576112667	-0,10094467	0,932422248	Tmc6
17224379	5,541682	5,68	5,431921	5,585937	5,308229	5,456628	5,551201	5,450264667	-0,10093633	0,932427634	Fev
17481320	3,98951	3,621731	3,60591	3,497393	3,804572	3,612515	3,7390503	3,63816	-0,10089033	0,932457365	Olfr654
17458020	7,20489	7,473107	7,308892	7,378059	7,162333	7,143858	7,328963	7,228083333	-0,10087967	0,932464259	Arhgef5
17517095	3,091417	2,958253	3,015963	2,839602	2,924946	2,998575	3,0218777	2,921041	-0,10083667	0,932492052	---
17386714	4,744166	4,748061	4,86358	4,606618	4,660532	4,786176	4,785269	4,684442	-0,100827	0,9324983	Ttc30b
17522479	5,362792	5,506264	5,518928	5,199313	5,661833	5,224365	5,4626613	5,361837	-0,10082433	0,932500023	Prss45
17356523	7,522429	7,833115	7,800271	7,452322	7,729399	7,671662	7,718605	7,617794333	-0,10081067	0,932508857	Sssca1 // Sssca1
17476938	5,343079	5,209476	5,473989	5,356469	5,109313	5,258349	5,3421813	5,241377	-0,10080433	0,932512951	---
17300207	3,945639	3,543917	3,728842	3,636686	3,553077	3,726316	3,739466	3,638693	-0,100773	0,932533204	---

17291525	7,729064	7,659199	7,204022	7,518921	7,474369	7,296745	7,5307617	7,430011667	-0,10075	0,932548071	Sox4
17471140	4,809891	5,065387	5,28359	4,969963	5,022541	4,864124	5,052956	4,952209333	-0,10074667	0,932550225	Ntf3
17530320	4,404492	4,396234	4,123568	4,255132	4,171163	4,195774	4,308098	4,207356333	-0,10074167	0,932553457	---
17411099	11,85871	11,99697	12,24285	12,04752	11,97946	11,76935	12,032843	11,93211	-0,10073333	0,932558844	---
17206083	4,104116	4,521101	4,178357	4,775486	4,11415	3,611799	4,267858	4,167145	-0,100713	0,932571988	---
17490917	6,354531	6,350407	6,327778	6,291864	6,329312	6,109426	6,3442387	6,243534	-0,10070467	0,932577374	Sec1
17464514	5,963723	5,904504	6,331616	5,88534	6,034639	5,977783	6,0666143	5,965920667	-0,10069367	0,932584485	Bicd1
17531075	6,423294	6,94066	6,81028	6,664324	6,668962	6,538871	6,7247447	6,624052333	-0,10069233	0,932585347	6230427J02Rik
17548205	3,283044	3,399688	3,830018	3,448498	3,598791	3,163413	3,50425	3,403567333	-0,10068267	0,932591596	Pdk1 // Pdk1
17281622	6,639108	6,579195	6,526336	6,296387	6,425737	6,720501	6,5815463	6,480875	-0,10067133	0,932598922	Cdkl1
17247533	11,03997	11,05438	10,89162	10,94312	10,84957	10,89128	10,995323	10,89465667	-0,10066667	0,932601938	Rab1
17325725	5,568182	5,15429	5,631198	5,601859	5,096584	5,353264	5,4512233	5,350569	-0,10065433	0,932609911	Drd3
17447095	10,30911	10,57803	10,58491	10,34886	10,38946	10,43182	10,490683	10,39004667	-0,10063667	0,932621331	---
17460099	5,747279	5,458554	5,947699	5,726807	5,423787	5,7011	5,717844	5,617231333	-0,10061267	0,932636846	Vax2
17248981	3,553194	3,612089	3,748275	3,573855	3,378326	3,659564	3,6378527	3,537248333	-0,10060433	0,932642233	Olfr1384
17548998	6,560431	7,13353	6,912242	6,7129	6,793503	6,79802	6,8687343	6,768141	-0,10059333	0,932649344	Tagln2 // Tagln2
17417210	3,687005	3,686961	3,896186	3,500967	3,573196	3,894227	3,7567173	3,65613	-0,10058733	0,932653223	Cyp4a30b-ps
17518142	7,653862	7,494697	7,552196	7,57607	7,441569	7,38138	7,5669183	7,466339667	-0,10057867	0,932658826	Tle3
17481590	4,873911	4,613075	4,797141	4,906864	4,431286	4,644272	4,7613757	4,660807333	-0,10056833	0,932665506	Olfr472
17439552	5,232349	4,971293	5,007625	5,008487	5,034195	4,866904	5,0704223	4,969862	-0,10056033	0,932670678	---
17389792	5,281945	4,972494	5,315778	4,822004	5,164009	5,28256	5,1900723	5,089524333	-0,100548	0,932678651	D2Ertd750e
17517456	10,07754	10,08375	10,0202	9,895447	9,986895	9,997509	10,060497	9,959950333	-0,10054633	0,932679729	Psma4
17400222	8,167187	8,090101	8,328297	8,091816	8,048846	8,143336	8,195195	8,094666	-0,100529	0,932690935	Vps72
17327995	9,223076	9,346023	9,300977	9,17208	9,158518	9,237925	9,2900253	9,189507667	-0,10051767	0,932698261	Carhsp1
17347203	6,238744	6,39935	6,422568	6,434559	6,339929	5,984638	6,353554	6,253042	-0,100512	0,932701925	Srd5a2
17402519	6,764453	6,572573	6,470517	6,359994	6,458385	6,687631	6,6025143	6,502003333	-0,100511	0,932702571	Tifa
17341451	4,181331	3,692349	3,636035	3,699867	3,767498	3,740961	3,8365717	3,736108667	-0,100463	0,932733604	Vmn2r113 // Vmn2r113
17537711	3,274234	3,07566	3,027537	2,977109	3,094288	3,004649	3,1258103	3,025348667	-0,10046167	0,932734466	---
17247067	7,879364	7,927828	7,703539	7,554317	7,775522	7,879527	7,8369103	7,736455333	-0,100455	0,932738776	Ykt6
17537722	9,237405	9,466763	9,171384	9,071013	9,26328	9,239913	9,2918507	9,191402	-0,10044867	0,932742871	Hnrnph2
17489522	5,552093	5,828009	5,62517	5,506467	5,633717	5,563764	5,668424	5,567982667	-0,10044133	0,932747612	Gm5058
17269241	4,851627	4,684865	4,489937	4,735789	4,53999	4,449458	4,6754763	4,575079	-0,10039733	0,93277606	Krtap4-13 // Krtap4-13
17442977	7,666675	7,955464	7,739563	7,725562	7,645994	7,688986	7,787234	7,686847333	-0,10038667	0,932782956	Crcp
17200163	3,032163	2,959124	2,987387	3,308611	2,718995	2,649984	2,9928913	2,89253	-0,10036133	0,932799336	---
17366814	5,996399	5,212221	5,47113	5,758745	5,3803	5,239672	5,5599167	5,459572333	-0,10034433	0,932810328	Mir669k // Mir669k
17366850	5,996399	5,212221	5,47113	5,758745	5,3803	5,239672	5,5599167	5,459572333	-0,10034433	0,932810328	Mir669k // Mir669k
17428430	8,061062	8,388124	8,218462	8,162706	7,966971	8,237	8,2225493	8,122225667	-0,10032367	0,93282369	Cmpk1
17466783	8,381052	8,4931	8,415202	8,270443	8,399038	8,318907	8,4297847	8,329462667	-0,100322	0,932824768	Skap2
17505367	7,653802	7,471782	7,188493	7,232979	7,407493	7,372697	7,4380257	7,337723	-0,10030267	0,932837269	Txnl4b
17207307	5,018224	4,30251	4,419777	3,8847	5,109161	4,445844	4,5801703	4,479901667	-0,10026867	0,932859253	---
17494688	3,790724	3,868106	3,88693	3,974893	3,573994	3,69609	3,8485867	3,748325667	-0,100261	0,93286421	Olfr695
17287426	5,412858	5,383276	5,296127	5,426168	5,11452	5,253817	5,364087	5,263835	-0,100252	0,93287003	Hrh2
17546410	2,888174	3,182858	2,778668	2,820455	2,899078	2,829478	2,9499	2,849670333	-0,10022967	0,932884471	---
17302617	7,921645	7,970727	8,032845	7,903329	7,824913	7,896333	7,9750723	7,874858333	-0,100214	0,932894602	---
17261750	6,577703	6,883945	7,027314	6,907028	6,750167	6,531143	6,829654	6,729446	-0,100208	0,932898482	Wwc1
17487023	3,985543	3,910876	3,910876	3,909698	3,944428	3,652587	3,935765	3,835571	-0,100194	0,932907535	Gm9519
17408085	4,792855	4,995465	5,012113	4,374896	5,25286	4,872112	4,9334777	4,833289333	-0,10018833	0,932911199	6330549D23Rik
17267700	4,354997	4,605323	4,755375	4,56702	4,500908	4,34723	4,5718983	4,471719333	-0,100179	0,932917234	---
17251423	4,389159	4,021271	4,517822	4,181223	4,423093	4,023419	4,3094173	4,209245	-0,10017233	0,932921545	Rnf222
17285513	3,827012	3,933678	4,072781	3,800174	3,944499	3,788299	3,9444903	3,844324	-0,10016633	0,932925425	---
17518672	10,27963	10,33452	10,33661	10,16747	10,21453	10,26835	10,31692	10,21678333	-0,10013667	0,932944609	Fam96a
17493807	6,137635	6,147039	6,208578	6,175027	6,032699	5,985124	6,1644173	6,064283333	-0,100134	0,932946334	Plekhb1
17517700	4,452446	4,509855	4,780659	4,416912	4,453036	4,572633	4,5809867	4,480860333	-0,10012633	0,932951292	Trcg1
17476702	5,163751	4,696163	4,657929	4,790197	4,931524	4,495778	4,839281	4,739166333	-0,10011467	0,932958836	---
17521711	4,626513	4,475516	4,873557	4,559675	4,583654	4,531957	4,6585287	4,558428667	-0,1001	0,932968321	1700102P08Rik
17207005	7,217706	7,119958	7,668925	7,143088	7,289326	7,273884	7,3355297	7,235432667	-0,100097	0,932970261	---
17545420	6,2865	6,200778	6,087265	5,854089	6,182672	6,237514	6,1915143	6,091425	-0,10008933	0,932975219	Ribc1
17351407	4,691764	4,516157	4,66052	4,41319	4,56714	4,587892	4,6228137	4,522740667	-0,100073	0,932985782	---
17205299	3,950144	3,833467	3,907494	3,728687	4,148437	3,513844	3,897035	3,796989333	-0,10004567	0,933003458	---
17249250	4,613207	4,382194	4,500779	4,494893	4,311169	4,389996	4,4987267	4,398686	-0,10004067	0,933006692	Olfr1378
17474227	4,938663	5,092142	5,15628	5,187032	5,134575	4,565368	5,0623617	4,962325	-0,10003667	0,933009278	Pnmal1
17291060	3,12725	3,552269	3,353422	3,120716	3,315031	3,29711	3,3443137	3,244285667	-0,100028	0,933014883	---
17240043	7,584326	7,45199	7,480516	7,508979	7,192895	7,514982	7,5056107	7,405618667	-0,099992	0,933038165	Gm20300 // Gm20300
17458010	3,336478	3,291198	3,434798	3,306553	3,343749	3,112274	3,354158	3,254192	-0,099966	0,933054981	Olfr435
17378311	7,822444	8,021564	7,44864	7,191686	8,101652	7,699434	7,764216	7,664257333	-0,09995867	0,933059723	---
17283830	6,215314	6,269289	6,157561	6,385004	5,593304	6,364077	6,2140547	6,114128333	-0,09992633	0,933080635	---
17374880	5,288017	5,116993	5,306089	5,257134	5,107827	5,046365	5,237033	5,137108667	-0,09992433	0,933081929	Tyro3
17351591	6,192308	6,313144	6,590728	6,225553	6,341828	6,229053	6,3653933	6,265478	-0,09991533	0,93308775	A730085E03Rik
17223834	3,714112	3,497858	3,424347	3,39893	3,459233	3,478411	3,545439	3,445524667	-0,09991433	0,933088396	Crygd
17311060	3,600538	3,632093	3,515087	3,541294	3,446565	3,460119	3,5825727	3,482659333	-0,09991333	0,933089043	---
17263463	7,823708	7,588188	7,654319	7,652226	7,55923	7,555031	7,6887383	7,588829	-0,09990933	0,93309163	Flcn
17247155	5,4598	5,470783	5,659911	5,510849	5,322451	5,457545	5,5301647	5,430281667	-0,099883	0,933108662	Myo1g // Myo1g
17355535	5,208608	5,247169	5,24851	5,053298	5,479402	4,871944	5,2347623	5,134881333	-0,099881	0,933109955	A330094K24Rik
17421732	4,126176	3,702893	3,56934	3,790382	3,600415	3,708019	3,7994697	3,699605333	-0,09986433	0,933120735	---
17411487	3,216783	3,01915	3,168221	2,980914	3,026143	3,097516	3,134718	3,034857667	-0,09986033	0,933123322	---
17225811	6,817524	6,727078	6,230052	6,834929	6,287614	6,352559	6,5915513	6,491700667	-0,09985067	0,933129575	---
17248860	8,373988	8,389109	7,92766	7,772445	8,266068	8,352712	8,2302523	8,130408333	-0,099844	0,933133887	Timd4
17331859	3,571323	3,83966	3,644049	3,507815	3,604068	3,643628	3,6850107	3,585170333	-0,09984033	0,933136258	LOC433047
17330803	3,57098	3,381358	3,388801	3,535062	3,295518	3,211135	3,4470463	3,347238333	-0,099808	0,933157172	---
17445280	8,694513	8,727461	8,438127	8,495331	8,575218	8,490173	8,6200337	8,520240667	-0,099793	0,933166874	Ankib1
17211522	3,150327	3,301951	2,925076	2,94577	3,016126	3,116103	3,1257847	3,025999667	-0,099785	0,933172049	---
17303757	9,497493	9,545411	9,498775	9,250854	9,431397	9,56009	9,513893	9,414113667	-0,09977933	0,933175714	Dnajc9
17221242	11,81426	11,90738	12,11819	11,92042	11,94158	11,67861	11,94661	11,84687	-0,09974	0,933201156	---
17223616	9,31839	9,486435	9,372604	9,468667	9,040793	9,368755	9,3924763	9,292738333	-0,099738	0,93320245	Raph1
17268093	4,367874	4,471168	4,476624	4,404446	4,474979	4,137049	4,4385553	4,338824667	-0,09973067	0,933207194	Gm11544
17538051	3,810807	3,748481	3,589135	3,529614	3,612386	3,707448	3,716141	3,616482667	-0,09965833	0,933253984	4930513O06Rik
17292276	10,81733	11,09087	11,10521	10,87159	10,90491	10,93804	11,00447	10,90484667	-0,09962333	0,933276625	Dek
17357126	9,043244	9,298608	9,335899	9,197021	9,102386	9,079477	9,225917	9,126294667	-0,09962233	0,933277272	Snhg1
17507842	3,673234	3,701838	3,634017	3,618108	3,508441	3,583673	3,6696963	3,570074	-0,09962233	0,933277272	Defb12
17205255	3,33228	3,309598	3,413188	3,200738	3,410933	3,144555	3,3516887	3,252075333	-0,09961333	0,933283094	---
17209445	3,33228	3,309598	3,413188	3,200738	3,410933	3,144555	3,3516887	3,252075333	-0,09961333	0,933283094	---
17273948	7,508492	7,312582	7,163416	7,130065	7,148602	7,40701	7,3281633	7,228559	-0,09960433	0,933288916	Sdc1
17517481	8,515846	8,567389	8,604979	8,375002	8,435695	8,578737	8,562738	8,463144667	-0,09959333	0,933296032	Ube2q2
17383835	7,245628	7,332593	7,445177	7,333592	7,338996	7,052018	7,3411127	7,241535333	-0,09957733	0,933306382	Uck1
17209963	5,70867	6,561336	6,132713	5,950852	6,663986	5,489204	6,1342397	6,034680667	-0,099559	0,933318243	---
17548802	3,958656	4,654567	4,244445	4,121581	4,564213	3,873282	4,2858893	4,186358667	-0,09953067	0,933336572	LOC100040263
17475061	5,480956	5,363419	5,523698	5,399336	5,409345	5,260852	5,4560243	5,356511	-0,09951333	0,933347786	Gm4598
17500593	4,89081	5,325253	5,732358	5,232048	5,274903	5,142934	5,3161403	5,216628333	-0,099512	0,933348649	Gm10063
17373155	8,414958	8,545092	8,526764	8,367975	8,433296	8,387025	8,4956047	8,396098667	-0,099506	0,93335253	Psmc3
17209051	3,620254	3,346052	3,872883	3,310574	3,654994	3,575147	3,613063	3,513571667	-0,09949133	0,933362019	---
17337919	6,249424	6,166321	6,442209	6,082902	6,14605	6,330536	6,2859847	6,186496	-0,09948867	0,933363744	Tcte1
17305789	9,853429	10,04599	9,99877	9,807656	9,858719	9,933374	9,966063	9,866583	-0,09948	0,933369351	Dlgap5
17317900	6,962362	6,870021	6,877955	6,898196	6,764039	6,749695	6,903446	6,803976667	-0,09946933	0,933376252	Gm10362
17363016	3,90681	3,798094	3,806241	3,758018	3,802251	3,652504	3,8370483	3,737591	-0,09945733	0,933384016	Oosp1
17256932	6,699798	6,977042	6,87906	6,855603	6,748274	6,653652	6,8519667	6,752509667	-0,099457	0,933384232	Tmub2
17283991	5,405739	5,247779	5,347751	5,223576	4,996652	5,482704	5,3337563	5,234310667	-0,09944567	0,933391564	---
17534555	7,353402	7,198952	7,180722	7,144681	7,190283	7,099806	7,2443587	7,144923333	-0,09943533	0,933398249	Sash3
17261247	6,169891	6,059676	6,292094	6,163157	6,077539	5,98266	6,173887	6,074452	-0,099435	0,933398465	A630052C17Rik
17549182	3,159042	3,234406	3,126909	3,010823	3,154505	3,056734	3,1734523	3,074020667	-0,09943167	0,933400622	---
17325919	8,973332	9,112471	8,795566	8,835161	8,831102	8,916855	8,9604563	8,861039333	-0,099417	0,933410111	Atg3
17312278	3,630687	3,772181	3,856187	3,656879	3,690809	3,613195	3,7530183	3,653627667	-0,09939067	0,933427148	---
17366631	4,536154	4,456411	4,141075	4,396657	4,2712	4,16763	4,37788	4,278495667	-0,09938433	0,933431246	1700061F12Rik
17497713	7,740016	8,047532	7,519629	7,733862	7,788509	7,486675	7,769059	7,669682	-0,099377	0,933435991	Ifitm2
17468261	5,740685	5,758928	5,617312	5,66919	5,548161	5,601467	5,7056417	5,606272667	-0,099369	0,933441167	Paip2b
17263148	3,939593	3,920763	4,11157	3,843167	3,967919	3,862733	3,990642	3,891273	-0,099369	0,933441167	Olfr30
17222878	7,854916	7,897352	8,019417	7,831544	7,832635	7,809415	7,923895	7,824531333	-0,09936367	0,933444618	Gm3940
17435419	4,455167	4,200401	4,248262	4,391474	3,935427	4,278852	4,3012767	4,201917667	-0,099359	0,933447637	1700022A21Rik
17207389	3,124666	3,063464	3,052959	2,884179	3,310443	2,748391	3,080363	2,981004333	-0,09935867	0,933447853	---
17446620	5,501994	5,354262	5,631191	5,494866	5,268921	5,42562	5,4958157	5,396469	-0,09934667	0,933455617	Dnajb6 // Dnajb6
17542023	3,802007	3,628946	3,651848	3,671942	3,546324	3,566507	3,694267	3,594924333	-0,09934267	0,933458205	Gm7073
17418168	5,132866	5,114429	5,156688	5,026198	5,111136	4,968736	5,134661	5,035356667	-0,09930433	0,933483008	Nt5c1a
17233794	6,549801	6,537537	6,493953	6,58613	6,278548	6,418763	6,527097	6,427813667	-0,09928333	0,933496596	Ddx21 // Ddx21
17530920	4,808883	4,846246	4,930731	4,808009	4,7063	4,773705	4,8619533	4,762671333	-0,099282	0,933497459	---
17206313	3,378995	4,835287	5,266931	4,729966	3,877263	4,576222	4,4937377	4,394483667	-0,099254	0,933515576	---
17247371	4,332368	4,272116	4,260388	4,210687	4,088874	4,267564	4,2882907	4,189041667	-0,099249	0,933518812	Pom121l12
17499560	8,24308	8,496814	8,164845	8,079833	8,111595	8,415581	8,3015797	8,202336333	-0,09924333	0,933522478	Agpat5
17391655	5,668677	5,71493	5,837419	5,548114	5,727535	5,647687	5,740342	5,641112	-0,09923	0,933531106	4930473A02Rik
17449635	10,27364	10,06218	9,996486	9,939982	10,14438	9,950286	10,110769	10,01154933	-0,09921933	0,933538008	---
17294416	8,097406	8,051884	7,846066	7,984282	7,965884	7,747535	7,998452	7,899233667	-0,09921833	0,933538655	Zfp825
17300441	6,625292	6,417017	6,676749	6,529931	6,335553	6,555926	6,5730193	6,473803333	-0,099216	0,933540165	Lrrc16b
17528537	4,274529	4,156139	4,066225	4,131861	3,968277	4,099136	4,165631	4,066424667	-0,09920633	0,93354642	LOC434412
17404474	8,44846	8,646205	8,085361	8,350187	8,171257	8,361001	8,393342	8,294148333	-0,09919367	0,933554617	1600012F09Rik
17208247	7,563182	7,678506	7,215707	6,842093	7,409391	7,908347	7,4857983	7,386610333	-0,099188	0,933558283	---
17482160	7,439627	7,749485	7,258881	7,417949	7,447693	7,284789	7,4826643	7,383477	-0,09918733	0,933558715	6330503K22Rik

17303578	8,950559	9,201937	9,030586	8,905999	8,990845	8,988685	9,0610273	8,961843	-0,09918433	0,933560656	Psmd6
17370330	3,319385	3,452257	3,183736	3,2916	3,080456	3,285779	3,3184593	3,219278333	-0,099181	0,933562813	Olfr339
17298738	8,035345	8,078143	7,833674	7,97768	7,820233	7,851728	7,9823873	7,883213667	-0,09917367	0,933567558	Gdf2
17475874	4,977938	4,887092	5,172864	4,981913	4,814253	4,944213	5,0126313	4,913459667	-0,09917167	0,933568853	Fbxo27
17301272	4,938931	5,045869	4,958193	4,843935	4,926877	4,874702	4,9809977	4,881838	-0,09915967	0,933576618	Prss51
17347846	7,716729	7,677939	7,724232	7,623491	7,645608	7,552332	7,7063	7,607143667	-0,09915633	0,933578775	Srbd1
17323473	8,558428	8,608009	8,503222	8,448619	8,34202	8,581555	8,556553	8,457398	-0,099155	0,933579638	Crkl
17419316	4,197484	3,80084	4,225787	4,106493	4,09353	3,726624	4,0747037	3,975549	-0,09915467	0,933579853	---
17363107	8,284336	8,435654	8,413233	8,301142	8,228527	8,306139	8,377741	8,278602667	-0,09913833	0,933590423	U05342
17238760	5,142254	4,462854	5,430341	5,183014	4,769094	4,785945	5,0118163	4,912684333	-0,099132	0,933594521	---
17265721	4,639005	4,605686	5,05636	4,60753	4,830948	4,565185	4,767017	4,667887667	-0,09912933	0,933596247	4732414G09Rik
17417817	3,447095	3,913449	3,681522	3,667007	3,466375	3,611347	3,6806887	3,581576333	-0,09911233	0,933607248	Olfr1340
17285668	9,922809	9,886562	9,811195	9,729603	9,766366	9,827261	9,873522	9,77441	-0,099112	0,933607464	---
17363644	7,829989	8,078185	7,718026	7,818489	7,826735	7,683682	7,8754	7,776302	-0,099098	0,933616524	Cbwd1
17375911	8,393325	8,315872	8,265477	8,162281	8,230911	8,284294	8,3248913	8,225828667	-0,09906267	0,933639389	Mrps5
17472465	3,999323	3,88491	3,71681	3,791238	3,719375	3,793252	3,8670143	3,767955	-0,09905933	0,933641546	---
17424452	4,594796	4,698886	4,174816	4,136046	4,513207	4,522155	4,4894993	4,390469333	-0,09903	0,93366053	Gm2564 // Gm2457 // Gm12407 // Gm2
17436054	5,62758	5,325654	5,449686	5,388228	5,393422	5,324205	5,46764	5,368618333	-0,09902167	0,933665923	Gtf3c2 // Gtf3c2
17333589	8,831627	9,034573	8,786291	8,721774	8,781989	8,851686	8,8841637	8,785149667	-0,099014	0,933670884	Chd1
17394160	4,863955	4,779338	5,034007	5,007814	4,683549	4,688906	4,8924333	4,793423	-0,09901033	0,933673257	Matn4
17518616	7,604174	7,668894	7,492467	7,504329	7,450288	7,513918	7,5885117	7,489511667	-0,099	0,933679945	Rbpms2
17441585	4,449029	4,280083	4,416334	4,311933	4,457464	4,079064	4,3818153	4,282820333	-0,098995	0,933683181	---
17525425	3,462977	3,052797	3,233192	3,045095	3,301273	3,105647	3,2496553	3,150671667	-0,09898367	0,933690516	Pate4
17341705	8,798326	8,59778	8,786224	8,496194	8,685183	8,704096	8,7274433	8,628491	-0,09895233	0,933710794	---
17320813	5,255314	5,357025	5,196397	5,248006	5,175316	5,088615	5,2695787	5,170645667	-0,098933	0,933723307	Nell2
17333410	5,307629	4,914036	4,614419	4,249145	4,994061	5,29611	4,9453613	4,846438667	-0,09892267	0,933729995	Smok4a
17202369	7,195171	6,507707	7,566829	6,798367	7,027759	7,146853	7,0899023	6,990993	-0,09890933	0,933738624	---
17480143	4,694428	4,809184	4,680022	4,617687	4,691779	4,577486	4,727878	4,628984	-0,098894	0,933748548	Dlg2
17467290	3,998468	4,005836	3,967536	3,856547	4,03462	3,78409	3,9906133	3,891752333	-0,098861	0,933769907	Vmn1r32
17442177	7,00844	6,967854	7,13472	7,021471	6,940367	6,852643	7,0370047	6,938160333	-0,09884433	0,933780694	Orai1
17545809	4,251255	3,958621	3,980643	4,099204	3,806365	3,988429	4,0635063	3,964666	-0,09884033	0,933783283	Gja6
17455216	3,289539	3,448309	3,84322	3,332241	3,676382	3,27595	3,5270227	3,428191	-0,09883167	0,933788893	1700001J03Rik
17496041	5,217204	4,641652	4,996391	5,055315	4,60931	4,894178	4,951749	4,852934333	-0,09881467	0,933799896	4933440M02Rik
17364845	4,877732	5,340071	5,188589	4,888198	5,142657	5,079094	5,135464	5,036649667	-0,09881433	0,933800112	D19Ertd386e // D19Ertd386e
17367338	4,009377	4,235009	4,086582	4,050591	3,949815	4,034155	4,1103227	4,011520333	-0,09880233	0,933807879	Armc3
17501170	4,78727	4,830935	4,842608	4,704416	4,822206	4,637836	4,820271	4,721486	-0,098785	0,933819098	Asb5
17210537	5,425596	5,693455	5,489472	5,520862	5,283995	5,507379	5,5361743	5,437412	-0,09876233	0,93383377	---
17209611	4,418374	4,483925	4,759546	4,31516	4,368059	4,682344	4,5539483	4,455187667	-0,09876067	0,933834849	---
17222601	5,688433	5,575461	5,73136	5,672607	5,531301	5,495067	5,6650847	5,566325	-0,09875967	0,933835496	Gm16894
17417400	6,289227	6,095253	6,286538	6,167475	6,064076	6,143189	6,2236727	6,124913333	-0,09875933	0,933835712	1700042G07Rik
17296248	9,466569	9,572109	9,313231	9,324995	9,351225	9,379544	9,4506363	9,351921333	-0,098715	0,933864409	Skiv2l2
17316278	4,164778	3,63862	4,230097	4,087755	3,892884	3,75672	4,011165	3,912453	-0,098712	0,933866351	---
17455969	4,559542	4,597284	4,817237	4,55094	4,671663	4,45543	4,658021	4,559344333	-0,09867667	0,933889222	---
17219762	5,071618	5,297508	5,00424	5,142785	4,894153	5,040438	5,1244553	5,025792	-0,09866333	0,933897853	Olfr414
17526287	5,522753	5,353558	5,559969	5,362867	5,293743	5,483685	5,47876	5,380098333	-0,09866167	0,933898932	Foxr1
17241553	5,154645	5,050024	5,006425	4,927106	5,168067	4,819988	5,0703647	4,971720333	-0,09864433	0,933910153	Mypn
17439530	3,774798	3,340138	3,56083	3,514041	3,507045	3,358818	3,5585887	3,459968	-0,09862067	0,933925473	---
17224270	7,842312	7,736365	7,942737	7,805429	7,751405	7,668718	7,8404713	7,741850667	-0,09862067	0,933925473	Tmbim1
17301211	3,062135	2,940817	3,110764	3,01341	3,069888	2,734574	3,0379053	2,939290667	-0,09861467	0,933929357	---
17401293	4,721416	4,608295	4,54236	4,525482	4,601924	4,448841	4,6240237	4,525415667	-0,098608	0,933933673	Gm15472
17316569	5,567763	5,578094	5,821229	5,690812	5,524217	5,456357	5,6556953	5,557128667	-0,09856667	0,933960431	Gm16136
17517390	9,202374	9,287829	9,07378	9,104294	8,954181	9,209878	9,1879943	9,089451	-0,09854333	0,933975536	Idh3a
17391971	11,52992	11,61542	11,64862	11,45861	11,54557	11,49417	11,597987	11,49945	-0,09853667	0,933979852	Pcna
17455652	5,644935	5,123054	5,038509	5,395713	4,712228	5,403001	5,2688327	5,170314	-0,09851867	0,933991505	LOC100862180 // LOC100862166 // Gm7
17424998	7,300956	7,354573	7,240108	6,912579	7,398278	7,289258	7,2985457	7,200038333	-0,09850733	0,933998842	Xpa
17518256	7,447655	7,340204	7,230814	7,180956	7,336566	7,20565	7,3395577	7,241057333	-0,09850033	0,934003374	Aagab
17245320	8,236679	8,056056	7,957033	7,973583	8,046333	7,934357	8,083256	7,984757667	-0,09849833	0,934004669	Cdc5l // Cdc5l
17551133	8,93362	9,038321	8,994102	8,871176	8,858049	8,941357	8,988681	8,890194	-0,098487	0,934012006	---
17390485	5,053173	4,773294	4,889412	4,865641	4,697124	4,857687	4,905293	4,806817333	-0,09847567	0,934019343	---
17472108	5,867561	5,417292	5,583918	5,690141	5,201396	5,68195	5,6229237	5,524495667	-0,098428	0,934050204	E330021D16Rik
17378321	3,270892	3,994832	2,973156	2,906405	3,453524	3,583673	3,41296	3,314534	-0,098426	0,934051499	---
17423187	3,118998	3,089074	3,249575	2,945583	2,982331	3,234455	3,152549	3,054123	-0,098426	0,934051499	---
17331228	3,007075	3,084231	2,995165	2,924065	2,9837	2,883446	3,0288237	2,930403667	-0,09842	0,934055383	Olfr176
17387831	3,690529	3,329426	3,477278	3,36243	3,502249	3,337316	3,4990777	3,400665	-0,09841267	0,934060131	Olfr1180
17367906	7,71576	8,067356	8,077196	8,314489	7,255077	7,995655	7,9534373	7,855073667	-0,09836367	0,934091856	---
17277879	8,642839	8,514827	8,360247	8,395417	8,348228	8,479226	8,505971	8,407623667	-0,09834733	0,934102432	Tdp1
17433301	4,893531	4,372414	4,799489	4,529096	4,813749	4,42755	4,688478	4,590131667	-0,09834633	0,934103079	---
17219751	3,516399	3,453524	3,527376	3,508646	3,280551	3,413076	3,4990997	3,400757667	-0,098342	0,934105885	1810030J14Rik // 1810030J14Rik
17229110	3,947704	3,619813	4,122095	3,985915	3,775136	3,633566	3,8965373	3,798205667	-0,09833167	0,934112575	---
17496283	10,69532	10,80846	10,76688	10,6322	10,68387	10,65964	10,756887	10,65857	-0,09831667	0,934122288	Eif3c
17533336	9,370921	9,596185	9,486541	9,388946	9,439855	9,329906	9,484549	9,386235667	-0,09831333	0,934124446	Atp6ap2
17236016	3,570994	3,748942	3,509929	3,372462	3,488612	3,673901	3,609955	3,511658333	-0,09829667	0,934135237	---
17445160	3,127495	3,218912	3,276754	3,199002	3,056237	3,073035	3,2077203	3,109424667	-0,09829567	0,934135885	Fry
17472899	4,839891	4,429214	4,834539	4,664693	4,588928	4,555185	4,7012147	4,602935333	-0,09827933	0,934146461	---
17510351	5,642461	5,341796	5,600217	5,401537	5,378123	5,509998	5,528158	5,429886	-0,098272	0,934151209	Tmem221
17384994	8,292592	8,312927	8,166225	8,117194	8,251307	8,108462	8,257248	8,158987667	-0,09826033	0,934158763	Orc4
17489954	4,024794	3,865324	4,341572	3,650719	4,179744	4,106548	4,07723	3,978970333	-0,09825967	0,934159195	LOC546957
17539646	4,630021	4,191721	4,522607	4,1741	4,476506	4,399047	4,4481163	4,349884333	-0,098232	0,93417711	---
17387969	3,968861	3,841134	3,652374	3,75081	3,655003	3,761861	3,8207897	3,722558	-0,09823167	0,934177325	Olfr1247
17236681	7,82987	7,977903	7,748302	7,57935	7,722386	7,95966	7,852025	7,753798667	-0,09822633	0,934180779	Ndufa12
17201143	4,036381	4,414185	4,201473	4,20005	4,220323	3,937018	4,2173463	4,119130333	-0,098216	0,93418747	---
17537283	4,833015	4,467397	4,461094	4,573888	4,487166	4,405827	4,5871687	4,488960333	-0,09820833	0,934192434	Dach2
17503596	9,20028	9,129356	9,26917	9,128457	9,098896	9,076843	9,199602	9,101398667	-0,09820333	0,934195672	Papd5
17509005	3,126293	3,100558	3,114973	2,869683	2,920631	3,256909	3,1139413	3,015741	-0,09820033	0,934197615	Adam26a
17416757	4,919531	5,812621	5,340568	4,935137	5,404069	5,438944	5,3575733	5,259383333	-0,09819	0,934204306	---
17371221	4,019038	4,15461	3,741345	3,841381	3,724744	4,054378	3,9716643	3,873501	-0,09816333	0,934221574	Scn2a1
17549614	6,147496	6,016909	5,866909	5,55477	6,016649	6,165428	6,010438	5,912282333	-0,09815567	0,934226538	---
17290188	2,912418	2,839739	2,939525	2,794882	2,655808	2,946594	2,8972273	2,799094667	-0,09813267	0,934241432	---
17336144	7,233179	6,967628	7,145506	7,013318	6,867863	7,170735	7,1154377	7,017305333	-0,09813233	0,934241648	Zfp414
17377675	9,949999	10,09651	10,01854	9,900687	9,973289	9,896784	10,021683	9,923586667	-0,09809633	0,934264961	Csnk2a1
17386340	6,109972	5,834171	6,063992	6,035641	5,872404	5,805803	6,0027117	5,904616	-0,09809567	0,934265393	1500002O10Rik
17284839	6,07106	5,787281	5,721972	5,903994	5,786177	5,595913	5,8601043	5,762028	-0,09807633	0,934277913	Itgb8
17315388	5,784219	6,434996	6,735004	6,213194	6,526515	5,920302	6,318073	6,220003667	-0,09806933	0,934282446	---
17481225	3,241409	3,298528	3,045261	3,0569	3,080333	3,153773	3,195066	3,097002	-0,098064	0,9342859	Olfr243
17514359	9,255026	9,468659	9,462205	9,288514	9,195786	9,407429	9,3952967	9,297243	-0,09805367	0,934292592	Alkbh8
17408875	3,384793	3,637443	3,568624	3,447494	3,550127	3,299106	3,5302867	3,432242333	-0,09804433	0,934298636	1700027A23Rik
17548207	9,159607	9,457827	9,305769	9,185579	9,153525	9,289978	9,3077343	9,209694	-0,09804033	0,934301226	---
17378328	9,284681	9,244415	9,433978	9,172899	9,412969	9,083094	9,3210247	9,222987333	-0,09803733	0,934303169	---
17407431	3,239752	3,266634	3,34331	2,971385	3,403323	3,18088	3,283232	3,185196	-0,098036	0,934304033	Lce1a1
17414828	5,355891	5,487424	5,526657	5,330453	5,60189	5,143522	5,4566573	5,358621667	-0,09803567	0,934304248	Trim32
17546923	3,566943	4,353857	3,903004	3,593912	4,319489	3,616342	3,941268	3,843247667	-0,09802033	0,934314179	Rbmy1a1
17313974	4,399942	4,863921	4,616336	4,522343	4,604539	4,45926	4,626733	4,528714	-0,098019	0,934315042	---
17513166	9,369025	9,330498	9,340386	9,136874	9,354117	9,25487	9,3466363	9,248620333	-0,098016	0,934316985	Cfdp1
17200113	5,632561	6,137168	6,471662	5,811014	5,767759	6,368578	6,0804637	5,982450333	-0,09801333	0,934318712	---
17282776	5,935367	5,974602	5,993756	5,822529	5,767656	6,019509	5,9679083	5,869898	-0,09801033	0,934320655	6430527G18Rik
17359079	4,931246	4,613513	4,955998	4,58648	4,795782	4,824479	4,8335857	4,735580333	-0,09800533	0,934323893	Cyp26c1
17364762	9,569139	9,945166	9,692412	9,710472	9,598046	9,604232	9,7355723	9,637583333	-0,097989	0,934334471	Exosc1
17417620	2,979577	3,051803	3,189444	3,036137	2,866532	3,024211	3,073608	2,975626667	-0,09798133	0,934339436	---
17517914	10,05758	10,07004	9,980242	9,852963	9,982138	9,978834	10,035954	9,937978333	-0,09797567	0,934343106	Nptn
17336595	6,127203	6,566538	6,449472	6,547297	6,094083	6,207921	6,381071	6,283100333	-0,09797067	0,934346344	Gpsm3
17384976	4,494018	4,658776	4,527233	4,327272	4,406825	4,652053	4,560009	4,46205	-0,097959	0,9343539	---
17545708	4,672589	4,835997	5,301819	5,059563	4,641033	4,815966	4,9368017	4,838854	-0,09794767	0,93436124	---
17548973	10,07792	10,091	9,850943	9,870105	9,923846	9,932107	10,006621	9,908686	-0,097935	0,934369444	Pten // Pten
17316845	7,083152	6,83622	7,126523	6,838427	7,030073	6,8836	7,0152983	6,917366667	-0,09793167	0,934371602	Sybu
17396093	4,615728	4,693995	4,86046	4,573576	4,694418	4,608444	4,7233943	4,625479333	-0,097915	0,934382397	Gm6300
17551083	4,014363	4,240016	4,381603	4,097448	4,258736	3,986065	4,211994	4,114083	-0,097911	0,934384987	---
17228102	7,649195	7,603362	7,850909	7,517094	7,59798	7,694679	7,7011553	7,603251	-0,09790433	0,934389305	Arpc5 // Arpc5
17377540	6,294467	6,141018	6,125596	6,295382	5,942327	6,029696	6,187027	6,089135	-0,097892	0,934397293	4921509C19Rik
17255604	5,201144	5,192003	5,184563	4,849083	5,298066	5,136893	5,19257	5,094680667	-0,09788933	0,93439902	Hoxb3
17400424	5,076769	4,676456	4,847566	5,004255	4,734268	4,568696	4,8669303	4,769073	-0,09785733	0,934419746	---
17515813	5,089836	5,124384	5,011954	4,876572	5,28104	4,774998	5,0753913	4,977536667	-0,09785467	0,934421473	Kcnj1
17431836	4,962805	4,899448	4,738724	4,732113	4,820949	4,754404	4,8669923	4,769155333	-0,097837	0,934432916	---
17249640	5,486195	5,48844	5,569381	5,516747	5,338483	5,395307	5,514672	5,416845667	-0,09782633	0,934439825	Acsl6
17470627	5,282355	5,349975	5,10926	5,03757	5,409592	5,000968	5,2471967	5,149376667	-0,09782	0,934443927	Clec4e
17284135	7,329432	7,500066	7,376931	7,574879	7,162474	7,175653	7,402143	7,304335333	-0,09780767	0,934451915	Xrcc3
17410612	7,73021	7,841154	7,747741	8,173811	7,194243	7,65764	7,773035	7,675231333	-0,09780367	0,934454506	Gm19693
17511464	3,323164	3,110419	3,332807	3,293393	2,96774	3,211851	3,2554633	3,157661333	-0,097802	0,934455586	---
17402797	9,613609	9,82972	9,728807	9,512877	9,657062	9,708852	9,7240453	9,626263667	-0,09778167	0,934468756	Cenpe
17202661	3,86629	3,453525	4,200724	3,319993	3,737902	4,169358	3,8401797	3,742417667	-0,097762	0,934481495	---
17398732	2,937702	3,113981	3,118685	3,025035	3,012819	2,839249	3,0567893	2,959034333	-0,097755	0,934486029	---
17204209	3,234948	3,246903	3,290392	3,230367	3,044502	3,204117	3,2574143	3,159662	-0,09775233	0,934487756	---
17504958	6,477754	6,780622	5,986547	6,107829	6,362548	6,481123	6,4149183	6,317166667	-0,09775167	0,934488188	Nutf2
17410982	4,638175	4,68385	4,569813	4,723122	4,446132	4,429345	4,6306127	4,532866333	-0,09774633	0,934491643	Gm20271
17348356	7,726821	7,689242	7,824446	7,593917	7,803931	7,549424	7,7468363	7,649090667	-0,09774567	0,934492074	Snrpd1

17268786	5,614807	5,759143	5,594833	5,460062	5,693389	5,52216	5,656261	5,558537	-0,097724	0,934506109	Neurod2
17424811	3,712032	3,677235	3,773598	3,595591	3,684074	3,590054	3,720955	3,623239667	-0,09771533	0,934511723	Olfr159
17493292	8,797992	8,791886	8,776067	8,686309	8,492432	8,894221	8,7886483	8,690987333	-0,097661	0,934546918	Tmem126a
17360599	5,344383	5,501837	5,676728	5,37032	5,5502	5,309476	5,5076493	5,409998667	-0,09765067	0,934553612	Adrb1
17301010	8,5982	8,668039	8,637161	8,52667	8,642053	8,441734	8,6344667	8,536819	-0,09764767	0,934555555	Mtmr6
17200347	3,105409	3,455424	3,39799	3,23077	3,421503	3,013662	3,3196077	3,221978333	-0,09762933	0,934567431	---
17200013	4,912636	5,069479	5,32028	4,426407	5,05061	5,532502	5,1007983	5,003173	-0,09762533	0,934570022	---
17516222	3,113935	3,115842	3,315359	3,066367	3,165637	3,020425	3,181712	3,084143	-0,097569	0,934606516	Olfr921
17537780	4,520919	4,169375	4,310485	3,972593	4,328794	4,406741	4,333593	4,236042667	-0,09755033	0,934618608	---
17515004	5,876313	6,205668	6,186225	5,932844	6,02293	6,01983	6,089402	5,991868	-0,097534	0,93462919	Fbxl12
17287206	10,01377	10,16982	10,04995	10,02172	9,965824	9,953615	10,077847	9,980386333	-0,09746033	0,934676915	Iars
17417767	8,642002	8,647749	8,67244	8,548674	8,574775	8,546388	8,6540637	8,556612333	-0,09745133	0,934682745	Elovl1
17468405	5,796717	5,745835	5,958922	5,786672	5,830513	5,591938	5,8338247	5,736374333	-0,09745033	0,934683393	Egr4
17446307	9,860342	9,868334	9,775994	9,731215	9,742526	9,738606	9,83489	9,737449	-0,097441	0,93468944	Rheb
17485975	5,195912	5,235439	5,371314	5,291772	4,99475	5,223844	5,267555	5,170122	-0,097433	0,934694623	Zfp579
17494774	3,586755	3,617548	3,453211	3,404209	3,57699	3,384025	3,5525047	3,455074667	-0,09743	0,934696567	Olfr471
17277177	5,532317	5,634203	5,588015	5,442198	5,472512	5,547569	5,584845	5,487426333	-0,09741867	0,93470391	Dnalc1
17365233	4,287031	4,730713	4,28096	4,326704	4,393455	4,286294	4,4329013	4,335484333	-0,097417	0,934704989	Fgf8
17356987	8,411822	8,292941	8,262961	8,091012	8,411684	8,172789	8,3225747	8,225161667	-0,097413	0,934707581	Ppp1r14b
17438423	10,72651	10,77273	10,7291	10,74101	10,61627	10,5789	10,74278	10,64539333	-0,09738667	0,934724642	Paics
17513548	4,880762	4,866745	5,219757	4,907557	5,225182	4,542377	4,989088	4,891705333	-0,09738267	0,934727234	---
17404923	5,778504	5,528995	5,773663	5,653459	5,543921	5,591642	5,6937207	5,596340667	-0,09738	0,934728962	Gm5148
17458885	4,68097	4,741708	4,89427	4,778343	4,696245	4,550226	4,772316	4,674938	-0,097378	0,934730257	Ppp1r17
17508512	5,305701	5,301729	5,670269	5,097379	5,553118	5,335142	5,4258997	5,328546333	-0,09735333	0,934746239	---
17284194	5,910577	5,76238	5,928061	5,594877	5,835976	5,878161	5,867006	5,769671333	-0,09733467	0,934758334	Tmem179
17507487	5,41888	4,92133	5,698282	5,254116	5,086591	5,405792	5,346164	5,248833	-0,097331	0,934760709	---
17443060	5,592546	5,430305	5,482203	5,32667	5,793602	5,092821	5,5016847	5,404364333	-0,09732033	0,934767621	---
17205779	7,14776	7,126069	7,44864	7,492175	6,973938	6,964466	7,240823	7,143526333	-0,09729667	0,934782955	---
17547817	4,291705	3,807737	4,084802	3,982638	3,840152	4,069598	4,0614147	3,964129333	-0,09728533	0,934790299	1700054O19Rik
17280893	9,795455	9,745375	9,386292	9,543562	9,488895	9,602879	9,642374	9,545112	-0,097262	0,934805417	Dnajb9
17308746	8,101444	8,149368	8,103076	7,969197	8,164235	7,928682	8,1179627	8,020704667	-0,097258	0,934808009	1200011I18Rik
17548166	3,145125	3,058992	3,116134	3,046187	2,965631	3,016822	3,1067503	3,009546667	-0,09720367	0,934843216	LOC100862491
17372777	3,905799	3,930296	3,77979	3,816686	3,785222	3,722454	3,8719617	3,774787333	-0,09717433	0,934862223	Olfr1026
17345519	8,067152	8,084011	8,139382	7,930847	8,163633	7,904636	8,0968483	7,999705333	-0,097143	0,934882528	Rrp36
17381300	6,55664	6,440455	6,085044	6,485429	5,993923	6,311368	6,360713	6,263573333	-0,09713967	0,934884688	---
17226622	8,721833	8,80937	8,811391	8,451525	8,803867	8,795898	8,7808647	8,683763333	-0,09710133	0,934909528	Cd55
17293717	4,398015	4,282811	4,147029	4,090704	4,297621	4,148258	4,2759517	4,178861	-0,09709067	0,934916441	1190003K10Rik
17215623	5,432685	5,020508	5,234841	5,172408	5,083533	5,141001	5,2293447	5,132314	-0,09703067	0,934955324	---
17306104	4,161349	4,471206	4,156964	4,367116	4,042032	4,089305	4,263173	4,166151	-0,097022	0,93496094	Ear11
17315397	7,194799	7,089145	7,194865	6,964039	7,208933	7,014781	7,159603	7,062584333	-0,09701867	0,9349631	Myg1
17450157	7,285115	7,429241	7,487364	7,369534	7,261253	7,279883	7,4005733	7,303556667	-0,09701667	0,934964397	Helq
17384559	4,462162	4,675738	4,675738	4,46483	4,400098	4,657688	4,604546	4,507538667	-0,09700733	0,934970445	Olfr341
17215101	6,75262	6,631665	6,823298	6,753714	6,562978	6,599875	6,735861	6,638855667	-0,09700533	0,934971741	Gm5258
17301086	5,511889	5,540199	5,506465	5,378522	5,307259	5,581768	5,5195177	5,422516333	-0,09700133	0,934974334	Sacs
17453535	6,180633	6,208248	6,456151	6,163811	6,059304	6,33099	6,2816773	6,184701667	-0,09697567	0,934990968	Vps37d
17265422	9,733524	9,884774	9,765045	9,576141	9,747364	9,768915	9,7944477	9,697473333	-0,09697433	0,934991832	C1qbp
17549378	5,746837	5,726778	5,763299	5,567813	5,791946	5,586246	5,745638	5,648668333	-0,09696967	0,934994856	---
17271692	6,71036	6,452486	6,950549	6,389535	6,654109	6,778888	6,704465	6,607510667	-0,09695433	0,935004794	---
17294899	6,025996	6,114778	5,996455	5,926564	5,779696	6,140117	6,045743	5,948792333	-0,09695067	0,93500717	Fam151b
17511293	5,617444	5,538256	5,811386	5,545151	5,706072	5,425094	5,6556953	5,558772333	-0,096923	0,935025101	Gm5741
17250852	5,975875	5,789722	5,742887	5,817312	5,781801	5,618623	5,8361613	5,739245333	-0,096916	0,935029638	B130011K05Rik
17548348	9,995983	10,28207	9,922934	9,787168	9,971342	10,15173	10,066996	9,97008	-0,09691567	0,935029854	Actg1 // Actg1
17322642	7,688614	7,698307	7,712414	7,482482	7,689621	7,636496	7,6997783	7,602866333	-0,096912	0,93503223	Ubn1
17304177	8,802834	8,783517	8,657636	8,61988	8,532025	8,801351	8,7479957	8,651085333	-0,09691033	0,93503331	D14Ertd449e
17453500	5,043925	4,663314	5,166858	4,92887	4,932109	4,722504	4,9580323	4,861161	-0,09687133	0,935058587	Wbscr25
17368387	10,19478	10,27438	10,38542	10,18177	10,25567	10,12655	10,28486	10,18799667	-0,09686333	0,935063772	---
17468959	10,80327	11,08007	10,84029	10,73577	10,74085	10,95643	10,907877	10,81101667	-0,09686	0,935065933	---
17373189	5,161635	5,440773	5,460826	5,300871	5,222703	5,249117	5,3544113	5,257563667	-0,09684767	0,935073926	Mybpc3
17207775	6,555961	6,745196	6,492001	6,688626	6,307189	6,506814	6,5977193	6,500876333	-0,096843	0,935076951	---
17288836	5,897744	5,427259	5,02563	6,103943	4,596515	5,359654	5,450211	5,353370667	-0,09684033	0,93507868	---
17208281	6,014461	6,784856	7,58497	7,193144	6,226346	6,674306	6,7947623	6,697932	-0,09683033	0,935085161	---
17288185	4,724278	4,252258	4,158442	4,391096	4,320177	4,133216	4,378326	4,281496333	-0,09682967	0,935085593	Olfr466
17233113	8,349186	8,329914	8,166998	8,005272	8,23015	8,320187	8,2820327	8,185203	-0,09682967	0,935085593	Hace1
17202807	6,984996	7,096492	6,511063	6,450953	7,031919	6,819194	6,8641837	6,767355333	-0,09682833	0,935086457	---
17280869	7,837008	7,976267	7,978536	7,786573	7,829094	7,885665	7,9306037	7,833777333	-0,09682633	0,935087754	Zfp277
17355228	5,100321	5,109785	5,429224	5,18531	5,159593	5,004011	5,21311	5,116304667	-0,09680533	0,935101365	Psmg2 // Psmg2
17293265	4,510081	4,239564	4,365207	4,224648	4,304068	4,295727	4,3716173	4,274814333	-0,096803	0,935102877	4932411G14Rik
17494677	4,753576	5,238906	4,872308	5,123154	4,81253	4,638716	4,95493	4,858133333	-0,09679667	0,935106982	Gm1966
17505453	8,371112	8,532393	8,475971	8,243848	8,423835	8,421479	8,4598253	8,363054	-0,09677133	0,935123403	Snord71
17401833	8,719931	8,679676	8,587385	8,544155	8,566459	8,586071	8,6623307	8,565561667	-0,096769	0,935124915	Slc25a24
17394446	7,250566	7,386669	7,445037	7,145249	7,308379	7,338398	7,3607573	7,264008667	-0,09674867	0,935138095	Zfp334
17524914	7,194293	7,304026	7,055884	6,956736	7,165976	7,14126	7,1847343	7,087990667	-0,09674367	0,935141336	Ecsit
17549248	4,771934	4,727086	4,915646	4,699897	4,835312	4,589264	4,8048887	4,708157667	-0,096731	0,935149546	---
17448168	3,31417	3,415513	3,530766	3,05821	3,489228	3,422859	3,4201497	3,323432333	-0,09671733	0,935158405	---
17405471	3,590249	3,853442	3,913107	3,586482	3,863739	3,616429	3,7855993	3,688883333	-0,096716	0,935159269	Gpr87
17421437	4,730092	4,534336	4,618383	4,565004	4,485078	4,542595	4,6276037	4,530892333	-0,09671133	0,935162294	Gm13247
17315395	10,18711	10,41209	10,18491	10,08397	10,24852	10,1615	10,26137	10,16466333	-0,09670667	0,935165319	---
17550747	3,611286	3,535915	3,484744	3,326241	3,433244	3,582385	3,5439817	3,44729	-0,09669167	0,935175042	---
17331514	3,42409	3,671165	3,36728	3,385432	3,52779	3,259306	3,4875117	3,390842667	-0,096669	0,935189735	---
17317208	9,067673	9,458889	9,063446	9,281636	9,000606	9,017777	9,1966693	9,100006333	-0,096663	0,935193625	---
17407669	8,890593	9,058118	8,869993	8,675566	9,020881	8,832368	8,939568	8,842938333	-0,09662967	0,935215232	Psmd4
17346503	5,050025	4,949233	5,358616	4,967781	5,209136	4,891083	5,1192913	5,022666667	-0,09662467	0,935218474	Tubb4a
17508688	5,507445	5,27154	5,114244	4,937737	5,403666	5,261958	5,297743	5,201120333	-0,09662267	0,93521977	2700090O03Rik
17448810	7,999653	9,829757	9,515414	10,24161	8,26063	8,552752	9,1149413	9,018330667	-0,09661067	0,935227549	---
17445381	8,298316	8,38453	8,137061	8,114184	8,192945	8,223004	8,2733023	8,176711	-0,09659133	0,935240082	Gtpbp10
17206027	5,124755	4,883276	5,001981	4,718247	4,760503	5,241523	5,0033373	4,906757667	-0,09657967	0,935247645	---
17267172	6,190954	5,900469	6,083705	5,94238	6,011196	5,931882	6,058376	5,961819333	-0,09655667	0,935262555	---
17418236	5,074824	5,042835	5,116251	4,790422	5,029963	5,123869	5,07797	4,981418	-0,096552	0,935265581	---
17433145	5,111274	5,215015	5,172127	4,981493	5,182394	5,044936	5,1661387	5,069607667	-0,096531	0,935279195	Rbp7
17502335	6,364805	6,111057	6,280451	6,124084	6,150321	6,192326	6,2521043	6,155577	-0,09652733	0,935281572	Zfp882
17324961	6,459405	6,40206	6,617481	6,296853	6,501596	6,390925	6,492982	6,396458	-0,096524	0,935283733	Rpl35a
17547994	4,475521	4,118232	4,313112	4,30517	4,075	4,237153	4,3022883	4,205774333	-0,096514	0,935290215	LOC100862171
17225179	8,329588	8,273058	8,410507	8,290005	8,331012	8,102596	8,3377177	8,241204333	-0,09651333	0,935290648	Pde6d
17543045	9,061098	9,096775	9,11189	8,935827	9,048347	8,99606	9,089921	8,993411333	-0,09650967	0,935293025	Gyk
17456868	4,585906	4,402601	4,514977	4,491787	4,561798	4,16039	4,5011613	4,404658333	-0,096503	0,935297347	Cpa2
17484565	3,887089	3,94633	3,740396	3,685458	4,028775	3,570083	3,8579383	3,761438667	-0,09649967	0,935299508	Olfr46
17471107	5,748518	5,747667	5,852058	5,555376	5,711221	5,792162	5,7827477	5,686253	-0,09649467	0,935302749	Plekhg6
17407501	4,300248	4,043863	4,427468	4,169651	4,13776	4,174756	4,257193	4,160722333	-0,09647067	0,935318309	Lce1m
17504413	5,505248	5,499853	5,568762	5,352057	5,442895	5,489605	5,524621	5,428185667	-0,09643533	0,935341216	Bean1
17249811	7,572884	7,281384	7,510042	7,333106	7,340052	7,401863	7,45477	7,358340333	-0,09642967	0,93534489	Slc36a1
17447145	8,296734	8,198143	8,146467	8,044671	8,123245	8,184156	8,2137813	8,117357333	-0,096424	0,935348564	---
17354728	4,359118	3,673757	4,052345	3,664227	4,073092	4,058669	4,0284067	3,931996	-0,09641067	0,935357208	---
17521648	5,159005	4,760853	5,044669	4,835474	4,896237	4,943599	4,9881757	4,89177	-0,09640567	0,93536045	Bsn // Bsn
17444150	3,018263	3,079603	2,904868	2,841701	2,972787	2,899072	3,0009113	2,90452	-0,09639133	0,935369743	---
17278128	4,693974	4,861814	4,798064	4,599391	4,597563	4,867751	4,7846173	4,688235	-0,09638233	0,935375578	Unc79
17530669	8,121555	8,166411	8,055252	7,993842	8,103077	7,957154	8,114406	8,018024333	-0,09638167	0,93537601	Acy1
17390665	5,048303	4,884472	5,114878	5,038378	4,695897	5,024263	5,0158843	4,919512667	-0,09637167	0,935382494	---
17202819	7,63871	7,428949	7,632693	7,343174	7,428822	7,639303	7,566784	7,470433	-0,096351	0,935395893	---
17509752	6,588039	6,524265	6,493993	6,554763	6,444063	6,318446	6,5354323	6,439090667	-0,09634167	0,935401945	---
17302970	6,203763	6,0591	5,795711	5,933022	5,841242	5,995317	6,0195247	5,923193667	-0,096331	0,935408861	---
17285242	5,610283	5,749449	6,410615	5,794016	5,798444	5,88891	5,923449	5,827123333	-0,09632567	0,935412319	---
17203751	4,067621	4,940446	4,399314	4,390404	3,823756	4,904306	4,469127	4,372822	-0,096305	0,935425719	---
17540027	11,94582	11,9281	11,91504	11,85318	11,83797	11,80895	11,929653	11,83336667	-0,09628667	0,935437606	Gm15453 // Rbm3
17367895	9,930978	9,866465	10,22163	10,23132	9,513058	9,985869	10,006358	9,910082333	-0,09627533	0,935444954	---
17438649	3,947354	3,861997	3,889484	4,063813	3,694365	3,651848	3,8996117	3,803342	-0,09626967	0,935448629	Csn1s2a
17465711	6,409633	6,497419	6,660106	6,262351	6,746122	6,269882	6,522386	6,426118333	-0,09626767	0,935449925	Akr1b3
17375387	3,703195	3,605783	3,93033	3,632165	3,632111	3,686239	3,746436	3,650171667	-0,09626433	0,935452087	4933406J08Rik
17208661	6,617261	6,370507	6,53028	6,529783	6,100534	6,599014	6,506016	6,409777	-0,096239	0,935468513	---
17299027	5,763322	5,73997	6,58097	6,071079	5,93898	5,785498	6,0280873	5,931852333	-0,096235	0,935471107	Gm2959
17534335	6,954484	7,103793	7,47574	7,113012	7,099011	7,033322	7,1780057	7,081781667	-0,096224	0,93547824	Mir3110 // Mir3110
17283624	7,639946	8,020351	8,025359	7,85567	7,848865	7,692472	7,8952187	7,799002333	-0,09621633	0,935483211	Serpina1a
17252800	8,231767	8,081845	7,926269	7,984987	7,90856	8,057711	8,0799603	7,983752667	-0,09620767	0,935488831	Mettl16
17397783	9,980647	10,05548	10,00717	9,896071	9,865805	9,992802	10,014432	9,918226	-0,09620633	0,935489695	Eif2a
17236490	5,763617	5,430723	5,877303	5,51249	5,539138	5,731414	5,6905477	5,594347333	-0,09620033	0,935493586	Tmpo
17269218	6,348133	6,220388	6,491668	6,428975	6,148983	6,193632	6,3533963	6,257196667	-0,09619967	0,935494018	---
17224942	4,531975	4,72342	4,749849	4,683238	4,496251	4,537188	4,6684147	4,572225667	-0,096189	0,935500935	Slc19a3
17277863	5,44875	5,349958	5,327437	5,26703	5,212459	5,358115	5,3753817	5,279201333	-0,09618033	0,935506555	---
17258155	5,685908	5,773568	5,759331	5,596465	5,709909	5,623907	5,7396023	5,643427	-0,09617533	0,935509797	Otop2
17441726	5,455053	5,41793	5,636256	5,303027	5,640446	5,277298	5,5030797	5,406923667	-0,096156	0,935522333	Rasal1
17265365	6,004547	5,77799	6,099054	5,661522	5,896863	6,034774	5,9605303	5,864386333	-0,096144	0,935530115	Inca1
17227261	5,202811	4,818497	5,34564	5,107059	4,93614	5,035355	5,122316	5,026184667	-0,09613133	0,935538329	Gpr37l1
17431350	7,867413	8,096733	8,184722	7,929834	8,042682	7,887984	8,0496227	7,9535	-0,09612267	0,935543949	Ldlrap1
17443342	5,154787	4,705558	5,213819	5,161465	4,971164	4,653169	5,0247213	4,928599333	-0,096122	0,935544381	Hspb1
17269006	4,712792	4,692463	4,768053	4,853039	4,48095	4,550992	4,724436	4,628327	-0,096109	0,935552811	Krt24

17467106	3,116182	3,093015	2,873337	2,833444	3,218497	2,742323	3,0275113	2,931421333	-0,09609	0,935565132	---
17296466	5,95031	5,721288	6,356945	5,823433	6,04168	5,875209	6,0095143	5,913440667	-0,09607367	0,935575724	Emb // Emb
17471936	4,637373	4,809312	4,970972	4,63372	4,831572	4,664144	4,8058857	4,709812	-0,09607367	0,935575724	Prpmp5
17207895	7,447283	7,526393	7,279285	7,387077	7,269333	7,308377	7,4176537	7,321595667	-0,096058	0,935585884	---
17461581	4,38676	4,197786	4,525893	4,260322	4,220798	4,34123	4,3701463	4,274116667	-0,09602967	0,935604258	Cpne9
17273804	9,508381	9,721701	9,564316	9,41794	9,291143	9,797242	9,5981327	9,502108333	-0,09602433	0,935607717	Gm6682
17206735	5,094318	5,6158	5,77211	5,292355	5,501872	5,399946	5,494076	5,398057667	-0,09601833	0,935611608	---
17250507	5,618745	5,122331	5,490139	5,466913	5,06506	5,411189	5,410405	5,314387333	-0,09601767	0,935612041	Mir5100
17315219	5,579164	5,452583	5,983559	5,391338	5,560062	5,775867	5,6717687	5,575755667	-0,096013	0,935615067	1700011A15Rik
17299589	5,922854	5,716631	5,924267	5,790895	6,011691	5,473202	5,854584	5,758596	-0,095988	0,93563128	Ear5
17509997	7,911566	8,080522	8,136279	7,920865	7,923114	7,996471	8,042789	7,946816667	-0,09597233	0,935641441	Ddx49
17547567	7,739776	7,886334	7,88564	7,679753	7,770533	7,773552	7,83725	7,741279333	-0,09597067	0,935642521	---
17549936	3,559355	3,453524	3,44084	3,303816	3,494795	3,367236	3,484573	3,388615667	-0,09595733	0,935651169	---
17234861	3,435204	3,528868	3,389981	3,419291	3,367316	3,279591	3,451351	3,355399333	-0,09595167	0,935654844	Olfr57
17496615	7,241476	7,372186	7,302998	7,154531	7,310316	7,163978	7,3055533	7,209608333	-0,095945	0,935659167	Tbc1d10b
17517888	6,046102	5,855963	5,932569	5,859939	5,665946	6,020942	5,944878	5,848942333	-0,09593567	0,935665221	Stoml1
17516231	3,936698	4,07887	4,102879	4,10384	3,842591	3,884235	4,0394823	3,943555333	-0,095927	0,935670841	Olfr923
17275255	4,327216	4,42711	4,253303	4,37651	4,001142	4,342224	4,3358763	4,239958667	-0,09591767	0,935676895	Nrcam
17545817	3,270741	3,13748	3,490139	3,235622	3,20717	3,167825	3,2994533	3,203539	-0,09591433	0,935679056	---
17468075	9,029156	9,129132	9,24312	9,133016	8,929411	9,051242	9,1338027	9,037889667	-0,095913	0,935679921	Htra2
17266728	3,693142	3,70449	4,015566	3,632957	3,785254	3,707252	3,8043993	3,708487667	-0,09591167	0,935680786	---
17503800	4,956799	4,799585	5,006333	4,725055	4,991074	4,758868	4,9209057	4,824999	-0,09590667	0,935684029	Irx3
17451443	9,378587	9,595667	9,543488	9,451666	9,327713	9,450675	9,505914	9,410018	-0,095896	0,935690947	Coro1c
17492495	7,061447	7,234808	7,495334	7,18712	7,201416	7,115389	7,263863	7,167975	-0,095888	0,935696135	Cib1
17323064	6,751106	8,135707	7,486733	7,736794	7,573962	6,775134	7,4578487	7,361963333	-0,09588533	0,935697865	---
17443890	3,574349	3,689483	3,623299	3,403742	3,687537	3,508229	3,6290437	3,533169333	-0,09587433	0,935704999	Zfp68 // Zfp68
17317688	5,987135	5,716725	6,017163	5,588298	5,962746	5,882378	5,9070077	5,811140667	-0,095867	0,935709756	Zfat
17377028	6,802936	6,668084	6,511161	6,525462	6,718848	6,450308	6,660727	6,564872667	-0,09585433	0,935717971	8430406I07Rik
17411966	4,112126	3,951576	3,998025	3,984766	3,951876	3,837609	4,0205757	3,924750333	-0,09582533	0,93573678	Calb1
17426348	5,318188	5,27036	5,172909	4,9328	5,313437	5,227798	5,253819	5,158011667	-0,09580733	0,935748455	Tnfsf15
17333703	3,853281	3,743808	3,892076	3,646538	3,825838	3,729466	3,8297217	3,733947333	-0,09577433	0,93576986	---
17365656	2,896021	3,023578	2,950204	2,868883	2,778994	2,934609	2,956601	2,860828667	-0,09577233	0,935771157	---
17240670	5,667415	6,216891	5,875887	5,742152	5,988572	5,742155	5,9200643	5,824293	-0,09577133	0,935771806	Lin28b
17340967	8,626369	8,444352	8,625278	8,595776	8,380882	8,432041	8,565333	8,469566333	-0,09576667	0,935774833	Acat2
17363374	9,638854	9,549743	9,411321	9,382086	9,553502	9,377035	9,533306	9,437541	-0,095765	0,935775914	Ostf1
17205763	4,380401	4,514112	4,358439	4,076158	4,655364	4,23414	4,4176507	4,321887333	-0,09576333	0,935776995	---
17418447	7,311995	7,393984	7,263581	7,081759	7,248979	7,351579	7,3231867	7,227439	-0,09574767	0,935787157	Meaf6
17349421	3,816802	3,632952	3,940481	3,702703	3,895594	3,504735	3,796745	3,701010667	-0,09573433	0,935795805	---
17450117	6,803926	6,70507	6,75344	6,684952	6,611981	6,678317	6,7541453	6,658416667	-0,09572867	0,935799481	---
17549132	3,882515	3,880947	4,070607	3,701654	3,877391	3,967917	3,9446897	3,848987333	-0,09570233	0,935816562	---
17208455	3,474404	3,7299	3,41525	3,902361	3,184609	3,245486	3,5398513	3,444152	-0,09569933	0,935818508	---
17348638	5,758152	6,063681	5,831086	5,767983	5,810475	5,787401	5,8843063	5,788619667	-0,09568667	0,935826724	Psma8
17218942	4,967437	5,052492	4,947145	5,015125	4,776503	4,888401	4,9890247	4,893343	-0,09568167	0,935829968	Gpr161
17516194	8,811708	9,061407	8,391029	8,576777	8,731615	8,66874	8,7547147	8,659044	-0,09567067	0,935837103	Vwa5a // Vwa5a
17286318	3,270435	3,278466	3,287882	2,997038	3,136406	3,416381	3,2789277	3,183275	-0,09565267	0,935848779	---
17546997	3,228256	3,879576	3,563027	3,247295	3,780819	3,355802	3,556953	3,461305333	-0,09564767	0,935852023	LOC100039753
17201139	7,902915	8,539249	8,464035	8,111285	8,072588	8,435458	8,3020663	8,206443667	-0,09562267	0,93586824	---
17269146	3,848664	3,579674	3,636703	3,814506	3,37993	3,583752	3,688347	3,592729333	-0,09561767	0,935871483	Krtap1-3
17499310	10,11439	10,28325	10,24607	10,05548	10,14231	10,15912	10,21457	10,11897	-0,0956	0,935882944	Tfdp1
17501508	6,599073	6,545976	6,566901	6,438603	6,470517	6,516062	6,57065	6,475060667	-0,09558933	0,935889863	Tmem192
17501214	3,474993	4,026551	3,9418	3,609703	3,897973	3,648977	3,814448	3,718884333	-0,09556367	0,935906514	LOC100862211
17220771	5,474385	5,722803	5,805388	5,347659	5,507413	5,860892	5,6675253	5,571988	-0,09553733	0,935923597	1700022P22Rik
17202801	6,110714	5,955887	5,310339	5,941149	5,228703	5,920538	5,7923133	5,696796667	-0,09551667	0,935937004	---
17250341	6,259763	6,528687	6,36447	6,421409	5,840163	6,604824	6,3843067	6,288798667	-0,095508	0,935942626	Tom1l2 // Tom1l2
17405005	8,021869	7,970242	7,918958	7,7609	7,863876	7,999812	7,9703563	7,874862667	-0,09549367	0,935951925	Pgrmc2
17322874	7,159615	7,034534	6,968874	6,930286	6,894916	7,051343	7,054341	6,958848333	-0,09549267	0,935952574	Clec16a
17504715	9,199194	9,175079	9,269958	9,13687	9,059956	9,160986	9,2147437	9,119270667	-0,095473	0,935965333	E2f4
17511453	8,066236	8,122251	7,814402	7,929475	7,885631	7,901399	8,000963	7,905501667	-0,09546133	0,935972902	N4bp1
17309061	6,186938	5,576332	5,693224	5,412893	6,031566	5,725681	5,8188313	5,72338	-0,09545133	0,935979389	---
17459370	3,295818	3,226818	3,439753	3,221864	3,221864	3,232347	3,3207963	3,225358333	-0,095438	0,93598804	---
17403829	5,738823	5,487343	5,729623	5,737107	5,53138	5,401008	5,6519297	5,556498333	-0,09543133	0,935992365	Negr1
17550791	3,404853	3,630394	3,760267	3,601175	3,346551	3,561506	3,5985047	3,503077333	-0,09542733	0,93599496	---
17411135	4,313868	4,492134	4,239349	4,23667	4,305557	4,216882	4,3484503	4,253036333	-0,095414	0,936003611	---
17262255	4,204806	3,931166	4,189004	3,984876	3,937797	4,116068	4,1083253	4,012913667	-0,09541167	0,936005124	Olfr1396
17217771	7,887205	7,612444	7,517134	7,667725	7,541835	7,520989	7,672261	7,576849667	-0,09541133	0,936005341	Gm3878
17551121	9,172593	9,374431	9,292411	9,072861	8,964464	9,515923	9,2798117	9,184416	-0,09539567	0,936015505	---
17459696	4,782769	4,792278	4,683331	4,442253	4,907047	4,622896	4,7527927	4,657398667	-0,095394	0,936016586	Gm15401
17280125	7,216196	7,436198	7,063216	7,073562	7,278737	7,077168	7,2385367	7,143155667	-0,095381	0,936025021	Pqlc3
17254395	8,48065	8,515303	8,242045	8,354405	8,192738	8,404739	8,412666	8,317294	-0,095372	0,93603086	Acaca
17539844	4,385238	3,996642	3,942207	3,893604	4,023741	4,120663	4,108029	4,012669333	-0,09535967	0,936038862	Ppp1r3f
17427585	10,24996	10,36442	10,27615	10,23468	10,18715	10,18275	10,296843	10,20152667	-0,09531667	0,936066761	Jak1
17248501	3,509851	3,708174	3,354932	3,697869	3,302667	3,286529	3,524319	3,429021667	-0,09529733	0,936079306	---
17408001	6,57213	6,519241	6,861372	6,617043	6,576118	6,47369	6,6509143	6,555617	-0,09529733	0,936079306	Bola1
17547499	5,347476	5,14965	5,315642	5,099104	5,313433	5,114543	5,2709227	5,175693333	-0,09522933	0,936123428	Adamts14 // Adamts14
17453617	7,676279	7,808321	8,004766	7,755765	7,846607	7,601314	7,8297887	7,734562	-0,09522667	0,936125158	Tmem120a
17466507	9,800358	9,811487	9,8258	9,761644	9,657606	9,732738	9,8125483	9,717329333	-0,095219	0,936130133	Ezh2
17328062	7,515265	7,614736	7,612151	7,644868	7,447002	7,364625	7,5807173	7,485498333	-0,095219	0,936130133	Emp2
17405130	5,863275	5,539793	5,50423	5,479149	5,627632	5,514872	5,635766	5,540551	-0,095215	0,936132728	Gm9442
17259516	9,018732	9,304374	9,24402	9,037148	9,310618	8,933777	9,189042	9,093847667	-0,09519433	0,936146139	---
17204989	4,809381	4,304466	5,373744	5,521767	3,430097	5,250229	4,829197	4,734031	-0,095166	0,936164524	---
17316700	10,31056	10,43326	10,22165	10,18223	10,19542	10,30235	10,321823	10,22666667	-0,09515667	0,93617058	Azin1
17381509	4,559725	4,905061	4,681795	4,493514	4,714763	4,652839	4,715527	4,620372	-0,095155	0,936171662	5031426D15Rik
17312800	6,74697	6,688079	6,843199	6,714283	6,673143	6,605368	6,759416	6,664264667	-0,09515133	0,936174041	Sh3bp1
17276254	9,805318	9,771125	9,886236	9,674231	9,677757	9,825294	9,820893	9,725760667	-0,09513233	0,93618637	---
17233811	7,965261	8,087436	8,043789	7,896141	7,941318	7,973673	8,032162	7,937044	-0,095118	0,936195672	Dna2
17217602	6,001779	5,690977	5,944035	5,712253	5,662423	5,976775	5,8789303	5,783817	-0,09511333	0,9361987	Nav1
17444938	6,447055	6,045354	6,168749	6,01817	6,028224	6,329429	6,220386	6,125274333	-0,09511167	0,936199781	---
17534669	3,874984	3,872528	4,048429	3,86138	3,947762	3,701471	3,9319803	3,836871	-0,09510933	0,936201296	---
17203687	5,07078	5,115364	5,80105	4,643438	4,935778	6,122671	5,3290647	5,233962333	-0,09510233	0,936205838	---
17349402	5,569756	5,981376	5,626207	5,580748	5,54984	5,761478	5,7257797	5,630688667	-0,095091	0,936213193	Pkd2l2
17549410	7,527542	7,777559	7,82199	7,65314	7,559253	7,629436	7,7090303	7,613943	-0,09508733	0,936215572	---
17549788	7,527542	7,777559	7,82199	7,65314	7,559253	7,629436	7,7090303	7,613943	-0,09508733	0,936215572	---
17216172	3,448042	3,252936	3,281023	3,071886	3,453524	3,171398	3,3273337	3,232269333	-0,09506433	0,936230498	Gm7133
17481958	3,67986	4,121428	3,992738	3,478253	4,2884	3,742199	3,931342	3,836284	-0,095058	0,936234608	---
17212888	9,070198	9,078876	8,816658	8,894971	8,80477	8,980828	8,9885773	8,893523	-0,09505433	0,936236987	Mob4
17548755	3,202303	3,296627	3,255241	3,243584	3,098588	3,126871	3,2513903	3,156347667	-0,09504267	0,936244558	Gm20265
17259512	8,987093	9,215143	9,173909	8,961759	9,092926	9,036385	9,1253817	9,030356667	-0,095025	0,936256023	Gps1
17463106	5,772542	5,856206	5,831117	5,932688	5,669261	5,573014	5,819955	5,724987667	-0,09496733	0,936293448	---
17229520	8,479468	8,62317	8,478107	8,51638	8,3384	8,441122	8,526915	8,431967333	-0,09494767	0,936306211	Uhmk1
17522726	5,052704	4,835831	4,786562	4,715562	5,035975	4,638782	4,891699	4,796773	-0,094926	0,936320273	Bcl2a1c
17508846	5,610039	4,907903	5,333066	5,383618	4,990398	5,192266	5,2836693	5,188760667	-0,09490867	0,936331522	Mir383
17407492	2,789692	3,113581	2,884402	2,828019	2,897045	2,777929	2,929225	2,834331	-0,094894	0,936341041	---
17414554	8,030458	8,147	8,060699	8,025385	8,010957	7,917183	8,0793857	7,984508333	-0,09487733	0,936351858	Prpf4
17336559	6,882349	6,7726	6,885963	6,834107	6,629746	6,792432	6,8469707	6,752095	-0,09487567	0,93635294	Notch4
17506332	7,729764	7,828804	7,649044	7,827408	7,467974	7,627611	7,7358707	7,640997667	-0,094873	0,936354671	Banp
17507435	7,137932	7,173891	7,038397	6,941284	7,056534	7,067802	7,11674	7,021873333	-0,09486667	0,936358781	Rab20
17469274	5,2757	5,074933	4,977425	4,841725	5,011445	5,190288	5,1093527	5,014486	-0,09486667	0,936358781	1700123L14Rik
17332473	6,45985	6,308604	6,649932	6,487184	6,556428	6,090197	6,4727953	6,377936333	-0,094859	0,936363757	Itgb2l
17404077	4,320177	4,691273	3,849075	4,02531	4,167076	4,383564	4,2868417	4,191983333	-0,09485833	0,93636419	Pmp2
17354233	8,434999	8,587047	8,27742	8,484653	8,249057	8,281225	8,4331553	8,338311667	-0,09484367	0,936373709	Pggt1b
17488343	5,295577	5,248446	5,261242	5,139171	5,307077	5,074541	5,2684217	5,173596333	-0,09482533	0,936385609	Gm4636
17202851	3,311559	2,990185	3,236137	3,619172	2,934358	2,699908	3,1792937	3,084479333	-0,09481433	0,936392748	---
17543829	9,22535	9,362152	9,251927	9,127359	9,234797	9,192869	9,2798097	9,185008333	-0,09480133	0,936401186	Rlim
17548462	5,298708	5,256791	5,402804	5,083828	5,293398	5,296673	5,3194343	5,224633	-0,09480133	0,936401186	D130050E23Rik
17551079	9,025648	9,248545	9,070682	9,042459	9,021359	8,996799	9,1149583	9,020205667	-0,09475267	0,936432774	---
17457308	4,135893	3,865559	3,982055	3,823158	3,806723	4,06938	3,9945023	3,899753667	-0,09474867	0,936435371	---
17528581	6,015024	6,048914	6,329539	6,020832	6,04155	6,046958	6,131159	6,036446667	-0,09471233	0,936458954	---
17305116	9,409772	9,150725	9,061353	9,003386	9,150258	9,184079	9,2072833	9,112574333	-0,094709	0,936461118	Gcap14
17525296	4,849145	4,65438	4,949421	4,61468	4,817447	4,736694	4,8176487	4,722940333	-0,09470833	0,936461551	Barx2
17349956	4,991634	4,631123	4,890827	4,785854	4,679766	4,763852	4,8378613	4,743157333	-0,094704	0,936464364	---
17479668	7,591341	7,864309	7,223403	7,47886	7,393427	7,522683	7,5596843	7,46499	-0,09469433	0,936470638	Pde8a
17278339	4,716499	4,727843	4,494123	4,427265	4,701943	4,525182	4,646155	4,551463333	-0,09469167	0,936472369	6230426M11Rik
17515839	4,118468	4,201869	4,195629	4,314494	3,982712	3,934725	4,1719887	4,077310333	-0,09467833	0,936481024	7630403G23Rik // 7630403G23Rik
17226142	6,607736	6,492184	6,384089	6,34836	6,58548	6,266198	6,4946697	6,400012667	-0,094657	0,936494872	Dsel
17527982	7,194732	6,883293	6,86487	6,805789	7,112928	6,740207	6,980965	6,886308	-0,094657	0,936494872	Mir5133
17514653	3,794449	3,639315	3,860252	3,660837	3,589509	3,759762	3,764672	3,670036	-0,094636	0,936508504	---
17396345	4,515098	4,624698	4,674119	4,453066	4,487172	4,589794	4,6046383	4,510010667	-0,09462767	0,936513913	---
17342595	7,540588	7,446662	7,519612	7,459178	7,352168	7,411666	7,5022873	7,407670667	-0,09461667	0,936521054	Nme4
17477191	5,552378	5,182704	5,813377	5,14117	5,571969	5,551471	5,516153	5,421536667	-0,09461633	0,93652127	Klk13
17312312	5,095644	5,04976	5,385947	5,024366	5,14914	5,074021	5,177117	5,082509	-0,094608	0,93652668	Mapk15
17339289	5,037324	5,172261	5,162093	4,896334	5,185222	5,006327	5,1238927	5,029294333	-0,09459833	0,936532955	L3mbtl4
17473248	5,94344	5,595779	5,913374	5,718594	5,545763	5,904457	5,817531	5,722938	-0,094593	0,936536417	Ttyh1
17505788	3,891679	3,566506	4,231544	3,637201	4,161703	3,607053	3,8965763	3,801985667	-0,09459067	0,936537932	---
17438742	3,599809	3,501081	3,621291	3,592963	3,494154	3,351327	3,5740603	3,479481333	-0,094579	0,936545506	Prol1

17234948	7,160659	7,159151	7,46487	6,940297	7,38743	7,173258	7,26156	7,166995	-0,094565	0,936554594	Fgf22
17275678	5,029601	4,84814	4,948851	4,69598	5,09093	4,756038	4,9421973	4,847649333	-0,094548	0,93656563	Sstr1
17490481	6,148353	6,366714	6,213194	6,027231	6,242662	6,174778	6,2427537	6,148223667	-0,09453	0,936577315	Cpt1c
17494303	4,768106	4,570214	4,574343	4,574343	4,521892	4,532859	4,6375543	4,543031333	-0,094523	0,936581859	Ubqlnl
17314888	10,10024	10,0017	10,16598	10,02624	9,925934	10,03219	10,089307	9,994788	-0,09451867	0,936584673	Gpd1
17220776	6,997557	7,162172	7,059124	7,010769	6,946194	6,978341	7,072951	6,978434667	-0,09451633	0,936586187	Nsl1
17300058	5,920747	6,37912	6,524488	6,384813	6,2142	5,941832	6,274785	6,180281667	-0,09450333	0,936594627	---
17250665	6,918715	6,97767	7,106446	6,963886	6,823728	6,931722	7,0009437	6,906445333	-0,09449833	0,936597873	Ttc19
17348684	4,200871	3,965637	4,245988	4,047505	4,136278	3,945221	4,1374987	4,043001333	-0,09449733	0,936598522	D830029L11
17508609	6,218953	6,272156	6,285337	6,194416	6,157996	6,140593	6,2588153	6,164335	-0,09448033	0,936609559	Nrg1
17205671	4,480155	4,454907	3,943211	3,920899	4,668915	4,00514	4,2927577	4,198318	-0,09443967	0,93663596	---
17401610	9,041848	9,342529	9,366017	9,097332	9,344827	9,024934	9,2501313	9,155697667	-0,09443367	0,936639855	Gstm5
17516178	3,636356	3,320822	3,220661	3,403374	3,280303	3,210903	3,392613	3,298193333	-0,09441967	0,936648945	---
17427709	3,805638	3,918477	3,774208	3,814306	3,568044	3,832776	3,8327743	3,738375333	-0,094399	0,936662362	4921539E11Rik
17475942	6,891982	6,911733	6,679663	6,649029	6,8062	6,74503	6,8277927	6,733419667	-0,094373	0,936679243	Rinl
17446028	3,337681	2,992948	3,41123	3,122776	3,180777	3,1552	3,2472863	3,152917667	-0,09436867	0,936682056	---
17499028	8,133375	8,157802	8,019474	7,944422	8,037329	8,045813	8,1035503	8,009188	-0,09436233	0,936686168	Ing1
17543920	4,337099	4,201025	4,457363	4,0482	4,330101	4,334123	4,331829	4,237474667	-0,09435433	0,936691362	5330434G04Rik
17306859	10,46885	10,2917	10,80058	10,62299	10,2766	10,3785	10,520377	10,42603	-0,09434667	0,93669634	---
17360462	11,11388	11,21305	11,15798	11,11987	10,92591	11,15618	11,161637	11,06732	-0,09431667	0,936715818	Acsl5
17429177	8,906956	9,012963	8,933233	8,886021	8,842074	8,842117	8,9510507	8,856737333	-0,09431333	0,936717983	Cdc20
17339902	3,865152	3,999668	4,369714	4,185173	3,708216	4,058237	4,078178	3,983875333	-0,09430267	0,936724908	Fam82a1
17405047	4,254422	4,417622	4,273098	4,220043	4,231462	4,210737	4,3150473	4,220747333	-0,0943	0,93672664	2610316D01Rik
17481207	3,490219	3,398884	3,370052	3,339254	3,303579	3,333494	3,4197183	3,325442333	-0,094276	0,936742223	Olfr608
17489977	4,731606	4,575846	4,180842	4,144323	4,605409	4,45576	4,496098	4,401830667	-0,09426733	0,93674785	Zfp939 // Zfp939
17410615	3,817682	3,936963	3,883105	3,586906	3,985289	3,782808	3,87925	3,785001	-0,094249	0,936759754	Dnajb14 // Dnajb14
17516997	8,165282	8,418488	7,334989	8,039221	7,76563	7,831254	7,9729197	7,878701667	-0,094218	0,936779883	Gm5617
17344978	4,652506	4,718172	4,74811	4,504581	4,523012	4,808561	4,7062627	4,612051333	-0,09421133	0,936784212	9130008F23Rik
17430799	7,199431	7,245224	6,989383	7,05169	7,007075	7,092677	7,1446793	7,050480667	-0,09419867	0,936792437	Trnau1ap
17373837	5,048352	4,932008	4,552257	4,74478	4,637446	4,86781	4,8442057	4,750012	-0,09419367	0,936795683	D430041D05Rik
17534330	7,757623	7,719851	7,734454	7,613854	7,646515	7,668994	7,7373093	7,643121	-0,09418833	0,936799147	Zbtb33
17291081	3,747808	3,270892	3,499125	3,432021	3,459018	3,344228	3,5059417	3,411755667	-0,094186	0,936800662	Vmn1r205
17291461	3,950258	3,674666	3,89888	3,589414	3,754531	3,897317	3,841268	3,747087333	-0,09418067	0,936804125	Prl7b1
17296615	4,736903	4,119394	3,971486	4,150402	4,165556	4,229297	4,2759277	4,181751667	-0,094176	0,936807155	---
17306785	9,494887	9,873351	9,70696	9,689942	9,588354	9,51442	9,6917327	9,597572	-0,09416067	0,936817112	Nedd8
17407643	6,137493	5,817131	5,966149	5,924237	5,878366	5,835758	5,973591	5,879453667	-0,09413733	0,936832263	Rfx5 // Rfx5
17484771	6,019162	6,30799	6,211345	5,981071	6,01035	6,264685	6,179499	6,085368667	-0,09413033	0,936836809	Lrrc56
17462985	3,179941	3,381325	3,376092	2,915873	3,555437	3,183763	3,3124527	3,218357667	-0,094095	0,936859754	---
17322352	6,771859	7,200382	6,930331	6,872401	7,030849	6,717068	6,967524	6,873439333	-0,09408467	0,936866464	Copz1 // Copz1
17234917	6,0356	5,836352	6,096109	6,004857	5,760272	5,920689	5,9893537	5,895272667	-0,094081	0,936868845	Gzmm
17232426	8,546142	8,737297	8,340226	8,316785	8,51371	8,510933	8,5412217	8,447142667	-0,094079	0,936870144	Echdc1
17527032	8,055834	8,024872	7,961104	7,833124	7,918933	8,007526	8,0139367	7,919861	-0,09407567	0,936872308	Ddx10
17240323	7,072089	6,895246	6,947949	6,768742	6,856729	7,007608	6,9717613	6,877693	-0,09406833	0,936877071	AI317395
17549626	6,105915	5,901434	6,1272	5,840653	6,132296	5,879414	6,0448497	5,950787667	-0,094062	0,936881183	---
17238424	10,49599	10,50852	10,42333	10,36076	10,38818	10,39676	10,475947	10,3819	-0,09404667	0,936891141	Cnpy2
17507668	6,387243	6,363422	6,592223	6,081131	6,569035	6,410629	6,4476293	6,353598333	-0,094031	0,936901315	2410022L05Rik
17439517	4,956452	4,628361	4,577967	4,686102	4,578868	4,615746	4,7209267	4,626905333	-0,09402133	0,936907593	Fgf5
17453513	8,430227	8,396618	8,458832	8,226365	8,38443	8,392847	8,428559	8,334547333	-0,09401167	0,93691387	Wbscr22
17419418	6,783739	6,431734	6,607631	6,49055	6,807739	6,242819	6,6077013	6,513702667	-0,09399867	0,936922313	---
17211280	8,23629	8,204277	8,179316	8,048029	8,158298	8,131589	8,2066277	8,112638667	-0,093989	0,936928591	Tmem70
17412121	3,240969	2,886099	3,053609	3,121495	2,970037	2,807286	3,0602257	2,966272667	-0,093953	0,93695197	---
17387407	8,219488	8,189109	8,283509	8,221253	8,149358	8,039645	8,230702	8,136752	-0,09395	0,936953919	Tfpi
17264792	6,93814	7,377516	7,228421	7,206795	6,94276	7,112687	7,181359	7,087414	-0,093945	0,936957166	Atp1b2
17444930	6,604768	6,620656	6,636316	6,481176	6,506804	6,591953	6,62058	6,526644333	-0,09393567	0,936963227	---
17528418	7,762163	7,701207	7,7194	7,522052	7,689404	7,689508	7,72759	7,633654667	-0,09393533	0,936963444	Lactb
17319753	6,261994	5,827255	6,013821	6,06237	5,925405	5,833496	6,0343567	5,940423667	-0,093933	0,936964959	---
17536003	3,730424	3,328604	3,027031	3,223606	3,101224	3,479435	3,3620197	3,268088333	-0,09393133	0,936966042	---
17469360	5,066304	5,007442	4,842162	4,70975	4,939714	4,984682	4,9719693	4,878048667	-0,09392067	0,936972969	Lmod3
17520604	4,689204	4,606706	4,628071	4,485506	4,623408	4,533319	4,641327	4,547411	-0,093916	0,936976	Trim42 // Trim42
17212792	8,395443	8,48851	8,204151	8,383917	8,195567	8,226918	8,3627013	8,268800667	-0,09390067	0,936985959	Hibch
17344216	5,8961	5,727192	5,634355	5,479971	6,022923	5,473085	5,752549	5,658659667	-0,09388933	0,936993319	Ly6g5b
17206903	6,444568	5,722141	5,175424	5,461566	6,218759	5,380147	5,780711	5,686824	-0,093887	0,936994835	---
17417926	6,972923	7,132064	6,928083	6,939355	6,911204	6,900896	7,0110233	6,917151667	-0,09387167	0,937004793	Foxj3
17546367	3,560801	4,460627	3,979901	3,725017	4,409119	3,585593	4,000443	3,906576333	-0,09386667	0,937008041	Rbmy1a1
17473622	3,466228	3,396335	3,531504	3,367275	3,363955	3,381325	3,464689	3,370851667	-0,09383733	0,937027093	---
17458790	5,12436	5,212413	5,247797	4,836542	5,251693	5,214849	5,1948567	5,101028	-0,09382867	0,937032722	Fam188b
17266847	4,679538	4,57158	4,649773	4,24508	4,624741	4,749675	4,6336303	4,539832	-0,09379833	0,937052423	1700003F17Rik
17507537	7,41818	7,50972	7,46422	7,373991	7,362316	7,374434	7,46404	7,370247	-0,093793	0,937055887	Pcid2
17291102	2,837904	3,03221	2,973282	2,661289	2,83559	3,065148	2,9477987	2,854009	-0,09378967	0,937058052	---
17443114	8,173685	8,375055	8,307423	8,21784	8,198519	8,158444	8,2853877	8,191601	-0,09378667	0,937060001	Rfc2
17296312	8,725883	8,677428	8,662603	8,613805	8,64273	8,528027	8,688638	8,594854	-0,093784	0,937061733	Snx18
17408829	8,035681	8,235107	8,110031	7,983793	7,970562	8,145151	8,1269397	8,033168667	-0,093771	0,937070177	Ddx20
17515994	3,799004	3,770876	3,833239	3,746309	3,774907	3,600644	3,8010397	3,707286667	-0,093753	0,937081868	Gm9513
17395091	6,424976	6,323359	6,67055	6,475767	6,259893	6,402039	6,4729617	6,379233	-0,09372867	0,937097674	Pmepa1
17254633	8,599187	8,728993	8,53525	8,59452	8,422686	8,565053	8,6211433	8,527419667	-0,09372367	0,937100922	Rnft1
17323061	8,668962	8,961191	8,699079	8,668894	8,615452	8,763828	8,7764107	8,682724667	-0,093686	0,937125388	Rrn3
17306418	8,494833	9,12507	8,601795	8,484637	8,899016	8,557001	8,740566	8,646884667	-0,09368133	0,93712842	---
17393546	3,998501	3,858801	4,033784	3,995966	3,856955	3,7574	3,9636953	3,870107	-0,09358833	0,937188831	---
17299542	8,558713	8,529254	8,640308	8,531453	8,433591	8,482493	8,5760917	8,482512333	-0,09357933	0,937194678	Tmem55b // Tmem55b
17493067	3,045506	3,517976	3,041564	3,388825	2,941068	2,994525	3,201682	3,108139333	-0,09354267	0,937218497	Vmn2r69
17247627	5,295789	5,308963	5,477086	5,315232	5,196295	5,289716	5,3606127	5,267081	-0,09353167	0,937225643	Tmem17
17204047	5,085052	5,010608	5,6249	5,476201	5,129811	4,834018	5,2401867	5,146676667	-0,09351	0,937239719	---
17436328	8,567062	8,62336	8,51282	8,396851	8,520146	8,50572	8,5677473	8,474239	-0,09350833	0,937240802	Yes1
17500327	9,582364	9,723505	9,901402	9,734301	9,657661	9,534827	9,735757	9,642263	-0,093494	0,937250113	Eif4ebp1
17329906	5,73236	5,590999	5,810537	5,482628	5,635677	5,735204	5,7112987	5,617836333	-0,09346233	0,937270686	Iqcg
17301852	9,514166	9,428787	9,544183	9,316693	9,429135	9,46093	9,495712	9,402252667	-0,09345933	0,937272635	Gm9199
17324402	8,392765	8,819967	8,57857	8,341591	8,542627	8,626754	8,5971007	8,503657333	-0,09344333	0,93728303	---
17549066	6,433255	6,332554	6,354851	6,321977	6,35052	6,167863	6,3735533	6,28012	-0,09343333	0,937289526	---
17200725	3,440689	2,967694	2,801988	2,898875	3,184227	2,847023	3,0701237	2,976708333	-0,09341533	0,937301221	---
17370924	4,419708	4,396486	4,532262	4,32399	4,372727	4,371495	4,4494853	4,356070667	-0,09341467	0,937301654	Galnt5
17230916	9,310173	9,39108	9,381742	9,28518	9,300609	9,216969	9,3609983	9,267586	-0,09341233	0,93730317	Pdcd5 // Pdcd5
17470976	5,001294	5,02463	5,332211	4,997133	5,032149	5,048633	5,1193783	5,025971667	-0,09340667	0,937306851	Lag3
17528615	6,913001	7,650317	7,42266	7,101995	7,04853	7,555248	7,3286593	7,235257667	-0,09340167	0,9373101	---
17465332	4,989939	4,398477	4,491759	4,602549	4,494817	4,502646	4,626725	4,533337333	-0,09338767	0,937319196	Lrrc4
17273140	6,434102	6,580164	6,68824	6,459207	6,278966	6,684176	6,567502	6,474116333	-0,09338567	0,937320495	---
17411361	4,057969	3,837339	4,002693	3,826006	3,827076	3,964764	3,9660003	3,872615333	-0,093385	0,937320928	---
17272845	5,449357	5,493789	5,770828	5,417449	5,559632	5,456845	5,5713247	5,477975333	-0,09334933	0,937344101	Cbx8
17419521	5,608055	5,607672	5,718601	5,639199	5,407017	5,608113	5,644776	5,551443	-0,093333	0,937354713	---
17238996	4,650379	4,76666	4,673566	4,509566	4,671653	4,629442	4,6968683	4,603553667	-0,09331467	0,937366625	Rgs17
17257591	11,20908	11,17824	11,44581	11,14037	11,35213	11,06081	11,27771	11,18443667	-0,09327333	0,937393481	Snord104
17544768	4,925095	4,879149	5,179778	4,780668	4,898996	5,024584	4,994674	4,901416	-0,093258	0,937403444	Glra4
17206707	6,1582	5,740068	5,775091	5,739992	5,98855	5,66505	5,8911197	5,797864	-0,09325567	0,93740496	---
17304518	7,317016	7,520433	7,390388	7,438626	7,234356	7,275247	7,409279	7,316076333	-0,09320267	0,937439398	2010107H07Rik
17278820	4,377604	4,920871	4,966408	4,623354	4,759047	4,602937	4,754961	4,661779333	-0,09318167	0,937453043	Mir494
17341276	8,182874	8,465065	7,873858	7,990973	8,249639	8,001646	8,1739323	8,080752667	-0,09317967	0,937454343	Fpr1
17433898	5,512803	6,188253	5,9249	5,718075	5,603576	6,024833	5,8753187	5,782161333	-0,09315733	0,937468855	Mxra8 // Mxra8
17392525	6,090942	5,867215	5,885519	5,906297	5,947272	5,710649	5,947892	5,854739333	-0,09315267	0,937471887	Nkx2-4
17457787	7,599635	7,50764	7,646152	7,546525	7,604454	7,323032	7,5844757	7,491337	-0,09313867	0,937480985	---
17306906	6,961293	6,986853	6,867697	6,757886	6,88465	6,893986	6,9386143	6,845507333	-0,093107	0,937501562	Ripk3
17312780	6,840806	6,717196	6,73811	6,696273	6,720675	6,599868	6,7653707	6,672272	-0,09309867	0,937506978	Gga1
17341342	3,25635	3,156797	3,243669	3,09821	3,122926	3,156398	3,2189387	3,125844667	-0,093094	0,93751001	Vmn1r228
17490274	6,800523	6,682832	6,72977	6,71453	6,239567	6,979753	6,7377083	6,644616667	-0,09309167	0,937511526	Spib
17308046	4,018343	4,328579	4,108167	3,816309	4,097094	4,262423	4,1516963	4,058608667	-0,09308767	0,937514126	Nefm
17315436	3,409792	3,499793	3,929854	3,486119	3,490973	3,583095	3,6131463	3,520062333	-0,093084	0,937516509	---
17453492	9,649342	9,912996	9,954631	9,753121	9,724761	9,759849	9,8389897	9,745910333	-0,09307933	0,937519541	Cldn13
17495802	4,658671	4,370963	4,769354	4,726695	4,476901	4,316156	4,5996627	4,506584	-0,09307867	0,937519974	Pdzd9
17500749	5,416862	5,177997	5,4433	5,276443	5,284558	5,197924	5,346053	5,252975	-0,093078	0,937520408	Mtus1
17326735	4,083672	4,483783	4,352246	4,181408	4,238111	4,220963	4,306567	4,213494	-0,093073	0,937523657	Chodl
17356670	4,065884	4,277846	4,050223	4,183851	4,019551	3,911339	4,1313177	4,038247	-0,09307067	0,937525173	---
17471502	6,190868	6,14904	6,118665	6,143445	6,042243	5,993691	6,1528577	6,059793	-0,09306467	0,937529072	Cd69
17298750	5,954886	5,849996	5,824181	5,90134	5,649445	5,799143	5,8763543	5,783309333	-0,093045	0,937541853	Gm5460
17489946	3,740062	4,02334	3,997384	3,787684	4,083072	3,610898	3,920262	3,827218	-0,093044	0,937542502	---
17204021	4,53871	4,519333	4,310108	4,639824	4,18887	4,260351	4,4560503	4,363015	-0,09303533	0,937548134	---
17300104	4,002644	3,934542	3,547466	3,639079	3,671675	3,894827	3,8282173	3,735193667	-0,09302367	0,937555716	---
17484550	6,260591	5,67241	5,993332	5,981482	5,761055	5,904744	5,9754443	5,882427	-0,09301733	0,937559832	Olfr525
17505913	8,868057	8,806157	8,741327	8,599536	8,737137	8,799847	8,8051803	8,712173333	-0,093007	0,937566547	Cenpn
17441808	9,363406	9,148021	9,449235	9,43072	9,277783	8,973145	9,3202207	9,227216	-0,09300467	0,937568064	---
17384569	3,367236	3,047925	3,332332	3,189692	3,260481	3,018315	3,2491643	3,156162667	-0,09300167	0,937570013	Olfr358
17356305	7,488067	7,446024	7,6106	7,507594	7,367898	7,390199	7,514897	7,421897	-0,093	0,937571096	Zdhhc24
17449673	7,464474	7,875628	7,489453	7,552693	7,520917	7,476954	7,6098517	7,516854667	-0,092997	0,937573046	Naaa
17286553	7,276163	7,370976	7,352868	7,15712	7,212497	7,351438	7,3333357	7,240351667	-0,092984	0,937581495	Cdyl
17436099	6,666306	6,946786	7,080386	6,790361	6,916632	6,707684	6,897826	6,804892333	-0,09293367	0,937614206	Krtcap3
17350552	7,05054	7,024283	6,863943	6,847202	6,790361	7,02241	6,9795887	6,886657667	-0,092931	0,937615939	Prr16
17543137	3,209279	3,366388	3,422579	3,279774	3,195837	3,243885	3,3327487	3,239832	-0,09291667	0,937625254	---

17292122	9,036425	9,049603	8,980038	8,885405	8,981133	8,9209	9,022022	8,929146	-0,092876	0,937651684	Ranbp9
17273743	6,269803	6,539597	6,516224	6,25496	6,46433	6,327767	6,4418747	6,349019	-0,09285567	0,9376649	2410017P09Rik
17388353	6,44437	6,422078	6,44907	6,400716	6,15028	6,485968	6,438506	6,345654667	-0,09285133	0,937667716	Gyltl1b
17441535	4,104154	3,826908	3,768084	3,67717	3,808826	3,934601	3,8997153	3,806865667	-0,09284967	0,937668799	---
17347263	3,290761	3,040564	3,240921	2,98501	3,294896	3,013811	3,1907487	3,097905667	-0,092843	0,937673132	---
17363139	3,115721	3,052502	3,017047	3,037317	2,74044	3,128997	3,0617567	2,968918	-0,09283867	0,937675949	Olfr1457
17215788	7,314854	7,452132	7,303584	7,310182	7,306997	7,174934	7,3568567	7,264037667	-0,092819	0,937688731	Asb1
17548012	10,47218	10,58831	10,59661	10,26962	10,49961	10,60943	10,552367	10,45955333	-0,09281333	0,937692414	Hmgn2 // Hmgn2 // Hmgn2 // Hmgn2 /.
17518326	4,667842	4,628117	4,667393	4,856937	4,545808	4,282181	4,6544507	4,561642	-0,09280867	0,937695447	---
17346349	7,514309	7,416406	7,420386	7,326559	7,360317	7,385823	7,450367	7,357566333	-0,09280067	0,937700647	Rfx2
17539062	3,580364	4,472751	3,654798	3,880969	3,629476	3,919071	3,9026377	3,809838667	-0,092799	0,93770173	---
17413470	4,41245	4,465644	4,509577	4,329388	4,34185	4,438045	4,462557	4,369761	-0,092796	0,93770368	Olfr155
17421571	7,372022	7,549223	7,530846	7,510365	7,366096	7,29726	7,4840303	7,391240333	-0,09279	0,93770758	Mad2l2
17324354	3,580027	3,913127	3,543722	3,746902	3,4774	3,534208	3,6789587	3,58617	-0,09278867	0,937708447	---
17201581	4,596019	4,439231	4,352104	4,45109	4,483604	4,174314	4,4624513	4,369669333	-0,092782	0,93771278	---
17371215	5,9722	6,04326	6,002784	5,865191	6,055129	5,819604	6,0060813	5,913308	-0,09277333	0,937718413	---
17201743	5,39807	4,660586	4,255931	4,559404	4,717572	4,759352	4,771529	4,678776	-0,092753	0,937731629	---
17201919	5,39807	4,660586	4,255931	4,559404	4,717572	4,759352	4,771529	4,678776	-0,092753	0,937731629	---
17208729	6,071935	5,812623	5,442973	5,823368	5,105453	6,12048	5,7758437	5,683100333	-0,09274333	0,937737912	---
17521483	3,794012	3,82029	4,085322	3,804695	3,922806	3,693915	3,8998747	3,807138667	-0,092736	0,937742679	---
17500808	10,66251	10,49936	10,43943	10,27997	10,42301	10,62013	10,533767	10,44103667	-0,09273	0,937746579	---
17471598	4,170986	4,034001	4,19169	4,139558	3,816577	4,162418	4,1322257	4,039517667	-0,092708	0,937760879	Klrc1
17303536	4,632301	4,223835	4,373046	4,237668	4,553617	4,159783	4,4097273	4,317022667	-0,09270467	0,937763046	---
17463591	3,097964	2,93562	2,895442	2,930464	2,837579	2,882954	2,976342	2,883665667	-0,09267633	0,937781463	---
17463593	3,097964	2,93562	2,895442	2,930464	2,837579	2,882954	2,976342	2,883665667	-0,09267633	0,937781463	---
17463595	3,097964	2,93562	2,895442	2,930464	2,837579	2,882954	2,976342	2,883665667	-0,09267633	0,937781463	---
17498962	6,996286	7,235184	7,074312	7,145932	7,104237	6,777593	7,1019273	7,009254	-0,09267333	0,937783413	Col4a2
17381355	3,317913	2,98494	3,206472	3,152729	3,091892	2,98673	3,169775	3,077117	-0,092658	0,93779338	---
17223574	8,146189	8,240753	7,928129	7,874676	8,04956	8,112962	8,1050237	8,012399333	-0,09262433	0,937815265	Sumo1
17315152	7,280072	7,128108	7,2882	7,279204	7,377833	6,761472	7,2321267	7,139503	-0,09262367	0,937815698	Acvr1b
17306532	4,563579	4,901391	4,670498	4,852485	4,47176	4,533401	4,7118227	4,619215333	-0,09260733	0,937826315	Myh6
17237084	8,041412	8,009202	7,797573	7,727145	7,858238	7,985029	7,9493957	7,856804	-0,09259167	0,9378365	Ccdc59
17287243	9,621572	10,04624	10,03752	9,981295	9,801165	9,645098	9,9017773	9,809186	-0,09259133	0,937836716	---
17346735	9,745063	9,684782	9,70997	9,548921	9,647556	9,665593	9,7132717	9,62069	-0,09258167	0,937843	Vapa
17548277	4,821895	4,918015	4,938526	4,889173	4,72304	4,788502	4,892812	4,800238333	-0,09257367	0,937848201	Gm19382
17468926	7,379793	7,4872	7,602452	7,392532	7,409355	7,389867	7,489815	7,397251333	-0,09256367	0,937854701	LOC100861972
17399663	9,081829	9,068725	9,017598	8,915047	8,98131	8,994123	9,0560507	8,963493333	-0,09255733	0,937858818	Jtb
17359055	9,9786	10,12711	10,02387	9,949198	9,947294	9,955449	10,043193	9,950647	-0,09254633	0,937865969	Exoc6
17389578	8,263229	8,288906	8,264718	8,217154	8,18188	8,140212	8,2722843	8,179748667	-0,09253567	0,937872904	Atpbd4
17200145	4,157287	3,763063	4,639083	4,299609	4,066224	3,916029	4,1864777	4,093954	-0,09252367	0,937880705	---
17455722	4,550612	4,641033	4,526785	4,676108	4,414382	4,350434	4,57281	4,480308	-0,092502	0,93789479	Gm7963 // LOC665729 // Gm6523
17510478	4,066024	4,231926	4,245196	4,027708	4,350742	3,887225	4,1810487	4,088558333	-0,09249033	0,937902374	---
17457866	3,286556	3,449468	3,470657	3,314262	3,251101	3,363936	3,402227	3,309766333	-0,09246067	0,937921661	Sva
17343823	3,861357	3,819403	4,209164	3,86073	3,81477	3,937192	3,963308	3,870897333	-0,09241067	0,937954168	---
17378329	10,22591	10,23168	10,01628	10,03767	10,05712	10,10185	10,157957	10,06554667	-0,09241	0,937954601	Dynlrb1
17486589	9,10562	9,315435	9,111195	9,097424	9,007352	9,150266	9,1774167	9,085014	-0,09240267	0,937959369	Slc27a5
17448373	4,464594	4,048428	4,180289	4,144724	4,141458	4,129927	4,2311037	4,138703	-0,09240067	0,937960669	---
17287682	8,074659	8,246559	8,10299	7,851148	8,061825	8,234076	8,1414027	8,049016333	-0,09238633	0,937969988	Grk6
17361638	5,770042	5,91913	5,794241	5,818225	5,651903	5,736159	5,8278043	5,735429	-0,09237533	0,937977139	Snx32
17305685	7,770728	7,9221	7,822921	7,637506	7,778373	7,822792	7,838583	7,746223667	-0,09235933	0,937987542	Ddhd1
17244529	3,518778	3,625754	3,693475	3,453133	3,601328	3,50652	3,612669	3,520327	-0,092342	0,937998812	4930473O22Rik
17200593	5,140079	4,559987	4,57072	4,813851	4,782945	4,397038	4,7569287	4,664611333	-0,09231733	0,938014849	---
17434381	9,120989	9,177778	9,091822	8,870547	9,062013	9,181089	9,1301963	9,037883	-0,09231333	0,93801745	Krit1
17544098	4,86129	4,718551	4,77228	5,148944	4,155825	4,770507	4,7840403	4,691758667	-0,09228167	0,938038039	Gm732
17232438	5,900208	6,461884	6,396613	6,237865	6,191182	6,052847	6,2529017	6,160631333	-0,09227033	0,938045408	---
17427155	6,454317	6,470043	6,410033	6,302498	6,419701	6,335386	6,4447977	6,352528333	-0,09226933	0,938046059	Cdkn2b
17356383	6,729546	6,311557	6,70766	6,599716	6,386966	6,485327	6,582921	6,490669667	-0,09225133	0,938057762	Gal3st3
17359466	6,481055	6,154785	6,405074	6,2071	6,251998	6,305089	6,3469713	6,254729	-0,09224233	0,938063614	Pgam1
17536343	4,373418	4,345719	4,394601	4,30865	4,175189	4,353232	4,371246	4,279023667	-0,09222233	0,938076619	Zxda // Zxda
17432488	4,630601	4,764258	4,929911	4,792144	4,534538	4,721423	4,7749233	4,682701667	-0,09222167	0,938077052	Pramef6
17458465	6,612469	6,426567	6,414018	6,229557	6,533957	6,412878	6,4843513	6,392130667	-0,09222067	0,938077702	Ccdc126
17387810	4,994095	4,597743	4,621305	4,730262	4,681438	4,524894	4,7377143	4,645531333	-0,092183	0,938102195	Olfr1166
17257680	3,403111	3,435985	3,443382	3,506313	3,178408	3,321214	3,4274927	3,335311667	-0,092181	0,938103495	---
17433656	5,583994	5,543536	5,469263	5,393458	5,636875	5,28997	5,5322643	5,440101	-0,09216333	0,938114983	5930403L14Rik
17256979	3,792265	3,744307	3,748196	3,700061	3,722697	3,585577	3,7615893	3,669445	-0,09214433	0,938127338	Gm1564
17402708	5,914793	6,054626	5,95237	5,81776	5,907557	5,92005	5,9739297	5,881789	-0,09214067	0,938129722	Aimp1 // Aimp1
17414301	9,577251	9,465005	9,627843	9,49804	9,48618	9,409462	9,5566997	9,464560667	-0,092139	0,938130806	Rad23b
17438569	3,919775	4,250492	3,94421	4,061958	3,962379	3,8138	4,038159	3,946045667	-0,09211333	0,938147496	---
17459731	3,731231	3,699954	3,318511	3,418746	3,602497	3,452113	3,583232	3,491118667	-0,09211333	0,938147496	---
17309457	5,420444	5,314192	5,383801	5,34386	5,233442	5,26481	5,3728123	5,280704	-0,09210833	0,938150747	Dzip1
17284694	4,405085	4,546321	4,203184	4,446395	4,181349	4,250532	4,3848633	4,292758667	-0,09210467	0,938153132	---
17212874	8,445176	8,663862	8,231847	8,293411	8,454052	8,317109	8,4469617	8,354857333	-0,09210433	0,938153348	Coq10b
17547400	2,683615	3,135739	3,160096	2,66939	3,094265	2,939525	2,99315	2,90106	-0,09209	0,938162669	---
17278502	4,036335	4,055215	4,034574	3,999974	3,986323	3,863626	4,0420413	3,949974333	-0,092067	0,938177626	---
17372052	8,431861	8,125678	8,359863	8,253813	8,188768	8,198651	8,3058007	8,213744	-0,09205667	0,938184345	---
17482366	6,628828	7,170191	6,940389	7,0741	6,913423	6,475808	6,913136	6,821110333	-0,09202567	0,938204505	Tmem159
17419044	9,94788	9,879812	9,902857	9,76923	9,858738	9,826551	9,910183	9,818173	-0,09201	0,938214693	Ptp4a2
17481612	5,874184	5,211764	5,065082	5,307336	5,222821	5,344853	5,3836767	5,29167	-0,09200667	0,938216861	Olfr491
17430945	7,465017	7,535612	7,630536	7,41579	7,482356	7,457006	7,5437217	7,451717333	-0,09200433	0,938218378	Fam76a
17264338	6,673512	6,826385	6,74409	6,711783	6,556277	6,699928	6,7479957	6,655996	-0,09199967	0,938221413	Gas7 // Gas7
17425123	5,590807	5,399297	5,421968	5,261078	5,2465	5,628526	5,4706907	5,378701333	-0,09198933	0,938228133	Anks6
17311443	3,771068	4,261586	4,423318	4,332692	3,737847	4,109487	4,1519907	4,060008667	-0,091982	0,938232902	---
17310201	9,308788	9,492765	9,214912	9,33121	9,200058	9,209292	9,3388217	9,246853333	-0,09196833	0,93824179	Lmbrd2
17376274	5,706639	6,021965	5,913228	5,737405	5,827564	5,801061	5,8806107	5,788676667	-0,091934	0,938264119	Nop56
17415728	11,99123	12,08876	12,12607	12,05244	11,91259	11,96523	12,068687	11,97675333	-0,09193333	0,938264553	Angptl3
17524967	4,644491	4,464406	4,438747	4,350831	4,347219	4,573815	4,5158813	4,423955	-0,09192633	0,938269105	Gm6607
17517198	3,67821	3,881117	4,015092	3,878631	3,840818	3,579228	3,8581397	3,766225667	-0,091914	0,938277126	---
17316390	8,948335	9,378822	9,21013	8,948656	9,264532	9,048361	9,1790957	9,087183	-0,09191267	0,938277993	Gm7429 // Gm6109 // Rpl30
17234027	3,944483	3,884386	3,860571	3,83915	3,759726	3,814828	3,89648	3,804568	-0,091912	0,938278427	---
17272174	6,927229	6,968813	6,740101	6,802171	6,831335	6,726962	6,8787143	6,786822667	-0,09189167	0,938291651	Trim65
17423030	5,61672	5,986752	5,926278	5,8003	6,057373	5,396422	5,84325	5,751365	-0,091885	0,938295987	Penk
17344862	3,663083	3,237222	3,67697	3,643421	3,321174	3,337075	3,5257583	3,43389	-0,09186833	0,938306827	Olfr101
17368550	8,89855	8,937551	8,956132	8,883278	8,841865	8,791606	8,9307443	8,838916333	-0,091828	0,938333059	Rxra
17519644	5,823892	5,792147	5,704032	5,61183	5,921077	5,511699	5,773357	5,681535333	-0,09182167	0,938337178	C920006O11Rik
17535110	4,055083	3,87474	3,983336	3,861323	3,867674	3,908699	3,971053	3,879232	-0,091821	0,938337612	---
17286732	10,45389	10,7258	10,7257	10,59155	10,52474	10,51364	10,63513	10,54331	-0,09182	0,938338262	Tmem14c
17294516	3,986812	4,20086	4,698442	3,97371	4,467126	4,169819	4,2953713	4,203551667	-0,09181967	0,938338479	---
17482590	7,012258	7,051538	7,04873	6,894022	7,073968	6,869123	7,0375087	6,945704333	-0,09180433	0,938348452	Polr3e
17302289	5,367703	5,369523	5,469716	5,31681	5,303825	5,310899	5,402314	5,310511333	-0,09180267	0,938349536	Pcdh17
17224952	3,236759	3,317335	3,274824	3,135532	3,270892	3,147106	3,276306	3,18451	-0,091796	0,938353872	---
17404150	3,453524	3,419344	3,317422	3,537818	3,220744	3,156379	3,3967633	3,304980333	-0,091783	0,938362328	---
17417066	3,414905	3,47006	3,381567	3,318867	3,275406	3,396941	3,4221773	3,330404667	-0,09177267	0,938369049	Skint2
17256673	9,322262	9,444344	9,222445	9,124594	9,260697	9,328456	9,3296837	9,237915667	-0,091768	0,938372084	Psme3
17501292	8,092297	8,013559	8,146704	7,972863	7,982616	8,021791	8,0841867	7,992422	-0,09176467	0,938374252	Hmgb2
17219178	3,516217	3,294423	3,469303	3,280404	3,427984	3,296283	3,4266477	3,334890333	-0,09175733	0,938379022	1700015E13Rik
17445895	6,826674	7,150099	6,884449	6,593345	7,171426	6,821179	6,9537407	6,861983333	-0,09175733	0,938379022	Psmc2 // Psmc2
17201275	4,315411	4,399331	4,677008	4,418483	4,206718	4,491287	4,4639167	4,372162667	-0,091754	0,93838119	---
17325721	8,324541	8,239305	8,229843	8,160822	8,211184	8,146467	8,264563	8,172824333	-0,09173867	0,938391164	---
17474270	3,82958	4,141942	4,463165	3,99073	4,165556	4,003187	4,1448957	4,053157667	-0,091738	0,938391597	Psg-ps1
17237313	4,674807	4,672999	4,870688	4,658077	4,68979	4,595417	4,739498	4,647761333	-0,09173667	0,938392464	Caps2
17528247	5,146868	4,682697	5,145877	4,9987	5,055637	4,645898	4,991814	4,900078333	-0,09173567	0,938393115	Kbtbd13
17307935	7,428616	7,359211	7,216833	7,16345	7,211766	7,354317	7,3348867	7,243177667	-0,091709	0,93841046	Ppp2r2a
17287330	9,11143	9,068559	8,86592	8,841797	8,978699	8,950344	9,015303	8,923613333	-0,09168967	0,938423036	Cks2
17267329	8,301846	8,417449	8,248149	8,076558	8,33784	8,278023	8,3224813	8,230807	-0,09167433	0,93843301	Vmp1
17406783	7,1717	7,132699	7,288099	7,06635	7,108925	7,142246	7,1974993	7,105840333	-0,091659	0,938442984	Lmna
17333672	8,101937	8,224697	8,081697	8,037855	8,016761	8,078765	8,1361103	8,044460333	-0,09165	0,938448838	Riok2
17440132	10,16498	10,18387	10,13708	9,976015	10,16218	10,07279	10,161977	10,07032833	-0,09164833	0,938449922	---
17333805	3,678674	3,651848	3,155746	3,44674	3,630208	3,134409	3,4954227	3,403785667	-0,091637	0,938457294	Vmn2r100
17489571	3,700151	3,468399	3,589545	3,533936	3,554468	3,39478	3,5860317	3,494394667	-0,091637	0,938457294	Abpb
17404366	5,276218	5,304472	5,505993	5,27353	5,234906	5,303352	5,3622277	5,270596	-0,09163167	0,938460764	Agtr1b
17521070	7,925864	7,917694	8,04975	7,813321	7,959192	7,845939	7,964436	7,872817333	-0,09161867	0,93846922	Mrpl3
17204063	7,542624	7,671093	7,843863	7,768328	7,694161	7,320251	7,68586	7,594246667	-0,09161333	0,938472689	---
17341426	3,168967	3,331116	3,270892	3,149924	3,140206	3,206142	3,2569917	3,165424	-0,09156767	0,938502396	Vmn2r111
17382530	5,52597	5,605746	5,549668	5,076099	5,510409	5,820202	5,5604613	5,468903333	-0,091558	0,938508684	AA543186
17409328	9,783128	10,04776	9,990524	9,861618	9,838796	9,846343	9,9404707	9,848919	-0,09155167	0,938512804	Sars
17406268	3,35136	3,269884	3,375694	3,214301	3,266053	3,242028	3,3323127	3,240794	-0,09151867	0,938534272	1700028M03Rik
17202871	6,780984	6,995793	6,86249	6,317936	6,969718	7,077063	6,8797557	6,788239	-0,09151667	0,938535573	---
17533736	3,692712	3,668773	3,577052	3,580278	3,467674	3,616111	3,646179	3,554687667	-0,09149133	0,938552054	Ssxa1
17391871	5,935575	6,031357	6,549046	6,04929	6,095383	6,096838	6,1719927	6,080503667	-0,091489	0,938553572	Spef1
17205885	4,438391	4,872685	3,91098	4,394944	4,054356	4,498329	4,407352	4,315876333	-0,09147567	0,938562246	---
17539027	7,604652	7,583874	7,092335	7,288296	7,231388	7,486773	7,4269537	7,335485667	-0,091468	0,938567233	2210013O21Rik
17213593	6,986265	7,430853	6,941025	6,972296	7,121004	6,990457	7,119381	7,027919	-0,091462	0,938571137	Snora41
17396790	3,307581	3,265364	3,488431	3,205262	3,159948	3,421799	3,353792	3,262336333	-0,09145567	0,938575257	---
17473914	3,828651	4,027487	3,727296	3,810636	3,698369	3,80015	3,8611447	3,769718333	-0,09142633	0,938594341	Cabp5

17301283	5,081616	4,987889	5,398655	4,836322	5,222847	5,134774	5,1560533	5,064647667	-0,09140567	0,938607786	Prss52
17319870	8,94311	8,909999	9,042905	8,868041	8,94013	8,813776	8,965338	8,873982333	-0,09135567	0,938640316	Ttll12
17238844	7,594062	7,714149	7,631255	7,536069	7,676967	7,452402	7,6464887	7,555146	-0,09134267	0,938648774	Syne1
17309455	3,733018	3,585142	3,957551	3,63938	3,7305	3,63188	3,7585703	3,667253333	-0,091317	0,938665474	---
17405825	5,359344	4,852195	5,846216	5,020595	5,756493	5,006758	5,352585	5,261282	-0,091303	0,938674583	---
17337637	2,958075	3,066502	2,900222	2,746553	2,939525	2,964836	2,974933	2,883638	-0,091295	0,938679788	Olfr138
17361771	7,501488	7,620856	7,69067	7,560105	7,423087	7,555938	7,604338	7,513043333	-0,09129467	0,938680005	Sssca1
17404172	2,901158	3,021773	2,986593	2,851383	2,952963	2,831301	2,9698413	2,878549	-0,09129233	0,938681523	---
17426497	9,650538	9,696789	9,692156	9,762485	9,681007	9,322117	9,6798277	9,588536333	-0,09129133	0,938682174	Megf9
17219222	5,404699	5,338431	5,555517	5,398068	5,309587	5,317179	5,4328823	5,341611333	-0,091271	0,938695403	Mpz
17363666	5,843134	5,585485	5,213993	5,763631	5,129553	5,475668	5,5475373	5,456284	-0,09125333	0,938706898	---
17322477	7,931974	7,382452	7,972627	7,588521	7,816056	7,608724	7,762351	7,671100333	-0,09125067	0,938708634	---
17305941	3,508134	3,804787	3,298491	3,332319	3,533571	3,471774	3,5371373	3,445888	-0,09124933	0,938709501	Olfr729
17432256	5,151157	5,149107	5,267098	4,993911	5,194862	5,104872	5,1891207	5,097881667	-0,091239	0,938716225	Slc25a34
17446458	4,494481	4,664685	4,590216	4,619427	4,354793	4,501554	4,5831273	4,491924667	-0,09120267	0,938739866	LOC100862368 // LOC100862359 // Gm1
17486674	5,236101	4,66129	4,919565	4,951904	5,117305	4,474234	4,9389853	4,847814333	-0,091171	0,938760471	Sult2a4
17479303	6,262591	6,766302	6,429545	6,381193	6,452466	6,351326	6,486146	6,394995	-0,091151	0,938773485	Aen
17250072	4,420702	3,959319	4,327065	4,208246	3,964746	4,260664	4,2356953	4,144552	-0,09114333	0,938778474	Olfr325
17208451	4,862638	5,694623	4,705448	5,497923	4,411591	5,079787	5,0875697	4,996433667	-0,091136	0,938783246	---
17207749	5,073465	5,164009	4,668663	5,059563	5,36541	4,207787	4,9687123	4,877586667	-0,09112567	0,93878997	---
17450897	4,727977	4,879591	4,762766	4,780786	4,563037	4,753135	4,7901113	4,698986	-0,09112533	0,938790187	Vmn2r12
17274292	4,117312	3,567001	3,65547	3,658455	3,651165	3,756834	3,7799277	3,688818	-0,09110967	0,938800382	Mir3066
17416154	4,353987	4,366055	4,755484	4,320096	4,490106	4,392026	4,491842	4,400742667	-0,09109933	0,938807106	LOC622167
17454139	3,968383	3,800618	4,152288	3,837089	4,092231	3,718717	3,973763	3,882679	-0,091084	0,938817084	Gm15498
17310078	9,646707	9,691703	9,661589	9,597216	9,567433	9,562108	9,6666663	9,575585667	-0,09108067	0,938819253	Nup155
17444481	5,682274	5,609804	5,515162	5,550215	5,521019	5,462792	5,6024133	5,511342	-0,09107133	0,938825326	Bhlha15
17305327	6,484598	6,486944	6,478201	6,562576	6,63232	5,981638	6,4832477	6,392178	-0,09106967	0,938826411	---
17471231	4,556323	4,634403	5,012835	4,50221	4,713584	4,714565	4,7345203	4,643453	-0,09106733	0,938827929	Prmt8
17333932	7,383884	6,899311	7,127542	7,129925	7,202066	6,80562	7,1369123	7,045870333	-0,091042	0,938844415	Zfp53
17387858	3,835538	3,726109	3,554154	3,695149	3,457564	3,690045	3,705267	3,614252667	-0,09101433	0,93886242	Olfr1208
17283964	3,847656	3,523223	3,852682	3,564412	3,595695	3,790578	3,741187	3,650228333	-0,09095867	0,938898646	Mir299
17298925	9,857665	10,09486	9,94869	9,703766	9,898705	10,02589	9,9670717	9,876120333	-0,09095133	0,938903419	---
17463619	3,723409	3,07233	3,051173	3,016418	3,27463	3,28305	3,282304	3,191366	-0,090938	0,938912096	Prp2
17258521	8,419024	8,29801	8,328877	8,106918	8,402103	8,264163	8,348637	8,257728	-0,090909	0,93893097	H3f3b // H3f3b
17508532	4,179532	4,141512	4,101983	4,088912	4,033771	4,027663	4,141009	4,050115333	-0,09089367	0,938940949	Got1l1
17510707	8,861184	8,904575	8,647173	8,706306	8,599664	8,834291	8,8043107	8,713420333	-0,09089033	0,938943118	Lsm6
17474746	2,991195	3,577439	3,148242	3,239908	2,98152	3,222802	3,2389587	3,148076667	-0,090882	0,938948542	Vmn1r123
17247877	3,577396	3,395364	3,66657	3,465527	3,560813	3,340354	3,5464433	3,455564667	-0,09087367	0,938950711	4933430M04Rik
17449958	11,71851	12,09344	11,87322	11,79035	11,74268	11,87954	11,895057	11,80419	-0,09086667	0,938958521	Mir703
17328829	9,380303	9,456278	9,427058	9,397707	9,305521	9,287848	9,421213	9,330358667	-0,09085433	0,938966549	Slc25a1
17484080	9,041203	8,962772	8,930967	8,828167	8,994328	8,839947	8,978314	8,887480667	-0,09083333	0,938980216	Fam175b
17384563	4,407375	3,24362	3,985553	3,919408	3,581214	3,863432	3,8788493	3,788018	-0,09083133	0,938981518	Olfr351
17342916	4,617265	4,722948	4,676859	4,4929	4,471151	4,780599	4,6723573	4,58155	-0,09080733	0,938997139	Slc26a8
17204499	3,25191	4,654896	3,546403	4,357124	2,921441	3,902226	3,8177363	3,726930333	-0,090806	0,938998006	---
17543150	8,81411	8,829559	8,879422	8,67846	8,763854	8,808381	8,8410303	8,750231667	-0,09079867	0,939002779	Pola1
17260898	6,946888	6,858974	6,829224	6,595117	6,72581	7,041775	6,878362	6,787567333	-0,09079467	0,939005383	1110067D22Rik
17380604	5,85709	5,730535	5,643254	5,799231	5,447776	5,7115	5,7436263	5,652835667	-0,09079067	0,939007986	9030418K01Rik
17283303	7,627165	7,510408	7,328367	7,410779	7,448636	7,334171	7,4886467	7,397862	-0,09078467	0,939011892	Ccdc88c
17505953	6,471187	6,749944	6,526298	6,508508	6,422007	6,544566	6,5824763	6,491693667	-0,09078267	0,939013193	Gan
17209541	4,607513	5,702586	5,053037	5,289762	4,508988	5,292056	5,1210453	5,030268667	-0,09077667	0,939017099	---
17527914	4,549751	4,640194	4,643508	4,306531	4,625754	4,628871	4,611151	4,520385333	-0,09076567	0,939024258	4933433G08Rik
17314085	7,990756	8,08007	8,211873	8,066378	7,927022	8,017034	8,094233	8,003478	-0,090755	0,939031201	Ppp6r2
17533348	5,917096	5,619438	5,96417	5,753081	5,807379	5,668034	5,833568	5,742831333	-0,09073667	0,939043134	Gm4984
17342958	5,946024	5,632954	6,060125	5,817935	5,707496	5,841516	5,879701	5,788982333	-0,09071867	0,93905485	Pxt1
17203261	3,899488	4,215922	4,529318	3,996721	4,459814	3,916051	4,2149093	4,124195333	-0,090714	0,939057888	---
17217965	3,372311	3,347351	3,323884	3,141979	3,310435	3,31899	3,3478487	3,257134667	-0,090714	0,939057888	---
17373105	7,556582	7,205809	7,625936	7,442903	7,326612	7,346681	7,4627757	7,372065333	-0,09071033	0,939060274	C1qtnf4
17324616	4,985483	4,516114	4,633065	4,754162	4,615652	4,492748	4,711554	4,620854	-0,0907	0,939067001	---
17370641	8,688273	8,660881	8,682321	8,617584	8,56022	8,581575	8,6771583	8,586459667	-0,09069867	0,939067868	Epc2
17297849	4,737512	4,559866	4,71506	4,669547	4,467496	4,603354	4,6708127	4,580131	-0,09068167	0,939078934	Gm10394
17253901	6,500837	6,576917	6,604912	6,597476	6,547606	6,265561	6,5608887	6,470214333	-0,09067433	0,939083707	Rnf135
17373596	4,040684	4,051628	4,17118	3,968144	4,083891	3,939437	4,0878307	3,997157333	-0,09067333	0,939084358	4921507L20Rik
17201655	7,099946	6,140003	6,618311	6,792161	6,188104	6,606018	6,61942	6,528761	-0,090659	0,939093688	---
17521722	7,087579	7,149882	7,308595	7,072894	7,046647	7,154548	7,1820187	7,091363	-0,09065567	0,939095858	Ccdc71
17514924	5,41634	4,593963	4,891093	5,020713	4,737648	4,871109	4,967132	4,87649	-0,090642	0,939104754	Olfr845
17330852	4,249536	4,343085	4,091907	4,185372	4,128601	4,098639	4,228176	4,137537333	-0,09063867	0,939106924	1700026J12Rik
17295270	10,85357	10,84493	10,88896	10,79493	10,77625	10,74439	10,862487	10,77185667	-0,09063	0,939112565	Nsa2
17410063	9,052628	9,310753	9,179125	9,126878	9,204119	8,93963	9,1808353	9,090209	-0,09062633	0,939114952	Snhg8
17396673	5,432982	5,494683	5,658687	5,280007	5,531754	5,502764	5,528784	5,438175	-0,090609	0,939126235	4930429B21Rik
17225871	3,800826	3,783504	3,684781	3,874764	3,478429	3,644118	3,7563703	3,665770333	-0,0906	0,939132094	Slco6c1
17528930	3,547465	3,203831	3,732917	3,637815	3,486419	3,088236	3,4947377	3,404156667	-0,090581	0,939144462	---
17417269	8,602734	8,749913	8,358806	8,505877	8,434492	8,49935	8,5704843	8,479906333	-0,090578	0,939146415	4732418C07Rik
17359143	3,801692	3,77135	3,749704	3,765802	3,678499	3,606765	3,7742487	3,683688667	-0,09056	0,939158133	Lgi1
17340998	6,647313	6,300723	6,366806	6,242942	6,592911	6,207316	6,4382807	6,347723	-0,09055767	0,939159652	Gpr31b
17397827	6,759233	6,612831	6,712392	6,581336	6,602629	6,628888	6,6948187	6,604284333	-0,09053433	0,939174841	Med12l
17494656	6,685315	7,479144	6,41088	6,828942	6,76184	6,712961	6,8584463	6,767914333	-0,090532	0,93917636	Gm4070 // Gm4070
17201757	4,20032	4,244856	4,006095	3,917929	4,071653	4,190119	4,1504237	4,059900333	-0,09052333	0,939182002	---
17201933	4,20032	4,244856	4,006095	3,917929	4,071653	4,190119	4,1504237	4,059900333	-0,09052333	0,939182002	---
17270410	6,083429	5,973465	6,380991	6,111979	6,055045	5,999335	6,1459617	6,055453	-0,09050867	0,93919155	Plcd3
17505475	5,84481	5,528622	5,182863	5,236012	5,738451	5,310357	5,518765	5,428273333	-0,09049167	0,939202617	---
17391139	4,444149	4,730277	4,46185	4,375598	4,347437	4,641799	4,5454253	4,454944667	-0,09048067	0,939209778	Usp50
17280626	4,818029	4,831348	4,949762	4,825486	4,812366	4,689849	4,8663797	4,775900333	-0,09047933	0,939210646	4933406C10Rik
17382032	4,884532	4,682972	4,771353	4,871914	4,641608	4,554001	4,779619	4,689174333	-0,09044467	0,939233215	BC061194
17425867	5,634312	5,419664	6,011072	5,90457	5,500619	5,388531	5,6883493	5,597906667	-0,09044267	0,939234517	---
17486465	3,474822	3,370027	3,544488	3,226981	3,851485	3,039591	3,4631123	3,372685667	-0,09042667	0,939244933	Vmn1r71
17511377	6,714326	6,821905	6,575368	6,589674	6,692682	6,557984	6,7038663	6,613446667	-0,09041967	0,93924949	Neto2
17536945	7,098577	6,802066	7,383427	7,068566	7,091395	6,852877	7,09469	7,004279333	-0,09041067	0,93925535	1700011M02Rik
17433305	4,886699	4,910325	4,832682	4,646106	4,842291	4,870128	4,8765687	4,786175	-0,09039367	0,939266418	1700045H11Rik
17333977	6,614895	6,575677	6,547566	6,412555	6,56101	6,4934	6,5793793	6,488988333	-0,090391	0,939268154	Zfp229
17481330	6,060684	5,828961	6,176614	5,821747	5,854271	6,119071	6,0220863	5,931696333	-0,09039	0,939268805	Olfr661
17521971	6,207214	6,140547	6,391497	6,156482	6,127254	6,184383	6,2464193	6,156039667	-0,09037967	0,939275532	Slc26a6
17550823	3,578592	3,347622	3,4896	3,360309	3,405133	3,37924	3,471938	3,381560667	-0,09037733	0,939277051	---
17312986	5,225957	5,386031	5,474092	5,449066	5,126984	5,238901	5,3620267	5,271650333	-0,09037633	0,939277703	---
17541612	9,011382	8,989415	8,512783	8,571677	8,809125	8,861651	8,83786	8,747484333	-0,09037567	0,939278137	Rap2c
17242046	6,724686	6,556028	6,549536	6,64712	6,57207	6,339962	6,6100833	6,519717333	-0,090366	0,93928443	Dip2a
17551101	8,823518	8,900372	8,817475	8,688326	8,751263	8,830694	8,8471217	8,756761	-0,09036067	0,939287903	---
17404174	4,512113	4,311946	3,871205	4,053347	4,053347	4,317492	4,2317547	4,141395333	-0,09035933	0,939288771	1810022K09Rik
17283225	6,540632	6,39274	6,604095	6,278969	6,63814	6,349301	6,512489	6,422136667	-0,09035233	0,939293328	BC002230
17447078	6,038718	6,633385	6,348763	6,176389	6,248232	6,325193	6,3402887	6,249938	-0,09035067	0,939294413	Ywhah // Ywhah
17494199	3,426143	3,314039	3,361058	3,359629	3,209607	3,260959	3,36708	3,276731667	-0,09034833	0,939295932	Olfr629
17316187	4,449581	4,354352	4,740273	4,663158	4,154729	4,455275	4,5147353	4,424387333	-0,090348	0,939296149	---
17480962	6,658738	6,507151	6,658738	6,561916	6,498851	6,492867	6,608209	6,517878	-0,090331	0,939307218	3200002M19Rik
17203187	3,958677	4,218045	4,078512	3,888936	3,993696	4,10167	4,085078	3,994767333	-0,09031067	0,939320456	---
17494172	6,333636	5,872457	6,051974	6,079347	5,861957	6,045843	6,0860223	5,995715667	-0,09030667	0,939323061	Olfr605
17286621	8,086295	8,390924	8,224762	7,952847	8,205016	8,273236	8,2339937	8,143699667	-0,090294	0,939331308	Riok1
17219635	3,396189	3,398858	2,931213	3,315989	3,201069	2,938354	3,2420867	3,151804	-0,09028267	0,939338687	Olfr1404
17517284	4,297938	4,635548	4,719513	4,224157	4,712074	4,445932	4,5509997	4,460721	-0,09027867	0,939341291	4930510E17Rik
17468247	5,080916	4,780684	4,763368	4,873654	4,744238	4,73634	4,8749893	4,784744	-0,09024533	0,939362995	1700124L16Rik
17384744	5,238805	4,567476	5,119474	4,855337	4,944472	4,855223	4,9752517	4,885010667	-0,090241	0,939365816	Rpl35
17270134	9,473633	9,486115	9,456685	9,281235	9,449537	9,414951	9,4721443	9,381907667	-0,09023667	0,939368638	Ubtf
17265777	3,085324	3,106743	3,112412	3,01881	2,989898	3,025112	3,101493	3,011273333	-0,09021967	0,939379707	Olfr381
17299170	6,221434	6,037578	5,807096	5,953419	5,848556	5,993499	6,022036	5,931824667	-0,09021133	0,939385133	---
17348681	4,10494	3,643431	3,645057	3,911424	3,669239	3,542147	3,7978093	3,707603333	-0,090206	0,939388606	---
17366618	5,3617	5,045296	5,079557	5,163856	5,016524	5,035644	5,1621843	5,072008	-0,09017633	0,939407923	Gm13391
17213948	3,986138	3,831938	3,882071	3,717883	3,945465	3,766322	3,900049	3,80989	-0,090159	0,93941921	---
17530229	4,740752	4,554061	4,411414	4,540465	4,338616	4,556704	4,5687423	4,478595	-0,09014733	0,939426807	---
17276921	4,322744	4,398795	4,470006	4,165111	4,341532	4,414486	4,3971817	4,307043	-0,09013867	0,93943245	Mir3067
17380021	7,201044	7,437256	7,284966	7,354964	7,09384	7,204095	7,3077553	7,217633	-0,09012233	0,939443086	Tshz2
17549258	4,14837	4,032986	4,221393	3,968956	4,267909	3,895531	4,1342497	4,044132	-0,09011767	0,939446125	---
17493130	3,533375	3,524001	3,309125	3,309125	3,333663	3,453524	3,4555003	3,365437333	-0,090063	0,939481723	Olfr307
17391598	7,758973	8,074166	8,035746	7,852384	8,033831	7,712517	7,956295	7,866244	-0,090051	0,939489537	Snrpb
17284362	6,946078	5,852214	7,008779	6,94346	5,784373	6,809098	6,602357	6,512310333	-0,09004667	0,939492359	---
17465006	5,147246	5,196599	4,914411	4,898553	5,25262	4,836969	5,0860853	4,996047333	-0,090038	0,939498003	Cttnbp2
17324643	9,657091	9,690752	9,565665	9,658111	9,538761	9,446526	9,637836	9,547799333	-0,09003667	0,939498871	Bdh1
17260727	4,086697	3,865195	3,779699	3,820001	3,905498	3,735993	3,9105303	3,820497333	-0,090033	0,939501259	1700046C09Rik
17267775	8,073426	8,1609	7,958091	7,901636	8,009848	8,010863	8,064139	7,974115667	-0,09002333	0,939507554	Utp18
17390212	4,848305	5,003894	4,865646	4,751144	4,795873	4,900802	4,9059483	4,815939667	-0,09000867	0,939517105	4931402G19Rik
17536351	2,945141	2,884955	2,954356	2,914432	2,786486	2,813553	2,9281507	2,838157	-0,08999367	0,939526874	---
17291104	3,665661	3,862282	3,763465	3,706432	3,624891	3,690166	3,7638027	3,673829667	-0,089973	0,939540333	Vmn1r212
17356974	5,914707	6,046886	6,099034	5,895497	6,014309	5,880916	6,020209	5,930240667	-0,08996833	0,939543372	Bad
17279001	7,213491	7,188722	7,423834	7,223532	7,185995	7,14662	7,275349	7,185382333	-0,08996667	0,939544457	Wdr20a
17353206	3,750995	3,625718	3,458067	3,52316	3,520871	3,520871	3,6115933	3,521634	-0,08995933	0,939549233	4930578E11Rik
17486418	3,985568	3,169087	3,773747	3,809298	3,272153	3,577105	3,6428007	3,552852	-0,08994867	0,939556179	Vmn2r51
17288876	10,07393	10,08689	9,84771	9,743311	9,845961	10,14947	10,002843	9,912914	-0,08992933	0,93956877	Arrdc3

17256991	4,878481	5,072253	5,138391	5,048123	4,818408	4,952814	5,0297083	4,939781667	-0,08992667	0,939570507	Adam11
17489318	5,111254	5,299341	5,001929	4,890472	5,005328	5,24698	5,137508	5,047593333	-0,08991467	0,939578322	Ffar1
17268066	5,753893	5,892159	6,357214	6,031772	5,564963	6,1369	6,0010887	5,911211667	-0,089877	0,939602854	Gm11545
17448048	3,380836	3,341731	3,149996	3,251763	3,228719	3,122548	3,2908543	3,20101	-0,08984433	0,939624129	---
17207733	4,201062	4,571285	4,013588	4,216593	3,840996	4,458817	4,2619783	4,172135333	-0,089843	0,939624998	---
17497113	7,186318	7,2554	7,271262	7,123576	6,973019	7,346867	7,23766	7,147820667	-0,08983933	0,939627386	Fam53b
17258239	7,533433	7,643871	7,610035	7,404257	7,561136	7,552434	7,5957797	7,505942333	-0,08983733	0,939628688	Nup85
17200007	4,688221	4,49994	4,843276	4,646002	4,951439	4,164516	4,6771457	4,587319	-0,08982667	0,939635635	---
17548658	11,91694	11,97377	11,92907	11,84819	11,82641	11,8757	11,939927	11,8501	-0,08982667	0,939635635	Prdx1
17453383	6,839641	6,806307	6,849168	6,605008	6,952922	6,667719	6,8317053	6,741883	-0,08982233	0,939638458	Clip2
17233319	3,917459	3,822448	3,933169	3,745059	3,846551	3,81202	3,8910253	3,80121	-0,08981533	0,939643017	Gm19395
17431516	6,543557	6,720467	6,877689	6,75287	6,594749	6,524667	6,7139043	6,624095333	-0,089809	0,939647142	1110049F12Rik
17399396	5,942345	5,824327	5,922597	5,842827	5,852558	5,724464	5,896423	5,806616333	-0,08980667	0,939648662	Dpm3
17366726	3,977548	4,177511	3,6498	3,763304	3,736143	4,036045	3,934953	3,845164	-0,089789	0,939660168	---
17345586	7,634098	8,074258	7,638956	7,720108	7,524742	7,833115	7,7824373	7,692655	-0,08978233	0,93966451	Rpl7l1
17543196	7,863093	7,928779	7,856367	7,827222	7,779035	7,772655	7,8827463	7,792970667	-0,08977567	0,939668853	Pdk3
17296723	4,765866	4,710527	4,352681	4,58688	4,234313	4,738599	4,6096913	4,519930667	-0,08976067	0,939678623	Gm3099
17447487	7,79993	7,924925	8,047708	7,715585	7,652339	8,13538	7,9241877	7,834434667	-0,089753	0,939683616	D5Ertd579e
17302925	4,471529	4,145231	4,29995	4,350208	4,231168	4,066106	4,30557	4,215827333	-0,08974267	0,939690347	---
17424878	3,905532	3,798257	3,783429	3,568559	3,654606	3,994832	3,8290727	3,739332333	-0,08974033	0,939691867	---
17294222	10,28773	10,59229	10,39464	10,29202	10,34326	10,37018	10,424887	10,33515333	-0,08973333	0,939696426	Rpl9
17392180	3,216401	3,321361	3,291984	3,104869	3,214795	3,240954	3,276582	3,186872667	-0,08970933	0,939712058	---
17244942	4,09749	4,393693	4,545407	4,226491	4,252141	4,28883	4,34553	4,255820667	-0,08970933	0,939712058	LOC629206
17399432	8,774069	8,605065	8,669374	8,71994	8,485014	8,574484	8,682836	8,593146	-0,08969	0,939724651	Shc1
17221999	3,105596	3,401938	3,115571	3,000116	3,322122	3,031811	3,2077017	3,118016333	-0,08968533	0,939727691	---
17251714	5,007629	4,721173	4,912745	4,739909	4,8301	4,802505	4,8805157	4,790838	-0,08967767	0,939732685	Sox15
17435920	7,553325	7,722005	7,716222	7,736862	7,509562	7,476111	7,6638507	7,574178333	-0,08967233	0,939736159	Emilin1
17265773	3,255342	3,552757	3,447371	3,219921	3,407447	3,359088	3,41849	3,328818667	-0,08967133	0,93973681	Olfr380
17392625	5,935463	5,675314	6,489974	5,967426	5,899497	5,964851	6,0335837	5,943924667	-0,089659	0,939744844	---
17301722	8,775862	8,739609	8,948561	8,586235	8,794219	8,814685	8,821344	8,731713	-0,089631	0,939763083	Bin3
17306975	9,189091	9,443568	9,512834	9,116802	9,623922	9,135886	9,381831	9,292203333	-0,08962767	0,939765254	Ctsg
17254249	8,664972	8,874986	8,944723	8,798005	8,702128	8,715698	8,828227	8,738610333	-0,08961667	0,93977242	Taf15
17289426	11,3193	11,51585	11,46299	11,3422	11,30255	11,38454	11,432713	11,34309667	-0,08961667	0,93977242	Gm5453
17399235	6,196982	5,869539	5,452975	5,892109	5,772684	5,585917	5,839832	5,750236667	-0,08959533	0,939786316	---
17204241	3,82331	3,377376	3,777542	3,662359	3,575781	3,47133	3,6594093	3,569823333	-0,089586	0,939792396	---
17361850	6,798323	6,622371	6,753011	6,44887	6,927674	6,528408	6,7245683	6,634984	-0,08958433	0,939793482	Cdc42ep2
17462191	10,30886	10,30527	10,24281	10,14579	10,19041	10,25201	10,285647	10,19607	-0,08957667	0,939798476	Hnrnpf
17472070	7,07276	6,982289	6,671432	6,985937	7,06468	6,407177	6,908827	6,819264667	-0,08956233	0,939807813	LOC100861778
17243868	7,253974	7,353079	7,456748	7,233254	7,323276	7,238593	7,3546003	7,265041	-0,08955933	0,939809767	Dram1
17471278	6,124973	6,400757	6,21307	6,060532	6,193721	6,215918	6,2462667	6,156723667	-0,089543	0,939820407	Tead4
17201789	3,951777	3,906527	4,873976	3,8252	4,398959	4,239505	4,2440933	4,154554667	-0,08953867	0,93982323	---
17201965	3,951777	3,906527	4,873976	3,8252	4,398959	4,239505	4,2440933	4,154554667	-0,08953867	0,93982323	---
17419041	4,710081	5,356791	5,235385	5,021388	5,084404	4,927921	5,1007523	5,011237667	-0,08951467	0,939838865	---
17236729	4,79235	4,652568	4,750044	4,801911	4,677276	4,447258	4,731654	4,642148333	-0,08950567	0,939844728	2310039L15Rik // 2310039L15Rik
17289889	10,06456	10,34943	9,98879	10,11517	10,04613	9,973011	10,13426	10,04477033	-0,08948967	0,939855151	Il6st
17438315	9,566895	9,312428	9,700294	9,531434	9,615206	9,164543	9,526539	9,437061	-0,089478	0,939862751	---
17412679	6,02252	5,256446	5,527742	5,467417	5,580009	5,490955	5,602236	5,512793667	-0,08944233	0,939885987	---
17218290	4,029437	3,921883	4,174863	4,129992	3,903356	3,824565	4,042061	3,952637667	-0,08942333	0,939898365	1700012A16Rik
17450477	3,879382	3,725409	3,565898	3,673854	3,62106	3,607513	3,723563	3,634142333	-0,08942067	0,939900103	Gbp6
17278509	3,919369	4,159437	4,161451	4,12982	3,987657	3,854543	4,0800857	3,990673333	-0,08941233	0,939905532	---
17524351	3,449318	2,973156	3,040533	3,100718	3,110076	2,98402	3,1543357	3,064938	-0,08939767	0,939915087	Olfr866
17341769	7,852484	7,773835	8,101089	7,880764	7,764581	7,813891	7,909136	7,819745333	-0,08939067	0,939919648	1600002H07Rik
17202013	3,379627	3,514088	3,473115	3,491082	3,16218	3,445403	3,45561	3,366221667	-0,08938833	0,939921168	---
17335352	5,552476	5,355251	5,664983	5,384924	5,322466	5,597229	5,5242367	5,434873	-0,08936367	0,939937238	Gm749
17444080	9,884326	9,960605	9,952396	9,79022	9,875665	9,863362	9,9324423	9,843082333	-0,08936	0,939939627	Eif3b
17536566	8,455941	8,599126	8,518496	8,362566	8,446192	8,496727	8,524521	8,435161667	-0,08935933	0,939940062	Kif4
17355298	4,306694	4,403553	4,161698	4,005245	4,488059	4,110596	4,2906483	4,2013	-0,08934833	0,939947228	2310002L13Rik
17322485	9,624564	9,658249	9,369542	9,309422	9,336991	9,737915	9,550785	9,461442667	-0,08934233	0,939951137	---
17362453	9,785621	9,912494	9,941534	9,804302	9,771282	9,796067	9,879883	9,790550333	-0,08933267	0,939957436	Slc3a2
17507910	6,743927	6,539631	6,879886	6,757161	6,465245	6,673157	6,721148	6,631854333	-0,08929367	0,939982846	Defa-rs1
17359327	4,308971	4,655068	4,53857	4,602688	4,381763	4,250334	4,5008697	4,411595	-0,08927467	0,939995225	Cyp2c50
17317127	4,287722	4,470886	4,423817	4,343727	4,331698	4,239192	4,3941417	4,304872333	-0,08926933	0,9399987	Gm9920
17282104	8,965373	8,909944	8,653026	8,580467	8,873299	8,806795	8,842781	8,753520333	-0,08926067	0,940004347	Atp6v1d
17398769	6,435476	6,394434	6,846027	6,492631	6,661671	6,253863	6,5586457	6,469388333	-0,08925733	0,940006519	Etv3
17210603	4,167847	4,703875	4,88258	4,474335	4,667941	4,344295	4,5847673	4,495523667	-0,08924367	0,940015423	---
17240181	5,845554	5,797359	5,716978	5,694596	5,822037	5,57559	5,7866303	5,697407667	-0,08922267	0,940029106	Fam26e
17357513	8,873091	8,907179	8,865034	9,114666	8,585087	8,6779	8,881768	8,792551	-0,089217	0,940032799	---
17300193	4,264005	4,55896	4,901934	4,34285	4,368284	4,746239	4,5749663	4,485791	-0,08917533	0,940059948	---
17542834	3,579487	3,545342	3,382039	3,410718	3,530337	3,298378	3,5022893	3,413144333	-0,089145	0,940079714	---
17352462	10,25768	10,13883	10,18461	10,13481	10,02542	10,15346	10,193707	10,10456333	-0,08914333	0,9400808	Kif5b
17355508	5,864371	5,934381	6,292443	5,955895	6,035314	5,832556	6,0303983	5,941255	-0,08914333	0,9400808	St8sia5
17468417	5,76513	5,88236	6,323977	6,118872	5,802687	5,782489	5,990489	5,901349333	-0,08913967	0,940083189	Cml3
17496063	7,845575	7,789404	7,909112	7,733444	7,737474	7,805756	7,8480303	7,758891333	-0,089139	0,940083623	Gtf3c1
17299341	8,406042	8,438438	8,393354	8,273476	8,311928	8,385037	8,4126113	8,323480333	-0,089131	0,940088836	Fbxo34
17362494	8,618947	8,73833	8,504858	8,603175	8,534504	8,457103	8,6207117	8,531594	-0,08911767	0,940097525	Polr2g
17435162	6,373798	6,35386	6,397434	6,253693	6,417509	6,186665	6,3750307	6,285955667	-0,089075	0,940125328	---
17201235	3,50324	2,845885	3,284574	2,790239	3,311191	3,26505	3,211233	3,12216	-0,089073	0,940126631	---
17264117	5,075669	5,006129	4,913139	4,89917	4,941135	4,887462	4,9983123	4,909255667	-0,08905667	0,940137275	Hs3st3a1 // Hs3st3a1
17369540	7,600193	7,677869	7,670548	7,652278	7,561472	7,467704	7,6495367	7,560484667	-0,089052	0,940140316	Exosc2
17472306	3,260478	3,502118	3,391683	3,213682	3,302967	3,370508	3,3847597	3,295719	-0,08904067	0,940147701	---
17203213	5,656744	5,009623	5,219699	5,443586	4,959338	5,216028	5,2953553	5,206317333	-0,089038	0,940149439	---
17372066	4,071806	3,970492	4,196368	4,094381	4,023367	3,853819	4,0795553	3,990522333	-0,089033	0,940152697	Hoxd9
17204181	5,398071	4,481506	3,832037	4,654041	4,481506	4,308971	4,570538	4,481506	-0,089032	0,940153349	---
17547575	5,541112	5,217647	5,02413	5,118038	5,192121	5,205644	5,260963	5,171934333	-0,08902867	0,940155521	---
17478985	8,816011	8,952856	8,912506	8,764252	8,801693	8,848351	8,893791	8,804765333	-0,08902567	0,940157476	Snrpa1
17467068	4,202971	4,32195	4,562995	4,294537	4,286422	4,240024	4,3626387	4,273661	-0,08897767	0,940188757	Neurod6
17444464	7,947461	8,031228	7,90961	7,953262	7,792871	7,875261	7,9627663	7,873798	-0,08896833	0,940194839	Lmtk2
17494692	3,520694	3,429365	3,39808	3,327935	3,36801	3,385313	3,4493797	3,360419333	-0,08896033	0,940200053	Olfr697
17308128	4,072678	4,001227	4,011456	3,953678	3,813317	4,051528	4,0284537	3,939507667	-0,088946	0,940209394	Nkx2-6
17311853	5,861537	5,701035	5,67189	5,813371	5,666965	5,487342	5,7448207	5,655892667	-0,088928	0,940221125	Pvt1
17249965	6,909284	7,006453	6,816634	6,748098	6,823553	6,893937	6,9107903	6,821862667	-0,08892767	0,940221342	Mrpl22
17286505	4,604662	4,570899	4,511664	4,536092	4,538151	4,346217	4,5624083	4,473486667	-0,08892167	0,940225252	---
17396702	7,463439	7,700677	7,609591	7,501153	7,340218	7,665587	7,5912357	7,502319333	-0,08891633	0,940228728	Zfp639
17479974	5,310154	5,090486	5,254982	5,014243	5,049694	5,32499	5,2185407	5,129642333	-0,08889833	0,940240459	Nox4
17327909	6,515432	6,385213	6,776529	6,29297	6,630532	6,487015	6,559058	6,470172333	-0,08888567	0,940248714	Ppl
17294736	3,44605	3,481405	3,384278	3,40667	3,241859	3,39663	3,4372443	3,348386333	-0,088858	0,940266745	---
17548411	3,984634	3,849521	4,148782	3,967732	4,003275	3,745364	3,9943123	3,905457	-0,08885533	0,940268483	Hpse // Hpse
17346572	7,37558	7,501348	7,397169	7,329618	7,418289	7,259653	7,424699	7,335853333	-0,08884567	0,940274784	Gpr108
17283242	5,445743	5,530329	5,442743	5,329158	5,411551	5,411607	5,4729383	5,384105333	-0,088833	0,940283039	Calm1 // Calm1
17502369	3,631996	4,308394	4,277862	3,826309	3,966475	4,15904	4,0727507	3,983941333	-0,08880933	0,940298464	Olfr373
17477046	5,04596	5,103083	4,267396	4,46732	4,967545	4,715159	4,8054797	4,716674667	-0,088805	0,940301289	Zfp936
17426638	6,131202	5,927481	6,062025	6,01319	5,931301	5,909873	6,040236	5,951454667	-0,08878133	0,940316714	Ptprd
17324742	4,207991	4,498294	4,526597	4,079494	4,519218	4,367837	4,4109607	4,322183	-0,08877767	0,940319104	---
17399162	6,692445	6,593073	6,735004	6,660524	6,488591	6,605104	6,6735073	6,584739667	-0,08876767	0,940325621	---
17237033	6,174708	5,727269	5,984449	6,309376	5,284494	6,026274	5,962142	5,873381333	-0,08876067	0,940330184	---
17333373	5,422374	5,226936	5,286702	5,309835	5,083637	5,276294	5,312004	5,223255333	-0,08874867	0,940338005	Gm10512
17380790	7,043097	7,165236	7,159233	6,973188	7,095603	7,032537	7,122522	7,033776	-0,088746	0,940339744	1600027N09Rik
17503559	8,69014	8,505257	8,469756	8,398598	8,506033	8,494287	8,555051	8,466306	-0,088745	0,940340395	Tmem188
17499155	5,320683	5,231346	5,542781	5,190142	5,383845	5,254624	5,3649367	5,276203667	-0,088733	0,940348217	Mcf2l
17275865	2,858603	2,822368	2,815713	2,741793	2,733837	2,754883	2,832228	2,743504333	-0,08872367	0,9403543	---
17358240	5,149415	4,937745	5,347299	4,956017	5,218723	4,99363	5,1448197	5,056123333	-0,08869633	0,940372117	Apba1
17309979	3,936388	3,796945	3,761326	3,715572	3,677166	3,835832	3,831553	3,742856667	-0,08869633	0,940372117	---
17545181	4,256069	3,931544	3,767509	4,006127	3,957932	3,724993	3,9850407	3,896350667	-0,08869	0,940376245	Il13ra2
17276151	6,296416	6,226616	6,261629	6,2423	6,136753	6,13955	6,2615537	6,172867667	-0,088686	0,940378852	---
17355439	3,317093	3,274019	3,14325	3,199539	3,190172	3,078648	3,2447873	3,156119667	-0,08866767	0,940390802	---
17375590	6,856073	6,479128	6,511718	6,441118	6,86525	6,27458	6,6156397	6,526982667	-0,088657	0,940397755	Slc24a5
17399995	9,990695	9,968868	9,989538	9,898449	9,941128	9,843615	9,9830337	9,894397333	-0,08863633	0,940411226	S100a11
17410830	10,39427	10,52347	10,31	10,21948	10,28815	10,45423	10,409247	10,32062	-0,08862667	0,940417528	Sh3glb1
17370475	4,337047	4,722329	4,727789	4,48449	4,667425	4,369371	4,5957217	4,507095333	-0,08862633	0,940417745	4933433P16Rik
17268454	6,865228	6,993674	6,934349	6,712248	6,942617	6,872518	6,9310837	6,842461	-0,08862267	0,940420135	Tbkbp1
17333323	11,35114	11,41812	11,40053	11,29343	11,31196	11,29855	11,38993	11,30131333	-0,08861667	0,940424046	Tcp1
17285330	3,944052	3,773582	3,939954	3,715285	3,896201	3,780259	3,8858627	3,797248333	-0,08861433	0,940425567	---
17466798	4,393095	4,171437	4,198758	4,3381	4,065898	4,093457	4,25443	4,165818333	-0,08861167	0,940427305	Hoxa1
17427746	7,856902	8,191957	7,538759	7,498833	7,737704	8,08526	7,8625393	7,773932333	-0,088607	0,940430347	Prkaa2
17337467	4,63462	4,590425	5,067655	4,686779	4,797484	4,542654	4,7642333	4,675639	-0,08859433	0,940438604	Trim31
17382709	4,905787	4,920561	5,031929	4,724065	4,957151	4,911325	4,952759	4,864180333	-0,08857867	0,940448817	4921530D09Rik
17471087	5,309543	5,293654	6,002158	5,617781	5,173378	5,548671	5,5351183	5,44661	-0,08850833	0,940494666	Tuba3a
17339989	11,21221	11,6711	11,33986	11,18445	11,24774	11,52555	11,407723	11,31924667	-0,08847667	0,94051531	---
17395863	5,955408	6,083239	6,09748	5,943594	5,913657	6,013567	6,0453757	5,956939333	-0,08843633	0,940541604	Zbtb46
17395488	9,915744	9,870528	9,949515	9,871228	9,804722	9,794533	9,911929	9,823494333	-0,08843467	0,940542691	Psma7
17341326	4,266972	4,163429	3,810003	4,104387	4,056093	3,814625	4,0801347	3,991701667	-0,088433	0,940543777	Vmn2r109
17516410	3,433285	4,38788	4,154015	4,239733	3,803376	3,666818	3,9917267	3,903309	-0,08841767	0,940553774	Mirlet7a-2
17232901	5,321703	5,493867	5,387798	5,303616	5,356805	5,277694	5,4011227	5,312705	-0,08841767	0,940553774	Ccdc162
17201191	8,987859	8,789401	8,606706	8,527807	8,767662	8,823313	8,7946553	8,706260667	-0,08839467	0,940568768	---

17275752	4,455984	4,703453	4,830429	4,69815	4,307758	4,71883	4,6632887	4,574912667	-0,088376	0,940580938	---
17295944	9,526536	9,810249	9,749282	9,794269	9,414731	9,612016	9,6953557	9,607005333	-0,08835033	0,940597672	Ppwd1
17481602	4,580116	4,093265	4,778034	4,397479	4,442436	4,346474	4,483805	4,395463	-0,088342	0,940603105	Olfr479
17246469	4,312866	4,415703	4,431814	4,348691	4,236499	4,310176	4,3867943	4,298455333	-0,088339	0,940605061	Neurod4
17519598	10,2836	10,17444	9,92661	9,973085	9,944064	10,20249	10,128217	10,03987967	-0,088337	0,940606365	Elovl5
17549264	3,994832	3,862058	4,122611	3,892678	4,09056	3,731306	3,993167	3,904848	-0,088319	0,940618101	---
17457860	3,2112	3,204973	3,585675	3,197971	3,376839	3,162102	3,3339493	3,245637333	-0,088312	0,940622665	Sval3
17234963	6,929614	6,730471	7,02451	6,905689	6,691391	6,822583	6,894865	6,806554333	-0,08831067	0,940623534	Palm
17524578	7,805891	7,468502	7,607405	7,472789	7,417717	7,726367	7,627266	7,538957667	-0,08830833	0,940625055	S1pr2
17526175	7,679923	7,579951	7,664618	7,562538	7,529361	7,567672	7,6414973	7,553190333	-0,088307	0,940625925	Abcg4
17302565	5,031107	4,609341	4,650996	4,817707	4,57366	4,635168	4,7638147	4,675511667	-0,088303	0,940628533	4930449E01Rik
17300108	3,68416	3,494044	3,529236	3,536081	3,57545	3,331002	3,5691467	3,480844333	-0,08830233	0,940628967	---
17517500	8,038686	8,183117	8,038646	8,016882	7,949936	8,028727	8,0868163	7,998515	-0,08830133	0,940629619	Fbxo22
17238884	5,37318	5,107877	5,572832	5,646502	5,093684	5,048887	5,3512963	5,263024333	-0,088272	0,940648745	Syne1
17541234	9,635643	9,825068	9,684994	9,626028	9,640321	9,614645	9,715235	9,626998	-0,088237	0,940671565	Thoc2
17200451	7,280706	6,902288	7,096314	6,853743	7,168031	6,992846	7,0931027	7,004873333	-0,08822933	0,940676564	---
17372929	3,375086	3,274917	3,303476	3,276738	3,23939	3,172688	3,3178263	3,229605333	-0,088221	0,940681998	Olfr1206
17371800	7,828024	7,866006	7,661141	7,601602	7,698765	7,790144	7,785057	7,696837	-0,08822	0,94068265	Itga6
17300175	3,729382	3,914159	4,043947	3,712167	3,821963	3,888749	3,8958293	3,807626333	-0,088203	0,940693734	---
17266004	6,210594	6,074866	6,113323	6,056808	6,010276	6,067108	6,1329277	6,044730667	-0,088197	0,940697646	4931413K12Rik
17465601	3,426133	2,96274	3,02564	3,067315	3,001409	3,081214	3,138171	3,049979333	-0,08819167	0,940701124	Mir29a
17284546	2,984589	2,9347	2,898438	2,84338	2,892934	2,816844	2,9392423	2,851052667	-0,08818967	0,940702428	---
17391483	2,748616	2,774894	2,703882	2,64032	2,85296	2,469585	2,742464	2,654288333	-0,08817567	0,940711557	---
17548746	7,35735	7,6226	7,420108	7,352509	7,467735	7,315289	7,466686	7,378511	-0,088175	0,940711992	Bace1
17389855	5,562037	5,626168	5,779818	5,557806	5,486494	5,659232	5,6560077	5,567844	-0,08816367	0,940719381	Gm14207
17327445	3,861115	4,046733	3,749763	3,843302	3,861594	3,688226	3,8858703	3,797707333	-0,088163	0,940719816	Dennd1b // Dennd1b
17500712	4,042219	3,994832	3,909283	3,955553	3,507	4,219323	3,9821113	3,893958667	-0,08815267	0,940726554	Adam39
17430503	8,466455	8,224139	8,312379	8,227068	8,249617	8,261837	8,3343243	8,246174	-0,08815033	0,940728076	Kpna6
17310944	7,613887	7,644057	7,639311	7,577409	7,520471	7,534926	7,6324183	7,544268667	-0,08814967	0,94072851	BC030476
17300047	3,137005	3,453762	3,042141	3,103815	3,205635	3,059058	3,2109693	3,122836	-0,08813333	0,940739161	---
17373844	4,667575	4,391696	4,836287	4,419657	4,620159	4,591343	4,6318527	4,543719667	-0,088133	0,940739378	4930500J02Rik
17352241	4,279181	3,885451	3,945994	4,00406	3,958135	3,884038	4,0368753	3,948744333	-0,088131	0,940740682	Fbxo15
17333023	7,366968	7,343008	7,54401	7,412299	7,324246	7,253055	7,4179953	7,329866667	-0,08812867	0,940742204	Tagap
17416811	10,22026	9,096155	9,839921	9,359275	9,742532	9,79025	9,7187787	9,630685667	-0,088093	0,940765461	Mir761
17204503	3,701234	3,420386	4,008785	3,548768	3,416859	3,900521	3,710135	3,622049333	-0,08808567	0,940770243	---
17404570	5,087086	4,931187	4,960376	4,883693	4,804296	5,026422	4,992883	4,904803667	-0,08807933	0,940774373	Slc7a14
17486230	6,34603	6,209235	5,68895	5,862743	6,151468	5,965845	6,081405	5,993352	-0,088053	0,940791545	Vmn2r34
17428416	3,07701	2,990322	3,311422	3,179586	2,913267	3,021807	3,1262513	3,03822	-0,08803133	0,940805674	---
17317417	2,92663	2,872248	2,86849	2,732264	2,667882	3,003163	2,8891227	2,801103	-0,08801967	0,940813282	---
17349960	3,151703	3,105	3,427284	3,180761	3,183612	3,055588	3,2279957	3,139987	-0,08800867	0,940820456	---
17479751	3,245778	3,162516	3,437189	3,168385	3,057679	3,355415	3,2818277	3,193826333	-0,08800133	0,940825238	---
17324400	7,443979	7,754499	7,254472	7,433339	7,407407	7,348228	7,4843167	7,396324667	-0,087992	0,940831325	Eif4a2
17473219	6,760883	6,56691	6,729793	6,542663	6,904063	6,346921	6,685862	6,597882333	-0,08797967	0,940839368	Tsen34
17494465	4,33775	4,16133	4,424679	4,218918	4,239715	4,201194	4,3079197	4,219942333	-0,08797733	0,940840889	Olfr683
17285691	12,91119	12,97069	13,05403	12,92722	12,87814	12,86664	12,978637	12,89066667	-0,08797	0,940845672	Hist1h2be // Hist1h2be
17262836	7,670749	7,593932	7,48959	7,455945	7,456832	7,577593	7,584757	7,49679	-0,087967	0,940847628	Cdc42se2
17232231	5,585844	5,377054	5,377054	5,509459	5,165447	5,401154	5,4466507	5,358686667	-0,087964	0,940849585	---
17357943	3,230191	2,93368	3,025593	3,020747	2,949843	2,954985	3,0631547	2,975191667	-0,087963	0,940850237	Olfr1496
17360486	7,634968	7,623696	7,740126	7,627235	7,548134	7,559627	7,6662633	7,578332	-0,08793133	0,940870888	Vti1a
17526102	8,755588	8,736156	8,872244	8,725522	8,650652	8,724056	8,787996	8,700076667	-0,08791933	0,940878714	Oaf
17299483	3,558265	3,497375	3,475955	3,407954	3,409311	3,450635	3,5105317	3,422633333	-0,08789833	0,94089241	Olfr738
17449288	3,579143	3,482278	3,793746	3,33699	3,723583	3,530913	3,618389	3,530495333	-0,08789367	0,940895453	Tmprss11bnl
17302096	4,797404	4,758811	4,780351	4,7748	4,679655	4,618475	4,7788553	4,690976667	-0,08787867	0,940905236	1700108F19Rik
17263151	5,452507	5,236476	5,239749	5,167717	5,233674	5,263713	5,3095773	5,221701333	-0,087876	0,940906975	Olfr332
17279858	5,130893	5,505965	5,50282	5,181814	5,11258	5,581693	5,3798927	5,292029	-0,08786367	0,940915019	Fkbp1b
17218254	10,44579	10,15134	10,43375	9,990516	10,34644	10,43035	10,343627	10,25576867	-0,087858	0,940918715	Arpc5
17248929	8,181353	8,21497	8,070415	7,99616	8,148505	8,058571	8,1555793	8,067745333	-0,087834	0,940934367	Zfp62
17515425	7,934858	8,2016	7,954667	7,87358	7,887228	8,066836	8,030375	7,942548	-0,087827	0,940938933	Zfp809
17282950	3,382125	3,322151	3,118261	3,275945	3,222264	3,06086	3,274179	3,186356333	-0,08782267	0,940941759	Gm5662
17548317	5,950534	5,867685	6,070727	5,795983	5,880708	5,948813	5,962982	5,875168	-0,087814	0,940947412	Alpl // Alpl
17202767	5,36378	4,533774	4,209667	4,136995	4,954546	4,752266	4,702407	4,614602333	-0,08780467	0,940953499	---
17291866	8,240011	8,332898	8,144796	8,200552	8,223338	8,030411	8,239235	8,151433667	-0,08780133	0,940955673	Lyrm4
17221991	3,348307	3,175475	2,961898	3,102503	3,044428	3,075376	3,1618933	3,074102333	-0,087791	0,940962413	---
17368286	6,904253	6,944724	7,218788	6,935369	6,905148	6,963909	7,0225883	6,934808667	-0,08777967	0,940969805	Snapc4
17366894	3,441271	3,49314	3,571401	3,53977	3,507665	3,195064	3,5019373	3,414166333	-0,087771	0,940975457	Mir467d // Mir467d
17320037	4,295498	4,436283	4,391947	4,391801	4,281214	4,187431	4,374576	4,286815333	-0,08776067	0,940982197	Smc1b
17497847	6,363756	6,137212	6,087399	6,061664	6,126312	6,137122	6,1961223	6,108366	-0,08775633	0,940985024	Sct
17550520	4,629338	4,641184	4,811012	4,650927	4,552937	4,614406	4,6938447	4,60609	-0,08775467	0,940986111	---
17208707	6,236337	6,029138	6,262388	5,979693	6,204785	6,080127	6,1759543	6,088201667	-0,08775267	0,940987415	---
17254681	9,205551	9,251026	9,275382	9,036259	9,152208	9,280235	9,2439863	9,156234	-0,08775233	0,940987632	Trim37
17380057	4,560723	4,291697	4,567141	4,399512	4,336522	4,420289	4,473187	4,385441	-0,087746	0,940991763	---
17332375	3,320875	3,755152	4,220496	3,790259	3,520077	3,723029	3,7655077	3,677788333	-0,08771933	0,941009157	2810404F17Rik
17210193	5,485625	5,62503	5,908525	5,544214	5,596985	5,61485	5,67306	5,585349667	-0,08771033	0,941015027	---
17281099	9,065347	8,968643	8,688776	8,658102	8,789297	9,01228	8,9075887	8,819893	-0,08769567	0,941024594	1110002B05Rik
17482124	7,09482	6,772187	6,96173	7,008142	6,479467	7,07814	6,9429123	6,855249667	-0,08766267	0,941046119	---
17486408	4,274762	4,249352	3,833711	3,988398	4,157575	3,948879	4,119275	4,031617333	-0,08765767	0,94104938	---
17204709	5,450083	5,734704	4,854647	5,624491	4,927105	5,224879	5,346478	5,258825	-0,087653	0,941052424	---
17516292	3,245775	3,190593	3,318065	3,124974	3,289198	3,077317	3,2514777	3,163829667	-0,087648	0,941055686	Olfr229
17517842	5,646514	5,573137	5,698242	5,521851	5,539999	5,593167	5,6392977	5,551672333	-0,08762533	0,941070471	E330033L03
17316820	3,666974	3,396614	3,569298	3,692568	3,304783	3,372669	3,5442953	3,456673333	-0,087622	0,941072645	Gm10373
17514122	8,149568	8,365302	7,972238	7,908231	8,163663	8,152366	8,1623693	8,074753333	-0,087616	0,941076559	2810004N23Rik
17274731	5,130513	4,666709	4,632183	4,782368	4,869628	4,514576	4,8098017	4,722190667	-0,087611	0,941079821	---
17530000	8,292162	8,431758	8,209248	8,209394	8,322419	8,138526	8,311056	8,223446333	-0,08760967	0,94108069	Pik3cb
17397738	3,421383	3,906319	3,710678	3,647928	3,488134	3,639503	3,67946	3,591855	-0,087605	0,941083735	---
17535681	5,454216	5,390424	5,011518	5,054749	5,329918	5,208771	5,285386	5,197812667	-0,08757333	0,941104391	Srpk3
17299006	5,905962	5,600407	5,368938	5,618034	5,443665	5,550909	5,6251023	5,537536	-0,08756633	0,941108957	---
17241729	3,02624	3,058159	3,077847	3,027791	2,91732	2,954491	3,054082	2,966534	-0,087548	0,941120917	Phyhipl
17286228	6,395898	5,966991	6,902904	6,402583	6,430467	6,17012	6,421931	6,33439	-0,087541	0,941125483	---
17210747	7,923009	7,725697	7,807176	7,955173	7,446019	7,792068	7,8186273	7,731086667	-0,08754067	0,941125701	---
17381911	6,617369	6,467779	6,682836	6,334493	6,756685	6,41419	6,589328	6,501789333	-0,08753867	0,941127005	Nsun6
17341440	8,341324	8,367093	8,202965	8,021043	8,509745	8,117983	8,303794	8,216257	-0,087537	0,941128093	Zfp945
17442925	10,95933	10,94189	10,80859	10,81782	10,82354	10,80589	10,90327	10,81575	-0,08752	0,941139182	Cct6a
17439557	6,725638	6,739904	6,950196	6,618359	6,674786	6,860058	6,805246	6,717734333	-0,08751167	0,941144619	Hnrnpd
17508006	6,224973	5,758565	5,959675	5,933917	5,887905	5,858879	5,981071	5,893567	-0,087504	0,94114962	Nek3
17491600	3,727684	3,928884	3,760229	3,76194	3,519332	3,873028	3,805599	3,7181	-0,087499	0,941152882	---
17516562	4,121975	3,879827	3,852726	3,877498	3,91643	3,798146	3,9515093	3,864024667	-0,08748467	0,941162232	---
17270937	8,47884	8,684824	8,683016	8,603357	8,498604	8,482273	8,61556	8,528078	-0,087482	0,941163972	Pecam1
17222256	7,219402	7,284623	7,154468	7,34378	6,863627	7,18865	7,2194977	7,132019	-0,08747867	0,941166147	Actr1b
17234008	5,624152	5,504511	5,560867	5,246825	5,507421	5,672859	5,5631767	5,475701667	-0,087475	0,941168539	Rtkn2
17372133	4,327368	3,902451	3,923836	3,956778	4,02168	3,912779	4,0512183	3,963745667	-0,08747267	0,941170061	---
17317608	3,744872	3,762457	3,746733	3,729707	3,694433	3,567513	3,751354	3,663884333	-0,08746967	0,941172018	Lrrc6
17489813	5,704157	5,882441	5,916548	5,538674	5,816362	5,885829	5,834382	5,746955	-0,087427	0,941199853	Pdcd5
17287764	9,1229	9,005013	8,920109	8,845049	9,032469	8,908267	9,0160073	8,928595	-0,08741233	0,941209421	Txndc15
17293827	4,093158	4,168016	3,684593	3,981119	3,815403	3,887013	3,9819223	3,894511667	-0,08741067	0,941210509	2410141K09Rik
17492978	9,553156	9,595416	9,476115	9,451037	9,470521	9,440915	9,5415623	9,454157667	-0,08740467	0,941214423	Fam108c
17252878	6,144689	5,874791	5,931457	5,855362	5,986439	5,846924	5,9836457	5,896241667	-0,087404	0,941214858	Mir212
17499962	6,75041	7,045702	6,893574	6,808466	6,799355	6,819673	6,896562	6,809164667	-0,08739733	0,941219207	Plat
17216642	5,57306	5,736959	5,538308	5,510628	5,604999	5,470521	5,616109	5,528716	-0,087393	0,941222034	Sctr
17330797	3,353989	3,478672	3,090665	3,324249	3,14484	3,192079	3,3077753	3,220389333	-0,087386	0,941226601	---
17235320	12,25371	12,08816	12,09215	12,06538	12,00043	12,10607	12,144673	12,05729333	-0,08738	0,941230516	Reep6
17231717	8,167526	8,15857	8,194543	8,056476	8,046036	8,155995	8,1735463	8,086169	-0,08737733	0,941232255	Fuca2
17548520	9,342815	9,585629	9,393163	9,245366	9,485295	9,328864	9,4405357	9,353175	-0,08736067	0,941243129	Eif4a3 // Eif4a3
17304162	8,813695	8,748072	8,631486	8,595739	8,54245	8,793015	8,7310843	8,643734667	-0,08734967	0,941250306	D14Ertd449e
17436551	4,386183	4,160344	4,332775	3,937291	4,518168	4,16183	4,2931007	4,205763	-0,08733767	0,941258135	4930557J02Rik
17205367	6,499242	5,687692	6,713302	6,565696	5,9798	6,09276	6,3000787	6,212752	-0,08732667	0,941265312	---
17342793	7,76944	7,916369	7,854019	7,753142	7,782336	7,742373	7,8466093	7,759283667	-0,08732567	0,941265964	Taf11
17547865	8,215718	7,279025	8,133514	7,452933	8,084154	7,829233	7,8760857	7,788773333	-0,08731233	0,941274663	BC023969
17451244	5,436011	5,315099	5,539879	5,492338	5,253586	5,283236	5,4303297	5,343053333	-0,08727633	0,941298151	1700034G24Rik
17414340	6,560131	6,329507	6,660036	6,321156	6,439601	6,527094	6,516558	6,429283667	-0,08727433	0,941299456	---
17445659	3,250798	3,277759	3,302691	3,123372	3,294313	3,151775	3,2770827	3,18982	-0,08726267	0,941307068	Gm17019
17394100	4,01126	3,859557	3,803049	3,89813	3,837109	3,676865	3,8912887	3,804034667	-0,087254	0,941312723	---
17549000	8,223302	8,284646	8,170515	8,087011	8,159921	8,16978	8,2261543	8,138904	-0,08725033	0,941315115	B230369F24Rik
17379674	6,197745	6,131116	6,417775	6,251525	6,171842	6,061581	6,2488787	6,161649333	-0,08722933	0,941328817	---
17367531	3,800187	3,563344	3,47289	3,532008	3,565165	3,477579	3,6121403	3,524917333	-0,087223	0,94133295	Mir3967
17299816	3,301708	3,355829	3,172562	3,536709	2,985073	3,046665	3,2766997	3,189482333	-0,08721733	0,941336647	---
17481317	4,315322	4,128086	4,121493	4,615745	3,926441	3,76107	4,1883003	4,101085333	-0,087215	0,94133817	Olfr653
17216777	8,124272	8,042583	8,106023	8,161911	8,065309	7,784018	8,0909593	8,003746	-0,08721333	0,941339257	---
17229970	2,948439	2,962361	3,062114	2,88403	2,998996	2,828279	2,9909713	2,903768333	-0,087203	0,941345999	Olfr1406
17318002	5,744236	5,523034	5,65167	5,512378	5,491107	5,653908	5,6396467	5,552464333	-0,08718233	0,941359484	Slurp1
17369911	5,987255	5,780236	6,301898	5,950522	5,895948	5,961373	6,0231297	5,935947667	-0,087182	0,941359702	Ak1
17439966	5,480753	5,641694	5,501677	5,35819	5,489432	5,514971	5,5413747	5,454197667	-0,087177	0,941362964	4930458A03Rik
17458928	3,547011	3,056848	3,145085	2,993522	3,294521	3,199381	3,249648	3,162474667	-0,08717333	0,941365357	Vmn1r5
17366542	7,385432	7,766961	7,517875	7,421657	7,430903	7,556191	7,556756	7,469583667	-0,08717233	0,941366009	Nudt5
17339137	4,866674	5,153975	4,922788	5,12335	5,092622	4,466071	4,9811457	4,894014333	-0,08713133	0,941392763	Gm4701

17390778	5,389781	4,793562	5,227369	5,026932	5,093789	5,028625	5,136904	5,049782	-0,087122	0,941398853	Duoxa1
17343057	5,501406	5,679568	5,413093	5,429171	5,458471	5,445068	5,5313557	5,444236667	-0,087119	0,94140081	Pim1
17486351	6,031265	5,686768	5,218621	5,558125	5,757148	5,360037	5,6455513	5,558436667	-0,08711467	0,941403638	Vmn2r29
17486367	6,031265	5,686768	5,218621	5,558125	5,757148	5,360037	5,6455513	5,558436667	-0,08711467	0,941403638	Vmn2r29
17486912	5,517328	5,222131	5,650301	5,100899	5,692177	5,335395	5,4632533	5,376157	-0,08709633	0,941415601	Gm19512
17494930	9,229343	9,285599	9,278013	9,232691	9,212624	9,086426	9,2643183	9,177247	-0,08707133	0,941431915	Tmem9b
17331042	8,599018	8,592711	8,596061	8,418984	8,623075	8,484526	8,59593	8,508861667	-0,08706833	0,941433872	---
17247485	3,731512	3,88453	4,072328	3,798143	3,825255	3,80378	3,8961233	3,809059333	-0,087064	0,9414367	---
17289986	5,260267	5,385143	5,398855	5,23094	5,28384	5,268302	5,3480883	5,261027333	-0,087061	0,941438658	Cdc20b
17240330	9,324648	9,273069	9,277628	8,976768	9,277628	9,35977	9,2917817	9,204722	-0,08705967	0,941439528	Slc16a10
17542997	3,553077	3,721472	3,483235	3,521951	3,487338	3,487338	3,585928	3,498875667	-0,08705233	0,941444313	Gm7173
17337314	7,927989	8,059083	8,056937	7,87414	7,856578	8,052166	8,0146697	7,927628	-0,08704167	0,941451274	Ppp1r10
17476599	5,437515	5,396836	5,070752	5,404181	4,996323	5,243521	5,301701	5,214675	-0,087026	0,941461498	Gm10640
17458950	3,755162	3,875299	3,582575	3,663137	3,520896	3,767975	3,7376787	3,650669333	-0,08700933	0,941472374	Vmn1r13
17217171	8,08113	8,146379	8,501516	8,212376	7,9714	8,284244	8,2430083	8,156006667	-0,08700167	0,941477377	---
17224256	8,587286	8,778517	8,775299	8,523166	8,776239	8,580741	8,7137007	8,626715333	-0,08698533	0,941488036	Aamp
17499293	7,10973	6,959299	7,007482	6,801312	7,097185	6,917122	7,0255037	6,938539667	-0,086964	0,941501958	Tmco3
17207075	4,249447	4,71187	4,409621	4,975274	3,729604	4,405168	4,4569793	4,370015333	-0,086964	0,941501958	---
17238974	9,027581	9,082659	9,193422	8,986869	8,960024	9,095894	9,1012207	9,014262333	-0,08695833	0,941505656	Fbxo5
17231423	8,047421	8,073296	7,860654	7,927179	7,837764	7,95566	7,9937903	7,906867667	-0,08692267	0,941528932	Akap12
17340393	4,78343	4,943914	4,825368	4,69988	4,763579	4,828568	4,850904	4,764009	-0,086895	0,941546988	Nrxn1 // Nrxn1
17493044	3,182898	3,475314	3,130545	3,249649	3,250599	3,027882	3,262919	3,176043333	-0,08687567	0,941559606	Vmn2r66
17414782	5,368666	5,42983	5,31904	5,293602	5,208751	5,354558	5,372512	5,285637	-0,086875	0,941560041	---
17539600	5,422493	5,492788	5,481903	5,276218	5,54234	5,318001	5,465728	5,378853	-0,086875	0,941560041	Gemin8
17387764	3,119955	3,084826	3,027832	3,03257	3,033041	2,906405	3,0775377	2,990672	-0,08686567	0,941566132	Olfr1134
17240444	4,295809	4,180829	4,388678	4,156827	4,258133	4,189796	4,2884387	4,201585333	-0,08685333	0,941574182	Ccdc162
17466505	8,517381	8,458238	8,259735	8,136589	8,522879	8,315429	8,4117847	8,324965667	-0,086819	0,941596589	Rbpj // Rbpj
17285851	11,48253	11,37694	11,16046	10,89139	11,31469	11,5534	11,339977	11,25316	-0,08681667	0,941598112	Hist1h2bc
17317225	8,569938	8,455645	8,356899	8,375341	8,344395	8,402394	8,4608273	8,374043333	-0,086784	0,941619433	Zhx1
17433665	5,761243	5,794156	5,686172	5,670448	5,633821	5,67695	5,7471903	5,660406333	-0,086784	0,941619433	Ttc34
17251933	9,784473	9,817569	9,780907	9,775032	9,685087	9,662498	9,7943163	9,707539	-0,08677733	0,941623784	Asgr1
17379547	5,784309	6,045524	6,122314	5,906838	5,974052	5,810927	5,984049	5,897272333	-0,08677667	0,941624219	Zswim3
17244697	6,147946	5,909094	6,193788	5,936552	6,195472	5,858566	6,0836093	5,996863333	-0,086746	0,941644235	---
17411892	7,240789	7,211652	7,153803	7,022347	7,068617	7,255084	7,2020813	7,115349333	-0,086732	0,941653373	Rbm12b
17210213	4,258008	5,407082	5,59452	5,503772	4,628322	4,867332	5,0865367	4,999808667	-0,086728	0,941655984	---
17547803	8,411249	8,638372	8,653276	8,476704	8,423162	8,542857	8,5676323	8,480907667	-0,08672467	0,94165816	Gm6340
17366437	7,310913	7,114953	6,834665	6,8231	7,086037	7,091225	7,0868437	7,000120667	-0,086723	0,941659247	Fam107b
17492858	3,363412	3,459167	3,736867	3,501678	3,309044	3,488569	3,5198153	3,433097	-0,08671833	0,941662293	---
17405010	5,207478	5,057342	5,323659	5,30822	5,029097	4,991045	5,1961597	5,109454	-0,08670567	0,941670561	---
17350187	3,063152	2,881563	3,019168	2,855231	2,914962	2,933591	2,987961	2,901261333	-0,08669967	0,941674477	Spg11
17374366	4,098436	4,145878	4,37284	4,214161	4,008459	4,134456	4,205718	4,119025333	-0,08669267	0,941679046	A530058N18Rik
17394102	5,176698	5,528611	5,266037	5,268537	5,329242	5,113496	5,323782	5,237091667	-0,08669033	0,941680569	Kcns1
17467164	3,093051	3,113788	3,089339	2,944771	3,050162	3,041204	3,098726	3,012045667	-0,08668033	0,941687097	---
17491710	3,087344	2,974279	3,087029	2,987113	2,985296	2,91622	3,0495507	2,962876333	-0,08667433	0,941691013	---
17407461	6,385928	5,499974	5,731444	5,687557	6,048852	5,620917	5,8724487	5,785775333	-0,08667333	0,941691666	Lce1h
17258580	4,41245	4,481975	4,617756	4,362251	4,361633	4,528342	4,5040603	4,417408667	-0,08665167	0,941705808	---
17236990	3,924236	3,919178	3,787163	3,722429	3,877268	3,770949	3,876859	3,790215333	-0,08664367	0,94171103	1700017N19Rik
17412653	7,663789	7,759355	7,692773	7,732233	7,634811	7,488972	7,7053057	7,618672	-0,08663367	0,941717558	Rars2
17494236	4,739242	4,729131	4,77345	4,528957	4,750579	4,702393	4,7472743	4,660643	-0,08663133	0,941719081	Hbb-bh2
17540584	5,199408	4,843426	4,802638	4,724819	4,870256	4,990578	4,9484907	4,861884333	-0,08660633	0,9417354	Gm5124 // Gm5124
17413523	5,76422	5,966267	5,896438	5,908493	5,665547	5,793075	5,8756417	5,789038333	-0,08660333	0,941737358	Gm12503
17453129	8,167527	7,997303	8,412309	8,388111	8,167373	7,761856	8,1923797	8,10578	-0,08659967	0,941739752	---
17294677	4,965293	5,191101	5,459914	4,796728	5,060899	5,498892	5,205436	5,118839667	-0,08659633	0,941741927	---
17385717	8,776536	8,340192	8,19347	8,320458	8,272054	8,457927	8,4367327	8,350146333	-0,08658633	0,941748455	---
17203945	4,810431	5,293884	4,961366	4,784484	5,209085	4,812369	5,0218937	4,935312667	-0,086581	0,941751937	---
17281485	7,423943	7,643959	7,694253	7,374604	7,558867	7,569012	7,587385	7,500827667	-0,08655733	0,941767386	Rps29
17462128	7,027441	7,348238	7,323298	7,096872	7,136783	7,205662	7,2329923	7,146439	-0,08655333	0,941769997	Alox5 // Alox5
17383813	5,201077	5,516538	5,498617	5,297885	5,493218	5,165476	5,4054107	5,318859667	-0,086551	0,94177152	Aif1l
17498951	4,9477	4,912569	4,917358	4,896614	4,803404	4,818023	4,9258757	4,839347	-0,08652867	0,941786099	Irs2
17354361	7,004874	7,00619	6,922528	6,843851	6,924072	6,906137	6,977864	6,891353333	-0,08651067	0,941797849	---
17296786	7,085874	7,123425	6,743679	6,785636	6,967813	6,940013	6,984326	6,897820667	-0,08650533	0,941801331	2610042L04Rik
17216061	3,537639	3,399082	4,238199	3,896949	3,563502	3,454994	3,7249733	3,638481667	-0,08649167	0,941810253	---
17285964	7,450111	7,594729	7,523953	7,451712	7,291098	7,566516	7,522931	7,436442	-0,086489	0,941811994	Fam65b
17307146	4,272042	4,206436	4,488412	4,379318	4,090851	4,237298	4,3222967	4,235822333	-0,08647433	0,941821568	---
17382410	5,171675	5,248335	5,17652	4,825241	5,18748	5,324389	5,1988433	5,11237	-0,08647333	0,941822221	4933433C11Rik
17397893	3,488162	3,36399	3,406095	3,308321	3,235188	3,455415	3,4194157	3,332974667	-0,086441	0,941843329	Gm8298
17381275	3,055543	2,97684	3,018914	3,074842	2,856051	2,861106	3,017099	2,930666333	-0,08643267	0,94184877	---
17210725	7,494697	6,845763	7,493362	7,300933	7,62465	6,64897	7,2779407	7,191517667	-0,086423	0,94185508	---
17347387	8,030787	8,362677	7,959491	7,944066	8,117324	8,0323	8,1176517	8,03123	-0,08642167	0,941855951	Cebpz
17377523	9,97111	10,18246	10,02511	9,736348	9,977357	10,20576	10,05956	9,973155	-0,086405	0,941866832	---
17536463	6,915986	7,091186	6,910288	6,956001	6,780337	6,921978	6,9724867	6,886105333	-0,08638133	0,941882283	Stard8
17525649	7,085925	6,894588	6,843834	6,785509	6,989347	6,79036	6,941449	6,855072	-0,086377	0,941885112	Nrgn
17482764	4,415207	4,744172	4,761527	4,844288	4,216132	4,601518	4,640302	4,553979333	-0,08632267	0,941920585	---
17392787	6,294467	6,09553	6,136592	6,296725	5,94434	6,026586	6,1755297	6,089217	-0,08631267	0,941927114	4921509C19Rik
17548367	8,087757	8,098747	8,19056	8,005335	8,027435	8,085375	8,125688	8,039381667	-0,08630633	0,941931249	---
17328895	6,447042	6,467826	6,156267	6,374356	6,422386	6,015603	6,357045	6,270781667	-0,08626333	0,941959324	---
17359962	9,068613	9,238855	9,130627	9,322352	8,471086	9,385909	9,1460317	9,059782333	-0,08624933	0,941968464	---
17542961	5,160831	4,840128	4,950833	5,427767	4,693856	4,571436	4,9839307	4,897686333	-0,08624433	0,941971729	---
17380749	4,987705	4,849706	5,486562	5,06969	5,073457	4,92211	5,107991	5,021752333	-0,08623867	0,941975429	Gm14318
17408536	5,444554	5,581944	5,558722	5,564442	5,337233	5,424866	5,5284067	5,442180333	-0,08622633	0,941983482	Mab21l3
17447028	4,438812	4,100512	4,577543	4,305539	4,247276	4,305382	4,372289	4,286065667	-0,08622333	0,941985441	---
17473685	5,551908	5,149219	5,141839	5,467115	5,026613	5,090575	5,2809887	5,194767667	-0,086221	0,941986964	---
17477254	4,956817	4,794373	4,931768	4,872595	4,836294	4,715508	4,8943193	4,808132333	-0,086187	0,942009164	Klk6
17311512	9,998686	9,855988	9,827047	9,657059	9,963976	9,802162	9,893907	9,807732333	-0,08617467	0,942017217	Mal2
17507706	3,647875	3,482388	3,314247	3,278524	3,569272	3,338194	3,4815033	3,39533	-0,08617333	0,942018088	---
17501260	9,868101	9,806456	9,942837	9,656466	9,830008	9,87241	9,8724647	9,786294667	-0,08617	0,942020264	Fbxo8
17465115	4,645021	4,873621	4,823637	4,712622	4,68638	4,68482	4,7807597	4,694607333	-0,08615233	0,9420318	Cadps2
17457320	3,553077	3,239601	3,153102	3,213012	3,190909	3,283406	3,31526	3,229109	-0,086151	0,942032671	---
17351681	9,267681	9,075523	9,097284	9,020926	9,143062	9,018049	9,1468293	9,060679	-0,08615033	0,942033106	Mex3c
17434807	3,233192	3,308717	2,969729	3,024098	3,179513	3,049577	3,170546	3,084396	-0,08615	0,942033324	LOC100862549
17550476	3,988059	3,728849	4,232323	3,942952	3,726447	4,021403	3,983077	3,896934	-0,086143	0,942037894	---
17471788	3,976176	4,108689	3,71079	3,827381	3,809725	3,900205	3,931885	3,845770333	-0,08611467	0,942056395	LOC100048471
17332595	6,458966	6,340719	6,386369	6,506597	6,258944	6,162173	6,3953513	6,309238	-0,08611333	0,942057266	Prdm15
17442071	8,455764	8,309411	8,233325	8,274077	8,210043	8,256064	8,3328333	8,246728	-0,08610533	0,94206249	Vps29
17220148	8,39228	8,65682	8,498275	8,294168	8,475535	8,519393	8,5157917	8,429698667	-0,086093	0,942070544	Lin9
17422389	5,317008	5,273924	5,758639	5,334106	5,304989	5,452235	5,449857	5,363776667	-0,08608033	0,942078815	---
17519088	10,70709	10,84361	10,8536	10,65088	10,68863	10,80663	10,801433	10,71538	-0,08605333	0,942096446	Adam10
17261645	6,957039	6,690925	6,798371	6,716228	6,695951	6,776031	6,815445	6,729403333	-0,08604167	0,942104064	Foxi1
17440707	5,380705	5,306924	5,450717	5,159684	5,469264	5,251285	5,3794487	5,293411	-0,08603767	0,942106677	Gm6588
17486644	3,998402	3,889699	3,980121	4,036046	3,751435	3,822631	3,956074	3,870037333	-0,08603667	0,94210733	Vmn1r85
17238594	9,148869	9,463717	9,320444	9,411884	9,206304	9,056759	9,31101	9,224982333	-0,08602767	0,942113207	Cd63
17215370	9,406202	9,804123	9,79895	9,763055	9,599891	9,388267	9,6697583	9,583737667	-0,08602067	0,942117778	---
17481236	4,175945	3,730487	3,608936	3,755777	3,948481	3,553077	3,838456	3,752445	-0,086011	0,942124091	Olfr65
17449093	4,104365	4,484829	4,380042	4,338954	4,061277	4,311045	4,3230787	4,237092	-0,08598667	0,942139981	---
17529690	4,177216	3,832893	3,940457	4,051618	3,79011	3,850908	3,983522	3,897545333	-0,08597667	0,942146512	---
17408646	3,112156	2,949163	3,233192	2,992252	2,927348	3,117085	3,0981703	3,012228333	-0,085942	0,942169151	---
17359020	10,59146	10,60682	10,59276	10,46914	10,50824	10,55588	10,597013	10,51108667	-0,08592667	0,942179164	Kif11
17240687	5,057686	4,773965	5,293326	5,030096	4,882207	4,954932	5,041659	4,955745	-0,085914	0,942187437	---
17236245	5,828192	5,435662	5,486743	5,649606	5,484528	5,358727	5,5835323	5,497620333	-0,085912	0,942188743	---
17478001	6,360874	6,301478	6,619579	6,054826	6,590153	6,379315	6,4273103	6,341431333	-0,085879	0,942210295	Fam83e
17293503	5,184584	5,72042	5,239799	5,1765	5,293873	5,416914	5,381601	5,295762333	-0,08583867	0,942236636	Zfp934 // Zfp934
17204691	6,374011	6,202789	6,151155	6,364935	6,343218	5,762329	6,2426517	6,156827333	-0,08582433	0,942245997	---
17465614	5,079525	4,989458	5,333547	5,151795	4,89406	5,099277	5,1341767	5,048377333	-0,08579933	0,942262326	Gm13845
17426511	4,353988	4,827245	4,32516	4,513983	4,426075	4,308971	4,502131	4,416343	-0,085788	0,942269728	---
17204177	3,528217	3,72558	3,658941	3,58569	3,733197	3,336511	3,6375793	3,551799333	-0,08578	0,942274953	---
17204727	5,41377	5,818698	5,877011	5,433628	4,918186	6,500342	5,7031597	5,617385333	-0,08577433	0,942278654	---
17326162	4,12305	4,131253	4,335732	4,004928	3,998151	4,329652	4,1966783	4,110910333	-0,085768	0,94228279	Gm16619
17411727	4,991909	4,808767	5,113484	4,860437	4,850935	4,945486	4,9713867	4,885619333	-0,08576733	0,942283226	Gdf6
17281993	4,257019	4,396946	4,152314	3,919523	4,396946	4,232567	4,2687597	4,183012	-0,08574767	0,942296071	Tex21
17414327	5,833888	6,015633	5,802649	5,907173	5,875113	5,612768	5,8840567	5,798351333	-0,08570533	0,942323721	Actl7a
17548767	4,743092	4,949708	4,770395	4,618274	4,770658	4,817151	4,821065	4,735361	-0,085704	0,942324592	BC043934
17483975	4,80871	4,774876	4,555326	4,532	4,77844	4,571368	4,7129707	4,627269333	-0,08570133	0,942326334	5430419D17Rik
17247369	3,320841	2,990182	2,934747	3,129336	3,008003	2,85135	3,0819233	2,996229667	-0,08569367	0,942331342	---
17304193	8,968719	8,917994	8,796401	8,79282	8,686586	8,946637	8,8943713	8,808681	-0,08569033	0,942333519	D14Ertd449e
17257065	9,879845	9,68384	9,531462	9,527426	9,791084	9,519566	9,6983823	9,612692	-0,08569033	0,942333519	---
17443062	3,857505	3,755979	4,293465	4,045455	3,975281	3,629208	3,968983	3,883314667	-0,08566833	0,942347889	4930563F08Rik
17307516	7,327656	6,877171	7,558885	7,042157	7,378188	7,086372	7,2545707	7,168905667	-0,085665	0,942350066	Mir16-1
17287650	6,20888	6,220541	6,36211	6,111492	6,253313	6,169733	6,2638437	6,178179333	-0,08566433	0,942350502	Rgs14
17495223	6,290265	6,26714	6,361709	6,073486	6,346175	6,24247	6,3063713	6,220710333	-0,085661	0,942352679	Btbd10
17520962	5,339794	4,923858	4,822964	5,033988	4,622621	5,173049	5,028872	4,943219333	-0,08565267	0,942358122	5830418P13Rik
17357790	5,922469	5,644462	5,79067	5,937273	5,547048	5,616325	5,785867	5,700215333	-0,08565167	0,942358775	Olfr235
17281946	9,105139	9,035742	8,858788	8,936012	8,833164	8,973602	8,9998897	8,914259333	-0,08563033	0,94237271	Ppp2r5e
17231086	5,639482	5,165453	5,207859	5,630329	4,917735	5,207859	5,337598	5,251974333	-0,08562367	0,942377065	---
17266038	6,787817	7,116289	6,892266	6,918	6,752626	6,868894	6,932124	6,846506667	-0,08561733	0,942381202	Fam101b

17222642	6,996484	6,877293	6,776455	6,933881	6,747004	6,712543	6,8834107	6,797809333	-0,08560133	0,942391653	Fhl2	
17547559	4,085605	4,017225	3,954884	3,970225	3,970425	3,86028	4,019238	3,933643333	-0,08559467	0,942396008	Dusp6 // Dusp6	
17377651	5,649224	5,574079	5,653252	5,412183	5,594321	5,613405	5,6255183	5,539969667	-0,08554867	0,942426057	Slc52a3	
17404021	8,186451	8,267695	8,406792	8,149852	8,230358	8,2241	8,2869793	8,201436667	-0,08554267	0,942429976	Mrps28	
17549954	3,188221	3,290338	3,17336	2,938883	3,301013	3,155439	3,2173063	3,131778333	-0,085528	0,942439557	---	
17267302	2,961459	3,210392	3,052899	2,831805	3,16768	2,968714	3,0749167	2,989399667	-0,085517	0,942446743	---	
17200483	5,09325	5,607843	5,685481	5,427453	5,209538	5,493066	5,4621913	5,376685667	-0,08550567	0,942454146	---	
17405435	6,841803	6,768522	6,764117	6,560544	6,613024	6,944365	6,7914807	6,705977667	-0,085503	0,942455889	Siah2	
17443004	8,732309	8,683964	8,705917	8,674159	8,674351	8,517185	8,7073967	8,621898333	-0,08549833	0,942458937	Rabgef1	
17463499	2,862059	2,799665	3,024708	2,79553	2,801133	2,833296	2,8954773	2,809986333	-0,085491	0,942463728	---	
17263123	6,494098	6,624748	6,750473	6,430468	6,544087	6,63835	6,6231063	6,537635	-0,08547133	0,942476575	Zfp672	
17480528	5,752559	5,417114	5,441728	5,366144	5,408622	5,580291	5,5371337	5,451685667	-0,085448	0,942491819	LOC100862647	
17404701	4,832881	4,456051	4,80504	4,879295	4,473442	4,484904	4,6979907	4,612547	-0,08544367	0,942494649	---	
17463727	6,28046	6,809268	6,393869	6,62266	6,59063	6,014016	6,4945323	6,409102	-0,08543033	0,94250336	Apold1	
17543336	7,848108	8,060781	8,042707	7,898415	7,892757	7,904182	7,9838653	7,898451333	-0,085414	0,942514031	Las1l	
17215807	3,614822	3,771194	3,957902	3,605841	3,758972	3,722887	3,781306	3,6959	-0,085406	0,942519257	Olfr1413	
17289941	7,528255	7,573627	7,370909	7,42219	7,388371	7,406108	7,4909303	7,405556333	-0,085374	0,942540163	Dhx29	
17356754	6,748504	6,628283	6,877594	6,685979	6,601192	6,711093	6,7514603	6,666088	-0,08537233	0,942541252	Cdc42bpg	
17550805	9,905251	9,839901	9,918681	9,716571	9,717821	9,973328	9,8879443	9,802573333	-0,085371	0,942542123	---	
17276007	10,21113	10,37663	10,28806	10,09658	10,27196	10,25117	10,29194	10,20657	-0,08537	0,942542776	Tmx1	
17318536	7,056087	6,988235	7,049853	7,069506	6,921518	6,847072	7,0313917	6,946032	-0,08535967	0,942549527	Fbxl6	
17250790	4,746903	4,830688	4,859838	4,711305	4,711305	4,758783	4,8124763	4,727131	-0,08534533	0,942558892	Fbxw10	
17525742	4,48955	4,12329	4,14667	4,349997	3,948685	4,20484	4,25317	4,167840667	-0,08532933	0,942569345	Olfr959	
17392087	8,244425	8,265863	8,143975	8,101171	8,172537	8,124577	8,2180877	8,132761667	-0,085326	0,942571523	Tmx4	
17341039	5,394073	4,944759	5,338496	5,154107	5,104756	5,162498	5,225776	5,140453667	-0,08532233	0,942573918	---	
17427222	4,682199	4,290818	4,340163	4,664865	4,051581	4,340793	4,4377267	4,352413	-0,08531367	0,942579581	---	
17244591	7,977407	8,142351	7,9249	7,879019	7,849473	8,060272	8,014886	7,929588	-0,085298	0,942589816	4930430F08Rik	
17439255	5,209002	4,816697	4,619663	4,827296	4,604382	4,9578	4,8817873	4,796492667	-0,08529467	0,942591994	LOC100862180 // LOC100862166 // Gm7	
17515315	8,785203	8,869236	8,940567	8,930444	8,532043	8,876647	8,865002	8,779711333	-0,08529067	0,942594608	Ldlr	
17524141	7,62744	7,764447	7,614225	7,525732	7,664047	7,560477	7,668704	7,583418667	-0,08528533	0,942598092	Med17	
17391212	8,959489	8,949505	9,059135	8,895867	8,94128	8,875214	8,9893763	8,904120333	-0,085256	0,942617258	Ncaph	
17505772	3,153102	3,07872	3,102498	3,068867	3,068739	2,940947	3,11144	3,026184333	-0,08525567	0,942617476	---	
17302588	7,135448	6,78054	6,98872	7,068493	6,972563	6,607886	6,968236	6,882980667	-0,08525533	0,942617693	---	
17311310	3,802226	3,650618	4,112492	3,857307	3,798331	3,653945	3,855112	3,769861	-0,085251	0,942620525	---	
17262250	5,930559	8,315456	5,516826	6,633116	6,782755	6,091234	6,5876137	6,502368333	-0,08524533	0,942624227	Tgtp2 // Tgtp1	
17460433	7,481685	7,35954	7,250481	7,265748	7,351293	7,218971	7,363902	7,278670667	-0,08523133	0,942633374	C87436	
17276614	7,336365	7,74188	7,640826	7,355276	7,447263	7,660839	7,5730237	7,487792667	-0,085231	0,942633592	Churc1	
17294244	4,834218	4,940848	4,944851	4,680155	5,127432	4,656713	4,906639	4,821433333	-0,08520567	0,942650145	Mrpl36 // Mrpl36	
17276666	4,301569	4,307364	4,380749	4,258841	4,277469	4,197767	4,329894	4,244692333	-0,08520167	0,942652758	Gm6657	
17267443	4,948282	4,757215	4,800656	4,947095	4,61834	4,685141	4,8353843	4,750192	-0,08519233	0,942658857	---	
17365861	7,573669	7,314139	7,215199	7,47274	6,76606	7,608724	7,367669	7,282508	-0,085161	0,94267933	---	
17509809	5,853173	5,709374	5,987273	5,893537	5,666254	5,734678	5,84994	5,764823	-0,085117	0,942708081	Gm10033	
17402895	10,5318	10,55923	10,50334	10,41599	10,42334	10,4997	10,531457	10,44634333	-0,08511333	0,942710477	Ube2d3	
17309907	9,843623	9,934519	9,627348	9,575624	9,851882	9,722712	9,80183	9,716739333	-0,08509067	0,942725288	Prkaa1	
17303625	7,666457	7,471758	7,208019	7,36494	7,441133	7,284923	7,4487447	7,363665333	-0,08507933	0,942732694	Nr1d2	
17239347	3,033989	3,014581	2,888603	2,987674	2,834425	2,859855	2,9790577	2,893984667	-0,085073	0,942736832	---	
17494286	3,810167	4,032043	3,750431	3,773723	3,744872	3,818829	3,8642137	3,779141333	-0,08507233	0,942737268	Olfr645	
17488470	7,212754	7,268058	7,182104	7,214742	7,046244	7,146756	7,220972	7,135914	-0,085058	0,942746634	Samd4b	
17395414	4,415196	4,18977	4,107004	4,244976	4,156159	4,055673	4,2373233	4,152269333	-0,085054	0,942749248	Sycp2	
17500375	4,68825	4,723074	4,766299	4,565648	4,648242	4,708571	4,7258743	4,640820333	-0,085054	0,942749248	4933433F19Rik // 4933433F19Rik	
17538806	7,902004	7,616096	7,929881	7,14318	8,113856	7,935784	7,8159937	7,73094	-0,08505367	0,942749466	---	
17444940	8,905382	8,891567	8,876352	8,849547	8,800348	8,768262	8,8911003	8,806052333	-0,085048	0,942753169	Uspl1	
17200147	3,325639	3,42495	3,898665	3,431344	3,28206	3,680709	3,5497513	3,464704333	-0,085047	0,942753822	---	
17398719	8,575141	9,05104	8,822369	8,634258	8,615309	8,943851	8,8161833	8,731139333	-0,085044	0,942755783	---	
17357504	7,660376	7,592669	7,641438	7,691119	7,57373	7,374544	7,6314943	7,546464333	-0,08503	0,942764931	---	
17448670	4,769365	4,802553	4,884092	4,885634	4,654206	4,661083	4,81867	4,733641	-0,085029	0,942765585	Zar1	
17268842	7,418762	7,817264	7,833663	7,809244	7,716484	7,288883	7,6898963	7,604870333	-0,085026	0,942767545	---	
17224121	4,059286	3,916008	3,916008	3,971658	3,876583	3,788006	3,9637673	3,878749	-0,08501833	0,942772555	4933417E11Rik	
17467823	10,53911	10,57697	10,42769	10,36324	10,42585	10,49967	10,51459	10,42958667	-0,08500333	0,942782358	Kcmf1	
17403866	6,759565	6,760975	6,8394	6,761963	6,733568	6,609412	6,7866467	6,701647667	-0,084999	0,942785189	Ptger3	
17538314	3,510128	3,315982	3,635096	3,340861	3,400437	3,464918	3,4870687	3,402072	-0,08499667	0,942786714	Rgag1	
17284700	2,985069	2,929984	3,116852	2,996604	2,940417	2,840017	3,010635	2,925679333	-0,08495567	0,942813508	---	
17445786	10,05312	10,26566	10,1219	9,975639	10,11945	10,09073	10,146893	10,06193967	-0,08495367	0,942814815	Ptpn12	
17482739	9,214533	9,293767	9,168773	9,111685	9,071662	9,238878	9,225691	9,140741667	-0,08494933	0,942817647	Plk1	
17219865	3,185924	3,554841	3,419363	3,082146	3,342674	3,480487	3,3867093	3,301769	-0,08494033	0,942823528	---	
17500752	5,524951	5,20752	5,242908	5,021388	5,320391	5,378853	5,3251263	5,240210667	-0,08491567	0,942839648	---	
17520771	3,180869	3,10323	3,080352	2,941581	3,127964	3,040171	3,1214837	3,036572	-0,08491167	0,942842262	4930519F24Rik	
17322700	5,872862	5,564992	6,013667	5,840674	5,677589	5,678701	5,8171737	5,732321333	-0,08485233	0,942881039	Rbfox1	
17440113	6,170295	6,13444	6,483476	6,28638	6,269161	5,978114	6,262737	6,177885	-0,084852	0,942881257	Ube2d2b	
17502665	5,495124	5,227757	5,631623	5,318201	5,288161	5,493677	5,4515013	5,366679667	-0,08482167	0,942901082	4933431K23Rik	
17537044	4,925089	3,926842	3,715472	4,557579	3,621908	4,133458	4,1891343	4,104315	-0,08481933	0,942902607	---	
17211194	4,022895	3,775774	3,909142	3,741995	3,893537	3,817864	3,9026037	3,817798667	-0,084805	0,942911975	4932411L15 // 4932411L15	
17363624	7,124063	7,156792	6,741587	6,918517	6,90667	6,942859	7,0074807	6,922682	-0,08479867	0,942916114	Fam122a	
17206351	4,448761	5,371051	4,575513	5,312804	4,112731	4,715395	4,7984417	4,713643333	-0,08479833	0,942916332	---	
17442765	5,795061	5,810833	5,907448	5,713475	5,80442	5,74107	5,8377807	5,752988333	-0,08479233	0,942920253	Tmem132c	
17450196	4,209645	4,600803	4,801888	4,470026	4,578966	4,308971	4,5374453	4,452654333	-0,084791	0,942921125	Agpat9	
17248019	8,550607	8,636099	8,663214	8,557866	8,591155	8,446552	8,61664	8,531857667	-0,08478233	0,942926789	Ccdc88a	
17541818	4,858229	4,725635	5,064777	4,638486	4,872993	4,882842	4,8828803	4,798107	-0,08477333	0,942932672	---	
17319529	6,641934	6,651661	6,508621	6,54231	6,441374	6,564218	6,6007387	6,515967333	-0,08477133	0,942933979	Tob2	
17205067	4,34176	4,109921	3,831933	4,944589	3,490901	3,593822	4,094538	4,009770667	-0,08476733	0,942936593	---	
17209257	4,34176	4,109921	3,831933	4,944589	3,490901	3,593822	4,094538	4,009770667	-0,08476733	0,942936593	---	
17232589	2,944557	2,769295	2,870885	2,85101	2,774224	2,705319	2,861579	2,776851	-0,084728	0,942962302	---	
17214142	8,347738	8,640297	8,152283	8,433468	8,152872	8,299812	8,380106	8,295384	-0,084722	0,942966223	Cxcr2	
17464801	4,000943	3,831676	4,001679	3,917203	4,048599	3,614377	3,944766	3,860059667	-0,08470633	0,942976463	---	
17301530	4,498844	4,173509	4,342837	4,310432	4,356002	4,094644	4,3383967	4,253692667	-0,084704	0,942977988	---	
17544835	5,48604	4,770916	5,005534	4,54773	5,384212	5,076503	5,0874967	5,002815	-0,08468167	0,942992586	---	
17205017	8,172155	8,216653	8,46036	8,027746	8,272419	8,29497	8,283056	8,198378333	-0,08467767	0,942995201	---	
17451813	5,39397	5,103727	5,467115	5,226704	5,355521	5,128555	5,321604	5,236926667	-0,08467733	0,942995418	4930562A09Rik	
17494438	4,25752	4,040312	3,973313	4,023386	4,142458	3,85128	4,0903817	4,005708	-0,08467367	0,942997815	Dub1a	
17281278	4,65146	4,419752	4,379004	4,47586	4,449839	4,270502	4,4834053	4,398733667	-0,08467167	0,942999122	---	
17486469	6,275763	6,019008	6,501141	6,582283	6,062167	5,897449	6,265304	6,180633	-0,084671	0,942999558	Zscan4a	
17550623	10,26789	10,22406	10,1748	10,15125	10,20228	10,05926	10,22225	10,13759667	-0,08465333	0,943011106	---	
17205273	8,644041	8,135334	8,800256	8,603648	8,711069	8,011017	8,5265437	8,441911333	-0,08463233	0,943024832	---	
17209463	8,644041	8,135334	8,800256	8,603648	8,711069	8,011017	8,5265437	8,441911333	-0,08463233	0,943024832	---	
17461793	9,469255	9,682886	9,539884	9,459905	9,538216	9,440021	9,5640083	9,479380667	-0,08462767	0,943027883	6720456B07Rik	
17205013	9,75792	9,912542	9,72939	9,650678	9,699892	9,795473	9,7999507	9,715347667	-0,084603	0,943044007	---	
17391456	10,10725	10,32214	10,4744	10,01939	10,51867	10,11193	10,301263	10,21666333	-0,0846	0,943045968	---	
17351509	3,38865	3,717051	3,681431	3,548063	3,47318	3,512097	3,5957107	3,511113333	-0,08459733	0,943047711	---	
17241318	8,94217	9,045884	9,197981	9,108373	8,89535	8,928566	9,0620117	8,977429667	-0,084582	0,943057734	Hk1	
17443261	6,479959	6,615591	6,499668	6,352901	6,441913	6,546825	6,5317393	6,447213	-0,08452633	0,943094122	Nsun5	
17357306	7,363177	7,473917	7,320445	7,347371	7,319791	7,236817	7,3858463	7,301326333	-0,08452	0,943098263	B3gat3	
17457987	2,897299	3,092202	3,280302	2,92911	3,009244	3,077904	3,0899343	3,005419333	-0,084515	0,943101531	Olfr444	
17430052	7,521422	7,582072	7,256251	7,455507	7,186743	7,463953	7,4532483	7,368734333	-0,084514	0,943102185	Eif2c3	
17372274	4,162484	4,272034	4,26458	4,019479	4,216228	4,209888	4,2330327	4,148531667	-0,084501	0,943110683	4930440I19Rik	
17232199	5,433419	4,861623	5,14862	5,079353	5,058362	5,052457	5,1478873	5,063390667	-0,08449667	0,943113516	---	
17321307	5,306748	5,653771	5,809175	5,485802	5,66948	5,360939	5,589898	5,505407	-0,084491	0,94311722	Rnd1	
17205089	3,342718	4,078984	3,439324	3,651653	3,133793	3,822144	3,620342	3,535863333	-0,08447867	0,943125283	---	
17209279	3,342718	4,078984	3,439324	3,651653	3,133793	3,822144	3,620342	3,535863333	-0,08447867	0,943125283	---	
17510311	6,429566	6,590064	6,614863	6,450669	6,430414	6,499978	6,544831	6,460353667	-0,08447733	0,943126154	Abhd8	
17450400	4,3041	4,114082	4,435568	4,283301	4,188147	4,128872	4,2845833	4,200106667	-0,08447667	0,94312659	---	
17200207	5,70495	5,997258	4,790248	5,910716	4,988049	5,340284	5,4974853	5,413016333	-0,084469	0,943131602	---	
17313039	5,026106	5,088042	5,14644	4,655248	5,292441	5,05954	5,0868627	5,002409667	-0,084453	0,943142062	---	11/03/17
17464776	7,595556	7,355239	7,464391	7,587575	7,300271	7,274051	7,4717287	7,387299	-0,08442967	0,943157316	Rpa3	
17519886	3,233192	2,946594	2,92538	3,270892	2,695626	2,885363	3,0350553	2,950627	-0,08442833	0,943158188	---	
17204679	6,334855	6,753641	6,424831	6,619322	6,745897	5,894835	6,5044423	6,420018	-0,08442433	0,943160802	---	
17402479	2,913804	2,995563	3,308858	3,00682	2,948908	3,009247	3,0727417	2,988325	-0,08441667	0,943165815	Mir302a	
17200421	5,432846	5,654348	5,239128	6,042706	5,030246	5,000164	5,4421073	5,357705333	-0,084402	0,943175403	---	
17340784	4,881435	4,44001	4,433889	4,66789	4,239436	4,594834	4,5851113	4,50072	-0,08439133	0,943182376	D17Ertd648e // D17Ertd648e	
17507698	5,564263	5,241278	5,626046	5,337025	5,361435	5,479969	5,4771957	5,392809667	-0,084386	0,943185863	Arhgef10	
17476680	4,268596	3,623449	3,669592	3,850011	3,726147	3,732331	3,853879	3,769496333	-0,08438267	0,943188042	Gm12776	
17442834	5,489187	5,612718	5,269834	5,357444	5,441441	5,319793	5,4572463	5,372892667	-0,08435367	0,943207002	Gpr133	
17298839	10,30502	10,32554	10,38665	10,22507	10,3132	10,2259	10,33907	10,25472333	-0,08434667	0,943211578	Wapal	
17426166	9,471485	9,243632	9,429104	9,309435	9,236177	9,345572	9,381407	9,297061333	-0,08434567	0,943212232	Cdc26	
17450911	3,403204	3,015294	2,941804	3,30875	2,852371	2,946184	3,1201007	3,035768333	-0,08433233	0,943220949	Vmn2r15	
17206099	2,799119	3,237674	3,403462	2,845096	3,221154	3,12101	3,1467517	3,06242	-0,08433167	0,943221385	---	
17549762	7,307084	6,813592	7,147304	7,11312	6,909085	6,992784	7,0893267	7,004996333	-0,08433033	0,943222257	---	
17381935	3,534088	2,934485	3,376108	3,174611	3,23273	3,184353	3,2815603	3,197231333	-0,084329	0,943223129	---	
17424813	3,928697	3,730507	3,826419	3,937874	3,648199	3,646589	3,828541	3,744220667	-0,08432033	0,943228795	Olfr29-ps1	
17533081	3,084508	3,057201	2,930679	2,888004	2,889197	3,04227	3,0241293	2,939823667	-0,08430567	0,943238384	---	
17204865	3,63646	3,44652	3,763968	3,714949	3,527617	3,351504	3,6156493	3,531356667	-0,08429267	0,943246883	---	
17372916	4,013274	3,91154	3,736853	3,887763	3,745694	3,775427	3,8872223	3,802961333	-0,084261	0,943267588	---	
17226991	7,860459	7,95523	7,980137	7,809856	7,88071	7,852504	7,931942	7,84769	-0,084252	0,943273472	Snrpe	
17358013	10,69702	10,55824	10,63423	10,22903	10,51005	10,89772	10,62983	10,5456	-0,08423	0,943287856	---	
17525363	7,315436	7,726945	7,375288	7,396801	7,424479	7,343742	7,4725563	7,388340667	-0,08421567	0,943297228	Foxred1	

17410190	9,134244	9,192302	9,020801	8,974569	9,086347	9,033821	9,1157823	9,031579	-0,08420333	0,943305292	Larp7
17352662	8,406651	8,535974	8,499215	8,326492	8,426888	8,435862	8,4806133	8,396414	-0,08419933	0,943307908	Esco1
17363997	6,620306	5,90524	5,62699	5,722777	6,10005	6,077126	6,0508453	5,966651	-0,08419433	0,943311177	Rnls
17538891	5,582986	5,408485	5,284229	5,651697	5,327842	5,043615	5,4252333	5,341051333	-0,084182	0,943319241	Iqsec2
17361041	6,786318	7,052576	7,038693	6,789365	7,117004	6,718676	6,9591957	6,875015	-0,08418067	0,943320113	Aldh3b1
17203171	3,889673	3,43529	3,393245	3,937432	3,173898	3,35435	3,572736	3,48856	-0,084176	0,943323164	---
17255620	5,202806	5,091723	5,06139	4,932404	5,159111	5,011876	5,1186397	5,034463667	-0,084176	0,943323164	Hoxb1
17548373	8,24634	8,324987	8,367443	8,228846	8,264339	8,193068	8,3129233	8,228751	-0,08417233	0,943325562	---
17313322	7,514476	7,73173	7,712447	7,71447	7,435047	7,556652	7,6528843	7,568723	-0,08416133	0,943332754	L3mbtl2
17514687	5,074983	5,630818	5,552512	5,619598	5,240768	5,145499	5,4194377	5,335288333	-0,08414933	0,943340601	Phxr4
17208517	5,154724	5,29494	4,8728	4,853838	5,148654	5,067571	5,107488	5,023354333	-0,08413367	0,943350845	---
17207271	5,339586	5,394014	4,94227	4,115229	5,488816	5,819495	5,22529	5,14118	-0,08411	0,94336632	---
17275490	8,239938	8,576741	8,259846	8,43959	8,146782	8,237835	8,3588417	8,274735667	-0,084106	0,943368936	Fam177a // 1700047I17Rik2
17275516	8,239938	8,576741	8,259846	8,43959	8,146782	8,237835	8,3588417	8,274735667	-0,084106	0,943368936	Fam177a // 1700047I17Rik2
17364367	7,988058	8,072226	7,912881	7,832314	7,89207	7,996496	7,991055	7,90696	-0,084095	0,943376128	Noc3l
17489534	3,562308	3,63052	3,54035	3,466573	3,562308	3,452029	3,577726	3,493636667	-0,08408933	0,943379834	Gm4692
17407864	8,047293	8,168507	7,949112	8,046121	8,01128	7,855246	8,0549707	7,970882333	-0,08408833	0,943380488	Tars2
17527977	8,257422	8,165608	8,002644	7,91721	8,220377	8,035955	8,1418913	8,057847333	-0,084044	0,943409478	Glce
17204699	7,602678	8,09697	7,456875	7,770104	7,214314	7,919995	7,718841	7,634804333	-0,08403667	0,943414273	---
17346522	5,885901	5,949097	5,88978	5,78069	5,803285	5,888766	5,9082593	5,824247	-0,08401233	0,943430186	Tnfsf14
17502206	5,806612	5,902642	6,036878	5,575298	5,920123	5,998741	5,9153773	5,831387333	-0,08399	0,94344479	Gtpbp3
17440457	7,007827	6,855811	6,868577	6,804745	6,752984	6,922631	6,9107383	6,826786667	-0,08395167	0,943469859	Pgam5 // Pgam5
17548831	11,12842	11,13983	11,2856	11,55564	11,09668	10,64968	11,184617	11,10066667	-0,08395	0,943470949	Erdr1 // Erdr1
17290684	4,926136	4,435219	4,704102	4,305149	4,882506	4,625978	4,6884857	4,604544333	-0,08394133	0,943476616	Gm2423
17355790	7,056057	6,92456	6,850084	6,835952	6,754131	6,988804	6,943567	6,859629	-0,083938	0,943478796	Tshz1
17237026	3,489827	3,26103	3,338067	3,26419	3,259487	3,313444	3,3629747	3,279040333	-0,08393433	0,943481194	Mir3059
17222326	9,353051	9,316158	9,141405	9,123211	9,237955	9,19767	9,2702047	9,186278667	-0,083926	0,943486644	6330578E17Rik
17275567	3,948784	3,495411	3,770787	3,614609	3,500516	3,848212	3,7383273	3,654445667	-0,08388167	0,943515637	Aldoart2
17338738	5,946023	5,954761	6,098866	5,779251	6,187881	5,780946	5,9998833	5,916026	-0,08385733	0,943531551	A230051N06Rik
17473166	5,517544	5,489195	5,491985	5,385587	5,257132	5,60445	5,4995747	5,415723	-0,08385167	0,943535257	Cacng6
17211369	3,962254	4,270216	4,038474	3,878136	3,959796	4,181468	4,0903147	4,006466667	-0,083848	0,943537655	Il17a
17236091	7,541145	7,962541	7,767944	7,506554	7,86173	7,651856	7,75721	7,67338	-0,08383	0,943549428	AI597468
17426752	3,568572	3,390562	3,607165	3,402546	3,37686	3,535467	3,5220997	3,438291	-0,08380867	0,94356338	---
17277876	4,459206	4,504256	4,645374	4,474708	4,556399	4,326336	4,5362787	4,452481	-0,08379767	0,943570574	---
17208985	3,780196	4,650786	4,810248	5,106273	4,021473	3,862117	4,4137433	4,329954333	-0,083789	0,943576243	---
17257556	6,436007	7,43396	7,076774	6,917135	6,744006	7,03427	6,982247	6,898470333	-0,08377667	0,943584309	---
17353106	3,833166	3,144947	3,474895	3,188791	3,736492	3,276403	3,484336	3,400562	-0,083774	0,943586053	---
17308842	6,543234	6,756833	6,69774	6,603753	6,554564	6,588173	6,6659357	6,582163333	-0,08377233	0,943587143	Dgkh
17220761	8,707753	8,767103	8,384228	8,530437	8,265435	8,811903	8,6196947	8,535925	-0,08376967	0,943588887	---
17519127	11,02376	11,41393	11,11135	11,05079	10,88404	11,36297	11,183013	11,09926667	-0,08374667	0,943603931	---
17261724	3,28021	2,940811	3,193378	3,17105	3,06256	2,929609	3,138133	3,054406333	-0,08372667	0,943617012	---
17431234	5,194559	5,272486	5,457259	5,213118	5,296396	5,163697	5,3081013	5,224403667	-0,08369767	0,94363598	Cnksr1
17539879	7,877867	7,814942	7,967121	7,934328	7,785705	7,688834	7,8866433	7,802955667	-0,08368767	0,943642521	Plp2
17269521	9,735238	9,791215	9,682383	9,685232	9,487403	9,785141	9,7362787	9,652592	-0,08368667	0,943643175	Acly
17548440	9,709306	9,88623	9,837248	9,755119	9,776395	9,650212	9,810928	9,727242	-0,083686	0,943643611	Imp3 // Imp3
17385030	8,39502	8,517617	8,367185	8,297721	8,392921	8,338138	8,4266073	8,342926667	-0,08368067	0,943647099	Mmadhc
17277278	6,13137	5,906484	6,110675	5,963127	6,081376	5,853076	6,0495097	5,965859667	-0,08365	0,943667158	Vsx2
17281635	3,574652	3,53621	3,620967	3,631796	3,529829	3,319268	3,5772763	3,493631	-0,08364533	0,943670211	Mir681
17350081	5,091167	4,924244	5,292332	5,063664	5,004597	4,988559	5,102581	5,01894	-0,083641	0,943673045	Kctd16
17485545	6,424569	6,418159	6,594271	6,229106	6,604009	6,352962	6,4789997	6,395359	-0,08364067	0,943673263	Leng1
17261187	4,941579	4,686141	4,717349	4,696758	4,696854	4,700579	4,7816897	4,698063667	-0,083626	0,943682857	5730522E02Rik
17484601	9,198332	9,237564	9,160652	9,149357	9,059904	9,136425	9,1988493	9,115228667	-0,08362067	0,943686345	1190003J15Rik
17205529	4,512994	4,862581	5,176514	5,025343	4,705757	4,570149	4,8506963	4,767083	-0,08361333	0,943691142	---
17408180	5,079111	5,033464	5,08278	5,02459	4,915054	5,004919	5,0651183	4,981521	-0,08359733	0,943701608	---
17377071	6,94729	6,996869	6,921261	6,850139	6,835061	6,929445	6,95514	6,871548333	-0,08359167	0,943705315	Polr3f
17371218	3,605469	3,649196	3,359604	3,503992	3,440016	3,419492	3,5380897	3,4545	-0,08358967	0,943706623	---
17256522	7,227693	7,38485	7,330153	7,230396	7,352005	7,109556	7,314232	7,230652333	-0,08357967	0,943713164	Coasy
17482717	6,323277	6,105385	6,278468	6,177392	5,987679	6,291378	6,23571	6,152149667	-0,08356033	0,943725811	Gga2 // Gga2
17201411	6,254392	6,272652	7,145333	6,694036	5,90818	6,8195	6,557459	6,473905333	-0,08355367	0,943730172	---
17330029	5,155503	5,746556	5,560284	5,292144	5,46071	5,458845	5,4874477	5,403899667	-0,083548	0,943733879	1700119H24Rik
17394318	7,847462	7,657793	7,803946	7,771228	7,653243	7,634143	7,7697337	7,686204667	-0,083529	0,943746308	Zfp335
17480420	7,243907	7,244682	7,200093	7,08456	7,204705	7,148839	7,2295607	7,146034667	-0,083526	0,94374827	Pak1
17533269	8,411281	8,544353	8,172365	8,311543	8,288218	8,277674	8,3759997	8,292478333	-0,08352133	0,943751323	Tspan7
17473671	4,289104	4,264668	4,007764	4,077495	3,994131	4,239378	4,1871787	4,103668	-0,08351067	0,943758301	Nlrp4b
17531685	4,903296	4,743056	4,479223	4,703487	4,488177	4,683414	4,708525	4,625026	-0,083499	0,943765933	Tdgf1
17205891	3,823438	3,335541	3,645894	4,220637	3,563514	2,770268	3,6016243	3,518139667	-0,08348467	0,943775309	---
17326650	5,253412	5,380483	5,265366	5,17146	5,446789	5,030563	5,2997537	5,216270667	-0,083483	0,943776399	---
17222354	3,877152	4,111823	4,040097	3,837273	3,92703	4,014332	4,0096907	3,926211667	-0,083479	0,943779016	4930594C11Rik
17528900	2,962329	2,949859	3,118352	2,820616	2,997231	2,962329	3,01018	2,926725333	-0,08345467	0,943794934	---
17467195	3,695481	4,066686	4,114136	3,772379	3,856869	3,996717	3,9587677	3,875321667	-0,083446	0,943800604	Vmn1r26
17469587	9,066711	9,094631	9,00212	8,925058	8,992791	8,995365	9,0544873	8,971071333	-0,083416	0,94382023	---
17471973	7,55096	7,642248	7,111274	7,128442	7,139627	7,786251	7,4348273	7,35144	-0,08338733	0,943838984	---
17514566	5,307435	5,442328	5,107221	5,27108	5,295838	5,039929	5,2856613	5,202282333	-0,083379	0,943844436	Mmp27
17500447	3,91289	3,696061	3,697225	3,882105	3,639284	3,534726	3,7687253	3,685371667	-0,08335367	0,94386101	---
17428209	4,078331	3,936994	3,872399	3,812307	4,036448	3,788955	3,9625747	3,879236667	-0,083338	0,94387126	9630013D21Rik
17372231	2,857013	2,790401	3,025067	2,89822	2,749258	2,775008	2,890827	2,807495333	-0,08333167	0,943875403	---
17480459	5,920717	5,313593	5,618639	5,666889	5,461823	5,474251	5,6176497	5,534321	-0,08332867	0,943877366	LOC330599
17320783	6,506723	6,576348	6,556735	6,545501	6,453361	6,390967	6,546602	6,463276333	-0,08332567	0,943879329	Pus7l
17367901	9,019651	8,100777	8,961513	8,084039	8,843989	8,903948	8,6939803	8,610658667	-0,08332167	0,943881946	---
17494325	6,574283	6,725255	6,438185	6,373954	6,764626	6,349186	6,579241	6,495922	-0,083319	0,94388369	Trim12c
17473728	3,959911	3,685135	3,936984	3,827672	3,754679	3,749805	3,8606767	3,777385333	-0,08329133	0,943901791	Vmn1r75
17427661	5,436227	5,661067	5,402722	5,326746	5,446144	5,477269	5,5000053	5,416719667	-0,08328567	0,943905499	Wdr78
17348791	4,246387	4,201915	4,507112	4,176965	4,30034	4,228274	4,3184713	4,235193	-0,08327833	0,943910297	Dsg3
17280392	7,418141	7,537189	7,479995	7,311808	7,588878	7,284845	7,4784417	7,395177	-0,08326467	0,943919238	Rps7
17545582	3,924364	3,603227	3,293338	3,504986	3,436775	3,629404	3,6069763	3,523721667	-0,08325467	0,943925781	Magea1
17271757	7,235018	7,054933	7,105324	7,039366	6,958694	7,14747	7,1317583	7,04851	-0,08324833	0,943929925	Gm11711
17448064	6,347283	6,683356	6,645169	6,292946	6,753752	6,379416	6,5586027	6,475371333	-0,08323133	0,943941048	Lgi2
17485739	3,503187	3,250038	3,45256	3,594393	3,141581	3,220207	3,4019283	3,318727	-0,08320133	0,943960677	Rdh13
17449473	13,34694	13,46118	13,39977	13,34944	13,28424	13,32471	13,40263	13,31946333	-0,08316667	0,94398336	Gc
17475303	5,232011	5,334593	5,628011	5,252865	5,234562	5,457762	5,398205	5,315063	-0,083142	0,9439995	4732471J01Rik
17456450	3,214321	3,302181	3,308418	3,151494	3,236356	3,187681	3,2749733	3,191843667	-0,08312967	0,94400757	Spam1
17258644	4,122889	4,048537	4,295946	3,983745	4,236986	3,997306	4,1557907	4,072679	-0,08311167	0,944019348	BC018473
17234865	4,054322	3,579366	3,628222	3,725479	3,844516	3,442596	3,75397	3,670863667	-0,08310633	0,944022838	Vmn2r80
17543246	4,941621	4,608349	4,778752	4,833418	4,483726	4,762265	4,7762407	4,693136333	-0,08310433	0,944024146	4932442L08Rik
17278345	10,68301	10,67702	10,62569	10,46114	10,64318	10,63209	10,661907	10,57880333	-0,08310333	0,944024801	Glrx5
17231040	7,024282	6,769501	6,728589	6,769501	6,826274	6,677297	6,8407907	6,757690667	-0,0831	0,944026982	Nenf
17387728	4,208175	3,557244	3,491524	3,962065	3,543781	3,50194	3,7523143	3,669262	-0,08305233	0,944058173	Olfr1101
17319619	9,886451	9,932707	9,688673	9,762401	9,763576	9,732764	9,8359437	9,752913667	-0,08303	0,944072788	Ndufa6
17527529	5,888575	5,627599	6,227078	5,973501	5,567221	5,953491	5,9144173	5,831404333	-0,083013	0,944083912	---
17301108	6,540968	6,587366	6,723984	6,507736	6,693465	6,402146	6,6174393	6,534449	-0,08299033	0,944098745	Arl11
17200975	8,484681	8,589897	8,739585	7,929144	8,897478	8,738589	8,604721	8,521737	-0,082984	0,94410289	---
17495097	10,40161	10,44105	10,40711	10,36873	10,27872	10,35338	10,41659	10,33361	-0,08298	0,944105507	Lyve1
17465535	6,167656	6,181576	6,141748	6,043535	6,124677	6,073876	6,16366	6,080696	-0,082964	0,944115978	Tsga14
17392452	7,840179	7,989224	7,946935	7,714204	7,950204	7,863074	7,925446	7,842494	-0,082952	0,944123831	Crnkl1
17443112	5,792214	5,799537	5,893598	5,684782	5,690261	5,861463	5,8284497	5,745502	-0,08294767	0,944126666	---
17240296	4,084815	3,948167	3,929711	3,980104	3,902121	3,83164	3,9875643	3,904621667	-0,08294267	0,944129939	2010001E11Rik
17428655	6,502663	6,502295	6,606442	6,423225	6,503189	6,436234	6,5371333	6,454216	-0,08291733	0,944146517	Gpbp1l1
17350084	3,83732	4,095024	4,567656	4,315385	4,179256	3,75661	4,1666667	4,083750333	-0,08291633	0,944147172	---
17503907	6,015111	6,171716	6,076965	5,978361	6,056485	5,980211	6,0879307	6,005019	-0,08291167	0,944150226	LOC100861643
17413216	5,062037	4,971159	4,932888	5,175745	4,557735	4,983911	4,9886947	4,905797	-0,08289767	0,944159388	---
17372880	4,378134	4,272027	4,476326	4,515598	4,265141	4,097092	4,3754957	4,292610333	-0,08288533	0,944167459	Olfr152
17202631	3,464534	4,827642	3,535351	4,042764	4,102835	3,433342	3,942509	3,859647	-0,082862	0,94418273	---
17526456	4,917774	4,783312	5,020203	4,746036	4,850307	4,876386	4,9070963	4,824243	-0,08285333	0,944188402	Cd3g
17231534	7,211889	7,417305	7,183045	7,138882	7,167307	7,257516	7,2707463	7,187901667	-0,08284467	0,944194074	Nup43
17270028	6,602486	6,448082	6,444773	6,316753	6,389173	6,540902	6,498447	6,415609333	-0,08283767	0,944198655	Mpp2
17222549	8,642902	8,613173	8,752353	8,51503	8,609493	8,635413	8,669476	8,586645333	-0,08283067	0,944203237	Rnf149
17513976	12,56676	12,61463	12,78122	12,44066	12,67836	12,5951	12,654203	12,57137333	-0,08283	0,944203673	Rn5s20 // Rn5s20
17536881	7,558442	7,549365	7,588264	7,467433	7,489634	7,490526	7,565357	7,482531	-0,082826	0,944206291	Pin4
17350011	9,781633	9,895383	9,751088	9,756443	9,58923	9,833962	9,809368	9,726545	-0,082823	0,944208254	Ndfip1
17488492	6,108148	5,824092	6,308573	6,064654	5,948246	5,979445	6,080271	5,997448333	-0,08282267	0,944208472	Nccrp1
17331577	4,230371	4,153515	3,971688	3,955243	4,099052	4,05282	4,1185247	4,035705	-0,08281967	0,944210436	1700041M19Rik
17401240	5,297562	4,889961	5,450475	5,169714	5,180105	5,039745	5,212666	5,129854667	-0,08281133	0,94421589	Dclre1b // Dclre1b
17451126	9,28762	9,228961	9,227495	9,103681	9,339865	9,052166	9,2480253	9,165237333	-0,082788	0,944231161	Ep400
17460250	2,93742	2,994423	2,962536	2,989025	2,813488	2,843621	2,964793	2,882044667	-0,08274833	0,944257123	---
17534899	5,422936	4,891974	4,821531	4,967145	5,091547	4,829566	5,0454803	4,962752667	-0,08272767	0,94427065	Ncrna00086
17409826	9,094163	9,082855	8,804004	8,696712	9,009057	9,027072	8,993674	8,910947	-0,082727	0,944271086	Snx7
17433265	4,596483	4,39958	4,60683	4,548708	4,410719	4,395323	4,5342977	4,451583333	-0,08271433	0,944279376	Car6
17260416	7,202958	7,573778	7,336388	7,273718	7,270732	7,320566	7,3710413	7,288338667	-0,08270267	0,944287013	Tbrg4
17262572	5,795514	5,596272	6,103617	5,81045	5,773465	5,663434	5,831801	5,749116333	-0,08268467	0,944298794	---
17394908	5,897288	5,19472	5,443854	5,563107	5,459438	5,265316	5,511954	5,429287	-0,082667	0,944310358	---
17470084	6,317518	5,627505	5,809419	6,281157	5,508072	5,717215	5,9181473	5,835481333	-0,082666	0,944311012	---
17437263	6,8135	7,108814	7,015889	6,895538	6,885409	6,909302	6,979401	6,896749667	-0,08265133	0,944320612	Med28
17430609	6,639111	6,799158	6,428751	6,437686	6,631582	6,549835	6,62234	6,539701	-0,082639	0,944328685	Serinc2
17313858	3,165324	3,216607	3,506018	3,142891	3,489123	3,008051	3,295983	3,213355	-0,082628	0,944335885	Mirlet7c-2

17472023	7,178909	6,826306	6,850288	6,674225	7,036616	6,896843	6,9518343	6,869228	-0,08260633	0,944350068	Mansc1
17293604	5,90097	5,669623	5,675442	5,669286	5,741276	5,587681	5,7486783	5,666081	-0,08259733	0,944355959	---
17289129	5,278802	4,594522	4,72436	4,806602	4,744957	4,798343	4,8658947	4,783300667	-0,082594	0,944358141	Gm4814
17268177	5,469478	5,56271	5,909472	5,669187	5,432011	5,592724	5,64722	5,564640667	-0,08257933	0,944367741	---
17229481	6,910033	6,905788	6,546581	6,762436	6,628601	6,72364	6,7874673	6,704892333	-0,082575	0,944370578	Ddr2
17203085	7,757266	7,159855	7,628603	7,57056	7,318611	7,408852	7,5152413	7,432674333	-0,082567	0,944375815	---
17200563	6,118988	5,402699	5,986493	6,63787	5,326897	5,295783	5,83606	5,753516667	-0,08254333	0,944391307	---
17431801	4,207684	3,957961	3,844419	3,952425	3,996528	3,813505	4,0033547	3,920819333	-0,08253533	0,944396544	Ubxn10
17281428	8,872933	8,847499	8,789035	8,618289	8,720868	8,922705	8,836489	8,753954	-0,082535	0,944396762	Mis18bp1
17548492	4,839109	4,619046	4,651487	4,466479	4,614697	4,780863	4,703214	4,620679667	-0,08253433	0,944397198	Gm8675
17254522	5,347698	4,864489	5,04668	4,909166	5,004466	5,097649	5,086289	5,003760333	-0,08252867	0,944400908	1700125H20Rik
17447737	7,986545	8,264498	7,942171	7,957412	7,971937	8,016307	8,0644047	7,981885333	-0,08251933	0,944407017	Rab28
17541516	4,317225	3,757013	3,792414	3,966137	3,938355	3,714602	3,9555507	3,873031333	-0,08251933	0,944407017	---
17316690	8,009079	8,128148	8,033796	7,924889	7,943619	8,054968	8,0570077	7,974492	-0,08251567	0,944409418	Klf10
17208203	3,966893	3,415519	3,592459	3,620167	3,34131	3,76588	3,6582903	3,575785667	-0,08250467	0,944416618	---
17266653	4,654902	4,512173	4,488074	4,618998	4,459319	4,329327	4,5517163	4,469214667	-0,08250167	0,944418582	BC037438 // BC037438
17307121	3,704787	3,552537	3,69496	3,566053	3,432803	3,705927	3,6507613	3,568261	-0,08250033	0,944419455	Gm4491
17268569	5,613297	5,697064	5,7385	5,436021	5,768969	5,596395	5,6829537	5,600461667	-0,082492	0,94442491	Srcin1
17549452	5,276218	4,889418	5,112069	5,113785	4,890827	5,025639	5,0925683	5,010083667	-0,08248467	0,944429711	---
17238703	3,078651	3,731833	3,205391	3,299208	3,140574	3,328655	3,338625	3,256145667	-0,08247933	0,944433202	Olfr822
17452360	6,624437	6,423671	6,749826	6,613306	6,248441	6,688785	6,5993113	6,516844	-0,08246733	0,944441058	Tctn1
17201589	4,695743	4,32994	4,354386	4,311082	4,214713	4,607001	4,460023	4,377598667	-0,08242433	0,944469208	---
17440754	10,89471	11,09149	10,84945	10,79464	11,14974	10,644	10,945217	10,86279333	-0,08242333	0,944469862	Rps28 // Rps28
17425784	4,749707	4,738765	4,663168	4,696511	4,714971	4,492901	4,7172133	4,634794333	-0,082419	0,944472699	AI481877
17228276	4,577646	4,641033	5,003654	4,758045	4,676679	4,540415	4,7407777	4,658379667	-0,082398	0,944486447	5830403L16Rik
17489508	5,821229	5,841509	5,756	5,718929	5,721479	5,731192	5,806246	5,723866667	-0,08237933	0,944498668	Gm5329
17213676	9,050245	9,319206	9,017811	9,010041	9,100721	9,029383	9,1290873	9,046715	-0,08237233	0,94450325	Creb1
17393743	5,651958	5,599895	5,768488	5,405862	5,633164	5,734199	5,673447	5,591075	-0,082372	0,944503469	Ghrh
17309360	4,296593	3,993695	4,495099	4,407872	4,092093	4,038329	4,2617957	4,179431333	-0,08236433	0,944508488	Slitrk1
17278614	9,112	9,047758	9,155169	9,052732	8,990057	9,02505	9,1049757	9,022613	-0,08236267	0,944509579	Yy1
17448557	3,921789	3,816675	3,93016	3,944309	3,7455	3,731742	3,8895413	3,807183667	-0,08235767	0,944512852	---
17286922	3,66407	3,576337	3,796113	3,455403	3,62319	3,710907	3,67884	3,5965	-0,08234	0,944524419	1700029N11Rik
17240586	5,349704	5,357563	5,340743	5,168786	5,318132	5,314079	5,3493367	5,266999	-0,08233767	0,944525946	Sobp
17206429	5,004077	4,777265	4,673073	4,975518	4,10792	5,123971	4,8181383	4,735803	-0,08233533	0,944527474	---
17370439	7,277877	7,181805	7,36908	7,259383	7,276586	7,045843	7,276254	7,193937333	-0,08231667	0,944539695	Lhx2
17541636	4,981328	5,05126	4,937841	4,711332	5,008501	5,003651	4,990143	4,907828	-0,0823150	0,944540786	Hs6st2
17266882	5,278687	4,606537	4,909555	4,955206	4,846942	4,745724	4,931593	4,849290667	-0,08230233	0,944549079	---
17354339	3,674477	3,718388	3,757321	3,676856	3,437246	3,789198	3,7167287	3,634433333	-0,08229533	0,944553662	1700065O20Rik
17444730	5,081931	4,85038	5,143957	4,727226	4,933786	5,168378	5,0254227	4,94313	-0,08229267	0,944555408	Wasf3
17374432	3,875322	3,992762	3,921724	3,798398	3,876565	3,868077	3,929936	3,84768	-0,082256	0,944579415	Tmco5
17209743	4,660684	4,447014	4,408552	4,589614	3,97274	4,70715	4,5054167	4,423168	-0,08224867	0,944584216	---
17202345	5,186241	5,838829	6,151226	5,775427	6,177724	4,97644	5,725432	5,643197	-0,082235	0,944593164	---
17542088	3,282035	3,048526	3,120856	3,123178	3,08261	2,998926	3,1504723	3,068238	-0,08223433	0,944593601	Mir465c-1
17542092	3,282035	3,048526	3,120856	3,123178	3,08261	2,998926	3,1504723	3,068238	-0,08223433	0,944593601	Mir465c-1
17407439	5,558911	5,427156	5,46539	5,269204	5,427156	5,508491	5,483819	5,401617	-0,082202	0,944614771	Lce1a2
17253233	3,627164	3,745725	3,272623	3,408727	3,472903	3,517284	3,548504	3,466304667	-0,08219933	0,944616517	---
17418094	3,628505	3,52231	3,440607	3,418746	3,553077	3,373064	3,530474	3,448295667	-0,08217833	0,944630267	9530002B09Rik
17447904	7,853455	7,786892	7,710042	7,423333	7,767282	7,913285	7,783463	7,7013	-0,082163	0,944640307	Qdpr
17548810	6,026076	6,113679	6,313273	6,28981	5,935265	5,981486	6,1510093	6,068853667	-0,08215567	0,944645108	---
17289691	3,309163	3,329101	3,585753	3,274541	3,309126	3,393883	3,4080057	3,32585	-0,08215567	0,944645108	1700006H21Rik
17345262	7,802099	7,767638	7,736946	7,593865	7,825469	7,64089	7,7688943	7,686741333	-0,082153	0,944646854	Tmem63b
17547799	5,973215	5,739753	5,984911	5,928219	5,780025	5,743185	5,899293	5,817143	-0,08215	0,944648819	Gm20242
17268469	9,815095	9,857842	9,739332	9,660036	9,805427	9,700362	9,8040897	9,721941667	-0,082148	0,944650128	Kpnb1
17207335	5,768868	5,874971	5,527254	6,072923	5,594311	5,257438	5,7236977	5,641557333	-0,08214033	0,944655148	---
17258153	3,695481	3,501326	3,90833	3,660703	3,449521	3,748543	3,7017123	3,619589	-0,08212333	0,94466628	Grin2c // Grin2c
17388667	6,494671	6,485646	6,537873	6,357316	6,437329	6,477221	6,5060633	6,423955333	-0,082108	0,94467632	B230118H07Rik
17249853	4,909479	4,965645	4,57387	4,683828	4,835252	4,683615	4,8163313	4,734231667	-0,08209967	0,944681777	---
17326979	5,206864	5,279083	5,413506	5,224133	5,207736	5,221292	5,2998177	5,217720333	-0,08209733	0,944683305	---
17511014	6,04987	6,116188	5,803855	5,983236	5,8238	5,916624	5,989971	5,907886667	-0,08208433	0,944691817	---
17349085	3,774742	3,477297	3,63746	3,651703	3,620769	3,370774	3,629833	3,547748667	-0,08208433	0,944691817	---
17312257	5,185496	5,008806	5,284802	5,108424	5,12093	5,003512	5,1597013	5,077622	-0,08207933	0,944695091	---
17400581	6,36993	6,306884	6,464566	6,359132	6,272261	6,26378	6,38046	6,298391	-0,082069	0,944701858	4930459I23Rik
17408033	6,36993	6,306884	6,464566	6,359132	6,272261	6,26378	6,38046	6,298391	-0,082069	0,944701858	4930459I23Rik
17548523	6,376951	6,361147	6,377295	6,389315	6,028849	6,451045	6,3717977	6,289736333	-0,08206133	0,944706878	Gemin6 // Gemin6
17401094	11,42141	11,47434	11,46812	11,31806	11,38755	11,4121	11,454623	11,37257	-0,08205333	0,944712116	Csde1
17548875	3,245922	3,167544	3,134998	3,083507	3,130985	3,08786	3,1828213	3,100784	-0,08203733	0,944722594	---
17284539	6,620759	6,35089	6,405571	6,133293	6,579353	6,418502	6,4590733	6,377049333	-0,082024	0,944731325	---
17475215	7,108752	7,366513	7,402488	7,688985	6,595272	7,347445	7,2925843	7,210567333	-0,082017	0,944735909	---
17546485	3,709245	3,551337	3,499078	3,603311	3,602497	3,307812	3,5865533	3,50454	-0,08201333	0,94473831	---
17329516	4,848557	4,964844	5,166836	4,923122	4,97664	4,834522	4,9934123	4,911428	-0,08198433	0,9447573	Fgf12
17282098	7,641665	7,103822	6,843409	7,521927	6,748668	7,072368	7,1962987	7,114321	-0,08197767	0,944761666	---
17217058	5,150773	5,329198	5,180759	5,273558	5,160386	4,980897	5,2202433	5,138280333	-0,081963	0,944771271	Slc26a9
17375608	3,898889	3,799324	3,930936	3,795141	3,805037	3,783094	3,876383	3,794424	-0,081959	0,94477389	Slc12a1
17394041	5,828994	5,904927	5,748034	5,740192	5,819547	5,676385	5,8273183	5,745374667	-0,08194367	0,944783932	0610008F07Rik
17547747	3,482985	3,513044	3,440754	3,420541	3,581203	3,189239	3,4789277	3,396994333	-0,08193333	0,944790699	LOC100861903
17336476	5,321313	5,176836	5,265218	5,031049	5,277126	5,209469	5,2544557	5,172548	-0,08190767	0,944807507	H2-Ob
17284471	3,372269	3,173636	3,557501	3,364644	3,1454	3,347643	3,367802	3,285895667	-0,08190633	0,94480838	---
17230877	3,889486	3,375414	3,42522	3,654616	3,219068	3,570719	3,5633733	3,481467667	-0,08190567	0,944808817	---
17212343	4,451962	4,545125	4,289854	4,129756	4,349741	4,561729	4,4289803	4,347075333	-0,081905	0,944809254	---
17275778	5,319024	5,944157	6,024413	5,519595	6,062458	5,459846	5,7625313	5,680633	-0,08189833	0,94481362	Gm527
17494469	4,36798	4,319578	4,246526	4,259439	4,17218	4,256809	4,3113613	4,229476	-0,08188533	0,944822133	Olfr685
17372908	2,867516	2,925886	2,915218	2,801856	2,852615	2,808507	2,9028733	2,820992667	-0,08188067	0,94482519	Olfr1186
17359716	7,268551	7,50532	7,333391	7,244301	7,318255	7,299111	7,3690873	7,287222333	-0,081865	0,94483545	Hif1an
17536278	4,075456	4,156233	4,074418	3,989422	3,652532	4,127322	4,0049567	3,923092	-0,08186467	0,944835668	AU015836
17506177	6,343683	6,499223	6,430259	6,440451	6,367875	6,21926	6,4243883	6,342528667	-0,08185967	0,944838943	Klhl36
17292598	5,457401	5,393439	5,341867	5,188159	5,428412	5,330584	5,397569	5,315718333	-0,08185067	0,944844837	Diras2
17382101	5,030103	4,952582	5,086962	5,019096	4,861772	4,943264	5,0232157	4,941377333	-0,08183833	0,944852914	Etl4 // Etl4
17531887	4,555144	4,591795	4,540927	4,601625	4,622571	4,218189	4,562622	4,480795	-0,081827	0,944860337	Ccr4
17281299	4,936462	5,08323	5,126885	5,030668	4,956133	4,914307	5,048859	4,967036	-0,081823	0,944862956	Nkx2-1
17363983	10,72965	10,60564	10,34595	10,27683	10,50185	10,6571	10,560413	10,47859333	-0,08182	0,944864921	Atad1
17308349	6,992692	7,234368	6,924198	6,771185	7,083745	7,050972	7,0504193	6,968634	-0,08178533	0,944887626	Polr3d
17467100	6,896271	6,766708	6,961465	6,719723	6,833732	6,825634	6,8748147	6,793029667	-0,081785	0,944887844	Gm8239
17201153	6,033252	7,497019	6,254819	6,620383	6,131917	6,787467	6,59503	6,513255667	-0,08177433	0,94489483	---
17461798	7,170355	7,148503	7,303411	7,096855	7,135169	7,144931	7,207423	7,125651667	-0,08177133	0,944896795	Vhl
17500441	5,758012	5,856549	5,691642	5,602386	5,707904	5,750608	5,7687343	5,686966	-0,08176833	0,94489876	Purg
17402711	7,932952	7,856356	7,79377	7,863035	7,771564	7,703301	7,861026	7,7793	-0,081726	0,944926487	Tbck
17327247	4,298859	3,817518	4,063036	3,919492	4,161523	3,853223	4,0598043	3,978079333	-0,081725	0,944927142	---
17231844	9,00355	8,925317	8,981476	8,857479	8,907378	8,900335	8,9701143	8,888397333	-0,081717	0,944932381	Perp
17230942	5,87376	5,571789	5,974694	5,794337	5,774742	5,606021	5,8067477	5,725033333	-0,08171433	0,944934128	---
17546488	5,159414	5,991804	6,157378	5,305217	6,379244	5,378999	5,769532	5,68782	-0,081712	0,944935656	Gm17361 // Gm17361
17382533	6,668715	6,606794	6,261727	6,43126	6,428059	6,432805	6,512412	6,430708	-0,081704	0,944940896	Dpp7
17336602	6,212808	6,251721	5,8154	6,177723	5,824257	6,032852	6,0933097	6,011610667	-0,081699	0,944944171	Pbx2
17407947	5,885379	6,040915	5,697957	5,914627	5,506643	5,957901	5,8747503	5,793057	-0,08169333	0,944947883	C920021L13Rik
17444913	6,346714	6,154962	6,172993	6,065575	6,239496	6,124521	6,2248897	6,143197333	-0,08169233	0,944948538	2210417A02Rik
17521296	2,983143	3,091578	2,819965	2,865655	2,823388	2,960621	2,9648953	2,883221333	-0,081674	0,944960546	---
17297000	4,957732	4,357322	3,676611	4,463088	4,173456	4,110163	4,330555	4,248902333	-0,08165267	0,944974519	4933406F09Rik
17270286	4,729231	5,048008	5,191342	4,844278	4,898519	4,980827	4,989527	4,907874667	-0,08165233	0,944974738	Gm11627
17488287	4,618434	4,575161	4,815565	4,630946	4,718491	4,414779	4,66972	4,588072	-0,081648	0,944977576	---
17358591	5,612369	5,631921	5,590149	5,545116	5,604373	5,440022	5,6114797	5,529837	-0,08164267	0,944981069	Mlana
17512413	7,07854	7,064227	6,868638	7,07188	6,785636	6,909016	7,0038017	6,922177333	-0,08162433	0,944993078	---
17471962	3,813155	3,836485	3,745183	3,529838	3,911809	3,708314	3,7982743	3,716653667	-0,08162067	0,94499548	Tas2r131
17453487	6,440604	6,227966	6,46056	6,543118	6,165986	6,17517	6,3763767	6,294758	-0,08161867	0,94499679	Wbscr28
17202017	6,705808	6,788255	6,889433	6,677504	6,732289	6,728853	6,7944987	6,712882	-0,08161667	0,9449981	---
17461423	10,11535	10,11487	10,05183	9,916196	9,963664	10,15742	10,094017	10,01242667	-0,08159	0,945015567	Arl8b
17431740	4,20437	4,476169	4,423135	4,199525	4,523973	4,13544	4,3678913	4,286312667	-0,08157867	0,945022991	---
17434585	10,39456	10,44893	10,34832	10,20121	10,47788	10,26801	10,39727	10,3157	-0,08157	0,945028668	Abcb4
17469541	5,60428	5,38069	5,280196	5,253329	5,421789	5,345341	5,421722	5,340153	-0,081569	0,945029323	Il5ra
17414738	3,693394	3,618063	3,404855	3,58612	3,227271	3,658231	3,572104	3,490540667	-0,08156333	0,945033035	Orm3
17287432	6,427814	6,513008	6,666141	6,551068	6,403788	6,407423	6,5356543	6,454093	-0,08156133	0,945034345	Cplx2 // Cplx2
17535930	9,294969	9,580531	9,505527	9,36721	9,362922	9,406281	9,4603423	9,378804333	-0,081538	0,94504963	Dkc1
17305167	10,42349	10,44725	10,37566	10,22303	10,45039	10,32842	10,415467	10,33394667	-0,08152	0,945061421	Ghitm
17246392	4,401893	4,156233	4,081612	4,319799	4,003338	4,072049	4,213246	4,131728667	-0,08151733	0,945063168	Olfr767
17461269	3,682787	3,408994	3,318302	3,376377	3,477207	3,311963	3,4700277	3,388515667	-0,081512	0,945066661	---
17486126	4,158821	4,080237	3,962133	3,889759	4,052091	4,014809	4,0670637	3,985553	-0,08151067	0,945067535	---
17313991	7,307272	7,120648	7,0353	7,010236	7,199118	7,009343	7,1544067	7,072899	-0,08150767	0,9450695	Zbed4
17398274	6,57888	6,439407	6,685134	6,426477	6,423679	6,608748	6,567807	6,486301333	-0,08150567	0,94507081	Ppm1l
17513311	7,846618	8,166439	8,111534	8,061998	7,969646	7,848466	8,0415303	7,960036667	-0,08149367	0,945078671	2310061C15Rik
17417306	6,546754	6,727881	6,489548	6,385705	6,514038	6,62003	6,588061	6,506591	-0,08147	0,945094175	Mob3c
17357918	3,655337	3,268341	3,675	3,460044	3,445463	3,4488	3,5328927	3,451435667	-0,081457	0,945102691	Olfr1471
17485702	5,632301	5,657005	5,9914	5,794422	5,67417	5,567746	5,7602353	5,678779333	-0,081456	0,945103346	Leng9
17248666	8,71946	8,581598	8,739977	8,52045	8,605172	8,671124	8,680345	8,598915333	-0,08142967	0,945120597	Rnf145
17447310	8,756432	8,67667	8,713354	8,671079	8,575524	8,655617	8,7154853	8,634073333	-0,081412	0,945132171	Lrpap1
17288859	5,688263	5,518101	5,52723	5,429465	5,57163	5,488299	5,5778647	5,496464667	-0,0814	0,945140032	A830082K12Rik
17306341	4,050066	3,618341	3,91166	3,82664	3,812872	3,69637	3,8600223	3,778627333	-0,081395	0,945143308	Olfr49

17408099	8,032134	7,995298	7,980536	7,87097	7,93937	7,953477	8,002656	7,921272333	-0,08138367	0,945150732	Polr3c
17299638	7,130113	7,22581	7,612163	7,049993	7,364395	7,309621	7,3226953	7,241336333	-0,081359	0,945166892	Ear6
17530294	10,6056	10,76672	10,62771	10,42883	10,55405	10,77312	10,666677	10,58533333	-0,08134333	0,945177156	---
17261156	4,584192	4,981827	4,121347	4,604906	4,520995	4,317476	4,5624553	4,481125667	-0,08132967	0,94518611	---
17284201	8,773667	9,00239	9,054562	8,863888	8,855321	8,867442	8,9435397	8,862217	-0,08132267	0,945190696	Akt1
17539040	3,777755	3,741465	3,405129	3,626303	3,578334	3,475783	3,6414497	3,56014	-0,08130967	0,945199213	Samt4
17481173	3,531288	3,346237	3,503046	3,391478	3,54209	3,203176	3,4601903	3,378914667	-0,08127567	0,945221489	Olfr585
17233145	2,858603	3,050578	2,987342	2,947861	2,866661	2,838185	2,9655077	2,884235667	-0,081272	0,945223891	---
17370360	3,453524	3,348995	3,290171	3,308607	3,237102	3,303208	3,36423	3,282972333	-0,08125767	0,945233282	Olfr357
17337219	4,016677	3,732865	4,040935	3,814386	3,917944	3,814386	3,930159	3,848905333	-0,08125367	0,945235903	---
17473463	5,252122	4,905819	4,87825	4,70902	5,215075	4,868365	5,0120637	4,93082	-0,08124367	0,945242455	Ccdc106
17490196	5,350661	5,314409	5,497304	5,32185	5,361156	5,23564	5,387458	5,306215333	-0,08124267	0,94524311	Acpt
17202417	5,405851	5,441621	5,460238	5,349794	5,160807	5,553407	5,4359033	5,354669333	-0,081234	0,945248788	---
17279993	5,474345	5,518032	5,675097	5,462481	5,352273	5,609027	5,5558247	5,474593667	-0,081231	0,945250754	4921511I17Rik
17431749	8,260802	8,086243	8,150238	7,981768	8,040783	8,23104	8,165761	8,084530333	-0,08123067	0,945250972	Pink1
17498208	6,153502	5,874824	6,22069	5,973867	5,840808	6,190658	6,0830053	6,001777667	-0,08122767	0,945252938	Trpm5
17485098	5,727846	5,696104	5,582728	5,636786	5,545809	5,580402	5,6688927	5,587665667	-0,081227	0,945253375	Muc5b
17254047	6,500739	6,822582	6,828036	6,624139	6,527058	6,756488	6,717119	6,635895	-0,081224	0,94525534	Ccl7
17478587	5,879304	6,049737	5,905895	5,826449	5,987015	5,777841	5,9449787	5,863768333	-0,08121033	0,945264295	A230056P14Rik
17539125	8,773639	8,852407	8,845968	8,741615	8,700394	8,786389	8,8240047	8,742799333	-0,08120533	0,945267571	Rps6ka3
17464208	4,258723	4,096846	4,331926	4,156356	4,235949	4,051586	4,229165	4,147963667	-0,08120133	0,945270192	---
17449553	9,801744	9,863446	9,504531	9,293522	9,416843	10,21576	9,7232403	9,642041667	-0,08119867	0,945271939	---
17249636	4,842022	4,842425	4,930833	4,752784	4,796147	4,822794	4,87176	4,790575	-0,081185	0,945280894	Csf2
17547589	5,496026	5,222835	5,48942	5,545094	5,218263	5,20139	5,4027603	5,321582333	-0,081178	0,94528548	Arhgap27 // Arhgap27
17415430	3,402416	3,699175	3,529947	3,745765	3,318092	3,324153	3,543846	3,46267	-0,081176	0,94528679	---
17303191	3,748444	4,094902	3,896408	3,828148	3,944918	3,723199	3,9132513	3,832088333	-0,081163	0,945295308	---
17380888	6,648902	6,676501	6,723675	6,589905	6,645247	6,570439	6,683026	6,601863667	-0,08116233	0,945295745	---
17504541	7,320085	7,580235	7,630202	7,656806	7,38639	7,243839	7,510174	7,429011667	-0,08116233	0,945295745	Ces2e
17265247	6,653703	6,816345	6,75665	6,629912	6,626715	6,726625	6,7422327	6,661084	-0,08114867	0,9453047	Pelp1
17391135	3,496752	3,606795	3,50257	3,210388	3,727977	3,424316	3,5353723	3,454227	-0,08114533	0,945306884	---
17391270	6,35093	6,338885	6,665817	6,297776	6,410755	6,403697	6,4518773	6,370742667	-0,08113467	0,945313873	Kcnip3
17341269	4,789343	4,847594	4,998369	4,806036	4,808639	4,777278	4,8784353	4,797317667	-0,08111767	0,945325013	Has1
17243429	5,454107	5,33714	5,211267	5,427296	5,25661	5,075309	5,3341713	5,253071667	-0,08109967	0,945336807	BC025920 // BC025920
17521262	3,420363	3,561055	3,847469	3,483934	3,449502	3,652207	3,609629	3,528547667	-0,08108133	0,94534882	---
17319736	4,86331	5,27385	5,247139	5,004259	5,079879	5,05692	5,1280997	5,047019333	-0,08108033	0,945349476	Mir3080 // Mir3080
17516515	6,144238	6,340657	6,589346	6,215227	6,361775	6,254	6,3580803	6,277000667	-0,08107967	0,945349912	Rnf26 // Rnf26
17201133	9,124509	9,190392	9,131192	8,963101	8,849122	9,390635	9,1486977	9,067619333	-0,08107833	0,945350786	---
17439131	8,527094	8,364587	8,476736	8,438939	8,471003	8,215306	8,456139	8,375082667	-0,08105633	0,945365202	Stbd1
17383858	6,352415	6,357445	6,528355	6,372282	6,231003	6,391818	6,4127383	6,331701	-0,08103733	0,945377652	Dnm1
17216527	4,324767	4,637346	4,255046	4,215074	4,311633	4,447341	4,4057197	4,324682667	-0,081037	0,945377871	Cntnap5a
17410722	4,700767	4,695279	4,931726	4,691606	4,782344	4,610751	4,775924	4,694900333	-0,08102367	0,945386608	Gm15688
17537773	3,297373	3,094642	3,152063	3,150682	3,172669	2,977683	3,1813593	3,100344667	-0,08101467	0,945392506	Pramel3
17494588	7,907149	8,097656	7,931425	7,935685	7,856741	7,900787	7,9787433	7,897737667	-0,08100567	0,945398403	Taf10
17285819	13,02872	13,03966	13,08911	12,92261	12,93251	13,05941	13,052497	12,97151	-0,08098667	0,945410854	Hist1h2af
17438480	3,629877	3,496011	3,382321	3,395054	3,524472	3,345735	3,5027363	3,421753667	-0,08098267	0,945413475	---
17221930	3,027269	2,94943	2,875683	2,82482	3,026634	2,757992	2,950794	2,869815333	-0,08097867	0,945416097	---
17378000	4,067841	3,903882	4,029497	3,806134	4,068514	3,883645	4,0004067	3,919431	-0,08097567	0,945418063	Efcab8
17371272	3,808833	3,744323	3,764127	3,786164	3,673074	3,615124	3,7724277	3,691454	-0,08097367	0,945419373	---
17208971	7,28264	7,039289	7,418369	7,546629	7,094914	6,855879	7,246766	7,165807333	-0,08095867	0,945429203	---
17209891	5,65833	5,401756	5,875621	5,794108	5,218194	5,680537	5,6452357	5,564279667	-0,080956	0,945430951	---
17509022	5,010178	4,632951	5,001981	4,759041	4,775239	4,867982	4,8817033	4,800754	-0,08094933	0,945435319	Gm2366
17523531	7,13804	7,301066	7,263061	6,857997	7,364012	7,237327	7,2340557	7,153112	-0,08094367	0,945439033	Tmem42
17419389	6,537188	6,953101	6,829757	6,692894	6,690673	6,693674	6,7733487	6,692413667	-0,080935	0,945444712	Snhg12
17484056	2,949886	3,02323	3,134866	2,973156	3,075559	2,816469	3,035994	2,955061333	-0,08093267	0,945446242	---
17361164	8,021626	8,093303	8,241561	8,230665	7,94289	7,940144	8,11883	8,037899667	-0,08093033	0,945447771	Rad9
17358891	8,243591	8,375556	8,300839	8,14506	8,182417	8,349771	8,306662	8,225749333	-0,08091267	0,945459348	Pcgf5
17209053	3,552599	5,052777	5,644813	5,069766	4,640932	4,296789	4,750063	4,669162333	-0,08090067	0,945467212	---
17201781	4,868377	5,255997	5,495006	5,323982	5,051321	5,001385	5,20646	5,125562667	-0,08089733	0,945469397	---
17201957	4,868377	5,255997	5,495006	5,323982	5,051321	5,001385	5,20646	5,125562667	-0,08089733	0,945469397	---
17422510	6,775785	6,716384	6,99281	6,608543	6,749549	6,884206	6,8283263	6,747432667	-0,08089367	0,9454718	2610002J02Rik
17525488	3,589932	3,372807	3,625129	3,405143	3,386967	3,5531	3,5292893	3,448403333	-0,080886	0,945476824	Gm17689
17532275	5,846137	6,25232	5,675401	5,610246	5,883951	6,037117	5,9246193	5,843771333	-0,080848	0,945501728	---
17460506	8,396827	8,396924	8,192689	8,303637	8,255999	8,184283	8,3288133	8,247973	-0,08084033	0,945506753	Gfpt1
17293521	7,283593	7,078828	7,160943	7,07402	7,063649	7,14318	7,1744547	7,093616333	-0,08083833	0,945508063	LOC100861712 // LOC100861712
17395189	4,618305	4,910915	4,755849	4,418642	4,888386	4,735528	4,7616897	4,680852	-0,08083767	0,9455085	Gm14393
17489202	6,373884	6,134566	6,288975	6,409751	6,015474	6,129691	6,2658083	6,184972	-0,08083633	0,945509374	Zbtb32
17227384	5,108043	5,380093	5,598964	5,24229	5,319675	5,282637	5,3623667	5,281534	-0,08083267	0,945511777	Pkp1
17202337	6,275095	6,051148	5,810939	6,027667	5,669628	6,197394	6,0457273	5,964896333	-0,080831	0,945512869	---
17223747	4,066012	3,923877	3,958782	3,937983	3,835018	3,933183	3,9828903	3,902061333	-0,080829	0,94551418	Mdh1b
17374077	5,533861	5,484986	5,638086	5,450935	5,480568	5,482968	5,552311	5,471490333	-0,08082067	0,945519642	Bdnf
17510701	9,256285	9,396274	9,216034	9,202518	9,296448	9,127191	9,289531	9,208719	-0,080812	0,945525322	Rbmxl1
17358051	4,947759	4,910575	5,01459	5,018922	4,788856	4,822782	4,9576413	4,876853333	-0,080788	0,945541051	Trpm6
17378575	4,794378	5,070575	4,931098	4,890189	4,756068	4,907443	4,932017	4,851233333	-0,08078367	0,945543891	4921517L17Rik
17457519	7,077687	6,988181	7,364072	7,125213	6,885424	7,176969	7,1433133	7,062535333	-0,080778	0,945547605	Dennd2a
17477178	5,810107	5,662931	5,721687	5,701841	5,712044	5,538523	5,731575	5,650802667	-0,08077233	0,945551319	Ctu1 // Ctu1
17517532	5,077866	5,005123	5,065486	4,786734	5,123772	4,995656	5,0494917	4,968720667	-0,080771	0,945552193	Isl2
17265783	4,968372	4,063631	4,137836	4,459326	3,873947	4,594271	4,3899463	4,309181333	-0,080765	0,945556125	Olfr385
17290041	4,480924	4,164223	4,112525	4,25224	4,085926	4,177216	4,2525573	4,171794	-0,08076333	0,945557218	---
17471804	3,102518	3,136768	2,834175	2,942259	3,141637	2,747374	3,024487	2,943756667	-0,08073033	0,945578847	Gm17776
17250193	4,90115	4,932757	5,072346	4,791057	4,79832	5,074735	4,968751	4,888037333	-0,08071367	0,94558977	Obscn
17538356	6,784458	6,861897	6,83375	6,737647	6,773295	6,727041	6,8267017	6,745994333	-0,08070733	0,945593921	Alg13
17352710	3,990763	4,038819	4,318073	4,251239	4,16047	3,693868	4,115885	4,035192333	-0,08069267	0,945603535	Cables1 // Cables1
17523081	6,093417	6,134878	6,360476	5,856697	6,293775	6,196239	6,196257	6,115570333	-0,08068667	0,945607467	Acvr2b
17485320	5,395526	5,349336	5,845189	5,408072	5,408911	5,531042	5,530017	5,449341667	-0,08067533	0,945614896	Kcnq1
17519579	9,940758	9,885729	9,974001	9,792906	9,862075	9,903482	9,933496	9,852821	-0,080675	0,945615114	Gclc
17256365	7,459811	7,537259	7,611938	7,547337	7,369734	7,449924	7,536336	7,455665	-0,080671	0,945617736	Fkbp10
17230023	4,64114	5,092793	4,295516	4,577217	4,6429	4,567326	4,676483	4,595814333	-0,08066867	0,945619265	Pydc4
17216219	3,865241	3,731222	3,7898	3,550068	3,79604	3,798193	3,795421	3,714767	-0,080654	0,945628879	1810006J02Rik
17393769	3,024969	3,140893	2,786022	2,933753	2,740652	3,035633	2,9839613	2,903346	-0,08061533	0,945654224	---
17460877	3,451443	2,859797	3,336478	3,285716	3,064856	3,055327	3,215906	3,135299667	-0,08060633	0,945660123	---
17426855	5,749767	5,696331	5,792226	5,746665	5,592573	5,657282	5,746108	5,665506667	-0,08060133	0,9456634	Ttc39b
17373616	3,897973	3,70893	3,829498	3,778358	3,71668	3,69962	3,8121337	3,731552667	-0,080581	0,945676729	Lrrc4c
17256162	7,978388	7,968489	8,0428	7,951287	7,935931	7,86074	7,996559	7,915986	-0,080573	0,945681972	Msl1
17226669	3,749532	4,003935	3,952538	3,809748	3,849721	3,804821	3,9020017	3,82143	-0,08057167	0,945682846	C4bp-ps1
17515132	7,960766	7,998895	7,979033	7,877082	8,011224	7,808707	7,9795647	7,899004333	-0,08056033	0,945690275	Atg4d
17471059	11,07925	11,18142	11,16094	11,0343	11,11203	11,03361	11,140537	11,05998	-0,08055667	0,945692679	Scarna10
17231757	3,453969	3,656484	3,180969	3,49647	3,269905	3,283386	3,430474	3,349920333	-0,08055367	0,945694645	---
17547759	3,97626	3,694353	3,653164	3,778656	3,749649	3,553817	3,7745923	3,694040667	-0,08055167	0,945695956	LOC100862265
17289843	4,434881	4,346232	4,330499	4,424272	4,136735	4,308971	4,3705373	4,289992667	-0,08054467	0,945700545	---
17548777	12,74497	12,6871	12,70548	12,68231	12,6352	12,57844	12,712517	12,63198333	-0,08053333	0,945707974	Eif1 // Eif1
17470661	3,210337	3,423764	3,178717	3,140479	3,354518	3,076241	3,2709393	3,190412667	-0,08052667	0,945712344	---
17425464	6,291823	6,431473	6,09928	5,744341	6,477031	6,359625	6,274192	6,193665667	-0,08052633	0,945712563	---
17337224	5,369713	5,316052	5,536439	5,109533	5,617943	5,253275	5,4074013	5,326917	-0,08048433	0,945740095	4833427F10Rik
17499409	5,039496	4,965872	5,049618	5,070988	4,912877	4,829694	5,0183287	4,937853	-0,08047567	0,945745776	---
17217789	8,029943	7,908751	8,009932	7,75032	7,841291	8,115631	7,9828753	7,902414	-0,08046133	0,945755172	Kif14
17229050	4,691893	4,675676	5,195695	4,688329	4,878436	4,755198	4,8544213	4,773987667	-0,08043367	0,945773309	Fmo6
17331907	3,699648	3,748789	3,662632	3,561443	3,770057	3,538302	3,7036897	3,623267333	-0,08042233	0,945780739	Krtap8-1
17201619	2,991369	2,948718	3,008762	2,774955	2,818468	3,114182	2,9829497	2,902535	-0,08041467	0,945785765	---
17312485	7,473652	7,269151	7,411741	7,457474	7,305462	7,150378	7,384848	7,304438	-0,08041	0,945788824	Hsf1
17219435	7,287067	7,281406	6,918217	7,188129	6,872924	7,184412	7,16223	7,081821667	-0,08040833	0,945789917	Slamf6
17288278	8,479344	8,779292	8,566956	8,638225	8,524171	8,421975	8,6085307	8,528123667	-0,080407	0,945790791	Ptdss1
17364588	4,716239	4,623702	4,590637	4,522683	4,545752	4,620925	4,643526	4,56312	-0,080406	0,945791447	Opalin
17548950	5,125146	4,923514	5,229213	4,946855	5,084884	5,004944	5,0926243	5,012227667	-0,08039667	0,945797565	Nenf // Nenf
17544595	3,869478	3,721574	3,74435	3,776878	3,522121	3,795218	3,7784673	3,698072333	-0,080395	0,945798658	3632454L22Rik
17420841	5,45147	5,460614	5,593506	5,379428	5,333705	5,55132	5,5018633	5,421484333	-0,080379	0,945809147	---
17520881	5,830558	5,663104	5,769162	5,720503	5,553009	5,748177	5,7542747	5,673896333	-0,08037833	0,945809584	Gm5627
17450180	8,184577	8,15692	8,027205	7,999948	8,159637	7,968032	8,1229007	8,042539	-0,08036167	0,945820511	Fam175a
17503948	7,769977	7,859698	7,683572	7,592718	7,831361	7,648129	7,7710823	7,690736	-0,08034633	0,945830563	Nup93
17479540	8,254232	8,452388	8,17034	8,127726	8,381188	8,127027	8,29232	8,211980333	-0,08033967	0,945834934	Ngrn
17482508	4,927381	5,121755	5,236207	4,934542	5,028653	5,081146	5,0951143	5,014780333	-0,080334	0,945838649	Vwa3a
17220214	6,152332	5,974812	5,982574	5,976425	6,155184	5,737117	6,0365727	5,956242	-0,08033067	0,945840835	Lefty1
17303916	7,268776	7,136091	7,165126	7,08946	7,066525	7,173091	7,1899977	7,109692	-0,08030567	0,945857225	Camk2g
17494418	3,034981	3,207292	3,256014	3,15416	3,01244	3,09081	3,1660957	3,085803333	-0,08029233	0,945865966	Trim30e-ps1
17450877	3,382853	3,370558	3,363842	3,253243	3,270892	3,352276	3,3724177	3,292137	-0,08028067	0,945873615	Vmn2r11
17353358	8,775444	9,088326	8,838197	8,8833	8,731516	8,846322	8,9006557	8,820379333	-0,08027633	0,945876457	Stard4
17535473	3,545877	3,958166	3,892503	3,79265	3,693345	3,669733	3,7988487	3,718576	-0,08027267	0,94587886	Pnma3 // Pnma3
17421384	8,117071	8,157992	8,061526	8,062801	8,051488	7,981494	8,1121963	8,031927667	-0,08026867	0,945881483	1700029I01Rik
17305719	8,530289	8,577902	8,629622	8,516769	8,583718	8,396541	8,579271	8,499009333	-0,08026167	0,945886072	Cnih
17341024	3,668841	3,576155	3,854947	3,661437	3,640532	3,557195	3,699981	3,619721333	-0,08025967	0,945887384	---
17282789	5,774376	5,918377	5,980262	5,817815	5,780813	5,833653	5,891005	5,810760333	-0,08024467	0,945897218	Ngb
17520979	4,155241	4,035759	4,025506	3,97268	3,934189	4,068908	4,0721687	3,991925667	-0,080243	0,945898311	4932413F04Rik
17439563	7,498652	7,504542	7,351799	7,371599	7,247117	7,495551	7,4516643	7,371422333	-0,080242	0,945898967	Enoph1
17361470	5,940589	5,937213	5,754838	5,705563	5,91162	5,774752	5,8775467	5,797311667	-0,080235	0,945903556	Klc2
17428698	4,259296	4,540514	4,432287	4,33325	4,375548	4,282608	4,410699	4,330468667	-0,08023033	0,945906616	AV051173

17494719	4,378641	4,307004	4,421342	4,134734	4,356777	4,374817	4,3689957	4,288776	-0,08021967	0,94591361	Olfr711
17323070	7,199886	7,712104	5,762578	5,310339	7,674934	7,44864	6,8915227	6,811304333	-0,08021833	0,945914484	---
17448752	8,730261	8,467694	8,503249	8,429847	8,765496	8,265326	8,567068	8,486889667	-0,08017833	0,945940711	---
17208081	9,423162	8,955229	9,518368	9,516071	9,100719	9,039435	9,2989197	9,218741667	-0,080178	0,945940929	---
17529417	4,907401	4,57295	4,628843	4,619298	4,552969	4,696399	4,7030647	4,622888667	-0,080176	0,94594224	Snap91
17548170	4,534001	4,629548	4,785377	4,596123	4,568298	4,54398	4,649642	4,569467	-0,080175	0,945942896	Gm19501
17249895	3,412409	3,591978	3,99391	3,457297	3,634713	3,665778	3,666099	3,585929333	-0,08016967	0,945946393	---
17300241	3,101826	3,047739	3,048167	2,957485	3,065144	2,934596	3,0659107	2,985741667	-0,080169	0,94594683	---
17234800	8,512551	8,753392	8,546969	8,506237	8,451019	8,615175	8,604304	8,524143667	-0,08016033	0,945952513	Lsm7
17315187	7,456302	7,572066	7,572916	7,488908	7,500371	7,371547	7,5337613	7,453608667	-0,08015267	0,94595754	9430023L20Rik
17495024	6,191095	6,12747	6,041791	6,138452	5,917663	6,063785	6,1201187	6,039966667	-0,080152	0,945957977	---
17202749	7,772702	7,431705	7,84157	7,671965	7,449064	7,684498	7,6819923	7,601842333	-0,08015	0,945959288	---
17406658	6,950976	7,611434	7,255736	6,998301	7,259659	7,319742	7,2727153	7,192567333	-0,080148	0,9459606	---
17449642	6,139546	6,763792	6,412866	6,216535	6,426095	6,433139	6,4387347	6,358589667	-0,080145	0,945962567	---
17414007	7,392076	7,537739	7,294305	7,334922	7,345277	7,303493	7,40804	7,327897333	-0,08014267	0,945964097	Hnrnpf // Hnrnpf
17240109	5,023313	5,193886	5,215335	5,302572	5,100467	4,789084	5,144178	5,064041	-0,080137	0,945967812	Nkain2
17548614	3,863925	3,803085	3,779369	3,752277	3,778688	3,675047	3,8154597	3,735337333	-0,08012233	0,945977429	Gm20115
17506353	6,114802	6,100839	5,927226	5,990984	5,920542	5,990984	6,0476223	5,967503333	-0,080119	0,945979615	Gm22
17348957	7,455159	7,5515	7,430005	7,189015	7,41152	7,595792	7,478888	7,398775667	-0,08011233	0,945983986	Zfp35
17277924	9,049129	9,068687	8,991583	8,835498	8,962775	9,070792	9,0364663	8,956355	-0,08011133	0,945984642	Calm3 // Calm2 // Calm1
17235077	9,428258	9,459274	9,53908	9,522182	9,371119	9,293001	9,4755373	9,395434	-0,08010333	0,945989888	Cnn2
17372862	3,686641	3,26288	3,535294	3,492025	3,506133	3,24638	3,4949383	3,414846	-0,08009233	0,9459971	Olfr153
17362050	5,852127	5,768052	5,865664	5,87652	5,652874	5,716173	5,8286143	5,748522333	-0,080092	0,945997319	Rps6ka4
17329298	5,458179	5,310308	5,193298	5,279886	5,441738	4,999917	5,320595	5,240513667	-0,08008133	0,946004313	Etv5
17341312	3,471874	3,336169	3,426442	3,359635	3,440449	3,194175	3,411495	3,331419667	-0,08007533	0,946008248	Vmn2r106
17415177	8,638481	8,586854	8,642662	8,556343	8,582582	8,488863	8,6226657	8,542596	-0,08006967	0,946011963	Rraga
17545516	4,740269	4,002609	4,063924	4,273738	3,944832	4,348053	4,268934	4,188874333	-0,08005967	0,946018521	---
17208399	5,859863	6,073215	6,210268	6,33123	5,4199	6,152088	6,047782	5,967739333	-0,08004267	0,946029668	---
17253839	9,686978	9,728683	9,811595	9,59308	9,609362	9,7847	9,7424187	9,662380667	-0,080038	0,946032728	Suz12
17334722	6,599819	6,594208	6,395121	6,476222	6,494977	6,377869	6,529716	6,449689333	-0,08002667	0,94604016	Rpusd1
17421665	8,642966	8,727962	8,612619	8,572908	8,542406	8,628195	8,6611823	8,581169667	-0,08001267	0,946049341	Exosc10
17528744	2,877661	3,017612	3,096603	2,84834	3,077572	2,825929	2,997292	2,917280333	-0,08001167	0,946049996	---
17269018	4,713746	4,915132	4,849104	4,719558	4,855698	4,662696	4,825994	4,745984	-0,08001	0,946051089	Krt25
17494503	7,132116	7,266473	7,296947	7,15514	7,139918	7,160482	7,2318453	7,151846667	-0,07999867	0,946058521	Prkcdbp
17413213	7,45315	7,45315	7,573059	7,475872	7,350931	7,412564	7,4931197	7,413122333	-0,07999733	0,946059395	Stoml2 // Stoml2
17300179	3,847115	3,677152	3,476528	3,512504	3,69659	3,551755	3,6669317	3,586949667	-0,079982	0,94606945	---
17484049	6,331022	6,233214	6,138006	6,139357	6,273812	6,049153	6,2340807	6,154107333	-0,07997333	0,946075134	Gpr26
17207601	7,881068	7,422546	6,809569	7,565221	7,082963	7,22514	7,371061	7,291108	-0,079953	0,946088468	---
17275576	7,819809	8,009264	7,76265	7,734559	7,824889	7,792419	7,8639077	7,783955667	-0,079952	0,946089124	Brms1l
17452139	5,839297	5,853759	6,047923	5,831387	5,749489	5,920302	5,9136597	5,833726	-0,07993367	0,946101146	Rph3a
17204633	6,123296	5,429463	5,831457	5,972727	5,921252	5,250437	5,7947387	5,714805333	-0,07993333	0,946101365	---
17337574	3,80939	3,731028	3,611981	3,611066	3,958308	3,343233	3,7174663	3,637535667	-0,07993067	0,946103114	Olfr109
17285696	4,401269	4,630422	4,439818	4,544187	4,16481	4,522748	4,490503	4,410581667	-0,07992133	0,946109234	Hist1h2bp
17357221	7,310678	8,385948	7,684511	7,881152	8,230215	7,030014	7,7937123	7,713793667	-0,07991867	0,946110983	---
17207905	4,533414	4,417756	4,928323	4,713939	4,116777	4,809109	4,6264977	4,546608333	-0,07988933	0,94613022	---
17214162	5,517251	5,653883	5,517704	5,509424	5,660484	5,279287	5,562946	5,483065	-0,079881	0,946135685	Gpbar1
17513181	6,175224	5,768486	6,279457	5,858613	6,368455	5,756487	6,074389	5,994518333	-0,07987067	0,946142462	Chst5
17313430	7,575519	7,421143	7,456102	7,47124	7,451707	7,29022	7,4842547	7,404389	-0,07986567	0,946145741	1700029P11Rik
17391241	3,777526	3,772772	3,903934	3,684383	3,664913	3,865363	3,8180773	3,738219667	-0,07985767	0,946150987	1810024B03Rik
17435505	4,313235	4,279268	4,32472	4,204781	4,15246	4,32044	4,305741	4,225893667	-0,07984733	0,946157764	Actr3b
17212406	7,810546	7,811358	7,491817	7,693302	7,600708	7,580171	7,7045737	7,624727	-0,07984667	0,946158202	Bivm
17359902	8,522259	8,730636	8,72739	8,590466	8,592021	8,55829	8,660095	8,580259	-0,079836	0,946165197	Nolc1
17290770	8,178568	8,130916	7,784803	7,882454	7,891731	8,080618	8,031429	7,951601	-0,079828	0,946170444	Mrpl32
17263087	7,024072	7,191802	7,265831	7,158689	6,951557	7,131991	7,1605683	7,080745667	-0,07982267	0,946173941	---
17389299	3,088494	3,152855	3,189692	3,224947	2,970222	2,996447	3,1436803	3,063872	-0,07980833	0,946183342	Olfr1309
17208063	6,203218	6,125648	5,666168	6,085236	5,618694	6,051773	5,9983447	5,918567667	-0,079777	0,946203892	---
17470301	9,649092	9,580444	9,273993	9,253025	9,38764	9,623563	9,5011763	9,421409333	-0,079767	0,94621045	Adipor2
17432145	5,169805	4,956285	5,132409	4,892217	4,878375	5,248642	5,0861663	5,006411333	-0,079755	0,946218321	---
17286181	3,506528	3,456756	3,589488	3,506528	3,602498	3,204521	3,5175907	3,437849	-0,07974167	0,946227066	Prl8a2
17261896	4,358807	4,36798	4,416757	4,178369	4,382195	4,343828	4,3811813	4,301464	-0,07971733	0,946243026	Gabra1
17547585	3,822801	3,83188	3,638007	3,767519	3,589573	3,696447	3,7642293	3,684513	-0,07971633	0,946243681	LOC100504826
17491885	4,630661	4,319233	4,434969	4,450387	4,472498	4,222863	4,461621	4,381916	-0,079705	0,946251115	---
17236255	4,663054	4,158989	4,582486	4,422817	4,298316	4,444282	4,4681763	4,388471667	-0,07970467	0,946251334	1700113H08Rik
17310950	6,610316	6,702657	6,465536	6,162326	6,722058	6,655012	6,5928363	6,513132	-0,07970433	0,946251552	Pop1
17543767	3,691921	3,803723	3,613396	3,707291	3,397384	3,765273	3,7030133	3,623316	-0,07969733	0,946256143	4930519F16Rik
17205707	3,930268	5,17515	4,848553	4,447147	4,539073	4,728662	4,6513237	4,571627333	-0,07969633	0,946256799	---
17535722	4,384792	4,913193	4,864302	4,680645	4,486636	4,755955	4,7207623	4,641078667	-0,07968367	0,946265107	Opn1mw
17246131	7,681384	7,550914	7,682741	7,608445	7,464665	7,602911	7,6383463	7,558673667	-0,07967267	0,946272322	Obfc2b
17524311	4,526548	4,621201	4,693886	4,869491	4,519772	4,213368	4,6138783	4,534210333	-0,079668	0,946275383	Muc16
17539110	4,448963	4,587617	4,469662	4,159845	4,565846	4,541672	4,5020807	4,422454333	-0,07962633	0,946302713	Smpx
17271257	8,157001	7,903815	8,119658	8,146526	7,803044	7,992064	8,060158	7,980544667	-0,07961333	0,94631124	---
17380089	4,409626	4,978716	4,596023	4,601134	4,528192	4,61626	4,661455	4,581862	-0,079593	0,946324578	Mc3r
17367652	6,038171	6,527082	5,867113	6,040211	6,11629	6,037147	6,144122	6,064549333	-0,07957267	0,946337915	Il1f9
17346069	5,662536	6,209793	6,331187	6,035691	5,890457	6,03878	6,0678387	5,988309333	-0,07952933	0,94636634	Zfp119b // Zfp119b
17205355	5,881423	5,656068	5,894371	5,642088	5,775436	5,77586	5,8106207	5,731128	-0,07949267	0,946390393	---
17533746	5,291319	5,263302	5,376835	5,094606	5,197716	5,400661	5,3104853	5,230994333	-0,079491	0,946391486	Agtr2
17522842	5,109241	4,96088	5,008096	5,076707	4,856039	4,907018	5,0260723	4,946588	-0,07948433	0,946395859	---
17341392	4,154363	3,744396	3,490184	4,165556	3,60036	3,384583	3,7963143	3,716833	-0,07948133	0,946397827	Zfp947
17345581	6,311208	6,21573	6,326106	6,316289	6,245277	6,053042	6,284348	6,204869333	-0,07947867	0,946399577	Ptcra
17283014	9,186993	9,228073	9,169678	9,065151	9,117236	9,163939	9,1949147	9,115442	-0,07947267	0,946403513	Gtf2a1
17300839	7,853808	8,091111	7,870112	7,845714	7,89787	7,833057	7,9383437	7,858880333	-0,07946333	0,946409635	Mphosph8
17362028	6,047014	5,926315	6,160604	5,816239	6,108981	5,970465	6,0446443	5,965228333	-0,079416	0,946440686	Nrxn2 // Nrxn2
17530802	7,331484	7,483085	7,307179	7,185405	7,277373	7,420727	7,373916	7,294501667	-0,07941433	0,94644178	Rbm15b
17419469	8,232841	8,290119	8,213629	8,041915	8,244978	8,211511	8,2455297	8,166134667	-0,079395	0,946454463	Rpa2
17510856	7,354307	7,481541	6,98641	7,145959	7,284513	7,153638	7,274086	7,194703333	-0,07938267	0,946462554	Il15
17208191	6,423637	6,242718	6,692287	6,758859	5,995244	6,366406	6,4528807	6,373503	-0,07937767	0,946465834	---
17374972	6,421175	6,662006	6,560141	6,575315	6,429427	6,400454	6,547774	6,468398667	-0,07937533	0,946467365	Jmjd7
17232119	3,900461	3,866824	4,144279	3,950976	3,860288	3,862179	3,9705213	3,891147667	-0,07937367	0,946468458	Slc2a12
17430304	4,661961	4,49395	4,606341	4,579018	4,571956	4,373191	4,5874173	4,508055	-0,07936233	0,946475894	1700086P04Rik
17332728	4,769813	4,92556	4,792761	4,94037	4,459949	4,849741	4,829378	4,75002	-0,079358	0,946478737	Cldn20
17265917	7,558547	7,513049	7,606628	7,318718	7,56952	7,551913	7,559408	7,480050333	-0,07935767	0,946478955	Dph1
17497276	4,600654	4,300284	4,436375	4,363051	4,356596	4,379596	4,445771	4,366414333	-0,07935667	0,946479611	---
17448467	6,178447	6,146035	6,11077	5,966507	6,288587	5,942102	6,145084	6,065732	-0,079352	0,946482673	Bend4
17260798	4,842747	4,736814	4,957393	4,848959	4,706186	4,743783	4,8456513	4,766309333	-0,079342	0,946489233	---
17442187	7,927078	7,926299	7,997275	7,772856	7,870143	7,969642	7,9502173	7,870880333	-0,079337	0,946492514	Tmem120b
17290398	10,22461	10,42582	10,16027	10,02717	10,29127	10,25426	10,270233	10,1909	-0,07933333	0,946494919	Gtpbp4
17271005	9,636576	9,514233	9,612283	9,501378	9,500787	9,523006	9,5876973	9,508390333	-0,079307	0,946512196	Smurf2
17280431	6,383159	6,375452	6,420721	6,409949	6,276038	6,255433	6,3931107	6,313806667	-0,079304	0,946514164	Tpo
17372840	3,94831	3,747976	3,572154	3,794681	3,783477	3,45246	3,7561467	3,676872667	-0,079274	0,946533846	Olfr1121
17439585	5,594923	6,204437	6,033852	6,008259	5,559388	6,02778	5,944404	5,865142333	-0,07926167	0,946541938	2310034O05Rik // 2310034O05Rik
17530137	5,861973	5,216059	5,366775	5,403666	5,336242	5,467115	5,4816023	5,402341	-0,07926133	0,946542157	Tmem22
17290629	9,09509	9,231836	9,070133	9,056494	9,127827	8,974982	9,132353	9,053101	-0,079252	0,94654828	Lgals8
17256513	5,773159	5,789795	5,906734	5,817842	5,710563	5,703549	5,8232293	5,743984667	-0,07924467	0,946553092	Hsd17b1
17372242	7,884674	8,068236	7,754996	7,948494	7,742729	7,778982	7,9026353	7,823401667	-0,07923367	0,946560309	Plekha3
17392627	6,590804	6,702666	6,640719	6,46201	6,656101	6,5784	6,6447297	6,565503667	-0,079226	0,946565339	Gm14133
17232929	10,31168	10,22604	10,37488	10,159	10,24883	10,26711	10,3042	10,22498	-0,07922	0,946569276	Snx3
17491728	4,311777	4,171932	4,243714	4,276901	4,104616	4,108265	4,2424743	4,163260667	-0,07921367	0,946573431	Mir344
17384826	4,808143	4,820878	4,674818	4,608086	4,793771	4,664354	4,7679463	4,688737	-0,07920933	0,946576274	Lrp1b
17290815	8,455948	8,509303	8,26558	8,172753	8,406254	8,414197	8,410277	8,331068	-0,079209	0,946576493	Rala
17494528	12,61167	12,95578	12,55873	12,59459	12,70696	12,58706	12,708727	12,62953667	-0,07919	0,946588959	Hpx
17350258	3,020767	3,110096	3,084008	2,92816	3,155282	2,893887	3,0716237	2,992443	-0,07918067	0,946595083	---
17530141	9,137163	8,988747	9,061103	9,023299	9,000061	8,926164	9,0623377	8,983174667	-0,079163	0,946606675	Pccb
17426636	3,673503	3,305239	3,279821	3,393638	3,480945	3,146543	3,419521	3,340375333	-0,07914567	0,946618048	---
17250129	6,495065	6,695265	6,7218	6,389057	6,491492	6,794149	6,6373767	6,558232667	-0,079144	0,946619141	2810021J22Rik
17440335	4,409786	3,618153	3,356521	3,615837	3,53904	3,992264	3,79482	3,715713667	-0,07910633	0,946643857	Vmn2r17
17473570	4,70543	4,050398	4,292678	4,345191	4,348277	4,117737	4,349502	4,270401667	-0,07910033	0,946647794	Gm6792
17235029	6,520111	6,262299	6,225819	6,19948	6,359034	6,212416	6,3360763	6,256976667	-0,07909967	0,946648231	Kiss1r
17264884	5,286025	6,059847	5,592834	5,621994	5,489473	5,589942	5,6462353	5,567136333	-0,079099	0,946648668	BC096441
17296595	5,709403	5,383186	5,115653	5,468239	5,262071	5,240651	5,4027473	5,323653667	-0,07909367	0,946652168	D830030K20Rik
17203417	7,877403	7,335426	7,90617	7,814357	7,41454	7,652824	7,706333	7,627240333	-0,07909267	0,946652824	---
17371053	3,598579	3,699841	3,924389	3,690678	3,841439	3,453487	3,7409363	3,661868	-0,07906833	0,946668791	---
17510735	6,88943	6,907598	6,991343	6,684099	6,96506	6,902028	6,929457	6,850395667	-0,07906133	0,946673384	Smad1
17413550	5,39435	5,204203	5,492548	5,261144	5,316283	5,276493	5,3637003	5,28464	-0,07906033	0,946674041	---
17263877	7,195168	7,110538	6,877737	6,837191	7,050013	7,059095	7,0611477	6,982099667	-0,079048	0,946682134	Prpsap2
17368920	7,125696	7,178187	7,002965	6,969868	7,218633	6,881236	7,1022827	7,023245667	-0,079037	0,946689352	Urm1
17339215	4,080804	3,990749	3,737048	3,85637	3,82648	3,888674	3,9362003	3,857174667	-0,07902567	0,946696789	LOC433134
17472180	5,173539	5,148055	5,299566	5,105461	5,114364	5,164289	5,2070533	5,128038	-0,07901533	0,946703569	C030030A07Rik
17232910	3,273573	3,374415	4,403801	3,736367	3,403802	3,674581	3,6839297	3,604916667	-0,079013	0,946705101	Mir3473b // Mir3473b
17447045	7,797448	7,544489	7,700073	7,638508	7,742132	7,424356	7,68067	7,601665333	-0,07900467	0,946710569	Pisd
17448682	4,306673	4,311925	4,337761	4,373683	4,229361	4,116302	4,3187863	4,239782	-0,07900433	0,946710788	Cnga1
17421242	3,049627	3,103741	3,157486	3,034494	2,985717	3,053682	3,103618	3,024631	-0,078987	0,946722162	Gm13103
17398912	4,543391	4,244714	4,101912	4,103993	4,333972	4,215158	4,2966723	4,217707667	-0,07896467	0,946736818	---
17382841	6,02278	6,159743	6,696218	6,579661	5,671858	6,390338	6,2929137	6,213952333	-0,07896133	0,946739005	C330006A16Rik
17210125	5,177156	5,900676	5,666248	5,593597	5,33877	5,574848	5,58136	5,502405	-0,078955	0,946743161	---

17371265	2,86968	2,855978	2,797147	2,718955	2,835685	2,731318	2,840935	2,761986	-0,078949	0,946747099	---
17511318	10,70244	10,70789	10,65912	10,53497	10,63698	10,66068	10,689817	10,61087667	-0,07894	0,946753005	Vps35
17202371	7,45193	7,196796	7,785297	7,191014	7,613985	7,392221	7,4780077	7,399073333	-0,07893433	0,946756723	---
17256655	5,320968	5,218196	5,386449	5,15319	5,334212	5,201413	5,3085377	5,229605	-0,07893267	0,946757817	Becn1
17495340	8,514951	8,683857	8,319712	8,331224	8,364244	8,586275	8,5061733	8,427247667	-0,07892567	0,946762411	Sox6
17219554	5,858942	5,597691	5,900991	5,661151	5,81063	5,649089	5,7858747	5,706956667	-0,078918	0,946767442	Igsf9
17225224	5,714458	5,894894	5,757261	5,621477	5,764621	5,743764	5,788871	5,709954	-0,078917	0,946768098	Ecel1
17344866	3,375338	3,215977	3,281733	3,414239	3,049379	3,172712	3,291016	3,21211	-0,078906	0,946775317	Olfr102
17205981	6,51812	6,478787	6,833165	6,793832	5,839296	6,960254	6,610024	6,531127333	-0,07889667	0,946781442	---
17220974	7,253873	7,205637	7,312154	7,096222	6,938101	7,500704	7,2572213	7,178342333	-0,078879	0,946793036	Plxna2
17324356	11,97819	12,18887	11,92756	11,99368	11,92029	11,94402	12,03154	11,95266333	-0,07887667	0,946794567	Kng1
17266629	8,22147	8,340342	8,219838	8,136442	8,212399	8,196209	8,26055	8,181683333	-0,07886667	0,94680113	Utp6
17363598	8,336774	8,229736	8,432825	8,250416	8,242373	8,270041	8,3331117	8,254276667	-0,078835	0,946821912	Fxn
17538807	4,255931	5,36541	5,040438	5,096822	3,018262	6,310274	4,8872597	4,808452667	-0,078807	0,946840289	---
17308456	9,750744	9,85931	9,894851	9,380869	10,02909	9,858533	9,8349683	9,756164	-0,07880433	0,946842039	---
17239687	5,850079	5,58047	5,573452	5,564697	5,536904	5,666001	5,6680003	5,589200667	-0,07879967	0,946845101	Gm6846
17246963	6,677896	7,099219	6,910935	6,927691	6,973874	6,550148	6,8960167	6,817237667	-0,078779	0,946858665	---
17288172	3,055587	3,071681	3,120691	2,908965	2,933945	3,168764	3,082653	3,003891333	-0,07876167	0,946870041	4930458L03Rik
17270721	5,393131	5,606955	5,659615	5,525804	5,528459	5,369326	5,5532337	5,474529667	-0,078704	0,94690789	Dcaf7 // Dcaf7
17314699	5,441418	5,157879	5,477047	5,048972	5,189325	5,601951	5,3587813	5,280082667	-0,07869867	0,94691139	Prph
17448997	5,370039	5,205591	5,196909	5,207561	5,060111	5,268901	5,257513	5,178857667	-0,07865533	0,946939833	Nmu
17501277	5,178043	5,164449	5,100404	5,29353	4,962208	4,951264	5,147632	5,069000667	-0,07863133	0,946955586	5033428I22Rik
17272926	5,709011	5,762025	5,875419	5,645953	5,788261	5,676397	5,7821517	5,703537	-0,07861467	0,946966525	Nptx1
17347533	8,142103	8,215014	8,040031	7,968339	8,116504	8,076462	8,1323827	8,053768333	-0,07861433	0,946966744	Sos1
17545135	5,373417	5,095158	5,227033	5,249566	4,991376	5,218893	5,2318693	5,153278333	-0,078591	0,94698206	Trpc5
17412793	4,563826	4,493526	4,867547	4,441283	4,925903	4,321945	4,641633	4,563043667	-0,07858933	0,946983154	Spink4
17506732	8,397893	8,396961	8,340164	8,215485	8,337561	8,346284	8,3783393	8,299776667	-0,07856267	0,947000658	Galnt2
17420422	6,073071	5,575628	5,915765	5,690937	5,918635	5,719278	5,8548213	5,776283333	-0,078538	0,94701685	2310026L22Rik
17539842	4,480212	4,409003	4,374345	4,517936	4,271733	4,238343	4,4211867	4,342670667	-0,078516	0,947031291	---
17337417	4,026099	3,548946	4,316557	4,023372	3,865118	3,767643	3,9638673	3,885377667	-0,07848967	0,947048577	---
17376441	4,193627	4,183487	3,730304	3,838096	4,064468	3,969396	4,035806	3,95732	-0,078486	0,947050984	---
17333169	5,933037	5,626708	5,862023	5,706167	5,699395	5,780748	5,807256	5,72877	-0,078486	0,947050984	1700010I14Rik
17525476	3,031034	3,070079	3,040121	2,826779	3,088405	2,990749	3,047078	2,968644333	-0,07843367	0,947085339	---
17204173	3,502463	4,875532	3,658941	4,212664	3,757764	3,831208	4,012312	3,933878667	-0,07843333	0,947085558	---
17529966	8,309051	8,424192	8,336117	8,291083	8,307586	8,235458	8,3564533	8,278042333	-0,078411	0,947100219	Mrps22
17373696	5,846581	5,925568	5,848001	5,65007	5,844602	5,89027	5,8733833	5,794980667	-0,07840267	0,94710569	Slc1a2
17427240	8,68117	8,790695	8,725754	8,631187	8,684361	8,646875	8,7325397	8,654141	-0,07839867	0,947108316	Plaa
17364007	3,233192	3,353311	3,189692	3,536116	3,048899	2,955995	3,2587317	3,180336667	-0,078395	0,947110723	---
17512119	2,959094	3,226557	2,853716	2,844364	3,018262	2,941576	3,0131223	2,934734	-0,07838833	0,947115099	---
17204007	3,030227	3,44886	3,617701	3,302374	3,288399	3,270921	3,365596	3,287231333	-0,07836467	0,947130636	---
17446213	4,084801	3,994832	4,177823	3,893951	4,050952	4,077459	4,0858187	4,007454	-0,07836467	0,947130636	Gbx1
17236821	4,085986	3,86815	3,747925	3,875808	3,765087	3,826098	3,900687	3,822331	-0,078356	0,947136326	Epyc
17341781	7,918598	7,977519	8,129106	7,954855	7,887774	7,947529	8,0084077	7,930052667	-0,078355	0,947136983	Ccnf
17525690	4,225013	3,676277	3,563426	3,649955	3,866749	3,713002	3,821572	3,743235333	-0,07833667	0,947149019	Olfr891
17257361	6,091565	6,466352	6,289069	6,097273	6,179667	6,335039	6,2823287	6,203993	-0,07833567	0,947149675	Mrc2
17424802	6,009523	5,509549	5,949401	5,830955	5,485463	5,917062	5,8228243	5,744493333	-0,078331	0,947152739	Olfr70
17284602	3,000703	3,426899	3,015781	3,34653	2,968087	2,893802	3,1477943	3,069473	-0,07832133	0,947159085	---
17423509	3,820901	3,6967	3,569881	3,676446	3,549693	3,626421	3,6958273	3,61752	-0,07830733	0,947168276	---
17537199	5,040331	4,677787	4,730576	4,949272	4,799383	4,465129	4,8162313	4,737928	-0,07830333	0,947170903	Gm6377
17488792	8,07786	8,125113	8,078703	7,996937	8,07499	7,974869	8,093892	8,015598667	-0,07829333	0,947177468	Kcnk6
17427108	9,330082	9,175677	9,076493	8,991587	9,173113	9,182749	9,194084	9,115816333	-0,07826767	0,947194319	Klhl9
17206239	6,804932	6,145717	6,601484	6,76945	6,348212	6,199672	6,5173777	6,439111333	-0,07826633	0,947195194	---
17209087	4,210738	3,939696	4,930834	4,118501	4,381765	4,346232	4,3604227	4,282166	-0,07825667	0,947201541	---
17523642	4,498209	4,18937	4,642416	4,466621	4,212293	4,416324	4,4433317	4,365079333	-0,07825233	0,947204386	Cxcr6
17389596	3,297877	3,406562	3,459713	3,311282	3,305913	3,312219	3,3880507	3,309804667	-0,078246	0,947208544	3110099E03Rik
17205681	4,003585	4,602025	5,187486	4,229935	4,189805	5,138644	4,5976987	4,519461333	-0,07823733	0,947214234	---
17204119	4,100362	4,8601	3,612304	4,477303	3,846084	4,01467	4,190922	4,112685667	-0,07823633	0,947214891	---
17266489	9,56703	9,616083	9,558601	9,60072	9,482819	9,423491	9,5805713	9,502343333	-0,078228	0,947220362	Tmem97
17435170	5,477994	5,500134	5,256211	5,31905	5,210773	5,469837	5,4114463	5,33322	-0,07822633	0,947221457	6030443J06Rik
17530755	7,235857	7,291951	7,454435	7,213256	7,305146	7,229197	7,3274143	7,249199667	-0,07821467	0,947229117	Rad54l2
17206219	5,693398	5,747092	5,672129	5,672605	5,482726	5,722667	5,7042063	5,625999333	-0,078207	0,94723415	---
17515884	7,44208	7,813995	7,443649	6,968349	7,801986	7,694797	7,5665747	7,488377333	-0,07819733	0,947240497	---
17301943	5,060476	4,873329	4,787009	4,782178	4,989631	4,714427	4,906938	4,828745333	-0,07819267	0,947243561	Gm10847
17220277	4,405798	4,176509	4,223736	4,239594	4,135558	4,196324	4,268681	4,190492	-0,078189	0,947245969	Dnahc14
17214269	4,614858	4,552556	4,723344	4,483817	4,645021	4,527394	4,6302527	4,552077333	-0,07817533	0,947254942	Plcd4
17432418	4,132054	3,879561	4,311262	3,855013	4,240539	3,992814	4,1076257	4,029455333	-0,07817033	0,947258225	---
17328543	4,011427	3,828336	3,707642	3,774655	3,927974	3,610293	3,849135	3,770974	-0,078161	0,947264353	4933404G15Rik
17430307	5,350022	5,20163	5,036526	5,365188	4,883753	5,104798	5,1960593	5,117913	-0,07814633	0,947273983	Hpca
17532953	7,62254	7,60598	7,568755	7,558974	7,478159	7,525749	7,5990917	7,520960667	-0,078131	0,947284051	Tfe3
17425525	4,825969	4,887721	4,668361	4,627664	4,812967	4,707041	4,794017	4,715890667	-0,07812633	0,947287115	6430704M03Rik
17339973	7,875753	8,016707	8,078738	7,615043	8,120759	8,001061	7,9903993	7,912287667	-0,07811167	0,947296746	Pkdcc
17379384	3,176046	3,154915	3,117199	2,955491	3,228872	3,029487	3,1493867	3,071283333	-0,07810333	0,947302217	Svs3a
17376541	7,071082	6,856554	6,972694	6,792872	6,892622	6,98056	6,9667767	6,888684667	-0,078092	0,947309659	Prnp
17397400	3,433795	3,255802	3,250514	3,121798	3,290009	3,294064	3,3133703	3,235290333	-0,07808	0,947317539	Gm20557
17461114	4,195158	4,292444	4,39738	4,309974	4,079928	4,26093	4,294994	4,216944	-0,07805	0,947337238	Fam19a1
17208445	6,284208	6,398339	6,701069	6,344724	6,653449	6,151312	6,4612053	6,383161667	-0,07804367	0,947341397	---
17290777	3,572838	3,51437	3,584439	3,575827	3,423836	3,437857	3,5572157	3,479173333	-0,07804233	0,947342272	4933412O06Rik
17264402	5,264642	5,058035	4,95718	4,981006	5,044735	5,020172	5,0932857	5,015304333	-0,07798133	0,947382328	---
17395108	6,792159	6,437297	6,526449	6,695502	6,355688	6,470771	6,5853017	6,507320333	-0,07798133	0,947382328	Ppp4r1l-ps
17257663	10,01731	10,00099	9,967212	9,874384	9,941161	9,936028	9,9951707	9,917191	-0,07797967	0,947383423	Psmd12
17272626	5,13718	5,184813	5,316026	4,975049	5,210763	5,218273	5,212673	5,134695	-0,077978	0,947384517	Dnahc17
17256066	6,744122	6,602538	6,84438	6,635748	6,760546	6,560822	6,7303467	6,652372	-0,07797467	0,947386706	Gm12
17235663	7,58479	7,847594	7,747202	7,688986	7,575616	7,68108	7,7265287	7,648560667	-0,077968	0,947391084	Pip5k1c
17390263	7,091723	7,213917	7,31845	6,973384	7,277914	7,138888	7,20803	7,130062	-0,077968	0,947391084	Cdan1
17415570	8,38464	8,454107	8,464392	8,380289	8,348342	8,340612	8,4343797	8,356414333	-0,07796533	0,947392835	Hook1
17471717	3,345515	3,050126	3,515354	3,340138	3,097262	3,239729	3,303665	3,225709667	-0,07795533	0,947399402	Klra11
17372388	4,696109	5,180147	4,840331	4,9005	5,034408	4,547832	4,905529	4,82758	-0,077949	0,947403561	Prdx6b
17543852	9,794916	9,765626	9,657434	9,637086	9,644918	9,702125	9,7393253	9,661376333	-0,077949	0,947403561	Abcb7
17272594	9,414969	9,259205	9,417695	9,457939	9,281472	9,118693	9,3639563	9,286034667	-0,07792167	0,947421511	Tk1
17304461	11,34252	11,44778	11,4541	11,4574	11,30354	11,2497	11,4148	11,33688	-0,07792	0,947422605	Itih1
17334922	6,238531	5,876369	6,20141	6,047191	5,821898	6,213493	6,1054367	6,027527333	-0,07790933	0,94742961	Neurl1b
17502609	4,132274	4,130088	4,17362	4,018053	4,167117	4,017165	4,1453273	4,067445	-0,07788233	0,947447342	1700007B14Rik
17238900	6,223991	6,089464	6,645618	6,397036	5,963963	6,36448	6,319691	6,241826333	-0,07786467	0,947458944	Syne1
17267209	7,43125	7,578734	7,595818	7,482583	7,497454	7,392174	7,5352673	7,457403667	-0,07786367	0,9474596	Brip1
17201455	6,745077	6,793561	6,360835	6,58383	6,473879	6,608258	6,6331577	6,555322333	-0,07783533	0,947478208	---
17547696	6,967262	7,276046	7,304724	7,329472	7,092747	6,892317	7,1826773	7,104845333	-0,077832	0,947480397	Polr2h // Polr2h
17287144	5,211813	5,200225	5,126355	5,177282	5,022415	5,105223	5,1794643	5,10164	-0,07782433	0,947485432	Dcun1d1
17521673	11,51968	11,41072	11,33867	11,24333	11,40503	11,38729	11,423023	11,34521667	-0,07780667	0,947497035	Rhoa
17282828	5,134204	5,154397	4,666396	5,134867	4,81465	4,772064	4,984999	4,907193667	-0,07780533	0,94749791	4933437F05Rik
17548504	8,349854	8,396276	8,468838	8,160501	8,427979	8,393152	8,4049893	8,327210667	-0,07777867	0,947515424	---
17312941	8,926494	8,706358	9,068986	8,819577	8,929644	8,719314	8,9006127	8,822845	-0,07776767	0,947522648	---
17204999	7,95285	7,729219	8,192838	7,971661	7,701078	7,968874	7,9583023	7,880537667	-0,07776467	0,947524619	---
17431586	2,951773	3,063457	3,062654	2,948606	3,0204	2,875596	3,0259613	2,948200667	-0,07776067	0,947527246	---
17358838	8,793329	8,704735	8,645178	8,563481	8,626884	8,719622	8,714414	8,636662333	-0,07775167	0,947533157	Kif20b
17395009	4,225339	4,039212	4,245557	4,046733	4,084826	4,145309	4,170036	4,092289333	-0,07774667	0,947536441	Cbln4
17232892	11,17092	11,16576	11,13803	11,03204	11,13682	11,07266	11,158237	11,08050667	-0,07773	0,947547387	Cd164
17276558	7,518753	7,668273	7,503496	7,469563	7,432487	7,555313	7,5635073	7,485787667	-0,07771967	0,947554174	Zbtb1
17394726	5,817616	5,869886	6,227272	5,638063	6,085292	5,958263	5,9715913	5,893872667	-0,07771867	0,947554831	---
17359623	6,079798	6,518045	6,424904	6,182	6,447775	6,159846	6,3409157	6,263207	-0,07770867	0,947561399	Cutc
17468957	5,550557	4,703321	5,361813	5,166355	5,170378	5,045846	5,2052303	5,127526333	-0,077704	0,947564464	---
17417946	5,643288	5,130922	5,457151	5,347889	5,284614	5,365746	5,4104537	5,332749667	-0,077704	0,947564464	Guca2a
17241409	10,54535	10,39387	10,41074	10,33821	10,38069	10,39804	10,449987	10,37231333	-0,07767333	0,947584606	Srgn
17344355	6,625525	6,409897	6,597379	6,618492	6,298433	6,482907	6,544267	6,466610667	-0,07765633	0,947595772	Vars2
17204573	3,387842	4,255931	3,61725	3,461071	4,165556	3,40143	3,7536743	3,676019	-0,07765533	0,947596429	---
17443353	5,014184	5,024605	5,342319	5,354951	5,070387	4,722812	5,127036	5,049383333	-0,07765267	0,94759818	Zp3
17494431	5,538506	5,059094	5,090282	4,94664	5,191442	5,316862	5,229294	5,151648	-0,077646	0,947602559	Dub3
17423854	8,123222	7,773532	7,924122	7,821027	7,910969	7,855972	7,940292	7,862656	-0,077636	0,947609128	Slc35a1
17382186	8,023037	8,043553	7,941236	7,933974	8,007049	7,833946	8,0026087	7,924989667	-0,077619	0,947620294	Spopl
17449516	7,878005	8,053961	7,721355	7,779036	7,734913	7,90653	7,8844403	7,806826333	-0,077614	0,947623578	Cox18
17211023	5,5094	5,638187	5,628834	5,758152	5,641501	5,143939	5,5921403	5,514530667	-0,07760967	0,947626424	3110035E14Rik
17235694	7,061865	6,793746	6,959915	6,887176	6,768416	6,92722	6,9385087	6,860937333	-0,07757133	0,947651604	Tbxa2r
17509001	3,53706	3,492188	3,290768	3,231061	3,631417	3,224964	3,4400053	3,362480667	-0,07752467	0,947682258	Adam26b
17372584	7,580817	7,558983	7,525158	7,456733	7,455059	7,520593	7,554986	7,477461667	-0,07752433	0,947682477	Med19
17537391	3,648806	3,392087	3,183271	3,458601	3,244758	3,288235	3,4080547	3,330531333	-0,07752333	0,947683134	---
17406179	4,61493	4,673189	4,675533	4,477939	4,648561	4,604615	4,6545507	4,577038333	-0,07751233	0,947690359	---
17476188	6,893943	6,571797	6,40525	6,178827	6,702217	6,757442	6,6236633	6,546162	-0,07750133	0,947697585	Zfp30
17529549	8,430383	8,606318	8,334848	8,265772	8,48739	8,385916	8,457183	8,379692667	-0,07749033	0,947704811	Snhg5
17462941	6,357538	6,454004	6,335551	6,111499	6,489171	6,314007	6,3823643	6,304892333	-0,077472	0,947716854	---
17424980	6,898735	7,100475	7,208944	7,024769	6,845477	7,105505	7,0693847	6,991917	-0,07746767	0,947719701	Tstd2
17464417	7,28993	7,380654	7,362689	7,059009	7,322358	7,419546	7,3444243	7,266971	-0,07745333	0,947729117	Klhdc5
17441453	4,687048	4,643794	4,790221	4,473189	4,764581	4,650968	4,707021	4,629579333	-0,07744167	0,947736781	Nos1
17283537	6,712254	7,077008	7,148861	6,688086	7,329963	6,687766	6,9793743	6,901938333	-0,077436	0,947740503	---
17210705	4,609404	4,59815	3,589144	4,448896	3,449429	4,666234	4,265566	4,188186333	-0,07737967	0,947777511	---
17549172	8,042537	7,741212	8,110079	7,850664	8,028923	7,782188	7,9646093	7,887258333	-0,077351	0,947796343	---

17529692	5,879607	6,192175	6,531882	6,193064	6,054424	6,12417	6,2012213	6,123886	-0,07733533	0,947806636	B430319G15Rik
17300217	3,445853	3,578585	3,715624	3,504284	3,571646	3,432225	3,5800207	3,502718333	-0,07730233	0,947828316	---
17544360	3,688495	3,929047	3,69495	3,715475	3,575984	3,78914	3,7708307	3,693533	-0,07729767	0,947831382	---
17368518	3,745094	3,779309	3,973599	3,73833	3,733729	3,794124	3,8326673	3,755394333	-0,077273	0,947847588	---
17221970	5,133843	5,241907	5,406272	4,992239	4,974476	5,58349	5,260674	5,183401667	-0,07727233	0,947848026	---
17359878	7,550564	7,863156	7,687888	7,565615	7,725434	7,578784	7,700536	7,623277667	-0,07725833	0,947857224	Pprc1
17222020	7,162269	7,171021	7,604993	7,151568	7,155582	7,399383	7,312761	7,235511	-0,07725	0,947862699	Bag2
17403423	7,693057	7,513299	7,634684	7,414889	7,631212	7,563253	7,61368	7,536451333	-0,07722867	0,947876715	Znhit6
17447510	9,708358	9,632471	9,716978	9,566431	9,508382	9,751344	9,6859357	9,608719	-0,07721667	0,947884599	Mrfap1
17205643	4,735517	5,442869	5,619149	4,769522	5,300159	5,496213	5,265845	5,188631333	-0,07721367	0,94788657	---
17304127	4,342348	4,708682	4,61405	4,477494	4,55718	4,398774	4,5550267	4,477816	-0,07721067	0,947888541	4930572O13Rik
17449073	7,79959	7,950414	7,813204	7,634931	7,757764	7,938891	7,8544027	7,777195333	-0,07720733	0,947890732	Noa1
17316350	4,802506	4,729093	4,374695	4,57748	4,520204	4,577096	4,6354313	4,55826	-0,07717133	0,947914385	1700084J12Rik
17449091	5,21224	5,052473	5,072658	5,072658	4,962289	5,070931	5,112457	5,035292667	-0,07716433	0,947918984	Pea15b
17452736	6,216194	6,091351	6,038583	5,982976	6,180987	5,950742	6,115376	6,038235	-0,077141	0,947934315	---
17547744	10,62984	10,54163	10,48719	10,32052	10,51803	10,58874	10,552887	10,47576333	-0,07712333	0,947945924	Cks2
17548303	10,62984	10,54163	10,48719	10,32052	10,51803	10,58874	10,552887	10,47576333	-0,07712333	0,947945924	Cks2 // Cks2
17548955	10,62984	10,54163	10,48719	10,32052	10,51803	10,58874	10,552887	10,47576333	-0,07712333	0,947945924	Cks2 // Cks2
17410227	10,00517	10,0903	9,785953	9,905104	9,63462	10,11037	9,9604743	9,883364667	-0,07710967	0,947954903	Ap1ar
17205055	4,568083	4,485396	4,187751	4,201951	4,406338	4,401618	4,4137433	4,336635667	-0,07710767	0,947956218	---
17209245	4,568083	4,485396	4,187751	4,201951	4,406338	4,401618	4,4137433	4,336635667	-0,07710767	0,947956218	---
17509629	10,12382	10,16773	10,04802	10,09265	10,0185	9,997103	10,11319	10,03608433	-0,07710567	0,947957532	Sc4mol
17367916	5,976801	5,693775	6,203728	5,99251	5,817928	5,832568	5,9581013	5,881002	-0,07709933	0,947961693	Lrrc26
17336516	5,771039	5,365678	5,51798	5,215275	5,657861	5,550276	5,5515657	5,474470667	-0,077095	0,947964541	Btnl2
17403058	5,84777	6,003318	6,089942	6,116929	5,689509	5,903369	5,9803433	5,903269	-0,07707433	0,94797812	Adh7
17402604	12,35382	12,43826	12,31447	12,21483	12,35603	12,30452	12,36885	12,29179333	-0,07705667	0,947989729	---
17239659	4,669789	4,61062	4,554746	4,522859	4,571979	4,509164	4,6117183	4,534667333	-0,077051	0,947993452	---
17522555	11,30077	11,41069	11,32753	11,22327	11,4202	11,16438	11,34633	11,26928333	-0,07704667	0,9479963	Ltf
17306983	3,630862	3,440869	3,156463	3,250867	3,211236	3,535015	3,409398	3,332372667	-0,07702533	0,948010318	Gzme
17383199	7,967113	7,97089	7,857507	7,736628	7,968647	7,859186	7,9318367	7,854820333	-0,07701633	0,948016232	Rexo4
17548528	6,30025	6,547963	6,527205	6,310658	6,459138	6,374599	6,4584727	6,381465	-0,07700767	0,948021927	Lrp6 // Lrp6
17415361	2,89733	2,765719	3,198773	2,798331	2,90874	2,923788	2,9539407	2,876953	-0,07698767	0,94803507	Ifna11
17331422	7,859878	7,78974	7,599641	7,664238	7,752598	7,601472	7,749753	7,672769333	-0,07698367	0,948037698	LOC100861916
17477648	6,493801	6,417235	6,829705	6,842874	6,32995	6,337018	6,580247	6,503280667	-0,07696633	0,948049089	Gm15545
17481487	4,545554	4,547065	4,77735	4,594808	4,473579	4,570737	4,623323	4,546374667	-0,07694833	0,948060917	Olfr701
17387732	3,76067	3,823297	3,418349	3,56821	3,658651	3,544612	3,6674387	3,590491	-0,07694767	0,948061355	Olfr1105
17236127	5,472028	5,639098	5,640291	5,714425	5,425593	5,380622	5,5838057	5,50688	-0,07692567	0,948075812	Gm6721
17349918	4,055538	4,029133	4,058539	3,947971	3,953859	4,010675	4,0477367	3,970835	-0,07690167	0,948091584	Pcdhb15
17331800	5,900469	6,138549	6,18336	6,129892	5,91023	5,951588	6,074126	5,997236667	-0,07688933	0,948099689	---
17384220	4,63913	3,863987	4,254292	4,033391	4,225181	4,268207	4,2524697	4,175593	-0,07687667	0,948108014	Gm13403
17218209	9,075292	8,946224	8,880972	8,797242	8,998646	8,875977	8,967496	8,890621667	-0,07687433	0,948109547	Edem3
17464424	3,985554	4,077916	3,914226	3,928824	3,838608	3,979644	3,9925653	3,915692	-0,07687333	0,948110204	E330012B07Rik
17383733	8,298155	8,458158	8,439843	8,204296	8,391306	8,369938	8,3987187	8,321846667	-0,076872	0,948111081	BC005624
17385365	5,476873	5,292399	5,805724	5,473466	5,544107	5,326859	5,5249987	5,448144	-0,07685467	0,948122472	Rprm
17248263	9,136026	9,087354	9,189055	9,081331	9,061655	9,038911	9,1374783	9,060632333	-0,076846	0,948128167	Hba-a2 // Hba-a1
17379498	4,637942	4,455266	4,740773	4,658485	4,475728	4,469236	4,611327	4,534483	-0,076844	0,948129482	Gm14316
17216721	4,538778	4,864329	4,484136	4,77771	4,040005	4,839011	4,629081	4,552242	-0,076839	0,948132768	---
17245448	7,62465	7,701242	7,65268	7,527633	7,742804	7,477634	7,659524	7,582690333	-0,07683367	0,948136273	Lemd3
17354585	3,005691	3,101873	3,000066	2,872236	3,124663	2,880235	3,0358767	2,959044667	-0,076832	0,948137368	---
17387485	9,781975	9,818323	9,734329	9,573573	9,731414	9,799145	9,778209	9,701377333	-0,07683167	0,948137587	Zdhhc5
17459952	7,68225	7,731038	7,906547	7,696765	7,710973	7,681639	7,7732783	7,696459	-0,07681933	0,948145693	Mogs
17448748	9,563873	9,012686	8,657098	8,525606	9,292755	9,18493	9,0778857	9,001097	-0,07678867	0,948165847	---
17207965	5,994666	5,941123	5,498768	5,992691	5,746229	5,465306	5,811519	5,734742	-0,076777	0,948173515	---
17438784	7,009356	7,32487	7,274792	7,126733	6,981763	7,270238	7,203006	7,126244667	-0,07676133	0,948183811	Utp3
17429880	7,548414	7,6126	7,962797	7,569571	7,602231	7,721763	7,707937	7,631188333	-0,07674867	0,948192136	Mtf1
17207007	3,96863	4,930336	4,603527	4,366741	4,954222	3,951297	4,500831	4,424086667	-0,07674433	0,948194984	---
17260914	2,965993	2,980307	3,322176	2,955122	3,175819	2,907338	3,089492	3,012759667	-0,07673233	0,948202871	---
17368349	5,488686	4,996188	5,605889	5,270088	5,266849	5,323684	5,3635877	5,286873667	-0,076714	0,948214921	Fam69b
17444916	3,328722	3,548246	3,624874	3,453525	3,306842	3,51141	3,500614	3,423925667	-0,07668833	0,94823179	4930505K14Rik // 4930505K14Rik
17377418	7,529489	7,581068	7,604526	7,352242	7,433371	7,699435	7,5716943	7,495016	-0,07667833	0,948238363	Cst3 // Cst3
17206065	6,064462	6,001342	6,202731	6,146505	6,056238	5,835781	6,0895117	6,012841333	-0,07667033	0,948243621	---
17444653	6,516687	6,198472	6,434889	6,111609	6,412572	6,395861	6,3833493	6,306680667	-0,07666867	0,948244717	Zkscan5
17522158	6,591019	7,128543	6,852396	6,743952	6,760784	6,837252	6,8573193	6,780662667	-0,07665667	0,948252604	Ccdc72 // Ccdc72
17268832	3,020644	3,050416	3,087191	3,019785	2,808246	3,100259	3,0527503	2,976096667	-0,07665367	0,948254576	---
17550450	9,317839	9,437275	9,475203	9,304598	9,36751	9,328271	9,4101057	9,333459667	-0,076646	0,948259615	---
17273348	8,901502	9,034105	8,973259	8,848078	8,715881	9,114976	8,969622	8,892978333	-0,07664367	0,948261149	Fasn
17349894	3,113646	3,316862	3,149453	3,158475	3,049694	3,141861	3,1933203	3,116676667	-0,07664367	0,948261149	Pcdhb9
17397520	5,32382	4,90028	5,495461	5,007984	5,205439	5,276218	5,2398537	5,163213667	-0,07664	0,948263559	---
17368106	5,829516	5,654254	5,946585	5,87127	5,647313	5,681865	5,8101183	5,733482667	-0,07663567	0,948266407	Gm996 // Gm996
17374747	6,813825	6,812421	6,807235	6,729451	6,678639	6,795504	6,8111603	6,734531333	-0,076629	0,948270789	Zfyve19
17327635	6,585727	6,700092	6,51685	6,548895	6,434266	6,589637	6,6008897	6,524266	-0,07662367	0,948274294	Slx4
17206339	3,365538	3,49693	3,640403	3,590793	3,378902	3,303313	3,500957	3,424336	-0,076621	0,948276047	---
17547890	4,963852	4,728591	4,851072	4,76001	4,848337	4,705346	4,8478383	4,771231	-0,07660733	0,94828503	Gm10864
17501325	3,827086	4,01225	3,773298	3,706511	3,903739	3,772593	3,870878	3,794281	-0,076597	0,948291822	---
17548808	7,189728	7,063344	6,953902	6,969598	7,046623	6,960987	7,0689913	6,992402667	-0,07658867	0,9482973	LOC100861693
17299582	4,373119	4,402316	4,445532	4,223101	4,385404	4,382744	4,406989	4,330416333	-0,07657267	0,948307817	Eddm3b
17284571	3,305344	3,292403	3,253725	3,225331	3,166167	3,23029	3,283824	3,207262667	-0,07656133	0,948315267	LOC382693 // LOC382693
17264515	6,620024	6,268122	6,715635	6,309742	6,648643	6,415838	6,5345937	6,458074333	-0,07651933	0,948342874	2310047M10Rik // 2310047M10Rik
17444047	6,38871	6,354425	6,554327	6,186321	6,575765	6,305826	6,4324873	6,355970667	-0,07651667	0,948344627	Elfn1
17315349	7,354641	7,372308	7,429093	7,172342	7,340874	7,413284	7,3853473	7,308833333	-0,076514	0,94834638	Mfsd5
17287115	7,04355	7,127814	7,172875	7,029376	7,0196	7,065728	7,1147463	7,038234667	-0,07651167	0,948347914	Bicd2
17388089	7,449894	7,239925	7,314154	7,147527	7,227652	7,399262	7,3346577	7,258147	-0,07651067	0,948348571	Ptpmt1
17394147	4,053677	3,803855	4,078283	3,83974	3,955553	3,911171	3,978605	3,902154667	-0,07645033	0,948388232	Svs4
17233720	9,770598	9,839548	9,609969	9,621102	9,721909	9,647771	9,7400383	9,663594	-0,07644433	0,948392176	Ppa1
17382203	6,145105	5,626826	5,686975	5,420551	5,914466	5,894598	5,8196353	5,743205	-0,07643033	0,94840138	Hnmt
17206143	5,409373	4,919503	5,323621	4,910144	5,412609	5,100495	5,217499	5,141082667	-0,07641633	0,948410583	---
17488436	6,370746	6,475296	6,36834	6,283169	6,357592	6,344407	6,404794	6,328389333	-0,07640467	0,948418253	Plekhg2
17247471	6,926679	6,678502	6,747293	6,748511	6,696012	6,678758	6,784158	6,707760333	-0,07639767	0,948422854	C1d
17456074	4,941508	4,639954	4,789394	4,618117	4,833501	4,690053	4,7902853	4,713890333	-0,076395	0,948424607	1110019D14Rik
17539158	9,461201	9,553518	9,377018	9,307121	9,534898	9,320613	9,4639123	9,387544	-0,07636833	0,948442138	Eif1ax
17261494	6,90889	6,832435	6,858983	6,656556	6,714554	7,000139	6,8667693	6,790416333	-0,076353	0,948452219	Nprl3
17412534	8,583643	8,464099	8,193492	8,114309	8,451301	8,446579	8,4137447	8,337396333	-0,07634833	0,948455287	Lyrm2
17500559	2,97326	3,097841	3,002503	2,823236	2,981244	3,04008	3,0245347	2,948186667	-0,076348	0,948455506	---
17536076	2,945755	2,926766	3,043956	2,672082	3,116141	2,899247	2,972159	2,895823333	-0,07633567	0,948463614	---
17479335	4,799161	4,492955	4,765622	4,705609	4,410526	4,712625	4,6859127	4,609586667	-0,076326	0,948469969	Acan
17270025	5,349807	4,997536	4,975646	5,097622	4,87763	5,11879	5,107663	5,031347333	-0,07631567	0,948476762	---
17434333	7,138284	7,226686	6,943871	7,119859	6,993762	6,966349	7,102947	7,026656667	-0,07629033	0,948493418	Pex1
17290104	2,851617	2,977124	3,114203	2,905105	2,820581	2,988402	2,9809813	2,904696	-0,07628533	0,948496705	---
17319324	5,075728	5,445317	5,173517	5,057155	5,264535	5,144068	5,2315207	5,155252667	-0,076268	0,948508101	Dnalc4
17337693	3,427587	3,185885	3,403435	3,493558	3,220034	3,074526	3,338969	3,262706	-0,076263	0,948511388	Gm20580
17200267	3,215681	3,048167	3,319473	3,36356	3,041064	2,949933	3,1944403	3,118185667	-0,07625467	0,948516867	---
17517050	5,108997	4,987947	5,155413	5,135741	4,839244	5,048612	5,084119	5,007865667	-0,07625333	0,948517743	Tmprss5
17298626	6,305096	6,131114	6,236267	6,447571	5,928802	6,067406	6,224159	6,147926333	-0,07623267	0,948531331	Fam170b
17356897	7,590601	7,463317	7,420377	7,22999	7,563657	7,45195	7,4914317	7,415199	-0,07623267	0,948531331	Rasgrp2
17547783	8,853857	8,775471	8,76642	8,734631	8,763395	8,669044	8,7985827	8,722356667	-0,076226	0,948535714	4933434E20Rik // 4933434E20Rik
17322578	4,833693	4,771867	4,857243	4,795146	4,737293	4,701716	4,8209343	4,744718333	-0,076216	0,948542289	4930562C15Rik // 4930562C15Rik
17205669	2,691317	3,527909	3,754204	3,333794	3,629851	2,781243	3,3244767	3,248296	-0,07618067	0,94856552	---
17495818	7,196915	7,178221	7,240941	7,288041	6,892655	7,20686	7,205359	7,129185333	-0,07617367	0,948570123	---
17409856	2,977639	2,892472	3,02702	2,930141	2,890609	2,847873	2,9657103	2,889541	-0,07616933	0,948572972	---
17491975	4,537484	4,533227	4,493721	4,436223	4,376732	4,522969	4,5214773	4,445308	-0,07616933	0,948572972	---
17222618	5,019871	5,318358	5,324232	5,067961	5,057384	5,308625	5,2208203	5,144656667	-0,07616367	0,948576698	2610017I09Rik
17447346	5,64817	5,46146	5,705209	5,45666	5,36541	5,764307	5,6049463	5,528792333	-0,076154	0,948583054	2310079F23Rik
17338371	7,602555	8,221925	8,176573	7,917828	8,06725	7,787525	8,000351	7,924201	-0,07615	0,948585684	Trem3
17227430	4,548337	4,540321	4,499512	4,70856	4,284521	4,366675	4,52939	4,453252	-0,076138	0,948593574	6530439I21 // 6530439I21
17550146	3,021239	3,056311	3,182163	2,949995	3,041643	3,039693	3,086571	3,010443667	-0,07612733	0,948600587	---
17280749	8,020294	8,000734	7,958282	7,985353	7,896815	7,868777	7,9931033	7,916981667	-0,07612167	0,948604313	Tspan13
17325637	6,064796	6,019693	5,959723	6,001754	5,952506	5,861623	6,0147373	5,938627667	-0,07610967	0,948612204	B4galt4
17222819	4,379601	4,097116	4,126474	4,159577	4,199863	4,015448	4,2010637	4,124962667	-0,076101	0,948617902	9330175M20Rik
17489943	5,595576	5,977101	5,559227	5,748677	5,364395	5,790552	5,7106347	5,634541333	-0,07609333	0,948622943	LOC100861686
17480922	7,040208	6,959297	6,774786	6,78386	6,805999	6,956165	6,9247637	6,848674667	-0,076089	0,948625793	Mir139
17285703	13,4542	13,42239	13,48205	13,35985	13,40499	13,36555	13,45288	13,37679667	-0,07608333	0,948629519	Hist1h2ao
17291023	13,4542	13,42239	13,48205	13,35985	13,40499	13,36555	13,45288	13,37679667	-0,07608333	0,948629519	Hist1h2ao
17474534	7,184908	6,976638	7,172485	7,108467	7,055927	6,941451	7,1113437	7,035281667	-0,076062	0,948643546	Opa3
17415715	10,02999	10,16212	9,983358	9,952852	9,977228	10,01724	10,058489	9,98244	-0,07604933	0,948651875	Usp1
17401308	6,95059	7,010824	6,671497	6,552253	6,904028	6,948493	6,877637	6,801591333	-0,07604567	0,948654286	Phtf1
17223728	3,568494	3,460006	3,221752	3,213683	3,57035	3,238117	3,4167507	3,340716667	-0,076034	0,948661958	---
17455274	6,965442	7,091302	6,948756	6,88262	6,956268	6,938511	7,0018333	6,925799667	-0,07603367	0,948662177	Mtif3
17284880	11,18688	11,20206	11,11415	11,11132	11,09317	11,07051	11,167697	11,09166667	-0,07603	0,948664588	Gdi2
17314435	14,07479	14,11834	14,14849	14,00701	14,01686	14,08967	14,113873	14,03784667	-0,07602667	0,94866678	Rn7sk // Rn7sk // Rn7sk // Rn7sk // Rn
17201683	5,113053	5,515641	4,112525	4,962171	4,722357	4,828643	4,9137397	4,837723667	-0,076016	0,948673794	---
17503546	4,562048	4,491805	4,567873	4,601529	4,30668	4,485484	4,5405753	4,464564333	-0,076011	0,948677082	4933402J07Rik
17283897	5,768836	5,863387	6,109688	5,570023	5,976603	5,967267	5,9139703	5,837964333	-0,076006	0,94868037	Ccdc85c
17313276	9,572771	9,641985	9,718702	9,405106	9,703701	9,596681	9,644486	9,568496	-0,07599	0,948690891	Rbx1
17227149	4,678697	4,825295	4,754264	4,498448	4,825295	4,706544	4,752752	4,676762333	-0,07598967	0,94869111	4931440L10Rik

17449155	8,143834	8,439329	8,248657	8,100307	8,083381	8,420202	8,2772733	8,201296667	-0,07597667	0,948699659	Cenpc1
17356556	9,436473	9,628944	9,660529	9,468134	9,531334	9,498552	9,5753153	9,49934	-0,07597533	0,948700536	Malat1 // Malat1
17549546	3,662115	3,859936	3,821987	3,778036	3,647613	3,690502	3,781346	3,705383667	-0,07596233	0,948709084	---
17549702	3,662115	3,859936	3,821987	3,778036	3,647613	3,690502	3,781346	3,705383667	-0,07596233	0,948709084	---
17472632	7,071931	6,95998	7,386405	7,139147	7,010237	7,041046	7,1394387	7,063476667	-0,075962	0,948709303	4930579D09Rik
17300075	4,73242	4,903801	4,975331	4,68494	4,704749	4,994034	4,8705173	4,794574333	-0,075943	0,948721798	---
17355984	8,898882	9,024412	8,936942	8,748689	8,817076	9,066679	8,953412	8,877481333	-0,07593067	0,948729908	Chka
17263223	4,988713	4,9185	5,189971	4,972968	5,008907	4,887547	5,0323947	4,956474	-0,07592067	0,948736484	Obscn
17228858	3,225737	2,918038	2,906405	3,038234	2,71296	3,071236	3,0167267	2,94081	-0,07591667	0,948739115	---
17201121	5,552864	5,795041	5,80548	5,480795	5,599218	5,845638	5,717795	5,641883667	-0,07591133	0,948742622	---
17499796	7,243618	6,80383	7,158237	7,056394	6,954475	6,967084	7,0685617	6,992651	-0,07591067	0,948743061	Gm15315
17434833	4,305202	4,438577	4,517204	4,548858	4,138481	4,345951	4,4203277	4,34443	-0,07589767	0,94875161	Pclo
17422264	4,141109	3,851219	4,300634	3,9051	4,097429	4,062752	4,097654	4,021760333	-0,07589367	0,94875424	---
17205543	4,360256	6,001148	5,127447	5,133955	4,74478	5,382543	5,1629503	5,087092667	-0,07585767	0,948777915	---
17517116	9,724873	9,717344	9,764389	9,655065	9,719876	9,604108	9,7355353	9,659683	-0,07585233	0,948781422	Timm8b
17223261	3,65922	3,743469	3,641176	3,51526	3,55263	3,748421	3,6812883	3,605437	-0,07585133	0,94878208	Boll
17319864	7,915432	7,925286	8,15285	7,900657	7,981824	7,883584	7,997856	7,922021667	-0,07583433	0,94879326	Mcat
17295926	8,990105	9,107342	8,932566	8,924923	8,931013	8,946581	9,0100043	8,934172333	-0,075832	0,948794795	2410002O22Rik
17480228	5,538308	5,325502	5,488976	5,532273	5,196198	5,396827	5,4509287	5,375099333	-0,07582933	0,948796548	---
17480636	7,245474	7,008873	6,847332	6,950833	6,855154	7,068205	7,033893	6,958064	-0,075829	0,948796768	Lipt2
17275754	3,867002	3,724895	3,630338	3,711563	3,587944	3,695269	3,740745	3,664925333	-0,07581967	0,948802906	---
17269126	4,045149	4,165556	4,250402	4,197183	4,016695	4,019783	4,1537023	4,077887	-0,07581533	0,948805756	Krtap3-3
17350952	8,61638	8,725985	8,64997	8,520965	8,522564	8,721393	8,6641117	8,588307333	-0,07580433	0,94881299	Rbm22
17516155	3,692158	3,78626	3,673171	3,52913	3,688237	3,706836	3,7171963	3,641401	-0,07579533	0,948818909	Olfr898
17451043	7,296583	7,725302	7,376785	7,255457	7,426509	7,489347	7,4662233	7,390437667	-0,07578567	0,948825266	Noc4l
17287035	4,519979	4,240489	4,289053	4,115848	4,488829	4,217494	4,3498403	4,274057	-0,07578333	0,948826801	E030046B03Rik
17383712	5,874649	5,609441	6,104686	5,815912	5,757782	5,787746	5,8629253	5,787146667	-0,07577867	0,94882987	Ptges
17203957	5,615296	5,928055	6,075612	5,484502	5,621051	6,286103	5,8729877	5,797218667	-0,075769	0,948836228	---
17208631	7,064271	7,032296	7,078852	6,882551	6,849621	7,215944	7,058473	6,982705333	-0,07576767	0,948837105	---
17272870	4,986934	5,019525	5,253671	4,902713	5,101961	5,028226	5,08671	5,010966667	-0,07574333	0,948853108	---
17259869	9,825687	9,943154	9,803186	9,639066	9,724959	9,980853	9,8573423	9,781626	-0,07571633	0,948870866	---
17507034	3,084207	2,89045	2,767395	3,022042	2,824945	2,667937	2,9140173	2,838308	-0,07570933	0,94887547	---
17351426	4,841899	4,651417	4,858676	4,700009	4,656608	4,768272	4,7839973	4,708296333	-0,075701	0,948880951	Gnal
17271710	5,913155	6,100544	5,842381	6,117112	5,780932	5,730955	5,9520267	5,876333	-0,07569367	0,948885775	Cd300lb
17373832	6,584592	6,481108	6,765969	6,532947	6,546681	6,525007	6,6105563	6,534878333	-0,075678	0,948896079	A930018P22Rik
17524328	3,141356	2,907701	3,003925	3,071597	2,947394	2,806991	3,0176607	2,941994	-0,07566667	0,948903533	Olfr843
17200705	6,175205	6,43099	6,252328	6,203102	5,923177	6,505288	6,2861743	6,210522333	-0,075652	0,94891318	---
17412323	3,792589	3,753497	3,555226	3,45241	3,873431	3,548528	3,7004373	3,624789667	-0,07564767	0,94891603	---
17301262	5,597697	5,800004	5,87304	5,77508	5,432193	5,836527	5,7569137	5,681266667	-0,075647	0,948916469	Rp1l1
17264046	6,539159	6,532139	6,549797	6,385726	6,61845	6,390079	6,540365	6,464751667	-0,07561333	0,948938613	BC046404
17544919	6,353616	6,582925	6,189243	5,961029	6,488233	6,449719	6,3752613	6,299660333	-0,075601	0,948946725	Nup62cl
17495541	5,857476	5,783239	6,069686	5,714917	5,845099	5,923599	5,903467	5,827871667	-0,07559533	0,948950452	Syt17
17416009	4,673865	4,363182	4,269383	4,422421	4,234805	4,422421	4,4354767	4,359882333	-0,07559433	0,94895111	Gm12794
17397373	3,356997	3,327829	3,58989	3,378416	3,258523	3,411033	3,4249053	3,349324	-0,07558133	0,948959661	---
17537375	3,224122	3,202356	3,578584	3,299644	3,173381	3,305298	3,3350207	3,259441	-0,07557967	0,948960757	---
17503681	7,121834	7,278819	6,900136	6,982406	7,041439	7,050287	7,100263	7,024710667	-0,07555233	0,948978737	Cyld
17231047	11,16974	11,00271	11,0737	10,96094	11,0148	11,04376	11,08205	11,0065	-0,07555	0,948980271	Ppp2r5a
17263070	4,642341	4,786092	4,550086	4,442401	4,756894	4,552588	4,6595063	4,583961	-0,07554533	0,948983341	---
17293135	8,553294	8,657742	8,561961	8,430088	8,605291	8,510996	8,590999	8,515458333	-0,07554067	0,948986411	2210016F16Rik // 2210016F16Rik
17309020	4,873744	4,556793	5,137523	4,471687	5,18147	4,688343	4,85602	4,7805	-0,07552	0,949000005	---
17476666	3,322636	3,084793	3,111702	3,006782	3,162805	3,123015	3,1730437	3,097534	-0,07550967	0,949006802	Gm12769
17343299	6,34856	6,133098	6,24359	6,182782	6,287838	6,028104	6,2417493	6,166241333	-0,075508	0,949007899	Notch3
17238179	3,894275	3,505894	3,773127	3,513393	3,773336	3,660069	3,724432	3,648932667	-0,07549933	0,9490136	Rdh18-ps
17434087	6,6111	6,494686	6,742473	6,595944	6,524795	6,501036	6,6160863	6,540591667	-0,07549467	0,949016669	Ccl19
17238722	4,763086	4,516029	4,629139	4,781503	4,62631	4,273974	4,6360847	4,560595667	-0,075489	0,949020397	H60c
17318013	5,926409	5,699868	5,820243	5,702401	5,687419	5,830266	5,8155067	5,740028667	-0,075478	0,949027633	Lynx1
17447726	4,632814	4,601865	4,628301	4,503008	4,601865	4,531695	4,6209933	4,545522667	-0,07547067	0,949032457	Hs3st1
17259443	5,335405	5,029453	5,218971	5,003601	5,203053	5,150772	5,1946097	5,119142	-0,07546767	0,94903443	Notum
17397494	5,135039	5,235881	5,118958	4,939329	5,196724	5,127469	5,1632927	5,087840667	-0,075452	0,949044736	---
17528673	6,824575	6,824575	6,703509	6,659173	6,769526	6,697608	6,7842197	6,708769	-0,07545067	0,949045613	Myzap
17548648	5,438688	5,391729	5,590673	5,444303	5,399627	5,350837	5,4736967	5,398255667	-0,075441	0,949051972	A430107J10Rik
17393187	4,698257	4,520714	4,725144	4,481655	4,51059	4,72563	4,6480383	4,572625	-0,07541333	0,949070173	---
17505938	7,553104	7,626514	7,52775	7,524281	7,51333	7,443533	7,5691227	7,493714667	-0,075408	0,949073681	Bcmo1
17371870	5,496099	5,705185	5,412986	5,463549	5,301774	5,622777	5,53809	5,4627	-0,07539	0,949085522	---
17482093	6,723029	7,178637	6,511145	6,806094	6,567259	6,813303	6,8042703	6,728885333	-0,075385	0,949088812	Gm10589
17218073	4,493357	4,395503	4,491997	4,61254	4,152705	4,389485	4,4602857	4,38491	-0,07537567	0,949094952	Pdc
17430958	3,215763	3,411981	3,561517	3,295305	3,266922	3,400938	3,3964203	3,321055	-0,07536533	0,94910175	---
17410323	7,619571	7,71216	7,649622	7,427232	7,591863	7,736186	7,660451	7,585093667	-0,07535733	0,949107013	Gar1
17322453	4,139169	4,120441	4,117663	4,054626	3,928463	4,168128	4,1257577	4,050405667	-0,075352	0,949110521	Olfr161
17287726	7,299407	7,42924	7,383715	7,121306	7,376807	7,388205	7,3707873	7,295439333	-0,075348	0,949113153	Caml
17512657	7,952193	7,771335	7,72529	7,747868	7,652883	7,82203	7,8162727	7,740927	-0,07534567	0,949114688	Slc7a6os
17345775	5,853152	6,157874	5,792907	5,656024	6,22558	5,69646	5,9346443	5,859354667	-0,07528967	0,94915153	A530064D06Rik // A530064D06Rik
17332290	5,762102	5,697342	5,680687	5,706367	5,662699	5,545198	5,713377	5,638088	-0,075289	0,949151968	Cldn14
17398717	5,564487	5,283051	5,350088	5,44824	5,081789	5,441791	5,3992087	5,32394	-0,07526867	0,949165346	---
17309368	4,250837	4,322802	4,331409	4,388176	3,99114	4,299955	4,3016827	4,226423667	-0,075259	0,949171705	Slitrk6
17375378	4,352798	4,34002	4,316022	4,123275	4,39142	4,268371	4,33628	4,261022	-0,075258	0,949172363	Trim69
17419060	5,008249	4,516599	5,259049	4,790904	5,00841	4,759004	4,9279657	4,852772667	-0,075193	0,949215129	E330017L17Rik
17360097	6,300406	6,266279	6,589254	6,41288	6,264643	6,252841	6,385313	6,310121333	-0,07519167	0,949216006	Cnnm2
17318045	4,361931	4,378358	4,252177	4,123271	4,419151	4,224537	4,330822	4,255653	-0,075169	0,94923092	Cyp11b1
17219867	3,962394	3,644243	4,056532	3,859328	3,818769	3,75958	3,887723	3,812565667	-0,07515733	0,949238596	---
17208907	3,376562	3,497931	3,730497	3,200252	3,363115	3,816152	3,5349967	3,459839667	-0,075157	0,949238815	---
17360942	5,317143	5,329086	5,255481	5,141043	5,312401	5,222852	5,30057	5,225432	-0,075138	0,949251317	Gal
17475592	5,835686	5,914505	6,027552	5,812651	5,851605	5,888161	5,9259143	5,850805667	-0,07510867	0,949270617	Sertad3
17497796	5,525343	5,386984	5,368362	5,386441	5,493746	5,175195	5,4268963	5,351794	-0,07510233	0,949274785	1600016N20Rik
17421968	5,259124	5,796895	5,452448	5,430663	5,560873	5,291656	5,5028223	5,427730667	-0,07509167	0,949281803	---
17405897	3,477546	3,624482	3,329861	3,410789	3,3201	3,475745	3,4772963	3,402211333	-0,075085	0,94928619	---
17341700	5,784953	6,286979	6,52295	6,640655	5,736397	5,992581	6,198294	6,123211	-0,075083	0,949287506	---
17254627	4,853116	4,855649	4,568313	4,575445	4,858704	4,617686	4,759026	4,683945	-0,075081	0,949288822	---
17469793	6,565361	6,337385	6,5453	6,821073	6,250775	6,151002	6,482682	6,407616667	-0,07506533	0,94929913	1700015O11Rik // 1700015O11Rik
17385767	5,270121	5,373909	5,223839	5,202535	5,164519	5,275658	5,2892897	5,214237333	-0,07505233	0,949307684	Fap
17523994	9,059713	9,025425	8,987529	8,890134	8,985327	8,972069	9,0242223	8,949176667	-0,07504567	0,949312071	Cep57
17313616	4,398263	4,061436	4,300111	4,135352	4,26683	4,1325	4,25327	4,178227333	-0,07504267	0,949314045	AW121686
17201027	8,257556	8,265208	8,435226	8,18606	8,012973	8,533875	8,31933	8,244302667	-0,07502733	0,949324135	---
17432912	7,96628	8,117598	7,863129	7,701592	8,084303	7,936085	7,9823357	7,907326667	-0,075009	0,949336199	Fbxo6
17218953	3,129647	2,998161	3,049969	3,036395	3,018262	2,898154	3,059259	2,984270333	-0,07498867	0,949349579	---
17551073	9,329601	9,306204	9,311243	9,170226	9,188329	9,363548	9,3156827	9,240701	-0,07498167	0,949354185	---
17546016	6,861109	6,94551	6,820613	6,69379	6,803337	6,905189	6,875744	6,800772	-0,074972	0,949360546	Ofd1
17210799	5,98514	5,310337	5,97806	5,05194	6,221835	5,774867	5,7578457	5,682880667	-0,074965	0,949365152	---
17282397	3,183543	3,339366	3,079958	3,24782	2,985217	3,145012	3,2009557	3,126016333	-0,07493933	0,949382043	---
17358466	5,055065	4,946679	5,095335	5,033797	4,909257	4,929213	5,0323597	4,957422333	-0,07493733	0,949383359	Slc1a1
17523359	5,170838	5,008462	5,067825	5,094728	5,056555	4,871056	5,082375	5,007446333	-0,07492867	0,949389062	Kbtbd5
17208115	3,801068	3,451827	3,217041	3,316479	3,320505	3,608279	3,4899787	3,415087667	-0,074891	0,949413849	---
17483230	5,063281	5,204133	5,25691	4,822036	5,279905	5,197719	5,1747747	5,099886667	-0,074888	0,949415824	2900092E17Rik // 2900092E17Rik
17387614	3,057212	2,942497	3,064648	2,825315	3,086584	2,927832	3,0214523	2,946577	-0,07487533	0,949424159	Olfr154
17257026	4,303908	4,251676	4,568653	4,268772	4,476682	4,154214	4,3747457	4,299889333	-0,07485633	0,949436663	Higd1b
17300959	7,333856	7,88824	7,557566	7,495577	7,496247	7,563317	7,5932207	7,518380333	-0,07484033	0,949447193	---
17222650	6,621719	6,673757	6,346317	6,270947	6,674585	6,471745	6,5472643	6,472425667	-0,07483867	0,94944829	Uxs1
17408470	6,012465	5,764032	5,776515	5,892715	5,681509	5,754288	5,851004	5,776170667	-0,07483333	0,9494518	Cd101
17252497	7,867803	7,930954	7,821209	7,748548	7,946553	7,700433	7,873322	7,798511333	-0,07481067	0,949466717	Atp2a3
17372755	5,551934	5,089778	4,947879	5,310339	4,890827	5,164009	5,1965303	5,121725	-0,07480533	0,949470227	Olfr1010
17363044	4,382852	3,626973	4,049437	3,845649	3,853813	4,135441	4,019754	3,944967667	-0,07478633	0,949482731	Olfr1417
17549928	7,421912	7,293142	7,68571	7,314908	7,416205	7,44534	7,4669213	7,392151	-0,07477033	0,949493261	---
17207679	5,001671	5,229611	4,144372	5,131998	4,314352	4,705067	4,7918847	4,717139	-0,07474567	0,949509496	---
17513806	6,869588	6,647214	7,137812	6,643833	7,10921	6,67737	6,8848713	6,810137667	-0,07473367	0,949517393	Cbfa2t3
17262650	7,629775	7,946679	7,902369	7,616103	7,840172	7,798355	7,8262743	7,751543333	-0,074731	0,949519149	Uqcrq
17428140	3,36414	3,007982	3,030666	3,200392	3,072447	2,905812	3,1342627	3,059550333	-0,07471233	0,949531434	---
17366373	8,934072	9,029178	8,787564	8,715777	8,907155	8,903794	8,916938	8,842242	-0,074696	0,949542184	Nmt2
17457125	4,911739	4,763681	4,87116	4,745018	4,83157	4,745921	4,84886	4,774169667	-0,07469033	0,949545914	---
17312829	9,439262	9,795301	10,00356	9,653926	9,76796	9,592173	9,746041	9,671353	-0,074688	0,94954745	Lgals1
17313705	7,880132	7,472404	7,587246	7,277424	7,715322	7,723002	7,646594	7,571916	-0,074678	0,949554031	Parvb
17332223	5,017094	5,140509	5,362843	4,852446	5,055474	5,388497	5,173482	5,098805667	-0,07467633	0,949555128	Kcne1
17493238	8,415464	8,567327	8,47029	8,407645	8,392433	8,428995	8,4843603	8,409691	-0,07466933	0,949559736	I7Rn6
17203057	3,921839	3,403774	3,372437	3,138004	3,875893	3,460157	3,5660167	3,491351333	-0,07466533	0,949562368	---
17351685	2,996732	3,025636	3,105364	2,981733	3,041928	2,880097	3,0425773	2,967919333	-0,074658	0,949567195	---
17355286	7,940972	7,50792	7,801692	7,605955	7,818972	7,601685	7,7501947	7,675537333	-0,07465733	0,949567634	Rab27b
17210677	3,178968	3,055937	3,426716	3,396866	2,859683	3,181147	3,2205403	3,145898667	-0,07464167	0,949577946	---
17349174	7,994542	8,268533	7,792398	7,775417	7,906519	8,149623	8,018491	7,943853	-0,074638	0,949580359	Polr2d
17548597	11,98961	12,10831	12,01076	12,0156	11,79351	12,0757	12,036227	11,96160333	-0,07462333	0,949590013	LOC100505014
17480100	4,458899	4,188859	4,388082	3,955553	4,461044	4,395414	4,34528	4,270670333	-0,07460967	0,949599008	---
17337458	9,762813	10,13642	10,09561	9,713135	9,960501	10,1514	9,998281	9,923678667	-0,07460233	0,949603835	Trim10
17229222	8,561722	8,633705	8,405923	8,336781	8,531586	8,509213	8,5337833	8,459193333	-0,07459	0,949611953	Tiprl
17240813	4,103771	3,97581	3,945886	4,094229	3,774651	3,932828	4,008489	3,933902667	-0,07458633	0,949614367	Zfa
17503106	6,193632	6,087982	6,065559	5,835485	6,000507	6,287448	6,1157243	6,041146667	-0,07457767	0,949620071	4930432K21Rik
17447623	5,554811	5,649386	5,6878	5,494134	5,431616	5,742552	5,6306657	5,556100667	-0,074565	0,949628409	Zbtb49

17475801	6,035101	6,317795	6,232517	6,016307	6,333466	6,01201	6,1951377	6,120594333	-0,07454333	0,949642671	Eid2
17240645	6,375685	6,334998	6,325563	6,238751	6,268773	6,305099	6,3454153	6,270874333	-0,074541	0,949644207	Prdm1
17220719	3,310378	3,565331	3,301785	3,290673	3,35397	3,309232	3,392498	3,317958333	-0,07453967	0,949645084	---
17325592	6,325087	6,282525	6,273146	6,205375	6,206323	6,245461	6,293586	6,219053	-0,074533	0,949649472	Popdc2
17256857	5,733946	5,663852	5,897218	5,649742	5,705691	5,716068	5,7650053	5,690500333	-0,074505	0,949667904	4930417O22Rik
17478789	7,147751	7,215776	7,248949	7,128024	7,160411	7,100527	7,2041587	7,129654	-0,07450467	0,949668123	Ndn
17203659	6,81893	6,94524	6,510067	7,093548	6,544778	6,412414	6,758079	6,68358	-0,074499	0,949671853	---
17455111	11,34026	11,43562	11,48577	11,42189	11,29704	11,31934	11,42055	11,34609	-0,07446	0,949697526	Cyp3a16
17278529	6,037398	5,933058	6,145116	6,004251	5,994224	5,893726	6,038524	5,964067	-0,074457	0,949699501	Hhipl1
17248716	3,894311	3,970691	3,920547	3,953789	3,948768	3,659631	3,9285163	3,854062667	-0,07445367	0,949701695	---
17262259	6,427115	6,217155	6,34774	6,328568	6,218022	6,222069	6,33067	6,256219667	-0,07445033	0,949703889	Btnl9
17327974	5,698267	5,321531	5,588225	5,560265	5,519304	5,305183	5,5360077	5,461584	-0,07442367	0,949721444	Cldn26
17216144	6,129732	5,997649	6,283815	6,216393	5,912117	6,059433	6,1370653	6,062647667	-0,07441767	0,949725393	Neu4
17510605	3,821215	3,833053	3,886798	3,574685	3,955553	3,787624	3,847022	3,772620667	-0,07440133	0,949736146	---
17442887	5,704705	5,55458	5,590664	5,539803	5,528751	5,558316	5,6166497	5,54229	-0,07435967	0,949763575	Mmp17
17309006	10,60508	10,59122	10,6911	10,47683	10,64476	10,54276	10,629133	10,55478333	-0,07435	0,949769939	Gm5088 // Gm5088
17275015	4,10457	4,211198	4,132771	3,936574	4,066402	4,222608	4,149513	4,075194667	-0,07431833	0,949790787	Agr2
17326448	3,313007	3,311341	3,370727	3,081503	3,481336	3,209356	3,3316917	3,257398333	-0,07429333	0,949807245	Olfr195
17299895	3,986098	3,511609	3,504574	3,592355	3,441334	3,745724	3,667427	3,593137667	-0,07428933	0,949809879	---
17417729	5,91963	6,260715	6,244554	6,023746	5,963492	6,214804	6,141633	6,067347333	-0,07428567	0,949812293	---
17512872	3,827026	3,574351	3,631813	3,7299	3,564785	3,515657	3,67773	3,603447333	-0,07428267	0,949814268	Calb2
17408546	7,201994	7,116708	7,253329	7,263188	7,104447	6,981668	7,190677	7,116434333	-0,07424267	0,949840603	Slc22a15
17374174	3,063374	2,912893	3,151504	2,965808	3,086918	2,852326	3,0425903	2,968350667	-0,07423967	0,949842578	Olfr1280
17468277	6,996384	6,923485	7,043168	6,834868	7,048859	6,856632	6,987679	6,913453	-0,074226	0,949851576	Dysf // Dysf
17231761	3,982323	3,897491	4,09118	3,860441	4,007518	3,88037	3,9903313	3,916109667	-0,07422167	0,949854429	---
17206247	7,479138	7,602795	7,315255	7,507014	7,584187	7,08334	7,4657293	7,391513667	-0,07421567	0,949858379	---
17322467	7,151052	6,938942	6,967135	7,200351	6,839136	6,795	7,019043	6,944829	-0,074214	0,949859476	Zfp174
17299119	4,416156	4,272073	3,984781	4,23051	4,058387	4,161473	4,2243367	4,150123333	-0,07421333	0,949859915	---
17491745	5,470145	4,748868	5,361765	5,087995	5,136172	5,133985	5,1935927	5,119384	-0,07420867	0,949862988	Mir344f
17389672	4,512115	4,36209	4,436788	4,325119	4,320819	4,44245	4,4369977	4,362796	-0,07420167	0,949867597	---
17522839	4,972706	4,867197	4,957614	4,984247	4,839448	4,751218	4,9325057	4,858304333	-0,07420133	0,949867816	---
17546774	11,18967	11,10539	11,23806	11,5584	10,97117	10,7811	11,177707	11,10355667	-0,07415	0,949901614	Erdr1
17271607	5,721569	5,54216	5,637639	5,45234	5,593752	5,632827	5,6337893	5,559639667	-0,07414967	0,949901834	Cpsf4l
17307157	8,951291	9,109351	8,814572	8,836941	8,969092	8,846738	8,9584047	8,884257	-0,07414767	0,949903151	Xpo4
17395298	4,175436	4,212562	4,32339	4,206279	4,303357	3,979358	4,2371293	4,162998	-0,07413133	0,949913905	---
17319465	10,24898	10,23842	10,06537	9,97875	10,24896	10,10275	10,184257	10,11015333	-0,07410333	0,949932341	St13
17411913	3,240504	3,434406	3,215243	3,185013	3,258832	3,224028	3,2967177	3,222624333	-0,07409333	0,949938926	---
17233489	6,072278	6,417478	6,222864	6,223723	6,023585	6,243035	6,23754	6,163447667	-0,07409233	0,949939584	Ccdc138
17204791	4,047925	4,478416	4,415047	4,12707	4,300234	4,291841	4,313796	4,239715	-0,074081	0,949947047	---
17532242	5,197001	5,174311	5,13876	4,932494	5,140257	5,215081	5,170024	5,095944	-0,07408	0,949947705	Gm2449
17455254	6,879967	7,372401	7,034691	6,771284	7,166855	7,126711	7,0956863	7,021616667	-0,07406967	0,949954509	Usp12
17333903	3,946572	3,815601	3,35666	3,500023	3,864331	3,532315	3,7062777	3,632223	-0,07405467	0,949964386	Vmn1r237
17240186	5,240867	5,172989	5,447478	5,15524	5,167286	5,316685	5,2871113	5,213070333	-0,074041	0,949973385	Fam26f
17516952	4,756561	4,657314	4,813491	4,635574	4,85124	4,518438	4,7424553	4,668417333	-0,074038	0,949975361	2900052N01Rik
17414583	4,905887	4,667667	4,760715	4,802848	4,57796	4,731383	4,7780897	4,704063667	-0,074026	0,949983262	4933430I17Rik
17306960	3,673608	3,635998	4,089409	3,779067	3,993916	3,404097	3,7996717	3,725693333	-0,07397833	0,95001465	Mcpt4
17379427	5,908069	5,910781	5,811631	5,951686	5,749964	5,706918	5,876827	5,802856	-0,073971	0,950019479	Dbndd2
17428249	6,131365	6,388012	6,489795	6,517394	6,066864	6,203167	6,3363907	6,262475	-0,07391567	0,950055917	---
17398895	5,349836	5,079137	5,41691	5,24016	5,263453	5,120543	5,281961	5,208052	-0,073909	0,950060307	Crabp2
17307991	7,633864	7,70727	7,520202	7,476311	7,497515	7,665825	7,6204453	7,546550333	-0,073895	0,950069527	Dock5
17498786	5,310981	5,395408	5,480866	5,247101	5,378437	5,340064	5,3957517	5,321867333	-0,07388433	0,950076551	Lrrc8e
17450545	4,233741	4,281262	4,396375	4,148355	4,311923	4,229498	4,3037927	4,229925333	-0,07386733	0,950087747	---
17512630	6,23035	6,472903	6,264759	6,185857	6,331292	6,229301	6,3226707	6,248816667	-0,073854	0,950096527	Ddx28
17454836	8,1113	8,280635	8,286928	8,110613	8,197113	8,149614	8,2262877	8,152446667	-0,073841	0,950105089	E130309D02Rik
17549584	3,756678	3,848141	3,747415	3,634564	3,751173	3,745002	3,784078	3,710246333	-0,07383167	0,950111235	---
17549854	3,756678	3,848141	3,747415	3,634564	3,751173	3,745002	3,784078	3,710246333	-0,07383167	0,950111235	---
17210135	4,591758	5,567734	5,483475	5,348184	4,8372	5,236116	5,2143223	5,1405	-0,07382233	0,950117382	---
17474921	4,979117	4,865247	4,817073	4,869859	4,738365	4,831748	4,8871457	4,813324	-0,07382167	0,950117821	---
17227025	5,840351	5,925185	5,813839	5,755012	5,805723	5,797236	5,8597917	5,785990333	-0,07380133	0,950131212	Lax1
17479575	10,07043	10,1624	10,21087	10,05441	10,04412	10,12378	10,1479	10,07410333	-0,07379667	0,950134285	Prc1
17335046	5,697871	5,509614	5,673142	5,642274	5,381531	5,635454	5,6268757	5,553086333	-0,07378933	0,950139115	Ggnbp1
17365162	7,348509	7,628301	7,472638	7,309215	7,305828	7,613042	7,4831493	7,409361667	-0,07378767	0,950140213	---
17488535	7,285501	7,21394	7,447384	7,244234	7,234575	7,246665	7,3156083	7,241824667	-0,07378367	0,950142847	Mrps12
17466861	4,697215	4,470821	4,826231	4,562375	4,537381	4,673255	4,6647557	4,591003667	-0,073752	0,950163702	Mira
17425179	3,317448	3,229021	3,142819	3,121604	3,163836	3,182631	3,2297627	3,156023667	-0,073739	0,950172264	Mir1958 // Mir1958
17404888	4,866846	4,396904	4,528058	4,438628	4,691483	4,440504	4,5972693	4,523538333	-0,073731	0,950177533	Il21
17390371	9,736238	9,794893	9,770649	9,562877	9,67611	9,841672	9,76726	9,693553	-0,073707	0,95019334	Epb4.2
17299516	8,876479	8,777827	8,935308	8,681291	8,869258	8,817953	8,8632047	8,789500667	-0,073704	0,950195316	---
17550759	3,189692	3,295207	3,241637	3,099532	3,209558	3,196345	3,2421787	3,168478333	-0,07370033	0,950197731	---
17525342	8,280826	8,169008	8,274326	8,034286	8,251312	8,217496	8,2413867	8,167698	-0,07368867	0,950205415	Dcps
17345150	3,526578	3,523706	3,325958	3,423576	3,375814	3,355837	3,4587473	3,385075667	-0,07367167	0,950216612	---
17350695	5,615852	5,249656	5,584792	5,425493	5,302032	5,501765	5,4834333	5,409763333	-0,07367	0,950217709	---
17252495	4,118317	4,525776	4,249716	4,133243	4,681194	3,858422	4,2979363	4,224286333	-0,07365	0,950230882	---
17321806	5,405772	5,556667	5,473415	5,442917	5,548764	5,223225	5,478618	5,404968667	-0,07364933	0,950231321	I730030J21Rik
17504393	4,730061	4,683145	4,697632	4,636205	4,617688	4,635997	4,7036127	4,629963333	-0,07364933	0,950231321	4933400L20Rik
17325291	9,159005	9,19829	9,025318	8,898325	9,045193	9,218155	9,1275377	9,053891	-0,07364667	0,950233078	Kpna1
17295685	8,45608	8,24925	8,137191	8,305132	8,228031	8,088475	8,2808403	8,207212667	-0,07362767	0,950245592	Marveld2
17390718	5,745324	5,504671	6,07863	5,879886	5,682383	5,545499	5,7762083	5,702589333	-0,073619	0,950251301	Patl2
17461469	4,03603	3,918494	3,932818	3,739055	3,95956	3,96787	3,9624473	3,888828333	-0,073619	0,950251301	---
17315960	9,860132	9,884756	9,789785	9,705796	9,79574	9,812321	9,844891	9,771285667	-0,07360533	0,950260303	Brix1
17201131	8,10941	8,331017	8,555472	7,967189	8,47548	8,332435	8,3319663	8,258368	-0,07359833	0,950264913	---
17291559	7,105968	7,418247	7,038815	6,987204	7,145558	7,209476	7,1876767	7,114079333	-0,07359733	0,950265572	E2f3
17308153	6,03063	6,011482	6,200968	5,891995	6,038377	6,091951	6,0810267	6,007441	-0,07358567	0,950273256	Synb
17501877	6,278109	5,926024	6,0321	6,242263	5,940473	5,832748	6,0787443	6,005161333	-0,073583	0,950275013	Homer3
17549530	4,570469	4,510143	4,688569	4,527885	4,589379	4,431196	4,589727	4,516153333	-0,07357367	0,950281161	---
17549682	4,570469	4,510143	4,688569	4,527885	4,589379	4,431196	4,589727	4,516153333	-0,07357367	0,950281161	---
17302709	6,083269	5,894312	5,791858	6,019741	5,953161	5,575835	5,9231463	5,849579	-0,07356733	0,950285332	---
17535707	5,284655	5,009163	4,774569	5,084884	4,515488	5,247364	5,0227957	4,949245333	-0,07355033	0,95029653	---
17518807	3,669577	3,961805	3,934036	3,766656	3,791935	3,786189	3,8551393	3,781593333	-0,073546	0,950299384	---
17205043	7,276176	7,001215	6,943385	7,059443	7,036863	6,903888	7,073592	7,000064667	-0,07352733	0,95031168	---
17209233	7,276176	7,001215	6,943385	7,059443	7,036863	6,903888	7,073592	7,000064667	-0,07352733	0,95031168	---
17231869	4,193597	4,179182	4,045311	4,210099	4,066565	3,920878	4,1393633	4,065847333	-0,073516	0,950319146	Il22ra2
17546429	3,335978	3,526689	3,27964	3,290962	3,312973	3,317836	3,380769	3,307257	-0,073512	0,95032178	---
17224342	7,013617	7,320743	7,221017	7,02076	7,087811	7,226274	7,1851257	7,111615	-0,07351067	0,950322659	Rnf25
17539048	3,401781	3,280011	3,40895	3,310771	3,332826	3,226638	3,3635807	3,290078333	-0,07350233	0,950328148	Gm8578
17331036	8,18707	8,177921	8,077185	7,988222	8,133834	8,099617	8,147392	8,073891	-0,073501	0,950329026	Rg9mtd1
17421271	4,143053	4,120183	4,556826	4,069308	4,426514	4,103771	4,273354	4,199864333	-0,07348967	0,950336492	Oog2
17482503	8,275969	8,176155	8,245347	8,131822	8,198989	8,14624	8,2324903	8,159017	-0,07347333	0,950347251	BC030336
17549166	3,464914	3,456707	3,810462	3,392394	3,613293	3,505999	3,577361	3,503895333	-0,07346567	0,950352301	---
17485198	3,429343	3,479105	3,819956	3,340628	3,371934	3,795475	3,5761347	3,502679	-0,07345567	0,950358889	---
17330201	4,896333	4,756892	4,791021	4,915359	4,67445	4,634071	4,8147487	4,741293333	-0,07345533	0,950359108	---
17494697	4,22858	4,044997	4,20857	4,03125	4,35406	3,876522	4,1607157	4,087277333	-0,07343833	0,950370307	Olfr699
17321227	7,652433	7,797628	7,827137	7,732134	7,564892	7,759944	7,759066	7,685656667	-0,07340933	0,950389411	Kansl2
17238061	6,770755	6,659137	6,857487	6,710265	6,712803	6,644084	6,7624597	6,689050667	-0,073409	0,95038963	---
17218027	9,753055	9,860498	9,678163	9,560467	9,734714	9,776324	9,7639053	9,690501667	-0,07340367	0,950393144	Uchl5
17278335	4,206085	4,142447	4,162549	4,106053	3,98517	4,199708	4,1703603	4,096977	-0,07338333	0,950406539	---
17306114	2,868234	2,796205	2,945511	2,686069	2,935627	2,768105	2,8699833	2,796600333	-0,073383	0,950406758	---
17218168	8,88891	8,807981	8,560547	8,611151	8,646919	8,779375	8,7524793	8,679148333	-0,073331	0,950441015	1190005F20Rik
17386347	9,957889	9,888014	9,942166	9,851482	9,868649	9,84796	9,9293563	9,856030333	-0,073326	0,950444309	Tlk1
17348626	4,753607	4,36097	4,848953	4,578108	4,675992	4,489488	4,65451	4,581196	-0,073314	0,950452214	8430422H06Rik
17317622	5,746866	5,901355	5,518297	5,695942	5,558025	5,692664	5,7221727	5,648877	-0,07329567	0,950464293	Tmem71
17397968	6,782752	6,900942	6,587345	6,816196	6,606998	6,627969	6,757013	6,683721	-0,073292	0,950466708	Arhgef26
17494725	3,908408	3,705118	3,66921	3,659339	3,687511	3,716012	3,760912	3,687620667	-0,07329133	0,950467147	Olfr2
17223581	4,712158	4,633668	4,622958	4,588864	4,607043	4,553028	4,6562613	4,582978333	-0,073283	0,950472638	Ica1l
17427164	5,919764	5,834824	5,918562	5,772659	5,814688	5,866001	5,89105	5,817782667	-0,07326733	0,950482959	Elavl2
17250930	4,226278	4,065049	4,296961	3,900212	4,420303	4,048097	4,196096	4,122870667	-0,07322533	0,95051063	9130409J20Rik
17280721	4,740441	4,510539	5,0009	4,602538	4,725234	4,704453	4,7506267	4,677408333	-0,07321833	0,950515242	---
17538751	11,23623	11,28739	11,25356	11,10592	11,15683	11,29482	11,25906	11,18585667	-0,07320333	0,950525125	---
17291012	13,26911	13,20641	13,27293	13,12665	13,20772	13,19451	13,249483	13,17629333	-0,07319	0,95053391	Hist1h2an
17203863	3,19671	2,910194	3,221309	2,963396	2,937029	3,208266	3,1094043	3,036230333	-0,073174	0,950544451	---
17348105	7,578935	7,673323	7,191751	7,420895	7,441741	7,36186	7,4813363	7,408165333	-0,073171	0,950546428	Bambi
17419222	8,551239	8,691265	8,595358	8,470463	8,686163	8,461723	8,6126207	8,539449667	-0,073171	0,950546428	Snrnp40
17493611	4,447303	4,659468	4,553891	4,392265	4,518752	4,530217	4,553554	4,480411333	-0,07314267	0,950565096	---
17313643	5,648387	5,988543	5,822121	5,696549	5,731458	5,811625	5,8196837	5,746544	-0,07313967	0,950567073	7530414M10Rik
17290155	7,234089	7,33989	7,54704	7,199586	7,428219	7,273803	7,373673	7,300536	-0,073137	0,95056883	3110070M22Rik
17511482	6,234424	6,464729	6,25611	6,325342	6,143349	6,267177	6,318421	6,245289333	-0,07313167	0,950572344	Zfp423
17283931	5,339836	5,205788	5,549781	4,917366	5,499363	5,459333	5,365135	5,292020667	-0,07311433	0,950583765	---
17406241	5,786724	5,679111	5,786377	5,464315	5,733615	5,834973	5,7507373	5,677634333	-0,073103	0,950591232	Rbm46
17533199	4,815644	5,017759	5,187159	4,971434	5,058238	4,771593	5,006854	4,933755	-0,073099	0,950593868	Lancl3
17503208	8,261737	8,428457	8,341042	8,210534	8,308696	8,292809	8,3437453	8,270679667	-0,07306567	0,950615831	Lyl1
17463298	6,759161	6,425664	7,241927	6,881467	7,005731	6,320368	6,8089173	6,735855333	-0,073062	0,950618247	---
17203505	4,452839	4,104558	4,824273	4,816309	4,047838	4,298391	4,4605567	4,387512667	-0,073044	0,950630108	---
17468900	4,457124	4,18601	4,348918	4,120197	4,179644	4,47314	4,330684	4,257660333	-0,07302367	0,950643506	Ccdc37
17362503	8,257257	8,385442	8,220056	8,324892	8,130105	8,188734	8,287585	8,214577	-0,073008	0,95065383	Ttc9c

17245663	8,069498	7,093237	7,289925	7,451821	7,523778	7,258039	7,48422	7,411212667	-0,07300733	0,950654269	---
17285829	6,623065	6,861966	6,500155	6,397065	6,631302	6,737829	6,6617287	6,588732	-0,07299667	0,950661298	Hist1h1d
17480407	8,87519	8,938779	9,016158	8,777916	8,935294	8,897931	8,9433757	8,870380333	-0,07299533	0,950662176	Clns1a
17455639	4,449352	4,571803	4,374146	4,263869	4,608393	4,304064	4,4651003	4,392108667	-0,07299167	0,950664592	---
17343550	3,294206	3,454132	3,545697	3,257274	3,572053	3,245741	3,431345	3,358356	-0,072989	0,95066635	Gm4461
17483133	5,288934	5,176536	4,916852	4,857708	5,219242	5,086473	5,1274407	5,054474333	-0,07296633	0,950681286	Fam57b
17210429	3,307562	3,789916	3,471795	3,237421	3,172852	3,940115	3,523091	3,450129333	-0,07296167	0,950684361	---
17333482	7,079779	7,226561	7,288979	7,057546	7,231336	7,087564	7,1984397	7,125482	-0,07295767	0,950686997	1600012H06Rik
17302634	5,612947	6,052748	5,528838	5,575	5,623117	5,777573	5,731511	5,658563333	-0,07294767	0,950693587	Mir18
17409816	5,386003	5,402849	5,399274	5,490688	5,055623	5,423004	5,396042	5,323105	-0,072937	0,950700616	D3Bwg0562e
17293165	5,317654	5,448454	5,410958	5,356482	5,35286	5,248929	5,3923553	5,319423667	-0,07293167	0,95070413	Slc28a3
17540544	8,488334	8,638722	8,437537	8,386868	8,409895	8,549115	8,521531	8,448626	-0,072905	0,950721703	Uxt
17283942	8,172537	8,858049	8,661262	8,571203	8,367873	8,534063	8,5639493	8,491046333	-0,072903	0,950723021	---
17480190	3,97879	4,176103	3,668445	3,512155	3,888777	4,203703	3,9411127	3,868211667	-0,072901	0,950724339	---
17284423	4,03528	3,73607	3,987608	3,956506	3,795701	3,788145	3,9196527	3,846784	-0,07286867	0,950745647	Ighg
17327502	3,878474	4,09652	4,078912	4,059601	4,015212	3,760497	4,0179687	3,945103333	-0,07286533	0,950747844	Sh3bgr // Sh3bgr
17309688	9,613515	9,50655	9,489093	9,415667	9,554087	9,420847	9,536386	9,463533667	-0,07285233	0,950756411	A2ld1
17331238	3,988354	3,86614	3,510127	3,873899	3,514179	3,758011	3,788207	3,715363	-0,072844	0,950761902	Olfr186
17231676	8,91584	9,200294	8,743486	8,748509	8,880405	9,012194	8,9532067	8,880369333	-0,07283733	0,950766296	Timm8a1
17531418	4,050192	3,936403	4,076698	4,10007	3,782982	3,961738	4,0210977	3,948263333	-0,07283433	0,950768273	Fbxw24
17231574	9,3995	9,583626	9,519738	9,402117	9,387814	9,49446	9,5009547	9,428130333	-0,07282433	0,950774863	Ppil4
17292536	4,342224	4,196265	4,254586	4,18442	4,220573	4,169631	4,2643583	4,191541333	-0,072817	0,950779696	4930518P08Rik
17548829	3,293263	3,379993	3,261851	3,225866	3,147887	3,342087	3,3117023	3,238913333	-0,072789	0,950798149	Gm4916
17313260	7,475421	7,634131	7,486812	7,488938	7,374837	7,51423	7,5321213	7,459335	-0,07278633	0,950799907	Xpnpep3
17499610	3,689194	3,688828	3,788138	3,506455	3,822618	3,618738	3,7220533	3,649270333	-0,072783	0,950802103	Defb5
17422988	6,311831	6,920937	6,384203	6,245938	6,649862	6,502866	6,5389903	6,466222	-0,07276833	0,950811769	Rps20
17309011	3,880103	3,693065	3,591201	3,61779	3,683345	3,644931	3,7214563	3,648688667	-0,07276767	0,950812209	---
17547709	7,656933	7,546492	7,663739	7,567579	7,527573	7,553749	7,622388	7,549633667	-0,07275433	0,950820996	---
17307215	8,496459	8,637752	8,475246	8,573364	8,437026	8,380811	8,5364857	8,463733667	-0,072752	0,950822534	Zdhhc20
17340148	6,64918	6,485861	6,443014	6,213194	6,72792	6,418699	6,5260183	6,453271	-0,07274733	0,95082561	1700106N22Rik
17330451	3,676758	3,933995	3,620579	3,547297	4,043555	3,422307	3,7437773	3,671053	-0,07272433	0,950840768	4932412D23Rik
17210745	2,882504	2,903982	3,290946	3,159143	2,914405	2,785776	3,0258107	2,953108	-0,07270267	0,950855048	---
17351655	5,31883	5,458052	5,386839	5,583884	5,197055	5,164737	5,387907	5,315225333	-0,07268167	0,950868889	Stard6
17406397	4,290134	4,268974	4,654234	4,15552	4,43911	4,400683	4,4044473	4,331771	-0,07267633	0,950872404	Dear1
17268356	8,139583	8,121927	8,191936	8,029173	8,176975	8,029331	8,1511487	8,078493	-0,07265567	0,950886026	Nfe2l1
17439003	7,13853	6,98262	6,828024	6,891932	7,015199	6,824085	6,983058	6,910405333	-0,07265267	0,950888003	Mthfd2l
17458454	8,14442	8,237037	8,237934	8,173023	8,036238	8,192173	8,2064637	8,133811333	-0,07265233	0,950888223	2410003K15Rik
17259585	10,68914	10,59221	10,52658	10,46963	10,57524	10,54515	10,602643	10,53000667	-0,07263667	0,950898549	Narf
17538990	5,42236	5,272824	5,043428	5,21515	5,072011	5,233545	5,246204	5,173568667	-0,07263533	0,950899427	Rragb
17491329	5,394063	5,193718	4,930833	5,096822	5,071432	5,132474	5,1728713	5,100242667	-0,07262867	0,950903822	Mrgpra2b
17370735	5,018155	5,233523	5,248007	5,434567	5,10195	4,745372	5,1665617	5,093963	-0,07259867	0,950923595	Tnfaip6
17528405	9,144925	9,32339	9,068996	8,986118	9,156249	9,17718	9,1791037	9,106515667	-0,072588	0,950930626	Rab8b
17481508	3,950122	4,023263	4,233768	4,352133	3,654398	3,982866	4,069051	3,996465667	-0,07258533	0,950932384	Olfr17
17365148	4,937407	5,014863	4,993728	4,859023	4,903523	4,96571	4,9819993	4,909418667	-0,07258067	0,95093546	---
17525465	4,513897	4,414105	4,376524	4,545547	4,058689	4,482578	4,434842	4,362271333	-0,07257067	0,950942051	Gm5916
17308783	9,595545	10,06939	9,710184	9,544038	9,653925	9,959497	9,7917063	9,719153333	-0,072553	0,950953696	Gm10132 // Gm3362 // Rpl23a
17250597	6,610245	6,692726	7,003418	6,824007	6,607615	6,657126	6,7687963	6,696249333	-0,072547	0,950957651	B9d1
17493250	8,926051	8,88059	8,955685	8,747311	8,889148	8,908241	8,9207753	8,848233333	-0,072542	0,950960947	Eed
17429288	4,62396	4,291964	4,444434	4,662818	4,302423	4,177496	4,4534527	4,380912333	-0,07254033	0,950962045	Olfr1329
17450998	4,64396	4,685021	4,521154	4,456596	4,649939	4,526025	4,6167117	4,544186667	-0,072525	0,950972152	Pole
17358128	4,231044	4,282272	4,143312	4,299826	4,276891	3,862339	4,218876	4,146352	-0,072524	0,950972812	---
17550142	6,491131	6,521289	6,190996	6,504794	6,207487	6,273583	6,4011387	6,328621333	-0,07251733	0,950977206	---
17471889	3,281133	3,69798	3,422875	3,532496	3,501092	3,150859	3,4673293	3,394815667	-0,07251367	0,950979623	Tas2r106
17378387	4,934153	5,035356	4,899209	4,694378	5,002119	4,954717	4,9562393	4,883738	-0,07250133	0,950987753	---
17320538	5,774649	5,599061	5,967978	5,768713	5,790417	5,565068	5,7805627	5,708066	-0,07249667	0,950990829	Arsa
17220866	6,665001	6,72504	6,583745	6,607094	6,57434	6,574865	6,6579287	6,585433	-0,07249567	0,950991488	Rd3
17202559	3,744872	3,789298	4,097571	3,744872	3,69128	3,978138	3,877247	3,804763333	-0,07248367	0,950999398	---
17526982	6,216423	6,011169	6,159244	6,100341	5,849204	6,219886	6,1289453	6,056477	-0,07246833	0,951009506	Gm684
17224771	6,498528	6,755499	6,514765	6,578059	6,49479	6,47859	6,5895973	6,517146333	-0,072451	0,951020932	Serpine2
17508052	10,13639	9,936756	9,949621	9,914051	9,992192	9,899174	10,007589	9,935139	-0,07245	0,951021591	Vdac3
17405724	5,577307	5,473798	5,335695	5,293314	5,612599	5,263625	5,4622667	5,389846	-0,07242067	0,951040928	Shox2
17520716	4,123235	4,127661	3,973332	4,137735	3,933389	3,935868	4,0747427	4,002330667	-0,072412	0,951046641	1600029I14Rik
17348450	10,08348	10,17973	10,04602	10,0723	10,08022	9,939542	10,103077	10,03068733	-0,07238933	0,951061583	---
17220489	8,140223	8,426804	8,22803	8,184307	8,235773	8,157825	8,265019	8,192635	-0,072384	0,951065099	Rab3gap2
17362279	8,132191	8,163341	8,134743	8,156321	8,036173	8,020647	8,143425	8,071047	-0,072378	0,951069054	Naa40
17514899	3,702866	3,675447	3,225883	3,30717	3,411884	3,66803	3,534732	3,462361333	-0,07237067	0,951073889	Mbd3l1
17221360	10,23595	10,03762	9,788273	9,870458	9,836508	10,13782	10,020614	9,948262	-0,07235233	0,951085975	Lactb2
17390866	5,00231	4,780204	5,00463	4,9061	4,601509	5,062483	4,929048	4,856697333	-0,07235067	0,951087074	---
17355871	3,602498	3,572073	3,582464	3,484639	3,558398	3,496968	3,5856783	3,513335	-0,07234333	0,951091908	---
17422092	6,402147	6,334257	6,31386	6,122789	6,405936	6,304531	6,350088	6,277752	-0,072336	0,951096743	Tnfrsf25
17205731	6,385965	5,961802	6,165459	5,781952	6,850991	5,663337	6,1710753	6,09876	-0,07231533	0,951110367	---
17379378	3,785176	3,570496	3,777192	3,677824	3,526238	3,711924	3,7109547	3,638662	-0,07229267	0,95112531	Wfdc12
17430989	8,091156	7,995706	8,064343	8,061421	7,98876	7,884171	8,0504017	7,978117333	-0,07228433	0,951130804	Tmem222
17311907	3,869239	3,78636	3,839728	3,606999	3,780073	3,891501	3,8317757	3,759524333	-0,07225133	0,951152561	---
17531751	4,264939	4,20992	4,334661	4,214508	4,177052	4,201208	4,26984	4,197589333	-0,07225067	0,951153	Arpp21
17489052	6,384089	6,337584	6,585684	6,398411	6,347167	6,345057	6,4357857	6,363545	-0,07224067	0,951159593	Aplp1
17380544	3,225594	3,288751	3,344127	3,365989	3,06396	3,211833	3,2861573	3,213927333	-0,07223	0,951166626	---
17275604	4,852054	4,915083	4,970718	4,694335	5,009886	4,816954	4,9126183	4,840391667	-0,07222667	0,951168823	Pax9
17547925	3,496329	4,011868	3,884326	3,680606	3,649722	3,845516	3,7975077	3,725281333	-0,07222633	0,951169043	Gm7167
17547620	9,230474	9,551191	9,38851	9,232394	9,242252	9,478851	9,3900583	9,317832333	-0,072226	0,951169263	Ftl1 // Ftl1
17262549	9,999887	10,15011	10,19005	10,02898	10,09003	10,00438	10,113349	10,04113	-0,072219	0,951173878	---
17357229	9,257833	9,253899	8,991584	9,031187	9,103345	9,152129	9,167772	9,095553667	-0,07221833	0,951174317	---
17232549	3,717816	3,700257	3,841289	4,033519	3,495553	3,513651	3,7531207	3,680907667	-0,072213	0,951177834	Hs3st5
17405261	7,824916	7,583326	7,409528	7,615873	7,435681	7,549777	7,6059233	7,533777	-0,07214633	0,951221789	Rfxap
17449060	4,763497	4,962786	4,761588	4,705855	4,883429	4,682165	4,8292903	4,757149667	-0,07214067	0,951225525	---
17226550	10,39062	10,55392	10,48376	10,42535	10,37411	10,41243	10,4761	10,40396333	-0,07213667	0,951228162	Mcm6
17319045	6,019662	5,998749	6,172834	5,876918	6,078181	6,019748	6,0637483	5,991615667	-0,07213267	0,951230799	Elfn2
17321790	5,436162	5,488641	5,529375	5,498781	5,613863	5,125192	5,484726	5,412612	-0,072114	0,951243107	Galnt6
17536191	4,647531	4,828127	4,845942	4,618064	4,718915	4,768333	4,7738667	4,701770667	-0,072096	0,951254976	5430427O19Rik
17548879	7,667868	7,90139	7,930581	7,619631	7,792138	7,871831	7,8332797	7,7612	-0,07207967	0,951265745	---
17484098	9,097358	8,960803	9,25386	8,856547	9,103303	9,135992	9,104007	9,031947333	-0,07205967	0,951278933	---
17223766	7,578893	7,755986	7,532314	7,504025	7,616412	7,530612	7,6223977	7,550349667	-0,072048	0,951286625	Klf7
17541217	6,559806	6,698688	6,612535	6,559178	6,809976	6,285838	6,6236763	6,551664	-0,07201233	0,951310144	C1galt1c1
17329282	9,936064	9,996861	9,954418	9,870787	9,892221	9,90835	9,9624477	9,890452667	-0,071995	0,951321573	Tra2b
17360852	3,9142	3,760538	4,010359	3,860184	3,794019	3,814913	3,8950323	3,823038667	-0,07199367	0,951322453	2700089I24Rik
17224587	8,975561	8,971001	9,024475	8,860668	8,89987	8,994555	8,9903457	8,918364333	-0,07198133	0,951330585	Dnpep
17293186	3,805205	3,620415	3,829205	3,660457	3,627818	3,750613	3,7516083	3,679629333	-0,071979	0,951332124	4930455J16Rik
17427126	5,532939	5,497535	5,430785	5,341184	5,423469	5,48072	5,4870863	5,415124333	-0,071962	0,951343334	---
17516689	2,995589	2,863296	2,864868	2,857744	2,90371	2,746477	2,9079177	2,835977	-0,07194067	0,951357402	---
17428129	5,100321	5,117408	4,997958	4,899134	4,980608	5,120142	5,0718957	4,999961333	-0,07193433	0,951361578	Kti12 // Kti12
17370343	4,423024	4,019503	4,49681	4,237879	4,458289	4,027409	4,3131123	4,241192333	-0,07192	0,95137103	Olfr347
17294544	3,690258	3,633826	3,565987	3,315205	3,632257	3,726879	3,6300237	3,558113667	-0,07191	0,951377625	D13Mgi7
17259314	4,941015	4,575032	4,842607	4,37527	4,821601	4,946073	4,786218	4,714314667	-0,07190333	0,951382021	Fscn2
17205475	7,066549	7,030685	7,305928	7,355449	6,880095	6,951993	7,1343873	7,062512333	-0,071875	0,951400705	---
17330380	8,868286	8,926621	8,777349	8,743716	8,897048	8,715879	8,8574187	8,785547667	-0,071871	0,951403343	4930455C21Rik
17470672	4,113353	3,73578	3,814418	3,810585	3,87732	3,76007	3,8878503	3,815991667	-0,07185867	0,951411477	---
17481618	3,096824	2,979912	3,153284	2,946978	3,036049	3,031513	3,0766733	3,004846667	-0,07182667	0,95143258	Olfr497
17538972	3,771638	3,722455	3,794688	3,552329	3,713009	3,808058	3,762927	3,691132	-0,071795	0,951453464	---
17377793	9,883145	9,840414	9,82708	9,641632	9,779862	9,913773	9,850213	9,778422333	-0,07179067	0,951456321	Tpx2
17547797	3,483162	3,392729	3,433165	3,461112	3,28486	3,347719	3,436352	3,364563667	-0,07178833	0,95145786	Gm19471
17302711	4,696234	4,696622	4,624331	4,63541	4,677158	4,489275	4,6723957	4,600614333	-0,07178133	0,951462477	Cldn10
17213189	3,600904	3,877715	3,571507	3,702105	3,460447	3,672251	3,6833753	3,611601	-0,07177433	0,951467093	Gm20257
17343538	3,722675	3,83933	3,706343	3,706343	3,661686	3,685015	3,756116	3,684348	-0,071768	0,95147127	4921501E09Rik
17259220	6,526872	6,424148	6,745987	6,458284	6,659944	6,363492	6,565669	6,493906667	-0,07176233	0,951475007	Chmp6
17465971	5,606583	5,669625	5,705248	5,71226	5,441409	5,612573	5,6604853	5,588747333	-0,071738	0,951491056	D630045J12Rik
17207547	3,269543	3,946633	3,202962	3,246145	3,470888	3,486966	3,473046	3,401333	-0,071713	0,951507544	---
17305143	4,959459	5,017877	4,854872	5,130834	4,726038	4,760219	4,9440693	4,872363667	-0,07170567	0,951512381	Cdhr1
17396401	5,544508	5,063352	5,191661	5,34812	5,188317	5,047989	5,266507	5,194808667	-0,07169833	0,951517217	---
17326250	8,610844	8,543954	8,425411	8,46693	8,389291	8,508905	8,5267363	8,455042	-0,07169433	0,951519855	Zbtb11
17221061	3,987214	3,754861	3,702743	3,810572	3,78259	3,63659	3,8149393	3,743250667	-0,07168867	0,951523593	Rp1 // Rp1
17466845	5,500208	5,476598	5,70791	5,481397	5,342375	5,645905	5,561572	5,489892333	-0,07167967	0,951529529	Hoxa7
17238091	6,037852	6,268867	6,681878	5,936281	6,566278	6,271033	6,3295323	6,257864	-0,07166833	0,951537004	1700012D01Rik
17401985	2,984045	2,821051	3,031581	2,73112	2,925279	2,965312	2,945559	2,873903667	-0,07165533	0,951545578	---
17249923	7,777481	7,85702	7,090837	7,369981	7,457881	7,682513	7,5751127	7,503458333	-0,07165433	0,951546237	---
17515025	4,302718	4,698949	4,255931	4,240252	4,352529	4,449859	4,4191993	4,347546667	-0,07165267	0,951547337	---
17247993	9,799215	9,931016	9,91176	9,779388	9,788027	9,859686	9,8806637	9,809033667	-0,07163	0,951562287	Smek2
17211033	4,624762	4,503003	4,546766	4,495402	4,464036	4,500266	4,558177	4,486568	-0,071609	0,951576138	1700034P13Rik
17488556	5,877043	5,943216	5,936571	5,797465	6,019778	5,724875	5,9189433	5,847372667	-0,07157067	0,951601422	Gm19897
17416600	5,739624	5,755241	5,976624	5,74522	5,84733	5,664361	5,8238297	5,752303667	-0,071526	0,951630885	Slc1a7
17400519	6,843874	6,907855	7,030253	6,842101	6,869869	6,85545	6,9273273	6,855806667	-0,07152067	0,951634403	---
17283936	9,083094	9,134696	8,942578	8,906956	9,265666	8,773211	9,053456	8,981944333	-0,07151167	0,95164034	---
17313427	5,470912	5,694305	5,805503	5,376047	5,226326	6,153846	5,6569067	5,585406333	-0,07150033	0,951647815	---
17403823	3,941607	3,634853	4,035628	3,759411	3,89278	3,745404	3,870696	3,799198333	-0,07149767	0,951649574	9330178D15Rik
17264824	8,913385	8,950192	8,994552	8,88663	8,890558	8,866451	8,9527097	8,881213	-0,07149667	0,951650234	Mpdu1

17212694	3,980239	4,008514	3,895683	3,943283	3,858076	3,868602	3,9614787	3,889987	-0,07149167	0,951653532	---
17470988	7,268392	7,116916	6,976304	6,990021	7,12218	7,035002	7,1205373	7,049067667	-0,07146967	0,951668044	Ptms
17387776	3,713591	3,605483	3,535345	3,425011	3,639307	3,575783	3,6181397	3,546700333	-0,07143933	0,951688054	Olfr1138
17229715	8,261396	8,183871	8,015659	7,960097	8,121771	8,16477	8,153642	8,082212667	-0,07142933	0,95169465	Ufc1
17458924	4,571467	4,691699	5,085859	4,81663	4,670238	4,647887	4,7830083	4,711585	-0,07142333	0,951698608	Vmn1r4
17495304	10,77011	10,65389	10,1514	10,45297	10,48533	10,42286	10,525133	10,45372	-0,07141333	0,951705205	---
17400805	10,06906	10,16396	10,02817	10,16894	9,961519	9,916497	10,087063	10,015652	-0,07141133	0,951706524	---
17481721	3,268696	3,529223	3,622456	3,703059	3,35371	3,14941	3,4734583	3,402059667	-0,07139867	0,95171488	---
17231063	4,34447	4,662514	4,779014	4,423884	4,63717	4,510781	4,5953327	4,523945	-0,07138767	0,951722137	---
17484578	4,351954	4,457372	4,239586	4,256785	4,432871	4,145097	4,3496373	4,278251	-0,07138633	0,951723016	Olfr539
17520116	4,780025	4,319862	4,573181	4,263869	4,42741	4,767635	4,5576893	4,486304667	-0,07138467	0,951724116	---
17367555	7,661343	7,628063	7,349969	7,362017	7,530563	7,532677	7,5464583	7,475085667	-0,07137267	0,951732032	Pdss1
17243051	7,976532	7,995335	7,888124	7,839627	7,900559	7,905687	7,9533303	7,881957667	-0,07137267	0,951732032	Mob3a
17504761	5,426228	5,438696	5,470587	5,493647	5,328355	5,299392	5,4451703	5,373798	-0,07137233	0,951732252	Slc9a5
17414802	6,3993	6,152838	6,529248	6,369244	6,152609	6,34547	6,360462	6,289107667	-0,07135433	0,951744127	Pappa
17391442	7,893939	7,19004	7,175143	7,873578	7,211289	6,960254	7,4197073	7,348373667	-0,07133367	0,95175776	---
17484040	10,55683	10,49313	10,37338	10,35427	10,44068	10,4144	10,474447	10,40311667	-0,07133	0,951760179	Bub3
17471778	3,159408	3,318069	3,079054	3,020712	3,113863	3,207993	3,1855103	3,114189333	-0,071321	0,951766117	Klra3
17270162	11,98975	12,05851	11,97929	11,91322	11,88905	12,01132	12,009183	11,93786333	-0,07132	0,951766776	Slc4a1
17302586	7,383054	6,943039	7,405032	7,025493	7,103487	7,388205	7,2437083	7,172395	-0,07131333	0,951771175	Gm4666 // Gm4664 // Gm4663 // Gm93
17219612	6,667146	6,478949	6,93931	6,448123	6,90214	6,521227	6,695135	6,62383	-0,071305	0,951776672	Vsig8
17484104	10,38577	10,49049	10,32686	10,41877	10,46655	10,10391	10,40104	10,32974333	-0,07129667	0,95178217	---
17442237	5,138307	4,780675	4,637369	4,622642	4,934632	4,785236	4,852117	4,780836667	-0,07128033	0,951792945	Wdr66
17343079	6,999883	7,138144	7,073364	6,998366	7,031054	6,96817	7,0704637	6,999196667	-0,071267	0,951801742	1110021J02Rik
17226916	5,729012	5,594143	5,627926	5,50682	5,612051	5,618459	5,6503603	5,57911	-0,07125033	0,951812738	Nfasc
17513491	8,099238	8,038139	8,242972	8,13413	8,154919	7,877571	8,126783	8,05554	-0,071243	0,951817576	Cotl1
17280173	5,302516	5,059472	5,235259	5,283206	5,024557	5,075814	5,1990823	5,127859	-0,07122333	0,951830551	1700030C10Rik
17398043	4,03956	3,945416	4,160429	4,230492	3,890211	3,811063	4,0484683	3,977255333	-0,071213	0,951837369	Vmn2r1
17203871	3,199157	2,744296	3,195467	3,313982	2,885273	2,726057	3,0463067	2,975104	-0,07120267	0,951844186	---
17548452	5,386061	5,045916	4,752841	5,134838	4,709505	5,126921	5,061606	4,990421333	-0,07118467	0,951856062	LOC100505155
17238571	8,217237	8,326284	8,227055	8,10901	8,255256	8,192802	8,2568587	8,185689333	-0,07116933	0,951866179	Dnajc14
17404464	4,104648	4,133839	3,609415	3,860918	3,836694	3,936793	3,9493007	3,878135	-0,07116567	0,951868598	Tmem212
17391554	7,246272	7,836415	7,560749	7,717924	7,460347	7,251672	7,547812	7,476647667	-0,07116433	0,951869478	Il1a
17407335	9,521612	9,259431	9,594855	9,292733	9,573484	9,29635	9,4586327	9,387522333	-0,07111033	0,951905107	---
17207765	5,617983	3,216169	3,343697	3,559678	4,4048	4,000075	4,059283	3,988184333	-0,07109867	0,951912804	---
17550194	2,990891	3,001259	2,98341	2,869794	2,990256	2,902244	2,9918533	2,920764667	-0,07108867	0,951919403	---
17511077	3,523174	3,65427	3,669492	3,532824	3,81816	3,282754	3,6156453	3,544579333	-0,071066	0,951934359	Mir24-2 // Mir24-2
17250744	10,53854	10,89655	10,9584	10,74104	10,84834	10,59093	10,79783	10,72677	-0,07106	0,951938318	Snord65
17254121	5,051628	4,840837	5,044487	4,916149	4,916958	4,89087	4,978984	4,907992333	-0,07099167	0,951983407	Fndc8
17503287	10,64787	10,77976	10,81596	10,59531	10,69906	10,73632	10,747863	10,67689667	-0,07096667	0,951999904	Prdx2
17548330	10,31353	10,72818	10,54336	10,43381	10,38088	10,55752	10,528357	10,45740333	-0,07095333	0,952008702	---
17542047	5,383616	5,38234	5,259789	5,126034	5,328381	5,358509	5,341915	5,270974667	-0,07094033	0,952017281	Slitrk4
17482560	7,950355	7,99752	8,094601	8,015732	7,888858	7,925187	8,0141587	7,943259	-0,07089967	0,952044117	Eef2k
17210789	3,162449	3,142852	3,191024	2,799554	3,404315	3,079827	3,1654417	3,094565333	-0,07087633	0,952059515	---
17481797	4,005802	3,83968	3,756708	3,645565	3,992219	3,751813	3,8673967	3,796532333	-0,07086433	0,952067434	Mrvi1
17366940	6,169003	5,399044	5,498644	5,870594	5,394446	5,634063	5,688897	5,618034333	-0,07086267	0,952068534	---
17205047	3,713701	3,33673	3,183817	2,994179	3,875272	3,152232	3,411416	3,340561	-0,070855	0,952073593	---
17209237	3,713701	3,33673	3,183817	2,994179	3,875272	3,152232	3,411416	3,340561	-0,070855	0,952073593	---
17336896	9,226631	9,491337	9,428687	9,315162	9,356738	9,262228	9,3822183	9,311376	-0,07084233	0,952081952	Clic1
17506291	7,837604	7,681316	7,585712	7,406258	7,741417	7,744491	7,701544	7,630722	-0,070822	0,952095371	Foxf1
17336980	5,826904	6,132638	5,870798	6,045863	5,925056	5,646976	5,9434467	5,872631667	-0,070815	0,952099991	D17H6S53E
17306318	3,078362	3,15992	3,19192	3,060572	2,966972	3,190253	3,1434007	3,072599	-0,07080167	0,95210879	Olfr1510
17446627	8,548926	8,534012	8,734904	8,496031	8,717027	8,392384	8,6059473	8,535147333	-0,0708	0,95210989	Tyms // Tyms
17532272	4,038809	3,738064	3,801582	3,835079	3,784599	3,746392	3,859485	3,78869	-0,070795	0,95211319	Gm5922
17511788	4,63144	4,079598	4,767448	4,59408	4,313954	4,358126	4,4928287	4,422053333	-0,07077533	0,952126169	Ces5a
17343681	8,787239	8,694934	8,722881	8,853377	8,355206	8,784161	8,735018	8,664248	-0,07077	0,952129689	---
17548199	4,010834	4,161963	4,286339	4,179535	4,074617	3,992687	4,1530453	4,082279667	-0,07076567	0,952132548	Gm20099
17418119	4,847445	5,007661	5,102978	4,797345	4,965756	4,982725	4,986028	4,915275333	-0,07075267	0,952141128	Mycl1
17203899	5,09069	4,943497	5,085755	5,292817	4,347877	5,267063	5,0399807	4,969252333	-0,07072833	0,952157188	---
17549908	6,061221	6,147339	6,335257	5,92452	6,370901	6,036226	6,1812723	6,110549	-0,07072333	0,952160487	---
17439116	7,263778	6,819682	7,110857	7,031771	6,893593	7,056832	7,0647723	6,994065333	-0,070707	0,952171267	Fam47e // Fam47e
17450515	9,74257	10,11534	7,229656	7,427701	9,672335	9,775412	9,0291887	8,958482667	-0,070706	0,952171927	Gbp11
17457504	6,120109	5,688769	5,798961	5,765229	5,93925	5,691319	5,8692797	5,798599333	-0,07068033	0,952188867	Rab19
17444016	5,276218	5,038356	5,129354	5,14676	5,135489	4,949725	5,147976	5,077324667	-0,07065133	0,952208008	Gpr30
17297125	7,521089	7,389858	7,201406	7,320823	7,234863	7,344715	7,3707843	7,300133667	-0,07065067	0,952208448	Ptprg
17402678	5,47251	5,577497	5,725071	5,395391	5,550704	5,61708	5,5916927	5,521058333	-0,07063433	0,952219228	LOC100503594
17447096	10,92063	10,80611	11,1179	10,87398	10,97522	10,7836	10,948213	10,8776	-0,07061333	0,952233089	---
17340626	4,678588	4,555174	4,740165	4,521748	4,733439	4,506911	4,6579757	4,587366	-0,07060967	0,952235509	---
17306625	6,385739	6,529986	6,2808	6,430995	6,454127	6,099612	6,3988417	6,328244667	-0,070597	0,95224387	Zfhx2
17547892	8,309023	8,418074	8,274014	8,256557	8,365585	8,167253	8,3337037	8,263131667	-0,070572	0,952260371	4930529C04Rik // 4930529C04Rik
17283422	8,278295	8,338452	8,153708	8,07163	8,216076	8,271074	8,2568183	8,18626	-0,07055833	0,952269392	Atxn3
17502583	9,68733	9,888453	9,835068	9,712818	9,832135	9,654259	9,803617	9,733070667	-0,07054633	0,952277312	Mcm5
17550038	3,373325	2,961119	3,298767	3,296641	3,034315	3,090622	3,2110703	3,140526	-0,07054433	0,952278633	---
17476998	10,15486	10,45346	10,23478	10,07599	10,15886	10,39664	10,281033	10,21049667	-0,07053667	0,952283693	Hmgb1
17467015	6,581019	6,835471	6,749517	6,397599	6,687472	6,869358	6,7220023	6,651476333	-0,070526	0,952290734	Nod1
17418904	9,086244	9,181592	8,966483	8,988762	9,00054	9,033504	9,0781063	9,007602	-0,07050433	0,952305036	Ak2
17236922	6,704128	6,617938	6,643813	6,616254	6,487645	6,650476	6,655293	6,584791667	-0,07050133	0,952307016	Gm4302
17236924	6,704128	6,617938	6,643813	6,616254	6,487645	6,650476	6,655293	6,584791667	-0,07050133	0,952307016	Gm4302 // Gm4302
17240689	2,821051	2,991073	2,784333	2,759093	2,884574	2,741376	2,8654857	2,795014333	-0,07047133	0,952326819	---
17219451	10,44124	10,82974	10,50352	10,48686	10,61473	10,4615	10,5915	10,52103	-0,07047	0,952327699	---
17292477	7,22842	7,15288	7,095725	6,978495	7,052586	7,234537	7,1590083	7,088539333	-0,070469	0,952328359	Cenpp
17518799	8,196477	8,144099	8,237511	8,089466	8,17393	8,103319	8,1926957	8,122238333	-0,07045733	0,95233606	Rps27l
17267932	5,416203	5,134693	5,400894	5,223822	5,39112	5,125509	5,3172633	5,246817	-0,07044633	0,952343322	Spata20
17533937	8,990953	8,763601	8,712946	8,988297	8,403971	8,863894	8,8225	8,752054	-0,070446	0,952343542	Gm2799
17369974	7,564007	7,60122	7,520473	7,399709	7,695051	7,379622	7,5619	7,491460667	-0,07043933	0,952347942	Tor2a
17223880	7,51647	7,610585	7,397242	7,451392	7,377438	7,48419	7,508099	7,437673333	-0,07042567	0,952356964	Kansl1l
17534051	9,064476	9,443513	8,781441	8,926422	9,039672	9,112093	9,0964767	9,026062333	-0,07041433	0,952364445	Il13ra1
17542119	3,000931	2,985651	2,988404	2,864899	3,010878	2,888004	2,991662	2,921260333	-0,07040167	0,952372807	Mir509
17514330	12,56782	12,46112	12,13988	12,43761	12,24909	12,27096	12,389607	12,31922	-0,07038667	0,952382709	---
17387585	4,051659	4,135211	3,829373	4,105335	3,888937	3,810835	4,0054143	3,935035667	-0,07037867	0,95238799	Olfr993
17477946	5,488678	5,520204	5,498045	5,453937	5,313612	5,528307	5,502309	5,431952	-0,070357	0,952402294	Mamstr
17266107	6,801596	6,652622	6,654279	6,668768	6,729324	6,499336	6,7028323	6,632476	-0,07035633	0,952402734	Abr
17516005	3,626642	3,724355	3,702254	3,671958	3,603214	3,567043	3,684417	3,614071667	-0,07034533	0,952409995	---
17235539	8,969609	8,833745	9,029405	8,921281	8,872847	8,827601	8,944253	8,873909667	-0,07034333	0,952411316	Map2k2
17550663	6,201959	6,205474	6,415414	6,100034	6,342931	6,168888	6,2742823	6,203951	-0,07033133	0,952419238	---
17289132	4,952771	4,532882	5,122647	4,862768	4,64927	4,885367	4,8694333	4,799135	-0,07029833	0,952441023	Cmya5 // Cmya5
17361304	5,950703	6,388265	6,220073	5,983101	6,138088	6,226966	6,186347	6,116051667	-0,07029533	0,952443004	Lrfn4
17282740	6,195266	5,820149	6,262088	5,942576	5,988862	6,135254	6,092501	6,022230667	-0,07027033	0,952459509	---
17232417	5,566503	5,539342	5,448026	5,609947	5,408856	5,324276	5,517957	5,447693	-0,070264	0,95246369	9330159F19Rik
17206407	3,893025	4,47807	3,957704	3,845509	3,518579	4,75394	4,1095997	4,039342667	-0,070257	0,952468311	---
17311871	3,743752	3,780509	4,004402	3,729935	3,804808	3,783168	3,8428877	3,772637	-0,07025067	0,952472493	4930449C09Rik
17307247	3,324144	3,537248	3,548038	3,468945	3,362455	3,367288	3,46981	3,399562667	-0,07024733	0,952474693	1700129C05Rik
17348145	4,055615	3,972882	4,621495	4,208319	4,180023	4,050914	4,216664	4,146418667	-0,07024533	0,952476014	Gm10556
17548622	9,998069	10,00177	10,07303	9,795326	10,0188	10,04813	10,02429	9,954085333	-0,07020433	0,952503083	Fam103a1
17303665	4,151208	3,661154	3,879554	3,9463	3,812485	3,722571	3,8973053	3,827118667	-0,07018667	0,952514747	Gm9637
17461448	4,560807	3,915188	4,13515	4,272668	4,064538	4,06343	4,203715	4,133545333	-0,07016967	0,952525971	Grm7
17269200	7,964032	7,902794	7,764987	7,906079	7,823667	7,691626	7,877271	7,807124	-0,070147	0,952540936	---
17335165	6,717315	6,421534	6,961824	6,761375	6,66586	6,463027	6,7002243	6,630087333	-0,070137	0,952547539	---
17267484	7,258799	7,223106	7,088366	7,08438	7,098616	7,176926	7,1900903	7,119974	-0,07011633	0,952561184	Rad51c
17527806	7,939721	8,007294	8,232938	8,074408	7,912961	7,982239	8,0599843	7,989869333	-0,070115	0,952562064	---
17405612	4,70444	4,359828	4,430677	4,522909	4,452239	4,309484	4,498315	4,428210667	-0,07010433	0,952569107	Vmn2r3
17464886	4,348025	4,244286	4,215088	4,094934	4,257895	4,244286	4,269133	4,199038333	-0,07009467	0,95257549	Vwde
17543131	3,892191	3,242653	3,712805	3,714299	3,566649	3,356486	3,615883	3,545811333	-0,07007167	0,952590676	Mageb5
17256579	4,852583	4,883974	4,97292	4,896334	4,794019	4,808953	4,903159	4,833102	-0,070057	0,952600361	Cntnap1
17468713	6,961949	7,204453	7,646088	7,435452	6,90594	7,260962	7,27083	7,200784667	-0,07004533	0,952608064	---
17243888	6,349065	6,592107	6,544236	6,450863	6,115153	6,709267	6,495136	6,425094333	-0,07004167	0,952610485	Chpt1
17332336	10,09102	10,19745	10,32045	10,27188	10,04442	10,08262	10,202973	10,13297333	-0,07	0,952637998	---
17342219	8,734571	8,748308	8,614	8,563833	8,723865	8,599192	8,6989597	8,628963333	-0,06999633	0,952640419	Ube2i
17372936	3,228126	3,194691	3,336592	3,215225	3,216884	3,117367	3,2531363	3,183158667	-0,06997767	0,952652745	Olfr1257
17448712	7,780282	7,513736	7,327559	7,481356	7,484569	7,445758	7,5405257	7,470561	-0,06996467	0,95266133	Tec
17511130	6,652319	7,05615	6,835062	6,597085	6,964236	6,772331	6,8478437	6,777884	-0,06995967	0,952664631	Ier2
17290967	4,735569	4,559065	4,702879	4,6785	4,54973	4,559464	4,6658377	4,595898	-0,06993967	0,952677838	Zfp389
17411054	9,3904	9,400874	9,230474	9,130838	9,30104	9,380052	9,3405827	9,270643333	-0,06993933	0,952678058	Rpf1
17247691	5,009067	4,799743	4,78542	4,88666	4,808048	4,689708	4,8647433	4,794805333	-0,069938	0,952678939	1700061J23Rik
17432572	4,000711	3,81575	4,266654	4,066651	3,9774	3,829269	4,027705	3,957773333	-0,06993167	0,952683121	Gm13124
17325849	7,748719	7,910925	7,799586	7,733783	7,710601	7,805089	7,8197433	7,749824333	-0,069919	0,952691485	BC027231
17481139	4,813787	3,971617	4,230681	4,488443	4,146251	4,171647	4,338695	4,268780333	-0,06991467	0,952694347	Olfr556
17221122	4,181254	4,372888	4,452976	4,344481	4,153238	4,299664	4,335706	4,265794333	-0,06991167	0,952696328	Sntg1
17223313	7,384756	7,370448	7,251512	7,284917	7,293867	7,218204	7,335572	7,265662667	-0,06990933	0,952697869	1110034B05Rik
17537975	3,924387	4,003725	3,407762	3,707177	3,631889	3,787081	3,7786247	3,708715667	-0,069909	0,952698089	---
17218845	6,657276	6,039289	6,094335	6,064493	6,330586	6,186096	6,2636333	6,193725	-0,06990833	0,952698529	Selp
17202547	6,509306	6,451222	6,349892	5,974638	6,569436	6,556628	6,4368067	6,366900667	-0,069906	0,95270007	---
17298729	2,839454	2,939525	2,975183	2,873871	2,836185	2,834393	2,918054	2,848149667	-0,06990433	0,952701171	---
17407363	11,85946	11,91086	11,78088	11,74036	11,92211	11,67903	11,8504	11,7805	-0,0699	0,952704032	S100a9

17437117	4,035738	4,270428	4,117027	4,19037	4,212667	3,810457	4,1410643	4,071164667	-0,06989967	0,952704252	---
17532597	13,28739	13,40685	13,38486	13,29557	13,22393	13,34992	13,3597	13,28980667	-0,06989333	0,952708435	---
17308160	4,559496	4,429662	4,586652	4,372286	4,544499	4,449352	4,52527	4,455379	-0,069891	0,952709975	Gm16677
17480018	9,174953	9,304103	9,010124	9,008642	9,092453	9,178481	9,16306	9,093192	-0,069868	0,952725164	Ctsc
17455752	7,678468	7,59871	7,494099	7,549765	7,532216	7,479722	7,5904257	7,520567667	-0,069858	0,952731768	Ccdc132
17240691	3,897973	3,651499	3,270892	3,621133	3,403245	3,586464	3,606788	3,536947333	-0,06984067	0,952743215	C130030K03Rik
17207781	3,973616	3,836678	3,689767	4,20174	3,497545	3,591313	3,8333537	3,763532667	-0,069821	0,952756202	---
17335338	5,163826	4,786092	5,505333	5,49983	4,691767	5,054196	5,1517503	5,081931	-0,06981933	0,952757303	---
17419831	8,994385	9,171475	9,147822	8,991173	9,029588	9,083517	9,1045607	9,034759333	-0,06980133	0,95276919	Syf2
17205767	3,33391	3,380339	3,724431	3,49713	3,580855	3,151355	3,47956	3,40978	-0,06978	0,952783279	---
17384632	8,555985	8,559754	8,334445	8,455233	8,303884	8,481747	8,4833947	8,413621333	-0,06977333	0,952787682	Strbp
17389645	8,60679	8,879007	8,802999	8,623237	8,619873	8,836378	8,762932	8,693162667	-0,06976933	0,952790324	---
17347074	6,076146	5,761006	5,970382	5,939336	5,792254	5,866641	5,9358447	5,866077	-0,06976767	0,952791424	---
17234803	8,569603	8,622255	8,37642	8,363276	8,511913	8,48383	8,5227593	8,453006333	-0,069753	0,952801111	Cstb
17545148	4,748463	4,644285	4,609854	4,856898	4,355637	4,580813	4,667534	4,597782667	-0,06975133	0,952802211	Mageb16-ps1
17268898	6,335147	6,353534	6,482884	6,409952	6,267578	6,284822	6,3905217	6,320784	-0,06973767	0,952811237	Msl1
17337945	4,445432	4,549531	4,608803	4,355702	4,588799	4,450076	4,5345887	4,464859	-0,06972967	0,952816521	Slc29a1
17204179	5,47482	4,964748	4,946623	4,982341	5,328728	4,865973	5,1287303	5,059014	-0,06971633	0,952825327	---
17226166	6,46298	6,534211	6,48987	6,510425	6,443411	6,3241	6,495687	6,425978667	-0,06970833	0,95283061	2900060B14Rik
17472185	8,40081	8,811076	8,564702	8,594835	8,458608	8,51412	8,592196	8,522521	-0,069675	0,952852626	Art4
17226098	3,015555	2,873888	3,013781	2,94203	2,741336	3,010885	2,9677413	2,898083667	-0,06965767	0,952864074	Serpinb3b
17447146	7,482946	8,022328	7,646446	7,713554	7,572814	7,656384	7,71724	7,647584	-0,069656	0,952865175	---
17337631	3,607251	4,111004	3,778013	3,604561	3,753544	3,929207	3,8320893	3,762437333	-0,069652	0,952867817	Olfr134
17206991	4,077052	3,849685	4,039637	4,108672	4,109075	3,539678	3,9887913	3,919141667	-0,06964967	0,952869358	---
17202903	6,271104	5,858298	6,594882	6,245202	5,569507	6,700649	6,241428	6,171786	-0,069642	0,952874421	---
17228353	7,711408	7,509697	7,4814	7,47851	7,331565	7,683568	7,5675017	7,497881	-0,06962067	0,952888512	Mr1
17536906	5,071808	4,156938	4,146398	4,254059	4,340156	4,57208	4,4583813	4,388765	-0,06961633	0,952891374	---
17254508	5,64964	5,273103	5,717953	5,40554	5,617495	5,40885	5,5468987	5,477295	-0,06960367	0,95289974	Car4
17321758	6,947833	6,446327	6,788118	6,907753	6,479883	6,585837	6,727426	6,657824333	-0,06960167	0,952901061	C330013E15Rik
17387473	9,521611	9,481406	9,358837	9,291213	9,339565	9,522287	9,4539513	9,384355	-0,06959633	0,952904584	Tmx2
17232049	4,938092	4,976864	5,323803	5,210512	4,879428	4,940032	5,0795863	5,009990667	-0,06959567	0,952905024	Gm5420
17203069	5,871336	5,12402	5,937826	5,510282	5,134247	6,079885	5,644394	5,574804667	-0,06958933	0,952909207	---
17337953	4,751372	4,868462	5,043647	5,24008	4,537694	4,676993	4,887827	4,818255667	-0,06957133	0,952921097	E030047D23Rik // E030047D23Rik
17294934	5,459009	5,438124	5,299244	5,252215	5,453991	5,281463	5,3987923	5,329223	-0,06956933	0,952922418	Thbs4
17349016	6,778025	7,038964	6,714716	6,633667	6,848908	6,840454	6,8439017	6,774343	-0,06955867	0,952929463	Mocos
17524847	7,928546	7,965189	8,179188	7,86797	8,036954	7,959335	8,0243077	7,954753	-0,06955467	0,952932105	Epor
17235556	7,300833	7,338458	7,362459	7,250468	7,318641	7,223987	7,3339167	7,264365333	-0,06955133	0,952934307	Zbtb7a
17328459	7,852866	7,939156	7,657423	7,776297	7,727048	7,73749	7,8164817	7,746945	-0,06953667	0,952943995	2310008H04Rik
17447036	4,597349	5,18572	5,080306	4,958937	4,644985	5,0509	4,9544583	4,884940667	-0,06951767	0,952956545	E230008O15Rik // E230008O15Rik
17464941	3,001589	3,053274	3,151244	2,818868	3,135633	3,04306	3,0687023	2,999187	-0,06951533	0,952958086	---
17284243	8,901733	9,139451	9,219733	9,069007	9,099518	8,883896	9,0869723	9,017473667	-0,06949867	0,952969095	Cdca4
17375833	7,537691	7,562024	7,41946	7,426633	7,45239	7,43172	7,5063917	7,436914333	-0,06947733	0,952983187	Tmem127
17456841	8,401069	8,266684	8,322022	8,219588	8,355858	8,205938	8,329925	8,260461333	-0,06946367	0,952992215	Klhdc10
17379187	7,91663	7,973957	7,854125	7,902098	7,87491	7,759326	7,914904	7,845444667	-0,06945933	0,952995077	Ift52
17431440	6,135665	6,233543	6,44682	6,139865	6,179036	6,288756	6,2720093	6,202552333	-0,069457	0,952996618	Rcan3
17551075	9,425539	9,366615	9,292612	9,308461	9,245418	9,322289	9,3614887	9,292056	-0,06943267	0,953012692	---
17247882	6,8715	6,79775	6,832376	6,971926	6,725015	6,596404	6,8338753	6,764448333	-0,069427	0,953016436	---
17519794	10,30859	10,24917	10,2176	10,22294	10,14684	10,19735	10,258453	10,18904333	-0,06941	0,953027666	Senp6
17536997	4,71177	4,540565	4,788492	4,665285	4,353541	4,813812	4,6802757	4,610879333	-0,06939633	0,953036694	Zcchc13
17489420	10,30413	10,02073	9,933818	10,137	9,92131	9,992234	10,086226	10,016848	-0,069378	0,953048805	Fxyd1
17504023	5,32643	5,704866	5,294909	5,65108	5,264332	5,202705	5,4420683	5,372705667	-0,06936267	0,953058934	Nlrc5
17359934	8,699937	8,361308	8,393126	8,290887	8,372234	8,583165	8,4847903	8,415428667	-0,06936167	0,953059595	---
17471980	4,319874	4,262229	4,225206	4,268515	3,977998	4,35277	4,269103	4,199761	-0,069342	0,953072587	Kap
17408156	4,656393	4,326889	4,453878	4,502106	4,384873	4,342176	4,4790533	4,409718333	-0,069335	0,953077211	Gja8
17214759	7,96086	7,948106	8,096458	7,94152	8,014352	7,841578	8,001808	7,932483333	-0,06932467	0,953084037	Rhbdd1
17542419	6,289164	6,185343	6,143911	6,216764	6,236526	5,957158	6,2061393	6,136816	-0,06932333	0,953084918	L1cam
17247509	4,30045	4,073504	4,112546	4,155667	4,049678	4,073202	4,1621667	4,092849	-0,06931767	0,953088662	4933406G16Rik // 4933406G16Rik
17435702	7,074628	6,997352	6,860415	6,899986	6,95902	6,865541	6,977465	6,908182333	-0,06928267	0,953111784	Dnajb6
17309653	5,352475	5,433846	4,947566	5,301827	5,086806	5,137414	5,244629	5,175349	-0,06928	0,953113546	A330035P11Rik
17275392	6,331595	6,457986	6,375434	6,128256	6,417268	6,411723	6,3883383	6,319082333	-0,069256	0,953129402	Ap4s1
17447979	7,801466	7,57209	7,697845	7,633987	7,571089	7,65858	7,690467	7,621218667	-0,06924833	0,953134467	Gpr125
17309905	8,388009	9,085268	8,86831	8,762732	8,727965	8,643238	8,780529	8,711311667	-0,06921733	0,953154947	Snord72
17501283	4,679407	4,922467	4,636906	4,830432	4,61564	4,58506	4,74626	4,677044	-0,069216	0,953155828	Scrg1
17521794	9,057563	9,336478	9,236876	9,117855	9,188215	9,117254	9,2103057	9,141108	-0,06919767	0,953167941	Qars
17547785	4,258551	3,95823	3,984037	3,995224	3,925977	4,07203	4,0669393	3,997743667	-0,06919567	0,953169262	Gm20064
17227278	7,511328	7,311227	7,394822	7,239226	7,438873	7,331735	7,4057923	7,336611333	-0,069181	0,953178952	Rnpep
17462310	6,690205	6,446382	6,577486	6,74534	6,29232	6,468908	6,5713577	6,502189333	-0,06916833	0,953187321	Ninj2
17314738	7,839882	7,731467	7,61414	7,578171	7,749851	7,649981	7,7284963	7,659334333	-0,069162	0,953191506	Spats2
17363788	4,193544	3,993811	4,416279	4,039295	4,077068	4,279788	4,2012113	4,132050333	-0,069161	0,953192166	Gm9883 // Gm9883
17422992	3,939702	3,881344	3,820851	3,963261	3,736018	3,735137	3,8806323	3,811472	-0,06916033	0,953192607	Mos
17319806	8,077124	8,06575	8,008112	7,947614	8,030405	7,965502	8,0503287	7,981173667	-0,069155	0,95319613	Arfgap3
17459324	4,537776	4,62794	4,467828	4,596598	4,223678	4,60583	4,5445147	4,475368667	-0,069146	0,953202077	---
17378260	8,681239	8,794063	8,713029	8,628277	8,725831	8,626831	8,7294437	8,660313	-0,06913067	0,953212208	Chmp4b
17308182	7,956204	7,911427	8,102267	7,946732	7,844419	7,971385	7,989966	7,920845333	-0,06912067	0,953218815	Chmp7
17459895	11,77195	11,64005	11,68743	11,64782	11,61302	11,63124	11,69981	11,63069333	-0,06911667	0,953221458	---
17536005	8,51228	8,612456	8,39689	8,471821	8,432933	8,409534	8,5072087	8,438096	-0,06911267	0,953224101	Tbl1x
17482450	3,509607	3,482348	3,379573	3,306291	3,421247	3,436666	3,457176	3,388068	-0,069108	0,953227184	Abca16
17364187	5,457619	5,358984	5,568784	5,307012	5,516514	5,354601	5,4617957	5,392709	-0,06908667	0,95324128	---
17314375	5,520735	5,455568	5,567021	5,471799	5,335509	5,528791	5,5144413	5,445366333	-0,069075	0,953248988	Muc19 // Muc19
17308675	4,471582	4,528874	4,862557	4,524918	4,798693	4,332181	4,6210043	4,551930667	-0,06907367	0,953249869	Spert
17501544	4,42312	4,390562	4,574841	4,251992	4,489426	4,439885	4,462841	4,393767667	-0,06907333	0,95325009	Npy1r
17365384	9,35998	9,302145	9,16586	9,373697	9,092494	9,154641	9,275995	9,206944	-0,069051	0,953264846	Actr1a
17359486	2,939618	2,987709	3,061946	2,954589	2,983488	2,84405	2,9964243	2,927375667	-0,06904867	0,953266388	---
17280270	6,078799	5,997401	5,957236	5,877139	5,869431	6,079759	6,0111453	5,942109667	-0,06903567	0,953274978	Gm9292
17409284	5,538162	5,439795	5,939181	5,681732	5,460774	5,567575	5,639046	5,570027	-0,069019	0,953285991	Celsr2
17383127	8,092413	8,194403	8,366959	8,162102	8,326856	7,957788	8,217925	8,148915333	-0,06900967	0,953292158	Snora43
17533134	4,103924	3,994722	4,296084	4,686494	3,726526	3,774684	4,1315767	4,062568	-0,06900867	0,953292819	Ssxb1
17487218	7,319373	6,994918	6,985603	6,91583	6,998716	7,178328	7,0999647	7,030958	-0,06900667	0,95329414	Cd3eap
17548934	8,175696	8,292814	8,141184	8,171425	8,215111	8,016159	8,2032313	8,134231667	-0,06899967	0,953298766	Zbed4 // Zbed4
17225175	6,086284	5,960996	6,295552	6,049646	6,063621	6,022664	6,1142773	6,045310333	-0,068967	0,953320351	Nmur1
17474875	3,162263	3,180348	3,039038	3,102321	3,119709	2,952734	3,1272163	3,058254667	-0,06896167	0,953323875	V1rd19
17449417	4,447576	4,582561	4,161841	4,059951	4,510962	4,414203	4,397326	4,328372	-0,068954	0,953328941	Sult1e1
17247845	4,415815	4,306675	4,514557	4,350683	4,295907	4,383599	4,412349	4,343396333	-0,06895267	0,953329823	Papolg
17452719	5,853627	5,808679	5,921946	5,857364	5,734397	5,785638	5,8614173	5,792466333	-0,068951	0,953330924	Abcb9
17291812	5,061052	4,72944	4,990896	4,868341	4,898407	4,807825	4,9271293	4,858191	-0,06893833	0,953339294	1700026J04Rik
17289792	3,902798	3,573045	3,951446	3,891104	3,708079	3,621318	3,8090963	3,740167	-0,06892933	0,953345241	Mir1904
17203919	3,082483	4,487799	3,597428	3,490675	3,722361	3,747939	3,72257	3,653658333	-0,06891167	0,953356916	---
17237505	3,358216	3,264425	3,35778	3,322008	3,139082	3,312597	3,326807	3,257895667	-0,06891133	0,953357136	---
17426022	6,10136	5,59972	5,314841	5,807853	5,37319	5,628163	5,6719737	5,603068667	-0,068905	0,953361321	Mup15 // Mup2
17251251	5,631907	5,96744	6,056939	5,898674	5,695035	5,855904	5,8854287	5,816537667	-0,068891	0,953370573	Rcvrn
17221251	10,71598	10,79403	10,59978	10,66609	10,66536	10,57169	10,703263	10,63438	-0,06888333	0,953375639	---
17426076	3,841708	3,663729	3,543406	3,494704	3,631434	3,716123	3,6829477	3,614087	-0,06886067	0,953390618	Mup5
17507559	6,982169	7,014151	7,154238	7,020196	7,014064	6,90973	7,050186	6,98133	-0,068856	0,953393702	Grtp1
17502249	8,056904	8,201221	8,273046	8,132823	8,017494	8,174301	8,177057	8,108206	-0,068851	0,953397006	Pgls
17549126	3,595695	3,704713	3,5975	3,593215	3,568721	3,529438	3,632636	3,563791333	-0,06884467	0,953401191	---
17345109	6,198313	6,143418	6,341758	6,252167	6,208306	6,016502	6,2278297	6,158991667	-0,068838	0,953405597	Slc25a27
17222051	4,638337	4,581016	5,084258	4,86727	4,401864	4,828002	4,7678703	4,699045333	-0,068825	0,953414188	Gm15455 // Gm15455 // Gm15455
17549486	4,327927	4,055011	4,609374	4,241438	4,061934	4,482483	4,3307707	4,261951667	-0,068819	0,953418153	---
17549634	4,327927	4,055011	4,609374	4,241438	4,061934	4,482483	4,3307707	4,261951667	-0,068819	0,953418153	---
17406129	7,43677	7,023341	7,131626	7,165553	7,130792	7,088941	7,1972457	7,128428667	-0,068817	0,953419475	Tmem144
17324274	8,76811	8,970221	8,851274	8,826195	8,698953	8,858049	8,8632017	8,794399	-0,06880267	0,953428947	Senp2
17511277	8,435378	8,558622	8,519608	8,329639	8,466162	8,511419	8,504536	8,43574	-0,068796	0,953433353	Asna1
17494156	4,229723	3,911783	3,8234	3,93979	3,81928	3,999469	3,988302	3,919513	-0,068789	0,953437979	Olfr586
17231229	10,35115	10,38949	10,52947	10,40849	10,31829	10,33702	10,42337	10,3546	-0,06877	0,953450536	Hsd11b1
17531900	7,762594	8,000974	7,781813	7,756141	7,830989	7,75199	7,8484603	7,779706667	-0,06875367	0,95346133	Cnot10
17334861	6,838244	6,889349	7,338498	7,069341	6,98696	6,803546	7,0220303	6,953282333	-0,068748	0,953465075	Mrpl28
17351625	4,754847	4,652211	4,683668	4,725173	4,691483	4,467873	4,6969087	4,628176333	-0,06873233	0,953475429	---
17383366	5,565799	5,636667	5,621972	5,443221	5,655305	5,519725	5,608146	5,539417	-0,068729	0,953477632	---
17274120	11,21874	11,44241	11,32438	11,2762	11,35179	11,15139	11,32851	11,25979333	-0,06871667	0,953485784	---
17500005	9,573975	9,74586	9,765261	9,59551	9,561614	9,721823	9,695032	9,626315667	-0,06871633	0,953486004	Ank1
17472467	7,475467	7,4394	7,403471	7,366166	7,47095	7,275081	7,439446	7,370732333	-0,06871367	0,953487766	Recql
17287099	5,79619	5,498982	5,805001	5,683322	5,644889	5,565873	5,7000577	5,631361333	-0,06869633	0,953499222	---
17399044	7,902943	8,146467	8,033737	7,924697	7,973909	7,97846	8,0277157	7,959022	-0,06869367	0,953500985	Smg5
17201229	4,581214	4,559236	4,278093	4,322155	4,410627	4,479886	4,4728477	4,404222667	-0,068625	0,953546369	---
17407647	5,778426	5,688522	5,332115	5,453485	5,473537	5,666167	5,5996877	5,531063	-0,06862467	0,953546589	Pi4kb
17515551	6,536176	6,723535	6,611256	6,660681	6,465079	6,539339	6,6236557	6,555033	-0,06862267	0,953547911	B3gat1
17355460	5,250955	5,1116	5,068184	5,195028	4,966754	5,063096	5,1435797	5,074959333	-0,06862033	0,953549453	---
17228960	4,201092	4,121974	4,17768	4,222759	4,064578	4,007562	4,1669153	4,098299667	-0,06861567	0,953552537	7420461P10Rik
17277534	4,560388	4,507589	4,688939	4,650887	4,580296	4,319984	4,5856387	4,517055667	-0,068583	0,953574129	Gm6772
17526297	5,781002	5,754748	5,827127	5,884183	5,498625	5,774326	5,7876257	5,719044667	-0,068581	0,953575451	Upk2
17277907	9,530694	9,698266	9,730886	9,492261	9,633676	9,628181	9,653282	9,584706	-0,068576	0,953578756	Psmc1
17264777	6,944724	7,064801	7,166374	6,937444	7,110683	6,922058	7,058633	6,990061667	-0,06857133	0,95358184	Wrap53
17510057	6,827679	6,893413	6,952204	6,862844	6,772039	6,832702	6,8910987	6,822528333	-0,06857033	0,953582501	Klhl26
17232734	8,391102	8,30309	8,293791	8,200677	8,229706	8,351959	8,3293277	8,260780667	-0,068547	0,953597924	Cdk19

17467181	3,070189	2,875769	2,766901	2,888974	2,848073	2,770179	2,9042863	2,835742	-0,06854433	0,953599687	Vmn1r21
17465393	4,877123	4,873833	4,954484	4,79529	4,869277	4,835275	4,9018133	4,833280667	-0,06853267	0,953607398	---
17362895	3,682586	3,62763	3,406317	3,472421	3,805145	3,233373	3,5721777	3,503646333	-0,06853133	0,953608279	1700025F22Rik
17221396	6,439425	6,580273	6,363489	6,31491	6,59736	6,265337	6,4610623	6,392535667	-0,06852667	0,953611364	Msc
17475640	8,7874	8,426356	8,598707	8,582466	8,480009	8,544497	8,6041543	8,535657333	-0,068497	0,953630974	Akt2
17387987	3,939776	3,808712	3,410815	3,714462	3,743867	3,495568	3,7197677	3,651299	-0,06846867	0,953649702	Olfr1251
17489251	7,231587	7,491869	7,762478	7,464544	7,479631	7,336368	7,4953113	7,426847667	-0,06846367	0,953653007	Haus5
17222679	7,281679	7,039625	7,070685	7,003596	7,11373	7,069282	7,130663	7,062202667	-0,06846033	0,953655211	1700029F09Rik
17284474	4,738049	3,94876	3,986613	4,120691	4,207929	4,139534	4,224474	4,156051333	-0,06842267	0,95368011	---
17317574	4,458346	4,262339	4,301713	4,251688	4,269681	4,29577	4,3407993	4,272379667	-0,06841967	0,953682093	Hhla1
17423321	6,851321	6,94574	6,956553	6,839311	6,980318	6,728734	6,9178713	6,849454333	-0,068417	0,953683856	Pdp1
17292732	7,144852	7,024887	7,329969	7,068037	7,184281	7,042172	7,1665693	7,098163333	-0,068406	0,953691127	Rnf44
17235360	6,912969	6,624506	6,997764	6,728927	6,774511	6,826647	6,8450797	6,776695	-0,06838467	0,95370523	Onecut3
17376466	6,893904	6,811089	6,928521	6,83146	6,801145	6,795794	6,877838	6,809466333	-0,06837167	0,953713823	2310035K24Rik
17437097	5,537978	5,547925	5,677805	5,806975	5,281947	5,469682	5,5879027	5,519534667	-0,068368	0,953716247	Otop1
17542963	3,002145	2,848781	2,851068	2,716291	2,919848	2,860778	2,9006647	2,832305667	-0,068359	0,953722197	---
17320907	8,650902	8,707794	8,593039	8,563327	8,603104	8,580248	8,6505783	8,582226333	-0,068352	0,953726824	Slc38a1
17235835	6,165187	6,262267	6,312901	6,167763	6,207066	6,160498	6,246785	6,178442333	-0,06834267	0,953732994	Gm8290
17549036	3,453524	3,453524	3,488731	3,382496	3,354753	3,453524	3,4652597	3,396924333	-0,06833533	0,953737842	---
17361589	8,431363	8,206571	8,402934	8,172245	8,357538	8,306101	8,346956	8,278628	-0,068328	0,95374269	Drap1
17403237	6,391771	7,026964	5,575888	6,472147	6,138083	6,179423	6,331541	6,263217667	-0,06832333	0,953745775	Gbp3
17299842	5,397194	5,040424	5,06546	5,363411	5,00561	4,929095	5,1676927	5,099372	-0,06832067	0,953747538	---
17425888	5,5181	5,04426	4,816236	5,179429	4,795382	5,198835	5,1261987	5,057882	-0,06831667	0,953750183	Mup7
17294368	9,223428	9,344364	9,241312	9,25416	9,184036	9,166019	9,2697013	9,201405	-0,06829633	0,953763625	Pdcd6
17355135	4,768445	4,433134	4,598765	4,584091	4,521808	4,48963	4,6001147	4,531843	-0,06827167	0,953779932	Mc4r
17548190	5,787776	5,392422	5,228431	5,500064	5,368906	5,334877	5,469543	5,401282333	-0,06826067	0,953787204	LOC100862073
17204793	3,566651	4,124568	3,627631	3,338157	4,259253	3,516665	3,77295	3,704691667	-0,06825833	0,953788747	---
17490912	4,591663	4,369731	4,580013	4,55093	4,405145	4,380587	4,5138023	4,445554	-0,06824833	0,953795358	Fut2
17211548	5,206779	5,183346	5,194523	5,243344	5,074026	5,062536	5,1948827	5,126635333	-0,06824733	0,953796019	Khdrbs2
17259760	4,961894	4,85098	4,823071	4,953126	4,531574	4,946509	4,8786483	4,810403	-0,06824533	0,953797342	Gm12592
17264151	3,475111	2,831359	3,465767	3,220774	3,10678	3,239954	3,2574123	3,189169333	-0,068243	0,953798884	---
17216563	9,210361	9,488675	9,326824	9,284137	9,240862	9,296139	9,3419533	9,273712667	-0,06824067	0,953800427	Mki67ip
17301823	8,080901	8,01591	7,844932	7,616168	8,038109	8,082747	7,980581	7,912341333	-0,06823967	0,953801088	Gfra2
17459153	4,978644	4,684899	4,770874	4,779455	4,809111	4,641141	4,8114723	4,743235667	-0,06823667	0,953803071	Atoh1
17315387	10,7973	10,79385	10,62068	10,53166	10,82525	10,65028	10,737277	10,66906333	-0,06821333	0,953818498	---
17359676	3,533649	3,292188	3,310704	3,465563	3,271542	3,194855	3,378847	3,310653333	-0,06819367	0,9538315	---
17460665	7,780354	7,758165	7,834202	7,647857	7,750611	7,769769	7,790907	7,722745667	-0,06816133	0,953852877	Mgll
17214983	9,526728	9,605764	9,572989	9,430555	9,55983	9,510623	9,5684937	9,500336	-0,06815767	0,953855302	Cab39
17422832	6,2389	6,377678	6,785046	6,470386	6,368902	6,357868	6,467208	6,399052	-0,068156	0,953856404	Fam132a
17333658	7,663088	7,673153	7,526054	7,56784	7,307166	7,782825	7,620765	7,552610333	-0,06815467	0,953857285	Gm6712 // Gm6712
17541225	7,041826	7,252908	7,247967	7,247384	7,032488	7,058396	7,1809003	7,112756	-0,06814433	0,953864117	---
17337375	6,918817	6,417384	6,730453	6,727154	6,600081	6,535005	6,6888847	6,620746667	-0,068138	0,953868305	Gm6034
17430861	6,270393	6,282625	6,196046	6,165684	6,232026	6,146943	6,249688	6,181551	-0,068137	0,953868966	Sesn2
17546402	2,795015	2,843028	2,827945	2,848	2,651187	2,762401	2,821996	2,753862667	-0,06813333	0,95387139	LOC100040054
17349100	7,998623	8,191201	8,076257	7,901672	8,10125	8,058849	8,0886937	8,020590333	-0,06810333	0,953891225	Pik3c3
17470457	6,927118	7,021044	6,937606	6,906542	6,998285	6,776639	6,9619227	6,893822	-0,06810067	0,953892989	Bid
17391460	7,165126	6,508142	7,057325	7,057325	6,489531	6,979436	6,9101977	6,842097333	-0,06810033	0,953893209	---
17472343	4,060458	4,374473	4,681642	4,300543	4,537863	4,073923	4,372191	4,304109667	-0,06808133	0,953905772	---
17211790	9,15099	9,106275	9,122691	8,981591	9,011355	9,182774	9,126652	9,058573333	-0,06807867	0,953907535	Hs6st1
17512891	6,052557	5,82473	6,335354	5,974855	6,072002	5,96158	6,0708803	6,002812333	-0,068068	0,953914588	LOC100861869
17467072	4,961555	4,669169	4,859591	4,822482	4,687302	4,776354	4,830105	4,762046	-0,068059	0,953920539	Pde1c
17373670	5,867329	5,487578	5,56044	5,743507	5,422064	5,54561	5,638449	5,570393667	-0,06805533	0,953922963	---
17241796	3,887065	4,295222	4,546232	4,041496	4,488059	3,994832	4,2428397	4,174795667	-0,068044	0,953930457	Gm6362
17316574	6,073842	6,007869	6,136425	6,106225	5,922629	5,98516	6,072712	6,004671333	-0,06804067	0,953932661	Ncald
17547694	10,14161	10,24879	10,02632	10,01505	10,13508	10,06248	10,138907	10,07087	-0,06803667	0,953935306	2900097C17Rik // 2900097C17Rik
17411489	6,627975	6,598176	6,306288	6,497485	6,448489	6,38236	6,510813	6,442778	-0,068035	0,953936408	Vmn1r2
17381283	8,417415	8,329638	8,334388	8,420891	8,196503	8,259987	8,3604803	8,292460333	-0,06802	0,953946326	Prpf18
17321007	6,05585	5,607031	6,000515	5,897488	5,783756	5,778115	5,8877987	5,819786333	-0,06801233	0,953951395	Rapgef3
17272004	8,869744	8,86894	8,89992	8,727945	8,857133	8,849499	8,8795347	8,811525667	-0,068009	0,953953599	Grb2
17401142	5,637653	5,427107	5,724141	5,496131	5,599154	5,489591	5,5963003	5,528292	-0,06800833	0,95395404	---
17393051	6,017224	5,619549	5,861787	5,746351	5,767242	5,780966	5,8328533	5,764853	-0,06800033	0,95395933	8430427H17Rik
17300929	10,48224	11,04838	10,83928	10,76558	10,55071	10,84961	10,789967	10,72196667	-0,068	0,953959551	---
17315505	4,574789	4,807918	4,888678	4,295086	4,922666	4,849645	4,7571283	4,689132333	-0,067996	0,953962195	Mir196a-2
17290455	2,869089	2,909767	3,089956	2,911104	2,831053	2,922679	2,9562707	2,888278667	-0,067992	0,95396484	---
17255543	5,420702	5,40174	5,42575	5,17102	5,397886	5,475539	5,416064	5,348148333	-0,06791567	0,954015316	Gm53
17447440	4,801297	4,773486	4,76239	4,850847	4,667624	4,614969	4,7790577	4,711146667	-0,067911	0,954018402	2210406O10Rik
17426956	8,238108	8,170854	8,214384	8,11982	8,095832	8,203983	8,207782	8,139878333	-0,06790367	0,954023252	Haus6
17300802	7,454096	7,377663	7,120299	7,185141	7,278372	7,284859	7,3173527	7,249457333	-0,06789533	0,954028762	Parp4
17487796	8,873658	8,878592	8,814258	8,706988	8,815194	8,840647	8,8555027	8,787609667	-0,067893	0,954030305	Rabac1
17325234	6,612206	6,166319	6,218277	6,279812	6,234639	6,278725	6,3322673	6,264392	-0,06787533	0,954041988	Sema5b
17204217	2,816842	4,235261	3,823958	3,512218	3,8037	3,356535	3,6253537	3,557484333	-0,06786933	0,954045956	---
17386697	3,583833	3,546047	3,672362	3,583404	3,378446	3,636785	3,6007473	3,532878333	-0,067869	0,954046176	---
17398634	8,610062	8,660535	8,611816	8,496434	8,594088	8,588343	8,627471	8,559621667	-0,06784933	0,954059182	Sh3d19
17365145	5,217279	5,635444	5,546038	5,417002	5,388321	5,38989	5,4662537	5,398404333	-0,06784933	0,954059182	---
17296912	3,788273	3,56612	2,85231	3,12508	3,520103	3,358024	3,4022343	3,334402333	-0,067832	0,954070644	---
17221660	2,858603	2,903003	2,845471	2,897202	2,69938	2,807	2,8690257	2,801194	-0,06783167	0,954070865	---
17518780	5,597073	5,545523	5,630011	5,468338	5,569263	5,531512	5,590869	5,523037667	-0,06783133	0,954071085	Car12
17342359	6,359288	6,336315	6,350896	6,264216	6,352279	6,226518	6,348833	6,281004333	-0,06782867	0,954072849	Haghl
17422785	6,238421	6,302896	6,284646	6,304132	5,97289	6,345456	6,275321	6,207492667	-0,06782833	0,954073069	Acap3
17207549	5,624026	4,275125	5,610261	4,63119	5,199849	5,4749	5,169804	5,101979667	-0,06782433	0,954075714	---
17271203	4,493176	4,72545	4,614151	4,536094	4,488059	4,605156	4,6109257	4,543103	-0,06782267	0,954076817	E030025P04Rik
17247849	6,067001	5,677581	5,837444	5,608731	5,902615	5,867215	5,8606753	5,792853667	-0,06782167	0,954077478	---
17329163	4,800671	4,919569	5,479149	5,091209	4,891719	5,013027	5,066463	4,998651667	-0,06781133	0,954084312	Camk2n2
17366902	4,109785	4,359433	4,565104	4,26122	4,319641	4,250095	4,344774	4,276985333	-0,06778867	0,954099302	Mir669c // Mir669c
17504327	5,882859	6,054764	6,233461	5,872054	6,051694	6,043971	6,057028	5,989239667	-0,06778833	0,954099522	Ndrg4
17505034	9,04045	9,087677	9,013813	8,986475	9,003256	8,94885	9,0473133	8,979527	-0,06778633	0,954100845	Nfatc3
17290259	5,581737	5,517973	5,407124	5,46685	5,453907	5,382753	5,502278	5,434503333	-0,06777467	0,95410856	Ucn3
17495135	11,71306	11,72307	11,62661	11,57677	11,65354	11,62914	11,68758	11,61981667	-0,06776333	0,954116056	Eif4g2
17200679	2,642338	3,185313	3,445572	2,907428	3,128537	3,034034	3,0910743	3,023333	-0,06774133	0,954130605	---
17457116	4,125901	3,901036	3,916503	4,029314	3,921925	3,789013	3,9811467	3,913417333	-0,06772933	0,954138541	---
17535390	7,215916	7,294024	7,185231	7,189637	7,209304	7,093055	7,2317237	7,163998667	-0,067725	0,954141407	Prrg3
17489905	6,821352	6,996702	6,919836	7,007868	6,945233	6,581626	6,91263	6,844909	-0,067721	0,954144053	Plekhf1
17275047	6,014576	5,83296	5,966955	5,877401	5,87715	5,856782	5,9381637	5,870444333	-0,06771933	0,954145155	Ispd
17248654	5,488785	5,49106	5,446995	5,541267	5,38833	5,294136	5,4756133	5,407911	-0,06770233	0,954156398	Il12b
17370745	8,784149	8,974072	8,749482	8,694351	8,849728	8,760628	8,835901	8,768235667	-0,06766533	0,954180869	Rif1
17548624	4,783309	4,771124	5,230582	4,904248	4,738103	4,93972	4,9283383	4,860690333	-0,067648	0,954192333	LOC100861900
17546040	9,296341	9,217446	9,185472	9,094714	9,161863	9,239773	9,2330863	9,16545	-0,06763633	0,95420005	Rab9
17427104	3,7805	4,111892	3,99675	4,169641	3,534722	3,981918	3,9630473	3,895427	-0,06762033	0,954210632	Ifnab
17349554	10,3307	10,39938	10,38337	10,27765	10,31968	10,31329	10,37115	10,30354	-0,06761	0,954217467	Matr3
17405843	3,145757	3,252662	3,329358	3,143558	3,31784	3,063571	3,2425923	3,174989667	-0,06760267	0,954222317	---
17407412	5,443954	5,268869	6,643718	6,075639	5,475957	5,602183	5,7855137	5,717926333	-0,06758733	0,954232459	Sprr1a
17483740	7,45675	7,239532	7,335276	7,371186	7,365858	7,091753	7,3438527	7,276265667	-0,067587	0,954232679	Inpp5f
17206361	3,686794	3,328966	3,606632	3,614747	3,66184	3,143098	3,5407973	3,473228333	-0,067569	0,954244585	---
17411848	4,536817	4,442068	4,695699	4,325871	4,624329	4,521748	4,5581947	4,490649333	-0,06754533	0,954260239	Cdh17
17425018	6,872157	6,89508	7,018261	6,685269	6,984787	6,91282	6,9284993	6,860958667	-0,06754067	0,954263326	5830415F09Rik
17478175	7,007344	6,685728	6,928794	6,829918	6,996261	6,593071	6,8739553	6,806416667	-0,06753867	0,954264649	Myod1
17358009	9,747559	9,518683	9,496607	9,261878	9,678721	9,619667	9,5876163	9,520088667	-0,06752767	0,954271925	---
17414250	7,759094	7,921738	7,649772	7,603383	7,766701	7,757942	7,776868	7,709342	-0,067526	0,954273027	Tmem38b
17503932	5,143064	5,220249	5,298281	5,187625	5,092344	5,179066	5,2205313	5,153011667	-0,06751967	0,954277216	Mt3
17376026	8,296643	8,442809	8,297336	8,294582	8,240681	8,299044	8,345596	8,278102333	-0,06749367	0,954294414	Tmem87b
17386421	5,881642	6,2249	6,165087	6,05877	6,046733	5,963646	6,090543	6,023049667	-0,06749333	0,954294635	Hat1 // Hat1
17299995	4,463727	4,55413	4,47649	4,526806	4,547801	4,217302	4,4981157	4,430636333	-0,06747933	0,954303895	---
17206147	4,809493	3,550944	3,819642	4,232243	3,896827	3,848609	4,0600263	3,992559667	-0,06746667	0,954312274	---
17391054	3,624161	3,698986	3,608341	3,581797	3,70696	3,440352	3,6438293	3,576369667	-0,06745967	0,954316904	---
17503442	2,711085	2,855158	2,944967	2,846699	2,822895	2,639274	2,83707	2,769622667	-0,06744733	0,954325063	---
17500168	5,079211	5,033401	5,082219	5,130712	4,831392	5,030393	5,0649437	4,997499	-0,06744467	0,954326827	---
17207381	7,329618	7,441741	7,011065	7,472396	6,98165	7,126069	7,260808	7,193371667	-0,06743633	0,954332339	---
17226736	8,699838	8,878913	8,60844	8,62384	8,714125	8,646947	8,7290637	8,661637333	-0,06742633	0,954338954	Mapkapk2
17340382	4,354521	4,217134	4,289918	4,081365	4,405272	4,172659	4,287191	4,219765333	-0,06742567	0,954339395	Gm4719
17444609	6,085596	5,811121	5,897055	6,024795	5,730978	5,835743	5,9312573	5,863838667	-0,06741867	0,954344026	Pdap1
17519357	8,833889	8,936163	8,866143	8,981965	8,616538	8,835469	8,8787317	8,811324	-0,06740767	0,954351302	Onecut1
17455602	3,717719	3,86937	3,874004	3,85809	3,645321	3,755475	3,8203643	3,752962	-0,06740233	0,95435483	Vmn2r18
17353488	8,458791	8,77018	8,416183	8,408192	8,47426	8,560499	8,5483847	8,480983667	-0,067401	0,954355712	Cdc23
17503572	8,094582	8,263983	8,030039	7,998588	8,166489	8,021341	8,1295347	8,062139333	-0,06739533	0,954359461	Heatr3
17372679	9,119537	9,191387	9,269243	9,045303	9,195179	9,137547	9,193389	9,126009667	-0,06737933	0,954370045	Ssrp1
17515703	4,986329	4,87187	5,211744	4,924055	5,072782	4,87102	5,0233143	4,955952333	-0,067362	0,954381511	Adamts8
17316392	12,0121	11,87646	11,65517	11,73173	11,70916	11,90076	11,84791	11,78055	-0,06736	0,954382834	Hrsp12
17301556	3,360035	2,98955	3,160706	3,153102	3,094266	3,060853	3,170097	3,102740333	-0,06735667	0,95438504	---
17468113	7,17019	7,143502	7,156102	6,944368	7,14504	7,178321	7,156598	7,089243	-0,067355	0,954386142	Ino80b
17536218	2,907044	2,879339	3,09844	2,87865	2,954707	2,849414	2,9616077	2,894257	-0,06735067	0,954389009	Mageb2
17392959	5,515845	5,565237	5,702267	5,38564	5,728083	5,467603	5,5944497	5,527108667	-0,067341	0,954395404	Defb29
17276732	10,23235	10,44199	10,16303	10,10596	10,21743	10,31196	10,279123	10,21178333	-0,06734	0,954396065	Eif2s1
17294167	7,722154	8,114734	7,842323	7,693701	7,902177	7,881322	7,8930703	7,825733333	-0,067337	0,95439805	BC018507
17317381	4,924417	4,728295	4,997569	4,827296	4,678155	4,942825	4,883427	4,816092	-0,067335	0,954399373	Gm19510

17322939	5,974602	6,170515	5,96116	5,916479	6,048852	5,938946	6,0354257	5,968092333	-0,06733333	0,954400475	2610020C07Rik
17470095	5,234094	4,915093	5,396434	5,226689	5,254399	4,862537	5,1818737	5,114541667	-0,067332	0,954401357	Fxyd4
17285631	4,813272	4,856062	4,622535	4,787444	4,590392	4,712043	4,7639563	4,696626333	-0,06733	0,95440268	Gpx6
17497010	4,783499	4,603843	4,685006	4,589496	4,587278	4,693606	4,6907827	4,62346	-0,06732267	0,954407532	4933402N03Rik
17525947	6,04521	5,859921	5,902277	5,786962	5,987593	5,830901	5,9358027	5,868485333	-0,06731733	0,95441106	4930546K05Rik
17539748	6,807321	6,351634	6,713749	6,630759	6,328681	6,711326	6,6242347	6,556922	-0,06731267	0,954414147	Gm14354
17285731	4,556864	4,553286	4,673446	4,56214	4,442845	4,576681	4,594532	4,527222	-0,06731	0,954415911	---
17335878	7,071008	7,302178	7,130061	7,104374	7,153678	7,0433	7,167749	7,100450667	-0,06729833	0,95442363	Pknox1
17417660	6,554404	6,541908	6,682435	6,397125	6,601469	6,578264	6,5929157	6,525619333	-0,06729633	0,954424953	Tmem53
17326731	4,600151	4,311434	4,34688	4,249538	4,452452	4,354623	4,4194883	4,352204333	-0,067284	0,954433112	4930570E03Rik
17524752	8,645875	8,669166	8,834509	8,566401	8,69844	8,682869	8,7165167	8,649236667	-0,06728	0,954435758	Spc24
17524357	3,457271	2,939525	3,304243	3,288684	2,986304	3,224221	3,2336797	3,166403	-0,06727667	0,954437963	Olfr870
17275925	9,63893	9,765084	9,67977	9,636737	9,627975	9,617252	9,6945947	9,627321333	-0,06727333	0,954440169	Arf6
17258023	8,435448	8,572086	8,486799	8,438148	8,435279	8,419088	8,498111	8,430838333	-0,06727267	0,95444061	Ttyh2
17495187	2,961225	3,142722	3,145909	3,130876	2,873478	3,043704	3,0832853	3,016019333	-0,067266	0,95444502	---
17548108	7,909175	7,82655	7,757566	7,685266	7,907759	7,698503	7,831097	7,763842667	-0,06725433	0,954452738	Nutf2 // Nutf2
17548696	7,909175	7,82655	7,757566	7,685266	7,907759	7,698503	7,831097	7,763842667	-0,06725433	0,954452738	Nutf2 // Nutf2
17427729	10,65834	10,62733	10,53479	10,42864	10,6176	10,57246	10,60682	10,53956667	-0,06725333	0,9544534	C8a
17314011	7,950623	7,662743	7,670414	7,702577	7,653338	7,726139	7,76126	7,694018	-0,067242	0,954460898	Pim3
17267446	5,957206	6,134285	6,100578	6,126373	5,977987	5,885997	6,064023	5,996785667	-0,06723733	0,954463985	1200011M11Rik
17411065	4,764608	4,592782	4,718769	4,66646	4,615998	4,592015	4,692053	4,624824333	-0,06722867	0,954469719	4930503B20Rik
17379003	3,397786	3,694059	3,771925	3,696604	3,531966	3,43353	3,6212567	3,554033333	-0,06722333	0,954473248	9130015L21Rik
17279026	6,473612	6,785634	6,562535	6,47838	6,539206	6,602543	6,6072603	6,540043	-0,06721733	0,954477217	Tecpr2
17289728	7,486939	7,600244	7,659056	7,392504	7,510295	7,641828	7,5820797	7,514875667	-0,067204	0,954486038	Depdc1b
17491777	3,652625	3,6898	3,81506	3,66772	3,570212	3,717958	3,7191617	3,651963333	-0,06719833	0,954489787	4930554H23Rik
17533780	6,69658	6,737436	6,200537	6,502846	6,554471	6,375643	6,544851	6,477653333	-0,06719767	0,954490228	Wdr44
17340485	8,49876	8,600539	8,473394	8,496196	8,424461	8,450529	8,524231	8,457062	-0,067169	0,954509195	Tmem242
17226420	11,43242	11,39285	11,32447	11,27544	11,33334	11,33946	11,383247	11,31608	-0,06716667	0,954510738	Actr3
17219339	6,816936	7,036093	6,806116	6,807511	6,855717	6,794432	6,8863817	6,81922	-0,06716167	0,954514046	Usf1
17406892	5,38086	5,16793	5,680278	5,415397	5,278958	5,333235	5,4096893	5,34253	-0,06715933	0,95451559	Rusc1
17324396	9,998981	10,15232	10,18724	10,06958	10,08237	9,985114	10,112847	10,045688	-0,067159	0,954515811	Eif4a2
17335616	10,08283	10,04361	10,0475	9,877045	10,04813	10,04729	10,05798	9,990821667	-0,06715833	0,954516252	Zfand3
17355159	5,841607	5,705935	5,614634	5,542528	5,748085	5,670106	5,7207253	5,653573	-0,06715233	0,954520222	B430212C06Rik
17347650	9,572701	9,516193	9,519327	9,530331	9,282489	9,593964	9,5360737	9,468928	-0,06714567	0,954524632	Haao
17420866	4,713696	4,630108	4,680429	4,495248	4,615467	4,712145	4,6747443	4,60762	-0,06712433	0,954538747	Padi1 // Padi1
17201043	8,32851	8,46191	8,621614	8,07187	8,428666	8,710151	8,470678	8,403562333	-0,06711567	0,954544481	---
17367644	4,206282	3,820054	3,66569	3,920327	3,775265	3,795113	3,897342	3,830235	-0,067107	0,954550216	Il1f8
17396162	12,87291	12,87682	12,91054	12,71021	12,85219	12,89657	12,886757	12,81965667	-0,0671	0,954554847	Car2
17438324	9,742532	9,670483	9,621883	9,44907	9,704535	9,680075	9,6782993	9,611226667	-0,06707267	0,954572932	---
17471968	3,307872	3,244996	3,220204	3,14558	3,288862	3,137442	3,2576907	3,190628	-0,06706267	0,954579549	Tas2r103
17525727	3,418048	3,298514	3,380156	3,319603	3,320524	3,255409	3,3655727	3,298512	-0,06706067	0,954580872	Olfr952
17480568	9,400043	9,458258	9,534067	9,425756	9,357621	9,407855	9,4641227	9,397077333	-0,06704533	0,954591018	Arrb1
17327757	7,77472	7,86692	7,837821	7,672578	7,808985	7,796846	7,826487	7,759469667	-0,06701733	0,954609545	Pam16
17464786	4,870314	4,538341	4,680304	4,989985	4,380093	4,517873	4,6963197	4,629317	-0,06700267	0,95461925	---
17314666	5,438254	5,587588	5,756046	5,593135	5,474888	5,512883	5,5939627	5,526968667	-0,066994	0,954624984	Wnt1
17211243	9,485242	9,703671	9,527376	9,455704	9,548724	9,510897	9,5720963	9,505108333	-0,066988	0,954628954	Terf1
17543916	5,410827	5,509867	5,424429	5,41944	5,281736	5,443081	5,4483743	5,381419	-0,06695533	0,95465057	E530001F21Rik
17437488	3,085682	3,182964	3,050698	3,062479	2,993231	3,062822	3,106448	3,039510667	-0,06693733	0,954662481	---
17202887	4,115928	3,905092	3,81662	3,909475	3,967858	3,759499	3,94588	3,878944	-0,066936	0,954663363	---
17270567	9,272883	9,120204	9,149742	9,19902	9,061729	9,081281	9,180943	9,11401	-0,066933	0,954665349	Gosr2
17200065	3,624248	3,252404	3,522824	3,30666	3,755509	3,136509	3,466492	3,399559333	-0,06693267	0,954665569	---
17497811	7,207717	6,687469	7,245614	7,250498	6,970191	6,71932	7,0469333	6,980003	-0,06693033	0,954667113	Mir210
17547990	7,027775	7,314385	7,217492	7,079738	7,157232	7,121899	7,1865507	7,119623	-0,06692767	0,954668878	Mrps18c
17344933	4,964137	4,929368	5,122765	4,985866	4,773518	5,056139	5,0054233	4,938507667	-0,06691567	0,954676819	Gm20581
17546614	5,382493	5,985389	6,272864	5,715476	6,059579	5,664977	5,8802487	5,813344	-0,06690467	0,954684098	LOC100504515
17539586	5,756787	6,085704	5,776695	5,677525	5,675104	6,065881	5,873062	5,80617	-0,066892	0,95469248	Fancb
17317398	4,437333	4,303148	4,447667	4,256968	4,483884	4,246706	4,3960493	4,329186	-0,06686333	0,95471145	A1bg
17348451	9,343805	9,105508	9,282724	8,982972	9,256893	9,291595	9,2440123	9,177153333	-0,066859	0,954714317	---
17318670	6,382098	6,324755	6,517004	6,174817	6,414196	6,434293	6,4079523	6,341102	-0,06685033	0,954720053	Recql4
17341029	5,276218	5,217376	5,561388	5,419899	5,434036	5,000573	5,3516607	5,284836	-0,06682467	0,954737038	2700054A10Rik
17305034	5,301517	5,243211	4,979278	5,276782	4,910458	5,136298	5,1746687	5,107846	-0,06682267	0,954738362	Sncg
17353619	7,379703	7,110065	7,159566	6,984617	7,138488	7,325849	7,2164447	7,149651333	-0,06679333	0,954757774	2010001M09Rik
17202861	3,781079	4,747941	3,709624	4,500054	4,04095	3,497279	4,079548	4,012761	-0,066787	0,954761965	---
17441425	5,493896	4,985994	5,284982	5,507706	5,131178	4,925651	5,2549573	5,188178333	-0,066779	0,954767259	---
17508779	5,191554	5,005747	5,31038	4,962245	5,161107	5,184028	5,169227	5,10246	-0,066767	0,954775201	Cldn23
17246055	3,242587	3,332301	3,35101	3,197727	3,364913	3,162983	3,3086327	3,241874333	-0,06675833	0,954780937	---
17529645	3,713346	3,588177	3,827776	3,444169	3,849677	3,635194	3,7097663	3,643013333	-0,066753	0,954784466	Mir184
17254532	6,651397	6,39677	6,558346	6,339756	6,663275	6,403294	6,5355043	6,468775	-0,06672933	0,954800129	D630032N06Rik
17286882	8,106953	8,145822	8,060095	7,955014	8,104966	8,052718	8,10429	8,037566	-0,066724	0,954803659	Nol7
17530105	3,384561	3,650739	3,562427	3,48301	3,37885	3,535728	3,5325757	3,465862667	-0,066713	0,954810939	---
17534222	3,223535	3,189692	3,585752	3,220575	3,309126	3,269158	3,332993	3,266286333	-0,06670667	0,95481513	Rhox3c
17481725	8,664501	8,863033	8,878692	8,693268	8,79086	8,721999	8,8020753	8,735375667	-0,06669967	0,954819763	Zfp143
17388794	4,407063	4,21515	4,193041	4,228871	4,265461	4,120827	4,2717513	4,205053	-0,06669833	0,954820646	A930006I01Rik
17208179	5,840258	4,941801	5,732874	5,641931	5,188287	5,484643	5,5049777	5,438287	-0,06669067	0,95482572	---
17341702	10,74692	10,54326	10,5204	10,42393	10,58976	10,59682	10,603527	10,53683667	-0,06669	0,954826161	---
17265268	7,404078	7,474618	7,422691	7,641855	7,160424	7,299053	7,4337957	7,367110667	-0,066685	0,95482947	Cxcl16
17508861	5,313347	5,101904	5,336793	5,316163	4,93957	5,296347	5,2506813	5,184026667	-0,06665467	0,954849546	Fgf20
17486025	3,947477	3,95429	3,848616	3,809612	3,730604	4,010218	3,9167943	3,850144667	-0,06664967	0,954852855	Nlrp2
17242516	8,25134	7,957252	7,816339	8,174723	7,800378	7,849883	8,0083103	7,941661333	-0,066649	0,954853297	Pdxk
17229348	4,270723	3,920444	4,06988	4,014833	4,129571	3,916739	4,0870157	4,020381	-0,06663467	0,954862783	Gm4846
17381657	9,383776	9,376644	9,305739	9,111292	9,354412	9,400577	9,3553863	9,288760333	-0,066626	0,954868519	Rbm17
17280932	3,187707	3,264676	3,121016	3,109744	3,118268	3,145559	3,191133	3,124523667	-0,06660933	0,95487955	---
17443613	9,736468	9,808915	9,582497	9,515481	9,695278	9,717328	9,7092933	9,642695667	-0,06659767	0,954887272	Tfr2
17210055	3,51463	3,499199	3,349753	3,202816	4,063318	2,897665	3,4545273	3,387933	-0,06659433	0,954889479	---
17209861	6,346261	6,503231	6,369547	6,487885	5,986068	6,545374	6,4063463	6,339775667	-0,06657067	0,954905143	---
17469463	9,77294	9,529037	9,58375	9,595367	9,539352	9,551315	9,6285757	9,562011333	-0,06656433	0,954909335	Rybp
17315447	11,96682	11,80959	11,90184	11,8277	11,78865	11,86231	11,89275	11,82622	-0,06653	0,95493206	Pcbp2
17407594	7,792832	8,024867	7,916009	7,789132	7,771519	7,973472	7,911236	7,844707667	-0,06652833	0,954933164	Cgn
17530115	4,962741	4,849471	4,844337	4,787017	4,836895	4,833062	4,8855163	4,818991333	-0,066525	0,95493537	Il20rb
17327862	5,596196	5,409484	5,559299	5,264697	5,792963	5,307746	5,5216597	5,455135333	-0,06652433	0,954935811	Sept12
17412092	10,45865	10,22544	10,00138	10,05138	10,17849	10,25604	10,22849	10,16197	-0,06652	0,95493868	Ttpa
17375373	12,99749	12,98961	12,79099	12,877	12,81972	12,88182	12,92603	12,85951333	-0,06651667	0,954940886	B2m
17394063	5,981633	6,127732	6,070238	6,077537	5,928265	5,974267	6,0598677	5,993356333	-0,06651133	0,954944416	Ada
17394126	4,111572	4,032043	3,889484	3,902915	4,087408	3,843257	4,011033	3,944526667	-0,06650633	0,954947726	Wfdc15b
17365343	6,816196	6,58092	6,707152	6,834505	6,426497	6,643801	6,7014227	6,634934333	-0,06648833	0,95495964	4833438C02Rik
17282970	5,825815	5,134756	5,493054	5,529693	5,334096	5,390418	5,4845417	5,418069	-0,06647267	0,954970011	Dio2
17542349	7,554519	7,52083	7,521904	7,338582	7,555388	7,503928	7,5324177	7,465966	-0,06645167	0,954983911	Haus7
17228375	8,748802	8,748238	8,805723	8,726171	8,619211	8,758106	8,7675877	8,701162667	-0,066425	0,955001563	Xpr1
17486982	8,252034	8,324239	8,264545	8,159535	8,385481	8,096557	8,2802727	8,213857667	-0,066415	0,955008183	Ppp5c
17515395	6,004008	6,266404	6,412873	6,017504	6,301663	6,164896	6,2277617	6,161354333	-0,06640733	0,955013258	Gm16845
17242700	5,367958	5,066739	5,258452	5,433167	5,098892	4,961912	5,2310497	5,164657	-0,06639267	0,955022967	---
17452871	3,308578	3,313545	3,02922	3,05284	3,228778	3,170564	3,2171143	3,150727333	-0,066387	0,955026718	---
17202599	5,222941	4,877682	5,072078	5,224174	4,921087	4,828284	5,057567	4,991181667	-0,06638533	0,955027821	---
17406460	6,218964	6,286757	6,1376	6,19378	5,969748	6,280659	6,2144403	6,148062333	-0,066378	0,955032676	Dclk2
17219481	8,223302	9,017333	8,580027	8,45336	8,734219	8,434024	8,6068873	8,540534333	-0,066353	0,955049225	---
17319823	7,196042	7,189734	7,125865	7,000357	7,204735	7,1075	7,170547	7,104197333	-0,06634967	0,955051432	1700001L05Rik
17472073	5,051723	4,840227	4,987733	4,896959	4,881905	4,901796	4,9598943	4,893553333	-0,066341	0,955057169	Gsg1
17258887	6,789877	7,053341	6,864344	6,808464	6,863999	6,836098	6,9025207	6,836187	-0,06633367	0,955062024	Pgs1
17338536	4,852098	5,044536	4,72205	4,833511	4,805095	4,781149	4,8728947	4,806585	-0,06630967	0,955077912	Gm20098
17412871	7,696747	7,828036	7,582073	7,597701	7,632258	7,678013	7,7022853	7,635990667	-0,06629467	0,955087842	Nudt2
17284049	6,578295	6,489429	6,684114	6,486209	6,557471	6,509276	6,583946	6,517652	-0,066294	0,955088284	Ankrd9
17502554	6,377381	6,456124	6,594241	6,401995	6,366099	6,460918	6,4759153	6,409670667	-0,06624467	0,955120944	Tom1
17460393	8,058226	8,369149	8,056787	8,062022	8,027727	8,195685	8,1613873	8,095144667	-0,06624267	0,955122268	Fam136a
17455682	5,749166	5,25897	5,340168	5,430166	5,313332	5,406108	5,4494347	5,383202	-0,06623267	0,955128888	C87414 // C87414
17409386	8,897717	8,838089	8,94079	8,793276	8,812944	8,871679	8,8921987	8,825966333	-0,06623233	0,955129109	Gpsm2
17203419	7,109458	6,717782	7,328193	7,089265	6,84501	7,022501	7,051811	6,985592	-0,066219	0,955137936	---
17438155	4,905056	5,142456	4,994266	4,803385	5,094085	4,945677	5,013926	4,947715667	-0,06621033	0,955143674	Spata18
17481326	4,419802	3,761323	4,036649	3,976917	3,944485	4,097857	4,0725913	4,006419667	-0,06617167	0,955169274	Olfr657
17371148	3,969714	3,879238	3,942823	4,048132	3,575601	3,969531	3,9305917	3,864421333	-0,06617033	0,955170157	Tbr1
17246967	10,54761	10,70416	10,6452	10,49684	10,6065	10,59514	10,632323	10,56616	-0,06616333	0,955174791	Xbp1
17286340	4,802604	4,451968	4,781224	4,428501	4,642158	4,766704	4,6785987	4,612454333	-0,06614433	0,955187371	Foxc1
17447147	7,741961	7,821985	7,559243	7,556333	7,723279	7,645161	7,7077297	7,641591	-0,06613867	0,955191122	---
17354599	6,309098	5,836825	6,309676	6,115452	6,089517	6,052218	6,1518663	6,085729	-0,06613733	0,955192005	Myoz3
17353167	3,267899	3,448755	3,473546	3,205947	3,421059	3,364802	3,3967333	3,330602667	-0,06613067	0,955196419	---
17281490	6,925155	6,939505	6,910644	6,816082	6,952157	6,808679	6,9251013	6,858972667	-0,06612867	0,955197743	Rpl36al // Rpl36a
17499579	4,572511	4,529085	4,488325	4,089321	4,769393	4,532839	4,5299737	4,463851	-0,06612267	0,955201716	Spag11b
17291780	5,689095	5,576762	5,683206	5,561837	5,560064	5,628802	5,6496877	5,583567667	-0,06612	0,955203481	Tubb2b
17262736	4,78948	4,271027	4,599267	4,427517	4,561763	4,472188	4,553258	4,487156	-0,066102	0,955215399	---
17401790	4,797059	4,683981	4,942716	4,801657	4,63115	4,79266	4,8079187	4,741822333	-0,06609633	0,955219151	Aknad1
17384619	5,517546	5,607129	5,121725	5,252398	5,478908	5,316846	5,4154667	5,349384	-0,06608267	0,9552282	Snord90
17486240	5,430391	4,749411	4,898741	5,086047	4,944471	4,849798	5,026181	4,960105333	-0,06607567	0,955232835	Vmn2r35
17548018	7,788002	7,874383	7,595324	7,5422	7,879488	7,637805	7,7525697	7,686497667	-0,066072	0,955235263	Lnpep
17475777	8,777601	8,928561	8,885763	8,77179	8,831368	8,790587	8,863975	8,797915	-0,06606	0,955243208	Fbl
17363237	7,217933	7,315258	7,184105	7,122718	7,178093	7,218353	7,2390987	7,173054667	-0,066044	0,955253802	Pebp1

17448614	6,488768	6,329042	6,62016	6,511955	6,356226	6,371667	6,4793233	6,413282667	-0,06604067	0,955256009	Corin
17421233	2,925418	3,086918	3,269748	3,164224	3,002264	2,917481	3,094028	3,027989667	-0,06603833	0,955257554	Gm13023
17550514	4,988485	4,583713	4,567438	4,959272	4,658064	4,324193	4,713212	4,647176333	-0,06603567	0,95525932	---
17331692	7,038525	6,932959	6,843007	7,087369	6,672402	6,856658	6,9381637	6,872143	-0,06602067	0,955269252	Cyyr1
17229981	3,735108	4,203269	4,439939	3,670686	3,98419	4,525401	4,1261053	4,060092333	-0,066013	0,955274328	Gm11062
17463801	4,344315	4,155715	4,281684	4,327132	4,077466	4,179147	4,2605713	4,194581667	-0,06598967	0,955289779	Grin2b
17289787	10,68506	10,94082	10,94423	10,76803	10,803	10,80113	10,856703	10,79072	-0,06598333	0,955293972	Rps3a
17519568	4,351198	4,361206	4,342792	4,077862	4,417814	4,361631	4,351732	4,285769	-0,065963	0,955307436	---
17264153	4,512235	4,321487	4,74083	4,37712	4,351126	4,64845	4,5248507	4,458898667	-0,065952	0,95531472	---
17334253	9,623726	9,578705	9,596099	9,540134	9,481799	9,578772	9,59951	9,533568333	-0,06594167	0,955321563	Eci1
17505740	5,715306	5,628014	5,439255	5,456247	5,611656	5,516868	5,5941917	5,528257	-0,06593467	0,955326198	---
17484233	7,246377	7,370452	7,112775	7,173115	7,06625	7,292478	7,2432013	7,177281	-0,06592033	0,955335689	Ptpre
17537889	11,15897	11,0769	10,95967	10,9345	11,05416	11,00912	11,06518	10,99926	-0,06592	0,95533591	Wbp5
17421164	4,928439	4,663244	4,77344	4,900691	4,579698	4,686977	4,7883743	4,722455333	-0,065919	0,955336572	---
17449820	6,46382	6,928638	6,452875	6,458311	6,723006	6,466273	6,615111	6,549196667	-0,06591433	0,955339662	Gm16427
17520903	3,774706	4,141812	4,430154	3,802768	4,139544	4,206688	4,1155573	4,049666667	-0,06589067	0,955355334	---
17515402	5,452583	5,774864	6,852679	5,842768	5,846085	6,193632	6,0267087	5,960828333	-0,06588033	0,955362177	Cnn1
17491497	4,787724	4,65983	4,644721	4,647305	4,678694	4,568637	4,697425	4,631545333	-0,06587967	0,955362619	C230091D08Rik
17219148	5,008236	4,894743	4,981537	4,995757	4,917193	4,773938	4,9615053	4,895629333	-0,065876	0,955365047	1700084C01Rik
17210185	4,891103	4,400154	5,165907	4,67197	4,523493	5,064088	4,8190547	4,753183667	-0,065871	0,955368358	---
17262491	6,90606	6,786755	6,839719	6,776503	6,869409	6,689022	6,844178	6,778311333	-0,06586667	0,955371227	Rmnd5b
17317313	8,787974	8,853561	8,630886	8,654119	8,712696	8,70801	8,7574737	8,691608333	-0,06586533	0,95537211	Tatdn1
17443350	7,284752	7,591516	7,515649	7,354286	7,404812	7,435284	7,4639723	7,398127333	-0,065845	0,955385575	Ywhag // Ywhag
17290922	8,115085	8,337976	8,342791	8,368845	7,989475	8,240011	8,265284	8,199443667	-0,06584033	0,955388666	---
17374089	9,668633	9,532165	9,462935	9,490157	9,480392	9,495665	9,5545777	9,488738	-0,06583967	0,955389107	Lin7c
17548518	12,34812	12,29613	12,28059	12,17885	12,25465	12,29383	12,30828	12,24244333	-0,06583667	0,955391094	H3f3a // H3f3a
17203291	3,044534	3,957565	3,32099	3,53535	3,530834	3,059452	3,4410297	3,375212	-0,06581767	0,955403676	---
17337614	5,262766	5,128492	5,225122	5,209749	5,290215	4,918987	5,20546	5,139650333	-0,06580967	0,955408974	Olfr127
17293397	4,389104	4,424331	4,426879	4,180053	4,492756	4,370086	4,413438	4,347631667	-0,06580633	0,955411182	Ctsr
17525395	5,188311	5,136965	5,243209	4,978914	5,136026	5,25615	5,189495	5,123696667	-0,06579833	0,95541648	Gm1113
17337427	3,800463	3,698723	3,586329	3,576596	3,656914	3,654649	3,6951717	3,629386333	-0,06578533	0,955425089	H2-M10.5
17486543	6,307782	5,84948	6,411907	6,073668	6,200727	6,097467	6,189723	6,123954	-0,065769	0,955435906	Vmn2r54
17376974	5,019206	4,975293	5,067528	4,806548	5,041136	5,017062	5,0206757	4,954915333	-0,06576033	0,955441645	---
17201563	5,381852	5,253304	4,828957	4,790074	5,612798	4,864006	5,1547043	5,088959333	-0,065745	0,9554518	---
17433023	4,663926	4,907187	4,908457	4,82889	4,669223	4,784224	4,8265233	4,760779	-0,06574433	0,955452241	Cort
17500824	7,968674	7,755423	7,997664	8,041975	7,641661	7,840905	7,9072537	7,841513667	-0,06574	0,955455111	---
17275232	3,255756	3,208918	3,261177	3,04402	3,366021	3,118617	3,2419503	3,176219333	-0,065731	0,955461072	---
17298608	6,609367	6,394991	6,688656	6,417177	6,648006	6,430732	6,564338	6,498638333	-0,06569967	0,955481823	Prrxl1
17548153	9,086426	9,215095	9,248173	9,041095	9,046644	9,264868	9,1832313	9,117535667	-0,06569567	0,955484472	LOC100862111
17506546	6,39911	6,409413	6,51053	6,36654	6,188042	6,567386	6,4396843	6,373989333	-0,065695	0,955484914	4732415M23Rik
17286454	3,872076	4,00418	4,208372	3,797479	4,066104	4,02396	4,0282093	3,962514333	-0,065695	0,955484914	1110046J04Rik
17527996	5,664196	5,647026	5,791322	5,802405	5,722968	5,380087	5,700848	5,635153333	-0,06569467	0,955485135	Coro2b
17537576	3,73197	3,444837	3,691632	3,627214	3,467799	3,576347	3,622813	3,55712	-0,065693	0,955486239	---
17455627	5,276218	4,752242	5,035496	5,055468	4,992344	4,81912	5,0213187	4,955644	-0,06567467	0,955498381	BC061212 // Gm7978 // BC061212 // G
17359578	4,713924	4,794018	4,846178	4,846178	4,713181	4,597743	4,7847067	4,719034	-0,06567267	0,955499705	---
17476902	5,696947	4,738885	5,245855	5,50681	5,305452	4,672423	5,227229	5,161561667	-0,06566733	0,955503238	---
17516320	6,069837	5,536292	5,708572	5,812527	5,612741	5,692469	5,771567	5,705912333	-0,06565467	0,955511627	Zfp202
17202805	6,134809	6,170459	6,20169	6,649701	5,999813	5,66048	6,168986	6,103331333	-0,06565467	0,955511627	---
17340304	4,502861	4,58592	4,672991	4,753053	4,35601	4,455757	4,5872573	4,521606667	-0,06565067	0,955514276	Gm9405
17446424	5,873705	5,818235	5,786144	5,787889	5,840309	5,652943	5,826028	5,760380333	-0,06564767	0,955516263	Xrcc2
17450047	3,190872	3,239602	3,282779	3,189428	3,292396	3,03449	3,237751	3,172104667	-0,06564633	0,955517146	---
17387942	3,901628	3,38217	3,550594	3,660127	3,484917	3,492487	3,611464	3,545843667	-0,06562033	0,955534366	Olfr1239
17422109	5,714417	5,772291	6,022359	5,805742	5,757867	5,748652	5,8363557	5,770753667	-0,065602	0,955546509	Hes2
17412047	3,917918	3,67356	3,808586	3,671914	3,801652	3,729747	3,8000213	3,734437667	-0,06558367	0,955558652	Cngb3
17344852	3,95226	3,66708	3,748831	4,049039	3,486749	3,635645	3,7893903	3,723811	-0,06557933	0,955561522	Olfr97
17549444	3,867904	4,031264	3,84366	3,740411	3,821497	3,984184	3,914276	3,848697333	-0,06557867	0,955561964	---
17549832	3,867904	4,031264	3,84366	3,740411	3,821497	3,984184	3,914276	3,848697333	-0,06557867	0,955561964	---
17467128	9,54047	9,731635	9,430762	9,325721	9,532284	9,648147	9,5676223	9,502050667	-0,06557167	0,9555666	Nt5c3
17387819	3,038215	2,885121	3,180557	2,922466	2,900901	3,083832	3,034631	2,969066333	-0,06556467	0,955571237	---
17266421	4,564183	4,546783	4,542879	4,520733	4,608502	4,327934	4,5512817	4,485723	-0,06555867	0,955575211	---
17408040	4,114402	4,22083	4,451375	4,068171	4,127323	4,394439	4,2622023	4,196644333	-0,065558	0,955575652	Gm17580
17424119	6,675805	6,57788	6,874162	6,736039	6,576042	6,619183	6,7092823	6,643754667	-0,06552767	0,955595744	Aqp3
17416005	3,621994	3,684342	3,519362	3,679971	3,386896	3,562252	3,608566	3,543039667	-0,06552633	0,955596627	---
17411885	8,227249	8,243234	8,09027	8,021793	8,102556	8,23983	8,1869177	8,121393	-0,06552467	0,955597731	C430048L16Rik
17505197	9,94542	9,99748	9,795069	9,723932	9,942679	9,874815	9,9126563	9,847142	-0,06551433	0,955604576	Cirh1a
17450527	8,363849	8,585661	8,28539	8,308665	8,381415	8,34832	8,4116333	8,346133333	-0,0655	0,95561407	---
17259808	3,904636	3,718133	3,862462	3,694432	3,798896	3,795473	3,8284103	3,762933667	-0,06547667	0,955629525	---
17295362	9,26004	9,313585	9,213833	9,163698	9,283998	9,143358	9,262486	9,197018	-0,065468	0,955635266	Btf3
17277635	5,199914	4,873754	5,068299	5,169454	4,784352	4,991762	5,0473223	4,981856	-0,06546633	0,95563637	Gm8300
17234403	5,20249	5,357088	5,348782	5,280233	5,088029	5,343827	5,3027867	5,237363	-0,06542367	0,955664633	Ggt5
17333076	6,645691	6,457793	6,985076	6,916574	6,689328	6,286423	6,6961867	6,630775	-0,06541167	0,955672582	Gm8465
17417738	9,532897	9,812668	9,634067	9,529594	9,643761	9,610043	9,6598773	9,594466	-0,06541133	0,955672803	Hyi
17538074	4,764929	4,641033	4,821218	4,658141	4,775175	4,597637	4,7423933	4,676984333	-0,065409	0,955674348	Trap1a
17451780	4,981443	4,943191	5,06016	4,910532	4,969432	4,908608	4,9949313	4,929524	-0,06540733	0,955675452	Gm13842
17449649	4,649108	4,652264	4,64491	4,569534	4,688705	4,491888	4,6487607	4,583375667	-0,065385	0,955690246	U90926
17281791	8,265863	8,198603	8,198545	8,165515	8,088332	8,21304	8,2210037	8,155629	-0,06537467	0,955697092	2810055F11Rik
17281767	6,683331	6,977124	6,636253	6,655038	6,816563	6,629018	6,7655693	6,700206333	-0,065363	0,95570482	3110056K07Rik
17382937	6,945127	6,930326	7,115706	7,045943	6,950142	6,799046	6,997053	6,931710333	-0,06534267	0,95571829	Snapc4
17366067	7,064651	7,161371	6,956952	6,906576	7,003374	7,077048	7,0609913	6,995666	-0,06532533	0,955729773	D19Ertd737e // D19Ertd737e
17303706	7,363778	7,316539	7,432286	7,351803	7,346061	7,218895	7,3708677	7,305586333	-0,06528133	0,955758921	Gng2
17420846	8,467264	8,532248	8,549718	8,406767	8,455205	8,491453	8,51641	8,451141667	-0,06526833	0,955767534	Rcc2
17326504	8,238669	8,388214	8,22575	8,276749	8,217047	8,163035	8,284211	8,218943667	-0,06526733	0,955768196	---
17357233	9,117696	9,608714	9,261479	9,105542	9,246259	9,440304	9,3292963	9,264035	-0,06526133	0,955772171	---
17217035	12,1905	11,72939	12,03285	11,76695	12,0902	11,89981	11,984247	11,91898667	-0,06526	0,955773054	Ctse
17317233	9,702439	9,788633	9,75654	9,653774	9,72536	9,672741	9,749204	9,683958333	-0,06524567	0,95578255	Atad2
17207247	2,94797	3,875863	2,919825	3,408889	3,150065	2,989015	3,247886	3,182656333	-0,06522967	0,95579315	---
17200635	3,278464	3,353351	3,948562	3,698747	3,50725	3,178693	3,5267923	3,461563333	-0,065229	0,955793592	---
17359678	4,789943	4,846148	4,689689	4,714876	4,589379	4,825858	4,77526	4,710037667	-0,06522233	0,955798008	Scd3
17533890	5,06432	4,605572	4,343368	4,670165	4,522782	4,624653	4,6710867	4,605866667	-0,06522	0,955799554	---
17277837	6,784291	6,813349	6,876729	6,770701	6,694321	6,813772	6,8247897	6,759598	-0,06519167	0,955818326	Ttc8
17330662	4,625537	4,29332	4,613719	4,634235	4,200789	4,502008	4,5108587	4,445677333	-0,06518133	0,955825172	Tmprss7
17393004	6,171088	5,535084	6,110528	5,725694	5,996736	5,898754	5,9389	5,873728	-0,065172	0,955831355	Foxs1
17492947	5,117302	4,768113	5,080392	4,871525	4,873438	5,025351	4,9886023	4,923438	-0,06516433	0,955836435	9930013L23Rik
17496029	3,590473	3,597077	3,475564	3,549025	3,585112	3,333494	3,5543713	3,489210333	-0,065161	0,955838643	---
17227696	12,1819	12,17124	11,96112	12,0126	12,00898	12,09723	12,104753	12,03960333	-0,06515	0,955845931	Cfh
17433934	4,999311	4,894619	5,047272	4,883225	5,004563	4,857965	4,9804007	4,915251	-0,06514967	0,955846152	Tnfrsf18
17335138	5,151882	4,983397	5,349744	5,166103	5,051531	5,071952	5,1616743	5,096528667	-0,06514567	0,955848802	---
17364930	7,794126	7,156415	7,760414	8,026495	7,311977	7,177049	7,5703183	7,505173667	-0,06514467	0,955849465	---
17466382	3,834023	3,784641	3,692438	3,790343	3,642582	3,682755	3,7703673	3,705226667	-0,06514067	0,955852115	Olfr459
17246473	2,940345	3,148118	2,965293	3,005844	2,904111	2,948407	3,0179187	2,952787333	-0,06513133	0,955858299	Vmn2r84
17504143	6,582784	5,967858	6,622943	6,188483	6,446646	6,343063	6,391195	6,326064	-0,065131	0,95585852	Ciapin1 // Ciapin1
17329030	3,174487	3,064866	3,227476	3,348727	2,973396	2,949421	3,1556097	3,090514667	-0,065095	0,955882372	Olfr165
17291260	4,842162	4,762741	4,900899	4,758335	4,810198	4,742024	4,8352673	4,770185667	-0,06508167	0,955891206	Scgn
17329500	4,762035	4,807759	4,854706	4,769532	4,73725	4,722498	4,8081667	4,743093333	-0,06507333	0,955896727	Gm606
17204711	5,930274	4,195256	4,99447	4,4717	5,005836	5,447275	5,04	4,974937	-0,065063	0,955903574	---
17282465	6,79165	7,070295	7,249715	6,974378	7,017052	6,925072	7,03722	6,972167333	-0,06505267	0,955910421	C130039O16Rik
17403879	8,748315	8,721658	8,489316	8,501125	8,571833	8,691173	8,6530963	8,588043667	-0,06505267	0,955910421	Ankrd13c
17322829	7,324975	7,784084	7,74566	7,569724	7,520185	7,569724	7,6182397	7,553211	-0,06502867	0,955926323	Nubp1
17293665	7,887283	7,976953	7,942028	7,809021	7,887458	7,914781	7,9354213	7,87042	-0,06500133	0,955944434	Zfp367
17550657	3,975152	3,931271	4,120004	3,857553	4,074832	3,899069	4,008809	3,943818	-0,064991	0,955951281	---
17515196	7,883506	7,81234	7,895197	7,755007	7,874332	7,766741	7,863681	7,798693333	-0,06498767	0,95595349	Qtrt1
17532973	7,430166	7,681241	7,550679	7,564133	7,55829	7,344765	7,5540287	7,489062667	-0,064966	0,955967847	Gripap1
17460085	5,02262	5,13553	5,235714	5,054074	5,111894	5,033034	5,131288	5,066334	-0,064954	0,955975798	B230319C09Rik
17338540	3,69032	3,660037	3,92973	3,610951	3,670797	3,803498	3,760029	3,695082	-0,064947	0,955980437	Gm19585
17483733	7,038201	7,089628	7,110038	6,860342	7,109588	7,073099	7,079289	7,014343	-0,064946	0,955981099	Bag3
17211519	4,176092	4,037166	4,246017	4,145387	4,042172	4,076901	4,1530917	4,088153333	-0,06493833	0,955986179	---
17305662	9,23967	9,321907	9,282563	9,288494	9,220783	9,140059	9,28138	9,216445333	-0,06493467	0,955988609	Fermt2
17338852	7,828095	7,816244	7,947419	7,809526	7,744479	7,842986	7,8639193	7,798997	-0,06492233	0,955996782	Ranbp3
17287325	6,250237	6,756805	6,562176	6,529626	6,446486	6,398345	6,5230727	6,458152333	-0,06492033	0,955998107	S1pr3
17507829	3,122761	3,086359	3,127348	2,939525	3,118005	3,084208	3,112156	3,047246	-0,06491	0,956004954	Defb37
17549510	9,773241	10,14613	10,03076	10,00325	9,789406	9,962748	9,983377	9,918468	-0,064909	0,956005617	---
17469797	6,339686	5,91521	6,247645	6,156968	6,232632	5,918252	6,1675137	6,102617333	-0,06489633	0,956014011	Ghrl
17394803	6,676098	6,685819	7,160364	6,682878	6,855696	6,789092	6,8407603	6,775888667	-0,06487167	0,956030356	---
17420431	10,65196	10,54462	10,8636	10,73807	10,44152	10,68598	10,686727	10,62185667	-0,06487	0,956031461	---
17432387	7,361012	7,399551	7,281877	7,185541	7,368837	7,293493	7,34748	7,282623667	-0,06485633	0,956040517	Tmem51
17246209	6,475514	6,723352	6,798677	6,516682	6,560073	6,72623	6,6658477	6,600995	-0,06485267	0,956042947	Rpl41
17328422	3,329319	3,300665	3,351973	3,152791	3,375966	3,258661	3,327319	3,262472667	-0,06484633	0,956047144	---
17294421	10,13298	10,14149	10,12111	10,05255	10,0471	10,10141	10,13186	10,06702	-0,06484	0,956051341	Cast
17358503	9,257959	9,267319	9,391525	9,330163	9,355032	9,037148	9,305601	9,240781	-0,06482	0,956064595	Rcl1
17358749	3,878214	3,652718	3,801215	3,870277	3,639974	3,627455	3,7773823	3,712568667	-0,06481367	0,956068792	Lipn
17255097	5,293554	5,1387	5,144635	5,103436	5,108488	5,170608	5,1922963	5,127510667	-0,06478567	0,956087348	Car10
17366980	3,981999	4,047331	3,935003	3,864802	3,938517	3,966701	3,988111	3,92334	-0,064771	0,956097068	Gm13293
17520932	9,808864	9,768073	9,712415	9,667162	9,840878	9,587001	9,7631173	9,698347	-0,06477033	0,956097509	Topbp1
17439978	7,818841	7,946858	7,855061	7,70914	7,890352	7,82696	7,8735867	7,808817333	-0,06476933	0,956098172	Cdc7

17290938	5,958867	5,61798	5,510475	5,567079	6,048825	5,277143	5,695774	5,631015667	-0,06475833	0,956105462	---
17416897	8,819519	8,933736	8,74144	8,738326	8,804331	8,757843	8,831565	8,766833333	-0,06473167	0,956123135	Faf1
17318802	4,691976	4,4805	4,163511	4,426386	4,309379	4,406088	4,445329	4,380617667	-0,06471133	0,95613661	1700025B11Rik
17312196	5,024117	4,836393	5,066991	4,598624	5,194068	4,940713	4,9758337	4,911135	-0,06469867	0,956145005	Ly6k
17335609	4,894307	4,717721	4,915299	4,714442	4,761762	4,857055	4,8424423	4,777753	-0,06468933	0,956151191	---
17217821	7,47231	7,477759	7,397389	7,40488	7,392504	7,356011	7,4491527	7,384465	-0,06468767	0,956152295	Zfp281
17293085	8,656671	8,808134	8,807062	8,786556	8,541818	8,749455	8,757289	8,692609667	-0,06467933	0,956157818	Hnrnpa0
17335040	7,683163	7,393557	7,560598	7,402231	7,37457	7,666499	7,5457727	7,4811	-0,06467267	0,956162237	Ggnbp1
17468444	5,87509	5,469272	5,488097	5,865954	5,367928	5,404636	5,6108197	5,546172667	-0,064647	0,956179248	Cml2
17452453	5,679894	6,091577	6,362504	6,047714	5,958654	5,933671	6,0446583	5,980013	-0,06464533	0,956180352	---
17397601	5,040504	4,716456	5,03064	4,937002	4,628083	5,028596	4,9292	4,864560333	-0,06463967	0,956184108	---
17365953	5,114971	4,967502	5,239516	4,999907	5,131085	4,997105	5,1073297	5,042699	-0,06463067	0,956190073	B230217O12Rik
17415835	5,451128	5,634983	5,501785	5,472662	5,356993	5,564387	5,5292987	5,464680667	-0,064618	0,956198468	Ror1
17467124	6,334355	6,758634	6,578926	6,440119	6,54208	6,495898	6,557305	6,492699	-0,064606	0,956206422	Fkbp9 // Fkbp9
17209999	3,647238	3,573967	4,161805	3,467569	4,065514	3,656113	3,7943367	3,729732	-0,06460467	0,956207306	---
17372119	9,675243	9,706403	9,626803	9,481922	9,661335	9,671412	9,669483	9,604889667	-0,06459333	0,956214817	Mtx2
17547636	6,572255	6,273525	6,501502	6,325592	6,492618	6,335312	6,449094	6,384507333	-0,06458667	0,956219236	---
17331564	8,251752	8,382235	8,256256	8,070037	8,310177	8,316281	8,2967477	8,232165	-0,06458267	0,956221887	Nrip1
17202911	2,728599	3,737434	3,533848	3,247776	3,443055	3,115409	3,3332937	3,268746667	-0,064547	0,956245527	---
17249425	10,59649	10,77801	10,59309	10,53749	10,58228	10,65423	10,655863	10,59133333	-0,06453	0,956256795	Skp1a
17410304	3,140253	3,224107	3,348439	3,185003	3,037268	3,297033	3,2375997	3,173101333	-0,06449833	0,956277785	---
17524659	7,724904	7,788044	7,830616	7,79376	7,818526	7,537799	7,781188	7,716695	-0,064493	0,95628132	Keap1
17252887	4,264119	4,378358	4,558475	4,046421	4,722591	4,238469	4,4003173	4,335827	-0,06449033	0,956283088	Rpa1 // Rpa1
17282534	6,372776	6,288741	6,50868	6,393669	6,312495	6,270569	6,3900657	6,325577667	-0,064488	0,956284635	Abcd4
17521300	7,187018	7,333418	7,338188	7,183671	7,156971	7,324583	7,286208	7,221741667	-0,06446633	0,956298996	Cish
17495598	6,808532	6,766461	6,835939	6,610715	6,766132	6,840812	6,803644	6,739219667	-0,06442433	0,956326837	2310008H09Rik
17257595	5,06997	4,89589	4,924857	4,998411	4,928849	4,770194	4,9635723	4,899151333	-0,064421	0,956329046	---
17513908	6,384089	6,312776	6,444545	6,397212	6,263116	6,287852	6,38047	6,31606	-0,06441	0,956336338	Fanca
17303646	8,050577	8,298907	8,239477	8,159118	8,208969	8,027663	8,1963203	8,131916667	-0,06440367	0,956340536	Ube2e1
17364414	11,71051	11,78182	11,77075	11,68747	11,67143	11,71097	11,75436	11,68995667	-0,06440333	0,956340757	Cyp2c68
17403323	3,857503	3,78354	4,017911	4,162933	3,715179	3,587663	3,886318	3,821925	-0,064393	0,956347607	---
17479728	5,804995	5,804995	5,942983	5,635941	5,943894	5,780006	5,850991	5,786613667	-0,06437733	0,956357992	Fam103a1
17354502	6,171128	6,243972	6,257936	5,958932	6,360064	6,160913	6,2243453	6,159969667	-0,06437567	0,956359097	2210409D07Rik
17366081	8,487208	8,538588	8,352303	8,305325	8,448442	8,431213	8,4593663	8,394993333	-0,064373	0,956360865	2700078E11Rik
17240226	9,036295	8,991089	9,102343	8,98929	8,928198	9,019163	9,0432423	8,978883667	-0,06435867	0,956370366	Marcks
17535076	3,845303	3,847158	4,024796	3,845848	3,820348	3,857992	3,9057523	3,841396	-0,06435633	0,956371913	Zic3
17256607	6,799254	6,832433	6,986464	6,936136	6,755352	6,733609	6,872717	6,808365667	-0,06435133	0,956375228	Ramp2
17344221	10,62584	10,66094	10,65547	10,49875	10,66345	10,587	10,647417	10,58306667	-0,06435	0,956376112	Csnk2b
17402475	3,002692	3,108107	3,171567	3,032044	2,984557	3,072717	3,094122	3,029772667	-0,06434933	0,956376554	Mir302b
17497044	7,885363	7,977858	7,868239	7,842017	7,827478	7,868924	7,9104867	7,846139667	-0,064347	0,9563781	Ikzf5
17545075	5,170931	5,018476	5,000115	4,936946	5,048938	5,010623	5,063174	4,998835667	-0,06433833	0,956383846	Gm15032
17307663	6,582783	6,181499	6,800459	6,372557	6,595272	6,403901	6,5215803	6,457243333	-0,064337	0,95638473	Xkr6
17365621	3,421984	3,242688	3,173072	3,103958	3,371726	3,16908	3,279248	3,214921333	-0,06432667	0,95639158	---
17348877	5,706008	5,012787	5,366161	5,320134	5,300296	5,271593	5,361652	5,297341	-0,064311	0,956401965	---
17208583	3,583591	3,372462	3,704516	3,281077	3,446887	3,739676	3,553523	3,489213333	-0,06430967	0,956402849	---
17537632	4,45473	4,375268	4,642305	4,459056	4,437831	4,482513	4,4907677	4,426466667	-0,064301	0,956408595	Arl13a
17318461	8,450048	8,51533	8,335402	8,403701	8,291229	8,412951	8,4335933	8,369293667	-0,06429967	0,956409479	Sharpin
17361890	8,720484	8,798201	8,832382	8,770615	8,692778	8,694824	8,783689	8,719405667	-0,06428333	0,956420307	Capn1
17516752	4,560383	4,348395	4,568395	4,377434	4,512231	4,394723	4,492391	4,428129333	-0,06426167	0,95643467	BC049352
17497829	5,007761	4,793611	4,624	4,624239	4,890827	4,717546	4,8084573	4,744204	-0,06425333	0,956440195	Cdhr5
17352126	5,527223	5,490501	5,676307	5,613138	5,518597	5,369628	5,564677	5,500454333	-0,06422267	0,956460526	Rbfa
17545936	6,124306	6,100193	6,014788	5,993979	5,943984	6,108692	6,0797623	6,015551667	-0,06421067	0,956468481	Car5b
17480555	4,972821	4,948603	4,789813	4,905144	4,853258	4,760218	4,9037457	4,83954	-0,06420567	0,956471796	Klhl35
17418413	9,130765	9,210916	9,069744	9,13495	9,108191	8,975671	9,1371417	9,072937333	-0,06420433	0,95647268	Gnl2
17493643	6,463976	6,376847	6,329813	6,190128	6,444183	6,343759	6,390212	6,326023333	-0,06418867	0,956483067	Mogat2
17344881	3,44098	3,238823	3,442974	3,422421	3,259546	3,248373	3,374259	3,310113333	-0,06414567	0,956511576	Olfr112
17461477	6,358372	6,951398	6,495444	6,508352	6,674634	6,429842	6,601738	6,537609333	-0,06412867	0,956522847	Lmcd1
17410572	5,828385	5,320594	5,010266	5,117602	5,220747	5,628529	5,386415	5,322292667	-0,06412233	0,956527046	4933401H06Rik
17328107	4,350769	4,426515	4,407395	4,226907	4,433848	4,331573	4,394893	4,330776	-0,064117	0,956530582	Tnp2 // Tnp2
17409619	3,77233	3,462644	3,424112	3,57582	3,519233	3,371713	3,5530287	3,488922	-0,06410667	0,956537433	---
17205411	5,540688	5,380414	5,461681	5,355728	5,574387	5,26036	5,4609277	5,396825	-0,06410267	0,956540085	---
17341691	5,195926	4,909891	4,966619	4,941255	5,027038	4,911838	5,0241453	4,960043667	-0,06410167	0,956540748	---
17472893	5,884087	6,000996	6,13167	5,921757	6,057134	5,845599	6,0055843	5,941496667	-0,06408767	0,956550031	Gm5887
17208855	6,490907	6,030886	5,802059	5,805039	6,101453	6,2251	6,1079507	6,043864	-0,06408667	0,956550694	---
17526263	3,407722	3,363139	3,941113	3,615619	3,434703	3,469393	3,570658	3,506571667	-0,06408633	0,956550915	---
17422914	8,974367	8,957767	8,92487	8,989721	8,96018	8,714894	8,9523347	8,888265	-0,06406967	0,956561965	Noc2l
17544529	3,428666	3,341611	3,385911	3,288247	3,422765	3,253036	3,385396	3,321349333	-0,06404667	0,956577215	B230119M05Rik
17519303	5,00269	4,989847	4,858184	4,975059	4,861775	4,821794	4,9502403	4,886209333	-0,064031	0,956587603	Rsl24d1
17218446	4,601434	4,431112	4,549317	4,372566	4,316228	4,700993	4,5272877	4,463262333	-0,06402533	0,95659136	---
17326147	7,092553	7,183439	7,012993	7,033664	6,876565	7,186728	7,0963283	7,032319	-0,06400933	0,956601969	Ift57
17256473	9,024781	9,136014	9,054697	9,079161	8,922543	9,021847	9,0718307	9,007850333	-0,06398033	0,956621199	Atp6v0a1
17308476	12,03928	12,16491	12,27453	11,96079	12,17332	12,15267	12,159573	12,09559333	-0,06398	0,95662142	---
17204381	4,095355	4,192506	4,301082	4,196941	3,934158	4,265916	4,1963143	4,132338333	-0,063976	0,956624072	---
17307125	4,582207	4,270607	4,60725	4,330597	4,594859	4,342716	4,486688	4,422724	-0,063964	0,956632029	Gjb6
17209959	4,330806	4,044629	3,613782	4,132262	3,757641	3,907437	3,9964057	3,932446667	-0,063959	0,956635344	---
17548315	8,775676	8,931185	8,826532	8,42417	8,895161	9,022186	8,8444643	8,780505667	-0,06395867	0,956635565	LOC100862132
17208835	6,518304	6,160482	6,177033	6,785368	6,035315	5,843266	6,285273	6,221316333	-0,06395667	0,956636892	---
17439872	3,247568	3,615188	3,681956	3,742545	3,565279	3,045044	3,514904	3,450956	-0,063948	0,956642638	Zfp33b
17499983	8,50629	8,562061	8,369871	8,457924	8,343245	8,445277	8,4794073	8,415482	-0,06392533	0,956657769	Myst3
17280661	5,503294	5,616518	5,712811	5,586674	5,310343	5,743495	5,6107543	5,546837333	-0,063917	0,956663195	F730043M19Rik
17284995	4,408965	4,26916	4,443907	4,373445	4,311985	4,244863	4,3740107	4,310097667	-0,063913	0,956665847	---
17357981	5,394023	5,081837	5,210045	5,270547	4,996603	5,227024	5,228635	5,164724667	-0,06391033	0,956667615	Prune2
17334902	12,31431	12,36157	12,26421	12,24745	12,22669	12,27422	12,313363	12,24945333	-0,06391	0,956667836	---
17509146	7,354507	7,323059	7,309847	7,329508	7,207171	7,25916	7,3291377	7,265279667	-0,063858	0,956702319	Snx25
17439246	6,092526	5,666902	5,578133	5,947327	5,256076	5,942625	5,779187	5,715342667	-0,06384433	0,956711382	LOC100862180 // LOC100862166 // Gm7
17225775	6,865192	6,819301	6,738426	6,735811	6,638221	6,857372	6,8076397	6,743801333	-0,06383833	0,95671536	Thap4
17331852	3,26075	3,277917	3,181349	3,091861	3,255412	3,181349	3,2400053	3,176207333	-0,063798	0,956742108	Krtap13
17300390	4,475502	4,293333	4,66834	4,401363	4,416526	4,427896	4,4790583	4,415261667	-0,06379667	0,956742992	D830015G02Rik
17513963	3,463759	4,080454	3,788274	3,576596	3,797208	3,767325	3,7774957	3,713709667	-0,063786	0,956750066	---
17349367	3,209207	3,21067	3,203347	3,443675	2,99806	2,990139	3,2077413	3,143958	-0,06378333	0,956751834	---
17387660	3,004888	2,846391	3,600818	2,898521	3,306669	3,055571	3,150699	3,086920333	-0,06377867	0,956754929	Olfr1053
17295718	8,047823	8,2205	8,019999	8,006006	8,001608	8,089387	8,0961073	8,032333667	-0,06377367	0,956758245	Cdk7
17391259	5,659455	5,633742	5,917721	5,757435	5,509869	5,752314	5,7369727	5,673206	-0,06376667	0,956762887	Fahd2a
17422980	5,932157	6,016179	5,851933	5,786929	5,975324	5,846763	5,933423	5,869672	-0,063751	0,956773277	Rps20
17214564	6,091356	5,997362	6,253704	6,053022	6,006836	6,091356	6,1141407	6,050404667	-0,063736	0,956783225	Accn4
17548488	12,60432	12,54222	12,55837	12,43079	12,53799	12,54493	12,568303	12,50457	-0,06373333	0,956784993	Hmgb1
17372902	4,151277	4,532215	3,985142	3,936056	4,374504	4,166892	4,222878	4,159150667	-0,06372733	0,956788972	Olfr1178
17206071	4,654854	4,925833	4,917374	4,785657	4,162244	5,35899	4,832687	4,768963667	-0,06372333	0,956791625	---
17348250	9,781805	9,888404	9,856594	9,773983	9,777046	9,784636	9,8422677	9,778555	-0,06371267	0,956798699	Wac
17203669	6,431602	6,687561	6,704369	5,989981	7,03669	6,605726	6,607844	6,544132333	-0,06371167	0,956799362	---
17263073	5,491218	5,418653	5,518182	5,254646	5,477543	5,504764	5,4760177	5,412317667	-0,0637	0,9568071	---
17408135	8,384705	8,310181	8,392285	8,295311	8,369009	8,231758	8,3623903	8,298692667	-0,06369767	0,956808647	Gpr89
17362883	7,983712	8,398967	8,075933	8,229378	8,008361	8,029803	8,1528707	8,089180667	-0,06369	0,956813732	---
17206533	6,406189	6,138332	5,316018	5,626085	5,774383	6,269044	5,953513	5,889837333	-0,06367567	0,956823238	---
17527139	8,318785	8,354417	8,238549	8,209982	8,258821	8,251933	8,303917	8,240245333	-0,06367167	0,956825891	Cul5
17257327	3,34388	3,655556	4,098788	3,605995	3,773964	3,527257	3,699408	3,635738667	-0,06366933	0,956827438	---
17442958	5,724748	5,454555	5,579524	5,506354	5,474715	5,586775	5,5862757	5,522614667	-0,063661	0,956832965	2410018M08Rik
17447185	6,726505	6,633516	6,633616	6,682168	6,597712	6,522923	6,6645457	6,600934333	-0,06361133	0,956865906	Poln
17350262	3,696362	3,85138	3,888709	3,83635	3,713009	3,6963	3,8121503	3,748553	-0,06359733	0,956875192	Npy6r
17406279	5,907433	6,179429	6,058797	5,88798	5,906021	6,080925	6,048553	5,984975333	-0,06357767	0,956888236	Tlr2
17459306	4,241688	4,568656	3,506137	4,13855	3,809821	4,177444	4,1054937	4,041938333	-0,06355533	0,956903049	---
17223710	4,643647	4,806856	4,382013	4,593636	4,374159	4,67408	4,6108387	4,547291667	-0,063547	0,956908576	Gpr1
17510749	11,34317	11,33485	11,32073	11,1956	11,3541	11,25842	11,332917	11,26937333	-0,06354333	0,956911008	Abce1
17253494	5,522773	5,168376	5,195006	5,083693	5,454021	5,157882	5,295385	5,231865333	-0,06351967	0,956926706	Proca1
17367278	7,583874	7,617675	7,52536	7,570022	7,413293	7,553066	7,5756363	7,512127	-0,06350933	0,95693356	Commd3
17432687	5,843707	5,419839	5,847725	5,832593	5,602798	5,485373	5,703757	5,640254667	-0,06350233	0,956938203	Tnfrsf8
17457725	3,552824	3,568012	3,695447	3,562552	3,583311	3,479918	3,6054277	3,541927	-0,06350067	0,956939308	---
17461739	8,185621	8,321257	8,225401	8,210045	8,248838	8,08297	8,244093	8,180617667	-0,06347533	0,956956112	Fancd2
17547571	9,562782	9,476328	9,195936	9,26337	9,389784	9,391476	9,411682	9,34821	-0,063472	0,956958323	Ssbp1 // Ssbp1
17200965	7,804765	7,658004	6,773635	6,586298	7,609479	7,850325	7,4121347	7,348700667	-0,063434	0,956983529	---
17249379	5,113168	4,776428	4,837504	4,7641	4,842345	4,930417	4,9090333	4,845620667	-0,06341267	0,956997681	---
17548942	3,755255	3,81465	4,180411	3,847669	3,547567	4,164853	3,916772	3,853363	-0,063409	0,957000113	Gm19622
17200503	5,08645	4,247303	4,685812	4,873482	4,637191	4,318685	4,6731883	4,609786	-0,06340233	0,957004535	---
17526614	9,656227	9,746102	9,659637	9,568373	9,700849	9,602558	9,687322	9,623926667	-0,06339533	0,957009179	Pafah1b2
17536515	3,813405	3,568486	3,486243	3,587264	3,60242	3,488267	3,6227113	3,559317	-0,06339433	0,957009842	Mir676
17380340	8,211443	8,178747	8,084762	8,050182	8,163179	8,071414	8,1583173	8,094925	-0,06339233	0,957011169	Th1l
17512474	12,59188	12,80757	12,78969	12,72625	12,61389	12,65883	12,729713	12,66632333	-0,06339	0,957012716	Gm8841
17287175	7,67451	8,23273	8,18993	7,606314	8,041027	8,259664	8,03239	7,969001667	-0,06338833	0,957013822	Ogn
17525692	3,075839	3,365145	3,200648	3,234599	3,113716	3,103215	3,2138773	3,15051	-0,06336733	0,957027753	Olfr915
17307134	6,747972	6,918122	6,799664	6,906311	6,77385	6,595508	6,8219193	6,758556333	-0,063363	0,957030627	Cryl1
17432227	5,439934	5,384482	5,411028	5,470541	5,304268	5,270578	5,4118147	5,348462333	-0,06335233	0,957037703	Fblim1
17218907	7,370194	7,566739	7,708079	7,506503	7,577827	7,370637	7,5483373	7,484989	-0,06334833	0,957040356	Nme7
17270648	10,11643	10,11204	10,117	9,991133	10,07378	10,09056	10,115157	10,05182433	-0,06333233	0,95705097	Cdc27
17356961	4,088111	4,007649	4,249666	3,996484	3,973051	4,185903	4,115142	4,051812667	-0,06332933	0,957052961	Rps6ka4 // Rps6ka4

17270294	3,934615	4,024393	3,963894	3,933054	3,748067	4,051804	3,9743007	3,910975	-0,06332567	0,957055393	---
17370727	4,465075	3,672232	4,517967	4,30865	4,178686	3,977977	4,2184247	4,155104333	-0,06332033	0,957058931	Gm13498
17405795	8,133664	8,299131	8,064537	8,132283	8,099952	8,07515	8,1657773	8,102461667	-0,06331567	0,957062027	Kpna4
17521454	9,772657	9,776319	9,960031	9,739283	9,742183	9,837604	9,8363357	9,773023333	-0,06331233	0,957064238	Ifrd2
17238648	3,713456	3,369449	3,043061	3,403932	3,348672	3,183427	3,375322	3,312010333	-0,06331167	0,95706468	Olfr775
17302470	3,367236	3,26589	3,233192	3,290761	3,238194	3,147428	3,2887727	3,225461	-0,06331167	0,95706468	---
17549880	3,247188	3,268768	3,320242	3,166879	3,12197	3,357442	3,2787327	3,215430333	-0,06330233	0,957070872	---
17426565	4,561529	4,788587	4,615322	4,653345	4,536547	4,585646	4,655146	4,591846	-0,0633	0,95707242	Rasef
17359957	8,837171	8,762732	8,249612	8,319017	9,012686	8,328025	8,616505	8,553242667	-0,06326233	0,957097408	---
17231248	8,563011	8,327576	8,484749	8,255094	8,371507	8,558954	8,4584453	8,395185	-0,06326033	0,957098735	G0s2
17300209	4,359735	4,221741	4,430702	4,441869	4,149832	4,230709	4,3373927	4,274136667	-0,063256	0,95710161	---
17326510	8,236109	8,335101	8,075932	8,106106	8,127989	8,223302	8,215714	8,152465667	-0,06324833	0,957106696	Pros1
17528902	4,07076	3,956862	3,914259	3,864869	3,970963	3,916317	3,980627	3,917383	-0,063244	0,957109571	Gfral
17236878	7,606662	7,746636	7,789817	7,56464	7,717761	7,671071	7,7143717	7,651157333	-0,06321433	0,957129252	Galnt4
17418262	9,401991	9,676991	9,501881	9,371822	9,547464	9,471947	9,5269543	9,463744333	-0,06321	0,957132127	---
17332287	5,1318	5,043606	5,216281	5,218278	4,78991	5,193869	5,1305623	5,067352333	-0,06321	0,957132127	---
17456552	3,261134	3,208885	3,337616	3,378575	3,169215	3,070224	3,2692117	3,206004667	-0,063207	0,957134117	---
17389592	4,605961	4,717797	4,683543	4,715111	4,571812	4,530769	4,6691003	4,605897333	-0,063203	0,957136771	4930528P14Rik
17367052	4,124839	4,32063	4,238617	4,285353	4,167529	4,04166	4,2280287	4,164847333	-0,06318133	0,957151146	---
17447331	5,166044	5,240298	5,316419	5,218785	5,112236	5,202228	5,2409203	5,177749667	-0,06317067	0,957158222	Cpz
17227483	9,193316	9,428513	9,315549	9,322566	9,124698	9,300606	9,3124593	9,24929	-0,06316933	0,957159107	Camsap2
17435043	3,353548	2,931878	3,017532	2,994497	3,149237	2,969729	3,100986	3,037821	-0,063165	0,957161982	---
17371682	3,716379	3,909199	4,051402	3,741769	3,731697	4,014034	3,8923267	3,829166667	-0,06316	0,957165299	---
17474897	5,983732	5,576068	5,854693	5,967099	5,558545	5,699381	5,804831	5,741675	-0,063156	0,957167953	Zfp112
17541760	10,17029	10,01456	9,924764	9,844702	10,00536	10,0701	10,036538	9,973387333	-0,06315067	0,957171492	Mospd1
17381752	8,607244	8,928852	8,532359	8,681541	8,505756	8,691712	8,689485	8,626336333	-0,06314867	0,957172818	Fam188a
17342491	6,742691	6,109051	6,659238	6,560735	6,429428	6,331373	6,50366	6,440512	-0,063148	0,957173261	Wfikkn1
17386672	5,556015	5,910949	6,009109	5,757033	5,897117	5,632524	5,8253577	5,762224667	-0,063133	0,957183213	1700109F18Rik
17360817	5,570779	5,279517	5,238729	5,290933	5,270973	5,337765	5,3630083	5,299890333	-0,063118	0,957193165	Kcnk18
17536895	4,05859	4,040597	4,122262	4,00912	4,148702	3,874291	4,0738163	4,010704333	-0,063112	0,957197146	Dmrtc1b
17304155	6,679231	6,354081	6,819177	6,556018	6,500839	6,606454	6,6174963	6,554437	-0,06305933	0,95723209	Plac9
17304170	6,679231	6,354081	6,819177	6,556018	6,500839	6,606454	6,6174963	6,554437	-0,06305933	0,95723209	Plac9
17247543	8,646452	8,525275	8,540995	8,53026	8,479991	8,513301	8,5709073	8,507850667	-0,06305667	0,957233859	Sertad2
17496193	7,431365	7,439966	7,12072	7,403012	6,917421	7,482454	7,3306837	7,267629	-0,06305467	0,957235186	Cd19
17279805	7,52431	7,623923	7,562034	7,569096	7,434368	7,517678	7,570089	7,507047333	-0,06304167	0,957243812	Ncoa1
17536548	3,590278	3,595569	3,691691	3,621105	3,558378	3,50897	3,625846	3,562817667	-0,06302833	0,957252658	Arr3
17405119	4,554364	4,316652	4,324358	4,083394	4,604105	4,318848	4,398458	4,335449	-0,063009	0,957265486	Mgarp
17346036	5,246849	5,025896	5,276218	5,188348	5,032779	5,138822	5,1829877	5,119983	-0,06300467	0,957268362	Vmn2r118
17426032	5,619978	5,115575	4,959768	5,151425	5,177327	5,177598	5,2317737	5,168783333	-0,06299033	0,957277872	Mup19 // Mup16
17409736	7,682597	7,818	7,603417	7,67445	7,594493	7,646137	7,701338	7,63836	-0,062978	0,957286056	Slc35a3
17369218	7,486675	7,433233	7,368701	7,209504	7,251799	7,638388	7,4295363	7,366563667	-0,06297267	0,957289595	Dolpp1
17403844	2,911624	2,797972	2,938451	2,855391	2,793342	2,81041	2,8826823	2,819714333	-0,062968	0,957292691	---
17206175	5,063254	5,729237	4,962769	5,70175	4,465996	5,398651	5,2517533	5,188799	-0,06295433	0,95730176	---
17371011	4,54137	4,466019	4,688129	4,768239	4,325764	4,412686	4,5651727	4,502229667	-0,062943	0,95730928	Dapl1
17274015	6,950645	7,030451	6,783471	6,751278	6,842004	6,982461	6,9215223	6,858581	-0,06294133	0,957310386	Ttc32
17427563	6,982742	6,634468	6,778572	6,78423	6,717906	6,704824	6,798594	6,735653333	-0,06294067	0,957310828	Itgb3bp
17288463	7,101667	7,248741	7,054278	6,930087	7,128898	7,156885	7,1348953	7,071956667	-0,06293867	0,957312156	Mrpl36
17477468	6,619797	6,285291	6,679629	6,524177	6,352791	6,51895	6,528239	6,465306	-0,062933	0,957315916	Lrrc4b
17351919	8,174914	8,239991	7,966773	7,833946	8,185871	8,173075	8,127226	8,064297333	-0,06292867	0,957318791	Hdhd2
17523281	7,819146	7,922351	7,898739	7,713806	7,882592	7,855083	7,8800787	7,817160333	-0,06291833	0,957325648	Trak1
17414245	4,988234	5,158473	4,887421	5,014524	4,672498	5,158379	5,011376	4,948467	-0,062909	0,957331841	Tal2
17349601	8,974737	8,757948	8,956603	8,721318	8,847602	8,931661	8,8964293	8,833527	-0,06290233	0,957336265	Cxxc5
17501080	8,617852	8,868567	8,659068	8,536654	8,680331	8,739837	8,7151623	8,652274	-0,06288833	0,957345555	Rwdd4a
17329220	9,888523	9,804041	9,645001	9,668625	9,597154	9,883162	9,7791883	9,716313667	-0,06287467	0,957354624	Ehhadh
17387932	3,983766	3,926208	3,871749	3,73682	4,237012	3,619282	3,927241	3,864371333	-0,06286967	0,957357942	Olfr1233
17489247	8,404344	8,3241	8,774613	8,235955	8,70997	8,368537	8,501019	8,438154	-0,062865	0,957361039	---
17404935	4,178289	3,84853	3,645528	3,901976	3,912165	3,669622	3,8907823	3,827921	-0,06286133	0,957363472	---
17201085	6,09894	6,007388	6,00764	5,676967	6,355819	5,892616	6,0379893	5,975134	-0,06285533	0,957367454	---
17408845	9,933741	10,03062	9,829721	9,798079	9,872699	9,934775	9,9313607	9,868517667	-0,062843	0,957375638	Rap1a
17531080	8,062309	8,370255	8,300909	8,172289	8,169804	8,202857	8,244491	8,18165	-0,062841	0,957376965	Rnf123
17263609	8,310647	8,400924	8,245474	8,20456	8,250553	8,313416	8,319015	8,256176333	-0,06283867	0,957378514	Flii
17479317	10,40112	10,49263	10,35547	10,25602	10,40894	10,39581	10,416407	10,35359	-0,06281667	0,957393113	Isg20
17403551	12,25327	12,38127	12,2262	12,23122	12,14253	12,29856	12,286913	12,22410333	-0,06281	0,957397537	Uox
17514786	4,94485	5,156508	5,009925	5,022364	4,872953	5,027538	5,0370943	4,974285	-0,06280933	0,95739798	---
17206073	4,427052	3,957997	4,314883	4,181009	4,28407	4,046446	4,2333107	4,170508333	-0,06280233	0,957402625	---
17374238	10,61534	10,54841	10,5966	10,46231	10,59678	10,51287	10,586783	10,52398667	-0,06279667	0,957406385	Nop10
17475616	5,241683	5,045197	5,148748	5,239203	5,025405	4,982644	5,1452093	5,082417333	-0,062792	0,957409482	Hipk4
17432927	5,613804	5,437401	5,972862	5,626554	5,582589	5,626554	5,674689	5,611899	-0,06279	0,95741081	Fbxo44
17453205	8,884441	8,950531	8,926834	8,824837	8,858475	8,890162	8,920602	8,857824667	-0,06277733	0,957419216	Sbds
17530300	6,766949	7,265255	7,22192	6,892833	7,274826	6,898136	7,084708	7,021931667	-0,06277633	0,957419879	---
17393357	8,435053	8,708284	8,591717	8,628461	8,471613	8,446684	8,5783513	8,515586	-0,06276533	0,957427179	Eif6
17210869	9,861057	9,938726	9,906928	9,797381	9,824729	9,896366	9,902237	9,839492	-0,062745	0,957440673	Tcea1
17218933	3,541462	3,721722	3,652858	3,492953	3,52102	3,713842	3,6386807	3,575938333	-0,06274233	0,957442443	LOC433374
17426000	6,217018	5,885748	5,698064	5,969425	5,62495	6,018269	5,93361	5,870881333	-0,06272867	0,957451513	Mup13
17463454	5,809987	5,790903	5,760641	5,67871	5,841023	5,653677	5,787177	5,72447	-0,062707	0,957465892	Clec2i
17499279	11,16741	11,13595	11,08191	10,97217	11,09244	11,13255	11,128423	11,06572	-0,06270333	0,957468326	Lamp1
17207093	2,79039	3,095549	2,890476	2,892665	2,861767	2,833909	2,9254717	2,862780333	-0,06269133	0,95747629	---
17203967	5,636422	5,944222	5,312364	5,791204	5,842005	5,071738	5,6310027	5,568315667	-0,062687	0,957479166	---
17548375	7,84737	8,235321	7,987728	8,004724	7,982121	7,895592	8,023473	7,960812333	-0,06266067	0,957496643	---
17531183	5,235632	5,039252	5,145378	5,015873	5,109772	5,106649	5,1400873	5,077431333	-0,062656	0,95749974	Ccdc36
17241789	8,726077	8,525092	8,557834	8,484915	8,422001	8,714168	8,603001	8,540361333	-0,06263967	0,95751058	Cisd1
17314159	5,857329	6,009696	6,301536	5,934338	5,981113	6,065208	6,056187	5,993553	-0,062634	0,957514341	Chkb // Chkb
17317013	9,910771	10,0602	10,24811	9,941956	9,982569	10,10668	10,073027	10,01040167	-0,06262533	0,957520093	Gm10020
17525788	3,536275	3,804329	3,559595	3,61352	3,724386	3,374422	3,6333997	3,570776	-0,06262367	0,957521199	Olfr979
17355131	5,441679	5,578999	5,727653	5,794158	5,245612	5,5207	5,582777	5,520156667	-0,06262033	0,957523412	Mir694
17297161	7,724662	7,796451	7,64672	7,60963	7,62122	7,749135	7,722611	7,659995	-0,062616	0,957526288	3830406C13Rik
17237787	5,199886	4,918501	5,047861	5,042192	4,886825	5,04941	5,055416	4,992809	-0,062607	0,957532261	Fam19a2
17505790	4,589413	4,412714	4,462447	4,371316	4,36559	4,539858	4,4881913	4,425588	-0,06260333	0,957534695	Cntnap4
17550841	3,872538	3,744719	3,829608	3,791241	3,755558	3,71228	3,8156217	3,753026333	-0,06259533	0,957540004	---
17351634	6,181226	6,304228	6,268544	6,190634	6,134222	6,24136	6,2513327	6,188738667	-0,062594	0,957540889	Ccdc68
17527465	6,362886	6,232205	6,121629	6,315974	6,276793	5,936221	6,2389067	6,176329333	-0,06257733	0,957551951	Gm10658
17531693	8,430479	8,391379	8,170313	8,217146	8,028201	8,559155	8,3307237	8,268167333	-0,06255633	0,95756589	Rtp3
17359421	4,332738	4,289166	4,217826	4,260864	4,156235	4,234988	4,27991	4,217362333	-0,06254767	0,957571642	Dntt
17434621	3,59545	3,423146	3,438469	3,418048	3,312219	3,539171	3,4856883	3,423146	-0,06254233	0,957575182	---
17449880	6,000062	6,022151	6,031817	6,104448	5,92021	5,841769	6,01801	5,955475667	-0,06253433	0,957580492	A430089I19Rik
17387638	3,308694	3,455277	3,528377	3,469838	3,408334	3,226609	3,4307827	3,368260333	-0,06252233	0,957588457	Olfr1042
17406831	4,686179	4,781247	4,729211	4,663795	4,579762	4,765514	4,7322123	4,669690333	-0,062522	0,957588678	Syt11
17210691	3,247492	3,350069	3,328801	3,401019	3,50634	2,831512	3,3087873	3,246290333	-0,062497	0,957605272	---
17484263	3,79074	3,75012	3,624405	3,504068	3,755396	3,718365	3,721755	3,659276333	-0,06247867	0,957617441	6330420H09Rik
17440387	8,414071	8,357553	8,323833	8,286111	8,373168	8,248745	8,3651523	8,302674667	-0,06247767	0,957618105	Chfr
17241454	8,765092	8,869015	8,760695	8,708483	8,68304	8,815849	8,7982673	8,735790667	-0,06247667	0,957618769	Ddx50
17473595	4,963507	4,838958	4,869748	5,089458	4,654783	4,740549	4,8907377	4,828263333	-0,06247433	0,957620317	Zfp78
17541008	9,315577	9,597545	9,625463	9,610224	9,510504	9,230488	9,5128617	9,450405333	-0,06245633	0,957632265	Snora69
17224386	5,118794	4,973583	5,124792	4,844197	4,972388	5,21322	5,0723897	5,009935	-0,06245467	0,957633372	Cryba2
17202781	7,132375	6,762664	7,185866	7,146749	6,701264	7,045535	7,0269683	6,964516	-0,06245233	0,95763492	---
17527171	3,040012	3,480905	2,909862	2,968346	3,113231	3,161845	3,143593	3,081140667	-0,06245233	0,95763492	---
17446622	4,661298	4,652869	4,598117	4,596741	4,471116	4,657082	4,637428	4,574979667	-0,06244833	0,957637576	A230098N10Rik
17255454	5,949016	5,83851	6,053164	5,788756	5,725525	6,139123	5,9468967	5,884468	-0,06242867	0,95765063	B130006D01Rik
17401987	7,3682	7,262003	7,152281	7,195599	7,241523	7,158117	7,260828	7,198413	-0,062415	0,957659702	Dph5
17436237	7,078676	7,3695	7,502474	7,298565	7,346189	7,118654	7,3168833	7,254469333	-0,062414	0,957660366	Fosl2
17432287	8,349812	8,529463	8,397226	8,396092	8,37827	8,314916	8,4255003	8,363092667	-0,06240767	0,95766457	Ddi2
17262020	5,169188	5,261383	4,939832	5,06514	5,255191	4,86287	5,1234677	5,061067	-0,06240067	0,957669216	---
17331088	4,52161	4,594306	4,888525	4,71877	4,649181	4,449352	4,668147	4,605767667	-0,06237933	0,957683378	Gm19797
17503528	5,171572	4,820762	5,388754	5,036669	5,074982	5,082301	5,1270293	5,064650667	-0,06237867	0,95768382	Gm10638
17205189	3,541075	4,123989	5,123268	4,213559	4,178288	4,209357	4,2627773	4,200401333	-0,062376	0,95768559	---
17209379	3,541075	4,123989	5,123268	4,213559	4,178288	4,209357	4,2627773	4,200401333	-0,062376	0,95768559	---
17507234	2,951107	3,004387	2,881186	2,936205	2,861528	2,851823	2,94556	2,883185333	-0,06237467	0,957686476	---
17225813	3,297911	3,236485	3,210276	3,201039	3,162911	3,193649	3,248224	3,185866333	-0,06235767	0,957697761	---
17276662	5,433245	4,827845	5,757292	5,497824	5,197577	5,135937	5,3394607	5,277112667	-0,062348	0,957704178	---
17546073	3,353563	3,627053	3,611314	3,420961	3,410613	3,573364	3,5306433	3,468312667	-0,06233067	0,957715684	---
17274611	5,503294	5,539432	5,712413	5,587687	5,472529	5,507939	5,5850463	5,522718333	-0,062328	0,957717454	Gm17746
17460107	5,908669	5,814138	5,913462	5,858194	5,777174	5,813948	5,8787563	5,816438667	-0,06231767	0,957724314	Atp6v1b1
17487161	6,893824	6,656986	6,664602	6,776751	6,7164	6,535323	6,7384707	6,676158	-0,06231267	0,957727633	Qpctl
17340482	5,015107	4,738761	4,776376	4,706073	4,697252	4,940008	4,8434147	4,781111	-0,06230367	0,957733608	4930579D07Rik
17216931	4,169805	4,47869	4,136731	4,367155	4,118198	4,113002	4,261742	4,199451667	-0,06229033	0,957742459	---
17507573	5,507975	5,599101	5,828364	5,600426	5,786172	5,361996	5,6451467	5,582864667	-0,062282	0,957747991	Adprhl1
17211347	4,424139	3,870875	3,611674	4,025866	3,699456	3,994535	3,968896	3,906619	-0,062277	0,957751311	Tfap2b
17393684	9,276221	9,329132	9,244501	9,175132	9,213852	9,274053	9,2832847	9,221012333	-0,06227233	0,957754409	Rbl1
17380108	8,20669	8,166082	8,130371	8,093954	8,048565	8,173876	8,1677143	8,105465	-0,06224933	0,957769678	Cstf1
17262621	10,12607	10,21796	10,04211	9,950508	10,11007	10,13885	10,128713	10,066476	-0,06223733	0,957777644	Hspa4
17459312	6,126348	5,79974	6,221776	6,009246	6,049279	5,902656	6,049288	5,987060333	-0,06222767	0,957784062	---
17336829	7,16534	7,401125	7,356623	7,156555	7,323526	7,256325	7,307696	7,245468667	-0,06222733	0,957784283	Lsm2
17271724	4,258581	4,546491	4,360274	4,492391	4,126012	4,360274	4,3884487	4,326225667	-0,062223	0,95778716	Cd300ld
17429810	7,051694	6,76649	6,621306	6,731214	6,751001	6,770676	6,8131633	6,750963667	-0,06219967	0,957802651	Akirin1

17430767	8,243857	8,420331	8,384556	8,274328	8,269213	8,318604	8,3495813	8,287381667	-0,06219967	0,957802651	Ythdf2
17469040	6,915491	7,062793	7,052761	6,935246	7,03251	6,87669	7,0103483	6,948148667	-0,06219967	0,957802651	Xpc
17203961	7,48414	7,545509	7,555464	7,594745	7,399846	7,403926	7,528371	7,466172333	-0,06219867	0,957803315	---
17362261	8,349855	8,371042	8,422887	8,312704	8,267426	8,377196	8,3812613	8,319108667	-0,06215267	0,957833854	Otub1
17485772	5,45459	5,737189	5,611156	5,594351	5,477961	5,544188	5,6009783	5,538833333	-0,062145	0,957838944	Tnnt1
17262413	3,802812	3,683555	3,945234	3,766236	3,712732	3,766236	3,8105337	3,748401333	-0,06213233	0,957847354	Grm6
17520537	5,711931	5,955834	5,864052	5,778781	5,836664	5,730004	5,843939	5,781816333	-0,06212267	0,957853772	Gk5
17421033	5,888031	6,050654	5,845178	5,831826	5,926378	5,839296	5,9279543	5,865833333	-0,062121	0,957854879	Epha2
17372592	8,421181	8,418886	8,383799	8,225707	8,464303	8,347505	8,4079553	8,345838333	-0,062117	0,957857535	Ypel4
17419648	6,45012	6,08281	6,31376	6,332174	6,143863	6,184344	6,28223	6,220127	-0,062103	0,95786683	---
17451731	3,445664	3,478843	3,773302	3,649568	3,54894	3,313011	3,5659363	3,503839667	-0,06209667	0,957871035	---
17348295	9,337032	9,557055	9,324043	9,272284	9,262628	9,497007	9,4060433	9,343973	-0,06207033	0,957888519	Thoc1
17377478	6,951729	6,937864	6,794713	6,788897	6,867101	6,842158	6,8947687	6,832718667	-0,06205	0,957902019	Entpd6
17428082	5,001522	5,024084	5,18934	5,053056	5,06961	4,906172	5,0716487	5,009612667	-0,062036	0,957911315	---
17378784	8,66621	8,624098	8,63562	8,544173	8,519001	8,676649	8,641976	8,579941	-0,062035	0,957911979	Rprd1b
17233716	6,426593	6,456075	6,659401	6,358594	6,634633	6,362764	6,514023	6,451997	-0,062026	0,957917955	Npffr1
17387800	4,234689	3,986357	3,965019	3,894424	3,854867	4,2507	4,0620217	3,999997	-0,06202467	0,95791884	Olfr1162
17540109	3,690116	3,384692	3,6841	3,526999	3,281518	3,764327	3,5863027	3,524281333	-0,06202133	0,957921053	Ssxb6
17324674	8,815987	9,029118	8,714892	8,703403	8,939154	8,731393	8,8533323	8,791316667	-0,06201567	0,957924816	---
17416251	8,850802	8,910274	8,764402	8,748456	8,855751	8,735233	8,841826	8,779813333	-0,06201267	0,957926808	Usp24
17401846	7,128664	7,056861	6,969768	6,893929	7,110004	6,965327	7,0517643	6,989753333	-0,062011	0,957927914	Vav3
17270682	7,705647	7,487561	7,92934	7,700947	7,631235	7,60434	7,707516	7,645507333	-0,06200867	0,957929464	1700052K11Rik
17285801	2,714154	2,951605	3,098343	2,856575	2,939234	2,782308	2,9213673	2,859372333	-0,061995	0,957938538	Vmn1r221
17250301	5,313037	5,421334	5,331372	5,371406	5,264362	5,244015	5,3552477	5,293261	-0,06198667	0,957944071	Gm16062
17213943	3,02375	2,998684	2,949195	2,817074	3,093875	2,874729	2,990543	2,928559333	-0,06198367	0,957946063	---
17536448	3,01444	2,933871	3,311956	3,004688	3,125305	2,944341	3,0867557	3,024778	-0,06197767	0,957950047	---
17276817	3,290932	3,37587	3,313767	2,95298	3,45537	3,386302	3,3268563	3,264884	-0,06197233	0,957953589	---
17428799	8,851245	9,280513	9,168346	9,172909	9,066424	8,874861	9,1000347	9,038064667	-0,06197	0,957955138	LOC100862183 // Gm15501 // Rps8
17295381	4,795359	4,701658	4,785758	4,972829	4,422036	4,70206	4,760925	4,698975	-0,06195	0,957968418	Tmem174
17301854	6,890424	6,705168	6,821983	6,664285	6,651255	6,916222	6,8058583	6,743920667	-0,06193767	0,957976608	Rcbtb2
17238882	7,199811	7,544982	7,271902	7,365447	7,233381	7,232081	7,3388983	7,276969667	-0,06192867	0,957982584	Syne1
17284795	7,997256	7,899302	7,926765	7,847417	7,867505	7,922636	7,9411077	7,879186	-0,06192167	0,957987232	Sp4
17424319	7,517191	7,486409	7,464477	7,333966	7,475881	7,472478	7,489359	7,427441667	-0,06191733	0,95799011	Sigmar1
17355738	7,557148	7,438268	7,190014	7,480549	7,206939	7,312194	7,3951433	7,333227333	-0,061916	0,957990995	Zfp236
17348398	6,843139	6,928044	7,023904	7,015879	6,802189	6,791302	6,9316957	6,86979	-0,06190567	0,957997857	Mir1b
17389309	3,959868	3,79423	3,621608	3,859212	3,744872	3,585943	3,791902	3,730009	-0,061893	0,958006268	Olfr1313
17458520	10,08143	10,03258	10,0673	10,05583	10,03819	9,901652	10,060437	9,998557333	-0,06187933	0,958015343	Mpp6
17527606	6,166195	6,537365	6,597055	6,357962	6,57805	6,178986	6,4335383	6,371666	-0,06187233	0,958019991	Arid3b
17215429	6,776335	6,808873	6,800369	6,740033	6,710975	6,748972	6,7951923	6,733326667	-0,06186567	0,958024418	Ugt1a10
17549164	3,405684	3,596958	3,458413	3,430216	3,557638	3,287642	3,4870183	3,425165333	-0,061853	0,95803283	---
17533413	10,99186	11,31883	11,49978	11,02488	11,39809	11,20197	11,270157	11,20831333	-0,06184333	0,958039249	Rpl3
17201773	4,920858	4,885465	4,833489	4,876632	4,828669	4,748981	4,8799373	4,818094	-0,06184333	0,958039249	---
17201949	4,920858	4,885465	4,833489	4,876632	4,828669	4,748981	4,8799373	4,818094	-0,06184333	0,958039249	---
17295304	7,973416	8,291453	8,053294	8,143284	7,900886	8,088484	8,1060543	8,044218	-0,06183633	0,958043897	---
17374047	4,030912	3,891248	3,986264	3,966291	3,722092	4,034544	3,9694747	3,907642333	-0,06183233	0,958046554	Kcna4
17550637	2,797099	2,80284	2,865289	2,768263	2,803658	2,707844	2,8217427	2,759921667	-0,061821	0,95805408	---
17423799	4,701617	4,85764	4,697487	4,622369	4,785828	4,663098	4,752248	4,690431667	-0,06181633	0,958057179	---
17263362	7,382034	7,555105	7,584601	7,436614	7,489705	7,410069	7,5072467	7,445462667	-0,061784	0,958078651	Guk1
17326370	10,75674	10,81888	10,72278	10,74629	10,67686	10,68991	10,766133	10,70435333	-0,06178	0,958081307	Tomm70a
17278251	2,903877	3,169719	2,839076	2,906282	3,025416	2,795671	2,9708907	2,909123	-0,06176767	0,958089498	---
17329937	9,31356	9,225551	9,188488	9,162827	9,200728	9,178776	9,242533	9,180777	-0,061756	0,958097246	Umps
17404470	5,194676	4,621337	5,212869	4,942636	4,918357	4,982644	5,0096273	4,947879	-0,06174833	0,958102337	1700112D23Rik
17362689	8,350208	8,490541	8,239924	8,297091	8,253967	8,344393	8,3602243	8,298483667	-0,06174067	0,958107428	Sdhaf2
17544267	3,321769	3,326132	3,291411	3,249779	3,313104	3,191241	3,313104	3,251374667	-0,06172933	0,958114955	---
17489100	5,700743	5,661855	5,922348	5,715586	5,740843	5,643347	5,7616487	5,699925333	-0,06172333	0,95811894	Arhgap33
17202225	6,998775	6,687226	6,765582	6,776018	6,944825	6,545574	6,8171943	6,755472333	-0,061722	0,958119825	---
17292139	7,790986	7,966734	7,938641	7,743511	7,826672	7,941036	7,898787	7,837073	-0,061714	0,958125138	Ccdc90a
17382851	4,504398	4,668463	5,006296	4,848962	4,43939	4,705662	4,7263723	4,664671333	-0,061701	0,958133772	Lhx3
17456084	6,775689	6,703965	6,625961	6,738999	6,41977	6,761772	6,7018717	6,640180333	-0,06169133	0,958140192	Foxp2
17249168	4,91153	4,907366	5,238788	4,933283	4,923352	5,01604	5,019228	4,957558333	-0,06166967	0,958154581	---
17522107	5,326361	5,140462	5,211737	5,16594	5,066963	5,260761	5,2261867	5,164554667	-0,061632	0,958179598	Ucn2
17304010	4,697807	4,850877	4,400966	4,454075	4,820565	4,490157	4,6498833	4,588265667	-0,06161767	0,958189117	A830039N20Rik
17322911	10,52236	10,9241	10,63203	10,55057	10,65679	10,68629	10,69283	10,63121667	-0,06161333	0,958191995	---
17205191	4,2325	4,051157	4,412516	4,130441	4,408127	3,972791	4,2320577	4,170453	-0,06160467	0,958197752	---
17209381	4,2325	4,051157	4,412516	4,130441	4,408127	3,972791	4,2320577	4,170453	-0,06160467	0,958197752	---
17270807	6,928916	6,861625	6,863907	6,844044	6,837281	6,788389	6,884816	6,823238	-0,061578	0,958215463	Smarcd2
17510581	8,664244	8,779791	8,562962	8,733999	8,52688	8,561401	8,668999	8,607426667	-0,06157233	0,958219227	---
17218682	6,551466	6,765399	6,629789	6,557875	6,436408	6,767687	6,6488847	6,587323333	-0,06156133	0,958226533	Cenpl
17467730	8,677978	8,832095	8,520021	8,762366	8,557181	8,525867	8,676698	8,615138	-0,06156	0,958227418	Rnf181
17540332	7,760229	7,938131	7,801136	7,871942	7,793037	7,64992	7,8331653	7,771633	-0,06153233	0,958245795	Cask
17550582	7,502965	7,662335	7,276059	7,27166	7,682865	7,302265	7,480453	7,41893	-0,061523	0,958251994	---
17488157	8,064172	8,06631	8,198679	7,945647	8,266125	7,932841	8,1097203	8,048204333	-0,061516	0,958256643	Snrpa
17491362	4,108352	3,773938	3,796539	3,915014	3,814065	3,765229	3,892943	3,831436	-0,061507	0,958262621	Mrgprb2
17310357	5,301476	5,054283	5,533745	5,181705	5,01006	5,513235	5,2965013	5,235	-0,06150133	0,958266385	Slc45a2
17205231	4,337978	4,041401	5,478046	4,853389	4,655722	4,163873	4,6191417	4,557661333	-0,06148033	0,958280334	---
17209421	4,337978	4,041401	5,478046	4,853389	4,655722	4,163873	4,6191417	4,557661333	-0,06148033	0,958280334	---
17518342	5,892704	5,626354	5,786969	5,376801	5,857117	5,887676	5,7686757	5,707198	-0,06147767	0,958282105	Megf11
17419940	8,446589	8,613956	8,508355	8,480207	8,242712	8,661579	8,5229667	8,461499333	-0,06146733	0,958288969	Srsf10
17346850	4,584895	4,621589	4,679073	4,383007	4,769644	4,548524	4,628519	4,567058333	-0,06146067	0,958293397	9130404H23Rik
17308474	12,09443	12,12239	12,11689	11,81426	12,12749	12,29758	12,141237	12,07977667	-0,06146	0,95829384	---
17221768	8,963734	8,865284	8,807981	8,72138	8,804722	8,926628	8,8789997	8,817576667	-0,061423	0,958318417	B3gat2 // B3gat2
17539056	2,757598	2,85296	3,104593	2,859449	2,89196	2,77953	2,9050503	2,843646333	-0,061404	0,958331038	---
17439976	5,505998	5,44979	5,857882	5,391671	5,609842	5,628005	5,6045567	5,543172667	-0,061384	0,958344323	---
17276249	8,971685	8,918771	8,979489	8,833146	8,918364	8,934321	8,9566483	8,895277	-0,06137133	0,958352738	Ppm1a
17225210	5,81866	5,871596	5,710629	5,677833	5,781351	5,757591	5,800295	5,738925	-0,06137	0,958353623	Alpi
17344349	4,958968	4,863272	5,045136	4,92883	4,427435	5,027018	4,955792	4,894427667	-0,06136433	0,958357388	Dpcr1
17310546	2,998331	3,01918	3,109306	2,950101	2,991295	3,001351	3,0422723	2,980915667	-0,06135667	0,95836248	---
17464549	4,78503	4,468504	4,363862	4,51399	4,446794	4,472556	4,539132	4,47778	-0,061352	0,95836558	Calcr
17548707	4,526692	4,354908	4,792546	4,737456	4,385386	4,367263	4,5580487	4,496701667	-0,061347	0,958368902	LOC100503303
17513871	7,7927	7,947068	7,862143	7,728471	7,858629	7,830787	7,8673037	7,805962333	-0,06134133	0,958372666	Chmp1a
17271622	6,829144	6,904027	6,836946	6,656864	6,769742	6,959528	6,8567057	6,795378	-0,06132767	0,958381745	Cdc42ep4
17237275	4,659201	5,045514	4,656627	4,9706	4,432355	4,774427	4,787114	4,725794	-0,06132	0,958386838	Gm5176
17254188	7,7649	8,157054	7,685505	7,830917	7,670626	7,921971	7,869153	7,807838	-0,061315	0,958390159	Slfn3
17405365	7,33507	7,402869	7,365981	7,354502	7,285783	7,2797	7,3679733	7,306661667	-0,06131167	0,958392374	Wwtr1
17294930	3,967707	3,98244	3,846014	3,812117	3,965302	3,834807	3,9320537	3,870742	-0,06131167	0,958392374	Spz1
17268627	5,149924	5,189385	5,519726	5,273425	5,180944	5,220736	5,286345	5,225035	-0,06131	0,958393481	1700001P01Rik
17384587	9,219906	9,336789	9,15237	9,125914	9,231194	9,168082	9,236355	9,175063333	-0,06129167	0,95840566	Rc3h2
17364189	4,683084	4,305005	4,385064	4,421292	4,567085	4,200905	4,4577177	4,396427333	-0,06129033	0,958406546	F530104D19Rik
17284519	6,121057	5,889256	5,99787	5,868427	5,984642	5,971249	6,0027277	5,941439333	-0,06128833	0,958407874	---
17311064	4,655892	4,189627	4,655632	4,490403	4,266604	4,560298	4,5003837	4,439101667	-0,061282	0,958412082	Polr2k
17289009	4,564005	4,368016	4,247787	4,505119	4,187864	4,302993	4,3932693	4,331992	-0,06127733	0,958415182	Hapln1
17346926	5,334065	5,366962	5,425402	5,347272	5,293411	5,301993	5,3754763	5,314225333	-0,061251	0,958432676	C030034I22Rik
17419411	12,45352	12,55779	12,52108	12,42813	12,52183	12,39868	12,510797	12,44954667	-0,06125	0,95843334	Snord99
17515090	5,336391	5,068801	5,714972	5,270851	5,547426	5,118147	5,373388	5,312141333	-0,06124667	0,958435555	Icam5
17301689	5,823384	5,873153	5,742008	5,919437	5,383896	5,951479	5,8128483	5,751604	-0,06124433	0,958437105	4930480K23Rik
17438327	4,614239	3,815353	4,121196	4,308971	4,004701	4,053437	4,183596	4,122369667	-0,06122633	0,958449063	4930432L08Rik
17299864	7,173591	7,090035	7,390329	7,267504	7,027688	7,175089	7,217985	7,156760333	-0,06122467	0,95845017	---
17299941	7,173591	7,090035	7,390329	7,267504	7,027688	7,175089	7,217985	7,156760333	-0,06122467	0,95845017	---
17488312	6,285835	6,259892	6,526415	6,33253	6,395163	6,160777	6,3573807	6,296156667	-0,061224	0,958450613	Map3k10
17412879	6,262108	5,850592	6,194672	6,048454	6,185277	5,890001	6,1024573	6,041244	-0,06121333	0,9584577	---
17298942	3,620261	3,949107	4,187973	4,072646	3,868361	3,632698	3,9191137	3,857901667	-0,061212	0,958458585	---
17322697	5,362242	5,331265	5,081099	5,245386	5,267268	5,078325	5,258202	5,196993	-0,061209	0,958460578	Gm15983
17387940	3,9756	3,502226	4,294879	3,834292	4,03264	3,722158	3,924235	3,86303	-0,061205	0,958463236	Olfr1238
17254019	4,633193	4,702702	4,732487	4,704086	4,607299	4,57339	4,6894607	4,628258333	-0,06120233	0,958465007	---
17456969	5,096487	5,008501	5,320893	5,073374	4,986435	5,182485	5,1419603	5,080764667	-0,06119567	0,958469436	---
17329692	8,928022	8,974882	8,761687	8,719641	9,003691	8,757689	8,888197	8,827007	-0,06119	0,958473201	Lsg1
17403025	8,576604	8,764486	8,471205	8,617641	8,571998	8,439112	8,6040983	8,542917	-0,06118133	0,958478959	Lamtor3
17236739	7,188306	7,182916	6,985661	6,88427	7,089831	7,199347	7,118961	7,057816	-0,061145	0,958503098	Ube2n
17391883	6,228633	6,320363	6,378205	6,109673	6,460611	6,173532	6,309067	6,247938667	-0,06112833	0,958514171	Cenpb
17490218	5,511314	5,568594	6,064689	5,468049	5,74443	5,748783	5,7148657	5,653754	-0,06111167	0,958525244	1700008O03Rik
17477508	7,341592	7,165639	7,366719	7,097251	7,399529	7,193845	7,2913167	7,230208333	-0,06110833	0,958527459	Napsa
17548486	6,299098	6,567606	6,710829	6,484682	6,463087	6,446452	6,5258443	6,464740333	-0,061104	0,958530338	---
17279948	3,035664	2,999268	3,283921	3,165009	2,999268	2,97134	3,1062843	3,045205667	-0,06107867	0,95854717	---
17423047	4,386347	4,22283	4,187074	4,126017	4,273826	4,213192	4,265417	4,204345	-0,061072	0,958551599	---
17510563	8,417194	8,36603	8,381309	8,307773	8,365964	8,307583	8,3881777	8,327106667	-0,061071	0,958552264	Slc35e1
17420139	5,996863	5,75837	5,77137	5,819256	5,770664	5,753477	5,842201	5,781132333	-0,06106867	0,958553814	Lactbl1
17290865	7,190848	7,28692	7,292378	7,192823	7,152895	7,241254	7,2567153	7,195657333	-0,061058	0,958560901	Epdr1
17486308	6,201151	6,398435	6,026968	6,258782	6,010805	6,173804	6,2088513	6,147797	-0,06105433	0,958563337	Zfp772 // Zfp772 // Zfp772 // Zfp772
17486384	6,201151	6,398435	6,026968	6,258782	6,010805	6,173804	6,2088513	6,147797	-0,06105433	0,958563337	Zfp772 // Zfp772 // Zfp772 // Zfp772
17259401	7,544432	7,633639	7,472096	7,64185	7,39434	7,430854	7,5500557	7,489014667	-0,061041	0,958572196	Gcgr
17445246	5,57881	5,641007	5,50359	5,514405	5,488883	5,536999	5,574469	5,513429	-0,06104	0,958572861	1700028E10Rik
17547793	3,392328	3,399884	3,36039	3,245112	3,453525	3,270892	3,3842007	3,323176333	-0,06102433	0,95858327	Fam107a // Fam107a
17202199	5,081486	5,132761	4,968842	4,846031	4,86748	5,286518	5,0610297	5,000009667	-0,06102	0,95858615	---
17539574	4,729108	4,553615	4,812771	4,63592	4,610735	4,665819	4,698498	4,637491333	-0,06100667	0,958595009	Asb9

17260438	5,337248	5,226922	5,728715	5,24444	5,486484	5,378943	5,4309617	5,369955667	-0,061006	0,958595452	---
17476604	3,579136	3,21849	3,153833	3,206383	3,518507	3,043571	3,317153	3,256153667	-0,06099933	0,958599881	Abpe
17547905	4,988692	4,959073	4,697111	4,929416	4,607517	4,924979	4,8816253	4,820637333	-0,060988	0,958607412	Gm20555
17251634	4,205155	4,306497	4,650298	4,299424	4,504501	4,175115	4,3873167	4,326346667	-0,06097	0,958619372	A030009H04Rik
17549268	3,8103	3,838196	4,087435	4,054986	3,801892	3,696184	3,911977	3,851020667	-0,06095633	0,958628453	---
17305727	8,920043	9,124309	8,968915	8,89797	8,928334	9,004094	9,0044223	8,943466	-0,06095633	0,958628453	Gmfb
17240945	11,3779	11,32974	11,29533	11,22261	11,32854	11,26897	11,334323	11,27337333	-0,06095	0,958632662	Serinc1
17305945	3,674593	3,271707	3,013421	3,270892	3,23991	3,266078	3,319907	3,25896	-0,060947	0,958634655	Olfr731
17290056	10,03908	10,00785	10,0268	10,09883	9,918004	9,874097	10,024577	9,963643667	-0,060933	0,958643958	Mocs2
17238494	8,939584	8,85237	8,992091	8,8737	8,865285	8,862267	8,928015	8,867084	-0,060931	0,958645287	Smarcc2
17459797	3,788486	3,376826	3,520549	3,479535	3,576991	3,446576	3,5619537	3,501034	-0,06091967	0,958652818	---
17381791	4,470639	4,502906	4,517597	4,49992	4,435254	4,373265	4,4970473	4,436146333	-0,060901	0,958665221	Cubn
17500532	7,940001	7,636314	8,115526	7,767263	7,816056	7,925831	7,8972803	7,836383333	-0,060897	0,958667879	Gm4889
17206111	3,659459	3,777473	3,716684	3,681447	3,822936	3,466558	3,717872	3,656980333	-0,06089167	0,958671423	---
17408184	7,011048	7,230575	7,123487	7,130185	7,10904	6,943226	7,1217033	7,060817	-0,06088633	0,958674967	Chd1l
17307673	4,621245	4,696219	4,784919	4,841135	4,54329	4,535319	4,7007943	4,639914667	-0,06087967	0,958679397	Prss55
17467269	6,613929	6,68381	6,496587	6,48554	6,494259	6,63195	6,5981087	6,537249667	-0,060859	0,958693131	Hpgds
17431861	6,009468	5,931882	6,261147	6,027678	5,918395	6,073861	6,067499	6,006644667	-0,06085433	0,958696232	Nbl1
17289790	3,379353	3,282105	3,13068	3,138445	3,237998	3,233192	3,264046	3,203211667	-0,06083433	0,958709522	Mir582
17522761	8,634706	8,660082	8,554255	8,595574	8,511732	8,559284	8,6163477	8,55553	-0,06081767	0,958720598	Dync1li1
17380868	4,465847	4,432244	4,48266	4,442254	4,432244	4,323821	4,4602503	4,399439667	-0,06081067	0,958725249	---
17285875	3,423895	3,376842	3,52525	3,474082	3,26594	3,403572	3,4419957	3,381198	-0,06079767	0,958733888	Hist1h4b
17291374	7,735221	7,937047	7,601667	7,725614	7,711009	7,654934	7,7579783	7,697185667	-0,06079267	0,958737211	Mrs2
17421997	3,12127	3,015774	3,345809	3,132767	3,112182	3,055548	3,160951	3,100165667	-0,06078533	0,958742084	---
17209813	5,959779	4,830215	6,043702	5,541157	5,633614	5,476642	5,611232	5,550471	-0,060761	0,958758255	---
17520602	10,00564	10,48164	10,3748	10,24755	10,08872	10,34359	10,28736	10,22662	-0,06074	0,958772211	---
17244215	2,993462	2,879577	3,006697	2,967719	2,837201	2,892614	2,959912	2,899178	-0,060734	0,958776199	Mir135a-2
17500432	4,188994	4,426118	4,336309	4,27172	4,126562	4,370967	4,3171403	4,256416333	-0,060724	0,958782844	---
17241849	6,722411	6,769551	6,813865	6,684415	6,714199	6,725042	6,768609	6,707885333	-0,06072367	0,958783066	Susd2
17543281	5,211735	5,134394	5,469971	5,315587	5,124372	5,194018	5,2720333	5,211325667	-0,06070767	0,958793699	---
17221236	10,56155	10,32345	10,67473	10,4766	10,49213	10,40888	10,51991	10,45920333	-0,06070667	0,958794364	---
17342238	4,747737	4,489729	4,610126	4,614727	4,556459	4,494294	4,615864	4,55516	-0,060704	0,958796136	Tpsab1
17227583	4,117974	4,408511	4,0252	4,399092	3,901237	4,069294	4,183895	4,123207667	-0,06068733	0,958807213	Gm5833 // Gm5833
17318123	4,826432	5,091458	4,927823	4,902969	4,877936	4,882784	4,948571	4,887896333	-0,06067467	0,958815631	Ly6h
17510793	8,664155	8,793536	8,614377	8,509395	8,644797	8,735888	8,6906893	8,630026667	-0,06066267	0,958823606	Smarca5
17423631	4,526777	4,578225	4,998772	4,886912	4,605342	4,42954	4,701258	4,640598	-0,06066	0,958825378	Pou3f2
17208439	6,535457	6,338883	6,614466	6,752103	6,580383	5,974343	6,4962687	6,435609667	-0,060659	0,958826043	---
17330901	4,722481	4,479416	4,512828	4,716065	4,596992	4,21971	4,571575	4,510922333	-0,06065267	0,958830252	Ccdc54
17215035	9,814479	9,866527	9,862766	9,765096	9,762685	9,834034	9,847924	9,787271667	-0,06065233	0,958830474	Psmd1
17506095	4,14892	3,904163	4,245462	4,221738	4,054321	3,840533	4,099515	4,038864	-0,060651	0,95883136	Necab2
17514143	5,217616	5,071307	5,474061	5,230829	5,104594	5,24563	5,254328	5,193684333	-0,06064367	0,958836234	---
17467921	3,576264	3,41082	3,34888	3,466225	3,275987	3,411823	3,4453213	3,384678333	-0,060643	0,958836677	---
17475221	8,073366	7,382452	7,552196	7,310053	7,488015	8,028027	7,669338	7,608698333	-0,06063967	0,958838892	---
17256697	4,835558	4,97395	4,951594	4,940719	4,711752	4,926814	4,9203673	4,859761667	-0,06060567	0,958861489	Aoc3
17538753	7,096506	7,366513	7,429241	6,893061	7,731467	7,085925	7,29742	7,236817667	-0,06060233	0,958863705	---
17350591	5,35101	5,298956	5,347754	5,176997	5,33997	5,298956	5,3325733	5,271974333	-0,060599	0,95886592	Sncaip
17216745	6,318724	5,969955	6,129795	5,983276	6,152606	6,10082	6,1394913	6,078900667	-0,06059067	0,958871459	Gpr39
17550857	3,780408	3,348424	3,895235	3,573546	3,718038	3,550776	3,674689	3,61412	-0,060569	0,958885859	---
17316153	7,647247	7,572882	7,819044	7,53674	7,854334	7,466424	7,6797243	7,619166	-0,06055833	0,958892949	Acot10
17346401	4,294381	4,504293	4,457685	4,364623	4,36343	4,346675	4,4187863	4,358242667	-0,06054367	0,958902697	Acer1
17262755	7,189531	7,153915	7,146818	7,337106	6,925717	7,045895	7,1634213	7,102906	-0,06051533	0,95892153	Slc22a5
17239889	7,307241	7,36758	7,24253	7,241366	7,222543	7,271976	7,3057837	7,245295	-0,06048867	0,958939254	L3mbtl3
17234474	3,214265	3,177356	3,357584	3,235556	3,208822	3,12337	3,249735	3,189249333	-0,06048567	0,958941248	---
17267745	9,054213	8,970394	8,854385	8,853757	8,853861	8,990029	8,959664	8,899215667	-0,06044833	0,958966064	Tom1l1
17212061	4,807707	4,710377	4,701762	4,770808	4,72447	4,543275	4,7399487	4,679517667	-0,060431	0,958977585	---
17299575	10,04939	10,25356	9,558598	9,96198	9,753748	9,964636	9,9538493	9,893454667	-0,06039467	0,959001737	Rnase4
17201553	3,008015	3,219107	3,30235	3,146058	3,217799	2,984531	3,1764907	3,116129333	-0,06036133	0,959023895	---
17544271	3,947641	3,880524	3,766909	3,828146	3,699459	3,886403	3,8650247	3,804669333	-0,06035533	0,959027883	---
17549670	5,168156	5,518079	5,458238	5,536167	5,192667	5,234637	5,381491	5,321157	-0,060334	0,959042065	---
17223429	3,8753	4,002666	3,87959	3,79057	3,955553	3,830465	3,9191853	3,858862667	-0,06032267	0,959049599	Als2cr12
17206635	4,305396	4,220537	4,548264	4,134677	4,298957	4,459605	4,3580657	4,297746333	-0,06031933	0,959051815	---
17437570	5,926363	5,230763	5,725997	5,631509	5,407145	5,663536	5,6277077	5,567396667	-0,060311	0,959057354	G6pd2
17203921	5,974833	4,666171	5,250617	5,330671	5,069546	5,310473	5,297207	5,236896667	-0,06031033	0,959057797	---
17293013	3,997271	3,740957	3,760431	3,840805	3,883129	3,593795	3,8328863	3,772576333	-0,06031	0,959058019	Il9
17467840	4,557948	4,63507	4,635996	4,456763	4,558585	4,632737	4,6096713	4,549361667	-0,06030967	0,959058241	Dnahc6
17499376	7,065139	7,125589	7,18877	7,011929	7,185494	7,001173	7,1264993	7,066198667	-0,06030067	0,959064224	Upf3a
17202693	3,783284	4,551501	3,631531	5,155624	3,511407	3,118386	3,988772	3,928472333	-0,06029967	0,959064888	---
17216676	4,791675	4,984406	5,056818	4,874146	4,907366	4,870495	4,9442997	4,884002333	-0,06029733	0,959066439	En1
17239136	5,303683	5,064063	5,364666	5,312265	5,06344	5,175849	5,2441373	5,183851333	-0,060286	0,959073974	---
17437773	8,598642	8,613563	8,556809	8,544162	8,484575	8,559424	8,5896713	8,529387	-0,06028433	0,959075082	Lias
17207035	4,488944	5,204262	5,225732	5,050679	4,944335	4,743086	4,9729793	4,9127	-0,06027933	0,959078405	---
17515058	7,099432	7,524864	6,974691	7,166854	7,132763	7,118534	7,1996623	7,139383667	-0,06027867	0,959078849	Ppan
17254589	3,365517	3,359715	3,322233	3,28152	3,385031	3,200088	3,349155	3,288879667	-0,06027533	0,959081065	---
17526850	6,48285	6,491792	6,040929	6,304203	6,103944	6,426636	6,3385237	6,278261	-0,06026267	0,959089485	AU019823
17204681	6,494455	6,673396	6,820866	6,579928	6,980446	6,247564	6,6629057	6,602646	-0,06025967	0,95909148	---
17347873	4,983281	4,870992	5,225827	4,739069	5,115294	5,044969	5,0267	4,966444	-0,060256	0,959093917	2010106C02Rik
17234612	6,181846	6,106441	6,110569	6,292513	5,802648	6,122936	6,132952	6,072699	-0,060253	0,959095912	Slc19a1
17549536	6,331679	6,221151	5,961046	6,219438	6,169702	5,944077	6,171292	6,111072333	-0,06021967	0,959118072	---
17549700	6,331679	6,221151	5,961046	6,219438	6,169702	5,944077	6,171292	6,111072333	-0,06021967	0,959118072	---
17206029	7,205654	7,271841	7,822532	7,501699	7,341815	7,275895	7,4333423	7,373136333	-0,060206	0,959127157	---
17257310	7,943621	8,108206	8,178932	7,895906	8,012306	8,141943	8,0769197	8,016718333	-0,06020133	0,95913026	Mettl2
17233078	9,772383	9,603682	9,545717	9,543721	9,631796	9,565666	9,640594	9,580394333	-0,06019967	0,959131368	Prep
17365778	8,871667	8,99826	8,880063	8,911452	8,793645	8,864309	8,9166633	8,856468667	-0,06019467	0,959134692	Zdhhc6
17287760	9,27873	9,23509	9,331913	9,244815	9,244012	9,176324	9,281911	9,221717	-0,060194	0,959135135	B230219D22Rik
17364078	2,880431	3,415751	2,875561	2,909931	3,03374	3,047503	3,0572477	2,997058	-0,06018967	0,959138016	---
17548123	6,053607	6,394137	5,576318	5,655888	5,954821	6,232791	6,0080207	5,947833333	-0,06018733	0,959139567	Ehd1 // Ehd1
17549382	5,707328	5,440548	5,794971	5,677482	5,466503	5,61833	5,6476157	5,587438333	-0,06017733	0,959146216	---
17549766	5,707328	5,440548	5,794971	5,677482	5,466503	5,61833	5,6476157	5,587438333	-0,06017733	0,959146216	---
17508787	6,147222	6,66956	6,111255	6,303614	6,361327	6,082576	6,3093457	6,249172333	-0,06017333	0,959148875	Lonrf1
17209739	5,309405	5,000091	5,350705	5,146996	5,244724	5,087979	5,220067	5,159899667	-0,06016733	0,959152864	---
17317309	5,134511	5,036155	4,662776	5,000099	4,759237	4,89361	4,9444807	4,884315333	-0,06016533	0,959154194	Ube2d4
17377038	4,177948	4,349173	4,411467	4,027868	4,340034	4,390202	4,3128627	4,252701333	-0,06016133	0,959156853	---
17529367	6,584946	6,456143	6,423302	6,660899	6,399038	6,223978	6,4881303	6,427971667	-0,06015867	0,959158626	Ube2cbp
17337349	6,796757	6,917566	6,767435	6,704915	6,919137	6,677243	6,8272527	6,767098333	-0,06015433	0,959161507	Gnl1
17202125	4,044283	3,350354	3,117302	3,626333	3,352163	3,352985	3,5039797	3,443827	-0,06015267	0,959162615	---
17401414	3,355701	3,314362	3,358314	3,262534	3,208386	3,37709	3,3427923	3,28267	-0,06012233	0,959182782	Gm5547
17222411	4,699536	4,535319	4,612885	4,509061	4,718226	4,440093	4,6159133	4,555793333	-0,06012	0,959184333	Lyg2
17228701	4,019975	3,247244	3,63935	3,668074	3,34753	3,710605	3,635523	3,575403	-0,06012	0,959184333	---
17438759	4,423581	4,433634	4,590054	4,432336	4,456715	4,377859	4,482423	4,422303333	-0,06011967	0,959184555	Ambn
17513006	7,069453	7,28686	7,605064	7,529014	6,74229	7,50973	7,320459	7,260344667	-0,06011433	0,959188101	Mir3473d // Mir3473d
17227401	5,562408	5,425757	5,277054	5,608205	5,291997	5,184676	5,4217397	5,361626	-0,06011367	0,959188544	Igfn1
17548244	6,629769	6,643242	6,688522	6,702091	6,475416	6,603688	6,6538443	6,593731667	-0,06011267	0,959189209	Gm19816
17285559	7,716351	7,703218	7,408135	7,606589	7,567864	7,472947	7,6092347	7,549133333	-0,06010133	0,959196744	Elmo1
17400732	7,048913	7,088053	7,114846	7,052051	6,973973	7,045521	7,0839373	7,023848333	-0,060089	0,959204944	Pdzk1
17288425	4,47816	4,305531	4,3854	4,353	4,299885	4,33597	4,389697	4,329618333	-0,06007867	0,959211814	4930520P13Rik // 4930520P13Rik
17428803	8,823681	8,945229	8,983017	8,788902	8,92817	8,854689	8,917309	8,857253667	-0,06005533	0,959227328	Kif2c
17208215	7,350724	7,104599	7,798984	7,761723	7,005869	7,306563	7,4181023	7,358051667	-0,06005067	0,959230431	---
17385483	8,474055	8,58612	8,51783	8,472709	8,403061	8,522117	8,5260017	8,465962333	-0,06003933	0,959237967	Mrps18b // Mrps18b
17547561	4,176893	4,241745	4,24938	4,053007	4,230716	4,204199	4,2226727	4,162640667	-0,060032	0,959242842	Gm10744
17428116	8,828568	8,831988	8,667733	8,805526	8,642483	8,700197	8,7760963	8,716068667	-0,06002767	0,959245724	Btf3l4
17354132	4,806303	4,557827	4,693132	4,712118	4,485736	4,679335	4,685754	4,625729667	-0,06002433	0,95924794	Jakmip2
17508766	8,819069	9,029676	8,787565	8,61593	8,887423	8,952888	8,87877	8,818747	-0,060023	0,959248826	Eri1
17205735	5,902221	5,255586	6,229151	5,632461	6,057333	5,517185	5,7956527	5,735659667	-0,059993	0,959268774	---
17300151	5,03976	4,856674	5,13847	5,062331	4,660626	5,131981	5,0116347	4,951646	-0,05998867	0,959271655	---
17371460	7,008256	7,206712	7,19227	7,02048	7,032558	7,174307	7,135746	7,075781667	-0,05996433	0,959287835	Ppig
17353131	7,293687	7,434027	7,36769	7,163695	7,36539	7,386433	7,3651347	7,305172667	-0,059962	0,959289386	Ino80c
17424952	6,600697	6,54542	6,012039	6,269939	6,335266	6,373083	6,386052	6,326096	-0,059956	0,959293376	Shb
17327491	6,928218	6,739963	6,875032	6,797862	6,613893	6,951597	6,8477377	6,787784	-0,05995367	0,959294927	Wrb
17217566	7,276267	7,581746	7,647298	7,251873	7,589389	7,484243	7,5017703	7,441835	-0,05993533	0,959307118	Ptpn7
17206867	6,931682	6,99193	6,935072	6,559625	7,062689	7,056573	6,9528947	6,892962333	-0,05993233	0,959309113	---
17495812	4,442019	4,694199	4,502243	4,33145	4,527763	4,599499	4,5461537	4,486237333	-0,05991633	0,959319752	9030407P20Rik
17548987	8,403605	8,534822	8,563901	8,635494	8,162622	8,524469	8,500776	8,440861667	-0,05991433	0,959321082	2810025M15Rik // 2810025M15Rik
17547666	5,022217	4,966687	5,073731	4,803261	5,075397	5,004238	5,0208783	4,960965333	-0,059913	0,959321968	Synj2bp // Synj2bp
17524022	4,939366	4,824738	4,83193	4,693431	4,950302	4,772591	4,8653447	4,805441333	-0,05990333	0,959328396	Kdm4d
17481606	5,193015	5,640791	5,0932	5,04702	5,346598	5,353687	5,309002	5,249101667	-0,05990033	0,959330391	Olfr481
17252282	5,169522	4,967333	4,963843	5,089472	4,947724	4,883816	5,033566	4,973670667	-0,05989533	0,959333716	9230020A06Rik
17251091	4,575487	3,877699	4,122266	4,16713	4,083563	4,145087	4,1918173	4,131926667	-0,05989067	0,959336819	Myh4
17291070	2,846766	2,888288	2,890938	2,916554	2,859246	2,670567	2,8753307	2,815455667	-0,059875	0,959347237	Vmn1r192
17200283	6,610086	6,603741	6,999856	6,961861	6,665769	6,406447	6,7378943	6,678025667	-0,05986867	0,959351448	---
17360532	4,862053	4,720337	4,730398	4,728275	4,662341	4,742582	4,7709293	4,711066	-0,05986333	0,959354995	Ppnr
17467799	9,504071	9,528714	9,321481	9,358419	9,472128	9,344136	9,451422	9,391561	-0,059861	0,959356546	Tgoln1
17492474	4,623921	4,102623	4,589643	4,454148	4,443478	4,23898	4,438729	4,378868667	-0,05986033	0,95935699	LOC100859962
17204565	4,972454	4,821065	5,255853	4,887525	5,014805	4,967523	5,0164573	4,956617667	-0,05983967	0,959370733	---
17437790	10,17709	10,2257	9,986734	10,00449	10,07015	10,13539	10,129841	10,07001	-0,05983133	0,959376274	Ube2k

17350869	10,00385	9,935898	9,967327	9,888481	9,90917	9,929955	9,969025	9,909202	-0,059823	0,959381816	Isoc1
17205493	3,110754	3,621179	3,719474	3,593455	3,258419	3,42007	3,4838023	3,423981333	-0,059821	0,959383146	---
17269167	3,635662	3,068658	3,170304	3,541903	3,052404	3,100886	3,2915413	3,231731	-0,05981033	0,959390239	Krtap4-2
17452709	5,558129	5,569256	5,825123	5,546298	5,488317	5,738469	5,650836	5,591028	-0,059808	0,959391791	Gpr81
17414529	3,631214	3,575187	3,54296	3,640862	3,544081	3,38502	3,5831203	3,523321	-0,05979933	0,959397554	---
17333891	5,535344	5,457184	5,709298	5,730488	5,254192	5,53777	5,5672753	5,507483333	-0,059792	0,959402431	Gm7736
17487884	8,231395	8,340862	8,272853	8,17221	8,169497	8,324029	8,2817033	8,221912	-0,05979133	0,959402874	Gsk3a
17419880	6,037012	6,431498	5,7582	5,954801	5,952087	6,140455	6,07557	6,015781	-0,059789	0,959404426	Il28ra
17354290	7,877037	7,978379	7,999079	7,815615	7,968225	7,891331	7,9514983	7,891723667	-0,05977467	0,959413958	Atg12
17548104	6,22498	6,232244	6,244391	6,051695	6,195802	6,274848	6,2338717	6,174115	-0,05975667	0,959425928	Hps1 // Hps1
17423713	10,04407	9,983738	9,942791	9,861278	9,927875	10,00219	9,9901997	9,930447667	-0,059752	0,959429031	Manea
17341378	8,377305	8,556589	8,314493	8,276306	8,418457	8,37441	8,416129	8,356391	-0,059738	0,959438342	Zfp942
17467575	8,563107	8,548875	8,589908	8,444487	8,540609	8,537592	8,5672967	8,507562667	-0,059734	0,959441002	Thnsl2
17267996	6,144834	6,009763	6,325939	6,068124	6,150511	6,082749	6,1601787	6,100461333	-0,05971733	0,959452086	Rsad1
17440768	6,252073	6,471289	6,270978	6,42063	6,121104	6,273492	6,3314467	6,271742	-0,05970467	0,95946051	Selplg // Selplg
17340401	3,388205	3,391877	3,438569	3,305486	3,354524	3,379528	3,406217	3,346512667	-0,05970433	0,959460732	---
17250500	5,198455	5,471751	5,461708	5,485654	5,165635	5,301569	5,3773047	5,317619333	-0,05968533	0,959473368	Smcr7
17459126	4,086833	4,143066	4,005624	4,050062	3,900853	4,105576	4,0785077	4,018830333	-0,05967733	0,959478688	Grid2
17355139	6,129178	5,971542	5,719677	5,837227	6,045513	5,758637	5,9401323	5,880459	-0,05967333	0,959481348	Gm19784
17311945	8,743933	8,940074	8,710896	8,711277	8,767248	8,737373	8,798301	8,738632667	-0,05966833	0,959484674	Phf20l1
17315133	6,033486	5,90772	6,184865	5,951745	5,90696	6,088364	6,0420237	5,982356333	-0,05966733	0,959485339	Acvrl1
17228099	3,858328	3,618736	3,937445	3,479591	4,11417	3,641779	3,8048363	3,74518	-0,05965633	0,959492654	---
17549120	5,157488	4,884863	5,252524	5,057095	5,104098	4,954789	5,0982917	5,038660667	-0,059631	0,959509503	---
17549122	5,157488	4,884863	5,252524	5,057095	5,104098	4,954789	5,0982917	5,038660667	-0,059631	0,959509503	---
17305221	10,95199	11,25617	10,79478	11,09865	10,61061	11,11479	11,00098	10,94135	-0,05963	0,959510168	5730469M10Rik
17318426	3,297892	3,230088	3,338579	3,480487	3,00861	3,198601	3,288853	3,229232667	-0,05962033	0,959516597	Mir3079 // Mir3079
17438980	3,857524	3,611028	3,93507	3,74521	3,816995	3,662564	3,8012073	3,741589667	-0,05961767	0,959518371	Cxcl15
17519972	8,312214	8,368467	8,156311	8,297568	8,312873	8,047702	8,2789973	8,219381	-0,05961633	0,959519258	Dopey1
17354074	3,949233	3,638976	3,91822	3,923326	3,592091	3,81217	3,8354763	3,775862333	-0,059614	0,959520809	Gpr151
17515523	10,88233	11,00512	10,78551	10,73053	10,8707	10,89304	10,890987	10,83142333	-0,05956333	0,959554508	Sept7
17415401	3,732356	3,803554	3,572322	3,476754	3,945514	3,507284	3,702744	3,643184	-0,05956	0,959556725	Dmrta1
17410984	5,287105	5,041559	5,684899	5,380469	5,244901	5,20955	5,3378543	5,278306667	-0,05954767	0,959564928	Wdr63
17221993	5,1387	4,87851	4,859681	4,829743	4,903327	4,965234	4,9589637	4,899434667	-0,059529	0,959577344	Gm9839
17422688	4,305548	4,225253	4,295377	4,217489	4,184323	4,24584	4,2753927	4,215884	-0,05950867	0,959590868	Tmem88b // Tmem88b
17492280	7,320281	7,331643	7,221175	6,90929	7,422201	7,363095	7,291033	7,231528667	-0,05950433	0,95959375	Mrpl46
17206703	5,319763	5,950127	5,723084	5,999893	5,614671	5,199934	5,6643247	5,604832667	-0,059492	0,959601954	---
17201397	8,731126	8,087269	8,918937	8,76635	7,962986	8,829527	8,5791107	8,519621	-0,05948967	0,959603506	---
17295233	11,13111	11,02879	11,01192	11,09434	10,76497	11,13406	11,057273	10,99779	-0,05948333	0,959607718	Hmgcr
17294562	4,514907	4,446708	4,59374	4,369109	4,446797	4,561039	4,5184517	4,458981667	-0,05947	0,959616587	Gm4211
17217380	11,96206	12,30575	12,17637	12,00588	12,06898	12,19092	12,14806	12,08859333	-0,05946667	0,959618804	Gm8618
17311145	3,127322	3,181019	3,005308	2,95957	3,017952	3,157775	3,1045497	3,045099	-0,05945067	0,959629447	---
17293309	4,029524	3,863075	4,047968	3,777989	4,019942	3,964336	3,980189	3,920755667	-0,05943333	0,959640976	Gm19866
17540610	4,002492	3,857406	3,992236	3,835681	4,141124	3,697047	3,9507113	3,891284	-0,05942733	0,959644967	---
17223811	6,124907	5,736361	6,169369	6,004487	5,91892	5,928965	6,0102123	5,950790667	-0,05942167	0,959648737	---
17500380	4,967305	5,076531	4,999899	4,993545	4,930273	4,941685	5,0145783	4,955167667	-0,05941067	0,959656054	Dusp26
17480515	4,67722	4,597957	4,758823	4,517524	4,402877	4,935401	4,678	4,618600667	-0,05939933	0,959663592	Mtap6
17467310	4,837413	4,812473	4,978957	4,846625	4,761511	4,842675	4,876281	4,816937	-0,059344	0,9597004	Gng12
17467435	5,411658	5,360312	5,365375	5,673345	4,630495	5,655477	5,379115	5,319772333	-0,05934267	0,959701287	Igkv4-70
17221238	9,216799	9,355032	8,861671	9,055793	9,273847	8,925866	9,1445007	9,085168667	-0,059332	0,959708383	---
17479162	7,867354	8,100947	7,689186	7,787311	7,8895	7,802763	7,885829	7,826524667	-0,05930433	0,959726787	Kansl2 // Kansl2
17365714	11,10794	11,21072	11,09873	11,08931	11,07081	11,07938	11,13913	11,07983333	-0,05929667	0,959731888	Nutf2 // Nutf2
17361558	7,301844	7,490489	7,31359	7,461319	7,272248	7,194512	7,368641	7,309359667	-0,05928133	0,959742088	Sart1
17542965	3,358071	3,359842	3,798086	3,571578	3,354766	3,411831	3,505333	3,446058333	-0,05927467	0,959746523	---
17461157	4,204879	3,981984	3,974903	4,019096	3,938195	4,026665	4,053922	3,994652	-0,05927	0,959749627	---
17273751	8,510896	8,575497	8,452438	8,413498	8,506539	8,440997	8,5129437	8,453678	-0,05926567	0,95975251	Itsn2
17396593	10,16301	10,25765	10,15412	10,04158	10,18468	10,17076	10,191593	10,13234	-0,05925333	0,959760715	Sec62
17419407	8,312532	7,674573	8,227943	7,929435	8,28945	7,818403	8,0716827	8,012429333	-0,05925333	0,959760715	Snora44
17277552	6,244352	6,563332	6,56694	6,10605	6,479112	6,611745	6,458208	6,398969	-0,059239	0,95977025	2310044G17Rik
17529222	3,717828	3,662727	3,800283	3,630837	3,801461	3,570876	3,726946	3,667724667	-0,05922133	0,959782003	Mei4
17329450	3,131364	3,095344	3,231855	3,101652	3,229301	2,949955	3,1528543	3,093636	-0,05921833	0,959783999	---
17216133	5,071932	5,074108	4,837768	4,915952	4,799132	5,091089	4,9946027	4,935391	-0,05921167	0,959788434	Gm6086
17223852	4,22237	4,441869	4,841665	4,436764	4,327282	4,564309	4,501968	4,442785	-0,059183	0,959807506	D630023F18Rik
17383985	5,383239	5,252741	5,407008	5,185535	5,182053	5,497908	5,3476627	5,288498667	-0,059164	0,959820146	Ttc16
17230873	3,664238	3,855898	4,247615	3,832882	4,003251	3,75417	3,9225837	3,863434333	-0,05914933	0,959829904	9330162B11Rik
17450904	3,212507	3,347263	3,070792	3,101084	3,259941	3,092114	3,2101873	3,151046333	-0,059141	0,959835448	Vmn2r14
17284417	3,942789	3,828917	3,513095	3,513741	3,877743	3,715899	3,7616003	3,702461	-0,05913933	0,959836557	---
17238663	2,953856	3,064675	3,270892	3,04803	3,185259	2,878752	3,0964743	3,037347	-0,05912733	0,959844541	Olfr784
17366874	6,07118	5,831348	5,616458	5,875867	5,798221	5,667545	5,839662	5,780544333	-0,05911767	0,959850972	Mir669k
17494714	3,674708	3,430081	3,804238	3,546124	3,678728	3,506842	3,6363423	3,577231333	-0,059111	0,959855408	Olfr710
17366890	3,36395	3,179831	3,051266	3,193144	3,02949	3,195099	3,198349	3,139244333	-0,05910467	0,959859621	---
17476951	5,518648	5,292804	5,521179	5,695694	5,279328	5,180299	5,4442103	5,385107	-0,05910333	0,959860508	Gm5592
17446688	5,892527	5,794143	5,868143	5,671129	5,880766	5,825725	5,8516043	5,79254	-0,05906433	0,959886456	Otof
17545588	4,021301	3,189887	3,107689	3,549896	3,362759	3,229056	3,4396257	3,380570333	-0,05905533	0,959892444	1700042B14Rik // 1700042B14Rik
17540050	9,958501	9,908319	9,994882	10,026	9,842465	9,816095	9,9539007	9,894853333	-0,05904733	0,959897767	Ebp
17419272	6,415971	6,331963	6,595345	6,472004	6,412296	6,281898	6,4477597	6,388732667	-0,059027	0,959911296	---
17299514	3,866013	3,495113	3,476358	3,573096	3,408059	3,679265	3,6124947	3,553473333	-0,05902133	0,959915067	---
17361385	6,423774	6,235319	6,051783	6,175424	6,139304	6,219116	6,2369587	6,177948	-0,05901067	0,959922164	Bbs1
17528350	6,375506	6,333986	6,31582	6,328899	6,119372	6,400018	6,3417707	6,282763	-0,05900767	0,95992416	Fbxl22
17528245	2,991163	2,686863	3,182151	3,080043	2,967721	2,635523	2,9533923	2,894429	-0,05896333	0,959953658	Rnu5g // Rnu5g // Rnu5g // Rnu5g
17398430	9,523517	9,716026	9,342515	9,455227	9,511986	9,438003	9,5273527	9,468405333	-0,05894733	0,959964305	Ctso
17407522	5,915346	5,720922	5,829778	5,601172	5,827453	5,860604	5,8220153	5,763076333	-0,058939	0,95996985	Tdpoz5
17407529	5,915346	5,720922	5,829778	5,601172	5,827453	5,860604	5,8220153	5,763076333	-0,058939	0,95996985	Tdpoz5
17549374	3,88385	4,019254	3,925685	3,987609	3,807312	3,857084	3,9429297	3,884001667	-0,058928	0,959977169	---
17549750	3,88385	4,019254	3,925685	3,987609	3,807312	3,857084	3,9429297	3,884001667	-0,058928	0,959977169	---
17247635	4,146292	4,241611	4,048981	4,069039	4,079058	4,112013	4,145628	4,086703333	-0,05892467	0,959979387	Gm12052
17354891	3,621372	3,291427	3,237462	3,22574	3,437879	3,309906	3,3834203	3,324508333	-0,058912	0,959987816	Spink13
17529842	3,232023	3,536725	3,181987	3,283064	3,444558	3,046381	3,3169117	3,258001	-0,05891067	0,959988703	---
17474322	3,374291	3,338227	3,429832	3,437532	3,309125	3,218984	3,3807833	3,321880333	-0,058903	0,959993804	Ceacam13
17237094	5,483321	5,391541	5,508869	5,388081	5,406235	5,412779	5,4612437	5,402365	-0,05887867	0,960009996	Ppfia2
17235249	6,757051	6,92183	7,008451	6,78658	6,881938	6,842184	6,8957773	6,836900667	-0,05887667	0,960011327	Mum1
17331232	3,256763	3,492426	3,234042	3,265719	3,264828	3,276061	3,3277437	3,268869333	-0,05887433	0,96001288	Olfr180
17266315	5,955923	5,407423	6,120288	5,828541	5,863608	5,614863	5,827878	5,769004	-0,058874	0,960013102	Gm11190
17453541	5,569973	5,675519	5,598485	5,414564	5,671828	5,580965	5,614659	5,555785667	-0,05887333	0,960013545	Fzd9
17497055	11,90069	12,00709	12,03294	11,95645	11,92605	11,88161	11,98024	11,92137	-0,05887	0,960015763	Rpl7a
17378898	4,262631	4,198266	4,388039	4,178105	4,169993	4,324255	4,2829787	4,224117667	-0,058861	0,960021752	Adig
17440569	5,684597	5,577338	5,614117	5,521653	5,60433	5,573524	5,6253507	5,566502333	-0,05884833	0,960030181	Gm15559
17323649	5,367689	5,259966	5,299001	5,173711	5,310105	5,266316	5,3088853	5,250044	-0,05884133	0,960034839	Rtn4r
17404011	6,787305	6,561318	6,633246	6,568697	6,573685	6,663006	6,660623	6,601796	-0,058827	0,960044377	Hey1
17345317	7,776785	7,832342	8,277896	7,981862	7,624537	8,104186	7,962341	7,903528333	-0,05881267	0,960053916	---
17279198	7,539336	7,600666	7,42599	7,446152	7,369061	7,574343	7,5219973	7,463185333	-0,058812	0,960054359	2810002N01Rik
17208541	7,648639	7,373779	7,655984	7,296751	7,909024	7,296208	7,5594673	7,500661	-0,05880633	0,96005813	---
17235757	6,048572	6,077539	5,795797	6,053237	5,745151	5,947102	5,9739693	5,915163333	-0,058806	0,960058352	Tle2
17532623	11,1102	11,42732	11,36594	11,13914	11,28514	11,30278	11,301153	11,24235333	-0,0588	0,960062345	---
17234852	4,225442	3,585483	3,671541	3,814631	3,735894	3,755544	3,8274887	3,768689667	-0,058799	0,96006301	Olfr1353
17391089	6,698861	6,432623	6,610027	6,585015	6,587177	6,39296	6,5805037	6,521717333	-0,05878633	0,960071439	Slc27a2
17293557	11,77885	11,80991	11,85621	11,72749	11,69928	11,84186	11,81499	11,75621	-0,05878	0,960075654	Fbp1
17326005	2,882872	3,452585	3,27995	2,994037	3,078618	3,366416	3,2051357	3,146357	-0,05877867	0,960076541	---
17390806	5,587632	5,053581	5,703966	5,630551	5,09967	5,438665	5,448393	5,389628667	-0,05876433	0,96008608	---
17514328	11,94556	12,02637	11,52491	11,92531	11,69313	11,70214	11,83228	11,77352667	-0,05875333	0,9600934	---
17362904	4,598458	4,561949	4,6655	4,534262	4,530263	4,585147	4,6086357	4,549890667	-0,058745	0,960098946	Ms4a13
17303008	6,004749	5,835012	5,59246	5,753106	5,642229	5,860651	5,8107403	5,751995333	-0,058745	0,960098946	---
17429454	7,768455	8,040983	7,854557	7,804796	7,875754	7,80724	7,8879983	7,829263333	-0,058735	0,960105601	Ctps
17422629	7,908547	7,899899	7,766321	7,799014	7,81305	7,786512	7,8582557	7,799525333	-0,05873033	0,960108707	Cdk11b
17201313	3,185851	2,67388	3,046314	2,870655	3,104269	2,754935	2,9686817	2,909953	-0,05872867	0,960109816	---
17429596	3,9286	3,827738	3,710833	3,956447	3,958916	3,375669	3,8223903	3,763677333	-0,058713	0,960120242	---
17399843	3,97089	3,904503	3,664485	3,772811	3,796994	3,793938	3,846626	3,787914333	-0,05871167	0,960121129	4930529C04Rik
17302056	4,254417	4,357163	4,557713	4,377244	4,330541	4,285439	4,3897643	4,331074667	-0,05868967	0,96013577	Kctd4
17549842	5,561624	5,439705	5,629843	5,394242	5,567742	5,493122	5,543724	5,485035333	-0,05868867	0,960136436	---
17216158	7,383121	7,166407	7,436516	7,240774	7,30044	7,268768	7,3286813	7,269994	-0,05868733	0,960137323	Fam174a
17413787	3,72464	4,077647	3,943583	3,808457	3,850885	3,910477	3,91529	3,856606333	-0,05868367	0,960139764	---
17279154	12,40456	12,28791	12,40983	12,27469	12,38916	12,26244	12,367433	12,30876333	-0,05867	0,960148859	---
17506418	8,152246	8,2954	8,325279	8,108001	8,294293	8,194622	8,2576417	8,198972	-0,05866967	0,960149081	Cdt1
17292543	4,28293	4,864492	5,007211	4,378499	4,850026	4,750126	4,718211	4,659550333	-0,05866067	0,960155071	Hist1h2al
17399887	3,058259	2,888958	3,142906	2,989384	2,970952	2,953818	3,030041	2,971384667	-0,05865633	0,960157955	Sprr2i
17361463	9,684667	9,699001	9,715915	9,697821	9,59938	9,626462	9,699861	9,641221	-0,05864	0,960168825	Rab1b
17515952	7,612526	7,752915	7,67269	7,530171	7,744836	7,587214	7,679377	7,620740333	-0,05863667	0,960171043	Pus3
17315635	8,140877	8,116485	8,086184	8,040139	7,970414	8,157104	8,1145153	8,055885667	-0,05862967	0,960175702	Ghr
17404993	4,479184	4,593665	4,854941	4,432323	4,808527	4,511091	4,6425967	4,583980333	-0,05861633	0,960184576	Gm10731
17322039	4,58774	5,159222	4,542834	4,64052	4,829001	4,644438	4,7632653	4,704653	-0,05861233	0,960187238	Krt1
17547761	3,202106	3,189411	3,42345	3,265347	3,208435	3,165401	3,2716557	3,213061	-0,05859467	0,960198996	Gm20311
17240269	5,946902	5,948745	6,085862	5,93633	5,940749	5,928656	5,9938363	5,935245	-0,05859133	0,960201215	E130307A14Rik
17502908	3,565955	3,583389	3,88819	3,553819	3,670299	3,637675	3,679178	3,620597667	-0,05858033	0,960208536	4933434I20Rik
17296565	5,587367	5,949385	5,563685	5,832881	5,881309	5,210568	5,7001457	5,641586	-0,05855967	0,960222291	Gm2888
17427725	3,018262	3,486027	3,336092	3,103693	3,334635	3,226411	3,280127	3,221579667	-0,05854733	0,9602305	---

17500535	5,976296	5,608593	5,966426	5,759759	5,848292	5,767644	5,8504383	5,791898333	-0,05854	0,960235381	Dusp4
17438597	4,19131	4,042069	4,157677	3,988328	3,935038	4,292105	4,130352	4,071823667	-0,05852833	0,960243146	---
17298906	3,246068	3,143568	3,075983	3,091095	3,109554	3,089406	3,1552063	3,096685	-0,05852133	0,960247805	---
17343070	5,549296	5,677997	5,801336	5,573713	5,653401	5,625983	5,6762097	5,617699	-0,05851067	0,960254905	0610038L08Rik
17405458	6,093547	6,076426	5,865384	6,035864	5,667441	6,156526	6,0117857	5,953277	-0,05850867	0,960256236	Gpr171
17207613	5,399765	5,976947	5,303428	5,099929	6,028733	5,375963	5,5600467	5,501541667	-0,058505	0,960258677	---
17220051	6,600282	6,618318	6,584906	6,512924	6,50851	6,606561	6,6011687	6,542665	-0,05850367	0,960259564	---
17263423	7,639359	7,818928	7,785947	7,669273	7,820756	7,578738	7,748078	7,689589	-0,058489	0,960269326	Zkscan17
17257063	10,37381	10,26482	10,43186	10,31431	10,19694	10,3838	10,35683	10,29835	-0,05848	0,960275317	---
17323705	3,663792	3,464375	3,521414	3,4814	3,486006	3,506737	3,5498603	3,491381	-0,05847933	0,960275761	---
17436999	5,759993	5,955643	6,265855	5,970359	5,897234	5,93847	5,9938303	5,935354333	-0,058476	0,960277979	Crmp1
17468157	7,580173	7,532831	7,652551	7,571682	7,560237	7,458242	7,5885183	7,530053667	-0,05846467	0,960285523	Tet3
17206395	4,238546	4,224026	4,15993	4,251625	3,981912	4,213602	4,2075007	4,149046333	-0,05845433	0,960292401	---
17273191	5,441754	5,952319	5,486276	5,422966	5,747386	5,534658	5,626783	5,568336667	-0,05844633	0,960297726	Alyref
17436507	9,065877	9,09645	9,049335	8,955579	9,028638	9,052113	9,070554	9,01211	-0,058444	0,960299279	Whsc1
17499949	5,450287	5,453667	5,413289	5,399961	5,404559	5,337435	5,439081	5,380651667	-0,05842933	0,960309042	Dkk4
17446828	5,741045	5,447674	5,555646	5,659605	5,559138	5,350344	5,581455	5,523029	-0,058426	0,960311261	Trim54
17215561	5,10534	4,653196	5,147349	4,986446	4,69482	5,049364	4,9686283	4,91021	-0,05841833	0,960316364	1700067G17Rik // 1700067G17Rik
17270783	8,891235	9,115868	8,999195	8,903733	9,10309	8,824232	9,0020993	8,943685	-0,05841433	0,960319027	Ftsj3
17464429	7,532063	7,729136	7,625765	7,456243	7,682165	7,573369	7,628988	7,570592333	-0,05839567	0,960331452	Ccdc91
17265853	9,433122	9,464825	9,393971	9,300429	9,340967	9,475361	9,4306393	9,372252333	-0,058387	0,960337221	Pafah1b1
17346001	7,180883	7,203957	7,00708	6,879978	7,244029	7,092791	7,13064	7,072266	-0,058374	0,960345874	---
17224546	4,983169	5,005434	4,987954	5,094445	4,831038	4,875973	4,9921857	4,933818667	-0,058367	0,960350534	A630095N17Rik
17201111	5,088993	5,901616	5,775197	5,367387	5,620472	5,602858	5,588602	5,530239	-0,058363	0,960353197	---
17245624	10,28731	10,33228	10,38397	10,18629	10,30374	10,33847	10,33452	10,27616667	-0,05835333	0,960359632	Usp15
17438995	5,889053	6,037611	6,029682	6,079282	5,958392	5,743621	5,9854487	5,927098333	-0,05835033	0,960361629	Cxcl2
17205335	3,808454	4,750706	4,348273	4,355019	4,35352	4,023875	4,3024777	4,244138	-0,05833967	0,960368729	---
17499780	4,98529	5,043895	4,992468	4,911412	4,92157	5,013667	5,0072177	4,948883	-0,05833467	0,960372057	Gm14850
17383610	5,789909	5,365144	5,908165	5,470461	5,710039	5,707754	5,6877393	5,629418	-0,05832133	0,960380933	1700084E18Rik
17333292	4,873175	4,260577	4,640307	4,642571	4,466159	4,49037	4,591353	4,533033333	-0,05831967	0,960382043	Slc22a2
17270062	6,08545	6,110301	6,060757	5,966008	5,999593	6,115967	6,0855027	6,027189333	-0,05831333	0,960386259	Tmem101
17279157	12,06612	12,09201	12,01112	11,84976	11,86182	12,2828	12,056417	11,99812667	-0,05829	0,960401792	---
17239763	3,106204	2,821052	2,978289	2,939525	2,932385	2,858772	2,968515	2,910227333	-0,05828767	0,960403345	Taar8b
17342750	7,853552	7,91083	7,771586	7,794816	7,922354	7,644064	7,8453227	7,787078	-0,05824467	0,96043197	Nudt3
17240220	6,804367	7,198296	7,107451	7,024334	6,900753	7,010327	7,0367047	6,978471333	-0,05823333	0,960439515	Amd2
17311147	4,865449	4,869882	4,975976	4,901805	4,97649	4,658335	4,903769	4,845543333	-0,05822567	0,960444619	Odf1
17298805	12,63346	12,55721	12,60259	12,61509	12,4896	12,51397	12,597753	12,53955333	-0,0582	0,960461706	Glud1
17423711	5,218048	5,255259	5,334391	5,223464	5,009419	5,400222	5,2692327	5,211035	-0,05819767	0,96046326	---
17273511	8,387013	8,342249	8,335652	8,351233	8,248518	8,290606	8,3549713	8,296785667	-0,05818567	0,960471249	Wdr45l
17366008	5,09345	4,948198	5,344108	5,094591	4,991392	5,125229	5,1285853	5,070404	-0,05818133	0,960474134	Hspa12a
17548110	6,987921	7,305116	7,539401	7,329834	7,055272	7,272816	7,2774793	7,219307333	-0,058172	0,960480347	Ppnr
17491433	3,055632	3,350281	2,991889	3,238595	3,016801	2,967908	3,1326007	3,074434667	-0,058166	0,960484342	---
17217592	5,180008	5,247772	5,0414	5,285237	4,978357	5,031131	5,1563933	5,098241667	-0,05815167	0,960493884	---
17389256	3,325271	3,918431	3,434562	3,516748	3,545448	3,441648	3,5594213	3,501281333	-0,05814	0,960501652	Olfr1275
17342708	7,108777	6,912313	7,027574	7,00606	7,021284	6,846936	7,0162213	6,958093333	-0,058128	0,960509641	Lemd2
17473047	8,522602	8,446814	8,448753	8,347261	8,438944	8,457623	8,472723	8,414609333	-0,05811367	0,960519184	Fam60a
17500867	4,99571	4,846714	5,03431	4,885502	4,708055	5,108836	4,9589113	4,900797667	-0,05811367	0,960519184	Mtnr1a
17548127	8,3706	8,393075	8,161139	8,134285	8,211186	8,405038	8,3082713	8,250169667	-0,05810167	0,960527173	2700078E11Rik
17288231	7,315994	7,29608	7,342374	7,281328	7,209852	7,288992	7,3181493	7,260057333	-0,058092	0,960533609	---
17382820	7,73841	7,702535	7,850396	7,746917	7,645848	7,724312	7,7637803	7,705692333	-0,058088	0,960536272	---
17326563	3,163818	3,139674	3,222799	3,134518	3,110373	3,10714	3,1754303	3,117343667	-0,05808667	0,96053716	---
17356685	6,162903	6,385895	6,328808	6,13541	6,471776	6,096204	6,2925353	6,234463333	-0,058072	0,960546925	Gpha2
17331477	3,212632	3,305043	3,246665	3,481302	3,167902	2,940924	3,25478	3,196709333	-0,05807067	0,960547813	---
17475209	8,886271	8,674858	8,211183	8,939154	8,295486	8,3635	8,5907707	8,532713333	-0,05805733	0,96055669	---
17395852	7,640471	8,068082	7,935012	7,790639	7,728309	7,950454	7,8811883	7,823134	-0,05805433	0,960558688	Arfrp1
17511124	5,064434	4,533195	4,733158	4,558802	4,828799	4,769025	4,776929	4,718875333	-0,05805367	0,960559132	---
17449638	9,228012	9,823715	9,604855	9,416886	9,443962	9,621595	9,552194	9,494147667	-0,05804633	0,960564014	---
17549684	5,281216	5,459827	5,252996	5,294888	5,102217	5,422816	5,3313463	5,273307	-0,05803933	0,960568675	---
17459947	9,132622	9,148144	9,155376	9,100262	9,073753	9,088012	9,1453807	9,087342333	-0,05803833	0,960569341	Mrpl53
17220548	10,12194	10,22873	10,19936	10,08683	10,18445	10,10464	10,183343	10,12530667	-0,05803667	0,96057045	Eprs
17446202	5,611799	5,659008	5,845535	5,578737	5,639121	5,724401	5,7054473	5,647419667	-0,05802767	0,960576443	Tmub1
17437725	4,042219	4,665783	4,766199	4,103604	4,880284	4,316267	4,4914003	4,433385	-0,05801533	0,960584655	---
17217469	7,447303	7,595889	7,530684	7,517335	7,442901	7,439596	7,5246253	7,466610667	-0,05801467	0,960585098	Klhl12
17550584	14,0189	14,01339	14,03021	13,94231	13,9519	13,99429	14,020833	13,96283333	-0,058	0,960594864	---
17357719	5,003617	4,851842	4,895135	4,728031	5,257631	4,590978	4,9168647	4,85888	-0,05798467	0,960605073	Gm97
17349087	3,282079	3,170723	3,182952	3,068583	3,189692	3,203542	3,211918	3,153939	-0,057979	0,960608847	---
17268047	6,300941	6,333045	6,341774	6,272325	6,27548	6,254087	6,3252533	6,267297333	-0,057956	0,960624161	Xylt2
17350559	6,038455	6,169694	6,218434	5,980246	6,133897	6,138577	6,1421943	6,08424	-0,05795433	0,960625271	Ftmt
17489098	11,18284	11,38826	11,30863	11,28474	11,32508	11,09606	11,293243	11,23529333	-0,05795	0,960628156	---
17342109	7,529228	7,448687	7,379694	7,491686	7,265173	7,426901	7,4525363	7,394586667	-0,05794967	0,960628378	Cramp1l
17446123	5,636462	5,571488	5,990611	5,521443	5,592461	5,910811	5,7328537	5,674905	-0,05794867	0,960629044	Kcnh2
17230546	4,574131	4,476088	4,689824	4,625052	4,61323	4,328048	4,5800143	4,52211	-0,05790433	0,960658564	Ccdc121
17204131	7,187975	7,013947	7,138243	7,004995	7,399907	6,761622	7,1133883	7,055508	-0,05788033	0,960674545	---
17214685	8,089931	8,299297	7,978389	8,020737	7,930644	8,242735	8,122539	8,064705333	-0,05783367	0,960705621	Acsl3
17383467	5,248118	5,375779	5,68999	5,513587	5,289918	5,336886	5,4379623	5,380130333	-0,057832	0,960706731	Ntng2
17226676	7,486654	7,649083	7,496501	7,489486	7,517552	7,451706	7,5440793	7,486248	-0,05783133	0,960707174	Pfkfb2
17525746	7,482769	7,290631	7,193543	7,367042	7,199414	7,227026	7,3223143	7,264494	-0,05782033	0,9607145	AW551984
17346004	3,532335	3,583575	3,817178	3,724498	3,456097	3,579142	3,6443627	3,586579	-0,05778367	0,960738917	Gm324
17248078	9,038065	9,017333	8,785796	9,142588	8,560416	8,964886	8,9470647	8,889296667	-0,057768	0,96074935	---
17262830	4,090043	4,308518	4,101724	4,223865	4,031262	4,071861	4,1667617	4,108996	-0,05776567	0,960750904	Acsl6
17246396	4,297268	4,493112	4,387764	4,3323	4,170365	4,502201	4,3927147	4,334955333	-0,05775933	0,960755121	Olfr768
17345124	8,668476	8,830741	8,696463	8,703402	8,601933	8,717075	8,7318933	8,674136667	-0,05775667	0,960756897	Enpp4
17432463	2,975419	3,034817	2,821822	2,987578	2,823347	2,847873	2,9440193	2,886266	-0,05775333	0,960759117	Gm13088
17410506	2,993989	2,854683	2,834993	2,94573	2,822559	2,742168	2,894555	2,836819	-0,057736	0,96077066	---
17545179	3,764073	4,257249	4,279948	3,993791	4,377468	3,756845	4,1004233	4,042701333	-0,057722	0,960779984	---
17299595	5,427012	5,229525	5,342907	5,306251	5,392394	5,127701	5,333148	5,275448667	-0,05769933	0,960795079	Gm7247
17419026	6,384783	5,938742	6,129892	5,893062	6,193632	6,193632	6,151139	6,093442	-0,057697	0,960796633	---
17205509	5,299521	5,602179	5,870599	5,472095	5,511289	5,615828	5,5907663	5,533070667	-0,05769567	0,960797521	---
17341883	8,874762	8,904457	8,956845	8,846939	8,779665	8,93642	8,9120213	8,854341333	-0,05768	0,960807954	Traf7
17214023	8,346609	8,613293	8,254343	8,329094	8,316166	8,395984	8,4047483	8,347081333	-0,057667	0,960816612	Gm5528
17237581	2,99902	2,743661	3,012588	2,903911	2,764828	2,913622	2,918423	2,860787	-0,057636	0,960837258	Il22
17548836	7,553813	7,46335	7,526692	7,314777	7,65059	7,405623	7,5146183	7,456996667	-0,05762167	0,960846804	1810037I17Rik // 1810037I17Rik
17389456	3,603476	3,74077	3,622074	3,565813	3,536819	3,690837	3,65544	3,597823	-0,057617	0,960849912	---
17424279	5,778749	5,900847	5,680511	5,680556	5,745151	5,761563	5,7867023	5,72909	-0,05761233	0,96085302	Cntfr
17225627	5,005619	4,507642	4,741055	4,738896	4,688112	4,654534	4,7514387	4,693847333	-0,05759133	0,960867007	9430060I03Rik
17247389	9,949282	10,20178	10,12197	10,08317	10,19642	9,820675	10,091011	10,03342167	-0,057589	0,960868561	Egfr
17543438	4,376102	4,221963	4,397955	4,350766	4,235634	4,236857	4,3320067	4,274419	-0,05758767	0,960869449	Gm14812
17389138	4,158359	3,479846	3,707979	3,648296	3,60253	3,922632	3,7820613	3,724486	-0,05757533	0,960877663	1700024P03Rik
17395933	8,203841	8,089342	8,01011	7,926376	8,180272	8,023928	8,1010977	8,043525333	-0,05757233	0,960879661	Rgs19
17338879	4,592863	4,592321	4,58189	4,581734	4,66296	4,34967	4,5890247	4,531454667	-0,05757	0,960881215	Fut4-ps1
17516740	4,682383	4,963744	4,900229	4,726515	4,897766	4,749419	4,8487853	4,791233333	-0,057552	0,960893204	Scn4b
17313581	8,80512	8,770459	8,934562	8,904135	8,939901	8,493482	8,8367137	8,779172667	-0,057541	0,96090053	---
17550034	8,80512	8,770459	8,934562	8,904135	8,939901	8,493482	8,8367137	8,779172667	-0,057541	0,96090053	---
17310970	2,768031	2,994354	2,855759	2,889313	2,805425	2,750842	2,8727147	2,815193333	-0,05752133	0,960913629	Pop1
17231690	8,190034	8,174672	8,317149	8,206118	8,217041	8,086177	8,227285	8,169778667	-0,05750633	0,96092362	Sf3b5
17364165	2,844436	2,984457	3,152876	3,007898	2,973156	2,828216	2,993923	2,936423333	-0,05749967	0,96092806	---
17362527	5,51137	4,873033	5,500349	5,176098	5,553525	4,982644	5,2949173	5,237422333	-0,057495	0,960931169	Rom1
17286548	4,53521	4,76189	4,800698	4,612536	4,700522	4,612342	4,699266	4,6418	-0,057466	0,960950485	1700011B04Rik
17501202	5,995208	6,221865	6,485782	6,379942	6,095819	6,054697	6,234285	6,176819333	-0,05746567	0,960950707	---
17540368	3,158336	3,536193	3,27697	3,295987	3,36537	3,137747	3,323833	3,266368	-0,057465	0,960951151	---
17549072	6,668289	6,535489	6,727338	6,670685	6,636257	6,451781	6,6437053	6,586241	-0,05746433	0,960951595	---
17421030	5,562775	5,810936	5,736159	5,330993	5,63768	5,968809	5,70329	5,645827333	-0,05746267	0,960952705	Gm13056
17398016	2,904966	2,724503	3,096151	2,88808	2,801792	2,863402	2,90854	2,851091333	-0,05744867	0,96096203	---
17202423	6,43918	6,205042	6,529062	6,315174	6,242312	6,443576	6,3910947	6,333687333	-0,05740733	0,960989562	---
17543229	7,260188	6,937352	6,89159	7,229997	6,904473	6,782446	7,02971	6,972305333	-0,05740467	0,960991339	Eif2s3x
17416967	6,32742	6,408539	6,25824	6,467042	6,206799	6,148205	6,3313997	6,274015333	-0,05738433	0,961004883	Spata6
17517244	4,822877	4,58723	4,596124	4,816721	4,596124	4,421257	4,6687437	4,611367333	-0,05737633	0,961010212	4933407I05Rik
17395830	7,985688	7,959379	7,940693	7,865305	7,855182	7,993163	7,96192	7,90455	-0,05737	0,961014431	Gmeb2
17375220	8,397965	8,507814	8,316207	8,315708	8,381161	8,353015	8,4073287	8,349961333	-0,05736733	0,961016207	Tubgcp4
17291229	4,707205	5,093501	4,896278	4,794568	4,999114	4,7312	4,8989947	4,841627333	-0,05736733	0,961016207	Hist1h4a
17526683	9,225482	9,313958	9,178736	9,079472	9,300344	9,166269	9,239392	9,182028333	-0,05736367	0,96101865	Rbm7
17422269	8,028582	8,247108	8,355753	8,148612	8,247183	8,063561	8,210481	8,153118667	-0,05736233	0,961019538	A430005L14Rik
17494507	6,661651	6,870869	7,172383	6,703152	6,842649	6,987038	6,9016343	6,844279667	-0,05735467	0,961024645	Smpd1 // Smpd1
17255588	6,149157	5,917001	6,100131	5,951589	6,110512	5,932199	6,0554297	5,9981	-0,05732967	0,961041298	Hoxb5
17414727	5,2822	4,90015	5,23911	5,043554	4,993055	5,212889	5,1404867	5,083166	-0,05732067	0,961047293	Mir455
17456053	8,65199	8,840306	8,614665	8,67132	8,65835	8,605331	8,7023203	8,645000333	-0,05732	0,961047738	Tmem106b
17539099	4,145315	4,033947	4,552984	4,271462	4,191129	4,097752	4,244082	4,186781	-0,057301	0,961060394	4930503H13Rik
17414908	3,63982	3,580651	3,609703	3,649638	3,250577	3,75806	3,610058	3,552758333	-0,05729967	0,961061283	Gm11240
17241810	4,129181	4,076742	4,27057	4,026042	4,108653	4,169919	4,158831	4,101538	-0,057293	0,961065724	Rtdr1
17404911	6,395455	6,385283	6,449704	6,404028	6,263994	6,390564	6,4101473	6,352862	-0,05728533	0,961070831	Nudt6
17363390	4,868429	4,619655	4,383915	4,615753	4,628508	4,455891	4,6239997	4,566717333	-0,05728233	0,961072829	Rorb
17373611	3,386167	3,198009	3,179324	3,207089	3,147394	3,237746	3,2546667	3,197409667	-0,057257	0,961089706	---
17203775	3,278464	3,792209	3,159588	3,685255	3,246604	3,126644	3,410087	3,352834333	-0,05725267	0,961092593	---
17437355	7,246881	7,062613	6,976927	6,942255	7,016839	7,155577	7,0954737	7,038223667	-0,05725	0,961094369	Pacrgl

17544491	8,077009	8,312476	7,979593	7,918343	8,171822	8,107267	8,123026	8,065810667	-0,05721533	0,961117464	Btk
17417478	3,106687	3,460886	3,155678	2,994184	3,330154	3,227277	3,2410837	3,183871667	-0,057212	0,961119684	---
17223985	5,736177	5,512579	5,38122	5,543591	5,492085	5,422664	5,5433253	5,486113333	-0,057212	0,961119684	Ikzf2
17437547	3,601444	3,737434	3,743121	3,677453	3,581097	3,651848	3,6939997	3,636799333	-0,05720033	0,961127456	Gm10440
17454657	3,01366	2,945446	3,127448	2,957876	2,997597	2,959498	3,0288513	2,971657	-0,05719433	0,961131454	---
17331021	6,656168	6,828142	6,76025	6,650642	6,686218	6,736124	6,7481867	6,690994667	-0,057192	0,961133008	Pcnp
17498897	6,582705	6,976116	6,433211	6,675089	6,662496	6,48288	6,6640107	6,606821667	-0,057189	0,961135007	Tnfsf13b
17374210	3,431687	3,69205	3,423484	3,244386	3,970991	3,160285	3,5157403	3,458554	-0,05718633	0,961136783	---
17471431	3,988348	4,720799	4,028462	4,19698	4,151799	4,217286	4,2458697	4,188688333	-0,05718133	0,961140114	Klrb1a
17243661	7,525022	7,748515	7,608222	7,406192	7,60322	7,700822	7,627253	7,570078	-0,057175	0,961144334	Prdm4
17348833	13,88772	13,89514	13,88732	13,88931	13,81262	13,79674	13,89006	13,83289	-0,05717	0,961147665	Ttr
17516349	4,541248	4,7135	4,676295	4,478065	4,784386	4,497089	4,643681	4,586513333	-0,05716767	0,961149219	---
17303353	6,275837	6,089939	5,901264	6,142402	5,788191	6,164978	6,0890133	6,031857	-0,05715633	0,96115677	Gm3696 // Gm3696
17511878	5,531445	5,621464	5,905253	5,747594	5,512414	5,626695	5,686054	5,628901	-0,057153	0,961158991	Pllp
17302136	3,332042	3,046491	4,298939	3,323132	3,782032	3,400935	3,5591573	3,502033	-0,05712433	0,961178089	---
17434764	5,42457	5,368536	5,570246	5,319343	5,443067	5,429577	5,4544507	5,397329	-0,05712167	0,961179866	Sema3a
17439367	5,818213	5,891506	6,167329	5,693861	5,975888	6,03596	5,959016	5,901903	-0,057113	0,96118564	Cxcl13
17268338	6,580609	6,437922	6,668339	6,509078	6,506034	6,500425	6,56229	6,505179	-0,057111	0,961186972	Snx11
17479834	6,285201	6,153178	5,762513	6,05018	5,996072	5,983308	6,066964	6,009853333	-0,05711067	0,961187195	Mex3b
17545486	3,108806	2,794113	3,074732	2,971607	3,097319	2,73743	2,9925503	2,935452	-0,05709833	0,961195412	---
17336322	6,710092	6,948502	6,889111	6,541757	7,095145	6,739512	6,849235	6,792138	-0,057097	0,9611963	Rxrb
17376372	6,656054	6,510858	6,769445	6,657385	6,554316	6,55339	6,6454523	6,588363667	-0,05708867	0,961201852	Oxt
17477754	4,855996	4,867071	4,918731	5,031438	4,671219	4,767878	4,8805993	4,823511667	-0,05708767	0,961202518	Slc6a16
17517268	5,597968	5,537417	5,702519	5,629664	5,629136	5,40787	5,6126347	5,555556667	-0,057078	0,961208959	---
17468678	8,716251	8,628453	8,672379	8,513896	8,680276	8,651701	8,672361	8,615291	-0,05707	0,961214289	Isy1
17402174	4,491825	4,427558	4,755824	4,643552	4,453887	4,40658	4,5584023	4,501339667	-0,05706267	0,961219175	4930432M17Rik // 4930432M17Rik
17376908	6,347926	6,038334	6,23997	6,206955	6,128622	6,119514	6,2087433	6,151697	-0,05704633	0,961230057	Ism1
17374055	3,217224	3,335079	3,095176	3,310747	3,215052	2,950543	3,2158263	3,158780667	-0,05704567	0,961230501	---
17273485	8,297369	8,354151	8,411674	8,356599	8,310414	8,225083	8,354398	8,297365333	-0,05703267	0,961239163	1110031I02Rik
17492722	6,402505	6,241172	6,342117	6,179268	6,330283	6,305146	6,328598	6,271565667	-0,05703233	0,961239385	Nmb
17354755	3,461805	3,248228	3,382773	3,268128	3,290838	3,362782	3,3642687	3,307249333	-0,05701933	0,961248047	Mir378 // Mir378
17200117	7,977314	7,538266	7,929435	7,710348	7,371874	8,191757	7,815005	7,757993	-0,057012	0,961252933	---
17519345	5,550728	5,121936	5,307509	5,342819	5,475292	4,991029	5,3267243	5,269713333	-0,057011	0,961253599	---
17501228	7,3159	7,271317	7,255409	7,162904	7,109939	7,398766	7,2808753	7,223869667	-0,05700567	0,961257153	---
17381626	4,741492	4,849179	4,76488	4,788192	4,605718	4,790697	4,7851837	4,728202333	-0,05698133	0,961273366	8030442B05Rik
17208733	5,0729	4,215689	5,67626	4,641429	4,964487	5,187993	4,988283	4,931303	-0,05698	0,961274254	---
17367978	5,680214	5,598892	5,484547	5,525243	5,433615	5,63388	5,5878843	5,530912667	-0,05697167	0,961279807	Npdc1
17208229	7,659557	8,184983	7,698588	7,747221	7,983221	7,64184	7,8477093	7,790760667	-0,05694867	0,961295132	---
17341242	4,383117	4,90823	4,655009	4,49542	4,704786	4,57532	4,6487853	4,591842	-0,05694333	0,961298686	Riok2 // Riok2
17252668	5,406137	5,15042	5,368959	5,25147	5,395868	5,107372	5,3085053	5,25157	-0,05693533	0,961304016	Trpv3
17202961	6,241813	5,478129	5,997282	6,097889	5,736688	5,711882	5,9057413	5,848819667	-0,05692167	0,961313123	---
17218680	12,06056	12,3955	12,24813	12,1035	12,19162	12,23832	12,23473	12,17781333	-0,05691667	0,961316455	Snord47
17229502	8,963329	8,901222	8,934841	8,825987	8,970811	8,831868	8,9331307	8,876222	-0,05690867	0,961321785	Uap1
17392437	7,905457	8,049288	8,034212	7,865678	8,022403	7,930162	7,996319	7,939414333	-0,05690467	0,961324451	Rbbp9
17418439	7,883934	7,929386	7,754144	7,814533	7,865221	7,717015	7,8558213	7,798923	-0,05689833	0,961328671	Snip1
17514049	7,786678	7,68459	7,813371	7,652787	7,801391	7,659827	7,7615463	7,704668333	-0,056878	0,96134222	Taf5l
17280609	7,830459	7,905791	7,580719	7,606335	7,762016	7,777986	7,772323	7,715445667	-0,05687733	0,961342664	Pik3cg
17221235	6,874737	7,350395	6,51867	6,59029	7,243382	6,739512	6,9146007	6,857728	-0,05687267	0,961345774	---
17544102	7,787044	7,904787	7,638198	7,674597	7,722387	7,762428	7,7766763	7,719804	-0,05687233	0,961345996	Brwd3
17306945	4,368436	4,610273	4,185273	4,467698	4,405516	4,120163	4,387994	4,331125667	-0,05686833	0,961348661	Mcpt-ps1
17403846	4,590132	4,401741	4,986253	4,645607	4,641114	4,520806	4,6593753	4,602509	-0,05686633	0,961349994	---
17517911	6,917135	6,816797	7,159702	6,753122	7,094997	6,874925	6,9645447	6,907681333	-0,05686333	0,961351993	---
17439094	4,364914	4,611339	4,600891	4,551132	4,455179	4,400288	4,5257147	4,468866333	-0,05684833	0,961361988	Art3
17388012	4,3603	4,160841	4,244062	4,379554	4,205963	4,009143	4,2550677	4,19822	-0,05684767	0,961362433	Olfr140
17370947	4,265094	4,093599	4,142223	4,109721	4,225452	3,995203	4,166972	4,110125333	-0,05684667	0,961363099	Gm13544
17337585	2,895554	3,04651	3,034445	3,025271	2,958173	2,82254	2,9921697	2,935328	-0,05684167	0,961366431	Olfr119
17229590	5,25828	5,105079	5,454692	5,064235	5,215654	5,36779	5,2726837	5,215893	-0,05679067	0,961400416	Fcrlb
17213446	7,336636	7,270334	7,163686	7,255761	7,176547	7,168003	7,2568853	7,200103667	-0,05678167	0,961406414	Abi2
17448832	5,352122	5,249662	5,234241	5,232372	5,447851	4,985492	5,278675	5,221905	-0,05677	0,961414189	Lrrc66
17550643	2,857857	2,85026	2,90085	2,813632	2,860957	2,764141	2,8696557	2,81291	-0,05674567	0,961430404	---
17475064	4,186465	4,424128	4,489306	4,277013	4,135818	4,516856	4,366633	4,309895667	-0,05673733	0,961435958	Gm1096
17440971	4,795031	4,53684	5,16823	4,617232	5,237551	4,475146	4,833367	4,776643	-0,056724	0,961444843	---
17213265	5,728401	5,575364	5,393958	5,754468	5,351057	5,422059	5,5659077	5,509194667	-0,056713	0,961452174	Fzd7
17316996	10,51786	10,4656	10,5191	10,38646	10,48368	10,46234	10,500853	10,44416	-0,05669333	0,961465281	Rad21
17330947	3,955168	3,782664	3,76578	3,688665	3,756108	3,888764	3,8345373	3,777845667	-0,05669167	0,961466391	Zpld1
17385389	3,775018	3,520016	3,889484	3,585615	3,853464	3,575481	3,7281727	3,67152	-0,05665267	0,961492383	BB557941
17435737	4,817961	4,775106	4,932412	4,614662	5,036207	4,704681	4,8418263	4,785183333	-0,056643	0,961498825	Fam59b
17548292	4,17834	4,22983	4,16457	4,045791	4,232584	4,124476	4,1909133	4,134283667	-0,05662967	0,961507711	Gm2401
17230706	6,848847	6,648181	6,824807	6,893061	6,611091	6,647833	6,773945	6,717328333	-0,05661667	0,961516376	1700112H15Rik // 1700112H15Rik
17433555	7,123333	7,054442	7,284475	7,149175	7,076368	7,066861	7,1540833	7,097468	-0,05661533	0,961517264	1190007F08Rik
17241968	8,373338	8,037341	8,263105	8,210251	8,119711	8,173995	8,2245947	8,167985667	-0,056609	0,961521485	Smarcb1
17545657	7,33302	7,043288	7,119986	7,123475	7,089412	7,113589	7,1654313	7,108825333	-0,056606	0,961523485	Sms
17379942	5,527267	5,52078	5,426938	5,617981	5,228086	5,459114	5,4916617	5,435060333	-0,05660133	0,961526595	A530013C23Rik
17388901	5,610383	5,408956	5,500852	5,521369	5,268448	5,56058	5,5067303	5,450132333	-0,056598	0,961528816	D430041D05Rik
17537038	4,425657	4,477375	4,481633	4,219343	4,589379	4,406175	4,461555	4,404965667	-0,05658933	0,961534593	Fgf16
17306087	4,825649	4,721276	5,019595	5,00049	4,914851	4,481471	4,8555067	4,798937333	-0,05656933	0,961547922	Rnase11
17352493	8,151929	8,182175	8,078917	7,980519	8,120003	8,142833	8,1376737	8,081118333	-0,05655533	0,961557253	Epc1
17488370	4,015503	4,127445	4,521336	4,108956	4,098527	4,287142	4,221428	4,164875	-0,056553	0,961558809	BC089491
17313158	5,273817	5,018065	5,197286	5,048529	5,209814	5,061209	5,163056	5,106517333	-0,05653867	0,961568362	Fam83f
17469887	8,867804	8,928473	8,611986	8,695885	8,766228	8,776541	8,8027543	8,746218	-0,05653633	0,961569917	2510049J12Rik
17475805	5,788994	5,956376	5,948443	5,916126	5,705744	5,902424	5,8979377	5,841431333	-0,05650633	0,961589912	Eid2b
17535181	3,871682	3,721678	3,715865	3,530718	3,937076	3,671984	3,7697417	3,713259333	-0,05648233	0,961605909	---
17219086	9,215615	9,442917	9,028737	9,092178	9,241203	9,184444	9,2290897	9,172608333	-0,05648133	0,961606576	Tmco1
17421142	6,500916	6,842165	6,743905	6,549093	6,54447	6,824022	6,695662	6,639195	-0,056467	0,961616129	---
17235518	5,071432	4,985903	4,752242	4,939055	4,800945	4,900177	4,9365257	4,880059	-0,05646667	0,961616352	---
17547523	4,181687	4,160127	4,295536	4,183295	4,167994	4,116671	4,21245	4,155986667	-0,05646333	0,961618573	Gm10752
17365410	7,718822	7,52729	7,445278	7,310956	7,379279	7,831806	7,5637967	7,507347	-0,05644967	0,961627683	Cyp17a1
17473514	4,60897	5,289295	5,253145	4,771067	5,197208	5,013795	5,05047	4,994023333	-0,05644667	0,961629683	Gm5065
17460090	4,43433	4,35659	4,447161	4,270681	4,481137	4,316933	4,4126937	4,356250333	-0,05644333	0,961631904	LOC100861870
17483870	5,148391	5,292417	5,169984	5,134152	5,171606	5,135713	5,2035973	5,147157	-0,05644033	0,961633904	Btbd16
17387681	3,252347	3,184906	3,226825	3,120201	3,179402	3,195159	3,2213593	3,164920667	-0,05643867	0,961635015	Olfr1066
17548002	3,997096	3,955778	3,803088	3,754936	3,910988	3,920732	3,918654	3,862218667	-0,05643533	0,961637237	Gm9753
17495309	4,893968	4,833868	4,869529	4,800024	4,851681	4,7764	4,8657883	4,809368333	-0,05642	0,961647457	4933406I18Rik
17205385	5,414843	5,008038	5,063701	5,569968	4,948729	4,798627	5,162194	5,105774667	-0,05641933	0,961647902	---
17334730	5,460957	5,468548	5,643569	5,522552	5,492176	5,38909	5,524358	5,467939333	-0,05641867	0,961648346	Mslnl
17217536	5,876392	5,69203	5,918535	5,711536	5,849108	5,757062	5,8289857	5,772568667	-0,056417	0,961649457	Syt2
17233286	4,346557	4,300836	4,168368	4,210285	4,252983	4,183252	4,2719203	4,215506667	-0,05641367	0,961651679	Nepn
17254089	7,23394	7,32637	7,199691	7,279906	6,964077	7,34683	7,2533337	7,196937667	-0,056396	0,961663455	Zfp830
17441265	7,657854	7,90028	7,81222	7,630466	7,720016	7,850685	7,790118	7,733722333	-0,05639567	0,961663677	Rab35
17209699	6,022525	6,061249	5,786878	5,908494	5,298505	6,494477	5,956884	5,900492	-0,056392	0,961666121	---
17425364	4,698923	4,632706	4,833206	4,593683	4,826596	4,575438	4,7216117	4,665239	-0,05637267	0,961679009	AI427809
17433944	5,78796	5,554796	5,624396	5,632486	5,549276	5,616329	5,6557173	5,599363667	-0,05635367	0,961691674	Ttll10
17442332	8,077712	8,144751	8,098588	8,029882	8,108147	8,013967	8,107017	8,050665333	-0,05635167	0,961693007	Kntc1
17507440	7,55742	7,485945	7,730635	7,433961	7,596973	7,574022	7,5913333	7,534985333	-0,056348	0,961695451	Cars2
17296225	4,370241	4,256165	4,435141	4,279063	4,267676	4,345766	4,353849	4,297501667	-0,05634733	0,961695896	Ddx4
17261161	4,775841	4,772346	5,099097	4,731766	5,071432	4,675055	4,882428	4,826084333	-0,05634367	0,96169834	Bcl11a
17404821	11,1271	11,12395	11,14429	11,04125	11,05428	11,13078	11,13178	11,07543667	-0,05634333	0,961698562	Ccna2
17498461	6,571856	6,810899	6,554738	6,535687	6,829948	6,402847	6,645831	6,589494	-0,056337	0,961702784	Fadd
17225196	4,949028	5,051235	5,380002	5,024067	5,053102	5,134105	5,126755	5,070424667	-0,05633033	0,961707228	Alppl2
17466262	3,578801	3,713975	3,763884	3,709314	3,657962	3,520399	3,6855533	3,629225	-0,05632833	0,961708561	1700074P13Rik
17276787	6,096926	6,045325	5,862319	5,942326	5,962497	5,930838	6,0015233	5,945220333	-0,056303	0,961725448	Rdh12
17406433	6,615402	6,352062	6,799687	6,662992	6,386966	6,548355	6,5890503	6,532771	-0,05627933	0,961741225	Prss48
17512169	3,140032	3,442424	3,27193	3,324272	3,202883	3,158443	3,2847953	3,228532667	-0,05626267	0,961752336	---
17408974	8,239473	7,966986	8,393667	8,127701	8,060256	8,243385	8,200042	8,143780667	-0,05626133	0,961753225	Cym
17335865	8,232122	8,119054	8,145748	8,087137	8,068171	8,172834	8,1656413	8,109380667	-0,05626067	0,961753669	Ndufv3
17528947	4,752241	4,707014	4,739225	4,613978	4,792757	4,62297	4,7328267	4,676568333	-0,05625833	0,961755224	2310046A06Rik
17532797	3,959337	3,86512	3,727721	3,889498	3,626748	3,867166	3,850726	3,794470667	-0,05625533	0,961757224	---
17498748	6,309594	6,482553	5,780352	6,337942	5,901405	6,16439	6,190833	6,134579	-0,056254	0,961758113	---
17363239	8,663555	8,760989	8,694559	8,662727	8,635139	8,652504	8,7063677	8,650123333	-0,05624433	0,961764557	Vps13a
17244486	6,488498	6,492607	6,433518	6,547652	6,403592	6,294678	6,471541	6,415307333	-0,05623367	0,961771668	Socs2
17308462	11,73345	11,79243	11,62918	11,42354	11,82589	11,73695	11,718353	11,66212667	-0,05622667	0,961776335	---
17318076	4,148948	4,116752	4,210886	4,160524	4,127332	4,020069	4,158862	4,102641667	-0,05622033	0,961780557	Ly6i
17462738	7,045088	6,768427	6,592803	6,709154	6,887095	6,641542	6,802106	6,745930333	-0,05617567	0,961810335	Clec4a3
17335874	4,741468	4,891835	4,940029	4,804593	4,893229	4,707016	4,8577773	4,801612667	-0,05616467	0,961817668	4833413E03Rik
17550867	3,727044	3,719257	3,415649	3,553808	3,588044	3,551618	3,62065	3,56449	-0,05616	0,961820779	---
17246569	5,049754	5,304368	5,453903	5,166852	5,035811	5,436887	5,2693417	5,213183333	-0,05615833	0,961821891	Gm11944
17306814	3,355491	3,361593	3,322834	3,20755	3,271031	3,392876	3,3466393	3,290485667	-0,05615367	0,961825002	---
17375654	4,658927	4,278615	4,251328	4,436171	4,224276	4,36	4,39629	4,340149	-0,056141	0,961833446	---
17455622	5,744402	5,694127	5,791968	5,667634	5,65855	5,735902	5,743499	5,687362	-0,056137	0,961836113	A430089I19Rik
17350007	6,759709	6,895415	7,047712	6,899633	7,001535	6,633258	6,9009453	6,844808667	-0,05613667	0,961836335	---
17529496	8,393763	8,421783	8,054336	8,103661	8,267141	8,330695	8,2899607	8,233832333	-0,05612833	0,961841891	Snx14
17397396	3,39089	3,372524	3,486898	3,49208	3,217332	3,372524	3,4167707	3,360645333	-0,05612533	0,961843891	---
17458561	8,400502	8,123755	8,262495	8,168365	8,37728	8,07274	8,2622507	8,206128333	-0,05612233	0,961845891	Cbx3
17446100	8,384007	8,197194	8,302537	8,204541	8,229398	8,28145	8,2945793	8,238463	-0,05611633	0,961849892	Fam126a

17269158	3,602498	3,647549	3,675852	3,576596	3,851146	3,329811	3,6419663	3,585851	-0,05611533	0,961850558	Krtap2-4
17452689	8,812504	8,890738	8,714322	8,809817	8,660474	8,778939	8,8058547	8,749743333	-0,05611133	0,961853225	Rsrc2
17517031	8,160338	8,126559	8,227657	8,111385	8,116487	8,118359	8,171518	8,115410333	-0,05610767	0,96185567	Zw10
17266911	4,946424	5,135851	5,207408	4,987453	5,252705	4,881205	5,096561	5,040454333	-0,05610667	0,961856337	Mmp28
17201225	9,04715	9,206813	8,68376	8,512155	8,838833	9,418424	8,979241	8,923137333	-0,05610367	0,961858337	---
17328425	6,414042	6,835252	6,754981	6,652135	6,616346	6,567488	6,6680917	6,611989667	-0,056102	0,961859448	Ube2v2
17345038	9,942628	10,15108	9,762162	9,977216	9,649272	10,06109	9,9519567	9,895859333	-0,05609733	0,961862559	Cd2ap
17217580	7,344812	7,458387	7,159555	7,319115	7,638988	6,836362	7,320918	7,264821667	-0,05609633	0,961863226	Arl8a
17262915	5,949051	5,710437	5,950033	5,779369	5,853891	5,807994	5,8698403	5,813751333	-0,056089	0,961868115	Ccdc69
17466195	8,371563	8,359914	8,242887	8,220166	8,386505	8,19944	8,324788	8,268703667	-0,05608433	0,961871226	Mrps33
17246310	8,220006	8,0159	8,048195	8,069114	8,058825	7,98792	8,0947003	8,038619667	-0,05608067	0,961873671	Dgka
17317296	8,164129	8,094757	8,107752	8,058025	8,104712	8,035689	8,1222127	8,066142	-0,05607067	0,961880338	Tmem65
17467433	3,052595	2,804229	2,886929	2,78262	2,924919	2,868012	2,9145843	2,858517	-0,05606733	0,961882561	---
17283780	5,319383	5,047656	5,487128	5,213218	5,163713	5,309103	5,2847223	5,228678	-0,05604433	0,961897896	Gm2721
17449939	8,971726	9,322596	8,978252	8,922594	9,144481	9,037378	9,090858	9,034817667	-0,05604033	0,961900562	Antxr2
17522785	6,12674	6,010418	6,02998	6,043478	6,002718	5,95288	6,0557127	5,999692	-0,05602067	0,961913675	---
17416761	8,587426	8,856168	8,592329	8,493227	8,668545	8,706125	8,678641	8,622632333	-0,05600867	0,961921676	Kti12
17502090	2,955092	3,002134	2,997003	2,930252	2,900901	2,955092	2,984743	2,928748333	-0,05599467	0,961931011	Gm3643
17381519	3,191837	3,277968	3,384198	3,192956	3,08502	3,408113	3,2846677	3,228696333	-0,05597133	0,961946569	---
17335952	6,611288	7,186004	6,871466	6,924063	6,729939	6,846888	6,889586	6,83363	-0,055956	0,961956792	Cyp4f17
17340917	4,990322	4,725413	5,079836	4,867023	4,878794	4,881919	4,931857	4,875912	-0,055945	0,961964127	Pnldc1
17253422	5,770139	6,408823	5,965877	5,7438	6,165988	6,067217	6,0482797	5,992335	-0,05594467	0,961964349	Dhrs13
17338650	7,401518	7,449318	7,624775	7,299065	7,53081	7,477917	7,4918703	7,435930667	-0,05593967	0,961967683	Ccdc94
17545567	4,261334	4,081388	4,207393	3,9124	4,256821	4,213087	4,1833717	4,127436	-0,05593567	0,96197035	Magea2
17501500	2,815119	2,903276	3,046128	3,003224	2,815119	2,778389	2,9215077	2,865577333	-0,05593033	0,961973907	---
17444970	4,765249	4,796027	4,809942	4,689435	4,771127	4,742865	4,790406	4,734475667	-0,05593033	0,961973907	6330406I15Rik
17220597	4,902667	5,144135	4,715545	4,639516	5,023005	4,932037	4,9207823	4,864852667	-0,05592967	0,961974351	A430105J06Rik
17321351	9,571942	9,713388	9,621521	9,604284	9,649506	9,485298	9,635617	9,579696	-0,055921	0,96198013	Prkag1
17485724	6,99884	6,793851	7,154771	6,668784	6,959403	7,151578	6,9824873	6,926588333	-0,055899	0,9619948	Rdh13
17299196	7,651116	7,590559	7,776451	7,524364	7,63345	7,692618	7,6727087	7,616810667	-0,055898	0,961995466	Ero1l // Ero1l
17502366	3,574731	3,619201	3,55418	3,592442	3,49258	3,495402	3,582704	3,526808	-0,055896	0,9619968	Olfr372
17511389	9,644684	9,823026	9,60982	9,556777	9,648916	9,704187	9,69251	9,636626667	-0,05588333	0,962005246	Itfg1
17331910	4,026051	3,84153	3,724585	3,804276	3,864274	3,756021	3,8640553	3,808190333	-0,055865	0,962017471	Krtap7-1
17312127	9,769585	9,700356	9,720568	9,560213	9,700003	9,762711	9,7301697	9,674309	-0,05586067	0,962020361	Ptp4a3
17520687	7,761747	7,927397	7,663168	7,641836	7,757628	7,785319	7,784104	7,728261	-0,055843	0,962032141	Cep70
17261016	7,307198	7,282303	7,420195	7,262949	7,356919	7,222312	7,3365653	7,280726667	-0,05583867	0,962035031	Commd1
17259311	5,083655	5,193876	5,344247	5,188437	5,087405	5,178566	5,2072593	5,151469333	-0,05579	0,962067484	0610009L18Rik
17543393	4,441112	4,373288	4,573016	4,049978	4,900551	4,269526	4,462472	4,406685	-0,055787	0,962069484	Gm9059
17428228	4,212274	4,124691	4,211803	4,227608	4,053306	4,100518	4,1829227	4,127144	-0,05577867	0,962075042	Elavl4
17290324	7,11007	7,129649	7,186743	6,983919	7,244622	7,120638	7,142154	7,086393	-0,055761	0,962086823	Pfkp
17392973	4,642804	4,39692	4,544879	4,377816	4,497044	4,5425	4,528201	4,472453333	-0,05574767	0,962095714	Defb45
17453160	8,324372	8,429084	8,502067	8,435305	8,263857	8,389369	8,4185077	8,362777	-0,05573067	0,962107051	Gusb
17448735	5,623774	5,841745	5,952893	5,554058	5,870677	5,826498	5,8061373	5,750411	-0,05572633	0,962109941	Slain2 // Slain2
17367921	5,743276	5,522435	5,743276	5,624959	5,548177	5,668676	5,6696623	5,613937333	-0,055725	0,96211083	Grin1 // Grin1
17314577	6,84262	7,070874	6,941515	6,93199	6,937146	6,818707	6,9516697	6,895947667	-0,055722	0,962112831	Pfkm
17384182	6,075015	6,193857	6,017611	5,944676	6,019387	6,150968	6,0940653	6,038343667	-0,05572167	0,962113053	Zbtb43
17366047	3,909941	4,023902	3,909611	3,915795	3,861694	3,898802	3,947818	3,892097	-0,055721	0,962113498	Emx2os
17299185	4,629343	4,495322	4,49098	4,405274	4,602653	4,440566	4,5385483	4,482831	-0,05571733	0,962115943	Gpr137c
17386448	5,265139	5,505568	5,55449	5,464854	5,34362	5,349615	5,4417323	5,386029667	-0,05570267	0,962125724	---
17214380	5,978284	5,730981	5,873468	5,605126	5,93831	5,872206	5,860911	5,805214	-0,055697	0,962129503	Wnt6
17249057	8,021902	8,268747	8,011869	8,055144	8,016582	8,063737	8,1008393	8,045154333	-0,055685	0,962137506	Mapk9
17493088	3,145683	2,977479	3,19102	3,072747	2,957014	3,117395	3,1047273	3,049052	-0,05567533	0,962143953	Vmn2r73
17471814	3,167095	3,30993	3,274414	3,2079	3,230738	3,1458	3,2504797	3,194812667	-0,055667	0,96214951	Gm6600
17208065	3,359665	2,982133	3,451103	2,859506	3,396095	3,370339	3,2643003	3,208646667	-0,05565367	0,962158403	---
17443083	6,912179	6,958998	7,018639	6,77378	6,99261	6,956532	6,963272	6,907640667	-0,05563133	0,962173297	Gtf2ird2
17493141	3,504254	3,426395	3,998949	3,836405	3,584504	3,341802	3,6431993	3,587570333	-0,055629	0,962174853	Olfr298
17200193	6,143806	6,095213	6,489531	6,376842	5,927543	6,257284	6,24285	6,187223	-0,055627	0,962176187	---
17203933	3,864782	3,302251	3,816156	3,591482	3,18846	4,036427	3,661063	3,605456333	-0,05560667	0,962189748	---
17262669	7,441801	7,517662	7,470141	7,160909	7,683629	7,418268	7,4765347	7,420935333	-0,05559933	0,962194639	Sowaha
17355278	3,063129	3,125259	3,125259	3,037667	2,98747	3,121716	3,104549	3,048951	-0,055598	0,962195528	---
17474434	6,214888	6,40684	6,161487	6,356208	6,000401	6,25983	6,2610717	6,205479667	-0,055592	0,96219953	Rsph6a
17526266	7,423626	8,143688	7,979384	7,437567	7,812131	8,130235	7,8488993	7,793311	-0,05558833	0,962201976	---
17254670	8,557659	8,747194	8,520756	8,529218	8,530401	8,599236	8,6085363	8,552951667	-0,05558467	0,962204421	Fam33a
17340712	5,770944	5,546705	5,734694	5,737102	5,658373	5,490148	5,6841143	5,628541	-0,05557333	0,96221198	Gm1604b
17202455	2,939525	2,689253	3,083236	3,135768	2,655457	2,7541	2,9040047	2,848441667	-0,055563	0,962218872	---
17248331	8,375311	8,306183	8,116362	8,143345	8,239305	8,248537	8,265952	8,210395667	-0,05555633	0,962223318	Fbxw11
17277794	8,411159	8,525056	8,199857	8,16482	8,491834	8,312751	8,3786907	8,323135	-0,05555567	0,962223763	Gpr65
17216436	4,129022	4,265658	4,571548	4,525406	4,079648	4,194511	4,322076	4,266521667	-0,05555433	0,962224652	Serpinb11
17525688	4,081475	3,952835	4,235925	4,346232	3,843845	3,913495	4,0900783	4,034524	-0,05555433	0,962224652	---
17484229	5,607119	5,736159	6,063276	5,872498	5,650904	5,716508	5,8021847	5,746636667	-0,055548	0,962228876	Foxi2
17491507	5,309396	4,599779	4,255647	4,805081	4,529888	4,663247	4,7216073	4,666072	-0,05553533	0,962237324	---
17391909	5,34242	5,326917	5,284677	5,344791	5,230686	5,211976	5,3180047	5,262484333	-0,05552033	0,962247329	5330413P13Rik
17210159	3,045084	3,151656	3,073797	2,818989	3,049589	3,235446	3,090179	3,034674667	-0,05550433	0,962258001	---
17379104	3,287792	3,741097	3,285918	3,310449	3,403473	3,43439	3,438269	3,382770667	-0,05549833	0,962262003	---
17285765	2,874768	2,882527	2,91161	2,777858	2,851911	2,872664	2,889635	2,834144333	-0,05549067	0,962267116	Vmn1r200
17270297	4,869473	4,404959	4,562256	4,688145	4,472395	4,509708	4,6122293	4,556749333	-0,05548	0,962274231	2810433D01Rik
17391094	7,330041	7,618842	7,351245	7,238217	7,394334	7,501139	7,433376	7,377896667	-0,05547933	0,962274676	Hdc
17355780	4,733514	4,818614	4,988811	4,786321	4,812657	4,605765	4,7903797	4,734914333	-0,05546533	0,962284014	1700095A13Rik
17287552	3,864403	3,765659	4,015801	3,904344	4,020427	3,554728	3,8819543	3,826499667	-0,05545467	0,962291128	5330429C05Rik
17247695	10,1927	10,21027	10,09954	10,1313	10,06161	10,14326	10,167503	10,11205667	-0,05544667	0,962296464	Xpo1
17322598	4,626249	4,663375	4,701482	4,645	4,666981	4,512799	4,663702	4,60826	-0,055442	0,962299577	Gm10914 // Gm10914
17389736	6,432492	6,155439	6,498384	6,151513	6,41795	6,350551	6,362105	6,306671333	-0,05543367	0,962305136	Gm1337
17283477	7,234529	7,111208	7,19333	7,1618	7,086433	7,124541	7,179689	7,124258	-0,055431	0,962306914	Moap1
17475861	6,203709	6,130054	6,326542	6,712574	5,35571	6,425784	6,2201017	6,164689333	-0,05541233	0,962319366	1700028B04Rik
17228896	3,991845	3,866158	3,725981	3,996231	3,518234	3,903294	3,861328	3,805919667	-0,05540833	0,962322034	4930558K02Rik
17211752	6,074214	6,010437	6,159626	6,020846	6,106805	5,950435	6,0814257	6,026028667	-0,055397	0,962329593	Arhgef4
17326428	10,84204	10,80771	10,86621	10,88859	10,70872	10,7525	10,838653	10,78327	-0,05538333	0,96233871	Cpox
17231495	4,523829	4,515704	4,502989	4,698106	4,418253	4,260055	4,514174	4,458804667	-0,05536933	0,962348048	Oprm1
17489244	8,115223	7,816056	7,822444	7,801847	8,09331	7,692477	7,9179077	7,862544667	-0,055363	0,962352273	---
17544088	8,269265	8,295761	7,932955	8,15155	7,945178	8,234575	8,1659937	8,110636333	-0,05535733	0,962356053	2610002M06Rik
17216301	7,532894	7,548146	7,691162	7,584665	7,492364	7,529103	7,590734	7,535377333	-0,05535667	0,962356498	---
17548112	11,35868	11,25267	11,16089	11,16352	11,26744	11,17523	11,257413	11,20206333	-0,05535	0,962360945	Morf4l2 // Morf4l2
17529580	5,292673	5,041319	5,103957	5,206621	5,045431	5,019866	5,145983	5,090639333	-0,05534367	0,962365169	---
17291704	6,042693	5,805016	5,908166	5,982065	5,74369	5,864093	5,918625	5,863282667	-0,05534233	0,962366059	Serpinb1c
17468509	3,553248	3,490435	3,546713	3,535062	3,441873	3,447462	3,530132	3,474799	-0,055333	0,962372285	---
17310772	5,101456	4,968239	5,302693	5,10345	5,042887	5,060074	5,1241293	5,068803667	-0,05532567	0,962377176	Ctnnd2
17372111	5,115633	4,490017	4,730798	4,803044	4,697964	4,66957	4,778816	4,723526	-0,05529	0,962400969	---
17550821	3,842374	3,827115	4,247842	3,616811	4,043077	4,09165	3,9724437	3,917179333	-0,05526433	0,962418091	---
17441083	8,95149	8,826523	9,033241	8,786223	8,930244	8,929012	8,9370847	8,881826333	-0,05525833	0,962422093	Sppl3
17255487	6,910204	6,937788	6,99456	7,150651	6,667475	6,858686	6,9475173	6,892270667	-0,05524667	0,962429876	Gip
17364663	2,86414	2,904603	2,894462	2,811668	2,781544	2,904262	2,887735	2,832491333	-0,05524367	0,962431878	---
17541776	3,172808	3,083785	3,091749	3,10727	3,028533	3,04684	3,116114	3,060881	-0,055233	0,962438993	Cxx1a
17240020	12,92127	12,90345	12,94638	12,85639	12,90978	12,83924	12,9237	12,86847	-0,05523	0,962440995	Rpl12
17542180	8,319319	8,496692	8,271185	8,197294	8,276012	8,448246	8,3623987	8,307184	-0,05521467	0,962451224	Cd99l2
17493980	6,822261	6,489136	6,553851	6,639709	6,376058	6,683882	6,6217493	6,566549667	-0,05519967	0,962461231	Lrrc51
17398023	9,845282	9,915337	9,814134	9,757704	9,769213	9,882245	9,858251	9,803054	-0,055197	0,96246301	Gmps
17362138	6,724905	6,651335	6,798324	6,619506	6,701975	6,687498	6,7248547	6,669659667	-0,055195	0,962464344	Gpr137
17468271	3,672056	3,908752	3,538572	3,551294	3,794051	3,608476	3,70646	3,651273667	-0,05518633	0,962470126	---
17488951	8,266468	8,138231	8,145828	8,217718	8,170019	7,997302	8,183509	8,128346333	-0,05516267	0,962485915	Zfp146
17469941	4,732406	5,154031	5,000252	4,855348	4,847975	5,017885	4,9622297	4,907069333	-0,05516033	0,962487471	BC060267
17549242	3,622353	3,609703	3,56083	3,792771	3,22878	3,605876	3,5976287	3,542475667	-0,055153	0,962492364	---
17312942	9,416952	9,340074	9,38591	9,393077	9,292756	9,291655	9,3809787	9,325829333	-0,05514933	0,96249481	---
17236100	3,335173	3,336487	3,370118	3,218157	3,421698	3,236491	3,3472593	3,292115333	-0,055144	0,962498368	---
17549958	3,335173	3,336487	3,370118	3,218157	3,421698	3,236491	3,3472593	3,292115333	-0,055144	0,962498368	---
17325321	3,15217	3,058601	3,006169	3,044864	3,143087	2,863574	3,0723133	3,017175	-0,05513833	0,962502149	---
17210337	7,908637	7,382452	7,617321	7,768157	7,269642	7,705206	7,6361367	7,581001667	-0,055135	0,962504373	---
17368389	8,668319	8,672056	8,612157	8,680679	8,555696	8,550766	8,650844	8,595713667	-0,05513033	0,962507486	Surf2
17325531	3,109372	3,057745	3,111498	3,029415	3,013027	3,070821	3,0928717	3,037754333	-0,05511733	0,962516159	---
17247015	7,295855	7,434337	7,466151	7,268987	7,362049	7,399956	7,398781	7,343664	-0,055117	0,962516382	Urgcp
17282025	10,32441	10,38525	10,38574	10,24921	10,22087	10,45999	10,365133	10,31002333	-0,05511	0,962521052	Spnb1
17387991	3,250899	3,10286	3,406786	2,986492	3,429845	3,178886	3,253515	3,198407667	-0,05510733	0,962522831	Olfr1252
17544324	3,004005	3,052278	3,270892	2,971852	3,210627	2,979376	3,1090583	3,053951667	-0,05510667	0,962523276	Vmn2r121
17236378	9,082374	9,048482	9,156519	9,025623	9,077947	9,018486	9,0957917	9,040685333	-0,05510633	0,962523498	Arl1
17312716	6,613832	6,509401	6,718943	6,643301	6,481174	6,5524	6,6140587	6,558958333	-0,05510033	0,962527501	Csf2rb
17419608	6,405725	6,257559	6,480377	6,339844	6,316317	6,32223	6,3812203	6,326130333	-0,05509	0,962534395	Fam46b
17544333	11,973	12,07199	12,2457	12,10423	12,09398	11,92726	12,096897	12,04182333	-0,05507333	0,962545515	Rps12 // Rps12
17544335	11,973	12,07199	12,2457	12,10423	12,09398	11,92726	12,096897	12,04182333	-0,05507333	0,962545515	Rps12 // Rps12
17544337	11,973	12,07199	12,2457	12,10423	12,09398	11,92726	12,096897	12,04182333	-0,05507333	0,962545515	Rps12 // Rps12
17544339	11,973	12,07199	12,2457	12,10423	12,09398	11,92726	12,096897	12,04182333	-0,05507333	0,962545515	Rps12 // Rps12
17544341	11,973	12,07199	12,2457	12,10423	12,09398	11,92726	12,096897	12,04182333	-0,05507333	0,962545515	Rps12 // Rps12
17544343	11,973	12,07199	12,2457	12,10423	12,09398	11,92726	12,096897	12,04182333	-0,05507333	0,962545515	Rps12 // Rps12
17321838	4,23082	4,333426	4,38894	4,654041	4,186474	3,947465	4,3177287	4,26266	-0,05506867	0,962548628	Krt83
17500543	8,837342	8,803797	8,861569	8,72453	8,793582	8,819394	8,834236	8,779168667	-0,05506733	0,962549518	Ppp1r3b
17501094	4,67368	4,706292	4,595166	4,718379	4,641473	4,450117	4,6583793	4,603323	-0,05505633	0,962556857	---

17286788	6,027829	5,787524	5,857564	5,686787	6,126414	5,69457	5,8909723	5,835923667	-0,05504867	0,962561972	---
17492064	2,776227	3,071195	2,973156	2,780037	3,095363	2,780037	2,9401927	2,885145667	-0,055047	0,962563084	---
17246418	5,336075	5,109068	5,318456	5,355571	5,133893	5,109091	5,254533	5,199518333	-0,05501467	0,962584657	Olfr796
17266157	9,588144	10,04241	9,722363	9,796654	9,703209	9,688089	9,7843057	9,729317333	-0,05498833	0,962602227	Cpd
17424421	6,911304	6,752811	6,954466	6,89136	6,789735	6,772523	6,8728603	6,817872667	-0,05498767	0,962602672	Ccl19
17434190	6,911304	6,752811	6,954466	6,89136	6,789735	6,772523	6,8728603	6,817872667	-0,05498767	0,962602672	Ccl19
17451811	5,513869	5,073395	5,304992	5,478092	5,146763	5,102451	5,2974187	5,242435333	-0,05498333	0,962605563	---
17374488	9,339617	9,536724	9,179099	9,266795	9,436983	9,186777	9,3518133	9,296851667	-0,05496167	0,96262002	Thbs1
17200495	6,511989	7,265649	7,081297	7,358811	6,556518	6,778735	6,9529783	6,898021333	-0,054957	0,962623134	---
17247823	8,591581	8,629736	8,475063	8,538703	8,571737	8,421086	8,56546	8,510508667	-0,05495133	0,962626915	Pus10
17205527	7,690274	6,707231	6,952998	7,420537	6,848376	6,916736	7,1168343	7,061883	-0,05495133	0,962626915	---
17247921	7,153907	6,890532	7,040251	6,991328	6,971362	6,957252	7,02823	6,973314	-0,054916	0,962650491	Vrk2 // Vrk2
17388897	4,129731	4,081504	4,313226	4,18043	4,081504	4,097781	4,1748203	4,119905	-0,05491533	0,962650936	---
17234720	5,689606	5,660358	5,899121	5,737961	5,659275	5,687105	5,749695	5,694780333	-0,05491467	0,962651381	C330046G03Rik
17346664	4,583987	4,160155	4,003574	4,327527	4,314747	3,940718	4,2492387	4,194330667	-0,054908	0,962655829	---
17247852	8,772904	8,732279	8,741792	8,684567	8,685844	8,711907	8,7489917	8,694106	-0,05488567	0,962670732	Bcl11a
17502423	8,544881	8,641001	8,669517	8,557178	8,493647	8,639968	8,6184663	8,563597667	-0,05486867	0,962682075	Ap1m1
17244997	4,251172	3,927551	3,98899	3,996265	4,040506	3,966367	4,0559043	4,001046	-0,05485833	0,962688971	Tph2
17319501	9,278637	9,373585	9,342442	9,205136	9,349857	9,275136	9,3315547	9,276709667	-0,054845	0,962697868	Rangap1
17294250	5,034416	5,179221	5,236648	5,130052	5,328428	4,827296	5,150095	5,095258667	-0,05483633	0,962703651	Slc6a18
17300565	6,353535	5,633504	5,833379	5,854656	5,81112	5,990155	5,9401393	5,885310333	-0,054829	0,962708544	Rnf31
17513446	5,72027	5,909166	5,835357	5,715919	5,718525	5,865903	5,8215977	5,766782333	-0,05481533	0,962717664	Hsdl1
17482041	5,538308	5,000725	5,416537	5,360089	5,241522	5,189522	5,3185233	5,263711	-0,05481233	0,962719666	Calcb
17514754	4,955	5,085936	5,119566	4,920463	5,255177	4,820462	5,0535007	4,998700667	-0,0548	0,962727896	---
17210487	3,759355	3,389123	3,711263	3,47914	3,19251	4,023706	3,6199137	3,565118667	-0,054795	0,962731233	---
17238736	3,712343	3,489152	3,915752	3,735563	3,583049	3,634276	3,705749	3,650962667	-0,05478633	0,962737016	---
17542072	2,922515	2,943108	2,941673	2,907568	2,857174	2,878199	2,9357653	2,880980333	-0,054785	0,962737906	Mir471
17391287	5,931377	5,461473	6,061766	5,572026	5,823845	5,894517	5,8182053	5,763462667	-0,05474267	0,962766156	Prom2
17400412	7,832745	8,131443	7,525438	7,746288	7,8482	7,730957	7,8298753	7,775148333	-0,054727	0,962776611	Ensa
17330306	7,138244	7,283352	7,157374	7,082935	7,174235	7,157646	7,19299	7,138272	-0,054718	0,962782617	Gtf2e1
17384992	5,346015	5,475594	5,774528	5,484589	5,671298	5,276097	5,5320457	5,477328	-0,05471767	0,96278284	---
17537766	5,321312	4,949266	4,892461	5,389513	4,635474	4,973922	5,0543463	4,999636333	-0,05471	0,962787956	---
17544629	5,321312	4,949266	4,892461	5,389513	4,635474	4,973922	5,0543463	4,999636333	-0,05471	0,962787956	---
17333852	3,249118	3,340492	3,183182	3,189692	3,309126	3,109846	3,2575973	3,202888	-0,05470933	0,962788401	Vmn1r230
17364392	3,741563	3,99797	3,832021	3,92397	3,616196	3,867274	3,8571847	3,80248	-0,05470467	0,962791515	Cyp2c38
17207933	6,513415	5,935966	6,592054	5,767423	6,790361	6,319545	6,347145	6,292443	-0,054702	0,962793295	---
17468708	12,81392	12,54086	12,48991	12,60989	12,55793	12,51278	12,614897	12,5602	-0,05469667	0,962796854	---
17482247	4,388176	4,217528	4,274154	4,289316	4,217426	4,2091	4,293286	4,238614	-0,054672	0,962813316	Acsm2
17338684	7,867828	7,879262	7,925224	7,826221	7,946102	7,735995	7,8907713	7,836106	-0,05466533	0,962817765	Mpnd
17339466	4,227448	4,513472	4,430927	4,333336	4,42108	4,253498	4,3906157	4,335971333	-0,05464433	0,96283178	Spdya
17399817	6,678473	6,58705	6,751782	6,635853	6,617956	6,599566	6,672435	6,617791667	-0,05464333	0,962832448	S100a7a
17333696	3,70512	3,362398	3,535063	3,535063	3,692839	3,210752	3,5341937	3,479551333	-0,05464233	0,962833115	Vmn2r90
17423507	3,101153	3,242314	3,121825	3,068164	3,143386	3,089862	3,1550973	3,100470667	-0,05462667	0,962843571	---
17549724	5,602032	5,952929	5,815713	5,597013	5,739866	5,869927	5,7902247	5,735602	-0,05462267	0,96284624	---
17549726	5,602032	5,952929	5,815713	5,597013	5,739866	5,869927	5,7902247	5,735602	-0,05462267	0,96284624	---
17234363	4,142302	4,255931	4,121065	3,927653	4,305107	4,122671	4,1730993	4,118477	-0,05462233	0,962846463	4933407G14Rik
17526809	5,129884	5,206898	5,275495	5,227273	5,075731	5,145477	5,2040923	5,149493667	-0,05459867	0,962862258	1600029D21Rik // 1600029D21Rik
17262136	4,426524	4,143746	4,252492	4,204586	4,174554	4,27983	4,274254	4,219656667	-0,05459733	0,962863148	Gm4926
17508626	9,482033	9,658441	9,640548	9,552012	9,556795	9,508481	9,593674	9,539096	-0,054578	0,962876051	Gm5908
17493095	3,773893	3,460487	3,709269	3,54779	3,416655	3,815495	3,647883	3,593313333	-0,05456967	0,962881613	Vmn2r74
17548432	5,69059	5,968987	6,172242	5,971409	5,872544	5,824177	5,9439397	5,889376667	-0,054563	0,962886062	Gm10091
17475054	6,309428	6,104993	6,316234	6,191728	6,144938	6,230357	6,2435517	6,189007667	-0,054544	0,962898743	Lypd3
17253274	5,078498	4,544044	4,900385	4,852355	4,763161	4,743815	4,8409757	4,786443667	-0,054532	0,962906753	2210008F06Rik
17530128	7,57843	7,726443	7,549313	7,577081	7,461272	7,652238	7,618062	7,563530333	-0,05453167	0,962906975	Nck1
17269266	6,675989	6,600077	6,701799	6,689418	6,581709	6,543223	6,6592883	6,604783333	-0,054505	0,962924773	Krtap17-1
17345290	4,787587	4,690832	4,749596	4,637822	4,744096	4,682586	4,7426717	4,688168	-0,05450367	0,962925663	1600014C23Rik
17297227	7,080219	7,326991	7,100294	7,013561	7,184465	7,146075	7,169168	7,114700333	-0,05446767	0,962949692	Slc4a7
17238650	3,509637	3,158263	3,319891	3,465577	3,156468	3,20236	3,3292637	3,274801667	-0,054462	0,962953474	Olfr776
17206109	4,85029	4,510143	4,556449	5,102999	4,381765	4,268733	4,6389607	4,584499	-0,05446167	0,962953697	---
17289194	3,331629	3,064148	3,017676	3,103092	3,11971	3,0273	3,1378177	3,083367333	-0,05445033	0,962961261	---
17529962	6,28848	6,430515	6,540257	6,398272	6,362594	6,335046	6,4197507	6,365304	-0,05444667	0,962963709	4930579K19Rik
17263482	8,65973	8,686331	8,711832	8,611851	8,723392	8,55936	8,6859643	8,631534333	-0,05443	0,962974833	Cops3
17200177	5,702336	5,286549	5,508153	5,330516	5,485925	5,517382	5,4990127	5,444607667	-0,054405	0,962991521	---
17325540	5,362496	5,414332	5,403117	5,357894	5,187838	5,471036	5,393315	5,338922667	-0,05439233	0,962999976	Gpr156
17533234	7,390121	7,193179	6,700898	7,006082	6,981511	7,133451	7,0947327	7,040348	-0,05438467	0,963005093	Sytl5
17437552	2,863728	2,762903	3,116605	2,88014	2,858224	2,841813	2,914412	2,860059	-0,054353	0,963026231	---
17458948	3,981416	3,841763	3,79169	3,875733	3,829941	3,746155	3,871623	3,817276333	-0,05434667	0,963030459	Vmn1r12
17401117	8,403173	8,317144	8,342361	8,162036	8,40233	8,335281	8,354226	8,299882333	-0,05434367	0,963032461	Nras
17300760	4,166503	4,053535	3,798722	3,982435	3,810958	4,062348	4,0062533	3,951913667	-0,05433967	0,963035131	Rnf17
17549696	4,055134	4,354659	4,318381	4,063314	4,291178	4,210738	4,2427247	4,18841	-0,05431467	0,96305182	---
17436035	3,98371	4,083403	4,2253	4,305258	3,956088	3,868144	4,097471	4,043163333	-0,05430767	0,963056492	Dnajc5g
17526206	6,804281	6,85876	6,828511	6,64238	6,647301	7,038954	6,8305173	6,776211667	-0,05430567	0,963057827	C2cd2l
17497724	9,44972	9,622613	9,254693	9,239236	9,634039	9,290836	9,442342	9,388037	-0,054305	0,963058272	Ifitm6
17248754	6,160404	6,364116	6,175995	6,173605	6,07233	6,291779	6,233505	6,179238	-0,054267	0,963083639	Adam19
17457804	9,035933	8,452298	9,366735	9,098312	8,978295	8,615584	8,9516553	8,897397	-0,05425833	0,963089425	LOC665506
17538281	4,719571	4,476762	4,787296	4,524432	4,511938	4,784499	4,6612097	4,606956333	-0,05425333	0,963092763	Irs4 // Irs4
17425941	6,288563	5,633531	5,411956	5,785907	5,536004	5,849401	5,7780167	5,723770667	-0,054246	0,963097658	Mup1
17361814	7,014452	6,95047	6,953792	6,955444	6,952032	6,848523	6,9729047	6,918666333	-0,05423833	0,963102776	Frmd8
17362973	6,51013	6,969279	6,517962	6,598754	6,659599	6,576357	6,6657903	6,61157	-0,05422033	0,963114793	Ms4a6d
17459389	5,708867	5,176551	5,453817	5,710001	5,305602	5,160995	5,4464117	5,392199333	-0,05421233	0,963120133	---
17292103	5,367804	5,071918	5,359442	5,058865	5,480017	5,097761	5,266388	5,212214333	-0,05417367	0,963145947	---
17501224	7,27867	7,330667	7,2162	7,222599	7,116183	7,324248	7,275179	7,22101	-0,054169	0,963149062	---
17299826	6,249992	5,770855	6,137718	6,325342	5,824402	5,846348	6,052855	5,998697333	-0,05415767	0,963156629	---
17480324	5,676711	5,570149	5,509953	5,235302	5,668946	5,690107	5,5856043	5,531451667	-0,05415267	0,963159967	Kctd21
17468706	11,31691	11,38161	11,25356	11,2693	11,2292	11,29114	11,31736	11,26321333	-0,05414667	0,963163972	---
17430397	10,12017	10,20344	10,1009	9,976519	10,09342	10,19214	10,141503	10,08735967	-0,05414367	0,963165975	Hdac1
17282251	3,191272	3,575867	3,773115	3,488161	3,340706	3,548979	3,513418	3,459282	-0,054136	0,963171094	---
17233071	7,433079	7,658525	7,296082	6,577758	8,040866	7,606663	7,462562	7,408429	-0,054133	0,963173096	---
17259422	6,929325	6,901126	6,878852	6,903934	6,873179	6,769814	6,903101	6,848975667	-0,05412533	0,963178215	Anapc11
17494221	11,99852	12,29081	12,31763	12,13522	12,06631	12,2431	12,20232	12,14821	-0,05411	0,963188452	Hbb-b1
17431259	6,081095	6,019722	5,957747	5,764093	6,023158	6,109001	6,0195213	5,965417333	-0,054104	0,963192458	E130218I03Rik // E130218I03Rik
17357230	10,60451	10,74345	10,88431	10,68267	10,65718	10,73013	10,74409	10,68999333	-0,05409667	0,963197354	---
17339928	6,903676	7,224281	6,805746	6,892214	6,968071	6,91121	6,977901	6,923831667	-0,05406933	0,963215602	Gemin6
17283968	3,070915	3,050059	3,019252	2,947926	3,05535	2,974762	3,046742	2,992679333	-0,05406267	0,963220054	Mir376a // Mir376a
17248412	5,39613	5,484467	5,351159	5,364488	5,202476	5,502629	5,4105853	5,356531	-0,05405433	0,963225617	Fam196b
17353346	4,644887	4,688926	4,869044	4,742644	4,626734	4,671331	4,7342857	4,680236333	-0,05404933	0,963228956	4930455D15Rik
17491310	3,113776	3,113343	3,14178	3,044821	3,141372	3,020602	3,1229663	3,068931667	-0,05403467	0,963238748	Mrgpra6
17520675	5,917205	5,519078	5,965922	5,530428	5,72751	5,982208	5,800735	5,746715333	-0,05401967	0,963248763	Foxl2
17471201	7,219677	7,085457	7,18221	7,275377	7,009417	7,040572	7,162448	7,108455333	-0,05399267	0,96326679	Rad51ap1
17448411	4,291425	4,091892	4,23002	4,138628	3,954976	4,357804	4,2044457	4,150469333	-0,05397633	0,963277696	1700126H18Rik
17201215	5,051683	4,898826	5,451434	5,283037	4,238857	5,718136	5,133981	5,08001	-0,053971	0,963281257	---
17319248	5,697286	5,915371	5,780628	5,758286	5,677893	5,795281	5,7977617	5,74382	-0,05394167	0,963300843	Dmc1
17352106	3,585369	3,714417	3,695983	3,749721	3,470287	3,613946	3,6652563	3,611318	-0,05393833	0,963303069	---
17542163	3,259275	3,333705	3,494988	3,409556	3,323205	3,193406	3,362656	3,308722333	-0,05393367	0,963306185	4930567H17Rik
17315198	3,790703	3,768342	3,723634	3,763558	3,540355	3,817018	3,760893	3,706977	-0,053916	0,963317981	Mir1941
17275332	9,053637	8,985693	8,875573	8,799685	8,937365	9,016275	8,9716343	8,917775	-0,05385933	0,963355819	G2e3
17263506	5,116539	5,343241	5,118783	5,176541	5,117771	5,122697	5,1928543	5,139003	-0,05385133	0,963361161	1700013G23Rik
17500514	6,196123	6,365339	6,329757	6,142663	6,350982	6,23604	6,297073	6,243228333	-0,05384467	0,963365613	---
17473796	11,26673	11,39121	11,54689	11,42819	11,39593	11,21918	11,40161	11,34776667	-0,05384333	0,963366503	Rps5
17443797	6,301593	6,309633	6,311823	6,422702	6,259434	6,07945	6,307683	6,253862	-0,053821	0,963381417	Mblac1
17200093	4,631133	5,450119	5,555579	4,7537	5,492544	5,229153	5,212277	5,158465667	-0,05381133	0,963387872	---
17396223	5,676297	5,865834	5,87695	5,768367	5,546821	5,942496	5,8063603	5,752561333	-0,053799	0,963396108	Pde7a
17206601	3,748065	2,973876	3,519237	3,394112	3,267941	3,41775	3,413726	3,359934333	-0,05379167	0,963401005	---
17241396	9,878615	9,913513	9,892452	9,78513	9,868066	9,870031	9,89486	9,841075667	-0,05378433	0,963405902	Vps26a
17304993	5,642282	5,35009	5,582645	5,525652	5,425471	5,462563	5,5250057	5,471228667	-0,053777	0,963410799	Zfp488
17254707	5,328398	4,932738	5,294806	5,209387	5,112977	5,072259	5,185314	5,131541	-0,053773	0,96341347	Tex14
17377621	6,523017	6,495058	6,48516	6,477697	6,451919	6,412309	6,5010783	6,447308333	-0,05377	0,963415473	5430405G05Rik
17300185	3,482932	3,369024	3,375138	3,345295	3,354419	3,366079	3,4090313	3,355264333	-0,053767	0,963417477	---
17283649	11,40231	11,51576	11,52993	11,48942	11,44802	11,34927	11,482667	11,42890333	-0,05376333	0,963419925	Serpina11
17210143	4,931105	4,615856	4,969792	4,910157	4,743848	4,701458	4,8389177	4,785154333	-0,05376333	0,963419925	---
17478082	8,44546	8,42396	8,378139	8,326859	8,461115	8,298302	8,415853	8,362092	-0,053761	0,963421483	Nomo1
17490104	3,226022	3,091169	3,114026	2,843221	3,332548	3,094223	3,143739	3,089997333	-0,05374167	0,963434394	Vmn2r63
17380729	7,471103	7,486779	7,399146	7,256153	7,398158	7,541538	7,4523427	7,398616333	-0,05372633	0,963444634	Adrm1
17223446	10,47563	10,54946	10,58673	10,19707	10,581	10,67265	10,537273	10,48357333	-0,0537	0,96346222	Trak2
17353444	4,198998	4,256032	4,293288	3,995389	4,324023	4,267851	4,2494393	4,195754333	-0,053685	0,963472237	Nme5
17501520	5,75338	5,682347	5,629613	5,50342	5,924267	5,476598	5,6884467	5,634761667	-0,053685	0,963472237	
17269980	5,969863	5,681714	6,163127	5,867298	6,015757	5,770622	5,9382347	5,884559	-0,05367567	0,96347847	Sost
17325206	5,997415	6,237653	5,839296	6,022095	5,825806	6,065494	6,024788	5,971131667	-0,05365633	0,963491381	Adcy5
17402556	3,83888	4,208733	4,063614	4,028097	4,03733	3,884852	4,0370757	3,983426333	-0,05364933	0,963496056	Gm5712 // Gm5712
17232927	3,92715	3,744416	4,142173	3,90237	3,869049	3,88138	3,937913	3,884266333	-0,05364667	0,963497837	---
17277251	4,880675	4,669665	4,698034	4,581448	4,824511	4,681488	4,749458	4,695815667	-0,05364233	0,963500731	2900006K08Rik
17234433	5,897789	6,054843	5,836815	6,152785	5,716758	5,759034	5,9298157	5,876192333	-0,05362333	0,963513421	Mmp11
17489273	3,333154	3,644716	3,516666	3,380872	3,551095	3,401722	3,4981787	3,444563	-0,05361567	0,963518541	4930479M11Rik
17217562	6,144155	5,589895	6,116438	5,857746	6,080572	5,751344	5,9501627	5,896554	-0,05360867	0,963523216	Gm10535

03/02/17

17397421	3,340902	3,332546	3,51148	3,48494	3,363698	3,17553	3,394976	3,341389333	-0,05358667	0,963537909	Slc7a11 // Slc7a11
17463638	3,84539	3,897973	3,971938	3,924076	3,866978	3,763526	3,9051003	3,851526667	-0,05357367	0,963546591	Tas2r116
17374686	6,588115	6,939076	6,884768	6,628819	6,758926	6,86351	6,8039863	6,750418333	-0,053568	0,963550376	Rpusd2
17357150	9,710726	9,816753	9,547729	9,534184	9,671033	9,709295	9,691736	9,638170667	-0,05356533	0,963552157	Wdr74
17344497	8,211619	8,293169	8,124321	8,133425	8,224444	8,110552	8,209703	8,156140333	-0,05356267	0,963553938	Mrps18b
17424094	8,820141	8,969347	8,89403	8,8305	8,83028	8,862128	8,894506	8,840969333	-0,05353667	0,963571303	Bag1
17549824	5,178388	5,183341	5,338599	5,232213	4,873259	5,434279	5,2334427	5,179917	-0,05352567	0,96357865	---
17268137	6,425005	6,008198	6,160561	6,220956	6,135659	6,076632	6,1979213	6,144415667	-0,05350567	0,963592008	Itga3
17542967	3,347467	3,282378	3,298235	3,250164	3,242736	3,274684	3,30936	3,255861333	-0,05349867	0,963596684	---
17491366	3,905962	3,577945	3,523421	3,651848	3,632029	3,56303	3,6691093	3,615635667	-0,05347367	0,963613382	Mrgprb3
17387685	7,362379	7,465064	7,524674	7,712104	7,397091	7,082542	7,4507057	7,397245667	-0,05346	0,96362251	Olfr1077-ps1
17308684	3,490093	3,494826	3,452844	3,392002	3,237013	3,648384	3,4792543	3,425799667	-0,05345467	0,963626072	---
17398233	10,7524	10,81521	10,93931	10,73538	10,76512	10,84607	10,83564	10,78219	-0,05345	0,963629189	Smc4
17511167	8,663801	8,618497	8,587	8,478088	8,522151	8,708735	8,6230993	8,569658	-0,05344133	0,963634978	Rad23a
17459490	7,659557	7,52895	7,521494	7,401611	7,555708	7,592393	7,5700003	7,516570667	-0,05342967	0,963642771	Rnf103
17282102	3,117234	3,244513	3,233609	3,120125	3,085605	3,229347	3,198452	3,145025667	-0,05342633	0,963644997	---
17361454	4,966517	4,670183	5,178006	4,997212	4,978575	4,678671	4,9382353	4,884819333	-0,053416	0,963651899	Cnih2
17268240	5,892518	5,647473	5,844947	5,816418	5,601048	5,807225	5,7949793	5,741563667	-0,05341567	0,963652122	4833417C18Rik
17515128	6,194876	6,148495	6,323277	6,142636	6,258385	6,105514	6,222216	6,168845	-0,053371	0,963681958	Keap1
17447689	5,27114	5,051835	5,150446	5,200216	5,112844	5,000258	5,157807	5,104439333	-0,05336767	0,963684184	Zfp518b
17533729	3,395494	3,567881	3,509971	3,369062	3,531058	3,41314	3,4911153	3,437753333	-0,053362	0,96368797	Spaca5
17284894	8,014521	8,008793	7,714752	7,814864	7,739398	8,023729	7,9126887	7,859330333	-0,05335833	0,963690419	Asb13
17391587	6,118919	5,988324	6,041819	5,934754	6,078512	5,975761	6,0496873	5,996342333	-0,053345	0,963699325	---
17519785	4,327301	4,378386	3,894449	4,511217	3,809617	4,119284	4,2000453	4,146706	-0,05333933	0,96370311	4930429F24Rik
17391574	5,231984	4,706383	5,200673	4,982447	5,141554	4,855049	5,0463467	4,993016667	-0,05333	0,963709345	F830045P16Rik
17301200	3,76438	3,820454	3,810009	3,897413	3,773627	3,563815	3,798281	3,744951667	-0,05332933	0,96370979	Defb42
17303821	5,902252	6,088859	5,896672	6,001198	5,764902	5,961749	5,9625943	5,909283	-0,05331133	0,963721814	Zmynd17
17211541	3,60674	3,472628	3,468668	3,533705	3,377599	3,476799	3,516012	3,462701	-0,053311	0,963722037	4930521A18Rik
17305456	8,283092	7,999627	8,518752	8,373222	8,207087	8,061238	8,267157	8,213849	-0,053308	0,963724041	---
17291136	4,903659	5,021094	4,781198	4,779918	4,836267	4,929906	4,9019837	4,848697	-0,05328667	0,963738292	C230035I16Rik
17369948	8,882362	8,898641	8,876423	8,798767	8,714475	8,984368	8,8858087	8,832536667	-0,053272	0,963748089	Mir1954 // Mir1954
17361250	9,144003	9,124067	9,184468	9,06988	9,15544	9,067429	9,150846	9,097583	-0,053263	0,963754102	Kdm2a
17540897	4,424255	4,352179	4,495566	4,376582	4,404817	4,330825	4,424	4,370741333	-0,05325867	0,963756996	---
17417573	7,191959	7,105761	7,249172	7,171908	6,998813	7,216399	7,1822973	7,12904	-0,05325733	0,963757887	Hectd3
17547996	4,685498	4,761238	4,658307	4,79171	4,577107	4,57651	4,701681	4,648442333	-0,05323867	0,963770357	---
17467700	5,522395	5,527895	5,318683	5,51629	5,202826	5,490167	5,4563243	5,403094333	-0,05323	0,963776147	4933431G14Rik // 4933431G14Rik
17449887	6,14038	5,966817	6,066327	6,180129	5,866882	5,966839	6,0578413	6,004616667	-0,05322467	0,963779709	A430089I19Rik
17420438	10,304	10,20399	10,17745	10,18903	10,26192	10,07484	10,22848	10,17526333	-0,05321667	0,963785054	---
17210984	10,41129	10,32091	10,24026	10,27339	10,24479	10,29466	10,324153	10,27094667	-0,05320667	0,963791734	Pcmtd1
17258642	11,00057	11,16623	11,14371	11,03586	11,05462	11,06043	11,103503	11,05030333	-0,0532	0,963796188	Snord1b
17313721	7,901797	7,733947	7,789639	7,723641	7,902344	7,639963	7,808461	7,755316	-0,053145	0,963832932	Parvg
17387542	5,250088	4,833288	5,255777	4,85254	5,140337	5,186866	5,113051	5,059914333	-0,05313667	0,963838499	P2rx3
17379113	6,110008	5,843434	6,216085	6,048891	5,960395	6,000845	6,056509	6,003377	-0,053132	0,963841617	C330008G21Rik
17265326	6,030058	6,29656	6,246819	6,056449	6,244893	6,11271	6,1911457	6,138017333	-0,05312833	0,963844066	---
17404704	4,417098	4,106346	4,522015	4,211808	4,311744	4,362566	4,3484863	4,295372667	-0,05311367	0,963853865	Gm3143 // Gm3143
17548834	3,235903	3,360146	3,299532	3,127292	3,44678	3,162174	3,298527	3,245415333	-0,05311167	0,963855201	---
17472909	4,52962	4,465092	4,543143	4,542706	4,407153	4,428662	4,5126183	4,459507	-0,05311133	0,963855424	Gm6288
17377405	3,568015	3,395504	3,661624	3,466799	3,421662	3,577391	3,5417143	3,488617333	-0,053097	0,963865	Cst13
17401386	3,993257	4,027357	4,209536	3,916525	4,020593	4,133752	4,0767167	4,023623333	-0,05309333	0,96386745	4930564D02Rik
17428094	7,336823	7,472788	7,511546	7,364691	7,444108	7,353097	7,4403857	7,387298667	-0,053087	0,963871681	Zfyve9
17413399	5,37804	5,313597	5,379089	5,38407	5,29124	5,23619	5,3569087	5,303833333	-0,05307533	0,963879475	---
17494273	4,203717	3,97093	3,89452	3,961263	3,85159	4,097237	4,0230557	3,97003	-0,05302567	0,963912659	Olfr642
17237142	8,921836	9,031186	9,000068	9,013573	8,996043	8,784407	8,9843633	8,931341	-0,05302233	0,963914886	Ppp1r12a
17355270	4,830659	4,789552	4,788431	4,832376	4,487297	4,929915	4,8028807	4,749862667	-0,053018	0,963917781	4930546C10Rik
17416830	10,46339	10,59523	10,56798	10,41021	10,55272	10,50462	10,5422	10,48918333	-0,05301667	0,963918672	Eps15
17497554	8,059896	8,015324	8,135634	8,079807	8,040409	7,931622	8,0702847	8,017279333	-0,05300533	0,963926244	Tubgcp2
17532599	13,81799	13,85596	13,81083	13,73365	13,75963	13,83249	13,82826	13,77525667	-0,05300333	0,963927581	---
17453647	5,814138	5,875764	5,902498	5,795957	5,756467	5,88097	5,8641333	5,811131333	-0,053002	0,963928471	---
17319770	8,780408	8,822138	8,783887	8,705275	8,750839	8,771326	8,7954777	8,74248	-0,05299767	0,963931367	Poldip3
17285842	5,52169	5,345804	5,591379	4,779568	5,676427	5,843955	5,486291	5,433316667	-0,05297433	0,963946957	Hist2h3b // Hist1h3e // Hist1h3b // Hist1
17233741	8,823489	9,073307	8,694456	8,606602	9,033243	8,792493	8,8637507	8,810779333	-0,05297133	0,963948961	---
17473925	3,602498	3,588448	3,769269	3,595264	3,622376	3,583673	3,653405	3,600437667	-0,05296733	0,963951634	Bsph1
17220608	6,009105	6,11201	5,939432	5,985128	6,014355	5,902269	6,0201823	5,967250667	-0,05293167	0,963975465	Gpatch2
17251299	4,876245	4,681784	4,296015	4,55964	4,569147	4,566468	4,6180147	4,565085	-0,05292967	0,963976802	---
17523952	4,301426	4,240832	4,408017	4,162493	4,369256	4,259757	4,3167583	4,263835333	-0,052923	0,963981256	Cntn5
17443024	7,392567	7,420276	7,45102	7,31393	7,482486	7,308688	7,4212877	7,368368	-0,05291967	0,963983484	Tyw1
17550835	3,250056	3,322201	3,479588	3,153102	3,466275	3,273816	3,350615	3,297731	-0,052884	0,964007316	---
17267766	3,546011	3,501816	4,067469	3,668333	3,762403	3,525925	3,7050987	3,652220333	-0,05287833	0,964011102	---
17314629	3,381662	3,590194	3,377162	3,348936	3,488166	3,35332	3,4496727	3,396807333	-0,05286533	0,964019789	4930415O20Rik
17479168	4,140555	4,107736	4,362306	4,174542	4,093737	4,183733	4,2035323	4,150670667	-0,05286167	0,964022239	A730056A06Rik
17415629	5,016004	4,501645	5,498991	4,791055	5,281252	4,785757	5,0055467	4,952688	-0,05285867	0,964024244	---
17372458	3,210386	3,055028	3,095951	3,046941	3,147443	3,008441	3,120455	3,067608333	-0,05284667	0,964032262	---
17332181	7,991058	8,149315	7,929035	8,044369	7,994123	7,872402	8,023136	7,970298	-0,052838	0,964038053	Cryzl1
17292333	5,627461	5,702191	5,69511	5,622276	5,574833	5,669181	5,6749207	5,622096667	-0,052824	0,964047408	Ptpdc1
17478919	7,385448	7,766751	7,941787	7,540835	7,723908	7,670792	7,6979953	7,645178333	-0,052817	0,964052086	Tarsl2
17402111	4,364802	4,464415	4,288479	4,223811	4,341917	4,393538	4,3725653	4,319755333	-0,05281	0,964056764	Gm9916
17417240	8,11559	7,853965	8,067926	8,317041	7,641606	7,920408	8,0124937	7,959685	-0,05280867	0,964057655	Cyp4a31
17501230	6,270338	6,324267	6,352102	6,273179	6,270469	6,244655	6,315569	6,262767667	-0,05280133	0,964062555	Gm19464
17423728	4,448911	4,398594	4,569077	4,35113	4,484082	4,422982	4,472194	4,419398	-0,052796	0,964066119	4930548K13Rik // 4930548K13Rik
17467973	3,701056	3,501931	3,683934	3,579635	3,569971	3,579019	3,6289737	3,576208333	-0,05276533	0,964086612	Reg3g
17551153	4,902014	4,820228	4,749077	5,054964	4,641242	4,616821	4,823773	4,771009	-0,052764	0,964087503	---
17489503	5,439343	5,163127	5,338822	5,201098	5,130487	5,451425	5,313764	5,261003333	-0,05276067	0,96408973	Gm4684 // Gm4684
17506933	8,847151	8,458191	8,60595	8,477488	8,679558	8,596047	8,6370973	8,584364333	-0,052733	0,964108219	1810063B05Rik
17219164	5,26338	5,271706	4,921862	5,085938	5,164009	5,048812	5,152316	5,099586333	-0,05272967	0,964110447	Sh2d1b2
17415290	3,131934	3,465425	3,470375	3,263413	3,256901	3,389242	3,3559113	3,303185333	-0,052726	0,964112897	Mir491
17398368	4,956849	4,731696	4,698824	4,635658	4,706118	4,887444	4,7957897	4,743073333	-0,05271633	0,964119357	4921511C10Rik
17496848	3,018262	3,236495	3,308054	3,328724	3,089496	2,986459	3,1876037	3,134893	-0,05271067	0,964123144	---
17513092	5,536113	5,717609	5,617547	5,550482	5,646851	5,515836	5,6237563	5,571056333	-0,0527	0,964130272	Fa2h
17292101	3,886047	3,710649	3,711536	3,775985	3,80587	3,568302	3,7694107	3,716719	-0,05269167	0,964135841	---
17304131	6,078608	6,073481	6,298318	6,114909	6,074408	6,103015	6,1501357	6,097444	-0,05269167	0,964135841	---
17351652	4,029979	3,668943	3,826179	3,816195	3,746449	3,80446	3,8417003	3,789034667	-0,05266567	0,964153217	---
17204281	2,846894	2,9311	4,046899	3,42792	3,043937	3,19504	3,2749643	3,222299	-0,05266533	0,96415344	---
17219670	3,601759	4,444148	3,862803	4,228117	3,849058	3,67355	3,96957	3,916908333	-0,05266167	0,96415589	Pydc3 // Pydc3
17205033	5,739436	4,968508	5,745883	5,555816	5,293981	5,446056	5,484609	5,431951	-0,052658	0,96415834	---
17209223	5,739436	4,968508	5,745883	5,555816	5,293981	5,446056	5,484609	5,431951	-0,052658	0,96415834	---
17347801	8,351713	8,350288	7,987326	8,065253	8,215037	8,251078	8,2297757	8,177122667	-0,052653	0,964161682	Prepl
17448021	10,34647	10,52994	10,44331	10,44003	10,35209	10,36965	10,439907	10,38725667	-0,05265	0,964163687	Dhx15
17299243	3,725403	3,480229	3,56083	3,495368	3,55884	3,554327	3,5888207	3,536178333	-0,05264233	0,964168811	---
17385063	2,992322	3,093344	3,201956	3,115457	3,131783	2,882504	3,095874	3,043248	-0,052626	0,964179726	---
17538575	11,05591	11,26318	11,16576	11,05972	11,11215	11,15511	11,161617	11,10899333	-0,05262333	0,964181509	Alas2
17201541	4,448703	4,75937	4,30451	4,45794	4,462472	4,434302	4,5041943	4,451571333	-0,052623	0,964181731	---
17415798	5,990545	5,939186	5,919735	5,88863	5,895025	5,907976	5,949822	5,897210333	-0,05261167	0,964189306	Efcab7
17276182	8,942977	8,756189	8,725391	8,8234	8,774603	8,668822	8,8081857	8,755608333	-0,05257733	0,964212252	Jkamp
17217678	5,301782	5,388598	5,329561	5,095586	5,447909	5,318729	5,3399803	5,287408	-0,05257233	0,964215594	Cacna1s
17472581	4,260148	3,937409	4,352139	3,981	4,266166	4,144849	4,183232	4,130671667	-0,05256033	0,964223614	Gm766
17509235	8,51747	8,75313	8,449224	8,455835	8,663635	8,442707	8,5732747	8,520725667	-0,052549	0,964231188	D030016E14Rik
17288219	7,300015	7,424232	7,396698	7,124425	7,322261	7,516619	7,3736483	7,321101667	-0,05254667	0,964232748	Gm10139 // Gm10139
17460152	9,242348	9,205262	9,21778	9,210891	9,060972	9,235915	9,2217967	9,169259333	-0,05253733	0,964238986	Zfml
17502457	5,104565	5,090247	5,102768	5,097651	5,113317	4,929001	5,0991933	5,046656333	-0,052537	0,964239209	Med26
17548036	5,86656	5,893585	5,89083	5,655248	5,952786	5,885345	5,8836583	5,831126333	-0,052532	0,96424255	Gm9258
17533294	6,765357	6,643457	6,624946	6,639429	6,623448	6,613301	6,67792	6,625392667	-0,05252733	0,964245669	Gm14474
17430388	4,230151	4,17477	4,452351	4,245671	4,221477	4,232574	4,2857573	4,233240667	-0,05251667	0,964252799	Tssk3
17493308	3,93791	3,656264	3,74735	3,976294	3,669898	3,537791	3,780508	3,727994333	-0,05251367	0,964254804	---
17486635	5,81914	5,32081	5,746841	5,724317	5,460583	5,544415	5,6289303	5,576438333	-0,052492	0,964269285	Mzf1
17341699	9,603048	9,645098	9,380869	9,273458	9,671874	9,526216	9,543005	9,490516	-0,052489	0,96427129	---
17441652	4,324535	4,560919	4,562501	4,286379	4,418273	4,585841	4,4826517	4,430164333	-0,05248733	0,964272404	Lhx5
17512350	5,607119	5,627151	5,640178	5,659465	5,584149	5,47339	5,624816	5,572334667	-0,05248133	0,964276415	4931428F04Rik
17206909	6,266021	6,352037	6,500333	6,539293	6,53181	5,889878	6,372797	6,320327	-0,05247	0,96428399	---
17448781	5,704705	6,75072	6,467963	6,465245	6,307243	5,993499	6,307796	6,255329	-0,052467	0,964285995	---
17333289	3,375845	3,383857	3,227404	3,218041	3,22956	3,382124	3,3290353	3,276575	-0,05246033	0,964290451	C030013G03Rik // C030013G03Rik
17537271	3,80228	3,949107	4,082889	3,890307	3,821157	3,965444	3,9447587	3,892302667	-0,052456	0,964293347	Zfp711
17320203	5,078254	5,057573	5,242651	5,148323	5,088911	4,983893	5,1261593	5,073709	-0,05245033	0,964297135	Ttll8
17490532	7,06829	6,756483	7,163899	7,042039	7,062865	6,726429	6,996224	6,943777667	-0,05244633	0,964299808	Scaf1
17418187	8,184166	8,171977	8,304112	8,084039	8,239552	8,179342	8,220085	8,167644333	-0,05244067	0,964303596	Pabpc4
17278713	3,640608	3,572966	3,58105	3,155918	3,798754	3,682639	3,598208	3,545770333	-0,05243767	0,964305601	Rtl1 // Rtl1
17353554	12,27549	12,26436	12,23088	12,20318	12,21117	12,19909	12,25691	12,20448	-0,05243	0,964310726	Hspa9
17409649	10,69262	10,63059	10,59887	10,57487	10,66347	10,52645	10,640693	10,58826333	-0,05243	0,964310726	Vcam1
17431174	8,772358	8,626622	8,497624	8,530938	8,6283	8,580119	8,6322013	8,579785667	-0,05241567	0,964320306	Cd52
17329568	4,667963	4,614758	4,806508	4,662586	4,677975	4,591444	4,6964097	4,644001667	-0,052408	0,964325431	Atp13a4
17397268	8,740242	8,922613	8,919744	8,771002	8,798138	8,856255	8,8608663	8,808465	-0,05240133	0,964329887	Plk4
17343483	6,178298	6,04641	6,289408	6,13462	5,968867	6,253453	6,171372	6,11898	-0,052392	0,964336126	Zfp870
17356447	7,06424	7,135149	7,15678	7,291036	6,984726	6,923263	7,118723	7,066341667	-0,05238133	0,964343256	Efemp2
17392956	3,672987	3,078138	3,523641	3,5272	3,375098	3,215331	3,424922	3,372543	-0,052379	0,964344815	Defb26
17482083	9,5966	9,589172	9,498817	9,500303	9,550182	9,476975	9,5615297	9,509153333	-0,05237633	0,964346598	1110004F10Rik

17229644	6,744884	6,656965	6,902549	6,679664	6,729773	6,737839	6,7681327	6,715758667	-0,052374	0,964348157	Tomm40l
17210527	2,674052	3,377439	3,56083	2,813301	3,064609	3,577303	3,204107	3,151737667	-0,05236933	0,964351277	---
17345542	8,048956	8,122078	8,039011	7,821459	8,158549	8,072981	8,070015	8,017663	-0,052352	0,964362863	Ppp2r5d
17514906	2,855742	2,906775	2,811769	2,805317	2,850745	2,761171	2,8580953	2,805744333	-0,052351	0,964363532	---
17374451	5,475755	5,152052	5,175456	5,363938	5,225789	5,056484	5,2677543	5,215403667	-0,05235067	0,964363754	---
17428142	9,169121	9,137947	9,019416	9,022664	9,124407	9,022385	9,108828	9,056485333	-0,05234267	0,964369102	Osbpl9
17203977	6,413485	6,766469	6,79395	6,69897	6,54231	6,575622	6,657968	6,605634	-0,052334	0,964374895	---
17207739	3,358049	4,721283	3,383048	4,278385	3,676204	3,350798	3,8207933	3,768462333	-0,052331	0,964376901	---
17303958	5,2274	5,275793	5,296285	5,286909	5,050609	5,305017	5,2664927	5,214178333	-0,05231433	0,964388042	Dupd1
17309535	5,838745	5,991328	5,851414	5,840708	5,900307	5,783583	5,893829	5,841532667	-0,05229633	0,964400074	Mbnl2
17208147	4,632237	4,90187	4,438873	4,962463	4,256186	4,597442	4,65766	4,605363667	-0,05229633	0,964400074	---
17299767	3,919331	3,900522	3,94367	4,21653	3,786991	3,603116	3,9211743	3,868879	-0,05229533	0,964400742	Olfr1509
17335900	5,995288	5,919897	5,84019	5,745634	6,001756	5,851109	5,9184583	5,866166333	-0,052292	0,964402971	---
17279534	5,451106	5,177385	5,609597	5,329578	5,320657	5,431026	5,412696	5,360420333	-0,05227567	0,964413889	---
17211984	4,540313	4,397795	4,537863	4,584705	4,640182	4,094357	4,4919903	4,439748	-0,05224233	0,964436172	---
17548912	10,67653	10,62432	10,47762	10,5142	10,50385	10,6037	10,592823	10,54058333	-0,05224	0,964437732	Cbx3 // Cbx3
17330314	8,195561	8,248091	8,019917	7,993025	8,123048	8,190783	8,154523	8,102285333	-0,05223767	0,964439292	Ndufb4
17273159	4,82181	4,913138	5,305062	4,846871	5,276218	4,760218	5,0133367	4,961102333	-0,05223433	0,96444152	Ppp1r27
17548133	10,56007	10,9373	10,90034	10,81318	10,72498	10,70287	10,799237	10,74701	-0,05222667	0,964446645	---
17202935	4,507386	4,453229	4,882293	4,8701	4,315145	4,50105	4,6143027	4,562098333	-0,05220433	0,964461575	---
17514853	6,705749	7,041369	6,878862	6,856122	6,771131	6,842116	6,8753267	6,823123	-0,05220367	0,964462021	Slc36a4
17335435	6,017808	6,155876	6,531607	6,167292	6,098034	6,283425	6,235097	6,182917	-0,05218	0,964477843	---
17221544	4,179447	3,977558	4,168912	4,104087	4,076862	3,988496	4,108639	4,056481667	-0,05215733	0,964492996	Tfap2d
17410031	5,365795	5,245042	5,613001	5,200336	5,349587	5,517462	5,407946	5,355795	-0,052151	0,96449723	Synpo2
17389704	4,680565	5,05339	4,667115	4,747226	4,717359	4,780066	4,8003567	4,748217	-0,05213967	0,964504807	A930104D05Rik
17535734	3,439956	2,675952	2,993496	2,927043	2,836474	3,189484	3,036468	2,984333667	-0,05213433	0,964508372	---
17399876	4,177296	4,064938	4,511601	4,20325	4,163224	4,230987	4,2512783	4,199153667	-0,05212467	0,964514835	Sprr2f
17549368	5,504698	5,834517	6,061872	5,711276	5,755249	5,778202	5,8003623	5,748242333	-0,05212	0,964517955	---
17549746	5,504698	5,834517	6,061872	5,711276	5,755249	5,778202	5,8003623	5,748242333	-0,05212	0,964517955	---
17339554	8,671172	8,548943	8,55778	8,589162	8,490112	8,542289	8,5926317	8,540521	-0,05211067	0,964524195	Lbh
17279088	6,126512	6,162035	6,147232	5,916558	6,213398	6,149529	6,1452597	6,093161667	-0,052098	0,964532663	Amn
17366754	4,004303	4,101352	3,50473	4,206144	3,315062	3,93289	3,8701283	3,818032	-0,05209633	0,964533777	Mir669a-1 // Mir669a-1
17366766	4,004303	4,101352	3,50473	4,206144	3,315062	3,93289	3,8701283	3,818032	-0,05209633	0,964533777	Mir669a-1 // Mir669a-1
17366776	4,004303	4,101352	3,50473	4,206144	3,315062	3,93289	3,8701283	3,818032	-0,05209633	0,964533777	Mir669a-1 // Mir669a-1
17366786	4,004303	4,101352	3,50473	4,206144	3,315062	3,93289	3,8701283	3,818032	-0,05209633	0,964533777	Mir669a-1 // Mir669a-1
17366798	4,004303	4,101352	3,50473	4,206144	3,315062	3,93289	3,8701283	3,818032	-0,05209633	0,964533777	Mir669a-1 // Mir669a-1
17366822	4,004303	4,101352	3,50473	4,206144	3,315062	3,93289	3,8701283	3,818032	-0,05209633	0,964533777	Mir669a-1 // Mir669a-1
17366834	4,004303	4,101352	3,50473	4,206144	3,315062	3,93289	3,8701283	3,818032	-0,05209633	0,964533777	Mir669a-1 // Mir669a-1
17366858	4,004303	4,101352	3,50473	4,206144	3,315062	3,93289	3,8701283	3,818032	-0,05209633	0,964533777	Mir669a-1 // Mir669a-1
17366870	4,004303	4,101352	3,50473	4,206144	3,315062	3,93289	3,8701283	3,818032	-0,05209633	0,964533777	Mir669a-1 // Mir669a-1
17366882	4,004303	4,101352	3,50473	4,206144	3,315062	3,93289	3,8701283	3,818032	-0,05209633	0,964533777	Mir669a-1 // Mir669a-1
17366908	4,004303	4,101352	3,50473	4,206144	3,315062	3,93289	3,8701283	3,818032	-0,05209633	0,964533777	Mir669a-1 // Mir669a-1
17366920	4,004303	4,101352	3,50473	4,206144	3,315062	3,93289	3,8701283	3,818032	-0,05209633	0,964533777	Mir669a-1 // Mir669a-1
17529229	2,990992	3,007552	3,123099	3,063287	2,927643	2,974432	3,0405477	2,988454	-0,05209367	0,96453556	---
17357801	4,456511	3,998589	4,324008	4,323291	4,252102	4,047457	4,2597027	4,207616667	-0,052086	0,964540686	Olfr1441
17392633	5,225247	5,650468	5,542796	5,218325	5,47269	5,571371	5,472837	5,420795333	-0,05204167	0,964570326	Zfp120 // Zfp120
17207099	3,499334	3,330931	3,378736	3,266226	3,477529	3,309126	3,4030003	3,350960333	-0,05204	0,964571441	---
17402977	3,561119	3,620055	3,443643	3,394487	3,552005	3,522206	3,5416057	3,489566	-0,05203967	0,964571663	---
17452873	7,797706	8,061277	8,000879	7,920845	7,938328	7,844589	7,9532873	7,901254	-0,05203333	0,964575898	Eif2b1
17506752	2,956969	3,182292	2,911892	2,9045	3,04637	2,944232	3,017051	2,965034	-0,052017	0,964586818	---
17241981	5,836306	5,926929	6,001292	5,735572	5,979658	5,893274	5,921509	5,869501333	-0,05200767	0,964593059	Mmp11
17509591	5,031878	4,845589	4,990294	4,876276	4,919171	4,916367	4,9559203	4,903938	-0,05198233	0,964609997	Tll1
17333872	6,737584	6,885446	6,711664	6,686683	6,878877	6,613234	6,7782313	6,726264667	-0,05196667	0,964620472	Zfp160
17427928	5,890664	5,870051	6,099994	5,893298	5,936467	5,875083	5,9535697	5,901616	-0,05195367	0,964629164	Dio1
17459712	4,130691	3,813649	3,77917	3,718918	3,978034	3,870744	3,9078367	3,855898667	-0,051938	0,964639639	Gm20560
17363154	4,103854	3,953605	3,483532	3,857157	3,766465	3,761589	3,846997	3,795070333	-0,05192667	0,964647217	Olfr1475
17434141	6,543142	6,296621	6,683295	6,519432	6,325003	6,522917	6,507686	6,455784	-0,051902	0,96466371	Ccl19
17227810	3,438023	3,527135	3,460742	3,424343	3,483575	3,362293	3,4753	3,423403667	-0,05189633	0,964667499	---
17246577	5,874774	5,810896	5,869185	5,921932	5,827775	5,649518	5,8516183	5,799741667	-0,05187667	0,96468065	Pik3ip1
17374137	4,105331	4,138167	4,168805	4,172001	4,042615	4,042139	4,1374343	4,085585	-0,05184933	0,964698927	Slc5a12
17387672	2,786518	3,086382	3,189512	2,992045	2,910593	3,004241	3,020804	2,968959667	-0,05184433	0,96470227	Olfr1058
17242856	6,80923	7,340844	7,131864	7,070695	7,100592	6,955172	7,0939793	7,042153	-0,05182633	0,964714307	2310011J03Rik
17281477	3,721076	3,478396	3,573906	3,732045	3,486413	3,399453	3,591126	3,539303667	-0,05182233	0,964716981	---
17458385	5,612846	5,60439	5,57901	5,640837	5,286128	5,713885	5,5987487	5,54695	-0,05179867	0,964732807	Gimap5
17424034	3,869468	4,093532	3,691924	3,840228	3,905784	3,753519	3,8849747	3,833177	-0,05179767	0,964733476	4933428C19Rik // 4933428C19Rik
17378440	5,482994	6,046844	5,896408	5,734409	5,788404	5,748074	5,8087487	5,756962333	-0,05178633	0,964741055	Procr
17234716	5,09689	4,567762	4,957929	4,743337	5,036233	4,68773	4,8741937	4,822433333	-0,05176033	0,964758441	Gm10100
17269343	4,040563	4,337238	4,083148	4,013481	4,149793	4,142406	4,1536497	4,101893333	-0,05175633	0,964761116	---
17370269	4,179262	4,370976	4,355401	4,089267	4,402646	4,258488	4,3018797	4,250133667	-0,051746	0,964768026	9030204H09Rik
17379990	4,360913	4,384897	4,831313	4,388447	4,439078	4,594411	4,5257077	4,473978667	-0,051729	0,964779395	---
17426251	5,891743	5,603348	5,815381	5,80887	5,634486	5,711943	5,7701573	5,718433	-0,05172433	0,964782515	Kif12
17404109	8,898458	9,010294	8,837955	8,865631	8,839123	8,886802	8,915569	8,863852	-0,051717	0,964787419	Impa1
17205203	6,254818	6,024207	6,541655	6,15891	6,105115	6,401511	6,27356	6,221845333	-0,05171467	0,96478898	---
17209393	6,254818	6,024207	6,541655	6,15891	6,105115	6,401511	6,27356	6,221845333	-0,05171467	0,96478898	---
17536848	3,825488	3,954236	4,246272	3,998934	3,812256	4,059671	4,0086653	3,956953667	-0,05171167	0,964790986	8030474K03Rik
17423337	4,083868	4,062224	3,818449	4,246981	3,703469	3,859092	3,9881803	3,936514	-0,05166633	0,964821303	---
17456934	11,28284	11,19524	11,11227	11,19309	11,05555	11,1868	11,196783	11,14514667	-0,05163667	0,964841143	Mest
17433610	6,301579	6,401834	6,341574	6,160587	6,358981	6,370518	6,348329	6,296695333	-0,05163367	0,964843149	Arhgef16
17543214	6,813124	6,864583	6,916485	6,833274	6,794403	6,81163	6,8647307	6,813102333	-0,05162833	0,964846716	Zfx
17232181	4,483706	4,331009	4,393875	4,614216	4,220791	4,218728	4,4028633	4,351245	-0,05161833	0,964853404	Taar4
17448135	4,464962	4,325379	4,375489	4,239702	4,313178	4,458119	4,38861	4,336999667	-0,05161033	0,964858754	Cckar
17291343	7,246031	7,150689	7,327423	7,173635	7,181481	7,214211	7,241381	7,189775667	-0,05160533	0,964862098	Gmnn
17382489	7,768545	7,879495	7,927817	7,764177	7,843259	7,813607	7,858619	7,807014333	-0,05160467	0,964862544	Ssna1
17358706	11,06717	11,07783	10,94463	10,93223	10,97266	11,03003	11,029877	10,97830667	-0,05157	0,964885729	Pten
17412330	5,121601	4,743805	4,827543	4,800304	4,875058	4,862893	4,8976497	4,846085	-0,05156467	0,964889296	Lasc1 // Lasc1
17480439	4,581343	4,525166	4,538571	4,449923	4,604492	4,436012	4,54836	4,496809	-0,051551	0,964898437	Gdpd4
17220934	5,960513	5,801689	5,688477	5,6757	5,886611	5,733733	5,816893	5,765348	-0,051545	0,964902449	---
17296124	7,329705	6,773232	7,057326	7,107779	6,856891	7,040985	7,053421	7,001885	-0,051536	0,964908469	---
17548119	5,514989	5,381143	5,317138	5,263067	5,406561	5,389049	5,4044233	5,352892333	-0,051531	0,964911813	Gm10815
17441587	4,710528	4,799522	5,008633	4,585529	4,785004	4,993563	4,839561	4,788032	-0,051529	0,964913151	G630008E18
17209161	3,488983	4,197963	4,049281	3,661209	3,832943	4,087492	3,9120757	3,860548	-0,05152767	0,964914042	---
17362609	6,859857	6,860788	6,919454	6,819581	6,929061	6,736892	6,880033	6,828511333	-0,05152167	0,964918055	Fen1
17234747	6,513415	6,088281	6,183754	6,218175	6,435448	5,977266	6,2618167	6,210296333	-0,05152033	0,964918947	1810043G02Rik
17389962	6,065315	5,93104	6,087172	5,988646	5,981	5,959343	6,0278423	5,976329667	-0,05151267	0,964924075	Rpap1
17244191	9,987987	10,02591	10,03259	9,939515	9,956171	9,996266	10,015496	9,963984	-0,05151167	0,964924744	Tmpo
17525722	3,965736	3,725183	3,760499	3,908149	3,767019	3,62175	3,8171393	3,765639333	-0,0515	0,964932547	Olfr947-ps1
17333107	4,847333	4,49086	4,853806	4,74936	4,92516	4,363037	4,7306663	4,679185667	-0,05148067	0,964945478	T2
17296104	6,222934	6,240121	6,063895	6,024364	6,303044	6,04515	6,17565	6,124186	-0,051464	0,964956625	Ndufaf2
17462830	3,842773	3,09192	3,160444	3,625409	2,943975	3,371384	3,3650457	3,313589333	-0,05145633	0,964961753	---
17529003	4,530784	4,423763	4,258865	4,424182	4,344559	4,290302	4,4044707	4,353014333	-0,05145633	0,964961753	Omt2a
17460938	5,277959	5,216413	5,403013	5,226779	5,253742	5,262507	5,2991283	5,247676	-0,05145233	0,964964429	---
17231619	3,45424	3,533818	3,809049	3,300533	3,518066	3,824157	3,5990357	3,547585333	-0,05145033	0,964965766	---
17546055	6,29441	6,244153	6,015886	6,122543	5,731179	6,546394	6,1848163	6,133372	-0,05144433	0,96496978	Egfl6
17262340	11,31855	11,62709	11,46128	11,33177	11,27219	11,64863	11,468973	11,41753	-0,05144333	0,964970448	---
17550394	11,0011	11,25539	11,20263	11,04068	11,16072	11,10342	11,15304	11,10160667	-0,05143333	0,964977137	---
17264482	6,32987	6,652903	6,705096	6,489963	6,633327	6,410323	6,562623	6,511204333	-0,05141867	0,964986947	Pfas
17284509	4,063729	3,896184	4,005614	3,992451	4,000248	3,818637	3,988509	3,937112	-0,051397	0,96500144	---
17240685	3,046152	2,9105	2,930868	2,846127	2,970685	2,916523	2,9625067	2,911111667	-0,051395	0,965002778	---
17405754	3,514011	3,450102	3,446679	3,495961	3,307138	3,453524	3,470264	3,418874333	-0,05138967	0,965006345	---
17313091	6,760902	6,815048	6,755911	6,912277	6,583576	6,681857	6,777287	6,725903333	-0,05138367	0,965010358	Smcr7l
17375885	5,11937	4,867807	5,023265	4,932996	5,051091	4,872237	5,0034807	4,952108	-0,05137267	0,965017716	Gpat2
17290121	9,451486	9,458843	9,518334	9,441198	9,398619	9,434742	9,476221	9,424853	-0,051368	0,965020838	Paip1
17542718	6,198693	5,958389	6,263416	6,146403	6,061118	6,05891	6,140166	6,088810333	-0,05135567	0,965029088	DXBay18 // DXBay18
17319134	5,079021	5,433676	5,304819	5,31988	5,257785	5,085787	5,2725053	5,221150667	-0,05135467	0,965029756	Sox10
17213702	8,010436	7,985826	7,817933	7,756946	7,902933	8,000254	7,938065	7,886711	-0,051354	0,965030202	Pikfyve
17394454	8,725624	9,482935	9,936469	9,826155	9,277564	8,887249	9,381676	9,330322667	-0,05135333	0,965030648	Ocstamp
17220787	5,395031	5,081196	5,371119	5,086016	5,199988	5,407324	5,2824487	5,231109333	-0,05133933	0,965040013	Batf3
17396064	3,84539	3,224125	3,71822	3,57314	3,277144	3,783456	3,5959117	3,54458	-0,05133167	0,965045141	Gm9833
17205533	2,784333	4,346232	4,537863	3,651848	3,651848	4,210738	3,889476	3,838144667	-0,05133133	0,965045364	---
17314848	4,61789	4,577854	5,060022	4,827682	4,621687	4,652419	4,751922	4,700596	-0,051326	0,965048932	Aqp6
17294691	3,884115	3,764686	3,752829	3,754945	3,707979	3,78473	3,8005433	3,749218	-0,05132533	0,965049378	A230107N01Rik
17254015	4,989518	4,92685	4,88094	5,06959	4,798777	4,774967	4,932436	4,881111333	-0,05132467	0,965049824	---
17308672	5,235575	5,167064	4,80701	4,857524	5,272425	4,925746	5,069883	5,018565	-0,051318	0,965054283	---
17358212	5,287195	5,278311	5,224212	5,136053	5,375622	5,124101	5,2632393	5,211925333	-0,051314	0,965056959	2410080I02Rik
17485266	5,796923	5,776407	5,936872	5,697223	5,789776	5,86931	5,836734	5,785436333	-0,05129767	0,965067885	Igf2as
17421120	7,556429	7,483404	7,431534	7,346568	7,420846	7,550076	7,4904557	7,439163333	-0,05129233	0,965071453	Casp9
[truncated: 3,747,704 more chars]
